# ^13^C-metabolic flux ratio and novel carbon path analyses confirmed that *Trichoderma reesei *uses primarily the respirative pathway also on the preferred carbon source glucose

**DOI:** 10.1186/1752-0509-3-104

**Published:** 2009-10-29

**Authors:** Paula Jouhten, Esa Pitkänen, Tiina Pakula, Markku Saloheimo, Merja Penttilä, Hannu Maaheimo

**Affiliations:** 1 VTT Technical Research Centre of Finland, Espoo, Finland; 2 Department of Computer Science, University of Helsinki, Helsinki, Finland

## Abstract

**Background:**

The filamentous fungus *Trichoderma reesei *is an important host organism for industrial enzyme production. It is adapted to nutrient poor environments where it is capable of producing large amounts of hydrolytic enzymes. In its natural environment *T. reesei *is expected to benefit from high energy yield from utilization of respirative metabolic pathway. However, *T. reesei *lacks metabolic pathway reconstructions and the utilization of the respirative pathway has not been investigated on the level of *in vivo *fluxes.

**Results:**

The biosynthetic pathways of amino acids in *T. reesei *supported by genome-level evidence were reconstructed with computational carbon path analysis. The pathway reconstructions were a prerequisite for analysis of *in vivo *fluxes. The distribution of *in vivo *fluxes in both wild type strain and *cre1*, a key regulator of carbon catabolite repression, deletion strain were quantitatively studied by performing ^13^C-labeling on both repressive carbon source glucose and non-repressive carbon source sorbitol. In addition, the ^13^C-labeling on sorbitol was performed both in the presence and absence of sophorose that induces the expression of cellulase genes. Carbon path analyses and the ^13^C-labeling patterns of proteinogenic amino acids indicated high similarity between biosynthetic pathways of amino acids in *T. reesei *and yeast *Saccharomyces cerevisiae*. In contrast to *S. cerevisiae*, however, mitochondrial rather than cytosolic biosynthesis of Asp was observed under all studied conditions. The relative anaplerotic flux to the TCA cycle was low and thus characteristic to respiratory metabolism in both strains and independent of the carbon source. Only minor differences were observed in the flux distributions of the wild type and *cre1 *deletion strain. Furthermore, the induction of the hydrolytic gene expression did not show altered flux distributions and did not affect the relative amino acid requirements or relative anabolic and respirative activities of the TCA cycle.

**Conclusion:**

High similarity between the biosynthetic pathways of amino acids in *T. reesei *and yeast *S. cerevisiae *was concluded. *In vivo *flux distributions confirmed that *T. reesei *uses primarily the respirative pathway also when growing on the repressive carbon source glucose in contrast to *Saccharomyces cerevisiae*, which substantially diminishes the respirative pathway flux under glucose repression.

## Background

The industrially important protein producer, the filamentous fungus *Trichoderma reesei*, a clonal derivative of the ascomycete *Hypocrea jecorina*, is adapted to growth in nutrient poor environments, where it is able to use complex plant material as carbon source. *T. reesei *and a number of other filamentous fungi and cellulolytic bacteria produce and secrete plant polymer hydrolyzing enzymes, such as cellulases and hemicellulases, into their surroundings to break down the polymers into easily metabolizable monomers [1].

Because of its ability to synthesize and secrete large amounts of proteins, *T. reesei *has gained industrial importance in production of enzymes of native and heterologous origin. Carbon catabolite repression (CCR) of *T. reesei *negatively regulates the powerful production machinery of the hydrolytic enzymes when a preferred carbon source, such as glucose, is available. Inducers of hydrolytic enzyme expression are often small oligosaccharides or derivative parts of the polymers from the environment of the fungus. The inductive signaling leads to synthesis of specific sets of enzymes [2,3]. In *T. reesei*, D-xylose, xylobiose, sophorose, and lactose have been observed to trigger production of particular enzyme sets [4,5]. Sophorose, a molecule of two beta-1,2-linked glucose units, is an efficient inducer of cellulose gene expression at low concentration (1-2 mM) when *T. reesei *is growing on a non-repressing carbon source, such as sorbitol or glycerol [6]. However, in high glucose concentrations CCR overrules the inductive signals in *T. reesei *[6].

Sorbitol as a carbon source neither provokes CCR nor triggers the cellulase gene expression in *T. reesei *[6]. Nevertheless, cellulase production is positively correlated with the ability of different *T. reesei *strains to grow on D-sorbitol [7], which could be converted to L-sorbose [8] that induces cellulase expression in *T. reesei *[9]. In *T. reesei *L-arabinitol 4-dehydrogenase (Lad1) is involved in the initial oxidization of D-sorbitol at C2 to convert it to D-fructose [10]. A specific sorbitol dehydrogenase converts sorbitol to fructose in *Aspergilli *fungi [11,2].

Cre1 is the key mediator protein of CCR in *T. reesei *[12,13]. *Trichoderma *Cre1 has a 95% sequence similarity with *Aspergillus *CreA in regions of the zinc-finger and proline-serine-threonine-rich domain and the complete sequences are 46% identical [13]. Cre1 is structurally also highly similar to Mig1, a key protein in glucose repression in yeast *Saccharomyces cerevisiae *[12,13]. However, the functional dissimilarities observed between Cre1/CreA and Mig1, in spite of the sequence and structural similarity, have led to the conclusion that glucose repression functionalities in filamentous fungi and yeasts have evolved separately [14,15]. Pfeiffer *et al *argued that the evolution from unicellular to undifferentiated multicellular organisms, like *T. reesei*, has been facilitated by the general preference of high yield energy generation through respiration even in the presence of a preferred carbon source [16]. In contrast to CCR regulation in *S. cerevisiae*, it has been shown that in *T. reesei *CCR does not cause repression of genes encoding the TCA cycle enzymes or respiratory pathway components [17,18]. David *et al *observed differences in the distribution of intracellular carbon fluxes in central carbon metabolism between *A. nidulans *reference and a carbon repression deletion mutant (*creA*Δ4) strains when they were grown on glucose [19].

Despite the industrial importance of *T. reesei*, its genome has only recently been sequenced [20] and its metabolism, beyond that related to protein production and secretion, is narrowly studied. In the present work, computational carbon path analysis [21] was utilized to reconstruct the biosynthetic pathways of amino acids. That was essential for quantitative flux analysis, as no metabolic network model was available for *T. reesei*. The localizations of the key reactions in the biosynthetic pathways of amino acids were determined from the ^13^C-labeling patterns of proteinogenic amino acids and by computational estimation of targeting peptide sequences. The intracellular metabolic flux ratios in the central carbon metabolism were determined utilizing fractional ^13^C-labeling and metabolic flux ratio (METAFoR) analysis [22] in a wild type (QM6a) strain and in a Δ*cre1 *mutant strain (L161a), when grown on the repressive carbon source glucose and on the neutral carbon source sorbitol. Additionally, the effect of sophorose induction of cellulase gene expression on the relative fluxes in the central carbon metabolism was quantified. To the authors' knowledge this is the first time that the metabolic pathways of *T. reesei *have been reconstructed and *in vivo *fluxes in the central carbon metabolism of *T. reesei *have been quantitatively studied.

## Results and Discussion

### ^13^C-labeling in batch cultures

Metabolic flux ratio (METAFoR) analysis was performed for the *T. reesei *wild type (QM6a) and Δ*cre1 *(L161a) strains growing in minimal medium in flasks with fractional [U-^13^C]glucose and on fractional [U-^13^C]sorbitol with and without induction of cellulase gene expression by sophorose. Since ^13^C-metabolic flux ratio (METAFoR) analysis is based on biosynthetically directed fractional (BDF) labeling of the constituents of biomass biopolymers, it requires constant intracellular flux distribution during the labeling [22-28]. Constant flux distribution can be achieved in a chemostat culture, where the specific growth rate is constant, or in a batch cultivation during exponential growth. In the exponential growth phase when the cells are growing at their maximum specific growth rate and the changes in the extracellular conditions are still insignificant a quasi-steady state can be assumed [26,19]. Precultivations were performed to determine the exponential growth phases and the maximum specific growth rates for the *T. reesei *wild type and Δ*cre1 *strains (data not shown). Cultures with different growth profiles were then sampled for quantitative flux analysis at equivalent growth stages, in the exponential phase. The maximum specific growth rates of *T. reesei *on glucose were 0.15 ± 0.01 h^-1 ^and 0.12 ± 0.01 h^-1 ^for the wild type and Δ*cre1 *strains, respectively. When grown on sorbitol the maximum specific growth rates for the wild type and Δ*cre1 *strains were 0.03 ± 0.02 h^-1 ^and 0.06 ± 0.01 h^-1^, respectively. The maximum specific growth rates of *A. nidulans *wild type strain and that of a CreA deletion strain have been observed to be 0.25 h^-1 ^and 0.11 h^-1^, respectively, when grown on glucose [19].

### Reconstruction of pathways through the metabolic network leading to amino acid synthesis

In order to quantify the in vivo flux ratios in the central carbon metabolism of *T. reesei *by ^13^C-labelling and METAFoR approach [22], it was necessary to obtain a model of the amino acid biosynthesis pathways. However, no curated metabolic model exists for *T. reesei*. Thus, the pathways for synthesis of the carbon backbones of the proteinogenic amino acids from the carbon source molecules in *T. reesei *were reconstructed by *ReTrace *pathway analysis [21]. ReTrace analysis results are summarized in Table 1 and fully reported in Additional File 1.

**Table 1:** Summary of the ReTrace [21] analysis of the amino acid biosynthetic routes in *T. reesei*.

**Amino acid**	**Precursors**	**Paths**	**Zo**	**AvgSc**	**BestSize**	**AvgSize**	**MinPoor**
Ala	Pyr	227	1	652	1	16.8	0

Arg	Oga	134	1	811	9	15.1	0

Asp	Oaa	121	1	757	1	13.5	0

Glu	Oga	36	1	426	1	12.6	0

Gly	Ser	260	1	1128	1	16.1	0

Gly	Thr	71	1	522	2	12.6	0

His	R5P	21	1	774	22	25.8	0

Ile	OAA, Pyr	483	1	797	14	18.7	0

Leu	AcCoA, Pyr	916	1	356	13	19.1	1

Lys	AcCoA, Oga	347	0.67	834	11	14.6	0

Phe	E4P, PEP	348	1	679	12	19.2	0

Pro	Oga	119	1	673	3	14.4	0

Ser	3PG	69	1	670	3	15.3	0

Thr	Oaa	97	1	768	7	2.5	0

Tyr	E4P, PEP	156	1	654	19	19.6	0

Paths paths found, Zo highest fraction of transferred atoms, AvgSc average reaction scores, BestSize the size of the pathway achieving Zo reported, AvgSize average pathway size, MinPoor minimum number of reactions with a score under 50

**Figure 1:**
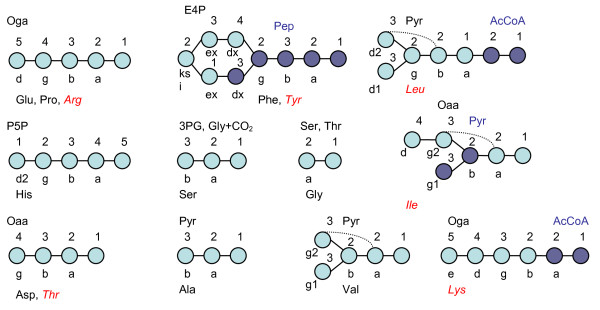
**Origins of proteinogenic amino acids**. The origins of the carbon backbones of the proteinogenic amino acids utilized in METAFoR analysis [26] and for which the biosynthetic pathways were reconstructed by computational pathway analysis method ReTrace [21]. The amino acids for which the biosynthetic pathway was not directly found by ReTrace are denoted in red italics. The amino acid carbons are denoted in the following way: a = α, b = β, g = γ, d = δ, e = ε, ksi = ξ.

ReTrace analysis confirmed, for most of the proteinogenic amino acids, that the biosynthetic pathways of amino acids identical to the pathways in *S. cerevisiae *are present also in *T. reesei*. Therefore the carbon backbones of the proteinogenic amino acids in *T. reesei *evidently originate from the precursor metabolites similar to the ones in *S. cerevisiae *[26] (Figure 1). For some proteinogenic amino acids (Arg, Ile, Leu, Thr, Tyr) ReTrace was not directly able to identify the biosynthetic routes that are active in *S. cerevisiae *because alternative reactions with higher scores strongly directed the search or because of errors in the atom mapping in the KEGG reaction database. However, the manual inspection of all the pathways identified directly from the carbon source or from different precursors, confirmed that the biosynthetic pathways for all proteinogenic amino acids relevant for METAFoR analysis, except for Arg and Lys, that are known to operate in *S. cerevisiae*, are also present in *T. reesei*.

The fungal biosynthetic pathway of Lys from Oga [29] was not found by ReTrace because of inconsistencies in the atom mapping in the KEGG reaction database. However, because the reactions of the alternative biosynthetic route of Lys which is active, for example in bacteria, did not gain good scores for presence in *T. reesei*, the fungal pathway was assumed prior to the ^13^C-pathway analysis. Pathways from Oga to Arg were identified by ReTrace but the pathway known to be active in *S. cerevisiae *was not found among them. Most of the identified pathways were directed through 1-pyrroline-5-carboxylate dehydrogenase reaction (1.5.1.12) in the reverse direction, which forms a false shortcut path between Oga and Arg.

After the unsuccessful direct search of pathway from Oaa to Thr, Thr biosynthesis pathway was searched from Asp, an intermediate in the pathway from Oaa to Thr. Genome level evidence of the presence of the pathway was found. The biosynthetic pathway of Ile that is active in *S. cerevisiae *was not found directly from precursors Oaa and Pyr because the pathway proceeds first from Oaa to Thr and that pathway was not directly identified as discussed above. The reactions further from Thr were identified with high scores for genome level evidence of their presence in *T. reesei *and thus, the pathway that is known to be active in *S. cerevisiae *is evidently present also in *T. reesei*. Tyr biosynthesis pathway was found from precursors downstream to 3-(4-hydroxyphenyl)pyruvate and only the transamination was lacking from a complete pathway. However, a high scoring hit for a transaminase sequence was separately searched and identified in the genome of *T. reesei*. Most of the high scoring alternative pathways could be excluded because only the anabolic pathways are active in the exponential growth and in absence of amino acids in the medium.

### ^13^C-pathway analysis and prediction of subcellular localization of key enzymes

**Figure 2:**
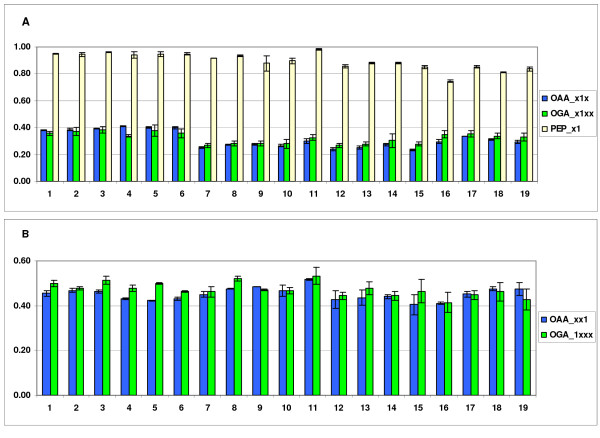
**Comparison of the fractions of corresponding intact bonds in amino acid precursors Oaa, Oga and Pep in *T. reesei***. The data is taken from all replicates of fractional [U-^13^C]glucose or [U-^13^C]sorbitol experiments performed (1-3 wild type on glucose, 4-6 Δ*cre1 *on glucose, 7-9 wild type on sorbitol, 10-12 wild type on sorbitol (sophorose experiment control), 13-15 wild type on sorbitol (sophorose induction), 16-17 Δ*cre1 *on sorbitol (sophorose experiment control), 18-19 Δ*cre1 *on sorbitol (sophorose induction)). Oaa data was detected from Asp-Cα, -Cβ and Thr-Cα, Oga data from Glu-Cα, -Cβ and Pro-Cα, -Cβ and Pep data from Phe and Tyr-Cα. Fractions of intact bonds in Oaa, Oga and Pep were calculated from combinations of fragmentomers. A) OAA_x1x is the fraction of Oaa molecules with an intact bond at C2-C3, OGA_x1xx is the fraction of Oga molecules with an intact bond at C2-C3 and PEP_x1 is the fraction of Pep molecules with an intact bond at C2-C3. B) OAA_xx1 is the fraction of Oaa molecules with an intact bond at C3-C4 and OGA_1xxx is the fraction of Oga molecules with an intact bond at C1-C2. Error bars are ± SEMs. The carbon chain of Oaa_mit _remains intact in the TCA cycle except that C1 is cleaved in the synthesis of Oga. Almost the entire labeling pattern of Oaa_mit _can be assessed from the labeling pattern determined for Oga. If Asp and Thr synthesis originates from Oaa_mit_, the fractions of corresponding Oaa and Oga intact fragments in the figures should match.

The pathways of amino acid biosynthesis reconstructed in *T. reesei *corresponded to the pathways utilized by *S. cerevisiae*. The fragmentomer data from ^13^C-labeling of proteinogenic amino acids provided further confirmation for this (see Methods for the definition of fragmentomer data). The ^13^C-labeling patterns of the carbon backbones of proteinogenic amino acids originate from the ^13^C-labeling of their precursor metabolites in central carbon metabolism and thus, the ^13^C-labeling patterns of amino acids can be propagated to the precursors to identify the active pathways. In particular, the Lys ^13^C-labeling pattern indicated its synthesis from Oga via α-aminoadipate pathway, as in yeasts [30]. However, in contrast to *S. cerevisiae *and a number of other yeast [26,31], the ^13^C-labeling pattern of Asp indicated that it primarily originated from mitochondrial Oaa under all the studied conditions (Figure 2). Mitochondrial Asp synthesis has previously been observed in *Yarrowia lipolytica *[31]. Furthermore, identical ^13^C-labeling patterns were observed in Asp and Thr. This confirmed Thr synthesis from Asp and excluded a contribution from the reversible threonine aldolase reaction [32].

A three carbon fragment of mitochondrial Oaa (Oaa_mit_) (C2-C3-C4) remains intact in the synthesis of the TCA cycle intermediate Oga and therefore the ^13^C-labeling pattern of Oaa_mit _can be partially observed in Glu that originates from Oga. In the exponential growth phase it is reasonable to assume unidirectional transport of Oaa across the mitochondrial membrane, which has previously been experimentally shown in *S. cerevisiae *[26]. When the backward transport from Oaa_mit _to Oaa_cyt _is negligible, a three-carbon fragment of Oaa_cyt _(C1-C2-C3) is produced from Pep, a precursor of Phe and Tyr, via glycolysis and by pyruvate carboxylase. The fractions of intact Cα-Cβ bonds in Asp and Thr were highly similar to the corresponding intact carbon fragments in Oga, propagated from Glu, but clearly different from the corresponding intact carbon fragments in Pep, propagated from Phe, Tyr in *T. reesei*, indicating the primarily mitochondrial origin of Asp. Since the C3-C4 bond of Oaa_mit _remains intact in the TCA cycle, Oaa_mit _serving as a precursor for Asp and Thr biosynthesis was further supported by the high similarity in the fraction of molecules having the corresponding C-C fragment intact, i.e. the C3-C4 fragment of Oaa, propagated from Asp, Thr, and C1-C2 fragment of Oga, propagated from Glu, (OAA_xx1 and OGA_1xxx, respectively, Figure 2).

Additional support for mitochondrial Asp synthesis was obtained from sequence analysis. Evidence of mitochondrial targeting peptide sequence was identified in one of the *T. reesei *genome sequences with homology to the aspartate aminotransferases in *S. cerevisiae *by TargetP analysis [33,34] (Additional file 2). This strongly supported the mitochondrial localization of one of the encoded enzymes. However, no evidence of mitochondrial targeting peptide was identified by TargetP analysis of the *T. reesei *sequence with homology to the *S. cerevisiae *pyruvate carboxylase that produces Oaa. Thus, Oaa_mit _could originate both from transport across the mitochondrial membrane and from the TCA cycle.

Pyruvate is a precursor of Ala and Val biosynthesis. If the pyruvate pools in cytosol and mitochondria possess significantly different ^13^C-labeling patterns, for example as a result of malic enzyme flux, a mitochondrial localization of pyruvate-based amino acid synthesis can be confirmed from the ^13^C-labeling data [31]. However, the fractions of intact two-carbon fragments Pyr C1-C2 and C2-C3 observed in Ala and Val and the corresponding two-carbon fragments in Pep, a direct precursor of Pyr_cyt_, observed in Phe and Tyr, were not significantly different under the studied conditions. Therefore, the ^13^C-labeling patterns could not be utilized to assess the localization of the synthesis of pyruvate-based amino acids. Strong evidence of a mitochondrial targeting sequence in the *T. reesei *sequence that showed homology to the acetolactate synthase in *S. cerevisiae *was identified by TargetP [33,34] (Additional file 2). In yeast the first enzyme in Val biosynthesis, the acetolactate synthase, has been reported to be localized in mitochondria [35], whereas cytosolic and mitochondrial isoenzymes of alanine aminotransferase have been observed [30].

**Figure 3:**
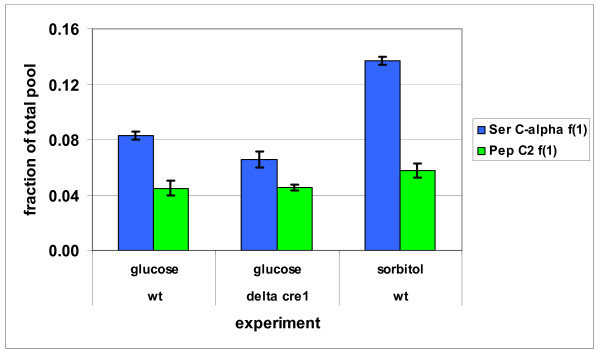
**Effect of the reversible glycine cleavage pathway**. Effect of the reversible glycine cleavage pathway in *T. reesei *wild type (wt) and Δ*cre1 *strains on the ^13^C-labeling pattern of Ser. The fraction of Ser-Cα *f(1) *fragmentomer from the total pool of Ser, compared to the corresponding fraction in Pep (C2) observed from Phe and Tyr-Cα *f(1) *fragmentomers. Error bars are ± SEMs.

Ser originates from glycolytic intermediate 3-phosphoglycerate and can be further converted to Gly and a C1 unit by the reversible reaction of serine hydroxymethyl transferase (SHMT). Gly could also originate from threonine aldolase or from the reversible reaction of the glycine cleavage pathway (i.e., C1 + CO_2_). In *S. cerevisiae *glycine cleavage pathway is active inside mitochondria [36] and although both mitochondrial and cytosolic isoenzymes of SHMT exist in *S. cerevisiae *[37,38], the effect of the glycine cleavage pathway on the Ser-Cα *f(1) *fraction has not been observed in *S. cerevisiae *batch cultures grown on glucose [26]. In *T. reesei *the activity of the glycine cleavage pathway was observed in the ^13^C-labeling pattern of Ser, since Ser-Cα *f(1) *fragmentomer fraction of Ser molecules with both carbon bonds cleaved was higher than the fraction of fully cleaved Pep, a three carbon lower glycolytic intermediate. The fraction of fully cleaved Pep molecules was observed in Phe and Tyr-Cα *f(1) *fragmentomer fractions (Figure 3). Two *T. reesei *sequences were observed to have homology to the *S. cerevisiae *SHMT sequences. In one of them a strong evidence of a mitochondrial targeting pre-sequence was found by TargetP [33,34] (Additional file 2). Therefore, SHMT activity likely occurs in both cytosolic and mitochondrial compartments of *T. reesei*. The Ser ^13^C-labeling pattern observed in *T. reesei *indicates either a partially cytosolic localization of the glycine cleavage pathway or protein synthesis occurring primarily from a mitochondrial pool of Ser. TargetP analysis of the *T. reesei *sequence homological to sequence of the *S. cerevisiae *glycine dehydrogenase, the p-subunit of the Gly cleavage system, showed no clear indication of a mitochondrial targeting pre-sequence [33,34] (Additional file 2).

Amino acids belonging to the Glu amino acid family, Glu, Pro and Arg, showed a highly similar ^13^C-labeling, as expected, in both strains grown on glucose and in the wild type strain culture grown on sorbitol (data not shown). In contrast, a significant variation was observed in the Glu, Pro and Arg fragmentomers in cultures when their pre-cultures were mixed before sophorose induction experiment. This observation may be explained by differential mobilization of amino acids from cellular compartments resulting from the perturbation when the cultures were mixed prior to the induction period.

### METAFoR analysis of *T. reesei*

The relative fluxes merging at the branching points of central carbon metabolism of *T. reesei *were determined by BDF ^13^C-labeling with glucose and sugar alcohol sorbitol as carbon sources. The flux ratios of the wild type and Δ*cre1 T. reesei *strains determined in batch cultures on glucose, on sorbitol and on sorbitol with sophorose induction of expression of cellulase genes are shown in Table 2.

**Table 2:** Metabolic flux ratios of *T. reesei *wild type (wt) and Δ*cre1 *strains in aerobic batch cultures on glucose and on sorbitol with and without sophorose induction of cellulase gene expression.

**strain**	**wt**		**Δ*cre1***		**wt**		**wt**		**Δ*cre1***		**Δ*cre1***	
**carbon source**	**glucose**		**glucose**		**sorbitol**		**sorbitol**		**sorbitol**		**sorbitol**	

fraction of total pool (%)		sd		sd		sd	sophorose	sd	control	sd	sophorose	sd

Pep from PPP (UB wo PEPck)	39	2	47	4	36	7	37	7	45	9	46	2

R5P from T3P and S7P	51	1	42	1	72	3	70	1	79	4	79	1

R5P from E4P	25	2	23	1	46	2	48	5	54	3	44	2

Ser from Gly and C1	80	0	85	2	44	2	41	15	54	1	52	0

Gly from CO_2 _and C1	12	1	14	1	6	2	16	21	8	1	10	3

Oaa_mit _from Pep	35	1	33	2	26	3	26	4	42	5	39	7

MAE (UB)	4	0	9	1	12	2	2	2	6	5	1	nd

MAE (LB)	2	0	6	1	9	1	2	2	4	3	0	nd

sd standard deviation, UB upper bound, LB lower bound

### Respiratory pathway flux of *T. reesei*

The relative anaplerotic flux (the fraction of OAA_mit _from Pep, Table 2) describes the relative activities of the biosynthetic and respirative carbon fluxes in the TCA cycle. The anaplerotic flux replenishes carbons to the TCA cycle flux by importing C4 compounds as there is drain of carbon to biosynthesis. David *et al *concluded that the TCA cycle was more active in Δ*creA *mutants of *A. nidulans *than in the wild type when grown on glucose [19]. However, they suggested that it resulted from a higher ATP demand in the deletion strain possibly caused by active futile cycles instead of derepression of the respirative pathway flux. No significant difference was observed in the anaplerotic flux ratios between the *T. reesei *wild type and Δ*cre1 *strains grown on glucose. Thus, in *T. reesei *the TCA cycle was as active relative to biosynthesis in the Δ*cre1 *strain as in the wild type strain and so, Cre1 does not mediate repression of respirative pathway flux in *T. reesei *either.

A difference was observed between the two *T. reesei *strains when grown on sorbitol. The relative anaplerotic flux was 26% in the wild type strain and 42% in the Δ*cre1 *strain (Table 2). This may indicate that there was a difference in the specific growth rates of the two strains on sorbitol.

Previously, an excess of glucose has been found to only partially repress the gene expression of the enzymes of the TCA cycle and the components of the respiratory chain in *T. reesei *[17]. That is in contrast to the effect of excess of glucose on *S. cerevisiae*, where glucose repression extensively downregulates the respiratory pathway at the transcriptional level [18]. The anaplerotic flux ratio in *T. reesei *wild type strain was higher on glucose (35%) than that on sorbitol (26%) (Table 2). The results indicated a higher activity of respiratory metabolism relative to biosynthesis on the non-repressing carbon source sorbitol than that on the repressing carbon source glucose. A complete oxidation of sorbitol, that is a more reduced carbon source than glucose, results in a higher relative flux of electrons per carbon source molecule to the respiratory chain than during growth on glucose. Thus, if *T. reesei *respired at maximum rate during the batch growth on glucose, fluxes producing reduced cofactors, for example biosynthetic pathway fluxes or the TCA cycle fluxes, would have decreased on sorbitol.

Small fractions of Pyr_mit _originating from malate via the action of the malic enzyme were observed in both strains under almost all conditions (Table 2).

### Pentose phosphate pathway (PPP) of *T. reesei*

A lower fraction of triose phosphates originated from pentose phosphates in the wild type strain (39%) than in the Δ*cre1 *strain (47%) when grown on glucose (Table 2). In batch cultures under excess glucose conditions, the gluconeogenesis by phosphoenolpyruvate carboxykinase and the reverse transport of Oaa across the mitochondrial membrane are assumed to have negligible fluxes [18,26]. For this purpose the fraction of Pep originating from the pentose phosphate pathway (PPP) was calculated neglecting any contribution of phosphoenolpyruvate carboxykinase to the ^13^C-labeling pattern of Pep, propagated from Phe, Tyr. The fraction of Pep originating from PPP represents the flux via PPP relative to the total flux to Pep. However, this fraction is not a direct measure of the flux through the oxidative branch of the PPP but includes molecules that have only gone through reversible reactions in the non-oxidative PPP. Furthermore, the standard deviation is always high because only 40% of the triose phosphates that originate from the PPP have different ^13^C-labeling patterns than the triose phosphates originating from glycolysis.

The differences in the relative flux through the PPP to the triose phosphates can be caused by differences in the glycolytic rate or in NADPH demands, since the oxidative branch of the PPP is usually the main source of cytosolic NADPH. A low glycolytic rate could allow the label scrambling in the non-oxidative part of the PPP to affect the ^13^C-labeling patterns of a large fraction of triose phosphates.

The reversible fluxes through the reactions of transketolase and transaldolase, observed in the ^13^C-labeling patterns of pentose phosphates that can be detected in His, were clearly different in glucose and sorbitol cultivations (Table 2). The fraction of pentose phosphates that had gone through a transketolase reaction (R5P from T3P and S7P) was 51% and 42% when glucose was the carbon source for the wild type and the Δ*cre1 *strains, respectively. When grown on sorbitol the fractions were higher, 72% and 79% for the wild type and the Δ*cre1 *strains, respectively. The fraction of pentose phosphates cleaved in the transaldolase and transketolase reactions (R5P from E4P) was 25% and 23% when grown on glucose, whereas when sorbitol was the carbon source they were 46% and 54% for the wild type and the Δ*cre1 *strains, respectively. The higher fractions of pentose phosphates cleaved in the reactions of transketolase or transaldolase when grown on sorbitol could be a result of entrance of sorbitol in the central carbon metabolism and into the PPP directly in a form of fructose 6-phosphate [10].

**Figure 4:**
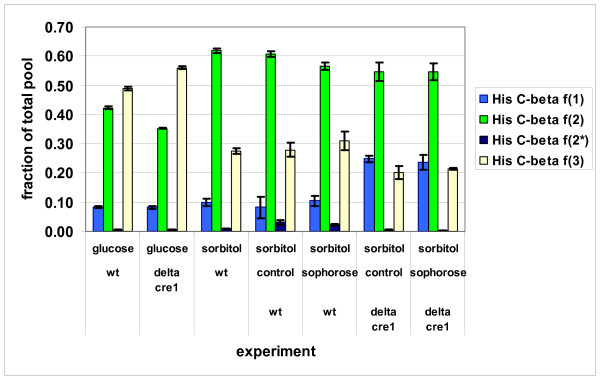
**^13^C-His-Cβ centered contiguous ^13^C-fragments in *T. reesei***. Fractions of ^13^C-His-Cβ centered contiguous ^13^C-fragments in *T. reesei *in different genetic, wild type (wt) and Δ*cre1 *mutant. Cultures were grown on glucose (glucose repressed) or sorbitol (derepressed). Sorbitol grown cultures were grown with or without induction of cellulase gene expression by the addition of sophorose to some cultures. His-Cβ *f*(1) denotes fragments with C-C bonds cleaved on both sides of Cβ, His-Cβ *f*(2) and *f*(2*) denote fragments with Cβ-Cα and Cβ-Cγ preserved, respectively, and His- Cβ *f*(3) denotes fragments were both bonds are intact. Error bars represent ± SEM.

Figure 4 shows the relative abundances of the contiguous ^13^C-fragments around His-Cβ, which originate from fragments around ribose 5-phosphate C3. When sorbitol was the carbon source lower fractions of fully intact His fragments and higher fractions of His fragments cleaved in the reversible reactions of transaldolase and transketolase were observed in both strains than when grown on glucose. This indicated higher relative fluxes in the non-oxidative part of the PPP when compared to the rate of withdrawal of pentose phosphates to His biosynthesis. When sorbitol was the carbon source the relative activity of the non-oxidative PPP compared to the rate of biosynthetic drain of pentose phosphates was even higher in the Δ*cre1 *strain than in the wild type strain. The fraction of fully cleaved His-Cβ *f*(1) fragments was higher in the Δ*cre1 *strain than in the wild type strain when they were grown on sorbitol (Figure 4). Correspondingly, lower fractions of fully intact His-Cβ *f*(3) were observed in the Δ*cre1 *strain than in the wild type strain.

### Effect of sophorose induction of cellulase gene expression on metabolic fluxes

Induction of cellulase gene expression with sophorose did not cause any significant changes in the metabolic flux distributions in the central carbon metabolism of *T. reesei*. Therefore, the induction of cellulase gene expression did not affect the relative fluxes to different amino acid families or the ratio of anabolic and catabolic activity of the central carbon metabolism. Alteration in the relative biosynthetic rates of different amino acids would have occurred if the amino acid composition of the induced cellulases had been significantly different from the amino acid composition of the proteins generally produced by *T. reesei *which was not observed.

### Flux ratio profiles of *T. reesei*, *S. cerevisiae *and *Pichia stipitis *indicate differences in preferred utilization of pathways

The anaplerotic flux ratios determined hereby in the wild type and Δ*cre1 T. reesei *strains in batch cultures, both grown on glucose, were substantially lower and similar to what has been observed in fully respiratory metabolism in *S. cerevisiae *in glucose-limited chemostat cultures, where there is no glucose repression [27] (Table 3). The extensive glucose repression of the TCA cycle and the respiratory pathway activity in *S. cerevisiae *result in high anaplerotic ratio in batch cultures on glucose [26]. The anaplerotic flux ratios in the *T. reesei *strains with glucose as a carbon source were also similar to the ones observed in *P. stipitis*, both when grown on glucose in batch cultures and in glucose-limited chemostat cultures [27]. *P. stipitis *completely lacks aerobic alcoholic fermentation.

**Table 3:** Metabolic flux ratios of *T. reesei *wild type (wt) and Δ*cre1 *strains in compared with the corresponding flux ratios observed in the crabtree positive and negative yeasts *S. cerevisiae *and *P. stipitis *[26,27].

**organism**	***T. reesei***		***T. reesei***		***T. reesei***		***T. reesei***		***S. cerevisiae***		***S. cerevisiae***		***P.stipitis***		***P.stipitis***	
**strain**	**wt**		**Δ*cre1***		**wt**		**Δ*cre1***									

carbon source	glucose		glucose		sorbitol		sorbitol		glucose		glucose		glucose		glucose	

culture	batch		batch		batch		batch		batch		chemostat		batch		chemostat	

reference							control		[26]		[27]		[27]		[27]	

fraction of total pool (%)		sd		sd		sd		sd		sd		sd		sd		sd

Pep from PPP (UB wo PEPck)	39	2	47	4	36	7	45	9	0-4		40	8	57	9	61	11

R5P from T3P and S7P	51	1	42	1	72	3	79	4	68	2	59	2	57	2	72	2

R5P from E4P	25	2	23	1	46	2	54	3	10	2	33	2	35	2	43	2

Oaa_mit _from Pep	35	1	33	2	26	3	42	5	76	4	31	2	36	2	32	2

MAE (UB)	4	0	9	1	12	2	6	5	25-30		<13		<6		<7	

MAE (LB)	2	0	6	1	9	1	4	3	nd	nd	nd	nd	nd	nd	nd	nd

It has previously been determined that glucose does not cause extensive repression of the gene expression of the TCA cycle and the respiratory pathway components in *T. reesei *[17] as it does in *S. cerevisiae *[18]. The ^13^C-labeling and METAFoR analysis results on the level of *in vivo *fluxes confirmed that for highly efficient energy generation through complete oxidation of carbon source *T. reesei *indeed uses primarily the respirative pathway also when growing on a preferred carbon source glucose. The regulatory differences between *T. reesei *and *S. cerevisiae *have been explained as adaptation to different growth environments. *S. cerevisiae *is adapted to nutrient rich environments in which it has competitive advantage from fast nutrient utilization and a high rate of ATP production through the fermentative pathway, whereas *T. reesei *is adapted to nutrient poor environments where it benefits from high energy yield [17,16]. It has also been postulated that undifferentiated multicellular organisms, of which *T. reesei *is an example, have gotten evolutionary advantage from preferring the high energy yield from respiratory metabolism [16].

## Conclusion

Biosynthetic pathways of *T. reesei *were reconstructed for most of the proteinogenic amino acids by using a computational carbon path analysis method ReTrace. The method was used to search for pathways from a metabolic network consisting of all reactions found in a comprehensive metabolic reaction database, and to subsequently rank the pathways according to the degree of support from the *T. reesei*'s genome [21]. Contiguous pathways, identical to the amino acid biosynthetic routes of *S. cerevisiae*, were found with high genome-level evidence. The ^13^C-labeling patterns observed in this study were in good accordance with the compartmentalized model of eukaryotic central carbon metabolism, originally developed for *S. cerevisiae *[26]. However, in contrast to *S. cerevisiae*, Asp synthesis was observed to occur primarily from the mitochondrial pool of Oaa in both *T. reesei *strains under all the studied conditions.

The *T. reesei *wild type strain is known to exhibit carbon catabolite repression of hydrolytic gene expression during growth on glucose, whereas in the Δ*cre1 *strain the repression is partially disturbed [13]. The respirative pathway in *T. reesei *does not become transcriptionally downregulated by the carbon catabolite repression as in *S. cerevisiae *[17]. However, it is the *in vivo *fluxes that are the ultimate phenotype of an organism. In the present work, the effect of carbon catabolite repression on *in vivo *fluxes in *T. reesei *was, for the first time, quantitatively studied. The relative anaplerotic flux to the respirative pathway flux was characteristic to primarily respiratory metabolism in the both *T. reesei *strains under all studied conditions. Thus, *T. reesei *utilizes primarily respiratory metabolism also when growing on a preferred carbon source glucose. However, the observed relative anaplerotic fluxes suggested that the respirative activity of the TCA cycle is even slightly higher when *T. reesei *grows on the neutral carbon source sorbitol than when it grows on glucose. Only minor differences were observed between the *in vivo *flux distributions of the wild type and the Δ*cre1 T. reesei *strains. This indicates, that Cre1, the key repressor of utilization of alternative carbon sources, does not mediate carbon source dependent metabolic state alterations in the central carbon metabolism of *T. reesei*. The induction of cellulase gene expression with sophorose did not result in significant changes in the relative requirements of proteinogenic amino acids or in the ratio of anabolic and oxidative activities of the TCA cycle.

## Methods

### Strains, media and culture conditions

Biosynthetically directed fractional (BDF) ^13^C-labeling of proteins was carried out for the *T. reesei *QM6a (wild type) [39] and *T. reesei *QM6a with deleted *cre1 *gene (unpublished). Both strains were cultivated in triplicate on two different carbon sources: glucose and sorbitol. Glucose cultivations were carried out in 2 l flasks in 200 ml of minimal medium ((NH_4_)_2_SO_4 _7.6 g/l, KH_2_PO_4 _15.0 g/l, 2.4 mM MgSO_4_, 4.1 mM CaCl_2_, CoCl_2 _3.7 mg/l, FeSO_4_·7H_2_O 5 mg/l, ZnSO_4_·7H_2_O 1.4 mg/l, MnSO_4_·7H_2_O 1.6 mg/l, pH adjusted to 4.8 with KOH) supplemented with 2% (w/v) glucose containing 10% (w/w) [U-^13^C]glucose.

**Figure 5:**
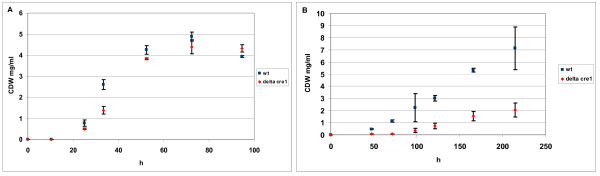
***T. reesei *growth curves**. Growth curves of *T. reesei *wild type (wt) and *Δcre1 *strains (A) on glucose and (B) on sorbitol. Error bars are standard deviations of three replicates.

The 200 ml cultures were inoculated with 8 × 10^7 ^spores and cultivated at +28°C with constant agitation at 250 rpm. After 35 h of cultivation, during the exponential growth phase (Figure 5), 30 ml and 50 ml samples were withdrawn for dry weight determination and for NMR experiments, respectively. Mycelium from the samples was collected by filtration through Whatmann GF/B filters and washed twice with the sampling volume of water. For dry weight determination the mycelium was dried in an oven at +106°C overnight and weighed.

The BDF ^13^C-labeling of the wild type strain on sorbitol was carried out in three replicates with 2% (w/v) sorbitol containing10% (w/w) [U-^13^C]sorbitol, similarly as in the glucose cultivations. After 104 h of incubation, in the exponential growth phase (Figure 5), 30 ml and 50 ml samples were withdrawn for dry weight determination and for NMR experiments, respectively.

BDF ^13^C-labeling on sorbitol was also carried out with induction of cellulase gene expression by sophorose. Six 2 l flasks of each strain were inoculated, with 2% (w/v) sorbitol as the sole carbon source in minimal medium (see above). After 76 h for the wild type and after 114 h for the Δ*cre1 *mutant, in exponential growth phase (Figure 5), the six cultures were combined, a 30 ml sample for dry weight determination was withdrawn and then the culture broth was redivided into six flasks. The final concentration of 1 mM sophorose was introduced into three of the six replicate 2 l flasks to induce cellulase gene expression. An identical volume of water was added to the three control cultures. Three hours after the induction, when cellulase gene expression was expected to be at a moderate level [6], 0.4 g of [U-^13^C]sorbitol was added to all six cultures to initiate BDF ^13^C-labeling. The addition of 0.4 g of [U-^13^C]sorbitol at this time was estimated to result in a [U-^13^C]sorbitol fraction of about 10% of the total sorbitol in the culture medium. After 24 h from the addition of the [U-^13^C]sorbitol, still during the early-exponential growth phase (Figure 5), 30 ml and 50 ml samples were withdrawn for dry weight determination and NMR experiments, respectively. Thereby the ^13^C-labeled fraction of biomass was synthesized in the induced conditions and the information of the pathways that were active when the cellulase gene expression was induced was recorded in the labelling patterns of proteinogenic amino acids.

### Nuclear Magnetic Resonance (NMR) spectroscopy experiments

The filtered mycelial samples were suspended into 10 ml of 6 M HCl and the biomass was hydrolysed in sealed glass tubes at +110°C for 22 h. The suspensions were dried and dissolved in H_2_O for filtration through 0.2 μm filters. The filtrates were vacuum-dried and dissolved in D_2_O for NMR experiments. The pH of the samples was below 1 due to residual HCl.

^13^C-HSQC NMR spectra were acquired at +40°C on a Varian Inova spectrometer operating at a ^1^H-resonance frequency of 600 MHz essentially as described [22]. For each sample two spectra were acquired focusing on the aliphatic and aromatic regions. For the aliphatic spectra, a matrix of 1024 × 1500 (f2 × f1) complex data points was acquired and zero-filled to 4096 complex data points in f1. The spectral widths were 6000 Hz and 5100 Hz in the ^1^H- and ^13^C-dimensions, respectively. The narrow spectral width in the ^13^C-dimension leads back-folding of part of the signals to the empty regions of the spectrum. For the aromatic region, a matrix of 1024 × 800 complex data points was acquired and zero-filled to 2048 complex data points in f1. The spectral widths for the aromatic spectra were 6000 Hz and 2815 Hz in the ^1^H- and ^13^C-dimensions, respectively. All spectra were weighted with a cosine function in both dimensions prior to the Fourier transformation. The spectra were processed using the standard Varian spectrometer software VNMR (version 6.1, C).

### Metabolic Flux Ratio (METAFoR) analysis

The software FCAL (R.W. Glaser; FCAL 2.3.1) [25] was used for the integration of ^13^C-scalar fine structures of proteinogenic amino acid carbon signals in the ^13^C-HSQC NMR spectra and the calculation of relative abundances of intact carbon fragments originating from a single source molecule of glucose. The nomenclature used here for the intact carbon fragments, fragmentomers, has been described previously [22]. Briefly, *f*^(1) ^represents the fraction of molecules in which the observed carbon atom and the neighboring carbons originate from different source molecules of glucose, *f*^(2) ^the fraction of molecules in which the observed carbon atom and one of the two neighboring atoms originate from the same source molecule of glucose, and *f*^(3) ^the fraction of molecules in which the observed carbon atom and both neighboring carbons originate from the same source molecule of glucose. If the observed carbon exhibits significantly different ^13^C-^13^C scalar coupling constants with the neighboring carbons, *f*^(2) ^and *f*^(2*) ^can be distinguished. The fraction of molecules with a conserved bond between the observed carbon atom and the neighboring carbon with the smaller coupling is represented by *f*^(2)^. *f*^(2*) ^then denotes the fraction of molecules where the bond is conserved between the observed carbon and the neighboring carbon with the larger coupling. If the observed carbon is located at the end of a carbon chain, *f*^(1) ^and *f*^(2) ^fragmentomers can be observed indicating the conservation of the two terminal carbon fragment of the molecule.

**Figure 6:**
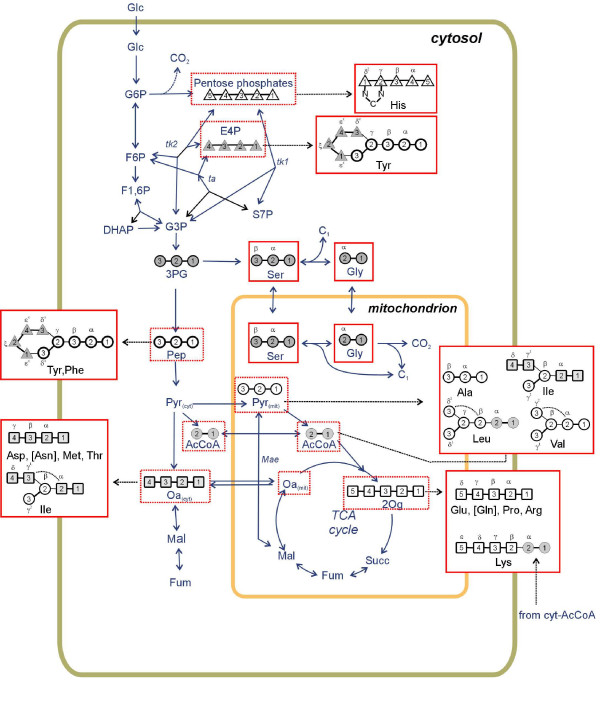
**Metabolic network model**. Eukaryotic central carbon metabolism network model [26].

The degree of^13^C-labeling of the biomass amino acids was determined from the ^13^C-scalar fine structures of Leu-Cβ and Val-Cγ_2_. The biomass was assumed to be fully produced from the fractionally labelled carbon source in the glucose experiments and in sorbitol experiments without sophorose induction because the dry weight of the inoculum was negligible. For the sorbitol experiments with sophorose induction the fraction of labeled biomass was estimated from the dry weight measurement (data not shown). The model of the central carbon metabolism network used in the METAFoR analysis was the one previously developed for eukaryotic metabolism of the yeast *S. cerevisiae *[26] (Figure 6). Fragmentomer information obtained from proteinogenic amino acids can be traced back to the metabolic intermediates in central carbon metabolism through the amino acid synthesis pathways to assess ratios of intracellular fluxes which merge at a metabolic network junction [26]. The biosynthetic pathways of amino acids in *T. reesei *were reconstructed with carbon path analysis method ReTrace [21] described in the next section.

### Carbon path analysis

The reconstruction of amino acid biosynthetic pathways from their precursors in *T. reesei *was performed with ReTrace. ReTrace is a recent computational pathway analysis method [21], which can be queried to discover branching metabolic pathways in a universal metabolic database. Specifically, ReTrace aims to find pathways which transfer as many atoms from source to target metabolites as possible.

The reaction database used in ReTrace analysis was KEGG LIGAND, downloaded in March 2009 [40]. Reaction database contained 7827 reactions and 15400 compounds. *Atom mappings*, that describe how atoms are transferred in a reaction from substrate to product metabolites, were defined for 33795 substrate-product pairs in the RPAIR database, which is a subdatabase of KEGG. All reactions were considered bidirectional. To compute reaction scores, a database consisting of 101136 sequences annotated with an EC number in UniProt version 9.3 [41] was queried with the 9129 protein sequences in *T. reesei *genome [20] by blastp [42] using e-value cutoff 10 to detect remote homologs. Each reaction in the KEGG database was assigned a score by taking the maximum BLAST score over all UniProt-*Trichoderma *sequence pairs, where UniProt sequence had been annotated with an EC number corresponding to the reaction. A total of 3974 reactions received a score in this procedure, while the remaining 3853 reactions were assigned a zero score. Reaction scores reflected the degree of evidence from the detection of sequence homology that there exists an enzyme catalyzing the reaction in *T. reesei*.

For a majority of pathway queries, maximum search depth was set to 3 and number of pathways searched at depths 1, 2 and 3 to 50, 10 and 1, respectively. In other words, ReTrace search comprised pathways with three branches or less. In particular, more alternative routes (k = 50) were searched at the initial first level (depth 1) than at subsequent levels to reduce the computational complexity. However, in queries involving Asp, Phe, Thr and Tyr, search time with these parameters exceeded a few hours due to branching. These queries were resolved by setting k = 1 already at the second level, while keeping k = 50 at the first level.

Typically, the queries took from 30 minutes to 2 hours CPU time each on computers running Intel Xeon X5355 CPUs. Queries were performed on a cluster of 10 CPUs with four cores each, reducing the total time required. Parameter choices affect the computation time significantly: although it is possible to obtain results on, for example, existence of complete pathways in a matter of seconds by setting k = 1 at each level, in this study a more exhaustive approach was adopted.

### Localization of amino acid biosynthetic enzymes in *T. reesei*

TargetP, a machine learning method based on neural networks, which predicts both chloroplast and mitochondrial targeting peptides and secretory signal peptides, was utilized to predict the probable subcellular localization of some amino acid biosynthetic enzymes in *T. reesei *[33,34]. The prediction performance of non-plant mitochondrial targeting peptides with TargetP has been measured to be 80 - 90% sensitivity and 70% specificity [33]. TargetP reported, for each analyzed peptide sequence, the probability that the peptide contained some signal peptide (SP), a mitochondrial targeting peptide (mTP) or cytosolic targeting peptide (cTP) presequence. In addition, a numerical reliability class (RC) between 1 and 5 was reported. The reliability class was derived from the difference of highest and second-highest probabilities assigned to the prediction classes SP, mTP, cTP or "other". The class "other" indicates the probability that no subcellular location sorting signal was found. If the difference was greater than 0.8, RC equals 1; if the difference was below 0.2, RC equals 5.

## Abbreviations

AcCoA: acetyl coenzyme A; Ala L: Alanine; Arg L: Arginine, Asp L: Aspartic acid; BDF: biosynthetically directed fractional; CCR: carbon catabolite repression: E4P: erythrose 4-phosphate; Glu L: Glutamic acid; Gly: Glycine; His L: Histidine; HSQC: heteronuclear single quantum correlation; Ile L: Isoleucine, Leu L: Leucine; Lys L: Lysine; Oaa: oxaloacetate; Oaa_cyt_: cytosolic oxaloacetate; Oaa_mit_: mitochondrial oxaloacetate; Oga: oxoglutarate; Pep: phosphoenol pyruvate: Phe L: Phenylalanine; PPP: pentose phosphate pathway; Pro L: Proline; Pyr: pyruvate; Pyr_cyt_: cytosolic pyruvate: Pyr_mit_: mitochondrial pyruvate; SEM: standard error of the mean: Ser L: Serine; Thr L: Threonine; Tyr L: Tyrosine.

## Authors' contributions

PJ participated in the design of the study, performed the cultivations, carried out the NMR experiments and performed the ^13^C-metabolic flux ratio analysis, EP performed the ReTrace carbon path analysis, PJ and EP interpreted the results of the computational pathway analysis and wrote the manuscript, HM participated in the design of the study and the NMR experiments, TP, MS and MP participated in the design of the study. All authors read and approved the final manuscript.

## Supplementary Material

Additional file 1 **Pathways discovered in ReTrace carbon path analysis**. Graphical and tabular representations of amino acid synthesis pathways discovered in ReTrace carbon path analysis [21]. Self-contained web site: unpack zip archive and open index.html with a web browser. Click here for file

Additional file 2 **Estimation of subcellular localization of key enzymes by TargetP**. TargetP machine learning program [33,34] was used to predict the subcellular localization of key enzymes of amino acid biosynthetic routes in *T. reesei*. Targeting sequences were predicted for *T. reesei *sequences that showed the highest homology to the aspartate aminotransferase, acetolactate synthase, serine hydroxymethyltransferase and glycine dehydrogenase in *S. cerevisiae*. Click here for file

## References

[B1] Kumar R, Singh S, Singh OV (2008). Bioconversion of lignocellulosic biomass: biochemical and molecular perspectives. J Ind Microbiol Biotechnol.

[B2] Ilmén M (1997). Molecular mechanisms of glucose repression in the filamentous fungus *Trichoderma reesei*. PhD thesis.

[B3] Aro N, Pakula T, Penttilä M (2005). Transcriptional regulation of plant cell wall degradation by filamentous fungi. FEMS Microbiol Rev.

[B4] Stricker AR, Mach RL, de Graaf LH (2008). Regulation of transcription of cellulases- and hemicellulases-encoding genes in *Aspergillus niger *and *Hypocrea jecorina *(*Trichoderma reesei*). Appl Microbiol Biotechnol.

[B5] Margolles-Clark M, Ilmén M, Penttilä M (1997). Expression patterns of ten hemicellulase genes from filamentous fungus *Trichoderma reesei *on various carbon sources. J Biotechnol.

[B6] Ilmén M, Saloheimo A, Onnela M-L, Penttilä ME (1997). Regulation of Cellulase Gene Expression in the Filamentous Fungus *Trichoderma reesei*. Appl Env Microbiol.

[B7] Druzhinina IS, Schmoll M, Seiboth B, Kubicek P (2006). Global Carbon Utilization Profiles of Wild-Type, Mutant, and Transformant Strains of *Hypcrea jecorina*. Appl Env Microbiol.

[B8] Richard P, Putkonen M, Väänänen R, Londesborough J, Penttilä M (2002). The missing link in the fungal L-arabinose catabolic pathway: identification of the L-xylulose reductase gene. Biochemistry.

[B9] Nogawa M, Goto M, Okada H, Morikawa Y (2001). L-sorbose induces cellulase gene transcription in the cellulolytic fungus *Trichoderma reesei*. Curr Genet.

[B10] Pail M, Peterbauer T, Seiboth B, Hametner C, Druzhinina I, Kubicek CP (2004). The metabolic role and evolution of L-arabinitol 4-dehydrogenase of *Hypocrea*. jecorina.

[B11] Bailey C, Arst HN (1975). Carbon catabolite repression in *Aspergillus nidulans*. Eur J Biochem.

[B12] Strauss J, Mach RL, Zeilinger S, Hartler G, Stöffler G, Wolschek M, Kubicek CP (1995). Cre1, the carbon catabolite repressor protein from *Trichoderma reesei*. FEBS Lett.

[B13] Ilmén M, Thrane C, Penttilä M (1996). The glucose repressor gene *cre1 *of *Trichoderma*: Isolation and expression of a full length and truncated mutant form. Mol Gen Genet.

[B14] Cziferszky A, Mach RL, Kubicek CP (2002). Phosphorylation Positively Regulates DNA Binding of the Carbon Catabolite Repressor Cre1 of *Hypocrea jecorina (Trichoderma reesei)*. J Biol Chem.

[B15] Vautard G, Cotton P, Fèvre M (1999). The glucose repressor CRE1 from *Sclerotina sclerotiorum *is functionally related to CREA from *Aspergillus nidulans *but not to the Mig proteins from *Saccharomyces cerevisiae*. FEBS Letters.

[B16] Pfeiffer T, Schuster S, Bonhoeffer S (2001). Cooperation and Competition in the Evolution of ATP-producing Pathways. Science.

[B17] Chambergo FS, Bonaccorsi ED, Ferreira AJS, Ramos ASP, Ribamar Ferreira Júnior J, Abrahao-Neto J, Farah JPS, El-Dorry H (2002). Elucidation of the Metabolic Fate of Glucose in the Filamentous Fungus *Trichoderma reesei *Using Expressed Sequence Tag (EST) Analysis and cDNA Microarrays. J Biol Chem.

[B18] Gancedo JM (1998). Yeast carbon catabolite repression. Microbiol Mol Biol Rev.

[B19] David H, Krogh AM, Roca C, Åkesson M, Nielsen J (2005). CreA influences the metabolic fluxes of *Aspergillus nidulans *during growth on glucose and xylose. Microbiology.

[B20] Martinez D, Berka RM, Henrissat B, Saloheimo M, Arvas M, Baker SE, Chapman J, Chertkov O, Coutinho PM, Cullen D, Danchin EGJ, Grigoriev IV, Harris P, Jackson M, Kubicek CP, Han CS, Ho I, Larrondo LF, de Leon AL, Magnuson JK, Merino S, Misra M, Nelson B, Putnam N, Robbertse B, Salamov AA, Schmoll M, Terry A, Thayer N, Westerholm-Parviainen A, Schoch CL, Yao J, Barbote R, Nelson MA, Detter C, Bruce D, Kuske CR, Xie G, Richardson P, Rokhsar DS, Lucas SM, Rubin EM, Dunn-Coleman N, Ward M, Brettin TS (2008). Genome sequencing and analysis of the biomass-degrading *Trichoderma reesei *(syn. *Hypocrea jecorina*). Nat Biotechnol.

[B21] Pitkänen E, Jouhten P, Rousu J (2009). Inferring branching pathways in genome-scale metabolic networks with ReTrace. BMC Syst Biol.

[B22] Szyperski T (1995). Biosynthetically directed fractional ^13^C-labelling of proteinogenic amino acids. An efficient tool to investigate intermediary metabolism. Eur J Biochem.

[B23] Sauer U, Hatzimanikatis V, Bailey JE, Hochuli M, Szyperski T, Wüthrich K (1997). Metabolic fluxes in riboflavin-producing *Bacillus subtilis*. Nat Biotechnol.

[B24] Sauer U, Lasko DR, Fiaux J, Hochuli M, Glaser R, Szyperski T, Wüthrich K, Bailey JE (1999). Metabolic flux ratio analysis of genetic and environmental modulations of *Escherichia coli *central carbon metabolism. J Bacteriol.

[B25] Szyperski T, Glaser RW, Hochuli M, Fiaux J, Sauer U, Bailey JE, Wüthrich K (1999). Bioreaction network topology and metabolic flux ratio analysis by biosynthetic fractional ^13^C labelling and two-dimensional NMR spectroscopy. Metab Eng.

[B26] Maaheimo H, Fiaux J, Ìakar PZ, Bailey JE, Sauer U, Szyperski T (2001). Central carbon metabolism of *Saccharomyces cerevisiae *explored by biosynthetic fractional ^13^C labelling of common amino acids. Eur J Biochem.

[B27] Fiaux J, Ìakar PZ, Sonderegger M, Wüthrich K, Szyperski T, Sauer U (2003). Metabolic-Flux Profiling of the Yeasts *Saccharomyces cerevisiae *and *Pichia stipitis*. Eukaryot Cell.

[B28] Sola A, Maaheimo H, Ylonen K, Ferrer P, Szyperski T (2004). Amino acid biosynthesis and metabolic flux profiling of *Pichia pastoris*. Eur J Biochem.

[B29] Xu H, Qian BAJ, West AH, Cook PF (2006). The α-Aminoadipate Pathway for Lysine Biosynthesis in Fungi. Cell Biochem Biophys.

[B30] Broquist HP (1971). Lysine biosynthesis (Yeast). Methods Enzymol.

[B31] Blank LM, Lehmbeck F, Sauer U (2005). Metabolic-flux analysis of fourteen hemiascomycetous yeasts. FEMS Yeast Res.

[B32] Monschau N, Stahmann K-P, Sahm H, McNeil JB, Bognar AL (1997). Identification of *Saccharomyces cerevisiae *GLY1 as a threonine aldolase: a key enzyme in glycine biosynthesis. FEMS Microbiology Letters.

[B33] Emanuelsson O, Brunak S, von Heijnen G, Nielsen H (2007). Locating proteins in the cell using TargetP, SignalP and related tools. Nature Protocols.

[B34] Emanuelsson O, Nielsen H, Brunak B, von Heijnen G (2000). Predicting subcellular localization of proteins based on their N-terminal amino acid sequenc. J Mol Biol.

[B35] Huh WK, Falvo JV, Gerke LC, Carroll AS, Howson RW, Weissman JS, O'shea EK (2003). Global analysis of protein localization in budding yeast. Nature.

[B36] Christensen KE, MacKenzie RE (2006). Mitochondrial one-carbon metabolism is adapted to the specific needs of yeast, plants and mammals. Bio Essays.

[B37] McNeil JB, McIntosh EM, Taylor BV, Zhang F, Tang S, Bognar AL (1994). Cloning and Molecular Characterization of Three Genes, Including Two Genes Encoding Serine Hydroxymethyltransferases, Whose Inactivation Is Required to Render Yeast Auxotrophic for Glycine. J Biol Chem.

[B38] McNeil JB, Bognar AL, Pearlman RE (1996). *In vivo *analysis of folate coenzymes and their compartmentation in *Saccharomyces cerevisiae*. Genetics.

[B39] Mandels M, Reese ET (1957). Induction of cellulase in *Trichoderma viride *as influenced by carbon sources and metals. J Bacteriol.

[B40] Kanehisa M, Araki M, Goto S, Hattori M, Hirakawa M, Itoh M, Katayama T, Kawashima S, Okuda S, Tokimatsu T, Yamanishi Y (2008). KEGG for linking genomes to life and the environment. Nucleic Acids Res.

[B41] The UniProt Consortium (2007). The Universal Protein Resource (UniProt). Nucleic Acids Res.

[B42] Altschul SF, Madden TL, Schaffer AA, Zhang J, Zhang Z, Miller W, Lipman DJ (1997). Gapped BLAST and PSI-BLAST: a new generation of protein database search programs. Nucleic Acids Res.

[B43] Eppstein D (1994). Finding the k shortest paths. 35th IEEE Symp Foundations of Comp Sci, Santa Fe.

