# Supplementary material for: 13C-metabolic flux ratio and novel carbon path analyses confirmed that Trichoderma reesei uses primarily the respirative pathway also on the preferred carbon source glucose
Source: BMC Syst Biol. 2009 Oct 29;3:104. doi: 10.1186/1752-0509-3-104 (PMC2776023; doi:10.1186/1752-0509-3-104)
Supplement: Additional file 1 — Pathways discovered in ReTrace carbon path analysis. Graphical and tabular representations of amino acid synthesis pathways discovered in ReTrace carbon path analysis [21]. Self-contained web site: unpack zip archive and open index.html with a web browser. [file 1752-0509-3-104-S1.zip › AF1-treesei/pathways-C00026-to-C00624.html]

Pathways from C00026 to C00624


**Pathways from C00026 to C00624**

**Sources:** 2-Oxoglutarate; (C00026)

**Target:**N-Acetyl-L-glutamate; (C00624)

|  | Composite mapping | Z | Average score | Rpairs | Reactions | Zero scores | Scores under threshold |
| --- | --- | --- | --- | --- | --- | --- | --- |
| Path 1 | C00026->C00624:[1->1,2->2,3->4,5->10,5->5,8->6,8->9] | 1.00 | 413.428571429 | 10 | 28 | 0 | 0 |
| Path 2 | C00026->C00624:[1->1,2->2,3->4,5->10,5->5,8->6,8->9] | 1.00 | 175.031413613 | 24 | 191 | 0 | 0 |
| Path 3 | C00026->C00624:[1->1,2->2,3->4,5->10,5->5,8->6,8->9] | 1.00 | 272.040935673 | 17 | 171 | 0 | 0 |
| Path 4 | C00026->C00624:[1->1,2->2,3->4,5->10,5->5,8->6,8->9] | 1.00 | 227.446808511 | 23 | 141 | 0 | 0 |
| Path 5 | C00026->C00624:[1->1,2->2,3->4,3->6,5->10,5->5,8->6,8->9] | 1.00 | 255.229357798 | 22 | 109 | 0 | 0 |
| Path 6 | C00026->C00624:[1->1,2->2,3->4,5->10,5->5,8->6,8->9] | 1.00 | 363.333333333 | 9 | 93 | 0 | 0 |
| Path 7 | C00026->C00624:[1->1,1->10,2->2,3->4,5->5,8->6,8->9] | 1.00 | 272.761006289 | 15 | 159 | 0 | 0 |
| Path 8 | C00026->C00624:[1->1,1->4,2->2,5->10,5->5,8->6,8->9] | 1.00 | 203.752 | 23 | 125 | 0 | 0 |
| Path 9 | C00026->C00624:[1->1,1->4,2->2,5->10,5->5,8->6,8->9] | 1.00 | 195.775700935 | 21 | 107 | 0 | 0 |
| Path 10 | C00026->C00624:[1->1,1->4,2->2,5->10,5->5,8->6,8->9] | 1.00 | 192.58490566 | 21 | 106 | 0 | 0 |
| Path 11 | C00026->C00624:[1->1,2->2,3->4,5->10,5->5,8->6,8->9] | 1.00 | 271.333333333 | 13 | 150 | 0 | 0 |
| Path 12 | C00026->C00624:[1->1,2->2,3->4,5->10,5->5,8->6,8->9] | 1.00 | 151.977272727 | 19 | 176 | 0 | 0 |
| Path 13 | C00026->C00624:[1->1,1->6,2->10,2->2,3->4,5->10,5->5,8->6,8->9] | 1.00 | 192.491803279 | 25 | 183 | 0 | 0 |
| Path 14 | C00026->C00624:[1->1,2->2,3->4,5->10,5->5,8->6,8->9] | 1.00 | 324.307692308 | 20 | 65 | 0 | 0 |
| Path 15 | C00026->C00624:[1->1,2->2,3->4,5->10,5->5,8->6,8->9] | 1.00 | 229.605633803 | 23 | 142 | 0 | 0 |
| Path 16 | C00026->C00624:[1->1,1->6,2->10,2->2,3->4,5->5,8->9] | 1.00 | 247.109756098 | 15 | 164 | 0 | 0 |
| Path 17 | C00026->C00624:[1->1,2->2,3->4,5->10,5->5,8->6,8->9] | 1.00 | 269.098684211 | 14 | 152 | 0 | 0 |
| Path 18 | C00026->C00624:[1->1,2->2,3->4,5->10,5->5,8->6,8->9] | 1.00 | 206.860465116 | 20 | 129 | 0 | 0 |
| Path 19 | C00026->C00624:[1->1,2->2,3->4,5->10,5->5,8->6,8->9] | 1.00 | 247.925287356 | 20 | 174 | 0 | 0 |
| Path 20 | C00026->C00624:[1->1,2->2,3->4,5->10,5->5,8->6,8->9] | 1.00 | 548.153846154 | 8 | 13 | 0 | 0 |
| Path 21 | C00026->C00624:[1->1,2->2,3->4,5->10,5->5,8->6,8->9] | 1.00 | 226.588607595 | 10 | 158 | 0 | 0 |
| Path 22 | C00026->C00624:[1->1,2->2,3->4,5->10,5->5,8->6,8->9] | 1.00 | 235.842857143 | 7 | 140 | 0 | 0 |
| Path 23 | C00026->C00624:[1->1,1->4,2->2,5->10,5->5,8->6,8->9] | 1.00 | 350.737704918 | 21 | 61 | 0 | 0 |
| Path 24 | C00026->C00624:[1->1,1->4,2->2,5->10,5->5,8->6,8->9] | 1.00 | 351.131147541 | 22 | 61 | 0 | 0 |
| Path 25 | C00026->C00624:[1->1,2->2,3->4,5->10,5->5,8->6,8->9] | 1.00 | 331.264150943 | 18 | 53 | 0 | 0 |
| Path 26 | C00026->C00624:[1->1,1->10,2->2,3->4,5->5,8->6,8->9] | 1.00 | 387.96460177 | 16 | 113 | 0 | 0 |
| Path 27 | C00026->C00624:[1->1,2->2,3->4,5->10,5->5,8->6,8->9] | 1.00 | 173.142105263 | 24 | 190 | 0 | 0 |
| Path 28 | C00026->C00624:[1->1,1->4,2->2,5->10,5->5,8->6,8->9] | 1.00 | 203.56 | 22 | 125 | 0 | 0 |
| Path 29 | C00026->C00624:[1->1,2->2,3->4,3->6,5->10,5->5,8->6,8->9] | 1.00 | 358.593495935 | 22 | 123 | 0 | 0 |
| Path 30 | C00026->C00624:[1->1,1->6,2->10,2->2,3->4,5->10,5->5,8->6,8->9] | 1.00 | 169.066298343 | 22 | 181 | 0 | 0 |
| Path 31 | C00026->C00624:[1->1,1->6,2->10,2->2,3->4,5->10,5->5,8->6,8->9] | 1.00 | 176.492462312 | 24 | 199 | 0 | 0 |
| Path 32 | C00026->C00624:[1->1,2->2,3->4,5->10,5->5,8->6,8->9] | 1.00 | 231.722222222 | 25 | 144 | 0 | 0 |
| Path 33 | C00026->C00624:[1->1,2->2,3->4,5->10,5->5,8->6,8->9] | 1.00 | 340.703125 | 21 | 128 | 0 | 0 |
| Path 34 | C00026->C00624:[1->1,2->2,3->4,5->10,5->5,8->6,8->9] | 1.00 | 144.053333333 | 8 | 75 | 0 | 0 |
| Path 35 | C00026->C00624:[1->1,2->2,3->4,5->10,5->5,8->6,8->9] | 1.00 | 184.394117647 | 22 | 170 | 0 | 0 |
| Path 36 | C00026->C00624:[1->1,2->2,3->4,5->10,5->5,8->6,8->9] | 1.00 | 591.833333333 | 7 | 12 | 0 | 0 |
| Path 37 | C00026->C00624:[1->1,2->2,3->4,5->10,5->5,8->6,8->9] | 1.00 | 233.806896552 | 25 | 145 | 0 | 0 |
| Path 38 | C00026->C00624:[1->1,1->6,2->10,2->2,3->4,5->10,5->5,8->6,8->9] | 1.00 | 171.071428571 | 22 | 182 | 0 | 0 |
| Path 39 | C00026->C00624:[1->1,2->2,3->4,5->10,5->5,8->6,8->9] | 1.00 | 256.596774194 | 23 | 124 | 0 | 0 |
| Path 40 | C00026->C00624:[1->1,1->6,2->10,2->2,3->4,5->5,8->9] | 1.00 | 247.510989011 | 17 | 182 | 0 | 0 |
| Path 41 | C00026->C00624:[1->1,2->2,3->4,3->6,5->10,5->5,8->6,8->9] | 1.00 | 221.728 | 21 | 125 | 0 | 0 |
| Path 42 | C00026->C00624:[1->1,2->2,3->4,5->10,5->5,8->6,8->9] | 1.00 | 267.2 | 16 | 170 | 0 | 0 |
| Path 43 | C00026->C00624:[1->1,2->2,3->4,5->10,5->5,8->6,8->9] | 1.00 | 209.376923077 | 20 | 130 | 0 | 0 |
| Path 44 | C00026->C00624:[1->1,2->2,3->4,5->10,5->5,8->6,8->9] | 1.00 | 296.87804878 | 15 | 41 | 0 | 0 |
| Path 45 | C00026->C00624:[1->1,2->2,3->4,5->10,5->5,8->6,8->9] | 1.00 | 479.171428571 | 18 | 35 | 0 | 0 |
| Path 46 | C00026->C00624:[1->1,2->2,3->4,5->10,5->5,8->6,8->9] | 1.00 | 241.936170213 | 8 | 141 | 0 | 0 |
| Path 47 | C00026->C00624:[1->1,2->2,3->4,5->10,5->5,8->6,8->9] | 1.00 | 299.106382979 | 12 | 47 | 0 | 0 |
| Path 48 | C00026->C00624:[1->1,1->4,2->2,5->10,5->5,8->6,8->9] | 1.00 | 347.683333333 | 21 | 60 | 0 | 0 |
| Path 49 | C00026->C00624:[1->1,1->4,2->2,5->10,5->5,8->6,8->9] | 1.00 | 195.551401869 | 20 | 107 | 0 | 0 |
| Path 50 | C00026->C00624:[1->1,2->2,3->4,5->10,5->5,8->6,8->9] | 1.00 | 380.727272727 | 6 | 77 | 0 | 0 |
| Path 51 | C00026->C00624:[1->1,2->2,3->4,5->10,5->5,8->6,8->9] | 1.00 | 168.978609626 | 22 | 187 | 0 | 0 |
| Path 52 | C00026->C00624:[1->1,2->2,3->4,5->10,5->5,8->6,8->9] | 1.00 | 587.214285714 | 9 | 14 | 0 | 0 |
| Path 53 | C00026->C00624:[1->1,2->2,3->4,5->10,5->5,8->6,8->9] | 1.00 | 412.333333333 | 10 | 30 | 0 | 0 |
| Path 54 | C00026->C00624:[1->1,2->2,3->4,5->10,5->5,8->6,8->9] | 1.00 | 323.857142857 | 11 | 112 | 0 | 0 |
| Path 55 | C00026->C00624:[1->1,2->2,3->4,5->10,5->5,8->6,8->9] | 1.00 | 230.162962963 | 23 | 135 | 0 | 0 |
| Path 56 | C00026->C00624:[1->1,2->2,3->4,5->10,5->5,8->6,8->9] | 1.00 | 356.265957447 | 8 | 94 | 0 | 0 |
| Path 57 | C00026->C00624:[1->1,1->4,2->2,5->10,5->5,8->6,8->9] | 1.00 | 201.088709677 | 23 | 124 | 0 | 0 |
| Path 58 | C00026->C00624:[1->1,2->2,3->4,5->10,5->5,8->6,8->9] | 1.00 | 261.983193277 | 24 | 119 | 0 | 0 |
| Path 59 | C00026->C00624:[1->1,1->6,2->10,2->2,3->4,5->10,5->5,8->6,8->9] | 1.00 | 185.666666667 | 19 | 111 | 0 | 0 |
| Path 60 | C00026->C00624:[1->1,1->6,2->10,2->2,3->4,5->10,5->5,8->6,8->9] | 1.00 | 188.186746988 | 23 | 166 | 0 | 0 |
| Path 61 | C00026->C00624:[1->1,2->2,3->4,5->10,5->5,8->6,8->9] | 1.00 | 376.153846154 | 7 | 78 | 0 | 0 |
| Path 62 | C00026->C00624:[1->1,2->2,3->4,5->10,5->5,8->6,8->9] | 1.00 | 336.696428571 | 20 | 56 | 0 | 0 |
| Path 63 | C00026->C00624:[1->1,1->10,2->2,3->4,5->5,8->6,8->9] | 1.00 | 225.053191489 | 16 | 94 | 0 | 0 |
| Path 64 | C00026->C00624:[1->1,2->2,3->4,5->10,5->5,8->6,8->9] | 1.00 | 267.63253012 | 15 | 166 | 0 | 0 |
| Path 65 | C00026->C00624:[1->1,1->6,2->10,2->2,3->4,5->10,5->5,8->6,8->9] | 1.00 | 218.031578947 | 20 | 95 | 0 | 0 |
| Path 66 | C00026->C00624:[1->1,2->2,3->4,5->10,5->5,8->6,8->9] | 1.00 | 388.793103448 | 9 | 29 | 0 | 0 |
| Path 67 | C00026->C00624:[1->1,2->2,3->4,5->10,5->5,8->6,8->9] | 1.00 | 270.786982249 | 16 | 169 | 0 | 0 |
| Path 68 | C00026->C00624:[1->1,2->2,3->4,5->10,5->5,8->6,8->9] | 1.00 | 156.565789474 | 9 | 76 | 0 | 0 |
| Path 69 | C00026->C00624:[1->1,1->6,2->10,2->2,3->4,5->10,5->5,8->6,8->9] | 1.00 | 194.347826087 | 25 | 184 | 0 | 0 |
| Path 70 | C00026->C00624:[1->1,1->10,2->2,3->4,5->5,8->6,8->9] | 1.00 | 450.541666667 | 17 | 48 | 0 | 0 |
| Path 71 | C00026->C00624:[1->1,2->2,3->4,5->10,5->5,8->6,8->9] | 1.00 | 265.805369128 | 12 | 149 | 0 | 0 |
| Path 72 | C00026->C00624:[1->1,2->2,3->4,5->10,5->5,8->6,8->9] | 1.00 | 274.496732026 | 15 | 153 | 0 | 0 |
| Path 73 | C00026->C00624:[1->1,2->2,3->4,5->10,5->5,8->6,8->9] | 1.00 | 269.172619048 | 15 | 168 | 0 | 0 |
| Path 74 | C00026->C00624:[1->1,2->2,3->4,5->10,5->5,8->6,8->9] | 1.00 | 254.341463415 | 23 | 123 | 0 | 0 |
| Path 75 | C00026->C00624:[1->1,2->2,3->4,5->10,5->5,8->6,8->9] | 1.00 | 149.794285714 | 19 | 175 | 0 | 0 |
| Path 76 | C00026->C00624:[1->1,2->2,3->4,5->10,5->5,8->6,8->9] | 1.00 | 146.096774194 | 11 | 93 | 0 | 0 |
| Path 77 | C00026->C00624:[1->1,2->2,3->4,5->10,5->5,8->6,8->9] | 1.00 | 264.22754491 | 14 | 167 | 0 | 0 |
| Path 78 | C00026->C00624:[1->1,1->4,2->2,5->10,5->5,8->6,8->9] | 1.00 | 348.083333333 | 22 | 60 | 0 | 0 |
| Path 79 | C00026->C00624:[1->1,2->2,3->4,5->10,5->5,8->6,8->9] | 1.00 | 264.25 | 24 | 120 | 0 | 0 |
| Path 80 | C00026->C00624:[1->1,2->2,3->4,5->10,5->5,8->6,8->9] | 1.00 | 385.253164557 | 8 | 79 | 0 | 0 |
| Path 81 | C00026->C00624:[1->1,2->2,3->4,5->10,5->5,8->6,8->9] | 1.00 | 364.042105263 | 9 | 95 | 0 | 0 |
| Path 82 | C00026->C00624:[1->1,2->2,3->4,5->10,5->5,8->6,8->9] | 1.00 | 182.325443787 | 22 | 169 | 0 | 0 |
| Path 83 | C00026->C00624:[1->1,1->4,2->2,5->10,5->5,8->6,8->9] | 1.00 | 192.358490566 | 20 | 106 | 0 | 0 |
| Path 84 | C00026->C00624:[1->1,2->2,3->4,5->10,5->5,8->6,8->9] | 1.00 | 232.397058824 | 23 | 136 | 0 | 0 |
| Path 85 | C00026->C00624:[1->1,2->2,3->4,5->10,5->5,8->6,8->9] | 1.00 | 170.920212766 | 22 | 188 | 0 | 0 |
| Path 86 | C00026->C00624:[1->1,1->4,2->2,5->10,5->5,8->6,8->9] | 1.00 | 200.89516129 | 22 | 124 | 0 | 0 |
| Path 87 | C00026->C00624:[1->1,1->6,2->10,2->2,3->4,5->10,5->5,8->6,8->9] | 1.00 | 178.28 | 24 | 200 | 0 | 0 |
| Path 88 | C00026->C00624:[1->1,1->6,2->10,2->2,3->4,5->10,5->5,8->6,8->9] | 1.00 | 186.090909091 | 23 | 165 | 0 | 0 |
| Path 89 | C00026->C00624:[1->1,1->4,2->2,5->5,8->9] | 0.71 | 390.888888889 | 16 | 36 | 0 | 0 |
| Path 90 | C00026->C00624:[1->1,2->10,2->2,3->4,3->6,5->5,8->9] | 1.00 | 390.037735849 | 17 | 106 | 0 | 0 |
| Path 91 | C00026->C00624:[1->1,2->2,3->4,5->10,5->5,8->6,8->9] | 1.00 | 168.316770186 | 18 | 161 | 0 | 0 |
| Path 92 | C00026->C00624:[1->1,2->2,3->4,3->6,5->5,8->9] | 0.86 | 192.684210526 | 12 | 133 | 0 | 0 |
| Path 93 | C00026->C00624:[1->1,1->6,2->10,2->2,3->4,5->5,8->9] | 1.00 | 262.654761905 | 16 | 168 | 0 | 0 |
| Path 94 | C00026->C00624:[1->1,1->10,2->2,2->6,5->5,8->9] | 0.86 | 225.611650485 | 15 | 103 | 0 | 0 |
| Path 95 | C00026->C00624:[1->1,1->4,2->2,5->10,5->5,8->6,8->9] | 1.00 | 190.8 | 20 | 105 | 0 | 0 |
| Path 96 | C00026->C00624:[1->1,1->10,2->2,3->4,3->6,5->5,8->9] | 1.00 | 225.485714286 | 19 | 105 | 0 | 0 |
| Path 97 | C00026->C00624:[1->1,1->10,2->2,2->6,3->4,5->5,8->9] | 1.00 | 214.877192982 | 15 | 114 | 0 | 0 |
| Path 98 | C00026->C00624:[1->1,2->10,2->2,3->4,5->5,5->6,8->9] | 1.00 | 151.05952381 | 10 | 84 | 0 | 0 |
| Path 99 | C00026->C00624:[5->10,8->4,8->6] | 0.43 | 192.580246914 | 14 | 81 | 0 | 0 |
| Path 100 | C00026->C00624:[1->1,2->10,2->2,3->4,5->5,5->6,8->9] | 1.00 | 234.248322148 | 9 | 149 | 0 | 0 |
| Path 101 | C00026->C00624:[3->4,3->6] | 0.29 | 142.135483871 | 14 | 155 | 0 | 0 |
| Path 102 | C00026->C00624:[5->10,8->6] | 0.29 | 651.6 | 7 | 10 | 0 | 0 |
| Path 103 | C00026->C00624:[1->10,1->6,2->10,8->4] | 0.43 | 213.254901961 | 15 | 102 | 0 | 0 |
| Path 104 | C00026->C00624:[1->1,1->10,2->2,2->6,3->4,5->5,8->9] | 1.00 | 305.78 | 16 | 50 | 0 | 0 |
| Path 105 | C00026->C00624:[1->1,1->6,2->10,2->2,3->4,5->5,8->9] | 1.00 | 237.48245614 | 20 | 114 | 0 | 0 |
| Path 106 | C00026->C00624:[1->1,2->2,3->4,5->10,5->5,8->6,8->9] | 1.00 | 294.8 | 14 | 40 | 0 | 0 |
| Path 107 | C00026->C00624:[1->1,2->2,3->4,5->5,8->9] | 0.71 | 403.125 | 5 | 8 | 0 | 0 |
| Path 108 | C00026->C00624:[1->1,2->2,3->4,5->5,8->9] | 0.71 | 348.479452055 | 4 | 73 | 0 | 0 |
| Path 109 | C00026->C00624:[1->1,2->2,3->4,3->6,5->5,8->9] | 0.86 | 162.00952381 | 11 | 105 | 0 | 0 |
| Path 110 | C00026->C00624:[1->1,2->2,3->4,5->5,8->9] | 0.71 | 218.487179487 | 22 | 234 | 0 | 2 |
| Path 111 | C00026->C00624:[1->1,2->2,3->4,5->5,8->9] | 0.71 | 251.714285714 | 11 | 147 | 0 | 0 |
| Path 112 | C00026->C00624:[1->1,1->6,2->10,2->2,3->4,5->5,8->9] | 1.00 | 272.964125561 | 19 | 223 | 0 | 0 |
| Path 113 | C00026->C00624:[1->1,2->2,3->4,5->5,8->9] | 0.71 | 148.829113924 | 17 | 158 | 0 | 0 |
| Path 114 | C00026->C00624:[1->1,2->2,5->10,5->5,8->6,8->9] | 0.86 | 426.363636364 | 13 | 33 | 0 | 0 |
| Path 115 | C00026->C00624:[1->1,2->2,3->4,5->5,8->6,8->9] | 0.86 | 170.714285714 | 12 | 84 | 0 | 0 |
| Path 116 | C00026->C00624:[1->1,2->2,3->4,3->6,5->10,5->5,8->6,8->9] | 1.00 | 214.45814978 | 18 | 227 | 0 | 2 |
| Path 117 | C00026->C00624:[5->10,8->4,8->6] | 0.43 | 663.9375 | 12 | 16 | 0 | 0 |
| Path 118 | C00026->C00624:[1->1,1->6,2->10,2->2,3->4,5->5,8->9] | 1.00 | 157.953367876 | 20 | 193 | 0 | 0 |
| Path 119 | C00026->C00624:[5->10,8->6] | 0.29 | 287.279069767 | 10 | 43 | 0 | 0 |
| Path 120 | C00026->C00624:[1->1,2->2,3->4,5->5,8->9] | 0.71 | 379.708737864 | 14 | 103 | 0 | 0 |
| Path 121 | C00026->C00624:[1->1,2->2,3->4,5->5,8->9] | 0.71 | 152.632911392 | 16 | 158 | 0 | 0 |
| Path 122 | C00026->C00624:[1->10,1->6,2->10,3->6,8->4] | 0.43 | 396.839285714 | 16 | 56 | 0 | 0 |
| Path 123 | C00026->C00624:[1->1,1->6,2->10,2->2,3->4,5->5,8->9] | 1.00 | 148.98816568 | 14 | 169 | 0 | 0 |
| Path 124 | C00026->C00624:[5->10,8->4,8->6] | 0.43 | 183.012048193 | 13 | 83 | 0 | 0 |
| Path 125 | C00026->C00624:[2->2,3->1,3->4,5->10,5->5,8->6,8->9] | 1.00 | 259.918181818 | 19 | 110 | 0 | 0 |
| Path 126 | C00026->C00624:[1->1,1->10,2->2,2->6,5->5,8->9] | 0.86 | 221.631578947 | 19 | 95 | 0 | 0 |
| Path 127 | C00026->C00624:[1->1,1->6,2->10,2->2,3->4,5->5,8->9] | 1.00 | 204.92248062 | 19 | 129 | 0 | 0 |
| Path 128 | C00026->C00624:[1->1,2->2,3->4,5->5,8->9] | 0.71 | 192.735849057 | 15 | 106 | 0 | 0 |
| Path 129 | C00026->C00624:[1->1,1->10,2->2,2->6,5->5,8->9] | 0.86 | 191.798245614 | 20 | 114 | 0 | 0 |
| Path 130 | C00026->C00624:[1->1,1->10,2->2,2->6,3->4,5->5,8->9] | 1.00 | 159.407608696 | 20 | 184 | 0 | 0 |
| Path 131 | C00026->C00624:[1->1,1->4,2->2,5->5,8->9] | 0.71 | 363.933333333 | 11 | 30 | 0 | 0 |
| Path 132 | C00026->C00624:[1->1,1->10,2->2,2->6,3->4,5->5,8->9] | 1.00 | 215.956521739 | 21 | 138 | 0 | 0 |
| Path 133 | C00026->C00624:[1->1,1->10,2->2,2->6,3->4,5->5,8->9] | 1.00 | 214.575539568 | 22 | 139 | 0 | 0 |
| Path 134 | C00026->C00624:[8->4] | 0.14 | 356.375 | 11 | 32 | 0 | 0 |
| Path 135 | C00026->C00624:[1->1,1->6,2->10,2->2,3->4,5->5,8->9] | 1.00 | 163.949685535 | 19 | 159 | 0 | 0 |
| Path 136 | C00026->C00624:[1->1,2->2,3->4,5->10,5->5,8->6,8->9] | 1.00 | 210.419047619 | 14 | 105 | 0 | 0 |
| Path 137 | C00026->C00624:[1->1,2->2,3->4,5->10,5->5,8->6,8->9] | 1.00 | 340.393700787 | 20 | 127 | 0 | 0 |
| Path 138 | C00026->C00624:[1->1,1->6,2->10,2->2,3->4,5->5,8->9] | 1.00 | 148.365714286 | 18 | 175 | 0 | 0 |
| Path 139 | C00026->C00624:[1->1,1->6,2->10,2->2,3->4,5->5,8->9] | 1.00 | 376.852941176 | 13 | 102 | 0 | 0 |
| Path 140 | C00026->C00624:[1->1,2->2,3->4,5->5,8->9] | 0.71 | 167.952662722 | 17 | 169 | 0 | 0 |
| Path 141 | C00026->C00624:[5->10,8->4,8->6] | 0.43 | 646.846153846 | 9 | 13 | 0 | 0 |
| Path 142 | C00026->C00624:[1->1,1->10,2->2,2->6,3->4,5->5,8->9] | 1.00 | 300.254901961 | 17 | 51 | 0 | 0 |
| Path 143 | C00026->C00624:[1->1,1->6,2->10,2->2,3->4,5->5,8->9] | 1.00 | 318.2 | 10 | 35 | 0 | 0 |
| Path 144 | C00026->C00624:[1->1,1->10,2->2,3->4,3->6,5->5,8->9] | 1.00 | 270.95 | 12 | 160 | 0 | 0 |
| Path 145 | C00026->C00624:[5->10,8->4,8->6] | 0.43 | 423.27027027 | 14 | 37 | 0 | 0 |
| Path 146 | C00026->C00624:[1->1,2->2,3->4,5->10,5->5,8->6,8->9] | 1.00 | 170.380368098 | 20 | 163 | 0 | 0 |
| Path 147 | C00026->C00624:[1->1,2->2,3->4,5->10,5->5,8->6,8->9] | 1.00 | 148.471264368 | 18 | 174 | 0 | 0 |
| Path 148 | C00026->C00624:[1->1,2->2,3->4,3->6,5->5,8->9] | 0.86 | 240.844311377 | 10 | 167 | 0 | 0 |
| Path 149 | C00026->C00624:[1->1,1->4,2->2,5->10,5->5,8->6,8->9] | 1.00 | 202.330645161 | 22 | 124 | 0 | 0 |
| Path 150 | C00026->C00624:[1->1,2->2,3->4,5->5,8->9] | 0.71 | 228.551020408 | 22 | 245 | 0 | 2 |
| Path 151 | C00026->C00624:[5->10,8->4,8->6] | 0.43 | 456.677419355 | 13 | 31 | 0 | 0 |
| Path 152 | C00026->C00624:[1->1,1->6,2->10,2->2,3->4,5->5,8->9] | 1.00 | 207.453846154 | 19 | 130 | 0 | 0 |
| Path 153 | C00026->C00624:[1->1,2->2,3->4,5->10,5->5,8->6,8->9] | 1.00 | 205.5078125 | 19 | 128 | 0 | 0 |
| Path 154 | C00026->C00624:[1->1,1->6,2->10,2->2,5->5,8->9] | 0.86 | 326.637931034 | 18 | 58 | 0 | 0 |
| Path 155 | C00026->C00624:[1->1,2->2,5->10,5->5,8->6,8->9] | 0.86 | 405.46875 | 12 | 32 | 0 | 0 |
| Path 156 | C00026->C00624:[5->10,8->4,8->6] | 0.43 | 536.264705882 | 15 | 34 | 0 | 0 |
| Path 157 | C00026->C00624:[1->1,1->10,2->2,3->4,3->6,5->5,8->9] | 1.00 | 205.530612245 | 13 | 98 | 0 | 0 |
| Path 158 | C00026->C00624:[5->10,8->6] | 0.29 | 141.583333333 | 7 | 72 | 0 | 0 |
| Path 159 | C00026->C00624:[1->1,1->10,2->2,2->6,3->4,5->5,8->9] | 1.00 | 392.689655172 | 19 | 116 | 0 | 0 |
| Path 160 | C00026->C00624:[5->10,8->6] | 0.29 | 128.154929577 | 6 | 71 | 0 | 0 |
| Path 161 | C00026->C00624:[1->1,1->6,2->10,2->2,3->4,5->5,8->9] | 1.00 | 259.333333333 | 15 | 165 | 0 | 0 |
| Path 162 | C00026->C00624:[1->1,1->10,2->2,2->6,5->5,8->9] | 0.86 | 333.014925373 | 20 | 67 | 0 | 0 |
| Path 163 | C00026->C00624:[1->1,1->4,2->2,5->10,5->5,8->6,8->9] | 1.00 | 181.530864198 | 13 | 81 | 0 | 0 |
| Path 164 | C00026->C00624:[1->1,2->2,5->5,8->4,8->9] | 0.71 | 190.076086957 | 12 | 92 | 0 | 0 |
| Path 165 | C00026->C00624:[1->1,1->6,2->10,2->2,5->5,8->9] | 0.86 | 397.121212121 | 12 | 33 | 0 | 0 |
| Path 166 | C00026->C00624:[1->1,2->2,2->6,5->5,8->9] | 0.71 | 199.056603774 | 16 | 106 | 0 | 0 |
| Path 167 | C00026->C00624:[1->1,1->4,2->2,5->5,8->9] | 0.71 | 391.555555556 | 17 | 36 | 0 | 0 |
| Path 168 | C00026->C00624:[8->6] | 0.14 | 385.470588235 | 11 | 34 | 0 | 0 |
| Path 169 | C00026->C00624:[1->1,1->6,2->10,2->2,3->4,5->5,8->9] | 1.00 | 201.228571429 | 13 | 105 | 0 | 0 |
| Path 170 | C00026->C00624:[5->10,8->4,8->6] | 0.43 | 443.15625 | 14 | 32 | 0 | 0 |
| Path 171 | C00026->C00624:[5->10,8->4,8->6] | 0.43 | 660.944444444 | 13 | 18 | 0 | 0 |
| Path 172 | C00026->C00624:[1->1,2->2,3->4,3->6,5->5,8->9] | 0.86 | 151.443181818 | 9 | 88 | 0 | 0 |
| Path 173 | C00026->C00624:[1->1,2->2,3->4,3->6,5->10,5->5,8->9] | 1.00 | 210.702222222 | 17 | 225 | 0 | 2 |
| Path 174 | C00026->C00624:[1->1,1->6,2->10,2->2,3->4,3->6,5->5,8->9] | 1.00 | 214.871369295 | 17 | 241 | 0 | 0 |
| Path 175 | C00026->C00624:[1->1,1->6,2->10,2->2,3->4,5->5,8->9] | 1.00 | 150.556818182 | 18 | 176 | 0 | 0 |
| Path 176 | C00026->C00624:[1->1,2->2,3->4,5->5,8->9] | 0.71 | 255.024390244 | 14 | 164 | 0 | 0 |
| Path 177 | C00026->C00624:[1->1,1->6,2->10,2->2,3->4,5->5,8->9] | 1.00 | 255.957831325 | 14 | 166 | 0 | 0 |
| Path 178 | C00026->C00624:[1->1,1->6,2->10,2->2,5->5,8->9] | 0.86 | 176.855670103 | 13 | 97 | 0 | 0 |
| Path 179 | C00026->C00624:[1->1,1->4,2->2,5->5,8->9] | 0.71 | 181.46 | 18 | 100 | 0 | 0 |
| Path 180 | C00026->C00624:[1->1,1->10,2->2,3->4,3->6,5->5,8->9] | 1.00 | 380.552083333 | 14 | 96 | 0 | 0 |
| Path 181 | C00026->C00624:[1->6,2->10,3->4] | 0.43 | 240.43030303 | 18 | 165 | 0 | 0 |
| Path 182 | C00026->C00624:[1->1,2->2,5->5,8->4,8->9] | 0.71 | 173.121212121 | 14 | 99 | 0 | 0 |
| Path 183 | C00026->C00624:[1->1,2->2,3->4,5->5,8->9] | 0.71 | 239.479674797 | 17 | 123 | 0 | 0 |
| Path 184 | C00026->C00624:[1->1,2->2,3->4,3->6,5->5,8->9] | 0.86 | 173.0 | 17 | 129 | 0 | 0 |
| Path 185 | C00026->C00624:[1->1,1->10,2->2,3->4,3->6,5->5,8->9] | 1.00 | 223.272727273 | 24 | 242 | 0 | 2 |
| Path 186 | C00026->C00624:[1->1,1->10,2->2,3->4,3->6,5->5,8->9] | 1.00 | 348.658333333 | 20 | 120 | 0 | 0 |
| Path 187 | C00026->C00624:[1->1,1->10,2->2,2->6,3->4,5->5,8->9] | 1.00 | 155.209876543 | 16 | 162 | 0 | 0 |
| Path 188 | C00026->C00624:[1->1,1->4,2->2,5->10,5->5,8->6,8->9] | 1.00 | 350.65 | 21 | 60 | 0 | 0 |
| Path 189 | C00026->C00624:[1->1,2->2,3->4,5->10,5->5,8->6,8->9] | 1.00 | 575.538461538 | 8 | 13 | 0 | 0 |
| Path 190 | C00026->C00624:[1->1,1->6,2->10,2->2,5->5,8->9] | 0.86 | 453.547619048 | 19 | 42 | 0 | 0 |
| Path 191 | C00026->C00624:[1->1,1->10,2->2,3->4,3->6,5->5,8->9] | 1.00 | 402.516853933 | 12 | 89 | 0 | 0 |
| Path 192 | C00026->C00624:[1->1,2->2,3->4,5->10,5->5,8->6,8->9] | 1.00 | 185.860465116 | 18 | 172 | 0 | 0 |
| Path 193 | C00026->C00624:[1->1,1->10,2->2,3->4,3->6,5->5,8->9] | 1.00 | 208.590163934 | 19 | 122 | 0 | 0 |
| Path 194 | C00026->C00624:[1->1,1->10,2->2,2->6,3->4,5->5,8->9] | 1.00 | 160.693548387 | 21 | 186 | 0 | 0 |
| Path 195 | C00026->C00624:[5->10,8->4,8->6] | 0.43 | 433.0 | 12 | 32 | 0 | 0 |
| Path 196 | C00026->C00624:[5->10,8->4,8->6] | 0.43 | 171.602564103 | 11 | 78 | 0 | 0 |
| Path 197 | C00026->C00624:[1->1,1->10,2->2,2->6,5->5,8->9] | 0.86 | 415.947368421 | 16 | 57 | 0 | 0 |
| Path 198 | C00026->C00624:[1->1,1->10,2->2,2->6,3->4,5->5,8->9] | 1.00 | 174.125748503 | 20 | 167 | 0 | 0 |
| Path 199 | C00026->C00624:[1->1,1->10,2->2,2->6,3->4,5->5,8->9] | 1.00 | 272.38121547 | 19 | 181 | 0 | 0 |
| Path 200 | C00026->C00624:[2->2,3->1,3->4,5->10,5->5,8->6,8->9] | 1.00 | 263.207207207 | 19 | 111 | 0 | 0 |
| Path 201 | C00026->C00624:[1->1,2->2,3->4,5->5,8->9] | 0.71 | 214.160714286 | 18 | 112 | 0 | 0 |
| Path 202 | C00026->C00624:[1->1,2->2,3->4,5->10,5->5,8->6,8->9] | 1.00 | 258.6875 | 21 | 128 | 0 | 0 |
| Path 203 | C00026->C00624:[1->1,2->2,3->4,5->5,8->9] | 0.71 | 171.50887574 | 16 | 169 | 0 | 0 |
| Path 204 | C00026->C00624:[1->1,2->2,3->4,5->5,8->9] | 0.71 | 316.647058824 | 10 | 17 | 0 | 0 |
| Path 205 | C00026->C00624:[1->1,2->2,3->4,3->6,5->5,8->9] | 0.86 | 177.484210526 | 15 | 95 | 0 | 0 |
| Path 206 | C00026->C00624:[1->1,2->2,3->4,3->6,5->10,5->5,8->9] | 1.00 | 218.320175439 | 19 | 228 | 0 | 2 |
| Path 207 | C00026->C00624:[1->1,1->10,2->2,2->6,3->4,5->5,8->9] | 1.00 | 241.8 | 21 | 120 | 0 | 0 |
| Path 208 | C00026->C00624:[1->1,1->6,2->10,2->2,3->4,5->5,8->9] | 1.00 | 236.895104895 | 8 | 143 | 0 | 0 |
| Path 209 | C00026->C00624:[1->1,1->10,2->2,2->6,3->4,5->5,8->9] | 1.00 | 244.214876033 | 21 | 121 | 0 | 0 |
| Path 210 | C00026->C00624:[1->1,1->6,2->10,2->2,3->4,5->5,8->9] | 1.00 | 174.848314607 | 21 | 178 | 0 | 0 |
| Path 211 | C00026->C00624:[1->1,1->10,2->2,2->6,5->5,8->9] | 0.86 | 328.470588235 | 21 | 68 | 0 | 0 |
| Path 212 | C00026->C00624:[1->1,2->2,3->4,5->5,8->9] | 0.71 | 220.629166667 | 19 | 240 | 0 | 2 |
| Path 213 | C00026->C00624:[1->1,1->6,2->10,2->2,3->4,3->6,5->5,8->9] | 1.00 | 217.393822394 | 19 | 259 | 0 | 0 |
| Path 214 | C00026->C00624:[1->1,1->4,2->2,5->10,5->5,8->6,8->9] | 1.00 | 194.191919192 | 15 | 99 | 0 | 0 |
| Path 215 | C00026->C00624:[1->1,1->6,2->10,2->2,3->4,5->5,8->9] | 1.00 | 195.474576271 | 21 | 177 | 0 | 0 |
| Path 216 | C00026->C00624:[1->1,2->2,5->10,5->5,8->4,8->6,8->9] | 1.00 | 224.164948454 | 15 | 97 | 0 | 0 |
| Path 217 | C00026->C00624:[1->1,2->10,2->2,3->4,5->5,5->6,8->9] | 1.00 | 134.402298851 | 10 | 87 | 0 | 0 |
| Path 218 | C00026->C00624:[1->1,2->2,5->10,5->5,8->4,8->6,8->9] | 1.00 | 205.730769231 | 17 | 104 | 0 | 0 |
| Path 219 | C00026->C00624:[1->1,1->10,1->6,2->10,2->2,3->4,5->5,8->9] | 1.00 | 232.81027668 | 24 | 253 | 0 | 2 |
| Path 220 | C00026->C00624:[1->1,1->6,2->10,2->2,5->5,8->9] | 0.86 | 207.402298851 | 18 | 87 | 0 | 0 |
| Path 221 | C00026->C00624:[1->1,1->6,2->10,2->2,5->5,8->9] | 0.86 | 177.634615385 | 17 | 104 | 0 | 0 |
| Path 222 | C00026->C00624:[1->1,1->6,2->10,2->2,3->4,5->5,8->9] | 1.00 | 224.535315985 | 18 | 269 | 0 | 0 |
| Path 223 | C00026->C00624:[1->1,2->2,3->4,5->10,5->5,8->6,8->9] | 1.00 | 395.875 | 14 | 104 | 0 | 0 |
| Path 224 | C00026->C00624:[1->1,1->6,2->10,2->2,3->4,5->5,8->9] | 1.00 | 149.512820513 | 9 | 78 | 0 | 0 |
| Path 225 | C00026->C00624:[1->1,1->6,2->10,2->2,3->4,5->5,8->9] | 1.00 | 234.85840708 | 20 | 113 | 0 | 0 |
| Path 226 | C00026->C00624:[1->1,2->2,5->5,8->4,8->9] | 0.71 | 200.072727273 | 14 | 110 | 0 | 0 |
| Path 227 | C00026->C00624:[5->10,8->4,8->6] | 0.43 | 606.6875 | 11 | 16 | 0 | 0 |
| Path 228 | C00026->C00624:[1->1,2->2,3->4,3->6,5->5,8->9] | 0.86 | 318.4 | 16 | 110 | 0 | 0 |
| Path 229 | C00026->C00624:[1->1,1->4,2->2,5->5,8->9] | 0.71 | 137.460526316 | 10 | 76 | 0 | 0 |
| Path 230 | C00026->C00624:[1->1,1->4,2->2,5->10,5->5,8->6,8->9] | 1.00 | 202.137096774 | 21 | 124 | 0 | 0 |
| Path 231 | C00026->C00624:[1->1,1->4,2->2,5->5,8->9] | 0.71 | 170.590361446 | 16 | 83 | 0 | 0 |
| Path 232 | C00026->C00624:[1->1,1->6,2->10,2->2,3->4,5->5,8->9] | 1.00 | 383.027522936 | 17 | 109 | 0 | 0 |
| Path 233 | C00026->C00624:[1->1,2->2,3->4,3->6,5->5,8->9] | 0.86 | 367.202531646 | 8 | 79 | 0 | 0 |
| Path 234 | C00026->C00624:[1->1,1->10,2->2,2->6,3->4,5->5,8->9] | 1.00 | 477.347826087 | 10 | 23 | 0 | 0 |
| Path 235 | C00026->C00624:[1->1,1->6,2->10,2->2,3->4,5->5,8->9] | 1.00 | 385.346153846 | 15 | 104 | 0 | 0 |
| Path 236 | C00026->C00624:[1->1,1->6,2->10,2->2,3->4,5->5,8->9] | 1.00 | 379.15625 | 10 | 32 | 0 | 0 |
| Path 237 | C00026->C00624:[1->1,2->2,3->4,5->10,5->5,8->6,8->9] | 1.00 | 136.853333333 | 12 | 150 | 0 | 0 |
| Path 238 | C00026->C00624:[1->1,2->2,5->10,5->5,8->4,8->6,8->9] | 1.00 | 237.306930693 | 18 | 101 | 0 | 0 |
| Path 239 | C00026->C00624:[1->1,1->4,2->2,5->5,8->9] | 0.71 | 181.22 | 17 | 100 | 0 | 0 |
| Path 240 | C00026->C00624:[8->6] | 0.14 | 157.9375 | 10 | 80 | 0 | 0 |
| Path 241 | C00026->C00624:[1->1,1->6,2->10,2->2,3->4,5->5,8->9] | 1.00 | 256.540540541 | 12 | 148 | 0 | 0 |
| Path 242 | C00026->C00624:[2->10,3->4,3->6] | 0.43 | 233.804878049 | 18 | 123 | 0 | 0 |
| Path 243 | C00026->C00624:[1->1,1->6,2->10,2->2,3->4,3->6,5->5,8->9] | 1.00 | 267.023529412 | 18 | 170 | 0 | 0 |
| Path 244 | C00026->C00624:[1->1,1->6,2->10,2->2,5->5,8->9] | 0.86 | 174.174757282 | 17 | 103 | 0 | 0 |
| Path 245 | C00026->C00624:[1->1,1->6,2->10,2->2,5->5,8->9] | 0.86 | 185.628099174 | 19 | 121 | 0 | 0 |
| Path 246 | C00026->C00624:[1->1,1->4,2->2,5->10,5->5,8->6,8->9] | 1.00 | 347.542372881 | 21 | 59 | 0 | 0 |
| Path 247 | C00026->C00624:[5->10,8->4,8->6] | 0.43 | 471.941176471 | 14 | 34 | 0 | 0 |
| Path 248 | C00026->C00624:[1->1,2->2,3->4,5->10,5->5,8->6,8->9] | 1.00 | 239.739130435 | 19 | 115 | 0 | 0 |
| Path 249 | C00026->C00624:[1->1,2->2,3->4,5->10,5->5,8->6,8->9] | 1.00 | 241.393162393 | 21 | 117 | 0 | 0 |
| Path 250 | C00026->C00624:[1->1,2->2,2->6,5->5,8->9] | 0.71 | 188.397727273 | 14 | 88 | 0 | 0 |
| Path 251 | C00026->C00624:[1->1,2->2,3->4,5->10,5->5,8->6,8->9] | 1.00 | 201.913461538 | 13 | 104 | 0 | 0 |
| Path 252 | C00026->C00624:[1->1,2->2,3->4,5->5,8->9] | 0.71 | 354.048076923 | 16 | 104 | 0 | 0 |
| Path 253 | C00026->C00624:[1->10,3->6,8->4] | 0.43 | 385.644444444 | 16 | 45 | 0 | 0 |
| Path 254 | C00026->C00624:[1->1,1->10,2->2,2->6,5->5,8->9] | 0.86 | 325.402985075 | 21 | 67 | 0 | 0 |
| Path 255 | C00026->C00624:[1->1,2->2,3->4,3->6,5->5,8->9] | 0.86 | 168.505882353 | 9 | 85 | 0 | 0 |
| Path 256 | C00026->C00624:[1->1,2->2,3->4,3->6,5->5,8->9] | 0.86 | 204.792307692 | 12 | 130 | 0 | 0 |
| Path 257 | C00026->C00624:[1->1,2->2,5->5,8->9] | 0.57 | 364.882352941 | 14 | 34 | 0 | 0 |
| Path 258 | C00026->C00624:[1->1,1->10,2->2,2->6,3->4,5->5,8->9] | 1.00 | 220.818965517 | 17 | 116 | 0 | 0 |
| Path 259 | C00026->C00624:[1->1,1->6,2->10,2->2,5->5,8->9] | 0.86 | 451.585365854 | 19 | 41 | 0 | 0 |
| Path 260 | C00026->C00624:[1->1,1->10,2->2,2->6,3->4,5->5,8->9] | 1.00 | 149.372670807 | 15 | 161 | 0 | 0 |
| Path 261 | C00026->C00624:[1->1,2->2,3->4,5->5,8->9] | 0.71 | 251.654545455 | 13 | 165 | 0 | 0 |
| Path 262 | C00026->C00624:[1->1,2->2,3->4,3->6,5->5,8->9] | 0.86 | 243.58 | 8 | 150 | 0 | 0 |
| Path 263 | C00026->C00624:[1->1,1->10,2->2,2->6,5->5,8->9] | 0.86 | 193.283185841 | 19 | 113 | 0 | 0 |
| Path 264 | C00026->C00624:[1->1,1->10,2->2,2->6,5->5,8->9] | 0.86 | 181.829545455 | 13 | 88 | 0 | 0 |
| Path 265 | C00026->C00624:[1->1,2->2,3->4,3->6,5->5,8->9] | 0.86 | 345.558139535 | 10 | 86 | 0 | 0 |
| Path 266 | C00026->C00624:[1->1,1->10,2->2,2->6,3->4,5->5,8->9] | 1.00 | 274.72392638 | 17 | 163 | 0 | 0 |
| Path 267 | C00026->C00624:[1->1,2->2,5->10,5->5,8->4,8->6,8->9] | 1.00 | 196.220930233 | 15 | 86 | 0 | 0 |
| Path 268 | C00026->C00624:[1->1,1->10,2->2,2->6,5->5,8->9] | 0.86 | 383.627906977 | 15 | 43 | 0 | 0 |
| Path 269 | C00026->C00624:[1->1,1->4,2->2,5->5,8->9] | 0.71 | 184.712871287 | 17 | 101 | 0 | 0 |
| Path 270 | C00026->C00624:[1->1,1->6,2->10,2->2,3->4,3->6,5->5,8->9] | 1.00 | 217.44214876 | 17 | 242 | 0 | 0 |
| Path 271 | C00026->C00624:[1->1,1->6,2->10,2->2,3->4,3->6,5->5,8->9] | 1.00 | 214.732142857 | 15 | 224 | 0 | 0 |
| Path 272 | C00026->C00624:[5->10,8->4,8->6] | 0.43 | 602.357142857 | 10 | 14 | 0 | 0 |
| Path 273 | C00026->C00624:[5->10,8->4,8->6] | 0.43 | 169.734177215 | 12 | 79 | 0 | 0 |
| Path 274 | C00026->C00624:[1->1,2->2,5->10,5->5,8->6,8->9] | 0.86 | 330.087719298 | 18 | 57 | 0 | 0 |
| Path 275 | C00026->C00624:[1->1,1->10,2->2,2->6,5->5,8->9] | 0.86 | 190.222222222 | 15 | 90 | 0 | 0 |
| Path 276 | C00026->C00624:[3->4,3->6] | 0.29 | 206.440366972 | 15 | 109 | 0 | 0 |
| Path 277 | C00026->C00624:[1->1,2->2,3->4,3->6,5->10,5->5,8->6,8->9] | 1.00 | 219.353982301 | 18 | 226 | 0 | 2 |
| Path 278 | C00026->C00624:[1->1,1->6,2->10,2->2,3->4,3->6,5->5,8->9] | 1.00 | 275.54954955 | 20 | 222 | 0 | 0 |
| Path 279 | C00026->C00624:[1->6,2->10,3->4,3->6] | 0.43 | 167.12568306 | 16 | 183 | 0 | 0 |
| Path 280 | C00026->C00624:[1->1,2->10,2->2,3->4,3->6,5->5,8->9] | 1.00 | 270.907216495 | 19 | 194 | 0 | 0 |
| Path 281 | C00026->C00624:[1->1,2->2,3->4,5->5,8->6,8->9] | 0.86 | 245.32885906 | 11 | 149 | 0 | 0 |
| Path 282 | C00026->C00624:[1->1,1->6,2->10,2->2,5->5,8->9] | 0.86 | 323.0 | 18 | 57 | 0 | 0 |
| Path 283 | C00026->C00624:[1->1,1->6,2->10,2->2,3->4,5->5,8->9] | 1.00 | 268.506493506 | 15 | 154 | 0 | 0 |
| Path 284 | C00026->C00624:[1->1,1->4,2->2,5->10,5->5,8->6,8->9] | 1.00 | 193.811320755 | 19 | 106 | 0 | 0 |
| Path 285 | C00026->C00624:[1->1,2->2,5->10,5->5,8->6,8->9] | 0.86 | 172.139240506 | 12 | 79 | 0 | 0 |
| Path 286 | C00026->C00624:[1->1,2->2,3->4,3->6,5->5,8->9] | 0.86 | 166.366071429 | 15 | 112 | 0 | 0 |
| Path 287 | C00026->C00624:[1->1,1->4,2->2,5->5,8->9] | 0.71 | 165.865853659 | 15 | 82 | 0 | 0 |
| Path 288 | C00026->C00624:[1->1,1->6,2->10,2->2,5->5,8->9] | 0.86 | 159.924050633 | 11 | 79 | 0 | 0 |
| Path 289 | C00026->C00624:[1->1,1->10,2->2,2->6,3->4,5->5,8->9] | 1.00 | 216.857142857 | 22 | 140 | 0 | 0 |
| Path 290 | C00026->C00624:[1->1,2->2,3->4,5->5,8->9] | 0.71 | 371.01980198 | 12 | 101 | 0 | 0 |
| Path 291 | C00026->C00624:[1->1,2->2,5->10,5->5,8->6,8->9] | 0.86 | 160.307692308 | 11 | 78 | 0 | 0 |
| Path 292 | C00026->C00624:[1->1,2->2,3->4,3->6,5->5,8->9] | 0.86 | 195.177083333 | 16 | 96 | 0 | 0 |
| Path 293 | C00026->C00624:[1->1,1->6,2->10,2->2,3->4,5->5,8->9] | 1.00 | 278.694915254 | 16 | 59 | 0 | 0 |
| Path 294 | C00026->C00624:[1->1,1->4,2->2,5->10,5->5,8->6,8->9] | 1.00 | 433.571428571 | 14 | 35 | 0 | 0 |
| Path 295 | C00026->C00624:[1->1,1->6,2->10,2->2,3->4,5->5,8->9] | 1.00 | 136.80794702 | 12 | 151 | 0 | 0 |
| Path 296 | C00026->C00624:[1->1,2->2,3->4,3->6,5->5,8->9] | 0.86 | 219.795744681 | 23 | 235 | 0 | 2 |
| Path 297 | C00026->C00624:[1->1,2->2,3->4,3->6,5->10,5->5,8->9] | 1.00 | 218.49339207 | 19 | 227 | 0 | 2 |
| Path 298 | C00026->C00624:[1->1,1->4,2->2,5->5,8->9] | 0.71 | 395.405405405 | 17 | 37 | 0 | 0 |
| Path 299 | C00026->C00624:[1->1,1->6,2->10,2->2,3->4,5->5,8->9] | 1.00 | 263.546875 | 21 | 128 | 0 | 0 |
| Path 300 | C00026->C00624:[1->1,1->10,2->2,2->6,3->4,5->5,8->9] | 1.00 | 171.957831325 | 20 | 166 | 0 | 0 |
| Path 301 | C00026->C00624:[1->1,1->4,2->2,5->5,8->9] | 0.71 | 166.158536585 | 16 | 82 | 0 | 0 |
| Path 302 | C00026->C00624:[1->1,1->6,2->10,2->2,3->4,5->5,8->9] | 1.00 | 266.691860465 | 17 | 172 | 0 | 0 |
| Path 303 | C00026->C00624:[5->10,8->6] | 0.29 | 602.333333333 | 6 | 9 | 0 | 0 |
| Path 304 | C00026->C00624:[1->1,1->10,2->2,2->6,3->4,5->5,8->9] | 1.00 | 377.193181818 | 9 | 88 | 0 | 0 |
| Path 305 | C00026->C00624:[5->10,8->6] | 0.29 | 133.505617978 | 9 | 89 | 0 | 0 |
| Path 306 | C00026->C00624:[1->1,2->2,5->10,5->5,8->6,8->9] | 0.86 | 326.446428571 | 18 | 56 | 0 | 0 |
| Path 307 | C00026->C00624:[1->1,2->2,3->4,5->5,8->9] | 0.71 | 112.213793103 | 9 | 145 | 0 | 0 |
| Path 308 | C00026->C00624:[1->1,2->2,3->4,5->5,8->9] | 0.71 | 234.593495935 | 18 | 123 | 0 | 0 |
| Path 309 | C00026->C00624:[1->1,2->2,5->10,5->5,8->4,8->6,8->9] | 1.00 | 228.391304348 | 17 | 115 | 0 | 0 |
| Path 310 | C00026->C00624:[1->1,2->2,3->4,5->5,8->9] | 0.71 | 189.485714286 | 15 | 105 | 0 | 0 |
| Path 311 | C00026->C00624:[1->1,2->2,3->4,5->10,5->5,8->6,8->9] | 1.00 | 389.087378641 | 13 | 103 | 0 | 0 |
| Path 312 | C00026->C00624:[1->1,1->6,2->10,2->2,3->4,5->5,8->9] | 1.00 | 266.316129032 | 16 | 155 | 0 | 0 |
| Path 313 | C00026->C00624:[1->1,2->2,3->4,5->10,5->5,8->6,8->9] | 1.00 | 380.717948718 | 7 | 78 | 0 | 0 |
| Path 314 | C00026->C00624:[1->10,2->10,3->6,8->4] | 0.43 | 474.428571429 | 17 | 42 | 0 | 0 |
| Path 315 | C00026->C00624:[1->1,2->2,3->4,5->10,5->5,8->6,8->9] | 1.00 | 397.266666667 | 15 | 105 | 0 | 0 |
| Path 316 | C00026->C00624:[5->10,8->4,8->6] | 0.43 | 194.6875 | 13 | 80 | 0 | 0 |
| Path 317 | C00026->C00624:[1->1,1->6,2->10,2->2,3->4,5->5,8->9] | 1.00 | 354.092783505 | 9 | 97 | 0 | 0 |
| Path 318 | C00026->C00624:[1->10,3->6,8->4] | 0.43 | 185.527472527 | 15 | 91 | 0 | 0 |
| Path 319 | C00026->C00624:[3->4,3->6] | 0.29 | 225.987012987 | 18 | 154 | 0 | 0 |
| Path 320 | C00026->C00624:[1->1,2->2,3->4,5->10,5->5,8->6,8->9] | 1.00 | 390.320754717 | 15 | 106 | 0 | 0 |
| Path 321 | C00026->C00624:[1->1,2->2,2->6,3->4,5->5,8->9] | 0.86 | 153.76875 | 15 | 160 | 0 | 0 |
| Path 322 | C00026->C00624:[1->1,2->2,3->4,3->6,5->5,8->9] | 0.86 | 197.025477707 | 18 | 157 | 0 | 0 |
| Path 323 | C00026->C00624:[1->1,1->4,2->2,5->5,8->9] | 0.71 | 184.95049505 | 18 | 101 | 0 | 0 |
| Path 324 | C00026->C00624:[1->1,1->10,2->2,2->6,3->4,5->5,8->9] | 1.00 | 383.961538462 | 10 | 26 | 0 | 0 |
| Path 325 | C00026->C00624:[1->1,2->2,2->6,3->4,5->5,8->9] | 0.86 | 163.617977528 | 17 | 178 | 0 | 0 |
| Path 326 | C00026->C00624:[1->1,2->2,5->10,5->5,8->6,8->9] | 0.86 | 174.607843137 | 17 | 102 | 0 | 0 |
| Path 327 | C00026->C00624:[1->1,1->4,2->2,5->5,8->9] | 0.71 | 170.301204819 | 15 | 83 | 0 | 0 |
| Path 328 | C00026->C00624:[1->1,2->2,3->4,5->5,8->9] | 0.71 | 128.642384106 | 14 | 151 | 0 | 0 |
| Path 329 | C00026->C00624:[1->1,1->10,2->2,3->4,3->6,5->5,8->9] | 1.00 | 222.505263158 | 13 | 95 | 0 | 0 |
| Path 330 | C00026->C00624:[5->10,8->6] | 0.29 | 674.625 | 5 | 8 | 0 | 0 |
| Path 331 | C00026->C00624:[1->1,1->6,2->10,2->2,3->4,5->5,8->9] | 1.00 | 180.629213483 | 16 | 89 | 0 | 0 |
| Path 332 | C00026->C00624:[1->1,2->10,2->2,3->4,3->6,5->5,8->9] | 1.00 | 272.870786517 | 16 | 178 | 0 | 0 |
| Path 333 | C00026->C00624:[3->4,3->6] | 0.29 | 207.825396825 | 17 | 126 | 0 | 0 |
| Path 334 | C00026->C00624:[1->1,2->2,3->4,5->5,8->6,8->9] | 0.86 | 389.763157895 | 13 | 38 | 0 | 0 |
| Path 335 | C00026->C00624:[1->1,1->10,2->2,2->6,5->5,8->9] | 0.86 | 329.96969697 | 20 | 66 | 0 | 0 |
| Path 336 | C00026->C00624:[1->1,2->2,3->4,5->10,5->5,8->6,8->9] | 1.00 | 150.674285714 | 18 | 175 | 0 | 0 |
| Path 337 | C00026->C00624:[1->1,1->6,2->10,2->2,3->4,5->5,8->9] | 1.00 | 131.679012346 | 9 | 81 | 0 | 0 |
| Path 338 | C00026->C00624:[1->1,1->6,2->10,2->2,3->4,3->6,5->5,8->9] | 1.00 | 265.176470588 | 15 | 153 | 0 | 0 |
| Path 339 | C00026->C00624:[1->1,1->10,2->2,2->6,3->4,5->5,8->9] | 1.00 | 161.432432432 | 20 | 185 | 0 | 0 |
| Path 340 | C00026->C00624:[1->1,1->4,2->2,5->10,5->5,8->6,8->9] | 1.00 | 194.037735849 | 20 | 106 | 0 | 0 |
| Path 341 | C00026->C00624:[1->1,2->2,3->4,3->6,5->5,8->9] | 0.86 | 176.539215686 | 11 | 102 | 0 | 0 |
| Path 342 | C00026->C00624:[1->1,1->6,2->10,2->2,3->4,5->5,8->9] | 1.00 | 152.114285714 | 15 | 105 | 0 | 0 |
| Path 343 | C00026->C00624:[1->1,1->6,2->10,2->2,3->4,3->6,5->5,8->9] | 1.00 | 263.701754386 | 17 | 171 | 0 | 0 |
| Path 344 | C00026->C00624:[1->1,1->6,2->10,2->2,5->5,8->9] | 0.86 | 217.91509434 | 20 | 106 | 0 | 0 |
| Path 345 | C00026->C00624:[1->1,1->10,2->2,2->6,5->5,8->9] | 0.86 | 188.769911504 | 20 | 113 | 0 | 0 |
| Path 346 | C00026->C00624:[1->1,2->2,3->4,5->5,8->6,8->9] | 0.86 | 359.466019417 | 12 | 103 | 0 | 0 |
| Path 347 | C00026->C00624:[1->1,1->10,2->2,2->6,3->4,5->5,8->9] | 1.00 | 245.992248062 | 17 | 129 | 0 | 0 |
| Path 348 | C00026->C00624:[5->10,8->4,8->6] | 0.43 | 427.708333333 | 14 | 48 | 0 | 0 |
| Path 349 | C00026->C00624:[1->1,2->2,3->4,5->10,5->5,8->6,8->9] | 1.00 | 405.375 | 8 | 16 | 0 | 0 |
| Path 350 | C00026->C00624:[1->1,2->2,5->5,8->4,8->9] | 0.71 | 155.777777778 | 12 | 81 | 0 | 0 |
| Path 351 | C00026->C00624:[1->1,1->10,2->2,2->6,5->5,8->9] | 0.86 | 437.333333333 | 20 | 48 | 0 | 0 |
| Path 352 | C00026->C00624:[1->1,1->4,2->2,5->5,8->9] | 0.71 | 159.234042553 | 12 | 94 | 0 | 0 |
| Path 353 | C00026->C00624:[1->1,2->2,5->5,8->9] | 0.57 | 322.888888889 | 9 | 27 | 0 | 0 |
| Path 354 | C00026->C00624:[2->2,3->1,3->4,5->5,8->9] | 0.71 | 242.898148148 | 17 | 108 | 0 | 0 |
| Path 355 | C00026->C00624:[1->1,1->4,2->2,5->5,8->9] | 0.71 | 394.756756757 | 16 | 37 | 0 | 0 |
| Path 356 | C00026->C00624:[1->1,1->10,2->2,2->6,5->5,8->9] | 0.86 | 190.241071429 | 19 | 112 | 0 | 0 |
| Path 357 | C00026->C00624:[1->1,1->6,2->10,2->2,3->4,5->5,8->9] | 1.00 | 264.739884393 | 18 | 173 | 0 | 0 |
| Path 358 | C00026->C00624:[1->6,2->10,3->4] | 0.43 | 185.781990521 | 17 | 211 | 0 | 0 |
| Path 359 | C00026->C00624:[1->1,2->2,3->4,5->5,8->9] | 0.71 | 169.111111111 | 10 | 99 | 0 | 0 |
| Path 360 | C00026->C00624:[1->1,2->2,5->5,8->9] | 0.57 | 112.97260274 | 8 | 73 | 0 | 0 |
| Path 361 | C00026->C00624:[1->1,1->4,2->2,5->10,5->5,8->6,8->9] | 1.00 | 199.634146341 | 22 | 123 | 0 | 0 |
| Path 362 | C00026->C00624:[5->10,8->4,8->6] | 0.43 | 459.142857143 | 15 | 35 | 0 | 0 |
| Path 363 | C00026->C00624:[2->10,3->4,3->6] | 0.43 | 247.509933775 | 19 | 151 | 0 | 0 |
| Path 364 | C00026->C00624:[1->1,2->2,3->4,3->6,5->5,8->9] | 0.86 | 250.446153846 | 11 | 195 | 0 | 0 |
| Path 365 | C00026->C00624:[1->1,1->4,2->2,5->10,5->5,8->6,8->9] | 1.00 | 347.13559322 | 20 | 59 | 0 | 0 |
| Path 366 | C00026->C00624:[1->1,1->6,2->10,2->2,3->4,5->5,8->9] | 1.00 | 166.2625 | 19 | 160 | 0 | 0 |
| Path 367 | C00026->C00624:[1->1,1->6,2->10,2->2,5->5,8->9] | 0.86 | 253.095238095 | 20 | 105 | 0 | 0 |
| Path 368 | C00026->C00624:[1->1,1->10,2->2,2->6,5->5,8->9] | 0.86 | 439.306122449 | 20 | 49 | 0 | 0 |
| Path 369 | C00026->C00624:[1->1,1->6,2->10,2->2,5->5,8->9] | 0.86 | 459.107142857 | 20 | 56 | 0 | 0 |
| Path 370 | C00026->C00624:[1->1,2->2,5->5,8->9] | 0.57 | 149.1875 | 13 | 80 | 0 | 0 |
| Path 371 | C00026->C00624:[1->1,1->10,2->2,2->6,3->4,5->5,8->9] | 1.00 | 178.64 | 16 | 175 | 0 | 0 |
| Path 372 | C00026->C00624:[5->10,8->6] | 0.29 | 411.291666667 | 8 | 24 | 0 | 0 |
| Path 373 | C00026->C00624:[3->4,3->6] | 0.29 | 149.505813953 | 16 | 172 | 0 | 0 |
| Path 374 | C00026->C00624:[1->1,1->6,2->10,2->2,3->4,5->5,8->9] | 1.00 | 226.205574913 | 20 | 287 | 0 | 0 |
| Path 375 | C00026->C00624:[1->1,1->6,2->10,2->2,5->5,8->9] | 0.86 | 211.113636364 | 18 | 88 | 0 | 0 |
| Path 376 | C00026->C00624:[1->1,1->4,2->2,5->10,5->5,8->6,8->9] | 1.00 | 190.571428571 | 19 | 105 | 0 | 0 |
| Path 377 | C00026->C00624:[1->1,1->6,2->10,2->2,5->5,8->9] | 0.86 | 188.483606557 | 19 | 122 | 0 | 0 |
| Path 378 | C00026->C00624:[1->1,2->2,5->10,5->5,8->6,8->9] | 0.86 | 178.097087379 | 17 | 103 | 0 | 0 |
| Path 379 | C00026->C00624:[1->1,2->2,3->4,5->10,5->5,8->6,8->9] | 1.00 | 187.591954023 | 20 | 174 | 0 | 0 |
| Path 380 | C00026->C00624:[5->10,8->4,8->6] | 0.43 | 420.606060606 | 13 | 33 | 0 | 0 |
| Path 381 | C00026->C00624:[1->1,1->4,2->2,5->10,5->5,8->6,8->9] | 1.00 | 199.43902439 | 21 | 123 | 0 | 0 |
| Path 382 | C00026->C00624:[1->1,1->6,2->10,2->2,3->4,5->5,8->9] | 1.00 | 347.43220339 | 16 | 118 | 0 | 0 |
| Path 383 | C00026->C00624:[5->10,8->6] | 0.29 | 410.192307692 | 8 | 26 | 0 | 0 |
| Path 384 | C00026->C00624:[1->1,2->2,3->4,5->5,8->9] | 0.71 | 219.526785714 | 17 | 112 | 0 | 0 |
| Path 385 | C00026->C00624:[1->1,2->2,3->4,5->10,5->5,8->6,8->9] | 1.00 | 143.198675497 | 13 | 151 | 0 | 0 |
| Path 386 | C00026->C00624:[1->1,1->6,2->10,2->2,3->4,5->5,8->9] | 1.00 | 228.048951049 | 21 | 286 | 0 | 0 |
| Path 387 | C00026->C00624:[1->1,2->2,3->4,5->10,5->5,8->6,8->9] | 1.00 | 257.452380952 | 19 | 126 | 0 | 0 |
| Path 388 | C00026->C00624:[1->1,1->10,2->2,2->6,3->4,5->5,8->9] | 1.00 | 213.217391304 | 16 | 115 | 0 | 0 |
| Path 389 | C00026->C00624:[1->1,1->6,2->10,2->2,3->4,5->5,8->9] | 1.00 | 172.81920904 | 21 | 177 | 0 | 0 |
| Path 390 | C00026->C00624:[1->1,1->6,2->10,2->2,3->4,3->6,5->5,8->9] | 1.00 | 219.593360996 | 18 | 241 | 0 | 0 |
| Path 391 | C00026->C00624:[2->2,3->1,3->4,5->5,8->9] | 0.71 | 231.752380952 | 16 | 105 | 0 | 0 |
| Path 392 | C00026->C00624:[5->10,8->4,8->6] | 0.43 | 213.393617021 | 13 | 94 | 0 | 0 |
| Path 393 | C00026->C00624:[1->1,2->2,5->5,8->4,8->9] | 0.71 | 205.322916667 | 15 | 96 | 0 | 0 |
| Path 394 | C00026->C00624:[8->4] | 0.14 | 481.931034483 | 12 | 29 | 0 | 0 |
| Path 395 | C00026->C00624:[1->6,2->10,3->4,3->6] | 0.43 | 226.678832117 | 17 | 137 | 0 | 0 |
| Path 396 | C00026->C00624:[1->1,2->2,3->4,5->5,8->9] | 0.71 | 202.636363636 | 5 | 11 | 0 | 0 |
| Path 397 | C00026->C00624:[1->1,1->10,2->2,2->6,5->5,8->9] | 0.86 | 392.19047619 | 14 | 42 | 0 | 0 |
| Path 398 | C00026->C00624:[8->4] | 0.14 | 177.550561798 | 10 | 89 | 0 | 0 |
| Path 399 | C00026->C00624:[1->1,1->10,2->2,2->6,3->4,5->5,8->9] | 1.00 | 275.566666667 | 20 | 180 | 0 | 0 |
| Path 400 | C00026->C00624:[1->1,2->2,5->5,8->9] | 0.57 | 144.316455696 | 13 | 79 | 0 | 0 |
| Path 401 | C00026->C00624:[1->1,1->6,2->10,2->2,3->4,5->5,8->9] | 1.00 | 270.011695906 | 18 | 171 | 0 | 0 |
| Path 402 | C00026->C00624:[1->1,1->10,2->2,2->6,5->5,8->9] | 0.86 | 399.795454545 | 16 | 44 | 0 | 0 |
| Path 403 | C00026->C00624:[1->1,2->2,3->4,5->5,8->9] | 0.71 | 242.333333333 | 15 | 150 | 0 | 0 |
| Path 404 | C00026->C00624:[3->4,3->6] | 0.29 | 171.655 | 17 | 200 | 0 | 0 |
| Path 405 | C00026->C00624:[5->10,8->6] | 0.29 | 382.8 | 7 | 25 | 0 | 0 |
| Path 406 | C00026->C00624:[1->1,1->10,2->2,2->6,3->4,5->5,8->9] | 1.00 | 386.760683761 | 18 | 117 | 0 | 0 |
| Path 407 | C00026->C00624:[1->1,2->2,3->4,5->5,8->9] | 0.71 | 377.74 | 13 | 100 | 0 | 0 |
| Path 408 | C00026->C00624:[1->1,1->10,2->2,2->6,3->4,5->5,8->9] | 1.00 | 218.244604317 | 21 | 139 | 0 | 0 |
| Path 409 | C00026->C00624:[1->1,1->6,2->10,2->2,3->4,3->6,5->5,8->9] | 1.00 | 267.974358974 | 18 | 195 | 0 | 0 |
| Path 410 | C00026->C00624:[1->1,1->10,2->2,2->6,5->5,8->9] | 0.86 | 218.308510638 | 19 | 94 | 0 | 0 |
| Path 411 | C00026->C00624:[1->1,1->6,2->10,2->2,3->4,5->5,8->9] | 1.00 | 159.891752577 | 20 | 194 | 0 | 0 |
| Path 412 | C00026->C00624:[1->1,2->2,3->4,3->6,5->5,8->9] | 0.86 | 370.048192771 | 10 | 83 | 0 | 0 |
| Path 413 | C00026->C00624:[1->1,1->10,2->2,2->6,3->4,5->5,8->9] | 1.00 | 150.15625 | 14 | 160 | 0 | 0 |
| Path 414 | C00026->C00624:[1->1,1->6,2->10,2->2,3->4,3->6,5->5,8->9] | 1.00 | 219.403100775 | 20 | 258 | 0 | 0 |
| Path 415 | C00026->C00624:[1->1,1->10,2->2,2->6,5->5,8->9] | 0.86 | 180.056179775 | 14 | 89 | 0 | 0 |
| Path 416 | C00026->C00624:[1->1,2->2,3->4,3->6,5->10,5->5,8->6,8->9] | 1.00 | 211.535714286 | 16 | 224 | 0 | 2 |
| Path 417 | C00026->C00624:[1->1,2->2,3->4,5->10,5->5,8->6,8->9] | 1.00 | 208.054263566 | 19 | 129 | 0 | 0 |
| Path 418 | C00026->C00624:[8->4] | 0.14 | 378.441860465 | 11 | 43 | 0 | 0 |
| Path 419 | C00026->C00624:[1->1,1->10,2->2,3->4,3->6,5->5,8->9] | 1.00 | 403.537634409 | 14 | 93 | 0 | 0 |
| Path 420 | C00026->C00624:[1->1,1->6,2->10,2->2,3->4,3->6,5->5,8->9] | 1.00 | 383.579439252 | 16 | 107 | 0 | 0 |
| Path 421 | C00026->C00624:[8->4] | 0.14 | 140.166666667 | 10 | 78 | 0 | 0 |
| Path 422 | C00026->C00624:[1->1,2->2,3->4,5->5,8->9] | 0.71 | 131.309210526 | 14 | 152 | 0 | 0 |
| Path 423 | C00026->C00624:[1->1,1->6,2->10,2->2,5->5,8->9] | 0.86 | 214.904761905 | 20 | 105 | 0 | 0 |
| Path 424 | C00026->C00624:[1->1,1->10,2->2,3->4,3->6,5->5,8->9] | 1.00 | 241.056603774 | 20 | 106 | 0 | 0 |
| Path 425 | C00026->C00624:[1->1,2->2,3->4,5->5,8->9] | 0.71 | 209.965065502 | 19 | 229 | 0 | 2 |
| Path 426 | C00026->C00624:[1->1,2->2,3->4,3->6,5->10,5->5,8->6,8->9] | 1.00 | 222.148471616 | 20 | 229 | 0 | 2 |
| Path 427 | C00026->C00624:[1->1,2->10,2->2,3->4,3->6,5->5,8->9] | 1.00 | 276.11299435 | 17 | 177 | 0 | 0 |
| Path 428 | C00026->C00624:[1->1,1->10,2->2,2->6,3->4,5->5,8->9] | 1.00 | 453.21875 | 16 | 32 | 0 | 0 |
| Path 429 | C00026->C00624:[1->1,2->2,5->5,8->9] | 0.57 | 359.757575758 | 14 | 33 | 0 | 0 |
| Path 430 | C00026->C00624:[1->1,2->2,3->4,5->10,5->5,8->6,8->9] | 1.00 | 247.161849711 | 19 | 173 | 0 | 0 |
| Path 431 | C00026->C00624:[1->1,1->10,2->2,2->6,3->4,5->5,8->9] | 1.00 | 158.675675676 | 21 | 185 | 0 | 0 |
| Path 432 | C00026->C00624:[1->1,2->2,3->4,5->10,5->5,8->6,8->9] | 1.00 | 396.906542056 | 16 | 107 | 0 | 0 |
| Path 433 | C00026->C00624:[1->1,1->4,2->2,5->10,5->5,8->6,8->9] | 1.00 | 350.25 | 20 | 60 | 0 | 0 |
