# Supplementary material for: 13C-metabolic flux ratio and novel carbon path analyses confirmed that Trichoderma reesei uses primarily the respirative pathway also on the preferred carbon source glucose
Source: BMC Syst Biol. 2009 Oct 29;3:104. doi: 10.1186/1752-0509-3-104 (PMC2776023; doi:10.1186/1752-0509-3-104)
Supplement: Additional file 1 — Pathways discovered in ReTrace carbon path analysis. Graphical and tabular representations of amino acid synthesis pathways discovered in ReTrace carbon path analysis [21]. Self-contained web site: unpack zip archive and open index.html with a web browser. [file 1752-0509-3-104-S1.zip › AF1-treesei/pathways-C00031-to-C00183.html]

Pathways from C00031 to C00183


**Pathways from C00031 to C00183**

**Sources:** D-Glucose; (C00031)

**Target:**L-Valine; (C00183)

|  | Composite mapping | Z | Average score | Rpairs | Reactions | Zero scores | Scores under threshold |
| --- | --- | --- | --- | --- | --- | --- | --- |
| Path 1 | C00031->C00183:[4->3,7->6,9->1] | 0.60 | 400.15 | 14 | 40 | 0 | 1 |
| Path 2 | C00031->C00183:[2->3,4->2,7->5,7->6,9->1] | 1.00 | 403.476744186 | 29 | 86 | 0 | 1 |
| Path 3 | C00031->C00183:[4->3,7->5,7->6,9->1,9->2] | 1.00 | 403.34 | 19 | 50 | 0 | 1 |
| Path 4 | C00031->C00183:[4->3,7->5,7->6,9->1,9->2] | 1.00 | 386.387755102 | 18 | 49 | 0 | 1 |
| Path 5 | C00031->C00183:[4->3,7->5,7->6,9->1,9->2] | 1.00 | 409.79245283 | 20 | 53 | 0 | 1 |
| Path 6 | C00031->C00183:[7->5,9->1,9->3] | 0.60 | 286.035087719 | 14 | 57 | 0 | 1 |
| Path 7 | C00031->C00183:[4->2,7->6,9->2] | 0.40 | 441.228571429 | 19 | 35 | 0 | 1 |
| Path 8 | C00031->C00183:[4->3,7->3,7->6,9->1] | 0.60 | 286.611111111 | 20 | 72 | 0 | 2 |
| Path 9 | C00031->C00183:[4->2,4->5] | 0.40 | 462.859375 | 21 | 64 | 0 | 1 |
| Path 10 | C00031->C00183:[4->3,7->5,7->6,9->1,9->2] | 1.00 | 467.943661972 | 22 | 71 | 0 | 1 |
| Path 11 | C00031->C00183:[4->3,7->6,9->1] | 0.60 | 395.880952381 | 14 | 42 | 0 | 1 |
| Path 12 | C00031->C00183:[2->3,4->2,7->5,7->6,9->1] | 1.00 | 482.184615385 | 28 | 65 | 0 | 1 |
| Path 13 | C00031->C00183:[4->2,4->6] | 0.40 | 456.237288136 | 22 | 59 | 0 | 1 |
| Path 14 | C00031->C00183:[7->6,9->2] | 0.40 | 343.4 | 9 | 20 | 0 | 1 |
| Path 15 | C00031->C00183:[4->3,7->3,7->5,7->6,9->1,9->2] | 1.00 | 304.282051282 | 23 | 78 | 0 | 2 |
| Path 16 | C00031->C00183:[1->1,2->3,4->5,7->6,9->2] | 1.00 | 453.779661017 | 22 | 59 | 0 | 1 |
| Path 17 | C00031->C00183:[7->3,7->6,9->1] | 0.60 | 374.028571429 | 12 | 35 | 0 | 1 |
| Path 18 | C00031->C00183:[4->3,7->5,7->6,9->1,9->2] | 1.00 | 365.793650794 | 23 | 63 | 0 | 2 |
| Path 19 | C00031->C00183:[4->2,4->3,7->5,7->6,9->1] | 1.00 | 342.941176471 | 20 | 68 | 0 | 1 |
| Path 20 | C00031->C00183:[2->3,4->2,7->5,7->6,9->1] | 1.00 | 421.369047619 | 26 | 84 | 0 | 1 |
| Path 21 | C00031->C00183:[1->1,1->2,2->3,4->5,4->6] | 1.00 | 480.655172414 | 20 | 58 | 0 | 1 |
| Path 22 | C00031->C00183:[4->3,7->5,7->6,9->1,9->2] | 1.00 | 322.535714286 | 26 | 84 | 0 | 2 |
| Path 23 | C00031->C00183:[2->3,4->2,7->5,7->6,9->1] | 1.00 | 374.37804878 | 27 | 82 | 0 | 1 |
| Path 24 | C00031->C00183:[4->2,4->5] | 0.40 | 472.127272727 | 22 | 55 | 0 | 1 |
| Path 25 | C00031->C00183:[4->2,4->6] | 0.40 | 462.859375 | 21 | 64 | 0 | 1 |
| Path 26 | C00031->C00183:[2->3,4->2,7->5,7->6,9->1] | 1.00 | 508.1 | 24 | 60 | 0 | 1 |
| Path 27 | C00031->C00183:[4->3,7->3,7->5,7->6,9->1,9->2] | 1.00 | 384.757142857 | 28 | 70 | 0 | 2 |
| Path 28 | C00031->C00183:[4->2,4->6] | 0.40 | 463.05 | 22 | 60 | 0 | 1 |
| Path 29 | C00031->C00183:[4->3,7->5,7->6,9->1,9->2] | 1.00 | 409.180555556 | 30 | 72 | 0 | 2 |
| Path 30 | C00031->C00183:[4->3,7->6,9->1] | 0.60 | 285.287671233 | 19 | 73 | 0 | 2 |
| Path 31 | C00031->C00183:[7->6,9->1,9->3] | 0.60 | 290.315789474 | 14 | 57 | 0 | 1 |
| Path 32 | C00031->C00183:[4->3,7->5,7->6,9->1,9->2] | 1.00 | 392.294117647 | 18 | 51 | 0 | 1 |
| Path 33 | C00031->C00183:[7->3,7->5,7->6,9->1,9->2] | 1.00 | 394.5 | 20 | 48 | 0 | 1 |
| Path 34 | C00031->C00183:[7->6,9->1,9->3] | 0.60 | 314.112903226 | 16 | 62 | 0 | 1 |
| Path 35 | C00031->C00183:[7->6,9->2] | 0.40 | 384.02173913 | 19 | 46 | 0 | 1 |
| Path 36 | C00031->C00183:[4->2,7->5,9->2] | 0.40 | 374.720930233 | 18 | 43 | 0 | 1 |
| Path 37 | C00031->C00183:[2->3,7->5,7->6,9->1,9->2] | 1.00 | 479.979166667 | 18 | 48 | 0 | 1 |
| Path 38 | C00031->C00183:[2->3,7->5,7->6,9->1,9->2] | 1.00 | 480.4375 | 19 | 48 | 0 | 1 |
| Path 39 | C00031->C00183:[1->1,2->3,4->5,7->6,9->2] | 1.00 | 471.672727273 | 21 | 55 | 0 | 1 |
| Path 40 | C00031->C00183:[1->1,2->3,4->5,7->6,9->2] | 1.00 | 462.568965517 | 18 | 58 | 0 | 1 |
| Path 41 | C00031->C00183:[4->3,7->5,7->6,9->1,9->2] | 1.00 | 402.307692308 | 21 | 52 | 0 | 1 |
| Path 42 | C00031->C00183:[1->1,1->2,2->3,4->5,4->6] | 1.00 | 464.634615385 | 16 | 52 | 0 | 1 |
| Path 43 | C00031->C00183:[4->3,7->5,7->6,9->1,9->2] | 1.00 | 395.632653061 | 18 | 49 | 0 | 1 |
| Path 44 | C00031->C00183:[4->3,7->6,9->1] | 0.60 | 386.538461538 | 13 | 39 | 0 | 1 |
| Path 45 | C00031->C00183:[2->3,4->2,7->5,7->6,9->1] | 1.00 | 418.404494382 | 27 | 89 | 0 | 1 |
| Path 46 | C00031->C00183:[7->5,9->1,9->3] | 0.60 | 305.355932203 | 16 | 59 | 0 | 1 |
| Path 47 | C00031->C00183:[2->3,7->5,7->6,9->1,9->2] | 1.00 | 414.416666667 | 18 | 48 | 0 | 1 |
| Path 48 | C00031->C00183:[1->1,2->3,4->5,7->6,9->2] | 1.00 | 470.508474576 | 19 | 59 | 0 | 1 |
| Path 49 | C00031->C00183:[7->6,9->2] | 0.40 | 404.2 | 18 | 40 | 0 | 1 |
| Path 50 | C00031->C00183:[1->1,2->3,4->5,7->6,9->2] | 1.00 | 462.759259259 | 19 | 54 | 0 | 1 |
| Path 51 | C00031->C00183:[4->3,7->5,7->6,9->1,9->2] | 1.00 | 384.479166667 | 17 | 48 | 0 | 1 |
| Path 52 | C00031->C00183:[2->3,4->2,7->5,7->6,9->1] | 1.00 | 417.814814815 | 25 | 81 | 0 | 1 |
| Path 53 | C00031->C00183:[7->6,9->2] | 0.40 | 395.027027027 | 17 | 37 | 0 | 1 |
| Path 54 | C00031->C00183:[4->2,4->6] | 0.40 | 459.193548387 | 23 | 62 | 0 | 1 |
| Path 55 | C00031->C00183:[4->3,7->5,7->6,9->1,9->2] | 1.00 | 392.402777778 | 29 | 72 | 0 | 2 |
| Path 56 | C00031->C00183:[4->2,4->6] | 0.40 | 474.465517241 | 23 | 58 | 0 | 1 |
| Path 57 | C00031->C00183:[1->1,2->3,4->5,7->6,9->2] | 1.00 | 404.481481481 | 19 | 54 | 0 | 1 |
| Path 58 | C00031->C00183:[1->1,2->3,4->5,7->6,9->2] | 1.00 | 471.272727273 | 20 | 55 | 0 | 1 |
| Path 59 | C00031->C00183:[7->5,9->2] | 0.40 | 462.217391304 | 21 | 46 | 0 | 1 |
| Path 60 | C00031->C00183:[5->3,7->6,9->1] | 0.60 | 374.706896552 | 17 | 58 | 0 | 1 |
| Path 61 | C00031->C00183:[4->3,7->5,7->6,9->1,9->2] | 1.00 | 404.058823529 | 18 | 51 | 0 | 1 |
| Path 62 | C00031->C00183:[5->3,7->5,9->1] | 0.60 | 384.461538462 | 16 | 52 | 0 | 1 |
| Path 63 | C00031->C00183:[4->3,7->5,7->6,9->1,9->2] | 1.00 | 419.953488372 | 17 | 43 | 0 | 1 |
| Path 64 | C00031->C00183:[2->3,4->2,7->5,7->6,9->1] | 1.00 | 448.229508197 | 26 | 61 | 0 | 1 |
| Path 65 | C00031->C00183:[4->3,7->5,7->6,9->1,9->2] | 1.00 | 396.979166667 | 17 | 48 | 0 | 1 |
| Path 66 | C00031->C00183:[2->3,4->2,7->5,7->6,9->1] | 1.00 | 413.024390244 | 28 | 82 | 0 | 1 |
| Path 67 | C00031->C00183:[4->3,7->5,7->6,9->1,9->2] | 1.00 | 398.682926829 | 15 | 41 | 0 | 1 |
| Path 68 | C00031->C00183:[4->3,7->5,7->6,9->1,9->2] | 1.00 | 457.076923077 | 24 | 78 | 0 | 1 |
| Path 69 | C00031->C00183:[1->1,1->2,2->3,4->5,4->6] | 1.00 | 478.78 | 15 | 50 | 0 | 1 |
| Path 70 | C00031->C00183:[4->2,4->6] | 0.40 | 472.127272727 | 22 | 55 | 0 | 1 |
| Path 71 | C00031->C00183:[7->6,9->1,9->3] | 0.60 | 270.529411765 | 16 | 68 | 0 | 1 |
| Path 72 | C00031->C00183:[7->3,7->6,9->1] | 0.60 | 389.5 | 13 | 36 | 0 | 1 |
| Path 73 | C00031->C00183:[2->3,4->2,7->5,7->6,9->1] | 1.00 | 412.756097561 | 27 | 82 | 0 | 1 |
| Path 74 | C00031->C00183:[4->3,7->5,7->6,9->1,9->2] | 1.00 | 398.578947368 | 31 | 76 | 0 | 2 |
| Path 75 | C00031->C00183:[7->5,9->1,9->3] | 0.60 | 402.12195122 | 15 | 41 | 0 | 1 |
| Path 76 | C00031->C00183:[4->3,7->5,7->6,9->1,9->2] | 1.00 | 347.368421053 | 24 | 76 | 0 | 1 |
| Path 77 | C00031->C00183:[4->2,4->5] | 0.40 | 456.237288136 | 22 | 59 | 0 | 1 |
| Path 78 | C00031->C00183:[7->6,9->2] | 0.40 | 312.473684211 | 8 | 19 | 0 | 1 |
| Path 79 | C00031->C00183:[4->3,7->6,9->1] | 0.60 | 374.206896552 | 21 | 58 | 0 | 2 |
| Path 80 | C00031->C00183:[7->3,7->5,7->6,9->1,9->2] | 1.00 | 387.977272727 | 16 | 44 | 0 | 1 |
| Path 81 | C00031->C00183:[7->6,9->1,9->3] | 0.60 | 352.575 | 12 | 40 | 0 | 1 |
| Path 82 | C00031->C00183:[4->3,7->5,7->6,9->1,9->2] | 1.00 | 415.34 | 19 | 50 | 0 | 1 |
| Path 83 | C00031->C00183:[4->3,7->5,7->6,9->1,9->2] | 1.00 | 311.364864865 | 21 | 74 | 0 | 2 |
| Path 84 | C00031->C00183:[2->3,4->2,7->5,7->6,9->1] | 1.00 | 379.423529412 | 28 | 85 | 0 | 1 |
| Path 85 | C00031->C00183:[1->1,2->3,4->5,7->6,9->2] | 1.00 | 471.433962264 | 17 | 53 | 0 | 1 |
| Path 86 | C00031->C00183:[4->3,7->3,7->6,9->1] | 0.60 | 377.438596491 | 22 | 57 | 0 | 2 |
| Path 87 | C00031->C00183:[4->2,4->3,7->5,7->6,9->1] | 1.00 | 366.105263158 | 26 | 76 | 0 | 1 |
| Path 88 | C00031->C00183:[7->6,9->1,9->3] | 0.60 | 347.708333333 | 15 | 48 | 0 | 1 |
| Path 89 | C00031->C00183:[1->1,2->3,4->5,7->6,9->2] | 1.00 | 479.944444444 | 18 | 54 | 0 | 1 |
| Path 90 | C00031->C00183:[1->1,2->3,4->5,7->6,9->2] | 1.00 | 414.054545455 | 20 | 55 | 0 | 1 |
| Path 91 | C00031->C00183:[1->1,2->3,4->5,7->6,9->2] | 1.00 | 445.551724138 | 21 | 58 | 0 | 1 |
| Path 92 | C00031->C00183:[4->3,7->5,7->6,9->1,9->2] | 1.00 | 291.8 | 20 | 75 | 0 | 2 |
| Path 93 | C00031->C00183:[2->3,7->5,7->6,9->1,9->2] | 1.00 | 478.442307692 | 17 | 52 | 0 | 1 |
| Path 94 | C00031->C00183:[7->6,9->2] | 0.40 | 380.138888889 | 16 | 36 | 0 | 1 |
| Path 95 | C00031->C00183:[4->1,4->3,7->6,9->1] | 0.60 | 288.986111111 | 19 | 72 | 0 | 2 |
| Path 96 | C00031->C00183:[7->3,7->6,9->1] | 0.60 | 369.5 | 14 | 42 | 0 | 1 |
| Path 97 | C00031->C00183:[4->3,7->5,7->6,9->1,9->2] | 1.00 | 408.581818182 | 22 | 55 | 0 | 1 |
| Path 98 | C00031->C00183:[4->3,7->3,7->6,9->1] | 0.60 | 386.114754098 | 24 | 61 | 0 | 2 |
| Path 99 | C00031->C00183:[7->6,9->1,9->3] | 0.60 | 395.0 | 14 | 40 | 0 | 1 |
| Path 100 | C00031->C00183:[1->1,1->2,2->3,4->5,4->6] | 1.00 | 500.87037037 | 19 | 54 | 0 | 1 |
| Path 101 | C00031->C00183:[4->2,7->6,9->2] | 0.40 | 374.720930233 | 18 | 43 | 0 | 1 |
| Path 102 | C00031->C00183:[4->3,7->5,7->6,9->1,9->2] | 1.00 | 403.264705882 | 28 | 68 | 0 | 2 |
| Path 103 | C00031->C00183:[4->3,7->5,7->6,9->1,9->2] | 1.00 | 384.479166667 | 17 | 48 | 0 | 1 |
| Path 104 | C00031->C00183:[7->5,9->2] | 0.40 | 343.4 | 9 | 20 | 0 | 1 |
| Path 105 | C00031->C00183:[4->3,7->5,7->6,9->1,9->2] | 1.00 | 418.022222222 | 19 | 45 | 0 | 1 |
| Path 106 | C00031->C00183:[4->3,7->3,7->5,7->6,9->1,9->2] | 1.00 | 377.181818182 | 26 | 66 | 0 | 2 |
| Path 107 | C00031->C00183:[2->3,7->5,7->6,9->1,9->2] | 1.00 | 490.127659574 | 16 | 47 | 0 | 1 |
| Path 108 | C00031->C00183:[4->3,7->6,9->1] | 0.60 | 388.87755102 | 16 | 49 | 0 | 1 |
| Path 109 | C00031->C00183:[4->2,4->5] | 0.40 | 463.05 | 22 | 60 | 0 | 1 |
| Path 110 | C00031->C00183:[1->1,1->2,2->3,4->5,4->6] | 1.00 | 500.462962963 | 18 | 54 | 0 | 1 |
| Path 111 | C00031->C00183:[7->3,7->5,7->6,9->1,9->2] | 1.00 | 374.340909091 | 16 | 44 | 0 | 1 |
| Path 112 | C00031->C00183:[7->6,9->2] | 0.40 | 390.692307692 | 17 | 39 | 0 | 1 |
| Path 113 | C00031->C00183:[7->6,9->1,9->3] | 0.60 | 252.787878788 | 14 | 66 | 0 | 1 |
| Path 114 | C00031->C00183:[7->6,9->1,9->3] | 0.60 | 407.863636364 | 15 | 44 | 0 | 1 |
| Path 115 | C00031->C00183:[7->6,9->1,9->3] | 0.60 | 376.547619048 | 14 | 42 | 0 | 1 |
| Path 116 | C00031->C00183:[2->3,4->2,7->5,7->6,9->1] | 1.00 | 500.180327869 | 27 | 61 | 0 | 1 |
| Path 117 | C00031->C00183:[1->1,2->3,4->6,7->5,9->2] | 1.00 | 462.568965517 | 18 | 58 | 0 | 1 |
| Path 118 | C00031->C00183:[4->1,4->3,7->6,9->1] | 0.60 | 304.310810811 | 21 | 74 | 0 | 2 |
| Path 119 | C00031->C00183:[1->1,2->3,4->5,7->6,9->2] | 1.00 | 463.166666667 | 20 | 54 | 0 | 1 |
| Path 120 | C00031->C00183:[4->3,7->5,7->6,9->1,9->2] | 1.00 | 338.347222222 | 22 | 72 | 0 | 1 |
| Path 121 | C00031->C00183:[2->3,4->2,7->5,7->6,9->1] | 1.00 | 407.314606742 | 30 | 89 | 0 | 1 |
| Path 122 | C00031->C00183:[4->2,7->6,9->2] | 0.40 | 427.285714286 | 20 | 42 | 0 | 1 |
| Path 123 | C00031->C00183:[4->3,7->5,7->6,9->1,9->2] | 1.00 | 474.375 | 23 | 72 | 0 | 1 |
| Path 124 | C00031->C00183:[2->3,4->2,7->5,7->6,9->1] | 1.00 | 416.705882353 | 29 | 85 | 0 | 1 |
| Path 125 | C00031->C00183:[2->3,4->2,7->5,7->6,9->1] | 1.00 | 497.369230769 | 25 | 65 | 0 | 1 |
| Path 126 | C00031->C00183:[4->2,7->6,9->2] | 0.40 | 447.236842105 | 20 | 38 | 0 | 1 |
| Path 127 | C00031->C00183:[4->3,7->6,9->1] | 0.60 | 272.275362319 | 17 | 69 | 0 | 2 |
| Path 128 | C00031->C00183:[4->2,4->3,7->5,7->6,9->1] | 1.00 | 357.6 | 22 | 70 | 0 | 1 |
| Path 129 | C00031->C00183:[4->2,7->5,9->2] | 0.40 | 441.228571429 | 19 | 35 | 0 | 1 |
| Path 130 | C00031->C00183:[7->5,9->2] | 0.40 | 395.027027027 | 17 | 37 | 0 | 1 |
| Path 131 | C00031->C00183:[4->3,7->5,7->6,9->1,9->2] | 1.00 | 302.835443038 | 22 | 79 | 0 | 2 |
| Path 132 | C00031->C00183:[7->6,9->1,9->3] | 0.60 | 296.05 | 14 | 60 | 0 | 1 |
| Path 133 | C00031->C00183:[7->6,9->1,9->3] | 0.60 | 298.775862069 | 15 | 58 | 0 | 1 |
| Path 134 | C00031->C00183:[1->2,4->2,4->6] | 0.40 | 465.298507463 | 22 | 67 | 0 | 1 |
| Path 135 | C00031->C00183:[2->3,7->5,7->6,9->1,9->2] | 1.00 | 459.461538462 | 20 | 52 | 0 | 1 |
| Path 136 | C00031->C00183:[2->3,4->2,7->5,7->6,9->1] | 1.00 | 414.953488372 | 26 | 86 | 0 | 1 |
| Path 137 | C00031->C00183:[4->2,7->6,9->2] | 0.40 | 420.358974359 | 19 | 39 | 0 | 1 |
| Path 138 | C00031->C00183:[7->6,9->2] | 0.40 | 465.591836735 | 22 | 49 | 0 | 1 |
| Path 139 | C00031->C00183:[4->3,5->1,5->2,9->5,9->6] | 1.00 | 471.72972973 | 25 | 74 | 0 | 1 |
| Path 140 | C00031->C00183:[4->3,7->5,7->6,9->1,9->2] | 1.00 | 365.793650794 | 23 | 63 | 0 | 2 |
| Path 141 | C00031->C00183:[4->2,4->3,7->5,7->6,9->1] | 1.00 | 365.824324324 | 24 | 74 | 0 | 1 |
| Path 142 | C00031->C00183:[4->2,7->5,9->2] | 0.40 | 420.358974359 | 19 | 39 | 0 | 1 |
| Path 143 | C00031->C00183:[1->1,1->2,2->3,4->5,4->6] | 1.00 | 467.966101695 | 17 | 59 | 0 | 1 |
| Path 144 | C00031->C00183:[7->5,9->1,9->3] | 0.60 | 360.731707317 | 13 | 41 | 0 | 1 |
| Path 145 | C00031->C00183:[5->3,7->6,9->1] | 0.60 | 378.529411765 | 15 | 51 | 0 | 1 |
| Path 146 | C00031->C00183:[7->5,9->2] | 0.40 | 312.473684211 | 8 | 19 | 0 | 1 |
| Path 147 | C00031->C00183:[1->1,2->3,4->6] | 0.60 | 472.25 | 13 | 48 | 0 | 1 |
| Path 148 | C00031->C00183:[2->3,4->2,7->5,7->6,9->1] | 1.00 | 499.819672131 | 26 | 61 | 0 | 1 |
| Path 149 | C00031->C00183:[4->3,7->6,9->1] | 0.60 | 380.5 | 15 | 46 | 0 | 1 |
| Path 150 | C00031->C00183:[4->2,4->3,7->5,7->6,9->1] | 1.00 | 352.208333333 | 22 | 72 | 0 | 1 |
| Path 151 | C00031->C00183:[7->5,9->2] | 0.40 | 380.138888889 | 16 | 36 | 0 | 1 |
| Path 152 | C00031->C00183:[4->3,7->6,9->1] | 0.60 | 364.166666667 | 19 | 54 | 0 | 2 |
| Path 153 | C00031->C00183:[7->6,9->1,9->3] | 0.60 | 278.875 | 13 | 56 | 0 | 1 |
| Path 154 | C00031->C00183:[4->3,7->5,9->1] | 0.60 | 394.05 | 14 | 40 | 0 | 1 |
| Path 155 | C00031->C00183:[7->6,9->2] | 0.40 | 321.730769231 | 10 | 26 | 0 | 1 |
| Path 156 | C00031->C00183:[7->6,9->1,9->3] | 0.60 | 416.952380952 | 16 | 42 | 0 | 1 |
| Path 157 | C00031->C00183:[1->2,4->6] | 0.40 | 465.425531915 | 13 | 47 | 0 | 1 |
| Path 158 | C00031->C00183:[7->6,9->1,9->3] | 0.60 | 269.0 | 15 | 70 | 0 | 1 |
| Path 159 | C00031->C00183:[7->6,9->2] | 0.40 | 203.954545455 | 10 | 44 | 0 | 1 |
| Path 160 | C00031->C00183:[4->3,7->5,7->6,9->1,9->2] | 1.00 | 374.388059701 | 25 | 67 | 0 | 2 |
| Path 161 | C00031->C00183:[7->3,7->5,7->6,9->1,9->2] | 1.00 | 395.282608696 | 18 | 46 | 0 | 1 |
| Path 162 | C00031->C00183:[5->3,7->6,9->1] | 0.60 | 389.153846154 | 16 | 52 | 0 | 1 |
| Path 163 | C00031->C00183:[4->2,4->6] | 0.40 | 465.634920635 | 23 | 63 | 0 | 1 |
| Path 164 | C00031->C00183:[7->6,9->2] | 0.40 | 462.217391304 | 21 | 46 | 0 | 1 |
| Path 165 | C00031->C00183:[4->3,7->6,9->1] | 0.60 | 293.279411765 | 18 | 68 | 0 | 2 |
| Path 166 | C00031->C00183:[2->3,4->2,7->5,7->6,9->1] | 1.00 | 416.447058824 | 28 | 85 | 0 | 1 |
| Path 167 | C00031->C00183:[4->2,4->3,7->5,7->6,9->1] | 1.00 | 358.125 | 24 | 72 | 0 | 1 |
| Path 168 | C00031->C00183:[7->5,9->1,9->3] | 0.60 | 259.268656716 | 15 | 67 | 0 | 1 |
| Path 169 | C00031->C00183:[4->3,7->5,7->6,9->1,9->2] | 1.00 | 405.192307692 | 21 | 52 | 0 | 1 |
| Path 170 | C00031->C00183:[4->3,7->5,9->1] | 0.60 | 402.651162791 | 15 | 43 | 0 | 1 |
| Path 171 | C00031->C00183:[4->3,7->6,9->1] | 0.60 | 408.325581395 | 15 | 43 | 0 | 1 |
| Path 172 | C00031->C00183:[7->3,7->5,9->1] | 0.60 | 382.722222222 | 13 | 36 | 0 | 1 |
| Path 173 | C00031->C00183:[7->5,7->6,9->1,9->2] | 0.80 | 379.711111111 | 17 | 45 | 0 | 1 |
| Path 174 | C00031->C00183:[4->3,7->5,7->6,9->1,9->2] | 1.00 | 340.873239437 | 21 | 71 | 0 | 1 |
| Path 175 | C00031->C00183:[4->1,4->3] | 0.40 | 391.510638298 | 17 | 47 | 0 | 2 |
| Path 176 | C00031->C00183:[2->3,4->2,7->5,7->6,9->1] | 1.00 | 382.928571429 | 27 | 98 | 0 | 1 |
| Path 177 | C00031->C00183:[1->1,2->3,4->5] | 0.60 | 478.795918367 | 14 | 49 | 0 | 1 |
| Path 178 | C00031->C00183:[4->3,7->3,7->5,9->1] | 0.60 | 293.547945205 | 21 | 73 | 0 | 2 |
| Path 179 | C00031->C00183:[7->5,7->6,9->1,9->2] | 0.80 | 356.634146341 | 13 | 41 | 0 | 1 |
| Path 180 | C00031->C00183:[4->1,4->3,7->6,9->2] | 0.80 | 409.147540984 | 24 | 61 | 0 | 2 |
| Path 181 | C00031->C00183:[4->3,7->5,9->1] | 0.60 | 381.305084746 | 22 | 59 | 0 | 2 |
| Path 182 | C00031->C00183:[1->1,2->3,4->2,4->5,4->6] | 1.00 | 372.135416667 | 27 | 96 | 0 | 1 |
| Path 183 | C00031->C00183:[4->3,7->5,9->1] | 0.60 | 409.731707317 | 15 | 41 | 0 | 1 |
| Path 184 | C00031->C00183:[4->3,7->5,9->1] | 0.60 | 396.7 | 14 | 40 | 0 | 1 |
| Path 185 | C00031->C00183:[4->3,7->5,9->1] | 0.60 | 371.963636364 | 20 | 55 | 0 | 2 |
| Path 186 | C00031->C00183:[7->3,7->6,9->1] | 0.60 | 265.016393443 | 15 | 61 | 0 | 2 |
| Path 187 | C00031->C00183:[4->1,4->3,7->6,9->2] | 0.80 | 415.338461538 | 26 | 65 | 0 | 2 |
| Path 188 | C00031->C00183:[7->5,7->6,9->1,9->2] | 0.80 | 383.044444444 | 17 | 45 | 0 | 1 |
| Path 189 | C00031->C00183:[4->3,5->1,9->5] | 0.60 | 408.186046512 | 17 | 43 | 0 | 1 |
| Path 190 | C00031->C00183:[4->3,7->5,9->1] | 0.60 | 417.068181818 | 16 | 44 | 0 | 1 |
| Path 191 | C00031->C00183:[4->3,5->1,9->5] | 0.60 | 415.304347826 | 18 | 46 | 0 | 1 |
| Path 192 | C00031->C00183:[4->3,7->5,9->1] | 0.60 | 397.454545455 | 21 | 55 | 0 | 2 |
| Path 193 | C00031->C00183:[4->1,4->3,7->6,9->2] | 0.80 | 425.983333333 | 25 | 60 | 0 | 2 |
| Path 194 | C00031->C00183:[1->1,2->3,4->2,4->5,4->6] | 1.00 | 405.582278481 | 25 | 79 | 0 | 1 |
| Path 195 | C00031->C00183:[7->5,7->6,9->1,9->2] | 0.80 | 393.813953488 | 15 | 43 | 0 | 1 |
| Path 196 | C00031->C00183:[4->3,7->5,7->6,9->1,9->2] | 1.00 | 299.267605634 | 19 | 71 | 0 | 2 |
| Path 197 | C00031->C00183:[7->6,9->1] | 0.40 | 368.878787879 | 10 | 33 | 0 | 1 |
| Path 198 | C00031->C00183:[7->5,7->6,9->1,9->2] | 0.80 | 395.138888889 | 13 | 36 | 0 | 1 |
| Path 199 | C00031->C00183:[4->3,4->5,7->6,9->2] | 0.80 | 237.223214286 | 22 | 112 | 0 | 1 |
| Path 200 | C00031->C00183:[4->2,4->3,7->5,7->6,9->1] | 1.00 | 350.309859155 | 21 | 71 | 0 | 1 |
| Path 201 | C00031->C00183:[1->1,2->3,4->2,4->5,4->6] | 1.00 | 380.495049505 | 26 | 101 | 0 | 1 |
| Path 202 | C00031->C00183:[7->5,7->6,9->1,9->2] | 0.80 | 368.029411765 | 11 | 34 | 0 | 1 |
| Path 203 | C00031->C00183:[7->5,7->6,9->1,9->2] | 0.80 | 394.157894737 | 15 | 38 | 0 | 1 |
| Path 204 | C00031->C00183:[4->1,4->3,7->1,7->3] | 0.40 | 388.716981132 | 19 | 53 | 0 | 2 |
| Path 205 | C00031->C00183:[7->5,7->6,9->1,9->2] | 0.80 | 370.30952381 | 14 | 42 | 0 | 1 |
| Path 206 | C00031->C00183:[1->1,2->3,4->2,4->5,4->6] | 1.00 | 509.413793103 | 22 | 58 | 0 | 1 |
| Path 207 | C00031->C00183:[4->1,4->3,7->1,7->3] | 0.40 | 389.078431373 | 19 | 51 | 0 | 2 |
| Path 208 | C00031->C00183:[4->3] | 0.20 | 313.434782609 | 10 | 23 | 0 | 2 |
| Path 209 | C00031->C00183:[7->3,7->5,9->1] | 0.60 | 379.348837209 | 15 | 43 | 0 | 1 |
| Path 210 | C00031->C00183:[4->1,4->3,7->1,7->3] | 0.40 | 372.363636364 | 20 | 55 | 0 | 2 |
| Path 211 | C00031->C00183:[7->5,9->1] | 0.40 | 256.101694915 | 13 | 59 | 0 | 2 |
| Path 212 | C00031->C00183:[7->5,7->6,9->1,9->2] | 0.80 | 272.109375 | 15 | 64 | 0 | 2 |
| Path 213 | C00031->C00183:[4->2,4->3,7->5,7->6,9->1] | 1.00 | 358.375 | 22 | 72 | 0 | 1 |
| Path 214 | C00031->C00183:[4->3,7->5,9->1] | 0.60 | 292.148648649 | 20 | 74 | 0 | 2 |
| Path 215 | C00031->C00183:[7->6,9->1,9->3] | 0.60 | 286.492063492 | 16 | 63 | 0 | 2 |
| Path 216 | C00031->C00183:[4->3,4->6,7->5,9->2] | 0.80 | 237.223214286 | 22 | 112 | 0 | 1 |
| Path 217 | C00031->C00183:[2->3,4->2,7->5,7->6,9->1] | 1.00 | 379.313131313 | 30 | 99 | 0 | 1 |
| Path 218 | C00031->C00183:[4->1,4->3] | 0.40 | 406.0 | 16 | 43 | 0 | 2 |
| Path 219 | C00031->C00183:[4->3,4->6,7->5,9->2] | 0.80 | 246.620689655 | 24 | 116 | 0 | 1 |
| Path 220 | C00031->C00183:[4->1,4->3,7->6,9->2] | 0.80 | 393.769230769 | 25 | 65 | 0 | 2 |
| Path 221 | C00031->C00183:[4->3,7->5,9->1] | 0.60 | 345.661764706 | 21 | 68 | 0 | 2 |
| Path 222 | C00031->C00183:[4->1,4->3] | 0.40 | 349.052631579 | 17 | 57 | 0 | 2 |
| Path 223 | C00031->C00183:[1->1,2->3,4->2,4->5,4->6] | 1.00 | 371.9875 | 25 | 80 | 0 | 1 |
| Path 224 | C00031->C00183:[4->2,4->3,7->5,7->6,9->1] | 1.00 | 351.463768116 | 21 | 69 | 0 | 1 |
| Path 225 | C00031->C00183:[4->3,7->5,7->6,9->1,9->2] | 1.00 | 334.1125 | 25 | 80 | 0 | 2 |
| Path 226 | C00031->C00183:[2->3,7->5,7->6,9->1,9->2] | 1.00 | 448.154929577 | 20 | 71 | 0 | 1 |
| Path 227 | C00031->C00183:[2->3,7->5,7->6,9->1,9->2] | 1.00 | 466.492307692 | 19 | 65 | 0 | 1 |
| Path 228 | C00031->C00183:[4->1,4->3,7->6,9->2] | 0.80 | 369.129032258 | 22 | 62 | 0 | 2 |
| Path 229 | C00031->C00183:[4->3,7->5,9->2] | 0.60 | 481.5 | 21 | 48 | 0 | 1 |
| Path 230 | C00031->C00183:[4->3,7->3,7->5,9->1] | 0.60 | 384.603448276 | 23 | 58 | 0 | 2 |
| Path 231 | C00031->C00183:[4->1,4->3,7->1,7->3] | 0.40 | 374.586206897 | 21 | 58 | 0 | 2 |
| Path 232 | C00031->C00183:[4->1,4->3,7->1,7->3] | 0.40 | 396.203703704 | 20 | 54 | 0 | 2 |
| Path 233 | C00031->C00183:[4->3] | 0.20 | 282.85 | 9 | 20 | 0 | 2 |
| Path 234 | C00031->C00183:[7->3,7->6,9->1] | 0.60 | 258.507692308 | 16 | 65 | 0 | 2 |
| Path 235 | C00031->C00183:[1->1,2->3,4->5] | 0.60 | 410.533333333 | 15 | 45 | 0 | 1 |
| Path 236 | C00031->C00183:[1->1,2->3,4->2,4->5,4->6] | 1.00 | 377.216494845 | 27 | 97 | 0 | 1 |
| Path 237 | C00031->C00183:[7->3] | 0.20 | 227.291666667 | 9 | 24 | 0 | 2 |
| Path 238 | C00031->C00183:[5->1,7->3,9->5] | 0.60 | 399.179487179 | 16 | 39 | 0 | 1 |
| Path 239 | C00031->C00183:[4->3,7->5,9->2] | 0.60 | 471.38 | 21 | 50 | 0 | 1 |
| Path 240 | C00031->C00183:[4->3,7->6,9->1] | 0.60 | 289.735294118 | 17 | 68 | 0 | 2 |
| Path 241 | C00031->C00183:[7->5,9->2] | 0.40 | 355.392857143 | 11 | 28 | 0 | 1 |
| Path 242 | C00031->C00183:[7->3,7->5,9->1] | 0.60 | 273.532258065 | 16 | 62 | 0 | 2 |
| Path 243 | C00031->C00183:[1->1,2->3,4->2,4->5,4->6] | 1.00 | 413.642857143 | 24 | 84 | 0 | 1 |
| Path 244 | C00031->C00183:[4->3,7->5,9->1] | 0.60 | 297.028985507 | 18 | 69 | 0 | 2 |
| Path 245 | C00031->C00183:[4->2,4->3,5->1,9->5,9->6] | 1.00 | 358.864864865 | 24 | 74 | 0 | 1 |
| Path 246 | C00031->C00183:[4->2,4->3,5->1,9->5,9->6] | 1.00 | 360.573333333 | 25 | 75 | 0 | 1 |
| Path 247 | C00031->C00183:[4->1,4->3] | 0.40 | 359.590163934 | 19 | 61 | 0 | 2 |
| Path 248 | C00031->C00183:[1->1,2->3,4->2,4->5,4->6] | 1.00 | 447.491525424 | 24 | 59 | 0 | 1 |
| Path 249 | C00031->C00183:[1->1,2->3,4->2,4->5,4->6] | 1.00 | 513.574074074 | 24 | 54 | 0 | 1 |
| Path 250 | C00031->C00183:[1->1,2->3,4->5] | 0.60 | 480.466666667 | 15 | 45 | 0 | 1 |
| Path 251 | C00031->C00183:[4->1,4->3,7->6,9->2] | 0.80 | 421.175438596 | 24 | 57 | 0 | 2 |
| Path 252 | C00031->C00183:[1->1,2->3,4->5] | 0.60 | 491.318181818 | 13 | 44 | 0 | 1 |
| Path 253 | C00031->C00183:[4->3,7->5,9->1] | 0.60 | 395.796296296 | 20 | 54 | 0 | 2 |
| Path 254 | C00031->C00183:[4->3,7->5,9->1] | 0.60 | 385.882352941 | 23 | 85 | 0 | 2 |
| Path 255 | C00031->C00183:[1->1,2->3,4->5] | 0.60 | 495.125 | 15 | 40 | 0 | 1 |
| Path 256 | C00031->C00183:[4->3,7->6,9->1] | 0.60 | 380.56 | 18 | 50 | 0 | 2 |
| Path 257 | C00031->C00183:[4->1,4->3,7->6,9->2] | 0.80 | 410.507692308 | 27 | 65 | 0 | 2 |
| Path 258 | C00031->C00183:[4->1,4->3,7->5,9->2] | 0.80 | 369.129032258 | 22 | 62 | 0 | 2 |
| Path 259 | C00031->C00183:[4->3,7->3,7->5,9->1] | 0.60 | 346.546666667 | 25 | 75 | 0 | 2 |
| Path 260 | C00031->C00183:[7->6,9->1] | 0.40 | 350.512820513 | 11 | 39 | 0 | 1 |
| Path 261 | C00031->C00183:[4->3,7->5,9->1] | 0.60 | 289.479452055 | 19 | 73 | 0 | 2 |
| Path 262 | C00031->C00183:[4->3,7->3,7->5,9->1] | 0.60 | 393.738636364 | 26 | 88 | 0 | 2 |
| Path 263 | C00031->C00183:[4->2] | 0.20 | 415.285714286 | 13 | 28 | 0 | 1 |
| Path 264 | C00031->C00183:[1->1,2->3,4->2,4->5,4->6] | 1.00 | 377.97826087 | 27 | 92 | 0 | 1 |
| Path 265 | C00031->C00183:[1->1,2->3,4->2,4->5,4->6] | 1.00 | 500.830508475 | 24 | 59 | 0 | 1 |
| Path 266 | C00031->C00183:[4->3,7->5,7->6,9->1,9->2] | 1.00 | 349.88 | 23 | 75 | 0 | 1 |
| Path 267 | C00031->C00183:[4->1,4->3] | 0.40 | 404.071428571 | 15 | 42 | 0 | 2 |
| Path 268 | C00031->C00183:[4->1,4->3] | 0.40 | 382.931818182 | 16 | 44 | 0 | 2 |
| Path 269 | C00031->C00183:[1->1,2->3,4->5] | 0.60 | 471.727272727 | 15 | 44 | 0 | 1 |
| Path 270 | C00031->C00183:[7->6,9->1] | 0.40 | 246.844827586 | 12 | 58 | 0 | 2 |
| Path 271 | C00031->C00183:[4->1,4->3] | 0.40 | 347.261538462 | 20 | 65 | 0 | 2 |
| Path 272 | C00031->C00183:[4->1,4->3,7->6,9->2] | 0.80 | 357.06097561 | 27 | 82 | 0 | 2 |
| Path 273 | C00031->C00183:[7->5,9->1] | 0.40 | 364.696969697 | 10 | 33 | 0 | 1 |
| Path 274 | C00031->C00183:[2->3,4->2,7->5,7->6,9->1] | 1.00 | 379.090909091 | 29 | 99 | 0 | 1 |
| Path 275 | C00031->C00183:[2->3,5->1,5->2,9->5,9->6] | 1.00 | 463.805970149 | 21 | 67 | 0 | 1 |
| Path 276 | C00031->C00183:[4->3,4->5,7->6,9->2] | 0.80 | 246.620689655 | 24 | 116 | 0 | 1 |
| Path 277 | C00031->C00183:[7->5,9->1] | 0.40 | 381.352941176 | 11 | 34 | 0 | 1 |
| Path 278 | C00031->C00183:[4->1,4->3,7->6,9->2] | 0.80 | 367.205128205 | 26 | 78 | 0 | 2 |
| Path 279 | C00031->C00183:[4->1,4->3] | 0.40 | 405.3 | 15 | 40 | 0 | 2 |
| Path 280 | C00031->C00183:[4->2] | 0.20 | 403.04 | 12 | 25 | 0 | 1 |
| Path 281 | C00031->C00183:[4->3,7->5,9->1] | 0.60 | 279.714285714 | 18 | 70 | 0 | 2 |
| Path 282 | C00031->C00183:[4->1,4->3] | 0.40 | 336.606557377 | 18 | 61 | 0 | 2 |
| Path 283 | C00031->C00183:[7->5,7->6,9->1,9->2] | 0.80 | 359.523809524 | 14 | 42 | 0 | 1 |
| Path 284 | C00031->C00183:[4->1,4->3,7->1,7->3] | 0.40 | 381.0 | 18 | 50 | 0 | 2 |
| Path 285 | C00031->C00183:[5->1,9->5] | 0.40 | 381.083333333 | 13 | 36 | 0 | 1 |
| Path 286 | C00031->C00183:[4->3,4->6,7->5,9->2] | 0.80 | 244.814159292 | 23 | 113 | 0 | 1 |
| Path 287 | C00031->C00183:[1->1,2->3,4->2,4->5,4->6] | 1.00 | 416.493670886 | 23 | 79 | 0 | 1 |
| Path 288 | C00031->C00183:[4->3,7->5,9->1] | 0.60 | 405.11627907 | 15 | 43 | 0 | 1 |
| Path 289 | C00031->C00183:[4->3,7->6,9->1] | 0.60 | 390.12962963 | 20 | 54 | 0 | 2 |
| Path 290 | C00031->C00183:[4->3,7->5,9->1] | 0.60 | 344.486842105 | 24 | 76 | 0 | 2 |
| Path 291 | C00031->C00183:[7->5,9->1] | 0.40 | 361.575 | 12 | 40 | 0 | 1 |
| Path 292 | C00031->C00183:[1->1,2->3,4->2,4->5,4->6] | 1.00 | 415.156626506 | 26 | 83 | 0 | 1 |
| Path 293 | C00031->C00183:[4->3,7->6,9->1] | 0.60 | 388.301886792 | 19 | 53 | 0 | 2 |
| Path 294 | C00031->C00183:[4->3,7->5,7->6,9->1,9->2] | 1.00 | 330.7 | 25 | 80 | 0 | 2 |
| Path 295 | C00031->C00183:[4->1,4->3,7->6,9->2] | 0.80 | 345.773333333 | 23 | 75 | 0 | 2 |
| Path 296 | C00031->C00183:[1->1,2->3,4->2,4->5,4->6] | 1.00 | 409.670731707 | 26 | 82 | 0 | 1 |
| Path 297 | C00031->C00183:[4->1,4->3,7->1,7->3] | 0.40 | 335.611111111 | 22 | 72 | 0 | 2 |
| Path 298 | C00031->C00183:[1->1,2->3,4->2,4->5,4->6] | 1.00 | 498.238095238 | 23 | 63 | 0 | 1 |
| Path 299 | C00031->C00183:[4->3,7->5,9->1] | 0.60 | 335.305555556 | 22 | 72 | 0 | 2 |
| Path 300 | C00031->C00183:[4->1,4->3,7->6,9->2] | 0.80 | 390.180327869 | 22 | 61 | 0 | 2 |
| Path 301 | C00031->C00183:[7->6,9->2] | 0.40 | 355.392857143 | 11 | 28 | 0 | 1 |
| Path 302 | C00031->C00183:[1->1,2->3,4->2,4->5,4->6] | 1.00 | 418.487179487 | 26 | 78 | 0 | 1 |
| Path 303 | C00031->C00183:[4->3,7->5,7->6,9->1,9->2] | 1.00 | 358.842696629 | 31 | 89 | 0 | 2 |
| Path 304 | C00031->C00183:[1->1,2->3,4->2,4->5,4->6] | 1.00 | 414.533333333 | 25 | 75 | 0 | 1 |
| Path 305 | C00031->C00183:[4->1,4->3] | 0.40 | 373.395348837 | 15 | 43 | 0 | 2 |
| Path 306 | C00031->C00183:[4->2,4->3,5->1,9->5,9->6] | 1.00 | 352.169014085 | 23 | 71 | 0 | 1 |
| Path 307 | C00031->C00183:[4->3,7->6,9->2] | 0.60 | 468.446808511 | 20 | 47 | 0 | 1 |
| Path 308 | C00031->C00183:[4->3,7->6,9->2] | 0.60 | 481.5 | 21 | 48 | 0 | 1 |
| Path 309 | C00031->C00183:[4->1,4->3] | 0.40 | 393.5 | 18 | 48 | 0 | 2 |
| Path 310 | C00031->C00183:[1->1,2->3,4->2,4->5,4->6] | 1.00 | 417.218390805 | 25 | 87 | 0 | 1 |
| Path 311 | C00031->C00183:[4->1,4->3,7->6,9->2] | 0.80 | 404.0 | 25 | 61 | 0 | 2 |
| Path 312 | C00031->C00183:[7->3,7->5,9->1] | 0.60 | 266.606060606 | 17 | 66 | 0 | 2 |
| Path 313 | C00031->C00183:[4->3,7->5,7->6,9->1,9->2] | 1.00 | 365.952941176 | 30 | 85 | 0 | 2 |
| Path 314 | C00031->C00183:[4->3,7->3,7->5,9->1] | 0.60 | 392.677419355 | 25 | 62 | 0 | 2 |
| Path 315 | C00031->C00183:[1->1,2->3,4->2,4->5,4->6] | 1.00 | 420.182926829 | 24 | 82 | 0 | 1 |
| Path 316 | C00031->C00183:[4->1,4->3] | 0.40 | 413.11627907 | 16 | 43 | 0 | 2 |
| Path 317 | C00031->C00183:[4->3,7->5,9->1] | 0.60 | 388.647058824 | 19 | 51 | 0 | 2 |
| Path 318 | C00031->C00183:[4->1,4->3] | 0.40 | 395.358974359 | 14 | 39 | 0 | 2 |
| Path 319 | C00031->C00183:[4->3,7->5,9->1] | 0.60 | 389.276595745 | 16 | 47 | 0 | 1 |
| Path 320 | C00031->C00183:[4->1,4->3,7->1,7->3] | 0.40 | 364.574074074 | 19 | 54 | 0 | 2 |
| Path 321 | C00031->C00183:[4->1,4->3] | 0.40 | 385.0 | 17 | 47 | 0 | 2 |
| Path 322 | C00031->C00183:[4->3,7->5,9->1] | 0.60 | 300.52173913 | 19 | 69 | 0 | 2 |
| Path 323 | C00031->C00183:[4->2] | 0.20 | 403.04 | 12 | 25 | 0 | 1 |
| Path 324 | C00031->C00183:[7->5,7->6,9->1,9->2] | 0.80 | 309.904109589 | 21 | 73 | 0 | 2 |
| Path 325 | C00031->C00183:[4->1,4->3,7->6,9->2] | 0.80 | 414.21875 | 25 | 64 | 0 | 2 |
| Path 326 | C00031->C00183:[1->1,2->3,4->2,4->5,4->6] | 1.00 | 381.114583333 | 25 | 96 | 0 | 1 |
| Path 327 | C00031->C00183:[7->5,7->6,9->1,9->2] | 0.80 | 371.268292683 | 13 | 41 | 0 | 1 |
| Path 328 | C00031->C00183:[4->1,4->3,7->6,9->2] | 0.80 | 374.202702703 | 26 | 74 | 0 | 2 |
| Path 329 | C00031->C00183:[4->3,4->5,7->6,9->2] | 0.80 | 244.814159292 | 23 | 113 | 0 | 1 |
| Path 330 | C00031->C00183:[2->3,4->2,7->5,7->6,9->1] | 1.00 | 382.233009709 | 28 | 103 | 0 | 1 |
| Path 331 | C00031->C00183:[7->5,7->6,9->1,9->2] | 0.80 | 379.860465116 | 15 | 43 | 0 | 1 |
| Path 332 | C00031->C00183:[4->3,7->5,9->1] | 0.60 | 396.96 | 17 | 50 | 0 | 1 |
| Path 333 | C00031->C00183:[4->3,7->5,9->2] | 0.60 | 468.446808511 | 20 | 47 | 0 | 1 |
| Path 334 | C00031->C00183:[7->5,9->1] | 0.40 | 361.484848485 | 10 | 33 | 0 | 1 |
| Path 335 | C00031->C00183:[4->1,4->3,7->6,9->2] | 0.80 | 377.651515152 | 24 | 66 | 0 | 2 |
| Path 336 | C00031->C00183:[4->3,7->6,9->1] | 0.60 | 282.486111111 | 18 | 72 | 0 | 2 |
| Path 337 | C00031->C00183:[4->2,4->3,7->5,7->6,9->1] | 1.00 | 360.136986301 | 23 | 73 | 0 | 1 |
| Path 338 | C00031->C00183:[1->1,2->3,4->2,4->5,4->6] | 1.00 | 377.240963855 | 26 | 83 | 0 | 1 |
| Path 339 | C00031->C00183:[4->1,4->3,7->6,9->2] | 0.80 | 391.741935484 | 23 | 62 | 0 | 2 |
| Path 340 | C00031->C00183:[7->3,7->5,9->1] | 0.60 | 385.666666667 | 13 | 36 | 0 | 1 |
| Path 341 | C00031->C00183:[4->3,7->5,9->1] | 0.60 | 391.449438202 | 25 | 89 | 0 | 2 |
| Path 342 | C00031->C00183:[4->1,4->3,7->6,9->2] | 0.80 | 363.179487179 | 27 | 78 | 0 | 2 |
| Path 343 | C00031->C00183:[4->3,7->5,9->1] | 0.60 | 287.015151515 | 17 | 66 | 0 | 2 |
| Path 344 | C00031->C00183:[7->6,9->1,9->3] | 0.60 | 267.819672131 | 14 | 61 | 0 | 2 |
| Path 345 | C00031->C00183:[1->1,2->3,4->2,4->5,4->6] | 1.00 | 494.551724138 | 24 | 58 | 0 | 1 |
| Path 346 | C00031->C00183:[2->3,4->2,7->5,7->6,9->1] | 1.00 | 372.650485437 | 31 | 103 | 0 | 1 |
| Path 347 | C00031->C00183:[4->1,4->3,7->5,9->2] | 0.80 | 383.603448276 | 21 | 58 | 0 | 2 |
| Path 348 | C00031->C00183:[7->6,9->1] | 0.40 | 351.3125 | 9 | 32 | 0 | 1 |
| Path 349 | C00031->C00183:[4->3,7->5,7->6,9->1,9->2] | 1.00 | 348.027027027 | 22 | 74 | 0 | 1 |
| Path 350 | C00031->C00183:[4->1,4->3,7->6,9->2] | 0.80 | 400.492753623 | 27 | 69 | 0 | 2 |
| Path 351 | C00031->C00183:[4->1,4->3] | 0.40 | 382.782608696 | 16 | 46 | 0 | 2 |
| Path 352 | C00031->C00183:[4->1,4->3,7->1,7->3] | 0.40 | 381.677966102 | 22 | 59 | 0 | 2 |
| Path 353 | C00031->C00183:[1->1,2->3,4->2,4->5,4->6] | 1.00 | 411.325 | 25 | 80 | 0 | 1 |
| Path 354 | C00031->C00183:[4->1,4->3,7->6,9->2] | 0.80 | 383.603448276 | 21 | 58 | 0 | 2 |
| Path 355 | C00031->C00183:[4->3,7->6,9->2] | 0.60 | 471.38 | 21 | 50 | 0 | 1 |
| Path 356 | C00031->C00183:[7->5,7->6,9->1,9->2] | 0.80 | 356.634146341 | 13 | 41 | 0 | 1 |
| Path 357 | C00031->C00183:[4->1,4->3,7->6,9->2] | 0.80 | 336.329113924 | 24 | 79 | 0 | 2 |
| Path 358 | C00031->C00183:[4->1,4->3] | 0.40 | 414.795454545 | 17 | 44 | 0 | 2 |
| Path 359 | C00031->C00183:[4->1,4->3,7->6,9->2] | 0.80 | 426.983606557 | 26 | 61 | 0 | 2 |
| Path 360 | C00031->C00183:[4->1,4->3,7->1,7->3] | 0.40 | 345.985294118 | 21 | 68 | 0 | 2 |
| Path 361 | C00031->C00183:[4->3,7->6,9->1] | 0.60 | 279.230769231 | 16 | 65 | 0 | 2 |
| Path 362 | C00031->C00183:[7->3,7->5,9->1] | 0.60 | 400.405405405 | 14 | 37 | 0 | 1 |
| Path 363 | C00031->C00183:[2->3,7->5,7->6,9->1,9->2] | 1.00 | 459.234375 | 18 | 64 | 0 | 1 |
