# Supplementary material for: 13C-metabolic flux ratio and novel carbon path analyses confirmed that Trichoderma reesei uses primarily the respirative pathway also on the preferred carbon source glucose
Source: BMC Syst Biol. 2009 Oct 29;3:104. doi: 10.1186/1752-0509-3-104 (PMC2776023; doi:10.1186/1752-0509-3-104)
Supplement: Additional file 1 — Pathways discovered in ReTrace carbon path analysis. Graphical and tabular representations of amino acid synthesis pathways discovered in ReTrace carbon path analysis [21]. Self-contained web site: unpack zip archive and open index.html with a web browser. [file 1752-0509-3-104-S1.zip › AF1-treesei/pathways-C00026-to-C00148.html]

Pathways from C00026 to C00148


**Pathways from C00026 to C00148**

**Sources:** 2-Oxoglutarate; (C00026)

**Target:**L-Proline; (C00148)

|  | Composite mapping | Z | Average score | Rpairs | Reactions | Zero scores | Scores under threshold |
| --- | --- | --- | --- | --- | --- | --- | --- |
| Path 1 | C00026->C00148:[1->1,2->3,5->7,8->2,8->8] | 1.00 | 185.975903614 | 13 | 83 | 0 | 0 |
| Path 2 | C00026->C00148:[1->1,2->3,5->7,8->2,8->8] | 1.00 | 622.761904762 | 14 | 21 | 0 | 0 |
| Path 3 | C00026->C00148:[1->1,2->3,3->2,5->7,8->8] | 1.00 | 244.363636364 | 5 | 11 | 0 | 0 |
| Path 4 | C00026->C00148:[1->1,2->3,3->2,5->7,8->8] | 1.00 | 175.723529412 | 15 | 170 | 0 | 0 |
| Path 5 | C00026->C00148:[1->1,1->2,2->3,5->7,8->8] | 1.00 | 376.575757576 | 12 | 33 | 0 | 0 |
| Path 6 | C00026->C00148:[1->1,2->3,5->7,8->2,8->8] | 1.00 | 197.06097561 | 13 | 82 | 0 | 0 |
| Path 7 | C00026->C00148:[1->1,2->3,5->7,8->2,8->8] | 1.00 | 202.918367347 | 15 | 98 | 0 | 0 |
| Path 8 | C00026->C00148:[1->1,2->3,5->7,8->2,8->8] | 1.00 | 199.957894737 | 13 | 95 | 0 | 0 |
| Path 9 | C00026->C00148:[1->1,2->3,3->2,5->7,8->8] | 1.00 | 199.97029703 | 13 | 101 | 0 | 0 |
| Path 10 | C00026->C00148:[1->1,2->3,3->2,5->7,8->8] | 1.00 | 436.405405405 | 12 | 37 | 0 | 0 |
| Path 11 | C00026->C00148:[1->1,2->3,5->7,8->2,8->8] | 1.00 | 187.951219512 | 12 | 82 | 0 | 0 |
| Path 12 | C00026->C00148:[1->1,2->3,3->2,5->7,8->8] | 1.00 | 413.913978495 | 16 | 93 | 0 | 0 |
| Path 13 | C00026->C00148:[1->1,2->3,3->2,5->7,8->8] | 1.00 | 355.77027027 | 3 | 74 | 0 | 0 |
| Path 14 | C00026->C00148:[1->1,2->3,3->2,5->7,8->8] | 1.00 | 193.508474576 | 19 | 177 | 0 | 0 |
| Path 15 | C00026->C00148:[1->1,2->3,5->7,8->2,8->8] | 1.00 | 210.881818182 | 14 | 110 | 0 | 0 |
| Path 16 | C00026->C00148:[1->1,2->3,5->7,8->2,8->8] | 1.00 | 206.8 | 15 | 100 | 0 | 0 |
| Path 17 | C00026->C00148:[1->1,2->3,3->2,5->7,8->8] | 1.00 | 367.329268293 | 10 | 82 | 0 | 0 |
| Path 18 | C00026->C00148:[1->1,2->3,3->2,5->7,8->8] | 1.00 | 473.208333333 | 12 | 24 | 0 | 0 |
| Path 19 | C00026->C00148:[1->1,2->3,3->2,5->7,8->8] | 1.00 | 460.5 | 5 | 8 | 0 | 0 |
| Path 20 | C00026->C00148:[1->1,2->3,3->2,5->7,8->8] | 1.00 | 163.452380952 | 9 | 84 | 0 | 0 |
| Path 21 | C00026->C00148:[1->1,2->3,3->2,5->7,8->8] | 1.00 | 228.106557377 | 17 | 244 | 0 | 0 |
| Path 22 | C00026->C00148:[2->3,3->1,3->2,5->7,8->8] | 1.00 | 234.305263158 | 16 | 95 | 0 | 0 |
| Path 23 | C00026->C00148:[1->1,2->2,2->3,5->7,8->8] | 1.00 | 516.0 | 15 | 30 | 0 | 0 |
| Path 24 | C00026->C00148:[1->1,2->3,3->2,5->7,8->8] | 1.00 | 282.883977901 | 17 | 181 | 0 | 0 |
| Path 25 | C00026->C00148:[1->1,2->3,5->7,8->2,8->8] | 1.00 | 220.155555556 | 17 | 90 | 0 | 0 |
| Path 26 | C00026->C00148:[1->1,2->3,3->2,5->7,8->8] | 1.00 | 354.767123288 | 4 | 73 | 0 | 0 |
| Path 27 | C00026->C00148:[1->1,2->3,3->2,5->7,8->8] | 1.00 | 230.591093117 | 19 | 247 | 0 | 0 |
| Path 28 | C00026->C00148:[1->1,2->3,3->2,5->7,8->8] | 1.00 | 190.11299435 | 20 | 177 | 0 | 0 |
| Path 29 | C00026->C00148:[1->1,2->3,3->2,5->7,8->8] | 1.00 | 432.207920792 | 21 | 101 | 0 | 0 |
| Path 30 | C00026->C00148:[2->3,3->1,3->2,5->7,8->8] | 1.00 | 253.63 | 19 | 100 | 0 | 0 |
| Path 31 | C00026->C00148:[1->1,2->3,3->2,5->7,8->8] | 1.00 | 188.86746988 | 11 | 83 | 0 | 0 |
| Path 32 | C00026->C00148:[1->1,2->3,3->2,5->7,8->8] | 1.00 | 376.088235294 | 11 | 102 | 0 | 0 |
| Path 33 | C00026->C00148:[1->1,2->3,3->2,5->7,8->8] | 1.00 | 255.487951807 | 12 | 166 | 0 | 0 |
| Path 34 | C00026->C00148:[2->3,3->1,3->2,5->7,8->8] | 1.00 | 243.270833333 | 17 | 96 | 0 | 0 |
| Path 35 | C00026->C00148:[2->3,3->1,3->2,5->7,8->8] | 1.00 | 238.375 | 16 | 96 | 0 | 0 |
| Path 36 | C00026->C00148:[2->3,3->1,3->2,5->7,8->8] | 1.00 | 241.287234043 | 15 | 94 | 0 | 0 |
| Path 37 | C00026->C00148:[1->1,2->3,5->7,8->2,8->8] | 1.00 | 197.828282828 | 14 | 99 | 0 | 0 |
| Path 38 | C00026->C00148:[1->1,2->3,3->2,5->7,8->8] | 1.00 | 239.862903226 | 17 | 124 | 0 | 0 |
| Path 39 | C00026->C00148:[1->1,2->3,3->2,5->7,8->8] | 1.00 | 244.709677419 | 16 | 124 | 0 | 0 |
| Path 40 | C00026->C00148:[2->3,3->1,3->2,5->7,8->8] | 1.00 | 232.107526882 | 14 | 93 | 0 | 0 |
| Path 41 | C00026->C00148:[1->1,2->3,3->2,5->7,8->8] | 1.00 | 272.353293413 | 15 | 167 | 0 | 0 |
| Path 42 | C00026->C00148:[1->1,2->3,3->2,5->7,8->8] | 1.00 | 256.013513514 | 10 | 148 | 0 | 0 |
| Path 43 | C00026->C00148:[2->3,3->1,3->2,5->7,8->8] | 1.00 | 264.67961165 | 20 | 103 | 0 | 0 |
| Path 44 | C00026->C00148:[1->1,2->3,3->2,5->7,8->8] | 1.00 | 244.260162602 | 18 | 123 | 0 | 0 |
| Path 45 | C00026->C00148:[1->1,2->3,3->2,5->7,8->8] | 1.00 | 287.346153846 | 18 | 182 | 0 | 0 |
| Path 46 | C00026->C00148:[1->1,2->3,5->7,8->2,8->8] | 1.00 | 227.53 | 15 | 100 | 0 | 0 |
| Path 47 | C00026->C00148:[1->1,2->3,5->7,8->2,8->8] | 1.00 | 571.761904762 | 14 | 21 | 0 | 0 |
| Path 48 | C00026->C00148:[1->1,2->3,5->7,8->2,8->8] | 1.00 | 599.15 | 13 | 20 | 0 | 0 |
| Path 49 | C00026->C00148:[1->1,2->3,5->7,8->2,8->8] | 1.00 | 185.975308642 | 12 | 81 | 0 | 0 |
| Path 50 | C00026->C00148:[1->1,2->3,5->7,8->2,8->8] | 1.00 | 197.580645161 | 11 | 93 | 0 | 0 |
| Path 51 | C00026->C00148:[1->1,2->3,3->2,5->7,8->8] | 1.00 | 225.843621399 | 17 | 243 | 0 | 0 |
| Path 52 | C00026->C00148:[1->1,2->3,3->1,3->2,5->7,8->8] | 1.00 | 249.146341463 | 17 | 123 | 0 | 0 |
| Path 53 | C00026->C00148:[1->1,2->3,3->2,5->7,8->8] | 1.00 | 234.076612903 | 20 | 248 | 0 | 0 |
| Path 54 | C00026->C00148:[1->1,2->3,3->2,5->7,8->8] | 1.00 | 230.591093117 | 19 | 247 | 0 | 0 |
| Path 55 | C00026->C00148:[2->3,3->1,3->2,5->7,8->8] | 1.00 | 225.64516129 | 15 | 93 | 0 | 0 |
| Path 56 | C00026->C00148:[1->1,2->3,3->2,5->7,8->8] | 1.00 | 227.307017544 | 17 | 228 | 0 | 0 |
| Path 57 | C00026->C00148:[1->1,2->3,5->7,8->2,8->8] | 1.00 | 198.879518072 | 13 | 83 | 0 | 0 |
| Path 58 | C00026->C00148:[1->1,2->3,5->7,8->2,8->8] | 1.00 | 206.27027027 | 13 | 111 | 0 | 0 |
| Path 59 | C00026->C00148:[1->1,2->3,3->2,5->7,8->8] | 1.00 | 289.382857143 | 20 | 175 | 0 | 0 |
| Path 60 | C00026->C00148:[1->1,2->3,3->2,5->7,8->8] | 1.00 | 223.484581498 | 16 | 227 | 0 | 0 |
| Path 61 | C00026->C00148:[1->1,2->3,3->2,5->7,8->8] | 1.00 | 228.106557377 | 17 | 244 | 0 | 0 |
| Path 62 | C00026->C00148:[1->1,2->3,3->2,5->7,8->8] | 1.00 | 457.0 | 4 | 9 | 0 | 0 |
| Path 63 | C00026->C00148:[1->1,2->3,3->2,5->7,8->8] | 1.00 | 225.843621399 | 17 | 243 | 0 | 0 |
| Path 64 | C00026->C00148:[1->1,1->2,2->3,5->7,8->8] | 1.00 | 151.341772152 | 11 | 79 | 0 | 0 |
| Path 65 | C00026->C00148:[1->1,2->3,3->2,5->7,8->8] | 1.00 | 234.076612903 | 20 | 248 | 0 | 0 |
| Path 66 | C00026->C00148:[1->1,2->3,3->2,5->7,8->8] | 1.00 | 172.188235294 | 16 | 170 | 0 | 0 |
| Path 67 | C00026->C00148:[1->1,2->3,3->2,5->7,8->8] | 1.00 | 216.044534413 | 15 | 247 | 0 | 0 |
| Path 68 | C00026->C00148:[1->1,2->3,3->2,5->7,8->8] | 1.00 | 279.87150838 | 16 | 179 | 0 | 0 |
| Path 69 | C00026->C00148:[8->2] | 0.20 | 624.263157895 | 12 | 19 | 0 | 0 |
| Path 70 | C00026->C00148:[1->1,2->3,3->2,5->7,8->8] | 1.00 | 275.292134831 | 15 | 178 | 0 | 0 |
| Path 71 | C00026->C00148:[1->1,2->3,3->2,5->7,8->8] | 1.00 | 224.409836066 | 17 | 244 | 0 | 0 |
| Path 72 | C00026->C00148:[1->1,2->3,3->2,5->7,8->8] | 1.00 | 217.947368421 | 15 | 247 | 0 | 0 |
| Path 73 | C00026->C00148:[1->1,2->3,3->2,5->7,8->8] | 1.00 | 223.901234568 | 17 | 243 | 0 | 0 |
| Path 74 | C00026->C00148:[1->1,2->3,3->2,5->7,8->8] | 1.00 | 222.471111111 | 15 | 225 | 0 | 0 |
| Path 75 | C00026->C00148:[8->2] | 0.20 | 673.25 | 10 | 16 | 0 | 0 |
| Path 76 | C00026->C00148:[8->2] | 0.20 | 645.133333333 | 9 | 15 | 0 | 0 |
| Path 77 | C00026->C00148:[1->1,2->3,3->2,5->7,8->8] | 1.00 | 222.278761062 | 15 | 226 | 0 | 0 |
| Path 78 | C00026->C00148:[1->1,2->3,3->2,5->7,8->8] | 1.00 | 219.5 | 15 | 240 | 0 | 0 |
| Path 79 | C00026->C00148:[1->1,2->3,3->2,5->7,8->8] | 1.00 | 218.343096234 | 14 | 239 | 0 | 0 |
| Path 80 | C00026->C00148:[1->1,2->3,3->2,5->7,8->8] | 1.00 | 226.123348018 | 16 | 227 | 0 | 0 |
| Path 81 | C00026->C00148:[8->2] | 0.20 | 456.483870968 | 12 | 31 | 0 | 0 |
| Path 82 | C00026->C00148:[1->1,2->3,3->2,5->7,8->8] | 1.00 | 227.963265306 | 18 | 245 | 0 | 0 |
| Path 83 | C00026->C00148:[1->1,2->3,3->2,5->7,8->8] | 1.00 | 219.149779736 | 17 | 227 | 0 | 2 |
| Path 84 | C00026->C00148:[1->1,2->3,3->2,5->7,8->8] | 1.00 | 224.727272727 | 16 | 242 | 0 | 0 |
| Path 85 | C00026->C00148:[1->1,2->3,3->2,5->7,8->8] | 1.00 | 218.575892857 | 14 | 224 | 0 | 0 |
| Path 86 | C00026->C00148:[1->1,2->3,3->2,5->7,8->8] | 1.00 | 277.707865169 | 15 | 178 | 0 | 0 |
| Path 87 | C00026->C00148:[8->2] | 0.20 | 406.093023256 | 11 | 43 | 0 | 0 |
| Path 88 | C00026->C00148:[1->1,2->3,3->2,5->7,8->8] | 1.00 | 282.273743017 | 16 | 179 | 0 | 0 |
| Path 89 | C00026->C00148:[1->1,2->3,3->2,5->7,8->8] | 1.00 | 215.403587444 | 13 | 223 | 0 | 0 |
| Path 90 | C00026->C00148:[1->1,2->3,3->2,5->7,8->8] | 1.00 | 278.657303371 | 15 | 178 | 0 | 0 |
| Path 91 | C00026->C00148:[1->1,2->3,3->2,5->7,8->8] | 1.00 | 223.252173913 | 19 | 230 | 0 | 2 |
| Path 92 | C00026->C00148:[1->1,2->3,3->2,5->7,8->8] | 1.00 | 219.5 | 15 | 240 | 0 | 0 |
| Path 93 | C00026->C00148:[1->1,2->3,3->2,5->7,8->8] | 1.00 | 221.817427386 | 15 | 241 | 0 | 0 |
| Path 94 | C00026->C00148:[1->1,2->3,3->2,5->7,8->8] | 1.00 | 219.330357143 | 14 | 224 | 0 | 0 |
| Path 95 | C00026->C00148:[8->2] | 0.20 | 452.878787879 | 12 | 33 | 0 | 0 |
| Path 96 | C00026->C00148:[1->1,2->3,3->2,5->7,8->8] | 1.00 | 227.025974026 | 20 | 231 | 0 | 2 |
| Path 97 | C00026->C00148:[1->1,2->3,3->2,5->7,8->8] | 1.00 | 224.409836066 | 17 | 244 | 0 | 0 |
| Path 98 | C00026->C00148:[1->1,2->3,3->2,5->7,8->8] | 1.00 | 227.963265306 | 18 | 245 | 0 | 0 |
| Path 99 | C00026->C00148:[8->2] | 0.20 | 171.525641026 | 10 | 78 | 0 | 0 |
| Path 100 | C00026->C00148:[1->1,2->3,3->2,5->7,8->8] | 1.00 | 281.7 | 16 | 180 | 0 | 0 |
| Path 101 | C00026->C00148:[8->2] | 0.20 | 606.3125 | 10 | 16 | 0 | 0 |
| Path 102 | C00026->C00148:[8->2] | 0.20 | 390.022727273 | 10 | 44 | 0 | 0 |
| Path 103 | C00026->C00148:[1->1,2->3,5->7,8->8] | 0.80 | 475.5 | 8 | 12 | 0 | 0 |
| Path 104 | C00026->C00148:[1->1,2->3,3->2,5->7,8->8] | 1.00 | 221.817427386 | 15 | 241 | 0 | 0 |
| Path 105 | C00026->C00148:[8->2] | 0.20 | 185.444444444 | 9 | 90 | 0 | 0 |
| Path 106 | C00026->C00148:[8->2] | 0.20 | 432.8125 | 11 | 32 | 0 | 0 |
| Path 107 | C00026->C00148:[1->1,2->3,3->2,5->7,8->8] | 1.00 | 224.727272727 | 16 | 242 | 0 | 0 |
| Path 108 | C00026->C00148:[1->1,2->3,3->2,5->7,8->8] | 1.00 | 216.65625 | 14 | 224 | 0 | 0 |
| Path 109 | C00026->C00148:[1->1,2->3,3->2,5->7,8->8] | 1.00 | 286.193370166 | 17 | 181 | 0 | 0 |
| Path 110 | C00026->C00148:[1->1,2->3,3->2,5->7,8->8] | 1.00 | 225.948497854 | 21 | 233 | 0 | 2 |
| Path 111 | C00026->C00148:[8->2] | 0.20 | 202.688172043 | 11 | 93 | 0 | 0 |
| Path 112 | C00026->C00148:[1->1,2->3,3->2,5->7,8->8] | 1.00 | 214.431718062 | 17 | 227 | 0 | 2 |
| Path 113 | C00026->C00148:[1->1,2->3,3->2,5->7,8->8] | 1.00 | 218.343096234 | 14 | 239 | 0 | 0 |
| Path 114 | C00026->C00148:[8->2] | 0.20 | 183.215189873 | 11 | 79 | 0 | 0 |
| Path 115 | C00026->C00148:[1->1,2->3,3->2,5->7,8->8] | 1.00 | 220.56 | 15 | 225 | 0 | 0 |
| Path 116 | C00026->C00148:[1->1,2->3,3->2,5->7,8->8] | 1.00 | 215.274336283 | 16 | 226 | 0 | 2 |
| Path 117 | C00026->C00148:[1->1,2->3,3->2,5->7,8->8] | 1.00 | 274.04519774 | 14 | 177 | 0 | 0 |
| Path 118 | C00026->C00148:[1->1,2->3,3->2,5->7,8->8] | 1.00 | 214.906504065 | 14 | 246 | 0 | 0 |
| Path 119 | C00026->C00148:[1->1,2->3,3->2,5->7,8->8] | 1.00 | 223.901234568 | 17 | 243 | 0 | 0 |
