# Supplementary material for: 13C-metabolic flux ratio and novel carbon path analyses confirmed that Trichoderma reesei uses primarily the respirative pathway also on the preferred carbon source glucose
Source: BMC Syst Biol. 2009 Oct 29;3:104. doi: 10.1186/1752-0509-3-104 (PMC2776023; doi:10.1186/1752-0509-3-104)
Supplement: Additional file 1 — Pathways discovered in ReTrace carbon path analysis. Graphical and tabular representations of amino acid synthesis pathways discovered in ReTrace carbon path analysis [21]. Self-contained web site: unpack zip archive and open index.html with a web browser. [file 1752-0509-3-104-S1.zip › AF1-treesei/pathways-C00022-to-C00041.html]

Pathways from C00022 to C00041


**Pathways from C00022 to C00041**

**Sources:** Pyruvate; (C00022)

**Target:**L-Alanine; (C00041)

|  | Composite mapping | Z | Average score | Rpairs | Reactions | Zero scores | Scores under threshold |
| --- | --- | --- | --- | --- | --- | --- | --- |
| Path 1 | C00022->C00041:[1->1,2->2,3->3] | 1.00 | 407.054794521 | 17 | 73 | 0 | 0 |
| Path 2 | C00022->C00041:[1->1,2->2,3->3] | 1.00 | 324.868131868 | 14 | 91 | 0 | 0 |
| Path 3 | C00022->C00041:[1->1,2->2,3->3] | 1.00 | 551.325581395 | 13 | 43 | 0 | 0 |
| Path 4 | C00022->C00041:[1->1,2->2,3->3] | 1.00 | 619.838709677 | 17 | 31 | 0 | 0 |
| Path 5 | C00022->C00041:[1->1,2->2,3->3] | 1.00 | 469.577777778 | 13 | 45 | 0 | 0 |
| Path 6 | C00022->C00041:[1->1,2->2,3->3] | 1.00 | 500.14893617 | 17 | 47 | 0 | 0 |
| Path 7 | C00022->C00041:[1->1,2->2,3->3] | 1.00 | 568.75862069 | 12 | 29 | 0 | 0 |
| Path 8 | C00022->C00041:[1->1,2->2,3->3] | 1.00 | 469.068181818 | 17 | 44 | 0 | 0 |
| Path 9 | C00022->C00041:[1->1,2->2,3->3] | 1.00 | 388.493150685 | 15 | 73 | 0 | 0 |
| Path 10 | C00022->C00041:[1->1,2->2,3->3] | 1.00 | 562.578947368 | 14 | 38 | 0 | 0 |
| Path 11 | C00022->C00041:[1->1,2->2,3->3] | 1.00 | 354.507936508 | 12 | 63 | 0 | 0 |
| Path 12 | C00022->C00041:[1->1,2->2,3->3] | 1.00 | 473.017857143 | 14 | 56 | 0 | 0 |
| Path 13 | C00022->C00041:[1->1,2->2,3->3] | 1.00 | 533.27027027 | 13 | 37 | 0 | 0 |
| Path 14 | C00022->C00041:[1->1,2->2,3->3] | 1.00 | 99.1739130435 | 1 | 23 | 0 | 0 |
| Path 15 | C00022->C00041:[1->1,2->2,3->3] | 1.00 | 311.523255814 | 15 | 86 | 0 | 0 |
| Path 16 | C00022->C00041:[1->1,2->2,3->3] | 1.00 | 376.294117647 | 16 | 68 | 0 | 0 |
| Path 17 | C00022->C00041:[1->1,2->2,3->3] | 1.00 | 495.173913043 | 14 | 46 | 0 | 0 |
| Path 18 | C00022->C00041:[1->1,2->2,3->3] | 1.00 | 416.693877551 | 16 | 49 | 0 | 0 |
| Path 19 | C00022->C00041:[1->1,2->2,3->3] | 1.00 | 471.319148936 | 15 | 47 | 0 | 0 |
| Path 20 | C00022->C00041:[1->1,2->2,3->3] | 1.00 | 405.5 | 16 | 74 | 0 | 0 |
| Path 21 | C00022->C00041:[1->1,2->2,3->3] | 1.00 | 441.674418605 | 16 | 43 | 0 | 0 |
| Path 22 | C00022->C00041:[1->1,2->2,3->3] | 1.00 | 396.220588235 | 18 | 68 | 0 | 0 |
| Path 23 | C00022->C00041:[1->1,2->2,3->3] | 1.00 | 359.307692308 | 14 | 65 | 0 | 0 |
| Path 24 | C00022->C00041:[1->1,2->2,3->3] | 1.00 | 495.8125 | 16 | 48 | 0 | 0 |
| Path 25 | C00022->C00041:[1->1,1->3,2->2] | 1.00 | 454.380952381 | 18 | 63 | 0 | 1 |
| Path 26 | C00022->C00041:[1->1,2->2,3->3] | 1.00 | 331.06557377 | 15 | 61 | 0 | 0 |
| Path 27 | C00022->C00041:[1->1,2->2,3->3] | 1.00 | 215.6 | 2 | 5 | 0 | 0 |
| Path 28 | C00022->C00041:[1->1,2->2,3->3] | 1.00 | 651.9375 | 18 | 32 | 0 | 0 |
| Path 29 | C00022->C00041:[1->1,2->2,3->3] | 1.00 | 536.194444444 | 15 | 36 | 0 | 0 |
| Path 30 | C00022->C00041:[1->1,2->2,3->3] | 1.00 | 593.096774194 | 14 | 31 | 0 | 0 |
| Path 31 | C00022->C00041:[1->1,2->2,3->3] | 1.00 | 516.196078431 | 16 | 51 | 0 | 0 |
| Path 32 | C00022->C00041:[1->1,2->2,3->3] | 1.00 | 394.710144928 | 17 | 69 | 0 | 0 |
| Path 33 | C00022->C00041:[1->1,2->2,3->3] | 1.00 | 499.688888889 | 15 | 45 | 0 | 0 |
| Path 34 | C00022->C00041:[1->1,2->2,3->3] | 1.00 | 451.672727273 | 13 | 55 | 0 | 0 |
| Path 35 | C00022->C00041:[1->1,2->2,3->3] | 1.00 | 356.780821918 | 12 | 73 | 0 | 0 |
| Path 36 | C00022->C00041:[1->1,1->3,2->2] | 1.00 | 445.5 | 17 | 64 | 0 | 0 |
| Path 37 | C00022->C00041:[1->1,2->2,3->3] | 1.00 | 380.618181818 | 12 | 55 | 0 | 0 |
| Path 38 | C00022->C00041:[1->1,2->2,3->3] | 1.00 | 516.567567568 | 15 | 37 | 0 | 0 |
| Path 39 | C00022->C00041:[1->1,2->2,3->3] | 1.00 | 476.309090909 | 15 | 55 | 0 | 0 |
| Path 40 | C00022->C00041:[1->1,2->2,3->3] | 1.00 | 582.133333333 | 14 | 30 | 0 | 0 |
| Path 41 | C00022->C00041:[1->1,2->2,3->3] | 1.00 | 290.910714286 | 19 | 112 | 0 | 0 |
| Path 42 | C00022->C00041:[1->1,2->2,3->3] | 1.00 | 332.24137931 | 18 | 58 | 0 | 1 |
| Path 43 | C00022->C00041:[1->1,2->2,3->3] | 1.00 | 497.035087719 | 27 | 57 | 0 | 1 |
| Path 44 | C00022->C00041:[1->1,2->2,3->3] | 1.00 | 272.025641026 | 17 | 117 | 0 | 0 |
| Path 45 | C00022->C00041:[3->1] | 0.33 | 435.70212766 | 12 | 47 | 0 | 0 |
| Path 46 | C00022->C00041:[1->1,2->2,3->3] | 1.00 | 263.596899225 | 20 | 129 | 0 | 0 |
| Path 47 | C00022->C00041:[1->1,2->2,3->3] | 1.00 | 457.511627907 | 16 | 43 | 0 | 0 |
| Path 48 | C00022->C00041:[1->1,2->2,3->3] | 1.00 | 412.761904762 | 17 | 63 | 0 | 0 |
| Path 49 | C00022->C00041:[1->1,2->2,3->3] | 1.00 | 368.762886598 | 22 | 97 | 0 | 1 |
| Path 50 | C00022->C00041:[1->1,2->2,3->3] | 1.00 | 539.289473684 | 13 | 38 | 0 | 0 |
| Path 51 | C00022->C00041:[1->1,2->2,3->3] | 1.00 | 368.835294118 | 19 | 85 | 0 | 0 |
| Path 52 | C00022->C00041:[1->1,2->2,3->3] | 1.00 | 532.948717949 | 12 | 39 | 0 | 0 |
| Path 53 | C00022->C00041:[1->1,2->2,3->3] | 1.00 | 503.631578947 | 11 | 38 | 0 | 0 |
| Path 54 | C00022->C00041:[1->1,2->2,3->3] | 1.00 | 348.924528302 | 15 | 53 | 0 | 0 |
| Path 55 | C00022->C00041:[1->1,2->2,3->3] | 1.00 | 327.416666667 | 19 | 60 | 0 | 1 |
| Path 56 | C00022->C00041:[1->1,2->2,3->3] | 1.00 | 500.980392157 | 13 | 51 | 0 | 0 |
| Path 57 | C00022->C00041:[1->1,2->2,3->3] | 1.00 | 303.724637681 | 16 | 69 | 0 | 0 |
| Path 58 | C00022->C00041:[3->2] | 0.33 | 550.181818182 | 9 | 11 | 0 | 0 |
| Path 59 | C00022->C00041:[1->1,2->2,3->3] | 1.00 | 246.846153846 | 21 | 143 | 0 | 1 |
| Path 60 | C00022->C00041:[1->1,2->2,3->3] | 1.00 | 386.803278689 | 14 | 61 | 0 | 0 |
| Path 61 | C00022->C00041:[1->1,2->2,3->3] | 1.00 | 366.637931034 | 14 | 58 | 0 | 0 |
| Path 62 | C00022->C00041:[1->1,2->2,3->3] | 1.00 | 546.321428571 | 14 | 28 | 0 | 0 |
| Path 63 | C00022->C00041:[1->1,2->2,3->3] | 1.00 | 521.25 | 12 | 36 | 0 | 0 |
| Path 64 | C00022->C00041:[1->1,2->2,3->3] | 1.00 | 290.722772277 | 16 | 101 | 0 | 0 |
| Path 65 | C00022->C00041:[1->1,2->2,3->3] | 1.00 | 376.023255814 | 11 | 43 | 0 | 0 |
| Path 66 | C00022->C00041:[1->1,2->2,3->3] | 1.00 | 638.961538462 | 13 | 26 | 0 | 0 |
| Path 67 | C00022->C00041:[3->2] | 0.33 | 477.875 | 10 | 16 | 0 | 0 |
| Path 68 | C00022->C00041:[1->1,2->2,3->3] | 1.00 | 272.741935484 | 19 | 124 | 0 | 0 |
| Path 69 | C00022->C00041:[1->1,2->2,3->3] | 1.00 | 352.938461538 | 22 | 65 | 0 | 1 |
| Path 70 | C00022->C00041:[1->1,2->2,3->3] | 1.00 | 307.984848485 | 14 | 66 | 0 | 0 |
| Path 71 | C00022->C00041:[1->1,2->2,3->3] | 1.00 | 393.12 | 26 | 75 | 0 | 1 |
| Path 72 | C00022->C00041:[1->1,2->2,3->3] | 1.00 | 526.0 | 13 | 27 | 0 | 0 |
| Path 73 | C00022->C00041:[1->1,2->2,3->3] | 1.00 | 309.333333333 | 18 | 99 | 0 | 0 |
| Path 74 | C00022->C00041:[1->1,2->2,3->3] | 1.00 | 380.847619048 | 27 | 105 | 0 | 1 |
| Path 75 | C00022->C00041:[1->1,2->2,3->3] | 1.00 | 473.15625 | 14 | 32 | 0 | 0 |
| Path 76 | C00022->C00041:[1->1,2->2,3->3] | 1.00 | 439.095238095 | 20 | 42 | 0 | 1 |
| Path 77 | C00022->C00041:[1->2,3->1] | 0.67 | 375.958333333 | 18 | 72 | 0 | 1 |
| Path 78 | C00022->C00041:[2->2,3->1] | 0.67 | 427.847457627 | 13 | 59 | 0 | 0 |
| Path 79 | C00022->C00041:[1->1,2->2,3->3] | 1.00 | 334.576923077 | 14 | 52 | 0 | 0 |
| Path 80 | C00022->C00041:[1->1,2->2,3->3] | 1.00 | 274.648648649 | 18 | 111 | 0 | 0 |
| Path 81 | C00022->C00041:[1->1,2->2,3->3] | 1.00 | 391.253968254 | 15 | 63 | 0 | 0 |
| Path 82 | C00022->C00041:[1->1,2->2,3->3] | 1.00 | 354.104477612 | 24 | 67 | 0 | 1 |
| Path 83 | C00022->C00041:[1->1,2->2,3->3] | 1.00 | 365.024096386 | 18 | 83 | 0 | 0 |
| Path 84 | C00022->C00041:[1->1,2->2,3->3] | 1.00 | 307.8375 | 16 | 80 | 0 | 0 |
| Path 85 | C00022->C00041:[1->1,2->2,3->3] | 1.00 | 308.421568627 | 17 | 102 | 0 | 0 |
| Path 86 | C00022->C00041:[1->1,2->2,3->3] | 1.00 | 354.051948052 | 16 | 77 | 0 | 0 |
| Path 87 | C00022->C00041:[1->1,2->2,3->3] | 1.00 | 359.443181818 | 24 | 88 | 0 | 1 |
| Path 88 | C00022->C00041:[1->1,2->2,3->3] | 1.00 | 298.607843137 | 17 | 102 | 0 | 0 |
| Path 89 | C00022->C00041:[1->1,2->2,3->3] | 1.00 | 340.467741935 | 14 | 62 | 0 | 0 |
| Path 90 | C00022->C00041:[1->1,2->2,3->3] | 1.00 | 284.836538462 | 16 | 104 | 0 | 0 |
| Path 91 | C00022->C00041:[1->1,2->2,3->3] | 1.00 | 327.2 | 19 | 100 | 0 | 0 |
| Path 92 | C00022->C00041:[1->1,2->2,3->3] | 1.00 | 276.382022472 | 15 | 89 | 0 | 0 |
| Path 93 | C00022->C00041:[1->1,2->2,3->3] | 1.00 | 315.065420561 | 21 | 107 | 0 | 0 |
| Path 94 | C00022->C00041:[1->1,2->2,3->3] | 1.00 | 313.898734177 | 13 | 79 | 0 | 0 |
| Path 95 | C00022->C00041:[1->1,2->2,3->3] | 1.00 | 360.19047619 | 18 | 84 | 0 | 0 |
| Path 96 | C00022->C00041:[1->1,2->2,3->3] | 1.00 | 341.941176471 | 14 | 51 | 0 | 0 |
| Path 97 | C00022->C00041:[1->1,2->2,3->3] | 1.00 | 360.175824176 | 26 | 91 | 0 | 1 |
| Path 98 | C00022->C00041:[1->1,2->2,3->3] | 1.00 | 334.641975309 | 16 | 81 | 0 | 0 |
| Path 99 | C00022->C00041:[1->1,2->2,3->3] | 1.00 | 235.802919708 | 25 | 137 | 0 | 1 |
| Path 100 | C00022->C00041:[1->1,2->2,3->3] | 1.00 | 269.282894737 | 25 | 152 | 0 | 1 |
| Path 101 | C00022->C00041:[1->2,3->1] | 0.67 | 436.12962963 | 16 | 54 | 0 | 1 |
| Path 102 | C00022->C00041:[1->2,3->1] | 0.67 | 452.288461538 | 16 | 52 | 0 | 0 |
| Path 103 | C00022->C00041:[1->1,2->2,3->3] | 1.00 | 394.269230769 | 26 | 78 | 0 | 1 |
| Path 104 | C00022->C00041:[1->1,2->2,3->3] | 1.00 | 326.88 | 13 | 50 | 0 | 0 |
| Path 105 | C00022->C00041:[1->1,2->2,3->3] | 1.00 | 352.444444444 | 15 | 63 | 0 | 0 |
| Path 106 | C00022->C00041:[1->1,1->3,2->2] | 1.00 | 456.673076923 | 16 | 52 | 0 | 0 |
| Path 107 | C00022->C00041:[1->1,2->2,3->3] | 1.00 | 466.688888889 | 16 | 45 | 0 | 0 |
| Path 108 | C00022->C00041:[1->1,2->2,3->3] | 1.00 | 377.806451613 | 17 | 62 | 0 | 0 |
| Path 109 | C00022->C00041:[1->2,3->1] | 0.67 | 369.246575342 | 17 | 73 | 0 | 0 |
| Path 110 | C00022->C00041:[1->1,2->2,3->3] | 1.00 | 294.268907563 | 19 | 119 | 0 | 0 |
| Path 111 | C00022->C00041:[1->1,2->2,3->3] | 1.00 | 405.25 | 15 | 48 | 0 | 0 |
| Path 112 | C00022->C00041:[1->1,2->2,3->3] | 1.00 | 478.06 | 12 | 50 | 0 | 0 |
| Path 113 | C00022->C00041:[1->1,2->2,3->3] | 1.00 | 261.708609272 | 26 | 151 | 0 | 1 |
| Path 114 | C00022->C00041:[1->1,2->2,3->3] | 1.00 | 343.914634146 | 17 | 82 | 0 | 0 |
| Path 115 | C00022->C00041:[1->1,2->2,3->3] | 1.00 | 494.0 | 16 | 35 | 0 | 0 |
| Path 116 | C00022->C00041:[1->1,2->2,3->3] | 1.00 | 298.725806452 | 16 | 62 | 0 | 0 |
| Path 117 | C00022->C00041:[1->1,2->2,3->3] | 1.00 | 350.5 | 23 | 70 | 0 | 1 |
| Path 118 | C00022->C00041:[1->1,2->2,3->3] | 1.00 | 311.881188119 | 17 | 101 | 0 | 0 |
| Path 119 | C00022->C00041:[1->1,2->2,3->3] | 1.00 | 397.22972973 | 26 | 74 | 0 | 1 |
| Path 120 | C00022->C00041:[1->1,2->2,3->3] | 1.00 | 394.774647887 | 25 | 71 | 0 | 1 |
| Path 121 | C00022->C00041:[1->1,2->2,3->3] | 1.00 | 492.0 | 15 | 33 | 0 | 0 |
| Path 122 | C00022->C00041:[1->1,2->2,3->3] | 1.00 | 389.19047619 | 18 | 63 | 0 | 0 |
| Path 123 | C00022->C00041:[1->1,2->2,3->3] | 1.00 | 344.302631579 | 15 | 76 | 0 | 0 |
| Path 124 | C00022->C00041:[1->1,2->2,3->3] | 1.00 | 531.627906977 | 15 | 43 | 0 | 0 |
| Path 125 | C00022->C00041:[1->1,2->2,3->3] | 1.00 | 373.18 | 16 | 50 | 0 | 0 |
| Path 126 | C00022->C00041:[1->1,2->2,3->3] | 1.00 | 283.133333333 | 19 | 120 | 0 | 0 |
| Path 127 | C00022->C00041:[1->1,2->2,3->3] | 1.00 | 373.6 | 13 | 60 | 0 | 0 |
| Path 128 | C00022->C00041:[1->1,2->2,3->3] | 1.00 | 324.420560748 | 21 | 107 | 0 | 0 |
| Path 129 | C00022->C00041:[1->1,2->2,3->3] | 1.00 | 505.16 | 14 | 50 | 0 | 0 |
| Path 130 | C00022->C00041:[1->1,2->2,3->3] | 1.00 | 329.507042254 | 16 | 71 | 0 | 0 |
| Path 131 | C00022->C00041:[1->1,2->2,3->3] | 1.00 | 498.418604651 | 14 | 43 | 0 | 0 |
| Path 132 | C00022->C00041:[1->1,2->2,3->3] | 1.00 | 321.303921569 | 20 | 102 | 0 | 0 |
| Path 133 | C00022->C00041:[1->1,2->2,3->3] | 1.00 | 493.727272727 | 13 | 44 | 0 | 0 |
| Path 134 | C00022->C00041:[2->2] | 0.33 | 230.914893617 | 11 | 94 | 0 | 0 |
| Path 135 | C00022->C00041:[1->1,2->2,3->3] | 1.00 | 452.409090909 | 15 | 44 | 0 | 0 |
| Path 136 | C00022->C00041:[1->1,2->2,3->3] | 1.00 | 319.160493827 | 14 | 81 | 0 | 0 |
| Path 137 | C00022->C00041:[1->1,2->2,3->3] | 1.00 | 316.058252427 | 18 | 103 | 0 | 0 |
| Path 138 | C00022->C00041:[1->1,2->2,3->3] | 1.00 | 398.428571429 | 25 | 70 | 0 | 1 |
| Path 139 | C00022->C00041:[1->1,2->2,3->3] | 1.00 | 275.4875 | 18 | 80 | 0 | 0 |
| Path 140 | C00022->C00041:[1->1,2->2,3->3] | 1.00 | 331.174603175 | 19 | 63 | 0 | 1 |
| Path 141 | C00022->C00041:[1->1,2->2,3->3] | 1.00 | 299.631578947 | 15 | 95 | 0 | 0 |
| Path 142 | C00022->C00041:[1->1,2->2,3->3] | 1.00 | 259.808080808 | 17 | 99 | 0 | 0 |
| Path 143 | C00022->C00041:[1->1,2->2,3->3] | 1.00 | 466.906976744 | 12 | 43 | 0 | 0 |
| Path 144 | C00022->C00041:[1->1,2->2,3->3] | 1.00 | 348.852459016 | 11 | 61 | 0 | 0 |
| Path 145 | C00022->C00041:[1->1,2->2,3->3] | 1.00 | 361.976190476 | 10 | 42 | 0 | 0 |
| Path 146 | C00022->C00041:[1->1,2->2,3->3] | 1.00 | 288.185185185 | 13 | 54 | 0 | 0 |
| Path 147 | C00022->C00041:[1->1,2->2,3->3] | 1.00 | 310.014084507 | 17 | 71 | 0 | 0 |
| Path 148 | C00022->C00041:[1->1,2->2,3->3] | 1.00 | 330.610169492 | 19 | 59 | 0 | 1 |
| Path 149 | C00022->C00041:[1->1,2->2,3->3] | 1.00 | 486.333333333 | 14 | 39 | 0 | 0 |
| Path 150 | C00022->C00041:[1->1,2->2,3->3] | 1.00 | 641.806451613 | 17 | 31 | 0 | 0 |
| Path 151 | C00022->C00041:[1->1,2->2,3->3] | 1.00 | 392.603773585 | 18 | 53 | 0 | 0 |
| Path 152 | C00022->C00041:[1->1,2->2,3->3] | 1.00 | 317.555555556 | 17 | 81 | 0 | 0 |
| Path 153 | C00022->C00041:[1->1,2->2,3->3] | 1.00 | 598.64 | 12 | 25 | 0 | 0 |
| Path 154 | C00022->C00041:[1->1,2->2,3->3] | 1.00 | 291.170212766 | 14 | 94 | 0 | 0 |
| Path 155 | C00022->C00041:[1->1,2->2,3->3] | 1.00 | 410.875 | 16 | 64 | 0 | 0 |
| Path 156 | C00022->C00041:[1->1,2->2,3->3] | 1.00 | 400.782608696 | 24 | 69 | 0 | 1 |
| Path 157 | C00022->C00041:[1->1,2->2,3->3] | 1.00 | 625.88 | 12 | 25 | 0 | 0 |
| Path 158 | C00022->C00041:[1->1,2->2,3->3] | 1.00 | 551.675675676 | 13 | 37 | 0 | 0 |
| Path 159 | C00022->C00041:[1->2,3->1] | 0.67 | 445.214285714 | 18 | 56 | 0 | 1 |
| Path 160 | C00022->C00041:[1->1,2->2,3->3] | 1.00 | 369.777777778 | 11 | 54 | 0 | 0 |
| Path 161 | C00022->C00041:[1->1,2->2,3->3] | 1.00 | 294.04 | 16 | 100 | 0 | 0 |
| Path 162 | C00022->C00041:[1->1,2->2,3->3] | 1.00 | 392.265822785 | 27 | 79 | 0 | 1 |
| Path 163 | C00022->C00041:[1->1,2->2,3->3] | 1.00 | 501.55 | 15 | 40 | 0 | 0 |
| Path 164 | C00022->C00041:[1->1,2->2,3->3] | 1.00 | 506.150943396 | 26 | 53 | 0 | 1 |
| Path 165 | C00022->C00041:[1->1,2->2,3->3] | 1.00 | 503.798245614 | 18 | 114 | 0 | 0 |
| Path 166 | C00022->C00041:[1->1,2->2,3->3] | 1.00 | 390.58490566 | 26 | 106 | 0 | 1 |
| Path 167 | C00022->C00041:[1->1,2->2,3->3] | 1.00 | 277.692307692 | 21 | 130 | 0 | 0 |
| Path 168 | C00022->C00041:[1->1,2->2,3->3] | 1.00 | 285.949494949 | 15 | 99 | 0 | 0 |
| Path 169 | C00022->C00041:[1->1,2->2,3->3] | 1.00 | 399.438356164 | 25 | 73 | 0 | 1 |
| Path 170 | C00022->C00041:[1->1,2->2,3->3] | 1.00 | 537.333333333 | 16 | 42 | 0 | 0 |
| Path 171 | C00022->C00041:[1->1,2->2,3->3] | 1.00 | 313.643564356 | 19 | 101 | 0 | 0 |
| Path 172 | C00022->C00041:[1->1,2->2,3->3] | 1.00 | 217.720930233 | 20 | 129 | 0 | 1 |
| Path 173 | C00022->C00041:[1->1,2->2,3->3] | 1.00 | 398.428571429 | 25 | 70 | 0 | 1 |
| Path 174 | C00022->C00041:[1->1,2->2,3->3] | 1.00 | 395.243243243 | 25 | 74 | 0 | 1 |
| Path 175 | C00022->C00041:[1->1,2->2,3->3] | 1.00 | 351.652777778 | 25 | 72 | 0 | 1 |
| Path 176 | C00022->C00041:[1->1,2->2,3->3] | 1.00 | 232.537313433 | 23 | 134 | 0 | 1 |
| Path 177 | C00022->C00041:[1->1,1->3,2->2] | 1.00 | 467.862745098 | 17 | 51 | 0 | 1 |
| Path 178 | C00022->C00041:[1->1,2->2,3->3] | 1.00 | 379.096153846 | 17 | 52 | 0 | 0 |
| Path 179 | C00022->C00041:[1->1,2->2,3->3] | 1.00 | 279.32 | 20 | 125 | 0 | 0 |
| Path 180 | C00022->C00041:[1->1,2->2,3->3] | 1.00 | 334.801980198 | 20 | 101 | 0 | 0 |
| Path 181 | C00022->C00041:[1->1,2->2,3->3] | 1.00 | 304.05 | 16 | 100 | 0 | 0 |
| Path 182 | C00022->C00041:[1->1,2->2,3->3] | 1.00 | 358.695652174 | 13 | 46 | 0 | 0 |
| Path 183 | C00022->C00041:[1->1,2->2,3->3] | 1.00 | 397.22972973 | 26 | 74 | 0 | 1 |
| Path 184 | C00022->C00041:[1->1,2->2,3->3] | 1.00 | 353.859649123 | 13 | 57 | 0 | 0 |
| Path 185 | C00022->C00041:[1->1,2->2,3->3] | 1.00 | 339.277108434 | 17 | 83 | 0 | 0 |
| Path 186 | C00022->C00041:[1->1,2->2,3->3] | 1.00 | 572.0 | 12 | 27 | 0 | 0 |
| Path 187 | C00022->C00041:[3->1] | 0.33 | 447.652173913 | 13 | 46 | 0 | 1 |
| Path 188 | C00022->C00041:[1->2,3->1] | 0.67 | 426.127272727 | 15 | 55 | 0 | 0 |
| Path 189 | C00022->C00041:[1->1,2->2,3->3] | 1.00 | 354.104477612 | 24 | 67 | 0 | 1 |
| Path 190 | C00022->C00041:[1->1,2->2,3->3] | 1.00 | 317.19 | 19 | 100 | 0 | 0 |
| Path 191 | C00022->C00041:[1->1,2->2,3->3] | 1.00 | 505.071428571 | 14 | 42 | 0 | 0 |
| Path 192 | C00022->C00041:[1->1,2->2,3->3] | 1.00 | 363.232142857 | 10 | 56 | 0 | 0 |
| Path 193 | C00022->C00041:[1->1,2->2,3->3] | 1.00 | 476.323529412 | 15 | 34 | 0 | 0 |
| Path 194 | C00022->C00041:[1->1,2->2,3->3] | 1.00 | 453.22 | 25 | 50 | 0 | 1 |
| Path 195 | C00022->C00041:[1->1,2->2,3->3] | 1.00 | 608.3 | 16 | 30 | 0 | 0 |
| Path 196 | C00022->C00041:[1->1,2->2,3->3] | 1.00 | 350.941176471 | 24 | 68 | 0 | 1 |
| Path 197 | C00022->C00041:[1->1,2->2,3->3] | 1.00 | 409.016393443 | 16 | 61 | 0 | 0 |
| Path 198 | C00022->C00041:[1->1,2->2,3->3] | 1.00 | 331.555555556 | 22 | 108 | 0 | 0 |
| Path 199 | C00022->C00041:[1->1,2->2,3->3] | 1.00 | 387.333333333 | 17 | 51 | 0 | 0 |
| Path 200 | C00022->C00041:[1->1,2->2,3->3] | 1.00 | 393.786666667 | 26 | 75 | 0 | 1 |
| Path 201 | C00022->C00041:[2->2,3->1] | 0.67 | 437.189655172 | 14 | 58 | 0 | 1 |
| Path 202 | C00022->C00041:[1->1,2->2,3->3] | 1.00 | 583.333333333 | 11 | 24 | 0 | 0 |
| Path 203 | C00022->C00041:[1->1,2->2,3->3] | 1.00 | 344.060240964 | 21 | 83 | 0 | 1 |
| Path 204 | C00022->C00041:[1->1,2->2,3->3] | 1.00 | 557.44 | 11 | 25 | 0 | 0 |
| Path 205 | C00022->C00041:[1->1,2->2,3->3] | 1.00 | 340.138888889 | 17 | 72 | 0 | 0 |
| Path 206 | C00022->C00041:[1->1,2->2,3->3] | 1.00 | 276.31092437 | 18 | 119 | 0 | 0 |
| Path 207 | C00022->C00041:[1->1,2->2,3->3] | 1.00 | 329.6875 | 20 | 64 | 0 | 1 |
| Path 208 | C00022->C00041:[1->1,2->2,3->3] | 1.00 | 287.483050847 | 18 | 118 | 0 | 0 |
| Path 209 | C00022->C00041:[1->1,2->2,3->3] | 1.00 | 330.610169492 | 19 | 59 | 0 | 1 |
| Path 210 | C00022->C00041:[1->1,2->2,3->3] | 1.00 | 298.8 | 16 | 70 | 0 | 0 |
| Path 211 | C00022->C00041:[1->1,2->2,3->3] | 1.00 | 407.129032258 | 15 | 62 | 0 | 0 |
| Path 212 | C00022->C00041:[1->2,3->1] | 0.67 | 435.403508772 | 17 | 57 | 0 | 0 |
| Path 213 | C00022->C00041:[1->1,2->2,3->3] | 1.00 | 320.483333333 | 14 | 60 | 0 | 0 |
| Path 214 | C00022->C00041:[1->1,2->2,3->3] | 1.00 | 292.088235294 | 15 | 68 | 0 | 0 |
| Path 215 | C00022->C00041:[1->1,2->2,3->3] | 1.00 | 293.738095238 | 21 | 126 | 0 | 0 |
| Path 216 | C00022->C00041:[1->1,2->2,3->3] | 1.00 | 369.205882353 | 11 | 68 | 0 | 0 |
| Path 217 | C00022->C00041:[1->1,2->2,3->3] | 1.00 | 348.273809524 | 18 | 84 | 0 | 0 |
| Path 218 | C00022->C00041:[1->1,2->2,3->3] | 1.00 | 285.477777778 | 16 | 90 | 0 | 0 |
| Path 219 | C00022->C00041:[1->1,2->2,3->3] | 1.00 | 342.333333333 | 12 | 45 | 0 | 0 |
| Path 220 | C00022->C00041:[1->1,2->2,3->3] | 1.00 | 267.252427184 | 15 | 103 | 0 | 0 |
| Path 221 | C00022->C00041:[1->1,2->2,3->3] | 1.00 | 287.328 | 20 | 125 | 0 | 0 |
| Path 222 | C00022->C00041:[1->1,2->2,3->3] | 1.00 | 279.0 | 18 | 118 | 0 | 0 |
| Path 223 | C00022->C00041:[1->1,2->2,3->3] | 1.00 | 307.70754717 | 20 | 106 | 0 | 0 |
| Path 224 | C00022->C00041:[1->2,3->1] | 0.67 | 463.392156863 | 17 | 51 | 0 | 1 |
| Path 225 | C00022->C00041:[1->1,2->2,3->3] | 1.00 | 356.12195122 | 17 | 82 | 0 | 0 |
| Path 226 | C00022->C00041:[1->1,2->2,3->3] | 1.00 | 429.19047619 | 15 | 42 | 0 | 0 |
| Path 227 | C00022->C00041:[1->1,2->2,3->3] | 1.00 | 633.381578947 | 16 | 76 | 0 | 0 |
