# Supplementary material for: 13C-metabolic flux ratio and novel carbon path analyses confirmed that Trichoderma reesei uses primarily the respirative pathway also on the preferred carbon source glucose
Source: BMC Syst Biol. 2009 Oct 29;3:104. doi: 10.1186/1752-0509-3-104 (PMC2776023; doi:10.1186/1752-0509-3-104)
Supplement: Additional file 1 — Pathways discovered in ReTrace carbon path analysis. Graphical and tabular representations of amino acid synthesis pathways discovered in ReTrace carbon path analysis [21]. Self-contained web site: unpack zip archive and open index.html with a web browser. [file 1752-0509-3-104-S1.zip › AF1-treesei/pathways-C00117-to-C00135.html]

Pathways from C00117 to C00135


**Pathways from C00117 to C00135**

**Sources:** D-Ribose 5-phosphate; (C00117)

**Target:**L-Histidine; (C00135)

|  | Composite mapping | Z | Average score | Rpairs | Reactions | Zero scores | Scores under threshold |
| --- | --- | --- | --- | --- | --- | --- | --- |
| Path 1 | C00117->C00135:[10->1,10->7,12->3,6->8,7->5,8->2] | 1.00 | 391.72 | 24 | 150 | 0 | 0 |
| Path 2 | C00117->C00135:[10->1,12->3,6->7,6->8,7->5,8->2] | 1.00 | 377.937931034 | 24 | 145 | 0 | 0 |
| Path 3 | C00117->C00135:[10->1,12->3,6->7,6->8,7->5,8->2] | 1.00 | 388.572413793 | 25 | 145 | 0 | 0 |
| Path 4 | C00117->C00135:[10->1,12->3,6->7,6->8,7->5,8->2] | 1.00 | 385.748251748 | 25 | 143 | 0 | 0 |
| Path 5 | C00117->C00135:[10->1,12->3,6->7,6->8,7->5,8->2] | 1.00 | 392.236111111 | 25 | 144 | 0 | 0 |
| Path 6 | C00117->C00135:[10->1,12->3,6->7,6->8,7->5,8->2] | 1.00 | 378.155844156 | 27 | 154 | 0 | 0 |
| Path 7 | C00117->C00135:[10->1,12->3,6->7,6->8,7->5,8->2] | 1.00 | 387.725352113 | 24 | 142 | 0 | 0 |
| Path 8 | C00117->C00135:[10->1,12->3,12->7,6->8,7->5,8->2] | 1.00 | 376.826388889 | 23 | 144 | 0 | 0 |
| Path 9 | C00117->C00135:[10->1,12->3,12->7,6->8,7->5,8->2] | 1.00 | 377.718309859 | 22 | 142 | 0 | 0 |
| Path 10 | C00117->C00135:[10->1,12->3,6->7,6->8,7->5,8->2] | 1.00 | 385.380952381 | 25 | 147 | 0 | 0 |
| Path 11 | C00117->C00135:[10->1,12->3,6->7,6->8,7->5,8->2] | 1.00 | 689.290322581 | 25 | 31 | 0 | 0 |
| Path 12 | C00117->C00135:[6->8,7->5,8->2,8->7] | 0.67 | 421.413793103 | 31 | 174 | 0 | 0 |
| Path 13 | C00117->C00135:[6->8,7->5,8->2] | 0.50 | 774.0 | 20 | 23 | 0 | 0 |
| Path 14 | C00117->C00135:[6->7,6->8,7->5,8->2] | 0.67 | 418.873333333 | 31 | 150 | 0 | 0 |
| Path 15 | C00117->C00135:[6->7,6->8,7->5,8->2] | 0.67 | 409.253164557 | 32 | 158 | 0 | 0 |
| Path 16 | C00117->C00135:[6->7,6->8,7->5,8->2] | 0.67 | 419.815789474 | 32 | 152 | 0 | 0 |
| Path 17 | C00117->C00135:[10->1,12->3,6->8,7->5,8->2] | 0.83 | 613.454545455 | 11 | 11 | 0 | 0 |
| Path 18 | C00117->C00135:[6->7,6->8,7->5,8->2] | 0.67 | 412.584415584 | 31 | 154 | 0 | 0 |
| Path 19 | C00117->C00135:[6->7,6->8,7->5,8->2] | 0.67 | 415.731543624 | 30 | 149 | 0 | 0 |
| Path 20 | C00117->C00135:[6->7,6->8,7->5,8->2] | 0.67 | 430.683544304 | 32 | 158 | 0 | 0 |
| Path 21 | C00117->C00135:[6->7] | 0.17 | 387.524822695 | 23 | 141 | 0 | 0 |
