# Supplementary material for: 13C-metabolic flux ratio and novel carbon path analyses confirmed that Trichoderma reesei uses primarily the respirative pathway also on the preferred carbon source glucose
Source: BMC Syst Biol. 2009 Oct 29;3:104. doi: 10.1186/1752-0509-3-104 (PMC2776023; doi:10.1186/1752-0509-3-104)
Supplement: Additional file 1 — Pathways discovered in ReTrace carbon path analysis. Graphical and tabular representations of amino acid synthesis pathways discovered in ReTrace carbon path analysis [21]. Self-contained web site: unpack zip archive and open index.html with a web browser. [file 1752-0509-3-104-S1.zip › AF1-treesei/pathways-C00031-to-C00251.html]

Pathways from C00031 to C00251


**Pathways from C00031 to C00251**

**Sources:** D-Glucose; (C00031)

**Target:**Chorismate; (C00251)

|  | Composite mapping | Z | Average score | Rpairs | Reactions | Zero scores | Scores under threshold |
| --- | --- | --- | --- | --- | --- | --- | --- |
| Path 1 | C00031->C00251:[1->6,2->3,4->10,4->11,4->9,5->1,7->12,7->2,9->5,9->8] | 1.00 | 582.107692308 | 24 | 65 | 0 | 0 |
| Path 2 | C00031->C00251:[1->6,2->3,4->10,4->11,4->9,5->1,7->12,7->2,9->5,9->8] | 1.00 | 602.49122807 | 22 | 57 | 0 | 1 |
| Path 3 | C00031->C00251:[1->6,2->3,4->10,4->11,4->9,5->1,7->12,7->2,9->5,9->8] | 1.00 | 570.578125 | 25 | 64 | 0 | 0 |
| Path 4 | C00031->C00251:[1->6,2->3,4->10,4->11,4->9,5->1,7->12,7->2,9->5,9->8] | 1.00 | 552.691176471 | 25 | 68 | 0 | 0 |
| Path 5 | C00031->C00251:[1->5,1->6,1->8,2->10,2->11,2->3,4->12,4->2,4->9,5->1] | 1.00 | 570.085106383 | 23 | 47 | 0 | 0 |
| Path 6 | C00031->C00251:[1->6,2->3,4->10,4->11,4->9,5->1,7->12,7->2,9->5,9->8] | 1.00 | 544.106060606 | 24 | 66 | 0 | 0 |
| Path 7 | C00031->C00251:[1->6,2->3,4->10,4->11,4->9,5->1,7->12,7->2,9->5,9->8] | 1.00 | 601.129032258 | 25 | 62 | 0 | 0 |
| Path 8 | C00031->C00251:[1->6,2->3,4->10,4->11,4->9,5->1,7->12,7->2,9->5,9->8] | 1.00 | 572.438356164 | 30 | 73 | 0 | 0 |
| Path 9 | C00031->C00251:[1->5,1->6,1->8,2->10,2->11,2->3,4->12,4->2,4->9,5->1] | 1.00 | 599.622222222 | 23 | 45 | 0 | 0 |
| Path 10 | C00031->C00251:[1->5,1->6,1->8,2->10,2->11,2->3,4->12,4->2,4->9,5->1] | 1.00 | 627.58974359 | 18 | 39 | 0 | 0 |
