# Supplementary material for: 13C-metabolic flux ratio and novel carbon path analyses confirmed that Trichoderma reesei uses primarily the respirative pathway also on the preferred carbon source glucose
Source: BMC Syst Biol. 2009 Oct 29;3:104. doi: 10.1186/1752-0509-3-104 (PMC2776023; doi:10.1186/1752-0509-3-104)
Supplement: Additional file 1 — Pathways discovered in ReTrace carbon path analysis. Graphical and tabular representations of amino acid synthesis pathways discovered in ReTrace carbon path analysis [21]. Self-contained web site: unpack zip archive and open index.html with a web browser. [file 1752-0509-3-104-S1.zip › AF1-treesei/pathways-C00031-to-C00123.html]

Pathways from C00031 to C00123


**Pathways from C00031 to C00123**

**Sources:** D-Glucose; (C00031)

**Target:**L-Leucine; (C00123)

|  | Composite mapping | Z | Average score | Rpairs | Reactions | Zero scores | Scores under threshold |
| --- | --- | --- | --- | --- | --- | --- | --- |
| Path 1 | C00031->C00123:[7->2,7->9,9->1,9->3,9->5] | 0.83 | 284.6 | 25 | 65 | 0 | 1 |
| Path 2 | C00031->C00123:[7->3,7->8,7->9,9->5] | 0.67 | 251.48 | 19 | 50 | 0 | 1 |
| Path 3 | C00031->C00123:[4->3,7->2,7->8,9->1] | 0.67 | 304.887096774 | 24 | 62 | 0 | 1 |
| Path 4 | C00031->C00123:[4->1,4->3,7->2,7->8,9->1] | 0.67 | 350.945205479 | 29 | 73 | 0 | 1 |
| Path 5 | C00031->C00123:[4->1,4->3,4->5,7->2,7->9,9->1,9->5] | 0.83 | 347.898734177 | 34 | 79 | 0 | 1 |
| Path 6 | C00031->C00123:[4->1,4->3,7->2,7->8,9->1] | 0.67 | 326.582089552 | 29 | 67 | 0 | 1 |
| Path 7 | C00031->C00123:[4->3,7->8,7->9,9->5] | 0.67 | 291.385964912 | 23 | 57 | 0 | 1 |
| Path 8 | C00031->C00123:[4->3,7->8,7->9,9->5] | 0.67 | 269.592592593 | 20 | 54 | 0 | 1 |
| Path 9 | C00031->C00123:[4->3,7->8,7->9,9->5] | 0.67 | 334.119402985 | 25 | 67 | 0 | 1 |
| Path 10 | C00031->C00123:[4->3,4->5,7->8,7->9,9->5] | 0.67 | 339.90625 | 28 | 64 | 0 | 1 |
| Path 11 | C00031->C00123:[4->1,4->3,7->1,7->2,7->3,7->8,9->1] | 0.67 | 310.096774194 | 26 | 62 | 0 | 1 |
| Path 12 | C00031->C00123:[4->1,4->3,7->2,7->8,9->1] | 0.67 | 339.465753425 | 32 | 73 | 0 | 1 |
| Path 13 | C00031->C00123:[1->1,4->2,4->3,4->8] | 0.67 | 435.257575758 | 24 | 66 | 0 | 1 |
| Path 14 | C00031->C00123:[4->3,7->8,7->9,9->5] | 0.67 | 266.867924528 | 19 | 53 | 0 | 1 |
| Path 15 | C00031->C00123:[4->3,4->5,7->3,7->5,7->8,7->9,9->5] | 0.67 | 310.096774194 | 26 | 62 | 0 | 1 |
| Path 16 | C00031->C00123:[7->2,7->3,7->9,9->1,9->5] | 0.83 | 271.553571429 | 21 | 56 | 0 | 1 |
| Path 17 | C00031->C00123:[4->3,7->2,7->8,9->1] | 0.67 | 334.119402985 | 25 | 67 | 0 | 1 |
| Path 18 | C00031->C00123:[4->1,4->3,7->2,7->8,9->1] | 0.67 | 325.046153846 | 27 | 65 | 0 | 1 |
| Path 19 | C00031->C00123:[2->3,7->2,7->9,9->1,9->5] | 0.83 | 376.214285714 | 29 | 98 | 0 | 1 |
| Path 20 | C00031->C00123:[4->3,7->2,7->8,9->1] | 0.67 | 351.11827957 | 28 | 93 | 0 | 1 |
| Path 21 | C00031->C00123:[4->3,7->8,7->9,9->5] | 0.67 | 280.285714286 | 20 | 56 | 0 | 1 |
| Path 22 | C00031->C00123:[4->3,7->2,7->9,9->1,9->5] | 0.83 | 351.11827957 | 28 | 93 | 0 | 1 |
| Path 23 | C00031->C00123:[4->1,4->3,4->5,7->2,7->9,9->1,9->5] | 0.83 | 330.064935065 | 32 | 77 | 0 | 1 |
| Path 24 | C00031->C00123:[4->1,4->3,4->5,7->2,7->9,9->1,9->5] | 0.83 | 297.161764706 | 26 | 68 | 0 | 1 |
| Path 25 | C00031->C00123:[4->1,4->3,4->5,7->1,7->2,7->3,7->5,7->9,9->1,9->5] | 0.83 | 308.128571429 | 28 | 70 | 0 | 1 |
| Path 26 | C00031->C00123:[4->3,7->2,7->3,7->9,9->1,9->5] | 0.83 | 333.597938144 | 30 | 97 | 0 | 1 |
| Path 27 | C00031->C00123:[4->1,4->3,7->2,7->8,9->1] | 0.67 | 294.525423729 | 23 | 59 | 0 | 1 |
| Path 28 | C00031->C00123:[4->3,7->8,7->9,9->5] | 0.67 | 312.129032258 | 25 | 62 | 0 | 1 |
| Path 29 | C00031->C00123:[4->3,7->2,7->9,9->1,9->5] | 0.83 | 304.887096774 | 24 | 62 | 0 | 1 |
| Path 30 | C00031->C00123:[7->2,7->3,7->9,9->1,9->5] | 0.83 | 344.606741573 | 27 | 89 | 0 | 1 |
| Path 31 | C00031->C00123:[7->2,7->9,9->1,9->3,9->5] | 0.83 | 235.366666667 | 20 | 60 | 0 | 1 |
| Path 32 | C00031->C00123:[2->3,7->2,7->9,9->1,9->5] | 0.83 | 340.132653061 | 25 | 98 | 0 | 1 |
| Path 33 | C00031->C00123:[4->3,7->8,7->9,9->5] | 0.67 | 319.327586207 | 24 | 58 | 0 | 1 |
| Path 34 | C00031->C00123:[7->2,7->9,9->1,9->3,9->5] | 0.83 | 316.707692308 | 26 | 65 | 0 | 1 |
| Path 35 | C00031->C00123:[4->3,7->8,7->9,9->5] | 0.67 | 313.746031746 | 26 | 63 | 0 | 1 |
| Path 36 | C00031->C00123:[7->8,7->9,9->3,9->5] | 0.67 | 251.653061224 | 17 | 49 | 0 | 1 |
| Path 37 | C00031->C00123:[1->1,4->2,4->3,4->8,5->2,5->8,9->1] | 0.67 | 371.819148936 | 31 | 94 | 0 | 1 |
| Path 38 | C00031->C00123:[4->3,7->2,7->8,9->1] | 0.67 | 286.516666667 | 22 | 60 | 0 | 1 |
| Path 39 | C00031->C00123:[7->2,7->9,9->1,9->3,9->5] | 0.83 | 346.528089888 | 26 | 89 | 0 | 1 |
| Path 40 | C00031->C00123:[7->2,7->3,7->8,9->1] | 0.67 | 323.841269841 | 24 | 63 | 0 | 1 |
| Path 41 | C00031->C00123:[7->2,7->9,9->1,9->3,9->5] | 0.83 | 344.444444444 | 27 | 90 | 0 | 1 |
| Path 42 | C00031->C00123:[7->2,7->3,7->8,9->1] | 0.67 | 248.163265306 | 18 | 49 | 0 | 1 |
| Path 43 | C00031->C00123:[4->3,4->5,7->8,7->9,9->5] | 0.67 | 312.93442623 | 25 | 61 | 0 | 1 |
| Path 44 | C00031->C00123:[4->3,7->2,7->8,9->1] | 0.67 | 367.197802198 | 28 | 91 | 0 | 1 |
| Path 45 | C00031->C00123:[7->2,7->9,9->1,9->3,9->5] | 0.83 | 243.0 | 18 | 54 | 0 | 1 |
| Path 46 | C00031->C00123:[4->3,7->2,7->9,9->1,9->5] | 0.83 | 332.132653061 | 29 | 98 | 0 | 1 |
| Path 47 | C00031->C00123:[7->2,7->3,7->8,9->1] | 0.67 | 282.666666667 | 20 | 51 | 0 | 1 |
| Path 48 | C00031->C00123:[4->3,7->2,7->9,9->1,9->5] | 0.83 | 331.464646465 | 29 | 99 | 0 | 1 |
| Path 49 | C00031->C00123:[4->1,4->3,4->5,7->2,7->9,9->1,9->5] | 0.83 | 320.219178082 | 30 | 73 | 0 | 1 |
| Path 50 | C00031->C00123:[4->3,7->2,7->8,9->1] | 0.67 | 445.083333333 | 28 | 72 | 0 | 1 |
| Path 51 | C00031->C00123:[7->1,7->2,7->3,7->5,7->9,9->1,9->5] | 0.83 | 310.959459459 | 30 | 74 | 0 | 1 |
| Path 52 | C00031->C00123:[4->1,4->3,7->2,7->8,9->1] | 0.67 | 368.168674699 | 34 | 83 | 0 | 1 |
| Path 53 | C00031->C00123:[7->2,7->9,9->1,9->3,9->5] | 0.83 | 264.892857143 | 20 | 56 | 0 | 1 |
| Path 54 | C00031->C00123:[4->1,4->3,7->2,7->8,9->1] | 0.67 | 448.506849315 | 29 | 73 | 0 | 1 |
| Path 55 | C00031->C00123:[4->3,7->8,7->9,9->5] | 0.67 | 298.181818182 | 21 | 55 | 0 | 1 |
| Path 56 | C00031->C00123:[4->3,4->5,7->8,7->9,9->5] | 0.67 | 387.454545455 | 29 | 88 | 0 | 1 |
| Path 57 | C00031->C00123:[4->1,4->3,4->5,7->2,7->9,9->1,9->5] | 0.83 | 326.739130435 | 29 | 69 | 0 | 1 |
| Path 58 | C00031->C00123:[7->2,7->9,9->1,9->3,9->5] | 0.83 | 300.953125 | 25 | 64 | 0 | 1 |
| Path 59 | C00031->C00123:[7->2,7->3,7->9,9->1,9->5] | 0.83 | 232.516666667 | 21 | 60 | 0 | 1 |
| Path 60 | C00031->C00123:[4->1,4->3,7->2,7->8,9->1] | 0.67 | 417.0 | 26 | 47 | 0 | 1 |
| Path 61 | C00031->C00123:[4->3,7->2,7->8,9->1] | 0.67 | 302.683333333 | 24 | 60 | 0 | 1 |
| Path 62 | C00031->C00123:[7->2,7->9,9->1,9->3,9->5] | 0.83 | 296.593220339 | 23 | 59 | 0 | 1 |
| Path 63 | C00031->C00123:[4->3,7->2,7->8,9->1] | 0.67 | 266.867924528 | 19 | 53 | 0 | 1 |
| Path 64 | C00031->C00123:[2->3,7->2,7->9,9->1,9->5] | 0.83 | 375.87628866 | 28 | 97 | 0 | 1 |
| Path 65 | C00031->C00123:[4->3,7->2,7->8,9->1] | 0.67 | 256.677966102 | 21 | 59 | 0 | 1 |
| Path 66 | C00031->C00123:[4->3,7->2,7->9,9->1,9->5] | 0.83 | 270.278688525 | 21 | 61 | 0 | 1 |
| Path 67 | C00031->C00123:[4->3,7->2,7->9,9->1,9->5] | 0.83 | 324.574468085 | 27 | 94 | 0 | 1 |
| Path 68 | C00031->C00123:[4->1,4->3,7->2,7->8,9->1] | 0.67 | 453.975903614 | 34 | 83 | 0 | 1 |
| Path 69 | C00031->C00123:[4->3,7->2,7->9,9->1,9->5] | 0.83 | 294.328358209 | 25 | 67 | 0 | 1 |
| Path 70 | C00031->C00123:[4->3,5->1,9->2,9->8] | 0.67 | 368.648351648 | 30 | 91 | 0 | 1 |
| Path 71 | C00031->C00123:[4->1,4->3,4->5,7->2,7->9,9->1,9->5] | 0.83 | 298.735294118 | 25 | 68 | 0 | 1 |
| Path 72 | C00031->C00123:[4->3,7->2,7->8,9->1] | 0.67 | 368.471910112 | 28 | 89 | 0 | 1 |
| Path 73 | C00031->C00123:[4->1,4->3,7->2,7->8,9->1] | 0.67 | 332.132352941 | 29 | 68 | 0 | 1 |
| Path 74 | C00031->C00123:[4->3,7->2,7->8,9->1] | 0.67 | 319.327586207 | 24 | 58 | 0 | 1 |
| Path 75 | C00031->C00123:[4->1,4->3,7->2,7->8,9->1] | 0.67 | 339.90625 | 28 | 64 | 0 | 1 |
| Path 76 | C00031->C00123:[7->2,7->9,9->1,9->3,9->5] | 0.83 | 323.852459016 | 25 | 61 | 0 | 1 |
| Path 77 | C00031->C00123:[7->2,7->9,9->1,9->3,9->5] | 0.83 | 280.144927536 | 26 | 69 | 0 | 1 |
| Path 78 | C00031->C00123:[4->3,7->2,7->9,9->1,9->5] | 0.83 | 261.0 | 23 | 67 | 0 | 1 |
| Path 79 | C00031->C00123:[7->2,7->3,7->9,9->1,9->5] | 0.83 | 288.238095238 | 25 | 63 | 0 | 1 |
| Path 80 | C00031->C00123:[4->3,7->2,7->8,9->1] | 0.67 | 257.5 | 20 | 58 | 0 | 1 |
| Path 81 | C00031->C00123:[7->2,7->9,9->1,9->3,9->5] | 0.83 | 282.65625 | 24 | 64 | 0 | 1 |
| Path 82 | C00031->C00123:[4->1,4->3,4->5,7->2,7->9,9->1,9->5] | 0.83 | 333.844155844 | 32 | 77 | 0 | 1 |
| Path 83 | C00031->C00123:[7->2,7->9,9->1,9->3,9->5] | 0.83 | 307.166666667 | 24 | 60 | 0 | 1 |
| Path 84 | C00031->C00123:[4->3,7->8,7->9,9->5] | 0.67 | 288.290909091 | 21 | 55 | 0 | 1 |
| Path 85 | C00031->C00123:[2->3,7->2,7->9,9->1,9->5] | 0.83 | 365.768421053 | 26 | 95 | 0 | 1 |
| Path 86 | C00031->C00123:[4->3,7->2,7->9,9->1,9->5] | 0.83 | 267.378787879 | 24 | 66 | 0 | 1 |
| Path 87 | C00031->C00123:[7->2,7->3,7->8,9->1] | 0.67 | 275.943396226 | 22 | 53 | 0 | 1 |
| Path 88 | C00031->C00123:[4->3,7->2,7->9,9->1,9->5] | 0.83 | 278.246376812 | 25 | 69 | 0 | 1 |
| Path 89 | C00031->C00123:[4->1,4->3,7->2,7->8,9->1] | 0.67 | 323.420289855 | 28 | 69 | 0 | 1 |
| Path 90 | C00031->C00123:[7->2,7->3,7->9,9->1,9->5] | 0.83 | 283.431034483 | 23 | 58 | 0 | 1 |
| Path 91 | C00031->C00123:[4->3,7->2,7->8,9->1] | 0.67 | 298.181818182 | 21 | 55 | 0 | 1 |
| Path 92 | C00031->C00123:[4->1,4->3,4->5,7->2,7->9,9->1,9->5] | 0.83 | 312.256756757 | 29 | 74 | 0 | 1 |
| Path 93 | C00031->C00123:[7->2,7->3,7->9,9->1,9->5] | 0.83 | 291.706896552 | 23 | 58 | 0 | 1 |
| Path 94 | C00031->C00123:[7->8,7->9,9->3,9->5] | 0.67 | 254.9 | 18 | 50 | 0 | 1 |
| Path 95 | C00031->C00123:[7->2,7->9,9->1,9->3,9->5] | 0.83 | 290.952380952 | 24 | 63 | 0 | 1 |
| Path 96 | C00031->C00123:[4->3,7->2,7->8,9->1] | 0.67 | 309.517241379 | 22 | 58 | 0 | 1 |
| Path 97 | C00031->C00123:[4->1,4->3,7->2,7->8,9->1] | 0.67 | 424.509433962 | 29 | 53 | 0 | 1 |
| Path 98 | C00031->C00123:[4->3,7->2,7->9,9->1,9->5] | 0.83 | 320.246753247 | 30 | 77 | 0 | 1 |
| Path 99 | C00031->C00123:[4->3,7->2,7->8,9->1] | 0.67 | 277.45 | 22 | 60 | 0 | 1 |
| Path 100 | C00031->C00123:[4->1,4->3,4->5,7->1,7->2,7->3,7->5,7->9,9->1,9->5] | 0.83 | 298.76119403 | 27 | 67 | 0 | 1 |
| Path 101 | C00031->C00123:[4->3,7->2,7->9,9->1,9->5] | 0.83 | 286.516666667 | 22 | 60 | 0 | 1 |
| Path 102 | C00031->C00123:[7->2,7->9,9->1,9->3,9->5] | 0.83 | 255.387096774 | 22 | 62 | 0 | 1 |
| Path 103 | C00031->C00123:[4->3,7->8,7->9,9->5] | 0.67 | 259.3 | 22 | 60 | 0 | 1 |
| Path 104 | C00031->C00123:[2->3,7->2,7->9,9->1,9->5] | 0.83 | 349.376344086 | 24 | 93 | 0 | 1 |
| Path 105 | C00031->C00123:[7->2,7->3,7->9,9->1,9->5] | 0.83 | 261.839285714 | 21 | 56 | 0 | 1 |
| Path 106 | C00031->C00123:[4->3,7->2,7->9,9->1,9->5] | 0.83 | 248.984375 | 22 | 64 | 0 | 1 |
| Path 107 | C00031->C00123:[4->3,7->2,7->8,9->1] | 0.67 | 300.137931034 | 22 | 58 | 0 | 1 |
| Path 108 | C00031->C00123:[4->3,7->2,7->8,9->1] | 0.67 | 248.984375 | 22 | 64 | 0 | 1 |
| Path 109 | C00031->C00123:[7->2,7->3,7->9,9->1,9->5] | 0.83 | 252.629032258 | 23 | 62 | 0 | 1 |
| Path 110 | C00031->C00123:[4->3,7->2,7->8,9->1] | 0.67 | 288.290909091 | 21 | 55 | 0 | 1 |
| Path 111 | C00031->C00123:[7->2,7->8,9->1,9->3] | 0.67 | 251.653061224 | 17 | 49 | 0 | 1 |
| Path 112 | C00031->C00123:[4->1,4->3,4->5,7->2,7->9,9->1,9->5] | 0.83 | 310.458333333 | 27 | 72 | 0 | 1 |
| Path 113 | C00031->C00123:[2->3,7->2,7->9,9->1,9->5] | 0.83 | 360.042105263 | 26 | 95 | 0 | 1 |
| Path 114 | C00031->C00123:[4->1,4->3,4->5,7->2,7->9,9->1,9->5] | 0.83 | 336.767123288 | 31 | 73 | 0 | 1 |
| Path 115 | C00031->C00123:[7->2,7->9,9->1,9->3,9->5] | 0.83 | 294.655172414 | 22 | 58 | 0 | 1 |
| Path 116 | C00031->C00123:[4->3,7->2,7->8,9->1] | 0.67 | 341.971428571 | 26 | 70 | 0 | 1 |
| Path 117 | C00031->C00123:[4->3,7->2,7->9,9->1,9->5] | 0.83 | 257.5 | 20 | 58 | 0 | 1 |
| Path 118 | C00031->C00123:[7->2,7->9,9->1,9->3,9->5] | 0.83 | 357.725274725 | 28 | 91 | 0 | 1 |
| Path 119 | C00031->C00123:[4->1,4->3,7->2,7->8,9->1] | 0.67 | 314.952380952 | 27 | 63 | 0 | 1 |
| Path 120 | C00031->C00123:[4->3,7->2,7->8,9->1] | 0.67 | 362.079545455 | 27 | 88 | 0 | 1 |
| Path 121 | C00031->C00123:[7->2,7->9,9->1,9->3,9->5] | 0.83 | 363.703296703 | 28 | 91 | 0 | 1 |
| Path 122 | C00031->C00123:[4->3,7->8,7->9,9->5] | 0.67 | 362.079545455 | 27 | 88 | 0 | 1 |
| Path 123 | C00031->C00123:[4->3,7->2,7->8,9->1] | 0.67 | 280.285714286 | 20 | 56 | 0 | 1 |
| Path 124 | C00031->C00123:[4->3,7->8,7->9,9->5] | 0.67 | 256.677966102 | 21 | 59 | 0 | 1 |
| Path 125 | C00031->C00123:[4->3,7->2,7->9,9->1,9->5] | 0.83 | 297.507936508 | 23 | 63 | 0 | 1 |
| Path 126 | C00031->C00123:[2->3,7->2,7->9,9->1,9->5] | 0.83 | 338.303030303 | 26 | 99 | 0 | 1 |
| Path 127 | C00031->C00123:[4->1,4->3,4->5,7->2,7->9,9->1,9->5] | 0.83 | 283.875 | 24 | 64 | 0 | 1 |
| Path 128 | C00031->C00123:[4->3,7->2,7->9,9->1,9->5] | 0.83 | 316.121212121 | 26 | 66 | 0 | 1 |
| Path 129 | C00031->C00123:[2->3,7->2,7->9,9->1,9->5] | 0.83 | 369.607843137 | 30 | 102 | 0 | 1 |
| Path 130 | C00031->C00123:[4->1,4->3,7->2,7->8,9->1] | 0.67 | 451.688311688 | 31 | 77 | 0 | 1 |
| Path 131 | C00031->C00123:[4->3,7->2,7->9,9->1,9->5] | 0.83 | 356.3125 | 29 | 96 | 0 | 1 |
| Path 132 | C00031->C00123:[7->2,7->3,7->8,9->1] | 0.67 | 272.0 | 20 | 51 | 0 | 1 |
| Path 133 | C00031->C00123:[4->3,7->2,7->8,9->1] | 0.67 | 306.53968254 | 25 | 63 | 0 | 1 |
| Path 134 | C00031->C00123:[4->1,4->3,7->2,7->8,9->1] | 0.67 | 308.19047619 | 25 | 63 | 0 | 1 |
| Path 135 | C00031->C00123:[4->3,7->2,7->9,9->1,9->5] | 0.83 | 306.53968254 | 25 | 63 | 0 | 1 |
| Path 136 | C00031->C00123:[4->3,7->2,7->9,9->1,9->5] | 0.83 | 306.857142857 | 32 | 98 | 0 | 1 |
| Path 137 | C00031->C00123:[4->3,4->5,7->8,7->9,9->5] | 0.67 | 294.525423729 | 23 | 59 | 0 | 1 |
| Path 138 | C00031->C00123:[2->3,7->2,7->9,9->1,9->5] | 0.83 | 348.554455446 | 28 | 101 | 0 | 1 |
| Path 139 | C00031->C00123:[7->2,7->9,9->1,9->3,9->5] | 0.83 | 264.161290323 | 22 | 62 | 0 | 1 |
| Path 140 | C00031->C00123:[7->2,7->3,7->8,9->1] | 0.67 | 355.702380952 | 26 | 84 | 0 | 1 |
| Path 141 | C00031->C00123:[4->3,4->5,7->8,7->9,9->5] | 0.67 | 332.132352941 | 29 | 68 | 0 | 1 |
| Path 142 | C00031->C00123:[4->3,7->2,7->9,9->1,9->5] | 0.83 | 335.645833333 | 29 | 96 | 0 | 1 |
| Path 143 | C00031->C00123:[4->3,7->2,7->9,9->1,9->5] | 0.83 | 300.641791045 | 26 | 67 | 0 | 1 |
| Path 144 | C00031->C00123:[7->2,7->9,9->1,9->3,9->5] | 0.83 | 274.607142857 | 20 | 56 | 0 | 1 |
| Path 145 | C00031->C00123:[4->1,4->3,4->5,7->2,7->9,9->1,9->5] | 0.83 | 313.128571429 | 28 | 70 | 0 | 1 |
| Path 146 | C00031->C00123:[4->3,4->5,7->8,7->9,9->5] | 0.67 | 308.19047619 | 25 | 63 | 0 | 1 |
| Path 147 | C00031->C00123:[4->3,7->8,7->9,9->5] | 0.67 | 362.662921348 | 28 | 89 | 0 | 1 |
| Path 148 | C00031->C00123:[4->3,7->2,7->9,9->1,9->5] | 0.83 | 277.45 | 22 | 60 | 0 | 1 |
| Path 149 | C00031->C00123:[4->1,4->3,7->2,7->8,9->1] | 0.67 | 408.372093023 | 24 | 43 | 0 | 1 |
| Path 150 | C00031->C00123:[4->3,7->2,7->8,9->1] | 0.67 | 300.641791045 | 26 | 67 | 0 | 1 |
| Path 151 | C00031->C00123:[4->3,7->2,7->9,9->1,9->5] | 0.83 | 288.873015873 | 23 | 63 | 0 | 1 |
| Path 152 | C00031->C00123:[4->3,7->8,7->9,9->5] | 0.67 | 320.93220339 | 25 | 59 | 0 | 1 |
| Path 153 | C00031->C00123:[4->1,4->3,7->2,7->8,9->1] | 0.67 | 338.422535211 | 30 | 71 | 0 | 1 |
| Path 154 | C00031->C00123:[4->1,4->3,7->2,7->8,9->1] | 0.67 | 387.454545455 | 29 | 88 | 0 | 1 |
| Path 155 | C00031->C00123:[4->3,7->8,7->9,9->5] | 0.67 | 290.535714286 | 22 | 56 | 0 | 1 |
| Path 156 | C00031->C00123:[4->3,7->2,7->9,9->1,9->5] | 0.83 | 309.928571429 | 27 | 70 | 0 | 1 |
| Path 157 | C00031->C00123:[4->3,7->2,7->8,9->1] | 0.67 | 291.385964912 | 23 | 57 | 0 | 1 |
| Path 158 | C00031->C00123:[4->3,7->8,7->9,9->5] | 0.67 | 282.631578947 | 21 | 57 | 0 | 1 |
| Path 159 | C00031->C00123:[4->1,4->3,4->5,7->2,7->9,9->1,9->5] | 0.83 | 301.212121212 | 26 | 66 | 0 | 1 |
| Path 160 | C00031->C00123:[4->3,7->2,7->8,9->1] | 0.67 | 312.129032258 | 25 | 62 | 0 | 1 |
| Path 161 | C00031->C00123:[4->1,4->3,7->2,7->8,9->1] | 0.67 | 312.93442623 | 25 | 61 | 0 | 1 |
| Path 162 | C00031->C00123:[4->3,7->2,7->8,9->1] | 0.67 | 267.378787879 | 24 | 66 | 0 | 1 |
| Path 163 | C00031->C00123:[4->3,7->2,7->9,9->1,9->5] | 0.83 | 314.692307692 | 25 | 65 | 0 | 1 |
| Path 164 | C00031->C00123:[4->1,4->3,4->5,7->2,7->9,9->1,9->5] | 0.83 | 323.756410256 | 30 | 78 | 0 | 1 |
| Path 165 | C00031->C00123:[7->3,7->8,7->9,9->5] | 0.67 | 248.163265306 | 18 | 49 | 0 | 1 |
| Path 166 | C00031->C00123:[7->2,7->3,7->9,9->1,9->5] | 0.83 | 239.833333333 | 19 | 54 | 0 | 1 |
| Path 167 | C00031->C00123:[4->1,4->3,4->5,7->2,7->9,9->1,9->5] | 0.83 | 326.565789474 | 31 | 76 | 0 | 1 |
| Path 168 | C00031->C00123:[4->1,4->3,7->2,7->8,9->1] | 0.67 | 359.194805195 | 31 | 77 | 0 | 1 |
| Path 169 | C00031->C00123:[7->2,7->8,9->1,9->3] | 0.67 | 311.666666667 | 28 | 78 | 0 | 2 |
| Path 170 | C00031->C00123:[4->1,4->3,7->2] | 0.50 | 442.048192771 | 31 | 83 | 0 | 2 |
| Path 171 | C00031->C00123:[4->1,4->3,7->1,7->3] | 0.33 | 390.05 | 21 | 40 | 0 | 1 |
| Path 172 | C00031->C00123:[2->3,7->2,7->8,7->9,9->1,9->5] | 1.00 | 306.888888889 | 29 | 90 | 0 | 2 |
| Path 173 | C00031->C00123:[4->1,4->3,7->2] | 0.50 | 437.325842697 | 33 | 89 | 0 | 2 |
| Path 174 | C00031->C00123:[7->8,9->3] | 0.33 | 238.887096774 | 20 | 62 | 0 | 2 |
| Path 175 | C00031->C00123:[4->3,7->2,7->9,9->1,9->5] | 0.83 | 371.607142857 | 35 | 112 | 0 | 2 |
| Path 176 | C00031->C00123:[4->3,7->2,7->8,9->1] | 0.67 | 416.224489796 | 26 | 49 | 0 | 1 |
| Path 177 | C00031->C00123:[4->3,7->8] | 0.33 | 380.0 | 21 | 41 | 0 | 2 |
| Path 178 | C00031->C00123:[7->2,9->1,9->3] | 0.50 | 222.464285714 | 19 | 56 | 0 | 2 |
| Path 179 | C00031->C00123:[4->1,4->3,7->2] | 0.50 | 382.965517241 | 27 | 58 | 0 | 2 |
| Path 180 | C00031->C00123:[2->3,7->2,7->8,9->1] | 0.67 | 352.694117647 | 23 | 85 | 0 | 2 |
| Path 181 | C00031->C00123:[4->1,4->3] | 0.33 | 343.446808511 | 20 | 47 | 0 | 1 |
| Path 182 | C00031->C00123:[5->3,7->2,7->8,9->1] | 0.67 | 365.510204082 | 30 | 98 | 0 | 2 |
| Path 183 | C00031->C00123:[4->1,4->3,7->1,7->2,7->3] | 0.50 | 390.0 | 25 | 45 | 0 | 2 |
| Path 184 | C00031->C00123:[7->8,7->9,9->5] | 0.50 | 346.049382716 | 23 | 81 | 0 | 1 |
| Path 185 | C00031->C00123:[4->1,4->3,7->2,7->8] | 0.67 | 395.315789474 | 21 | 38 | 0 | 1 |
| Path 186 | C00031->C00123:[4->3,7->8] | 0.33 | 268.654545455 | 20 | 55 | 0 | 2 |
| Path 187 | C00031->C00123:[7->2,7->3,7->9,9->1,9->5] | 0.83 | 233.428571429 | 20 | 56 | 0 | 2 |
| Path 188 | C00031->C00123:[2->3,7->8] | 0.33 | 348.288888889 | 27 | 90 | 0 | 2 |
| Path 189 | C00031->C00123:[5->3,7->2,7->8,7->9,9->1,9->5] | 1.00 | 278.376811594 | 24 | 69 | 0 | 2 |
| Path 190 | C00031->C00123:[4->1,4->2,4->3] | 0.50 | 399.519480519 | 30 | 77 | 0 | 2 |
| Path 191 | C00031->C00123:[7->2,7->3,7->8,9->1] | 0.67 | 264.555555556 | 22 | 54 | 0 | 2 |
| Path 192 | C00031->C00123:[2->3,5->8] | 0.33 | 340.808510638 | 30 | 94 | 0 | 2 |
| Path 193 | C00031->C00123:[5->3,7->8,7->9,9->5] | 0.67 | 276.294117647 | 23 | 68 | 0 | 2 |
| Path 194 | C00031->C00123:[4->1,4->3] | 0.33 | 406.454545455 | 19 | 33 | 0 | 1 |
| Path 195 | C00031->C00123:[1->1,4->2,4->3] | 0.50 | 426.661538462 | 23 | 65 | 0 | 1 |
| Path 196 | C00031->C00123:[7->8,9->3] | 0.33 | 264.378947368 | 22 | 95 | 0 | 2 |
| Path 197 | C00031->C00123:[2->3,7->2,7->8,9->1] | 0.67 | 352.912087912 | 25 | 91 | 0 | 2 |
| Path 198 | C00031->C00123:[7->2,7->3,7->8,7->9,9->1,9->5] | 1.00 | 248.483333333 | 24 | 60 | 0 | 2 |
| Path 199 | C00031->C00123:[5->3,7->2,7->8,9->1] | 0.67 | 276.294117647 | 23 | 68 | 0 | 2 |
| Path 200 | C00031->C00123:[7->8,7->9,9->5] | 0.50 | 284.517857143 | 22 | 56 | 0 | 1 |
| Path 201 | C00031->C00123:[4->1,4->3,7->2] | 0.50 | 340.534482759 | 24 | 58 | 0 | 1 |
| Path 202 | C00031->C00123:[4->1,4->3,5->2,5->8] | 0.67 | 354.782051282 | 30 | 78 | 0 | 1 |
| Path 203 | C00031->C00123:[7->2,7->8,9->1,9->3] | 0.67 | 298.506493506 | 27 | 77 | 0 | 2 |
| Path 204 | C00031->C00123:[7->2,9->1,9->3] | 0.50 | 389.176470588 | 19 | 34 | 0 | 1 |
| Path 205 | C00031->C00123:[2->3,7->2,7->8,7->9,9->1,9->5] | 1.00 | 308.968085106 | 29 | 94 | 0 | 2 |
| Path 206 | C00031->C00123:[7->2,7->8,9->1,9->3] | 0.67 | 361.528089888 | 29 | 89 | 0 | 2 |
| Path 207 | C00031->C00123:[7->2,7->8,9->1,9->3] | 0.67 | 266.237288136 | 22 | 59 | 0 | 2 |
| Path 208 | C00031->C00123:[7->2,7->9,9->1,9->3,9->5] | 0.83 | 340.241758242 | 27 | 91 | 0 | 2 |
| Path 209 | C00031->C00123:[4->3,7->2,7->8,9->1] | 0.67 | 292.15 | 23 | 60 | 0 | 2 |
| Path 210 | C00031->C00123:[7->2,7->8,9->1,9->3] | 0.67 | 245.096153846 | 19 | 52 | 0 | 2 |
| Path 211 | C00031->C00123:[7->2,9->1,9->3] | 0.50 | 253.269230769 | 19 | 52 | 0 | 2 |
| Path 212 | C00031->C00123:[4->3,5->8] | 0.33 | 318.0 | 24 | 67 | 0 | 2 |
| Path 213 | C00031->C00123:[4->1,4->3] | 0.33 | 327.976744186 | 18 | 43 | 0 | 1 |
| Path 214 | C00031->C00123:[2->3,7->2,7->8,7->9,9->1,9->5] | 1.00 | 303.549450549 | 31 | 91 | 0 | 2 |
| Path 215 | C00031->C00123:[2->3,7->2,7->8,9->1] | 0.67 | 367.948453608 | 29 | 97 | 0 | 2 |
| Path 216 | C00031->C00123:[9->3] | 0.17 | 216.386363636 | 12 | 44 | 0 | 2 |
| Path 217 | C00031->C00123:[7->1,7->3] | 0.33 | 387.363636364 | 23 | 44 | 0 | 1 |
| Path 218 | C00031->C00123:[2->3,7->2,7->8,7->9,9->1,9->5] | 1.00 | 305.617977528 | 28 | 89 | 0 | 2 |
| Path 219 | C00031->C00123:[4->1,4->3,4->5,7->2,7->9,9->1,9->5] | 0.83 | 285.857142857 | 23 | 63 | 0 | 1 |
| Path 220 | C00031->C00123:[5->3,7->2,7->8,9->1] | 0.67 | 298.985714286 | 25 | 70 | 0 | 2 |
| Path 221 | C00031->C00123:[4->3,7->2,7->8,7->9,9->1,9->3,9->5] | 1.00 | 373.867256637 | 38 | 113 | 0 | 2 |
| Path 222 | C00031->C00123:[7->2,7->8,9->1] | 0.50 | 288.0 | 20 | 51 | 0 | 1 |
| Path 223 | C00031->C00123:[4->3,7->8,7->9,9->5] | 0.67 | 273.050847458 | 22 | 59 | 0 | 2 |
| Path 224 | C00031->C00123:[4->3,7->2,7->8,7->9,9->1,9->5] | 1.00 | 341.123809524 | 37 | 105 | 0 | 2 |
| Path 225 | C00031->C00123:[4->3,7->2,9->1] | 0.50 | 260.052631579 | 20 | 57 | 0 | 2 |
| Path 226 | C00031->C00123:[4->1,4->3,7->2] | 0.50 | 419.808510638 | 26 | 47 | 0 | 1 |
| Path 227 | C00031->C00123:[7->2,9->1,9->3] | 0.50 | 296.490566038 | 21 | 53 | 0 | 1 |
| Path 228 | C00031->C00123:[2->3,7->2,7->8,9->1] | 0.67 | 317.94047619 | 28 | 84 | 0 | 2 |
| Path 229 | C00031->C00123:[4->8] | 0.17 | 421.965517241 | 19 | 58 | 0 | 1 |
| Path 230 | C00031->C00123:[2->3,7->2,7->8,9->1] | 0.67 | 342.065934066 | 28 | 91 | 0 | 2 |
| Path 231 | C00031->C00123:[5->3,7->2,9->1] | 0.50 | 346.855555556 | 24 | 90 | 0 | 2 |
| Path 232 | C00031->C00123:[7->2,9->1,9->3] | 0.50 | 283.288135593 | 23 | 59 | 0 | 1 |
| Path 233 | C00031->C00123:[2->3,7->2,9->1] | 0.50 | 341.035294118 | 24 | 85 | 0 | 2 |
| Path 234 | C00031->C00123:[7->3] | 0.17 | 253.666666667 | 9 | 21 | 0 | 2 |
| Path 235 | C00031->C00123:[1->1,4->2,4->8] | 0.50 | 421.93220339 | 20 | 59 | 0 | 1 |
| Path 236 | C00031->C00123:[7->2,9->1,9->3] | 0.50 | 326.727272727 | 23 | 55 | 0 | 1 |
| Path 237 | C00031->C00123:[5->3,7->2,7->8,7->9,9->1,9->5] | 1.00 | 276.04 | 26 | 75 | 0 | 2 |
| Path 238 | C00031->C00123:[4->1,4->3,7->2,7->8,9->1] | 0.67 | 334.608695652 | 28 | 69 | 0 | 1 |
| Path 239 | C00031->C00123:[7->2,7->9,9->1,9->5] | 0.67 | 276.410714286 | 21 | 56 | 0 | 1 |
| Path 240 | C00031->C00123:[7->2,7->8,9->1,9->3] | 0.67 | 305.549295775 | 26 | 71 | 0 | 2 |
| Path 241 | C00031->C00123:[4->3,7->2,7->8,7->9,9->1,9->5] | 1.00 | 322.208791209 | 35 | 91 | 0 | 2 |
| Path 242 | C00031->C00123:[4->3,7->2,7->9,9->1,9->3,9->5] | 0.83 | 371.069565217 | 36 | 115 | 0 | 2 |
| Path 243 | C00031->C00123:[4->1,4->3,7->2] | 0.50 | 393.582089552 | 23 | 67 | 0 | 1 |
| Path 244 | C00031->C00123:[7->8,9->3] | 0.33 | 295.757009346 | 26 | 107 | 0 | 2 |
| Path 245 | C00031->C00123:[4->1,4->3,7->2] | 0.50 | 389.551020408 | 26 | 49 | 0 | 2 |
| Path 246 | C00031->C00123:[7->2,7->3,9->1] | 0.50 | 354.65625 | 18 | 32 | 0 | 1 |
| Path 247 | C00031->C00123:[7->2,7->9,9->1,9->3,9->5] | 0.83 | 257.844827586 | 21 | 58 | 0 | 2 |
| Path 248 | C00031->C00123:[4->1,4->3,7->1,7->2,7->3] | 0.50 | 396.925 | 23 | 40 | 0 | 1 |
| Path 249 | C00031->C00123:[4->3,7->2,7->8,7->9,9->1,9->5] | 1.00 | 295.25 | 29 | 72 | 0 | 2 |
| Path 250 | C00031->C00123:[4->3,7->2,7->8,9->1] | 0.67 | 301.081967213 | 24 | 61 | 0 | 2 |
| Path 251 | C00031->C00123:[9->3] | 0.17 | 366.724137931 | 14 | 29 | 0 | 2 |
| Path 252 | C00031->C00123:[2->3,7->8] | 0.33 | 419.150684932 | 27 | 73 | 0 | 2 |
| Path 253 | C00031->C00123:[4->1,4->3,4->5,7->2,7->9,9->1,9->5] | 0.83 | 367.398148148 | 33 | 108 | 0 | 2 |
| Path 254 | C00031->C00123:[7->2,9->1] | 0.33 | 286.870967742 | 15 | 31 | 0 | 1 |
| Path 255 | C00031->C00123:[4->1,4->3,4->5,7->2,7->8,7->9,9->1,9->5] | 1.00 | 370.401785714 | 37 | 112 | 0 | 2 |
| Path 256 | C00031->C00123:[4->3,4->8] | 0.33 | 439.102941176 | 24 | 68 | 0 | 1 |
| Path 257 | C00031->C00123:[7->2,7->8,9->1,9->3] | 0.67 | 298.578947368 | 26 | 76 | 0 | 2 |
| Path 258 | C00031->C00123:[7->3,7->8] | 0.33 | 250.823529412 | 19 | 51 | 0 | 2 |
| Path 259 | C00031->C00123:[2->3,7->8,7->9,9->5] | 0.67 | 352.694117647 | 23 | 85 | 0 | 2 |
| Path 260 | C00031->C00123:[7->2,9->1,9->3] | 0.50 | 329.473684211 | 19 | 38 | 0 | 1 |
| Path 261 | C00031->C00123:[2->3,7->2,9->1] | 0.50 | 376.956043956 | 26 | 91 | 0 | 1 |
| Path 262 | C00031->C00123:[4->3,7->2,7->8,7->9,9->1,9->3,9->5] | 1.00 | 338.754545455 | 41 | 110 | 0 | 2 |
| Path 263 | C00031->C00123:[7->2,9->1,9->3] | 0.50 | 241.236363636 | 19 | 55 | 0 | 1 |
| Path 264 | C00031->C00123:[7->2,9->1,9->3] | 0.50 | 278.492063492 | 24 | 63 | 0 | 1 |
| Path 265 | C00031->C00123:[7->8,7->9,9->5] | 0.50 | 253.816326531 | 18 | 49 | 0 | 1 |
| Path 266 | C00031->C00123:[4->1,4->3] | 0.33 | 415.266666667 | 18 | 30 | 0 | 1 |
| Path 267 | C00031->C00123:[7->2,7->8,9->1] | 0.50 | 346.049382716 | 23 | 81 | 0 | 1 |
| Path 268 | C00031->C00123:[7->2,7->8,9->1] | 0.50 | 261.8125 | 17 | 48 | 0 | 1 |
| Path 269 | C00031->C00123:[4->1,4->2,4->3] | 0.50 | 399.538461538 | 29 | 78 | 0 | 2 |
| Path 270 | C00031->C00123:[4->1,4->3,7->2,9->1] | 0.50 | 502.361702128 | 28 | 47 | 0 | 1 |
| Path 271 | C00031->C00123:[5->3,7->2,7->8,9->1] | 0.67 | 353.304347826 | 26 | 92 | 0 | 2 |
| Path 272 | C00031->C00123:[4->3,4->5] | 0.33 | 433.935483871 | 19 | 31 | 0 | 1 |
| Path 273 | C00031->C00123:[4->3,7->2,7->9,9->1,9->5] | 0.83 | 334.019417476 | 36 | 103 | 0 | 2 |
| Path 274 | C00031->C00123:[7->2,7->9,9->1,9->5] | 0.67 | 274.0 | 20 | 55 | 0 | 1 |
| Path 275 | C00031->C00123:[4->3,7->2,7->9,9->1,9->5] | 0.83 | 338.728971963 | 35 | 107 | 0 | 2 |
| Path 276 | C00031->C00123:[7->2,7->8,7->9,9->1,9->3,9->5] | 1.00 | 255.597222222 | 26 | 72 | 0 | 2 |
| Path 277 | C00031->C00123:[4->3,7->2,7->8,9->1] | 0.67 | 333.589041096 | 28 | 73 | 0 | 2 |
| Path 278 | C00031->C00123:[2->3,7->2,9->1] | 0.50 | 352.954022989 | 22 | 87 | 0 | 1 |
| Path 279 | C00031->C00123:[2->3,7->2,7->8,9->1] | 0.67 | 374.952380952 | 34 | 105 | 0 | 2 |
| Path 280 | C00031->C00123:[9->8] | 0.17 | 241.145833333 | 17 | 48 | 0 | 1 |
| Path 281 | C00031->C00123:[7->8,9->3] | 0.33 | 288.391891892 | 24 | 74 | 0 | 2 |
| Path 282 | C00031->C00123:[4->3,7->2,9->1] | 0.50 | 279.160714286 | 20 | 56 | 0 | 1 |
| Path 283 | C00031->C00123:[4->3,7->2,7->8,7->9,9->1,9->5] | 1.00 | 365.447368421 | 40 | 114 | 0 | 2 |
| Path 284 | C00031->C00123:[2->3,7->2,7->8,9->1] | 0.67 | 369.636363636 | 26 | 88 | 0 | 2 |
| Path 285 | C00031->C00123:[9->3] | 0.17 | 196.074074074 | 13 | 54 | 0 | 2 |
| Path 286 | C00031->C00123:[4->1,4->2,4->3] | 0.50 | 437.962025316 | 27 | 79 | 0 | 2 |
| Path 287 | C00031->C00123:[4->1,4->2,4->3] | 0.50 | 434.363636364 | 29 | 77 | 0 | 2 |
| Path 288 | C00031->C00123:[2->3,5->8] | 0.33 | 324.208791209 | 28 | 91 | 0 | 2 |
| Path 289 | C00031->C00123:[2->3,7->2,9->1] | 0.50 | 304.011764706 | 24 | 85 | 0 | 2 |
| Path 290 | C00031->C00123:[4->1,4->3,7->1,7->2,7->3] | 0.50 | 380.904761905 | 24 | 42 | 0 | 2 |
| Path 291 | C00031->C00123:[2->3,7->8] | 0.33 | 431.086956522 | 25 | 69 | 0 | 2 |
| Path 292 | C00031->C00123:[4->1,4->2,4->3] | 0.50 | 442.042857143 | 28 | 70 | 0 | 2 |
| Path 293 | C00031->C00123:[7->2,9->1,9->3] | 0.50 | 229.16 | 17 | 50 | 0 | 2 |
| Path 294 | C00031->C00123:[5->3,7->2,7->8,9->1] | 0.67 | 276.044776119 | 22 | 67 | 0 | 2 |
| Path 295 | C00031->C00123:[4->3,7->2,7->8,9->1] | 0.67 | 273.050847458 | 22 | 59 | 0 | 2 |
| Path 296 | C00031->C00123:[7->2,7->8,7->9,9->1,9->3,9->5] | 1.00 | 248.396226415 | 20 | 53 | 0 | 2 |
| Path 297 | C00031->C00123:[2->3,7->2,7->9,9->1,9->5] | 0.83 | 341.888888889 | 24 | 90 | 0 | 2 |
| Path 298 | C00031->C00123:[4->3,7->2,7->8,7->9,9->1,9->5] | 1.00 | 275.5 | 23 | 60 | 0 | 2 |
| Path 299 | C00031->C00123:[2->3,7->8] | 0.33 | 307.15 | 23 | 80 | 0 | 2 |
| Path 300 | C00031->C00123:[1->3,4->8,5->8,9->3] | 0.33 | 360.868131868 | 29 | 91 | 0 | 2 |
| Path 301 | C00031->C00123:[4->1,4->3,4->5,7->1,7->2,7->3,7->5,7->9,9->1,9->5] | 0.83 | 291.855072464 | 28 | 69 | 0 | 2 |
| Path 302 | C00031->C00123:[4->1,4->3,7->2] | 0.50 | 437.649350649 | 30 | 77 | 0 | 2 |
| Path 303 | C00031->C00123:[4->1,4->3,7->2] | 0.50 | 330.163934426 | 24 | 61 | 0 | 1 |
| Path 304 | C00031->C00123:[4->1,4->3,7->2] | 0.50 | 386.093333333 | 26 | 75 | 0 | 1 |
| Path 305 | C00031->C00123:[7->2,9->1,9->3] | 0.50 | 228.462962963 | 18 | 54 | 0 | 1 |
| Path 306 | C00031->C00123:[4->3,7->2,7->9,9->1,9->3,9->5] | 0.83 | 358.504587156 | 36 | 109 | 0 | 2 |
| Path 307 | C00031->C00123:[4->1,4->3] | 0.33 | 534.606060606 | 22 | 33 | 0 | 1 |
| Path 308 | C00031->C00123:[2->3,7->2,7->8,9->1] | 0.67 | 375.740384615 | 33 | 104 | 0 | 2 |
| Path 309 | C00031->C00123:[4->1,4->3,7->2] | 0.50 | 393.657894737 | 21 | 38 | 0 | 1 |
| Path 310 | C00031->C00123:[2->3,7->2,7->8,9->1] | 0.67 | 365.516853933 | 28 | 89 | 0 | 2 |
| Path 311 | C00031->C00123:[4->1,4->3,7->2,7->8,9->1] | 0.67 | 307.610169492 | 23 | 59 | 0 | 1 |
| Path 312 | C00031->C00123:[7->2,7->3,7->8,9->1] | 0.67 | 264.018867925 | 21 | 53 | 0 | 2 |
| Path 313 | C00031->C00123:[4->1,4->3,7->2] | 0.50 | 383.14893617 | 23 | 47 | 0 | 1 |
| Path 314 | C00031->C00123:[7->2,9->1] | 0.33 | 222.782608696 | 15 | 46 | 0 | 1 |
| Path 315 | C00031->C00123:[7->8,9->3] | 0.33 | 433.826086957 | 26 | 69 | 0 | 2 |
| Path 316 | C00031->C00123:[7->8,9->3] | 0.33 | 310.486842105 | 26 | 76 | 0 | 2 |
| Path 317 | C00031->C00123:[5->3,7->8] | 0.33 | 432.081081081 | 25 | 74 | 0 | 2 |
| Path 318 | C00031->C00123:[4->1,4->3,7->2] | 0.50 | 398.736111111 | 29 | 72 | 0 | 2 |
| Path 319 | C00031->C00123:[7->8,9->3] | 0.33 | 386.861111111 | 20 | 36 | 0 | 2 |
| Path 320 | C00031->C00123:[7->8,9->3] | 0.33 | 276.672727273 | 19 | 55 | 0 | 2 |
| Path 321 | C00031->C00123:[2->3,7->8,7->9,9->5] | 0.67 | 342.611111111 | 27 | 90 | 0 | 2 |
| Path 322 | C00031->C00123:[2->3,7->2,7->8,9->1] | 0.67 | 364.5 | 26 | 92 | 0 | 2 |
| Path 323 | C00031->C00123:[7->8,9->3] | 0.33 | 356.76744186 | 26 | 86 | 0 | 2 |
| Path 324 | C00031->C00123:[5->3,7->8,7->9,9->5] | 0.67 | 276.044776119 | 22 | 67 | 0 | 2 |
| Path 325 | C00031->C00123:[4->3,7->2,9->1] | 0.50 | 334.894736842 | 19 | 38 | 0 | 1 |
| Path 326 | C00031->C00123:[7->2,7->8,7->9,9->1,9->3,9->5] | 1.00 | 279.612903226 | 25 | 62 | 0 | 2 |
| Path 327 | C00031->C00123:[4->1,4->3,7->2] | 0.50 | 410.38 | 26 | 50 | 0 | 1 |
| Path 328 | C00031->C00123:[7->2,7->3,7->8,9->1] | 0.67 | 315.738461538 | 25 | 65 | 0 | 2 |
| Path 329 | C00031->C00123:[4->1,4->3,4->5,7->2,7->9,9->1,9->5] | 0.83 | 358.259259259 | 36 | 108 | 0 | 2 |
| Path 330 | C00031->C00123:[2->3,7->2,7->8,9->1] | 0.67 | 330.579545455 | 27 | 88 | 0 | 2 |
| Path 331 | C00031->C00123:[7->2,7->8,9->1,9->3] | 0.67 | 317.984848485 | 25 | 66 | 0 | 2 |
| Path 332 | C00031->C00123:[7->2,7->8,7->9,9->1,9->3,9->5] | 1.00 | 300.578947368 | 24 | 57 | 0 | 2 |
| Path 333 | C00031->C00123:[4->1,4->3,7->2] | 0.50 | 386.863636364 | 23 | 44 | 0 | 1 |
| Path 334 | C00031->C00123:[4->1,4->3,7->2,7->8,9->1] | 0.67 | 296.862068966 | 22 | 58 | 0 | 1 |
| Path 335 | C00031->C00123:[7->2,7->8,9->1] | 0.50 | 276.410714286 | 21 | 56 | 0 | 1 |
| Path 336 | C00031->C00123:[4->8,7->3] | 0.33 | 431.524590164 | 22 | 61 | 0 | 1 |
| Path 337 | C00031->C00123:[7->2,7->8,7->9,9->1,9->3,9->5] | 1.00 | 245.61971831 | 25 | 71 | 0 | 2 |
| Path 338 | C00031->C00123:[5->8,7->3] | 0.33 | 314.770491803 | 22 | 61 | 0 | 1 |
| Path 339 | C00031->C00123:[2->3,7->2,7->8,9->1] | 0.67 | 363.731182796 | 27 | 93 | 0 | 2 |
| Path 340 | C00031->C00123:[4->1,4->3,7->1,7->3] | 0.33 | 379.72972973 | 20 | 37 | 0 | 1 |
| Path 341 | C00031->C00123:[7->2,7->8,7->9,9->1,9->3,9->5] | 1.00 | 281.0 | 25 | 65 | 0 | 2 |
| Path 342 | C00031->C00123:[7->2,7->8,9->1] | 0.50 | 255.52 | 19 | 50 | 0 | 1 |
| Path 343 | C00031->C00123:[2->3] | 0.17 | 419.561403509 | 16 | 57 | 0 | 2 |
| Path 344 | C00031->C00123:[2->3,7->8,7->9,9->5] | 0.67 | 312.034883721 | 25 | 86 | 0 | 2 |
| Path 345 | C00031->C00123:[4->1,4->3,7->2] | 0.50 | 396.325 | 34 | 80 | 0 | 2 |
| Path 346 | C00031->C00123:[4->1,4->2,4->3] | 0.50 | 394.435897436 | 32 | 78 | 0 | 2 |
| Path 347 | C00031->C00123:[4->3,7->2,9->1] | 0.50 | 348.243902439 | 20 | 41 | 0 | 1 |
| Path 348 | C00031->C00123:[4->1,4->3,7->2] | 0.50 | 403.553191489 | 25 | 47 | 0 | 1 |
| Path 349 | C00031->C00123:[2->3,7->2,7->8,9->1] | 0.67 | 366.590909091 | 28 | 88 | 0 | 2 |
| Path 350 | C00031->C00123:[7->2,7->8,9->1,9->3] | 0.67 | 267.722222222 | 21 | 54 | 0 | 2 |
| Path 351 | C00031->C00123:[7->2,7->8,9->1,9->3] | 0.67 | 266.278350515 | 24 | 97 | 0 | 2 |
| Path 352 | C00031->C00123:[4->3,7->2,7->8,7->9,9->1,9->5] | 1.00 | 370.981818182 | 38 | 110 | 0 | 2 |
| Path 353 | C00031->C00123:[4->3,7->2,9->1] | 0.50 | 292.31372549 | 21 | 51 | 0 | 1 |
| Path 354 | C00031->C00123:[4->1,4->3,7->2] | 0.50 | 433.52 | 28 | 50 | 0 | 1 |
| Path 355 | C00031->C00123:[5->3,7->8] | 0.33 | 365.16 | 22 | 50 | 0 | 2 |
| Path 356 | C00031->C00123:[2->3,7->2,9->1] | 0.50 | 341.294117647 | 25 | 85 | 0 | 2 |
| Path 357 | C00031->C00123:[2->3,7->8,7->9,9->5] | 0.67 | 348.627906977 | 25 | 86 | 0 | 2 |
| Path 358 | C00031->C00123:[7->8,9->3] | 0.33 | 330.375 | 20 | 40 | 0 | 2 |
| Path 359 | C00031->C00123:[7->2,7->8,9->1,9->3] | 0.67 | 221.62295082 | 19 | 61 | 0 | 2 |
| Path 360 | C00031->C00123:[7->2,7->3,9->1] | 0.50 | 225.74 | 18 | 50 | 0 | 2 |
| Path 361 | C00031->C00123:[7->2,7->8,9->1,9->3] | 0.67 | 298.446428571 | 23 | 56 | 0 | 2 |
| Path 362 | C00031->C00123:[2->3,7->2,7->8,9->1] | 0.67 | 311.816091954 | 26 | 87 | 0 | 2 |
| Path 363 | C00031->C00123:[7->8,9->3] | 0.33 | 254.206896552 | 21 | 58 | 0 | 2 |
| Path 364 | C00031->C00123:[4->1,4->3,7->2] | 0.50 | 440.941176471 | 31 | 85 | 0 | 2 |
| Path 365 | C00031->C00123:[2->3,7->2,7->8,9->1] | 0.67 | 364.264367816 | 25 | 87 | 0 | 2 |
| Path 366 | C00031->C00123:[4->3,7->2,9->1] | 0.50 | 245.759259259 | 19 | 54 | 0 | 2 |
| Path 367 | C00031->C00123:[4->1,4->3,7->2] | 0.50 | 440.240963855 | 29 | 83 | 0 | 2 |
| Path 368 | C00031->C00123:[7->2,7->8,9->1,9->3] | 0.67 | 236.0 | 20 | 57 | 0 | 2 |
| Path 369 | C00031->C00123:[4->3,7->2,7->8,7->9,9->1,9->5] | 1.00 | 275.298507463 | 26 | 67 | 0 | 2 |
| Path 370 | C00031->C00123:[7->2,9->1,9->3] | 0.50 | 302.484848485 | 16 | 33 | 0 | 1 |
| Path 371 | C00031->C00123:[2->3,7->2,9->1] | 0.50 | 299.975 | 23 | 80 | 0 | 2 |
| Path 372 | C00031->C00123:[4->3,7->2,7->8,9->1] | 0.67 | 326.188405797 | 26 | 69 | 0 | 2 |
| Path 373 | C00031->C00123:[4->3,7->2,7->8,9->1,9->3] | 0.67 | 310.953846154 | 25 | 65 | 0 | 1 |
| Path 374 | C00031->C00123:[4->3,7->2,7->8,7->9,9->1,9->3,9->5] | 1.00 | 373.644067797 | 39 | 118 | 0 | 2 |
| Path 375 | C00031->C00123:[4->1,4->2,4->3] | 0.50 | 445.136986301 | 29 | 73 | 0 | 2 |
| Path 376 | C00031->C00123:[5->3] | 0.17 | 327.052631579 | 13 | 38 | 0 | 2 |
| Path 377 | C00031->C00123:[7->2,7->8,9->1,9->3] | 0.67 | 327.134328358 | 26 | 67 | 0 | 2 |
| Path 378 | C00031->C00123:[7->2,7->9,9->1,9->3,9->5] | 0.83 | 286.85 | 23 | 60 | 0 | 2 |
| Path 379 | C00031->C00123:[2->3] | 0.17 | 357.132075472 | 17 | 53 | 0 | 2 |
| Path 380 | C00031->C00123:[7->8,9->3] | 0.33 | 240.64 | 17 | 50 | 0 | 2 |
| Path 381 | C00031->C00123:[1->1,4->2,7->3] | 0.50 | 422.114754098 | 22 | 61 | 0 | 1 |
| Path 382 | C00031->C00123:[1->1,4->2,4->8,5->2,5->8,9->1] | 0.50 | 357.67816092 | 27 | 87 | 0 | 1 |
| Path 383 | C00031->C00123:[4->3,9->8] | 0.33 | 280.145454545 | 21 | 55 | 0 | 1 |
| Path 384 | C00031->C00123:[4->3,7->2,7->8,9->1] | 0.67 | 280.517241379 | 23 | 58 | 0 | 2 |
| Path 385 | C00031->C00123:[2->3,7->2,9->1] | 0.50 | 296.308641975 | 25 | 81 | 0 | 2 |
| Path 386 | C00031->C00123:[4->1,4->3,4->5,7->2,7->9,9->1,9->5] | 0.83 | 310.023529412 | 31 | 85 | 0 | 2 |
| Path 387 | C00031->C00123:[2->3,7->2,9->1] | 0.50 | 345.05952381 | 22 | 84 | 0 | 2 |
| Path 388 | C00031->C00123:[4->1,4->3,7->1,7->2,7->3] | 0.50 | 343.175438596 | 25 | 57 | 0 | 1 |
| Path 389 | C00031->C00123:[4->3] | 0.17 | 325.235294118 | 9 | 17 | 0 | 2 |
| Path 390 | C00031->C00123:[7->2,9->1,9->3] | 0.50 | 245.483870968 | 20 | 93 | 0 | 2 |
| Path 391 | C00031->C00123:[4->1,4->3,4->5,7->2,7->9,9->1,9->5] | 0.83 | 331.808080808 | 33 | 99 | 0 | 2 |
| Path 392 | C00031->C00123:[4->3,5->8] | 0.33 | 326.542857143 | 25 | 70 | 0 | 2 |
| Path 393 | C00031->C00123:[7->2,7->8,9->1] | 0.50 | 271.833333333 | 22 | 60 | 0 | 1 |
| Path 394 | C00031->C00123:[4->3,7->2,7->8,7->9,9->1,9->3,9->5] | 1.00 | 341.834862385 | 39 | 109 | 0 | 2 |
| Path 395 | C00031->C00123:[2->3,7->2,7->8,7->9,9->1,9->5] | 1.00 | 342.446808511 | 29 | 94 | 0 | 2 |
| Path 396 | C00031->C00123:[4->1,4->2,4->3] | 0.50 | 436.012195122 | 31 | 82 | 0 | 2 |
| Path 397 | C00031->C00123:[2->3,7->8,7->9,9->5] | 0.67 | 304.674698795 | 27 | 83 | 0 | 2 |
| Path 398 | C00031->C00123:[4->3,7->2,7->9,9->1,9->5] | 0.83 | 269.777777778 | 26 | 81 | 0 | 1 |
| Path 399 | C00031->C00123:[4->1,4->3,7->2] | 0.50 | 381.37254902 | 25 | 51 | 0 | 1 |
| Path 400 | C00031->C00123:[7->2,7->8,9->1] | 0.50 | 217.519230769 | 17 | 52 | 0 | 1 |
| Path 401 | C00031->C00123:[7->2,7->8,9->1,9->3] | 0.67 | 290.146666667 | 25 | 75 | 0 | 2 |
| Path 402 | C00031->C00123:[7->2,9->1] | 0.33 | 251.704545455 | 17 | 44 | 0 | 1 |
| Path 403 | C00031->C00123:[7->8,7->9,9->3,9->5] | 0.67 | 350.825581395 | 26 | 86 | 0 | 2 |
| Path 404 | C00031->C00123:[7->8,7->9,9->5] | 0.50 | 288.0 | 20 | 51 | 0 | 1 |
| Path 405 | C00031->C00123:[7->8,9->3] | 0.33 | 394.871794872 | 20 | 39 | 0 | 2 |
| Path 406 | C00031->C00123:[7->2,7->8,7->9,9->1,9->3,9->5] | 1.00 | 248.474576271 | 22 | 59 | 0 | 2 |
| Path 407 | C00031->C00123:[5->3,7->2,7->8,9->1] | 0.67 | 364.76344086 | 27 | 93 | 0 | 2 |
| Path 408 | C00031->C00123:[2->3,7->8] | 0.33 | 303.395061728 | 25 | 81 | 0 | 2 |
| Path 409 | C00031->C00123:[2->3,7->8] | 0.33 | 351.892857143 | 22 | 84 | 0 | 2 |
| Path 410 | C00031->C00123:[4->3,7->2,7->9,9->1,9->3,9->5] | 0.83 | 300.042857143 | 26 | 70 | 0 | 1 |
| Path 411 | C00031->C00123:[4->3,7->8] | 0.33 | 369.157894737 | 20 | 38 | 0 | 2 |
| Path 412 | C00031->C00123:[4->1,4->3,7->2,9->1] | 0.50 | 380.609375 | 28 | 64 | 0 | 1 |
| Path 413 | C00031->C00123:[4->1,4->3] | 0.33 | 433.935483871 | 19 | 31 | 0 | 1 |
| Path 414 | C00031->C00123:[2->3,7->2,7->8,9->1] | 0.67 | 359.404494382 | 28 | 89 | 0 | 2 |
| Path 415 | C00031->C00123:[2->3,7->2,7->8,9->1] | 0.67 | 308.353658537 | 25 | 82 | 0 | 2 |
| Path 416 | C00031->C00123:[4->3,7->2,7->8,9->1] | 0.67 | 259.363636364 | 20 | 55 | 0 | 2 |
| Path 417 | C00031->C00123:[7->2,7->8,7->9,9->1,9->3,9->5] | 1.00 | 270.838709677 | 25 | 62 | 0 | 2 |
| Path 418 | C00031->C00123:[4->3,4->5,7->8,7->9,9->5] | 0.67 | 310.596774194 | 24 | 62 | 0 | 1 |
| Path 419 | C00031->C00123:[7->8,9->3] | 0.33 | 341.363636364 | 25 | 66 | 0 | 2 |
| Path 420 | C00031->C00123:[2->3,7->2,7->8,9->1] | 0.67 | 327.573033708 | 30 | 89 | 0 | 2 |
| Path 421 | C00031->C00123:[4->3,7->2,7->8,9->1] | 0.67 | 301.216666667 | 23 | 60 | 0 | 2 |
| Path 422 | C00031->C00123:[4->3,9->8] | 0.33 | 292.413793103 | 22 | 58 | 0 | 1 |
| Path 423 | C00031->C00123:[4->3,7->2,7->8,7->9,9->1,9->5] | 1.00 | 337.933962264 | 39 | 106 | 0 | 2 |
| Path 424 | C00031->C00123:[2->3,7->2,7->8,7->9,9->1,9->5] | 1.00 | 343.263157895 | 30 | 95 | 0 | 2 |
| Path 425 | C00031->C00123:[4->1,4->3,7->2] | 0.50 | 418.425 | 23 | 40 | 0 | 1 |
| Path 426 | C00031->C00123:[4->1,4->3,7->2,7->8,9->1] | 0.67 | 414.30952381 | 23 | 42 | 0 | 1 |
| Path 427 | C00031->C00123:[7->8,7->9,9->3,9->5] | 0.67 | 277.796296296 | 21 | 54 | 0 | 2 |
| Path 428 | C00031->C00123:[2->3,7->8,7->9,9->5] | 0.67 | 353.577777778 | 24 | 90 | 0 | 2 |
| Path 429 | C00031->C00123:[4->3,7->2,7->9,9->1,9->3,9->5] | 0.83 | 338.141509434 | 36 | 106 | 0 | 2 |
| Path 430 | C00031->C00123:[1->1,2->3,4->2] | 0.50 | 405.885714286 | 20 | 70 | 0 | 1 |
| Path 431 | C00031->C00123:[7->3,7->8] | 0.33 | 258.591836735 | 18 | 49 | 0 | 1 |
| Path 432 | C00031->C00123:[4->1,4->3,4->5,7->2,7->8,7->9,9->1,9->5] | 1.00 | 315.556818182 | 34 | 88 | 0 | 2 |
| Path 433 | C00031->C00123:[4->1,4->3,4->5,7->2,7->8,7->9,9->1,9->5] | 1.00 | 370.311320755 | 35 | 106 | 0 | 2 |
| Path 434 | C00031->C00123:[7->8,7->9,9->3,9->5] | 0.67 | 241.761904762 | 21 | 63 | 0 | 2 |
| Path 435 | C00031->C00123:[4->1,4->3,7->2] | 0.50 | 406.985507246 | 25 | 69 | 0 | 1 |
| Path 436 | C00031->C00123:[4->1,4->3,7->2] | 0.50 | 425.325581395 | 24 | 43 | 0 | 1 |
| Path 437 | C00031->C00123:[5->3,7->2,7->8,9->1] | 0.67 | 300.628571429 | 25 | 70 | 0 | 2 |
| Path 438 | C00031->C00123:[4->3,7->2,7->9,9->1,9->3,9->5] | 0.83 | 339.513513514 | 37 | 111 | 0 | 2 |
| Path 439 | C00031->C00123:[4->1,4->3,7->2] | 0.50 | 441.55952381 | 30 | 84 | 0 | 2 |
| Path 440 | C00031->C00123:[4->3,7->2,9->1] | 0.50 | 318.783783784 | 18 | 37 | 0 | 1 |
| Path 441 | C00031->C00123:[7->1,7->3] | 0.33 | 373.152173913 | 24 | 46 | 0 | 2 |
| Path 442 | C00031->C00123:[5->3,7->8] | 0.33 | 283.671641791 | 22 | 67 | 0 | 2 |
| Path 443 | C00031->C00123:[2->3,7->2,9->1] | 0.50 | 359.522727273 | 23 | 88 | 0 | 1 |
| Path 444 | C00031->C00123:[7->8,7->9,9->3,9->5] | 0.67 | 244.156862745 | 18 | 51 | 0 | 2 |
| Path 445 | C00031->C00123:[4->1,4->3,7->2] | 0.50 | 437.35 | 28 | 80 | 0 | 2 |
| Path 446 | C00031->C00123:[2->3,7->8,7->9,9->5] | 0.67 | 311.816091954 | 26 | 87 | 0 | 2 |
| Path 447 | C00031->C00123:[4->1,4->3] | 0.33 | 376.428571429 | 17 | 28 | 0 | 2 |
| Path 448 | C00031->C00123:[4->3,7->2,7->8,7->9,9->1,9->5] | 1.00 | 263.953125 | 25 | 64 | 0 | 2 |
| Path 449 | C00031->C00123:[4->1,4->3,7->2] | 0.50 | 385.3125 | 24 | 48 | 0 | 1 |
| Path 450 | C00031->C00123:[4->1,4->3,7->1,7->3] | 0.33 | 374.357142857 | 22 | 42 | 0 | 2 |
| Path 451 | C00031->C00123:[4->1,4->3,7->2,7->8,9->1] | 0.67 | 422.608695652 | 25 | 46 | 0 | 1 |
| Path 452 | C00031->C00123:[7->2,7->8,9->1,9->3] | 0.67 | 222.774193548 | 20 | 62 | 0 | 2 |
| Path 453 | C00031->C00123:[2->3,7->8] | 0.33 | 347.788235294 | 24 | 85 | 0 | 2 |
| Path 454 | C00031->C00123:[4->3,7->8] | 0.33 | 396.384615385 | 20 | 39 | 0 | 1 |
| Path 455 | C00031->C00123:[7->8,7->9,9->3,9->5] | 0.67 | 279.473684211 | 21 | 57 | 0 | 2 |
| Path 456 | C00031->C00123:[7->1,7->2,7->3] | 0.50 | 401.510638298 | 26 | 47 | 0 | 1 |
| Path 457 | C00031->C00123:[7->3,7->8] | 0.33 | 372.59375 | 18 | 32 | 0 | 1 |
| Path 458 | C00031->C00123:[4->3,7->2,7->8,7->9,9->1,9->5] | 1.00 | 371.181818182 | 39 | 110 | 0 | 2 |
| Path 459 | C00031->C00123:[2->3,7->2,9->1] | 0.50 | 435.070422535 | 23 | 71 | 0 | 1 |
| Path 460 | C00031->C00123:[7->2,7->8,9->1,9->3] | 0.67 | 367.640449438 | 29 | 89 | 0 | 2 |
| Path 461 | C00031->C00123:[4->3,7->2,7->9,9->1,9->5] | 0.83 | 368.140186916 | 35 | 107 | 0 | 2 |
| Path 462 | C00031->C00123:[2->3] | 0.17 | 424.980769231 | 16 | 52 | 0 | 2 |
| Path 463 | C00031->C00123:[4->1,4->2,4->3] | 0.50 | 391.154929577 | 29 | 71 | 0 | 2 |
| Path 464 | C00031->C00123:[5->3,7->8] | 0.33 | 273.863636364 | 21 | 66 | 0 | 2 |
| Path 465 | C00031->C00123:[2->3,7->2,7->8,9->1] | 0.67 | 360.159090909 | 27 | 88 | 0 | 2 |
| Path 466 | C00031->C00123:[4->3,7->2,9->1] | 0.50 | 304.814814815 | 22 | 54 | 0 | 1 |
| Path 467 | C00031->C00123:[4->1,4->3,7->1,7->3] | 0.33 | 395.894736842 | 21 | 38 | 0 | 1 |
| Path 468 | C00031->C00123:[7->2,9->1] | 0.33 | 347.7 | 16 | 30 | 0 | 1 |
| Path 469 | C00031->C00123:[4->1,4->3,4->5,7->2,7->8,7->9,9->1,9->5] | 1.00 | 367.08411215 | 38 | 107 | 0 | 2 |
| Path 470 | C00031->C00123:[2->3,7->2,9->1] | 0.50 | 441.958333333 | 24 | 72 | 0 | 1 |
| Path 471 | C00031->C00123:[4->1,4->3,4->5,7->2,7->8,7->9,9->1,9->5] | 1.00 | 297.876712329 | 30 | 73 | 0 | 2 |
| Path 472 | C00031->C00123:[2->3,7->8,7->9,9->5] | 0.67 | 347.988505747 | 26 | 87 | 0 | 2 |
| Path 473 | C00031->C00123:[4->3,7->8] | 0.33 | 263.923076923 | 18 | 52 | 0 | 1 |
| Path 474 | C00031->C00123:[4->3,7->8] | 0.33 | 276.509433962 | 19 | 53 | 0 | 1 |
| Path 475 | C00031->C00123:[7->2,7->3,9->1] | 0.50 | 232.625 | 17 | 48 | 0 | 1 |
| Path 476 | C00031->C00123:[2->3,7->2,7->8,7->9,9->1,9->5] | 1.00 | 338.01010101 | 32 | 99 | 0 | 2 |
| Path 477 | C00031->C00123:[4->1,4->3,7->2] | 0.50 | 404.714285714 | 23 | 42 | 0 | 1 |
| Path 478 | C00031->C00123:[2->3,7->2,7->8,9->1] | 0.67 | 367.183673469 | 30 | 98 | 0 | 2 |
| Path 479 | C00031->C00123:[7->1,7->2,7->3] | 0.50 | 387.591836735 | 27 | 49 | 0 | 2 |
| Path 480 | C00031->C00123:[4->3,7->2,7->8,9->1] | 0.67 | 272.706896552 | 21 | 58 | 0 | 2 |
| Path 481 | C00031->C00123:[7->2,7->3,7->8,7->9,9->1,9->5] | 1.00 | 245.576271186 | 23 | 59 | 0 | 2 |
| Path 482 | C00031->C00123:[2->3,7->2,7->8,9->1] | 0.67 | 353.11827957 | 30 | 93 | 0 | 2 |
| Path 483 | C00031->C00123:[4->1,4->3,7->2] | 0.50 | 414.541666667 | 26 | 48 | 0 | 1 |
| Path 484 | C00031->C00123:[4->1,4->3,9->2,9->8] | 0.67 | 309.868852459 | 25 | 61 | 0 | 1 |
| Path 485 | C00031->C00123:[5->3] | 0.17 | 421.677419355 | 16 | 62 | 0 | 2 |
| Path 486 | C00031->C00123:[2->3,7->2,7->8,9->1] | 0.67 | 358.967741935 | 30 | 93 | 0 | 2 |
| Path 487 | C00031->C00123:[4->1,4->3,4->5,7->2,7->9,9->1,9->5] | 0.83 | 328.52 | 35 | 100 | 0 | 2 |
| Path 488 | C00031->C00123:[7->2,9->1,9->3] | 0.50 | 350.072289157 | 24 | 83 | 0 | 1 |
| Path 489 | C00031->C00123:[4->1,4->2,4->3] | 0.50 | 431.0 | 28 | 74 | 0 | 2 |
| Path 490 | C00031->C00123:[7->8,9->3] | 0.33 | 230.0 | 19 | 61 | 0 | 2 |
| Path 491 | C00031->C00123:[4->1,4->3,7->2] | 0.50 | 443.368421053 | 28 | 76 | 0 | 2 |
| Path 492 | C00031->C00123:[4->3,7->2,7->8,7->9,9->1,9->3,9->5] | 1.00 | 370.807017544 | 41 | 114 | 0 | 2 |
| Path 493 | C00031->C00123:[7->8,7->9,9->3,9->5] | 0.67 | 277.509433962 | 20 | 53 | 0 | 2 |
| Path 494 | C00031->C00123:[2->3,7->2,7->8,7->9,9->1,9->5] | 1.00 | 310.136842105 | 30 | 95 | 0 | 2 |
| Path 495 | C00031->C00123:[2->3,7->2,7->8,7->9,9->1,9->5] | 1.00 | 342.680851064 | 30 | 94 | 0 | 2 |
| Path 496 | C00031->C00123:[7->8,7->9,9->5] | 0.50 | 255.52 | 19 | 50 | 0 | 1 |
| Path 497 | C00031->C00123:[5->3,5->8] | 0.33 | 290.117647059 | 23 | 68 | 0 | 2 |
| Path 498 | C00031->C00123:[7->8] | 0.17 | 219.8 | 14 | 45 | 0 | 1 |
| Path 499 | C00031->C00123:[7->8,7->9,9->3,9->5] | 0.67 | 298.545454545 | 22 | 55 | 0 | 2 |
| Path 500 | C00031->C00123:[7->8,7->9,9->3,9->5] | 0.67 | 298.446428571 | 23 | 56 | 0 | 2 |
| Path 501 | C00031->C00123:[2->3,7->2,7->8,7->9,9->1,9->5] | 1.00 | 346.096774194 | 27 | 93 | 0 | 2 |
| Path 502 | C00031->C00123:[7->2,7->8,9->1,9->3] | 0.67 | 341.128571429 | 26 | 70 | 0 | 2 |
| Path 503 | C00031->C00123:[4->3,7->2,9->1] | 0.50 | 267.309090909 | 19 | 55 | 0 | 1 |
| Path 504 | C00031->C00123:[7->2,7->8,9->1,9->3] | 0.67 | 271.666666667 | 23 | 96 | 0 | 2 |
| Path 505 | C00031->C00123:[4->3,7->2,7->9,9->1,9->5] | 0.83 | 351.409090909 | 27 | 66 | 0 | 1 |
| Path 506 | C00031->C00123:[4->3,7->2,7->9,9->1,9->5] | 0.83 | 313.670886076 | 31 | 79 | 0 | 2 |
| Path 507 | C00031->C00123:[4->1,4->3,4->5,7->2,7->9,9->1,9->5] | 0.83 | 305.631578947 | 30 | 76 | 0 | 2 |
| Path 508 | C00031->C00123:[7->8,7->9,9->3,9->5] | 0.67 | 236.982758621 | 21 | 58 | 0 | 2 |
| Path 509 | C00031->C00123:[4->1,4->3,4->5,7->2,7->8,7->9,9->1,9->5] | 1.00 | 311.962025316 | 33 | 79 | 0 | 2 |
| Path 510 | C00031->C00123:[4->1,4->3] | 0.33 | 422.703703704 | 17 | 27 | 0 | 1 |
| Path 511 | C00031->C00123:[5->3,7->2,7->8,9->1] | 0.67 | 292.857142857 | 25 | 70 | 0 | 2 |
| Path 512 | C00031->C00123:[7->2,7->8,7->9,9->1,9->3,9->5] | 1.00 | 253.661538462 | 23 | 65 | 0 | 2 |
| Path 513 | C00031->C00123:[7->2,7->8,9->1,9->3] | 0.67 | 257.016949153 | 22 | 59 | 0 | 2 |
| Path 514 | C00031->C00123:[7->2,7->8,9->1] | 0.50 | 250.479166667 | 17 | 48 | 0 | 1 |
| Path 515 | C00031->C00123:[2->3,7->8] | 0.33 | 385.47826087 | 25 | 69 | 0 | 2 |
| Path 516 | C00031->C00123:[5->3,7->8] | 0.33 | 359.582417582 | 25 | 91 | 0 | 2 |
| Path 517 | C00031->C00123:[4->1,4->3,7->2] | 0.50 | 407.3125 | 30 | 96 | 0 | 1 |
| Path 518 | C00031->C00123:[2->3,7->8] | 0.33 | 432.671232877 | 24 | 73 | 0 | 2 |
| Path 519 | C00031->C00123:[7->8,9->3] | 0.33 | 251.655913978 | 20 | 93 | 0 | 2 |
| Path 520 | C00031->C00123:[7->8,7->9,9->5] | 0.50 | 290.423076923 | 21 | 52 | 0 | 1 |
| Path 521 | C00031->C00123:[4->1,4->3,7->1,7->2,7->3] | 0.50 | 403.128571429 | 26 | 70 | 0 | 1 |
| Path 522 | C00031->C00123:[2->3,7->2,7->8,7->9,9->1,9->5] | 1.00 | 353.641304348 | 26 | 92 | 0 | 2 |
| Path 523 | C00031->C00123:[4->3,4->5,7->8,7->9] | 0.67 | 395.315789474 | 21 | 38 | 0 | 1 |
| Path 524 | C00031->C00123:[4->1,4->2,4->3,4->8] | 0.67 | 447.901408451 | 27 | 71 | 0 | 1 |
| Path 525 | C00031->C00123:[2->3,7->2,7->8,9->1] | 0.67 | 324.416666667 | 28 | 84 | 0 | 2 |
| Path 526 | C00031->C00123:[4->1,4->2,4->3] | 0.50 | 362.202898551 | 26 | 69 | 0 | 1 |
| Path 527 | C00031->C00123:[7->2,7->8,7->9,9->1,9->3,9->5] | 1.00 | 255.215686275 | 25 | 102 | 0 | 2 |
| Path 528 | C00031->C00123:[4->1,4->3,4->5,7->2,7->9,9->1,9->5] | 0.83 | 364.048076923 | 35 | 104 | 0 | 2 |
| Path 529 | C00031->C00123:[2->3,7->2,7->8,7->9,9->1,9->5] | 1.00 | 346.882978723 | 28 | 94 | 0 | 2 |
| Path 530 | C00031->C00123:[4->1,4->3] | 0.33 | 407.342105263 | 22 | 38 | 0 | 2 |
| Path 531 | C00031->C00123:[7->2,7->8,7->9,9->1,9->3,9->5] | 1.00 | 268.393442623 | 24 | 61 | 0 | 2 |
| Path 532 | C00031->C00123:[7->2,7->8,7->9,9->1,9->3,9->5] | 1.00 | 295.682539683 | 26 | 63 | 0 | 2 |
| Path 533 | C00031->C00123:[7->2,7->8,9->1,9->3] | 0.67 | 299.016949153 | 23 | 59 | 0 | 2 |
| Path 534 | C00031->C00123:[5->3,7->2,7->8,9->1] | 0.67 | 353.967032967 | 25 | 91 | 0 | 2 |
| Path 535 | C00031->C00123:[2->3,7->2,7->8,9->1] | 0.67 | 370.517241379 | 25 | 87 | 0 | 2 |
| Path 536 | C00031->C00123:[5->3,7->2,9->1] | 0.50 | 265.166666667 | 21 | 66 | 0 | 2 |
| Path 537 | C00031->C00123:[7->2,7->8,9->1,9->3] | 0.67 | 351.191176471 | 27 | 68 | 0 | 2 |
| Path 538 | C00031->C00123:[7->2,9->1] | 0.33 | 327.586206897 | 15 | 29 | 0 | 1 |
| Path 539 | C00031->C00123:[4->3,7->2,9->1] | 0.50 | 395.4 | 21 | 40 | 0 | 1 |
| Path 540 | C00031->C00123:[4->3,7->8] | 0.33 | 281.517241379 | 21 | 58 | 0 | 2 |
| Path 541 | C00031->C00123:[7->2,7->3,7->8,9->1] | 0.67 | 274.283018868 | 21 | 53 | 0 | 2 |
| Path 542 | C00031->C00123:[7->2,7->8,9->1,9->3] | 0.67 | 242.5625 | 22 | 64 | 0 | 2 |
| Path 543 | C00031->C00123:[2->3,7->2,9->1] | 0.50 | 414.835616438 | 23 | 73 | 0 | 1 |
| Path 544 | C00031->C00123:[4->3,7->2,7->8,9->1] | 0.67 | 334.152777778 | 27 | 72 | 0 | 2 |
| Path 545 | C00031->C00123:[2->3,7->2,7->8,7->9,9->1,9->5] | 1.00 | 309.698795181 | 26 | 83 | 0 | 2 |
| Path 546 | C00031->C00123:[7->2,7->9,9->1,9->5] | 0.67 | 234.13559322 | 20 | 59 | 0 | 1 |
| Path 547 | C00031->C00123:[2->3,7->2,7->9,9->1,9->5] | 0.83 | 303.582417582 | 26 | 91 | 0 | 2 |
| Path 548 | C00031->C00123:[5->3,7->8] | 0.33 | 353.233333333 | 24 | 90 | 0 | 2 |
| Path 549 | C00031->C00123:[4->3,7->2,7->8,9->1] | 0.67 | 307.704918033 | 23 | 61 | 0 | 1 |
| Path 550 | C00031->C00123:[7->2,7->9,9->1,9->5] | 0.67 | 212.315789474 | 18 | 57 | 0 | 1 |
| Path 551 | C00031->C00123:[4->3,7->2,7->8,9->1] | 0.67 | 351.409090909 | 27 | 66 | 0 | 1 |
| Path 552 | C00031->C00123:[2->3,7->2,7->8,9->1] | 0.67 | 312.034883721 | 25 | 86 | 0 | 2 |
| Path 553 | C00031->C00123:[7->2,7->8,9->1] | 0.50 | 274.0 | 20 | 55 | 0 | 1 |
| Path 554 | C00031->C00123:[4->3,7->2,7->8,7->9,9->1,9->5] | 1.00 | 262.771929825 | 22 | 57 | 0 | 2 |
| Path 555 | C00031->C00123:[4->1,4->3] | 0.33 | 538.034482759 | 20 | 29 | 0 | 1 |
| Path 556 | C00031->C00123:[7->2,7->8,9->1,9->3] | 0.67 | 236.982758621 | 21 | 58 | 0 | 2 |
| Path 557 | C00031->C00123:[4->3,4->5,4->8,4->9] | 0.67 | 469.090909091 | 26 | 66 | 0 | 1 |
| Path 558 | C00031->C00123:[4->1,4->3,4->5,7->1,7->2,7->3,7->5,7->8,7->9,9->1,9->5] | 1.00 | 299.375 | 31 | 72 | 0 | 2 |
| Path 559 | C00031->C00123:[4->1,4->3,7->2] | 0.50 | 398.210526316 | 32 | 76 | 0 | 2 |
| Path 560 | C00031->C00123:[7->2,7->3,7->8,7->9,9->1,9->5] | 1.00 | 245.169811321 | 21 | 53 | 0 | 2 |
| Path 561 | C00031->C00123:[2->3,7->2,7->8,9->1] | 0.67 | 359.684782609 | 29 | 92 | 0 | 2 |
| Path 562 | C00031->C00123:[4->1,4->3] | 0.33 | 400.730769231 | 16 | 26 | 0 | 1 |
| Path 563 | C00031->C00123:[4->3,7->2,7->8,7->9,9->1,9->5] | 1.00 | 273.106060606 | 25 | 66 | 0 | 2 |
| Path 564 | C00031->C00123:[5->3,7->2,7->8,9->1] | 0.67 | 364.0 | 28 | 94 | 0 | 2 |
| Path 565 | C00031->C00123:[7->2,7->9,9->1,9->3,9->5] | 0.83 | 236.482142857 | 19 | 56 | 0 | 2 |
| Path 566 | C00031->C00123:[2->3,7->2,7->8,9->1] | 0.67 | 367.107526882 | 29 | 93 | 0 | 2 |
| Path 567 | C00031->C00123:[7->8,7->9,9->5] | 0.50 | 228.191489362 | 16 | 47 | 0 | 1 |
| Path 568 | C00031->C00123:[4->3,7->2,7->9,9->1,9->5] | 0.83 | 296.378787879 | 24 | 66 | 0 | 1 |
| Path 569 | C00031->C00123:[2->3,7->2,7->8,9->1] | 0.67 | 330.157303371 | 28 | 89 | 0 | 2 |
| Path 570 | C00031->C00123:[2->3,7->8,7->9,9->5] | 0.67 | 348.88372093 | 26 | 86 | 0 | 2 |
| Path 571 | C00031->C00123:[2->3,7->2,7->8,9->1] | 0.67 | 304.817073171 | 26 | 82 | 0 | 2 |
| Path 572 | C00031->C00123:[7->8,7->9,9->3,9->5] | 0.67 | 251.0625 | 22 | 64 | 0 | 2 |
| Path 573 | C00031->C00123:[4->1,4->3,7->2] | 0.50 | 396.923076923 | 27 | 52 | 0 | 2 |
| Path 574 | C00031->C00123:[2->3,7->8] | 0.33 | 358.705882353 | 23 | 85 | 0 | 2 |
| Path 575 | C00031->C00123:[7->2,7->9,9->1,9->3,9->5] | 0.83 | 267.224137931 | 21 | 58 | 0 | 2 |
| Path 576 | C00031->C00123:[4->1,4->2,4->3] | 0.50 | 396.27027027 | 30 | 74 | 0 | 2 |
| Path 577 | C00031->C00123:[7->2,7->8,7->9,9->1,9->3,9->5] | 1.00 | 248.041666667 | 26 | 72 | 0 | 2 |
| Path 578 | C00031->C00123:[2->3,7->2,7->8,7->9,9->1,9->5] | 1.00 | 347.244897959 | 28 | 98 | 0 | 2 |
| Path 579 | C00031->C00123:[4->3,7->2,7->9,9->1,9->5] | 0.83 | 287.550724638 | 26 | 69 | 0 | 2 |
| Path 580 | C00031->C00123:[5->3,5->8] | 0.33 | 361.326315789 | 27 | 95 | 0 | 2 |
| Path 581 | C00031->C00123:[4->3,7->2,7->8,9->1] | 0.67 | 292.163934426 | 24 | 61 | 0 | 2 |
| Path 582 | C00031->C00123:[5->3,7->2,7->8,9->1] | 0.67 | 369.787234043 | 28 | 94 | 0 | 2 |
| Path 583 | C00031->C00123:[7->2,7->9,9->1,9->3,9->5] | 0.83 | 229.725806452 | 21 | 62 | 0 | 2 |
| Path 584 | C00031->C00123:[2->3,5->8] | 0.33 | 377.785714286 | 29 | 98 | 0 | 2 |
| Path 585 | C00031->C00123:[4->3,7->2,7->9,9->1,9->5] | 0.83 | 317.090909091 | 32 | 88 | 0 | 2 |
| Path 586 | C00031->C00123:[4->1,4->3,7->2] | 0.50 | 374.0 | 22 | 44 | 0 | 1 |
| Path 587 | C00031->C00123:[9->3] | 0.17 | 281.586206897 | 12 | 29 | 0 | 2 |
| Path 588 | C00031->C00123:[7->2,7->8,7->9,9->1,9->3,9->5] | 1.00 | 245.292307692 | 23 | 65 | 0 | 2 |
| Path 589 | C00031->C00123:[4->1,4->3,7->2] | 0.50 | 396.203703704 | 27 | 54 | 0 | 1 |
| Path 590 | C00031->C00123:[7->8,7->9,9->5] | 0.50 | 282.163636364 | 21 | 55 | 0 | 1 |
| Path 591 | C00031->C00123:[4->3,7->2,7->8,9->1] | 0.67 | 325.714285714 | 27 | 70 | 0 | 2 |
| Path 592 | C00031->C00123:[2->3,7->2,9->1] | 0.50 | 346.382022472 | 23 | 89 | 0 | 2 |
| Path 593 | C00031->C00123:[4->1,4->3,7->2] | 0.50 | 438.146341463 | 30 | 82 | 0 | 2 |
| Path 594 | C00031->C00123:[7->1,7->2,7->3,7->5,7->8,7->9,9->1,9->5] | 1.00 | 310.746835443 | 34 | 79 | 0 | 2 |
| Path 595 | C00031->C00123:[7->2,9->1,9->3] | 0.50 | 299.320754717 | 21 | 53 | 0 | 1 |
| Path 596 | C00031->C00123:[4->1,4->3,4->5,7->1,7->2,7->3,7->5,7->9,9->1,9->5] | 0.83 | 301.25 | 29 | 72 | 0 | 2 |
| Path 597 | C00031->C00123:[4->1,4->3,4->5,7->2,7->9,9->1,9->5] | 0.83 | 349.506666667 | 31 | 75 | 0 | 1 |
| Path 598 | C00031->C00123:[7->2,7->3,7->8,9->1] | 0.67 | 240.803921569 | 19 | 51 | 0 | 2 |
| Path 599 | C00031->C00123:[4->3,7->2,7->3,7->9,9->1,9->5] | 0.83 | 362.927536232 | 30 | 69 | 0 | 1 |
| Path 600 | C00031->C00123:[2->3,7->2,7->8,9->1] | 0.67 | 352.0 | 24 | 86 | 0 | 2 |
| Path 601 | C00031->C00123:[7->2,7->8,7->9,9->1,9->3,9->5] | 1.00 | 243.636363636 | 25 | 66 | 0 | 2 |
| Path 602 | C00031->C00123:[2->3,7->2,7->8,9->1] | 0.67 | 369.580645161 | 27 | 93 | 0 | 2 |
| Path 603 | C00031->C00123:[2->3,7->2,7->9,9->1,9->5] | 0.83 | 343.294736842 | 25 | 95 | 0 | 2 |
| Path 604 | C00031->C00123:[2->3] | 0.17 | 427.0 | 14 | 56 | 0 | 2 |
| Path 605 | C00031->C00123:[4->1,4->2,4->3] | 0.50 | 394.826666667 | 28 | 75 | 0 | 2 |
| Path 606 | C00031->C00123:[7->2,7->8,9->1,9->3] | 0.67 | 362.306818182 | 28 | 88 | 0 | 2 |
| Path 607 | C00031->C00123:[2->3] | 0.17 | 363.942307692 | 15 | 52 | 0 | 2 |
| Path 608 | C00031->C00123:[7->2,7->8,9->1,9->3] | 0.67 | 271.886597938 | 24 | 97 | 0 | 2 |
| Path 609 | C00031->C00123:[7->2,9->1] | 0.33 | 250.541666667 | 17 | 48 | 0 | 1 |
| Path 610 | C00031->C00123:[4->1,4->3,7->1,7->2,7->3] | 0.50 | 405.325581395 | 24 | 43 | 0 | 1 |
| Path 611 | C00031->C00123:[4->3,7->2,7->8,7->9,9->1,9->3,9->5] | 1.00 | 373.773109244 | 40 | 119 | 0 | 2 |
| Path 612 | C00031->C00123:[4->3,4->5,7->8,7->9] | 0.67 | 332.912280702 | 25 | 57 | 0 | 1 |
| Path 613 | C00031->C00123:[2->3,7->2,7->8,7->9,9->1,9->5] | 1.00 | 342.913043478 | 29 | 92 | 0 | 2 |
| Path 614 | C00031->C00123:[9->3] | 0.17 | 283.447368421 | 14 | 38 | 0 | 2 |
| Path 615 | C00031->C00123:[7->8,9->3] | 0.33 | 247.661290323 | 20 | 62 | 0 | 2 |
| Path 616 | C00031->C00123:[7->8,7->9,9->5] | 0.50 | 309.216666667 | 21 | 60 | 0 | 1 |
| Path 617 | C00031->C00123:[4->3,7->8] | 0.33 | 270.122807018 | 20 | 57 | 0 | 2 |
| Path 618 | C00031->C00123:[7->8,7->9,9->3,9->5] | 0.67 | 253.446808511 | 21 | 94 | 0 | 2 |
| Path 619 | C00031->C00123:[7->8,7->9,9->5] | 0.50 | 250.479166667 | 17 | 48 | 0 | 1 |
| Path 620 | C00031->C00123:[1->1,4->2] | 0.33 | 412.068965517 | 19 | 58 | 0 | 1 |
| Path 621 | C00031->C00123:[7->8,9->3] | 0.33 | 316.78125 | 23 | 64 | 0 | 2 |
| Path 622 | C00031->C00123:[7->2,7->8,7->9,9->1,9->3,9->5] | 1.00 | 240.923076923 | 24 | 65 | 0 | 2 |
| Path 623 | C00031->C00123:[2->3,7->2,7->8,7->9,9->1,9->5] | 1.00 | 348.806818182 | 27 | 88 | 0 | 2 |
| Path 624 | C00031->C00123:[4->1,4->3,7->2] | 0.50 | 402.707317073 | 22 | 41 | 0 | 1 |
| Path 625 | C00031->C00123:[7->2,7->3,7->8,9->1] | 0.67 | 274.62962963 | 22 | 54 | 0 | 2 |
| Path 626 | C00031->C00123:[7->2,7->9,9->1,9->5] | 0.67 | 335.127906977 | 24 | 86 | 0 | 1 |
| Path 627 | C00031->C00123:[7->2,9->1,9->3] | 0.50 | 318.661016949 | 24 | 59 | 0 | 1 |
| Path 628 | C00031->C00123:[4->1,4->3,7->1,7->2,7->3,7->8,9->1] | 0.67 | 312.573770492 | 25 | 61 | 0 | 1 |
| Path 629 | C00031->C00123:[2->3,7->2,9->1] | 0.50 | 335.292134831 | 26 | 89 | 0 | 2 |
| Path 630 | C00031->C00123:[7->2,7->9,9->1,9->5] | 0.67 | 217.68627451 | 16 | 51 | 0 | 1 |
| Path 631 | C00031->C00123:[4->3,7->8] | 0.33 | 289.410714286 | 20 | 56 | 0 | 1 |
| Path 632 | C00031->C00123:[7->2,7->8,7->9,9->1,9->3,9->5] | 1.00 | 270.490909091 | 22 | 55 | 0 | 2 |
| Path 633 | C00031->C00123:[7->2,7->8,9->1,9->3] | 0.67 | 253.863157895 | 22 | 95 | 0 | 2 |
| Path 634 | C00031->C00123:[4->1,4->3,7->2,7->8,9->1] | 0.67 | 320.444444444 | 25 | 63 | 0 | 1 |
| Path 635 | C00031->C00123:[7->8,7->9,9->5] | 0.50 | 217.519230769 | 17 | 52 | 0 | 1 |
| Path 636 | C00031->C00123:[4->3,7->2,7->8,9->1] | 0.67 | 289.896551724 | 23 | 58 | 0 | 2 |
| Path 637 | C00031->C00123:[7->2,7->8,9->1,9->3] | 0.67 | 269.030769231 | 23 | 65 | 0 | 2 |
| Path 638 | C00031->C00123:[7->2,7->8,7->9,9->1,9->3,9->5] | 1.00 | 230.671428571 | 24 | 70 | 0 | 2 |
| Path 639 | C00031->C00123:[7->2,7->8,9->1] | 0.50 | 335.127906977 | 24 | 86 | 0 | 1 |
| Path 640 | C00031->C00123:[2->3,7->2,7->9,9->1,9->5] | 0.83 | 338.164835165 | 26 | 91 | 0 | 2 |
| Path 641 | C00031->C00123:[4->1,4->3,7->1,7->3] | 0.33 | 363.358974359 | 21 | 39 | 0 | 2 |
| Path 642 | C00031->C00123:[4->1,4->3,7->2] | 0.50 | 389.573770492 | 28 | 61 | 0 | 2 |
| Path 643 | C00031->C00123:[7->2,7->8,9->1,9->3] | 0.67 | 368.488636364 | 28 | 88 | 0 | 2 |
| Path 644 | C00031->C00123:[7->2,9->1,9->3] | 0.50 | 360.0 | 17 | 32 | 0 | 1 |
| Path 645 | C00031->C00123:[7->3,7->8] | 0.33 | 237.22 | 18 | 50 | 0 | 2 |
| Path 646 | C00031->C00123:[9->3] | 0.17 | 343.0 | 13 | 31 | 0 | 2 |
| Path 647 | C00031->C00123:[4->1,4->3] | 0.33 | 415.390243902 | 23 | 41 | 0 | 2 |
| Path 648 | C00031->C00123:[4->3,7->2,9->1] | 0.50 | 300.189655172 | 22 | 58 | 0 | 1 |
| Path 649 | C00031->C00123:[5->3,7->2,7->8,9->1] | 0.67 | 298.901408451 | 26 | 71 | 0 | 2 |
| Path 650 | C00031->C00123:[4->1,4->3,7->2] | 0.50 | 338.963636364 | 23 | 55 | 0 | 1 |
| Path 651 | C00031->C00123:[7->8,9->3] | 0.33 | 218.316666667 | 18 | 60 | 0 | 2 |
| Path 652 | C00031->C00123:[2->3,7->2,7->8,9->1] | 0.67 | 327.965909091 | 29 | 88 | 0 | 2 |
| Path 653 | C00031->C00123:[4->1,4->3,7->2,7->8] | 0.67 | 332.912280702 | 25 | 57 | 0 | 1 |
| Path 654 | C00031->C00123:[7->2,7->8,9->1,9->3] | 0.67 | 350.16091954 | 27 | 87 | 0 | 2 |
| Path 655 | C00031->C00123:[4->1,4->3,7->2] | 0.50 | 438.75308642 | 29 | 81 | 0 | 2 |
| Path 656 | C00031->C00123:[4->1,4->2,4->3] | 0.50 | 440.865853659 | 28 | 82 | 0 | 2 |
| Path 657 | C00031->C00123:[7->2,7->8,9->1,9->3] | 0.67 | 266.683333333 | 23 | 60 | 0 | 2 |
| Path 658 | C00031->C00123:[4->3,7->8,7->9,9->5] | 0.67 | 272.706896552 | 21 | 58 | 0 | 2 |
| Path 659 | C00031->C00123:[4->3,7->2,9->1] | 0.50 | 288.345454545 | 21 | 55 | 0 | 1 |
| Path 660 | C00031->C00123:[4->1,4->3,7->2,7->8,9->1] | 0.67 | 325.838235294 | 27 | 68 | 0 | 1 |
| Path 661 | C00031->C00123:[7->2,7->8,7->9,9->1,9->3,9->5] | 1.00 | 344.457446809 | 30 | 94 | 0 | 2 |
| Path 662 | C00031->C00123:[4->1,4->3,7->2] | 0.50 | 398.955555556 | 24 | 45 | 0 | 1 |
| Path 663 | C00031->C00123:[4->3,7->2,9->1] | 0.50 | 385.513513514 | 20 | 37 | 0 | 1 |
| Path 664 | C00031->C00123:[1->1,4->2,4->3] | 0.50 | 430.661764706 | 24 | 68 | 0 | 1 |
| Path 665 | C00031->C00123:[7->2,9->1,9->3] | 0.50 | 355.8 | 21 | 40 | 0 | 1 |
| Path 666 | C00031->C00123:[7->2,7->8,9->1,9->3] | 0.67 | 318.369230769 | 24 | 65 | 0 | 2 |
| Path 667 | C00031->C00123:[2->3,7->2,7->8,9->1] | 0.67 | 347.988505747 | 26 | 87 | 0 | 2 |
| Path 668 | C00031->C00123:[7->8,9->3] | 0.33 | 274.769230769 | 19 | 52 | 0 | 2 |
| Path 669 | C00031->C00123:[7->8,9->3] | 0.33 | 359.264705882 | 18 | 34 | 0 | 2 |
| Path 670 | C00031->C00123:[4->1,4->3,4->5,7->2,7->8,7->9,9->1,9->5] | 1.00 | 335.941176471 | 36 | 102 | 0 | 2 |
| Path 671 | C00031->C00123:[4->1,4->2,4->3,4->8] | 0.67 | 469.090909091 | 26 | 66 | 0 | 1 |
| Path 672 | C00031->C00123:[2->3,7->2,7->8,9->1] | 0.67 | 348.24137931 | 27 | 87 | 0 | 2 |
| Path 673 | C00031->C00123:[4->3,7->2,7->8,7->9,9->1,9->3,9->5] | 1.00 | 343.00877193 | 40 | 114 | 0 | 2 |
| Path 674 | C00031->C00123:[7->2,7->8,7->9,9->1,9->3,9->5] | 1.00 | 256.815533981 | 26 | 103 | 0 | 2 |
| Path 675 | C00031->C00123:[4->1,4->3,7->2] | 0.50 | 429.80952381 | 33 | 84 | 0 | 2 |
| Path 676 | C00031->C00123:[2->3,7->8,7->9,9->5] | 0.67 | 352.912087912 | 25 | 91 | 0 | 2 |
| Path 677 | C00031->C00123:[4->1,4->2,4->3] | 0.50 | 405.978723404 | 28 | 94 | 0 | 1 |
| Path 678 | C00031->C00123:[7->3,7->8,7->9,9->5] | 0.67 | 240.803921569 | 19 | 51 | 0 | 2 |
| Path 679 | C00031->C00123:[7->8,7->9,9->3,9->5] | 0.67 | 267.722222222 | 21 | 54 | 0 | 2 |
| Path 680 | C00031->C00123:[4->1,4->3,7->1,7->3] | 0.33 | 328.407407407 | 22 | 54 | 0 | 1 |
| Path 681 | C00031->C00123:[4->1,4->3,4->5,7->2,7->9,9->1,9->5] | 0.83 | 299.223880597 | 25 | 67 | 0 | 1 |
| Path 682 | C00031->C00123:[4->3,7->2,7->9,9->1,9->3,9->5] | 0.83 | 368.063063063 | 38 | 111 | 0 | 2 |
| Path 683 | C00031->C00123:[2->3,7->2,7->9,9->1,9->5] | 0.83 | 299.802325581 | 25 | 86 | 0 | 2 |
| Path 684 | C00031->C00123:[2->3,7->2,7->8,7->9,9->1,9->5] | 1.00 | 347.97979798 | 29 | 99 | 0 | 2 |
| Path 685 | C00031->C00123:[7->8,9->3] | 0.33 | 232.714285714 | 19 | 56 | 0 | 2 |
| Path 686 | C00031->C00123:[4->3,7->2,7->9,9->1,9->5] | 0.83 | 263.619047619 | 22 | 63 | 0 | 2 |
| Path 687 | C00031->C00123:[4->1,4->3,7->2] | 0.50 | 379.135135135 | 20 | 37 | 0 | 1 |
| Path 688 | C00031->C00123:[2->3,7->8,7->9,9->5] | 0.67 | 352.0 | 24 | 86 | 0 | 2 |
| Path 689 | C00031->C00123:[2->3,7->8,7->9,9->5] | 0.67 | 342.065934066 | 28 | 91 | 0 | 2 |
| Path 690 | C00031->C00123:[4->1,4->2,4->3] | 0.50 | 397.634146341 | 31 | 82 | 0 | 2 |
| Path 691 | C00031->C00123:[2->3,7->2,7->8,9->1] | 0.67 | 353.577777778 | 24 | 90 | 0 | 2 |
| Path 692 | C00031->C00123:[7->3,7->8] | 0.33 | 244.583333333 | 17 | 48 | 0 | 1 |
| Path 693 | C00031->C00123:[7->3,9->8] | 0.33 | 263.215686275 | 20 | 51 | 0 | 1 |
| Path 694 | C00031->C00123:[4->1,4->3,7->2] | 0.50 | 426.06097561 | 32 | 82 | 0 | 2 |
| Path 695 | C00031->C00123:[5->8,7->3] | 0.33 | 306.698412698 | 23 | 63 | 0 | 2 |
| Path 696 | C00031->C00123:[4->3,7->2,9->1] | 0.50 | 381.666666667 | 20 | 39 | 0 | 1 |
| Path 697 | C00031->C00123:[7->8,7->9,9->3,9->5] | 0.67 | 350.16091954 | 27 | 87 | 0 | 2 |
| Path 698 | C00031->C00123:[7->2,7->8,9->1,9->3] | 0.67 | 342.537313433 | 26 | 67 | 0 | 2 |
| Path 699 | C00031->C00123:[4->3,7->2,7->8,7->9,9->1,9->5] | 1.00 | 261.476190476 | 24 | 63 | 0 | 2 |
| Path 700 | C00031->C00123:[7->2,7->9,9->1,9->3,9->5] | 0.83 | 269.31147541 | 21 | 61 | 0 | 2 |
| Path 701 | C00031->C00123:[7->2,7->9,9->1,9->3,9->5] | 0.83 | 248.636363636 | 22 | 99 | 0 | 2 |
| Path 702 | C00031->C00123:[7->2,7->8,9->1,9->3] | 0.67 | 350.347826087 | 28 | 69 | 0 | 2 |
| Path 703 | C00031->C00123:[4->1,4->3,7->2] | 0.50 | 401.303797468 | 32 | 79 | 0 | 2 |
| Path 704 | C00031->C00123:[5->3,7->2,7->9,9->1,9->5] | 0.83 | 267.861111111 | 23 | 72 | 0 | 2 |
| Path 705 | C00031->C00123:[4->1,4->3,5->2,5->8] | 0.67 | 356.24 | 29 | 75 | 0 | 1 |
| Path 706 | C00031->C00123:[7->2,7->8,9->1,9->3] | 0.67 | 241.761904762 | 21 | 63 | 0 | 2 |
| Path 707 | C00031->C00123:[2->3] | 0.17 | 411.607142857 | 16 | 56 | 0 | 2 |
| Path 708 | C00031->C00123:[7->2,7->8,9->1,9->3] | 0.67 | 251.0625 | 22 | 64 | 0 | 2 |
| Path 709 | C00031->C00123:[7->2,7->8,7->9,9->1,9->3,9->5] | 1.00 | 278.828125 | 24 | 64 | 0 | 2 |
| Path 710 | C00031->C00123:[4->1,4->3,4->5,7->2,7->9,9->1,9->5] | 0.83 | 379.225352113 | 31 | 71 | 0 | 1 |
| Path 711 | C00031->C00123:[7->8,7->9,9->5] | 0.50 | 221.226415094 | 18 | 53 | 0 | 1 |
| Path 712 | C00031->C00123:[7->8,7->9,9->3,9->5] | 0.67 | 267.245283019 | 20 | 53 | 0 | 2 |
| Path 713 | C00031->C00123:[4->1,4->2,4->3] | 0.50 | 401.657534247 | 28 | 73 | 0 | 2 |
| Path 714 | C00031->C00123:[7->2,9->1,9->3] | 0.50 | 356.988095238 | 25 | 84 | 0 | 1 |
| Path 715 | C00031->C00123:[4->3,7->2,7->9,9->1,9->3,9->5] | 0.83 | 371.224137931 | 37 | 116 | 0 | 2 |
| Path 716 | C00031->C00123:[4->1,4->3] | 0.33 | 379.857142857 | 25 | 56 | 0 | 2 |
| Path 717 | C00031->C00123:[2->3,7->8] | 0.33 | 317.976744186 | 25 | 86 | 0 | 2 |
| Path 718 | C00031->C00123:[4->1,4->3,7->2] | 0.50 | 399.357142857 | 33 | 84 | 0 | 2 |
| Path 719 | C00031->C00123:[4->1,4->3,7->2] | 0.50 | 400.126760563 | 25 | 71 | 0 | 1 |
| Path 720 | C00031->C00123:[4->1,4->3,4->5,7->2,7->9,9->1,9->5] | 0.83 | 333.576923077 | 34 | 104 | 0 | 2 |
| Path 721 | C00031->C00123:[7->2,7->8,7->9,9->1,9->3,9->5] | 1.00 | 240.084745763 | 22 | 59 | 0 | 2 |
| Path 722 | C00031->C00123:[7->2,7->8,9->1,9->3] | 0.67 | 266.0 | 23 | 96 | 0 | 2 |
| Path 723 | C00031->C00123:[7->8,9->3] | 0.33 | 301.863636364 | 19 | 44 | 0 | 2 |
| Path 724 | C00031->C00123:[4->1,4->3,7->2] | 0.50 | 440.6375 | 31 | 80 | 0 | 2 |
| Path 725 | C00031->C00123:[4->3,7->2,7->9,9->1,9->3,9->5] | 0.83 | 335.009345794 | 38 | 107 | 0 | 2 |
| Path 726 | C00031->C00123:[2->3,7->2,7->8,9->1] | 0.67 | 353.77173913 | 29 | 92 | 0 | 2 |
| Path 727 | C00031->C00123:[5->3,7->2,7->8,9->1] | 0.67 | 366.257731959 | 29 | 97 | 0 | 2 |
| Path 728 | C00031->C00123:[4->3,7->2,7->8,7->9,9->1,9->5] | 1.00 | 342.372727273 | 38 | 110 | 0 | 2 |
| Path 729 | C00031->C00123:[7->8,7->9,9->5] | 0.50 | 346.87804878 | 24 | 82 | 0 | 1 |
| Path 730 | C00031->C00123:[7->2,7->9,9->1,9->5] | 0.67 | 252.037735849 | 18 | 53 | 0 | 1 |
| Path 731 | C00031->C00123:[7->8,7->9,9->3,9->5] | 0.67 | 245.096153846 | 19 | 52 | 0 | 2 |
| Path 732 | C00031->C00123:[4->1,4->3,7->2] | 0.50 | 401.3 | 31 | 80 | 0 | 2 |
| Path 733 | C00031->C00123:[7->2,7->8,9->1] | 0.50 | 224.152173913 | 15 | 46 | 0 | 1 |
| Path 734 | C00031->C00123:[7->8,9->3] | 0.33 | 290.418918919 | 24 | 74 | 0 | 2 |
| Path 735 | C00031->C00123:[4->3,7->2,7->8,9->1] | 0.67 | 320.348484848 | 26 | 66 | 0 | 1 |
| Path 736 | C00031->C00123:[5->3,7->8,7->9,9->5] | 0.67 | 353.304347826 | 26 | 92 | 0 | 2 |
| Path 737 | C00031->C00123:[4->1,4->3,7->2] | 0.50 | 387.784615385 | 30 | 65 | 0 | 2 |
| Path 738 | C00031->C00123:[4->1,4->3,7->2,7->8] | 0.67 | 326.672131148 | 25 | 61 | 0 | 1 |
| Path 739 | C00031->C00123:[4->1,4->3] | 0.33 | 393.09375 | 19 | 32 | 0 | 2 |
| Path 740 | C00031->C00123:[4->1,4->3,7->2,7->8,9->1] | 0.67 | 421.177777778 | 24 | 45 | 0 | 1 |
| Path 741 | C00031->C00123:[7->2,7->8,9->1,9->3] | 0.67 | 296.907407407 | 27 | 108 | 0 | 2 |
| Path 742 | C00031->C00123:[7->2,7->8,9->1] | 0.50 | 217.68627451 | 16 | 51 | 0 | 1 |
| Path 743 | C00031->C00123:[2->3,7->2,7->8,9->1] | 0.67 | 342.611111111 | 27 | 90 | 0 | 2 |
| Path 744 | C00031->C00123:[7->2,9->1,9->3] | 0.50 | 271.86 | 18 | 50 | 0 | 1 |
| Path 745 | C00031->C00123:[4->1,4->3,7->2] | 0.50 | 452.9 | 25 | 40 | 0 | 1 |
| Path 746 | C00031->C00123:[2->3,7->8] | 0.33 | 437.382352941 | 23 | 68 | 0 | 2 |
| Path 747 | C00031->C00123:[7->8,7->9,9->5] | 0.50 | 261.8125 | 17 | 48 | 0 | 1 |
| Path 748 | C00031->C00123:[2->3,7->2,7->8,9->1] | 0.67 | 348.88372093 | 26 | 86 | 0 | 2 |
| Path 749 | C00031->C00123:[7->2,9->1,9->3] | 0.50 | 266.236363636 | 19 | 55 | 0 | 2 |
| Path 750 | C00031->C00123:[7->2,7->8,9->1] | 0.50 | 212.315789474 | 18 | 57 | 0 | 1 |
| Path 751 | C00031->C00123:[7->2,7->8,7->9,9->1,9->3,9->5] | 1.00 | 225.904761905 | 21 | 63 | 0 | 2 |
| Path 752 | C00031->C00123:[4->3,7->2,7->8,7->9,9->1,9->3,9->5] | 1.00 | 361.553571429 | 39 | 112 | 0 | 2 |
| Path 753 | C00031->C00123:[5->3,7->2,7->8,9->1] | 0.67 | 370.612903226 | 27 | 93 | 0 | 2 |
| Path 754 | C00031->C00123:[7->2,9->1,9->3] | 0.50 | 292.894736842 | 22 | 57 | 0 | 1 |
| Path 755 | C00031->C00123:[2->3,7->2,7->8,7->9,9->1,9->5] | 1.00 | 313.045454545 | 27 | 88 | 0 | 2 |
| Path 756 | C00031->C00123:[7->2,7->8,9->1,9->3] | 0.67 | 250.396825397 | 21 | 63 | 0 | 2 |
| Path 757 | C00031->C00123:[2->3,7->2,9->1] | 0.50 | 369.831578947 | 27 | 95 | 0 | 1 |
| Path 758 | C00031->C00123:[2->3,7->2,7->8,9->1] | 0.67 | 360.409090909 | 28 | 88 | 0 | 2 |
| Path 759 | C00031->C00123:[7->2,7->3,9->1] | 0.50 | 372.121212121 | 19 | 33 | 0 | 1 |
| Path 760 | C00031->C00123:[4->3,4->5,7->8,7->9,9->5] | 0.67 | 307.610169492 | 23 | 59 | 0 | 1 |
| Path 761 | C00031->C00123:[4->1,4->2,4->3] | 0.50 | 441.358024691 | 29 | 81 | 0 | 2 |
| Path 762 | C00031->C00123:[7->3,7->8,7->9,9->5] | 0.67 | 241.807692308 | 20 | 52 | 0 | 2 |
| Path 763 | C00031->C00123:[2->3,7->2,7->8,9->1] | 0.67 | 321.392857143 | 27 | 84 | 0 | 2 |
| Path 764 | C00031->C00123:[4->3,7->2,7->8,7->9,9->1,9->3,9->5] | 1.00 | 323.821052632 | 37 | 95 | 0 | 2 |
| Path 765 | C00031->C00123:[2->3,7->8] | 0.33 | 341.741573034 | 26 | 89 | 0 | 2 |
| Path 766 | C00031->C00123:[7->8,7->9,9->3,9->5] | 0.67 | 222.774193548 | 20 | 62 | 0 | 2 |
| Path 767 | C00031->C00123:[4->1,4->3,7->1,7->3] | 0.33 | 480.85 | 24 | 40 | 0 | 1 |
| Path 768 | C00031->C00123:[2->3,7->8] | 0.33 | 354.825581395 | 26 | 86 | 0 | 2 |
| Path 769 | C00031->C00123:[7->2,7->8,7->9,9->1,9->3,9->5] | 1.00 | 281.896551724 | 22 | 58 | 0 | 2 |
| Path 770 | C00031->C00123:[4->1,4->3,7->2] | 0.50 | 437.875 | 32 | 88 | 0 | 2 |
| Path 771 | C00031->C00123:[4->1,4->3,7->2] | 0.50 | 403.48 | 30 | 75 | 0 | 2 |
| Path 772 | C00031->C00123:[4->3,7->2,7->9,9->1,9->5] | 0.83 | 271.1625 | 25 | 80 | 0 | 1 |
| Path 773 | C00031->C00123:[4->1,4->3] | 0.33 | 385.174603175 | 28 | 63 | 0 | 2 |
| Path 774 | C00031->C00123:[4->1,4->3,7->2,9->1] | 0.50 | 371.873015873 | 27 | 63 | 0 | 1 |
| Path 775 | C00031->C00123:[9->3] | 0.17 | 209.436363636 | 14 | 55 | 0 | 2 |
| Path 776 | C00031->C00123:[5->3,7->2,7->8,7->9,9->1,9->5] | 1.00 | 354.021505376 | 27 | 93 | 0 | 2 |
| Path 777 | C00031->C00123:[2->3,7->8] | 0.33 | 314.851851852 | 24 | 81 | 0 | 2 |
| Path 778 | C00031->C00123:[2->3] | 0.17 | 364.350877193 | 16 | 57 | 0 | 2 |
| Path 779 | C00031->C00123:[2->3,7->8,7->9,9->5] | 0.67 | 348.24137931 | 27 | 87 | 0 | 2 |
| Path 780 | C00031->C00123:[4->3,7->2,7->8,7->9,9->1,9->3,9->5] | 1.00 | 370.614035088 | 40 | 114 | 0 | 2 |
| Path 781 | C00031->C00123:[4->1,4->3,7->2] | 0.50 | 445.625 | 25 | 40 | 0 | 1 |
| Path 782 | C00031->C00123:[2->3,7->8] | 0.33 | 380.892307692 | 26 | 65 | 0 | 2 |
| Path 783 | C00031->C00123:[2->3,7->2,7->8,9->1] | 0.67 | 381.356435644 | 32 | 101 | 0 | 2 |
| Path 784 | C00031->C00123:[2->3,7->8] | 0.33 | 311.048780488 | 26 | 82 | 0 | 2 |
| Path 785 | C00031->C00123:[4->3,7->2,7->8,9->1] | 0.67 | 289.842105263 | 22 | 57 | 0 | 2 |
| Path 786 | C00031->C00123:[2->3,5->8] | 0.33 | 362.744444444 | 26 | 90 | 0 | 2 |
| Path 787 | C00031->C00123:[7->2,7->8,9->1,9->3] | 0.67 | 269.393939394 | 24 | 66 | 0 | 2 |
| Path 788 | C00031->C00123:[5->3,7->2,7->8,9->1] | 0.67 | 292.855072464 | 24 | 69 | 0 | 2 |
| Path 789 | C00031->C00123:[4->3,7->2,7->9,9->1,9->3,9->5] | 0.83 | 371.181818182 | 35 | 110 | 0 | 2 |
| Path 790 | C00031->C00123:[7->8,7->9,9->3,9->5] | 0.67 | 279.232142857 | 20 | 56 | 0 | 2 |
| Path 791 | C00031->C00123:[4->3,7->2,7->9,9->1,9->5] | 0.83 | 371.58490566 | 33 | 106 | 0 | 2 |
| Path 792 | C00031->C00123:[7->8,7->9,9->3,9->5] | 0.67 | 250.396825397 | 21 | 63 | 0 | 2 |
| Path 793 | C00031->C00123:[4->3,7->8] | 0.33 | 386.305555556 | 19 | 36 | 0 | 1 |
| Path 794 | C00031->C00123:[7->2,7->9,9->1,9->3,9->5] | 0.83 | 243.617647059 | 22 | 68 | 0 | 2 |
| Path 795 | C00031->C00123:[4->1,4->3] | 0.33 | 495.775 | 30 | 80 | 0 | 1 |
| Path 796 | C00031->C00123:[7->2,7->8,9->1,9->3] | 0.67 | 340.450704225 | 27 | 71 | 0 | 2 |
| Path 797 | C00031->C00123:[2->3,7->2,7->8,9->1] | 0.67 | 330.150537634 | 30 | 93 | 0 | 2 |
| Path 798 | C00031->C00123:[4->3,7->2,7->9,9->1,9->5] | 0.83 | 337.264705882 | 34 | 102 | 0 | 2 |
| Path 799 | C00031->C00123:[4->1,4->3,4->5,7->1,7->2,7->3,7->5,7->8,7->9,9->1,9->5] | 1.00 | 308.093333333 | 32 | 75 | 0 | 2 |
| Path 800 | C00031->C00123:[7->2,7->8,9->1,9->3] | 0.67 | 350.825581395 | 26 | 86 | 0 | 2 |
| Path 801 | C00031->C00123:[2->3,7->2,7->8,9->1] | 0.67 | 328.289156627 | 26 | 83 | 0 | 2 |
| Path 802 | C00031->C00123:[2->3,7->2,7->8,9->1] | 0.67 | 381.574257426 | 33 | 101 | 0 | 2 |
| Path 803 | C00031->C00123:[7->2,9->1,9->3] | 0.50 | 250.367346939 | 17 | 49 | 0 | 1 |
| Path 804 | C00031->C00123:[7->2,7->8,7->9,9->1,9->3,9->5] | 1.00 | 350.954545455 | 28 | 88 | 0 | 2 |
| Path 805 | C00031->C00123:[4->1,4->3,7->2] | 0.50 | 437.935064935 | 31 | 77 | 0 | 2 |
| Path 806 | C00031->C00123:[7->2,7->3,9->1] | 0.50 | 315.941176471 | 18 | 34 | 0 | 1 |
| Path 807 | C00031->C00123:[2->3,7->2,7->8,9->1] | 0.67 | 382.46 | 32 | 100 | 0 | 2 |
| Path 808 | C00031->C00123:[7->2,9->1,9->3] | 0.50 | 436.402985075 | 25 | 67 | 0 | 1 |
| Path 809 | C00031->C00123:[2->3,7->2,7->8,9->1] | 0.67 | 370.413043478 | 26 | 92 | 0 | 2 |
| Path 810 | C00031->C00123:[7->2,7->8,9->1,9->3] | 0.67 | 327.651515152 | 25 | 66 | 0 | 2 |
| Path 811 | C00031->C00123:[7->2,9->1,9->3] | 0.50 | 290.263157895 | 22 | 57 | 0 | 1 |
| Path 812 | C00031->C00123:[4->3,7->2,7->8,9->1] | 0.67 | 259.964285714 | 21 | 56 | 0 | 2 |
| Path 813 | C00031->C00123:[7->2,7->8,9->1] | 0.50 | 241.773584906 | 18 | 53 | 0 | 1 |
| Path 814 | C00031->C00123:[4->3,7->2,7->9,9->1,9->5] | 0.83 | 360.457142857 | 29 | 70 | 0 | 1 |
| Path 815 | C00031->C00123:[7->8,9->3] | 0.33 | 264.307692308 | 19 | 52 | 0 | 2 |
| Path 816 | C00031->C00123:[7->2,7->8,7->9,9->1,9->3,9->5] | 1.00 | 297.625 | 27 | 64 | 0 | 2 |
| Path 817 | C00031->C00123:[7->8,7->9,9->3,9->5] | 0.67 | 242.5625 | 22 | 64 | 0 | 2 |
| Path 818 | C00031->C00123:[4->3,4->8] | 0.33 | 435.492307692 | 23 | 65 | 0 | 1 |
| Path 819 | C00031->C00123:[4->1,4->3,9->2,9->8] | 0.67 | 319.59375 | 26 | 64 | 0 | 1 |
| Path 820 | C00031->C00123:[7->2,9->1,9->3] | 0.50 | 229.629032258 | 20 | 62 | 0 | 2 |
| Path 821 | C00031->C00123:[4->1,4->2,4->3] | 0.50 | 439.884615385 | 29 | 78 | 0 | 2 |
| Path 822 | C00031->C00123:[7->2,7->8,7->9,9->1,9->3,9->5] | 1.00 | 227.927536232 | 23 | 69 | 0 | 2 |
| Path 823 | C00031->C00123:[5->1,9->2,9->8] | 0.50 | 353.738095238 | 26 | 84 | 0 | 1 |
| Path 824 | C00031->C00123:[4->3,4->5,7->3,7->5,7->8,7->9,9->5] | 0.67 | 312.573770492 | 25 | 61 | 0 | 1 |
| Path 825 | C00031->C00123:[2->3,7->2,7->8,9->1] | 0.67 | 324.04494382 | 28 | 89 | 0 | 2 |
| Path 826 | C00031->C00123:[2->3,7->8,7->9,9->5] | 0.67 | 308.353658537 | 25 | 82 | 0 | 2 |
| Path 827 | C00031->C00123:[7->8,7->9,9->3,9->5] | 0.67 | 221.62295082 | 19 | 61 | 0 | 2 |
| Path 828 | C00031->C00123:[4->1,4->3,7->2] | 0.50 | 446.17721519 | 29 | 79 | 0 | 2 |
| Path 829 | C00031->C00123:[7->2,7->8,9->1,9->3] | 0.67 | 311.909090909 | 27 | 77 | 0 | 2 |
| Path 830 | C00031->C00123:[9->3] | 0.17 | 240.149425287 | 15 | 87 | 0 | 2 |
| Path 831 | C00031->C00123:[2->3,7->2,7->8,9->1] | 0.67 | 367.913043478 | 28 | 92 | 0 | 2 |
| Path 832 | C00031->C00123:[2->3,5->8] | 0.33 | 378.010204082 | 30 | 98 | 0 | 2 |
| Path 833 | C00031->C00123:[2->3,7->2,7->8,9->1] | 0.67 | 327.869047619 | 27 | 84 | 0 | 2 |
| Path 834 | C00031->C00123:[2->3,7->2,7->8,9->1] | 0.67 | 359.651685393 | 29 | 89 | 0 | 2 |
| Path 835 | C00031->C00123:[2->3,7->2,7->8,9->1] | 0.67 | 345.670103093 | 33 | 97 | 0 | 2 |
| Path 836 | C00031->C00123:[4->3,7->2,9->1] | 0.50 | 370.361111111 | 19 | 36 | 0 | 1 |
| Path 837 | C00031->C00123:[4->1,4->2,4->3,4->8] | 0.67 | 451.2 | 29 | 75 | 0 | 1 |
| Path 838 | C00031->C00123:[4->1,4->2,4->3] | 0.50 | 445.558441558 | 27 | 77 | 0 | 2 |
| Path 839 | C00031->C00123:[7->2,7->8,9->1] | 0.50 | 434.046153846 | 24 | 65 | 0 | 1 |
| Path 840 | C00031->C00123:[4->1,4->2,4->3] | 0.50 | 441.23943662 | 28 | 71 | 0 | 2 |
| Path 841 | C00031->C00123:[4->3,7->2,7->8,9->1] | 0.67 | 280.298245614 | 22 | 57 | 0 | 2 |
| Path 842 | C00031->C00123:[4->3,7->8] | 0.33 | 256.388888889 | 19 | 54 | 0 | 2 |
| Path 843 | C00031->C00123:[4->1,4->2,4->3,4->8] | 0.67 | 450.716216216 | 28 | 74 | 0 | 1 |
| Path 844 | C00031->C00123:[2->3,5->8] | 0.33 | 321.627906977 | 27 | 86 | 0 | 2 |
| Path 845 | C00031->C00123:[4->3,7->8,7->9,9->5] | 0.67 | 259.964285714 | 21 | 56 | 0 | 2 |
| Path 846 | C00031->C00123:[5->3,7->2,7->8,7->9,9->1,9->5] | 1.00 | 347.666666667 | 29 | 99 | 0 | 2 |
| Path 847 | C00031->C00123:[7->2,9->1,9->3] | 0.50 | 343.258823529 | 25 | 85 | 0 | 2 |
| Path 848 | C00031->C00123:[7->2,9->1,9->3] | 0.50 | 236.1875 | 16 | 48 | 0 | 1 |
| Path 849 | C00031->C00123:[2->3,7->2,7->8,7->9,9->1,9->5] | 1.00 | 352.781609195 | 25 | 87 | 0 | 2 |
| Path 850 | C00031->C00123:[4->3] | 0.17 | 354.05 | 10 | 20 | 0 | 2 |
| Path 851 | C00031->C00123:[4->3,7->8] | 0.33 | 277.745454545 | 19 | 55 | 0 | 1 |
| Path 852 | C00031->C00123:[2->3,7->2,7->8,9->1] | 0.67 | 304.674698795 | 27 | 83 | 0 | 2 |
| Path 853 | C00031->C00123:[7->2,7->8,7->9,9->1,9->3,9->5] | 1.00 | 280.381818182 | 22 | 55 | 0 | 2 |
| Path 854 | C00031->C00123:[2->3,7->2,7->8,9->1] | 0.67 | 363.454545455 | 26 | 88 | 0 | 2 |
| Path 855 | C00031->C00123:[7->2,9->1,9->3] | 0.50 | 294.326923077 | 20 | 52 | 0 | 1 |
| Path 856 | C00031->C00123:[7->8,9->3] | 0.33 | 319.125 | 23 | 64 | 0 | 2 |
| Path 857 | C00031->C00123:[4->1,4->3,9->2,9->8] | 0.67 | 322.169230769 | 27 | 65 | 0 | 1 |
| Path 858 | C00031->C00123:[7->2,7->3,9->1] | 0.50 | 297.303030303 | 17 | 33 | 0 | 1 |
| Path 859 | C00031->C00123:[4->1,4->3,7->2] | 0.50 | 438.5 | 23 | 36 | 0 | 1 |
| Path 860 | C00031->C00123:[7->2,7->8,9->1] | 0.50 | 353.182926829 | 24 | 82 | 0 | 1 |
| Path 861 | C00031->C00123:[7->2,7->8,9->1,9->3] | 0.67 | 366.021505376 | 31 | 93 | 0 | 2 |
| Path 862 | C00031->C00123:[4->3,7->2,7->9,9->1,9->5] | 0.83 | 368.345794393 | 36 | 107 | 0 | 2 |
| Path 863 | C00031->C00123:[7->2,7->8,9->1,9->3] | 0.67 | 308.5 | 22 | 58 | 0 | 2 |
| Path 864 | C00031->C00123:[4->3,4->5,7->8,7->9] | 0.67 | 326.672131148 | 25 | 61 | 0 | 1 |
| Path 865 | C00031->C00123:[7->2,9->1,9->3] | 0.50 | 250.875 | 20 | 56 | 0 | 1 |
| Path 866 | C00031->C00123:[2->3,7->2,7->8,7->9,9->1,9->5] | 1.00 | 302.255555556 | 30 | 90 | 0 | 2 |
| Path 867 | C00031->C00123:[9->3] | 0.17 | 346.571428571 | 13 | 28 | 0 | 2 |
| Path 868 | C00031->C00123:[2->3,7->2,7->8,9->1] | 0.67 | 324.047058824 | 29 | 85 | 0 | 2 |
| Path 869 | C00031->C00123:[7->8,7->9,9->3,9->5] | 0.67 | 253.863157895 | 22 | 95 | 0 | 2 |
| Path 870 | C00031->C00123:[2->3,7->8] | 0.33 | 352.831460674 | 23 | 89 | 0 | 2 |
| Path 871 | C00031->C00123:[7->2,7->8,9->1,9->3] | 0.67 | 341.808823529 | 27 | 68 | 0 | 2 |
| Path 872 | C00031->C00123:[4->1,4->3,7->2,7->8,9->1] | 0.67 | 429.615384615 | 28 | 52 | 0 | 1 |
| Path 873 | C00031->C00123:[7->2,7->3,9->1] | 0.50 | 246.87755102 | 18 | 49 | 0 | 1 |
| Path 874 | C00031->C00123:[7->2,9->1] | 0.33 | 265.4 | 14 | 30 | 0 | 1 |
| Path 875 | C00031->C00123:[7->8,9->3] | 0.33 | 266.671875 | 22 | 64 | 0 | 2 |
| Path 876 | C00031->C00123:[2->3,7->2,7->8,9->1] | 0.67 | 308.543209877 | 24 | 81 | 0 | 2 |
| Path 877 | C00031->C00123:[7->2,7->8,9->1] | 0.50 | 252.037735849 | 18 | 53 | 0 | 1 |
| Path 878 | C00031->C00123:[4->1,4->3,7->2] | 0.50 | 389.5 | 21 | 40 | 0 | 1 |
| Path 879 | C00031->C00123:[4->3,4->5,7->3,7->5] | 0.33 | 395.894736842 | 21 | 38 | 0 | 1 |
| Path 880 | C00031->C00123:[7->8,7->9,9->5] | 0.50 | 224.152173913 | 15 | 46 | 0 | 1 |
| Path 881 | C00031->C00123:[4->1,4->3,4->5,7->2,7->9,9->1,9->5] | 0.83 | 367.596330275 | 34 | 109 | 0 | 2 |
| Path 882 | C00031->C00123:[2->3,7->2,7->8,9->1] | 0.67 | 346.21875 | 32 | 96 | 0 | 2 |
| Path 883 | C00031->C00123:[4->1,4->2,4->3] | 0.50 | 430.716049383 | 31 | 81 | 0 | 2 |
| Path 884 | C00031->C00123:[4->1,4->3,5->2,5->8] | 0.67 | 347.591549296 | 27 | 71 | 0 | 1 |
| Path 885 | C00031->C00123:[4->1,4->3] | 0.33 | 386.847457627 | 26 | 59 | 0 | 2 |
| Path 886 | C00031->C00123:[7->2,7->8,9->1,9->3] | 0.67 | 290.184210526 | 26 | 76 | 0 | 2 |
| Path 887 | C00031->C00123:[5->3,7->8,7->9,9->5] | 0.67 | 353.967032967 | 25 | 91 | 0 | 2 |
| Path 888 | C00031->C00123:[1->1,4->1,4->2,4->3,4->8] | 0.67 | 446.08974359 | 30 | 78 | 0 | 1 |
| Path 889 | C00031->C00123:[7->8,9->3] | 0.33 | 297.0 | 21 | 57 | 0 | 2 |
| Path 890 | C00031->C00123:[4->3,4->5] | 0.33 | 422.703703704 | 17 | 27 | 0 | 1 |
| Path 891 | C00031->C00123:[4->1,4->3] | 0.33 | 426.611111111 | 21 | 36 | 0 | 1 |
| Path 892 | C00031->C00123:[7->2,7->8,9->1,9->3] | 0.67 | 308.237288136 | 23 | 59 | 0 | 2 |
| Path 893 | C00031->C00123:[4->1,4->3,4->5,7->2,7->8,7->9,9->1,9->5] | 1.00 | 361.342342342 | 39 | 111 | 0 | 2 |
| Path 894 | C00031->C00123:[7->8,9->3] | 0.33 | 270.105263158 | 22 | 95 | 0 | 2 |
| Path 895 | C00031->C00123:[7->8,9->3] | 0.33 | 287.150943396 | 20 | 53 | 0 | 2 |
| Path 896 | C00031->C00123:[2->3,7->8] | 0.33 | 310.764705882 | 24 | 85 | 0 | 2 |
| Path 897 | C00031->C00123:[7->8] | 0.17 | 347.379310345 | 15 | 29 | 0 | 1 |
| Path 898 | C00031->C00123:[5->3,7->2,7->9,9->1,9->5] | 0.83 | 343.770833333 | 26 | 96 | 0 | 2 |
| Path 899 | C00031->C00123:[7->2,7->8,9->1,9->3] | 0.67 | 296.871559633 | 28 | 109 | 0 | 2 |
| Path 900 | C00031->C00123:[2->3,7->2,7->8,9->1] | 0.67 | 324.397727273 | 27 | 88 | 0 | 2 |
| Path 901 | C00031->C00123:[4->1,4->3,4->5,7->2,7->8,7->9,9->1,9->5] | 1.00 | 366.878504673 | 37 | 107 | 0 | 2 |
| Path 902 | C00031->C00123:[4->1,4->3,7->2] | 0.50 | 396.779220779 | 30 | 77 | 0 | 2 |
| Path 903 | C00031->C00123:[4->1,4->3,7->2] | 0.50 | 426.567901235 | 32 | 81 | 0 | 2 |
| Path 904 | C00031->C00123:[7->2,7->3,7->8,9->1] | 0.67 | 241.807692308 | 20 | 52 | 0 | 2 |
| Path 905 | C00031->C00123:[2->3,7->2,7->9,9->1,9->5] | 0.83 | 338.406593407 | 27 | 91 | 0 | 2 |
| Path 906 | C00031->C00123:[7->2,7->8,9->1,9->3] | 0.67 | 279.473684211 | 21 | 57 | 0 | 2 |
| Path 907 | C00031->C00123:[4->1,4->3,4->5,7->2,7->9,9->1,9->5] | 0.83 | 386.887323944 | 31 | 71 | 0 | 1 |
| Path 908 | C00031->C00123:[4->1,4->3,4->5,7->2,7->8,7->9,9->1,9->5] | 1.00 | 337.46728972 | 37 | 107 | 0 | 2 |
| Path 909 | C00031->C00123:[4->3,5->8] | 0.33 | 334.367647059 | 24 | 68 | 0 | 1 |
| Path 910 | C00031->C00123:[4->3,7->2,7->9,9->1,9->5] | 0.83 | 412.805555556 | 35 | 108 | 0 | 1 |
| Path 911 | C00031->C00123:[4->1,4->3,4->5,7->2,7->9,9->1,9->5] | 0.83 | 314.356164384 | 28 | 73 | 0 | 1 |
| Path 912 | C00031->C00123:[7->2,7->9,9->1,9->3,9->5] | 0.83 | 216.818181818 | 20 | 66 | 0 | 2 |
| Path 913 | C00031->C00123:[2->3,7->8,7->9,9->5] | 0.67 | 304.817073171 | 26 | 82 | 0 | 2 |
| Path 914 | C00031->C00123:[4->1,4->3,4->5,7->2,7->9,9->1,9->5] | 0.83 | 367.339805825 | 32 | 103 | 0 | 2 |
| Path 915 | C00031->C00123:[7->2,7->8,9->1,9->3] | 0.67 | 257.616666667 | 23 | 60 | 0 | 2 |
| Path 916 | C00031->C00123:[7->2,9->1,9->3] | 0.50 | 426.349206349 | 23 | 63 | 0 | 1 |
| Path 917 | C00031->C00123:[7->2,7->8,9->1] | 0.50 | 234.13559322 | 20 | 59 | 0 | 1 |
| Path 918 | C00031->C00123:[4->3,7->2,7->9,9->1,9->5] | 0.83 | 362.558558559 | 37 | 111 | 0 | 2 |
| Path 919 | C00031->C00123:[7->2,7->8,7->9,9->1,9->3,9->5] | 1.00 | 255.59375 | 23 | 96 | 0 | 2 |
| Path 920 | C00031->C00123:[7->2,7->8,9->1,9->3] | 0.67 | 279.232142857 | 20 | 56 | 0 | 2 |
| Path 921 | C00031->C00123:[4->1,4->3,5->2,5->8] | 0.67 | 365.654320988 | 32 | 81 | 0 | 1 |
| Path 922 | C00031->C00123:[7->8,9->3] | 0.33 | 303.914285714 | 25 | 70 | 0 | 2 |
| Path 923 | C00031->C00123:[2->3,5->8] | 0.33 | 371.333333333 | 31 | 102 | 0 | 2 |
| Path 924 | C00031->C00123:[4->1,4->3,7->2] | 0.50 | 362.794871795 | 21 | 39 | 0 | 2 |
| Path 925 | C00031->C00123:[7->8,7->9,9->3,9->5] | 0.67 | 236.0 | 20 | 57 | 0 | 2 |
| Path 926 | C00031->C00123:[4->3,7->2,7->9,9->1,9->3,9->5] | 0.83 | 318.97826087 | 34 | 92 | 0 | 2 |
| Path 927 | C00031->C00123:[4->1,4->3,7->2] | 0.50 | 444.564102564 | 24 | 39 | 0 | 1 |
| Path 928 | C00031->C00123:[7->2,7->8,9->1] | 0.50 | 309.216666667 | 21 | 60 | 0 | 1 |
| Path 929 | C00031->C00123:[2->3,7->2,7->8,7->9,9->1,9->5] | 1.00 | 343.494736842 | 31 | 95 | 0 | 2 |
| Path 930 | C00031->C00123:[7->2,9->1,9->3] | 0.50 | 285.666666667 | 21 | 54 | 0 | 2 |
| Path 931 | C00031->C00123:[5->3,7->2,7->8,7->9,9->1,9->5] | 1.00 | 277.934210526 | 27 | 76 | 0 | 2 |
| Path 932 | C00031->C00123:[2->3,7->2,9->1] | 0.50 | 407.666666667 | 22 | 72 | 0 | 1 |
| Path 933 | C00031->C00123:[2->3,5->8] | 0.33 | 363.052631579 | 27 | 95 | 0 | 2 |
| Path 934 | C00031->C00123:[4->1,4->3,7->2] | 0.50 | 373.833333333 | 22 | 42 | 0 | 2 |
| Path 935 | C00031->C00123:[7->2,7->8,9->1,9->3] | 0.67 | 299.120689655 | 22 | 58 | 0 | 2 |
| Path 936 | C00031->C00123:[4->3,7->8,7->9,9->5] | 0.67 | 259.363636364 | 20 | 55 | 0 | 2 |
| Path 937 | C00031->C00123:[2->3,7->2,7->8,7->9,9->1,9->5] | 1.00 | 306.047619048 | 28 | 84 | 0 | 2 |
| Path 938 | C00031->C00123:[4->1,4->3,5->2,5->8] | 0.67 | 354.472972973 | 28 | 74 | 0 | 1 |
| Path 939 | C00031->C00123:[2->3,7->8,7->9,9->5] | 0.67 | 308.543209877 | 24 | 81 | 0 | 2 |
| Path 940 | C00031->C00123:[4->1,4->3,7->2,7->8,9->1] | 0.67 | 310.596774194 | 24 | 62 | 0 | 1 |
| Path 941 | C00031->C00123:[4->1,4->3,4->5,7->2,7->9,9->1,9->5] | 0.83 | 365.405797101 | 29 | 69 | 0 | 1 |
| Path 942 | C00031->C00123:[4->1,4->3,7->2,9->1] | 0.50 | 459.854166667 | 27 | 48 | 0 | 1 |
| Path 943 | C00031->C00123:[4->1,4->3,9->2,9->8] | 0.67 | 335.788732394 | 30 | 71 | 0 | 1 |
| Path 944 | C00031->C00123:[2->3,7->2,7->8,9->1] | 0.67 | 382.24 | 31 | 100 | 0 | 2 |
| Path 945 | C00031->C00123:[4->1,4->3,7->2] | 0.50 | 328.0 | 22 | 54 | 0 | 1 |
| Path 946 | C00031->C00123:[4->1,4->3,4->5,7->2,7->8,7->9,9->1,9->5] | 1.00 | 285.594202899 | 28 | 69 | 0 | 2 |
| Path 947 | C00031->C00123:[7->2,7->8,9->1,9->3] | 0.67 | 277.509433962 | 20 | 53 | 0 | 2 |
| Path 948 | C00031->C00123:[4->1,4->2,4->3] | 0.50 | 442.648648649 | 26 | 74 | 0 | 2 |
| Path 949 | C00031->C00123:[4->1,4->3,4->5,7->2,7->8,7->9,9->1,9->5] | 1.00 | 332.708737864 | 38 | 103 | 0 | 2 |
| Path 950 | C00031->C00123:[4->3,7->2,7->9,9->1,9->5] | 0.83 | 371.45045045 | 34 | 111 | 0 | 2 |
| Path 951 | C00031->C00123:[4->1,4->2,4->3] | 0.50 | 396.7 | 27 | 70 | 0 | 2 |
| Path 952 | C00031->C00123:[4->1,4->3,7->2] | 0.50 | 440.9125 | 32 | 80 | 0 | 2 |
| Path 953 | C00031->C00123:[7->2,7->9,9->1,9->5] | 0.67 | 241.773584906 | 18 | 53 | 0 | 1 |
| Path 954 | C00031->C00123:[7->2,7->8,9->1,9->3] | 0.67 | 305.375 | 27 | 72 | 0 | 2 |
| Path 955 | C00031->C00123:[7->1,7->2,7->3,7->5,7->9,9->1,9->5] | 0.83 | 304.368421053 | 31 | 76 | 0 | 2 |
| Path 956 | C00031->C00123:[4->1,4->3,7->2] | 0.50 | 391.87804878 | 22 | 41 | 0 | 1 |
| Path 957 | C00031->C00123:[4->1,4->3,7->2] | 0.50 | 437.083333333 | 34 | 84 | 0 | 2 |
| Path 958 | C00031->C00123:[4->1,4->3,7->2] | 0.50 | 365.23943662 | 28 | 71 | 0 | 1 |
| Path 959 | C00031->C00123:[5->8] | 0.17 | 299.172413793 | 19 | 58 | 0 | 1 |
| Path 960 | C00031->C00123:[4->1,4->3,5->1,9->2,9->8] | 0.67 | 322.0 | 28 | 68 | 0 | 1 |
| Path 961 | C00031->C00123:[7->8,9->3] | 0.33 | 296.296296296 | 21 | 54 | 0 | 2 |
| Path 962 | C00031->C00123:[5->3,7->2,7->8,7->9,9->1,9->5] | 1.00 | 348.39 | 30 | 100 | 0 | 2 |
| Path 963 | C00031->C00123:[7->2,7->8,7->9,9->1,9->3,9->5] | 1.00 | 253.281690141 | 25 | 71 | 0 | 2 |
| Path 964 | C00031->C00123:[7->8,9->3] | 0.33 | 343.636363636 | 25 | 66 | 0 | 2 |
| Path 965 | C00031->C00123:[4->1,4->3,7->2] | 0.50 | 436.821428571 | 33 | 84 | 0 | 2 |
| Path 966 | C00031->C00123:[7->2,7->8,9->1,9->3] | 0.67 | 244.156862745 | 18 | 51 | 0 | 2 |
| Path 967 | C00031->C00123:[2->3,7->2,7->8,9->1] | 0.67 | 321.734939759 | 26 | 83 | 0 | 2 |
| Path 968 | C00031->C00123:[4->3,4->5,7->8,7->9,9->5] | 0.67 | 296.862068966 | 22 | 58 | 0 | 1 |
| Path 969 | C00031->C00123:[4->3,7->2,9->1] | 0.50 | 252.884615385 | 18 | 52 | 0 | 1 |
| Path 970 | C00031->C00123:[4->1,4->3,7->2] | 0.50 | 437.054054054 | 23 | 37 | 0 | 1 |
| Path 971 | C00031->C00123:[7->2,7->9,9->1,9->5] | 0.67 | 271.833333333 | 22 | 60 | 0 | 1 |
| Path 972 | C00031->C00123:[4->1,4->3] | 0.33 | 390.064516129 | 18 | 31 | 0 | 2 |
| Path 973 | C00031->C00123:[7->2,9->1,9->3] | 0.50 | 208.75 | 18 | 60 | 0 | 2 |
| Path 974 | C00031->C00123:[4->1,4->3,7->2] | 0.50 | 393.315068493 | 31 | 73 | 0 | 2 |
| Path 975 | C00031->C00123:[4->1,4->3,4->5,7->2,7->9,9->1,9->5] | 0.83 | 290.4 | 27 | 70 | 0 | 2 |
| Path 976 | C00031->C00123:[2->3,7->8] | 0.33 | 348.047058824 | 25 | 85 | 0 | 2 |
| Path 977 | C00031->C00123:[4->3,7->2,7->9,9->1,9->5] | 0.83 | 250.933333333 | 21 | 60 | 0 | 2 |
| Path 978 | C00031->C00123:[2->3,7->2,7->8,9->1] | 0.67 | 348.627906977 | 25 | 86 | 0 | 2 |
| Path 979 | C00031->C00123:[7->8,9->3] | 0.33 | 339.985507246 | 25 | 69 | 0 | 2 |
| Path 980 | C00031->C00123:[7->2,7->8,9->1,9->3] | 0.67 | 267.245283019 | 20 | 53 | 0 | 2 |
| Path 981 | C00031->C00123:[7->2,9->1,9->3] | 0.50 | 260.98 | 18 | 50 | 0 | 1 |
| Path 982 | C00031->C00123:[7->2,7->8,9->1,9->3] | 0.67 | 277.796296296 | 21 | 54 | 0 | 2 |
| Path 983 | C00031->C00123:[7->2,7->3,9->1] | 0.50 | 267.381818182 | 21 | 55 | 0 | 1 |
| Path 984 | C00031->C00123:[4->3,5->8] | 0.33 | 325.923076923 | 23 | 65 | 0 | 1 |
| Path 985 | C00031->C00123:[4->3,7->2,7->8,7->9,9->1,9->5] | 1.00 | 319.475609756 | 34 | 82 | 0 | 2 |
| Path 986 | C00031->C00123:[4->3,7->2,7->8,7->9,9->1,9->5] | 1.00 | 374.234782609 | 38 | 115 | 0 | 2 |
| Path 987 | C00031->C00123:[2->3,7->2,7->9,9->1,9->5] | 0.83 | 296.390804598 | 27 | 87 | 0 | 2 |
| Path 988 | C00031->C00123:[7->8,9->3] | 0.33 | 350.011764706 | 25 | 85 | 0 | 2 |
| Path 989 | C00031->C00123:[4->1,4->3,7->2] | 0.50 | 376.465116279 | 23 | 43 | 0 | 2 |
| Path 990 | C00031->C00123:[4->3,7->2,7->8,7->9,9->1,9->5] | 1.00 | 374.105263158 | 37 | 114 | 0 | 2 |
| Path 991 | C00031->C00123:[4->1,4->3] | 0.33 | 412.793103448 | 17 | 29 | 0 | 1 |
| Path 992 | C00031->C00123:[2->3,7->2,7->8,9->1] | 0.67 | 365.764044944 | 29 | 89 | 0 | 2 |
| Path 993 | C00031->C00123:[2->3,7->2,7->8,9->1] | 0.67 | 366.340909091 | 27 | 88 | 0 | 2 |
| Path 994 | C00031->C00123:[7->2,9->1,9->3] | 0.50 | 334.114285714 | 18 | 35 | 0 | 1 |
| Path 995 | C00031->C00123:[2->3,7->2,7->8,7->9,9->1,9->5] | 1.00 | 349.056818182 | 28 | 88 | 0 | 2 |
| Path 996 | C00031->C00123:[4->3,7->2,7->9,9->1,9->3,9->5] | 0.83 | 367.864864865 | 37 | 111 | 0 | 2 |
| Path 997 | C00031->C00123:[9->3] | 0.17 | 232.266666667 | 13 | 45 | 0 | 2 |
| Path 998 | C00031->C00123:[4->1,4->3,7->2] | 0.50 | 436.574712644 | 31 | 87 | 0 | 2 |
| Path 999 | C00031->C00123:[2->3,7->2,7->8,7->9,9->1,9->5] | 1.00 | 337.173469388 | 31 | 98 | 0 | 2 |
| Path 1000 | C00031->C00123:[7->2,7->8,9->1,9->3] | 0.67 | 253.446808511 | 21 | 94 | 0 | 2 |
| Path 1001 | C00031->C00123:[4->3,7->2,9->1] | 0.50 | 265.679245283 | 19 | 53 | 0 | 1 |
| Path 1002 | C00031->C00123:[7->2,7->8,9->1] | 0.50 | 282.163636364 | 21 | 55 | 0 | 1 |
| Path 1003 | C00031->C00123:[4->3,7->2,9->1] | 0.50 | 333.675 | 19 | 40 | 0 | 1 |
| Path 1004 | C00031->C00123:[4->1,4->2,4->3,4->8] | 0.67 | 453.580246914 | 32 | 81 | 0 | 1 |
| Path 1005 | C00031->C00123:[4->1,4->3,7->2,7->8,9->1] | 0.67 | 317.758064516 | 24 | 62 | 0 | 1 |
| Path 1006 | C00031->C00123:[2->3] | 0.17 | 422.213114754 | 15 | 61 | 0 | 2 |
| Path 1007 | C00031->C00123:[7->2,9->1] | 0.33 | 207.044444444 | 14 | 45 | 0 | 1 |
| Path 1008 | C00031->C00123:[7->2,7->8,7->9,9->1,9->3,9->5] | 1.00 | 251.333333333 | 23 | 60 | 0 | 2 |
| Path 1009 | C00031->C00123:[4->1,4->3,7->2] | 0.50 | 426.727272727 | 25 | 44 | 0 | 1 |
| Path 1010 | C00031->C00123:[7->3,7->8] | 0.33 | 354.235294118 | 19 | 34 | 0 | 2 |
| Path 1011 | C00031->C00123:[5->3,7->2,7->8,9->1] | 0.67 | 300.739130435 | 24 | 69 | 0 | 2 |
| Path 1012 | C00031->C00123:[2->3,7->2,7->9,9->1,9->5] | 0.83 | 332.905263158 | 28 | 95 | 0 | 2 |
| Path 1013 | C00031->C00123:[7->8,9->3] | 0.33 | 254.176470588 | 18 | 51 | 0 | 2 |
| Path 1014 | C00031->C00123:[7->2,7->3,7->8,9->1] | 0.67 | 315.393939394 | 26 | 66 | 0 | 2 |
| Path 1015 | C00031->C00123:[4->1,4->3] | 0.33 | 433.58974359 | 22 | 39 | 0 | 1 |
| Path 1016 | C00031->C00123:[7->2,7->3,9->1] | 0.50 | 259.72 | 19 | 50 | 0 | 1 |
| Path 1017 | C00031->C00123:[1->3] | 0.17 | 422.877192982 | 17 | 57 | 0 | 2 |
| Path 1018 | C00031->C00123:[7->2,9->1,9->3] | 0.50 | 238.403225806 | 20 | 62 | 0 | 2 |
| Path 1019 | C00031->C00123:[4->1,4->2,4->3] | 0.50 | 436.786666667 | 28 | 75 | 0 | 2 |
| Path 1020 | C00031->C00123:[4->1,4->3,4->5,7->2,7->9,9->1,9->5] | 0.83 | 363.836538462 | 34 | 104 | 0 | 2 |
| Path 1021 | C00031->C00123:[7->2,9->1,9->3] | 0.50 | 263.730769231 | 19 | 52 | 0 | 2 |
| Path 1022 | C00031->C00123:[4->1,4->3,4->5,7->2,7->9,9->1,9->5] | 0.83 | 277.106060606 | 25 | 66 | 0 | 2 |
| Path 1023 | C00031->C00123:[7->2,7->8,7->9,9->1,9->3,9->5] | 1.00 | 345.252631579 | 31 | 95 | 0 | 2 |
| Path 1024 | C00031->C00123:[7->2,9->1,9->3] | 0.50 | 284.784313725 | 19 | 51 | 0 | 1 |
| Path 1025 | C00031->C00123:[2->3,7->8] | 0.33 | 359.255555556 | 24 | 90 | 0 | 2 |
| Path 1026 | C00031->C00123:[4->1,4->2,4->3] | 0.50 | 437.127906977 | 30 | 86 | 0 | 2 |
| Path 1027 | C00031->C00123:[2->3,7->8] | 0.33 | 354.569767442 | 25 | 86 | 0 | 2 |
| Path 1028 | C00031->C00123:[7->2,7->8,7->9,9->1,9->3,9->5] | 1.00 | 277.31147541 | 24 | 61 | 0 | 2 |
| Path 1029 | C00031->C00123:[4->3,7->2,7->8,7->9,9->1,9->5] | 1.00 | 374.357798165 | 36 | 109 | 0 | 2 |
| Path 1030 | C00031->C00123:[7->8] | 0.17 | 235.260869565 | 15 | 46 | 0 | 1 |
| Path 1031 | C00031->C00123:[2->3,7->8] | 0.33 | 431.405797101 | 26 | 69 | 0 | 2 |
| Path 1032 | C00031->C00123:[7->2,7->8,9->1,9->3] | 0.67 | 298.545454545 | 22 | 55 | 0 | 2 |
| Path 1033 | C00031->C00123:[2->3,7->2,7->8,9->1] | 0.67 | 329.755319149 | 31 | 94 | 0 | 2 |
| Path 1034 | C00031->C00123:[7->2,7->8,9->1,9->3] | 0.67 | 365.244680851 | 32 | 94 | 0 | 2 |
| Path 1035 | C00031->C00123:[2->3,7->8] | 0.33 | 386.796875 | 24 | 64 | 0 | 2 |
| Path 1036 | C00031->C00123:[4->1,4->3,4->5,7->2,7->8,7->9,9->1,9->5] | 1.00 | 370.234234234 | 36 | 111 | 0 | 2 |
| Path 1037 | C00031->C00123:[2->3,7->2,7->8,9->1] | 0.67 | 317.647058824 | 29 | 85 | 0 | 2 |
| Path 1038 | C00031->C00123:[7->2,7->9,9->1,9->3,9->5] | 0.83 | 235.617647059 | 22 | 68 | 0 | 2 |
