# Supplementary material for: 13C-metabolic flux ratio and novel carbon path analyses confirmed that Trichoderma reesei uses primarily the respirative pathway also on the preferred carbon source glucose
Source: BMC Syst Biol. 2009 Oct 29;3:104. doi: 10.1186/1752-0509-3-104 (PMC2776023; doi:10.1186/1752-0509-3-104)
Supplement: Additional file 1 — Pathways discovered in ReTrace carbon path analysis. Graphical and tabular representations of amino acid synthesis pathways discovered in ReTrace carbon path analysis [21]. Self-contained web site: unpack zip archive and open index.html with a web browser. [file 1752-0509-3-104-S1.zip › AF1-treesei/pathways-C00279-C00074-to-C00079.html]

Pathways from C00279,C00074 to C00079


**Pathways from C00279-C00074 to C00079**

**Sources:** D-Erythrose 4-phosphate (C00279)
Phosphoenolpyruvate; (C00074)

**Target:**L-Phenylalanine; (C00079)

|  | Composite mapping | Z | Average score | Rpairs | Reactions | Zero scores | Scores under threshold |
| --- | --- | --- | --- | --- | --- | --- | --- |
| Path 1 | C00279->C00079:[10->7,6->3,7->6,8->10], C00074->C00079:[6->1,6->5,7->8,8->2,8->4] | 1.00 | 656.1 | 12 | 20 | 0 | 0 |
| Path 2 | C00279->C00079:[10->7,6->3,7->6,8->10], C00074->C00079:[6->1,6->5,7->8,8->2,8->4] | 1.00 | 589.743589744 | 19 | 39 | 0 | 0 |
| Path 3 | C00279->C00079:[10->7,6->3,7->6,8->10], C00074->C00079:[6->1,6->5,7->8,8->2,8->4] | 1.00 | 575.064516129 | 18 | 31 | 0 | 0 |
| Path 4 | C00074->C00079:[7->2,7->5,7->8] | 0.33 | 372.328767123 | 20 | 73 | 0 | 1 |
| Path 5 | C00074->C00079:[7->2,7->5,7->8] | 0.33 | 424.055555556 | 24 | 72 | 0 | 1 |
| Path 6 | C00074->C00079:[7->5,7->8] | 0.22 | 488.137254902 | 16 | 51 | 0 | 0 |
| Path 7 | C00074->C00079:[7->5,7->8] | 0.22 | 477.369565217 | 12 | 46 | 0 | 0 |
| Path 8 | C00074->C00079:[7->2,7->5,7->8] | 0.33 | 427.083333333 | 22 | 60 | 0 | 1 |
| Path 9 | C00074->C00079:[7->5,7->8] | 0.22 | 430.961538462 | 16 | 52 | 0 | 1 |
| Path 10 | C00074->C00079:[7->2,7->5,7->8] | 0.33 | 460.464285714 | 20 | 56 | 0 | 1 |
| Path 11 | C00279->C00079:[6->5,6->8], C00074->C00079:[6->5,6->8] | 0.22 | 403.136363636 | 21 | 66 | 0 | 1 |
| Path 12 | C00074->C00079:[7->2,7->5,7->8] | 0.33 | 460.265625 | 21 | 64 | 0 | 0 |
| Path 13 | C00074->C00079:[7->5,7->8] | 0.22 | 461.444444444 | 15 | 54 | 0 | 0 |
| Path 14 | C00074->C00079:[7->5,7->8] | 0.22 | 420.869565217 | 21 | 69 | 0 | 1 |
| Path 15 | C00074->C00079:[7->5,7->8] | 0.22 | 403.227272727 | 18 | 44 | 0 | 0 |
| Path 16 | C00074->C00079:[7->2,7->5,7->8] | 0.33 | 445.434210526 | 27 | 76 | 0 | 1 |
| Path 17 | C00074->C00079:[7->2,7->5,7->8] | 0.33 | 466.830769231 | 22 | 65 | 0 | 0 |
| Path 18 | C00074->C00079:[7->5,7->8] | 0.22 | 403.060606061 | 11 | 33 | 0 | 0 |
| Path 19 | C00074->C00079:[7->5,7->8] | 0.22 | 360.925373134 | 15 | 67 | 0 | 1 |
| Path 20 | C00074->C00079:[7->5,7->8] | 0.22 | 413.5 | 16 | 46 | 0 | 0 |
| Path 21 | C00074->C00079:[6->5,6->8] | 0.22 | 372.588235294 | 20 | 51 | 0 | 1 |
| Path 22 | C00074->C00079:[7->2,7->5,7->8] | 0.33 | 430.373333333 | 27 | 75 | 0 | 1 |
| Path 23 | C00074->C00079:[7->2,7->5,7->8] | 0.33 | 432.955555556 | 13 | 45 | 0 | 0 |
| Path 24 | C00074->C00079:[7->5,7->8] | 0.22 | 378.327272727 | 18 | 55 | 0 | 1 |
| Path 25 | C00279->C00079:[6->5,6->8], C00074->C00079:[6->5,6->8] | 0.22 | 408.228070175 | 19 | 57 | 0 | 1 |
| Path 26 | C00074->C00079:[7->2,7->5,7->8] | 0.33 | 402.864864865 | 24 | 74 | 0 | 1 |
| Path 27 | C00074->C00079:[7->5,7->8] | 0.22 | 436.244897959 | 11 | 49 | 0 | 0 |
| Path 28 | C00074->C00079:[7->2,7->5,7->8] | 0.33 | 449.360655738 | 19 | 61 | 0 | 1 |
| Path 29 | C00279->C00079:[6->5,6->8] | 0.22 | 423.4 | 13 | 25 | 0 | 0 |
| Path 30 | C00074->C00079:[7->2,7->5,7->8] | 0.33 | 426.253333333 | 26 | 75 | 0 | 1 |
| Path 31 | C00074->C00079:[7->8] | 0.11 | 386.344827586 | 8 | 29 | 0 | 1 |
| Path 32 | C00074->C00079:[7->5,7->8] | 0.22 | 343.72972973 | 11 | 37 | 0 | 1 |
| Path 33 | C00074->C00079:[6->5,6->8,8->2] | 0.33 | 434.555555556 | 18 | 54 | 0 | 3 |
| Path 34 | C00074->C00079:[6->5,7->8,8->2] | 0.33 | 462.625 | 17 | 48 | 0 | 3 |
| Path 35 | C00074->C00079:[7->2,7->5,7->8] | 0.33 | 451.46031746 | 23 | 63 | 0 | 1 |
| Path 36 | C00279->C00079:[6->5,6->8], C00074->C00079:[6->5,6->8] | 0.22 | 395.692307692 | 20 | 65 | 0 | 1 |
| Path 37 | C00074->C00079:[7->2,7->5,7->8] | 0.33 | 447.878787879 | 25 | 66 | 0 | 1 |
| Path 38 | C00074->C00079:[7->5,7->8] | 0.22 | 465.704545455 | 18 | 44 | 0 | 0 |
| Path 39 | C00279->C00079:[10->7,6->3,7->6,8->10], C00074->C00079:[6->1,6->5,7->8,8->2,8->4] | 1.00 | 546.459459459 | 14 | 37 | 0 | 2 |
| Path 40 | C00279->C00079:[6->5,6->8] | 0.22 | 431.073170732 | 20 | 41 | 0 | 1 |
| Path 41 | C00074->C00079:[7->2,7->5,7->8] | 0.33 | 438.166666667 | 20 | 60 | 0 | 1 |
| Path 42 | C00074->C00079:[7->2,7->5,7->8] | 0.33 | 415.592592593 | 17 | 54 | 0 | 1 |
| Path 43 | C00279->C00079:[6->5,6->8] | 0.22 | 424.888888889 | 15 | 27 | 0 | 1 |
| Path 44 | C00074->C00079:[7->2,7->5,7->8] | 0.33 | 413.807017544 | 19 | 57 | 0 | 1 |
| Path 45 | C00074->C00079:[7->2,7->5,7->8] | 0.33 | 421.196428571 | 18 | 56 | 0 | 0 |
| Path 46 | C00074->C00079:[7->2,7->5,7->8] | 0.33 | 456.8 | 18 | 60 | 0 | 0 |
| Path 47 | C00074->C00079:[7->5,7->8] | 0.22 | 437.203389831 | 17 | 59 | 0 | 1 |
| Path 48 | C00279->C00079:[6->5,6->8] | 0.22 | 441.230769231 | 14 | 26 | 0 | 0 |
| Path 49 | C00074->C00079:[7->5,7->8] | 0.22 | 362.22 | 15 | 50 | 0 | 1 |
| Path 50 | C00074->C00079:[7->2,7->5,7->8] | 0.33 | 450.47761194 | 25 | 67 | 0 | 1 |
| Path 51 | C00074->C00079:[7->5,7->8] | 0.22 | 494.76744186 | 18 | 43 | 0 | 1 |
| Path 52 | C00074->C00079:[7->2,7->5,7->8] | 0.33 | 426.93877551 | 16 | 49 | 0 | 1 |
| Path 53 | C00074->C00079:[7->2,7->5,7->8] | 0.33 | 463.675675676 | 28 | 74 | 0 | 1 |
| Path 54 | C00074->C00079:[6->5,6->8,7->5,7->8] | 0.22 | 443.104166667 | 16 | 48 | 0 | 1 |
| Path 55 | C00074->C00079:[7->5,7->8] | 0.22 | 467.92 | 15 | 50 | 0 | 1 |
| Path 56 | C00074->C00079:[7->5,7->8] | 0.22 | 421.714285714 | 13 | 49 | 0 | 1 |
| Path 57 | C00279->C00079:[6->8], C00074->C00079:[6->5,8->2] | 0.33 | 453.2 | 20 | 55 | 0 | 3 |
| Path 58 | C00074->C00079:[7->2,7->5,7->8] | 0.33 | 450.040540541 | 26 | 74 | 0 | 1 |
| Path 59 | C00074->C00079:[7->2,7->5,7->8] | 0.33 | 463.633333333 | 23 | 60 | 0 | 1 |
| Path 60 | C00074->C00079:[6->5,6->8,7->5,7->8] | 0.22 | 437.025641026 | 13 | 39 | 0 | 0 |
| Path 61 | C00074->C00079:[7->5,7->8] | 0.22 | 348.377777778 | 14 | 45 | 0 | 1 |
| Path 62 | C00074->C00079:[7->2,7->5,7->8] | 0.33 | 422.259259259 | 26 | 81 | 0 | 1 |
| Path 63 | C00074->C00079:[7->5,7->8] | 0.22 | 485.428571429 | 17 | 42 | 0 | 1 |
| Path 64 | C00074->C00079:[7->2,7->5,7->8] | 0.33 | 443.803571429 | 17 | 56 | 0 | 0 |
| Path 65 | C00074->C00079:[6->5,6->8,7->5,7->8] | 0.22 | 452.531914894 | 15 | 47 | 0 | 0 |
| Path 66 | C00074->C00079:[7->5,7->8] | 0.22 | 404.095238095 | 13 | 42 | 0 | 0 |
| Path 67 | C00074->C00079:[7->2,7->5,7->8] | 0.33 | 424.285714286 | 28 | 84 | 0 | 1 |
| Path 68 | C00074->C00079:[7->2,7->5,7->8] | 0.33 | 399.397590361 | 26 | 83 | 0 | 1 |
| Path 69 | C00279->C00079:[6->8] | 0.11 | 404.19047619 | 10 | 21 | 0 | 1 |
| Path 70 | C00074->C00079:[7->8] | 0.11 | 331.90625 | 7 | 32 | 0 | 1 |
| Path 71 | C00074->C00079:[7->5,7->8] | 0.22 | 431.638297872 | 14 | 47 | 0 | 1 |
| Path 72 | C00279->C00079:[6->2,6->5,6->8] | 0.33 | 412.1 | 17 | 30 | 0 | 0 |
| Path 73 | C00074->C00079:[7->2,7->5,7->8] | 0.33 | 367.970149254 | 20 | 67 | 0 | 1 |
| Path 74 | C00074->C00079:[7->5,7->8] | 0.22 | 445.672727273 | 16 | 55 | 0 | 1 |
| Path 75 | C00074->C00079:[6->5,7->8,8->2] | 0.33 | 435.724137931 | 16 | 58 | 0 | 3 |
| Path 76 | C00074->C00079:[7->2,7->5,7->8] | 0.33 | 372.728571429 | 22 | 70 | 0 | 1 |
| Path 77 | C00074->C00079:[7->2,7->5,7->8] | 0.33 | 354.2 | 22 | 80 | 0 | 1 |
| Path 78 | C00074->C00079:[7->5,7->8] | 0.22 | 423.574468085 | 17 | 47 | 0 | 0 |
| Path 79 | C00074->C00079:[6->5,6->8,7->5,7->8] | 0.22 | 426.1 | 14 | 40 | 0 | 1 |
| Path 80 | C00074->C00079:[6->5,6->8] | 0.22 | 429.702702703 | 12 | 37 | 0 | 1 |
| Path 81 | C00074->C00079:[6->5,7->8,8->2] | 0.33 | 449.525 | 15 | 40 | 0 | 3 |
| Path 82 | C00074->C00079:[7->2,7->5,7->8] | 0.33 | 421.901639344 | 20 | 61 | 0 | 1 |
| Path 83 | C00074->C00079:[6->5,6->8] | 0.22 | 339.266666667 | 15 | 60 | 0 | 1 |
| Path 84 | C00074->C00079:[7->2,7->5,7->8] | 0.33 | 490.363636364 | 26 | 66 | 0 | 1 |
| Path 85 | C00074->C00079:[7->5,7->8] | 0.22 | 427.52 | 12 | 50 | 0 | 1 |
| Path 86 | C00074->C00079:[7->2,7->5,7->8] | 0.33 | 423.433962264 | 16 | 53 | 0 | 0 |
| Path 87 | C00074->C00079:[7->2,7->5,7->8] | 0.33 | 365.923076923 | 23 | 78 | 0 | 1 |
| Path 88 | C00074->C00079:[7->2,7->5,7->8] | 0.33 | 465.431372549 | 16 | 51 | 0 | 0 |
| Path 89 | C00074->C00079:[7->2,7->5,7->8] | 0.33 | 464.365079365 | 25 | 63 | 0 | 1 |
| Path 90 | C00074->C00079:[7->5,7->8] | 0.22 | 394.76744186 | 14 | 43 | 0 | 1 |
| Path 91 | C00074->C00079:[6->5,6->8] | 0.22 | 367.755102041 | 14 | 49 | 0 | 1 |
| Path 92 | C00074->C00079:[7->2,7->5,7->8] | 0.33 | 436.549295775 | 26 | 71 | 0 | 1 |
| Path 93 | C00074->C00079:[7->2,7->5,7->8] | 0.33 | 393.451219512 | 25 | 82 | 0 | 1 |
| Path 94 | C00279->C00079:[6->8] | 0.11 | 426.136363636 | 11 | 22 | 0 | 1 |
| Path 95 | C00074->C00079:[7->5,7->8] | 0.22 | 480.16 | 15 | 50 | 0 | 0 |
| Path 96 | C00074->C00079:[7->5,7->8] | 0.22 | 430.433962264 | 14 | 53 | 0 | 1 |
| Path 97 | C00074->C00079:[7->2,7->5,7->8] | 0.33 | 459.757575758 | 23 | 66 | 0 | 1 |
| Path 98 | C00074->C00079:[7->5,7->8] | 0.22 | 354.517241379 | 16 | 58 | 0 | 1 |
| Path 99 | C00074->C00079:[7->2,7->5,7->8] | 0.33 | 418.896103896 | 27 | 77 | 0 | 1 |
| Path 100 | C00074->C00079:[7->2,7->5,7->8] | 0.33 | 481.869565217 | 20 | 46 | 0 | 0 |
| Path 101 | C00074->C00079:[7->2,7->5,7->8] | 0.33 | 380.141025641 | 26 | 78 | 0 | 1 |
| Path 102 | C00074->C00079:[7->5,7->8] | 0.22 | 424.924528302 | 14 | 53 | 0 | 1 |
| Path 103 | C00074->C00079:[7->2,7->5,7->8] | 0.33 | 484.261538462 | 25 | 65 | 0 | 1 |
| Path 104 | C00074->C00079:[7->2,7->5,7->8] | 0.33 | 446.555555556 | 22 | 63 | 0 | 1 |
| Path 105 | C00074->C00079:[7->2,7->5,7->8] | 0.33 | 456.897058824 | 26 | 68 | 0 | 1 |
| Path 106 | C00074->C00079:[6->5,6->8] | 0.22 | 472.382978723 | 18 | 47 | 0 | 1 |
| Path 107 | C00074->C00079:[7->2,7->5,7->8] | 0.33 | 426.298245614 | 17 | 57 | 0 | 0 |
| Path 108 | C00074->C00079:[6->5,7->8,8->2] | 0.33 | 468.966666667 | 8 | 30 | 0 | 2 |
| Path 109 | C00074->C00079:[7->5,7->8] | 0.22 | 469.983606557 | 21 | 61 | 0 | 0 |
| Path 110 | C00074->C00079:[7->5,7->8] | 0.22 | 404.70212766 | 17 | 47 | 0 | 1 |
| Path 111 | C00279->C00079:[6->5,6->8] | 0.22 | 457.740740741 | 15 | 27 | 0 | 0 |
| Path 112 | C00074->C00079:[7->2,7->5,7->8] | 0.33 | 421.177419355 | 18 | 62 | 0 | 0 |
| Path 113 | C00074->C00079:[7->2,7->5,7->8] | 0.33 | 431.928571429 | 19 | 56 | 0 | 0 |
| Path 114 | C00074->C00079:[7->5,7->8] | 0.22 | 438.372093023 | 11 | 43 | 0 | 0 |
| Path 115 | C00074->C00079:[7->2,7->5,7->8] | 0.33 | 449.31147541 | 19 | 61 | 0 | 1 |
| Path 116 | C00074->C00079:[7->2,7->5,7->8] | 0.33 | 432.243589744 | 29 | 78 | 0 | 1 |
| Path 117 | C00074->C00079:[7->2,7->5,7->8] | 0.33 | 457.548387097 | 24 | 62 | 0 | 1 |
| Path 118 | C00074->C00079:[7->2,7->5,7->8] | 0.33 | 450.75 | 21 | 64 | 0 | 1 |
| Path 119 | C00074->C00079:[7->2,7->5,7->8] | 0.33 | 428.0 | 23 | 73 | 0 | 1 |
| Path 120 | C00074->C00079:[6->5,6->8,7->5,7->8] | 0.22 | 422.755102041 | 16 | 49 | 0 | 1 |
| Path 121 | C00074->C00079:[7->5,7->8] | 0.22 | 444.74137931 | 16 | 58 | 0 | 0 |
| Path 122 | C00279->C00079:[6->8] | 0.11 | 354.310344828 | 11 | 29 | 0 | 1 |
| Path 123 | C00074->C00079:[7->2,7->5,7->8] | 0.33 | 444.933333333 | 20 | 60 | 0 | 1 |
| Path 124 | C00074->C00079:[7->2,7->5,7->8] | 0.33 | 424.350877193 | 20 | 57 | 0 | 1 |
| Path 125 | C00074->C00079:[7->8] | 0.11 | 343.409090909 | 8 | 22 | 0 | 1 |
| Path 126 | C00074->C00079:[7->2,7->5,7->8] | 0.33 | 435.121212121 | 22 | 66 | 0 | 0 |
| Path 127 | C00074->C00079:[7->2,7->5,7->8] | 0.33 | 415.869565217 | 23 | 69 | 0 | 1 |
| Path 128 | C00074->C00079:[7->2,7->5,7->8] | 0.33 | 402.174418605 | 28 | 86 | 0 | 1 |
| Path 129 | C00074->C00079:[7->2,7->5,7->8] | 0.33 | 420.981818182 | 16 | 55 | 0 | 1 |
| Path 130 | C00074->C00079:[7->5,7->8] | 0.22 | 478.75 | 17 | 52 | 0 | 1 |
| Path 131 | C00074->C00079:[7->5,7->8] | 0.22 | 373.677419355 | 16 | 62 | 0 | 1 |
| Path 132 | C00074->C00079:[7->2,7->5,7->8] | 0.33 | 361.4 | 21 | 75 | 0 | 1 |
| Path 133 | C00074->C00079:[7->8] | 0.11 | 432.191489362 | 12 | 47 | 0 | 1 |
| Path 134 | C00074->C00079:[7->5,7->8] | 0.22 | 455.442622951 | 21 | 61 | 0 | 1 |
| Path 135 | C00074->C00079:[7->2,7->5,7->8] | 0.33 | 435.476190476 | 22 | 63 | 0 | 1 |
| Path 136 | C00279->C00079:[10->7,6->3,7->6,8->10], C00074->C00079:[6->1,6->5,7->8,8->2,8->4] | 1.00 | 566.986111111 | 31 | 72 | 0 | 1 |
| Path 137 | C00074->C00079:[6->5,6->8] | 0.22 | 411.409090909 | 20 | 44 | 0 | 1 |
| Path 138 | C00074->C00079:[7->5,7->8] | 0.22 | 462.403225806 | 22 | 62 | 0 | 1 |
| Path 139 | C00074->C00079:[7->2,7->5,7->8] | 0.33 | 453.184615385 | 22 | 65 | 0 | 1 |
| Path 140 | C00074->C00079:[7->2,7->5,7->8] | 0.33 | 460.777777778 | 18 | 54 | 0 | 0 |
| Path 141 | C00074->C00079:[7->2,7->5,7->8] | 0.33 | 455.876923077 | 24 | 65 | 0 | 1 |
| Path 142 | C00074->C00079:[7->2,7->5,7->8] | 0.33 | 423.543478261 | 14 | 46 | 0 | 1 |
| Path 143 | C00074->C00079:[7->2,7->5,7->8] | 0.33 | 471.549295775 | 27 | 71 | 0 | 1 |
| Path 144 | C00074->C00079:[7->2,7->5,7->8] | 0.33 | 376.186666667 | 24 | 75 | 0 | 1 |
| Path 145 | C00074->C00079:[7->2,7->5,7->8] | 0.33 | 458.741935484 | 22 | 62 | 0 | 0 |
| Path 146 | C00074->C00079:[7->5,7->8] | 0.22 | 448.064516129 | 19 | 62 | 0 | 0 |
| Path 147 | C00074->C00079:[7->5,7->8] | 0.22 | 452.551020408 | 17 | 49 | 0 | 0 |
| Path 148 | C00074->C00079:[7->2,7->5,7->8] | 0.33 | 463.028169014 | 26 | 71 | 0 | 1 |
| Path 149 | C00074->C00079:[7->2,7->5,7->8] | 0.33 | 429.75 | 15 | 48 | 0 | 0 |
| Path 150 | C00074->C00079:[7->5,7->8] | 0.22 | 427.557377049 | 18 | 61 | 0 | 1 |
| Path 151 | C00074->C00079:[7->2,7->5,7->8] | 0.33 | 372.328358209 | 20 | 67 | 0 | 1 |
| Path 152 | C00074->C00079:[7->2,7->5,7->8] | 0.33 | 417.066666667 | 21 | 60 | 0 | 1 |
| Path 153 | C00279->C00079:[6->5,6->8] | 0.22 | 419.675 | 19 | 40 | 0 | 1 |
| Path 154 | C00074->C00079:[7->2,7->5,7->8] | 0.33 | 457.709090909 | 19 | 55 | 0 | 1 |
| Path 155 | C00074->C00079:[7->2,7->5,7->8] | 0.33 | 456.971428571 | 25 | 70 | 0 | 1 |
| Path 156 | C00279->C00079:[6->2,6->5,6->8] | 0.33 | 441.78125 | 19 | 32 | 0 | 0 |
| Path 157 | C00074->C00079:[7->5,7->8] | 0.22 | 365.985714286 | 17 | 70 | 0 | 1 |
| Path 158 | C00074->C00079:[6->5,6->8] | 0.22 | 385.476923077 | 20 | 65 | 0 | 1 |
| Path 159 | C00074->C00079:[7->5,7->8] | 0.22 | 471.510204082 | 14 | 49 | 0 | 0 |
| Path 160 | C00074->C00079:[7->5,7->8] | 0.22 | 445.545454545 | 18 | 44 | 0 | 1 |
| Path 161 | C00074->C00079:[7->2,7->5,7->8] | 0.33 | 365.293333333 | 21 | 75 | 0 | 1 |
| Path 162 | C00074->C00079:[7->2,7->5,7->8] | 0.33 | 439.24 | 25 | 75 | 0 | 1 |
| Path 163 | C00074->C00079:[7->2,7->5,7->8] | 0.33 | 376.539473684 | 22 | 76 | 0 | 1 |
| Path 164 | C00074->C00079:[7->5,7->8] | 0.22 | 419.431372549 | 18 | 51 | 0 | 0 |
| Path 165 | C00074->C00079:[7->2,7->5,7->8] | 0.33 | 424.326923077 | 18 | 52 | 0 | 1 |
| Path 166 | C00074->C00079:[7->2,7->5,7->8] | 0.33 | 433.3 | 20 | 60 | 0 | 1 |
| Path 167 | C00074->C00079:[7->2,7->5,7->8] | 0.33 | 471.220588235 | 25 | 68 | 0 | 1 |
| Path 168 | C00074->C00079:[7->2,7->5,7->8] | 0.33 | 455.671232877 | 26 | 73 | 0 | 1 |
| Path 169 | C00279->C00079:[6->5,6->8], C00074->C00079:[6->5,6->8] | 0.22 | 400.348837209 | 19 | 43 | 0 | 1 |
| Path 170 | C00074->C00079:[7->2,7->5,7->8] | 0.33 | 349.337662338 | 20 | 77 | 0 | 1 |
| Path 171 | C00074->C00079:[7->5,7->8] | 0.22 | 353.277777778 | 10 | 36 | 0 | 0 |
| Path 172 | C00074->C00079:[7->2,7->5,7->8] | 0.33 | 369.457831325 | 25 | 83 | 0 | 1 |
| Path 173 | C00074->C00079:[7->5,7->8] | 0.22 | 453.054545455 | 16 | 55 | 0 | 1 |
| Path 174 | C00074->C00079:[7->2,7->5,7->8] | 0.33 | 449.140625 | 23 | 64 | 0 | 1 |
| Path 175 | C00074->C00079:[7->2,7->5,7->8] | 0.33 | 453.775862069 | 21 | 58 | 0 | 1 |
| Path 176 | C00074->C00079:[7->2,7->5,7->8] | 0.33 | 441.164179104 | 23 | 67 | 0 | 0 |
| Path 177 | C00074->C00079:[7->5,7->8] | 0.22 | 497.268292683 | 16 | 41 | 0 | 0 |
| Path 178 | C00074->C00079:[7->2,7->5,7->8] | 0.33 | 469.642857143 | 25 | 70 | 0 | 0 |
| Path 179 | C00074->C00079:[7->2,7->5,7->8] | 0.33 | 454.432835821 | 26 | 67 | 0 | 1 |
| Path 180 | C00074->C00079:[7->5,7->8] | 0.22 | 394.266666667 | 19 | 45 | 0 | 1 |
| Path 181 | C00074->C00079:[7->2,7->5,7->8] | 0.33 | 475.172413793 | 22 | 58 | 0 | 1 |
| Path 182 | C00074->C00079:[7->5,7->8] | 0.22 | 366.486842105 | 17 | 76 | 0 | 1 |
| Path 183 | C00074->C00079:[7->5,7->8] | 0.22 | 463.214285714 | 14 | 42 | 0 | 0 |
| Path 184 | C00074->C00079:[7->5,7->8] | 0.22 | 435.045454545 | 12 | 44 | 0 | 1 |
| Path 185 | C00074->C00079:[7->5,7->8] | 0.22 | 433.581818182 | 18 | 55 | 0 | 1 |
| Path 186 | C00074->C00079:[7->2,7->5,7->8] | 0.33 | 360.555555556 | 19 | 72 | 0 | 1 |
| Path 187 | C00074->C00079:[7->5,7->8] | 0.22 | 414.75 | 18 | 48 | 0 | 1 |
| Path 188 | C00074->C00079:[6->5,6->8,8->2] | 0.33 | 370.047619048 | 18 | 63 | 0 | 3 |
| Path 189 | C00074->C00079:[7->2,7->5,7->8] | 0.33 | 428.777777778 | 15 | 54 | 0 | 0 |
| Path 190 | C00074->C00079:[7->2,7->5,7->8] | 0.33 | 456.480769231 | 17 | 52 | 0 | 1 |
| Path 191 | C00074->C00079:[7->8] | 0.11 | 444.0 | 7 | 27 | 0 | 1 |
| Path 192 | C00074->C00079:[7->2,7->5,7->8] | 0.33 | 378.333333333 | 26 | 90 | 0 | 1 |
| Path 193 | C00074->C00079:[7->2,7->5,7->8] | 0.33 | 432.985074627 | 23 | 67 | 0 | 1 |
| Path 194 | C00074->C00079:[7->2,7->5,7->8] | 0.33 | 409.32 | 25 | 75 | 0 | 1 |
| Path 195 | C00074->C00079:[7->2,7->5,7->8] | 0.33 | 385.353658537 | 25 | 82 | 0 | 1 |
| Path 196 | C00074->C00079:[7->5,7->8] | 0.22 | 459.724137931 | 19 | 58 | 0 | 1 |
| Path 197 | C00074->C00079:[7->2,7->5,7->8] | 0.33 | 472.060606061 | 23 | 66 | 0 | 0 |
| Path 198 | C00074->C00079:[7->2,7->5,7->8] | 0.33 | 483.50877193 | 21 | 57 | 0 | 0 |
| Path 199 | C00074->C00079:[7->5,7->8] | 0.22 | 435.714285714 | 23 | 70 | 0 | 1 |
| Path 200 | C00074->C00079:[7->2,7->5,7->8] | 0.33 | 490.904761905 | 24 | 63 | 0 | 1 |
| Path 201 | C00074->C00079:[7->2,7->5,7->8] | 0.33 | 430.013157895 | 25 | 76 | 0 | 1 |
| Path 202 | C00279->C00079:[10->7,6->3,7->6,8->10], C00074->C00079:[6->1,6->5,7->8,8->2,8->4] | 1.00 | 678.906976744 | 22 | 43 | 0 | 0 |
| Path 203 | C00074->C00079:[7->5,7->8] | 0.22 | 506.547619048 | 17 | 42 | 0 | 0 |
| Path 204 | C00074->C00079:[7->5,7->8] | 0.22 | 454.35483871 | 10 | 31 | 0 | 0 |
| Path 205 | C00074->C00079:[7->2,7->5,7->8] | 0.33 | 428.626865672 | 23 | 67 | 0 | 1 |
| Path 206 | C00074->C00079:[6->5,7->8,8->2] | 0.33 | 438.433333333 | 20 | 60 | 0 | 3 |
| Path 207 | C00074->C00079:[7->2,7->5,7->8] | 0.33 | 454.769230769 | 24 | 65 | 0 | 0 |
| Path 208 | C00074->C00079:[7->2,7->5,7->8] | 0.33 | 433.944444444 | 22 | 72 | 0 | 0 |
| Path 209 | C00074->C00079:[7->5,7->8] | 0.22 | 422.816666667 | 19 | 60 | 0 | 1 |
| Path 210 | C00074->C00079:[7->5,7->8] | 0.22 | 368.322033898 | 14 | 59 | 0 | 1 |
| Path 211 | C00074->C00079:[7->2,7->5,7->8] | 0.33 | 440.644067797 | 19 | 59 | 0 | 0 |
| Path 212 | C00074->C00079:[7->2,7->5,7->8] | 0.33 | 456.85 | 18 | 60 | 0 | 0 |
| Path 213 | C00074->C00079:[7->2,7->5,7->8] | 0.33 | 463.594202899 | 24 | 69 | 0 | 0 |
| Path 214 | C00074->C00079:[7->2,7->5,7->8] | 0.33 | 416.459459459 | 25 | 74 | 0 | 1 |
| Path 215 | C00074->C00079:[7->2,7->5,7->8] | 0.33 | 449.231884058 | 27 | 69 | 0 | 1 |
| Path 216 | C00074->C00079:[7->2,7->5,7->8] | 0.33 | 412.0 | 27 | 78 | 0 | 1 |
| Path 217 | C00074->C00079:[6->5,6->8,8->2] | 0.33 | 439.888888889 | 16 | 45 | 0 | 3 |
| Path 218 | C00074->C00079:[7->2,7->5,7->8] | 0.33 | 367.328125 | 18 | 64 | 0 | 1 |
| Path 219 | C00074->C00079:[7->2,7->5,7->8] | 0.33 | 484.516129032 | 23 | 62 | 0 | 1 |
| Path 220 | C00074->C00079:[7->5,7->8] | 0.22 | 436.203703704 | 17 | 54 | 0 | 0 |
| Path 221 | C00074->C00079:[7->2,7->5,7->8] | 0.33 | 420.352941176 | 25 | 68 | 0 | 1 |
| Path 222 | C00074->C00079:[7->5,7->8] | 0.22 | 434.683333333 | 17 | 60 | 0 | 0 |
| Path 223 | C00074->C00079:[7->2,7->5,7->8] | 0.33 | 418.929824561 | 19 | 57 | 0 | 1 |
| Path 224 | C00074->C00079:[7->2,7->5,7->8] | 0.33 | 461.318181818 | 25 | 66 | 0 | 0 |
| Path 225 | C00074->C00079:[7->2,7->5,7->8] | 0.33 | 471.491525424 | 22 | 59 | 0 | 0 |
| Path 226 | C00074->C00079:[7->2,7->5,7->8] | 0.33 | 414.492063492 | 19 | 63 | 0 | 1 |
| Path 227 | C00074->C00079:[7->2,7->5,7->8] | 0.33 | 365.814814815 | 21 | 81 | 0 | 1 |
| Path 228 | C00074->C00079:[7->2,7->5,7->8] | 0.33 | 437.666666667 | 20 | 63 | 0 | 0 |
| Path 229 | C00074->C00079:[7->2,7->5,7->8] | 0.33 | 450.703125 | 21 | 64 | 0 | 1 |
| Path 230 | C00074->C00079:[6->5,7->8,8->2] | 0.33 | 440.836734694 | 14 | 49 | 0 | 3 |
| Path 231 | C00074->C00079:[7->2,7->5,7->8] | 0.33 | 461.406779661 | 22 | 59 | 0 | 1 |
| Path 232 | C00074->C00079:[7->5,7->8] | 0.22 | 455.189189189 | 18 | 37 | 0 | 0 |
| Path 233 | C00074->C00079:[7->2,7->5,7->8] | 0.33 | 420.979591837 | 16 | 49 | 0 | 1 |
| Path 234 | C00074->C00079:[7->2,7->5,7->8] | 0.33 | 452.4 | 19 | 55 | 0 | 1 |
| Path 235 | C00074->C00079:[7->2,7->5,7->8] | 0.33 | 407.823529412 | 20 | 51 | 0 | 0 |
| Path 236 | C00074->C00079:[7->5,7->8] | 0.22 | 439.225806452 | 19 | 62 | 0 | 1 |
| Path 237 | C00074->C00079:[7->2,7->5,7->8] | 0.33 | 369.857142857 | 23 | 84 | 0 | 1 |
| Path 238 | C00074->C00079:[7->5,7->8] | 0.22 | 442.625 | 9 | 40 | 0 | 0 |
| Path 239 | C00074->C00079:[7->2,7->5,7->8] | 0.33 | 451.168831169 | 28 | 77 | 0 | 1 |
| Path 240 | C00074->C00079:[7->2,7->5,7->8] | 0.33 | 465.014925373 | 24 | 67 | 0 | 1 |
| Path 241 | C00074->C00079:[7->5,7->8] | 0.22 | 443.194444444 | 17 | 36 | 0 | 0 |
| Path 242 | C00074->C00079:[7->2,7->5,7->8] | 0.33 | 405.831168831 | 26 | 77 | 0 | 1 |
| Path 243 | C00074->C00079:[7->2,7->5,7->8] | 0.33 | 445.458333333 | 21 | 48 | 0 | 0 |
| Path 244 | C00074->C00079:[7->8] | 0.11 | 433.555555556 | 6 | 36 | 0 | 1 |
| Path 245 | C00074->C00079:[7->2,7->5,7->8] | 0.33 | 440.769230769 | 27 | 78 | 0 | 1 |
| Path 246 | C00279->C00079:[6->8], C00074->C00079:[6->5,8->2] | 0.33 | 500.541666667 | 20 | 48 | 0 | 3 |
| Path 247 | C00279->C00079:[6->8], C00074->C00079:[6->5,8->2] | 0.33 | 492.319148936 | 19 | 47 | 0 | 3 |
| Path 248 | C00074->C00079:[7->5,7->8] | 0.22 | 404.166666667 | 13 | 42 | 0 | 0 |
| Path 249 | C00074->C00079:[7->2,7->5,7->8] | 0.33 | 436.01754386 | 18 | 57 | 0 | 1 |
| Path 250 | C00074->C00079:[7->5,7->8] | 0.22 | 430.5 | 12 | 48 | 0 | 0 |
| Path 251 | C00074->C00079:[6->5,6->8] | 0.22 | 425.434782609 | 14 | 46 | 0 | 1 |
| Path 252 | C00279->C00079:[6->5,6->8] | 0.22 | 441.392857143 | 16 | 28 | 0 | 1 |
| Path 253 | C00279->C00079:[6->5,6->8], C00074->C00079:[6->5,6->8] | 0.22 | 376.808219178 | 21 | 73 | 0 | 1 |
| Path 254 | C00074->C00079:[7->5,7->8] | 0.22 | 452.441860465 | 15 | 43 | 0 | 1 |
| Path 255 | C00074->C00079:[7->2,7->5,7->8] | 0.33 | 461.260869565 | 23 | 69 | 0 | 0 |
| Path 256 | C00074->C00079:[7->2,7->5,7->8] | 0.33 | 424.579710145 | 24 | 69 | 0 | 1 |
| Path 257 | C00074->C00079:[7->2,7->5,7->8] | 0.33 | 430.785714286 | 25 | 70 | 0 | 1 |
| Path 258 | C00074->C00079:[7->5,7->8] | 0.22 | 433.096153846 | 13 | 52 | 0 | 0 |
| Path 259 | C00074->C00079:[7->2,7->5,7->8] | 0.33 | 444.054794521 | 25 | 73 | 0 | 1 |
| Path 260 | C00074->C00079:[6->5,6->8] | 0.22 | 482.652173913 | 17 | 46 | 0 | 0 |
| Path 261 | C00074->C00079:[7->2,7->5,7->8] | 0.33 | 376.811594203 | 21 | 69 | 0 | 1 |
| Path 262 | C00074->C00079:[7->2,7->5,7->8] | 0.33 | 454.323529412 | 24 | 68 | 0 | 1 |
| Path 263 | C00074->C00079:[7->2,7->5,7->8] | 0.33 | 428.6 | 21 | 65 | 0 | 0 |
| Path 264 | C00074->C00079:[7->5,7->8] | 0.22 | 440.952380952 | 20 | 63 | 0 | 1 |
| Path 265 | C00074->C00079:[7->2,7->5,7->8] | 0.33 | 467.947368421 | 21 | 57 | 0 | 1 |
| Path 266 | C00074->C00079:[7->2,7->5,7->8] | 0.33 | 460.594202899 | 25 | 69 | 0 | 1 |
| Path 267 | C00074->C00079:[7->5,7->8] | 0.22 | 370.346153846 | 21 | 78 | 0 | 1 |
| Path 268 | C00074->C00079:[7->2,7->5,7->8] | 0.33 | 475.360655738 | 24 | 61 | 0 | 1 |
| Path 269 | C00074->C00079:[7->5,7->8] | 0.22 | 373.558823529 | 16 | 68 | 0 | 1 |
| Path 270 | C00074->C00079:[7->2,7->5,7->8] | 0.33 | 417.424242424 | 21 | 66 | 0 | 1 |
| Path 271 | C00074->C00079:[7->5,7->8] | 0.22 | 455.906976744 | 17 | 43 | 0 | 0 |
| Path 272 | C00074->C00079:[7->2,7->5,7->8] | 0.33 | 457.876712329 | 27 | 73 | 0 | 1 |
| Path 273 | C00074->C00079:[7->5,7->8] | 0.22 | 478.763636364 | 19 | 55 | 0 | 1 |
| Path 274 | C00074->C00079:[7->2,7->5,7->8] | 0.33 | 423.982758621 | 18 | 58 | 0 | 1 |
| Path 275 | C00074->C00079:[7->2,7->5,7->8] | 0.33 | 498.822580645 | 23 | 62 | 0 | 0 |
| Path 276 | C00074->C00079:[7->2,7->5,7->8] | 0.33 | 413.0 | 21 | 66 | 0 | 1 |
| Path 277 | C00279->C00079:[10->7,6->3,7->6,8->10], C00074->C00079:[6->1,6->5,7->8,8->2,8->4] | 1.00 | 588.339622642 | 21 | 53 | 0 | 0 |
| Path 278 | C00074->C00079:[7->2,7->5,7->8] | 0.33 | 381.580645161 | 28 | 93 | 0 | 1 |
| Path 279 | C00074->C00079:[7->5,7->8] | 0.22 | 452.450980392 | 13 | 51 | 0 | 0 |
| Path 280 | C00074->C00079:[7->5,7->8] | 0.22 | 431.829268293 | 10 | 41 | 0 | 1 |
| Path 281 | C00074->C00079:[7->5,7->8] | 0.22 | 467.789473684 | 18 | 57 | 0 | 0 |
| Path 282 | C00074->C00079:[7->5,7->8] | 0.22 | 470.745098039 | 16 | 51 | 0 | 1 |
| Path 283 | C00074->C00079:[7->2,7->5,7->8] | 0.33 | 463.0 | 23 | 64 | 0 | 0 |
| Path 284 | C00279->C00079:[6->5,6->8] | 0.22 | 363.882352941 | 15 | 34 | 0 | 1 |
| Path 285 | C00279->C00079:[6->5,6->8] | 0.22 | 430.435897436 | 18 | 39 | 0 | 0 |
| Path 286 | C00074->C00079:[6->8] | 0.11 | 346.4 | 10 | 50 | 0 | 1 |
| Path 287 | C00074->C00079:[7->2,7->5,7->8] | 0.33 | 372.166666667 | 19 | 66 | 0 | 1 |
| Path 288 | C00074->C00079:[6->5,6->8] | 0.22 | 436.163636364 | 19 | 55 | 0 | 1 |
| Path 289 | C00279->C00079:[6->5,6->8] | 0.22 | 407.115384615 | 14 | 26 | 0 | 1 |
| Path 290 | C00074->C00079:[7->2,7->5,7->8] | 0.33 | 452.712121212 | 25 | 66 | 0 | 1 |
| Path 291 | C00074->C00079:[7->5,7->8] | 0.22 | 385.333333333 | 17 | 54 | 0 | 0 |
| Path 292 | C00074->C00079:[7->2,7->5,7->8] | 0.33 | 445.175675676 | 24 | 74 | 0 | 0 |
| Path 293 | C00074->C00079:[7->2,7->5,7->8] | 0.33 | 430.828125 | 21 | 64 | 0 | 1 |
| Path 294 | C00074->C00079:[7->5,7->8] | 0.22 | 439.411764706 | 15 | 51 | 0 | 0 |
| Path 295 | C00074->C00079:[7->2,7->5,7->8] | 0.33 | 396.470588235 | 27 | 85 | 0 | 1 |
| Path 296 | C00074->C00079:[7->5,7->8] | 0.22 | 455.355555556 | 19 | 45 | 0 | 1 |
| Path 297 | C00074->C00079:[7->5,7->8] | 0.22 | 394.697674419 | 14 | 43 | 0 | 1 |
| Path 298 | C00074->C00079:[7->2,7->5,7->8] | 0.33 | 424.920634921 | 24 | 63 | 0 | 1 |
| Path 299 | C00074->C00079:[6->5,6->8] | 0.22 | 416.482758621 | 20 | 58 | 0 | 1 |
| Path 300 | C00074->C00079:[6->5,6->8,8->2] | 0.33 | 379.309090909 | 17 | 55 | 0 | 3 |
| Path 301 | C00074->C00079:[7->2,7->5,7->8] | 0.33 | 418.948275862 | 18 | 58 | 0 | 1 |
| Path 302 | C00074->C00079:[7->2,7->5,7->8] | 0.33 | 456.26984127 | 22 | 63 | 0 | 0 |
| Path 303 | C00074->C00079:[7->5,7->8] | 0.22 | 425.019230769 | 15 | 52 | 0 | 1 |
| Path 304 | C00074->C00079:[7->2,7->5,7->8] | 0.33 | 422.216666667 | 22 | 60 | 0 | 1 |
| Path 305 | C00074->C00079:[7->5,7->8] | 0.22 | 411.365384615 | 19 | 52 | 0 | 1 |
| Path 306 | C00074->C00079:[7->5,7->8] | 0.22 | 371.230769231 | 11 | 26 | 0 | 0 |
| Path 307 | C00074->C00079:[7->2,7->5,7->8] | 0.33 | 430.132352941 | 23 | 68 | 0 | 1 |
| Path 308 | C00074->C00079:[6->5,6->8] | 0.22 | 491.255319149 | 18 | 47 | 0 | 0 |
| Path 309 | C00074->C00079:[7->2,7->5,7->8] | 0.33 | 422.106060606 | 22 | 66 | 0 | 1 |
| Path 310 | C00074->C00079:[7->2,7->5,7->8] | 0.33 | 429.372881356 | 21 | 59 | 0 | 0 |
| Path 311 | C00074->C00079:[7->2,7->5,7->8] | 0.33 | 476.303571429 | 20 | 56 | 0 | 0 |
| Path 312 | C00074->C00079:[7->2,7->5,7->8] | 0.33 | 434.676470588 | 24 | 68 | 0 | 1 |
| Path 313 | C00074->C00079:[7->2,7->5,7->8] | 0.33 | 432.512195122 | 21 | 41 | 0 | 0 |
| Path 314 | C00074->C00079:[6->5,6->8] | 0.22 | 481.020833333 | 19 | 48 | 0 | 1 |
| Path 315 | C00074->C00079:[7->2,7->5,7->8] | 0.33 | 456.457627119 | 22 | 59 | 0 | 1 |
| Path 316 | C00074->C00079:[7->5,7->8] | 0.22 | 377.657142857 | 20 | 70 | 0 | 1 |
| Path 317 | C00074->C00079:[7->2,7->5,7->8] | 0.33 | 388.658823529 | 27 | 85 | 0 | 1 |
| Path 318 | C00074->C00079:[7->5,7->8] | 0.22 | 431.216216216 | 18 | 37 | 0 | 1 |
| Path 319 | C00074->C00079:[7->2,7->5,7->8] | 0.33 | 468.5 | 23 | 60 | 0 | 1 |
| Path 320 | C00279->C00079:[6->5,6->8] | 0.22 | 441.85 | 19 | 40 | 0 | 0 |
| Path 321 | C00074->C00079:[6->8] | 0.11 | 314.821428571 | 9 | 28 | 0 | 1 |
| Path 322 | C00074->C00079:[7->5,7->8] | 0.22 | 443.5 | 18 | 50 | 0 | 1 |
| Path 323 | C00074->C00079:[7->5,7->8] | 0.22 | 426.929824561 | 14 | 57 | 0 | 0 |
| Path 324 | C00074->C00079:[7->2,7->5,7->8] | 0.33 | 465.614285714 | 26 | 70 | 0 | 1 |
| Path 325 | C00074->C00079:[7->2,7->5,7->8] | 0.33 | 478.253731343 | 24 | 67 | 0 | 0 |
| Path 326 | C00074->C00079:[7->2,7->5,7->8] | 0.33 | 492.459016393 | 22 | 61 | 0 | 0 |
| Path 327 | C00074->C00079:[7->5,7->8] | 0.22 | 462.08 | 15 | 50 | 0 | 1 |
| Path 328 | C00074->C00079:[7->2,7->5,7->8] | 0.33 | 455.485714286 | 28 | 70 | 0 | 1 |
| Path 329 | C00074->C00079:[7->5,7->8] | 0.22 | 422.491803279 | 17 | 61 | 0 | 1 |
| Path 330 | C00074->C00079:[7->2,7->5,7->8] | 0.33 | 468.836363636 | 19 | 55 | 0 | 0 |
| Path 331 | C00074->C00079:[7->2,7->5,7->8] | 0.33 | 454.671428571 | 24 | 70 | 0 | 1 |
| Path 332 | C00074->C00079:[7->2,7->5,7->8] | 0.33 | 373.279069767 | 27 | 86 | 0 | 1 |
| Path 333 | C00074->C00079:[7->2,7->5,7->8] | 0.33 | 452.474576271 | 19 | 59 | 0 | 0 |
| Path 334 | C00074->C00079:[7->5,7->8] | 0.22 | 443.210526316 | 19 | 38 | 0 | 1 |
| Path 335 | C00074->C00079:[7->5,7->8] | 0.22 | 463.033333333 | 20 | 60 | 0 | 0 |
| Path 336 | C00074->C00079:[7->5,7->8] | 0.22 | 443.75 | 14 | 52 | 0 | 1 |
| Path 337 | C00074->C00079:[7->5,7->8] | 0.22 | 467.212765957 | 13 | 47 | 0 | 1 |
| Path 338 | C00074->C00079:[6->8] | 0.11 | 354.023809524 | 9 | 42 | 0 | 1 |
| Path 339 | C00074->C00079:[7->5,7->8] | 0.22 | 419.568965517 | 15 | 58 | 0 | 1 |
| Path 340 | C00074->C00079:[7->5,7->8] | 0.22 | 440.15625 | 11 | 32 | 0 | 1 |
| Path 341 | C00074->C00079:[7->5,7->8] | 0.22 | 428.272727273 | 18 | 55 | 0 | 1 |
| Path 342 | C00074->C00079:[7->2,7->5,7->8] | 0.33 | 434.5625 | 20 | 64 | 0 | 0 |
| Path 343 | C00074->C00079:[7->5,7->8] | 0.22 | 471.203703704 | 18 | 54 | 0 | 1 |
| Path 344 | C00074->C00079:[6->8] | 0.11 | 431.3125 | 8 | 32 | 0 | 1 |
| Path 345 | C00074->C00079:[7->2,7->5,7->8] | 0.33 | 427.876923077 | 21 | 65 | 0 | 1 |
| Path 346 | C00074->C00079:[7->2,7->5,7->8] | 0.33 | 417.646153846 | 23 | 65 | 0 | 1 |
| Path 347 | C00074->C00079:[7->5,7->8] | 0.22 | 428.409090909 | 12 | 44 | 0 | 1 |
| Path 348 | C00074->C00079:[7->2,7->5,7->8] | 0.33 | 464.327586207 | 21 | 58 | 0 | 0 |
