# Supplementary material for: 13C-metabolic flux ratio and novel carbon path analyses confirmed that Trichoderma reesei uses primarily the respirative pathway also on the preferred carbon source glucose
Source: BMC Syst Biol. 2009 Oct 29;3:104. doi: 10.1186/1752-0509-3-104 (PMC2776023; doi:10.1186/1752-0509-3-104)
Supplement: Additional file 1 — Pathways discovered in ReTrace carbon path analysis. Graphical and tabular representations of amino acid synthesis pathways discovered in ReTrace carbon path analysis [21]. Self-contained web site: unpack zip archive and open index.html with a web browser. [file 1752-0509-3-104-S1.zip › AF1-treesei/pathways-C00031-to-C00082.html]

Pathways from C00031 to C00082


**Pathways from C00031 to C00082**

**Sources:** D-Glucose; (C00031)

**Target:**L-Tyrosine; (C00082)

|  | Composite mapping | Z | Average score | Rpairs | Reactions | Zero scores | Scores under threshold |
| --- | --- | --- | --- | --- | --- | --- | --- |
| Path 1 | C00031->C00082:[4->1,4->2] | 0.22 | 271.762711864 | 23 | 59 | 1 | 2 |
| Path 2 | C00031->C00082:[9->2] | 0.11 | 227.341463415 | 10 | 41 | 0 | 0 |
| Path 3 | C00031->C00082:[4->2] | 0.11 | 380.642857143 | 7 | 14 | 0 | 0 |
| Path 4 | C00031->C00082:[4->1,4->2] | 0.22 | 328.36 | 16 | 25 | 1 | 2 |
| Path 5 | C00031->C00082:[4->1,4->2,7->1,7->2] | 0.22 | 288.435483871 | 26 | 62 | 1 | 2 |
| Path 6 | C00031->C00082:[4->2] | 0.11 | 348.428571429 | 8 | 14 | 0 | 1 |
| Path 7 | C00031->C00082:[4->1,4->2] | 0.22 | 262.218181818 | 21 | 55 | 1 | 2 |
| Path 8 | C00031->C00082:[4->1,4->2] | 0.22 | 286.873015873 | 25 | 63 | 1 | 2 |
| Path 9 | C00031->C00082:[4->1,4->2] | 0.22 | 351.464285714 | 17 | 28 | 1 | 2 |
