# Supplementary material for: 13C-metabolic flux ratio and novel carbon path analyses confirmed that Trichoderma reesei uses primarily the respirative pathway also on the preferred carbon source glucose
Source: BMC Syst Biol. 2009 Oct 29;3:104. doi: 10.1186/1752-0509-3-104 (PMC2776023; doi:10.1186/1752-0509-3-104)
Supplement: Additional file 1 — Pathways discovered in ReTrace carbon path analysis. Graphical and tabular representations of amino acid synthesis pathways discovered in ReTrace carbon path analysis [21]. Self-contained web site: unpack zip archive and open index.html with a web browser. [file 1752-0509-3-104-S1.zip › AF1-treesei/pathways-C00117-to-C00002.html]

Pathways from C00117 to C00002


**Pathways from C00117 to C00002**

**Sources:** D-Ribose 5-phosphate; (C00117)

**Target:**ATP; (C00002)

|  | Composite mapping | Z | Average score | Rpairs | Reactions | Zero scores | Scores under threshold |
| --- | --- | --- | --- | --- | --- | --- | --- |
| Path 1 | C00117->C00002:[10->8,12->3,6->17,7->12,8->13] | 0.50 | 309.265895954 | 10 | 346 | 0 | 0 |
| Path 2 | C00117->C00002:[10->8,12->3,6->17,7->12,8->13] | 0.50 | 682.733333333 | 7 | 15 | 0 | 0 |
| Path 3 | C00117->C00002:[10->8,12->3,6->17,7->12,8->13] | 0.50 | 940.2 | 5 | 5 | 0 | 0 |
| Path 4 | C00117->C00002:[10->8,12->3,6->17,7->12,8->13] | 0.50 | 520.526315789 | 6 | 19 | 0 | 0 |
| Path 5 | C00117->C00002:[10->8,12->3,6->17,7->12,8->13] | 0.50 | 271.608695652 | 7 | 46 | 0 | 0 |
| Path 6 | C00117->C00002:[10->8,12->3,6->17,7->12,8->13] | 0.50 | 668.714285714 | 6 | 14 | 0 | 0 |
| Path 7 | C00117->C00002:[10->8,12->3,6->11,6->17,7->12,8->13] | 0.60 | 562.7 | 21 | 30 | 0 | 0 |
| Path 8 | C00117->C00002:[10->8,12->3,6->10,6->11,6->17,7->12,8->13] | 0.70 | 200.7090301 | 24 | 299 | 0 | 0 |
| Path 9 | C00117->C00002:[10->11,10->8,12->3,6->17,7->12,8->13] | 0.60 | 347.244274809 | 18 | 131 | 0 | 1 |
| Path 10 | C00117->C00002:[10->8,12->3,6->10,6->11,6->17,6->2,6->4,7->12,8->13] | 0.90 | 372.696969697 | 35 | 165 | 0 | 1 |
| Path 11 | C00117->C00002:[10->8,12->3,6->11,6->17,7->12,8->13] | 0.60 | 359.26056338 | 19 | 142 | 0 | 0 |
| Path 12 | C00117->C00002:[10->8,12->3,6->10,6->11,6->17,6->2,6->4,7->12,8->13] | 0.90 | 626.270833333 | 35 | 48 | 0 | 1 |
| Path 13 | C00117->C00002:[10->8,12->3,6->10,6->11,6->17,7->12,8->13] | 0.70 | 380.813333333 | 25 | 150 | 0 | 0 |
| Path 14 | C00117->C00002:[10->8,12->3,6->10,6->11,6->17,6->2,6->4,7->12,8->13] | 0.90 | 224.798722045 | 36 | 313 | 0 | 1 |
| Path 15 | C00117->C00002:[10->8,12->3,6->11,6->17,7->12,8->13] | 0.60 | 357.62962963 | 17 | 135 | 0 | 0 |
| Path 16 | C00117->C00002:[10->8,12->3,6->11,6->17,7->12,8->13] | 0.60 | 357.028169014 | 19 | 142 | 0 | 0 |
| Path 17 | C00117->C00002:[10->8,12->3,6->10,6->11,6->17,7->12,8->13] | 0.70 | 203.276094276 | 25 | 297 | 0 | 0 |
| Path 18 | C00117->C00002:[10->8,12->3,6->10,6->11,6->17,7->12,8->13] | 0.70 | 382.951724138 | 24 | 145 | 0 | 0 |
| Path 19 | C00117->C00002:[10->8,12->3,6->10,6->11,6->17,6->2,6->4,7->12,8->13] | 0.90 | 400.968553459 | 34 | 159 | 0 | 1 |
| Path 20 | C00117->C00002:[10->8,12->3,6->17,7->12,8->13] | 0.50 | 314.700854701 | 4 | 117 | 0 | 0 |
| Path 21 | C00117->C00002:[10->8,12->3,6->10,6->11,6->17,7->12,8->13] | 0.70 | 349.369127517 | 23 | 149 | 0 | 0 |
| Path 22 | C00117->C00002:[10->8,12->3,6->11,6->17,7->12,8->13] | 0.60 | 382.368794326 | 20 | 141 | 0 | 0 |
| Path 23 | C00117->C00002:[10->11] | 0.10 | 376.773049645 | 14 | 141 | 0 | 0 |
| Path 24 | C00117->C00002:[10->8,12->11,12->3,6->17,7->12,8->13] | 0.60 | 515.548387097 | 19 | 31 | 0 | 0 |
| Path 25 | C00117->C00002:[10->11,10->4,10->8,12->3,6->10,6->11,6->17,6->2,6->4,7->12,8->13] | 0.90 | 238.358433735 | 41 | 332 | 0 | 1 |
| Path 26 | C00117->C00002:[10->8,12->3,6->10,6->11,6->17,7->12,8->13] | 0.70 | 352.774834437 | 25 | 151 | 0 | 0 |
| Path 27 | C00117->C00002:[10->11,10->8,12->3,6->17,7->12,8->13] | 0.60 | 386.677852349 | 20 | 149 | 0 | 0 |
| Path 28 | C00117->C00002:[10->8,12->3,6->10,6->11,6->17,6->2,6->4,7->12,8->13] | 0.90 | 373.576687117 | 33 | 163 | 0 | 2 |
| Path 29 | C00117->C00002:[10->11,10->8,12->11,12->3,6->10,6->17,7->12,8->13] | 0.70 | 366.440251572 | 29 | 159 | 0 | 0 |
| Path 30 | C00117->C00002:[10->8,12->3,6->10,6->11,6->17,7->12,8->13] | 0.70 | 207.81270903 | 26 | 299 | 0 | 0 |
| Path 31 | C00117->C00002:[10->8,12->3,6->10,6->11,6->17,6->2,6->4,7->12,8->13] | 0.90 | 220.575562701 | 35 | 311 | 0 | 1 |
| Path 32 | C00117->C00002:[10->8,12->3,6->11,6->17,7->12,8->13] | 0.60 | 357.560283688 | 18 | 141 | 0 | 1 |
| Path 33 | C00117->C00002:[10->8,12->3,6->10,6->11,6->17,6->2,6->4,7->12,8->13] | 0.90 | 220.479365079 | 36 | 315 | 0 | 1 |
| Path 34 | C00117->C00002:[10->8,12->3,6->10,6->11,6->17,7->12,8->13] | 0.70 | 634.25 | 20 | 28 | 0 | 0 |
| Path 35 | C00117->C00002:[10->8,12->3,6->10,6->11,6->17,6->2,6->4,7->12,8->13] | 0.90 | 395.036809816 | 35 | 163 | 0 | 1 |
| Path 36 | C00117->C00002:[10->8,12->3,6->10,6->11,6->17,6->2,6->4,7->12,8->13] | 0.90 | 566.29787234 | 34 | 47 | 0 | 1 |
| Path 37 | C00117->C00002:[10->8,12->3,6->10,6->11,6->17,6->2,6->4,7->12,8->13] | 0.90 | 524.58490566 | 35 | 53 | 0 | 1 |
| Path 38 | C00117->C00002:[10->12,10->13,10->17] | 0.30 | 395.241134752 | 22 | 141 | 0 | 0 |
| Path 39 | C00117->C00002:[10->8,12->3,6->10,6->11,6->17,7->12,8->13] | 0.70 | 642.205882353 | 25 | 34 | 0 | 0 |
| Path 40 | C00117->C00002:[10->8,12->3,6->10,6->11,6->17,6->2,6->4,7->12,8->13] | 0.90 | 377.502994012 | 36 | 167 | 0 | 1 |
| Path 41 | C00117->C00002:[10->8,12->3,6->10,6->11,6->17,6->2,6->4,7->12,8->13] | 0.90 | 376.557575758 | 34 | 165 | 0 | 1 |
| Path 42 | C00117->C00002:[10->8,12->11,12->3,6->17,7->12,8->13] | 0.60 | 529.482758621 | 18 | 29 | 0 | 0 |
| Path 43 | C00117->C00002:[10->8,12->3,6->10,6->11,6->17,7->12,8->13] | 0.70 | 203.405315615 | 26 | 301 | 0 | 0 |
| Path 44 | C00117->C00002:[10->8,12->3,6->10,6->11,6->17,7->12,8->13] | 0.70 | 562.722222222 | 26 | 36 | 0 | 0 |
| Path 45 | C00117->C00002:[10->8,12->3,6->10,6->11,6->17,6->2,6->4,7->12,8->13] | 0.90 | 220.162420382 | 35 | 314 | 0 | 1 |
| Path 46 | C00117->C00002:[10->11,10->4,10->8,12->3,6->10,6->11,6->17,6->2,6->4,7->12,8->13] | 0.90 | 407.346590909 | 38 | 176 | 0 | 1 |
| Path 47 | C00117->C00002:[10->8,12->3,6->10,6->11,6->17,7->12,8->13] | 0.70 | 642.323529412 | 25 | 34 | 0 | 0 |
| Path 48 | C00117->C00002:[10->8,12->3,6->10,6->11,6->17,6->2,6->4,7->12,8->13] | 0.90 | 369.828220859 | 33 | 163 | 0 | 1 |
| Path 49 | C00117->C00002:[10->8,12->3,6->10,6->11,6->17,7->12,8->13] | 0.70 | 541.028571429 | 24 | 35 | 0 | 0 |
| Path 50 | C00117->C00002:[10->8,12->3,6->11,6->17,7->12,8->13] | 0.60 | 372.625 | 20 | 144 | 0 | 0 |
| Path 51 | C00117->C00002:[10->8,12->3,6->10,6->11,6->17,7->12,8->13] | 0.70 | 634.392857143 | 20 | 28 | 0 | 0 |
| Path 52 | C00117->C00002:[10->11,10->4,10->8,12->3,6->10,6->17,7->12,8->13] | 0.80 | 408.460606061 | 33 | 165 | 0 | 0 |
| Path 53 | C00117->C00002:[10->8,12->3,6->11,6->17,7->12,8->13] | 0.60 | 587.129032258 | 21 | 31 | 0 | 0 |
| Path 54 | C00117->C00002:[10->8,12->3,6->10,6->11,6->17,6->2,6->4,7->12,8->13] | 0.90 | 223.971608833 | 37 | 317 | 0 | 1 |
| Path 55 | C00117->C00002:[10->8,12->3,6->10,6->11,6->17,6->2,6->4,7->12,8->13] | 0.90 | 400.968553459 | 34 | 159 | 0 | 1 |
| Path 56 | C00117->C00002:[10->10,10->11,10->2,10->4,10->8,12->10,12->11,12->2,12->3,12->4,6->10,6->17,6->2,7->12,8->13] | 0.90 | 229.611620795 | 41 | 327 | 0 | 1 |
| Path 57 | C00117->C00002:[10->12,10->13,12->17] | 0.30 | 393.034965035 | 20 | 143 | 0 | 0 |
| Path 58 | C00117->C00002:[10->11,10->8,12->11,12->3,6->10,6->17,7->12,8->13] | 0.70 | 214.30420712 | 30 | 309 | 0 | 0 |
| Path 59 | C00117->C00002:[10->8,12->3,6->10,6->11,6->17,7->12,8->13] | 0.70 | 370.158273381 | 19 | 139 | 0 | 0 |
| Path 60 | C00117->C00002:[10->11,10->8,12->3,6->17,7->12,8->13] | 0.60 | 553.432432432 | 20 | 37 | 0 | 0 |
| Path 61 | C00117->C00002:[10->13,10->17] | 0.20 | 397.186206897 | 20 | 145 | 0 | 0 |
| Path 62 | C00117->C00002:[10->8,12->10,12->11,12->2,12->3,12->4,6->10,6->17,6->2,7->12,8->13] | 0.90 | 380.0 | 39 | 175 | 0 | 1 |
| Path 63 | C00117->C00002:[10->8,12->3,6->10,6->11,6->17,7->12,8->13] | 0.70 | 356.993377483 | 24 | 151 | 0 | 0 |
| Path 64 | C00117->C00002:[10->11,10->8,12->3,6->17,7->12,8->13] | 0.60 | 362.66 | 19 | 150 | 0 | 0 |
| Path 65 | C00117->C00002:[10->8,12->3,6->10,6->11,6->17,7->12,8->13] | 0.70 | 387.810810811 | 26 | 148 | 0 | 0 |
| Path 66 | C00117->C00002:[10->8,12->3,6->11,6->17,7->12,8->13] | 0.60 | 674.033333333 | 21 | 30 | 0 | 0 |
| Path 67 | C00117->C00002:[10->8,12->11,12->3,6->17,7->12,8->13] | 0.60 | 347.021126761 | 17 | 142 | 0 | 0 |
| Path 68 | C00117->C00002:[10->11,10->4,10->8,12->3,6->10,6->17,6->2,7->12,8->13] | 0.90 | 416.711864407 | 40 | 177 | 0 | 1 |
| Path 69 | C00117->C00002:[10->8,12->3,6->17,7->12,8->13] | 0.50 | 348.5859375 | 12 | 128 | 0 | 0 |
| Path 70 | C00117->C00002:[10->8,12->3,6->10,6->11,6->17,7->12,8->13] | 0.70 | 205.521594684 | 25 | 301 | 0 | 0 |
| Path 71 | C00117->C00002:[10->8,12->3,6->10,6->11,6->17,6->2,6->4,7->12,8->13] | 0.90 | 218.012779553 | 34 | 313 | 0 | 1 |
| Path 72 | C00117->C00002:[10->12,10->13,10->17] | 0.30 | 396.79020979 | 20 | 143 | 0 | 0 |
| Path 73 | C00117->C00002:[10->8,12->11,12->3,12->4,6->10,6->17,6->2,7->12,8->13] | 0.90 | 550.706896552 | 39 | 58 | 0 | 1 |
| Path 74 | C00117->C00002:[10->8,12->3,6->10,6->11,6->17,6->2,6->4,7->12,8->13] | 0.90 | 626.354166667 | 35 | 48 | 0 | 1 |
| Path 75 | C00117->C00002:[10->11,10->8,12->3,6->17,7->12,8->13] | 0.60 | 207.85 | 20 | 300 | 0 | 0 |
| Path 76 | C00117->C00002:[10->10,10->11,10->2,10->4,10->8,12->10,12->11,12->2,12->3,12->4,6->10,6->17,6->2,7->12,8->13] | 0.90 | 399.676136364 | 41 | 176 | 0 | 1 |
| Path 77 | C00117->C00002:[10->8,12->10,12->11,12->2,12->3,12->4,6->10,6->17,6->2,7->12,8->13] | 0.90 | 400.666666667 | 40 | 174 | 0 | 1 |
| Path 78 | C00117->C00002:[6->10,6->11] | 0.20 | 382.775510204 | 28 | 147 | 0 | 1 |
| Path 79 | C00117->C00002:[10->11,10->4,10->8,12->3,6->10,6->17,6->2,7->12,8->13] | 0.90 | 563.384615385 | 40 | 65 | 0 | 1 |
| Path 80 | C00117->C00002:[10->8,12->3,6->10,6->11,6->17,6->2,6->4,7->12,8->13] | 0.90 | 556.6875 | 35 | 48 | 0 | 1 |
| Path 81 | C00117->C00002:[10->8,12->3,6->10,6->11,6->17,7->12,8->13] | 0.70 | 525.894736842 | 25 | 38 | 0 | 0 |
| Path 82 | C00117->C00002:[10->8,12->3,6->11,6->17,7->12,8->13] | 0.60 | 380.191780822 | 21 | 146 | 0 | 0 |
| Path 83 | C00117->C00002:[10->8,12->3,6->10,6->11,6->17,6->2,6->4,7->12,8->13] | 0.90 | 378.968553459 | 32 | 159 | 0 | 2 |
| Path 84 | C00117->C00002:[10->8,12->3,6->10,6->11,6->17,6->2,6->4,7->12,8->13] | 0.90 | 395.036809816 | 35 | 163 | 0 | 1 |
| Path 85 | C00117->C00002:[10->8,12->3,6->10,6->11,6->17,6->2,6->4,7->12,8->13] | 0.90 | 373.018292683 | 34 | 164 | 0 | 1 |
| Path 86 | C00117->C00002:[10->8,12->3,6->10,6->11,6->17,6->2,6->4,7->12,8->13] | 0.90 | 398.221556886 | 41 | 167 | 0 | 2 |
| Path 87 | C00117->C00002:[10->8,12->3,6->10,6->11,6->17,7->12,8->13] | 0.70 | 358.827586207 | 22 | 145 | 0 | 1 |
| Path 88 | C00117->C00002:[10->11,10->4,10->8,12->11,12->3,12->4,6->10,6->17,6->2,7->12,8->13] | 0.90 | 384.335260116 | 39 | 173 | 0 | 1 |
| Path 89 | C00117->C00002:[10->8,12->3,6->10,6->11,6->17,7->12,8->13] | 0.70 | 382.951724138 | 24 | 145 | 0 | 0 |
| Path 90 | C00117->C00002:[10->8,12->3,6->11,6->17,7->12,8->13] | 0.60 | 355.265734266 | 20 | 143 | 0 | 0 |
| Path 91 | C00117->C00002:[10->8,12->3,6->10,6->11,6->17,6->2,6->4,7->12,8->13] | 0.90 | 222.501587302 | 35 | 315 | 0 | 1 |
| Path 92 | C00117->C00002:[10->8,12->3,6->10,6->11,6->17,6->2,6->4,7->12,8->13] | 0.90 | 220.948387097 | 34 | 310 | 0 | 1 |
| Path 93 | C00117->C00002:[10->8,12->3,6->17,7->12,8->13] | 0.50 | 521.166666667 | 5 | 6 | 0 | 0 |
| Path 94 | C00117->C00002:[10->12,10->13,12->17] | 0.30 | 376.242647059 | 18 | 136 | 0 | 0 |
| Path 95 | C00117->C00002:[10->8,12->3,6->10,6->11,6->17,6->2,6->4,7->12,8->13] | 0.90 | 376.664596273 | 34 | 161 | 0 | 1 |
| Path 96 | C00117->C00002:[10->8,12->3,6->10,6->11,6->17,7->12,8->13] | 0.70 | 541.142857143 | 24 | 35 | 0 | 0 |
| Path 97 | C00117->C00002:[10->11,10->8,12->3,6->17,7->12,8->13] | 0.60 | 374.409722222 | 17 | 144 | 0 | 0 |
| Path 98 | C00117->C00002:[10->8,12->3,6->11,6->17,7->12,8->13] | 0.60 | 540.294117647 | 21 | 34 | 0 | 0 |
| Path 99 | C00117->C00002:[10->8,12->11,12->3,12->4,6->10,6->17,6->2,7->12,8->13] | 0.90 | 385.16374269 | 38 | 171 | 0 | 1 |
| Path 100 | C00117->C00002:[10->8,12->3,6->10,6->11,6->17,6->2,6->4,7->12,8->13] | 0.90 | 554.408163265 | 34 | 49 | 0 | 1 |
| Path 101 | C00117->C00002:[10->8,12->3,6->11,6->17,7->12,8->13] | 0.60 | 642.192307692 | 18 | 26 | 0 | 0 |
| Path 102 | C00117->C00002:[10->8,12->3,6->10,6->11,6->17,7->12,8->13] | 0.70 | 358.301369863 | 23 | 146 | 0 | 0 |
| Path 103 | C00117->C00002:[10->11,10->4,10->8,12->3,6->10,6->17,7->12,8->13] | 0.80 | 406.122580645 | 34 | 155 | 0 | 0 |
| Path 104 | C00117->C00002:[10->8,12->3,6->10,6->11,6->17,6->2,6->4,7->12,8->13] | 0.90 | 380.34375 | 33 | 160 | 0 | 1 |
| Path 105 | C00117->C00002:[10->11,6->17,7->12,8->13] | 0.40 | 403.422535211 | 25 | 142 | 0 | 0 |
| Path 106 | C00117->C00002:[10->8,12->3,6->10,6->11,6->17,7->12,8->13] | 0.70 | 634.392857143 | 20 | 28 | 0 | 0 |
| Path 107 | C00117->C00002:[10->8,12->3,6->10,6->11,6->17,6->2,6->4,7->12,8->13] | 0.90 | 534.37254902 | 35 | 51 | 0 | 1 |
| Path 108 | C00117->C00002:[10->8,12->3,6->17,7->12,8->13] | 0.50 | 559.0 | 6 | 7 | 0 | 0 |
| Path 109 | C00117->C00002:[10->8,12->3,6->11,6->17,7->12,8->13] | 0.60 | 558.903225806 | 20 | 31 | 0 | 0 |
| Path 110 | C00117->C00002:[10->11,10->4,10->8,12->3,6->10,6->17,6->2,7->12,8->13] | 0.90 | 413.11627907 | 37 | 172 | 0 | 1 |
| Path 111 | C00117->C00002:[10->8,12->3,6->11,6->17,7->12,8->13] | 0.60 | 674.166666667 | 21 | 30 | 0 | 0 |
| Path 112 | C00117->C00002:[10->8,12->3,6->10,6->11,6->17,7->12,8->13] | 0.70 | 543.970588235 | 25 | 34 | 0 | 0 |
| Path 113 | C00117->C00002:[10->8,12->3,6->10,6->11,6->17,6->2,6->4,7->12,8->13] | 0.90 | 539.277777778 | 37 | 54 | 0 | 1 |
| Path 114 | C00117->C00002:[10->8,12->3,6->11,6->17,7->12,8->13] | 0.60 | 347.875862069 | 19 | 145 | 0 | 0 |
| Path 115 | C00117->C00002:[10->8,12->3,6->11,6->17,7->12,8->13] | 0.60 | 382.368794326 | 20 | 141 | 0 | 0 |
| Path 116 | C00117->C00002:[10->13,10->17] | 0.20 | 407.324503311 | 24 | 151 | 0 | 0 |
| Path 117 | C00117->C00002:[10->8,12->10,12->11,12->2,12->3,12->4,6->10,6->17,6->2,7->12,8->13] | 0.90 | 229.095384615 | 40 | 325 | 0 | 1 |
| Path 118 | C00117->C00002:[10->8,12->3,6->10,6->11,6->17,6->2,6->4,7->12,8->13] | 0.90 | 526.115384615 | 36 | 52 | 0 | 1 |
| Path 119 | C00117->C00002:[10->8,12->3,6->10,6->11,6->17,7->12,8->13] | 0.70 | 356.578231293 | 24 | 147 | 0 | 0 |
| Path 120 | C00117->C00002:[10->8,12->3,6->10,6->11,6->17,7->12,8->13] | 0.70 | 381.047945205 | 25 | 146 | 0 | 0 |
| Path 121 | C00117->C00002:[10->11] | 0.10 | 349.728682171 | 16 | 129 | 0 | 1 |
| Path 122 | C00117->C00002:[10->11,10->4,10->8,12->3,6->10,6->17,6->2,6->4,7->12,8->13] | 0.90 | 405.096385542 | 39 | 166 | 0 | 1 |
| Path 123 | C00117->C00002:[10->8,12->3,6->10,6->11,6->17,6->2,6->4,7->12,8->13] | 0.90 | 530.25 | 36 | 52 | 0 | 1 |
| Path 124 | C00117->C00002:[10->8,12->3,6->10,6->11,6->17,6->2,6->4,7->12,8->13] | 0.90 | 554.408163265 | 34 | 49 | 0 | 1 |
| Path 125 | C00117->C00002:[12->13,12->17] | 0.20 | 399.993103448 | 24 | 145 | 0 | 0 |
| Path 126 | C00117->C00002:[10->8,12->3,6->17,7->12,8->13] | 0.50 | 312.25 | 3 | 116 | 0 | 1 |
| Path 127 | C00117->C00002:[10->8,12->3,6->10,6->11,6->17,6->2,6->4,7->12,8->13] | 0.90 | 554.326530612 | 34 | 49 | 0 | 1 |
| Path 128 | C00117->C00002:[10->11,10->8,12->3,6->10,6->17,7->12,8->13] | 0.70 | 223.796178344 | 30 | 314 | 0 | 0 |
| Path 129 | C00117->C00002:[10->11,6->17,7->12,8->13] | 0.40 | 406.138157895 | 24 | 152 | 0 | 0 |
| Path 130 | C00117->C00002:[10->13] | 0.10 | 393.264285714 | 17 | 140 | 0 | 0 |
| Path 131 | C00117->C00002:[10->8,12->3,6->10,6->11,6->17,6->2,6->4,7->12,8->13] | 0.90 | 524.58490566 | 35 | 53 | 0 | 1 |
| Path 132 | C00117->C00002:[10->8,12->3,6->11,6->17,7->12,8->13] | 0.60 | 361.965277778 | 20 | 144 | 0 | 0 |
| Path 133 | C00117->C00002:[10->8,12->11,12->3,6->10,6->17,7->12,8->13] | 0.70 | 213.657980456 | 29 | 307 | 0 | 0 |
| Path 134 | C00117->C00002:[10->11,10->8,12->3,6->17,7->12,8->13] | 0.60 | 355.630769231 | 16 | 130 | 0 | 0 |
| Path 135 | C00117->C00002:[10->8,12->10,12->11,12->2,12->3,12->4,6->10,6->17,6->2,7->12,8->13] | 0.90 | 525.451612903 | 40 | 62 | 0 | 1 |
| Path 136 | C00117->C00002:[10->11,10->4,10->8,12->3,6->10,6->17,6->2,7->12,8->13] | 0.90 | 396.303370787 | 39 | 178 | 0 | 1 |
| Path 137 | C00117->C00002:[10->8,12->3,6->10,6->11,6->17,7->12,8->13] | 0.70 | 360.47260274 | 23 | 146 | 0 | 0 |
| Path 138 | C00117->C00002:[10->8,12->11,12->3,12->4,6->10,6->17,6->2,7->12,8->13] | 0.90 | 406.347058824 | 39 | 170 | 0 | 1 |
| Path 139 | C00117->C00002:[10->8,12->3,6->11,6->17,7->12,8->13] | 0.60 | 516.25 | 20 | 32 | 0 | 0 |
| Path 140 | C00117->C00002:[10->8,12->3,6->11,6->17,7->12,8->13] | 0.60 | 578.482758621 | 20 | 29 | 0 | 0 |
| Path 141 | C00117->C00002:[10->8,12->3,6->11,6->17,7->12,8->13] | 0.60 | 558.903225806 | 20 | 31 | 0 | 0 |
| Path 142 | C00117->C00002:[10->11,10->4,10->8,12->3,6->10,6->17,6->2,7->12,8->13] | 0.90 | 411.166666667 | 38 | 162 | 0 | 1 |
| Path 143 | C00117->C00002:[10->8,12->11,12->3,6->10,6->17,7->12,8->13] | 0.70 | 390.083333333 | 29 | 156 | 0 | 0 |
| Path 144 | C00117->C00002:[10->8,12->11,12->3,6->10,6->17,7->12,8->13] | 0.70 | 538.977272727 | 29 | 44 | 0 | 0 |
| Path 145 | C00117->C00002:[10->8,12->3,6->10,6->11,6->17,7->12,8->13] | 0.70 | 202.69 | 25 | 300 | 0 | 0 |
| Path 146 | C00117->C00002:[10->8,12->3,6->10,6->11,6->17,6->2,6->4,7->12,8->13] | 0.90 | 391.962962963 | 34 | 162 | 0 | 1 |
| Path 147 | C00117->C00002:[10->8,12->3,6->10,6->11,6->17,7->12,8->13] | 0.70 | 541.142857143 | 24 | 35 | 0 | 0 |
| Path 148 | C00117->C00002:[10->11,6->10] | 0.20 | 393.819354839 | 24 | 155 | 0 | 0 |
| Path 149 | C00117->C00002:[10->8,12->3,6->17,7->12,8->13] | 0.50 | 166.407407407 | 6 | 270 | 0 | 0 |
| Path 150 | C00117->C00002:[10->12,12->13,12->17] | 0.30 | 411.688311688 | 28 | 154 | 0 | 0 |
| Path 151 | C00117->C00002:[10->8,12->3,6->10,6->11,6->17,6->2,6->4,7->12,8->13] | 0.90 | 378.3625 | 33 | 160 | 0 | 1 |
| Path 152 | C00117->C00002:[10->8,12->3,6->10,6->11,6->17,7->12,8->13] | 0.70 | 363.624161074 | 25 | 149 | 0 | 0 |
| Path 153 | C00117->C00002:[10->13] | 0.10 | 379.126865672 | 16 | 134 | 0 | 0 |
| Path 154 | C00117->C00002:[10->10,10->11,10->2,10->4,10->8,12->10,12->11,12->2,12->3,12->4,6->10,6->17,6->2,7->12,8->13] | 0.90 | 379.248587571 | 40 | 177 | 0 | 1 |
| Path 155 | C00117->C00002:[10->8,12->3,6->17,7->12,8->13] | 0.50 | 436.0 | 6 | 7 | 0 | 0 |
| Path 156 | C00117->C00002:[10->8,12->3,6->17,7->12,8->13] | 0.50 | 308.116666667 | 5 | 120 | 0 | 0 |
| Path 157 | C00117->C00002:[10->8,12->3,6->11,6->17,7->12,8->13] | 0.60 | 200.869863014 | 20 | 292 | 0 | 0 |
| Path 158 | C00117->C00002:[10->8,12->3,6->10,6->11,6->17,6->2,6->4,7->12,8->13] | 0.90 | 374.951219512 | 34 | 164 | 0 | 1 |
| Path 159 | C00117->C00002:[10->8,12->3,6->10,6->11,6->17,6->2,6->4,7->12,8->13] | 0.90 | 527.2 | 34 | 50 | 0 | 1 |
| Path 160 | C00117->C00002:[10->8,12->11,12->3,6->17,7->12,8->13] | 0.60 | 196.003424658 | 18 | 292 | 0 | 0 |
| Path 161 | C00117->C00002:[10->8,12->3,6->17,7->12,8->13] | 0.50 | 316.899159664 | 6 | 119 | 0 | 0 |
| Path 162 | C00117->C00002:[10->8,12->3,6->10,6->11,6->17,7->12,8->13] | 0.70 | 360.189781022 | 19 | 137 | 0 | 0 |
| Path 163 | C00117->C00002:[10->8,12->3,6->11,6->17,7->12,8->13] | 0.60 | 355.727891156 | 20 | 147 | 0 | 0 |
| Path 164 | C00117->C00002:[10->8,12->3,6->10,6->11,6->17,6->2,6->4,7->12,8->13] | 0.90 | 405.074074074 | 36 | 162 | 0 | 1 |
| Path 165 | C00117->C00002:[10->8,12->3,6->10,6->17,7->12,8->13] | 0.60 | 357.630136986 | 23 | 146 | 0 | 0 |
| Path 166 | C00117->C00002:[10->8,12->3,6->11,6->17,7->12,8->13] | 0.60 | 367.781021898 | 17 | 137 | 0 | 0 |
| Path 167 | C00117->C00002:[10->10,10->11,10->2,10->4,10->8,12->10,12->11,12->2,12->3,12->4,6->10,6->17,6->2,7->12,8->13] | 0.90 | 518.828125 | 41 | 64 | 0 | 1 |
| Path 168 | C00117->C00002:[10->11,10->8,12->3,6->10,6->17,7->12,8->13] | 0.70 | 379.975609756 | 29 | 164 | 0 | 0 |
| Path 169 | C00117->C00002:[10->8,12->3,6->17,7->12,8->13] | 0.50 | 521.833333333 | 5 | 6 | 0 | 0 |
| Path 170 | C00117->C00002:[10->8,12->3,6->11,6->17,7->12,8->13] | 0.60 | 558.774193548 | 20 | 31 | 0 | 0 |
| Path 171 | C00117->C00002:[10->8,12->3,6->10,6->11,6->17,6->2,6->4,7->12,8->13] | 0.90 | 569.68 | 36 | 50 | 0 | 1 |
| Path 172 | C00117->C00002:[10->8,12->3,6->10,6->11,6->17,6->2,6->4,7->12,8->13] | 0.90 | 590.423076923 | 36 | 52 | 0 | 1 |
| Path 173 | C00117->C00002:[10->8,12->3,6->17,7->12,8->13] | 0.50 | 559.571428571 | 6 | 7 | 0 | 0 |
| Path 174 | C00117->C00002:[10->8,12->3,6->10,6->11,6->17,7->12,8->13] | 0.70 | 368.264285714 | 20 | 140 | 0 | 0 |
| Path 175 | C00117->C00002:[10->8,12->11,12->3,6->17,7->12,8->13] | 0.60 | 372.290780142 | 18 | 141 | 0 | 0 |
| Path 176 | C00117->C00002:[10->8,12->3,6->10,6->11,6->17,6->2,6->4,7->12,8->13] | 0.90 | 590.423076923 | 36 | 52 | 0 | 1 |
| Path 177 | C00117->C00002:[10->12,10->13,12->17] | 0.30 | 390.302816901 | 19 | 142 | 0 | 0 |
| Path 178 | C00117->C00002:[10->8,12->3,6->10,6->11,6->17,6->2,6->4,7->12,8->13] | 0.90 | 371.393939394 | 35 | 165 | 0 | 1 |
| Path 179 | C00117->C00002:[10->11,10->4,10->8,12->11,12->3,12->4,6->10,6->17,6->2,7->12,8->13] | 0.90 | 542.8 | 40 | 60 | 0 | 1 |
| Path 180 | C00117->C00002:[10->8,12->3,6->11,6->17,7->12,8->13] | 0.60 | 197.959322034 | 20 | 295 | 0 | 0 |
| Path 181 | C00117->C00002:[10->8,12->3,6->17,7->12,8->13] | 0.50 | 324.313559322 | 6 | 118 | 0 | 0 |
| Path 182 | C00117->C00002:[10->11,10->8,12->3,6->10,6->17,7->12,8->13] | 0.70 | 402.036809816 | 30 | 163 | 0 | 0 |
| Path 183 | C00117->C00002:[10->8,12->3,6->10,6->11,6->17,6->2,6->4,7->12,8->13] | 0.90 | 404.085889571 | 40 | 163 | 0 | 2 |
| Path 184 | C00117->C00002:[10->11,10->4,10->8,12->3,6->10,6->17,6->2,7->12,8->13] | 0.90 | 239.323170732 | 40 | 328 | 0 | 1 |
| Path 185 | C00117->C00002:[10->8,12->11,12->3,6->17,7->12,8->13] | 0.60 | 371.468531469 | 19 | 143 | 0 | 0 |
| Path 186 | C00117->C00002:[10->11,10->8,12->11,12->3,6->10,6->17,7->12,8->13] | 0.70 | 389.113924051 | 30 | 158 | 0 | 0 |
| Path 187 | C00117->C00002:[10->11,10->4,10->8,12->3,6->10,6->11,6->17,6->2,6->4,7->12,8->13] | 0.90 | 391.093406593 | 40 | 182 | 0 | 1 |
| Path 188 | C00117->C00002:[10->8,12->3,6->10,6->11,6->17,7->12,8->13] | 0.70 | 511.594594595 | 25 | 37 | 0 | 0 |
| Path 189 | C00117->C00002:[10->8,12->3,6->17,7->12,8->13] | 0.50 | 318.694915254 | 5 | 118 | 0 | 0 |
| Path 190 | C00117->C00002:[10->8,12->3,6->10,6->17,7->12,8->13] | 0.60 | 203.277027027 | 24 | 296 | 0 | 0 |
| Path 191 | C00117->C00002:[10->8,12->3,6->11,6->17,7->12,8->13] | 0.60 | 365.876811594 | 18 | 138 | 0 | 0 |
| Path 192 | C00117->C00002:[10->8,12->3,6->10,6->11,6->17,7->12,8->13] | 0.70 | 642.323529412 | 25 | 34 | 0 | 0 |
| Path 193 | C00117->C00002:[10->11,10->8,12->3,6->10,6->17,7->12,8->13] | 0.70 | 556.745098039 | 30 | 51 | 0 | 0 |
| Path 194 | C00117->C00002:[10->11,6->10] | 0.20 | 390.310344828 | 25 | 145 | 0 | 0 |
| Path 195 | C00117->C00002:[10->8,12->3,6->11,6->17,7->12,8->13] | 0.60 | 642.038461538 | 18 | 26 | 0 | 0 |
| Path 196 | C00117->C00002:[10->11,10->4,10->8,12->3,6->10,6->11,6->17,6->2,6->4,7->12,8->13] | 0.90 | 411.022099448 | 41 | 181 | 0 | 1 |
| Path 197 | C00117->C00002:[10->8,12->3,6->10,6->11,6->17,6->2,6->4,7->12,8->13] | 0.90 | 219.796825397 | 36 | 315 | 0 | 1 |
| Path 198 | C00117->C00002:[10->8,12->3,6->10,6->11,6->17,6->2,6->4,7->12,8->13] | 0.90 | 399.15060241 | 37 | 166 | 0 | 1 |
| Path 199 | C00117->C00002:[10->8,12->3,6->10,6->11,6->17,6->2,6->4,7->12,8->13] | 0.90 | 626.354166667 | 35 | 48 | 0 | 1 |
| Path 200 | C00117->C00002:[10->8,12->3,6->10,6->11,6->17,6->2,6->4,7->12,8->13] | 0.90 | 524.509433962 | 35 | 53 | 0 | 1 |
| Path 201 | C00117->C00002:[10->11,10->4,10->8,12->11,12->3,12->4,6->10,6->17,6->2,7->12,8->13] | 0.90 | 230.482972136 | 40 | 323 | 0 | 1 |
| Path 202 | C00117->C00002:[10->8,12->3,6->10,6->11,6->17,6->2,6->4,7->12,8->13] | 0.90 | 398.463414634 | 35 | 164 | 0 | 1 |
| Path 203 | C00117->C00002:[10->8,12->3,6->10,6->11,6->17,7->12,8->13] | 0.70 | 373.459459459 | 24 | 148 | 0 | 0 |
| Path 204 | C00117->C00002:[10->8,12->3,6->10,6->11,6->17,7->12,8->13] | 0.70 | 503.722222222 | 24 | 36 | 0 | 0 |
| Path 205 | C00117->C00002:[10->8,12->3,6->10,6->11,6->17,7->12,8->13] | 0.70 | 203.608108108 | 24 | 296 | 0 | 0 |
| Path 206 | C00117->C00002:[10->11,10->8,12->11,12->3,6->10,6->17,7->12,8->13] | 0.70 | 529.173913043 | 30 | 46 | 0 | 0 |
| Path 207 | C00117->C00002:[10->8,12->3,6->10,6->11,6->17,7->12,8->13] | 0.70 | 557.272727273 | 24 | 33 | 0 | 0 |
| Path 208 | C00117->C00002:[10->8,12->3,6->10,6->11,6->17,6->2,6->4,7->12,8->13] | 0.90 | 542.5 | 35 | 52 | 0 | 1 |
| Path 209 | C00117->C00002:[10->11] | 0.10 | 357.811023622 | 13 | 127 | 0 | 0 |
| Path 210 | C00117->C00002:[12->13,12->17] | 0.20 | 412.271523179 | 25 | 151 | 0 | 0 |
| Path 211 | C00117->C00002:[10->8,12->3,6->11,6->17,7->12,8->13] | 0.60 | 204.350340136 | 21 | 294 | 0 | 0 |
| Path 212 | C00117->C00002:[10->8,12->11,12->3,6->10,6->17,7->12,8->13] | 0.70 | 367.114649682 | 28 | 157 | 0 | 0 |
| Path 213 | C00117->C00002:[10->8,12->3,6->10,6->11,6->17,7->12,8->13] | 0.70 | 509.131578947 | 26 | 38 | 0 | 0 |
| Path 214 | C00117->C00002:[10->8,12->3,6->11,6->17,7->12,8->13] | 0.60 | 380.415492958 | 21 | 142 | 0 | 0 |
| Path 215 | C00117->C00002:[10->11,10->4,10->8,12->3,6->10,6->11,6->17,6->2,6->4,7->12,8->13] | 0.90 | 539.956521739 | 41 | 69 | 0 | 1 |
| Path 216 | C00117->C00002:[10->8,12->3,6->11,6->17,7->12,8->13] | 0.60 | 642.192307692 | 18 | 26 | 0 | 0 |
| Path 217 | C00117->C00002:[10->8,12->3,6->17,7->12,8->13] | 0.50 | 521.833333333 | 5 | 6 | 0 | 0 |
| Path 218 | C00117->C00002:[10->8,12->3,6->10,6->17,7->12,8->13] | 0.60 | 554.303030303 | 24 | 33 | 0 | 0 |
| Path 219 | C00117->C00002:[10->8,12->3,6->11,6->17,7->12,8->13] | 0.60 | 202.855218855 | 21 | 297 | 0 | 0 |
| Path 220 | C00117->C00002:[10->8,12->11,12->3,6->17,7->12,8->13] | 0.60 | 346.555555556 | 18 | 144 | 0 | 0 |
| Path 221 | C00117->C00002:[10->11,10->4,10->8,12->11,12->3,12->4,6->10,6->17,6->2,7->12,8->13] | 0.90 | 405.26744186 | 40 | 172 | 0 | 1 |
| Path 222 | C00117->C00002:[10->8,12->3,6->10,6->11,6->17,6->2,6->4,7->12,8->13] | 0.90 | 393.268292683 | 36 | 164 | 0 | 1 |
| Path 223 | C00117->C00002:[10->8,12->3,6->10,6->11,6->17,7->12,8->13] | 0.70 | 352.34 | 24 | 150 | 0 | 0 |
| Path 224 | C00117->C00002:[10->8,12->3,6->10,6->11,6->17,6->2,6->4,7->12,8->13] | 0.90 | 590.346153846 | 36 | 52 | 0 | 1 |
| Path 225 | C00117->C00002:[10->8,12->11,12->3,6->17,7->12,8->13] | 0.60 | 196.802721088 | 19 | 294 | 0 | 0 |
| Path 226 | C00117->C00002:[10->8,12->3,6->11,6->17,7->12,8->13] | 0.60 | 674.166666667 | 21 | 30 | 0 | 0 |
| Path 227 | C00117->C00002:[10->8,12->3,6->17,7->12,8->13] | 0.50 | 559.571428571 | 6 | 7 | 0 | 0 |
| Path 228 | C00117->C00002:[10->8,12->3,6->10,6->11,6->17,6->2,6->4,7->12,8->13] | 0.90 | 382.858895706 | 35 | 163 | 0 | 1 |
| Path 229 | C00117->C00002:[10->8,12->3,6->11,6->17,7->12,8->13] | 0.60 | 386.986013986 | 21 | 143 | 0 | 0 |
| Path 230 | C00117->C00002:[10->8,12->11,12->3,12->4,6->10,6->17,6->2,7->12,8->13] | 0.90 | 229.965732087 | 39 | 321 | 0 | 1 |
| Path 231 | C00117->C00002:[10->8,12->3,6->10,6->11,6->17,6->4,7->12,8->13] | 0.80 | 398.756410256 | 36 | 156 | 0 | 1 |
| Path 232 | C00117->C00002:[10->8,12->3,6->11,6->17,7->12,8->13] | 0.60 | 200.542662116 | 21 | 293 | 0 | 0 |
| Path 233 | C00117->C00002:[10->8,12->3,6->10,6->11,6->17,6->2,6->4,7->12,8->13] | 0.90 | 399.11875 | 35 | 160 | 0 | 1 |
