# Supplementary material for: 13C-metabolic flux ratio and novel carbon path analyses confirmed that Trichoderma reesei uses primarily the respirative pathway also on the preferred carbon source glucose
Source: BMC Syst Biol. 2009 Oct 29;3:104. doi: 10.1186/1752-0509-3-104 (PMC2776023; doi:10.1186/1752-0509-3-104)
Supplement: Additional file 1 — Pathways discovered in ReTrace carbon path analysis. Graphical and tabular representations of amino acid synthesis pathways discovered in ReTrace carbon path analysis [21]. Self-contained web site: unpack zip archive and open index.html with a web browser. [file 1752-0509-3-104-S1.zip › AF1-treesei/pathways-C00031-to-C00037.html]

Pathways from C00031 to C00037


**Pathways from C00031 to C00037**

**Sources:** D-Glucose; (C00031)

**Target:**Glycine; (C00037)

|  | Composite mapping | Z | Average score | Rpairs | Reactions | Zero scores | Scores under threshold |
| --- | --- | --- | --- | --- | --- | --- | --- |
| Path 1 | C00031->C00037:[2->2,9->1] | 1.00 | 307.364705882 | 23 | 85 | 0 | 0 |
| Path 2 | C00031->C00037:[4->1,5->2,7->1] | 1.00 | 341.12371134 | 27 | 97 | 0 | 0 |
| Path 3 | C00031->C00037:[2->1,4->2] | 1.00 | 390.630434783 | 22 | 92 | 0 | 0 |
| Path 4 | C00031->C00037:[9->1,9->2] | 1.00 | 406.513513514 | 14 | 37 | 0 | 0 |
| Path 5 | C00031->C00037:[4->1,5->2] | 1.00 | 356.428571429 | 27 | 91 | 0 | 0 |
| Path 6 | C00031->C00037:[4->1,4->2] | 1.00 | 187.338461538 | 22 | 195 | 0 | 0 |
| Path 7 | C00031->C00037:[4->1,4->2] | 1.00 | 204.80620155 | 21 | 129 | 0 | 2 |
| Path 8 | C00031->C00037:[4->1,4->2] | 1.00 | 399.970588235 | 12 | 34 | 0 | 1 |
| Path 9 | C00031->C00037:[4->1,5->2] | 1.00 | 414.879310345 | 16 | 58 | 0 | 0 |
| Path 10 | C00031->C00037:[4->1,5->2] | 1.00 | 367.265306122 | 30 | 98 | 0 | 0 |
| Path 11 | C00031->C00037:[7->1,9->2] | 1.00 | 335.282051282 | 11 | 39 | 0 | 0 |
| Path 12 | C00031->C00037:[9->1,9->2] | 1.00 | 316.528301887 | 15 | 53 | 0 | 0 |
| Path 13 | C00031->C00037:[4->1,4->2] | 1.00 | 411.526315789 | 14 | 38 | 0 | 1 |
| Path 14 | C00031->C00037:[4->1,5->2,7->1] | 1.00 | 356.231578947 | 27 | 95 | 0 | 0 |
| Path 15 | C00031->C00037:[4->1,5->2,7->2] | 1.00 | 436.261538462 | 20 | 65 | 0 | 0 |
| Path 16 | C00031->C00037:[4->1,5->2] | 1.00 | 441.5 | 15 | 38 | 0 | 0 |
| Path 17 | C00031->C00037:[4->1,4->2] | 1.00 | 182.182291667 | 21 | 192 | 0 | 0 |
| Path 18 | C00031->C00037:[7->1,9->2] | 1.00 | 409.5 | 10 | 28 | 0 | 0 |
| Path 19 | C00031->C00037:[4->1,5->2] | 1.00 | 343.402298851 | 22 | 87 | 0 | 0 |
| Path 20 | C00031->C00037:[4->1,9->2] | 1.00 | 293.590909091 | 17 | 66 | 0 | 1 |
| Path 21 | C00031->C00037:[9->1,9->2] | 1.00 | 287.142857143 | 11 | 49 | 0 | 0 |
| Path 22 | C00031->C00037:[4->1,4->2] | 1.00 | 195.048 | 19 | 125 | 0 | 2 |
| Path 23 | C00031->C00037:[4->1,7->2] | 1.00 | 424.704918033 | 16 | 61 | 0 | 0 |
| Path 24 | C00031->C00037:[7->1,9->2] | 1.00 | 443.083333333 | 15 | 36 | 0 | 0 |
| Path 25 | C00031->C00037:[4->1,4->2] | 1.00 | 206.74015748 | 21 | 127 | 0 | 2 |
| Path 26 | C00031->C00037:[4->1,9->2] | 1.00 | 350.23255814 | 12 | 43 | 0 | 0 |
| Path 27 | C00031->C00037:[4->1,9->2] | 1.00 | 407.102564103 | 13 | 39 | 0 | 0 |
| Path 28 | C00031->C00037:[9->1,9->2] | 1.00 | 358.727272727 | 13 | 44 | 0 | 0 |
| Path 29 | C00031->C00037:[4->1,4->2] | 1.00 | 436.458333333 | 21 | 48 | 0 | 1 |
| Path 30 | C00031->C00037:[7->1,9->2] | 1.00 | 413.238095238 | 16 | 42 | 0 | 0 |
| Path 31 | C00031->C00037:[4->2,9->1] | 1.00 | 179.251282051 | 22 | 195 | 0 | 0 |
| Path 32 | C00031->C00037:[4->1,5->2] | 1.00 | 594.701030928 | 24 | 97 | 0 | 0 |
| Path 33 | C00031->C00037:[4->1,5->2] | 1.00 | 321.630252101 | 31 | 119 | 0 | 0 |
| Path 34 | C00031->C00037:[4->1,5->2] | 1.00 | 613.736842105 | 26 | 95 | 0 | 0 |
| Path 35 | C00031->C00037:[9->1,9->2] | 1.00 | 279.396825397 | 16 | 63 | 0 | 0 |
| Path 36 | C00031->C00037:[7->1,9->2] | 1.00 | 426.542857143 | 14 | 35 | 0 | 0 |
| Path 37 | C00031->C00037:[5->2,7->1] | 1.00 | 439.578947368 | 17 | 38 | 0 | 0 |
| Path 38 | C00031->C00037:[4->1,5->2] | 1.00 | 362.837209302 | 25 | 86 | 0 | 0 |
| Path 39 | C00031->C00037:[4->1,5->2] | 1.00 | 350.714285714 | 24 | 91 | 0 | 0 |
| Path 40 | C00031->C00037:[4->1,5->2] | 1.00 | 341.862068966 | 24 | 87 | 0 | 0 |
| Path 41 | C00031->C00037:[9->1,9->2] | 1.00 | 453.885714286 | 14 | 35 | 0 | 0 |
| Path 42 | C00031->C00037:[4->1,5->2] | 1.00 | 357.107142857 | 24 | 84 | 0 | 0 |
| Path 43 | C00031->C00037:[1->2,2->1] | 1.00 | 430.567567568 | 12 | 37 | 0 | 0 |
| Path 44 | C00031->C00037:[4->1,5->2] | 1.00 | 355.641304348 | 25 | 92 | 0 | 0 |
| Path 45 | C00031->C00037:[4->1,7->2] | 1.00 | 392.4 | 21 | 65 | 0 | 0 |
| Path 46 | C00031->C00037:[9->1,9->2] | 1.00 | 429.515151515 | 12 | 33 | 0 | 0 |
| Path 47 | C00031->C00037:[9->1,9->2] | 1.00 | 261.333333333 | 12 | 60 | 0 | 0 |
| Path 48 | C00031->C00037:[4->1,5->2,7->1] | 1.00 | 357.461538462 | 26 | 91 | 0 | 0 |
| Path 49 | C00031->C00037:[4->1,7->2] | 1.00 | 336.540229885 | 19 | 87 | 0 | 0 |
| Path 50 | C00031->C00037:[4->1,9->2] | 1.00 | 414.976190476 | 14 | 42 | 0 | 0 |
| Path 51 | C00031->C00037:[4->1,4->2] | 1.00 | 429.795454545 | 19 | 44 | 0 | 1 |
| Path 52 | C00031->C00037:[4->1,9->2] | 1.00 | 314.583333333 | 22 | 72 | 0 | 1 |
| Path 53 | C00031->C00037:[4->1,5->2,7->1] | 1.00 | 338.965517241 | 22 | 87 | 0 | 0 |
| Path 54 | C00031->C00037:[4->1,5->2] | 1.00 | 334.662790698 | 21 | 86 | 0 | 0 |
| Path 55 | C00031->C00037:[5->2,9->1] | 1.00 | 349.769230769 | 19 | 78 | 0 | 0 |
| Path 56 | C00031->C00037:[9->1,9->2] | 1.00 | 449.702702703 | 16 | 37 | 0 | 0 |
| Path 57 | C00031->C00037:[4->1,5->2] | 1.00 | 419.918032787 | 17 | 61 | 0 | 0 |
| Path 58 | C00031->C00037:[2->2,7->1] | 1.00 | 366.275362319 | 24 | 69 | 0 | 0 |
| Path 59 | C00031->C00037:[4->1,7->2] | 1.00 | 298.348623853 | 24 | 109 | 0 | 0 |
| Path 60 | C00031->C00037:[4->1,5->2] | 1.00 | 310.8125 | 27 | 112 | 0 | 0 |
| Path 61 | C00031->C00037:[4->1,4->2,7->1,7->2] | 1.00 | 392.571428571 | 18 | 49 | 0 | 1 |
| Path 62 | C00031->C00037:[4->1,7->2] | 1.00 | 330.2 | 20 | 85 | 0 | 0 |
| Path 63 | C00031->C00037:[4->1,5->2] | 1.00 | 365.550561798 | 26 | 89 | 0 | 0 |
| Path 64 | C00031->C00037:[9->1,9->2] | 1.00 | 239.242857143 | 13 | 70 | 0 | 0 |
| Path 65 | C00031->C00037:[2->2,4->1] | 1.00 | 360.515151515 | 21 | 66 | 0 | 0 |
| Path 66 | C00031->C00037:[4->1,7->2] | 1.00 | 351.357894737 | 27 | 95 | 0 | 0 |
| Path 67 | C00031->C00037:[4->1,5->2] | 1.00 | 349.241758242 | 26 | 91 | 0 | 0 |
| Path 68 | C00031->C00037:[9->1,9->2] | 1.00 | 256.559322034 | 12 | 59 | 0 | 0 |
| Path 69 | C00031->C00037:[2->2,9->1] | 1.00 | 344.47826087 | 22 | 69 | 0 | 0 |
| Path 70 | C00031->C00037:[9->1,9->2] | 1.00 | 369.804878049 | 13 | 41 | 0 | 0 |
| Path 71 | C00031->C00037:[4->1,5->2,7->1] | 1.00 | 354.723404255 | 27 | 94 | 0 | 0 |
| Path 72 | C00031->C00037:[9->1,9->2] | 1.00 | 280.516129032 | 14 | 62 | 0 | 0 |
| Path 73 | C00031->C00037:[4->1,7->1,7->2] | 1.00 | 339.954545455 | 23 | 88 | 0 | 0 |
| Path 74 | C00031->C00037:[4->1,5->2,7->1] | 1.00 | 363.927083333 | 28 | 96 | 0 | 0 |
| Path 75 | C00031->C00037:[9->1,9->2] | 1.00 | 322.409090909 | 11 | 44 | 0 | 0 |
| Path 76 | C00031->C00037:[4->1,5->2] | 1.00 | 317.752212389 | 28 | 113 | 0 | 0 |
| Path 77 | C00031->C00037:[4->1,5->2] | 1.00 | 364.045454545 | 26 | 88 | 0 | 0 |
| Path 78 | C00031->C00037:[4->1,9->2] | 1.00 | 303.180327869 | 16 | 61 | 0 | 1 |
| Path 79 | C00031->C00037:[2->2,4->1] | 1.00 | 367.333333333 | 22 | 69 | 0 | 0 |
| Path 80 | C00031->C00037:[7->2,9->1] | 1.00 | 169.8 | 23 | 220 | 0 | 0 |
| Path 81 | C00031->C00037:[7->1,9->2] | 1.00 | 359.543478261 | 15 | 46 | 0 | 0 |
| Path 82 | C00031->C00037:[2->2,7->1] | 1.00 | 351.774193548 | 20 | 62 | 0 | 0 |
| Path 83 | C00031->C00037:[4->1,5->2] | 1.00 | 342.444444444 | 23 | 90 | 0 | 0 |
| Path 84 | C00031->C00037:[7->1,9->2] | 1.00 | 430.620689655 | 11 | 29 | 0 | 0 |
| Path 85 | C00031->C00037:[7->1,9->2] | 1.00 | 396.942857143 | 12 | 35 | 0 | 0 |
| Path 86 | C00031->C00037:[4->1,5->2] | 1.00 | 593.319587629 | 26 | 97 | 0 | 0 |
| Path 87 | C00031->C00037:[4->1,4->2] | 1.00 | 196.886178862 | 19 | 123 | 0 | 2 |
| Path 88 | C00031->C00037:[2->2,9->1] | 1.00 | 370.705882353 | 23 | 68 | 0 | 0 |
| Path 89 | C00031->C00037:[4->2,9->1] | 1.00 | 187.592783505 | 23 | 194 | 0 | 0 |
| Path 90 | C00031->C00037:[5->2,7->1] | 1.00 | 427.129032258 | 13 | 31 | 0 | 0 |
| Path 91 | C00031->C00037:[4->1,7->2] | 1.00 | 588.178947368 | 22 | 95 | 0 | 0 |
| Path 92 | C00031->C00037:[5->2,9->1] | 1.00 | 341.038961039 | 18 | 77 | 0 | 0 |
| Path 93 | C00031->C00037:[1->2,2->1] | 1.00 | 510.195121951 | 11 | 41 | 0 | 0 |
| Path 94 | C00031->C00037:[4->1,7->1,9->2] | 1.00 | 295.184615385 | 18 | 65 | 0 | 1 |
| Path 95 | C00031->C00037:[4->1,5->2] | 1.00 | 317.017699115 | 28 | 113 | 0 | 0 |
| Path 96 | C00031->C00037:[4->1,9->2] | 1.00 | 420.3125 | 11 | 32 | 0 | 0 |
| Path 97 | C00031->C00037:[4->1,5->2] | 1.00 | 435.0 | 14 | 35 | 0 | 0 |
| Path 98 | C00031->C00037:[4->1,5->2] | 1.00 | 614.968085106 | 26 | 94 | 0 | 0 |
| Path 99 | C00031->C00037:[4->1,4->2,7->1,7->2] | 1.00 | 406.58490566 | 21 | 53 | 0 | 1 |
| Path 100 | C00031->C00037:[4->1,5->2] | 1.00 | 361.629213483 | 27 | 89 | 0 | 0 |
| Path 101 | C00031->C00037:[4->1,4->2] | 1.00 | 447.121212121 | 13 | 33 | 0 | 1 |
| Path 102 | C00031->C00037:[4->1,9->2] | 1.00 | 361.130434783 | 13 | 46 | 0 | 0 |
| Path 103 | C00031->C00037:[9->1,9->2] | 1.00 | 325.963636364 | 14 | 55 | 0 | 0 |
| Path 104 | C00031->C00037:[4->1,5->2] | 1.00 | 589.489583333 | 23 | 96 | 0 | 0 |
| Path 105 | C00031->C00037:[4->1,9->2] | 1.00 | 438.545454545 | 12 | 33 | 0 | 0 |
| Path 106 | C00031->C00037:[4->1,4->2] | 1.00 | 453.891891892 | 15 | 37 | 0 | 1 |
| Path 107 | C00031->C00037:[9->1,9->2] | 1.00 | 381.090909091 | 10 | 33 | 0 | 0 |
| Path 108 | C00031->C00037:[4->1,9->2] | 1.00 | 428.628571429 | 12 | 35 | 0 | 0 |
| Path 109 | C00031->C00037:[9->1,9->2] | 1.00 | 301.84 | 12 | 50 | 0 | 0 |
| Path 110 | C00031->C00037:[2->2,9->1] | 1.00 | 298.857142857 | 22 | 84 | 0 | 0 |
| Path 111 | C00031->C00037:[4->1,7->2] | 1.00 | 338.269662921 | 22 | 89 | 0 | 0 |
| Path 112 | C00031->C00037:[5->2,7->1] | 1.00 | 402.673076923 | 13 | 52 | 0 | 0 |
| Path 113 | C00031->C00037:[4->1,9->2] | 1.00 | 445.111111111 | 13 | 36 | 0 | 0 |
| Path 114 | C00031->C00037:[4->1,5->2,7->1] | 1.00 | 347.556818182 | 23 | 88 | 0 | 0 |
| Path 115 | C00031->C00037:[5->2,7->2,9->1] | 1.00 | 402.016129032 | 18 | 62 | 0 | 0 |
| Path 116 | C00031->C00037:[4->1,4->2,7->1,7->2] | 1.00 | 417.113636364 | 17 | 44 | 0 | 1 |
| Path 117 | C00031->C00037:[4->1,7->2] | 1.00 | 412.942028986 | 19 | 69 | 0 | 0 |
| Path 118 | C00031->C00037:[5->2,7->1] | 1.00 | 421.516666667 | 18 | 60 | 0 | 0 |
| Path 119 | C00031->C00037:[2->1,4->2] | 1.00 | 212.233333333 | 19 | 210 | 0 | 0 |
| Path 120 | C00031->C00037:[9->1,9->2] | 1.00 | 309.450980392 | 13 | 51 | 0 | 0 |
| Path 121 | C00031->C00037:[4->1,5->2] | 1.00 | 320.13559322 | 31 | 118 | 0 | 0 |
| Path 122 | C00031->C00037:[4->2,9->1] | 1.00 | 172.804761905 | 22 | 210 | 0 | 0 |
| Path 123 | C00031->C00037:[9->1,9->2] | 1.00 | 274.333333333 | 13 | 63 | 0 | 0 |
| Path 124 | C00031->C00037:[4->2,7->1] | 1.00 | 175.505319149 | 20 | 188 | 0 | 0 |
| Path 125 | C00031->C00037:[9->1,9->2] | 1.00 | 408.228571429 | 12 | 35 | 0 | 0 |
| Path 126 | C00031->C00037:[4->1,5->2] | 1.00 | 310.090909091 | 31 | 121 | 0 | 0 |
| Path 127 | C00031->C00037:[4->2,7->1] | 1.00 | 186.964102564 | 24 | 195 | 0 | 0 |
| Path 128 | C00031->C00037:[4->1,4->2,9->2] | 1.00 | 297.815384615 | 17 | 65 | 0 | 1 |
| Path 129 | C00031->C00037:[9->1,9->2] | 1.00 | 311.981132075 | 15 | 53 | 0 | 0 |
| Path 130 | C00031->C00037:[4->2,9->1] | 1.00 | 176.829383886 | 23 | 211 | 0 | 0 |
| Path 131 | C00031->C00037:[4->1,4->2,7->1,7->2] | 1.00 | 382.155555556 | 16 | 45 | 0 | 1 |
| Path 132 | C00031->C00037:[5->2,9->1] | 1.00 | 432.196721311 | 19 | 61 | 0 | 0 |
| Path 133 | C00031->C00037:[4->1,5->2] | 1.00 | 348.636363636 | 23 | 88 | 0 | 0 |
| Path 134 | C00031->C00037:[4->1,5->2] | 1.00 | 596.836734694 | 25 | 98 | 0 | 0 |
| Path 135 | C00031->C00037:[4->1,5->2] | 1.00 | 358.764705882 | 24 | 85 | 0 | 0 |
| Path 136 | C00031->C00037:[9->1,9->2] | 1.00 | 305.962264151 | 12 | 53 | 0 | 0 |
| Path 137 | C00031->C00037:[4->1,7->2] | 1.00 | 438.396825397 | 18 | 63 | 0 | 0 |
| Path 138 | C00031->C00037:[9->1,9->2] | 1.00 | 441.081081081 | 13 | 37 | 0 | 0 |
| Path 139 | C00031->C00037:[4->1,9->2] | 1.00 | 279.64516129 | 15 | 62 | 0 | 1 |
| Path 140 | C00031->C00037:[9->1,9->2] | 1.00 | 276.213114754 | 14 | 61 | 0 | 0 |
| Path 141 | C00031->C00037:[4->1,4->2] | 1.00 | 412.294117647 | 21 | 51 | 0 | 1 |
| Path 142 | C00031->C00037:[9->2] | 0.50 | 376.375 | 9 | 32 | 0 | 0 |
| Path 143 | C00031->C00037:[4->1,5->2] | 1.00 | 453.065217391 | 21 | 46 | 0 | 0 |
| Path 144 | C00031->C00037:[4->1,7->2] | 1.00 | 338.045454545 | 21 | 88 | 0 | 0 |
| Path 145 | C00031->C00037:[7->2] | 0.50 | 373.846153846 | 13 | 39 | 0 | 0 |
| Path 146 | C00031->C00037:[4->1] | 0.50 | 487.85 | 11 | 20 | 0 | 2 |
| Path 147 | C00031->C00037:[4->1,9->2] | 1.00 | 358.410958904 | 27 | 73 | 0 | 1 |
| Path 148 | C00031->C00037:[4->1,5->2,7->1] | 1.00 | 337.921348315 | 24 | 89 | 0 | 0 |
| Path 149 | C00031->C00037:[5->2,9->1] | 1.00 | 334.611940299 | 21 | 67 | 0 | 0 |
| Path 150 | C00031->C00037:[4->1,4->2,7->1,7->2] | 1.00 | 174.16751269 | 25 | 197 | 0 | 2 |
| Path 151 | C00031->C00037:[4->1,5->2] | 1.00 | 319.811965812 | 30 | 117 | 0 | 0 |
| Path 152 | C00031->C00037:[4->1,9->2] | 1.00 | 448.793650794 | 29 | 63 | 0 | 1 |
| Path 153 | C00031->C00037:[4->1,9->2] | 1.00 | 370.179487179 | 29 | 78 | 0 | 1 |
| Path 154 | C00031->C00037:[4->1,5->2] | 1.00 | 294.154545455 | 25 | 110 | 0 | 0 |
| Path 155 | C00031->C00037:[4->1,9->2] | 1.00 | 359.426666667 | 26 | 75 | 0 | 1 |
| Path 156 | C00031->C00037:[4->1,4->2] | 1.00 | 172.378947368 | 23 | 190 | 0 | 2 |
| Path 157 | C00031->C00037:[4->1,5->2] | 1.00 | 328.209302326 | 21 | 86 | 0 | 0 |
| Path 158 | C00031->C00037:[4->1,5->2] | 1.00 | 349.144444444 | 25 | 90 | 0 | 0 |
| Path 159 | C00031->C00037:[4->1,5->2,7->1] | 1.00 | 327.104651163 | 21 | 86 | 0 | 0 |
| Path 160 | C00031->C00037:[5->2,7->1] | 1.00 | 304.568181818 | 21 | 88 | 0 | 0 |
| Path 161 | C00031->C00037:[4->1] | 0.50 | 482.647058824 | 10 | 17 | 0 | 2 |
| Path 162 | C00031->C00037:[4->1,9->2] | 1.00 | 440.75862069 | 27 | 58 | 0 | 1 |
| Path 163 | C00031->C00037:[4->1,4->2] | 1.00 | 488.866666667 | 22 | 45 | 0 | 1 |
| Path 164 | C00031->C00037:[4->1,5->2] | 1.00 | 451.784313725 | 24 | 51 | 0 | 1 |
| Path 165 | C00031->C00037:[4->1,5->2] | 1.00 | 599.298969072 | 24 | 97 | 0 | 0 |
| Path 166 | C00031->C00037:[5->2,7->1] | 1.00 | 318.317647059 | 21 | 85 | 0 | 0 |
| Path 167 | C00031->C00037:[4->1,5->2] | 1.00 | 456.145833333 | 22 | 48 | 0 | 0 |
| Path 168 | C00031->C00037:[4->1,9->2] | 1.00 | 436.425925926 | 24 | 54 | 0 | 1 |
| Path 169 | C00031->C00037:[4->1,9->2] | 1.00 | 359.87012987 | 28 | 77 | 0 | 1 |
| Path 170 | C00031->C00037:[4->1,7->1,9->2] | 1.00 | 445.894736842 | 27 | 57 | 0 | 1 |
| Path 171 | C00031->C00037:[4->1] | 0.50 | 475.414634146 | 19 | 41 | 0 | 1 |
| Path 172 | C00031->C00037:[7->1,9->2] | 1.00 | 351.732394366 | 25 | 71 | 0 | 1 |
| Path 173 | C00031->C00037:[4->1,4->2,7->1,7->2] | 1.00 | 162.134831461 | 22 | 178 | 0 | 2 |
| Path 174 | C00031->C00037:[7->1,9->2] | 1.00 | 361.24137931 | 18 | 29 | 0 | 1 |
| Path 175 | C00031->C00037:[4->1,4->2] | 1.00 | 409.486486486 | 13 | 37 | 0 | 1 |
| Path 176 | C00031->C00037:[4->1,5->2] | 1.00 | 827.728813559 | 22 | 59 | 0 | 0 |
| Path 177 | C00031->C00037:[4->1,7->1,9->2] | 1.00 | 409.40625 | 27 | 64 | 0 | 1 |
| Path 178 | C00031->C00037:[4->1,5->2] | 1.00 | 342.261904762 | 23 | 84 | 0 | 0 |
| Path 179 | C00031->C00037:[4->1,5->2] | 1.00 | 300.621621622 | 26 | 111 | 0 | 0 |
| Path 180 | C00031->C00037:[4->1,5->2] | 1.00 | 591.926315789 | 22 | 95 | 0 | 0 |
| Path 181 | C00031->C00037:[7->1] | 0.50 | 379.523809524 | 10 | 21 | 0 | 2 |
| Path 182 | C00031->C00037:[5->2] | 0.50 | 399.731707317 | 15 | 41 | 0 | 0 |
| Path 183 | C00031->C00037:[4->1,5->2] | 1.00 | 355.615384615 | 24 | 91 | 0 | 0 |
| Path 184 | C00031->C00037:[4->1,9->2] | 1.00 | 458.461538462 | 23 | 52 | 0 | 1 |
| Path 185 | C00031->C00037:[4->1] | 0.50 | 472.105263158 | 18 | 38 | 0 | 1 |
| Path 186 | C00031->C00037:[4->1,4->2] | 1.00 | 159.864130435 | 19 | 184 | 0 | 2 |
| Path 187 | C00031->C00037:[4->1,5->2] | 1.00 | 341.674418605 | 23 | 86 | 0 | 0 |
| Path 188 | C00031->C00037:[7->2,9->1] | 1.00 | 302.111111111 | 17 | 63 | 0 | 0 |
| Path 189 | C00031->C00037:[4->1,5->2] | 1.00 | 595.770833333 | 25 | 96 | 0 | 0 |
| Path 190 | C00031->C00037:[4->1,4->2] | 1.00 | 419.381818182 | 23 | 55 | 0 | 1 |
| Path 191 | C00031->C00037:[4->1] | 0.50 | 251.619047619 | 8 | 21 | 0 | 1 |
| Path 192 | C00031->C00037:[4->1,4->2] | 1.00 | 441.205882353 | 13 | 34 | 0 | 1 |
| Path 193 | C00031->C00037:[4->1,9->2] | 1.00 | 392.25 | 27 | 68 | 0 | 1 |
| Path 194 | C00031->C00037:[4->1,5->2,7->1] | 1.00 | 322.473684211 | 25 | 95 | 0 | 0 |
| Path 195 | C00031->C00037:[7->1,7->2] | 1.00 | 406.642857143 | 16 | 42 | 0 | 1 |
| Path 196 | C00031->C00037:[4->1,5->2] | 1.00 | 343.23255814 | 21 | 86 | 0 | 0 |
| Path 197 | C00031->C00037:[4->1,5->2] | 1.00 | 406.957446809 | 18 | 47 | 0 | 0 |
| Path 198 | C00031->C00037:[4->1,9->2] | 1.00 | 474.407407407 | 25 | 54 | 0 | 1 |
| Path 199 | C00031->C00037:[4->1,7->2] | 1.00 | 590.627659574 | 21 | 94 | 0 | 0 |
| Path 200 | C00031->C00037:[4->1,4->2] | 1.00 | 163.774193548 | 20 | 186 | 0 | 2 |
| Path 201 | C00031->C00037:[5->2,7->1] | 1.00 | 320.406976744 | 21 | 86 | 0 | 0 |
| Path 202 | C00031->C00037:[4->1,4->2] | 1.00 | 151.125748503 | 17 | 167 | 0 | 2 |
| Path 203 | C00031->C00037:[4->1,5->2] | 1.00 | 329.25 | 21 | 88 | 0 | 0 |
| Path 204 | C00031->C00037:[4->1,5->2] | 1.00 | 350.633333333 | 23 | 90 | 0 | 0 |
| Path 205 | C00031->C00037:[4->1,7->2] | 1.00 | 338.604651163 | 18 | 86 | 0 | 0 |
| Path 206 | C00031->C00037:[4->1,9->2] | 1.00 | 290.615384615 | 16 | 65 | 0 | 1 |
| Path 207 | C00031->C00037:[4->1,4->2] | 1.00 | 184.888888889 | 29 | 225 | 0 | 2 |
| Path 208 | C00031->C00037:[4->1,9->2] | 1.00 | 456.588235294 | 23 | 51 | 0 | 1 |
| Path 209 | C00031->C00037:[7->1,7->2] | 1.00 | 391.166666667 | 16 | 42 | 0 | 0 |
| Path 210 | C00031->C00037:[4->1,5->2] | 1.00 | 294.680672269 | 29 | 119 | 0 | 0 |
| Path 211 | C00031->C00037:[4->1,9->2] | 1.00 | 384.90625 | 25 | 64 | 0 | 1 |
| Path 212 | C00031->C00037:[5->2,7->1] | 1.00 | 421.066666667 | 20 | 45 | 0 | 0 |
| Path 213 | C00031->C00037:[4->1,9->2] | 1.00 | 438.849056604 | 24 | 53 | 0 | 1 |
| Path 214 | C00031->C00037:[7->1,9->2] | 1.00 | 334.076923077 | 22 | 65 | 0 | 1 |
| Path 215 | C00031->C00037:[4->1,4->2,7->1,7->2] | 1.00 | 170.866666667 | 24 | 195 | 0 | 2 |
| Path 216 | C00031->C00037:[4->1,9->2] | 1.00 | 365.775 | 29 | 80 | 0 | 1 |
| Path 217 | C00031->C00037:[4->1,5->2] | 1.00 | 341.735632184 | 25 | 87 | 0 | 0 |
| Path 218 | C00031->C00037:[4->1,5->2] | 1.00 | 348.528735632 | 22 | 87 | 0 | 0 |
| Path 219 | C00031->C00037:[4->1,4->2] | 1.00 | 167.308510638 | 21 | 188 | 0 | 2 |
| Path 220 | C00031->C00037:[4->1,5->2] | 1.00 | 364.114942529 | 25 | 87 | 0 | 0 |
| Path 221 | C00031->C00037:[4->1,5->2] | 1.00 | 321.322033898 | 30 | 118 | 0 | 0 |
| Path 222 | C00031->C00037:[7->1,7->2] | 1.00 | 299.101265823 | 17 | 79 | 0 | 0 |
| Path 223 | C00031->C00037:[4->1,7->2] | 1.00 | 825.410714286 | 19 | 56 | 0 | 0 |
| Path 224 | C00031->C00037:[4->1,5->2] | 1.00 | 304.5 | 29 | 116 | 0 | 0 |
| Path 225 | C00031->C00037:[5->2] | 0.50 | 396.463414634 | 17 | 41 | 0 | 0 |
| Path 226 | C00031->C00037:[4->1,4->2,7->1,7->2] | 1.00 | 456.545454545 | 25 | 55 | 0 | 1 |
| Path 227 | C00031->C00037:[4->1,7->2] | 1.00 | 329.869047619 | 19 | 84 | 0 | 0 |
| Path 228 | C00031->C00037:[5->2] | 0.50 | 436.102564103 | 17 | 39 | 0 | 0 |
| Path 229 | C00031->C00037:[5->2,7->1] | 1.00 | 297.474358974 | 16 | 78 | 0 | 0 |
| Path 230 | C00031->C00037:[4->1,5->2] | 1.00 | 367.360824742 | 29 | 97 | 0 | 0 |
| Path 231 | C00031->C00037:[4->1,9->2] | 1.00 | 432.982758621 | 25 | 58 | 0 | 1 |
| Path 232 | C00031->C00037:[4->1,7->2] | 1.00 | 315.872093023 | 19 | 86 | 0 | 0 |
| Path 233 | C00031->C00037:[7->1,9->2] | 1.00 | 431.66 | 23 | 50 | 0 | 1 |
| Path 234 | C00031->C00037:[4->1,5->2] | 1.00 | 345.747126437 | 24 | 87 | 0 | 0 |
| Path 235 | C00031->C00037:[4->1,5->2] | 1.00 | 309.691666667 | 30 | 120 | 0 | 0 |
| Path 236 | C00031->C00037:[5->2] | 0.50 | 382.35 | 14 | 40 | 0 | 0 |
| Path 237 | C00031->C00037:[4->1,7->2] | 1.00 | 359.354166667 | 15 | 48 | 0 | 0 |
| Path 238 | C00031->C00037:[4->1,9->2] | 1.00 | 493.34 | 25 | 50 | 0 | 1 |
| Path 239 | C00031->C00037:[7->1,7->2] | 1.00 | 405.959183673 | 20 | 49 | 0 | 0 |
| Path 240 | C00031->C00037:[2->2] | 0.50 | 338.322033898 | 17 | 59 | 0 | 0 |
| Path 241 | C00031->C00037:[1->2,4->1] | 1.00 | 323.86 | 17 | 50 | 0 | 1 |
| Path 242 | C00031->C00037:[4->1] | 0.50 | 147.245508982 | 16 | 167 | 0 | 2 |
| Path 243 | C00031->C00037:[4->1,9->2] | 1.00 | 422.490196078 | 22 | 51 | 0 | 1 |
| Path 244 | C00031->C00037:[4->1,5->2,7->1] | 1.00 | 318.070588235 | 20 | 85 | 0 | 0 |
| Path 245 | C00031->C00037:[4->1,5->2,7->1] | 1.00 | 337.505376344 | 25 | 93 | 0 | 0 |
| Path 246 | C00031->C00037:[4->1,7->1,9->2] | 1.00 | 449.466666667 | 28 | 60 | 0 | 1 |
| Path 247 | C00031->C00037:[7->1,7->2] | 1.00 | 438.333333333 | 20 | 45 | 0 | 1 |
| Path 248 | C00031->C00037:[4->1,5->2] | 1.00 | 316.651785714 | 27 | 112 | 0 | 0 |
| Path 249 | C00031->C00037:[4->1,4->2] | 1.00 | 177.434554974 | 22 | 191 | 0 | 2 |
| Path 250 | C00031->C00037:[4->1,7->1,9->2] | 1.00 | 394.774647887 | 29 | 71 | 0 | 1 |
| Path 251 | C00031->C00037:[7->1,9->2] | 1.00 | 453.234042553 | 22 | 47 | 0 | 1 |
| Path 252 | C00031->C00037:[4->1,5->2,7->1] | 1.00 | 354.688172043 | 26 | 93 | 0 | 0 |
| Path 253 | C00031->C00037:[4->1,4->2,7->1,7->2] | 1.00 | 191.878640777 | 28 | 206 | 0 | 2 |
| Path 254 | C00031->C00037:[7->1,9->2] | 1.00 | 448.62745098 | 24 | 51 | 0 | 1 |
| Path 255 | C00031->C00037:[7->1] | 0.50 | 421.547619048 | 18 | 42 | 0 | 1 |
| Path 256 | C00031->C00037:[4->1,5->2] | 1.00 | 418.5 | 21 | 48 | 0 | 0 |
| Path 257 | C00031->C00037:[7->1,9->2] | 1.00 | 460.717391304 | 22 | 46 | 0 | 1 |
| Path 258 | C00031->C00037:[4->1,5->2] | 1.00 | 357.096385542 | 23 | 83 | 0 | 0 |
| Path 259 | C00031->C00037:[4->1,9->2] | 1.00 | 379.405405405 | 28 | 74 | 0 | 1 |
| Path 260 | C00031->C00037:[4->1,5->2,7->1] | 1.00 | 347.436781609 | 22 | 87 | 0 | 0 |
| Path 261 | C00031->C00037:[4->1,5->2] | 1.00 | 434.192307692 | 21 | 52 | 0 | 0 |
| Path 262 | C00031->C00037:[1->2,4->1] | 1.00 | 334.811320755 | 18 | 53 | 0 | 1 |
| Path 263 | C00031->C00037:[4->1,5->2] | 1.00 | 329.516853933 | 24 | 89 | 0 | 0 |
| Path 264 | C00031->C00037:[4->1,5->2] | 1.00 | 421.291666667 | 19 | 48 | 0 | 0 |
| Path 265 | C00031->C00037:[7->1,7->2] | 1.00 | 351.684210526 | 12 | 38 | 0 | 1 |
| Path 266 | C00031->C00037:[4->1,5->2] | 1.00 | 429.102040816 | 20 | 49 | 0 | 0 |
| Path 267 | C00031->C00037:[4->1,5->2] | 1.00 | 334.388235294 | 20 | 85 | 0 | 0 |
| Path 268 | C00031->C00037:[4->1] | 0.50 | 493.2 | 9 | 15 | 0 | 1 |
| Path 269 | C00031->C00037:[4->1,9->2] | 1.00 | 406.030769231 | 26 | 65 | 0 | 1 |
| Path 270 | C00031->C00037:[4->1,4->2] | 1.00 | 174.063492063 | 21 | 189 | 0 | 2 |
| Path 271 | C00031->C00037:[4->1,9->2] | 1.00 | 447.411764706 | 22 | 51 | 0 | 1 |
| Path 272 | C00031->C00037:[4->1,4->2] | 1.00 | 176.114155251 | 26 | 219 | 0 | 2 |
| Path 273 | C00031->C00037:[4->1,4->2] | 1.00 | 160.894736842 | 18 | 171 | 0 | 2 |
| Path 274 | C00031->C00037:[4->1] | 0.50 | 140.475609756 | 15 | 164 | 0 | 2 |
| Path 275 | C00031->C00037:[4->1,7->1,9->2] | 1.00 | 445.53125 | 29 | 64 | 0 | 1 |
| Path 276 | C00031->C00037:[4->1,7->2] | 1.00 | 332.52688172 | 25 | 93 | 0 | 0 |
| Path 277 | C00031->C00037:[4->1,5->2] | 1.00 | 336.865168539 | 25 | 89 | 0 | 0 |
| Path 278 | C00031->C00037:[4->1,5->2] | 1.00 | 306.153846154 | 29 | 117 | 0 | 0 |
| Path 279 | C00031->C00037:[4->1,4->2] | 1.00 | 461.069767442 | 19 | 43 | 0 | 1 |
| Path 280 | C00031->C00037:[7->1,9->2] | 1.00 | 441.75 | 27 | 56 | 0 | 1 |
| Path 281 | C00031->C00037:[4->1,7->2] | 1.00 | 407.448979592 | 18 | 49 | 0 | 0 |
| Path 282 | C00031->C00037:[4->1,4->2] | 1.00 | 391.380952381 | 19 | 63 | 0 | 0 |
| Path 283 | C00031->C00037:[4->1,5->2] | 1.00 | 342.294117647 | 23 | 85 | 0 | 0 |
| Path 284 | C00031->C00037:[4->1,7->1,9->2] | 1.00 | 468.192982456 | 27 | 57 | 0 | 1 |
| Path 285 | C00031->C00037:[5->2,9->1] | 1.00 | 339.547945205 | 24 | 73 | 0 | 0 |
| Path 286 | C00031->C00037:[4->1,4->2] | 1.00 | 488.909090909 | 21 | 44 | 0 | 1 |
| Path 287 | C00031->C00037:[4->1,4->2] | 1.00 | 157.588235294 | 18 | 170 | 0 | 2 |
| Path 288 | C00031->C00037:[4->1,9->2] | 1.00 | 352.592105263 | 25 | 76 | 0 | 1 |
| Path 289 | C00031->C00037:[4->1,4->2,7->1,7->2] | 1.00 | 411.636363636 | 16 | 44 | 0 | 1 |
| Path 290 | C00031->C00037:[4->1,4->2,7->1,7->2] | 1.00 | 453.038461538 | 24 | 52 | 0 | 1 |
| Path 291 | C00031->C00037:[4->1,9->2] | 1.00 | 299.229508197 | 15 | 61 | 0 | 1 |
| Path 292 | C00031->C00037:[4->1,9->2] | 1.00 | 443.041666667 | 21 | 48 | 0 | 1 |
| Path 293 | C00031->C00037:[4->1,5->2] | 1.00 | 828.137931034 | 21 | 58 | 0 | 0 |
| Path 294 | C00031->C00037:[4->1,9->2] | 1.00 | 345.821917808 | 24 | 73 | 0 | 1 |
| Path 295 | C00031->C00037:[4->1,5->2] | 1.00 | 344.104651163 | 23 | 86 | 0 | 0 |
| Path 296 | C00031->C00037:[4->1,4->2,7->1,7->2] | 1.00 | 168.022099448 | 23 | 181 | 0 | 2 |
| Path 297 | C00031->C00037:[4->1,5->2] | 1.00 | 424.31372549 | 22 | 51 | 0 | 0 |
| Path 298 | C00031->C00037:[4->1,7->2] | 1.00 | 434.338709677 | 17 | 62 | 0 | 0 |
| Path 299 | C00031->C00037:[7->1,9->2] | 1.00 | 363.378378378 | 28 | 74 | 0 | 1 |
| Path 300 | C00031->C00037:[4->1,4->2] | 1.00 | 181.020725389 | 23 | 193 | 0 | 2 |
| Path 301 | C00031->C00037:[5->2] | 0.50 | 406.857142857 | 10 | 28 | 0 | 0 |
| Path 302 | C00031->C00037:[4->1,7->2] | 1.00 | 308.590361446 | 18 | 83 | 0 | 0 |
| Path 303 | C00031->C00037:[4->1,7->1,9->2] | 1.00 | 395.268656716 | 28 | 67 | 0 | 1 |
| Path 304 | C00031->C00037:[4->1,4->2,7->1,7->2] | 1.00 | 397.177419355 | 25 | 62 | 0 | 1 |
| Path 305 | C00031->C00037:[4->1,9->2] | 1.00 | 471.882352941 | 24 | 51 | 0 | 1 |
| Path 306 | C00031->C00037:[4->1,5->2] | 1.00 | 322.611764706 | 20 | 85 | 0 | 0 |
| Path 307 | C00031->C00037:[4->1,5->2] | 1.00 | 310.387387387 | 26 | 111 | 0 | 0 |
| Path 308 | C00031->C00037:[4->1,4->2,7->1,7->2] | 1.00 | 177.67839196 | 26 | 199 | 0 | 2 |
| Path 309 | C00031->C00037:[4->1,9->2] | 1.00 | 384.864197531 | 30 | 81 | 0 | 1 |
| Path 310 | C00031->C00037:[4->1,5->2] | 1.00 | 362.894117647 | 24 | 85 | 0 | 0 |
| Path 311 | C00031->C00037:[7->1,9->2] | 1.00 | 365.260869565 | 26 | 69 | 0 | 1 |
| Path 312 | C00031->C00037:[4->1,9->2] | 1.00 | 443.053571429 | 25 | 56 | 0 | 1 |
| Path 313 | C00031->C00037:[4->1,4->2,7->1,7->2] | 1.00 | 188.897058824 | 27 | 204 | 0 | 2 |
| Path 314 | C00031->C00037:[4->1,9->2] | 1.00 | 370.975609756 | 30 | 82 | 0 | 1 |
| Path 315 | C00031->C00037:[4->1,5->2] | 1.00 | 313.416666667 | 19 | 84 | 0 | 0 |
| Path 316 | C00031->C00037:[4->1,4->2] | 1.00 | 159.65497076 | 20 | 171 | 0 | 2 |
| Path 317 | C00031->C00037:[4->1,9->2] | 1.00 | 454.857142857 | 22 | 49 | 0 | 1 |
| Path 318 | C00031->C00037:[4->1,7->2] | 1.00 | 317.83908046 | 20 | 87 | 0 | 0 |
| Path 319 | C00031->C00037:[7->1,9->2] | 1.00 | 350.434782609 | 26 | 69 | 0 | 1 |
| Path 320 | C00031->C00037:[5->2,7->1] | 1.00 | 448.021276596 | 23 | 47 | 0 | 1 |
| Path 321 | C00031->C00037:[4->1,4->2] | 1.00 | 179.009049774 | 27 | 221 | 0 | 2 |
| Path 322 | C00031->C00037:[7->1,9->2] | 1.00 | 352.410958904 | 27 | 73 | 0 | 1 |
| Path 323 | C00031->C00037:[4->1,4->2] | 1.00 | 165.123655914 | 21 | 186 | 0 | 2 |
| Path 324 | C00031->C00037:[4->1,5->2] | 1.00 | 334.584269663 | 22 | 89 | 0 | 0 |
| Path 325 | C00031->C00037:[4->1,9->2] | 1.00 | 384.779220779 | 29 | 77 | 0 | 1 |
| Path 326 | C00031->C00037:[4->1,5->2] | 1.00 | 321.035294118 | 22 | 85 | 0 | 0 |
| Path 327 | C00031->C00037:[4->1,5->2,7->1] | 1.00 | 363.989473684 | 27 | 95 | 0 | 0 |
| Path 328 | C00031->C00037:[4->1,9->2] | 1.00 | 460.454545455 | 25 | 55 | 0 | 1 |
| Path 329 | C00031->C00037:[4->1,7->2] | 1.00 | 372.87755102 | 16 | 49 | 0 | 0 |
| Path 330 | C00031->C00037:[4->2] | 0.50 | 168.356756757 | 17 | 185 | 0 | 0 |
| Path 331 | C00031->C00037:[4->1,5->2] | 1.00 | 616.457446809 | 25 | 94 | 0 | 0 |
| Path 332 | C00031->C00037:[4->1,5->2,7->1] | 1.00 | 345.563829787 | 26 | 94 | 0 | 0 |
| Path 333 | C00031->C00037:[5->2,7->1] | 1.00 | 434.837209302 | 20 | 43 | 0 | 0 |
| Path 334 | C00031->C00037:[4->1,5->2] | 1.00 | 320.448275862 | 20 | 87 | 0 | 0 |
| Path 335 | C00031->C00037:[7->1,9->2] | 1.00 | 298.032786885 | 17 | 61 | 0 | 1 |
| Path 336 | C00031->C00037:[4->1,4->2,7->1,7->2] | 1.00 | 176.140703518 | 25 | 199 | 0 | 2 |
| Path 337 | C00031->C00037:[7->1,9->2] | 1.00 | 349.656716418 | 24 | 67 | 0 | 1 |
| Path 338 | C00031->C00037:[4->1,4->2] | 1.00 | 439.818181818 | 12 | 33 | 0 | 1 |
| Path 339 | C00031->C00037:[4->1,5->2] | 1.00 | 868.767857143 | 23 | 56 | 0 | 0 |
| Path 340 | C00031->C00037:[4->1,4->2] | 1.00 | 432.066666667 | 11 | 30 | 0 | 1 |
| Path 341 | C00031->C00037:[4->1,5->2,7->1] | 1.00 | 356.212765957 | 26 | 94 | 0 | 0 |
| Path 342 | C00031->C00037:[9->2] | 0.50 | 409.192307692 | 8 | 26 | 0 | 0 |
| Path 343 | C00031->C00037:[4->1,5->2] | 1.00 | 597.166666667 | 23 | 96 | 0 | 0 |
| Path 344 | C00031->C00037:[4->1,9->2] | 1.00 | 376.141176471 | 31 | 85 | 0 | 1 |
| Path 345 | C00031->C00037:[4->1,5->2] | 1.00 | 331.02247191 | 22 | 89 | 0 | 0 |
| Path 346 | C00031->C00037:[5->2,7->1] | 1.00 | 320.024390244 | 20 | 82 | 0 | 0 |
| Path 347 | C00031->C00037:[4->1,9->2] | 1.00 | 436.581818182 | 26 | 55 | 0 | 1 |
| Path 348 | C00031->C00037:[4->1,5->2] | 1.00 | 825.827586207 | 23 | 58 | 0 | 0 |
| Path 349 | C00031->C00037:[4->1,9->2] | 1.00 | 427.759259259 | 23 | 54 | 0 | 1 |
| Path 350 | C00031->C00037:[4->1,5->2] | 1.00 | 343.976744186 | 24 | 86 | 0 | 0 |
| Path 351 | C00031->C00037:[4->1,5->2] | 1.00 | 337.843373494 | 22 | 83 | 0 | 0 |
| Path 352 | C00031->C00037:[4->1,7->2] | 1.00 | 280.301369863 | 17 | 73 | 0 | 0 |
| Path 353 | C00031->C00037:[7->1,9->2] | 1.00 | 337.115942029 | 23 | 69 | 0 | 1 |
| Path 354 | C00031->C00037:[9->2] | 0.50 | 252.31372549 | 10 | 51 | 0 | 1 |
| Path 355 | C00031->C00037:[4->1,9->2] | 1.00 | 389.222222222 | 20 | 36 | 0 | 1 |
| Path 356 | C00031->C00037:[4->1,7->1,9->2] | 1.00 | 465.462962963 | 26 | 54 | 0 | 1 |
| Path 357 | C00031->C00037:[5->2,7->1] | 1.00 | 413.6875 | 20 | 48 | 0 | 0 |
| Path 358 | C00031->C00037:[4->1,9->2] | 1.00 | 364.684210526 | 28 | 76 | 0 | 1 |
| Path 359 | C00031->C00037:[4->1,5->2] | 1.00 | 335.890243902 | 22 | 82 | 0 | 0 |
| Path 360 | C00031->C00037:[7->1,9->2] | 1.00 | 451.2 | 21 | 45 | 0 | 1 |
| Path 361 | C00031->C00037:[4->1,9->2] | 1.00 | 491.808510638 | 24 | 47 | 0 | 1 |
| Path 362 | C00031->C00037:[4->1,9->2] | 1.00 | 357.901408451 | 25 | 71 | 0 | 1 |
| Path 363 | C00031->C00037:[4->1,5->2] | 1.00 | 349.354166667 | 28 | 96 | 0 | 0 |
| Path 364 | C00031->C00037:[4->1,5->2] | 1.00 | 361.670454545 | 26 | 88 | 0 | 0 |
| Path 365 | C00031->C00037:[5->2,9->1] | 1.00 | 333.373134328 | 21 | 67 | 0 | 0 |
| Path 366 | C00031->C00037:[4->1,4->2] | 1.00 | 486.829268293 | 20 | 41 | 0 | 1 |
| Path 367 | C00031->C00037:[9->1,9->2] | 1.00 | 275.703703704 | 12 | 54 | 0 | 1 |
| Path 368 | C00031->C00037:[7->1,9->2] | 1.00 | 364.564102564 | 29 | 78 | 0 | 1 |
| Path 369 | C00031->C00037:[5->2,9->1] | 1.00 | 337.347222222 | 24 | 72 | 0 | 0 |
| Path 370 | C00031->C00037:[4->1,9->2] | 1.00 | 437.131147541 | 26 | 61 | 0 | 1 |
| Path 371 | C00031->C00037:[4->1,5->2,7->1] | 1.00 | 338.744186047 | 21 | 86 | 0 | 0 |
| Path 372 | C00031->C00037:[7->1,9->2] | 1.00 | 366.424657534 | 27 | 73 | 0 | 1 |
| Path 373 | C00031->C00037:[7->1] | 0.50 | 350.6875 | 8 | 16 | 0 | 1 |
| Path 374 | C00031->C00037:[4->1,4->2,7->1,7->2] | 1.00 | 170.907407407 | 15 | 108 | 0 | 2 |
| Path 375 | C00031->C00037:[4->1,9->2] | 1.00 | 437.935483871 | 27 | 62 | 0 | 1 |
| Path 376 | C00031->C00037:[4->1,5->2] | 1.00 | 823.456140351 | 20 | 57 | 0 | 0 |
| Path 377 | C00031->C00037:[4->1,9->2] | 1.00 | 170.274509804 | 24 | 204 | 0 | 2 |
| Path 378 | C00031->C00037:[4->1] | 0.50 | 284.833333333 | 9 | 24 | 0 | 1 |
| Path 379 | C00031->C00037:[4->1,9->2] | 1.00 | 364.364864865 | 26 | 74 | 0 | 1 |
| Path 380 | C00031->C00037:[4->1,9->2] | 1.00 | 375.62962963 | 30 | 81 | 0 | 1 |
| Path 381 | C00031->C00037:[4->1,4->2] | 1.00 | 168.868571429 | 20 | 175 | 0 | 2 |
| Path 382 | C00031->C00037:[7->1,9->2] | 1.00 | 428.314814815 | 24 | 54 | 0 | 1 |
| Path 383 | C00031->C00037:[4->1,4->2] | 1.00 | 184.251282051 | 24 | 195 | 0 | 2 |
| Path 384 | C00031->C00037:[4->1,9->2] | 1.00 | 385.0 | 26 | 68 | 0 | 1 |
| Path 385 | C00031->C00037:[4->1,4->2,7->1,7->2] | 1.00 | 171.290816327 | 23 | 196 | 0 | 2 |
| Path 386 | C00031->C00037:[4->1,4->2,7->1,7->2] | 1.00 | 169.297435897 | 23 | 195 | 0 | 2 |
| Path 387 | C00031->C00037:[4->1,4->2,7->1,7->2] | 1.00 | 167.889502762 | 22 | 181 | 0 | 2 |
| Path 388 | C00031->C00037:[4->1,9->2] | 1.00 | 287.948275862 | 14 | 58 | 0 | 1 |
| Path 389 | C00031->C00037:[4->1,4->2,7->1,7->2] | 1.00 | 172.893401015 | 24 | 197 | 0 | 2 |
| Path 390 | C00031->C00037:[4->1,4->2,7->1,7->2] | 1.00 | 403.902439024 | 15 | 41 | 0 | 1 |
| Path 391 | C00031->C00037:[4->1,5->2] | 1.00 | 457.0 | 22 | 49 | 0 | 0 |
| Path 392 | C00031->C00037:[4->1,4->2,7->1,7->2] | 1.00 | 185.584158416 | 26 | 202 | 0 | 2 |
| Path 393 | C00031->C00037:[4->1,7->1,7->2] | 1.00 | 319.325581395 | 21 | 86 | 0 | 0 |
| Path 394 | C00031->C00037:[4->1,5->2] | 1.00 | 455.425925926 | 25 | 54 | 0 | 1 |
| Path 395 | C00031->C00037:[4->1,5->2,7->1] | 1.00 | 335.760869565 | 25 | 92 | 0 | 0 |
| Path 396 | C00031->C00037:[4->1,5->2] | 1.00 | 413.58 | 19 | 50 | 0 | 0 |
| Path 397 | C00031->C00037:[7->1,9->2] | 1.00 | 264.724137931 | 14 | 58 | 0 | 1 |
| Path 398 | C00031->C00037:[4->1,7->2] | 1.00 | 357.745454545 | 17 | 55 | 0 | 0 |
| Path 399 | C00031->C00037:[7->1,9->2] | 1.00 | 272.537037037 | 13 | 54 | 0 | 1 |
| Path 400 | C00031->C00037:[4->1,4->2,7->1,7->2] | 1.00 | 180.845771144 | 27 | 201 | 0 | 2 |
| Path 401 | C00031->C00037:[4->1,9->2] | 1.00 | 325.618181818 | 18 | 55 | 0 | 1 |
| Path 402 | C00031->C00037:[4->1,4->2] | 1.00 | 153.195876289 | 11 | 97 | 0 | 2 |
| Path 403 | C00031->C00037:[5->2] | 0.50 | 396.666666667 | 12 | 51 | 0 | 0 |
| Path 404 | C00031->C00037:[4->1,5->2] | 1.00 | 617.731182796 | 25 | 93 | 0 | 0 |
| Path 405 | C00031->C00037:[4->1,9->2] | 1.00 | 379.769230769 | 29 | 78 | 0 | 1 |
| Path 406 | C00031->C00037:[4->1,4->2] | 1.00 | 151.269461078 | 18 | 167 | 0 | 2 |
| Path 407 | C00031->C00037:[4->1,9->2] | 1.00 | 445.366666667 | 28 | 60 | 0 | 1 |
| Path 408 | C00031->C00037:[4->1,5->2] | 1.00 | 426.941176471 | 20 | 51 | 0 | 0 |
| Path 409 | C00031->C00037:[4->1,5->2] | 1.00 | 322.340909091 | 21 | 88 | 0 | 0 |
| Path 410 | C00031->C00037:[4->1,9->2] | 1.00 | 440.684210526 | 25 | 57 | 0 | 1 |
| Path 411 | C00031->C00037:[4->1,9->2] | 1.00 | 165.094527363 | 23 | 201 | 0 | 2 |
| Path 412 | C00031->C00037:[4->1,4->2,7->1,7->2] | 1.00 | 170.93956044 | 22 | 182 | 0 | 2 |
| Path 413 | C00031->C00037:[4->1,4->2,7->1,7->2] | 1.00 | 176.556756757 | 23 | 185 | 0 | 2 |
| Path 414 | C00031->C00037:[4->1,9->2] | 1.00 | 314.557692308 | 17 | 52 | 0 | 1 |
| Path 415 | C00031->C00037:[7->1,7->2] | 1.00 | 374.323529412 | 11 | 34 | 0 | 1 |
| Path 416 | C00031->C00037:[4->1,4->2,7->1,7->2] | 1.00 | 162.0 | 21 | 178 | 0 | 2 |
| Path 417 | C00031->C00037:[4->1,4->2,7->1,7->2] | 1.00 | 161.00952381 | 14 | 105 | 0 | 2 |
| Path 418 | C00031->C00037:[4->1] | 0.50 | 372.0 | 11 | 37 | 0 | 0 |
| Path 419 | C00031->C00037:[4->1,4->2] | 1.00 | 156.602040816 | 12 | 98 | 0 | 2 |
| Path 420 | C00031->C00037:[4->1,7->2] | 1.00 | 297.796296296 | 23 | 108 | 0 | 0 |
| Path 421 | C00031->C00037:[5->2,7->1] | 1.00 | 442.925 | 18 | 40 | 0 | 0 |
| Path 422 | C00031->C00037:[4->1,5->2] | 1.00 | 366.591836735 | 16 | 49 | 0 | 0 |
| Path 423 | C00031->C00037:[4->1,5->2] | 1.00 | 356.411111111 | 26 | 90 | 0 | 0 |
| Path 424 | C00031->C00037:[4->1,7->2] | 1.00 | 400.282608696 | 17 | 46 | 0 | 0 |
| Path 425 | C00031->C00037:[4->1,4->2] | 1.00 | 141.574468085 | 10 | 94 | 0 | 2 |
| Path 426 | C00031->C00037:[4->1,9->2] | 1.00 | 343.463768116 | 23 | 69 | 0 | 1 |
| Path 427 | C00031->C00037:[7->1,9->2] | 1.00 | 434.183673469 | 23 | 49 | 0 | 1 |
| Path 428 | C00031->C00037:[4->1,5->2,7->1] | 1.00 | 340.947916667 | 26 | 96 | 0 | 0 |
| Path 429 | C00031->C00037:[5->2,7->1] | 1.00 | 384.352941176 | 20 | 51 | 0 | 0 |
| Path 430 | C00031->C00037:[5->2,7->1] | 1.00 | 407.095238095 | 16 | 42 | 0 | 0 |
| Path 431 | C00031->C00037:[5->2,9->1] | 1.00 | 323.090909091 | 20 | 66 | 0 | 0 |
| Path 432 | C00031->C00037:[4->1,7->1,7->2] | 1.00 | 339.747126437 | 22 | 87 | 0 | 0 |
| Path 433 | C00031->C00037:[4->1,9->2] | 1.00 | 465.694915254 | 28 | 59 | 0 | 1 |
| Path 434 | C00031->C00037:[9->2] | 0.50 | 384.68 | 7 | 25 | 0 | 0 |
| Path 435 | C00031->C00037:[4->1,9->2] | 1.00 | 365.5 | 27 | 78 | 0 | 1 |
| Path 436 | C00031->C00037:[4->1] | 0.50 | 487.166666667 | 8 | 12 | 0 | 1 |
| Path 437 | C00031->C00037:[4->1,5->2] | 1.00 | 875.509090909 | 23 | 55 | 0 | 0 |
| Path 438 | C00031->C00037:[4->1,4->2] | 1.00 | 168.936170213 | 22 | 188 | 0 | 2 |
| Path 439 | C00031->C00037:[5->2,7->1] | 1.00 | 429.0 | 21 | 50 | 0 | 0 |
| Path 440 | C00031->C00037:[4->1,5->2] | 1.00 | 452.066666667 | 21 | 45 | 0 | 0 |
| Path 441 | C00031->C00037:[4->1,4->2,7->1,7->2] | 1.00 | 165.911917098 | 22 | 193 | 0 | 2 |
| Path 442 | C00031->C00037:[4->1,9->2] | 1.00 | 462.928571429 | 27 | 56 | 0 | 1 |
| Path 443 | C00031->C00037:[5->2,7->1] | 1.00 | 307.569620253 | 17 | 79 | 0 | 0 |
| Path 444 | C00031->C00037:[4->1,5->2] | 1.00 | 327.727272727 | 23 | 88 | 0 | 0 |
| Path 445 | C00031->C00037:[4->1,9->2] | 1.00 | 391.930555556 | 28 | 72 | 0 | 1 |
| Path 446 | C00031->C00037:[4->1,9->2] | 1.00 | 377.575757576 | 19 | 33 | 0 | 1 |
| Path 447 | C00031->C00037:[4->1,4->2] | 1.00 | 156.17032967 | 18 | 182 | 0 | 2 |
| Path 448 | C00031->C00037:[4->1,4->2] | 1.00 | 330.604166667 | 13 | 96 | 0 | 2 |
| Path 449 | C00031->C00037:[4->1,5->2] | 1.00 | 317.392857143 | 27 | 112 | 0 | 0 |
| Path 450 | C00031->C00037:[9->2] | 0.50 | 311.861111111 | 8 | 36 | 0 | 0 |
| Path 451 | C00031->C00037:[4->1,9->2] | 1.00 | 459.962962963 | 24 | 54 | 0 | 1 |
| Path 452 | C00031->C00037:[5->2] | 0.50 | 409.357142857 | 16 | 42 | 0 | 0 |
| Path 453 | C00031->C00037:[4->1,5->2] | 1.00 | 365.636363636 | 25 | 88 | 0 | 0 |
| Path 454 | C00031->C00037:[4->1,4->2,7->1,7->2] | 1.00 | 182.48 | 25 | 200 | 0 | 2 |
| Path 455 | C00031->C00037:[4->1,5->2] | 1.00 | 358.773809524 | 23 | 84 | 0 | 0 |
| Path 456 | C00031->C00037:[4->1,4->2] | 1.00 | 161.527173913 | 20 | 184 | 0 | 2 |
| Path 457 | C00031->C00037:[4->1,9->2] | 1.00 | 441.482758621 | 26 | 58 | 0 | 1 |
| Path 458 | C00031->C00037:[4->1,7->2] | 1.00 | 351.287234043 | 26 | 94 | 0 | 0 |
| Path 459 | C00031->C00037:[4->1,7->1,9->2] | 1.00 | 442.0 | 28 | 61 | 0 | 1 |
| Path 460 | C00031->C00037:[5->2,7->1] | 1.00 | 415.408163265 | 20 | 49 | 0 | 0 |
| Path 461 | C00031->C00037:[4->1,9->2] | 1.00 | 350.708333333 | 24 | 72 | 0 | 1 |
| Path 462 | C00031->C00037:[4->1,7->2] | 1.00 | 280.990654206 | 22 | 107 | 0 | 0 |
| Path 463 | C00031->C00037:[4->1,4->2] | 1.00 | 322.815217391 | 11 | 92 | 0 | 2 |
| Path 464 | C00031->C00037:[4->1,5->2] | 1.00 | 301.369369369 | 26 | 111 | 0 | 0 |
| Path 465 | C00031->C00037:[7->1,9->2] | 1.00 | 451.980392157 | 25 | 51 | 0 | 1 |
| Path 466 | C00031->C00037:[4->1,5->2] | 1.00 | 342.269662921 | 22 | 89 | 0 | 0 |
| Path 467 | C00031->C00037:[4->1,9->2] | 1.00 | 399.229508197 | 24 | 61 | 0 | 1 |
| Path 468 | C00031->C00037:[4->1,4->2,7->1,7->2] | 1.00 | 390.604166667 | 17 | 48 | 0 | 1 |
| Path 469 | C00031->C00037:[5->2,7->1] | 1.00 | 432.788461538 | 23 | 52 | 0 | 0 |
| Path 470 | C00031->C00037:[7->1,9->2] | 1.00 | 416.234042553 | 21 | 47 | 0 | 1 |
| Path 471 | C00031->C00037:[7->1,9->2] | 1.00 | 438.227272727 | 20 | 44 | 0 | 1 |
| Path 472 | C00031->C00037:[4->1,4->2] | 1.00 | 456.85 | 18 | 40 | 0 | 1 |
| Path 473 | C00031->C00037:[5->2,7->1] | 1.00 | 423.155555556 | 19 | 45 | 0 | 0 |
| Path 474 | C00031->C00037:[4->1,5->2,7->1] | 1.00 | 357.455555556 | 25 | 90 | 0 | 0 |
| Path 475 | C00031->C00037:[5->2,7->1] | 1.00 | 390.317073171 | 15 | 41 | 0 | 0 |
| Path 476 | C00031->C00037:[7->1,9->2] | 1.00 | 431.921568627 | 25 | 51 | 0 | 1 |
| Path 477 | C00031->C00037:[4->1,5->2] | 1.00 | 336.277777778 | 23 | 90 | 0 | 0 |
| Path 478 | C00031->C00037:[5->2,9->1] | 1.00 | 320.453333333 | 24 | 75 | 0 | 0 |
| Path 479 | C00031->C00037:[4->1,4->2] | 1.00 | 182.098654709 | 28 | 223 | 0 | 2 |
| Path 480 | C00031->C00037:[5->2] | 0.50 | 434.473684211 | 17 | 38 | 0 | 0 |
| Path 481 | C00031->C00037:[4->1,4->2] | 1.00 | 159.514619883 | 19 | 171 | 0 | 2 |
