# Supplementary material for: 13C-metabolic flux ratio and novel carbon path analyses confirmed that Trichoderma reesei uses primarily the respirative pathway also on the preferred carbon source glucose
Source: BMC Syst Biol. 2009 Oct 29;3:104. doi: 10.1186/1752-0509-3-104 (PMC2776023; doi:10.1186/1752-0509-3-104)
Supplement: Additional file 1 — Pathways discovered in ReTrace carbon path analysis. Graphical and tabular representations of amino acid synthesis pathways discovered in ReTrace carbon path analysis [21]. Self-contained web site: unpack zip archive and open index.html with a web browser. [file 1752-0509-3-104-S1.zip › AF1-treesei/pathways-C00031-to-C00152.html]

Pathways from C00031 to C00152


**Pathways from C00031 to C00152**

**Sources:** D-Glucose; (C00031)

**Target:**L-Asparagine; (C00152)

|  | Composite mapping | Z | Average score | Rpairs | Reactions | Zero scores | Scores under threshold |
| --- | --- | --- | --- | --- | --- | --- | --- |
| Path 1 | C00031->C00152:[1->1,2->3,2->5,4->2] | 1.00 | 380.64 | 14 | 75 | 0 | 0 |
| Path 2 | C00031->C00152:[1->1,2->3,4->2,9->5] | 1.00 | 319.65 | 17 | 60 | 0 | 0 |
| Path 3 | C00031->C00152:[1->1,2->3,4->2,9->5] | 1.00 | 310.017857143 | 19 | 56 | 0 | 0 |
| Path 4 | C00031->C00152:[4->3,4->5,7->2,9->1] | 1.00 | 324.584158416 | 21 | 101 | 0 | 1 |
| Path 5 | C00031->C00152:[4->5,5->3,7->2,9->1] | 1.00 | 347.627737226 | 29 | 137 | 0 | 0 |
| Path 6 | C00031->C00152:[4->3,4->5,7->2,7->3,7->5,9->1] | 1.00 | 427.391304348 | 24 | 69 | 0 | 1 |
| Path 7 | C00031->C00152:[5->3,7->2,9->1,9->5] | 1.00 | 315.46875 | 22 | 64 | 0 | 0 |
| Path 8 | C00031->C00152:[1->1,2->3,4->2,7->5] | 1.00 | 308.4 | 18 | 55 | 0 | 0 |
| Path 9 | C00031->C00152:[1->1,2->3,4->2,5->5] | 1.00 | 325.420289855 | 21 | 69 | 0 | 0 |
| Path 10 | C00031->C00152:[7->2,9->1,9->3,9->5] | 1.00 | 305.273809524 | 14 | 84 | 0 | 0 |
| Path 11 | C00031->C00152:[4->5,5->3,7->2,7->5,9->1] | 1.00 | 385.373913043 | 27 | 115 | 0 | 0 |
| Path 12 | C00031->C00152:[4->3,4->5,7->2,9->1] | 1.00 | 331.330188679 | 23 | 106 | 0 | 0 |
| Path 13 | C00031->C00152:[1->1,2->3,4->2,7->5] | 1.00 | 320.5 | 14 | 48 | 0 | 0 |
| Path 14 | C00031->C00152:[4->3,4->5,7->2,9->1] | 1.00 | 557.29245283 | 22 | 106 | 0 | 0 |
| Path 15 | C00031->C00152:[1->1,2->3,4->2,4->5] | 1.00 | 337.910714286 | 18 | 56 | 0 | 0 |
| Path 16 | C00031->C00152:[5->3,7->2,9->1,9->5] | 1.00 | 268.732394366 | 17 | 71 | 0 | 0 |
| Path 17 | C00031->C00152:[1->1,2->3,4->2,9->5] | 1.00 | 272.875 | 20 | 72 | 0 | 0 |
| Path 18 | C00031->C00152:[1->1,2->3,4->2,4->5] | 1.00 | 344.0 | 16 | 55 | 0 | 0 |
| Path 19 | C00031->C00152:[1->1,2->3,4->2,4->5] | 1.00 | 574.94214876 | 25 | 121 | 0 | 0 |
| Path 20 | C00031->C00152:[5->3,7->2,9->1,9->5] | 1.00 | 354.186440678 | 22 | 59 | 0 | 0 |
| Path 21 | C00031->C00152:[1->1,2->3,4->2,7->5] | 1.00 | 290.063829787 | 15 | 47 | 0 | 0 |
