# Supplementary material for: 13C-metabolic flux ratio and novel carbon path analyses confirmed that Trichoderma reesei uses primarily the respirative pathway also on the preferred carbon source glucose
Source: BMC Syst Biol. 2009 Oct 29;3:104. doi: 10.1186/1752-0509-3-104 (PMC2776023; doi:10.1186/1752-0509-3-104)
Supplement: Additional file 1 — Pathways discovered in ReTrace carbon path analysis. Graphical and tabular representations of amino acid synthesis pathways discovered in ReTrace carbon path analysis [21]. Self-contained web site: unpack zip archive and open index.html with a web browser. [file 1752-0509-3-104-S1.zip › AF1-treesei/pathways-C00031-to-C00097.html]

Pathways from C00031 to C00097


**Pathways from C00031 to C00097**

**Sources:** D-Glucose; (C00031)

**Target:**L-Cysteine; (C00097)

|  | Composite mapping | Z | Average score | Rpairs | Reactions | Zero scores | Scores under threshold |
| --- | --- | --- | --- | --- | --- | --- | --- |
| Path 1 | C00031->C00097:[1->1,2->2,4->3] | 1.00 | 421.962962963 | 12 | 27 | 0 | 0 |
| Path 2 | C00031->C00097:[4->1,4->2,7->3,9->1] | 1.00 | 283.984375 | 16 | 64 | 0 | 1 |
| Path 3 | C00031->C00097:[1->1,2->2,4->3] | 1.00 | 507.820512821 | 12 | 39 | 0 | 0 |
| Path 4 | C00031->C00097:[4->2,5->1,9->3] | 1.00 | 423.756756757 | 14 | 37 | 0 | 0 |
| Path 5 | C00031->C00097:[7->3,9->1,9->2] | 1.00 | 434.25 | 15 | 36 | 0 | 0 |
| Path 6 | C00031->C00097:[4->2,5->1,9->3] | 1.00 | 410.756756757 | 14 | 37 | 0 | 0 |
| Path 7 | C00031->C00097:[1->1,2->2,4->3] | 1.00 | 559.115384615 | 10 | 26 | 0 | 0 |
| Path 8 | C00031->C00097:[4->2,5->1,9->3] | 1.00 | 401.352941176 | 13 | 34 | 0 | 0 |
| Path 9 | C00031->C00097:[1->1,2->2,4->3] | 1.00 | 506.615384615 | 12 | 39 | 0 | 0 |
| Path 10 | C00031->C00097:[7->3,9->1,9->2] | 1.00 | 347.016393443 | 19 | 61 | 0 | 0 |
| Path 11 | C00031->C00097:[4->2,5->3,9->1] | 1.00 | 473.390243902 | 18 | 41 | 0 | 0 |
| Path 12 | C00031->C00097:[4->1,4->2,7->3,9->1] | 1.00 | 301.318181818 | 18 | 66 | 0 | 1 |
| Path 13 | C00031->C00097:[4->2,7->3,9->1] | 1.00 | 279.907692308 | 16 | 65 | 0 | 1 |
| Path 14 | C00031->C00097:[7->3,9->1,9->2] | 1.00 | 266.403225806 | 15 | 62 | 0 | 0 |
| Path 15 | C00031->C00097:[1->1,2->2,4->3] | 1.00 | 527.516129032 | 10 | 31 | 0 | 0 |
| Path 16 | C00031->C00097:[4->2,5->1,9->3] | 1.00 | 415.5 | 13 | 34 | 0 | 0 |
| Path 17 | C00031->C00097:[5->2,7->3,9->1] | 1.00 | 382.02 | 14 | 50 | 0 | 0 |
| Path 18 | C00031->C00097:[4->2,7->3,9->1] | 1.00 | 264.836065574 | 14 | 61 | 0 | 1 |
| Path 19 | C00031->C00097:[4->2,5->3,9->1] | 1.00 | 469.921052632 | 17 | 38 | 0 | 0 |
| Path 20 | C00031->C00097:[5->3,7->2,9->1] | 1.00 | 430.675675676 | 15 | 37 | 0 | 0 |
| Path 21 | C00031->C00097:[1->1,2->2,4->3] | 1.00 | 502.279069767 | 11 | 43 | 0 | 0 |
| Path 22 | C00031->C00097:[4->2,7->3,9->1] | 1.00 | 417.9375 | 11 | 32 | 0 | 0 |
| Path 23 | C00031->C00097:[1->1,2->2,4->3] | 1.00 | 499.536585366 | 11 | 41 | 0 | 0 |
| Path 24 | C00031->C00097:[4->2,7->3,9->1] | 1.00 | 411.617647059 | 11 | 34 | 0 | 0 |
| Path 25 | C00031->C00097:[1->1,2->2,4->3] | 1.00 | 398.193548387 | 12 | 31 | 0 | 0 |
| Path 26 | C00031->C00097:[7->3,9->1,9->2] | 1.00 | 268.619047619 | 16 | 63 | 0 | 0 |
| Path 27 | C00031->C00097:[7->3,9->1,9->2] | 1.00 | 358.46875 | 9 | 32 | 0 | 0 |
| Path 28 | C00031->C00097:[1->1,2->2,4->3] | 1.00 | 563.9375 | 12 | 32 | 0 | 0 |
| Path 29 | C00031->C00097:[7->3,9->1,9->2] | 1.00 | 261.258064516 | 12 | 62 | 0 | 0 |
| Path 30 | C00031->C00097:[1->1,2->2,4->3] | 1.00 | 528.387096774 | 11 | 31 | 0 | 0 |
| Path 31 | C00031->C00097:[7->3,9->1,9->2] | 1.00 | 387.111111111 | 13 | 36 | 0 | 0 |
| Path 32 | C00031->C00097:[7->3,9->1,9->2] | 1.00 | 262.733333333 | 13 | 60 | 0 | 0 |
| Path 33 | C00031->C00097:[4->2,7->3,9->1] | 1.00 | 400.56097561 | 13 | 41 | 0 | 0 |
| Path 34 | C00031->C00097:[1->1,2->2,4->3] | 1.00 | 425.923076923 | 12 | 39 | 0 | 0 |
| Path 35 | C00031->C00097:[7->3,9->1,9->2] | 1.00 | 284.0 | 11 | 49 | 0 | 0 |
| Path 36 | C00031->C00097:[7->3,9->1,9->2] | 1.00 | 425.388888889 | 12 | 36 | 0 | 0 |
| Path 37 | C00031->C00097:[1->1,2->2,4->3] | 1.00 | 426.0 | 10 | 31 | 0 | 0 |
| Path 38 | C00031->C00097:[4->2,5->3,9->1] | 1.00 | 447.80952381 | 17 | 42 | 0 | 0 |
| Path 39 | C00031->C00097:[5->2,5->3,9->1] | 1.00 | 426.321428571 | 20 | 56 | 0 | 0 |
| Path 40 | C00031->C00097:[7->3,9->1,9->2] | 1.00 | 351.45 | 12 | 40 | 0 | 0 |
| Path 41 | C00031->C00097:[7->3,9->1,9->2] | 1.00 | 311.056338028 | 20 | 71 | 0 | 0 |
| Path 42 | C00031->C00097:[1->1,2->2,4->3] | 1.00 | 529.088235294 | 12 | 34 | 0 | 0 |
| Path 43 | C00031->C00097:[7->3,9->1,9->2] | 1.00 | 343.111111111 | 17 | 63 | 0 | 0 |
| Path 44 | C00031->C00097:[4->2,5->3,9->1] | 1.00 | 452.444444444 | 18 | 45 | 0 | 0 |
| Path 45 | C00031->C00097:[1->1,2->2,4->3] | 1.00 | 472.606060606 | 12 | 33 | 0 | 0 |
| Path 46 | C00031->C00097:[5->1,7->2,9->3] | 1.00 | 375.785714286 | 10 | 28 | 0 | 0 |
| Path 47 | C00031->C00097:[7->3,9->1,9->2] | 1.00 | 471.133333333 | 20 | 45 | 0 | 0 |
| Path 48 | C00031->C00097:[7->3,9->1,9->2] | 1.00 | 310.726027397 | 18 | 73 | 0 | 0 |
| Path 49 | C00031->C00097:[7->3,9->1,9->2] | 1.00 | 463.808510638 | 18 | 47 | 0 | 0 |
| Path 50 | C00031->C00097:[7->3,9->1,9->2] | 1.00 | 344.790322581 | 16 | 62 | 0 | 0 |
| Path 51 | C00031->C00097:[7->3,9->1,9->2] | 1.00 | 297.115384615 | 14 | 52 | 0 | 0 |
| Path 52 | C00031->C00097:[7->3,9->1,9->2] | 1.00 | 305.546666667 | 21 | 75 | 0 | 0 |
| Path 53 | C00031->C00097:[4->2,5->1,9->3] | 1.00 | 474.212765957 | 21 | 47 | 0 | 0 |
| Path 54 | C00031->C00097:[1->1,2->2,4->3] | 1.00 | 505.444444444 | 11 | 36 | 0 | 0 |
| Path 55 | C00031->C00097:[4->2,7->2,7->3,9->1] | 1.00 | 395.240506329 | 22 | 79 | 0 | 1 |
| Path 56 | C00031->C00097:[7->3,9->1,9->2] | 1.00 | 433.486486486 | 16 | 37 | 0 | 0 |
| Path 57 | C00031->C00097:[1->1,2->2,4->3] | 1.00 | 498.421052632 | 12 | 38 | 0 | 0 |
| Path 58 | C00031->C00097:[1->1,2->2,4->3] | 1.00 | 519.868421053 | 10 | 38 | 0 | 0 |
| Path 59 | C00031->C00097:[4->2,4->3,9->1] | 1.00 | 383.553571429 | 19 | 56 | 0 | 0 |
| Path 60 | C00031->C00097:[4->2,7->3,9->1] | 1.00 | 401.387096774 | 10 | 31 | 0 | 0 |
| Path 61 | C00031->C00097:[4->2,7->2,7->3,9->1] | 1.00 | 385.346938776 | 19 | 49 | 0 | 1 |
| Path 62 | C00031->C00097:[7->3,9->1,9->2] | 1.00 | 428.543478261 | 15 | 46 | 0 | 0 |
| Path 63 | C00031->C00097:[7->3,9->1,9->2] | 1.00 | 344.557377049 | 19 | 61 | 0 | 0 |
| Path 64 | C00031->C00097:[4->2,5->3,9->1] | 1.00 | 452.363636364 | 15 | 44 | 0 | 0 |
| Path 65 | C00031->C00097:[7->3,9->1,9->2] | 1.00 | 290.980769231 | 11 | 52 | 0 | 0 |
| Path 66 | C00031->C00097:[1->1,2->2,4->3] | 1.00 | 519.8 | 9 | 35 | 0 | 0 |
| Path 67 | C00031->C00097:[7->2,7->3,9->1] | 1.00 | 379.029411765 | 11 | 34 | 0 | 0 |
| Path 68 | C00031->C00097:[7->3,9->1,9->2] | 1.00 | 345.492063492 | 17 | 63 | 0 | 0 |
| Path 69 | C00031->C00097:[4->2,5->1,9->3] | 1.00 | 472.791666667 | 22 | 48 | 0 | 0 |
| Path 70 | C00031->C00097:[1->1,2->3,4->2] | 1.00 | 450.955555556 | 16 | 45 | 0 | 0 |
| Path 71 | C00031->C00097:[7->3,9->1,9->2] | 1.00 | 449.632653061 | 21 | 49 | 0 | 0 |
| Path 72 | C00031->C00097:[4->2,7->2,7->3,9->1] | 1.00 | 281.3125 | 17 | 64 | 0 | 1 |
| Path 73 | C00031->C00097:[1->1,2->3,4->2] | 1.00 | 455.104166667 | 17 | 48 | 0 | 0 |
| Path 74 | C00031->C00097:[4->2,5->3,9->1] | 1.00 | 456.510638298 | 16 | 47 | 0 | 0 |
| Path 75 | C00031->C00097:[4->2,7->3,9->1] | 1.00 | 370.282608696 | 16 | 46 | 0 | 1 |
| Path 76 | C00031->C00097:[5->1,5->2,9->3] | 1.00 | 399.212765957 | 16 | 47 | 0 | 0 |
| Path 77 | C00031->C00097:[4->2,7->2,7->3,9->1] | 1.00 | 394.735849057 | 21 | 53 | 0 | 1 |
| Path 78 | C00031->C00097:[7->3,9->1,9->2] | 1.00 | 300.0 | 14 | 52 | 0 | 0 |
| Path 79 | C00031->C00097:[7->2,7->3,9->1] | 1.00 | 406.785714286 | 10 | 28 | 0 | 0 |
| Path 80 | C00031->C00097:[5->1,7->2,9->3] | 1.00 | 404.766666667 | 12 | 30 | 0 | 0 |
| Path 81 | C00031->C00097:[7->2,7->3,9->1] | 1.00 | 387.37037037 | 9 | 27 | 0 | 0 |
| Path 82 | C00031->C00097:[1->1,2->2,4->3] | 1.00 | 412.78125 | 12 | 32 | 0 | 0 |
| Path 83 | C00031->C00097:[7->3,9->1,9->2] | 1.00 | 338.446153846 | 20 | 65 | 0 | 0 |
| Path 84 | C00031->C00097:[4->2,4->3,9->1] | 1.00 | 389.901960784 | 18 | 51 | 0 | 0 |
| Path 85 | C00031->C00097:[7->3,9->1,9->2] | 1.00 | 415.0 | 19 | 49 | 0 | 0 |
| Path 86 | C00031->C00097:[7->3,9->1,9->2] | 1.00 | 242.275862069 | 11 | 58 | 0 | 0 |
| Path 87 | C00031->C00097:[7->3,9->1,9->2] | 1.00 | 299.169811321 | 15 | 53 | 0 | 0 |
| Path 88 | C00031->C00097:[7->3,9->1,9->2] | 1.00 | 311.907407407 | 13 | 54 | 0 | 0 |
| Path 89 | C00031->C00097:[7->3,9->1,9->2] | 1.00 | 387.735294118 | 11 | 34 | 0 | 0 |
| Path 90 | C00031->C00097:[5->2,5->3,9->1] | 1.00 | 407.244444444 | 14 | 45 | 0 | 0 |
| Path 91 | C00031->C00097:[1->1,2->2,4->3] | 1.00 | 516.266666667 | 10 | 30 | 0 | 0 |
| Path 92 | C00031->C00097:[7->2,7->3,9->1] | 1.00 | 476.853658537 | 18 | 41 | 0 | 0 |
| Path 93 | C00031->C00097:[7->3,9->1,9->2] | 1.00 | 293.94 | 12 | 50 | 0 | 0 |
| Path 94 | C00031->C00097:[7->3,9->1,9->2] | 1.00 | 465.434782609 | 17 | 46 | 0 | 0 |
| Path 95 | C00031->C00097:[4->2,7->3,9->1] | 1.00 | 391.342105263 | 12 | 38 | 0 | 0 |
| Path 96 | C00031->C00097:[4->2,7->3,9->1] | 1.00 | 381.44 | 18 | 50 | 0 | 1 |
| Path 97 | C00031->C00097:[4->2,7->3,9->1] | 1.00 | 288.516666667 | 15 | 60 | 0 | 1 |
| Path 98 | C00031->C00097:[5->1,5->2,9->3] | 1.00 | 399.065217391 | 15 | 46 | 0 | 0 |
| Path 99 | C00031->C00097:[7->3,9->1,9->2] | 1.00 | 270.520833333 | 10 | 48 | 0 | 0 |
| Path 100 | C00031->C00097:[7->3,9->1,9->2] | 1.00 | 336.138461538 | 20 | 65 | 0 | 0 |
| Path 101 | C00031->C00097:[7->3,9->1,9->2] | 1.00 | 387.621621622 | 14 | 37 | 0 | 0 |
| Path 102 | C00031->C00097:[5->2,7->3,9->1] | 1.00 | 400.090909091 | 13 | 44 | 0 | 0 |
| Path 103 | C00031->C00097:[4->2,7->3,9->1] | 1.00 | 392.675 | 21 | 80 | 0 | 1 |
| Path 104 | C00031->C00097:[7->3,9->1,9->2] | 1.00 | 411.5 | 11 | 32 | 0 | 0 |
| Path 105 | C00031->C00097:[1->1,2->3,4->2,4->3] | 1.00 | 493.351851852 | 22 | 54 | 0 | 0 |
| Path 106 | C00031->C00097:[5->2,5->3,9->1] | 1.00 | 443.134615385 | 19 | 52 | 0 | 0 |
| Path 107 | C00031->C00097:[7->3,9->1,9->2] | 1.00 | 437.647058824 | 13 | 34 | 0 | 0 |
| Path 108 | C00031->C00097:[7->3,9->1,9->2] | 1.00 | 342.370967742 | 16 | 62 | 0 | 0 |
| Path 109 | C00031->C00097:[1->1,2->2,2->3,5->2,5->3,9->1] | 1.00 | 409.957446809 | 16 | 47 | 0 | 0 |
| Path 110 | C00031->C00097:[1->1,2->3,7->2] | 1.00 | 446.56097561 | 15 | 41 | 0 | 0 |
| Path 111 | C00031->C00097:[5->3,7->2,9->1] | 1.00 | 420.357142857 | 17 | 42 | 0 | 0 |
| Path 112 | C00031->C00097:[1->1,2->2,4->3] | 1.00 | 500.9 | 10 | 40 | 0 | 0 |
| Path 113 | C00031->C00097:[1->1,2->2,4->3] | 1.00 | 418.027777778 | 11 | 36 | 0 | 0 |
| Path 114 | C00031->C00097:[7->3,9->1,9->2] | 1.00 | 309.638888889 | 17 | 72 | 0 | 0 |
| Path 115 | C00031->C00097:[4->2,4->3,9->1] | 1.00 | 398.618181818 | 20 | 55 | 0 | 0 |
| Path 116 | C00031->C00097:[5->2,7->3,9->1] | 1.00 | 387.744186047 | 12 | 43 | 0 | 0 |
| Path 117 | C00031->C00097:[5->3,7->2,9->1] | 1.00 | 448.0 | 14 | 40 | 0 | 0 |
| Path 118 | C00031->C00097:[4->2,7->3,9->1] | 1.00 | 480.979166667 | 20 | 48 | 0 | 0 |
| Path 119 | C00031->C00097:[4->2,7->3,9->1] | 1.00 | 478.555555556 | 19 | 45 | 0 | 0 |
| Path 120 | C00031->C00097:[1->1,2->2,4->3] | 1.00 | 495.171428571 | 11 | 35 | 0 | 0 |
| Path 121 | C00031->C00097:[1->1,2->2,4->3] | 1.00 | 473.545454545 | 11 | 33 | 0 | 0 |
| Path 122 | C00031->C00097:[7->3,9->1,9->2] | 1.00 | 433.422222222 | 18 | 45 | 0 | 0 |
| Path 123 | C00031->C00097:[7->3,9->1,9->2] | 1.00 | 427.70212766 | 16 | 47 | 0 | 0 |
| Path 124 | C00031->C00097:[4->2,7->3,9->1] | 1.00 | 386.513157895 | 19 | 76 | 0 | 1 |
| Path 125 | C00031->C00097:[7->3,9->1,9->2] | 1.00 | 302.0 | 15 | 53 | 0 | 0 |
| Path 126 | C00031->C00097:[4->2,7->3,9->1] | 1.00 | 426.457142857 | 12 | 35 | 0 | 0 |
| Path 127 | C00031->C00097:[5->3,9->1] | 0.67 | 452.387096774 | 13 | 31 | 0 | 0 |
| Path 128 | C00031->C00097:[4->2,4->3,7->2,7->3] | 0.67 | 489.722222222 | 19 | 36 | 1 | 1 |
| Path 129 | C00031->C00097:[4->2,4->3,7->2,7->3] | 0.67 | 487.212121212 | 18 | 33 | 1 | 1 |
| Path 130 | C00031->C00097:[7->2,7->3,9->1] | 1.00 | 334.681818182 | 23 | 66 | 0 | 0 |
| Path 131 | C00031->C00097:[4->1,4->2,4->3,7->1,7->2,7->3] | 1.00 | 467.613636364 | 23 | 44 | 1 | 1 |
| Path 132 | C00031->C00097:[4->2,4->3] | 0.67 | 151.119318182 | 23 | 176 | 2 | 3 |
| Path 133 | C00031->C00097:[4->1,4->2] | 0.67 | 383.542857143 | 12 | 35 | 0 | 1 |
| Path 134 | C00031->C00097:[4->1,4->2,7->1,7->2] | 0.67 | 381.88 | 18 | 50 | 0 | 1 |
| Path 135 | C00031->C00097:[4->1,4->2,4->3,7->1,7->2,7->3] | 1.00 | 463.804347826 | 25 | 46 | 1 | 1 |
| Path 136 | C00031->C00097:[4->1,4->2,4->3] | 1.00 | 499.59375 | 19 | 32 | 1 | 1 |
| Path 137 | C00031->C00097:[7->2,7->3,9->1] | 1.00 | 248.614035088 | 13 | 57 | 0 | 1 |
| Path 138 | C00031->C00097:[4->2,7->3,9->1] | 1.00 | 352.87012987 | 26 | 77 | 0 | 0 |
| Path 139 | C00031->C00097:[4->2,7->2,7->3,9->1] | 1.00 | 385.329113924 | 30 | 79 | 0 | 0 |
| Path 140 | C00031->C00097:[4->2,4->3] | 0.67 | 147.751412429 | 22 | 177 | 2 | 3 |
| Path 141 | C00031->C00097:[7->2,7->3,9->1] | 1.00 | 397.913043478 | 20 | 46 | 0 | 0 |
| Path 142 | C00031->C00097:[4->2,7->2,7->3,9->1] | 1.00 | 397.402777778 | 28 | 72 | 0 | 0 |
| Path 143 | C00031->C00097:[4->2,7->2,7->3,9->1] | 1.00 | 431.564516129 | 28 | 62 | 0 | 0 |
| Path 144 | C00031->C00097:[4->1,4->2] | 0.67 | 467.380952381 | 20 | 42 | 0 | 0 |
| Path 145 | C00031->C00097:[4->2,7->3,9->1] | 1.00 | 387.884057971 | 25 | 69 | 0 | 0 |
| Path 146 | C00031->C00097:[4->2,5->3] | 0.67 | 471.404255319 | 19 | 47 | 0 | 0 |
| Path 147 | C00031->C00097:[4->2,7->3,9->1] | 1.00 | 400.760869565 | 17 | 46 | 0 | 1 |
| Path 148 | C00031->C00097:[4->1,4->2,4->3] | 1.00 | 351.031746032 | 25 | 63 | 1 | 1 |
| Path 149 | C00031->C00097:[4->2,7->3,9->1] | 1.00 | 412.075471698 | 22 | 53 | 0 | 0 |
| Path 150 | C00031->C00097:[4->2,7->3,9->1] | 1.00 | 428.218181818 | 24 | 55 | 0 | 0 |
| Path 151 | C00031->C00097:[4->2,7->3,9->1] | 1.00 | 359.444444444 | 29 | 81 | 0 | 0 |
| Path 152 | C00031->C00097:[4->2,7->3,9->1] | 1.00 | 328.838235294 | 22 | 68 | 0 | 0 |
| Path 153 | C00031->C00097:[4->2,4->3,9->1] | 1.00 | 396.981481481 | 19 | 54 | 0 | 0 |
| Path 154 | C00031->C00097:[4->1,4->2,4->3] | 1.00 | 492.75862069 | 18 | 29 | 1 | 1 |
| Path 155 | C00031->C00097:[7->2,7->3,9->1] | 1.00 | 419.11627907 | 19 | 43 | 0 | 0 |
| Path 156 | C00031->C00097:[7->2,7->3,9->1] | 1.00 | 337.642857143 | 24 | 70 | 0 | 0 |
| Path 157 | C00031->C00097:[7->2,7->3,9->1] | 1.00 | 350.479452055 | 27 | 73 | 0 | 0 |
| Path 158 | C00031->C00097:[4->2,5->1,9->3] | 1.00 | 433.8 | 23 | 50 | 0 | 0 |
| Path 159 | C00031->C00097:[4->2,7->3,9->1] | 1.00 | 427.73015873 | 27 | 63 | 0 | 0 |
| Path 160 | C00031->C00097:[4->1,4->2] | 0.67 | 461.083333333 | 14 | 24 | 1 | 1 |
| Path 161 | C00031->C00097:[4->2,4->3,7->2,7->3] | 0.67 | 465.052631579 | 19 | 38 | 1 | 1 |
| Path 162 | C00031->C00097:[4->2,7->3,9->1] | 1.00 | 394.561643836 | 27 | 73 | 0 | 0 |
| Path 163 | C00031->C00097:[4->1,4->2,4->3,7->1,7->2,7->3] | 1.00 | 459.428571429 | 23 | 42 | 1 | 1 |
| Path 164 | C00031->C00097:[4->2,7->3,9->1] | 1.00 | 425.659574468 | 20 | 47 | 0 | 0 |
| Path 165 | C00031->C00097:[4->2,7->3,9->1] | 1.00 | 284.5 | 14 | 60 | 0 | 1 |
| Path 166 | C00031->C00097:[4->2,4->3] | 0.67 | 559.96 | 15 | 25 | 1 | 1 |
| Path 167 | C00031->C00097:[4->2,7->3,9->1] | 1.00 | 429.551724138 | 25 | 58 | 0 | 0 |
| Path 168 | C00031->C00097:[4->1,4->2,4->3] | 1.00 | 168.234693878 | 27 | 196 | 2 | 3 |
| Path 169 | C00031->C00097:[4->2,7->2,7->3,9->1] | 1.00 | 438.737704918 | 28 | 61 | 0 | 0 |
| Path 170 | C00031->C00097:[7->2,7->3,9->1] | 1.00 | 318.390625 | 21 | 64 | 0 | 0 |
| Path 171 | C00031->C00097:[4->2,7->3,9->1] | 1.00 | 424.763636364 | 24 | 55 | 0 | 0 |
| Path 172 | C00031->C00097:[4->2,7->3,9->1] | 1.00 | 372.236842105 | 28 | 76 | 0 | 0 |
| Path 173 | C00031->C00097:[4->2] | 0.33 | 529.380952381 | 12 | 21 | 1 | 1 |
| Path 174 | C00031->C00097:[4->1,4->2] | 0.67 | 402.428571429 | 24 | 63 | 0 | 0 |
| Path 175 | C00031->C00097:[4->1,4->2,4->3] | 1.00 | 361.384615385 | 26 | 65 | 1 | 1 |
| Path 176 | C00031->C00097:[4->1,4->2,4->3] | 1.00 | 351.177419355 | 24 | 62 | 1 | 1 |
| Path 177 | C00031->C00097:[7->3,9->1] | 0.67 | 356.064516129 | 8 | 31 | 0 | 0 |
| Path 178 | C00031->C00097:[5->1,9->3] | 0.67 | 461.375 | 17 | 40 | 0 | 0 |
| Path 179 | C00031->C00097:[7->2] | 0.33 | 398.87804878 | 17 | 41 | 0 | 0 |
| Path 180 | C00031->C00097:[4->1,4->2,4->3] | 1.00 | 161.119791667 | 25 | 192 | 2 | 3 |
| Path 181 | C00031->C00097:[4->2,7->3,9->1] | 1.00 | 430.525423729 | 26 | 59 | 0 | 0 |
| Path 182 | C00031->C00097:[4->2,7->3,9->1] | 1.00 | 473.130434783 | 23 | 46 | 0 | 0 |
| Path 183 | C00031->C00097:[4->2,7->3,9->1] | 1.00 | 475.836734694 | 24 | 49 | 0 | 0 |
| Path 184 | C00031->C00097:[4->1,4->2] | 0.67 | 468.035714286 | 16 | 28 | 1 | 1 |
| Path 185 | C00031->C00097:[4->2,5->3] | 0.67 | 468.272727273 | 18 | 44 | 0 | 0 |
| Path 186 | C00031->C00097:[4->2,5->1,9->3] | 1.00 | 438.528301887 | 24 | 53 | 0 | 0 |
| Path 187 | C00031->C00097:[4->1,4->2,7->1,7->2] | 0.67 | 370.760869565 | 16 | 46 | 0 | 1 |
| Path 188 | C00031->C00097:[4->2,7->3,9->1] | 1.00 | 347.0 | 27 | 76 | 0 | 0 |
| Path 189 | C00031->C00097:[4->1,4->2,7->1,7->2] | 0.67 | 384.928571429 | 26 | 70 | 0 | 0 |
| Path 190 | C00031->C00097:[4->2,7->3,9->1] | 1.00 | 448.454545455 | 26 | 55 | 0 | 0 |
| Path 191 | C00031->C00097:[7->2,7->3,9->1] | 1.00 | 435.84 | 24 | 50 | 0 | 0 |
| Path 192 | C00031->C00097:[5->3,9->1] | 0.67 | 434.351351351 | 11 | 37 | 0 | 0 |
| Path 193 | C00031->C00097:[4->2,7->3,9->1] | 1.00 | 405.76 | 21 | 50 | 0 | 0 |
| Path 194 | C00031->C00097:[4->2,7->3,9->1] | 1.00 | 421.351851852 | 25 | 54 | 0 | 0 |
| Path 195 | C00031->C00097:[4->3] | 0.33 | 585.8 | 10 | 15 | 0 | 0 |
| Path 196 | C00031->C00097:[4->2,7->3,9->1] | 1.00 | 436.258064516 | 28 | 62 | 0 | 0 |
| Path 197 | C00031->C00097:[4->2,7->3,9->1] | 1.00 | 447.854545455 | 24 | 55 | 0 | 0 |
| Path 198 | C00031->C00097:[4->2] | 0.33 | 531.388888889 | 11 | 18 | 1 | 1 |
| Path 199 | C00031->C00097:[4->1,4->2,4->3,7->1,7->2,7->3] | 1.00 | 448.025 | 22 | 40 | 1 | 1 |
| Path 200 | C00031->C00097:[4->2,4->3] | 0.67 | 358.459016393 | 22 | 61 | 1 | 1 |
| Path 201 | C00031->C00097:[4->2,7->3,9->1] | 1.00 | 343.9 | 24 | 70 | 0 | 0 |
| Path 202 | C00031->C00097:[4->2,7->3,9->1] | 1.00 | 351.02739726 | 25 | 73 | 0 | 0 |
| Path 203 | C00031->C00097:[4->2,7->3,9->1] | 1.00 | 398.844444444 | 16 | 45 | 0 | 1 |
| Path 204 | C00031->C00097:[7->2,7->3,9->1] | 1.00 | 352.350649351 | 28 | 77 | 0 | 0 |
| Path 205 | C00031->C00097:[4->2,4->3] | 0.67 | 519.766666667 | 16 | 30 | 1 | 1 |
| Path 206 | C00031->C00097:[4->1,4->2] | 0.67 | 341.910714286 | 16 | 56 | 0 | 1 |
| Path 207 | C00031->C00097:[4->2,7->3,9->1] | 1.00 | 332.041666667 | 23 | 72 | 0 | 0 |
| Path 208 | C00031->C00097:[4->1,4->2,4->3] | 1.00 | 164.855670103 | 26 | 194 | 2 | 3 |
| Path 209 | C00031->C00097:[4->2,7->2,7->3,9->1] | 1.00 | 341.909090909 | 21 | 66 | 0 | 1 |
| Path 210 | C00031->C00097:[4->1,4->2] | 0.67 | 394.105263158 | 13 | 38 | 0 | 1 |
| Path 211 | C00031->C00097:[4->2,7->2,7->3,9->1] | 1.00 | 434.672413793 | 27 | 58 | 0 | 0 |
| Path 212 | C00031->C00097:[4->2,4->3] | 0.67 | 520.037037037 | 15 | 27 | 1 | 1 |
| Path 213 | C00031->C00097:[4->3] | 0.33 | 574.388888889 | 11 | 18 | 0 | 0 |
| Path 214 | C00031->C00097:[4->2,7->3,9->1] | 1.00 | 382.697368421 | 28 | 76 | 0 | 0 |
| Path 215 | C00031->C00097:[4->2,7->3,9->1] | 1.00 | 372.95 | 29 | 80 | 0 | 0 |
| Path 216 | C00031->C00097:[5->1,9->3] | 0.67 | 363.444444444 | 9 | 27 | 0 | 0 |
| Path 217 | C00031->C00097:[4->1,4->2,4->3] | 1.00 | 505.828571429 | 21 | 35 | 1 | 1 |
| Path 218 | C00031->C00097:[4->1,4->2,4->3] | 1.00 | 160.877094972 | 25 | 179 | 2 | 3 |
| Path 219 | C00031->C00097:[4->2,7->3,9->1] | 1.00 | 339.641791045 | 20 | 67 | 0 | 1 |
| Path 220 | C00031->C00097:[5->1,9->3] | 0.67 | 381.259259259 | 9 | 27 | 0 | 0 |
| Path 221 | C00031->C00097:[7->3,9->1] | 0.67 | 381.64 | 7 | 25 | 0 | 0 |
| Path 222 | C00031->C00097:[4->2,7->3,9->1] | 1.00 | 351.706666667 | 27 | 75 | 0 | 0 |
| Path 223 | C00031->C00097:[7->2,7->3,9->1] | 1.00 | 430.75 | 20 | 44 | 0 | 0 |
| Path 224 | C00031->C00097:[4->2,7->3,9->1] | 1.00 | 452.017241379 | 27 | 58 | 0 | 0 |
| Path 225 | C00031->C00097:[4->2] | 0.33 | 452.7 | 18 | 40 | 0 | 0 |
| Path 226 | C00031->C00097:[4->1,4->2] | 0.67 | 421.735294118 | 12 | 34 | 0 | 1 |
| Path 227 | C00031->C00097:[4->2,7->3,9->1] | 1.00 | 346.202702703 | 25 | 74 | 0 | 0 |
| Path 228 | C00031->C00097:[4->2] | 0.33 | 420.076923077 | 8 | 13 | 1 | 1 |
| Path 229 | C00031->C00097:[4->1,4->2,4->3,7->1,7->2,7->3] | 1.00 | 456.23255814 | 23 | 43 | 1 | 1 |
| Path 230 | C00031->C00097:[7->2,7->3,9->1] | 1.00 | 352.930555556 | 26 | 72 | 0 | 0 |
| Path 231 | C00031->C00097:[4->2,7->3,9->1] | 1.00 | 423.076923077 | 23 | 52 | 0 | 0 |
| Path 232 | C00031->C00097:[4->2,4->3] | 0.67 | 565.772727273 | 14 | 22 | 1 | 1 |
| Path 233 | C00031->C00097:[4->1,4->2,4->3] | 1.00 | 157.511111111 | 24 | 180 | 2 | 3 |
| Path 234 | C00031->C00097:[4->2,7->2,7->3,9->1] | 1.00 | 435.523076923 | 29 | 65 | 0 | 0 |
| Path 235 | C00031->C00097:[4->1,4->2,4->3] | 1.00 | 513.454545455 | 19 | 33 | 1 | 1 |
| Path 236 | C00031->C00097:[4->1,4->2] | 0.67 | 329.0 | 14 | 52 | 0 | 1 |
| Path 237 | C00031->C00097:[4->1,4->2,7->1,7->2] | 0.67 | 419.914285714 | 18 | 35 | 1 | 1 |
| Path 238 | C00031->C00097:[4->1,4->2,4->3] | 1.00 | 157.594736842 | 24 | 190 | 2 | 3 |
| Path 239 | C00031->C00097:[4->2,7->3,9->1] | 1.00 | 353.46835443 | 28 | 79 | 0 | 0 |
| Path 240 | C00031->C00097:[4->1,4->2,4->3,7->1,7->2,7->3] | 1.00 | 453.636363636 | 24 | 44 | 1 | 1 |
| Path 241 | C00031->C00097:[4->1,4->2] | 0.67 | 395.152542373 | 22 | 59 | 0 | 0 |
| Path 242 | C00031->C00097:[7->2,7->3,9->1] | 1.00 | 338.722222222 | 26 | 72 | 0 | 0 |
| Path 243 | C00031->C00097:[4->2,7->3,9->1] | 1.00 | 366.273972603 | 27 | 73 | 0 | 0 |
| Path 244 | C00031->C00097:[4->2] | 0.33 | 438.3125 | 9 | 16 | 1 | 1 |
| Path 245 | C00031->C00097:[4->1,4->2] | 0.67 | 470.711111111 | 21 | 45 | 0 | 0 |
| Path 246 | C00031->C00097:[4->2,7->3,9->1] | 1.00 | 364.0125 | 29 | 80 | 0 | 0 |
| Path 247 | C00031->C00097:[4->2,7->3,9->1] | 1.00 | 426.403508772 | 26 | 57 | 0 | 0 |
| Path 248 | C00031->C00097:[4->1,4->2,4->3] | 1.00 | 360.507462687 | 27 | 67 | 1 | 1 |
| Path 249 | C00031->C00097:[4->2,7->3,9->1] | 1.00 | 272.245614035 | 13 | 57 | 0 | 1 |
| Path 250 | C00031->C00097:[4->1,4->2,4->3] | 1.00 | 360.787878788 | 26 | 66 | 1 | 1 |
| Path 251 | C00031->C00097:[4->2,4->3] | 0.67 | 362.0 | 21 | 56 | 1 | 1 |
| Path 252 | C00031->C00097:[4->2,7->3,9->1] | 1.00 | 390.380952381 | 15 | 42 | 0 | 1 |
| Path 253 | C00031->C00097:[7->2,7->3,9->1] | 1.00 | 440.933333333 | 21 | 45 | 0 | 0 |
| Path 254 | C00031->C00097:[4->1,4->2,7->1,7->2] | 0.67 | 427.605263158 | 19 | 38 | 1 | 1 |
| Path 255 | C00031->C00097:[4->2,7->3,9->1] | 1.00 | 448.553571429 | 25 | 56 | 0 | 0 |
| Path 256 | C00031->C00097:[4->2,4->3] | 0.67 | 369.898305085 | 22 | 59 | 1 | 1 |
| Path 257 | C00031->C00097:[4->2,7->3,9->1] | 1.00 | 340.338983051 | 17 | 59 | 0 | 1 |
| Path 258 | C00031->C00097:[4->2,7->3,9->1] | 1.00 | 358.038961039 | 28 | 77 | 0 | 0 |
| Path 259 | C00031->C00097:[4->1,4->2] | 0.67 | 343.145833333 | 13 | 48 | 0 | 1 |
| Path 260 | C00031->C00097:[4->1,4->2,7->1,7->2] | 0.67 | 443.089285714 | 25 | 56 | 0 | 0 |
| Path 261 | C00031->C00097:[4->1,4->2,4->3] | 1.00 | 494.818181818 | 20 | 33 | 1 | 1 |
| Path 262 | C00031->C00097:[4->2,7->3,9->1] | 1.00 | 457.867924528 | 24 | 53 | 0 | 0 |
| Path 263 | C00031->C00097:[4->1,4->2,7->1,7->2] | 0.67 | 399.333333333 | 16 | 45 | 0 | 1 |
| Path 264 | C00031->C00097:[4->2,7->3,9->1] | 1.00 | 432.13559322 | 27 | 59 | 0 | 0 |
| Path 265 | C00031->C00097:[7->3,9->1] | 0.67 | 465.842105263 | 15 | 38 | 0 | 0 |
| Path 266 | C00031->C00097:[5->3] | 0.33 | 453.27027027 | 14 | 37 | 0 | 0 |
| Path 267 | C00031->C00097:[4->2,7->3,9->1] | 1.00 | 454.3 | 23 | 50 | 0 | 0 |
| Path 268 | C00031->C00097:[4->1,4->2] | 0.67 | 423.6 | 13 | 35 | 0 | 1 |
| Path 269 | C00031->C00097:[4->2,7->2,7->3,9->1] | 1.00 | 456.568965517 | 27 | 58 | 0 | 0 |
| Path 270 | C00031->C00097:[7->3,9->1,9->2] | 1.00 | 258.58490566 | 11 | 53 | 0 | 1 |
| Path 271 | C00031->C00097:[5->3,9->1] | 0.67 | 427.857142857 | 13 | 35 | 0 | 0 |
| Path 272 | C00031->C00097:[7->3,9->1] | 0.67 | 233.7 | 9 | 50 | 0 | 1 |
| Path 273 | C00031->C00097:[4->2,4->3] | 0.67 | 518.709677419 | 17 | 31 | 1 | 1 |
| Path 274 | C00031->C00097:[4->1,4->2,7->1,7->2] | 0.67 | 329.19047619 | 18 | 63 | 0 | 1 |
| Path 275 | C00031->C00097:[4->1,4->2,7->1,7->2] | 0.67 | 390.904761905 | 15 | 42 | 0 | 1 |
| Path 276 | C00031->C00097:[4->2,7->3,9->1] | 1.00 | 382.8875 | 29 | 80 | 0 | 0 |
| Path 277 | C00031->C00097:[4->2,7->3,9->1] | 1.00 | 436.1875 | 21 | 48 | 0 | 0 |
| Path 278 | C00031->C00097:[4->2,4->3] | 0.67 | 557.153846154 | 16 | 26 | 1 | 1 |
| Path 279 | C00031->C00097:[5->1,9->3] | 0.67 | 460.024390244 | 18 | 41 | 0 | 0 |
| Path 280 | C00031->C00097:[4->2] | 0.33 | 447.459459459 | 17 | 37 | 0 | 0 |
| Path 281 | C00031->C00097:[4->2,7->2,7->3,9->1] | 1.00 | 453.254545455 | 26 | 55 | 0 | 0 |
| Path 282 | C00031->C00097:[7->2,7->3,9->1] | 1.00 | 431.24 | 23 | 50 | 0 | 0 |
| Path 283 | C00031->C00097:[4->1,4->2,4->3] | 1.00 | 360.793650794 | 24 | 63 | 1 | 1 |
| Path 284 | C00031->C00097:[4->2,7->3,9->1] | 1.00 | 276.671875 | 15 | 64 | 0 | 1 |
| Path 285 | C00031->C00097:[4->2,7->3,9->1] | 1.00 | 422.169491525 | 25 | 59 | 0 | 0 |
| Path 286 | C00031->C00097:[7->2,7->3,9->1] | 1.00 | 322.397058824 | 22 | 68 | 0 | 0 |
| Path 287 | C00031->C00097:[4->1,4->2,4->3] | 1.00 | 165.121827411 | 26 | 197 | 2 | 3 |
| Path 288 | C00031->C00097:[4->2,7->3,9->1] | 1.00 | 367.324675325 | 28 | 77 | 0 | 0 |
| Path 289 | C00031->C00097:[7->2,7->3,9->1] | 1.00 | 335.911764706 | 25 | 68 | 0 | 0 |
| Path 290 | C00031->C00097:[4->2,7->3,9->1] | 1.00 | 443.846153846 | 23 | 52 | 0 | 0 |
| Path 291 | C00031->C00097:[5->1,7->2,9->3] | 1.00 | 428.391304348 | 22 | 46 | 0 | 0 |
| Path 292 | C00031->C00097:[7->3,9->1] | 0.67 | 358.75 | 6 | 24 | 0 | 0 |
| Path 293 | C00031->C00097:[4->1,4->2,4->3] | 1.00 | 152.902857143 | 23 | 175 | 2 | 3 |
| Path 294 | C00031->C00097:[4->1,4->2,4->3,7->1,7->2,7->3] | 1.00 | 450.743589744 | 21 | 39 | 1 | 1 |
| Path 295 | C00031->C00097:[4->2,7->3,9->1] | 1.00 | 336.802816901 | 23 | 71 | 0 | 0 |
| Path 296 | C00031->C00097:[4->1,4->2,4->3,7->1,7->2,7->3] | 1.00 | 463.4 | 21 | 40 | 1 | 1 |
| Path 297 | C00031->C00097:[4->1,4->2,4->3] | 1.00 | 505.322580645 | 19 | 31 | 1 | 1 |
| Path 298 | C00031->C00097:[7->2,7->3,9->1] | 1.00 | 255.358490566 | 12 | 53 | 0 | 1 |
| Path 299 | C00031->C00097:[4->1,4->2] | 0.67 | 355.961538462 | 15 | 52 | 0 | 1 |
| Path 300 | C00031->C00097:[4->3] | 0.33 | 519.05 | 11 | 20 | 0 | 0 |
| Path 301 | C00031->C00097:[1->1,2->3,4->3] | 0.67 | 485.276595745 | 18 | 47 | 0 | 0 |
| Path 302 | C00031->C00097:[7->2,7->3,9->1] | 1.00 | 415.38 | 24 | 50 | 0 | 0 |
| Path 303 | C00031->C00097:[4->1,4->2,4->3] | 1.00 | 513.965517241 | 17 | 29 | 1 | 1 |
| Path 304 | C00031->C00097:[4->2,7->2,7->3,9->1] | 1.00 | 385.266666667 | 29 | 75 | 0 | 0 |
| Path 305 | C00031->C00097:[4->1,4->2,7->1,7->2] | 0.67 | 340.711864407 | 17 | 59 | 0 | 1 |
| Path 306 | C00031->C00097:[4->2,7->3,9->1] | 1.00 | 375.638888889 | 26 | 72 | 0 | 0 |
| Path 307 | C00031->C00097:[4->1,4->2,7->1,7->2] | 0.67 | 438.886792453 | 24 | 53 | 0 | 0 |
| Path 308 | C00031->C00097:[4->2,7->3,9->1] | 1.00 | 431.16 | 21 | 50 | 0 | 0 |
| Path 309 | C00031->C00097:[4->2,7->3,9->1] | 1.00 | 365.083333333 | 30 | 84 | 0 | 0 |
| Path 310 | C00031->C00097:[7->2,7->3,9->1] | 1.00 | 427.490909091 | 26 | 55 | 0 | 0 |
| Path 311 | C00031->C00097:[4->1,4->2] | 0.67 | 412.483870968 | 11 | 31 | 0 | 1 |
| Path 312 | C00031->C00097:[4->2,7->3,9->1] | 1.00 | 440.960784314 | 22 | 51 | 0 | 0 |
| Path 313 | C00031->C00097:[4->2,4->3,7->2,7->3] | 0.67 | 468.87804878 | 20 | 41 | 1 | 1 |
| Path 314 | C00031->C00097:[7->3,9->1,9->2] | 1.00 | 280.309090909 | 13 | 55 | 0 | 1 |
| Path 315 | C00031->C00097:[1->1,2->3] | 0.67 | 433.157894737 | 12 | 38 | 0 | 0 |
| Path 316 | C00031->C00097:[4->1,4->2] | 0.67 | 471.065217391 | 22 | 46 | 0 | 0 |
| Path 317 | C00031->C00097:[4->2,7->3,9->1] | 1.00 | 344.805555556 | 26 | 72 | 0 | 0 |
| Path 318 | C00031->C00097:[4->2,4->3] | 0.67 | 142.779069767 | 21 | 172 | 2 | 3 |
| Path 319 | C00031->C00097:[7->2,7->3,9->1] | 1.00 | 417.0 | 22 | 48 | 0 | 0 |
| Path 320 | C00031->C00097:[4->2,7->3,9->1] | 1.00 | 376.210526316 | 27 | 76 | 0 | 0 |
| Path 321 | C00031->C00097:[4->1,4->2,4->3] | 1.00 | 498.142857143 | 17 | 28 | 1 | 1 |
| Path 322 | C00031->C00097:[4->2,7->3,9->1] | 1.00 | 426.774193548 | 26 | 62 | 0 | 0 |
| Path 323 | C00031->C00097:[7->2,7->3,9->1] | 1.00 | 350.955882353 | 25 | 68 | 0 | 0 |
| Path 324 | C00031->C00097:[4->1,4->2,4->3] | 1.00 | 161.728205128 | 25 | 195 | 2 | 3 |
| Path 325 | C00031->C00097:[4->2,7->3,9->1] | 1.00 | 328.841269841 | 18 | 63 | 0 | 1 |
| Path 326 | C00031->C00097:[4->1,4->2] | 0.67 | 396.487179487 | 14 | 39 | 0 | 1 |
| Path 327 | C00031->C00097:[5->3,7->2] | 0.67 | 465.5 | 17 | 40 | 0 | 0 |
| Path 328 | C00031->C00097:[4->2,7->3,9->1] | 1.00 | 339.453333333 | 24 | 75 | 0 | 0 |
