# Supplementary material for: 13C-metabolic flux ratio and novel carbon path analyses confirmed that Trichoderma reesei uses primarily the respirative pathway also on the preferred carbon source glucose
Source: BMC Syst Biol. 2009 Oct 29;3:104. doi: 10.1186/1752-0509-3-104 (PMC2776023; doi:10.1186/1752-0509-3-104)
Supplement: Additional file 1 — Pathways discovered in ReTrace carbon path analysis. Graphical and tabular representations of amino acid synthesis pathways discovered in ReTrace carbon path analysis [21]. Self-contained web site: unpack zip archive and open index.html with a web browser. [file 1752-0509-3-104-S1.zip › AF1-treesei/pathways-C00031-to-C00049.html]

Pathways from C00031 to C00049


**Pathways from C00031 to C00049**

**Sources:** D-Glucose; (C00031)

**Target:**L-Aspartate; (C00049)

|  | Composite mapping | Z | Average score | Rpairs | Reactions | Zero scores | Scores under threshold |
| --- | --- | --- | --- | --- | --- | --- | --- |
| Path 1 | C00031->C00049:[4->3,4->5,7->2,9->1] | 1.00 | 295.417391304 | 22 | 115 | 0 | 0 |
| Path 2 | C00031->C00049:[1->1,2->3,4->2,9->5] | 1.00 | 313.134615385 | 17 | 52 | 0 | 0 |
| Path 3 | C00031->C00049:[4->3,4->5,7->2,9->1] | 1.00 | 333.970588235 | 22 | 102 | 0 | 0 |
| Path 4 | C00031->C00049:[5->3,7->2,9->1,9->5] | 1.00 | 326.839285714 | 20 | 56 | 0 | 0 |
| Path 5 | C00031->C00049:[1->1,2->3,4->2,9->5] | 1.00 | 336.62 | 19 | 50 | 0 | 0 |
| Path 6 | C00031->C00049:[4->3,4->5,7->2,9->1] | 1.00 | 333.021052632 | 20 | 95 | 0 | 0 |
| Path 7 | C00031->C00049:[4->3,4->5,7->2,9->1] | 1.00 | 390.25 | 14 | 64 | 0 | 0 |
| Path 8 | C00031->C00049:[1->1,2->3,4->2,9->5] | 1.00 | 317.711864407 | 20 | 59 | 0 | 0 |
| Path 9 | C00031->C00049:[1->1,2->3,4->2,5->5] | 1.00 | 305.075471698 | 15 | 53 | 0 | 0 |
| Path 10 | C00031->C00049:[1->1,2->3,4->2,4->5] | 1.00 | 321.875 | 16 | 48 | 0 | 0 |
| Path 11 | C00031->C00049:[5->3,7->2,7->5,9->1] | 1.00 | 331.2 | 15 | 50 | 0 | 0 |
| Path 12 | C00031->C00049:[1->1,2->3,4->2,4->5] | 1.00 | 342.723404255 | 17 | 47 | 0 | 0 |
| Path 13 | C00031->C00049:[7->2,9->1,9->3,9->5] | 1.00 | 320.719512195 | 15 | 82 | 0 | 0 |
| Path 14 | C00031->C00049:[4->3,4->5,7->2,9->1] | 1.00 | 339.255813953 | 16 | 86 | 0 | 0 |
| Path 15 | C00031->C00049:[5->3,5->5,7->2,9->1] | 1.00 | 377.0 | 15 | 75 | 0 | 0 |
| Path 16 | C00031->C00049:[1->1,2->3,4->2,4->5] | 1.00 | 600.518518519 | 25 | 108 | 0 | 0 |
| Path 17 | C00031->C00049:[1->1,2->3,4->2,5->5] | 1.00 | 338.338235294 | 21 | 68 | 0 | 0 |
| Path 18 | C00031->C00049:[1->1,2->3,4->2,9->5] | 1.00 | 349.431372549 | 18 | 51 | 0 | 0 |
| Path 19 | C00031->C00049:[1->1,2->3,4->2,4->5] | 1.00 | 339.833333333 | 14 | 48 | 0 | 0 |
| Path 20 | C00031->C00049:[4->3,4->5,7->2,9->1] | 1.00 | 339.766990291 | 23 | 103 | 0 | 0 |
| Path 21 | C00031->C00049:[5->3,7->2,9->1,9->5] | 1.00 | 269.014925373 | 16 | 67 | 0 | 0 |
| Path 22 | C00031->C00049:[1->1,2->3,4->2,9->5] | 1.00 | 313.557692308 | 18 | 52 | 0 | 0 |
| Path 23 | C00031->C00049:[4->3,4->5,7->2,9->1] | 1.00 | 395.940298507 | 15 | 67 | 0 | 0 |
| Path 24 | C00031->C00049:[4->3,4->5,5->1,9->2] | 1.00 | 565.096153846 | 23 | 104 | 0 | 0 |
| Path 25 | C00031->C00049:[1->1,2->3,4->2,4->5] | 1.00 | 529.893939394 | 19 | 66 | 0 | 0 |
| Path 26 | C00031->C00049:[1->1,2->3,2->5,4->2] | 1.00 | 402.759036145 | 20 | 83 | 0 | 0 |
| Path 27 | C00031->C00049:[7->2,7->3,7->5,9->1] | 1.00 | 373.915254237 | 12 | 59 | 0 | 0 |
| Path 28 | C00031->C00049:[1->1,2->3,4->2,9->5] | 1.00 | 319.215686275 | 15 | 51 | 0 | 0 |
| Path 29 | C00031->C00049:[1->1,2->3,4->2,4->5] | 1.00 | 353.2 | 18 | 50 | 0 | 0 |
| Path 30 | C00031->C00049:[1->1,2->3,4->2,4->5,7->5] | 1.00 | 376.137614679 | 27 | 109 | 0 | 0 |
| Path 31 | C00031->C00049:[5->3,7->2,9->1,9->5] | 1.00 | 320.557692308 | 16 | 52 | 0 | 0 |
| Path 32 | C00031->C00049:[1->1,2->3,4->2,9->5] | 1.00 | 269.75 | 20 | 72 | 0 | 0 |
| Path 33 | C00031->C00049:[7->2,9->1,9->3,9->5] | 1.00 | 387.03125 | 14 | 64 | 0 | 0 |
| Path 34 | C00031->C00049:[1->1,2->3,4->2,9->5] | 1.00 | 275.271428571 | 19 | 70 | 0 | 0 |
| Path 35 | C00031->C00049:[1->1,2->3,4->2,7->5] | 1.00 | 289.627906977 | 15 | 43 | 0 | 0 |
| Path 36 | C00031->C00049:[1->1,4->2,4->3,4->5] | 1.00 | 389.566929134 | 28 | 127 | 0 | 0 |
| Path 37 | C00031->C00049:[7->2,9->1,9->3,9->5] | 1.00 | 307.3375 | 13 | 80 | 0 | 0 |
| Path 38 | C00031->C00049:[1->1,2->3,4->2,4->5] | 1.00 | 393.5 | 18 | 98 | 0 | 0 |
| Path 39 | C00031->C00049:[4->3,4->5,7->2,9->1] | 1.00 | 381.666666667 | 13 | 63 | 0 | 0 |
| Path 40 | C00031->C00049:[1->1,2->3,4->2,7->5] | 1.00 | 532.830508475 | 17 | 59 | 0 | 0 |
| Path 41 | C00031->C00049:[4->3,4->5,7->2,7->3,7->5,9->1] | 1.00 | 341.235294118 | 24 | 102 | 0 | 0 |
| Path 42 | C00031->C00049:[1->1,2->3,4->2,9->5] | 1.00 | 325.709090909 | 19 | 55 | 0 | 0 |
| Path 43 | C00031->C00049:[1->1,2->3,4->2,7->5] | 1.00 | 389.138297872 | 17 | 94 | 0 | 0 |
| Path 44 | C00031->C00049:[1->1,2->3,4->2,7->5] | 1.00 | 311.882352941 | 17 | 51 | 0 | 0 |
| Path 45 | C00031->C00049:[1->1,2->3,4->2,4->5] | 1.00 | 585.572649573 | 24 | 117 | 0 | 0 |
| Path 46 | C00031->C00049:[1->1,2->3,4->2,4->5] | 1.00 | 387.954954955 | 24 | 111 | 0 | 0 |
| Path 47 | C00031->C00049:[4->5,5->3,7->2,9->1] | 1.00 | 335.538461538 | 18 | 52 | 0 | 0 |
| Path 48 | C00031->C00049:[5->3,7->2,9->1,9->5] | 1.00 | 324.771929825 | 17 | 57 | 0 | 0 |
| Path 49 | C00031->C00049:[5->3,7->2,9->1,9->5] | 1.00 | 275.930555556 | 17 | 72 | 0 | 0 |
| Path 50 | C00031->C00049:[1->1,2->3,4->2,7->5] | 1.00 | 848.76119403 | 18 | 67 | 0 | 0 |
| Path 51 | C00031->C00049:[1->1,2->3,4->2,4->5] | 1.00 | 342.547169811 | 15 | 53 | 0 | 0 |
| Path 52 | C00031->C00049:[1->1,2->3,4->2,4->5] | 1.00 | 341.510638298 | 17 | 47 | 0 | 0 |
| Path 53 | C00031->C00049:[5->3,7->2,7->5,9->1] | 1.00 | 310.106382979 | 16 | 47 | 0 | 0 |
| Path 54 | C00031->C00049:[4->5,5->3,7->2,9->1] | 1.00 | 352.561403509 | 17 | 57 | 0 | 0 |
| Path 55 | C00031->C00049:[1->1,2->3,2->5,4->2] | 1.00 | 407.706666667 | 16 | 75 | 0 | 0 |
| Path 56 | C00031->C00049:[1->1,2->3,4->2,5->5] | 1.00 | 310.551724138 | 16 | 58 | 0 | 0 |
| Path 57 | C00031->C00049:[5->3,5->5,7->2,9->1] | 1.00 | 339.072463768 | 22 | 69 | 0 | 0 |
| Path 58 | C00031->C00049:[4->3,4->5,7->2,9->1] | 1.00 | 568.794117647 | 21 | 102 | 0 | 0 |
| Path 59 | C00031->C00049:[4->3,4->5,7->2,7->3,7->5,9->1] | 1.00 | 737.863013699 | 24 | 73 | 0 | 1 |
| Path 60 | C00031->C00049:[4->3,4->5,7->2,9->1] | 1.00 | 551.055555556 | 22 | 108 | 0 | 0 |
| Path 61 | C00031->C00049:[4->3,4->5,7->2,9->1] | 1.00 | 297.283018868 | 18 | 106 | 0 | 0 |
| Path 62 | C00031->C00049:[5->3,5->5,7->2,9->1] | 1.00 | 347.153846154 | 21 | 65 | 0 | 0 |
| Path 63 | C00031->C00049:[1->1,2->3,4->2,5->5] | 1.00 | 334.357142857 | 18 | 56 | 0 | 0 |
| Path 64 | C00031->C00049:[1->1,2->3,4->2,9->5] | 1.00 | 273.073529412 | 18 | 68 | 0 | 0 |
| Path 65 | C00031->C00049:[1->1,2->3,4->2,9->5] | 1.00 | 271.621621622 | 20 | 74 | 0 | 0 |
