# Supplementary material for: 13C-metabolic flux ratio and novel carbon path analyses confirmed that Trichoderma reesei uses primarily the respirative pathway also on the preferred carbon source glucose
Source: BMC Syst Biol. 2009 Oct 29;3:104. doi: 10.1186/1752-0509-3-104 (PMC2776023; doi:10.1186/1752-0509-3-104)
Supplement: Additional file 1 — Pathways discovered in ReTrace carbon path analysis. Graphical and tabular representations of amino acid synthesis pathways discovered in ReTrace carbon path analysis [21]. Self-contained web site: unpack zip archive and open index.html with a web browser. [file 1752-0509-3-104-S1.zip › AF1-treesei/paths.html]

ReTrace results: Trichoderma reesei amino acid biosynthesis


## ReTrace results: Trichoderma reesei amino acid biosynthesis

Sort table by clicking any column header. Back to introduction.

| Source(s) | Target | Pathways | Summary | NumPathways | Zo | BestZo | BestAvgScore | AvgScores | #RPAIRs | #Reactions | MinZeroScores | ZeroScores | MinScoresUnderThr | ScoresUnderThr |
| --- | --- | --- | --- | --- | --- | --- | --- | --- | --- | --- | --- | --- | --- | --- |
| 2-Oxoglutarate | L-Glutamate | html | text | 36 | 1.00 (0.00) | 1.0 | 426.0 | 234.94 (48.71) | 12.64 (3.55) | 207.22 (53.25) | 0 | 0.00 (0.00) | 0 | 0.39 (0.79) |
| Pyruvate | L-Alanine | html | text | 227 | 0.97 (0.12) | 1.0 | 652.0 | 393.71 (97.67) | 16.75 (4.28) | 67.96 (28.60) | 0 | 0.00 (0.00) | 0 | 0.21 (0.41) |
| Oxaloacetate | L-Aspartate | html | text | 121 | 0.82 (0.18) | 1.0 | 757.0 | 376.17 (121.46) | 13.46 (4.39) | 79.55 (54.86) | 0 | 0.00 (0.00) | 0 | 0.38 (0.67) |
| 2-Oxoglutarate | L-Proline | html | text | 119 | 0.91 (0.25) | 1.0 | 673.0 | 278.63 (115.53) | 14.37 (3.57) | 143.87 (80.56) | 0 | 0.00 (0.00) | 0 | 0.10 (0.44) |
| 2-Oxoglutarate | L-Arginine | html | text | 134 | 0.62 (0.34) | 1.0 | 811.0 | 378.23 (157.90) | 15.06 (2.44) | 94.97 (51.68) | 0 | 0.00 (0.00) | 0 | 0.41 (0.49) |
| Pyruvate, Oxaloacetate | L-Isoleucine | html | text | 483 | 0.68 (0.23) | 1.0 | 797.0 | 428.45 (90.87) | 18.72 (4.12) | 49.50 (18.98) | 0 | 0.00 (0.00) | 0 | 0.53 (0.53) |
| Oxaloacetate | L-Threonine | html | text | 97 | 0.74 (0.22) | 1.0 | 768.0 | 486.70 (95.20) | 12.45 (2.89) | 36.07 (15.28) | 0 | 0.00 (0.00) | 0 | 0.68 (0.47) |
| D-Ribose 5-phosphate | ATP | html | text | 233 | 0.69 (0.20) | 0.9 | 940.0 | 414.59 (128.79) | 26.01 (9.76) | 138.18 (89.88) | 0 | 0.00 (0.00) | 0 | 0.40 (0.52) |
| Pyruvate | L-Valine | html | text | 285 | 0.71 (0.26) | 1.0 | 931.0 | 472.86 (110.98) | 17.03 (3.81) | 54.19 (24.75) | 0 | 0.00 (0.00) | 0 | 0.04 (0.20) |
| L-Serine | Glycine | html | text | 260 | 0.98 (0.10) | 1.0 | 1128.0 | 375.40 (145.29) | 16.10 (3.83) | 71.16 (43.26) | 0 | 0.00 (0.00) | 0 | 0.33 (0.65) |
| D-Ribose 5-phosphate | IMP | html | text | 320 | 0.57 (0.30) | 0.9 | 708.0 | 577.56 (62.31) | 25.73 (8.98) | 45.28 (20.16) | 0 | 0.00 (0.00) | 0 | 0.28 (0.45) |
| 3-Phospho-D-glycerate | L-Serine | html | text | 69 | 0.95 (0.16) | 1.0 | 670.0 | 470.68 (69.79) | 15.30 (4.51) | 48.71 (25.78) | 0 | 0.00 (0.00) | 0 | 0.06 (0.29) |
| L-Threonine | Glycine | html | text | 71 | 0.88 (0.21) | 1.0 | 522.0 | 368.83 (67.94) | 12.75 (4.19) | 39.97 (15.48) | 0 | 0.00 (0.00) | 0 | 0.20 (0.40) |
| D-Glucose | L-Alanine | html | text | 212 | 0.94 (0.15) | 1.0 | 526.0 | 307.68 (51.29) | 18.43 (6.50) | 72.10 (16.70) | 0 | 0.00 (0.00) | 0 | 0.65 (0.48) |
| D-Glucose | L-Glutamate | html | text | 872 | 0.74 (0.25) | 1.0 | 612.0 | 363.27 (77.82) | 22.79 (4.97) | 154.24 (56.89) | 0 | 0.00 (0.00) | 0 | 0.36 (0.81) |
| D-Glucose | L-Arginine | html | text | 192 | 0.50 (0.20) | 1.0 | 1635.0 | 355.88 (223.78) | 22.54 (5.81) | 162.79 (94.97) | 0 | 0.69 (0.95) | 0 | 1.03 (1.42) |
| D-Glucose | ATP | html | text | 783 | 0.38 (0.16) | 0.9 | 669.0 | 399.37 (78.98) | 26.24 (7.79) | 128.11 (54.94) | 0 | 0.00 (0.00) | 0 | 0.35 (0.48) |
| D-Glucose | L-Cysteine | html | text | 328 | 0.92 (0.17) | 1.0 | 586.0 | 398.66 (81.98) | 18.45 (5.71) | 54.30 (29.60) | 0 | 0.20 (0.49) | 0 | 0.38 (0.67) |
| D-Glucose | L-Aspartate | html | text | 64 | 1.00 (0.00) | 1.0 | 849.0 | 375.69 (108.71) | 18.38 (3.48) | 72.03 (22.27) | 0 | 0.00 (0.00) | 0 | 0.02 (0.12) |
| D-Glucose | Glycine | html | text | 481 | 0.97 (0.12) | 1.0 | 876.0 | 361.23 (112.34) | 20.80 (5.30) | 81.54 (46.53) | 0 | 0.00 (0.00) | 0 | 0.56 (0.70) |
| D-Erythrose 4-phosphate, Phosphoenolpyruvate | L-Phenylalanine | html | text | 348 | 0.29 (0.12) | 1.0 | 679.0 | 433.52 (43.09) | 19.16 (4.75) | 57.03 (14.03) | 0 | 0.00 (0.00) | 0 | 0.78 (0.62) |
| D-Glucose | L-Lysine | html | text | 46 | 0.71 (0.11) | 1.0 | 450.0 | 397.35 (26.77) | 24.39 (2.43) | 67.04 (13.96) | 0 | 0.00 (0.00) | 0 | 0.00 (0.00) |
| D-Glucose | L-Asparagine | html | text | 20 | 1.00 (0.00) | 1.0 | 575.0 | 355.50 (78.64) | 20.15 (4.15) | 78.95 (25.37) | 0 | 0.00 (0.00) | 0 | 0.10 (0.30) |
| D-Glucose | L-Threonine | html | text | 30 | 0.62 (0.15) | 0.75 | 544.0 | 408.47 (57.96) | 26.37 (4.11) | 49.73 (14.78) | 1 | 1.00 (0.00) | 2 | 2.00 (0.00) |
| Pyruvate, Acetyl-CoA | L-Leucine | html | text | 916 | 0.69 (0.23) | 1.0 | 1259.0 | 356.60 (123.33) | 19.17 (4.22) | 50.49 (25.77) | 0 | 0.00 (0.00) | 0 | 0.78 (0.42) |
| D-Glucose | L-Proline | html | text | 59 | 1.00 (0.00) | 1.0 | 444.0 | 382.90 (29.83) | 25.07 (3.68) | 138.37 (31.70) | 0 | 0.00 (0.00) | 0 | 0.00 (0.00) |
| D-Glucose | L-Serine | html | text | 113 | 0.96 (0.10) | 1.0 | 494.0 | 358.65 (56.13) | 13.78 (3.28) | 46.64 (13.38) | 0 | 0.00 (0.00) | 0 | 0.36 (0.48) |
| D-Glucose | Chorismate | html | text | 9 | 1.00 (0.00) | 1.0 | 602.0 | 577.22 (19.80) | 24.56 (2.17) | 60.78 (8.89) | 0 | 0.00 (0.00) | 0 | 0.11 (0.31) |
| Acetyl-CoA | alpha-D-Glucose 6-phosphate | html | text | 698 | 0.73 (0.22) | 1.0 | 912.0 | 432.06 (89.50) | 19.90 (4.99) | 80.24 (41.96) | 0 | 0.00 (0.00) | 0 | 0.64 (0.48) |
| D-Glucose | L-Valine | html | text | 363 | 0.73 (0.24) | 1.0 | 514.0 | 386.18 (60.95) | 19.58 (4.94) | 58.92 (18.03) | 0 | 0.00 (0.00) | 1 | 1.33 (0.47) |
| D-Glucose | Tryptophan | html | text | 0 | 0.00 (0.00) | 0.0 | 0.0 | 0.00 (0.00) | 0.00 (0.00) | 0.00 (0.00) | 2147483647 | 0.00 (0.00) | 2147483647 | 0.00 (0.00) |
| Acetyl-CoA | D-Glucose | html | text | 428 | 0.72 (0.26) | 1.0 | 618.0 | 405.67 (80.75) | 19.99 (5.06) | 93.72 (54.01) | 0 | 0.00 (0.00) | 0 | 0.59 (0.49) |
| D-Glucose | L-Methionine | html | text | 63 | 1.00 (0.00) | 1.0 | 388.0 | 355.84 (22.75) | 36.37 (3.60) | 113.68 (14.93) | 0 | 0.00 (0.00) | 0 | 0.00 (0.00) |
| D-Glucose | L-Phenylalanine | html | text | 242 | 0.38 (0.29) | 1.0 | 566.0 | 372.70 (93.73) | 22.59 (6.41) | 60.80 (19.52) | 0 | 1.51 (0.77) | 0 | 1.51 (0.77) |
| D-Erythrose 4-phosphate, Phosphoenolpyruvate | L-Tyrosine | html | text | 156 | 0.32 (0.21) | 1.0 | 654.0 | 463.44 (53.63) | 19.62 (5.00) | 51.99 (14.12) | 0 | 0.00 (0.00) | 0 | 0.15 (0.53) |
| D-Glucose | L-Tyrosine | html | text | 8 | 0.18 (0.05) | 0.22 | 381.0 | 299.12 (46.79) | 17.00 (7.31) | 41.62 (19.86) | 0 | 0.62 (0.48) | 0 | 1.38 (0.86) |
| D-Glucose | L-Isoleucine | html | text | 630 | 0.78 (0.23) | 1.0 | 517.0 | 430.41 (55.97) | 28.82 (6.88) | 63.29 (17.11) | 0 | 0.82 (0.38) | 0 | 0.89 (0.49) |
| Acetyl-CoA, 2-Oxoglutarate | L-Lysine | html | text | 347 | 0.51 (0.18) | 0.67 | 834.0 | 350.91 (152.45) | 14.63 (2.99) | 96.16 (49.05) | 0 | 0.00 (0.00) | 0 | 0.00 (0.00) |
| D-Glucose | L-Leucine | html | text | 1038 | 0.63 (0.21) | 1.0 | 538.0 | 328.65 (59.62) | 24.77 (5.14) | 69.51 (19.75) | 0 | 0.00 (0.00) | 1 | 1.58 (0.49) |
| D-Ribose 5-phosphate | L-Histidine | html | text | 21 | 0.82 (0.22) | 1.0 | 774.0 | 439.81 (107.15) | 25.81 (4.93) | 131.29 (45.45) | 0 | 0.00 (0.00) | 0 | 0.00 (0.00) |
| D-Glucose | L-Histidine | html | text | 91 | 0.84 (0.23) | 1.0 | 592.0 | 422.37 (82.93) | 25.80 (5.59) | 109.77 (59.56) | 1 | 1.10 (0.39) | 1 | 1.10 (0.39) |
| L-Aspartate | L-Threonine | html | text | 19 | 0.68 (0.18) | 1.0 | 399.0 | 305.26 (37.89) | 19.95 (4.72) | 56.47 (21.27) | 0 | 0.95 (0.22) | 0 | 1.89 (0.45) |
| 2-Oxoglutarate | N-Acetyl-L-glutamate | html | text | 12 | 0.71 (0.32) | 1.0 | 675.0 | 428.58 (159.74) | 6.33 (1.18) | 53.67 (41.05) | 0 | 0.00 (0.00) | 0 | 0.00 (0.00) |
| N-Acetyl-L-glutamate | L-Arginine | html | text | 0 | 0.00 (0.00) | 0.0 | 0.0 | 0.00 (0.00) | 0.00 (0.00) | 0.00 (0.00) | 2147483647 | 0.00 (0.00) | 2147483647 | 0.00 (0.00) |
