# Supplementary material for: 13C-metabolic flux ratio and novel carbon path analyses confirmed that Trichoderma reesei uses primarily the respirative pathway also on the preferred carbon source glucose
Source: BMC Syst Biol. 2009 Oct 29;3:104. doi: 10.1186/1752-0509-3-104 (PMC2776023; doi:10.1186/1752-0509-3-104)
Supplement: Additional file 1 — Pathways discovered in ReTrace carbon path analysis. Graphical and tabular representations of amino acid synthesis pathways discovered in ReTrace carbon path analysis [21]. Self-contained web site: unpack zip archive and open index.html with a web browser. [file 1752-0509-3-104-S1.zip › AF1-treesei/pathways-C00031-to-C00065.html]

Pathways from C00031 to C00065


**Pathways from C00031 to C00065**

**Sources:** D-Glucose; (C00031)

**Target:**L-Serine; (C00065)

|  | Composite mapping | Z | Average score | Rpairs | Reactions | Zero scores | Scores under threshold |
| --- | --- | --- | --- | --- | --- | --- | --- |
| Path 1 | C00031->C00065:[7->3,9->1,9->2] | 1.00 | 429.352941176 | 13 | 34 | 0 | 0 |
| Path 2 | C00031->C00065:[7->3,9->1,9->2] | 1.00 | 261.258064516 | 14 | 62 | 0 | 0 |
| Path 3 | C00031->C00065:[4->2,7->3,9->1] | 1.00 | 375.115384615 | 19 | 52 | 0 | 1 |
| Path 4 | C00031->C00065:[7->3,9->1,9->2] | 1.00 | 258.033333333 | 13 | 60 | 0 | 0 |
| Path 5 | C00031->C00065:[4->2,7->3,9->1] | 1.00 | 400.805555556 | 12 | 36 | 0 | 0 |
| Path 6 | C00031->C00065:[1->1,2->2,4->3] | 1.00 | 384.285714286 | 9 | 28 | 0 | 0 |
| Path 7 | C00031->C00065:[7->3,9->1,9->2] | 1.00 | 241.433333333 | 12 | 60 | 0 | 0 |
| Path 8 | C00031->C00065:[7->3,9->1,9->2] | 1.00 | 378.25 | 12 | 36 | 0 | 0 |
| Path 9 | C00031->C00065:[4->2,7->3,9->1] | 1.00 | 375.8 | 18 | 50 | 0 | 1 |
| Path 10 | C00031->C00065:[4->2,7->3,9->1] | 1.00 | 422.805555556 | 13 | 36 | 0 | 0 |
| Path 11 | C00031->C00065:[4->2,7->3,9->1] | 1.00 | 406.117647059 | 12 | 34 | 0 | 0 |
| Path 12 | C00031->C00065:[4->2,7->3,9->1] | 1.00 | 275.569230769 | 16 | 65 | 0 | 1 |
| Path 13 | C00031->C00065:[7->3,9->1,9->2] | 1.00 | 259.875 | 13 | 64 | 0 | 0 |
| Path 14 | C00031->C00065:[4->2,7->3,9->1] | 1.00 | 260.213114754 | 14 | 61 | 0 | 1 |
| Path 15 | C00031->C00065:[7->3,9->1,9->2] | 1.00 | 288.3 | 12 | 50 | 0 | 0 |
| Path 16 | C00031->C00065:[4->2,7->2,7->3,9->1] | 1.00 | 378.745098039 | 20 | 51 | 0 | 1 |
| Path 17 | C00031->C00065:[7->3,9->1,9->2] | 1.00 | 414.421052632 | 13 | 38 | 0 | 0 |
| Path 18 | C00031->C00065:[4->2,7->3,9->1] | 1.00 | 392.023255814 | 14 | 43 | 0 | 0 |
| Path 19 | C00031->C00065:[4->2,7->3,9->1] | 1.00 | 389.15 | 21 | 80 | 0 | 1 |
| Path 20 | C00031->C00065:[7->3,9->1,9->2] | 1.00 | 417.555555556 | 12 | 36 | 0 | 0 |
| Path 21 | C00031->C00065:[4->2,7->3,9->1] | 1.00 | 393.682926829 | 13 | 41 | 0 | 0 |
| Path 22 | C00031->C00065:[7->3,9->1,9->2] | 1.00 | 433.571428571 | 14 | 35 | 0 | 0 |
| Path 23 | C00031->C00065:[4->2,7->3,9->1] | 1.00 | 392.290322581 | 10 | 31 | 0 | 0 |
| Path 24 | C00031->C00065:[4->2,7->3,9->1] | 1.00 | 414.212121212 | 12 | 33 | 0 | 0 |
| Path 25 | C00031->C00065:[4->2,7->3,9->1] | 1.00 | 382.625 | 13 | 40 | 0 | 0 |
| Path 26 | C00031->C00065:[7->3,9->1,9->2] | 1.00 | 293.960784314 | 13 | 51 | 0 | 0 |
| Path 27 | C00031->C00065:[7->2,7->3,9->1] | 1.00 | 394.133333333 | 11 | 30 | 0 | 0 |
| Path 28 | C00031->C00065:[7->3,9->1,9->2] | 1.00 | 344.4 | 12 | 40 | 0 | 0 |
| Path 29 | C00031->C00065:[4->1,4->2,7->3,9->1] | 1.00 | 279.578125 | 16 | 64 | 0 | 1 |
| Path 30 | C00031->C00065:[7->2,7->3,9->1] | 1.00 | 384.071428571 | 10 | 28 | 0 | 0 |
| Path 31 | C00031->C00065:[4->2,7->2,7->3,9->1] | 1.00 | 389.41509434 | 21 | 53 | 0 | 1 |
| Path 32 | C00031->C00065:[7->2,7->3,9->1] | 1.00 | 402.931034483 | 11 | 29 | 0 | 0 |
| Path 33 | C00031->C00065:[4->1,4->2,7->3,9->1] | 1.00 | 297.045454545 | 18 | 66 | 0 | 1 |
| Path 34 | C00031->C00065:[7->2,7->3,9->1] | 1.00 | 370.027777778 | 12 | 36 | 0 | 0 |
| Path 35 | C00031->C00065:[7->3,9->1,9->2] | 1.00 | 308.517857143 | 14 | 56 | 0 | 0 |
| Path 36 | C00031->C00065:[7->3,9->1,9->2] | 1.00 | 407.96969697 | 12 | 33 | 0 | 0 |
| Path 37 | C00031->C00065:[4->2,7->3,9->1] | 1.00 | 363.895833333 | 17 | 48 | 0 | 1 |
| Path 38 | C00031->C00065:[5->2,7->3,9->1] | 1.00 | 392.130434783 | 14 | 46 | 0 | 0 |
| Path 39 | C00031->C00065:[4->2,7->3,9->1] | 1.00 | 383.921052632 | 12 | 38 | 0 | 0 |
| Path 40 | C00031->C00065:[5->2,7->3,9->1] | 1.00 | 385.636363636 | 13 | 44 | 0 | 0 |
| Path 41 | C00031->C00065:[4->2,7->2,7->3,9->1] | 1.00 | 391.670886076 | 22 | 79 | 0 | 1 |
| Path 42 | C00031->C00065:[7->3,9->1,9->2] | 1.00 | 281.37254902 | 12 | 51 | 0 | 0 |
| Path 43 | C00031->C00065:[5->2,7->3,9->1] | 1.00 | 393.681818182 | 13 | 44 | 0 | 0 |
| Path 44 | C00031->C00065:[7->3,9->1,9->2] | 1.00 | 400.058823529 | 12 | 34 | 0 | 0 |
| Path 45 | C00031->C00065:[4->2,7->3,9->1] | 1.00 | 382.166666667 | 20 | 78 | 0 | 1 |
| Path 46 | C00031->C00065:[7->3,9->1,9->2] | 1.00 | 271.020408163 | 11 | 49 | 0 | 0 |
| Path 47 | C00031->C00065:[4->2,7->3,9->1] | 1.00 | 409.125 | 11 | 32 | 0 | 0 |
| Path 48 | C00031->C00065:[7->3,9->1,9->2] | 1.00 | 263.262295082 | 14 | 61 | 0 | 0 |
| Path 49 | C00031->C00065:[4->2,7->3,9->1] | 1.00 | 418.4 | 12 | 35 | 0 | 0 |
| Path 50 | C00031->C00065:[4->2,7->2,7->3,9->1] | 1.00 | 388.272727273 | 22 | 55 | 0 | 1 |
| Path 51 | C00031->C00065:[4->2,7->3,9->1] | 1.00 | 388.390243902 | 22 | 82 | 0 | 1 |
| Path 52 | C00031->C00065:[7->2,7->3,9->1] | 1.00 | 370.735294118 | 11 | 34 | 0 | 0 |
| Path 53 | C00031->C00065:[4->2,7->3,9->1] | 1.00 | 415.135135135 | 13 | 37 | 0 | 0 |
| Path 54 | C00031->C00065:[5->2,7->3,9->1] | 1.00 | 397.755555556 | 14 | 45 | 0 | 0 |
| Path 55 | C00031->C00065:[4->2,7->2,7->3,9->1] | 1.00 | 390.839506173 | 23 | 81 | 0 | 1 |
| Path 56 | C00031->C00065:[7->3,9->1,9->2] | 1.00 | 379.441176471 | 11 | 34 | 0 | 0 |
| Path 57 | C00031->C00065:[5->2,7->3,9->1] | 1.00 | 381.186046512 | 12 | 43 | 0 | 0 |
| Path 58 | C00031->C00065:[7->2,7->3,9->1] | 1.00 | 396.714285714 | 10 | 28 | 0 | 0 |
| Path 59 | C00031->C00065:[7->3,9->1,9->2] | 1.00 | 264.645833333 | 10 | 48 | 0 | 0 |
| Path 60 | C00031->C00065:[4->2,7->3,9->1] | 1.00 | 398.0625 | 11 | 32 | 0 | 0 |
| Path 61 | C00031->C00065:[7->3,9->1,9->2] | 1.00 | 356.545454545 | 10 | 33 | 0 | 0 |
| Path 62 | C00031->C00065:[7->3,9->1,9->2] | 1.00 | 284.22 | 12 | 50 | 0 | 0 |
| Path 63 | C00031->C00065:[5->2,7->3,9->1] | 1.00 | 376.38 | 14 | 50 | 0 | 0 |
| Path 64 | C00031->C00065:[1->1,2->2,4->3] | 1.00 | 487.380952381 | 11 | 42 | 0 | 0 |
| Path 65 | C00031->C00065:[7->3,9->1,9->2] | 1.00 | 290.980769231 | 13 | 52 | 0 | 0 |
| Path 66 | C00031->C00065:[4->2,7->3,9->1] | 1.00 | 408.285714286 | 12 | 35 | 0 | 0 |
| Path 67 | C00031->C00065:[4->2,7->2,7->3,9->1] | 1.00 | 379.591836735 | 19 | 49 | 0 | 1 |
| Path 68 | C00031->C00065:[7->3,9->1,9->2] | 1.00 | 268.38 | 11 | 50 | 0 | 0 |
| Path 69 | C00031->C00065:[4->2,7->3,9->1] | 1.00 | 403.323529412 | 11 | 34 | 0 | 0 |
| Path 70 | C00031->C00065:[7->3,9->1,9->2] | 1.00 | 425.388888889 | 14 | 36 | 0 | 0 |
| Path 71 | C00031->C00065:[7->3,9->1,9->2] | 1.00 | 256.709677419 | 12 | 62 | 0 | 0 |
| Path 72 | C00031->C00065:[4->2,7->2,7->3,9->1] | 1.00 | 276.90625 | 17 | 64 | 0 | 1 |
| Path 73 | C00031->C00065:[7->3,9->1,9->2] | 1.00 | 350.147058824 | 10 | 34 | 0 | 0 |
| Path 74 | C00031->C00065:[5->2,7->3,9->1] | 1.00 | 375.673076923 | 15 | 52 | 0 | 0 |
| Path 75 | C00031->C00065:[4->2,7->3,9->1] | 1.00 | 390.212121212 | 11 | 33 | 0 | 0 |
| Path 76 | C00031->C00065:[7->3,9->1,9->2] | 1.00 | 345.047619048 | 13 | 42 | 0 | 0 |
| Path 77 | C00031->C00065:[7->3,9->1,9->2] | 1.00 | 385.085714286 | 12 | 35 | 0 | 0 |
| Path 78 | C00031->C00065:[5->2,7->3,9->1] | 1.00 | 380.155555556 | 13 | 45 | 0 | 0 |
| Path 79 | C00031->C00065:[4->2,7->3,9->1] | 1.00 | 283.816666667 | 15 | 60 | 0 | 1 |
| Path 80 | C00031->C00065:[7->3,9->1,9->2] | 1.00 | 288.240740741 | 12 | 54 | 0 | 0 |
| Path 81 | C00031->C00065:[4->2,7->3,9->1] | 1.00 | 364.152173913 | 16 | 46 | 0 | 1 |
| Path 82 | C00031->C00065:[7->3,9->1,9->2] | 1.00 | 278.244897959 | 11 | 49 | 0 | 0 |
| Path 83 | C00031->C00065:[7->3,9->1,9->2] | 1.00 | 402.6875 | 11 | 32 | 0 | 0 |
| Path 84 | C00031->C00065:[7->2,7->3,9->1] | 1.00 | 375.620689655 | 10 | 29 | 0 | 0 |
| Path 85 | C00031->C00065:[7->2,7->3,9->1] | 1.00 | 376.925925926 | 9 | 27 | 0 | 0 |
| Path 86 | C00031->C00065:[7->3,9->1,9->2] | 1.00 | 349.65625 | 9 | 32 | 0 | 0 |
| Path 87 | C00031->C00065:[7->3,9->1,9->2] | 1.00 | 243.169491525 | 12 | 59 | 0 | 0 |
| Path 88 | C00031->C00065:[7->3,9->1,9->2] | 1.00 | 237.413793103 | 11 | 58 | 0 | 0 |
| Path 89 | C00031->C00065:[4->2,7->3,9->1] | 1.00 | 382.802631579 | 19 | 76 | 0 | 1 |
| Path 90 | C00031->C00065:[1->1,2->2,4->3] | 1.00 | 493.85 | 10 | 40 | 0 | 0 |
| Path 91 | C00031->C00065:[7->3,9->1,9->2] | 1.00 | 306.685185185 | 13 | 54 | 0 | 0 |
| Path 92 | C00031->C00065:[7->3,9->1,9->2] | 1.00 | 285.557692308 | 11 | 52 | 0 | 0 |
| Path 93 | C00031->C00065:[4->1,4->2] | 0.67 | 324.851851852 | 15 | 54 | 0 | 1 |
| Path 94 | C00031->C00065:[4->2,7->3,9->1] | 1.00 | 383.666666667 | 15 | 42 | 0 | 1 |
| Path 95 | C00031->C00065:[4->2,7->3,9->1] | 1.00 | 324.365079365 | 18 | 63 | 0 | 1 |
| Path 96 | C00031->C00065:[4->2,7->3,9->1] | 1.00 | 335.432835821 | 20 | 67 | 0 | 1 |
| Path 97 | C00031->C00065:[4->1,4->2] | 0.67 | 403.387096774 | 11 | 31 | 0 | 1 |
| Path 98 | C00031->C00065:[7->3,9->1,9->2] | 1.00 | 275.181818182 | 13 | 55 | 0 | 1 |
| Path 99 | C00031->C00065:[4->1,4->2] | 0.67 | 337.270833333 | 13 | 48 | 0 | 1 |
| Path 100 | C00031->C00065:[4->2,7->3,9->1] | 1.00 | 394.630434783 | 17 | 46 | 0 | 1 |
| Path 101 | C00031->C00065:[4->1,4->2] | 0.67 | 412.432432432 | 14 | 37 | 0 | 1 |
| Path 102 | C00031->C00065:[4->1,4->2,7->1,7->2] | 0.67 | 364.354166667 | 17 | 48 | 0 | 1 |
| Path 103 | C00031->C00065:[4->1,4->2] | 0.67 | 400.636363636 | 12 | 33 | 0 | 1 |
| Path 104 | C00031->C00065:[4->1,4->2,7->1,7->2] | 0.67 | 364.630434783 | 16 | 46 | 0 | 1 |
| Path 105 | C00031->C00065:[4->1,4->2] | 0.67 | 410.361111111 | 13 | 36 | 0 | 1 |
| Path 106 | C00031->C00065:[4->2,7->3,9->1] | 1.00 | 391.106382979 | 17 | 47 | 0 | 1 |
| Path 107 | C00031->C00065:[4->1,4->2] | 0.67 | 375.485714286 | 12 | 35 | 0 | 1 |
| Path 108 | C00031->C00065:[4->2,7->3,9->1] | 1.00 | 382.5 | 16 | 44 | 0 | 1 |
| Path 109 | C00031->C00065:[4->2,7->2,7->3,9->1] | 1.00 | 338.235294118 | 22 | 68 | 0 | 1 |
| Path 110 | C00031->C00065:[4->2,7->3,9->1] | 1.00 | 272.265625 | 15 | 64 | 0 | 1 |
| Path 111 | C00031->C00065:[4->1,4->2] | 0.67 | 413.441176471 | 12 | 34 | 0 | 1 |
| Path 112 | C00031->C00065:[4->1,4->2,7->1,7->2] | 0.67 | 325.738461538 | 19 | 65 | 0 | 1 |
| Path 113 | C00031->C00065:[4->1,4->2] | 0.67 | 387.731707317 | 15 | 41 | 0 | 1 |
| Path 114 | C00031->C00065:[7->3,9->1] | 0.67 | 347.636363636 | 9 | 33 | 0 | 0 |
