# Supplementary material for: 13C-metabolic flux ratio and novel carbon path analyses confirmed that Trichoderma reesei uses primarily the respirative pathway also on the preferred carbon source glucose
Source: BMC Syst Biol. 2009 Oct 29;3:104. doi: 10.1186/1752-0509-3-104 (PMC2776023; doi:10.1186/1752-0509-3-104)
Supplement: Additional file 1 — Pathways discovered in ReTrace carbon path analysis. Graphical and tabular representations of amino acid synthesis pathways discovered in ReTrace carbon path analysis [21]. Self-contained web site: unpack zip archive and open index.html with a web browser. [file 1752-0509-3-104-S1.zip › AF1-treesei/pathways-C00036-to-C00049.html]

Pathways from C00036 to C00049


**Pathways from C00036 to C00049**

**Sources:** Oxaloacetate; (C00036)

**Target:**L-Aspartate; (C00049)

|  | Composite mapping | Z | Average score | Rpairs | Reactions | Zero scores | Scores under threshold |
| --- | --- | --- | --- | --- | --- | --- | --- |
| Path 1 | C00036->C00049:[2->2,5->1,5->5] | 0.75 | 664.0 | 9 | 19 | 0 | 0 |
| Path 2 | C00036->C00049:[2->2,5->1,5->5] | 0.75 | 495.304347826 | 9 | 23 | 0 | 0 |
| Path 3 | C00036->C00049:[2->2,5->1,5->5] | 0.75 | 354.904761905 | 7 | 21 | 0 | 0 |
| Path 4 | C00036->C00049:[2->2,3->1,3->5,5->1,5->5] | 0.75 | 409.322580645 | 14 | 62 | 0 | 0 |
| Path 5 | C00036->C00049:[2->2,3->1,3->5,5->1,5->5] | 0.75 | 461.628571429 | 10 | 35 | 0 | 0 |
| Path 6 | C00036->C00049:[2->2,5->1,5->5] | 0.75 | 363.14516129 | 12 | 62 | 0 | 0 |
| Path 7 | C00036->C00049:[2->2,3->5,5->1] | 0.75 | 491.238095238 | 9 | 21 | 0 | 0 |
| Path 8 | C00036->C00049:[2->2,5->1,5->5] | 0.75 | 454.686567164 | 18 | 67 | 0 | 0 |
| Path 9 | C00036->C00049:[2->2,3->1,3->5] | 0.75 | 414.61627907 | 14 | 86 | 0 | 0 |
| Path 10 | C00036->C00049:[2->2,5->1,5->5] | 0.75 | 443.079365079 | 15 | 63 | 0 | 0 |
| Path 11 | C00036->C00049:[2->2,5->1,5->5] | 0.75 | 539.620689655 | 12 | 29 | 0 | 0 |
| Path 12 | C00036->C00049:[2->2,5->1,5->5] | 0.75 | 424.383561644 | 14 | 73 | 0 | 0 |
| Path 13 | C00036->C00049:[1->2,2->1,3->5,5->3] | 1.00 | 457.647058824 | 7 | 17 | 0 | 0 |
| Path 14 | C00036->C00049:[5->1,5->2,5->5] | 0.75 | 378.21875 | 16 | 64 | 0 | 0 |
| Path 15 | C00036->C00049:[2->2,2->5,5->1] | 0.75 | 514.64 | 12 | 25 | 0 | 0 |
| Path 16 | C00036->C00049:[2->2,5->1,5->5] | 0.75 | 379.959459459 | 17 | 74 | 0 | 0 |
| Path 17 | C00036->C00049:[1->2,2->1,3->5,5->3] | 1.00 | 483.64516129 | 8 | 31 | 0 | 0 |
| Path 18 | C00036->C00049:[2->2,3->1,3->5] | 0.75 | 380.676470588 | 12 | 68 | 0 | 0 |
| Path 19 | C00036->C00049:[1->1,2->2,3->3,5->5] | 1.00 | 211.875 | 3 | 8 | 0 | 0 |
| Path 20 | C00036->C00049:[1->2,2->1,3->5,5->3] | 1.00 | 271.888888889 | 4 | 9 | 0 | 0 |
| Path 21 | C00036->C00049:[2->2,5->1,5->5] | 0.75 | 485.542857143 | 14 | 35 | 0 | 0 |
| Path 22 | C00036->C00049:[2->2,5->1,5->5] | 0.75 | 664.777777778 | 12 | 27 | 0 | 0 |
| Path 23 | C00036->C00049:[2->2,3->5,5->1] | 0.75 | 457.666666667 | 9 | 45 | 0 | 0 |
| Path 24 | C00036->C00049:[2->2,3->5,5->1] | 0.75 | 431.677419355 | 8 | 31 | 0 | 0 |
| Path 25 | C00036->C00049:[1->1,2->2,3->3,5->5] | 1.00 | 124.888888889 | 1 | 9 | 0 | 0 |
| Path 26 | C00036->C00049:[2->2,5->1,5->5] | 0.75 | 318.185185185 | 9 | 54 | 0 | 0 |
| Path 27 | C00036->C00049:[5->1,5->2,5->5] | 0.75 | 523.32 | 13 | 25 | 0 | 0 |
| Path 28 | C00036->C00049:[2->2,5->1,5->5] | 0.75 | 377.160714286 | 11 | 56 | 0 | 0 |
| Path 29 | C00036->C00049:[1->5,2->1,2->2,5->3] | 1.00 | 572.555555556 | 9 | 18 | 0 | 0 |
| Path 30 | C00036->C00049:[1->2,2->1,3->5,5->3] | 1.00 | 316.523809524 | 12 | 42 | 0 | 1 |
| Path 31 | C00036->C00049:[2->2,5->1,5->5] | 0.75 | 438.152542373 | 13 | 59 | 0 | 0 |
| Path 32 | C00036->C00049:[1->1,2->2,3->3,5->5] | 1.00 | 147.350877193 | 12 | 171 | 0 | 0 |
| Path 33 | C00036->C00049:[2->2,5->1,5->5] | 0.75 | 397.634920635 | 13 | 63 | 0 | 0 |
| Path 34 | C00036->C00049:[2->2,5->1,5->5] | 0.75 | 439.0 | 15 | 87 | 0 | 0 |
| Path 35 | C00036->C00049:[2->2,3->1,3->5,5->1,5->5] | 0.75 | 489.20754717 | 12 | 53 | 0 | 0 |
| Path 36 | C00036->C00049:[2->1,5->2,5->3] | 0.75 | 526.296296296 | 15 | 27 | 0 | 0 |
| Path 37 | C00036->C00049:[2->2,5->1,5->5] | 0.75 | 370.241935484 | 12 | 62 | 0 | 0 |
| Path 38 | C00036->C00049:[1->1,2->2,3->3,5->5] | 1.00 | 350.638297872 | 9 | 94 | 0 | 0 |
| Path 39 | C00036->C00049:[5->3,5->5] | 0.50 | 317.315789474 | 13 | 57 | 0 | 1 |
| Path 40 | C00036->C00049:[1->1,2->2,3->3,5->5] | 1.00 | 169.13 | 16 | 200 | 0 | 2 |
| Path 41 | C00036->C00049:[1->1,2->2,3->3,5->5] | 1.00 | 344.619402985 | 13 | 134 | 0 | 0 |
| Path 42 | C00036->C00049:[2->2,3->1,5->1,5->5] | 0.75 | 440.744680851 | 17 | 94 | 0 | 0 |
| Path 43 | C00036->C00049:[1->1,2->2,3->3,5->5] | 1.00 | 170.970873786 | 17 | 206 | 0 | 2 |
| Path 44 | C00036->C00049:[2->2,5->1,5->5] | 0.75 | 429.818181818 | 17 | 66 | 0 | 0 |
| Path 45 | C00036->C00049:[2->2,5->1,5->5] | 0.75 | 476.93877551 | 11 | 49 | 0 | 0 |
| Path 46 | C00036->C00049:[1->1,2->2,3->3,5->5] | 1.00 | 199.274509804 | 19 | 204 | 0 | 2 |
| Path 47 | C00036->C00049:[1->1,2->2,3->3,5->5] | 1.00 | 177.333333333 | 16 | 165 | 0 | 2 |
| Path 48 | C00036->C00049:[2->2,3->1,5->1,5->5] | 0.75 | 425.838709677 | 16 | 93 | 0 | 0 |
| Path 49 | C00036->C00049:[2->2,5->1,5->5] | 0.75 | 385.017857143 | 11 | 56 | 0 | 0 |
| Path 50 | C00036->C00049:[2->2,5->1,5->5] | 0.75 | 401.166666667 | 13 | 72 | 0 | 0 |
| Path 51 | C00036->C00049:[1->1,2->2,3->3,5->5] | 1.00 | 374.980392157 | 20 | 102 | 0 | 1 |
| Path 52 | C00036->C00049:[1->1,2->2,3->3,5->5] | 1.00 | 161.204545455 | 11 | 176 | 0 | 0 |
| Path 53 | C00036->C00049:[2->2,5->1,5->5] | 0.75 | 445.014285714 | 17 | 70 | 0 | 0 |
| Path 54 | C00036->C00049:[3->1,3->5,5->1,5->5] | 0.50 | 393.448275862 | 13 | 58 | 0 | 0 |
| Path 55 | C00036->C00049:[5->1,5->5] | 0.50 | 356.980769231 | 10 | 52 | 0 | 0 |
| Path 56 | C00036->C00049:[1->1,2->2,3->3,5->5] | 1.00 | 478.575757576 | 19 | 66 | 0 | 1 |
| Path 57 | C00036->C00049:[1->2,2->1,3->5,5->3] | 1.00 | 333.078947368 | 11 | 38 | 0 | 1 |
| Path 58 | C00036->C00049:[1->1,2->2,3->3,5->5] | 1.00 | 317.464285714 | 8 | 56 | 0 | 0 |
| Path 59 | C00036->C00049:[1->1,2->2,3->3,5->5] | 1.00 | 202.116022099 | 18 | 181 | 0 | 2 |
| Path 60 | C00036->C00049:[1->1,2->2,3->3,5->5] | 1.00 | 409.314285714 | 22 | 105 | 0 | 1 |
| Path 61 | C00036->C00049:[1->1,2->2,3->3,5->5] | 1.00 | 190.50462963 | 18 | 216 | 0 | 2 |
| Path 62 | C00036->C00049:[1->1,2->2,3->3,5->5] | 1.00 | 393.564814815 | 23 | 108 | 0 | 1 |
| Path 63 | C00036->C00049:[2->1,5->3] | 0.50 | 756.555555556 | 7 | 9 | 0 | 0 |
| Path 64 | C00036->C00049:[1->1,2->2,3->3,5->5] | 1.00 | 393.096153846 | 21 | 104 | 0 | 1 |
| Path 65 | C00036->C00049:[1->1,2->2,3->3,5->5] | 1.00 | 180.270531401 | 18 | 207 | 0 | 2 |
| Path 66 | C00036->C00049:[2->2,5->1,5->5] | 0.75 | 381.084507042 | 12 | 71 | 0 | 0 |
| Path 67 | C00036->C00049:[1->1,2->2,3->3,5->5] | 1.00 | 188.207920792 | 18 | 202 | 0 | 2 |
| Path 68 | C00036->C00049:[1->1,2->2,3->3,5->5] | 1.00 | 189.931034483 | 18 | 203 | 0 | 2 |
| Path 69 | C00036->C00049:[5->1,5->5] | 0.50 | 669.173913043 | 11 | 23 | 0 | 0 |
| Path 70 | C00036->C00049:[1->1,2->2,3->3,5->5] | 1.00 | 348.666666667 | 9 | 57 | 0 | 0 |
| Path 71 | C00036->C00049:[2->2,3->1,3->5,5->1,5->5] | 0.75 | 403.646341463 | 13 | 82 | 0 | 0 |
| Path 72 | C00036->C00049:[1->1,2->2,3->3,5->5] | 1.00 | 403.234693878 | 12 | 98 | 0 | 0 |
| Path 73 | C00036->C00049:[2->2,5->1,5->5] | 0.75 | 420.21875 | 14 | 64 | 0 | 0 |
| Path 74 | C00036->C00049:[5->3,5->5] | 0.50 | 386.888888889 | 10 | 18 | 0 | 1 |
| Path 75 | C00036->C00049:[2->2,5->1,5->5] | 0.75 | 346.360655738 | 11 | 61 | 0 | 0 |
| Path 76 | C00036->C00049:[2->2,5->1,5->5] | 0.75 | 392.985294118 | 16 | 68 | 0 | 0 |
| Path 77 | C00036->C00049:[2->2,5->1,5->5] | 0.75 | 416.419354839 | 14 | 62 | 0 | 0 |
| Path 78 | C00036->C00049:[1->1,2->2,3->3,5->5] | 1.00 | 187.289473684 | 12 | 190 | 0 | 0 |
| Path 79 | C00036->C00049:[2->2,5->1,5->5] | 0.75 | 403.176470588 | 13 | 85 | 0 | 0 |
| Path 80 | C00036->C00049:[5->3] | 0.25 | 401.692307692 | 6 | 13 | 0 | 0 |
| Path 81 | C00036->C00049:[2->2,5->1,5->5] | 0.75 | 397.888888889 | 13 | 63 | 0 | 0 |
| Path 82 | C00036->C00049:[2->2,5->1,5->5] | 0.75 | 358.8 | 10 | 55 | 0 | 0 |
| Path 83 | C00036->C00049:[5->1,5->5] | 0.50 | 411.784615385 | 15 | 65 | 0 | 0 |
| Path 84 | C00036->C00049:[5->3,5->5] | 0.50 | 427.666666667 | 18 | 42 | 0 | 1 |
| Path 85 | C00036->C00049:[1->1,2->2,3->3,5->5] | 1.00 | 497.819444444 | 22 | 72 | 0 | 1 |
| Path 86 | C00036->C00049:[1->1,2->2,3->3,5->5] | 1.00 | 377.654205607 | 22 | 107 | 0 | 1 |
| Path 87 | C00036->C00049:[1->1,2->2,3->3,5->5] | 1.00 | 391.689320388 | 21 | 103 | 0 | 1 |
| Path 88 | C00036->C00049:[2->2,5->1,5->5] | 0.75 | 408.92 | 18 | 75 | 0 | 0 |
| Path 89 | C00036->C00049:[1->1,2->2,3->3,5->5] | 1.00 | 168.329268293 | 12 | 164 | 0 | 0 |
| Path 90 | C00036->C00049:[2->2,5->1,5->5] | 0.75 | 424.985507246 | 16 | 69 | 0 | 0 |
| Path 91 | C00036->C00049:[2->2,5->1,5->5] | 0.75 | 427.578947368 | 19 | 76 | 0 | 0 |
| Path 92 | C00036->C00049:[1->1,2->2,3->3,5->5] | 1.00 | 321.785714286 | 15 | 70 | 0 | 0 |
| Path 93 | C00036->C00049:[2->2,5->1,5->5] | 0.75 | 408.323076923 | 16 | 65 | 0 | 0 |
| Path 94 | C00036->C00049:[2->2,5->1] | 0.50 | 403.933333333 | 6 | 15 | 0 | 0 |
| Path 95 | C00036->C00049:[1->1,2->2,3->3,5->5] | 1.00 | 349.956521739 | 6 | 23 | 0 | 0 |
| Path 96 | C00036->C00049:[1->1,2->2,3->3,5->5] | 1.00 | 331.45112782 | 12 | 133 | 0 | 0 |
| Path 97 | C00036->C00049:[1->1,2->2,3->3,5->5] | 1.00 | 199.285714286 | 19 | 217 | 0 | 2 |
| Path 98 | C00036->C00049:[1->1,2->2,3->3,5->5] | 1.00 | 285.4375 | 12 | 64 | 0 | 0 |
| Path 99 | C00036->C00049:[1->1,2->2,3->3,5->5] | 1.00 | 144.293413174 | 14 | 167 | 0 | 2 |
| Path 100 | C00036->C00049:[1->1,2->2,3->3,5->5] | 1.00 | 179.263157895 | 17 | 171 | 0 | 2 |
| Path 101 | C00036->C00049:[1->1,2->2,3->3,5->5] | 1.00 | 357.940594059 | 19 | 101 | 0 | 1 |
| Path 102 | C00036->C00049:[5->1,5->5] | 0.50 | 423.509090909 | 12 | 55 | 0 | 0 |
| Path 103 | C00036->C00049:[2->2,3->1,5->1,5->5] | 0.75 | 397.76 | 14 | 75 | 0 | 0 |
| Path 104 | C00036->C00049:[1->1,2->2,3->3,5->5] | 1.00 | 171.693989071 | 16 | 183 | 0 | 2 |
| Path 105 | C00036->C00049:[1->1,2->2,3->3,5->5] | 1.00 | 131.55625 | 9 | 160 | 0 | 0 |
| Path 106 | C00036->C00049:[1->1,2->2,3->3,5->5] | 1.00 | 250.96124031 | 22 | 129 | 0 | 1 |
| Path 107 | C00036->C00049:[2->2,5->1,5->5] | 0.75 | 416.235955056 | 15 | 89 | 0 | 0 |
| Path 108 | C00036->C00049:[1->1,2->2,3->3,5->5] | 1.00 | 314.638297872 | 11 | 47 | 0 | 1 |
| Path 109 | C00036->C00049:[5->1,5->5] | 0.50 | 381.508474576 | 12 | 59 | 0 | 0 |
| Path 110 | C00036->C00049:[2->2,5->1,5->5] | 0.75 | 393.295081967 | 13 | 61 | 0 | 0 |
| Path 111 | C00036->C00049:[5->1,5->5] | 0.50 | 464.947368421 | 8 | 19 | 0 | 0 |
| Path 112 | C00036->C00049:[5->1,5->5] | 0.50 | 344.086206897 | 11 | 58 | 0 | 0 |
| Path 113 | C00036->C00049:[1->1,2->2,3->3,5->5] | 1.00 | 161.668341709 | 12 | 199 | 0 | 0 |
| Path 114 | C00036->C00049:[1->1,2->2,3->3,5->5] | 1.00 | 629.142857143 | 8 | 21 | 0 | 0 |
| Path 115 | C00036->C00049:[5->3,5->5] | 0.50 | 359.074074074 | 21 | 81 | 0 | 1 |
| Path 116 | C00036->C00049:[5->1,5->5] | 0.50 | 523.64 | 11 | 25 | 0 | 0 |
| Path 117 | C00036->C00049:[2->2,5->1,5->5] | 0.75 | 419.73255814 | 14 | 86 | 0 | 0 |
| Path 118 | C00036->C00049:[1->1,2->2,3->3,5->5] | 1.00 | 359.339622642 | 14 | 53 | 0 | 1 |
| Path 119 | C00036->C00049:[5->3,5->5] | 0.50 | 533.02173913 | 21 | 46 | 0 | 1 |
| Path 120 | C00036->C00049:[2->2,3->1,5->1,5->5] | 0.75 | 416.565789474 | 15 | 76 | 0 | 0 |
| Path 121 | C00036->C00049:[1->1,2->2,3->3,5->5] | 1.00 | 256.854961832 | 23 | 131 | 0 | 1 |
