# Supplementary material for: 13C-metabolic flux ratio and novel carbon path analyses confirmed that Trichoderma reesei uses primarily the respirative pathway also on the preferred carbon source glucose
Source: BMC Syst Biol. 2009 Oct 29;3:104. doi: 10.1186/1752-0509-3-104 (PMC2776023; doi:10.1186/1752-0509-3-104)
Supplement: Additional file 1 — Pathways discovered in ReTrace carbon path analysis. Graphical and tabular representations of amino acid synthesis pathways discovered in ReTrace carbon path analysis [21]. Self-contained web site: unpack zip archive and open index.html with a web browser. [file 1752-0509-3-104-S1.zip › AF1-treesei/pathways-C00031-to-C00407.html]

Pathways from C00031 to C00407


**Pathways from C00031 to C00407**

**Sources:** D-Glucose; (C00031)

**Target:**L-Isoleucine; (C00407)

|  | Composite mapping | Z | Average score | Rpairs | Reactions | Zero scores | Scores under threshold |
| --- | --- | --- | --- | --- | --- | --- | --- |
| Path 1 | C00031->C00407:[1->2,1->5,4->6,4->9] | 0.67 | 505.327586207 | 24 | 58 | 0 | 0 |
| Path 2 | C00031->C00407:[7->6,9->2] | 0.33 | 346.293103448 | 21 | 58 | 0 | 0 |
| Path 3 | C00031->C00407:[1->2,4->1,4->3,4->6,7->9,9->5] | 1.00 | 463.12195122 | 34 | 82 | 1 | 1 |
| Path 4 | C00031->C00407:[4->3,4->5,7->6,7->9,9->2] | 0.83 | 367.47761194 | 28 | 67 | 1 | 1 |
| Path 5 | C00031->C00407:[4->3,4->5,7->6,7->9,9->2] | 0.83 | 391.507692308 | 30 | 65 | 1 | 1 |
| Path 6 | C00031->C00407:[4->3,7->6,7->9,9->2,9->5] | 0.83 | 500.782051282 | 35 | 78 | 1 | 1 |
| Path 7 | C00031->C00407:[4->1,4->3,7->6,7->9,9->2,9->5] | 1.00 | 476.351648352 | 40 | 91 | 1 | 1 |
| Path 8 | C00031->C00407:[4->1,4->3,7->6,7->9,9->2,9->5] | 1.00 | 462.278688525 | 35 | 61 | 1 | 1 |
| Path 9 | C00031->C00407:[1->2,4->1,4->3,4->6,7->9,9->5] | 1.00 | 480.772727273 | 39 | 88 | 1 | 1 |
| Path 10 | C00031->C00407:[4->1,4->3,7->6,7->9,9->2,9->5] | 1.00 | 439.957142857 | 39 | 70 | 1 | 1 |
| Path 11 | C00031->C00407:[4->3] | 0.17 | 478.148148148 | 17 | 27 | 1 | 1 |
| Path 12 | C00031->C00407:[1->2,4->6] | 0.33 | 516.95 | 17 | 40 | 0 | 0 |
| Path 13 | C00031->C00407:[1->2,4->1,4->3,4->6,7->9,9->5] | 1.00 | 448.2 | 38 | 85 | 1 | 1 |
| Path 14 | C00031->C00407:[1->2,4->1,4->3,4->6,7->9,9->5] | 1.00 | 436.301204819 | 39 | 83 | 1 | 1 |
| Path 15 | C00031->C00407:[1->2,4->5,4->6,4->9] | 0.67 | 416.158536585 | 27 | 82 | 0 | 0 |
| Path 16 | C00031->C00407:[4->1,4->3,7->6,7->9,9->2,9->5] | 1.00 | 422.714285714 | 30 | 56 | 1 | 1 |
| Path 17 | C00031->C00407:[4->1,4->3,7->6,7->9,9->2,9->5] | 1.00 | 424.2 | 35 | 65 | 1 | 1 |
| Path 18 | C00031->C00407:[4->3,5->6,5->9,9->2,9->5] | 0.83 | 455.310344828 | 28 | 58 | 1 | 1 |
| Path 19 | C00031->C00407:[1->2,4->1,4->3,4->6,7->1,7->3,7->9,9->5] | 1.00 | 473.235955056 | 40 | 89 | 1 | 1 |
| Path 20 | C00031->C00407:[2->3] | 0.17 | 349.426229508 | 17 | 61 | 1 | 2 |
| Path 21 | C00031->C00407:[4->3,5->2,5->5,9->6,9->9] | 0.83 | 429.8125 | 26 | 48 | 1 | 1 |
| Path 22 | C00031->C00407:[4->1,4->3,4->5,7->6,7->9,9->2] | 1.00 | 363.554054054 | 32 | 74 | 1 | 1 |
| Path 23 | C00031->C00407:[7->3,7->9,9->5] | 0.50 | 417.926829268 | 22 | 41 | 1 | 1 |
| Path 24 | C00031->C00407:[1->2,4->6,7->9,9->5] | 0.67 | 451.532258065 | 26 | 62 | 0 | 0 |
| Path 25 | C00031->C00407:[4->1,4->3,7->6,7->9,9->2,9->5] | 1.00 | 433.385964912 | 32 | 57 | 1 | 1 |
| Path 26 | C00031->C00407:[1->2,4->3,4->6,7->9,9->5] | 0.83 | 466.947368421 | 34 | 76 | 1 | 1 |
| Path 27 | C00031->C00407:[1->2,1->5,4->1,4->3,4->6,4->9] | 1.00 | 490.782051282 | 34 | 78 | 1 | 1 |
| Path 28 | C00031->C00407:[7->9,9->3,9->5] | 0.50 | 218.389830508 | 20 | 59 | 1 | 2 |
| Path 29 | C00031->C00407:[4->3,7->6,7->9,9->2,9->5] | 0.83 | 366.0 | 32 | 73 | 1 | 1 |
| Path 30 | C00031->C00407:[1->2,4->6] | 0.33 | 429.933333333 | 17 | 45 | 0 | 0 |
| Path 31 | C00031->C00407:[7->6,7->9,9->2,9->5] | 0.67 | 442.028571429 | 21 | 35 | 0 | 0 |
| Path 32 | C00031->C00407:[1->2,4->1,4->3,4->6,7->9,9->5] | 1.00 | 416.337662338 | 34 | 77 | 1 | 1 |
| Path 33 | C00031->C00407:[4->3,7->6,7->9,9->2,9->5] | 0.83 | 409.239130435 | 24 | 46 | 1 | 1 |
| Path 34 | C00031->C00407:[7->3,7->6,7->9,9->2,9->5] | 0.83 | 436.813953488 | 24 | 43 | 1 | 1 |
| Path 35 | C00031->C00407:[4->3,7->6,9->2] | 0.50 | 495.292682927 | 26 | 41 | 1 | 1 |
| Path 36 | C00031->C00407:[1->2,4->3,4->6] | 0.50 | 504.810344828 | 28 | 58 | 1 | 1 |
| Path 37 | C00031->C00407:[4->3,4->5,7->6,7->9,9->2] | 0.83 | 395.309859155 | 32 | 71 | 1 | 1 |
| Path 38 | C00031->C00407:[7->6,7->9,9->2,9->5] | 0.67 | 403.414634146 | 22 | 41 | 0 | 0 |
| Path 39 | C00031->C00407:[4->1,4->3,7->6,7->9,9->2,9->5] | 1.00 | 433.349206349 | 35 | 63 | 1 | 1 |
| Path 40 | C00031->C00407:[4->3,7->6,7->9,9->2,9->5] | 0.83 | 482.734177215 | 32 | 79 | 1 | 1 |
| Path 41 | C00031->C00407:[5->2,9->6] | 0.33 | 401.0 | 15 | 30 | 0 | 0 |
| Path 42 | C00031->C00407:[4->3,7->9,9->5] | 0.50 | 443.025641026 | 22 | 39 | 1 | 1 |
| Path 43 | C00031->C00407:[4->3,7->6,7->9,9->2,9->5] | 0.83 | 450.092592593 | 29 | 54 | 1 | 1 |
| Path 44 | C00031->C00407:[1->2,4->3,4->6,7->9,9->5] | 0.83 | 462.935897436 | 34 | 78 | 1 | 1 |
| Path 45 | C00031->C00407:[4->1,4->3,7->1,7->3,7->6,7->9,9->2,9->5] | 1.00 | 427.859375 | 36 | 64 | 1 | 1 |
| Path 46 | C00031->C00407:[1->2,4->1,4->3,4->6,7->9,9->5] | 1.00 | 473.563218391 | 38 | 87 | 1 | 1 |
| Path 47 | C00031->C00407:[1->2,4->1,4->3,4->6,7->9,9->5] | 1.00 | 445.160493827 | 36 | 81 | 1 | 1 |
| Path 48 | C00031->C00407:[7->3,7->9,9->5] | 0.50 | 221.018867925 | 19 | 53 | 1 | 2 |
| Path 49 | C00031->C00407:[4->1,4->3,7->6,7->9,9->2,9->5] | 1.00 | 453.634615385 | 31 | 52 | 1 | 1 |
| Path 50 | C00031->C00407:[1->2,4->1,4->3,4->6,7->9,9->5] | 1.00 | 459.811764706 | 35 | 85 | 1 | 1 |
| Path 51 | C00031->C00407:[7->6,7->9,9->2,9->5] | 0.67 | 443.916666667 | 20 | 36 | 0 | 0 |
| Path 52 | C00031->C00407:[1->2,4->1,4->3,4->6,7->9,9->5] | 1.00 | 468.244186047 | 37 | 86 | 1 | 1 |
| Path 53 | C00031->C00407:[1->2,4->3,4->6,7->9,9->5] | 0.83 | 433.905405405 | 32 | 74 | 1 | 1 |
| Path 54 | C00031->C00407:[4->3,7->6,7->9,9->2,9->5] | 0.83 | 386.891891892 | 33 | 74 | 1 | 1 |
| Path 55 | C00031->C00407:[4->3,7->6,7->9,9->2,9->5] | 0.83 | 481.368421053 | 31 | 76 | 1 | 1 |
| Path 56 | C00031->C00407:[7->6,7->9,9->2,9->5] | 0.67 | 420.3 | 21 | 40 | 0 | 0 |
| Path 57 | C00031->C00407:[4->3,7->9,9->5] | 0.50 | 406.325581395 | 23 | 43 | 1 | 1 |
| Path 58 | C00031->C00407:[4->1,4->3,7->6,7->9,9->2,9->5] | 1.00 | 437.655172414 | 32 | 58 | 1 | 1 |
| Path 59 | C00031->C00407:[4->1,4->3,7->6,7->9,9->2,9->5] | 1.00 | 493.5 | 38 | 82 | 1 | 1 |
| Path 60 | C00031->C00407:[4->1,4->3,7->6,7->9,9->2,9->5] | 1.00 | 433.29787234 | 27 | 47 | 1 | 1 |
| Path 61 | C00031->C00407:[4->3,7->6,7->9,9->2,9->5] | 0.83 | 441.734693878 | 27 | 49 | 1 | 1 |
| Path 62 | C00031->C00407:[4->1,4->3,7->6,7->9,9->2,9->5] | 1.00 | 356.701298701 | 33 | 77 | 1 | 1 |
| Path 63 | C00031->C00407:[5->6,5->9,7->3,9->2,9->5] | 0.83 | 452.296296296 | 27 | 54 | 1 | 1 |
| Path 64 | C00031->C00407:[1->2,4->1,4->3,4->6,7->9,9->5] | 1.00 | 459.122222222 | 42 | 90 | 1 | 1 |
| Path 65 | C00031->C00407:[1->2,4->1,4->3,4->6,7->9,9->5] | 1.00 | 470.891566265 | 35 | 83 | 1 | 1 |
| Path 66 | C00031->C00407:[4->1,4->3,7->6,7->9,9->2,9->5] | 1.00 | 459.793650794 | 37 | 63 | 1 | 1 |
| Path 67 | C00031->C00407:[4->1,4->3,7->6,7->9,9->2,9->5] | 1.00 | 403.132075472 | 28 | 53 | 1 | 1 |
| Path 68 | C00031->C00407:[1->2,4->6,7->3,7->9,9->5] | 0.83 | 480.560606061 | 28 | 66 | 1 | 1 |
| Path 69 | C00031->C00407:[1->2,4->3,4->6,7->9,9->5] | 0.83 | 488.090909091 | 32 | 77 | 1 | 1 |
| Path 70 | C00031->C00407:[4->1,4->3,7->1,7->3,7->6,7->9,9->2,9->5] | 1.00 | 451.897058824 | 39 | 68 | 1 | 1 |
| Path 71 | C00031->C00407:[1->2,1->5,4->3,4->6,4->9] | 0.83 | 511.138888889 | 32 | 72 | 1 | 1 |
| Path 72 | C00031->C00407:[4->3,4->5,7->6,7->9,9->2] | 0.83 | 358.939393939 | 27 | 66 | 1 | 1 |
| Path 73 | C00031->C00407:[1->2,4->1,4->3,4->6,7->1,7->3,7->9,9->5] | 1.00 | 469.178571429 | 41 | 84 | 1 | 1 |
| Path 74 | C00031->C00407:[9->3] | 0.17 | 264.03030303 | 13 | 33 | 1 | 2 |
| Path 75 | C00031->C00407:[4->1,4->3,7->6,7->9,9->2,9->5] | 1.00 | 438.393442623 | 34 | 61 | 1 | 1 |
| Path 76 | C00031->C00407:[4->1,4->3,7->6,7->9,9->2,9->5] | 1.00 | 418.087719298 | 32 | 57 | 1 | 1 |
| Path 77 | C00031->C00407:[4->3,7->6,7->9,9->2,9->5] | 0.83 | 439.553191489 | 25 | 47 | 1 | 1 |
| Path 78 | C00031->C00407:[2->3] | 0.17 | 404.646153846 | 16 | 65 | 1 | 2 |
| Path 79 | C00031->C00407:[7->6,7->9,9->2,9->5] | 0.67 | 394.730769231 | 15 | 26 | 0 | 0 |
| Path 80 | C00031->C00407:[4->1,4->3,7->6,7->9,9->2,9->5] | 1.00 | 390.884615385 | 27 | 52 | 1 | 1 |
| Path 81 | C00031->C00407:[4->1,4->3,7->1,7->3,7->6,7->9,9->2,9->5] | 1.00 | 446.442622951 | 35 | 61 | 1 | 1 |
| Path 82 | C00031->C00407:[4->3,7->6,7->9,9->2,9->5] | 0.83 | 437.346938776 | 25 | 49 | 1 | 1 |
| Path 83 | C00031->C00407:[4->1,4->3,4->5,7->6,7->9,9->2] | 1.00 | 360.260273973 | 31 | 73 | 1 | 1 |
| Path 84 | C00031->C00407:[4->3,7->6,7->9,9->2,9->5] | 0.83 | 447.314814815 | 29 | 54 | 1 | 1 |
| Path 85 | C00031->C00407:[4->1,4->3,7->6,7->9,9->2,9->5] | 1.00 | 424.543859649 | 30 | 57 | 1 | 1 |
| Path 86 | C00031->C00407:[7->6,7->9,9->2,9->5] | 0.67 | 401.0 | 21 | 37 | 0 | 0 |
| Path 87 | C00031->C00407:[4->1,4->3,7->6,7->9,9->2,9->5] | 1.00 | 483.675324675 | 34 | 77 | 1 | 1 |
| Path 88 | C00031->C00407:[4->5,7->6,7->9,9->2] | 0.67 | 349.35 | 24 | 60 | 0 | 0 |
| Path 89 | C00031->C00407:[4->2,5->6] | 0.33 | 392.117647059 | 25 | 68 | 0 | 0 |
| Path 90 | C00031->C00407:[1->2,4->3,4->6,7->9,9->5] | 0.83 | 476.432432432 | 32 | 74 | 1 | 1 |
| Path 91 | C00031->C00407:[7->9,9->5] | 0.33 | 391.4 | 14 | 25 | 0 | 0 |
| Path 92 | C00031->C00407:[4->1,4->3,7->6,7->9,9->2,9->5] | 1.00 | 423.965517241 | 32 | 58 | 1 | 1 |
| Path 93 | C00031->C00407:[4->3,7->6,7->9,9->2,9->5] | 0.83 | 487.207792208 | 32 | 77 | 1 | 1 |
| Path 94 | C00031->C00407:[7->6,7->9,9->2,9->5] | 0.67 | 354.125 | 16 | 32 | 0 | 0 |
| Path 95 | C00031->C00407:[2->3,7->9,9->5] | 0.50 | 290.80952381 | 26 | 84 | 1 | 2 |
| Path 96 | C00031->C00407:[1->2,4->1,4->3,4->6,7->9,9->5] | 1.00 | 467.348837209 | 36 | 86 | 1 | 1 |
| Path 97 | C00031->C00407:[2->3,7->9,9->5] | 0.50 | 298.5 | 25 | 88 | 1 | 2 |
| Path 98 | C00031->C00407:[4->2,4->5,7->6,7->9,9->2,9->5] | 0.67 | 369.866666667 | 26 | 60 | 0 | 0 |
| Path 99 | C00031->C00407:[4->3,7->6,7->9,9->2,9->5] | 0.83 | 440.040816327 | 27 | 49 | 1 | 1 |
| Path 100 | C00031->C00407:[1->2,4->1,4->3,4->6,7->9,9->5] | 1.00 | 457.207792208 | 34 | 77 | 1 | 1 |
| Path 101 | C00031->C00407:[4->1,4->3,5->2,5->5,9->6,9->9] | 1.00 | 477.7 | 41 | 90 | 1 | 1 |
| Path 102 | C00031->C00407:[4->1,4->3,7->6,7->9,9->2,9->5] | 1.00 | 413.157894737 | 31 | 57 | 1 | 1 |
| Path 103 | C00031->C00407:[4->3,7->6,7->9,9->2,9->5] | 0.83 | 420.340425532 | 25 | 47 | 1 | 1 |
| Path 104 | C00031->C00407:[1->2,4->1,4->3,4->6,7->9,9->5] | 1.00 | 457.345679012 | 33 | 81 | 1 | 1 |
| Path 105 | C00031->C00407:[4->3,7->6,7->9,9->2,9->5] | 0.83 | 351.492753623 | 28 | 69 | 1 | 1 |
| Path 106 | C00031->C00407:[7->6,7->9,9->2,9->5] | 0.67 | 440.763157895 | 22 | 38 | 0 | 0 |
| Path 107 | C00031->C00407:[1->2,4->3,4->6,7->9,9->5] | 0.83 | 474.704225352 | 31 | 71 | 1 | 1 |
| Path 108 | C00031->C00407:[1->2,4->3,4->5,4->6,4->9] | 0.83 | 424.550561798 | 31 | 89 | 1 | 1 |
| Path 109 | C00031->C00407:[7->6,7->9,9->2,9->5] | 0.67 | 437.688888889 | 21 | 45 | 0 | 0 |
| Path 110 | C00031->C00407:[7->6,7->9,9->2,9->5] | 0.67 | 402.4 | 19 | 35 | 0 | 0 |
| Path 111 | C00031->C00407:[7->9,9->3,9->5] | 0.50 | 242.260416667 | 21 | 96 | 1 | 2 |
| Path 112 | C00031->C00407:[4->1,4->3,7->6,7->9,9->2,9->5] | 1.00 | 359.328767123 | 31 | 73 | 1 | 1 |
| Path 113 | C00031->C00407:[4->1,4->3,7->6,7->9,9->2,9->5] | 1.00 | 464.451219512 | 34 | 82 | 1 | 1 |
| Path 114 | C00031->C00407:[1->2,4->5,4->6,4->9] | 0.67 | 373.265060241 | 29 | 83 | 0 | 0 |
| Path 115 | C00031->C00407:[4->1,4->3,7->6,7->9,9->2,9->5] | 1.00 | 419.6 | 30 | 55 | 1 | 1 |
| Path 116 | C00031->C00407:[4->3,7->6,7->9,9->2,9->5] | 0.83 | 454.941176471 | 29 | 51 | 1 | 1 |
| Path 117 | C00031->C00407:[4->3] | 0.17 | 482.066666667 | 18 | 30 | 1 | 1 |
| Path 118 | C00031->C00407:[7->6,7->9,9->2,9->5] | 0.67 | 351.166666667 | 24 | 60 | 0 | 0 |
| Path 119 | C00031->C00407:[4->3,7->6,7->9,9->2,9->5] | 0.83 | 436.963636364 | 30 | 55 | 1 | 1 |
| Path 120 | C00031->C00407:[4->1,4->3,4->5,7->6,7->9,9->2] | 1.00 | 349.875 | 30 | 72 | 1 | 1 |
| Path 121 | C00031->C00407:[1->2,4->3,4->5,4->6,4->9] | 0.83 | 421.574468085 | 32 | 94 | 1 | 1 |
| Path 122 | C00031->C00407:[1->2,4->3,4->6,7->9,9->5] | 0.83 | 483.594936709 | 32 | 79 | 1 | 1 |
| Path 123 | C00031->C00407:[1->2,4->1,4->3,4->6,7->9,9->5] | 1.00 | 422.935897436 | 35 | 78 | 1 | 1 |
| Path 124 | C00031->C00407:[4->1,4->3,7->6,7->9,9->2,9->5] | 1.00 | 463.411764706 | 31 | 51 | 1 | 1 |
| Path 125 | C00031->C00407:[4->1,4->3,7->6,7->9,9->2,9->5] | 1.00 | 351.386666667 | 31 | 75 | 1 | 1 |
| Path 126 | C00031->C00407:[4->3,7->6,7->9,9->2,9->5] | 0.83 | 358.402777778 | 29 | 72 | 1 | 1 |
| Path 127 | C00031->C00407:[1->2,4->1,4->3,4->6,7->9,9->5] | 1.00 | 474.21686747 | 39 | 83 | 1 | 1 |
| Path 128 | C00031->C00407:[4->3,7->6,9->2] | 0.50 | 493.552631579 | 25 | 38 | 1 | 1 |
| Path 129 | C00031->C00407:[1->2,4->6] | 0.33 | 511.159090909 | 15 | 44 | 0 | 0 |
| Path 130 | C00031->C00407:[4->3,7->6,7->9,9->2,9->5] | 0.83 | 457.882352941 | 29 | 51 | 1 | 1 |
| Path 131 | C00031->C00407:[4->1,4->3,7->6,7->9,9->2,9->5] | 1.00 | 421.049180328 | 33 | 61 | 1 | 1 |
| Path 132 | C00031->C00407:[4->3,7->6,9->2] | 0.50 | 448.2 | 23 | 40 | 1 | 1 |
| Path 133 | C00031->C00407:[4->1,4->3,7->9,9->5] | 0.67 | 393.78 | 25 | 50 | 1 | 1 |
| Path 134 | C00031->C00407:[1->2,4->6,7->3] | 0.50 | 491.285714286 | 26 | 63 | 1 | 1 |
| Path 135 | C00031->C00407:[4->1,4->3,7->6,7->9,9->2,9->5] | 1.00 | 436.01754386 | 32 | 57 | 1 | 1 |
| Path 136 | C00031->C00407:[1->2,4->3,4->6] | 0.50 | 446.96969697 | 29 | 66 | 1 | 1 |
| Path 137 | C00031->C00407:[4->1,4->3,7->6,7->9,9->2,9->5] | 1.00 | 481.431578947 | 43 | 95 | 1 | 1 |
| Path 138 | C00031->C00407:[4->1,4->3,7->1,7->3,7->6,7->9,9->2,9->5] | 1.00 | 443.579710145 | 40 | 69 | 1 | 1 |
| Path 139 | C00031->C00407:[4->1,4->3,4->5,7->6,7->9,9->2] | 1.00 | 419.194174757 | 38 | 103 | 1 | 1 |
| Path 140 | C00031->C00407:[1->2,4->6] | 0.33 | 508.84 | 17 | 50 | 0 | 0 |
| Path 141 | C00031->C00407:[4->1,4->3,7->1,7->3,7->6,7->9,9->2,9->5] | 1.00 | 434.540983607 | 35 | 61 | 1 | 1 |
| Path 142 | C00031->C00407:[1->2,4->3,4->6] | 0.50 | 494.651515152 | 29 | 66 | 1 | 1 |
| Path 143 | C00031->C00407:[1->2,1->5,4->1,4->3,4->6,4->9] | 1.00 | 500.060240964 | 38 | 83 | 1 | 1 |
| Path 144 | C00031->C00407:[1->2,1->5,4->1,4->3,4->6,4->9] | 1.00 | 477.243902439 | 36 | 82 | 1 | 1 |
| Path 145 | C00031->C00407:[4->1,4->3,7->6,7->9,9->2,9->5] | 1.00 | 422.086206897 | 32 | 58 | 1 | 1 |
| Path 146 | C00031->C00407:[1->2,1->5,4->1,4->3,4->6,4->9,5->6,5->9,9->2,9->5] | 1.00 | 467.875 | 38 | 88 | 1 | 1 |
| Path 147 | C00031->C00407:[4->1,4->3,7->1,7->3,7->6,7->9,9->2,9->5] | 1.00 | 451.359375 | 38 | 64 | 1 | 1 |
| Path 148 | C00031->C00407:[4->1,4->3,7->6,7->9,9->2,9->5] | 1.00 | 479.607142857 | 35 | 56 | 1 | 1 |
| Path 149 | C00031->C00407:[1->2,4->1,4->3,4->6,7->1,7->3,7->9,9->5] | 1.00 | 473.858823529 | 41 | 85 | 1 | 1 |
| Path 150 | C00031->C00407:[1->2,4->6,7->9,9->5] | 0.67 | 467.965517241 | 24 | 58 | 0 | 0 |
| Path 151 | C00031->C00407:[7->6,7->9,9->2,9->5] | 0.67 | 486.557692308 | 23 | 52 | 0 | 0 |
| Path 152 | C00031->C00407:[4->1,4->3,7->6,7->9,9->2,9->5] | 1.00 | 432.734375 | 34 | 64 | 1 | 1 |
| Path 153 | C00031->C00407:[7->3,7->6,7->9,9->2,9->5] | 0.83 | 458.272727273 | 28 | 55 | 1 | 1 |
| Path 154 | C00031->C00407:[9->3] | 0.17 | 235.604395604 | 16 | 91 | 1 | 2 |
| Path 155 | C00031->C00407:[7->6,9->2] | 0.33 | 507.7 | 14 | 20 | 0 | 0 |
| Path 156 | C00031->C00407:[1->2,1->5,4->3,4->6,4->9] | 0.83 | 495.5 | 28 | 68 | 1 | 1 |
| Path 157 | C00031->C00407:[4->1,4->3,7->6,7->9,9->2,9->5] | 1.00 | 376.6375 | 36 | 80 | 1 | 1 |
| Path 158 | C00031->C00407:[1->2,4->1,4->3,4->6,7->9,9->5] | 1.00 | 457.493506494 | 35 | 77 | 1 | 1 |
| Path 159 | C00031->C00407:[4->1,4->3,7->6,7->9,9->2,9->5] | 1.00 | 411.807017544 | 30 | 57 | 1 | 1 |
| Path 160 | C00031->C00407:[4->3,4->5] | 0.33 | 363.018518519 | 22 | 54 | 1 | 1 |
| Path 161 | C00031->C00407:[7->9,9->3,9->5] | 0.50 | 278.122807018 | 22 | 57 | 1 | 2 |
| Path 162 | C00031->C00407:[7->9,9->3,9->5] | 0.50 | 225.6 | 21 | 65 | 1 | 2 |
| Path 163 | C00031->C00407:[9->3] | 0.17 | 204.508474576 | 15 | 59 | 1 | 2 |
| Path 164 | C00031->C00407:[4->1,4->3,7->6,7->9,9->2,9->5] | 1.00 | 364.493506494 | 33 | 77 | 1 | 1 |
| Path 165 | C00031->C00407:[4->2,4->3,4->5,5->6,5->9] | 0.83 | 389.567901235 | 32 | 81 | 1 | 1 |
| Path 166 | C00031->C00407:[4->1,4->3,7->6,7->9,9->2,9->5] | 1.00 | 435.616666667 | 34 | 60 | 1 | 1 |
| Path 167 | C00031->C00407:[2->3,7->9,9->5] | 0.50 | 339.72826087 | 24 | 92 | 1 | 2 |
| Path 168 | C00031->C00407:[1->2,4->1,4->3,4->6,7->1,7->3,7->9,9->5] | 1.00 | 470.611764706 | 37 | 85 | 1 | 1 |
| Path 169 | C00031->C00407:[4->3,7->6,7->9,9->2,9->5] | 0.83 | 411.875 | 26 | 48 | 1 | 1 |
| Path 170 | C00031->C00407:[7->6,9->2] | 0.33 | 399.80952381 | 11 | 21 | 0 | 0 |
| Path 171 | C00031->C00407:[4->1,4->3,7->6,7->9,9->2,9->5] | 1.00 | 446.38961039 | 38 | 77 | 1 | 1 |
| Path 172 | C00031->C00407:[1->2,1->5,4->6,4->9] | 0.67 | 486.129032258 | 25 | 62 | 0 | 0 |
| Path 173 | C00031->C00407:[4->3,7->6,7->9,9->2,9->5] | 0.83 | 444.22 | 26 | 50 | 1 | 1 |
| Path 174 | C00031->C00407:[7->6,7->9,9->2,9->5] | 0.67 | 398.96969697 | 17 | 33 | 0 | 0 |
| Path 175 | C00031->C00407:[1->2,4->6,7->9,9->5] | 0.67 | 404.631578947 | 23 | 57 | 0 | 0 |
| Path 176 | C00031->C00407:[1->2,4->3,4->6,7->9,9->5] | 0.83 | 481.442857143 | 29 | 70 | 1 | 1 |
| Path 177 | C00031->C00407:[1->2,4->3,4->6] | 0.50 | 487.580645161 | 28 | 62 | 1 | 1 |
| Path 178 | C00031->C00407:[4->1,4->3,7->6,7->9,9->2,9->5] | 1.00 | 438.116666667 | 34 | 60 | 1 | 1 |
| Path 179 | C00031->C00407:[4->1,4->3,7->1,7->3,7->6,7->9,9->2,9->5] | 1.00 | 426.931034483 | 33 | 58 | 1 | 1 |
| Path 180 | C00031->C00407:[4->3,7->6,7->9,9->2,9->5] | 0.83 | 391.506024096 | 34 | 83 | 1 | 1 |
| Path 181 | C00031->C00407:[1->2,4->1,4->3,4->6,7->9,9->5] | 1.00 | 469.25974026 | 33 | 77 | 1 | 1 |
| Path 182 | C00031->C00407:[4->1,4->3,7->6,7->9,9->2,9->5] | 1.00 | 441.384615385 | 32 | 65 | 1 | 1 |
| Path 183 | C00031->C00407:[5->3] | 0.17 | 404.409090909 | 17 | 66 | 1 | 2 |
| Path 184 | C00031->C00407:[7->6,9->2] | 0.33 | 383.185185185 | 12 | 27 | 0 | 0 |
| Path 185 | C00031->C00407:[7->6,7->9,9->2,9->5] | 0.67 | 441.022222222 | 21 | 45 | 0 | 0 |
| Path 186 | C00031->C00407:[4->3,7->6,7->9,9->2,9->5] | 0.83 | 451.0 | 30 | 57 | 1 | 1 |
| Path 187 | C00031->C00407:[7->3,7->6,9->2] | 0.50 | 442.888888889 | 22 | 36 | 1 | 1 |
| Path 188 | C00031->C00407:[1->2,4->6,7->3] | 0.50 | 501.862068966 | 25 | 58 | 1 | 1 |
| Path 189 | C00031->C00407:[4->1,4->3,5->2,5->5,9->6,9->9] | 1.00 | 467.870588235 | 37 | 85 | 1 | 1 |
| Path 190 | C00031->C00407:[4->3,7->6,7->9,9->2,9->5] | 0.83 | 516.621621622 | 34 | 74 | 1 | 1 |
| Path 191 | C00031->C00407:[4->3,7->6,7->9,9->2,9->5] | 0.83 | 500.915492958 | 31 | 71 | 1 | 1 |
| Path 192 | C00031->C00407:[4->3,7->6,7->9,9->2,9->5] | 0.83 | 422.659574468 | 25 | 47 | 1 | 1 |
| Path 193 | C00031->C00407:[1->2,1->5,4->1,4->3,4->6,4->9] | 1.00 | 484.04 | 32 | 75 | 1 | 1 |
| Path 194 | C00031->C00407:[1->2,1->5,4->3,4->6,4->9] | 0.83 | 495.171052632 | 33 | 76 | 1 | 1 |
| Path 195 | C00031->C00407:[1->2,4->1,4->3,4->6,7->1,7->3,7->9,9->5] | 1.00 | 469.0 | 35 | 79 | 1 | 1 |
| Path 196 | C00031->C00407:[4->1,4->3,7->6,7->9,9->2,9->5] | 1.00 | 444.746268657 | 38 | 67 | 1 | 1 |
| Path 197 | C00031->C00407:[4->1,4->3,7->6,7->9,9->2,9->5] | 1.00 | 436.370967742 | 36 | 62 | 1 | 1 |
| Path 198 | C00031->C00407:[1->2,4->3,4->6,7->9,9->5] | 0.83 | 481.041666667 | 32 | 72 | 1 | 1 |
| Path 199 | C00031->C00407:[4->3,7->6,7->9,9->2,9->5] | 0.83 | 443.9 | 23 | 40 | 1 | 1 |
| Path 200 | C00031->C00407:[4->3,7->6,7->9,9->2,9->5] | 0.83 | 463.372881356 | 29 | 59 | 1 | 1 |
| Path 201 | C00031->C00407:[4->3,7->6,7->9,9->2,9->5] | 0.83 | 460.830508475 | 29 | 59 | 1 | 1 |
| Path 202 | C00031->C00407:[1->2,4->1,4->3,4->6,7->9,9->5] | 1.00 | 456.76744186 | 40 | 86 | 1 | 1 |
| Path 203 | C00031->C00407:[4->1,4->3,7->6,7->9,9->2,9->5] | 1.00 | 429.5 | 34 | 62 | 1 | 1 |
| Path 204 | C00031->C00407:[1->2,1->5,4->3,4->6,4->9] | 0.83 | 484.514285714 | 29 | 70 | 1 | 1 |
| Path 205 | C00031->C00407:[4->1,4->3,4->5,7->6,7->9,9->2] | 1.00 | 378.387755102 | 38 | 98 | 1 | 1 |
| Path 206 | C00031->C00407:[1->2,4->1,4->3,4->6,7->9,9->5] | 1.00 | 470.088888889 | 39 | 90 | 1 | 1 |
| Path 207 | C00031->C00407:[7->9,9->3,9->5] | 0.50 | 224.245283019 | 18 | 53 | 1 | 2 |
| Path 208 | C00031->C00407:[4->3,7->6,7->9,9->2,9->5] | 0.83 | 473.553191489 | 27 | 47 | 1 | 1 |
| Path 209 | C00031->C00407:[4->3,7->6,7->9,9->2,9->5] | 0.83 | 480.671428571 | 33 | 70 | 1 | 1 |
| Path 210 | C00031->C00407:[1->2,4->3,4->6,7->9,9->5] | 0.83 | 487.774647887 | 30 | 71 | 1 | 1 |
| Path 211 | C00031->C00407:[1->2,4->3,4->5,4->6,4->9] | 0.83 | 414.865168539 | 33 | 89 | 1 | 1 |
| Path 212 | C00031->C00407:[1->2,4->1,4->3,4->6,7->1,7->3,7->9,9->5] | 1.00 | 423.8375 | 37 | 80 | 1 | 1 |
| Path 213 | C00031->C00407:[9->3] | 0.17 | 224.469387755 | 14 | 49 | 1 | 2 |
| Path 214 | C00031->C00407:[4->3,7->6,7->9,9->2,9->5] | 0.83 | 463.523809524 | 25 | 42 | 1 | 1 |
| Path 215 | C00031->C00407:[1->2,4->1,4->3,4->6,7->9,9->5] | 1.00 | 475.597701149 | 38 | 87 | 1 | 1 |
| Path 216 | C00031->C00407:[7->3,7->6,7->9,9->2,9->5] | 0.83 | 454.466666667 | 26 | 45 | 1 | 1 |
| Path 217 | C00031->C00407:[4->3,7->6,7->9,9->2,9->5] | 0.83 | 469.358490566 | 29 | 53 | 1 | 1 |
| Path 218 | C00031->C00407:[1->2,4->3,4->6,7->9,9->5] | 0.83 | 475.58974359 | 31 | 78 | 1 | 1 |
| Path 219 | C00031->C00407:[9->3] | 0.17 | 191.982758621 | 14 | 58 | 1 | 2 |
| Path 220 | C00031->C00407:[4->3,7->9,9->5] | 0.50 | 448.333333333 | 23 | 42 | 1 | 1 |
| Path 221 | C00031->C00407:[4->3,7->6,7->9,9->2,9->5] | 0.83 | 437.843137255 | 29 | 51 | 1 | 1 |
| Path 222 | C00031->C00407:[4->2,4->3,4->5,7->6,7->9] | 0.83 | 363.196969697 | 27 | 66 | 1 | 1 |
| Path 223 | C00031->C00407:[7->6,7->9,9->2,9->5] | 0.67 | 383.647058824 | 18 | 34 | 0 | 0 |
| Path 224 | C00031->C00407:[7->3,7->6,7->9,9->2,9->5] | 0.83 | 431.733333333 | 26 | 45 | 1 | 1 |
| Path 225 | C00031->C00407:[1->2,4->1,4->3,4->6,7->9,9->5] | 1.00 | 468.646341463 | 38 | 82 | 1 | 1 |
| Path 226 | C00031->C00407:[4->1,4->3,7->6,7->9,9->2,9->5] | 1.00 | 472.355555556 | 39 | 90 | 1 | 1 |
| Path 227 | C00031->C00407:[4->1,4->3,7->6,7->9,9->2,9->5] | 1.00 | 493.461538462 | 42 | 91 | 1 | 1 |
| Path 228 | C00031->C00407:[4->1,4->3,7->6,7->9,9->2,9->5] | 1.00 | 445.982142857 | 31 | 56 | 1 | 1 |
| Path 229 | C00031->C00407:[7->6,7->9,9->2,9->5] | 0.67 | 404.342857143 | 19 | 35 | 0 | 0 |
| Path 230 | C00031->C00407:[1->2,4->1,4->3,4->6,7->9,9->5] | 1.00 | 459.802469136 | 36 | 81 | 1 | 1 |
| Path 231 | C00031->C00407:[4->3,4->5,7->9] | 0.50 | 364.176470588 | 27 | 68 | 1 | 1 |
| Path 232 | C00031->C00407:[4->1,4->3,7->6,7->9,9->2,9->5] | 1.00 | 400.941176471 | 26 | 51 | 1 | 1 |
| Path 233 | C00031->C00407:[4->1,4->3,7->6,7->9,9->2,9->5] | 1.00 | 401.075471698 | 28 | 53 | 1 | 1 |
| Path 234 | C00031->C00407:[7->6,7->9,9->2,9->5] | 0.67 | 371.606060606 | 17 | 33 | 0 | 0 |
| Path 235 | C00031->C00407:[7->6,7->9,9->2,9->5] | 0.67 | 492.49122807 | 23 | 57 | 0 | 0 |
| Path 236 | C00031->C00407:[1->2,4->6,7->9,9->5] | 0.67 | 443.672131148 | 25 | 61 | 0 | 0 |
| Path 237 | C00031->C00407:[1->2,4->1,4->3,4->6,7->1,7->3,7->9,9->5] | 1.00 | 480.288888889 | 41 | 90 | 1 | 1 |
| Path 238 | C00031->C00407:[4->1,4->3,7->6,7->9,9->2,9->5] | 1.00 | 408.394230769 | 37 | 104 | 1 | 1 |
| Path 239 | C00031->C00407:[1->2,4->1,4->3,4->6,7->1,7->3,7->9,9->5] | 1.00 | 451.273809524 | 39 | 84 | 1 | 1 |
| Path 240 | C00031->C00407:[4->3,4->5,7->9] | 0.50 | 357.107692308 | 26 | 65 | 1 | 1 |
| Path 241 | C00031->C00407:[4->1,4->3,7->6,7->9,9->2,9->5] | 1.00 | 408.854545455 | 28 | 55 | 1 | 1 |
| Path 242 | C00031->C00407:[7->9,9->5] | 0.33 | 375.032258065 | 15 | 31 | 0 | 0 |
| Path 243 | C00031->C00407:[4->3,4->5,7->6,7->9,9->2] | 0.83 | 421.195652174 | 36 | 92 | 1 | 1 |
| Path 244 | C00031->C00407:[9->3] | 0.17 | 320.34375 | 14 | 32 | 1 | 2 |
| Path 245 | C00031->C00407:[1->2,4->6,7->3] | 0.50 | 493.406779661 | 27 | 59 | 1 | 1 |
| Path 246 | C00031->C00407:[7->3,7->6,7->9,9->2,9->5] | 0.83 | 427.145833333 | 26 | 48 | 1 | 1 |
| Path 247 | C00031->C00407:[1->2,4->6,7->9,9->5] | 0.67 | 460.228070175 | 24 | 57 | 0 | 0 |
| Path 248 | C00031->C00407:[7->3,7->6,7->9,9->2,9->5] | 0.83 | 400.976190476 | 23 | 42 | 1 | 1 |
| Path 249 | C00031->C00407:[1->2,4->6,7->3] | 0.50 | 468.983870968 | 28 | 62 | 1 | 1 |
| Path 250 | C00031->C00407:[1->2,4->1,4->3,4->6,7->1,7->3,7->9,9->5] | 1.00 | 458.063291139 | 37 | 79 | 1 | 1 |
| Path 251 | C00031->C00407:[4->1,4->3,7->1,7->3,7->6,7->9,9->2,9->5] | 1.00 | 449.102941176 | 35 | 68 | 1 | 1 |
| Path 252 | C00031->C00407:[7->6,7->9,9->2,9->5] | 0.67 | 428.621621622 | 21 | 37 | 0 | 0 |
| Path 253 | C00031->C00407:[4->1,4->3,7->1,7->3,7->6,7->9,9->2,9->5] | 1.00 | 403.181818182 | 30 | 55 | 1 | 1 |
| Path 254 | C00031->C00407:[4->1,4->3,7->6,7->9,9->2,9->5] | 1.00 | 442.306451613 | 34 | 62 | 1 | 1 |
| Path 255 | C00031->C00407:[4->1,4->3] | 0.33 | 444.594594595 | 22 | 37 | 1 | 1 |
| Path 256 | C00031->C00407:[4->1,4->3,7->6,7->9,9->2,9->5] | 1.00 | 462.892307692 | 37 | 65 | 1 | 1 |
| Path 257 | C00031->C00407:[2->3,7->9,9->5] | 0.50 | 294.277108434 | 24 | 83 | 1 | 2 |
| Path 258 | C00031->C00407:[7->3,7->6,7->9,9->2,9->5] | 0.83 | 415.837209302 | 24 | 43 | 1 | 1 |
| Path 259 | C00031->C00407:[5->2,5->5,7->3,9->6,9->9] | 0.83 | 423.795454545 | 25 | 44 | 1 | 1 |
| Path 260 | C00031->C00407:[4->3,4->5,7->6,7->9,9->2] | 0.83 | 385.306666667 | 33 | 75 | 1 | 1 |
| Path 261 | C00031->C00407:[4->1,4->3,7->6,7->9,9->2,9->5] | 1.00 | 466.558139535 | 36 | 86 | 1 | 1 |
| Path 262 | C00031->C00407:[4->2,4->3,4->5] | 0.50 | 379.677966102 | 25 | 59 | 1 | 1 |
| Path 263 | C00031->C00407:[4->2,4->3,4->5,5->6,5->9] | 0.83 | 397.873417722 | 32 | 79 | 1 | 1 |
| Path 264 | C00031->C00407:[4->1,4->3,7->9,9->5] | 0.67 | 402.37037037 | 27 | 54 | 1 | 1 |
| Path 265 | C00031->C00407:[7->6,7->9,9->2,9->5] | 0.67 | 476.53968254 | 24 | 63 | 0 | 0 |
| Path 266 | C00031->C00407:[4->1,4->3,7->6,7->9,9->2,9->5] | 1.00 | 420.836363636 | 30 | 55 | 1 | 1 |
| Path 267 | C00031->C00407:[4->1,4->3,7->1,7->3,7->6,7->9,9->2,9->5] | 1.00 | 434.966101695 | 34 | 59 | 1 | 1 |
| Path 268 | C00031->C00407:[4->1,4->3,7->6,7->9,9->2,9->5] | 1.00 | 473.728395062 | 40 | 81 | 1 | 1 |
| Path 269 | C00031->C00407:[4->3,7->6,7->9,9->2,9->5] | 0.83 | 428.34 | 26 | 50 | 1 | 1 |
| Path 270 | C00031->C00407:[4->5,7->6,7->9,9->2] | 0.67 | 366.758064516 | 26 | 62 | 0 | 0 |
| Path 271 | C00031->C00407:[4->3,7->6,7->9,9->2,9->5] | 0.83 | 503.408450704 | 31 | 71 | 1 | 1 |
| Path 272 | C00031->C00407:[7->6,7->9,9->2,9->5] | 0.67 | 334.983606557 | 23 | 61 | 0 | 0 |
| Path 273 | C00031->C00407:[7->6,7->9,9->2,9->5] | 0.67 | 418.571428571 | 23 | 42 | 0 | 0 |
| Path 274 | C00031->C00407:[4->1,4->3,7->6,7->9,9->2,9->5] | 1.00 | 440.803921569 | 30 | 51 | 1 | 1 |
| Path 275 | C00031->C00407:[4->1,4->3,7->6,7->9,9->2,9->5] | 1.00 | 461.483333333 | 36 | 60 | 1 | 1 |
| Path 276 | C00031->C00407:[1->2,4->3,4->6] | 0.50 | 488.953846154 | 29 | 65 | 1 | 1 |
| Path 277 | C00031->C00407:[1->2,4->1,4->3,4->6,7->1,7->3,7->9,9->5] | 1.00 | 463.175 | 37 | 80 | 1 | 1 |
| Path 278 | C00031->C00407:[1->2,4->6,7->3] | 0.50 | 440.06779661 | 27 | 59 | 1 | 1 |
| Path 279 | C00031->C00407:[4->1,4->3,7->6,7->9,9->2,9->5] | 1.00 | 455.914285714 | 36 | 70 | 1 | 1 |
| Path 280 | C00031->C00407:[5->3] | 0.17 | 308.928571429 | 14 | 42 | 1 | 2 |
| Path 281 | C00031->C00407:[4->1,4->3,7->6,7->9,9->2,9->5] | 1.00 | 487.25974026 | 38 | 77 | 1 | 1 |
| Path 282 | C00031->C00407:[4->2] | 0.17 | 354.409090909 | 16 | 44 | 0 | 0 |
| Path 283 | C00031->C00407:[4->3,4->5,7->9] | 0.50 | 365.956521739 | 28 | 69 | 1 | 1 |
| Path 284 | C00031->C00407:[7->6,7->9,9->2,9->5] | 0.67 | 496.039215686 | 21 | 51 | 0 | 0 |
| Path 285 | C00031->C00407:[1->2,1->5,4->3,4->6,4->9] | 0.83 | 504.376811594 | 29 | 69 | 1 | 1 |
| Path 286 | C00031->C00407:[4->3,7->6,7->9,9->2,9->5] | 0.83 | 458.407407407 | 30 | 54 | 1 | 1 |
| Path 287 | C00031->C00407:[4->2,4->3,4->5] | 0.50 | 370.218181818 | 23 | 55 | 1 | 1 |
| Path 288 | C00031->C00407:[4->1,4->3,7->1,7->3,7->6,7->9,9->2,9->5] | 1.00 | 462.506849315 | 39 | 73 | 1 | 1 |
| Path 289 | C00031->C00407:[1->2,4->6] | 0.33 | 496.612244898 | 16 | 49 | 0 | 0 |
| Path 290 | C00031->C00407:[9->3] | 0.17 | 338.848484848 | 15 | 33 | 1 | 2 |
| Path 291 | C00031->C00407:[1->2,1->5,4->1,4->3,4->6,4->9] | 1.00 | 466.013157895 | 32 | 76 | 1 | 1 |
| Path 292 | C00031->C00407:[4->3] | 0.17 | 289.333333333 | 10 | 21 | 1 | 2 |
| Path 293 | C00031->C00407:[4->1,4->3,7->1,7->3,7->6,7->9,9->2,9->5] | 1.00 | 412.907407407 | 29 | 54 | 1 | 1 |
| Path 294 | C00031->C00407:[4->1,4->3,7->6,7->9,9->2,9->5] | 1.00 | 444.307692308 | 36 | 65 | 1 | 1 |
| Path 295 | C00031->C00407:[4->3,7->6,7->9,9->2,9->5] | 0.83 | 449.196969697 | 31 | 66 | 1 | 1 |
| Path 296 | C00031->C00407:[4->1,4->3,7->6,7->9,9->2,9->5] | 1.00 | 428.169491525 | 33 | 59 | 1 | 1 |
| Path 297 | C00031->C00407:[7->6,7->9,9->2,9->5] | 0.67 | 397.685714286 | 19 | 35 | 0 | 0 |
| Path 298 | C00031->C00407:[7->3,7->6,7->9,9->2,9->5] | 0.83 | 434.302325581 | 24 | 43 | 1 | 1 |
| Path 299 | C00031->C00407:[1->2,4->3,4->6] | 0.50 | 492.671428571 | 28 | 70 | 1 | 1 |
| Path 300 | C00031->C00407:[1->2,4->1,4->3,4->6,7->9,9->5] | 1.00 | 433.965116279 | 40 | 86 | 1 | 1 |
| Path 301 | C00031->C00407:[4->3] | 0.17 | 317.833333333 | 11 | 24 | 1 | 2 |
| Path 302 | C00031->C00407:[4->3,5->2,5->5,9->6,9->9] | 0.83 | 484.405063291 | 34 | 79 | 1 | 1 |
| Path 303 | C00031->C00407:[4->2,7->6] | 0.33 | 349.145454545 | 20 | 55 | 0 | 0 |
| Path 304 | C00031->C00407:[4->1,4->3,7->6,7->9,9->2,9->5] | 1.00 | 447.733333333 | 33 | 60 | 1 | 1 |
| Path 305 | C00031->C00407:[4->1,4->3,7->6,7->9,9->2,9->5] | 1.00 | 458.057142857 | 36 | 70 | 1 | 1 |
| Path 306 | C00031->C00407:[4->3,7->6,7->9,9->2,9->5] | 0.83 | 436.979591837 | 27 | 49 | 1 | 1 |
| Path 307 | C00031->C00407:[1->2,4->1,4->3,4->6,7->9,9->5] | 1.00 | 451.085365854 | 37 | 82 | 1 | 1 |
| Path 308 | C00031->C00407:[1->2,4->6,7->9,9->5] | 0.67 | 468.0 | 21 | 56 | 0 | 0 |
| Path 309 | C00031->C00407:[4->2,7->6] | 0.33 | 359.535714286 | 21 | 56 | 0 | 0 |
| Path 310 | C00031->C00407:[7->6,7->9,9->2,9->5] | 0.67 | 467.035714286 | 25 | 56 | 0 | 0 |
| Path 311 | C00031->C00407:[1->2,4->3,4->6] | 0.50 | 493.571428571 | 28 | 63 | 1 | 1 |
| Path 312 | C00031->C00407:[4->3,7->6,9->2] | 0.50 | 453.023255814 | 24 | 43 | 1 | 1 |
| Path 313 | C00031->C00407:[1->2,4->1,4->3,4->6,7->9,9->5] | 1.00 | 468.914634146 | 39 | 82 | 1 | 1 |
| Path 314 | C00031->C00407:[1->2,1->5,4->1,4->3,4->6,4->9] | 1.00 | 491.064102564 | 35 | 78 | 1 | 1 |
| Path 315 | C00031->C00407:[1->2,1->5,4->1,4->3,4->6,4->9,5->6,5->9,9->2,9->5] | 1.00 | 477.387096774 | 42 | 93 | 1 | 1 |
| Path 316 | C00031->C00407:[1->2,4->3,4->6] | 0.50 | 502.215384615 | 27 | 65 | 1 | 1 |
| Path 317 | C00031->C00407:[4->3,7->9,9->5] | 0.50 | 424.866666667 | 23 | 45 | 1 | 1 |
| Path 318 | C00031->C00407:[4->1,4->3,7->6,7->9,9->2,9->5] | 1.00 | 441.910447761 | 34 | 67 | 1 | 1 |
| Path 319 | C00031->C00407:[7->6,9->2] | 0.33 | 435.166666667 | 15 | 30 | 0 | 0 |
| Path 320 | C00031->C00407:[7->3,7->6,9->2] | 0.50 | 428.942857143 | 21 | 35 | 1 | 1 |
| Path 321 | C00031->C00407:[7->6,9->2] | 0.33 | 465.538461538 | 15 | 26 | 0 | 0 |
| Path 322 | C00031->C00407:[7->6,7->9,9->2,9->5] | 0.67 | 416.55 | 21 | 40 | 0 | 0 |
| Path 323 | C00031->C00407:[2->3] | 0.17 | 341.666666667 | 18 | 57 | 1 | 2 |
| Path 324 | C00031->C00407:[4->3,7->9,9->5] | 0.50 | 420.0 | 23 | 47 | 1 | 1 |
| Path 325 | C00031->C00407:[1->2,4->3,4->5,4->6,4->9] | 0.83 | 419.866666667 | 33 | 90 | 1 | 1 |
| Path 326 | C00031->C00407:[2->3] | 0.17 | 393.283333333 | 17 | 60 | 1 | 2 |
| Path 327 | C00031->C00407:[4->3,7->6,7->9,9->2,9->5] | 0.83 | 470.568181818 | 26 | 44 | 1 | 1 |
| Path 328 | C00031->C00407:[4->1,4->3,7->6,7->9,9->2,9->5] | 1.00 | 487.545454545 | 39 | 77 | 1 | 1 |
| Path 329 | C00031->C00407:[4->2,4->5,5->6,5->9] | 0.67 | 376.959459459 | 28 | 74 | 0 | 0 |
| Path 330 | C00031->C00407:[4->1,4->3,7->6,7->9,9->2,9->5] | 1.00 | 430.1 | 32 | 60 | 1 | 1 |
| Path 331 | C00031->C00407:[1->2,1->5,4->3,4->6,4->9] | 0.83 | 485.25974026 | 30 | 77 | 1 | 1 |
| Path 332 | C00031->C00407:[1->2,4->3,4->6] | 0.50 | 501.483870968 | 26 | 62 | 1 | 1 |
| Path 333 | C00031->C00407:[4->1,4->3,7->6,7->9,9->2,9->5] | 1.00 | 446.615384615 | 36 | 65 | 1 | 1 |
| Path 334 | C00031->C00407:[4->2,4->3,4->5,7->6,7->9,9->2,9->5] | 0.83 | 377.507042254 | 30 | 71 | 1 | 1 |
| Path 335 | C00031->C00407:[1->2,4->1,4->3,4->6,7->1,7->3,7->9,9->5] | 1.00 | 479.464285714 | 39 | 84 | 1 | 1 |
| Path 336 | C00031->C00407:[4->1,4->3,7->6,7->9,9->2,9->5] | 1.00 | 419.491803279 | 33 | 61 | 1 | 1 |
| Path 337 | C00031->C00407:[4->1,4->3,4->5,7->6,7->9,9->2] | 1.00 | 402.764705882 | 40 | 102 | 1 | 1 |
| Path 338 | C00031->C00407:[4->3,7->6,9->2] | 0.50 | 435.820512821 | 22 | 39 | 1 | 1 |
| Path 339 | C00031->C00407:[7->6,7->9,9->2,9->5] | 0.67 | 426.038461538 | 23 | 52 | 0 | 0 |
| Path 340 | C00031->C00407:[2->3] | 0.17 | 407.65 | 15 | 60 | 1 | 2 |
| Path 341 | C00031->C00407:[1->2,4->1,4->3,4->6,7->9,9->5] | 1.00 | 476.058823529 | 38 | 85 | 1 | 1 |
| Path 342 | C00031->C00407:[1->2,4->1,4->3,4->6,7->9,9->5] | 1.00 | 479.963414634 | 37 | 82 | 1 | 1 |
| Path 343 | C00031->C00407:[1->2,4->3,4->5,4->6,4->9] | 0.83 | 423.2 | 33 | 85 | 1 | 1 |
| Path 344 | C00031->C00407:[1->2,4->5,4->6,4->9] | 0.67 | 413.425287356 | 28 | 87 | 0 | 0 |
| Path 345 | C00031->C00407:[1->2,1->5,4->6,4->9] | 0.67 | 485.203703704 | 20 | 54 | 0 | 0 |
| Path 346 | C00031->C00407:[7->6,7->9,9->2,9->5] | 0.67 | 421.148148148 | 16 | 27 | 0 | 0 |
| Path 347 | C00031->C00407:[4->1,4->3,7->6,7->9,9->2,9->5] | 1.00 | 482.985915493 | 32 | 71 | 1 | 1 |
| Path 348 | C00031->C00407:[2->3,7->9,9->5] | 0.50 | 334.511363636 | 26 | 88 | 1 | 2 |
| Path 349 | C00031->C00407:[1->2,4->3,4->6,7->9,9->5] | 0.83 | 437.333333333 | 32 | 72 | 1 | 1 |
| Path 350 | C00031->C00407:[1->2,4->1,4->3,4->6,7->1,7->3,7->9,9->5] | 1.00 | 462.146067416 | 43 | 89 | 1 | 1 |
| Path 351 | C00031->C00407:[7->3,7->6,9->2] | 0.50 | 447.805555556 | 22 | 36 | 1 | 1 |
| Path 352 | C00031->C00407:[7->6,7->9,9->2,9->5] | 0.67 | 344.596774194 | 24 | 62 | 0 | 0 |
| Path 353 | C00031->C00407:[4->1,4->3,7->6,7->9,9->2,9->5] | 1.00 | 456.0 | 38 | 72 | 1 | 1 |
| Path 354 | C00031->C00407:[4->1,4->3,7->6,7->9,9->2,9->5] | 1.00 | 418.590163934 | 33 | 61 | 1 | 1 |
| Path 355 | C00031->C00407:[4->3,7->6,7->9,9->2,9->5] | 0.83 | 447.732142857 | 31 | 56 | 1 | 1 |
| Path 356 | C00031->C00407:[7->3,7->6,9->2] | 0.50 | 441.738095238 | 25 | 42 | 1 | 1 |
| Path 357 | C00031->C00407:[4->1,4->3,7->1,7->3,7->6,7->9,9->2,9->5] | 1.00 | 460.46031746 | 37 | 63 | 1 | 1 |
| Path 358 | C00031->C00407:[4->2,4->6] | 0.33 | 423.385542169 | 25 | 83 | 0 | 0 |
| Path 359 | C00031->C00407:[9->3] | 0.17 | 209.75 | 13 | 48 | 1 | 2 |
| Path 360 | C00031->C00407:[4->3,7->6,7->9,9->2,9->5] | 0.83 | 497.212121212 | 32 | 66 | 1 | 1 |
| Path 361 | C00031->C00407:[1->2,4->1,4->3,4->6,7->9,9->5] | 1.00 | 470.813953488 | 41 | 86 | 1 | 1 |
| Path 362 | C00031->C00407:[1->2,4->6,7->3,7->9,9->5] | 0.83 | 473.432835821 | 30 | 67 | 1 | 1 |
| Path 363 | C00031->C00407:[4->3,4->5,7->6,7->9,9->2] | 0.83 | 382.594202899 | 30 | 69 | 1 | 1 |
| Path 364 | C00031->C00407:[1->2,4->6,7->9,9->5] | 0.67 | 470.306451613 | 23 | 62 | 0 | 0 |
| Path 365 | C00031->C00407:[4->1,4->3,7->6,7->9,9->2,9->5] | 1.00 | 496.6375 | 37 | 80 | 1 | 1 |
| Path 366 | C00031->C00407:[4->1,4->3,7->1,7->3,7->6,7->9,9->2,9->5] | 1.00 | 419.070175439 | 32 | 57 | 1 | 1 |
| Path 367 | C00031->C00407:[1->2,4->6,7->9,9->5] | 0.67 | 459.842105263 | 23 | 57 | 0 | 0 |
| Path 368 | C00031->C00407:[4->3,7->6,7->9,9->2,9->5] | 0.83 | 496.878787879 | 31 | 66 | 1 | 1 |
| Path 369 | C00031->C00407:[1->2,4->5,4->6,4->9] | 0.67 | 405.646341463 | 29 | 82 | 0 | 0 |
| Path 370 | C00031->C00407:[4->3,7->6,7->9,9->2,9->5] | 0.83 | 374.154929577 | 30 | 71 | 1 | 1 |
| Path 371 | C00031->C00407:[4->3,7->6,7->9,9->2,9->5] | 0.83 | 418.081632653 | 34 | 98 | 1 | 1 |
| Path 372 | C00031->C00407:[4->3,7->6,9->2] | 0.50 | 469.19047619 | 25 | 42 | 1 | 1 |
| Path 373 | C00031->C00407:[4->3,7->6,7->9,9->2,9->5] | 0.83 | 422.488888889 | 25 | 45 | 1 | 1 |
| Path 374 | C00031->C00407:[4->1,4->3,7->6,7->9,9->2,9->5] | 1.00 | 491.341463415 | 38 | 82 | 1 | 1 |
| Path 375 | C00031->C00407:[1->2,4->6] | 0.33 | 491.568181818 | 17 | 44 | 0 | 0 |
| Path 376 | C00031->C00407:[4->1,4->3,4->5,7->6,7->9,9->2] | 1.00 | 384.0 | 35 | 77 | 1 | 1 |
| Path 377 | C00031->C00407:[1->2,1->5,4->6,4->9] | 0.67 | 471.839285714 | 21 | 56 | 0 | 0 |
| Path 378 | C00031->C00407:[4->3,5->2,5->5,9->6,9->9] | 0.83 | 434.960784314 | 27 | 51 | 1 | 1 |
| Path 379 | C00031->C00407:[1->2,4->3,4->6] | 0.50 | 443.619047619 | 28 | 63 | 1 | 1 |
| Path 380 | C00031->C00407:[1->2,1->5,4->1,4->3,4->6,4->9] | 1.00 | 486.316455696 | 35 | 79 | 1 | 1 |
| Path 381 | C00031->C00407:[7->6,9->2] | 0.33 | 462.708333333 | 14 | 24 | 0 | 0 |
| Path 382 | C00031->C00407:[4->3,7->9,9->5] | 0.50 | 240.315789474 | 20 | 57 | 1 | 2 |
| Path 383 | C00031->C00407:[1->2,1->5,4->1,4->3,4->6,4->9] | 1.00 | 486.873563218 | 40 | 87 | 1 | 1 |
| Path 384 | C00031->C00407:[1->2,4->5,4->6,4->9] | 0.67 | 421.318181818 | 29 | 88 | 0 | 0 |
| Path 385 | C00031->C00407:[7->9,9->3,9->5] | 0.50 | 205.587301587 | 19 | 63 | 1 | 2 |
| Path 386 | C00031->C00407:[1->2,1->5,4->1,4->3,4->6,4->9] | 1.00 | 477.049382716 | 36 | 81 | 1 | 1 |
| Path 387 | C00031->C00407:[7->3,7->6,9->2] | 0.50 | 454.945945946 | 23 | 37 | 1 | 1 |
| Path 388 | C00031->C00407:[4->1,4->3,7->6,7->9,9->2,9->5] | 1.00 | 430.596774194 | 34 | 62 | 1 | 1 |
| Path 389 | C00031->C00407:[4->1,4->3,7->6,7->9,9->2,9->5] | 1.00 | 475.460526316 | 33 | 76 | 1 | 1 |
| Path 390 | C00031->C00407:[4->1,4->3,7->6,7->9,9->2,9->5] | 1.00 | 491.97752809 | 42 | 89 | 1 | 1 |
| Path 391 | C00031->C00407:[1->2,4->1,4->3,4->6,7->9,9->5] | 1.00 | 474.481927711 | 40 | 83 | 1 | 1 |
| Path 392 | C00031->C00407:[7->9,9->3,9->5] | 0.50 | 336.409090909 | 26 | 88 | 1 | 2 |
| Path 393 | C00031->C00407:[4->1,4->3,7->6,7->9,9->2,9->5] | 1.00 | 443.303571429 | 31 | 56 | 1 | 1 |
| Path 394 | C00031->C00407:[4->3,7->9,9->5] | 0.50 | 422.459459459 | 22 | 37 | 1 | 1 |
| Path 395 | C00031->C00407:[2->3,7->9,9->5] | 0.50 | 334.261363636 | 25 | 88 | 1 | 2 |
| Path 396 | C00031->C00407:[7->3,7->6,7->9,9->2,9->5] | 0.83 | 421.659090909 | 25 | 44 | 1 | 1 |
| Path 397 | C00031->C00407:[4->1,4->3,7->6,7->9,9->2,9->5] | 1.00 | 476.625 | 35 | 72 | 1 | 1 |
| Path 398 | C00031->C00407:[4->3,7->6,9->2] | 0.50 | 472.4 | 26 | 45 | 1 | 1 |
| Path 399 | C00031->C00407:[1->2,4->6,7->3,7->9,9->5] | 0.83 | 426.462686567 | 30 | 67 | 1 | 1 |
| Path 400 | C00031->C00407:[1->2,4->6,7->9,9->5] | 0.67 | 413.706896552 | 24 | 58 | 0 | 0 |
| Path 401 | C00031->C00407:[4->3,7->6,7->9,9->2,9->5] | 0.83 | 455.058823529 | 27 | 51 | 1 | 1 |
| Path 402 | C00031->C00407:[4->1,4->3,7->6,7->9,9->2,9->5] | 1.00 | 462.473684211 | 36 | 76 | 1 | 1 |
| Path 403 | C00031->C00407:[4->3,7->6,9->2] | 0.50 | 441.642857143 | 23 | 42 | 1 | 1 |
| Path 404 | C00031->C00407:[7->6,7->9,9->2,9->5] | 0.67 | 495.257575758 | 27 | 66 | 0 | 0 |
| Path 405 | C00031->C00407:[4->1,4->3,7->6,7->9,9->2,9->5] | 1.00 | 439.5 | 34 | 60 | 1 | 1 |
| Path 406 | C00031->C00407:[4->3,7->6,7->9,9->2,9->5] | 0.83 | 466.48 | 28 | 50 | 1 | 1 |
| Path 407 | C00031->C00407:[1->5,4->3,4->9] | 0.50 | 496.685714286 | 28 | 70 | 1 | 1 |
| Path 408 | C00031->C00407:[4->3,4->5,7->6,7->9,9->2] | 0.83 | 429.649484536 | 35 | 97 | 1 | 1 |
| Path 409 | C00031->C00407:[4->1,4->3,7->6,7->9,9->2,9->5] | 1.00 | 432.611111111 | 34 | 72 | 1 | 1 |
| Path 410 | C00031->C00407:[1->2,4->3,4->5,4->6,4->9] | 0.83 | 428.8 | 33 | 95 | 1 | 1 |
| Path 411 | C00031->C00407:[1->2,4->6,7->3,7->9,9->5] | 0.83 | 467.46969697 | 30 | 66 | 1 | 1 |
| Path 412 | C00031->C00407:[4->1,4->3,7->6,7->9,9->2,9->5] | 1.00 | 485.913580247 | 37 | 81 | 1 | 1 |
| Path 413 | C00031->C00407:[4->3,7->6,7->9,9->2,9->5] | 0.83 | 428.416666667 | 26 | 48 | 1 | 1 |
| Path 414 | C00031->C00407:[4->3,7->6,7->9,9->2,9->5] | 0.83 | 362.958333333 | 29 | 72 | 1 | 1 |
| Path 415 | C00031->C00407:[1->3] | 0.17 | 404.114754098 | 18 | 61 | 1 | 2 |
| Path 416 | C00031->C00407:[7->6,7->9,9->2,9->5] | 0.67 | 369.483870968 | 15 | 31 | 0 | 0 |
| Path 417 | C00031->C00407:[4->1,4->3,7->6,7->9,9->2,9->5] | 1.00 | 474.816091954 | 38 | 87 | 1 | 1 |
| Path 418 | C00031->C00407:[4->3,4->5,7->6,7->9,9->2] | 0.83 | 373.558823529 | 29 | 68 | 1 | 1 |
| Path 419 | C00031->C00407:[4->1,4->3,7->6,7->9,9->2,9->5] | 1.00 | 431.631578947 | 31 | 57 | 1 | 1 |
| Path 420 | C00031->C00407:[1->2,4->6,7->3] | 0.50 | 479.947368421 | 26 | 57 | 1 | 1 |
| Path 421 | C00031->C00407:[4->1,4->3,7->1,7->3,7->6,7->9,9->2,9->5] | 1.00 | 414.553571429 | 31 | 56 | 1 | 1 |
| Path 422 | C00031->C00407:[4->1,4->3,7->1,7->3,7->6,7->9,9->2,9->5] | 1.00 | 437.274193548 | 36 | 62 | 1 | 1 |
| Path 423 | C00031->C00407:[4->3,7->6,7->9,9->2,9->5] | 0.83 | 404.844444444 | 25 | 45 | 1 | 1 |
| Path 424 | C00031->C00407:[4->3,7->6,7->9,9->2,9->5] | 0.83 | 502.25 | 35 | 80 | 1 | 1 |
| Path 425 | C00031->C00407:[4->3,5->6,5->9,9->2,9->5] | 0.83 | 458.360655738 | 29 | 61 | 1 | 1 |
| Path 426 | C00031->C00407:[4->1,4->3,7->6,7->9,9->2,9->5] | 1.00 | 480.0 | 39 | 88 | 1 | 1 |
| Path 427 | C00031->C00407:[4->3,7->6,7->9,9->2,9->5] | 0.83 | 439.690909091 | 30 | 55 | 1 | 1 |
| Path 428 | C00031->C00407:[1->2,4->1,4->3,4->6,7->9,9->5] | 1.00 | 465.5125 | 34 | 80 | 1 | 1 |
| Path 429 | C00031->C00407:[4->1,4->3,7->6,7->9,9->2,9->5] | 1.00 | 404.41509434 | 28 | 53 | 1 | 1 |
| Path 430 | C00031->C00407:[4->3,7->6,7->9,9->2,9->5] | 0.83 | 421.044444444 | 23 | 45 | 1 | 1 |
| Path 431 | C00031->C00407:[4->2,4->3,4->5,5->2,5->5,9->6,9->9] | 0.83 | 363.070422535 | 30 | 71 | 1 | 1 |
| Path 432 | C00031->C00407:[1->2,4->3,4->6] | 0.50 | 501.338028169 | 29 | 71 | 1 | 1 |
| Path 433 | C00031->C00407:[4->1,4->3,7->6,7->9,9->2,9->5] | 1.00 | 343.386666667 | 31 | 75 | 1 | 1 |
| Path 434 | C00031->C00407:[4->3,4->5,7->6,7->9,9->2] | 0.83 | 422.583333333 | 34 | 96 | 1 | 1 |
| Path 435 | C00031->C00407:[1->2,4->3,4->6] | 0.50 | 505.426229508 | 29 | 61 | 1 | 1 |
| Path 436 | C00031->C00407:[1->2,4->3,4->6,7->9,9->5] | 0.83 | 473.92 | 30 | 75 | 1 | 1 |
| Path 437 | C00031->C00407:[1->2,4->3,4->6,7->9,9->5] | 0.83 | 479.934210526 | 31 | 76 | 1 | 1 |
| Path 438 | C00031->C00407:[4->3,7->6,7->9,9->2,9->5] | 0.83 | 368.342857143 | 29 | 70 | 1 | 1 |
| Path 439 | C00031->C00407:[4->3,7->6,7->9,9->2,9->5] | 0.83 | 427.0625 | 24 | 48 | 1 | 1 |
| Path 440 | C00031->C00407:[4->3,7->6,7->9,9->2,9->5] | 0.83 | 440.346938776 | 27 | 49 | 1 | 1 |
| Path 441 | C00031->C00407:[4->3,7->6,9->2] | 0.50 | 457.139534884 | 24 | 43 | 1 | 1 |
| Path 442 | C00031->C00407:[4->3,7->6,7->9,9->2,9->5] | 0.83 | 460.770491803 | 31 | 61 | 1 | 1 |
| Path 443 | C00031->C00407:[7->9,9->3,9->5] | 0.50 | 247.218181818 | 20 | 55 | 1 | 2 |
| Path 444 | C00031->C00407:[7->9,9->3,9->5] | 0.50 | 257.109090909 | 20 | 55 | 1 | 2 |
| Path 445 | C00031->C00407:[4->1,4->3,7->6,7->9,9->2,9->5] | 1.00 | 438.366666667 | 34 | 60 | 1 | 1 |
| Path 446 | C00031->C00407:[4->3,4->5,7->6,7->9,9->2] | 0.83 | 386.75 | 35 | 92 | 1 | 1 |
| Path 447 | C00031->C00407:[4->1,4->3,7->6,7->9,9->2,9->5] | 1.00 | 482.880952381 | 38 | 84 | 1 | 1 |
| Path 448 | C00031->C00407:[1->2,4->6,7->9,9->5] | 0.67 | 467.451612903 | 23 | 62 | 0 | 0 |
| Path 449 | C00031->C00407:[4->1,4->3,7->6,7->9,9->2,9->5] | 1.00 | 381.97752809 | 37 | 89 | 1 | 1 |
| Path 450 | C00031->C00407:[7->6,9->2] | 0.33 | 423.954545455 | 12 | 22 | 0 | 0 |
| Path 451 | C00031->C00407:[4->1,4->3,7->6,7->9,9->2,9->5] | 1.00 | 505.352941176 | 41 | 85 | 1 | 1 |
| Path 452 | C00031->C00407:[4->1,4->3,7->6,7->9,9->2,9->5] | 1.00 | 493.631578947 | 36 | 76 | 1 | 1 |
| Path 453 | C00031->C00407:[4->1,4->3,7->1,7->3,7->6,7->9,9->2,9->5] | 1.00 | 432.559322034 | 33 | 59 | 1 | 1 |
| Path 454 | C00031->C00407:[7->3,7->6,7->9,9->2,9->5] | 0.83 | 441.934782609 | 27 | 46 | 1 | 1 |
| Path 455 | C00031->C00407:[4->1,4->3,7->6,7->9,9->2,9->5] | 1.00 | 351.810126582 | 33 | 79 | 1 | 1 |
| Path 456 | C00031->C00407:[4->3,7->6,7->9,9->2,9->5] | 0.83 | 494.771428571 | 30 | 70 | 1 | 1 |
| Path 457 | C00031->C00407:[4->3,7->9,9->5] | 0.50 | 254.166666667 | 21 | 60 | 1 | 2 |
| Path 458 | C00031->C00407:[4->3,7->6,7->9,9->2,9->5] | 0.83 | 429.7 | 26 | 50 | 1 | 1 |
| Path 459 | C00031->C00407:[4->2,4->3,4->5,7->6,7->9] | 0.83 | 371.671641791 | 28 | 67 | 1 | 1 |
| Path 460 | C00031->C00407:[1->2,1->5,4->1,4->3,4->6,4->9] | 1.00 | 475.608108108 | 31 | 74 | 1 | 1 |
| Path 461 | C00031->C00407:[1->2,1->5,4->6,4->9] | 0.67 | 474.158730159 | 22 | 63 | 0 | 0 |
| Path 462 | C00031->C00407:[4->3,7->6,7->9,9->2,9->5] | 0.83 | 504.476923077 | 29 | 65 | 1 | 1 |
| Path 463 | C00031->C00407:[1->2,4->1,4->3,4->6,7->9,9->5] | 1.00 | 470.558139535 | 40 | 86 | 1 | 1 |
| Path 464 | C00031->C00407:[4->2,4->5,7->6,7->9,9->2,9->5] | 0.67 | 361.609375 | 26 | 64 | 0 | 0 |
| Path 465 | C00031->C00407:[4->1,4->3,7->1,7->3,7->6,7->9,9->2,9->5] | 1.00 | 428.732142857 | 31 | 56 | 1 | 1 |
| Path 466 | C00031->C00407:[5->2,5->5,9->6,9->9] | 0.67 | 473.461538462 | 26 | 65 | 0 | 0 |
| Path 467 | C00031->C00407:[1->2,4->6,7->3,7->9,9->5] | 0.83 | 472.676056338 | 29 | 71 | 1 | 1 |
| Path 468 | C00031->C00407:[4->1,4->3] | 0.33 | 436.696969697 | 20 | 33 | 1 | 1 |
| Path 469 | C00031->C00407:[1->2,4->6,7->3,7->9,9->5] | 0.83 | 458.774647887 | 32 | 71 | 1 | 1 |
| Path 470 | C00031->C00407:[7->6,7->9,9->2,9->5] | 0.67 | 407.073170732 | 22 | 41 | 0 | 0 |
| Path 471 | C00031->C00407:[7->3,7->6,7->9,9->2,9->5] | 0.83 | 413.731707317 | 22 | 41 | 1 | 1 |
| Path 472 | C00031->C00407:[4->1,4->3,7->6,7->9,9->2,9->5] | 1.00 | 418.630434783 | 26 | 46 | 1 | 1 |
| Path 473 | C00031->C00407:[4->1,4->3,4->5,7->6,7->9,9->2] | 1.00 | 410.5 | 38 | 98 | 1 | 1 |
| Path 474 | C00031->C00407:[4->3,7->6,9->2] | 0.50 | 452.625 | 23 | 40 | 1 | 1 |
| Path 475 | C00031->C00407:[4->3,7->6,7->9,9->2,9->5] | 0.83 | 354.323529412 | 27 | 68 | 1 | 1 |
| Path 476 | C00031->C00407:[4->3,7->6,7->9,9->2,9->5] | 0.83 | 424.106382979 | 25 | 47 | 1 | 1 |
| Path 477 | C00031->C00407:[4->2,4->3,4->5,4->6,4->9] | 0.83 | 424.563829787 | 32 | 94 | 1 | 1 |
| Path 478 | C00031->C00407:[1->2,4->3,4->6] | 0.50 | 491.567164179 | 27 | 67 | 1 | 1 |
| Path 479 | C00031->C00407:[7->6,9->2] | 0.33 | 432.0 | 12 | 22 | 0 | 0 |
| Path 480 | C00031->C00407:[4->1,4->3,7->1,7->3,7->6,7->9,9->2,9->5] | 1.00 | 444.222222222 | 37 | 63 | 1 | 1 |
| Path 481 | C00031->C00407:[1->2,4->1,4->3,4->6,7->9,9->5] | 1.00 | 462.218390805 | 41 | 87 | 1 | 1 |
| Path 482 | C00031->C00407:[4->2,4->5,5->2,5->5,9->6,9->9] | 0.67 | 345.59375 | 26 | 64 | 0 | 0 |
| Path 483 | C00031->C00407:[1->2,1->5,4->1,4->3,4->6,4->9] | 1.00 | 468.265060241 | 33 | 83 | 1 | 1 |
| Path 484 | C00031->C00407:[4->1,4->3,7->6,7->9,9->2,9->5] | 1.00 | 476.319444444 | 34 | 72 | 1 | 1 |
| Path 485 | C00031->C00407:[1->2,4->1,4->3,4->6,7->9,9->5] | 1.00 | 477.098901099 | 40 | 91 | 1 | 1 |
| Path 486 | C00031->C00407:[4->3,4->5,7->6,7->9,9->2] | 0.83 | 412.302083333 | 37 | 96 | 1 | 1 |
| Path 487 | C00031->C00407:[4->1,4->3,7->6,7->9,9->2,9->5] | 1.00 | 452.85483871 | 36 | 62 | 1 | 1 |
| Path 488 | C00031->C00407:[7->3,7->6,7->9,9->2,9->5] | 0.83 | 432.333333333 | 29 | 51 | 1 | 1 |
| Path 489 | C00031->C00407:[1->5,4->3,4->9] | 0.50 | 495.76119403 | 27 | 67 | 1 | 1 |
| Path 490 | C00031->C00407:[5->3,7->9,9->5] | 0.50 | 259.826086957 | 22 | 69 | 1 | 2 |
| Path 491 | C00031->C00407:[4->1,4->3,7->6,7->9,9->2,9->5] | 1.00 | 368.595238095 | 37 | 84 | 1 | 1 |
| Path 492 | C00031->C00407:[7->3,7->6,7->9,9->2,9->5] | 0.83 | 417.418604651 | 24 | 43 | 1 | 1 |
| Path 493 | C00031->C00407:[1->2,1->5,4->6,4->9] | 0.67 | 504.948275862 | 23 | 58 | 0 | 0 |
| Path 494 | C00031->C00407:[4->1,4->3,7->1,7->3,7->6,7->9,9->2,9->5] | 1.00 | 415.767857143 | 31 | 56 | 1 | 1 |
| Path 495 | C00031->C00407:[4->1,4->3,7->6,7->9,9->2,9->5] | 1.00 | 440.25 | 36 | 64 | 1 | 1 |
| Path 496 | C00031->C00407:[4->5,7->6,7->9,9->2] | 0.67 | 352.3 | 24 | 60 | 0 | 0 |
| Path 497 | C00031->C00407:[1->2,4->3,4->6,7->9,9->5] | 0.83 | 481.347222222 | 33 | 72 | 1 | 1 |
| Path 498 | C00031->C00407:[4->1,4->3,7->6,7->9,9->2,9->5] | 1.00 | 425.137931034 | 32 | 58 | 1 | 1 |
| Path 499 | C00031->C00407:[7->3,7->6,7->9,9->2,9->5] | 0.83 | 443.42 | 28 | 50 | 1 | 1 |
| Path 500 | C00031->C00407:[1->2,4->6] | 0.33 | 499.866666667 | 17 | 45 | 0 | 0 |
| Path 501 | C00031->C00407:[1->2,4->3,4->6,7->9,9->5] | 0.83 | 476.72972973 | 33 | 74 | 1 | 1 |
| Path 502 | C00031->C00407:[4->3,7->6,7->9,9->2,9->5] | 0.83 | 432.130434783 | 24 | 46 | 1 | 1 |
| Path 503 | C00031->C00407:[4->1,4->3,7->6,7->9,9->2,9->5] | 1.00 | 458.824324324 | 38 | 74 | 1 | 1 |
| Path 504 | C00031->C00407:[7->3] | 0.17 | 234.96 | 10 | 25 | 1 | 2 |
| Path 505 | C00031->C00407:[1->2,4->3,4->6,7->9,9->5] | 0.83 | 482.263157895 | 31 | 76 | 1 | 1 |
| Path 506 | C00031->C00407:[7->6,7->9,9->2,9->5] | 0.67 | 478.828571429 | 28 | 70 | 0 | 0 |
| Path 507 | C00031->C00407:[4->1,4->3,7->6,7->9,9->2,9->5] | 1.00 | 435.727272727 | 37 | 66 | 1 | 1 |
| Path 508 | C00031->C00407:[1->2,4->1,4->3,4->6,7->9,9->5] | 1.00 | 460.074074074 | 37 | 81 | 1 | 1 |
| Path 509 | C00031->C00407:[4->3,7->6,7->9,9->2,9->5] | 0.83 | 491.844444444 | 28 | 45 | 1 | 1 |
| Path 510 | C00031->C00407:[7->6,7->9,9->2,9->5] | 0.67 | 484.660714286 | 22 | 56 | 0 | 0 |
| Path 511 | C00031->C00407:[4->3,7->6,7->9,9->2,9->5] | 0.83 | 433.117647059 | 27 | 51 | 1 | 1 |
| Path 512 | C00031->C00407:[1->2,4->3,4->6,7->9,9->5] | 0.83 | 430.38028169 | 31 | 71 | 1 | 1 |
| Path 513 | C00031->C00407:[4->3,7->6,7->9,9->2,9->5] | 0.83 | 468.653061224 | 29 | 49 | 1 | 1 |
| Path 514 | C00031->C00407:[4->1,4->3,7->6,7->9,9->2,9->5] | 1.00 | 418.113207547 | 28 | 53 | 1 | 1 |
| Path 515 | C00031->C00407:[4->1,4->3,7->6,7->9,9->2,9->5] | 1.00 | 438.0 | 37 | 66 | 1 | 1 |
| Path 516 | C00031->C00407:[4->3,4->5,7->6,7->9,9->2] | 0.83 | 370.119402985 | 28 | 67 | 1 | 1 |
| Path 517 | C00031->C00407:[7->6,7->9,9->2,9->5] | 0.67 | 438.595744681 | 23 | 47 | 0 | 0 |
| Path 518 | C00031->C00407:[1->2,4->1,4->3,4->6,7->1,7->3,7->9,9->5] | 1.00 | 436.835294118 | 41 | 85 | 1 | 1 |
| Path 519 | C00031->C00407:[4->1,4->3,4->5,7->6,7->9,9->2] | 1.00 | 412.441176471 | 37 | 102 | 1 | 1 |
| Path 520 | C00031->C00407:[4->1,4->3,7->6,7->9,9->2,9->5] | 1.00 | 428.516666667 | 32 | 60 | 1 | 1 |
| Path 521 | C00031->C00407:[4->3,7->9,9->5] | 0.50 | 430.645833333 | 24 | 48 | 1 | 1 |
| Path 522 | C00031->C00407:[4->1,4->3,7->6,7->9,9->2,9->5] | 1.00 | 454.03030303 | 38 | 66 | 1 | 1 |
| Path 523 | C00031->C00407:[4->1,4->3,7->1,7->3,7->6,7->9,9->2,9->5] | 1.00 | 436.587301587 | 35 | 63 | 1 | 1 |
| Path 524 | C00031->C00407:[4->3,4->5] | 0.33 | 371.140350877 | 23 | 57 | 1 | 1 |
| Path 525 | C00031->C00407:[1->2,4->6,7->9,9->5] | 0.67 | 468.344827586 | 25 | 58 | 0 | 0 |
| Path 526 | C00031->C00407:[4->3,7->9,9->5] | 0.50 | 413.363636364 | 22 | 44 | 1 | 1 |
| Path 527 | C00031->C00407:[7->3,7->6,7->9,9->2,9->5] | 0.83 | 426.047619048 | 23 | 42 | 1 | 1 |
| Path 528 | C00031->C00407:[4->5,7->6,7->9,9->2] | 0.67 | 339.491525424 | 23 | 59 | 0 | 0 |
| Path 529 | C00031->C00407:[1->2,4->6,7->3,7->9,9->5] | 0.83 | 481.5 | 30 | 72 | 1 | 1 |
| Path 530 | C00031->C00407:[4->1,4->3,7->6,7->9,9->2,9->5] | 1.00 | 448.101449275 | 38 | 69 | 1 | 1 |
| Path 531 | C00031->C00407:[4->3,7->6,7->9,9->2,9->5] | 0.83 | 360.188405797 | 28 | 69 | 1 | 1 |
| Path 532 | C00031->C00407:[7->6,9->2] | 0.33 | 402.75 | 13 | 28 | 0 | 0 |
| Path 533 | C00031->C00407:[7->6,7->9,9->2,9->5] | 0.67 | 427.678571429 | 17 | 28 | 0 | 0 |
| Path 534 | C00031->C00407:[1->2,4->1,4->3,4->6,7->1,7->3,7->9,9->5] | 1.00 | 463.023809524 | 36 | 84 | 1 | 1 |
| Path 535 | C00031->C00407:[4->1,4->3,7->1,7->3,7->6,7->9,9->2,9->5] | 1.00 | 423.316666667 | 34 | 60 | 1 | 1 |
| Path 536 | C00031->C00407:[4->1,4->3,7->6,7->9,9->2,9->5] | 1.00 | 450.435483871 | 36 | 62 | 1 | 1 |
| Path 537 | C00031->C00407:[4->1,4->3,4->5,7->6,7->9,9->2] | 1.00 | 375.296296296 | 36 | 81 | 1 | 1 |
| Path 538 | C00031->C00407:[4->1,4->3,7->6,7->9,9->2,9->5] | 1.00 | 443.844827586 | 33 | 58 | 1 | 1 |
| Path 539 | C00031->C00407:[1->2,4->5,4->6,4->9] | 0.67 | 411.180722892 | 29 | 83 | 0 | 0 |
| Path 540 | C00031->C00407:[4->1,4->3,7->6,7->9,9->2,9->5] | 1.00 | 408.111111111 | 29 | 54 | 1 | 1 |
| Path 541 | C00031->C00407:[4->3,7->9,9->5] | 0.50 | 429.575 | 23 | 40 | 1 | 1 |
| Path 542 | C00031->C00407:[4->1,4->3,7->6,7->9,9->2,9->5] | 1.00 | 422.915254237 | 32 | 59 | 1 | 1 |
| Path 543 | C00031->C00407:[4->2,4->3,4->5,7->6,7->9,9->2,9->5] | 0.83 | 385.850746269 | 30 | 67 | 1 | 1 |
| Path 544 | C00031->C00407:[1->2,4->3,4->6,7->9,9->5] | 0.83 | 460.76 | 33 | 75 | 1 | 1 |
| Path 545 | C00031->C00407:[4->3,7->6,7->9,9->2,9->5] | 0.83 | 441.120689655 | 31 | 58 | 1 | 1 |
| Path 546 | C00031->C00407:[4->3,7->6,7->9,9->2,9->5] | 0.83 | 415.857142857 | 25 | 49 | 1 | 1 |
| Path 547 | C00031->C00407:[1->2,1->5,4->3,4->6,4->9] | 0.83 | 510.833333333 | 31 | 72 | 1 | 1 |
| Path 548 | C00031->C00407:[7->6,7->9,9->2,9->5] | 0.67 | 374.909090909 | 17 | 33 | 0 | 0 |
| Path 549 | C00031->C00407:[4->3,7->6,7->9,9->2,9->5] | 0.83 | 451.166666667 | 26 | 48 | 1 | 1 |
| Path 550 | C00031->C00407:[4->1,4->3,7->6,7->9,9->2,9->5] | 1.00 | 436.854166667 | 28 | 48 | 1 | 1 |
| Path 551 | C00031->C00407:[1->2,4->1,4->3,4->6,7->9,9->5] | 1.00 | 463.282051282 | 35 | 78 | 1 | 1 |
| Path 552 | C00031->C00407:[4->3,7->6,7->9,9->2,9->5] | 0.83 | 466.192307692 | 30 | 52 | 1 | 1 |
| Path 553 | C00031->C00407:[4->3,7->6,7->9,9->2,9->5] | 0.83 | 365.704225352 | 30 | 71 | 1 | 1 |
| Path 554 | C00031->C00407:[7->6,7->9,9->2,9->5] | 0.67 | 493.25 | 27 | 64 | 0 | 0 |
| Path 555 | C00031->C00407:[4->3,7->6,7->9,9->2,9->5] | 0.83 | 369.104477612 | 28 | 67 | 1 | 1 |
| Path 556 | C00031->C00407:[4->1,4->3,7->6,7->9,9->2,9->5] | 1.00 | 470.072289157 | 35 | 83 | 1 | 1 |
| Path 557 | C00031->C00407:[1->2,1->5,4->1,4->3,4->6,4->9] | 1.00 | 494.0875 | 36 | 80 | 1 | 1 |
| Path 558 | C00031->C00407:[1->2,4->1,4->3,4->6,7->9,9->5] | 1.00 | 430.268292683 | 38 | 82 | 1 | 1 |
| Path 559 | C00031->C00407:[5->3,7->9,9->5] | 0.50 | 340.258064516 | 25 | 93 | 1 | 2 |
| Path 560 | C00031->C00407:[4->3,7->6,7->9,9->2,9->5] | 0.83 | 362.68115942 | 28 | 69 | 1 | 1 |
| Path 561 | C00031->C00407:[4->1,4->3,7->6,7->9,9->2,9->5] | 1.00 | 419.327272727 | 30 | 55 | 1 | 1 |
| Path 562 | C00031->C00407:[4->1,4->3,7->6,7->9,9->2,9->5] | 1.00 | 416.6 | 30 | 55 | 1 | 1 |
| Path 563 | C00031->C00407:[7->6,7->9,9->2,9->5] | 0.67 | 401.971428571 | 19 | 35 | 0 | 0 |
| Path 564 | C00031->C00407:[4->1,4->3,7->6,7->9,9->2,9->5] | 1.00 | 399.375 | 29 | 56 | 1 | 1 |
| Path 565 | C00031->C00407:[4->3,7->6,7->9,9->2,9->5] | 0.83 | 377.705128205 | 34 | 78 | 1 | 1 |
| Path 566 | C00031->C00407:[1->2,1->5,4->6,4->9,5->6,5->9,9->2,9->5] | 0.67 | 473.220588235 | 27 | 68 | 0 | 0 |
| Path 567 | C00031->C00407:[1->2,4->3,4->6,7->9,9->5] | 0.83 | 482.917808219 | 30 | 73 | 1 | 1 |
| Path 568 | C00031->C00407:[1->2,4->3,4->6,7->9,9->5] | 0.83 | 475.014084507 | 32 | 71 | 1 | 1 |
| Path 569 | C00031->C00407:[4->1,4->3,7->6,7->9,9->2,9->5] | 1.00 | 481.376623377 | 34 | 77 | 1 | 1 |
| Path 570 | C00031->C00407:[4->1,4->3,7->6,7->9,9->2,9->5] | 1.00 | 445.347826087 | 34 | 69 | 1 | 1 |
| Path 571 | C00031->C00407:[4->1,4->3,4->5,7->6,7->9,9->2] | 1.00 | 414.701030928 | 36 | 97 | 1 | 1 |
| Path 572 | C00031->C00407:[4->1,4->3,7->6,7->9,9->2,9->5] | 1.00 | 415.120689655 | 31 | 58 | 1 | 1 |
| Path 573 | C00031->C00407:[4->1,4->3,4->5,7->6,7->9,9->2] | 1.00 | 410.724489796 | 39 | 98 | 1 | 1 |
| Path 574 | C00031->C00407:[4->1,4->3,7->1,7->3,7->6,7->9,9->2,9->5] | 1.00 | 444.568965517 | 33 | 58 | 1 | 1 |
| Path 575 | C00031->C00407:[4->1,4->3,7->6,7->9,9->2,9->5] | 1.00 | 411.134615385 | 27 | 52 | 1 | 1 |
| Path 576 | C00031->C00407:[7->6,7->9,9->2,9->5] | 0.67 | 486.980769231 | 24 | 52 | 0 | 0 |
| Path 577 | C00031->C00407:[1->2,4->5,4->6,4->9] | 0.67 | 414.256410256 | 29 | 78 | 0 | 0 |
| Path 578 | C00031->C00407:[4->1,4->3,7->6,7->9,9->2,9->5] | 1.00 | 427.6 | 32 | 60 | 1 | 1 |
| Path 579 | C00031->C00407:[4->1,4->3,7->6,7->9,9->2,9->5] | 1.00 | 443.692307692 | 32 | 65 | 1 | 1 |
| Path 580 | C00031->C00407:[1->2,4->1,4->3,4->6,7->9,9->5] | 1.00 | 463.564102564 | 36 | 78 | 1 | 1 |
| Path 581 | C00031->C00407:[2->3,7->9,9->5] | 0.50 | 329.0 | 27 | 92 | 1 | 2 |
| Path 582 | C00031->C00407:[7->6,7->9,9->2,9->5] | 0.67 | 472.258064516 | 20 | 31 | 0 | 0 |
| Path 583 | C00031->C00407:[1->2,1->5,4->1,4->3,4->6,4->9] | 1.00 | 478.295454545 | 37 | 88 | 1 | 1 |
| Path 584 | C00031->C00407:[4->3,4->5,7->6,7->9,9->2] | 0.83 | 425.549450549 | 33 | 91 | 1 | 1 |
| Path 585 | C00031->C00407:[4->1,4->3,7->6,7->9,9->2,9->5] | 1.00 | 410.614035088 | 30 | 57 | 1 | 1 |
| Path 586 | C00031->C00407:[1->2,1->5,4->3,4->6,4->9,5->6,5->9,9->2,9->5] | 0.83 | 483.804878049 | 35 | 82 | 1 | 1 |
| Path 587 | C00031->C00407:[2->3] | 0.17 | 347.714285714 | 16 | 56 | 1 | 2 |
| Path 588 | C00031->C00407:[4->1,4->3,7->6,7->9,9->2,9->5] | 1.00 | 357.202531646 | 35 | 79 | 1 | 1 |
| Path 589 | C00031->C00407:[4->5,7->6,7->9,9->2] | 0.67 | 375.655172414 | 26 | 58 | 0 | 0 |
| Path 590 | C00031->C00407:[1->2,4->3,4->5,4->6,4->9] | 0.83 | 384.9 | 33 | 90 | 1 | 1 |
| Path 591 | C00031->C00407:[5->6,9->2] | 0.33 | 445.175 | 17 | 40 | 0 | 0 |
| Path 592 | C00031->C00407:[4->1,4->3,7->6,7->9,9->2,9->5] | 1.00 | 456.471698113 | 32 | 53 | 1 | 1 |
| Path 593 | C00031->C00407:[1->2,4->1,4->3,4->6,7->9,9->5] | 1.00 | 420.950617284 | 36 | 81 | 1 | 1 |
| Path 594 | C00031->C00407:[1->2,4->6,7->3] | 0.50 | 500.921875 | 27 | 64 | 1 | 1 |
| Path 595 | C00031->C00407:[4->3,7->6,7->9,9->2,9->5] | 0.83 | 427.854166667 | 26 | 48 | 1 | 1 |
| Path 596 | C00031->C00407:[4->1,4->3,7->6,7->9,9->2,9->5] | 1.00 | 484.662790698 | 38 | 86 | 1 | 1 |
| Path 597 | C00031->C00407:[7->6,7->9,9->2,9->5] | 0.67 | 469.209677419 | 23 | 62 | 0 | 0 |
| Path 598 | C00031->C00407:[7->6,7->9,9->2,9->5] | 0.67 | 512.283333333 | 26 | 60 | 0 | 0 |
| Path 599 | C00031->C00407:[1->2,4->6,7->9,9->5] | 0.67 | 459.852459016 | 22 | 61 | 0 | 0 |
| Path 600 | C00031->C00407:[4->3,4->5] | 0.33 | 373.137931034 | 24 | 58 | 1 | 1 |
| Path 601 | C00031->C00407:[7->6,7->9,9->2,9->5] | 0.67 | 424.567567568 | 21 | 37 | 0 | 0 |
| Path 602 | C00031->C00407:[7->3,7->9,9->5] | 0.50 | 436.971428571 | 21 | 35 | 1 | 1 |
| Path 603 | C00031->C00407:[4->1,4->3,7->6,7->9,9->2,9->5] | 1.00 | 428.612903226 | 34 | 62 | 1 | 1 |
| Path 604 | C00031->C00407:[1->2,4->1,4->3,4->6,7->9,9->5] | 1.00 | 463.184210526 | 32 | 76 | 1 | 1 |
| Path 605 | C00031->C00407:[2->3] | 0.17 | 401.016393443 | 17 | 61 | 1 | 2 |
| Path 606 | C00031->C00407:[1->2,4->6,7->9,9->5] | 0.67 | 477.619047619 | 24 | 63 | 0 | 0 |
| Path 607 | C00031->C00407:[4->1,4->3,7->6,7->9,9->2,9->5] | 1.00 | 444.818181818 | 32 | 55 | 1 | 1 |
| Path 608 | C00031->C00407:[4->3,7->6,7->9,9->2,9->5] | 0.83 | 463.564516129 | 30 | 62 | 1 | 1 |
| Path 609 | C00031->C00407:[7->6,7->9,9->2,9->5] | 0.67 | 439.75 | 20 | 36 | 0 | 0 |
| Path 610 | C00031->C00407:[4->1,4->3,7->1,7->3,7->6,7->9,9->2,9->5] | 1.00 | 433.426229508 | 35 | 61 | 1 | 1 |
| Path 611 | C00031->C00407:[4->3,7->6,7->9,9->2,9->5] | 0.83 | 360.164383562 | 30 | 73 | 1 | 1 |
| Path 612 | C00031->C00407:[1->2,4->3,4->6] | 0.50 | 500.632352941 | 28 | 68 | 1 | 1 |
| Path 613 | C00031->C00407:[4->3,7->6,7->9,9->2,9->5] | 0.83 | 488.226190476 | 36 | 84 | 1 | 1 |
| Path 614 | C00031->C00407:[2->3] | 0.17 | 404.392857143 | 17 | 56 | 1 | 2 |
| Path 615 | C00031->C00407:[1->2,4->6,7->9,9->5] | 0.67 | 476.122807018 | 22 | 57 | 0 | 0 |
| Path 616 | C00031->C00407:[4->3,4->5,7->6,7->9,9->2] | 0.83 | 420.956521739 | 35 | 92 | 1 | 1 |
| Path 617 | C00031->C00407:[7->6,7->9,9->2,9->5] | 0.67 | 387.03125 | 16 | 32 | 0 | 0 |
| Path 618 | C00031->C00407:[7->6,7->9,9->2,9->5] | 0.67 | 376.96969697 | 17 | 33 | 0 | 0 |
| Path 619 | C00031->C00407:[7->6,9->2] | 0.33 | 356.203389831 | 22 | 59 | 0 | 0 |
| Path 620 | C00031->C00407:[1->2,1->5,4->6,4->9] | 0.67 | 496.527272727 | 21 | 55 | 0 | 0 |
| Path 621 | C00031->C00407:[4->3,7->6,7->9,9->2,9->5] | 0.83 | 469.48 | 28 | 50 | 1 | 1 |
| Path 622 | C00031->C00407:[4->3,7->9,9->5] | 0.50 | 413.565217391 | 24 | 46 | 1 | 1 |
| Path 623 | C00031->C00407:[4->3,7->6,7->9,9->2,9->5] | 0.83 | 441.615384615 | 28 | 52 | 1 | 1 |
| Path 624 | C00031->C00407:[1->2,1->5,4->1,4->3,4->6,4->9] | 1.00 | 500.325301205 | 39 | 83 | 1 | 1 |
| Path 625 | C00031->C00407:[4->1,4->3,7->6,7->9,9->2,9->5] | 1.00 | 459.819672131 | 35 | 61 | 1 | 1 |
| Path 626 | C00031->C00407:[2->3,7->9,9->5] | 0.50 | 338.068965517 | 23 | 87 | 1 | 2 |
| Path 627 | C00031->C00407:[7->6,7->9,9->2,9->5] | 0.67 | 495.596491228 | 23 | 57 | 0 | 0 |
| Path 628 | C00031->C00407:[4->3,7->6,7->9,9->2,9->5] | 0.83 | 460.097560976 | 24 | 41 | 1 | 1 |
| Path 629 | C00031->C00407:[1->2,4->1,4->3,4->6,7->9,9->5] | 1.00 | 465.280487805 | 34 | 82 | 1 | 1 |
| Path 630 | C00031->C00407:[1->2,4->1,4->3,4->6,7->9,9->5] | 1.00 | 474.395061728 | 36 | 81 | 1 | 1 |
