# Supplementary material for: 13C-metabolic flux ratio and novel carbon path analyses confirmed that Trichoderma reesei uses primarily the respirative pathway also on the preferred carbon source glucose
Source: BMC Syst Biol. 2009 Oct 29;3:104. doi: 10.1186/1752-0509-3-104 (PMC2776023; doi:10.1186/1752-0509-3-104)
Supplement: Additional file 1 — Pathways discovered in ReTrace carbon path analysis. Graphical and tabular representations of amino acid synthesis pathways discovered in ReTrace carbon path analysis [21]. Self-contained web site: unpack zip archive and open index.html with a web browser. [file 1752-0509-3-104-S1.zip › AF1-treesei/pathways-C00279-C00074-to-C00082.html]

Pathways from C00279,C00074 to C00082


**Pathways from C00279-C00074 to C00082**

**Sources:** D-Erythrose 4-phosphate (C00279)
Phosphoenolpyruvate; (C00074)

**Target:**L-Tyrosine; (C00082)

|  | Composite mapping | Z | Average score | Rpairs | Reactions | Zero scores | Scores under threshold |
| --- | --- | --- | --- | --- | --- | --- | --- |
| Path 1 | C00279->C00082:[10->10,6->9,7->11,8->12], C00074->C00082:[6->1,6->7,7->2,8->3,8->8] | 1.00 | 536.711111111 | 20 | 45 | 0 | 0 |
| Path 2 | C00279->C00082:[10->10,6->2,6->9,7->11,8->12], C00074->C00082:[6->1,6->7,8->3,8->8] | 1.00 | 654.457142857 | 24 | 35 | 0 | 0 |
| Path 3 | C00279->C00082:[10->10,6->9,7->11,8->12], C00074->C00082:[6->1,6->7,7->2,8->3,8->8] | 1.00 | 594.673469388 | 19 | 49 | 0 | 0 |
| Path 4 | C00279->C00082:[10->10,6->9,7->11,8->12], C00074->C00082:[6->1,6->7,7->2,8->3,8->8] | 1.00 | 564.075 | 19 | 40 | 0 | 0 |
| Path 5 | C00279->C00082:[10->10,6->9,7->11,8->12], C00074->C00082:[6->1,6->2,6->7,8->3,8->8] | 1.00 | 489.698412698 | 23 | 63 | 0 | 0 |
| Path 6 | C00279->C00082:[10->10,6->2,6->9,7->11,8->12], C00074->C00082:[6->1,6->7,8->3,8->8] | 1.00 | 647.617647059 | 23 | 34 | 0 | 0 |
| Path 7 | C00279->C00082:[10->10,6->9,7->11,8->12], C00074->C00082:[6->1,6->7,7->2,8->3,8->8] | 1.00 | 602.457142857 | 21 | 35 | 0 | 0 |
| Path 8 | C00279->C00082:[10->10,6->9,7->11,8->12], C00074->C00082:[6->1,6->2,6->7,8->3,8->8] | 1.00 | 516.363636364 | 22 | 55 | 0 | 0 |
| Path 9 | C00279->C00082:[10->10,6->9,7->11,8->12], C00074->C00082:[6->1,6->2,6->7,8->3,8->8] | 1.00 | 607.4 | 21 | 45 | 0 | 0 |
| Path 10 | C00279->C00082:[10->10,6->9,7->11,8->12], C00074->C00082:[6->1,6->7,7->2,8->3,8->8] | 1.00 | 564.066666667 | 25 | 60 | 0 | 0 |
| Path 11 | C00279->C00082:[10->10,6->9,7->11,8->12], C00074->C00082:[6->1,6->2,6->7,8->3,8->8] | 1.00 | 545.024390244 | 22 | 41 | 0 | 0 |
| Path 12 | C00279->C00082:[10->10,6->2,6->9,7->11,8->12], C00074->C00082:[6->1,6->7,8->3,8->8] | 1.00 | 566.80952381 | 24 | 42 | 0 | 0 |
| Path 13 | C00279->C00082:[10->10,6->9,7->11,8->12], C00074->C00082:[6->1,6->7,7->2,8->3,8->8] | 1.00 | 637.975 | 20 | 40 | 0 | 0 |
| Path 14 | C00074->C00082:[7->1,7->2,7->3] | 0.33 | 472.054794521 | 28 | 73 | 0 | 0 |
| Path 15 | C00279->C00082:[6->1,6->2], C00074->C00082:[6->1,6->2] | 0.22 | 427.138461538 | 23 | 65 | 0 | 0 |
| Path 16 | C00074->C00082:[7->1,7->2,7->3] | 0.33 | 495.52238806 | 27 | 67 | 0 | 0 |
| Path 17 | C00074->C00082:[7->1,7->2] | 0.22 | 386.8 | 19 | 75 | 0 | 0 |
| Path 18 | C00074->C00082:[7->1,7->2] | 0.22 | 383.924242424 | 17 | 66 | 0 | 0 |
| Path 19 | C00074->C00082:[7->1,7->2,7->3] | 0.33 | 496.964285714 | 23 | 56 | 0 | 0 |
| Path 20 | C00074->C00082:[7->1,7->2,7->3] | 0.33 | 445.264150943 | 19 | 53 | 0 | 0 |
| Path 21 | C00074->C00082:[7->1,7->2,7->3] | 0.33 | 391.523809524 | 20 | 63 | 0 | 0 |
| Path 22 | C00074->C00082:[7->1,7->2] | 0.22 | 475.137254902 | 16 | 51 | 0 | 0 |
| Path 23 | C00074->C00082:[7->1,7->2] | 0.22 | 396.402985075 | 18 | 67 | 0 | 0 |
| Path 24 | C00074->C00082:[7->1,7->2,7->3] | 0.33 | 482.203703704 | 21 | 54 | 0 | 0 |
| Path 25 | C00074->C00082:[7->1,7->2] | 0.22 | 482.316666667 | 23 | 60 | 0 | 0 |
| Path 26 | C00074->C00082:[7->1,7->2] | 0.22 | 490.761904762 | 17 | 42 | 0 | 0 |
| Path 27 | C00074->C00082:[6->1,6->2,7->1,7->2] | 0.22 | 466.692307692 | 16 | 39 | 0 | 0 |
| Path 28 | C00074->C00082:[7->1,7->2] | 0.22 | 448.191489362 | 20 | 47 | 0 | 0 |
| Path 29 | C00074->C00082:[6->1,7->2,8->3] | 0.33 | 465.826086957 | 14 | 46 | 0 | 2 |
| Path 30 | C00279->C00082:[6->1,6->2] | 0.22 | 500.592592593 | 18 | 27 | 0 | 0 |
| Path 31 | C00074->C00082:[7->1,7->2,7->3] | 0.33 | 458.432835821 | 26 | 67 | 0 | 0 |
| Path 32 | C00074->C00082:[6->1,6->2,7->1,7->2] | 0.22 | 412.157894737 | 19 | 57 | 0 | 0 |
| Path 33 | C00074->C00082:[7->1,7->2] | 0.22 | 495.12244898 | 17 | 49 | 0 | 0 |
| Path 34 | C00074->C00082:[7->1,7->2] | 0.22 | 453.966666667 | 20 | 60 | 0 | 0 |
| Path 35 | C00074->C00082:[7->2] | 0.11 | 423.0 | 7 | 25 | 0 | 0 |
| Path 36 | C00279->C00082:[6->1,6->2] | 0.22 | 469.68 | 16 | 25 | 0 | 0 |
| Path 37 | C00074->C00082:[7->1,7->2,7->3] | 0.33 | 424.232876712 | 26 | 73 | 0 | 0 |
| Path 38 | C00279->C00082:[6->1,6->2], C00074->C00082:[6->1,6->2] | 0.22 | 436.178571429 | 21 | 56 | 0 | 0 |
| Path 39 | C00074->C00082:[7->1,7->2,7->3] | 0.33 | 452.651515152 | 25 | 66 | 0 | 0 |
| Path 40 | C00074->C00082:[7->1,7->2] | 0.22 | 406.759259259 | 20 | 54 | 0 | 0 |
| Path 41 | C00074->C00082:[7->1,7->2,7->3] | 0.33 | 393.569444444 | 22 | 72 | 0 | 0 |
| Path 42 | C00074->C00082:[6->2] | 0.11 | 470.464285714 | 7 | 28 | 0 | 0 |
| Path 43 | C00074->C00082:[7->1,7->2,7->3] | 0.33 | 511.426229508 | 25 | 61 | 0 | 0 |
| Path 44 | C00074->C00082:[7->1,7->2] | 0.22 | 466.725806452 | 22 | 62 | 0 | 0 |
| Path 45 | C00074->C00082:[7->1,7->2] | 0.22 | 381.035087719 | 18 | 57 | 0 | 0 |
| Path 46 | C00074->C00082:[7->1,7->2,7->3] | 0.33 | 450.013888889 | 25 | 72 | 0 | 0 |
| Path 47 | C00279->C00082:[6->1,6->2], C00074->C00082:[6->1,6->2] | 0.22 | 419.953125 | 22 | 64 | 0 | 0 |
| Path 48 | C00074->C00082:[7->1,7->2,7->3] | 0.33 | 460.810810811 | 27 | 74 | 0 | 0 |
| Path 49 | C00074->C00082:[7->1,7->2] | 0.22 | 447.228070175 | 17 | 57 | 0 | 0 |
| Path 50 | C00074->C00082:[7->1,7->2,7->3] | 0.33 | 460.254237288 | 22 | 59 | 0 | 0 |
| Path 51 | C00074->C00082:[7->1,7->2,7->3] | 0.33 | 488.117647059 | 19 | 51 | 0 | 0 |
| Path 52 | C00279->C00082:[6->2] | 0.11 | 485.888888889 | 10 | 18 | 0 | 0 |
| Path 53 | C00074->C00082:[6->1,6->2,8->3] | 0.33 | 385.666666667 | 18 | 60 | 0 | 2 |
| Path 54 | C00074->C00082:[7->1,7->2] | 0.22 | 454.604166667 | 15 | 48 | 0 | 0 |
| Path 55 | C00074->C00082:[7->1,7->2] | 0.22 | 471.55 | 12 | 40 | 0 | 0 |
| Path 56 | C00074->C00082:[7->1,7->2,7->3] | 0.33 | 463.632352941 | 24 | 68 | 0 | 0 |
| Path 57 | C00074->C00082:[6->1,6->2] | 0.22 | 444.087719298 | 22 | 57 | 0 | 0 |
| Path 58 | C00074->C00082:[7->1,7->2,7->3] | 0.33 | 466.291666667 | 27 | 72 | 0 | 0 |
| Path 59 | C00074->C00082:[7->1,7->2] | 0.22 | 450.636363636 | 18 | 44 | 0 | 0 |
| Path 60 | C00279->C00082:[6->1,6->2] | 0.22 | 485.730769231 | 17 | 26 | 0 | 0 |
| Path 61 | C00074->C00082:[7->1,7->2,7->3] | 0.33 | 453.854166667 | 18 | 48 | 0 | 0 |
| Path 62 | C00279->C00082:[6->2], C00074->C00082:[6->1,8->3] | 0.33 | 476.019230769 | 20 | 52 | 0 | 2 |
| Path 63 | C00279->C00082:[6->2] | 0.11 | 385.84 | 10 | 25 | 0 | 0 |
| Path 64 | C00074->C00082:[7->1,7->2,7->3] | 0.33 | 476.133333333 | 21 | 60 | 0 | 0 |
| Path 65 | C00074->C00082:[7->1,7->2] | 0.22 | 503.3 | 18 | 50 | 0 | 0 |
| Path 66 | C00074->C00082:[7->1,7->2,7->3] | 0.33 | 391.075757576 | 22 | 66 | 0 | 0 |
| Path 67 | C00074->C00082:[7->1,7->2] | 0.22 | 429.522727273 | 21 | 44 | 0 | 0 |
| Path 68 | C00074->C00082:[6->1,6->2,7->1,7->2] | 0.22 | 429.631578947 | 18 | 38 | 0 | 0 |
| Path 69 | C00074->C00082:[7->1,7->2] | 0.22 | 394.620689655 | 16 | 58 | 0 | 0 |
| Path 70 | C00074->C00082:[7->1,7->2] | 0.22 | 415.730769231 | 14 | 26 | 0 | 0 |
| Path 71 | C00074->C00082:[7->1,7->2] | 0.22 | 502.52173913 | 15 | 46 | 0 | 0 |
| Path 72 | C00074->C00082:[7->1,7->2,7->3] | 0.33 | 452.589285714 | 22 | 56 | 0 | 0 |
| Path 73 | C00074->C00082:[7->1,7->2,7->3] | 0.33 | 488.129032258 | 23 | 62 | 0 | 0 |
| Path 74 | C00074->C00082:[7->1,7->2,7->3] | 0.33 | 452.640625 | 23 | 64 | 0 | 0 |
| Path 75 | C00074->C00082:[7->1,7->2] | 0.22 | 462.098039216 | 18 | 51 | 0 | 0 |
| Path 76 | C00074->C00082:[7->1,7->2,7->3] | 0.33 | 446.4 | 24 | 65 | 0 | 0 |
| Path 77 | C00074->C00082:[7->1,7->2] | 0.22 | 476.163265306 | 20 | 49 | 0 | 0 |
| Path 78 | C00074->C00082:[6->2] | 0.11 | 341.083333333 | 8 | 24 | 0 | 0 |
| Path 79 | C00074->C00082:[7->1,7->2] | 0.22 | 385.416666667 | 13 | 36 | 0 | 0 |
| Path 80 | C00074->C00082:[7->1,7->2,7->3] | 0.33 | 481.078125 | 26 | 64 | 0 | 0 |
| Path 81 | C00074->C00082:[7->1,7->2] | 0.22 | 525.487804878 | 19 | 41 | 0 | 0 |
| Path 82 | C00074->C00082:[7->2] | 0.11 | 384.777777778 | 7 | 18 | 0 | 0 |
| Path 83 | C00074->C00082:[7->1,7->2,7->3] | 0.33 | 418.37804878 | 28 | 82 | 0 | 0 |
| Path 84 | C00074->C00082:[7->1,7->2] | 0.22 | 438.652173913 | 19 | 46 | 0 | 0 |
| Path 85 | C00074->C00082:[7->1,7->2,7->3] | 0.33 | 439.838709677 | 21 | 62 | 0 | 0 |
| Path 86 | C00074->C00082:[7->1,7->2] | 0.22 | 488.950819672 | 24 | 61 | 0 | 0 |
| Path 87 | C00074->C00082:[7->1,7->2,7->3] | 0.33 | 458.666666667 | 16 | 45 | 0 | 0 |
| Path 88 | C00074->C00082:[7->1,7->2,7->3] | 0.33 | 491.101694915 | 25 | 59 | 0 | 0 |
| Path 89 | C00074->C00082:[7->1,7->2,7->3] | 0.33 | 503.807017544 | 24 | 57 | 0 | 0 |
| Path 90 | C00074->C00082:[7->1,7->2,7->3] | 0.33 | 517.483870968 | 26 | 62 | 0 | 0 |
| Path 91 | C00074->C00082:[6->1,6->2,7->1,7->2] | 0.22 | 455.666666667 | 18 | 48 | 0 | 0 |
| Path 92 | C00074->C00082:[7->1,7->2,7->3] | 0.33 | 412.592592593 | 27 | 81 | 0 | 0 |
| Path 93 | C00074->C00082:[6->1,7->2,8->3] | 0.33 | 481.297297297 | 15 | 37 | 0 | 2 |
| Path 94 | C00074->C00082:[6->1,7->2,8->3] | 0.33 | 489.622222222 | 17 | 45 | 0 | 2 |
| Path 95 | C00074->C00082:[6->1,6->2,7->1,7->2] | 0.22 | 477.14893617 | 18 | 47 | 0 | 0 |
| Path 96 | C00074->C00082:[7->1,7->2,7->3] | 0.33 | 464.464285714 | 20 | 56 | 0 | 0 |
| Path 97 | C00279->C00082:[6->2], C00074->C00082:[6->1,8->3] | 0.33 | 530.066666667 | 20 | 45 | 0 | 2 |
| Path 98 | C00074->C00082:[6->1,6->2,8->3] | 0.33 | 456.725490196 | 18 | 51 | 0 | 2 |
| Path 99 | C00074->C00082:[7->1,7->2,7->3] | 0.33 | 430.486486486 | 27 | 74 | 0 | 0 |
| Path 100 | C00074->C00082:[7->2] | 0.11 | 356.857142857 | 6 | 28 | 0 | 0 |
| Path 101 | C00074->C00082:[7->1,7->2,7->3] | 0.33 | 396.182926829 | 25 | 82 | 0 | 0 |
| Path 102 | C00074->C00082:[6->1,6->2,8->3] | 0.33 | 397.865384615 | 17 | 52 | 0 | 2 |
| Path 103 | C00074->C00082:[7->1,7->2,7->3] | 0.33 | 450.203703704 | 18 | 54 | 0 | 0 |
| Path 104 | C00074->C00082:[7->1,7->2] | 0.22 | 510.823529412 | 19 | 51 | 0 | 0 |
| Path 105 | C00074->C00082:[7->1,7->2] | 0.22 | 475.333333333 | 20 | 36 | 0 | 0 |
| Path 106 | C00074->C00082:[7->1,7->2,7->3] | 0.33 | 381.929577465 | 21 | 71 | 0 | 0 |
| Path 107 | C00074->C00082:[7->1,7->2] | 0.22 | 482.813953488 | 20 | 43 | 0 | 0 |
| Path 108 | C00074->C00082:[6->1,6->2] | 0.22 | 507.804347826 | 20 | 46 | 0 | 0 |
| Path 109 | C00074->C00082:[7->1,7->2] | 0.22 | 486.459459459 | 21 | 37 | 0 | 0 |
| Path 110 | C00074->C00082:[7->1,7->2,7->3] | 0.33 | 478.34375 | 24 | 64 | 0 | 0 |
| Path 111 | C00074->C00082:[6->2] | 0.11 | 362.847826087 | 9 | 46 | 0 | 0 |
| Path 112 | C00074->C00082:[7->1,7->2,7->3] | 0.33 | 384.85 | 23 | 80 | 0 | 0 |
| Path 113 | C00074->C00082:[7->1,7->2,7->3] | 0.33 | 484.630769231 | 25 | 65 | 0 | 0 |
| Path 114 | C00074->C00082:[7->1,7->2,7->3] | 0.33 | 472.569230769 | 27 | 65 | 0 | 0 |
| Path 115 | C00074->C00082:[7->1,7->2] | 0.22 | 464.689655172 | 19 | 58 | 0 | 0 |
| Path 116 | C00074->C00082:[7->1,7->2,7->3] | 0.33 | 441.857142857 | 21 | 56 | 0 | 0 |
| Path 117 | C00074->C00082:[7->1,7->2,7->3] | 0.33 | 476.083333333 | 21 | 60 | 0 | 0 |
| Path 118 | C00279->C00082:[6->2] | 0.11 | 462.294117647 | 9 | 17 | 0 | 0 |
| Path 119 | C00074->C00082:[7->1,7->2,7->3] | 0.33 | 448.983050847 | 24 | 59 | 0 | 0 |
| Path 120 | C00279->C00082:[6->1,6->2], C00074->C00082:[6->1,6->2] | 0.22 | 437.428571429 | 21 | 42 | 0 | 0 |
| Path 121 | C00074->C00082:[7->1,7->2] | 0.22 | 399.138461538 | 20 | 65 | 0 | 0 |
| Path 122 | C00074->C00082:[6->1,7->2,8->3] | 0.33 | 458.473684211 | 20 | 57 | 0 | 2 |
| Path 123 | C00074->C00082:[7->1,7->2] | 0.22 | 431.714285714 | 16 | 42 | 0 | 0 |
| Path 124 | C00074->C00082:[7->1,7->2,7->3] | 0.33 | 405.891891892 | 24 | 74 | 0 | 0 |
| Path 125 | C00074->C00082:[7->2] | 0.11 | 468.09375 | 5 | 32 | 0 | 0 |
| Path 126 | C00279->C00082:[6->1,6->2] | 0.22 | 460.102564103 | 21 | 39 | 0 | 0 |
| Path 127 | C00074->C00082:[6->1,6->2] | 0.22 | 447.88372093 | 22 | 43 | 0 | 0 |
| Path 128 | C00074->C00082:[7->1,7->2,7->3] | 0.33 | 437.153846154 | 23 | 65 | 0 | 0 |
| Path 129 | C00074->C00082:[6->2] | 0.11 | 374.736842105 | 8 | 38 | 0 | 0 |
| Path 130 | C00074->C00082:[6->1,7->2,8->3] | 0.33 | 456.345454545 | 16 | 55 | 0 | 2 |
| Path 131 | C00279->C00082:[6->2], C00074->C00082:[6->1,8->3] | 0.33 | 521.954545455 | 19 | 44 | 0 | 2 |
| Path 132 | C00074->C00082:[7->1,7->2] | 0.22 | 457.62962963 | 20 | 54 | 0 | 0 |
| Path 133 | C00074->C00082:[7->2] | 0.11 | 493.869565217 | 6 | 23 | 0 | 0 |
| Path 134 | C00074->C00082:[7->1,7->2] | 0.22 | 431.642857143 | 16 | 42 | 0 | 0 |
| Path 135 | C00074->C00082:[6->1,6->2] | 0.22 | 515.872340426 | 21 | 47 | 0 | 0 |
| Path 136 | C00074->C00082:[7->1,7->2] | 0.22 | 534.095238095 | 20 | 42 | 0 | 0 |
| Path 137 | C00074->C00082:[7->1,7->2,7->3] | 0.33 | 489.590909091 | 26 | 66 | 0 | 0 |
| Path 138 | C00074->C00082:[7->1,7->2] | 0.22 | 492.0 | 21 | 44 | 0 | 0 |
| Path 139 | C00074->C00082:[7->1,7->2,7->3] | 0.33 | 489.872727273 | 22 | 55 | 0 | 0 |
| Path 140 | C00074->C00082:[7->1,7->2,7->3] | 0.33 | 484.275862069 | 24 | 58 | 0 | 0 |
| Path 141 | C00074->C00082:[7->1,7->2,7->3] | 0.33 | 486.171428571 | 28 | 70 | 0 | 0 |
| Path 142 | C00074->C00082:[7->1,7->2] | 0.22 | 459.857142857 | 14 | 49 | 0 | 0 |
| Path 143 | C00074->C00082:[7->1,7->2,7->3] | 0.33 | 474.634920635 | 25 | 63 | 0 | 0 |
| Path 144 | C00074->C00082:[7->1,7->2] | 0.22 | 465.279069767 | 14 | 43 | 0 | 0 |
| Path 145 | C00074->C00082:[7->1,7->2] | 0.22 | 382.590909091 | 16 | 44 | 0 | 0 |
| Path 146 | C00074->C00082:[7->2] | 0.11 | 457.76744186 | 11 | 43 | 0 | 0 |
| Path 147 | C00074->C00082:[7->1,7->2,7->3] | 0.33 | 480.362318841 | 27 | 69 | 0 | 0 |
| Path 148 | C00074->C00082:[6->1,6->2,8->3] | 0.33 | 467.19047619 | 16 | 42 | 0 | 2 |
| Path 149 | C00074->C00082:[7->1,7->2] | 0.22 | 491.677419355 | 13 | 31 | 0 | 0 |
| Path 150 | C00279->C00082:[6->1,6->2] | 0.22 | 470.775 | 22 | 40 | 0 | 0 |
| Path 151 | C00074->C00082:[7->1,7->2,7->3] | 0.33 | 456.031746032 | 23 | 63 | 0 | 0 |
| Path 152 | C00074->C00082:[7->1,7->2,7->3] | 0.33 | 446.596491228 | 20 | 57 | 0 | 0 |
| Path 153 | C00074->C00082:[7->1,7->2,7->3] | 0.33 | 381.918918919 | 23 | 74 | 0 | 0 |
| Path 154 | C00074->C00082:[7->1,7->2] | 0.22 | 455.346153846 | 16 | 52 | 0 | 0 |
| Path 155 | C00074->C00082:[7->1,7->2] | 0.22 | 393.224489796 | 17 | 49 | 0 | 0 |
| Path 156 | C00074->C00082:[7->1,7->2,7->3] | 0.33 | 478.848484848 | 28 | 66 | 0 | 0 |
