# Supplementary material for: 13C-metabolic flux ratio and novel carbon path analyses confirmed that Trichoderma reesei uses primarily the respirative pathway also on the preferred carbon source glucose
Source: BMC Syst Biol. 2009 Oct 29;3:104. doi: 10.1186/1752-0509-3-104 (PMC2776023; doi:10.1186/1752-0509-3-104)
Supplement: Additional file 1 — Pathways discovered in ReTrace carbon path analysis. Graphical and tabular representations of amino acid synthesis pathways discovered in ReTrace carbon path analysis [21]. Self-contained web site: unpack zip archive and open index.html with a web browser. [file 1752-0509-3-104-S1.zip › AF1-treesei/pathways-C00022-C00024-to-C00123.html]

Pathways from C00022,C00024 to C00123


**Pathways from C00022-C00024 to C00123**

**Sources:** Pyruvate; (C00022)
Acetyl-CoA; (C00024)

**Target:**L-Leucine; (C00123)

|  | Composite mapping | Z | Average score | Rpairs | Reactions | Zero scores | Scores under threshold |
| --- | --- | --- | --- | --- | --- | --- | --- |
| Path 1 | C00024->C00123:[49->5,50->8,50->9], C00022->C00123:[2->3] | 0.67 | 369.888888889 | 11 | 27 | 0 | 0 |
| Path 2 | C00024->C00123:[49->1,49->5,50->2,50->8,50->9], C00022->C00123:[2->3] | 1.00 | 352.333333333 | 13 | 33 | 0 | 0 |
| Path 3 | C00024->C00123:[49->1,49->5,50->2,50->9], C00022->C00123:[1->1,1->5,2->3,3->2,3->9] | 0.83 | 424.890909091 | 23 | 55 | 0 | 0 |
| Path 4 | C00024->C00123:[49->1,49->3,50->2,50->8] | 0.67 | 295.813953488 | 17 | 43 | 0 | 0 |
| Path 5 | C00024->C00123:[49->1,50->2,50->8], C00022->C00123:[1->1,2->3,3->2,3->8] | 0.67 | 378.378378378 | 15 | 37 | 0 | 0 |
| Path 6 | C00024->C00123:[49->3,49->5,50->8,50->9], C00022->C00123:[1->3,1->5,3->8,3->9] | 0.67 | 355.695652174 | 13 | 23 | 0 | 0 |
| Path 7 | C00024->C00123:[49->1,50->2,50->8], C00022->C00123:[1->1,2->3,3->2,3->8] | 0.67 | 418.770833333 | 19 | 48 | 0 | 0 |
| Path 8 | C00024->C00123:[49->1,49->3,50->2,50->8], C00022->C00123:[1->1,1->3,3->2,3->8] | 0.67 | 280.242424242 | 14 | 33 | 0 | 0 |
| Path 9 | C00024->C00123:[49->1,49->5,50->2,50->9], C00022->C00123:[2->3] | 0.83 | 381.358974359 | 17 | 39 | 0 | 0 |
| Path 10 | C00024->C00123:[49->5,50->8,50->9], C00022->C00123:[1->5,2->3,3->8,3->9] | 0.67 | 462.375 | 20 | 48 | 0 | 0 |
| Path 11 | C00024->C00123:[49->3,49->5,50->8,50->9], C00022->C00123:[1->3,1->5,3->8,3->9] | 0.67 | 277.70212766 | 17 | 47 | 0 | 0 |
| Path 12 | C00024->C00123:[49->1,49->3,49->5,50->2,50->9] | 0.83 | 271.52173913 | 16 | 46 | 0 | 0 |
| Path 13 | C00024->C00123:[49->1,50->2,50->8], C00022->C00123:[2->3] | 0.67 | 288.88 | 14 | 50 | 0 | 0 |
| Path 14 | C00024->C00123:[49->1,49->5,50->2,50->8,50->9], C00022->C00123:[2->3] | 1.00 | 468.354166667 | 20 | 48 | 0 | 0 |
| Path 15 | C00024->C00123:[49->5,50->8,50->9], C00022->C00123:[1->5,2->3,3->8,3->9] | 0.67 | 456.021276596 | 20 | 47 | 0 | 0 |
| Path 16 | C00024->C00123:[49->1,49->3,50->2,50->8], C00022->C00123:[1->1,1->3,3->2,3->8] | 0.67 | 274.739130435 | 16 | 46 | 0 | 0 |
| Path 17 | C00024->C00123:[49->5,50->8,50->9], C00022->C00123:[2->3] | 0.67 | 288.88 | 14 | 50 | 0 | 0 |
| Path 18 | C00024->C00123:[49->1,50->2,50->8], C00022->C00123:[2->3] | 0.67 | 347.029411765 | 14 | 34 | 0 | 0 |
| Path 19 | C00024->C00123:[49->1,50->2,50->8], C00022->C00123:[2->3] | 0.67 | 373.486486486 | 16 | 37 | 0 | 0 |
| Path 20 | C00024->C00123:[49->3,49->5,50->8,50->9] | 0.67 | 295.813953488 | 17 | 43 | 0 | 0 |
| Path 21 | C00024->C00123:[49->1,49->5,50->2,50->9], C00022->C00123:[2->3] | 0.83 | 374.925 | 17 | 40 | 0 | 0 |
| Path 22 | C00024->C00123:[49->5,50->8,50->9], C00022->C00123:[2->3] | 0.67 | 408.15625 | 14 | 32 | 0 | 0 |
| Path 23 | C00024->C00123:[49->1,49->5,50->2,50->9], C00022->C00123:[1->1,1->5,2->3,3->2,3->9] | 0.83 | 269.566666667 | 16 | 60 | 0 | 0 |
| Path 24 | C00024->C00123:[49->1,50->2,50->8], C00022->C00123:[1->1,2->3,3->2,3->8] | 0.67 | 316.675675676 | 13 | 37 | 0 | 0 |
| Path 25 | C00024->C00123:[49->1,49->5,50->2,50->9], C00022->C00123:[2->3] | 0.83 | 460.47826087 | 20 | 46 | 0 | 0 |
| Path 26 | C00024->C00123:[49->1,49->5,50->2,50->9], C00022->C00123:[1->1,1->5,2->3,3->2,3->9] | 0.83 | 418.770833333 | 19 | 48 | 0 | 0 |
| Path 27 | C00024->C00123:[49->1,49->3,49->5,50->2,50->9] | 0.83 | 349.260869565 | 13 | 23 | 0 | 0 |
| Path 28 | C00024->C00123:[49->1,49->5,50->2,50->9], C00022->C00123:[2->3] | 0.83 | 367.742857143 | 23 | 70 | 0 | 0 |
| Path 29 | C00024->C00123:[49->1,49->5,50->2,50->9], C00022->C00123:[2->3] | 0.83 | 347.029411765 | 14 | 34 | 0 | 0 |
| Path 30 | C00024->C00123:[49->1,50->2,50->8], C00022->C00123:[1->1,3->2,3->3,3->8] | 0.67 | 496.913043478 | 24 | 46 | 0 | 0 |
| Path 31 | C00024->C00123:[49->1,50->2,50->8], C00022->C00123:[1->1,2->3,3->2,3->8] | 0.67 | 463.404255319 | 19 | 47 | 0 | 0 |
| Path 32 | C00024->C00123:[49->1,49->5,50->2,50->9], C00022->C00123:[1->1,1->5,2->3,3->2,3->9] | 0.83 | 427.384615385 | 21 | 52 | 0 | 0 |
| Path 33 | C00024->C00123:[49->3,49->5,50->8,50->9], C00022->C00123:[1->3,1->5,3->8,3->9] | 0.67 | 244.732142857 | 17 | 56 | 0 | 0 |
| Path 34 | C00024->C00123:[49->1,50->2,50->8], C00022->C00123:[1->1,3->2,3->3,3->8] | 0.67 | 400.384615385 | 16 | 26 | 0 | 0 |
| Path 35 | C00024->C00123:[49->1,50->2,50->8], C00022->C00123:[1->1,3->2,3->3,3->8] | 0.67 | 428.620689655 | 18 | 29 | 0 | 0 |
| Path 36 | C00024->C00123:[49->1,50->2,50->8], C00022->C00123:[2->3] | 0.67 | 297.5 | 17 | 58 | 0 | 0 |
| Path 37 | C00024->C00123:[49->1,49->5,50->2,50->9], C00022->C00123:[2->3] | 0.83 | 336.8125 | 12 | 32 | 0 | 0 |
| Path 38 | C00024->C00123:[49->1,50->2,50->8], C00022->C00123:[1->1,2->3,3->2,3->8] | 0.67 | 279.690909091 | 15 | 55 | 0 | 0 |
| Path 39 | C00024->C00123:[49->1,50->2,50->8], C00022->C00123:[1->1,2->3,3->2,3->8] | 0.67 | 269.566666667 | 16 | 60 | 0 | 0 |
| Path 40 | C00024->C00123:[49->5,50->8,50->9], C00022->C00123:[2->3] | 0.67 | 310.641509434 | 16 | 53 | 0 | 0 |
| Path 41 | C00024->C00123:[49->1,49->5,50->2,50->9], C00022->C00123:[2->3] | 0.83 | 445.627906977 | 18 | 43 | 0 | 0 |
| Path 42 | C00024->C00123:[49->5,50->8,50->9], C00022->C00123:[1->5,2->3,3->8,3->9] | 0.67 | 378.378378378 | 15 | 37 | 0 | 0 |
| Path 43 | C00024->C00123:[49->1,50->2,50->8], C00022->C00123:[2->3] | 0.67 | 277.0 | 15 | 55 | 0 | 0 |
| Path 44 | C00024->C00123:[49->5,50->8,50->9], C00022->C00123:[2->3] | 0.67 | 496.214285714 | 18 | 42 | 0 | 0 |
| Path 45 | C00024->C00123:[49->5,50->8,50->9], C00022->C00123:[2->3] | 0.67 | 389.2 | 21 | 65 | 0 | 0 |
| Path 46 | C00024->C00123:[49->1,50->2,50->8], C00022->C00123:[1->1,2->3,3->2,3->8] | 0.67 | 343.425 | 15 | 40 | 0 | 0 |
| Path 47 | C00024->C00123:[49->1,49->3,50->2,50->8] | 0.67 | 285.341463415 | 15 | 41 | 0 | 0 |
| Path 48 | C00024->C00123:[49->1,49->5,50->2,50->9], C00022->C00123:[1->1,1->5,2->3,3->2,3->9] | 0.83 | 352.166666667 | 16 | 42 | 0 | 0 |
| Path 49 | C00024->C00123:[49->5,50->8,50->9], C00022->C00123:[2->3] | 0.67 | 400.233333333 | 13 | 30 | 0 | 0 |
| Path 50 | C00024->C00123:[49->1,50->2,50->8], C00022->C00123:[2->3] | 0.67 | 365.657142857 | 14 | 35 | 0 | 0 |
| Path 51 | C00024->C00123:[49->1,50->2,50->8], C00022->C00123:[1->1,2->3,3->2,3->8] | 0.67 | 341.4375 | 12 | 32 | 0 | 0 |
| Path 52 | C00024->C00123:[49->1,50->2,50->8], C00022->C00123:[1->1,2->3,3->2,3->8] | 0.67 | 198.206185567 | 18 | 97 | 0 | 0 |
| Path 53 | C00024->C00123:[49->3,49->5,50->8,50->9], C00022->C00123:[1->3,1->5,3->8,3->9] | 0.67 | 368.08 | 15 | 25 | 0 | 0 |
| Path 54 | C00024->C00123:[49->1,50->2,50->8], C00022->C00123:[2->3] | 0.67 | 310.641509434 | 16 | 53 | 0 | 0 |
| Path 55 | C00024->C00123:[49->5,50->8,50->9], C00022->C00123:[1->5,2->3,3->8,3->9] | 0.67 | 279.690909091 | 15 | 55 | 0 | 0 |
| Path 56 | C00024->C00123:[49->1,50->2,50->8], C00022->C00123:[1->1,3->2,3->3,3->8] | 0.67 | 518.634146341 | 23 | 41 | 0 | 0 |
| Path 57 | C00024->C00123:[49->1,49->3,49->5,50->2,50->9], C00022->C00123:[1->1,1->3,1->5,3->2,3->9] | 0.83 | 320.428571429 | 14 | 28 | 0 | 0 |
| Path 58 | C00024->C00123:[49->3,49->5,50->8,50->9], C00022->C00123:[1->3,1->5,3->8,3->9] | 0.67 | 369.846153846 | 16 | 26 | 0 | 0 |
| Path 59 | C00024->C00123:[49->1,49->5,50->2,50->9], C00022->C00123:[2->3] | 0.83 | 390.955223881 | 18 | 67 | 0 | 0 |
| Path 60 | C00024->C00123:[49->5,50->8,50->9], C00022->C00123:[1->5,2->3,3->8,3->9] | 0.67 | 341.4375 | 12 | 32 | 0 | 0 |
| Path 61 | C00024->C00123:[49->1,49->5,50->2,50->9], C00022->C00123:[2->3] | 0.83 | 374.378378378 | 15 | 37 | 0 | 0 |
| Path 62 | C00024->C00123:[49->3,49->5,50->8,50->9], C00022->C00123:[1->3,1->5,3->8,3->9] | 0.67 | 358.125 | 14 | 24 | 0 | 0 |
| Path 63 | C00024->C00123:[49->3,49->5,50->8,50->9], C00022->C00123:[1->3,1->5,3->8,3->9] | 0.67 | 274.739130435 | 16 | 46 | 0 | 0 |
| Path 64 | C00024->C00123:[49->1,49->5,50->2,50->9], C00022->C00123:[2->3] | 0.83 | 368.315068493 | 25 | 73 | 0 | 0 |
| Path 65 | C00024->C00123:[49->1,50->2,50->8], C00022->C00123:[2->3] | 0.67 | 496.214285714 | 18 | 42 | 0 | 0 |
| Path 66 | C00024->C00123:[49->3,49->5,50->8,50->9] | 0.67 | 285.341463415 | 15 | 41 | 0 | 0 |
| Path 67 | C00024->C00123:[49->1,50->2,50->8], C00022->C00123:[2->3] | 0.67 | 389.2 | 21 | 65 | 0 | 0 |
| Path 68 | C00024->C00123:[49->5,50->8,50->9], C00022->C00123:[1->5,2->3,3->8,3->9] | 0.67 | 282.089285714 | 16 | 56 | 0 | 0 |
| Path 69 | C00024->C00123:[49->5,50->8,50->9], C00022->C00123:[1->5,2->3,3->8,3->9] | 0.67 | 463.404255319 | 19 | 47 | 0 | 0 |
| Path 70 | C00024->C00123:[49->1,50->2,50->8], C00022->C00123:[1->1,2->3,3->2,3->8] | 0.67 | 456.021276596 | 20 | 47 | 0 | 0 |
| Path 71 | C00024->C00123:[49->1,49->5,50->2,50->9], C00022->C00123:[1->1,1->5,2->3,3->2,3->9] | 0.83 | 376.847222222 | 19 | 72 | 0 | 0 |
| Path 72 | C00024->C00123:[49->1,49->5,50->2,50->9], C00022->C00123:[1->1,1->5,2->3,3->2,3->9] | 0.83 | 316.675675676 | 13 | 37 | 0 | 0 |
| Path 73 | C00024->C00123:[49->5,50->8,50->9], C00022->C00123:[1->5,2->3,3->8,3->9] | 0.67 | 369.885714286 | 14 | 35 | 0 | 0 |
| Path 74 | C00024->C00123:[49->1,50->2,50->8], C00022->C00123:[2->3] | 0.67 | 457.454545455 | 19 | 44 | 0 | 0 |
| Path 75 | C00024->C00123:[49->5,50->8,50->9], C00022->C00123:[1->5,2->3,3->8,3->9] | 0.67 | 343.636363636 | 13 | 33 | 0 | 0 |
| Path 76 | C00024->C00123:[49->1,50->2,50->8], C00022->C00123:[1->1,3->2,3->3,3->8] | 0.67 | 428.620689655 | 18 | 29 | 0 | 0 |
| Path 77 | C00024->C00123:[49->1,50->2,50->8], C00022->C00123:[1->1,2->3,3->2,3->8] | 0.67 | 326.615384615 | 15 | 39 | 0 | 0 |
| Path 78 | C00024->C00123:[49->1,49->3,50->2,50->8], C00022->C00123:[1->1,1->3,3->2,3->8] | 0.67 | 355.695652174 | 13 | 23 | 0 | 0 |
| Path 79 | C00024->C00123:[49->1,50->2,50->8], C00022->C00123:[2->3] | 0.67 | 445.627906977 | 18 | 43 | 0 | 0 |
| Path 80 | C00024->C00123:[49->1,49->3,50->2,50->8], C00022->C00123:[1->1,1->3,3->2,3->8] | 0.67 | 244.732142857 | 17 | 56 | 0 | 0 |
| Path 81 | C00024->C00123:[49->1,49->5,50->2,50->9], C00022->C00123:[1->1,1->5,2->3,3->2,3->9] | 0.83 | 352.166666667 | 16 | 42 | 0 | 0 |
| Path 82 | C00024->C00123:[49->1,50->2,50->8], C00022->C00123:[2->3] | 0.67 | 400.233333333 | 13 | 30 | 0 | 0 |
| Path 83 | C00024->C00123:[49->1,49->5,50->2,50->8,50->9], C00022->C00123:[1->1,1->5,2->3,3->2,3->8,3->9] | 1.00 | 330.684210526 | 14 | 38 | 0 | 0 |
| Path 84 | C00024->C00123:[49->1,50->2,50->8], C00022->C00123:[2->3] | 0.67 | 408.15625 | 14 | 32 | 0 | 0 |
| Path 85 | C00024->C00123:[49->1,50->2,50->8], C00022->C00123:[1->1,3->2,3->3,3->8] | 0.67 | 387.225806452 | 17 | 31 | 0 | 0 |
| Path 86 | C00024->C00123:[49->1,50->2,50->8], C00022->C00123:[1->1,3->2,3->3,3->8] | 0.67 | 412.470588235 | 19 | 34 | 0 | 0 |
| Path 87 | C00024->C00123:[49->1,49->5,50->2,50->9], C00022->C00123:[2->3] | 0.83 | 305.15 | 18 | 60 | 0 | 0 |
| Path 88 | C00024->C00123:[49->3,49->5,50->8,50->9], C00022->C00123:[1->3,1->5,3->8,3->9] | 0.67 | 280.242424242 | 14 | 33 | 0 | 0 |
| Path 89 | C00024->C00123:[49->1,49->5,50->2,50->9], C00022->C00123:[1->1,1->5,2->3,3->2,3->9] | 0.83 | 354.133333333 | 18 | 45 | 0 | 0 |
| Path 90 | C00024->C00123:[49->1,50->2,50->8], C00022->C00123:[2->3] | 0.67 | 336.8125 | 12 | 32 | 0 | 0 |
| Path 91 | C00024->C00123:[49->1,50->2,50->8], C00022->C00123:[1->1,2->3,3->2,3->8] | 0.67 | 369.885714286 | 14 | 35 | 0 | 0 |
| Path 92 | C00024->C00123:[49->1,49->3,49->5,50->2,50->9] | 0.83 | 482.852941176 | 19 | 34 | 0 | 0 |
| Path 93 | C00024->C00123:[49->3,49->5,50->8,50->9], C00022->C00123:[1->3,1->5,3->8,3->9] | 0.67 | 284.176470588 | 15 | 34 | 0 | 0 |
| Path 94 | C00024->C00123:[49->1,49->5,50->2,50->9], C00022->C00123:[1->1,1->5,2->3,3->2,3->9] | 0.83 | 326.615384615 | 15 | 39 | 0 | 0 |
| Path 95 | C00024->C00123:[49->1,50->2,50->8], C00022->C00123:[1->1,2->3,3->2,3->8] | 0.67 | 212.46 | 20 | 100 | 0 | 0 |
| Path 96 | C00024->C00123:[49->1,49->5,50->2,50->9], C00022->C00123:[2->3] | 0.83 | 277.0 | 15 | 55 | 0 | 0 |
| Path 97 | C00024->C00123:[49->1,49->3,49->5,50->2,50->9] | 0.83 | 362.16 | 15 | 25 | 0 | 0 |
| Path 98 | C00024->C00123:[49->1,50->2,50->8], C00022->C00123:[3->3] | 0.67 | 478.791666667 | 17 | 24 | 0 | 0 |
| Path 99 | C00024->C00123:[49->1,49->5,50->2,50->8,50->9], C00022->C00123:[2->3] | 1.00 | 379.083333333 | 15 | 36 | 0 | 0 |
| Path 100 | C00024->C00123:[49->1,49->3,50->2,50->8], C00022->C00123:[1->1,1->3,3->2,3->8] | 0.67 | 368.08 | 15 | 25 | 0 | 0 |
| Path 101 | C00024->C00123:[49->5,50->8,50->9], C00022->C00123:[1->5,2->3,3->8,3->9] | 0.67 | 371.111111111 | 15 | 36 | 0 | 0 |
| Path 102 | C00024->C00123:[49->1,50->2,50->8], C00022->C00123:[2->3] | 0.67 | 369.888888889 | 11 | 27 | 0 | 0 |
| Path 103 | C00024->C00123:[49->1,50->2], C00022->C00123:[2->3] | 0.50 | 389.258064516 | 13 | 31 | 0 | 0 |
| Path 104 | C00024->C00123:[49->1,49->5,50->2,50->8,50->9], C00022->C00123:[1->1,1->5,2->3,3->2,3->8,3->9] | 1.00 | 203.851851852 | 24 | 108 | 0 | 1 |
| Path 105 | C00024->C00123:[49->1,49->5,50->2,50->9], C00022->C00123:[3->3] | 0.83 | 300.037735849 | 22 | 53 | 0 | 1 |
| Path 106 | C00024->C00123:[49->1,50->2,50->8], C00022->C00123:[2->3] | 0.67 | 469.382978723 | 19 | 47 | 0 | 0 |
| Path 107 | C00024->C00123:[49->1,49->5,50->2,50->9], C00022->C00123:[3->3] | 0.83 | 294.196428571 | 21 | 56 | 0 | 1 |
| Path 108 | C00024->C00123:[49->1,49->5,50->2,50->8,50->9], C00022->C00123:[3->3] | 1.00 | 318.55 | 27 | 60 | 0 | 1 |
| Path 109 | C00024->C00123:[50->8], C00022->C00123:[3->3] | 0.33 | 432.75 | 20 | 32 | 0 | 1 |
| Path 110 | C00024->C00123:[49->1,49->5,50->2,50->8,50->9], C00022->C00123:[1->1,1->5,2->3,3->2,3->8,3->9] | 1.00 | 237.379032258 | 27 | 124 | 0 | 1 |
| Path 111 | C00024->C00123:[49->1,49->5,50->2,50->3,50->8,50->9] | 1.00 | 343.440677966 | 24 | 59 | 0 | 1 |
| Path 112 | C00024->C00123:[49->1,50->2,50->8], C00022->C00123:[1->1,3->2,3->3,3->8] | 0.67 | 392.939393939 | 20 | 33 | 0 | 1 |
| Path 113 | C00024->C00123:[49->1,50->2,50->8], C00022->C00123:[1->1,3->2,3->3,3->8] | 0.67 | 432.371428571 | 21 | 35 | 0 | 1 |
| Path 114 | C00024->C00123:[50->8], C00022->C00123:[3->3,3->8] | 0.33 | 385.275862069 | 18 | 29 | 0 | 1 |
| Path 115 | C00024->C00123:[49->1,50->2], C00022->C00123:[1->1,2->3,3->2] | 0.50 | 314.905660377 | 16 | 53 | 0 | 0 |
| Path 116 | C00024->C00123:[49->1,49->3,49->5,50->2,50->9] | 0.83 | 437.121212121 | 17 | 33 | 0 | 1 |
| Path 117 | C00024->C00123:[49->1,50->2], C00022->C00123:[2->3] | 0.50 | 274.489795918 | 13 | 49 | 0 | 0 |
| Path 118 | C00024->C00123:[49->1,49->5,50->2,50->9], C00022->C00123:[2->3] | 0.83 | 349.720588235 | 22 | 68 | 0 | 1 |
| Path 119 | C00024->C00123:[49->1,49->3,50->2] | 0.50 | 1108.66666667 | 17 | 36 | 0 | 1 |
| Path 120 | C00024->C00123:[49->1,49->5,50->2,50->8,50->9], C00022->C00123:[2->3] | 1.00 | 217.896226415 | 25 | 106 | 0 | 1 |
| Path 121 | C00024->C00123:[49->1,49->3,49->5,50->2,50->8,50->9] | 1.00 | 695.52173913 | 26 | 69 | 0 | 1 |
| Path 122 | C00024->C00123:[50->8] | 0.17 | 343.0 | 9 | 14 | 0 | 0 |
| Path 123 | C00024->C00123:[49->1,49->3,50->2], C00022->C00123:[1->1,1->3,3->2] | 0.50 | 996.365853659 | 18 | 41 | 0 | 1 |
| Path 124 | C00024->C00123:[49->1,49->3,49->5,50->2,50->8,50->9], C00022->C00123:[1->1,1->3,1->5,3->2,3->8,3->9] | 1.00 | 387.641025641 | 21 | 39 | 0 | 1 |
| Path 125 | C00024->C00123:[49->1,49->5,50->2,50->9], C00022->C00123:[1->1,1->5,3->2,3->3,3->9] | 0.83 | 335.371428571 | 19 | 35 | 0 | 1 |
| Path 126 | C00024->C00123:[49->1,49->3,50->2], C00022->C00123:[1->1,1->3,3->2] | 0.50 | 348.103448276 | 22 | 58 | 0 | 1 |
| Path 127 | C00024->C00123:[49->1,50->2], C00022->C00123:[1->1,3->2,3->3] | 0.50 | 374.15625 | 19 | 32 | 0 | 1 |
| Path 128 | C00024->C00123:[50->8], C00022->C00123:[2->3,3->8] | 0.33 | 390.163636364 | 21 | 55 | 0 | 1 |
| Path 129 | C00024->C00123:[49->1,49->5,50->2,50->9], C00022->C00123:[1->1,1->5,2->3,3->2,3->9] | 0.83 | 378.109090909 | 23 | 55 | 0 | 1 |
| Path 130 | C00024->C00123:[49->1,49->5,50->2,50->8,50->9], C00022->C00123:[2->3] | 1.00 | 414.02 | 26 | 50 | 0 | 1 |
| Path 131 | C00024->C00123:[49->1,49->3,50->2] | 0.50 | 367.529411765 | 11 | 17 | 0 | 0 |
| Path 132 | C00024->C00123:[49->1,50->2,50->8] | 0.50 | 402.333333333 | 12 | 18 | 0 | 0 |
| Path 133 | C00024->C00123:[49->1,49->5,50->2,50->9], C00022->C00123:[1->1,1->5,2->3,3->2,3->9] | 0.83 | 207.46728972 | 22 | 107 | 0 | 1 |
| Path 134 | C00024->C00123:[49->1,49->5,50->2,50->9], C00022->C00123:[1->1,1->5,2->3,3->2,3->9] | 0.83 | 376.480769231 | 23 | 52 | 0 | 1 |
| Path 135 | C00024->C00123:[50->3,50->8] | 0.33 | 352.7 | 19 | 50 | 0 | 1 |
| Path 136 | C00024->C00123:[49->3,49->5,50->8,50->9], C00022->C00123:[1->3,1->5,3->8,3->9] | 0.67 | 307.753623188 | 18 | 69 | 0 | 1 |
| Path 137 | C00024->C00123:[49->5,50->8,50->9], C00022->C00123:[1->5,3->3,3->8,3->9] | 0.67 | 406.193548387 | 19 | 31 | 0 | 1 |
| Path 138 | C00024->C00123:[49->5,50->8,50->9], C00022->C00123:[1->5,2->3,3->8,3->9] | 0.67 | 325.171428571 | 14 | 35 | 0 | 1 |
| Path 139 | C00024->C00123:[49->1,50->2,50->8], C00022->C00123:[1->1,3->2,3->3,3->8] | 0.67 | 391.324324324 | 21 | 37 | 0 | 1 |
| Path 140 | C00024->C00123:[49->1,50->2,50->8], C00022->C00123:[1->1,3->2,3->3,3->8] | 0.67 | 406.193548387 | 19 | 31 | 0 | 1 |
| Path 141 | C00024->C00123:[49->1,50->2], C00022->C00123:[1->1,3->2,3->3] | 0.50 | 358.617647059 | 19 | 34 | 0 | 1 |
| Path 142 | C00024->C00123:[49->5,50->8,50->9], C00022->C00123:[1->5,2->3,3->8,3->9] | 0.67 | 196.243902439 | 23 | 123 | 0 | 1 |
| Path 143 | C00024->C00123:[49->1,49->5,50->2,50->9], C00022->C00123:[3->3] | 0.83 | 389.944444444 | 20 | 36 | 0 | 1 |
| Path 144 | C00024->C00123:[49->5,50->8,50->9], C00022->C00123:[1->5,3->3,3->8,3->9] | 0.67 | 394.055555556 | 20 | 36 | 0 | 1 |
| Path 145 | C00024->C00123:[49->3,49->5,50->8,50->9], C00022->C00123:[1->3,1->5,3->8,3->9] | 0.67 | 416.451612903 | 17 | 31 | 0 | 1 |
| Path 146 | C00024->C00123:[49->1,49->5,50->2,50->8,50->9], C00022->C00123:[3->3] | 1.00 | 395.444444444 | 23 | 36 | 0 | 1 |
| Path 147 | C00024->C00123:[49->5,50->8,50->9], C00022->C00123:[1->5,3->8,3->9] | 0.50 | 313.095238095 | 12 | 21 | 0 | 0 |
| Path 148 | C00024->C00123:[49->1,49->3,49->5,50->2,50->9], C00022->C00123:[2->3] | 0.83 | 319.862068966 | 24 | 58 | 0 | 1 |
| Path 149 | C00024->C00123:[49->1,49->3,49->5,50->2,50->8,50->9] | 1.00 | 417.029411765 | 20 | 34 | 0 | 1 |
| Path 150 | C00024->C00123:[49->1,49->3,49->5,50->2,50->9], C00022->C00123:[2->3] | 0.83 | 444.19047619 | 25 | 42 | 0 | 1 |
| Path 151 | C00024->C00123:[49->1,49->3,50->2,50->8], C00022->C00123:[1->1,1->3,3->2,3->8] | 0.67 | 269.527777778 | 16 | 36 | 0 | 1 |
| Path 152 | C00024->C00123:[49->1,49->3,50->2,50->8], C00022->C00123:[1->1,1->3,3->2,3->8] | 0.67 | 332.153846154 | 15 | 26 | 0 | 1 |
| Path 153 | C00024->C00123:[49->1,49->3,50->2] | 0.50 | 267.625 | 14 | 40 | 0 | 0 |
| Path 154 | C00024->C00123:[49->1,50->2,50->3,50->8], C00022->C00123:[1->1,3->2,3->3,3->8] | 0.67 | 338.368421053 | 22 | 57 | 0 | 1 |
| Path 155 | C00024->C00123:[49->1,49->3,49->5,50->2,50->9], C00022->C00123:[1->1,1->3,1->5,3->2,3->9] | 0.83 | 350.432835821 | 24 | 67 | 0 | 1 |
| Path 156 | C00024->C00123:[49->1,49->3,49->5,50->2,50->9] | 0.83 | 263.583333333 | 17 | 48 | 0 | 1 |
| Path 157 | C00024->C00123:[50->8], C00022->C00123:[3->3,3->8] | 0.33 | 393.314285714 | 19 | 35 | 0 | 1 |
| Path 158 | C00024->C00123:[49->5,50->8,50->9], C00022->C00123:[1->5,2->3,3->8,3->9] | 0.67 | 209.882352941 | 21 | 102 | 0 | 1 |
| Path 159 | C00024->C00123:[49->1,49->5,50->2,50->8,50->9], C00022->C00123:[1->1,1->5,2->3,3->2,3->8,3->9] | 1.00 | 383.41025641 | 28 | 78 | 0 | 1 |
| Path 160 | C00024->C00123:[49->1,49->3,49->5,50->2,50->8,50->9], C00022->C00123:[1->1,1->3,1->5,3->2,3->8,3->9] | 1.00 | 458.947368421 | 22 | 38 | 0 | 1 |
| Path 161 | C00024->C00123:[49->1,50->2,50->8], C00022->C00123:[1->1,2->3,3->2,3->8] | 0.67 | 374.698113208 | 20 | 53 | 0 | 1 |
| Path 162 | C00024->C00123:[49->3,50->8] | 0.33 | 286.774193548 | 15 | 62 | 0 | 1 |
| Path 163 | C00024->C00123:[49->3,49->5,50->8,50->9], C00022->C00123:[1->3,1->5,3->8,3->9] | 0.67 | 307.542857143 | 19 | 70 | 0 | 1 |
| Path 164 | C00024->C00123:[49->1,49->5,50->2,50->3,50->9], C00022->C00123:[1->1,1->5,3->2,3->3,3->9] | 0.83 | 324.344262295 | 22 | 61 | 0 | 1 |
| Path 165 | C00024->C00123:[50->8], C00022->C00123:[3->3,3->8] | 0.33 | 292.18 | 19 | 50 | 0 | 1 |
| Path 166 | C00024->C00123:[49->1,49->5,50->2,50->9], C00022->C00123:[3->3] | 0.83 | 313.474576271 | 23 | 59 | 0 | 1 |
| Path 167 | C00024->C00123:[49->1,49->5,50->2,50->9], C00022->C00123:[1->1,1->5,2->3,3->2,3->9] | 0.83 | 371.639344262 | 23 | 61 | 0 | 1 |
| Path 168 | C00024->C00123:[49->1,50->2], C00022->C00123:[1->1,2->3,3->2] | 0.50 | 395.840909091 | 20 | 44 | 0 | 1 |
| Path 169 | C00024->C00123:[49->1,49->5,50->2,50->8,50->9], C00022->C00123:[1->1,1->5,3->2,3->3,3->8,3->9] | 1.00 | 428.27027027 | 23 | 37 | 0 | 1 |
| Path 170 | C00024->C00123:[49->1,50->2], C00022->C00123:[1->1,3->2,3->3] | 0.50 | 284.163636364 | 20 | 55 | 0 | 1 |
| Path 171 | C00024->C00123:[49->1,49->5,50->2,50->9], C00022->C00123:[2->3] | 0.83 | 194.237704918 | 22 | 122 | 0 | 1 |
| Path 172 | C00024->C00123:[49->1,50->2,50->8], C00022->C00123:[1->1,3->2,3->3,3->8] | 0.67 | 366.441176471 | 19 | 34 | 0 | 1 |
| Path 173 | C00024->C00123:[49->1,49->3,49->5,50->2,50->9] | 0.83 | 257.67032967 | 20 | 91 | 0 | 1 |
| Path 174 | C00024->C00123:[50->3,50->8], C00022->C00123:[3->3,3->8] | 0.33 | 179.237623762 | 19 | 101 | 0 | 1 |
| Path 175 | C00024->C00123:[49->5,50->8,50->9] | 0.50 | 387.41509434 | 20 | 53 | 0 | 0 |
| Path 176 | C00024->C00123:[49->1,50->2,50->8], C00022->C00123:[1->1,3->2,3->3,3->8] | 0.67 | 296.839285714 | 21 | 56 | 0 | 1 |
| Path 177 | C00024->C00123:[49->1,49->5,50->2,50->3,50->9] | 0.83 | 182.656 | 23 | 125 | 0 | 1 |
| Path 178 | C00024->C00123:[49->1,49->5,50->2,50->3,50->8,50->9], C00022->C00123:[1->1,1->5,3->2,3->3,3->8,3->9] | 1.00 | 184.961538462 | 22 | 104 | 0 | 1 |
| Path 179 | C00024->C00123:[49->1,49->5,50->2,50->9], C00022->C00123:[2->3] | 0.83 | 205.736 | 24 | 125 | 0 | 1 |
| Path 180 | C00024->C00123:[49->5,50->8,50->9], C00022->C00123:[1->5,3->3,3->8,3->9] | 0.67 | 390.0 | 21 | 34 | 0 | 1 |
| Path 181 | C00024->C00123:[49->1,50->2,50->8] | 0.50 | 285.341463415 | 15 | 41 | 0 | 0 |
| Path 182 | C00024->C00123:[49->1,49->3,50->2], C00022->C00123:[2->3] | 0.50 | 383.954545455 | 14 | 22 | 0 | 0 |
| Path 183 | C00024->C00123:[49->1,49->3,49->5,50->2,50->9], C00022->C00123:[1->1,1->3,1->5,2->3,3->2,3->9] | 0.83 | 217.839285714 | 25 | 112 | 0 | 1 |
| Path 184 | C00024->C00123:[49->1,49->5,50->2,50->9], C00022->C00123:[1->1,1->5,3->2,3->9] | 0.67 | 278.08 | 12 | 25 | 0 | 0 |
| Path 185 | C00024->C00123:[49->1,49->5,50->2,50->8,50->9], C00022->C00123:[1->1,1->5,2->3,3->2,3->8,3->9] | 1.00 | 212.701923077 | 23 | 104 | 0 | 1 |
| Path 186 | C00024->C00123:[49->1,49->3,49->5,50->2,50->8,50->9] | 1.00 | 283.8 | 24 | 95 | 0 | 1 |
| Path 187 | C00024->C00123:[49->1,50->2], C00022->C00123:[2->3] | 0.50 | 328.145833333 | 15 | 48 | 0 | 0 |
| Path 188 | C00024->C00123:[49->1,49->5,50->2,50->9], C00022->C00123:[2->3] | 0.83 | 360.928571429 | 18 | 42 | 0 | 1 |
| Path 189 | C00024->C00123:[49->1,49->3,49->5,50->2,50->8,50->9], C00022->C00123:[1->1,1->3,1->5,3->2,3->8,3->9] | 1.00 | 335.407407407 | 16 | 27 | 0 | 1 |
| Path 190 | C00024->C00123:[49->1,50->2], C00022->C00123:[2->3] | 0.50 | 398.92 | 20 | 50 | 0 | 1 |
| Path 191 | C00024->C00123:[49->3,50->8] | 0.33 | 315.492063492 | 16 | 63 | 0 | 1 |
| Path 192 | C00024->C00123:[49->1,50->2,50->8], C00022->C00123:[1->1,2->3,3->2,3->8] | 0.67 | 390.696428571 | 22 | 56 | 0 | 1 |
| Path 193 | C00024->C00123:[49->3,50->8] | 0.33 | 489.407407407 | 15 | 27 | 0 | 1 |
| Path 194 | C00024->C00123:[49->1,50->2,50->8], C00022->C00123:[3->3] | 0.67 | 421.038461538 | 19 | 26 | 0 | 1 |
| Path 195 | C00024->C00123:[49->1,49->5,50->2,50->8,50->9], C00022->C00123:[1->1,1->5,3->2,3->3,3->8,3->9] | 1.00 | 403.181818182 | 21 | 33 | 0 | 1 |
| Path 196 | C00024->C00123:[49->1,50->2,50->8], C00022->C00123:[3->3] | 0.67 | 485.5 | 23 | 34 | 0 | 1 |
| Path 197 | C00024->C00123:[49->1,49->3,49->5,50->2,50->9] | 0.83 | 236.534482759 | 18 | 58 | 0 | 1 |
| Path 198 | C00024->C00123:[49->1,49->3,49->5,50->2,50->8,50->9], C00022->C00123:[1->1,1->3,1->5,3->2,3->8,3->9] | 1.00 | 434.872340426 | 24 | 47 | 0 | 1 |
| Path 199 | C00024->C00123:[49->1,49->5,50->2,50->8,50->9], C00022->C00123:[1->1,1->5,2->3,3->2,3->8,3->9] | 1.00 | 381.709090909 | 26 | 55 | 0 | 1 |
| Path 200 | C00024->C00123:[49->1,50->2,50->8], C00022->C00123:[1->1,2->3,3->2,3->8] | 0.67 | 195.450819672 | 22 | 122 | 0 | 1 |
| Path 201 | C00024->C00123:[49->1,49->5,50->2,50->8,50->9], C00022->C00123:[1->1,1->5,2->3,3->2,3->8,3->9] | 1.00 | 382.982758621 | 26 | 58 | 0 | 1 |
| Path 202 | C00024->C00123:[49->1,50->2,50->8], C00022->C00123:[1->1,3->2,3->8] | 0.50 | 486.142857143 | 18 | 35 | 0 | 0 |
| Path 203 | C00024->C00123:[49->1,50->2], C00022->C00123:[1->1,3->2,3->3] | 0.50 | 415.852941176 | 20 | 34 | 0 | 1 |
| Path 204 | C00024->C00123:[49->1,49->5,50->2,50->8,50->9], C00022->C00123:[2->3] | 1.00 | 200.912 | 25 | 125 | 0 | 1 |
| Path 205 | C00024->C00123:[49->1,49->3,49->5,50->2,50->8,50->9], C00022->C00123:[1->1,1->3,1->5,2->3,3->2,3->8,3->9] | 1.00 | 224.47826087 | 28 | 115 | 0 | 1 |
| Path 206 | C00024->C00123:[49->1,49->5,50->2,50->8,50->9], C00022->C00123:[1->1,1->5,3->2,3->3,3->8,3->9] | 1.00 | 380.5 | 24 | 40 | 0 | 1 |
| Path 207 | C00024->C00123:[49->1,50->2,50->8] | 0.50 | 367.913043478 | 13 | 23 | 0 | 0 |
| Path 208 | C00024->C00123:[49->1,50->2,50->8], C00022->C00123:[3->3] | 0.67 | 467.322580645 | 21 | 31 | 0 | 1 |
| Path 209 | C00022->C00123:[3->3] | 0.17 | 423.904761905 | 13 | 21 | 0 | 1 |
| Path 210 | C00024->C00123:[49->3,50->8], C00022->C00123:[1->3,3->8] | 0.33 | 442.28125 | 16 | 32 | 0 | 1 |
| Path 211 | C00024->C00123:[49->5,50->8,50->9], C00022->C00123:[1->5,3->3,3->8,3->9] | 0.67 | 386.433333333 | 19 | 30 | 0 | 1 |
| Path 212 | C00024->C00123:[49->1,49->3,50->2], C00022->C00123:[1->1,1->3,3->2] | 0.50 | 326.681818182 | 12 | 22 | 0 | 0 |
| Path 213 | C00024->C00123:[49->1,49->5,50->2,50->8,50->9], C00022->C00123:[1->1,1->5,3->2,3->3,3->8,3->9] | 1.00 | 372.590909091 | 24 | 44 | 0 | 1 |
| Path 214 | C00024->C00123:[49->5,50->8,50->9], C00022->C00123:[1->5,2->3,3->8,3->9] | 0.67 | 432.711111111 | 19 | 45 | 0 | 1 |
| Path 215 | C00024->C00123:[49->1,50->2,50->8] | 0.50 | 387.41509434 | 20 | 53 | 0 | 0 |
| Path 216 | C00024->C00123:[49->1,49->3,49->5,50->2,50->9] | 0.83 | 264.628571429 | 15 | 35 | 0 | 1 |
| Path 217 | C00024->C00123:[49->1,50->2,50->8], C00022->C00123:[1->1,3->2,3->3,3->8] | 0.67 | 391.324324324 | 21 | 37 | 0 | 1 |
| Path 218 | C00024->C00123:[49->3,49->5,50->8,50->9], C00022->C00123:[1->3,1->5,3->8,3->9] | 0.67 | 463.1 | 21 | 40 | 0 | 1 |
| Path 219 | C00024->C00123:[49->1,49->3,49->5,50->2,50->8,50->9] | 1.00 | 252.552238806 | 24 | 67 | 0 | 1 |
| Path 220 | C00024->C00123:[49->1,49->3,49->5,50->2,50->8,50->9] | 1.00 | 273.188679245 | 22 | 53 | 0 | 1 |
| Path 221 | C00024->C00123:[49->1,49->5,50->2,50->9], C00022->C00123:[3->3] | 0.83 | 388.454545455 | 20 | 33 | 0 | 1 |
| Path 222 | C00024->C00123:[49->1,49->5,50->2,50->9], C00022->C00123:[1->1,1->5,3->2,3->3,3->9] | 0.83 | 398.1 | 22 | 40 | 0 | 1 |
| Path 223 | C00024->C00123:[49->1,50->2,50->8] | 0.50 | 300.65 | 11 | 20 | 0 | 0 |
| Path 224 | C00024->C00123:[49->1,49->3,49->5,50->2,50->8,50->9] | 1.00 | 334.066666667 | 19 | 30 | 0 | 1 |
| Path 225 | C00024->C00123:[49->1,49->3,50->2], C00022->C00123:[1->1,1->3,2->3,3->2] | 0.50 | 213.330188679 | 23 | 106 | 0 | 1 |
| Path 226 | C00024->C00123:[50->8], C00022->C00123:[3->3] | 0.33 | 453.851851852 | 19 | 27 | 0 | 1 |
| Path 227 | C00024->C00123:[50->8], C00022->C00123:[2->3] | 0.33 | 205.932773109 | 22 | 119 | 0 | 1 |
| Path 228 | C00024->C00123:[49->1,49->5,50->2,50->9], C00022->C00123:[1->1,1->5,3->2,3->3,3->9] | 0.83 | 362.052631579 | 21 | 38 | 0 | 1 |
| Path 229 | C00024->C00123:[49->1,50->2,50->8], C00022->C00123:[3->3] | 0.67 | 306.304347826 | 19 | 46 | 0 | 1 |
| Path 230 | C00024->C00123:[49->1,49->5,50->2,50->8,50->9], C00022->C00123:[3->3] | 1.00 | 396.108108108 | 24 | 37 | 0 | 1 |
| Path 231 | C00024->C00123:[49->1,49->5,50->2,50->9], C00022->C00123:[1->1,1->5,3->2,3->3,3->9] | 0.83 | 349.45 | 21 | 40 | 0 | 1 |
| Path 232 | C00024->C00123:[49->1,49->3,49->5,50->2,50->8,50->9] | 1.00 | 264.521276596 | 23 | 94 | 0 | 1 |
| Path 233 | C00024->C00123:[49->1,50->2,50->8], C00022->C00123:[1->1,2->3,3->2,3->8] | 0.67 | 196.81 | 20 | 100 | 0 | 1 |
| Path 234 | C00024->C00123:[49->1,49->3,50->2], C00022->C00123:[1->1,1->3,2->3,3->2] | 0.50 | 380.58974359 | 21 | 39 | 0 | 1 |
| Path 235 | C00024->C00123:[49->1,49->3,50->2,50->8], C00022->C00123:[1->1,1->3,3->2,3->8] | 0.67 | 269.951219512 | 18 | 41 | 0 | 1 |
| Path 236 | C00024->C00123:[49->1,50->2,50->3], C00022->C00123:[1->1,3->2,3->3] | 0.50 | 327.272727273 | 20 | 55 | 0 | 1 |
| Path 237 | C00024->C00123:[49->1,50->2], C00022->C00123:[2->3] | 0.50 | 370.575757576 | 14 | 33 | 0 | 1 |
| Path 238 | C00024->C00123:[49->5,50->8,50->9] | 0.50 | 348.133333333 | 10 | 15 | 0 | 0 |
| Path 239 | C00024->C00123:[49->1,49->3,49->5,50->2,50->9], C00022->C00123:[1->1,1->3,1->5,3->2,3->9] | 0.83 | 404.315789474 | 18 | 38 | 0 | 1 |
| Path 240 | C00024->C00123:[49->3,49->5,50->8,50->9], C00022->C00123:[1->3,1->5,3->8,3->9] | 0.67 | 979.953488372 | 20 | 43 | 0 | 1 |
| Path 241 | C00024->C00123:[49->1,50->2], C00022->C00123:[1->1,2->3,3->2] | 0.50 | 362.846153846 | 16 | 39 | 0 | 0 |
| Path 242 | C00024->C00123:[49->1,50->2,50->8], C00022->C00123:[3->3] | 0.67 | 424.862068966 | 20 | 29 | 0 | 1 |
| Path 243 | C00024->C00123:[49->1,49->3,49->5,50->2,50->9], C00022->C00123:[2->3] | 0.83 | 397.857142857 | 24 | 42 | 0 | 1 |
| Path 244 | C00024->C00123:[49->1,50->2,50->8] | 0.50 | 362.16 | 15 | 25 | 0 | 0 |
| Path 245 | C00024->C00123:[49->1,49->5,50->2,50->3,50->8,50->9], C00022->C00123:[1->1,1->5,3->2,3->3,3->8,3->9] | 1.00 | 331.28125 | 25 | 64 | 0 | 1 |
| Path 246 | C00024->C00123:[49->5,50->8,50->9], C00022->C00123:[1->5,3->3,3->8,3->9] | 0.67 | 294.653846154 | 21 | 52 | 0 | 1 |
| Path 247 | C00024->C00123:[49->5,50->3,50->8,50->9], C00022->C00123:[1->5,3->3,3->8,3->9] | 0.67 | 182.67961165 | 21 | 103 | 0 | 1 |
| Path 248 | C00024->C00123:[49->1,49->5,50->2,50->8,50->9], C00022->C00123:[1->1,1->5,3->2,3->3,3->8,3->9] | 1.00 | 358.023255814 | 24 | 43 | 0 | 1 |
| Path 249 | C00024->C00123:[49->1,50->2,50->3] | 0.50 | 294.767123288 | 22 | 73 | 0 | 1 |
| Path 250 | C00024->C00123:[49->1,49->3,49->5,50->2,50->8,50->9] | 1.00 | 417.114285714 | 21 | 35 | 0 | 1 |
| Path 251 | C00024->C00123:[49->3,50->8], C00022->C00123:[1->3,3->8] | 0.33 | 1010.36585366 | 18 | 41 | 0 | 1 |
| Path 252 | C00024->C00123:[49->1,49->5,50->2,50->8,50->9], C00022->C00123:[1->1,1->5,2->3,3->2,3->8,3->9] | 1.00 | 389.517241379 | 24 | 58 | 0 | 1 |
| Path 253 | C00024->C00123:[49->1,49->5,50->2,50->9], C00022->C00123:[1->1,1->5,3->2,3->3,3->9] | 0.83 | 365.292682927 | 21 | 41 | 0 | 1 |
| Path 254 | C00024->C00123:[49->1,49->5,50->2,50->8,50->9], C00022->C00123:[1->1,1->5,3->2,3->3,3->8,3->9] | 1.00 | 294.283333333 | 25 | 60 | 0 | 1 |
| Path 255 | C00024->C00123:[49->1,50->2,50->3,50->8] | 0.67 | 182.418367347 | 20 | 98 | 0 | 1 |
| Path 256 | C00024->C00123:[49->1,49->3,49->5,50->2,50->9] | 0.83 | 305.608695652 | 18 | 69 | 0 | 1 |
| Path 257 | C00024->C00123:[49->1,49->3,49->5,50->2,50->8,50->9], C00022->C00123:[1->1,1->3,1->5,3->2,3->8,3->9] | 1.00 | 349.134328358 | 27 | 67 | 0 | 1 |
| Path 258 | C00024->C00123:[49->1,49->3,49->5,50->2,50->9] | 0.83 | 318.87037037 | 20 | 54 | 0 | 1 |
| Path 259 | C00024->C00123:[49->3] | 0.17 | 312.533333333 | 9 | 15 | 0 | 1 |
| Path 260 | C00024->C00123:[49->1,49->5,50->2,50->8,50->9], C00022->C00123:[1->1,1->5,2->3,3->2,3->8,3->9] | 1.00 | 318.976744186 | 18 | 43 | 0 | 1 |
| Path 261 | C00024->C00123:[49->1,50->2,50->8], C00022->C00123:[1->1,2->3,3->2,3->8] | 0.67 | 210.689320388 | 22 | 103 | 0 | 1 |
| Path 262 | C00024->C00123:[49->1,50->2,50->8], C00022->C00123:[2->3] | 0.67 | 195.777777778 | 21 | 117 | 0 | 1 |
| Path 263 | C00024->C00123:[49->1,49->5,50->2,50->8,50->9], C00022->C00123:[2->3] | 1.00 | 338.565789474 | 28 | 76 | 0 | 1 |
| Path 264 | C00024->C00123:[49->3,50->8], C00022->C00123:[1->3,3->8] | 0.33 | 330.125 | 13 | 24 | 0 | 1 |
| Path 265 | C00024->C00123:[49->1,49->5,50->2,50->8,50->9], C00022->C00123:[2->3] | 1.00 | 232.063380282 | 29 | 142 | 0 | 1 |
| Path 266 | C00024->C00123:[49->1,49->3,49->5,50->2,50->9] | 0.83 | 411.677419355 | 17 | 31 | 0 | 1 |
| Path 267 | C00024->C00123:[49->1,50->2,50->3,50->8] | 0.67 | 354.019607843 | 20 | 51 | 0 | 1 |
| Path 268 | C00024->C00123:[49->1,50->2,50->8], C00022->C00123:[3->3] | 0.67 | 432.363636364 | 21 | 33 | 0 | 1 |
| Path 269 | C00024->C00123:[49->1,49->5,50->2,50->9], C00022->C00123:[2->3] | 0.83 | 333.068493151 | 25 | 73 | 0 | 1 |
| Path 270 | C00024->C00123:[49->1,49->5,50->2,50->3,50->8,50->9] | 1.00 | 191.23255814 | 27 | 129 | 0 | 1 |
| Path 271 | C00024->C00123:[49->1,49->5,50->2,50->9], C00022->C00123:[1->1,1->5,3->2,3->9] | 0.67 | 237.6875 | 15 | 48 | 0 | 0 |
| Path 272 | C00022->C00123:[3->3] | 0.17 | 281.128205128 | 13 | 39 | 0 | 1 |
| Path 273 | C00024->C00123:[49->1,50->2], C00022->C00123:[2->3] | 0.50 | 374.656716418 | 23 | 67 | 0 | 0 |
| Path 274 | C00024->C00123:[49->1,49->5,50->2,50->8,50->9], C00022->C00123:[1->1,1->5,2->3,3->2,3->8,3->9] | 1.00 | 377.030769231 | 27 | 65 | 0 | 1 |
| Path 275 | C00024->C00123:[49->1,49->3,49->5,50->2,50->8,50->9], C00022->C00123:[1->1,1->3,1->5,2->3,3->2,3->8,3->9] | 1.00 | 382.581395349 | 25 | 43 | 0 | 1 |
| Path 276 | C00024->C00123:[49->3,49->5,50->8,50->9], C00022->C00123:[1->3,1->5,3->8,3->9] | 0.67 | 269.375 | 17 | 40 | 0 | 1 |
| Path 277 | C00024->C00123:[49->1,50->2,50->8] | 0.50 | 494.171428571 | 18 | 35 | 0 | 0 |
| Path 278 | C00024->C00123:[49->1,50->2,50->8], C00022->C00123:[1->1,3->2,3->8] | 0.50 | 426.0 | 18 | 36 | 0 | 0 |
| Path 279 | C00024->C00123:[50->8], C00022->C00123:[3->3] | 0.33 | 303.777777778 | 18 | 45 | 0 | 1 |
| Path 280 | C00024->C00123:[49->1,50->2,50->8], C00022->C00123:[2->3] | 0.67 | 211.020618557 | 20 | 97 | 0 | 1 |
| Path 281 | C00024->C00123:[49->1,49->3,49->5,50->2,50->9] | 0.83 | 265.675 | 17 | 40 | 0 | 1 |
| Path 282 | C00024->C00123:[49->1,49->3,50->2,50->8], C00022->C00123:[1->1,1->3,3->2,3->8] | 0.67 | 281.455882353 | 17 | 68 | 0 | 1 |
| Path 283 | C00024->C00123:[49->1,49->5,50->2,50->9] | 0.67 | 243.488372093 | 14 | 43 | 0 | 0 |
| Path 284 | C00024->C00123:[49->1,49->3,49->5,50->2,50->9], C00022->C00123:[2->3] | 0.83 | 319.777777778 | 25 | 63 | 0 | 1 |
| Path 285 | C00024->C00123:[49->1,50->2], C00022->C00123:[2->3] | 0.50 | 345.884615385 | 10 | 26 | 0 | 0 |
| Path 286 | C00024->C00123:[49->1,49->3,49->5,50->2,50->9], C00022->C00123:[1->1,1->3,1->5,2->3,3->2,3->9] | 0.83 | 371.093023256 | 24 | 43 | 0 | 1 |
| Path 287 | C00024->C00123:[49->1,50->2,50->8], C00022->C00123:[3->3] | 0.67 | 327.851851852 | 22 | 54 | 0 | 1 |
| Path 288 | C00024->C00123:[49->1,50->2], C00022->C00123:[3->3] | 0.50 | 455.172413793 | 19 | 29 | 0 | 1 |
| Path 289 | C00024->C00123:[49->1,49->3,50->2,50->8], C00022->C00123:[1->1,1->3,3->2,3->8] | 0.67 | 269.375 | 17 | 40 | 0 | 1 |
| Path 290 | C00024->C00123:[49->1,49->5,50->2,50->3,50->8,50->9], C00022->C00123:[1->1,1->5,3->2,3->3,3->8,3->9] | 1.00 | 190.54954955 | 25 | 111 | 0 | 1 |
| Path 291 | C00024->C00123:[49->1,49->5,50->2,50->8,50->9], C00022->C00123:[2->3] | 1.00 | 337.65060241 | 29 | 83 | 0 | 1 |
| Path 292 | C00024->C00123:[50->8], C00022->C00123:[2->3,3->8] | 0.33 | 207.801980198 | 20 | 101 | 0 | 1 |
| Path 293 | C00024->C00123:[49->1,49->3,50->2] | 0.50 | 321.541666667 | 18 | 48 | 0 | 1 |
| Path 294 | C00024->C00123:[49->1,50->2], C00022->C00123:[1->1,3->2,3->3] | 0.50 | 284.163636364 | 20 | 55 | 0 | 1 |
| Path 295 | C00024->C00123:[49->1,50->2,50->8] | 0.50 | 319.727272727 | 13 | 22 | 0 | 0 |
| Path 296 | C00024->C00123:[49->1,50->2] | 0.33 | 328.0625 | 11 | 16 | 0 | 0 |
| Path 297 | C00024->C00123:[49->1,49->5,50->2,50->8,50->9], C00022->C00123:[2->3] | 1.00 | 392.338983051 | 25 | 59 | 0 | 1 |
| Path 298 | C00024->C00123:[49->1,49->3,50->2], C00022->C00123:[1->1,1->3,3->2] | 0.50 | 297.661764706 | 17 | 68 | 0 | 1 |
| Path 299 | C00024->C00123:[49->1,49->5,50->2,50->8,50->9], C00022->C00123:[1->1,1->5,2->3,3->2,3->8,3->9] | 1.00 | 429.468085106 | 21 | 47 | 0 | 1 |
| Path 300 | C00024->C00123:[49->1,50->2], C00022->C00123:[1->1,3->2,3->3] | 0.50 | 349.125 | 17 | 32 | 0 | 1 |
| Path 301 | C00024->C00123:[49->1,49->3,50->2,50->8], C00022->C00123:[1->1,1->3,3->2,3->8] | 0.67 | 996.30952381 | 19 | 42 | 0 | 1 |
| Path 302 | C00024->C00123:[49->1,49->3,49->5,50->2,50->8,50->9] | 1.00 | 287.436619718 | 20 | 71 | 0 | 1 |
| Path 303 | C00024->C00123:[49->1,49->3,50->2], C00022->C00123:[1->1,1->3,3->2] | 0.50 | 355.639344262 | 22 | 61 | 0 | 1 |
| Path 304 | C00024->C00123:[49->3,50->8], C00022->C00123:[1->3,3->8] | 0.33 | 306.102941176 | 17 | 68 | 0 | 1 |
| Path 305 | C00024->C00123:[49->1,49->5,50->2,50->9], C00022->C00123:[3->3] | 0.83 | 301.655172414 | 23 | 58 | 0 | 1 |
| Path 306 | C00024->C00123:[49->1,50->2], C00022->C00123:[1->1,2->3,3->2] | 0.50 | 202.118811881 | 20 | 101 | 0 | 1 |
| Path 307 | C00024->C00123:[49->1,50->2], C00022->C00123:[2->3] | 0.50 | 266.901960784 | 14 | 51 | 0 | 1 |
| Path 308 | C00024->C00123:[49->1,49->3,50->2], C00022->C00123:[2->3] | 0.50 | 369.763636364 | 22 | 55 | 0 | 0 |
| Path 309 | C00024->C00123:[49->1,49->3,49->5,50->2,50->9], C00022->C00123:[1->1,1->3,1->5,3->2,3->9] | 0.83 | 343.359375 | 24 | 64 | 0 | 1 |
| Path 310 | C00024->C00123:[50->8], C00022->C00123:[3->3,3->8] | 0.33 | 428.928571429 | 17 | 28 | 0 | 0 |
| Path 311 | C00024->C00123:[49->5,50->8,50->9], C00022->C00123:[1->5,2->3,3->8,3->9] | 0.67 | 195.450819672 | 22 | 122 | 0 | 1 |
| Path 312 | C00024->C00123:[49->1,49->5,50->2,50->8,50->9], C00022->C00123:[1->1,1->5,3->2,3->3,3->8,3->9] | 1.00 | 364.641025641 | 24 | 39 | 0 | 1 |
| Path 313 | C00024->C00123:[49->1,49->3,49->5,50->2,50->8,50->9], C00022->C00123:[1->1,1->3,1->5,3->2,3->8,3->9] | 1.00 | 356.563380282 | 28 | 71 | 0 | 1 |
| Path 314 | C00024->C00123:[49->1,49->5,50->2,50->9], C00022->C00123:[3->3] | 0.83 | 381.5 | 19 | 30 | 0 | 1 |
| Path 315 | C00024->C00123:[50->8], C00022->C00123:[3->3,3->8] | 0.33 | 386.133333333 | 16 | 30 | 0 | 0 |
| Path 316 | C00024->C00123:[49->1,49->5,50->2,50->8,50->9], C00022->C00123:[2->3] | 1.00 | 405.363636364 | 27 | 55 | 0 | 1 |
| Path 317 | C00024->C00123:[49->1,50->2], C00022->C00123:[2->3] | 0.50 | 334.287671233 | 23 | 73 | 0 | 1 |
| Path 318 | C00024->C00123:[49->1,50->2], C00022->C00123:[2->3] | 0.50 | 469.648648649 | 16 | 37 | 0 | 0 |
| Path 319 | C00024->C00123:[49->1,49->5,50->2,50->9], C00022->C00123:[1->1,1->5,2->3,3->2,3->9] | 0.83 | 194.028846154 | 20 | 104 | 0 | 1 |
| Path 320 | C00024->C00123:[49->1,49->3,49->5,50->2,50->8,50->9], C00022->C00123:[1->1,1->3,1->5,3->2,3->8,3->9] | 1.00 | 273.590909091 | 20 | 44 | 0 | 1 |
| Path 321 | C00022->C00123:[3->3] | 0.17 | 421.708333333 | 13 | 24 | 0 | 1 |
| Path 322 | C00024->C00123:[49->1,49->3,49->5,50->2,50->8,50->9], C00022->C00123:[1->1,1->3,1->5,3->2,3->8,3->9] | 1.00 | 283.6 | 19 | 70 | 0 | 1 |
| Path 323 | C00024->C00123:[49->1,49->5,50->2,50->8,50->9], C00022->C00123:[2->3] | 1.00 | 440.745454545 | 28 | 55 | 0 | 1 |
| Path 324 | C00024->C00123:[49->3] | 0.17 | 329.076923077 | 7 | 13 | 0 | 1 |
| Path 325 | C00024->C00123:[49->5,50->8,50->9], C00022->C00123:[1->5,3->8,3->9] | 0.50 | 250.727272727 | 15 | 44 | 0 | 0 |
| Path 326 | C00024->C00123:[49->1,49->3,50->2] | 0.50 | 382.578947368 | 13 | 19 | 0 | 0 |
| Path 327 | C00024->C00123:[49->1,49->3,50->2] | 0.50 | 324.761904762 | 14 | 21 | 0 | 1 |
| Path 328 | C00024->C00123:[49->1,50->2,50->8] | 0.50 | 464.419354839 | 17 | 31 | 0 | 0 |
| Path 329 | C00024->C00123:[49->1,50->2], C00022->C00123:[1->1,3->2,3->3] | 0.50 | 376.914285714 | 19 | 35 | 0 | 1 |
| Path 330 | C00024->C00123:[49->1,50->2,50->3,50->8], C00022->C00123:[1->1,3->2,3->3,3->8] | 0.67 | 181.598039216 | 20 | 102 | 0 | 1 |
| Path 331 | C00024->C00123:[49->1,50->2,50->8], C00022->C00123:[1->1,2->3,3->2,3->8] | 0.67 | 326.117647059 | 13 | 34 | 0 | 1 |
| Path 332 | C00024->C00123:[49->1,50->2], C00022->C00123:[1->1,2->3,3->2] | 0.50 | 188.851239669 | 21 | 121 | 0 | 1 |
| Path 333 | C00024->C00123:[49->1,49->3,49->5,50->2,50->9] | 0.83 | 327.8 | 14 | 25 | 0 | 1 |
| Path 334 | C00024->C00123:[49->1,49->5,50->2,50->8,50->9], C00022->C00123:[1->1,1->5,3->2,3->3,3->8,3->9] | 1.00 | 371.30952381 | 25 | 42 | 0 | 1 |
| Path 335 | C00024->C00123:[49->1,49->3,49->5,50->2,50->8,50->9], C00022->C00123:[2->3] | 1.00 | 220.54887218 | 30 | 133 | 0 | 1 |
| Path 336 | C00024->C00123:[50->8], C00022->C00123:[3->3,3->8] | 0.33 | 375.5 | 19 | 34 | 0 | 1 |
| Path 337 | C00024->C00123:[49->1,49->5,50->2,50->8,50->9], C00022->C00123:[1->1,1->5,3->2,3->3,3->8,3->9] | 1.00 | 288.721311475 | 25 | 61 | 0 | 1 |
| Path 338 | C00024->C00123:[49->1,49->5,50->2,50->8,50->9], C00022->C00123:[2->3] | 1.00 | 355.805555556 | 26 | 72 | 0 | 1 |
| Path 339 | C00024->C00123:[49->1,49->3,49->5,50->2,50->9], C00022->C00123:[1->1,1->3,1->5,2->3,3->2,3->9] | 0.83 | 369.511111111 | 23 | 45 | 0 | 1 |
| Path 340 | C00024->C00123:[49->1,49->3,49->5,50->2,50->8,50->9], C00022->C00123:[2->3] | 1.00 | 402.822222222 | 27 | 45 | 0 | 1 |
| Path 341 | C00024->C00123:[49->3,50->8], C00022->C00123:[1->3,3->8] | 0.33 | 264.411764706 | 14 | 34 | 0 | 1 |
| Path 342 | C00024->C00123:[49->1,50->2], C00022->C00123:[3->3] | 0.50 | 309.82 | 21 | 50 | 0 | 1 |
| Path 343 | C00024->C00123:[49->5,50->3,50->8,50->9], C00022->C00123:[1->5,3->3,3->8,3->9] | 0.67 | 338.368421053 | 22 | 57 | 0 | 1 |
| Path 344 | C00024->C00123:[49->1,49->3,50->2], C00022->C00123:[1->1,1->3,3->2] | 0.50 | 298.423076923 | 15 | 26 | 0 | 1 |
| Path 345 | C00024->C00123:[49->1,50->2,50->8], C00022->C00123:[1->1,2->3,3->2,3->8] | 0.67 | 429.673913043 | 20 | 46 | 0 | 1 |
| Path 346 | C00024->C00123:[50->8], C00022->C00123:[3->3,3->8] | 0.33 | 392.09375 | 19 | 32 | 0 | 1 |
| Path 347 | C00024->C00123:[49->1,50->2], C00022->C00123:[2->3] | 0.50 | 223.691729323 | 24 | 133 | 0 | 1 |
| Path 348 | C00024->C00123:[49->1,49->3,50->2,50->8], C00022->C00123:[1->1,1->3,3->2,3->8] | 0.67 | 460.0 | 21 | 37 | 0 | 1 |
| Path 349 | C00024->C00123:[49->1,49->5,50->2,50->8,50->9], C00022->C00123:[3->3] | 1.00 | 407.676470588 | 22 | 34 | 0 | 1 |
| Path 350 | C00024->C00123:[49->1,50->2,50->8], C00022->C00123:[2->3] | 0.67 | 196.601694915 | 22 | 118 | 0 | 1 |
| Path 351 | C00024->C00123:[49->1,49->3,49->5,50->2,50->9], C00022->C00123:[1->1,1->3,1->5,2->3,3->2,3->9] | 0.83 | 375.85 | 22 | 40 | 0 | 1 |
| Path 352 | C00024->C00123:[49->1,49->3,50->2,50->8], C00022->C00123:[1->1,1->3,3->2,3->8] | 0.67 | 464.638888889 | 20 | 36 | 0 | 1 |
| Path 353 | C00024->C00123:[49->5,50->8,50->9], C00022->C00123:[1->5,3->3,3->8,3->9] | 0.67 | 296.771929825 | 22 | 57 | 0 | 1 |
| Path 354 | C00024->C00123:[49->1,50->2,50->8], C00022->C00123:[1->1,2->3,3->2,3->8] | 0.67 | 432.711111111 | 19 | 45 | 0 | 1 |
| Path 355 | C00024->C00123:[49->1,49->3,50->2] | 0.50 | 306.380952381 | 16 | 63 | 0 | 1 |
| Path 356 | C00024->C00123:[49->1,50->2], C00022->C00123:[2->3] | 0.50 | 326.964285714 | 11 | 28 | 0 | 1 |
| Path 357 | C00024->C00123:[49->1,49->5,50->2,50->8,50->9], C00022->C00123:[1->1,1->5,2->3,3->2,3->8,3->9] | 1.00 | 201.831775701 | 23 | 107 | 0 | 1 |
| Path 358 | C00024->C00123:[50->8], C00022->C00123:[2->3,3->8] | 0.33 | 193.551020408 | 18 | 98 | 0 | 1 |
| Path 359 | C00024->C00123:[49->1,49->5,50->2,50->8,50->9], C00022->C00123:[1->1,1->5,3->2,3->3,3->8,3->9] | 1.00 | 294.234375 | 25 | 64 | 0 | 1 |
| Path 360 | C00024->C00123:[49->1,49->3,49->5,50->2,50->9], C00022->C00123:[1->1,1->3,1->5,3->2,3->9] | 0.83 | 427.268292683 | 21 | 41 | 0 | 1 |
| Path 361 | C00024->C00123:[49->1,49->5,50->2,50->8,50->9], C00022->C00123:[1->1,1->5,3->2,3->3,3->8,3->9] | 1.00 | 292.152542373 | 24 | 59 | 0 | 1 |
| Path 362 | C00024->C00123:[49->1,49->3,50->2] | 0.50 | 254.858823529 | 18 | 85 | 0 | 1 |
| Path 363 | C00024->C00123:[50->3,50->8] | 0.33 | 178.791666667 | 18 | 96 | 0 | 1 |
| Path 364 | C00024->C00123:[49->1,49->5,50->2,50->8,50->9], C00022->C00123:[1->1,1->5,2->3,3->2,3->8,3->9] | 1.00 | 316.571428571 | 17 | 42 | 0 | 1 |
| Path 365 | C00024->C00123:[49->5,50->8,50->9] | 0.50 | 285.341463415 | 15 | 41 | 0 | 0 |
| Path 366 | C00024->C00123:[49->1,50->2,50->8], C00022->C00123:[1->1,2->3,3->2,3->8] | 0.67 | 325.171428571 | 14 | 35 | 0 | 1 |
| Path 367 | C00024->C00123:[49->3,50->8], C00022->C00123:[1->3,3->8] | 0.33 | 279.388059701 | 16 | 67 | 0 | 1 |
| Path 368 | C00024->C00123:[49->5,50->8,50->9] | 0.50 | 535.866666667 | 17 | 30 | 0 | 0 |
| Path 369 | C00024->C00123:[49->1,50->2], C00022->C00123:[1->1,3->2,3->3] | 0.50 | 277.115384615 | 20 | 52 | 0 | 1 |
| Path 370 | C00024->C00123:[49->1,49->3,49->5,50->2,50->8,50->9], C00022->C00123:[1->1,1->3,1->5,3->2,3->8,3->9] | 1.00 | 313.171428571 | 20 | 35 | 0 | 1 |
| Path 371 | C00024->C00123:[50->8], C00022->C00123:[3->8] | 0.17 | 302.157894737 | 10 | 19 | 0 | 0 |
| Path 372 | C00024->C00123:[49->1,49->3,50->2,50->8], C00022->C00123:[1->1,1->3,3->2,3->8] | 0.67 | 437.235294118 | 18 | 34 | 0 | 1 |
| Path 373 | C00024->C00123:[49->3] | 0.17 | 239.642857143 | 10 | 28 | 0 | 1 |
| Path 374 | C00024->C00123:[49->1,50->2], C00022->C00123:[1->1,3->2,3->3] | 0.50 | 354.740740741 | 16 | 27 | 0 | 1 |
| Path 375 | C00024->C00123:[49->3,49->5,50->8,50->9], C00022->C00123:[1->3,1->5,3->8,3->9] | 0.67 | 269.527777778 | 16 | 36 | 0 | 1 |
| Path 376 | C00024->C00123:[49->1,49->3,49->5,50->2,50->8,50->9], C00022->C00123:[1->1,1->3,1->5,3->2,3->8,3->9] | 1.00 | 355.657142857 | 27 | 70 | 0 | 1 |
| Path 377 | C00024->C00123:[49->1,50->2,50->3,50->8] | 0.67 | 352.846153846 | 21 | 52 | 0 | 1 |
| Path 378 | C00024->C00123:[49->1,50->2], C00022->C00123:[1->1,2->3,3->2] | 0.50 | 387.979591837 | 21 | 49 | 0 | 1 |
| Path 379 | C00024->C00123:[49->1,50->2] | 0.33 | 504.48 | 15 | 25 | 0 | 0 |
| Path 380 | C00024->C00123:[49->1,49->5,50->2,50->9], C00022->C00123:[2->3] | 0.83 | 388.053571429 | 22 | 56 | 0 | 1 |
| Path 381 | C00024->C00123:[49->1,49->3,50->2,50->8] | 0.67 | 1105.56756757 | 18 | 37 | 0 | 1 |
| Path 382 | C00024->C00123:[49->1,50->2,50->8], C00022->C00123:[1->1,3->2,3->3,3->8] | 0.67 | 376.771428571 | 20 | 35 | 0 | 1 |
| Path 383 | C00024->C00123:[49->1,50->2], C00022->C00123:[2->3] | 0.50 | 449.717948718 | 17 | 39 | 0 | 1 |
| Path 384 | C00024->C00123:[49->1,50->2,50->8], C00022->C00123:[2->3] | 0.67 | 380.28125 | 14 | 32 | 0 | 1 |
| Path 385 | C00024->C00123:[49->1,50->2,50->8] | 0.50 | 254.710526316 | 13 | 38 | 0 | 0 |
| Path 386 | C00024->C00123:[49->1,49->3,49->5,50->2,50->8,50->9] | 1.00 | 289.277777778 | 21 | 72 | 0 | 1 |
| Path 387 | C00024->C00123:[49->1,49->5,50->2,50->9], C00022->C00123:[1->1,1->5,2->3,3->2,3->9] | 0.83 | 379.853333333 | 25 | 75 | 0 | 1 |
| Path 388 | C00024->C00123:[49->1,49->5,50->2,50->9], C00022->C00123:[2->3] | 0.83 | 208.431372549 | 21 | 102 | 0 | 1 |
| Path 389 | C00024->C00123:[49->1,49->5,50->2,50->8,50->9], C00022->C00123:[1->1,1->5,3->2,3->3,3->8,3->9] | 1.00 | 392.078947368 | 22 | 38 | 0 | 1 |
| Path 390 | C00024->C00123:[49->1,49->3,50->2], C00022->C00123:[2->3] | 0.50 | 414.583333333 | 22 | 36 | 0 | 1 |
| Path 391 | C00024->C00123:[49->1,49->5,50->2,50->9], C00022->C00123:[1->1,1->5,3->2,3->3,3->9] | 0.83 | 282.5 | 21 | 56 | 0 | 1 |
| Path 392 | C00024->C00123:[49->1,49->3,49->5,50->2,50->8,50->9] | 1.00 | 958.088888889 | 22 | 45 | 0 | 1 |
| Path 393 | C00024->C00123:[49->1,50->2,50->8], C00022->C00123:[3->3] | 0.67 | 452.642857143 | 20 | 28 | 0 | 1 |
| Path 394 | C00024->C00123:[49->3,50->8] | 0.33 | 277.620689655 | 13 | 29 | 0 | 1 |
| Path 395 | C00024->C00123:[49->1,49->3,50->2], C00022->C00123:[1->1,1->3,2->3,3->2] | 0.50 | 454.567567568 | 20 | 37 | 0 | 0 |
| Path 396 | C00024->C00123:[49->1,49->3,49->5,50->2,50->8,50->9], C00022->C00123:[1->1,1->3,1->5,3->2,3->8,3->9] | 1.00 | 462.048780488 | 22 | 41 | 0 | 1 |
| Path 397 | C00024->C00123:[49->1,49->5,50->2,50->8,50->9], C00022->C00123:[2->3] | 1.00 | 280.233333333 | 19 | 60 | 0 | 1 |
| Path 398 | C00024->C00123:[49->1,49->3,49->5,50->2,50->8,50->9], C00022->C00123:[1->1,1->3,1->5,3->2,3->8,3->9] | 1.00 | 280.881578947 | 21 | 76 | 0 | 1 |
| Path 399 | C00024->C00123:[49->1,49->3,50->2,50->8] | 0.67 | 317.125 | 17 | 64 | 0 | 1 |
| Path 400 | C00024->C00123:[49->1,50->2,50->8], C00022->C00123:[1->1,3->2,3->3,3->8] | 0.67 | 402.65625 | 20 | 32 | 0 | 1 |
| Path 401 | C00024->C00123:[50->3,50->8], C00022->C00123:[3->3,3->8] | 0.33 | 337.709090909 | 20 | 55 | 0 | 1 |
| Path 402 | C00024->C00123:[49->1,49->5,50->2,50->9], C00022->C00123:[2->3] | 0.83 | 270.122807018 | 16 | 57 | 0 | 1 |
| Path 403 | C00024->C00123:[49->1,50->2,50->8], C00022->C00123:[1->1,2->3,3->2,3->8] | 0.67 | 209.882352941 | 21 | 102 | 0 | 1 |
| Path 404 | C00024->C00123:[49->1,49->3,49->5,50->2,50->8,50->9] | 1.00 | 345.316666667 | 24 | 60 | 0 | 1 |
| Path 405 | C00024->C00123:[49->1,49->3,49->5,50->2,50->8,50->9] | 1.00 | 326.947368421 | 23 | 57 | 0 | 1 |
| Path 406 | C00024->C00123:[49->1,50->2,50->8], C00022->C00123:[1->1,2->3,3->2,3->8] | 0.67 | 210.689320388 | 22 | 103 | 0 | 1 |
| Path 407 | C00024->C00123:[49->1,49->3,50->2], C00022->C00123:[2->3] | 0.50 | 322.122807018 | 23 | 57 | 0 | 1 |
| Path 408 | C00024->C00123:[49->1,49->5,50->2,50->3,50->8,50->9] | 1.00 | 344.716666667 | 25 | 60 | 0 | 1 |
| Path 409 | C00024->C00123:[49->1,49->3,50->2] | 0.50 | 256.295454545 | 17 | 44 | 0 | 1 |
| Path 410 | C00024->C00123:[49->1,49->3,50->2,50->8] | 0.67 | 281.028571429 | 16 | 35 | 0 | 1 |
| Path 411 | C00024->C00123:[50->8], C00022->C00123:[3->3,3->8] | 0.33 | 521.1 | 22 | 40 | 0 | 0 |
| Path 412 | C00024->C00123:[49->3,49->5,50->8,50->9], C00022->C00123:[1->3,1->5,3->8,3->9] | 0.67 | 996.30952381 | 19 | 42 | 0 | 1 |
| Path 413 | C00024->C00123:[49->1,49->5,50->2,50->8,50->9], C00022->C00123:[1->1,1->5,3->2,3->3,3->8,3->9] | 1.00 | 359.431818182 | 25 | 44 | 0 | 1 |
| Path 414 | C00024->C00123:[50->8], C00022->C00123:[3->3,3->8] | 0.33 | 363.0 | 17 | 29 | 0 | 1 |
| Path 415 | C00024->C00123:[50->8], C00022->C00123:[3->3] | 0.33 | 481.347826087 | 16 | 23 | 0 | 0 |
| Path 416 | C00024->C00123:[49->5,50->8,50->9], C00022->C00123:[1->5,2->3,3->8,3->9] | 0.67 | 326.117647059 | 13 | 34 | 0 | 1 |
| Path 417 | C00024->C00123:[49->1,50->2,50->8], C00022->C00123:[1->1,3->2,3->8] | 0.50 | 295.296296296 | 14 | 27 | 0 | 0 |
| Path 418 | C00024->C00123:[49->3] | 0.17 | 500.380952381 | 10 | 21 | 0 | 1 |
| Path 419 | C00024->C00123:[49->1,49->5,50->2,50->3,50->9], C00022->C00123:[1->1,1->5,3->2,3->3,3->9] | 0.83 | 180.504672897 | 21 | 107 | 0 | 1 |
| Path 420 | C00024->C00123:[49->1,49->5,50->2,50->8,50->9], C00022->C00123:[1->1,1->5,2->3,3->2,3->8,3->9] | 1.00 | 200.407692308 | 26 | 130 | 0 | 1 |
| Path 421 | C00024->C00123:[49->1,50->2], C00022->C00123:[1->1,3->2] | 0.33 | 271.947368421 | 10 | 19 | 0 | 0 |
| Path 422 | C00024->C00123:[49->1,49->5,50->2,50->9], C00022->C00123:[1->1,1->5,3->2,3->3,3->9] | 0.83 | 285.475409836 | 22 | 61 | 0 | 1 |
| Path 423 | C00024->C00123:[49->1,49->3,49->5,50->2,50->8,50->9], C00022->C00123:[2->3] | 1.00 | 326.712121212 | 28 | 66 | 0 | 1 |
| Path 424 | C00024->C00123:[49->1,49->3,50->2,50->8], C00022->C00123:[1->1,1->3,3->2,3->8] | 0.67 | 281.623188406 | 18 | 69 | 0 | 1 |
| Path 425 | C00024->C00123:[49->1,49->3,49->5,50->2,50->8,50->9], C00022->C00123:[2->3] | 1.00 | 413.325 | 26 | 40 | 0 | 1 |
| Path 426 | C00024->C00123:[49->1,49->5,50->2,50->8,50->9], C00022->C00123:[1->1,1->5,3->2,3->3,3->8,3->9] | 1.00 | 296.169230769 | 26 | 65 | 0 | 1 |
| Path 427 | C00024->C00123:[49->1,49->3,49->5,50->2,50->9], C00022->C00123:[1->1,1->3,1->5,3->2,3->9] | 0.83 | 380.583333333 | 18 | 36 | 0 | 1 |
| Path 428 | C00024->C00123:[49->1,49->5,50->2,50->8,50->9], C00022->C00123:[3->3] | 1.00 | 390.647058824 | 23 | 34 | 0 | 1 |
| Path 429 | C00024->C00123:[49->1,50->2,50->8], C00022->C00123:[3->3] | 0.67 | 428.264705882 | 22 | 34 | 0 | 1 |
| Path 430 | C00024->C00123:[49->1,49->3,50->2,50->8] | 0.67 | 352.47826087 | 16 | 23 | 0 | 1 |
| Path 431 | C00024->C00123:[49->1,50->2,50->8] | 0.50 | 271.52173913 | 16 | 46 | 0 | 0 |
| Path 432 | C00024->C00123:[49->1,49->5,50->2,50->8,50->9], C00022->C00123:[2->3] | 1.00 | 336.646341463 | 28 | 82 | 0 | 1 |
| Path 433 | C00024->C00123:[49->1,49->5,50->2,50->8,50->9], C00022->C00123:[2->3] | 1.00 | 339.957746479 | 27 | 71 | 0 | 1 |
| Path 434 | C00024->C00123:[49->1,49->5,50->2,50->3,50->9] | 0.83 | 336.535714286 | 21 | 56 | 0 | 1 |
| Path 435 | C00024->C00123:[49->1,49->5,50->2,50->8,50->9], C00022->C00123:[1->1,1->5,3->2,3->3,3->8,3->9] | 1.00 | 390.857142857 | 22 | 35 | 0 | 1 |
| Path 436 | C00022->C00123:[3->3] | 0.17 | 285.409090909 | 14 | 44 | 0 | 1 |
| Path 437 | C00024->C00123:[49->1,50->2] | 0.33 | 302.0 | 9 | 14 | 0 | 0 |
| Path 438 | C00024->C00123:[49->3,49->5,50->8,50->9], C00022->C00123:[1->3,1->5,3->8,3->9] | 0.67 | 332.153846154 | 15 | 26 | 0 | 1 |
| Path 439 | C00024->C00123:[49->1,49->5,50->2,50->3,50->8,50->9] | 1.00 | 302.891566265 | 28 | 83 | 0 | 1 |
| Path 440 | C00024->C00123:[49->1,49->5,50->2,50->8,50->9], C00022->C00123:[3->3] | 1.00 | 380.421052632 | 23 | 38 | 0 | 1 |
| Path 441 | C00024->C00123:[49->3] | 0.17 | 301.228070175 | 11 | 57 | 0 | 1 |
| Path 442 | C00024->C00123:[49->1,49->3,49->5,50->2,50->8,50->9], C00022->C00123:[1->1,1->3,1->5,3->2,3->8,3->9] | 1.00 | 273.523809524 | 19 | 42 | 0 | 1 |
| Path 443 | C00024->C00123:[49->1,49->3,50->2,50->8] | 0.67 | 480.24137931 | 17 | 29 | 0 | 1 |
| Path 444 | C00024->C00123:[49->1,49->3,49->5,50->2,50->8,50->9], C00022->C00123:[1->1,1->3,1->5,3->2,3->8,3->9] | 1.00 | 326.413793103 | 18 | 29 | 0 | 1 |
| Path 445 | C00024->C00123:[49->1,49->3,49->5,50->2,50->9], C00022->C00123:[2->3] | 0.83 | 365.428571429 | 16 | 28 | 0 | 0 |
| Path 446 | C00024->C00123:[49->1,49->5,50->2,50->9], C00022->C00123:[1->1,1->5,2->3,3->2,3->9] | 0.83 | 193.984251969 | 23 | 127 | 0 | 1 |
| Path 447 | C00024->C00123:[49->1,49->5,50->2,50->8,50->9], C00022->C00123:[2->3] | 1.00 | 368.355555556 | 21 | 45 | 0 | 1 |
| Path 448 | C00024->C00123:[50->8], C00022->C00123:[3->3,3->8] | 0.33 | 367.0625 | 17 | 32 | 0 | 1 |
| Path 449 | C00024->C00123:[49->1,49->5,50->2,50->9], C00022->C00123:[1->1,1->5,3->2,3->9] | 0.67 | 426.0 | 18 | 36 | 0 | 0 |
| Path 450 | C00024->C00123:[49->5,50->8,50->9], C00022->C00123:[1->5,3->3,3->8,3->9] | 0.67 | 376.771428571 | 20 | 35 | 0 | 1 |
| Path 451 | C00024->C00123:[49->1,50->2,50->8], C00022->C00123:[3->3] | 0.67 | 328.06 | 22 | 50 | 0 | 1 |
| Path 452 | C00024->C00123:[49->3,49->5,50->8,50->9], C00022->C00123:[1->3,1->5,3->8,3->9] | 0.67 | 437.235294118 | 18 | 34 | 0 | 1 |
| Path 453 | C00024->C00123:[49->1,50->2], C00022->C00123:[3->3] | 0.50 | 315.283018868 | 21 | 53 | 0 | 1 |
| Path 454 | C00024->C00123:[49->1,49->5,50->2,50->8,50->9], C00022->C00123:[3->3] | 1.00 | 431.631578947 | 24 | 38 | 0 | 1 |
| Path 455 | C00024->C00123:[49->1,49->5,50->2,50->9], C00022->C00123:[2->3] | 0.83 | 226.877697842 | 26 | 139 | 0 | 1 |
| Path 456 | C00024->C00123:[49->1,49->3,50->2,50->8] | 0.67 | 486.928571429 | 16 | 28 | 0 | 1 |
| Path 457 | C00024->C00123:[49->1,49->5,50->2,50->9], C00022->C00123:[1->1,1->5,3->2,3->3,3->9] | 0.83 | 371.75 | 20 | 36 | 0 | 1 |
| Path 458 | C00024->C00123:[49->1,50->2,50->8], C00022->C00123:[3->3] | 0.67 | 442.444444444 | 19 | 27 | 0 | 1 |
| Path 459 | C00024->C00123:[49->1,49->5,50->2,50->8,50->9], C00022->C00123:[3->3] | 1.00 | 323.068965517 | 26 | 58 | 0 | 1 |
| Path 460 | C00024->C00123:[49->1,49->5,50->2,50->8,50->9], C00022->C00123:[2->3] | 1.00 | 392.8 | 26 | 60 | 0 | 1 |
| Path 461 | C00024->C00123:[49->1,49->3,50->2], C00022->C00123:[1->1,1->3,2->3,3->2] | 0.50 | 347.62962963 | 15 | 27 | 0 | 0 |
| Path 462 | C00024->C00123:[49->1,49->3,49->5,50->2,50->8,50->9] | 1.00 | 250.903225806 | 22 | 62 | 0 | 1 |
| Path 463 | C00024->C00123:[49->1,49->3,49->5,50->2,50->9], C00022->C00123:[1->1,1->3,1->5,3->2,3->9] | 0.83 | 432.318181818 | 21 | 44 | 0 | 1 |
| Path 464 | C00024->C00123:[49->1,49->5,50->2,50->9] | 0.67 | 464.419354839 | 17 | 31 | 0 | 0 |
| Path 465 | C00024->C00123:[49->1,49->3,49->5,50->2,50->8,50->9], C00022->C00123:[1->1,1->3,1->5,3->2,3->8,3->9] | 1.00 | 434.5625 | 25 | 48 | 0 | 1 |
| Path 466 | C00024->C00123:[49->1,49->5,50->2,50->8,50->9], C00022->C00123:[1->1,1->5,2->3,3->2,3->8,3->9] | 1.00 | 362.693548387 | 25 | 62 | 0 | 1 |
| Path 467 | C00024->C00123:[49->1,49->5,50->2,50->8,50->9], C00022->C00123:[1->1,1->5,3->2,3->3,3->8,3->9] | 1.00 | 403.279069767 | 25 | 43 | 0 | 1 |
| Path 468 | C00024->C00123:[49->1,50->2,50->8], C00022->C00123:[2->3] | 0.67 | 463.325 | 18 | 40 | 0 | 1 |
| Path 469 | C00024->C00123:[49->1,50->2,50->8], C00022->C00123:[3->3] | 0.67 | 307.529411765 | 20 | 51 | 0 | 1 |
| Path 470 | C00024->C00123:[49->1,49->3,50->2] | 0.50 | 260.058823529 | 15 | 34 | 0 | 1 |
| Path 471 | C00022->C00123:[2->3] | 0.17 | 319.227272727 | 6 | 22 | 0 | 1 |
| Path 472 | C00024->C00123:[49->1,49->5,50->2,50->8,50->9], C00022->C00123:[2->3] | 1.00 | 282.524590164 | 20 | 61 | 0 | 1 |
| Path 473 | C00024->C00123:[49->1,49->3,50->2], C00022->C00123:[1->1,1->3,3->2] | 0.50 | 247.529411765 | 14 | 34 | 0 | 1 |
| Path 474 | C00024->C00123:[49->1,50->2], C00022->C00123:[2->3] | 0.50 | 357.642857143 | 12 | 28 | 0 | 0 |
| Path 475 | C00024->C00123:[49->1,49->5,50->2,50->9], C00022->C00123:[2->3] | 0.83 | 321.764705882 | 13 | 34 | 0 | 1 |
| Path 476 | C00024->C00123:[50->8], C00022->C00123:[2->3,3->8] | 0.33 | 323.272727273 | 12 | 33 | 0 | 1 |
| Path 477 | C00024->C00123:[49->1,50->2], C00022->C00123:[1->1,2->3,3->2] | 0.50 | 362.788461538 | 19 | 52 | 0 | 1 |
| Path 478 | C00024->C00123:[49->1,49->3,50->2,50->8] | 0.67 | 288.953125 | 17 | 64 | 0 | 1 |
| Path 479 | C00024->C00123:[49->1,49->5,50->2,50->9] | 0.67 | 300.65 | 11 | 20 | 0 | 0 |
| Path 480 | C00024->C00123:[50->8], C00022->C00123:[2->3] | 0.33 | 498.073170732 | 17 | 41 | 0 | 0 |
| Path 481 | C00024->C00123:[49->1,50->2,50->8], C00022->C00123:[2->3] | 0.67 | 208.421487603 | 24 | 121 | 0 | 1 |
| Path 482 | C00024->C00123:[49->1,49->3,49->5,50->2,50->8,50->9] | 1.00 | 250.015151515 | 23 | 66 | 0 | 1 |
| Path 483 | C00024->C00123:[49->1,50->2,50->8], C00022->C00123:[1->1,2->3,3->2,3->8] | 0.67 | 196.243902439 | 23 | 123 | 0 | 1 |
| Path 484 | C00024->C00123:[49->1,50->2], C00022->C00123:[2->3] | 0.50 | 202.864583333 | 19 | 96 | 0 | 1 |
| Path 485 | C00024->C00123:[49->1,49->5,50->2,50->8,50->9], C00022->C00123:[1->1,1->5,3->2,3->3,3->8,3->9] | 1.00 | 294.283333333 | 25 | 60 | 0 | 1 |
| Path 486 | C00024->C00123:[50->8], C00022->C00123:[3->3,3->8] | 0.33 | 392.09375 | 19 | 32 | 0 | 1 |
| Path 487 | C00024->C00123:[49->1,49->5,50->2,50->8,50->9], C00022->C00123:[1->1,1->5,2->3,3->2,3->8,3->9] | 1.00 | 216.540540541 | 26 | 111 | 0 | 1 |
| Path 488 | C00024->C00123:[49->1,49->5,50->2,50->8,50->9], C00022->C00123:[3->3] | 1.00 | 305.2 | 25 | 60 | 0 | 1 |
| Path 489 | C00024->C00123:[50->8] | 0.17 | 401.294117647 | 11 | 17 | 0 | 0 |
| Path 490 | C00024->C00123:[49->1,50->2,50->8], C00022->C00123:[3->3] | 0.67 | 473.133333333 | 20 | 30 | 0 | 1 |
| Path 491 | C00024->C00123:[49->1,49->5,50->2,50->8,50->9], C00022->C00123:[1->1,1->5,3->2,3->3,3->8,3->9] | 1.00 | 298.896551724 | 23 | 58 | 0 | 1 |
| Path 492 | C00024->C00123:[49->1,50->2,50->8], C00022->C00123:[3->3] | 0.67 | 427.322580645 | 19 | 31 | 0 | 1 |
| Path 493 | C00024->C00123:[49->1,50->2,50->8] | 0.50 | 348.133333333 | 10 | 15 | 0 | 0 |
| Path 494 | C00024->C00123:[49->1,49->3,49->5,50->2,50->8,50->9], C00022->C00123:[1->1,1->3,1->5,3->2,3->8,3->9] | 1.00 | 270.186046512 | 19 | 43 | 0 | 1 |
| Path 495 | C00024->C00123:[49->1,50->2,50->8], C00022->C00123:[1->1,3->2,3->3,3->8] | 0.67 | 362.580645161 | 19 | 31 | 0 | 1 |
| Path 496 | C00024->C00123:[50->8], C00022->C00123:[2->3] | 0.33 | 367.961538462 | 10 | 26 | 0 | 0 |
| Path 497 | C00024->C00123:[49->1,49->5,50->2,50->8,50->9], C00022->C00123:[3->3] | 1.00 | 301.814814815 | 23 | 54 | 0 | 1 |
| Path 498 | C00024->C00123:[49->1,50->2,50->8], C00022->C00123:[3->3] | 0.67 | 408.266666667 | 19 | 30 | 0 | 1 |
| Path 499 | C00024->C00123:[49->1,49->3,49->5,50->2,50->9] | 0.83 | 710.061538462 | 22 | 65 | 0 | 1 |
| Path 500 | C00024->C00123:[49->1,49->5,50->2,50->8,50->9], C00022->C00123:[1->1,1->5,3->2,3->3,3->8,3->9] | 1.00 | 348.076923077 | 23 | 39 | 0 | 1 |
| Path 501 | C00024->C00123:[49->1,49->3,50->2], C00022->C00123:[1->1,1->3,3->2] | 0.50 | 270.820895522 | 16 | 67 | 0 | 1 |
| Path 502 | C00024->C00123:[49->3,49->5,50->8,50->9], C00022->C00123:[1->3,1->5,3->8,3->9] | 0.67 | 441.606060606 | 17 | 33 | 0 | 1 |
| Path 503 | C00024->C00123:[49->3,49->5,50->8,50->9], C00022->C00123:[1->3,1->5,3->8,3->9] | 0.67 | 333.72 | 14 | 25 | 0 | 1 |
| Path 504 | C00024->C00123:[49->1,49->5,50->2,50->8,50->9], C00022->C00123:[1->1,1->5,2->3,3->2,3->8,3->9] | 1.00 | 376.359375 | 26 | 64 | 0 | 1 |
| Path 505 | C00024->C00123:[50->8], C00022->C00123:[3->3,3->8] | 0.33 | 405.733333333 | 18 | 30 | 0 | 1 |
| Path 506 | C00022->C00123:[3->3] | 0.17 | 340.043478261 | 12 | 23 | 0 | 1 |
| Path 507 | C00024->C00123:[49->1,49->5,50->2,50->8,50->9], C00022->C00123:[3->3] | 1.00 | 303.963636364 | 24 | 55 | 0 | 1 |
| Path 508 | C00024->C00123:[49->1,50->2], C00022->C00123:[1->1,2->3,3->2] | 0.50 | 444.326086957 | 19 | 46 | 0 | 0 |
| Path 509 | C00024->C00123:[49->5,50->8,50->9], C00022->C00123:[1->5,3->3,3->8,3->9] | 0.67 | 391.324324324 | 21 | 37 | 0 | 1 |
| Path 510 | C00024->C00123:[49->1,50->2], C00022->C00123:[2->3] | 0.50 | 371.5 | 16 | 36 | 0 | 1 |
| Path 511 | C00024->C00123:[49->5,50->8,50->9], C00022->C00123:[1->5,2->3,3->8,3->9] | 0.67 | 388.98245614 | 23 | 57 | 0 | 1 |
| Path 512 | C00024->C00123:[49->1,49->5,50->2,50->8,50->9], C00022->C00123:[1->1,1->5,3->2,3->3,3->8,3->9] | 1.00 | 296.169230769 | 26 | 65 | 0 | 1 |
| Path 513 | C00024->C00123:[49->1,49->5,50->2,50->8,50->9], C00022->C00123:[3->3] | 1.00 | 303.254237288 | 24 | 59 | 0 | 1 |
| Path 514 | C00024->C00123:[49->1,50->2,50->8], C00022->C00123:[1->1,3->2,3->3,3->8] | 0.67 | 383.419354839 | 20 | 31 | 0 | 1 |
| Path 515 | C00024->C00123:[49->1,49->5,50->2,50->8,50->9], C00022->C00123:[1->1,1->5,2->3,3->2,3->8,3->9] | 1.00 | 409.259259259 | 24 | 54 | 0 | 1 |
| Path 516 | C00024->C00123:[49->1,49->5,50->2,50->8,50->9], C00022->C00123:[1->1,1->5,2->3,3->2,3->8,3->9] | 1.00 | 202.083969466 | 27 | 131 | 0 | 1 |
| Path 517 | C00024->C00123:[49->1,49->3,49->5,50->2,50->8,50->9] | 1.00 | 314.02739726 | 22 | 73 | 0 | 1 |
| Path 518 | C00024->C00123:[49->3,50->8], C00022->C00123:[1->3,3->8] | 0.33 | 416.333333333 | 16 | 30 | 0 | 1 |
| Path 519 | C00024->C00123:[49->1,49->3,49->5,50->2,50->8,50->9], C00022->C00123:[1->1,1->3,1->5,3->2,3->8,3->9] | 1.00 | 309.126760563 | 20 | 71 | 0 | 1 |
| Path 520 | C00024->C00123:[49->1,49->5,50->2,50->8,50->9], C00022->C00123:[1->1,1->5,2->3,3->2,3->8,3->9] | 1.00 | 389.037735849 | 25 | 53 | 0 | 1 |
| Path 521 | C00024->C00123:[49->3,50->8] | 0.33 | 462.04 | 15 | 25 | 0 | 1 |
| Path 522 | C00024->C00123:[50->8], C00022->C00123:[3->3,3->8] | 0.33 | 428.928571429 | 17 | 28 | 0 | 0 |
| Path 523 | C00024->C00123:[49->1,50->2], C00022->C00123:[2->3] | 0.50 | 415.065217391 | 22 | 46 | 0 | 1 |
| Path 524 | C00024->C00123:[49->3,50->8] | 0.33 | 352.095238095 | 14 | 21 | 0 | 1 |
| Path 525 | C00024->C00123:[49->1,49->3,49->5,50->2,50->8,50->9], C00022->C00123:[1->1,1->3,1->5,3->2,3->8,3->9] | 1.00 | 436.742857143 | 19 | 35 | 0 | 1 |
| Path 526 | C00024->C00123:[50->8], C00022->C00123:[2->3,3->8] | 0.33 | 390.163636364 | 21 | 55 | 0 | 1 |
| Path 527 | C00024->C00123:[49->1,49->3,49->5,50->2,50->8,50->9], C00022->C00123:[2->3] | 1.00 | 446.066666667 | 28 | 45 | 0 | 1 |
| Path 528 | C00024->C00123:[49->1,49->3,49->5,50->2,50->8,50->9], C00022->C00123:[1->1,1->3,1->5,3->2,3->8,3->9] | 1.00 | 270.479166667 | 21 | 48 | 0 | 1 |
| Path 529 | C00024->C00123:[49->1,49->3,49->5,50->2,50->9] | 0.83 | 261.24 | 19 | 50 | 0 | 1 |
| Path 530 | C00024->C00123:[49->1,50->2,50->8], C00022->C00123:[1->1,2->3,3->2,3->8] | 0.67 | 209.882352941 | 21 | 102 | 0 | 1 |
| Path 531 | C00024->C00123:[49->1,50->2,50->8], C00022->C00123:[1->1,3->2,3->3,3->8] | 0.67 | 386.433333333 | 19 | 30 | 0 | 1 |
| Path 532 | C00024->C00123:[49->1,49->5,50->2,50->9], C00022->C00123:[1->1,1->5,3->2,3->3,3->9] | 0.83 | 285.475409836 | 22 | 61 | 0 | 1 |
| Path 533 | C00024->C00123:[49->1,49->5,50->2,50->9], C00022->C00123:[2->3] | 0.83 | 429.422222222 | 19 | 45 | 0 | 1 |
| Path 534 | C00024->C00123:[49->1,50->2,50->8], C00022->C00123:[1->1,2->3,3->2,3->8] | 0.67 | 373.185185185 | 21 | 54 | 0 | 1 |
| Path 535 | C00024->C00123:[49->1,49->5,50->2,50->3,50->8,50->9], C00022->C00123:[1->1,1->5,3->2,3->3,3->8,3->9] | 1.00 | 332.646153846 | 26 | 65 | 0 | 1 |
| Path 536 | C00024->C00123:[49->1,50->2], C00022->C00123:[1->1,2->3,3->2] | 0.50 | 440.489795918 | 21 | 49 | 0 | 0 |
| Path 537 | C00024->C00123:[49->1,49->3,49->5,50->2,50->9], C00022->C00123:[1->1,1->3,1->5,3->2,3->9] | 0.83 | 255.025 | 16 | 40 | 0 | 1 |
| Path 538 | C00022->C00123:[3->3] | 0.17 | 396.608695652 | 13 | 23 | 0 | 1 |
| Path 539 | C00024->C00123:[49->5,50->8,50->9], C00022->C00123:[1->5,3->8,3->9] | 0.50 | 484.138888889 | 19 | 36 | 0 | 0 |
| Path 540 | C00024->C00123:[49->1,50->2,50->8], C00022->C00123:[1->1,3->2,3->8] | 0.50 | 355.695652174 | 13 | 23 | 0 | 0 |
| Path 541 | C00024->C00123:[49->1,49->3,49->5,50->2,50->9], C00022->C00123:[1->1,1->3,1->5,3->2,3->9] | 0.83 | 298.25 | 17 | 32 | 0 | 1 |
| Path 542 | C00024->C00123:[50->8], C00022->C00123:[2->3,3->8] | 0.33 | 207.801980198 | 20 | 101 | 0 | 1 |
| Path 543 | C00024->C00123:[49->1,50->2,50->8], C00022->C00123:[2->3] | 0.67 | 408.326923077 | 22 | 52 | 0 | 1 |
| Path 544 | C00024->C00123:[49->1,49->3,50->2,50->8], C00022->C00123:[1->1,1->3,3->2,3->8] | 0.67 | 323.071428571 | 17 | 28 | 0 | 1 |
| Path 545 | C00024->C00123:[49->3,49->5,50->8,50->9], C00022->C00123:[1->3,1->5,3->8,3->9] | 0.67 | 281.455882353 | 17 | 68 | 0 | 1 |
| Path 546 | C00024->C00123:[50->3] | 0.17 | 339.295454545 | 14 | 44 | 0 | 1 |
| Path 547 | C00024->C00123:[50->8], C00022->C00123:[3->3] | 0.33 | 493.5625 | 21 | 32 | 0 | 1 |
| Path 548 | C00024->C00123:[49->1,49->3,50->2,50->8], C00022->C00123:[1->1,1->3,3->2,3->8] | 0.67 | 324.185185185 | 16 | 27 | 0 | 1 |
| Path 549 | C00024->C00123:[49->1,49->3,50->2] | 0.50 | 468.148148148 | 15 | 27 | 0 | 1 |
| Path 550 | C00024->C00123:[49->1,49->3,49->5,50->2,50->8,50->9] | 1.00 | 946.391304348 | 23 | 46 | 0 | 1 |
| Path 551 | C00024->C00123:[49->1,49->5,50->2,50->3,50->8,50->9], C00022->C00123:[1->1,1->5,3->2,3->3,3->8,3->9] | 1.00 | 339.775862069 | 23 | 58 | 0 | 1 |
| Path 552 | C00022->C00123:[2->3] | 0.17 | 363.760869565 | 14 | 46 | 0 | 1 |
| Path 553 | C00024->C00123:[49->1,49->5,50->2,50->8,50->9], C00022->C00123:[1->1,1->5,2->3,3->2,3->8,3->9] | 1.00 | 316.988235294 | 28 | 85 | 0 | 1 |
| Path 554 | C00024->C00123:[49->1,49->5,50->2,50->8,50->9], C00022->C00123:[3->3] | 1.00 | 309.267857143 | 25 | 56 | 0 | 1 |
| Path 555 | C00024->C00123:[49->1,49->3,49->5,50->2,50->8,50->9], C00022->C00123:[1->1,1->3,1->5,3->2,3->8,3->9] | 1.00 | 282.688311688 | 22 | 77 | 0 | 1 |
| Path 556 | C00024->C00123:[49->1,50->2,50->8], C00022->C00123:[2->3] | 0.67 | 377.914285714 | 14 | 35 | 0 | 0 |
| Path 557 | C00024->C00123:[49->1,50->2], C00022->C00123:[1->1,2->3,3->2] | 0.50 | 386.782608696 | 21 | 46 | 0 | 1 |
| Path 558 | C00024->C00123:[49->1,49->3,50->2], C00022->C00123:[1->1,1->3,2->3,3->2] | 0.50 | 384.733333333 | 24 | 60 | 0 | 1 |
| Path 559 | C00024->C00123:[50->8], C00022->C00123:[3->3,3->8] | 0.33 | 376.0 | 16 | 27 | 0 | 1 |
| Path 560 | C00024->C00123:[49->1,50->2], C00022->C00123:[3->3] | 0.50 | 402.5 | 17 | 24 | 0 | 1 |
| Path 561 | C00024->C00123:[49->1,50->2,50->8], C00022->C00123:[3->3] | 0.67 | 429.571428571 | 19 | 28 | 0 | 1 |
| Path 562 | C00024->C00123:[49->1,50->2,50->8], C00022->C00123:[1->1,3->2,3->3,3->8] | 0.67 | 402.65625 | 20 | 32 | 0 | 1 |
| Path 563 | C00024->C00123:[49->1,50->2,50->8], C00022->C00123:[1->1,3->2,3->3,3->8] | 0.67 | 390.0 | 21 | 34 | 0 | 1 |
| Path 564 | C00024->C00123:[49->1,50->2,50->8], C00022->C00123:[2->3] | 0.67 | 211.857142857 | 21 | 98 | 0 | 1 |
| Path 565 | C00024->C00123:[49->1,50->2,50->8] | 0.50 | 243.488372093 | 14 | 43 | 0 | 0 |
| Path 566 | C00024->C00123:[49->1,49->3,49->5,50->2,50->8,50->9], C00022->C00123:[1->1,1->3,1->5,3->2,3->8,3->9] | 1.00 | 350.176470588 | 28 | 68 | 0 | 1 |
| Path 567 | C00024->C00123:[49->3] | 0.17 | 465.526315789 | 10 | 19 | 0 | 1 |
| Path 568 | C00024->C00123:[49->1,49->3,49->5,50->2,50->8,50->9], C00022->C00123:[1->1,1->3,1->5,3->2,3->8,3->9] | 1.00 | 388.45 | 22 | 40 | 0 | 1 |
| Path 569 | C00024->C00123:[49->1,49->3,49->5,50->2,50->8,50->9], C00022->C00123:[1->1,1->3,1->5,3->2,3->8,3->9] | 1.00 | 430.111111111 | 25 | 45 | 0 | 1 |
| Path 570 | C00024->C00123:[49->1,49->3,50->2], C00022->C00123:[1->1,1->3,3->2] | 0.50 | 453.605263158 | 19 | 38 | 0 | 1 |
| Path 571 | C00024->C00123:[49->1,49->3,50->2,50->8] | 0.67 | 370.2 | 13 | 20 | 0 | 1 |
| Path 572 | C00024->C00123:[49->1,49->3,49->5,50->2,50->8,50->9], C00022->C00123:[1->1,1->3,1->5,3->2,3->8,3->9] | 1.00 | 322.676470588 | 19 | 34 | 0 | 1 |
| Path 573 | C00024->C00123:[49->1,49->5,50->2,50->9], C00022->C00123:[1->1,1->5,3->2,3->3,3->9] | 0.83 | 340.973684211 | 19 | 38 | 0 | 1 |
| Path 574 | C00024->C00123:[49->1,50->2,50->8], C00022->C00123:[3->3] | 0.67 | 491.333333333 | 22 | 33 | 0 | 1 |
| Path 575 | C00024->C00123:[49->1,50->2], C00022->C00123:[3->3] | 0.50 | 291.022222222 | 18 | 45 | 0 | 1 |
| Path 576 | C00024->C00123:[49->3,49->5,50->8,50->9], C00022->C00123:[1->3,1->5,3->8,3->9] | 0.67 | 460.0 | 21 | 37 | 0 | 1 |
| Path 577 | C00024->C00123:[49->1,49->3,50->2] | 0.50 | 277.516129032 | 15 | 62 | 0 | 1 |
| Path 578 | C00024->C00123:[49->1,49->5,50->2,50->9], C00022->C00123:[1->1,1->5,3->2,3->9] | 0.67 | 295.296296296 | 14 | 27 | 0 | 0 |
| Path 579 | C00024->C00123:[49->1,49->5,50->2,50->8,50->9], C00022->C00123:[1->1,1->5,2->3,3->2,3->8,3->9] | 1.00 | 361.754098361 | 24 | 61 | 0 | 1 |
| Path 580 | C00024->C00123:[49->1,50->2,50->8], C00022->C00123:[1->1,2->3,3->2,3->8] | 0.67 | 388.98245614 | 23 | 57 | 0 | 1 |
| Path 581 | C00024->C00123:[49->1,49->3,49->5,50->2,50->8,50->9], C00022->C00123:[1->1,1->3,1->5,3->2,3->8,3->9] | 1.00 | 316.138888889 | 21 | 36 | 0 | 1 |
| Path 582 | C00024->C00123:[50->8], C00022->C00123:[3->3,3->8] | 0.33 | 432.735294118 | 20 | 34 | 0 | 1 |
| Path 583 | C00024->C00123:[49->1,50->2,50->3] | 0.50 | 341.22 | 19 | 50 | 0 | 1 |
| Path 584 | C00024->C00123:[49->3,49->5,50->8,50->9], C00022->C00123:[1->3,1->5,3->8,3->9] | 0.67 | 464.638888889 | 20 | 36 | 0 | 1 |
| Path 585 | C00024->C00123:[49->5,50->8,50->9], C00022->C00123:[1->5,3->8,3->9] | 0.50 | 355.695652174 | 13 | 23 | 0 | 0 |
| Path 586 | C00022->C00123:[3->3] | 0.17 | 347.692307692 | 12 | 26 | 0 | 1 |
| Path 587 | C00024->C00123:[50->8], C00022->C00123:[2->3,3->8] | 0.33 | 433.0 | 18 | 44 | 0 | 1 |
| Path 588 | C00024->C00123:[49->1,49->3,49->5,50->2,50->8,50->9] | 1.00 | 278.634615385 | 21 | 52 | 0 | 1 |
| Path 589 | C00024->C00123:[49->1,49->3,49->5,50->2,50->8,50->9], C00022->C00123:[1->1,1->3,1->5,3->2,3->8,3->9] | 1.00 | 304.454545455 | 22 | 77 | 0 | 1 |
| Path 590 | C00024->C00123:[49->1,49->5,50->2,50->9], C00022->C00123:[1->1,1->5,3->2,3->3,3->9] | 0.83 | 344.333333333 | 18 | 33 | 0 | 1 |
| Path 591 | C00024->C00123:[49->1,50->2], C00022->C00123:[2->3] | 0.50 | 201.109243697 | 22 | 119 | 0 | 1 |
| Path 592 | C00024->C00123:[49->1,49->5,50->2,50->8,50->9], C00022->C00123:[3->3] | 1.00 | 322.73015873 | 27 | 63 | 0 | 1 |
| Path 593 | C00024->C00123:[49->1,49->5,50->2,50->8,50->9], C00022->C00123:[1->1,1->5,3->2,3->3,3->8,3->9] | 1.00 | 352.238095238 | 23 | 42 | 0 | 1 |
| Path 594 | C00024->C00123:[50->8], C00022->C00123:[2->3] | 0.33 | 208.84375 | 19 | 96 | 0 | 1 |
| Path 595 | C00024->C00123:[49->5,50->3,50->8,50->9], C00022->C00123:[1->5,3->3,3->8,3->9] | 0.67 | 339.178571429 | 21 | 56 | 0 | 1 |
| Path 596 | C00024->C00123:[49->1,49->3,49->5,50->2,50->8,50->9] | 1.00 | 328.551724138 | 24 | 58 | 0 | 1 |
| Path 597 | C00024->C00123:[49->1,49->5,50->2,50->9], C00022->C00123:[2->3] | 0.83 | 401.5 | 24 | 52 | 0 | 1 |
| Path 598 | C00024->C00123:[49->5,50->8,50->9], C00022->C00123:[1->5,3->3,3->8,3->9] | 0.67 | 402.65625 | 20 | 32 | 0 | 1 |
| Path 599 | C00024->C00123:[49->1,50->2], C00022->C00123:[1->1,2->3,3->2] | 0.50 | 361.277777778 | 14 | 36 | 0 | 0 |
| Path 600 | C00024->C00123:[49->1,50->2], C00022->C00123:[1->1,2->3,3->2] | 0.50 | 419.954545455 | 18 | 44 | 0 | 1 |
| Path 601 | C00024->C00123:[49->1,49->5,50->2,50->8,50->9], C00022->C00123:[1->1,1->5,2->3,3->2,3->8,3->9] | 1.00 | 409.056603774 | 23 | 53 | 0 | 1 |
| Path 602 | C00024->C00123:[49->1,49->3,49->5,50->2,50->9], C00022->C00123:[1->1,1->3,1->5,3->2,3->9] | 0.83 | 304.466666667 | 15 | 30 | 0 | 1 |
| Path 603 | C00024->C00123:[49->3,50->8], C00022->C00123:[1->3,3->8] | 0.33 | 468.710526316 | 19 | 38 | 0 | 1 |
| Path 604 | C00024->C00123:[49->1,49->5,50->2,50->8,50->9], C00022->C00123:[1->1,1->5,3->2,3->3,3->8,3->9] | 1.00 | 363.184210526 | 23 | 38 | 0 | 1 |
| Path 605 | C00024->C00123:[49->1,50->2], C00022->C00123:[1->1,3->2,3->3] | 0.50 | 386.6 | 18 | 30 | 0 | 1 |
| Path 606 | C00024->C00123:[49->1,49->3,50->2,50->8] | 0.67 | 281.361111111 | 17 | 36 | 0 | 1 |
| Path 607 | C00024->C00123:[49->1,50->2], C00022->C00123:[3->3] | 0.50 | 314.916666667 | 20 | 48 | 0 | 1 |
| Path 608 | C00024->C00123:[49->1,49->5,50->2,50->8,50->9], C00022->C00123:[2->3] | 1.00 | 211.984375 | 27 | 128 | 0 | 1 |
| Path 609 | C00024->C00123:[49->1,49->3,49->5,50->2,50->8,50->9] | 1.00 | 345.931034483 | 18 | 29 | 0 | 1 |
| Path 610 | C00024->C00123:[49->1,49->5,50->2,50->3,50->8,50->9], C00022->C00123:[1->1,1->5,3->2,3->3,3->8,3->9] | 1.00 | 188.463636364 | 24 | 110 | 0 | 1 |
| Path 611 | C00024->C00123:[49->1,49->3,50->2], C00022->C00123:[1->1,1->3,3->2] | 0.50 | 306.208333333 | 13 | 24 | 0 | 1 |
| Path 612 | C00024->C00123:[50->8], C00022->C00123:[2->3,3->8] | 0.33 | 193.595041322 | 21 | 121 | 0 | 1 |
| Path 613 | C00024->C00123:[49->1,49->5,50->2,50->8,50->9], C00022->C00123:[3->3] | 1.00 | 408.028571429 | 23 | 35 | 0 | 1 |
| Path 614 | C00024->C00123:[49->1,49->3,49->5,50->2,50->8,50->9], C00022->C00123:[1->1,1->3,1->5,3->2,3->8,3->9] | 1.00 | 409.547619048 | 22 | 42 | 0 | 1 |
| Path 615 | C00024->C00123:[49->1,49->5,50->2,50->9], C00022->C00123:[3->3] | 0.83 | 401.419354839 | 19 | 31 | 0 | 1 |
| Path 616 | C00024->C00123:[49->1,49->5,50->2,50->9], C00022->C00123:[1->1,1->5,3->2,3->3,3->9] | 0.83 | 279.224137931 | 22 | 58 | 0 | 1 |
| Path 617 | C00024->C00123:[50->8], C00022->C00123:[2->3,3->8] | 0.33 | 195.895833333 | 17 | 96 | 0 | 0 |
| Path 618 | C00024->C00123:[49->1,49->5,50->2,50->8,50->9], C00022->C00123:[1->1,1->5,3->2,3->3,3->8,3->9] | 1.00 | 292.152542373 | 24 | 59 | 0 | 1 |
| Path 619 | C00024->C00123:[49->1,49->5,50->2,50->8,50->9], C00022->C00123:[1->1,1->5,2->3,3->2,3->8,3->9] | 1.00 | 214.690909091 | 25 | 110 | 0 | 1 |
| Path 620 | C00024->C00123:[49->5,50->8,50->9], C00022->C00123:[1->5,2->3,3->8,3->9] | 0.67 | 210.689320388 | 22 | 103 | 0 | 1 |
| Path 621 | C00024->C00123:[49->1,49->3,50->2] | 0.50 | 233.315789474 | 18 | 57 | 0 | 1 |
| Path 622 | C00024->C00123:[49->1,49->3,49->5,50->2,50->8,50->9], C00022->C00123:[1->1,1->3,1->5,3->2,3->8,3->9] | 1.00 | 305.935897436 | 23 | 78 | 0 | 1 |
| Path 623 | C00024->C00123:[49->1,49->3,49->5,50->2,50->8,50->9] | 1.00 | 284.58974359 | 19 | 39 | 0 | 1 |
| Path 624 | C00024->C00123:[49->1,49->5,50->2,50->8,50->9], C00022->C00123:[3->3] | 1.00 | 396.875 | 24 | 40 | 0 | 1 |
| Path 625 | C00024->C00123:[49->1,49->3,49->5,50->2,50->9] | 0.83 | 279.279411765 | 17 | 68 | 0 | 1 |
| Path 626 | C00024->C00123:[49->1,49->3,49->5,50->2,50->9] | 0.83 | 239.428571429 | 20 | 63 | 0 | 1 |
| Path 627 | C00024->C00123:[49->1,49->3,49->5,50->2,50->9] | 0.83 | 337.178571429 | 20 | 56 | 0 | 1 |
| Path 628 | C00024->C00123:[49->1,49->3,50->2,50->8] | 0.67 | 288.888888889 | 16 | 63 | 0 | 1 |
| Path 629 | C00024->C00123:[50->8], C00022->C00123:[3->3] | 0.33 | 474.965517241 | 19 | 29 | 0 | 1 |
| Path 630 | C00024->C00123:[49->1,49->3,49->5,50->2,50->8,50->9] | 1.00 | 266.157894737 | 24 | 95 | 0 | 1 |
| Path 631 | C00024->C00123:[49->1,50->2,50->8], C00022->C00123:[3->3] | 0.67 | 307.25 | 21 | 52 | 0 | 1 |
| Path 632 | C00024->C00123:[49->1,50->2,50->8], C00022->C00123:[3->3] | 0.67 | 404.548387097 | 20 | 31 | 0 | 1 |
| Path 633 | C00024->C00123:[50->8], C00022->C00123:[3->3,3->8] | 0.33 | 405.733333333 | 18 | 30 | 0 | 1 |
| Path 634 | C00024->C00123:[49->1,49->5,50->2,50->9], C00022->C00123:[3->3] | 0.83 | 308.5 | 23 | 56 | 0 | 1 |
| Path 635 | C00024->C00123:[49->1,49->3,50->2] | 0.50 | 439.08 | 15 | 25 | 0 | 1 |
| Path 636 | C00024->C00123:[49->1,49->3,50->2], C00022->C00123:[2->3] | 0.50 | 430.032258065 | 21 | 31 | 0 | 1 |
| Path 637 | C00024->C00123:[49->1,49->5,50->2,50->8,50->9], C00022->C00123:[3->3] | 1.00 | 389.757575758 | 22 | 33 | 0 | 1 |
| Path 638 | C00022->C00123:[2->3] | 0.17 | 466.878787879 | 12 | 33 | 0 | 1 |
| Path 639 | C00024->C00123:[49->1,50->2,50->3] | 0.50 | 176.865546218 | 21 | 119 | 0 | 1 |
| Path 640 | C00024->C00123:[49->1,49->5,50->2,50->8,50->9], C00022->C00123:[1->1,1->5,3->2,3->3,3->8,3->9] | 1.00 | 384.5625 | 21 | 32 | 0 | 1 |
| Path 641 | C00024->C00123:[49->1,49->3,49->5,50->2,50->8,50->9] | 1.00 | 336.838709677 | 20 | 31 | 0 | 1 |
| Path 642 | C00024->C00123:[49->1,49->3,49->5,50->2,50->9], C00022->C00123:[2->3] | 0.83 | 408.540540541 | 23 | 37 | 0 | 1 |
| Path 643 | C00024->C00123:[49->1,50->2], C00022->C00123:[1->1,2->3,3->2] | 0.50 | 320.387096774 | 11 | 31 | 0 | 0 |
| Path 644 | C00024->C00123:[49->1,49->3,50->2], C00022->C00123:[1->1,1->3,3->2] | 0.50 | 449.514285714 | 19 | 35 | 0 | 1 |
| Path 645 | C00024->C00123:[49->1,50->2,50->8], C00022->C00123:[3->3] | 0.67 | 306.021276596 | 20 | 47 | 0 | 1 |
| Path 646 | C00024->C00123:[49->1,49->3,49->5,50->2,50->9] | 0.83 | 318.703703704 | 16 | 27 | 0 | 1 |
| Path 647 | C00024->C00123:[49->1,50->2], C00022->C00123:[2->3] | 0.50 | 188.896551724 | 20 | 116 | 0 | 1 |
| Path 648 | C00024->C00123:[49->1,50->2,50->8], C00022->C00123:[1->1,3->2,3->3,3->8] | 0.67 | 374.655172414 | 18 | 29 | 0 | 1 |
| Path 649 | C00024->C00123:[49->1,49->5,50->2,50->9] | 0.67 | 319.727272727 | 13 | 22 | 0 | 0 |
| Path 650 | C00024->C00123:[49->3] | 0.17 | 1259.33333333 | 12 | 30 | 0 | 1 |
| Path 651 | C00024->C00123:[49->1,49->5,50->2,50->8,50->9], C00022->C00123:[3->3] | 1.00 | 396.282051282 | 23 | 39 | 0 | 1 |
| Path 652 | C00024->C00123:[49->1,50->2], C00022->C00123:[3->3] | 0.50 | 293.8 | 19 | 50 | 0 | 1 |
| Path 653 | C00024->C00123:[49->3], C00022->C00123:[1->3] | 0.17 | 469.0 | 14 | 29 | 0 | 1 |
| Path 654 | C00024->C00123:[49->1,50->2,50->8], C00022->C00123:[1->1,2->3,3->2,3->8] | 0.67 | 388.98245614 | 23 | 57 | 0 | 1 |
| Path 655 | C00024->C00123:[49->1,50->2,50->8], C00022->C00123:[2->3] | 0.67 | 207.716666667 | 23 | 120 | 0 | 1 |
| Path 656 | C00024->C00123:[49->1,49->5,50->2,50->8,50->9], C00022->C00123:[3->3] | 1.00 | 316.830508475 | 26 | 59 | 0 | 1 |
| Path 657 | C00024->C00123:[49->1,50->2], C00022->C00123:[2->3] | 0.50 | 426.804878049 | 21 | 41 | 0 | 1 |
| Path 658 | C00024->C00123:[49->1,50->2,50->8], C00022->C00123:[1->1,3->2,3->3,3->8] | 0.67 | 394.055555556 | 20 | 36 | 0 | 1 |
| Path 659 | C00024->C00123:[49->1,49->3,50->2,50->8], C00022->C00123:[1->1,1->3,3->2,3->8] | 0.67 | 467.461538462 | 20 | 39 | 0 | 1 |
| Path 660 | C00024->C00123:[49->1,49->3,49->5,50->2,50->9], C00022->C00123:[2->3] | 0.83 | 214.738461538 | 27 | 130 | 0 | 1 |
| Path 661 | C00024->C00123:[49->1,49->3,49->5,50->2,50->8,50->9], C00022->C00123:[2->3] | 1.00 | 327.360655738 | 27 | 61 | 0 | 1 |
| Path 662 | C00024->C00123:[49->1,49->5,50->2,50->3,50->9] | 0.83 | 180.147058824 | 20 | 102 | 0 | 1 |
| Path 663 | C00024->C00123:[49->5,50->8,50->9], C00022->C00123:[1->5,3->3,3->8,3->9] | 0.67 | 374.444444444 | 21 | 36 | 0 | 1 |
| Path 664 | C00024->C00123:[49->1,50->2,50->8], C00022->C00123:[1->1,3->2,3->3,3->8] | 0.67 | 374.444444444 | 21 | 36 | 0 | 1 |
| Path 665 | C00024->C00123:[50->8], C00022->C00123:[2->3] | 0.33 | 347.464285714 | 11 | 28 | 0 | 1 |
| Path 666 | C00024->C00123:[49->5,50->8,50->9], C00022->C00123:[1->5,3->3,3->8,3->9] | 0.67 | 294.68627451 | 20 | 51 | 0 | 1 |
| Path 667 | C00024->C00123:[49->3,50->8], C00022->C00123:[1->3,3->8] | 0.33 | 265.512820513 | 16 | 39 | 0 | 1 |
| Path 668 | C00024->C00123:[49->1,49->3,50->2,50->8], C00022->C00123:[1->1,1->3,3->2,3->8] | 0.67 | 416.451612903 | 17 | 31 | 0 | 1 |
| Path 669 | C00024->C00123:[49->5,50->8,50->9], C00022->C00123:[1->5,3->8,3->9] | 0.50 | 358.125 | 14 | 24 | 0 | 0 |
| Path 670 | C00024->C00123:[49->1,50->2,50->8], C00022->C00123:[2->3] | 0.67 | 350.21875 | 12 | 32 | 0 | 0 |
| Path 671 | C00024->C00123:[49->1,50->2,50->8], C00022->C00123:[1->1,3->2,3->3,3->8] | 0.67 | 392.939393939 | 20 | 33 | 0 | 1 |
| Path 672 | C00024->C00123:[49->1,49->3,50->2,50->8], C00022->C00123:[1->1,1->3,3->2,3->8] | 0.67 | 441.606060606 | 17 | 33 | 0 | 1 |
| Path 673 | C00024->C00123:[49->1,49->3,50->2,50->8], C00022->C00123:[1->1,1->3,3->2,3->8] | 0.67 | 412.59375 | 18 | 32 | 0 | 1 |
| Path 674 | C00024->C00123:[49->1,49->3,49->5,50->2,50->8,50->9] | 1.00 | 312.555555556 | 21 | 72 | 0 | 1 |
| Path 675 | C00024->C00123:[49->1,49->3,49->5,50->2,50->8,50->9] | 1.00 | 440.055555556 | 20 | 36 | 0 | 1 |
| Path 676 | C00024->C00123:[49->1,49->5,50->2,50->8,50->9], C00022->C00123:[1->1,1->5,3->2,3->3,3->8,3->9] | 1.00 | 346.184210526 | 22 | 38 | 0 | 1 |
| Path 677 | C00024->C00123:[49->3] | 0.17 | 269.178571429 | 10 | 56 | 0 | 1 |
| Path 678 | C00024->C00123:[50->8], C00022->C00123:[3->3,3->8] | 0.33 | 393.314285714 | 19 | 35 | 0 | 1 |
| Path 679 | C00024->C00123:[49->1,50->2,50->8], C00022->C00123:[3->3] | 0.67 | 447.137931034 | 21 | 29 | 0 | 1 |
| Path 680 | C00024->C00123:[49->1,49->5,50->2,50->8,50->9], C00022->C00123:[1->1,1->5,3->2,3->3,3->8,3->9] | 1.00 | 290.838709677 | 26 | 62 | 0 | 1 |
| Path 681 | C00024->C00123:[49->1,49->5,50->2,50->9], C00022->C00123:[1->1,1->5,2->3,3->2,3->9] | 0.83 | 356.034482759 | 21 | 58 | 0 | 1 |
| Path 682 | C00024->C00123:[49->5,50->8,50->9], C00022->C00123:[1->5,3->3,3->8,3->9] | 0.67 | 296.839285714 | 21 | 56 | 0 | 1 |
| Path 683 | C00024->C00123:[50->8], C00022->C00123:[3->3] | 0.33 | 426.416666667 | 17 | 24 | 0 | 1 |
| Path 684 | C00024->C00123:[49->1,50->2], C00022->C00123:[2->3] | 0.50 | 336.253731343 | 23 | 67 | 0 | 1 |
| Path 685 | C00024->C00123:[49->1,49->5,50->2,50->8,50->9], C00022->C00123:[1->1,1->5,2->3,3->2,3->8,3->9] | 1.00 | 200.407692308 | 26 | 130 | 0 | 1 |
| Path 686 | C00024->C00123:[49->1,49->3,49->5,50->2,50->9], C00022->C00123:[1->1,1->3,1->5,3->2,3->9] | 0.83 | 297.648648649 | 19 | 74 | 0 | 1 |
| Path 687 | C00024->C00123:[49->3,49->5,50->8,50->9], C00022->C00123:[1->3,1->5,3->8,3->9] | 0.67 | 269.951219512 | 18 | 41 | 0 | 1 |
| Path 688 | C00024->C00123:[49->1,49->3,49->5,50->2,50->8,50->9] | 1.00 | 285.21875 | 25 | 96 | 0 | 1 |
| Path 689 | C00024->C00123:[49->1,49->3,49->5,50->2,50->8,50->9] | 1.00 | 283.272727273 | 21 | 44 | 0 | 1 |
| Path 690 | C00024->C00123:[49->1,50->2,50->8], C00022->C00123:[3->3] | 0.67 | 328.775510204 | 21 | 49 | 0 | 1 |
| Path 691 | C00024->C00123:[49->5,50->8,50->9], C00022->C00123:[1->5,2->3,3->8,3->9] | 0.67 | 429.673913043 | 20 | 46 | 0 | 1 |
| Path 692 | C00024->C00123:[49->1,49->5,50->2,50->8,50->9], C00022->C00123:[1->1,1->5,3->2,3->3,3->8,3->9] | 1.00 | 356.756756757 | 22 | 37 | 0 | 1 |
| Path 693 | C00024->C00123:[49->1,49->3,50->2,50->8], C00022->C00123:[1->1,1->3,3->2,3->8] | 0.67 | 307.753623188 | 18 | 69 | 0 | 1 |
| Path 694 | C00024->C00123:[49->1,49->3,49->5,50->2,50->8,50->9] | 1.00 | 344.050847458 | 23 | 59 | 0 | 1 |
| Path 695 | C00024->C00123:[49->5,50->3,50->8,50->9], C00022->C00123:[1->5,3->3,3->8,3->9] | 0.67 | 181.598039216 | 20 | 102 | 0 | 1 |
| Path 696 | C00024->C00123:[49->1,50->2,50->3,50->8], C00022->C00123:[1->1,3->2,3->3,3->8] | 0.67 | 339.178571429 | 21 | 56 | 0 | 1 |
| Path 697 | C00024->C00123:[50->8], C00022->C00123:[2->3,3->8] | 0.33 | 210.363636364 | 19 | 99 | 0 | 0 |
| Path 698 | C00024->C00123:[49->5,50->8,50->9], C00022->C00123:[1->5,3->8,3->9] | 0.50 | 486.142857143 | 18 | 35 | 0 | 0 |
| Path 699 | C00022->C00123:[3->3] | 0.17 | 481.217391304 | 14 | 23 | 0 | 1 |
| Path 700 | C00024->C00123:[49->5,50->8,50->9], C00022->C00123:[1->5,2->3,3->8,3->9] | 0.67 | 390.696428571 | 22 | 56 | 0 | 1 |
| Path 701 | C00024->C00123:[49->1,49->3,49->5,50->2,50->8,50->9] | 1.00 | 439.513513514 | 21 | 37 | 0 | 1 |
| Path 702 | C00024->C00123:[49->1,49->3,50->2,50->8] | 0.67 | 1084.18421053 | 19 | 38 | 0 | 1 |
| Path 703 | C00024->C00123:[49->1,49->3,49->5,50->2,50->8,50->9], C00022->C00123:[1->1,1->3,1->5,2->3,3->2,3->8,3->9] | 1.00 | 380.956521739 | 29 | 69 | 0 | 1 |
| Path 704 | C00024->C00123:[49->5,50->8,50->9], C00022->C00123:[1->5,3->3,3->8,3->9] | 0.67 | 392.939393939 | 20 | 33 | 0 | 1 |
| Path 705 | C00024->C00123:[49->1,49->5,50->2,50->8,50->9], C00022->C00123:[3->3] | 1.00 | 311.210526316 | 26 | 57 | 0 | 1 |
| Path 706 | C00024->C00123:[49->5,50->8,50->9], C00022->C00123:[1->5,3->3,3->8,3->9] | 0.67 | 428.5 | 22 | 36 | 0 | 1 |
| Path 707 | C00024->C00123:[49->3,50->8], C00022->C00123:[1->3,3->8] | 0.33 | 465.914285714 | 19 | 35 | 0 | 1 |
| Path 708 | C00024->C00123:[49->1,50->2,50->8], C00022->C00123:[2->3] | 0.67 | 410.588235294 | 21 | 51 | 0 | 1 |
| Path 709 | C00024->C00123:[49->1,49->3,50->2], C00022->C00123:[2->3] | 0.50 | 210.733870968 | 25 | 124 | 0 | 1 |
| Path 710 | C00024->C00123:[49->1,49->3,50->2,50->8] | 0.67 | 460.423076923 | 16 | 26 | 0 | 1 |
| Path 711 | C00024->C00123:[49->1,49->5,50->2,50->8,50->9], C00022->C00123:[1->1,1->5,2->3,3->2,3->8,3->9] | 1.00 | 202.083969466 | 27 | 131 | 0 | 1 |
| Path 712 | C00024->C00123:[49->5,50->8,50->9] | 0.50 | 402.333333333 | 12 | 18 | 0 | 0 |
| Path 713 | C00024->C00123:[49->1,49->5,50->2,50->9], C00022->C00123:[2->3] | 0.83 | 410.29787234 | 23 | 47 | 0 | 1 |
| Path 714 | C00024->C00123:[49->1,50->2], C00022->C00123:[1->1,2->3,3->2] | 0.50 | 384.060606061 | 17 | 66 | 0 | 0 |
| Path 715 | C00024->C00123:[49->1,49->3,49->5,50->2,50->8,50->9], C00022->C00123:[1->1,1->3,1->5,3->2,3->8,3->9] | 1.00 | 430.340909091 | 24 | 44 | 0 | 1 |
| Path 716 | C00024->C00123:[49->1,49->5,50->2,50->8,50->9], C00022->C00123:[1->1,1->5,3->2,3->3,3->8,3->9] | 1.00 | 370.12195122 | 24 | 41 | 0 | 1 |
| Path 717 | C00024->C00123:[49->1,49->5,50->2,50->8,50->9], C00022->C00123:[1->1,1->5,3->2,3->3,3->8,3->9] | 1.00 | 375.675675676 | 22 | 37 | 0 | 1 |
| Path 718 | C00024->C00123:[49->1,49->3,49->5,50->2,50->8,50->9], C00022->C00123:[1->1,1->3,1->5,2->3,3->2,3->8,3->9] | 1.00 | 377.695652174 | 27 | 46 | 0 | 1 |
| Path 719 | C00024->C00123:[50->8], C00022->C00123:[3->3,3->8] | 0.33 | 412.242424242 | 18 | 33 | 0 | 0 |
| Path 720 | C00024->C00123:[49->1,49->3,49->5,50->2,50->9], C00022->C00123:[1->1,1->3,1->5,3->2,3->9] | 0.83 | 257.022222222 | 18 | 45 | 0 | 1 |
| Path 721 | C00024->C00123:[49->1,49->5,50->2,50->9], C00022->C00123:[2->3] | 0.83 | 438.923076923 | 25 | 52 | 0 | 1 |
| Path 722 | C00024->C00123:[50->8], C00022->C00123:[3->3,3->8] | 0.33 | 498.622222222 | 23 | 45 | 0 | 0 |
| Path 723 | C00024->C00123:[49->1,50->2,50->8], C00022->C00123:[1->1,3->2,3->3,3->8] | 0.67 | 368.666666667 | 18 | 33 | 0 | 1 |
| Path 724 | C00024->C00123:[49->1,49->5,50->2,50->8,50->9], C00022->C00123:[1->1,1->5,3->2,3->3,3->8,3->9] | 1.00 | 297.018867925 | 22 | 53 | 0 | 1 |
| Path 725 | C00024->C00123:[49->5,50->8,50->9], C00022->C00123:[1->5,3->3,3->8,3->9] | 0.67 | 383.419354839 | 20 | 31 | 0 | 1 |
| Path 726 | C00024->C00123:[49->1,49->3,49->5,50->2,50->9], C00022->C00123:[1->1,1->3,1->5,2->3,3->2,3->9] | 0.83 | 376.803030303 | 26 | 66 | 0 | 1 |
| Path 727 | C00024->C00123:[49->1,50->2], C00022->C00123:[1->1,3->2,3->3] | 0.50 | 280.7 | 19 | 50 | 0 | 1 |
| Path 728 | C00024->C00123:[49->1,50->2,50->8], C00022->C00123:[1->1,3->2,3->8] | 0.50 | 237.6875 | 15 | 48 | 0 | 0 |
| Path 729 | C00024->C00123:[49->1,49->3,49->5,50->2,50->9], C00022->C00123:[1->1,1->3,1->5,3->2,3->9] | 0.83 | 273.01369863 | 18 | 73 | 0 | 1 |
| Path 730 | C00024->C00123:[49->1,49->3,49->5,50->2,50->9], C00022->C00123:[1->1,1->3,1->5,3->2,3->9] | 0.83 | 907.14893617 | 20 | 47 | 0 | 1 |
| Path 731 | C00024->C00123:[49->1,49->3,50->2] | 0.50 | 276.26744186 | 19 | 86 | 0 | 1 |
| Path 732 | C00024->C00123:[49->1,49->3,50->2], C00022->C00123:[2->3] | 0.50 | 322.442307692 | 22 | 52 | 0 | 1 |
| Path 733 | C00024->C00123:[49->1,49->5,50->2,50->3,50->8,50->9] | 1.00 | 301.463414634 | 27 | 82 | 0 | 1 |
| Path 734 | C00024->C00123:[49->1,49->5,50->2,50->9], C00022->C00123:[2->3] | 0.83 | 334.117647059 | 24 | 68 | 0 | 1 |
| Path 735 | C00024->C00123:[49->5,50->8,50->9] | 0.50 | 254.710526316 | 13 | 38 | 0 | 0 |
| Path 736 | C00024->C00123:[49->1,49->5,50->2,50->3,50->9] | 0.83 | 294.974683544 | 24 | 79 | 0 | 1 |
| Path 737 | C00024->C00123:[49->1,50->2,50->8], C00022->C00123:[1->1,3->2,3->8] | 0.50 | 278.08 | 12 | 25 | 0 | 0 |
| Path 738 | C00022->C00123:[2->3] | 0.17 | 404.863636364 | 15 | 44 | 0 | 1 |
| Path 739 | C00024->C00123:[49->1,49->3,50->2], C00022->C00123:[1->1,1->3,3->2] | 0.50 | 424.34375 | 16 | 32 | 0 | 1 |
| Path 740 | C00024->C00123:[49->1,49->5,50->2,50->8,50->9], C00022->C00123:[1->1,1->5,3->2,3->3,3->8,3->9] | 1.00 | 403.659090909 | 26 | 44 | 0 | 1 |
| Path 741 | C00024->C00123:[49->1,50->2,50->8], C00022->C00123:[2->3] | 0.67 | 348.066666667 | 13 | 30 | 0 | 1 |
| Path 742 | C00024->C00123:[49->1,50->2,50->8], C00022->C00123:[3->3] | 0.67 | 426.16 | 18 | 25 | 0 | 1 |
| Path 743 | C00024->C00123:[49->1,49->5,50->2,50->8,50->9], C00022->C00123:[3->3] | 1.00 | 431.333333333 | 25 | 39 | 0 | 1 |
| Path 744 | C00024->C00123:[49->1,50->2,50->3,50->8], C00022->C00123:[1->1,3->2,3->3,3->8] | 0.67 | 182.67961165 | 21 | 103 | 0 | 1 |
| Path 745 | C00024->C00123:[49->1,49->3,50->2], C00022->C00123:[1->1,1->3,2->3,3->2] | 0.50 | 383.027027027 | 22 | 37 | 0 | 1 |
| Path 746 | C00024->C00123:[49->1,49->5,50->2,50->8,50->9], C00022->C00123:[1->1,1->5,3->2,3->3,3->8,3->9] | 1.00 | 355.0 | 21 | 36 | 0 | 1 |
| Path 747 | C00024->C00123:[49->1,49->5,50->2,50->9], C00022->C00123:[3->3] | 0.83 | 372.542857143 | 20 | 35 | 0 | 1 |
| Path 748 | C00024->C00123:[49->1,50->2], C00022->C00123:[2->3] | 0.50 | 374.328125 | 21 | 64 | 0 | 0 |
| Path 749 | C00024->C00123:[49->1,49->3,49->5,50->2,50->8,50->9], C00022->C00123:[1->1,1->3,1->5,3->2,3->8,3->9] | 1.00 | 319.727272727 | 18 | 33 | 0 | 1 |
| Path 750 | C00024->C00123:[49->1,50->2,50->8], C00022->C00123:[3->3] | 0.67 | 327.218181818 | 23 | 55 | 0 | 1 |
| Path 751 | C00024->C00123:[49->1,50->2], C00022->C00123:[1->1,2->3,3->2] | 0.50 | 310.96 | 22 | 75 | 0 | 1 |
| Path 752 | C00024->C00123:[50->8], C00022->C00123:[3->3] | 0.33 | 449.32 | 17 | 25 | 0 | 1 |
| Path 753 | C00024->C00123:[49->1,50->2], C00022->C00123:[3->3] | 0.50 | 300.361702128 | 20 | 47 | 0 | 1 |
| Path 754 | C00024->C00123:[49->1,49->5,50->2,50->8,50->9], C00022->C00123:[2->3] | 1.00 | 333.972972973 | 16 | 37 | 0 | 1 |
| Path 755 | C00024->C00123:[49->1,50->2,50->8], C00022->C00123:[1->1,2->3,3->2,3->8] | 0.67 | 390.696428571 | 22 | 56 | 0 | 1 |
| Path 756 | C00024->C00123:[49->3,50->8], C00022->C00123:[1->3,3->8] | 0.33 | 320.5 | 15 | 26 | 0 | 1 |
| Path 757 | C00024->C00123:[49->1,49->3,50->2] | 0.50 | 522.571428571 | 17 | 28 | 0 | 0 |
| Path 758 | C00024->C00123:[49->1,49->5,50->2,50->9], C00022->C00123:[2->3] | 0.83 | 331.493670886 | 25 | 79 | 0 | 1 |
| Path 759 | C00024->C00123:[49->1,49->5,50->2,50->8,50->9], C00022->C00123:[3->3] | 1.00 | 310.049180328 | 26 | 61 | 0 | 1 |
| Path 760 | C00024->C00123:[49->1,49->3,50->2] | 0.50 | 337.368421053 | 12 | 19 | 0 | 1 |
| Path 761 | C00024->C00123:[49->1,49->3,49->5,50->2,50->8,50->9], C00022->C00123:[1->1,1->3,1->5,3->2,3->8,3->9] | 1.00 | 409.292682927 | 21 | 41 | 0 | 1 |
| Path 762 | C00024->C00123:[49->1,50->2], C00022->C00123:[1->1,2->3,3->2] | 0.50 | 305.878787879 | 12 | 33 | 0 | 1 |
| Path 763 | C00022->C00123:[3->3] | 0.17 | 448.789473684 | 12 | 19 | 0 | 1 |
| Path 764 | C00024->C00123:[49->1,50->2,50->8], C00022->C00123:[1->1,3->2,3->3,3->8] | 0.67 | 294.653846154 | 21 | 52 | 0 | 1 |
| Path 765 | C00022->C00123:[3->3] | 0.17 | 354.571428571 | 11 | 21 | 0 | 1 |
| Path 766 | C00024->C00123:[49->1,50->2,50->8], C00022->C00123:[1->1,3->2,3->3,3->8] | 0.67 | 428.5 | 22 | 36 | 0 | 1 |
| Path 767 | C00024->C00123:[49->1,49->3,50->2,50->8], C00022->C00123:[1->1,1->3,3->2,3->8] | 0.67 | 463.1 | 21 | 40 | 0 | 1 |
| Path 768 | C00024->C00123:[49->1,49->3,49->5,50->2,50->8,50->9] | 1.00 | 280.093023256 | 20 | 43 | 0 | 1 |
| Path 769 | C00024->C00123:[50->8], C00022->C00123:[3->3] | 0.33 | 427.566666667 | 18 | 30 | 0 | 1 |
| Path 770 | C00024->C00123:[49->1,50->2,50->8], C00022->C00123:[1->1,3->2,3->8] | 0.50 | 308.05 | 11 | 20 | 0 | 0 |
| Path 771 | C00024->C00123:[50->8], C00022->C00123:[3->3,3->8] | 0.33 | 399.6 | 15 | 25 | 0 | 0 |
| Path 772 | C00024->C00123:[49->1,50->2], C00022->C00123:[2->3] | 0.50 | 388.588235294 | 15 | 34 | 0 | 0 |
| Path 773 | C00024->C00123:[49->1,49->5,50->2,50->8,50->9], C00022->C00123:[2->3] | 1.00 | 367.404761905 | 19 | 42 | 0 | 1 |
| Path 774 | C00024->C00123:[49->1,49->5,50->2,50->9], C00022->C00123:[3->3] | 0.83 | 312.981481481 | 22 | 54 | 0 | 1 |
| Path 775 | C00024->C00123:[50->8], C00022->C00123:[2->3] | 0.33 | 193.844827586 | 20 | 116 | 0 | 1 |
| Path 776 | C00024->C00123:[49->1,50->2], C00022->C00123:[2->3] | 0.50 | 400.147540984 | 16 | 61 | 0 | 0 |
| Path 777 | C00024->C00123:[49->1,50->2], C00022->C00123:[1->1,2->3,3->2] | 0.50 | 387.014492754 | 23 | 69 | 0 | 1 |
| Path 778 | C00022->C00123:[2->3] | 0.17 | 176.760869565 | 13 | 92 | 0 | 1 |
| Path 779 | C00024->C00123:[49->1,50->2,50->8], C00022->C00123:[3->3] | 0.67 | 448.192307692 | 18 | 26 | 0 | 1 |
| Path 780 | C00024->C00123:[49->1,49->3,50->2,50->8], C00022->C00123:[1->1,1->3,3->2,3->8] | 0.67 | 333.72 | 14 | 25 | 0 | 1 |
| Path 781 | C00024->C00123:[49->3,49->5,50->8,50->9], C00022->C00123:[1->3,1->5,3->8,3->9] | 0.67 | 323.071428571 | 17 | 28 | 0 | 1 |
| Path 782 | C00024->C00123:[49->3,49->5,50->8,50->9], C00022->C00123:[1->3,1->5,3->8,3->9] | 0.67 | 467.461538462 | 20 | 39 | 0 | 1 |
| Path 783 | C00024->C00123:[49->1,50->2,50->8], C00022->C00123:[1->1,3->2,3->3,3->8] | 0.67 | 364.9 | 18 | 30 | 0 | 1 |
| Path 784 | C00024->C00123:[49->1,50->2], C00022->C00123:[3->3] | 0.50 | 426.36 | 17 | 25 | 0 | 1 |
| Path 785 | C00024->C00123:[49->1,50->2], C00022->C00123:[1->1,3->2,3->3] | 0.50 | 343.206896552 | 17 | 29 | 0 | 1 |
| Path 786 | C00024->C00123:[49->1,50->2,50->8], C00022->C00123:[1->1,2->3,3->2,3->8] | 0.67 | 328.27027027 | 13 | 37 | 0 | 0 |
| Path 787 | C00024->C00123:[49->1,49->3,50->2,50->8] | 0.67 | 366.523809524 | 14 | 21 | 0 | 1 |
| Path 788 | C00024->C00123:[49->1,49->3,50->2,50->8] | 0.67 | 454.222222222 | 17 | 27 | 0 | 1 |
| Path 789 | C00024->C00123:[49->1,49->5,50->2,50->9], C00022->C00123:[2->3] | 0.83 | 324.740740741 | 17 | 54 | 0 | 0 |
| Path 790 | C00022->C00123:[3->3] | 0.17 | 418.222222222 | 12 | 18 | 0 | 1 |
| Path 791 | C00024->C00123:[49->3,50->8] | 0.33 | 1124.61111111 | 17 | 36 | 0 | 1 |
| Path 792 | C00024->C00123:[49->1,49->5,50->2,50->8,50->9], C00022->C00123:[2->3] | 1.00 | 431.857142857 | 23 | 49 | 0 | 1 |
| Path 793 | C00024->C00123:[49->1,49->5,50->2,50->8,50->9], C00022->C00123:[1->1,1->5,3->2,3->3,3->8,3->9] | 1.00 | 379.487179487 | 23 | 39 | 0 | 1 |
| Path 794 | C00024->C00123:[49->1,49->5,50->2,50->9], C00022->C00123:[3->3] | 0.83 | 291.784313725 | 20 | 51 | 0 | 1 |
| Path 795 | C00024->C00123:[49->1,50->2,50->8], C00022->C00123:[1->1,3->2,3->8] | 0.50 | 246.930232558 | 14 | 43 | 0 | 0 |
| Path 796 | C00024->C00123:[49->1,49->5,50->2,50->8,50->9], C00022->C00123:[2->3] | 1.00 | 336.236842105 | 17 | 38 | 0 | 1 |
| Path 797 | C00024->C00123:[49->1,50->2,50->8], C00022->C00123:[2->3] | 0.67 | 349.965517241 | 12 | 29 | 0 | 1 |
| Path 798 | C00024->C00123:[49->1,49->3,50->2,50->8], C00022->C00123:[1->1,1->3,3->2,3->8] | 0.67 | 979.953488372 | 20 | 43 | 0 | 1 |
| Path 799 | C00024->C00123:[49->3] | 0.17 | 232.391304348 | 8 | 23 | 0 | 1 |
| Path 800 | C00024->C00123:[49->1,49->5,50->2,50->9], C00022->C00123:[3->3] | 0.83 | 428.142857143 | 21 | 35 | 0 | 1 |
| Path 801 | C00024->C00123:[49->1,49->3,49->5,50->2,50->9] | 0.83 | 277.652173913 | 21 | 92 | 0 | 1 |
| Path 802 | C00024->C00123:[49->3,49->5,50->8,50->9], C00022->C00123:[1->3,1->5,3->8,3->9] | 0.67 | 324.185185185 | 16 | 27 | 0 | 1 |
| Path 803 | C00024->C00123:[49->1,49->5,50->2,50->8,50->9], C00022->C00123:[1->1,1->5,2->3,3->2,3->8,3->9] | 1.00 | 315.761904762 | 27 | 84 | 0 | 1 |
| Path 804 | C00024->C00123:[49->1,49->5,50->2,50->8,50->9], C00022->C00123:[2->3] | 1.00 | 202.650793651 | 26 | 126 | 0 | 1 |
| Path 805 | C00024->C00123:[49->1,49->3,50->2] | 0.50 | 258.738095238 | 15 | 42 | 0 | 1 |
| Path 806 | C00024->C00123:[49->1,49->3,49->5,50->2,50->8,50->9], C00022->C00123:[1->1,1->3,1->5,3->2,3->8,3->9] | 1.00 | 967.227272727 | 21 | 44 | 0 | 1 |
| Path 807 | C00024->C00123:[50->8], C00022->C00123:[2->3] | 0.33 | 464.435897436 | 17 | 39 | 0 | 1 |
| Path 808 | C00024->C00123:[49->1,49->5,50->2,50->9], C00022->C00123:[1->1,1->5,2->3,3->2,3->9] | 0.83 | 309.962962963 | 24 | 81 | 0 | 1 |
| Path 809 | C00024->C00123:[49->1,49->5,50->2,50->8,50->9], C00022->C00123:[3->3] | 1.00 | 321.368421053 | 25 | 57 | 0 | 1 |
| Path 810 | C00024->C00123:[50->8], C00022->C00123:[2->3] | 0.33 | 410.4 | 20 | 50 | 0 | 1 |
| Path 811 | C00024->C00123:[49->1,50->2,50->8], C00022->C00123:[1->1,3->2,3->3,3->8] | 0.67 | 390.0 | 21 | 34 | 0 | 1 |
| Path 812 | C00024->C00123:[49->1,50->2,50->8], C00022->C00123:[2->3] | 0.67 | 377.636363636 | 15 | 33 | 0 | 1 |
| Path 813 | C00024->C00123:[49->1,50->2,50->8] | 0.50 | 480.09375 | 18 | 32 | 0 | 0 |
| Path 814 | C00024->C00123:[49->1,50->2,50->8] | 0.50 | 535.866666667 | 17 | 30 | 0 | 0 |
| Path 815 | C00024->C00123:[49->1,49->5,50->2,50->9], C00022->C00123:[1->1,1->5,2->3,3->2,3->9] | 0.83 | 405.26 | 20 | 50 | 0 | 1 |
| Path 816 | C00024->C00123:[49->1,49->3,49->5,50->2,50->8,50->9] | 1.00 | 343.285714286 | 17 | 28 | 0 | 1 |
| Path 817 | C00024->C00123:[49->1,49->3,49->5,50->2,50->8,50->9] | 1.00 | 699.573529412 | 25 | 68 | 0 | 1 |
| Path 818 | C00024->C00123:[49->1,49->3,50->2] | 0.50 | 341.94 | 18 | 50 | 0 | 1 |
| Path 819 | C00024->C00123:[49->1,50->2,50->3,50->8] | 0.67 | 181.278350515 | 19 | 97 | 0 | 1 |
| Path 820 | C00024->C00123:[49->1,49->3,50->2,50->8] | 0.67 | 282.709677419 | 15 | 31 | 0 | 1 |
| Path 821 | C00024->C00123:[49->1,50->2], C00022->C00123:[2->3] | 0.50 | 354.774193548 | 20 | 62 | 0 | 1 |
| Path 822 | C00024->C00123:[49->1,50->2,50->8] | 0.50 | 349.260869565 | 13 | 23 | 0 | 0 |
| Path 823 | C00024->C00123:[49->1,49->5,50->2,50->8,50->9], C00022->C00123:[2->3] | 1.00 | 354.901408451 | 25 | 71 | 0 | 1 |
| Path 824 | C00024->C00123:[49->1,49->3,49->5,50->2,50->9] | 0.83 | 992.785714286 | 19 | 42 | 0 | 1 |
| Path 825 | C00024->C00123:[50->8], C00022->C00123:[2->3,3->8] | 0.33 | 338.903225806 | 11 | 31 | 0 | 0 |
| Path 826 | C00024->C00123:[49->3,50->8] | 0.33 | 367.578947368 | 12 | 19 | 0 | 1 |
| Path 827 | C00024->C00123:[49->1,50->2], C00022->C00123:[2->3] | 0.50 | 457.369565217 | 23 | 46 | 0 | 1 |
| Path 828 | C00024->C00123:[49->1,49->5,50->2,50->8,50->9], C00022->C00123:[1->1,1->5,2->3,3->2,3->8,3->9] | 1.00 | 198.048387097 | 24 | 124 | 0 | 1 |
| Path 829 | C00024->C00123:[49->1,49->5,50->2,50->8,50->9], C00022->C00123:[3->3] | 1.00 | 321.161290323 | 26 | 62 | 0 | 1 |
| Path 830 | C00024->C00123:[49->1,50->2], C00022->C00123:[3->3] | 0.50 | 408.433333333 | 18 | 30 | 0 | 1 |
| Path 831 | C00024->C00123:[49->3], C00022->C00123:[1->3] | 0.17 | 472.03125 | 14 | 32 | 0 | 1 |
| Path 832 | C00024->C00123:[49->1,49->3,50->2,50->8] | 0.67 | 282.366666667 | 14 | 30 | 0 | 1 |
| Path 833 | C00024->C00123:[49->1,49->5,50->2,50->8,50->9], C00022->C00123:[1->1,1->5,3->2,3->3,3->8,3->9] | 1.00 | 373.644444444 | 25 | 45 | 0 | 1 |
| Path 834 | C00024->C00123:[50->8], C00022->C00123:[2->3] | 0.33 | 399.551724138 | 12 | 29 | 0 | 0 |
| Path 835 | C00024->C00123:[49->1,49->5,50->2,50->9], C00022->C00123:[1->1,1->5,2->3,3->2,3->9] | 0.83 | 231.553719008 | 24 | 121 | 0 | 1 |
| Path 836 | C00024->C00123:[50->8] | 0.17 | 539.862068966 | 16 | 29 | 0 | 0 |
| Path 837 | C00024->C00123:[49->1,50->2], C00022->C00123:[1->1,3->2,3->3] | 0.50 | 365.482758621 | 18 | 29 | 0 | 1 |
| Path 838 | C00024->C00123:[49->1,50->2], C00022->C00123:[1->1,2->3,3->2] | 0.50 | 228.113043478 | 22 | 115 | 0 | 1 |
| Path 839 | C00024->C00123:[49->1,50->2,50->8], C00022->C00123:[1->1,3->2,3->8] | 0.50 | 320.428571429 | 14 | 28 | 0 | 0 |
| Path 840 | C00024->C00123:[49->1,50->2,50->8], C00022->C00123:[1->1,3->2,3->3,3->8] | 0.67 | 294.68627451 | 20 | 51 | 0 | 1 |
| Path 841 | C00024->C00123:[49->1,49->5,50->2,50->3,50->8,50->9] | 1.00 | 188.495238095 | 23 | 105 | 0 | 1 |
| Path 842 | C00024->C00123:[49->1,49->3,50->2,50->8], C00022->C00123:[1->1,1->3,3->2,3->8] | 0.67 | 268.857142857 | 15 | 35 | 0 | 1 |
| Path 843 | C00024->C00123:[49->1,49->5,50->2,50->8,50->9], C00022->C00123:[2->3] | 1.00 | 213.596899225 | 28 | 129 | 0 | 1 |
| Path 844 | C00024->C00123:[49->1,49->5,50->2,50->8,50->9], C00022->C00123:[3->3] | 1.00 | 311.822580645 | 27 | 62 | 0 | 1 |
| Path 845 | C00024->C00123:[49->1,50->2], C00022->C00123:[2->3] | 0.50 | 337.661290323 | 22 | 62 | 0 | 1 |
| Path 846 | C00024->C00123:[49->1,49->3,49->5,50->2,50->8,50->9], C00022->C00123:[1->1,1->3,1->5,3->2,3->8,3->9] | 1.00 | 872.019607843 | 24 | 51 | 0 | 1 |
| Path 847 | C00024->C00123:[49->1,49->3,50->2] | 0.50 | 752.016949153 | 20 | 59 | 0 | 1 |
| Path 848 | C00024->C00123:[49->1,49->3,50->2] | 0.50 | 257.827586207 | 13 | 29 | 0 | 1 |
| Path 849 | C00024->C00123:[49->1,49->5,50->2,50->8,50->9], C00022->C00123:[2->3] | 1.00 | 432.104166667 | 22 | 48 | 0 | 1 |
| Path 850 | C00024->C00123:[49->1,49->5,50->2,50->8,50->9], C00022->C00123:[1->1,1->5,3->2,3->3,3->8,3->9] | 1.00 | 350.585365854 | 22 | 41 | 0 | 1 |
| Path 851 | C00024->C00123:[49->1,49->3,50->2], C00022->C00123:[1->1,1->3,3->2] | 0.50 | 250.794871795 | 16 | 39 | 0 | 1 |
| Path 852 | C00024->C00123:[49->1,50->2,50->8] | 0.50 | 322.1 | 11 | 20 | 0 | 0 |
| Path 853 | C00022->C00123:[2->3] | 0.17 | 192.7 | 14 | 90 | 0 | 1 |
| Path 854 | C00024->C00123:[49->1,50->2,50->8], C00022->C00123:[1->1,3->2,3->3,3->8] | 0.67 | 394.055555556 | 20 | 36 | 0 | 1 |
| Path 855 | C00024->C00123:[50->8], C00022->C00123:[3->3] | 0.33 | 326.113207547 | 21 | 53 | 0 | 1 |
| Path 856 | C00024->C00123:[49->1,50->2], C00022->C00123:[1->1,2->3,3->2] | 0.50 | 379.727272727 | 21 | 55 | 0 | 1 |
| Path 857 | C00024->C00123:[49->1,50->2], C00022->C00123:[3->3] | 0.50 | 388.068965517 | 18 | 29 | 0 | 1 |
| Path 858 | C00024->C00123:[49->1,50->2,50->8], C00022->C00123:[1->1,3->2,3->8] | 0.50 | 295.24 | 12 | 25 | 0 | 0 |
| Path 859 | C00024->C00123:[49->1,50->2,50->8], C00022->C00123:[1->1,3->2,3->3,3->8] | 0.67 | 406.193548387 | 19 | 31 | 0 | 1 |
| Path 860 | C00024->C00123:[49->3,49->5,50->8,50->9], C00022->C00123:[1->3,1->5,3->8,3->9] | 0.67 | 268.857142857 | 15 | 35 | 0 | 1 |
| Path 861 | C00024->C00123:[49->1,49->3,49->5,50->2,50->8,50->9] | 1.00 | 248.131147541 | 21 | 61 | 0 | 1 |
| Path 862 | C00024->C00123:[49->1,50->2,50->3], C00022->C00123:[1->1,3->2,3->3] | 0.50 | 173.554455446 | 19 | 101 | 0 | 1 |
| Path 863 | C00024->C00123:[49->1,50->2], C00022->C00123:[1->1,2->3,3->2] | 0.50 | 188.851239669 | 21 | 121 | 0 | 1 |
| Path 864 | C00024->C00123:[49->1,49->3,49->5,50->2,50->8,50->9], C00022->C00123:[1->1,1->3,1->5,3->2,3->8,3->9] | 1.00 | 273.594594595 | 17 | 37 | 0 | 1 |
| Path 865 | C00024->C00123:[49->1,50->2,50->8], C00022->C00123:[1->1,3->2,3->3,3->8] | 0.67 | 296.771929825 | 22 | 57 | 0 | 1 |
| Path 866 | C00022->C00123:[2->3] | 0.17 | 179.818181818 | 15 | 110 | 0 | 1 |
| Path 867 | C00024->C00123:[50->8], C00022->C00123:[2->3] | 0.33 | 379.0 | 13 | 31 | 0 | 1 |
| Path 868 | C00024->C00123:[49->1,49->3,50->2,50->8] | 0.67 | 355.181818182 | 15 | 22 | 0 | 1 |
| Path 869 | C00024->C00123:[49->1,49->3,50->2], C00022->C00123:[1->1,1->3,2->3,3->2] | 0.50 | 389.676470588 | 20 | 34 | 0 | 1 |
| Path 870 | C00024->C00123:[49->1,50->2,50->3] | 0.50 | 172.8125 | 18 | 96 | 0 | 1 |
| Path 871 | C00024->C00123:[49->1,49->5,50->2,50->3,50->8,50->9] | 1.00 | 190.679245283 | 24 | 106 | 0 | 1 |
| Path 872 | C00024->C00123:[49->1,49->5,50->2,50->8,50->9], C00022->C00123:[1->1,1->5,3->2,3->3,3->8,3->9] | 1.00 | 294.234375 | 25 | 64 | 0 | 1 |
| Path 873 | C00024->C00123:[50->8], C00022->C00123:[3->3] | 0.33 | 429.925925926 | 18 | 27 | 0 | 1 |
| Path 874 | C00024->C00123:[49->1,49->5,50->2,50->9], C00022->C00123:[1->1,1->5,2->3,3->2,3->9] | 0.83 | 193.984251969 | 23 | 127 | 0 | 1 |
| Path 875 | C00024->C00123:[49->1,50->2] | 0.33 | 234.72972973 | 12 | 37 | 0 | 0 |
| Path 876 | C00024->C00123:[49->1,49->3,49->5,50->2,50->8,50->9] | 1.00 | 275.907407407 | 23 | 54 | 0 | 1 |
| Path 877 | C00024->C00123:[49->1,49->5,50->2,50->9], C00022->C00123:[1->1,1->5,2->3,3->2,3->9] | 0.83 | 384.04 | 22 | 50 | 0 | 1 |
| Path 878 | C00024->C00123:[49->1,49->5,50->2,50->9], C00022->C00123:[1->1,1->5,2->3,3->2,3->9] | 0.83 | 304.58974359 | 14 | 39 | 0 | 1 |
| Path 879 | C00024->C00123:[49->1,49->3,49->5,50->2,50->8,50->9] | 1.00 | 275.862745098 | 20 | 51 | 0 | 1 |
| Path 880 | C00024->C00123:[49->5,50->8,50->9], C00022->C00123:[1->5,3->3,3->8,3->9] | 0.67 | 432.371428571 | 21 | 35 | 0 | 1 |
| Path 881 | C00024->C00123:[50->8], C00022->C00123:[3->3] | 0.33 | 326.875 | 20 | 48 | 0 | 1 |
| Path 882 | C00024->C00123:[49->5,50->8,50->9], C00022->C00123:[1->5,3->8,3->9] | 0.50 | 246.930232558 | 14 | 43 | 0 | 0 |
| Path 883 | C00024->C00123:[49->1,49->5,50->2,50->3,50->8,50->9] | 1.00 | 189.4453125 | 26 | 128 | 0 | 1 |
| Path 884 | C00024->C00123:[49->1,50->2,50->8], C00022->C00123:[1->1,3->2,3->3,3->8] | 0.67 | 377.571428571 | 17 | 28 | 0 | 1 |
| Path 885 | C00024->C00123:[49->1,50->2], C00022->C00123:[3->3] | 0.50 | 408.666666667 | 18 | 27 | 0 | 1 |
| Path 886 | C00024->C00123:[49->1,49->3,50->2], C00022->C00123:[2->3] | 0.50 | 468.638888889 | 23 | 36 | 0 | 1 |
| Path 887 | C00024->C00123:[50->8], C00022->C00123:[3->3,3->8] | 0.33 | 294.6 | 20 | 55 | 0 | 1 |
| Path 888 | C00024->C00123:[49->1,49->5,50->2,50->9], C00022->C00123:[1->1,1->5,3->2,3->3,3->9] | 0.83 | 282.5 | 21 | 56 | 0 | 1 |
| Path 889 | C00024->C00123:[49->1,50->2,50->8], C00022->C00123:[2->3] | 0.67 | 459.170731707 | 19 | 41 | 0 | 1 |
| Path 890 | C00024->C00123:[49->1,49->3,50->2,50->8], C00022->C00123:[1->1,1->3,3->2,3->8] | 0.67 | 307.542857143 | 19 | 70 | 0 | 1 |
| Path 891 | C00024->C00123:[49->1,49->3,49->5,50->2,50->8,50->9], C00022->C00123:[1->1,1->3,1->5,3->2,3->8,3->9] | 1.00 | 881.06 | 23 | 50 | 0 | 1 |
| Path 892 | C00024->C00123:[50->8], C00022->C00123:[3->3] | 0.33 | 305.28 | 19 | 50 | 0 | 1 |
| Path 893 | C00024->C00123:[49->5,50->8,50->9], C00022->C00123:[1->5,3->8,3->9] | 0.50 | 308.05 | 11 | 20 | 0 | 0 |
| Path 894 | C00024->C00123:[49->1,49->3,49->5,50->2,50->8,50->9], C00022->C00123:[1->1,1->3,1->5,3->2,3->8,3->9] | 1.00 | 412.818181818 | 19 | 33 | 0 | 1 |
| Path 895 | C00024->C00123:[50->8], C00022->C00123:[3->3] | 0.33 | 407.862068966 | 18 | 29 | 0 | 1 |
| Path 896 | C00024->C00123:[49->1,49->5,50->2,50->9], C00022->C00123:[1->1,1->5,3->2,3->3,3->9] | 0.83 | 353.828571429 | 20 | 35 | 0 | 1 |
| Path 897 | C00024->C00123:[49->3,49->5,50->8,50->9], C00022->C00123:[1->3,1->5,3->8,3->9] | 0.67 | 412.59375 | 18 | 32 | 0 | 1 |
| Path 898 | C00024->C00123:[49->3,49->5,50->8,50->9], C00022->C00123:[1->3,1->5,3->8,3->9] | 0.67 | 281.623188406 | 18 | 69 | 0 | 1 |
| Path 899 | C00024->C00123:[49->1,50->2,50->8], C00022->C00123:[3->3] | 0.67 | 423.125 | 20 | 32 | 0 | 1 |
| Path 900 | C00024->C00123:[49->1,49->3,49->5,50->2,50->8,50->9], C00022->C00123:[1->1,1->3,1->5,3->2,3->8,3->9] | 1.00 | 273.530612245 | 22 | 49 | 0 | 1 |
| Path 901 | C00024->C00123:[49->1,49->3,49->5,50->2,50->8,50->9], C00022->C00123:[1->1,1->3,1->5,2->3,3->2,3->8,3->9] | 1.00 | 375.9375 | 26 | 48 | 0 | 1 |
| Path 902 | C00024->C00123:[49->1,49->5,50->2,50->8,50->9], C00022->C00123:[3->3] | 1.00 | 381.435897436 | 24 | 39 | 0 | 1 |
| Path 903 | C00024->C00123:[49->1,50->2,50->8], C00022->C00123:[1->1,2->3,3->2,3->8] | 0.67 | 195.838383838 | 19 | 99 | 0 | 1 |
| Path 904 | C00024->C00123:[49->1,50->2], C00022->C00123:[1->1,3->2,3->3] | 0.50 | 280.7 | 19 | 50 | 0 | 1 |
| Path 905 | C00024->C00123:[49->1,49->5,50->2,50->8,50->9], C00022->C00123:[1->1,1->5,2->3,3->2,3->8,3->9] | 1.00 | 327.805555556 | 15 | 36 | 0 | 1 |
| Path 906 | C00024->C00123:[49->1,49->3,50->2], C00022->C00123:[1->1,1->3,3->2] | 0.50 | 397.2 | 16 | 30 | 0 | 1 |
| Path 907 | C00024->C00123:[49->1,49->3,50->2,50->8] | 0.67 | 316.753846154 | 18 | 65 | 0 | 1 |
| Path 908 | C00024->C00123:[50->8], C00022->C00123:[2->3,3->8] | 0.33 | 373.826923077 | 19 | 52 | 0 | 1 |
| Path 909 | C00024->C00123:[49->1,50->2], C00022->C00123:[1->1,2->3,3->2] | 0.50 | 187.693877551 | 18 | 98 | 0 | 1 |
| Path 910 | C00024->C00123:[49->3,50->8] | 0.33 | 276.941176471 | 15 | 34 | 0 | 1 |
| Path 911 | C00024->C00123:[49->1,49->5,50->2,50->9], C00022->C00123:[2->3] | 0.83 | 359.333333333 | 16 | 39 | 0 | 1 |
| Path 912 | C00024->C00123:[49->1,49->3,50->2] | 0.50 | 229.5 | 16 | 52 | 0 | 1 |
| Path 913 | C00024->C00123:[50->3] | 0.17 | 160.644444444 | 13 | 90 | 0 | 1 |
| Path 914 | C00024->C00123:[49->1,50->2], C00022->C00123:[3->3] | 0.50 | 302.134615385 | 21 | 52 | 0 | 1 |
| Path 915 | C00024->C00123:[49->1,49->5,50->2,50->8,50->9], C00022->C00123:[2->3] | 1.00 | 215.971428571 | 24 | 105 | 0 | 1 |
| Path 916 | C00024->C00123:[49->1,49->3,49->5,50->2,50->8,50->9] | 1.00 | 281.026315789 | 18 | 38 | 0 | 1 |
