# Supplementary material for: 13C-metabolic flux ratio and novel carbon path analyses confirmed that Trichoderma reesei uses primarily the respirative pathway also on the preferred carbon source glucose
Source: BMC Syst Biol. 2009 Oct 29;3:104. doi: 10.1186/1752-0509-3-104 (PMC2776023; doi:10.1186/1752-0509-3-104)
Supplement: Additional file 1 — Pathways discovered in ReTrace carbon path analysis. Graphical and tabular representations of amino acid synthesis pathways discovered in ReTrace carbon path analysis [21]. Self-contained web site: unpack zip archive and open index.html with a web browser. [file 1752-0509-3-104-S1.zip › AF1-treesei/pathways-C00031-to-C00062.html]

Pathways from C00031 to C00062


**Pathways from C00031 to C00062**

**Sources:** D-Glucose; (C00031)

**Target:**L-Arginine; (C00062)

|  | Composite mapping | Z | Average score | Rpairs | Reactions | Zero scores | Scores under threshold |
| --- | --- | --- | --- | --- | --- | --- | --- |
| Path 1 | C00031->C00062:[4->10,4->5,7->2,7->3,9->1,9->4] | 1.00 | 356.916230366 | 28 | 191 | 0 | 0 |
| Path 2 | C00031->C00062:[4->10,4->5,7->2,7->3,9->1,9->4] | 1.00 | 366.993506494 | 26 | 154 | 0 | 0 |
| Path 3 | C00031->C00062:[4->10,4->5,7->2,7->3,9->1,9->4] | 1.00 | 539.384146341 | 29 | 164 | 0 | 0 |
| Path 4 | C00031->C00062:[4->10,4->5,7->10,7->2,7->3,7->5,9->1,9->4] | 1.00 | 360.927835052 | 31 | 194 | 0 | 0 |
| Path 5 | C00031->C00062:[4->10,4->5,7->2,7->3,9->1,9->4] | 1.00 | 361.37012987 | 25 | 154 | 0 | 0 |
| Path 6 | C00031->C00062:[4->10,4->5,7->2,7->3,9->1,9->4] | 1.00 | 360.051282051 | 30 | 195 | 0 | 0 |
| Path 7 | C00031->C00062:[4->10,4->5,7->10,7->2,7->3,7->5,9->1,9->4] | 1.00 | 371.757961783 | 29 | 157 | 0 | 0 |
| Path 8 | C00031->C00062:[4->10,4->5,7->2,7->3,9->1,9->4] | 1.00 | 365.126582278 | 27 | 158 | 0 | 0 |
| Path 9 | C00031->C00062:[4->10,4->5,7->2,7->3,9->1,9->4] | 1.00 | 370.607594937 | 28 | 158 | 0 | 0 |
| Path 10 | C00031->C00062:[4->10,4->5,7->10,7->2,7->3,7->5,9->1,9->4] | 1.00 | 366.242038217 | 28 | 157 | 0 | 0 |
| Path 11 | C00031->C00062:[7->10,7->5] | 0.33 | 179.858974359 | 19 | 234 | 2 | 3 |
| Path 12 | C00031->C00062:[4->10,4->3,4->5] | 0.50 | 200.008368201 | 24 | 239 | 2 | 3 |
| Path 13 | C00031->C00062:[4->10,4->5] | 0.33 | 1556.61538462 | 14 | 26 | 0 | 0 |
| Path 14 | C00031->C00062:[4->10,4->3,4->5,7->10,7->3,7->5] | 0.50 | 219.612903226 | 25 | 279 | 0 | 0 |
| Path 15 | C00031->C00062:[4->10,4->5,7->10,7->5] | 0.33 | 194.557377049 | 24 | 244 | 2 | 3 |
| Path 16 | C00031->C00062:[4->10,4->5] | 0.33 | 187.630434783 | 20 | 230 | 2 | 3 |
| Path 17 | C00031->C00062:[4->10,4->3,4->5,7->10,7->3,7->5] | 0.50 | 478.769230769 | 21 | 39 | 0 | 0 |
| Path 18 | C00031->C00062:[4->10,4->3,4->5] | 0.50 | 560.8125 | 20 | 32 | 0 | 0 |
| Path 19 | C00031->C00062:[4->10,7->2,9->4] | 0.50 | 341.08 | 21 | 125 | 0 | 0 |
| Path 20 | C00031->C00062:[7->10,7->2,9->4] | 0.50 | 328.801652893 | 19 | 121 | 0 | 0 |
| Path 21 | C00031->C00062:[7->10,7->5] | 0.33 | 313.554216867 | 19 | 83 | 0 | 0 |
| Path 22 | C00031->C00062:[4->10,4->5,7->2,9->4] | 0.67 | 334.528571429 | 23 | 140 | 0 | 0 |
| Path 23 | C00031->C00062:[4->10,4->5] | 0.33 | 479.0 | 10 | 16 | 0 | 0 |
| Path 24 | C00031->C00062:[4->10,4->3,4->5] | 0.50 | 318.748120301 | 30 | 266 | 2 | 3 |
| Path 25 | C00031->C00062:[4->10,4->5] | 0.33 | 327.1 | 18 | 50 | 0 | 0 |
| Path 26 | C00031->C00062:[4->10,4->5,7->2,7->3,9->1,9->4] | 1.00 | 338.197802198 | 29 | 182 | 0 | 0 |
| Path 27 | C00031->C00062:[4->10,4->3,4->5] | 0.50 | 314.446153846 | 27 | 260 | 2 | 3 |
| Path 28 | C00031->C00062:[4->10,4->3,4->5] | 0.50 | 363.349514563 | 29 | 103 | 0 | 0 |
| Path 29 | C00031->C00062:[4->10,4->3,4->5] | 0.50 | 189.788546256 | 16 | 227 | 0 | 0 |
| Path 30 | C00031->C00062:[4->10,4->5,7->10,7->5] | 0.33 | 450.407407407 | 15 | 27 | 0 | 0 |
| Path 31 | C00031->C00062:[4->10] | 0.17 | 1635.08333333 | 12 | 24 | 0 | 0 |
| Path 32 | C00031->C00062:[4->10,4->5] | 0.33 | 530.631578947 | 12 | 19 | 0 | 0 |
| Path 33 | C00031->C00062:[4->10,4->5,7->10,7->5] | 0.33 | 457.1 | 16 | 30 | 0 | 0 |
| Path 34 | C00031->C00062:[4->10,4->3,4->5,7->10,7->3,7->5] | 0.50 | 205.033057851 | 23 | 242 | 0 | 0 |
| Path 35 | C00031->C00062:[4->10] | 0.17 | 521.428571429 | 9 | 14 | 0 | 0 |
| Path 36 | C00031->C00062:[7->10,7->5] | 0.33 | 201.501845018 | 22 | 271 | 2 | 3 |
| Path 37 | C00031->C00062:[4->10,4->3,4->5] | 0.50 | 215.078358209 | 21 | 268 | 0 | 0 |
| Path 38 | C00031->C00062:[4->10,4->3,4->5] | 0.50 | 323.356846473 | 22 | 241 | 0 | 0 |
| Path 39 | C00031->C00062:[4->10,4->5,7->10,7->2,7->5,9->4] | 0.67 | 346.496503497 | 27 | 143 | 0 | 0 |
| Path 40 | C00031->C00062:[4->10,4->5] | 0.33 | 209.771217712 | 24 | 271 | 2 | 3 |
| Path 41 | C00031->C00062:[7->10,7->5] | 0.33 | 293.586956522 | 16 | 46 | 0 | 0 |
| Path 42 | C00031->C00062:[4->10,4->3,4->5,7->10,7->3,7->5] | 0.50 | 218.777385159 | 29 | 283 | 2 | 3 |
| Path 43 | C00031->C00062:[4->10,4->5,7->10,7->5] | 0.33 | 194.132780083 | 24 | 241 | 2 | 3 |
| Path 44 | C00031->C00062:[4->10,4->3,4->5,7->10,7->3,7->5] | 0.50 | 199.911764706 | 21 | 238 | 0 | 0 |
| Path 45 | C00031->C00062:[4->10,4->3,4->5] | 0.50 | 218.557971014 | 27 | 276 | 2 | 3 |
| Path 46 | C00031->C00062:[4->10,4->3,4->5,7->10,7->3,7->5] | 0.50 | 204.308943089 | 27 | 246 | 2 | 3 |
| Path 47 | C00031->C00062:[4->10,4->3,4->5] | 0.50 | 317.40530303 | 29 | 264 | 2 | 3 |
| Path 48 | C00031->C00062:[4->10,4->5] | 0.33 | 485.052631579 | 11 | 19 | 0 | 0 |
| Path 49 | C00031->C00062:[4->10,4->3,4->5] | 0.50 | 193.603524229 | 17 | 227 | 0 | 0 |
| Path 50 | C00031->C00062:[4->10,4->5,7->10,7->2,7->3,7->5,9->1,9->4] | 1.00 | 339.016574586 | 30 | 181 | 0 | 0 |
| Path 51 | C00031->C00062:[4->10,4->3,4->5] | 0.50 | 424.015384615 | 20 | 65 | 0 | 0 |
| Path 52 | C00031->C00062:[4->10,4->5] | 0.33 | 321.942528736 | 20 | 87 | 0 | 0 |
| Path 53 | C00031->C00062:[4->10,4->3,4->5] | 0.50 | 199.256 | 24 | 250 | 2 | 3 |
| Path 54 | C00031->C00062:[4->10,4->3,4->5,7->10,7->3,7->5] | 0.50 | 209.373584906 | 30 | 265 | 2 | 3 |
| Path 55 | C00031->C00062:[4->10,4->3,4->5] | 0.50 | 1259.18421053 | 21 | 38 | 0 | 0 |
| Path 56 | C00031->C00062:[4->10,4->5] | 0.33 | 388.142857143 | 14 | 56 | 0 | 0 |
| Path 57 | C00031->C00062:[4->10,4->5,7->2,7->3,9->1,9->4] | 1.00 | 334.342696629 | 27 | 178 | 0 | 0 |
| Path 58 | C00031->C00062:[4->10,4->3,4->5] | 0.50 | 214.275735294 | 25 | 272 | 2 | 3 |
| Path 59 | C00031->C00062:[4->10,4->3,4->5,7->10,7->3,7->5] | 0.50 | 222.311258278 | 32 | 302 | 2 | 3 |
| Path 60 | C00031->C00062:[7->10] | 0.17 | 394.333333333 | 9 | 18 | 0 | 0 |
| Path 61 | C00031->C00062:[4->10,4->3,4->5] | 0.50 | 201.611111111 | 25 | 252 | 2 | 3 |
| Path 62 | C00031->C00062:[4->10,4->3,4->5,7->10,7->3,7->5] | 0.50 | 206.105660377 | 29 | 265 | 2 | 3 |
| Path 63 | C00031->C00062:[4->10,4->3,4->5] | 0.50 | 195.792 | 23 | 250 | 2 | 3 |
| Path 64 | C00031->C00062:[4->10,4->3,4->5] | 0.50 | 198.174603175 | 24 | 252 | 2 | 3 |
| Path 65 | C00031->C00062:[4->10,4->5] | 0.33 | 337.867924528 | 19 | 53 | 0 | 0 |
| Path 66 | C00031->C00062:[4->10,4->3,4->5,7->10,7->3,7->5] | 0.50 | 201.45210728 | 27 | 261 | 2 | 3 |
| Path 67 | C00031->C00062:[4->10,4->5,7->2,7->3,9->1,9->4] | 1.00 | 344.124137931 | 27 | 145 | 0 | 0 |
| Path 68 | C00031->C00062:[4->10,4->3,4->5] | 0.50 | 199.077922078 | 19 | 231 | 0 | 0 |
| Path 69 | C00031->C00062:[4->10,4->3,4->5] | 0.50 | 204.145669291 | 26 | 254 | 2 | 3 |
| Path 70 | C00031->C00062:[4->10,4->3,4->5] | 0.50 | 206.42578125 | 27 | 256 | 2 | 3 |
| Path 71 | C00031->C00062:[4->10,4->3,4->5,7->10,7->3,7->5] | 0.50 | 481.651162791 | 23 | 43 | 0 | 0 |
| Path 72 | C00031->C00062:[4->10,4->5,7->2,7->3,9->1,9->4] | 1.00 | 333.283687943 | 24 | 141 | 0 | 0 |
| Path 73 | C00031->C00062:[4->10,4->5] | 0.33 | 191.875536481 | 21 | 233 | 2 | 3 |
| Path 74 | C00031->C00062:[7->10,7->5] | 0.33 | 183.55982906 | 20 | 234 | 2 | 3 |
| Path 75 | C00031->C00062:[4->10,4->3,4->5] | 0.50 | 428.985507246 | 22 | 69 | 0 | 0 |
| Path 76 | C00031->C00062:[4->10,4->5,7->10,7->5] | 0.33 | 190.539419087 | 23 | 241 | 2 | 3 |
| Path 77 | C00031->C00062:[4->10,4->3,4->5] | 0.50 | 1187.80952381 | 23 | 42 | 0 | 0 |
| Path 78 | C00031->C00062:[4->10,7->2,9->4] | 0.50 | 338.43030303 | 24 | 165 | 0 | 0 |
| Path 79 | C00031->C00062:[4->10,4->3,4->5] | 0.50 | 377.348484848 | 26 | 66 | 0 | 0 |
| Path 80 | C00031->C00062:[4->10,4->3,4->5,7->10,7->3,7->5] | 0.50 | 222.832752613 | 31 | 287 | 2 | 3 |
| Path 81 | C00031->C00062:[4->10,7->2,9->4] | 0.50 | 335.117283951 | 23 | 162 | 0 | 0 |
| Path 82 | C00031->C00062:[4->10,4->3,4->5] | 0.50 | 214.059925094 | 20 | 267 | 0 | 0 |
| Path 83 | C00031->C00062:[4->10,7->2,9->4] | 0.50 | 345.2109375 | 22 | 128 | 0 | 0 |
| Path 84 | C00031->C00062:[4->10,4->3,4->5,7->10,7->3,7->5] | 0.50 | 500.974358974 | 22 | 39 | 0 | 0 |
| Path 85 | C00031->C00062:[4->10,4->3,4->5] | 0.50 | 357.434343434 | 27 | 99 | 0 | 0 |
| Path 86 | C00031->C00062:[4->10,4->5,7->2,9->4] | 0.67 | 345.409722222 | 26 | 144 | 0 | 0 |
| Path 87 | C00031->C00062:[4->10,4->5,7->2,9->4] | 0.67 | 339.395833333 | 25 | 144 | 0 | 0 |
| Path 88 | C00031->C00062:[4->10,4->3,4->5,7->10,7->3,7->5] | 0.50 | 196.273109244 | 20 | 238 | 0 | 0 |
| Path 89 | C00031->C00062:[4->10,4->3,4->5] | 0.50 | 390.46969697 | 27 | 66 | 0 | 0 |
| Path 90 | C00031->C00062:[4->10,4->5] | 0.33 | 485.15 | 12 | 20 | 0 | 0 |
| Path 91 | C00031->C00062:[4->10,4->3,4->5,7->10,7->3,7->5] | 0.50 | 218.453020134 | 30 | 298 | 2 | 3 |
| Path 92 | C00031->C00062:[4->10,4->3,4->5] | 0.50 | 200.736220472 | 25 | 254 | 2 | 3 |
| Path 93 | C00031->C00062:[4->10,4->3,4->5] | 0.50 | 320.210970464 | 20 | 237 | 0 | 0 |
| Path 94 | C00031->C00062:[4->10,4->5] | 0.33 | 316.131147541 | 25 | 244 | 2 | 3 |
| Path 95 | C00031->C00062:[4->10,7->2,9->4] | 0.50 | 552.422222222 | 24 | 135 | 0 | 0 |
| Path 96 | C00031->C00062:[4->10,4->5] | 0.33 | 189.435897436 | 21 | 234 | 2 | 3 |
| Path 97 | C00031->C00062:[4->10,7->2,9->4] | 0.50 | 334.152 | 20 | 125 | 0 | 0 |
| Path 98 | C00031->C00062:[4->10,4->3,4->5,7->10,7->3,7->5] | 0.50 | 380.230769231 | 27 | 65 | 0 | 0 |
| Path 99 | C00031->C00062:[4->10,4->5,7->10,7->5] | 0.33 | 1191.97297297 | 18 | 37 | 0 | 0 |
| Path 100 | C00031->C00062:[4->10,4->3,4->5,7->10,7->3,7->5] | 0.50 | 410.421052632 | 24 | 76 | 0 | 0 |
| Path 101 | C00031->C00062:[4->10,4->3,4->5] | 0.50 | 368.806451613 | 24 | 62 | 0 | 0 |
| Path 102 | C00031->C00062:[4->10,4->3,4->5,7->10,7->3,7->5] | 0.50 | 393.553846154 | 28 | 65 | 0 | 0 |
| Path 103 | C00031->C00062:[4->10,4->5] | 0.33 | 208.744444444 | 23 | 270 | 2 | 3 |
| Path 104 | C00031->C00062:[4->10,4->3,4->5,7->10,7->3,7->5] | 0.50 | 215.392727273 | 23 | 275 | 0 | 0 |
| Path 105 | C00031->C00062:[4->10,4->5,7->2,9->4] | 0.67 | 335.333333333 | 26 | 177 | 0 | 0 |
| Path 106 | C00031->C00062:[4->10,4->3,4->5,7->10,7->3,7->5] | 0.50 | 208.277153558 | 30 | 267 | 2 | 3 |
| Path 107 | C00031->C00062:[4->10,4->3,4->5] | 0.50 | 216.124567474 | 27 | 289 | 2 | 3 |
| Path 108 | C00031->C00062:[4->10,4->5,7->10,7->2,7->5,9->4] | 0.67 | 340.440559441 | 26 | 143 | 0 | 0 |
| Path 109 | C00031->C00062:[4->10,4->5] | 0.33 | 309.78 | 17 | 50 | 0 | 0 |
| Path 110 | C00031->C00062:[4->10,4->3,4->5] | 0.50 | 320.697959184 | 26 | 245 | 2 | 3 |
| Path 111 | C00031->C00062:[4->10,4->3,4->5] | 0.50 | 194.060869565 | 17 | 230 | 0 | 0 |
| Path 112 | C00031->C00062:[7->10] | 0.17 | 340.945454545 | 11 | 55 | 0 | 0 |
| Path 113 | C00031->C00062:[4->10,4->5,7->2,7->3,9->1,9->4] | 1.00 | 528.483443709 | 28 | 151 | 0 | 0 |
| Path 114 | C00031->C00062:[4->10,4->5] | 0.33 | 205.277153558 | 22 | 267 | 2 | 3 |
| Path 115 | C00031->C00062:[4->10,4->5] | 0.33 | 380.830188679 | 13 | 53 | 0 | 0 |
| Path 116 | C00031->C00062:[7->10,7->5] | 0.33 | 416.4 | 11 | 20 | 0 | 0 |
| Path 117 | C00031->C00062:[4->10,4->5] | 0.33 | 188.158798283 | 20 | 233 | 2 | 3 |
| Path 118 | C00031->C00062:[4->10,4->5,7->2,9->4] | 0.67 | 340.714285714 | 24 | 140 | 0 | 0 |
| Path 119 | C00031->C00062:[4->10] | 0.17 | 379.740740741 | 12 | 54 | 0 | 0 |
| Path 120 | C00031->C00062:[4->10,4->3,4->5,7->10,7->3,7->5] | 0.50 | 200.269709544 | 21 | 241 | 0 | 0 |
| Path 121 | C00031->C00062:[4->10,4->5,7->10,7->5] | 0.33 | 418.333333333 | 14 | 27 | 0 | 0 |
| Path 122 | C00031->C00062:[4->10,4->5,7->10,7->5] | 0.33 | 213.494661922 | 27 | 281 | 2 | 3 |
| Path 123 | C00031->C00062:[7->10,7->2,9->4] | 0.50 | 335.958677686 | 20 | 121 | 0 | 0 |
| Path 124 | C00031->C00062:[7->10,7->5] | 0.33 | 373.1 | 10 | 20 | 0 | 0 |
| Path 125 | C00031->C00062:[4->10,4->5] | 0.33 | 389.877192982 | 15 | 57 | 0 | 0 |
| Path 126 | C00031->C00062:[4->10,4->5] | 0.33 | 193.136752137 | 22 | 234 | 2 | 3 |
| Path 127 | C00031->C00062:[4->10,4->3,4->5,7->10,7->3,7->5] | 0.50 | 203.863070539 | 22 | 241 | 0 | 0 |
| Path 128 | C00031->C00062:[4->10,4->3,4->5,7->10,7->3,7->5] | 0.50 | 321.1015625 | 30 | 256 | 2 | 3 |
| Path 129 | C00031->C00062:[4->10,4->3,4->5] | 0.50 | 210.613636364 | 19 | 264 | 0 | 0 |
| Path 130 | C00031->C00062:[4->10,4->3,4->5,7->10,7->3,7->5] | 0.50 | 201.454545455 | 22 | 242 | 0 | 0 |
| Path 131 | C00031->C00062:[4->10,4->3,4->5] | 0.50 | 194.736170213 | 22 | 235 | 2 | 3 |
| Path 132 | C00031->C00062:[4->10,4->5,7->10,7->5] | 0.33 | 372.109375 | 17 | 64 | 0 | 0 |
| Path 133 | C00031->C00062:[4->10,4->3,4->5] | 0.50 | 197.826086957 | 18 | 230 | 0 | 0 |
| Path 134 | C00031->C00062:[4->10,4->3,4->5,7->10,7->3,7->5] | 0.50 | 205.732 | 28 | 250 | 2 | 3 |
| Path 135 | C00031->C00062:[7->10,7->5] | 0.33 | 312.413043478 | 17 | 46 | 0 | 0 |
| Path 136 | C00031->C00062:[4->10,4->3,4->5] | 0.50 | 323.734939759 | 28 | 249 | 2 | 3 |
| Path 137 | C00031->C00062:[4->10,4->3,4->5] | 0.50 | 533.75 | 19 | 32 | 0 | 0 |
| Path 138 | C00031->C00062:[4->10,4->5,7->10,7->5] | 0.33 | 198.106557377 | 25 | 244 | 2 | 3 |
| Path 139 | C00031->C00062:[4->10,4->3,4->5,7->10,7->3,7->5] | 0.50 | 316.406593407 | 32 | 273 | 2 | 3 |
| Path 140 | C00031->C00062:[4->10,4->5,7->10,7->5] | 0.33 | 378.611940299 | 18 | 67 | 0 | 0 |
| Path 141 | C00031->C00062:[4->10,4->3,4->5] | 0.50 | 203.631799163 | 25 | 239 | 2 | 3 |
| Path 142 | C00031->C00062:[4->10,4->5,7->2,9->4] | 0.67 | 530.946666667 | 27 | 150 | 0 | 0 |
| Path 143 | C00031->C00062:[4->10] | 0.17 | 469.764705882 | 9 | 17 | 0 | 0 |
| Path 144 | C00031->C00062:[4->10,4->3,4->5,7->10,7->3,7->5] | 0.50 | 501.790697674 | 24 | 43 | 0 | 0 |
| Path 145 | C00031->C00062:[4->10,4->3,4->5,7->10,7->3,7->5] | 0.50 | 365.049019608 | 30 | 102 | 0 | 0 |
| Path 146 | C00031->C00062:[4->10,4->5,7->2,7->3,9->1,9->4] | 1.00 | 339.425531915 | 25 | 141 | 0 | 0 |
| Path 147 | C00031->C00062:[4->10,4->5,7->10,7->2,7->3,7->5,9->1,9->4] | 1.00 | 339.180555556 | 27 | 144 | 0 | 0 |
| Path 148 | C00031->C00062:[4->10,4->5,7->10,7->2,7->5,9->4] | 0.67 | 340.016666667 | 29 | 180 | 0 | 0 |
| Path 149 | C00031->C00062:[7->10,7->2,9->4] | 0.50 | 331.044303797 | 22 | 158 | 0 | 0 |
| Path 150 | C00031->C00062:[4->10,4->3,4->5] | 0.50 | 195.329004329 | 18 | 231 | 0 | 0 |
| Path 151 | C00031->C00062:[4->10,4->3,4->5] | 0.50 | 203.04296875 | 26 | 256 | 2 | 3 |
| Path 152 | C00031->C00062:[4->10,4->3,4->5] | 0.50 | 220.133105802 | 29 | 293 | 2 | 3 |
| Path 153 | C00031->C00062:[4->10,4->3,4->5] | 0.50 | 214.174216028 | 26 | 287 | 2 | 3 |
| Path 154 | C00031->C00062:[4->10,4->3,4->5] | 0.50 | 198.421276596 | 23 | 235 | 2 | 3 |
| Path 155 | C00031->C00062:[4->10,4->3,4->5,7->10,7->3,7->5] | 0.50 | 209.196 | 29 | 250 | 2 | 3 |
| Path 156 | C00031->C00062:[4->10,7->2,9->4] | 0.50 | 338.4453125 | 21 | 128 | 0 | 0 |
| Path 157 | C00031->C00062:[4->10,4->3,4->5,7->10,7->3,7->5] | 0.50 | 200.788617886 | 26 | 246 | 2 | 3 |
| Path 158 | C00031->C00062:[4->10,4->5] | 0.33 | 533.125 | 11 | 16 | 0 | 0 |
| Path 159 | C00031->C00062:[4->10,4->5] | 0.33 | 328.455555556 | 21 | 90 | 0 | 0 |
| Path 160 | C00031->C00062:[4->10,4->3,4->5,7->10,7->3,7->5] | 0.50 | 315.081180812 | 31 | 271 | 2 | 3 |
| Path 161 | C00031->C00062:[4->10] | 0.17 | 459.571428571 | 8 | 14 | 0 | 0 |
| Path 162 | C00031->C00062:[4->10,4->3,4->5,7->10,7->3,7->5] | 0.50 | 415.3875 | 26 | 80 | 0 | 0 |
| Path 163 | C00031->C00062:[4->10,4->5,7->10,7->5] | 0.33 | 428.233333333 | 15 | 30 | 0 | 0 |
| Path 164 | C00031->C00062:[4->10,4->3,4->5,7->10,7->3,7->5] | 0.50 | 224.111842105 | 33 | 304 | 2 | 3 |
| Path 165 | C00031->C00062:[4->10,4->5,7->10,7->5] | 0.33 | 313.657370518 | 27 | 251 | 2 | 3 |
| Path 166 | C00031->C00062:[4->10,4->3,4->5,7->10,7->3,7->5] | 0.50 | 211.520599251 | 31 | 267 | 2 | 3 |
| Path 167 | C00031->C00062:[4->10,4->5] | 0.33 | 1417.03333333 | 16 | 30 | 0 | 0 |
| Path 168 | C00031->C00062:[4->10,4->3,4->5,7->10,7->3,7->5] | 0.50 | 203.692015209 | 28 | 263 | 2 | 3 |
| Path 169 | C00031->C00062:[4->10,4->5] | 0.33 | 312.904166667 | 23 | 240 | 2 | 3 |
| Path 170 | C00031->C00062:[4->10,4->3,4->5,7->10,7->3,7->5] | 0.50 | 206.984790875 | 29 | 263 | 2 | 3 |
| Path 171 | C00031->C00062:[4->10,4->3,4->5] | 0.50 | 773.25 | 28 | 72 | 0 | 0 |
| Path 172 | C00031->C00062:[4->10,4->5,7->2,7->3,9->1,9->4] | 1.00 | 338.151724138 | 26 | 145 | 0 | 0 |
| Path 173 | C00031->C00062:[4->10,4->3,4->5] | 0.50 | 537.178571429 | 17 | 28 | 0 | 0 |
| Path 174 | C00031->C00062:[4->10,4->5,7->10,7->2,7->3,7->5,9->1,9->4] | 1.00 | 345.194444444 | 28 | 144 | 0 | 0 |
| Path 175 | C00031->C00062:[4->10,4->3,4->5,7->10,7->3,7->5] | 0.50 | 204.770114943 | 28 | 261 | 2 | 3 |
| Path 176 | C00031->C00062:[4->10,4->3,4->5,7->10,7->3,7->5] | 0.50 | 220.303333333 | 31 | 300 | 2 | 3 |
| Path 177 | C00031->C00062:[4->10,4->5] | 0.33 | 528.45 | 13 | 20 | 0 | 0 |
| Path 178 | C00031->C00062:[7->10,7->5] | 0.33 | 350.561403509 | 13 | 57 | 0 | 0 |
| Path 179 | C00031->C00062:[4->10,4->5,7->10,7->5] | 0.33 | 210.215827338 | 26 | 278 | 2 | 3 |
| Path 180 | C00031->C00062:[4->10] | 0.17 | 520.705882353 | 10 | 17 | 0 | 0 |
| Path 181 | C00031->C00062:[4->10,4->3,4->5] | 0.50 | 218.237113402 | 28 | 291 | 2 | 3 |
| Path 182 | C00031->C00062:[4->10] | 0.17 | 371.647058824 | 11 | 51 | 0 | 0 |
| Path 183 | C00031->C00062:[4->10,4->3,4->5] | 0.50 | 568.107142857 | 18 | 28 | 0 | 0 |
| Path 184 | C00031->C00062:[4->10,4->3,4->5] | 0.50 | 315.832061069 | 28 | 262 | 2 | 3 |
| Path 185 | C00031->C00062:[4->10,4->3,4->5] | 0.50 | 382.774193548 | 25 | 62 | 0 | 0 |
| Path 186 | C00031->C00062:[7->10] | 0.17 | 346.222222222 | 8 | 18 | 0 | 0 |
| Path 187 | C00031->C00062:[4->10,4->3,4->5,7->10,7->3,7->5] | 0.50 | 218.651079137 | 24 | 278 | 0 | 0 |
| Path 188 | C00031->C00062:[4->10,4->5,7->2,9->4] | 0.67 | 339.187845304 | 28 | 181 | 0 | 0 |
| Path 189 | C00031->C00062:[4->10,4->3,4->5,7->10,7->3,7->5] | 0.50 | 1050.6122449 | 25 | 49 | 0 | 0 |
| Path 190 | C00031->C00062:[4->10,4->5] | 0.33 | 183.865217391 | 19 | 230 | 2 | 3 |
| Path 191 | C00031->C00062:[4->10,4->5] | 0.33 | 804.95 | 21 | 60 | 0 | 0 |
| Path 192 | C00031->C00062:[4->10,4->5] | 0.33 | 321.528301887 | 18 | 53 | 0 | 0 |
