# Supplementary material for: 13C-metabolic flux ratio and novel carbon path analyses confirmed that Trichoderma reesei uses primarily the respirative pathway also on the preferred carbon source glucose
Source: BMC Syst Biol. 2009 Oct 29;3:104. doi: 10.1186/1752-0509-3-104 (PMC2776023; doi:10.1186/1752-0509-3-104)
Supplement: Additional file 1 — Pathways discovered in ReTrace carbon path analysis. Graphical and tabular representations of amino acid synthesis pathways discovered in ReTrace carbon path analysis [21]. Self-contained web site: unpack zip archive and open index.html with a web browser. [file 1752-0509-3-104-S1.zip › AF1-treesei/pathways-C00117-to-C00130.html]

Pathways from C00117 to C00130


**Pathways from C00117 to C00130**

**Sources:** D-Ribose 5-phosphate; (C00117)

**Target:**IMP; (C00130)

|  | Composite mapping | Z | Average score | Rpairs | Reactions | Zero scores | Scores under threshold |
| --- | --- | --- | --- | --- | --- | --- | --- |
| Path 1 | C00117->C00130:[10->8,12->3,6->17,7->12,8->13] | 0.50 | 338.4 | 4 | 5 | 0 | 0 |
| Path 2 | C00117->C00130:[6->10,6->11] | 0.20 | 494.567567568 | 28 | 74 | 0 | 0 |
| Path 3 | C00117->C00130:[6->11] | 0.10 | 657.666666667 | 15 | 24 | 0 | 0 |
| Path 4 | C00117->C00130:[10->8,12->3,6->10,6->11,6->17,6->2,6->4,7->12,8->13] | 0.90 | 566.75 | 34 | 52 | 0 | 1 |
| Path 5 | C00117->C00130:[10->8,12->3,6->10,6->11,6->17,6->2,6->4,7->12,8->13] | 0.90 | 540.93442623 | 37 | 61 | 0 | 1 |
| Path 6 | C00117->C00130:[10->8,12->3,6->11,6->17,7->12,8->13] | 0.60 | 620.869565217 | 17 | 23 | 0 | 0 |
| Path 7 | C00117->C00130:[6->11,6->17,7->12,8->13] | 0.40 | 651.4375 | 25 | 48 | 0 | 0 |
| Path 8 | C00117->C00130:[6->10,6->11] | 0.20 | 588.818181818 | 22 | 33 | 0 | 0 |
| Path 9 | C00117->C00130:[6->10] | 0.10 | 616.434782609 | 16 | 23 | 0 | 0 |
| Path 10 | C00117->C00130:[10->8,12->3,6->10,6->11,6->17,6->2,6->4,7->12,8->13] | 0.90 | 564.62745098 | 33 | 51 | 0 | 1 |
| Path 11 | C00117->C00130:[10->8,12->3,6->11,6->17,7->12,8->13] | 0.60 | 619.875 | 16 | 24 | 0 | 0 |
| Path 12 | C00117->C00130:[6->11,6->17,7->12,8->13] | 0.40 | 606.763157895 | 23 | 38 | 0 | 0 |
| Path 13 | C00117->C00130:[10->8,12->3,6->10,6->11,6->17,6->2,6->4,7->12,8->13] | 0.90 | 599.761904762 | 30 | 42 | 0 | 1 |
| Path 14 | C00117->C00130:[6->11] | 0.10 | 677.45 | 13 | 20 | 0 | 0 |
| Path 15 | C00117->C00130:[6->11,6->17,7->12,8->13] | 0.40 | 701.344827586 | 21 | 29 | 0 | 0 |
| Path 16 | C00117->C00130:[10->11,10->8,12->3,6->11,6->17,7->12,8->13] | 0.60 | 570.475 | 22 | 40 | 0 | 0 |
| Path 17 | C00117->C00130:[10->8,12->3,6->10,6->11,6->17,6->4,7->12,8->13] | 0.80 | 587.609756098 | 28 | 41 | 0 | 0 |
| Path 18 | C00117->C00130:[6->10,6->11] | 0.20 | 623.75 | 20 | 28 | 0 | 0 |
| Path 19 | C00117->C00130:[6->10,6->11] | 0.20 | 656.233333333 | 22 | 30 | 0 | 0 |
| Path 20 | C00117->C00130:[6->11] | 0.10 | 680.636363636 | 14 | 22 | 0 | 0 |
| Path 21 | C00117->C00130:[10->8,12->3,6->10,6->11,6->17,6->4,7->12,8->13] | 0.80 | 612.475 | 28 | 40 | 0 | 0 |
| Path 22 | C00117->C00130:[6->10,6->11] | 0.20 | 566.785714286 | 17 | 28 | 0 | 0 |
| Path 23 | C00117->C00130:[6->10,6->11] | 0.20 | 639.928571429 | 20 | 28 | 0 | 0 |
| Path 24 | C00117->C00130:[6->11] | 0.10 | 535.148148148 | 13 | 27 | 0 | 0 |
| Path 25 | C00117->C00130:[6->11,6->17,7->12,8->13] | 0.40 | 707.533333333 | 22 | 30 | 0 | 0 |
| Path 26 | C00117->C00130:[10->8,12->3,6->10,6->11,6->17,6->4,7->12,8->13] | 0.80 | 546.653846154 | 31 | 52 | 0 | 0 |
| Path 27 | C00117->C00130:[6->11,6->17,7->12,8->13] | 0.40 | 643.27027027 | 24 | 37 | 0 | 0 |
| Path 28 | C00117->C00130:[10->8,12->3,6->10,6->11,6->17,6->4,7->12,8->10,8->11,8->13,8->4] | 0.80 | 554.3 | 37 | 80 | 0 | 0 |
| Path 29 | C00117->C00130:[6->10,6->11] | 0.20 | 547.862068966 | 18 | 29 | 0 | 0 |
| Path 30 | C00117->C00130:[10->8,12->3,6->10,6->17,6->2,7->12,8->13] | 0.70 | 634.5 | 34 | 46 | 0 | 1 |
| Path 31 | C00117->C00130:[10->8,12->3,6->10,6->11,6->17,6->2,6->4,7->12,8->13] | 0.90 | 553.436363636 | 36 | 55 | 0 | 1 |
| Path 32 | C00117->C00130:[10->8,12->3,6->11,6->17,7->12,8->13] | 0.60 | 533.666666667 | 17 | 27 | 0 | 0 |
| Path 33 | C00117->C00130:[10->8,12->3,6->10,6->11,6->17,6->2,6->4,7->12,8->13] | 0.90 | 558.930232558 | 29 | 43 | 0 | 1 |
| Path 34 | C00117->C00130:[6->11] | 0.10 | 666.421052632 | 12 | 19 | 0 | 0 |
| Path 35 | C00117->C00130:[10->8,12->3,6->10,6->17,7->12,8->13] | 0.60 | 544.365853659 | 26 | 41 | 0 | 0 |
| Path 36 | C00117->C00130:[10->8,12->3,6->10,6->11,6->17,6->2,6->4,7->12,8->13] | 0.90 | 568.021276596 | 32 | 47 | 0 | 1 |
| Path 37 | C00117->C00130:[6->10,6->11] | 0.20 | 539.230769231 | 16 | 26 | 0 | 0 |
| Path 38 | C00117->C00130:[10->8,12->3,6->10,6->11,6->17,6->2,6->4,7->12,8->13] | 0.90 | 557.904761905 | 39 | 63 | 0 | 1 |
| Path 39 | C00117->C00130:[6->11] | 0.10 | 547.379310345 | 14 | 29 | 0 | 0 |
| Path 40 | C00117->C00130:[10->8,12->3,6->10,6->11,6->17,6->2,6->4,7->12,8->13] | 0.90 | 623.154929577 | 41 | 71 | 0 | 1 |
| Path 41 | C00117->C00130:[10->8,12->3,6->10,6->17,7->12,8->13] | 0.60 | 629.970588235 | 26 | 34 | 0 | 0 |
| Path 42 | C00117->C00130:[6->10,6->11] | 0.20 | 522.206349206 | 25 | 63 | 0 | 0 |
| Path 43 | C00117->C00130:[10->8,12->3,6->10,6->11,6->17,6->2,6->4,7->12,8->13] | 0.90 | 570.849315068 | 40 | 73 | 0 | 1 |
| Path 44 | C00117->C00130:[10->8,12->3,6->10,6->17,7->12,8->13] | 0.60 | 622.181818182 | 25 | 33 | 0 | 0 |
| Path 45 | C00117->C00130:[6->10,6->11] | 0.20 | 607.096774194 | 20 | 31 | 0 | 0 |
| Path 46 | C00117->C00130:[10->8,12->3,6->11,6->17,7->12,8->13] | 0.60 | 630.56 | 17 | 25 | 0 | 0 |
| Path 47 | C00117->C00130:[10->8,12->3,6->10,6->11,6->17,6->4,7->12,8->13] | 0.80 | 615.142857143 | 26 | 35 | 0 | 0 |
| Path 48 | C00117->C00130:[10->8,12->3,6->10,6->11,6->17,6->2,6->4,7->12,8->13] | 0.90 | 576.216216216 | 41 | 74 | 0 | 1 |
| Path 49 | C00117->C00130:[10->8,12->3,6->10,6->17,6->2,7->12,8->13] | 0.70 | 606.046511628 | 31 | 43 | 0 | 1 |
| Path 50 | C00117->C00130:[10->8,12->3,6->11,6->17,7->12,8->13] | 0.60 | 582.826086957 | 17 | 23 | 0 | 0 |
| Path 51 | C00117->C00130:[6->11] | 0.10 | 501.34375 | 14 | 32 | 0 | 0 |
| Path 52 | C00117->C00130:[6->11,6->17,7->12,8->13] | 0.40 | 699.818181818 | 24 | 33 | 0 | 0 |
| Path 53 | C00117->C00130:[10->8,12->3,6->10,6->11,6->17,6->2,6->4,7->12,8->13] | 0.90 | 600.708333333 | 33 | 48 | 0 | 1 |
| Path 54 | C00117->C00130:[10->8,12->3,6->10,6->11,6->17,6->2,6->4,7->12,8->13] | 0.90 | 548.558139535 | 39 | 86 | 0 | 1 |
| Path 55 | C00117->C00130:[10->8,12->3,6->10,6->11,6->17,6->2,6->4,7->12,8->13] | 0.90 | 608.407894737 | 40 | 76 | 0 | 1 |
| Path 56 | C00117->C00130:[10->8,12->3,6->10,6->11,6->17,6->4,7->12,8->13] | 0.80 | 513.057471264 | 35 | 87 | 0 | 0 |
| Path 57 | C00117->C00130:[10->8,12->3,6->11,6->17,7->12,8->13] | 0.60 | 578.571428571 | 20 | 42 | 0 | 0 |
| Path 58 | C00117->C00130:[6->10,6->11] | 0.20 | 522.453125 | 25 | 64 | 0 | 0 |
| Path 59 | C00117->C00130:[10->8,12->3,6->17,7->12,8->13] | 0.50 | 224.333333333 | 4 | 6 | 0 | 1 |
| Path 60 | C00117->C00130:[10->8,12->3,6->11,6->17,7->12,8->13] | 0.60 | 606.208333333 | 20 | 48 | 0 | 0 |
| Path 61 | C00117->C00130:[10->8,12->3,6->10,6->11,6->17,6->2,6->4,7->12,8->13] | 0.90 | 592.428571429 | 34 | 49 | 0 | 1 |
| Path 62 | C00117->C00130:[10->8,12->3,6->10,6->11,6->17,6->2,6->4,7->12,8->13] | 0.90 | 549.428571429 | 41 | 91 | 0 | 1 |
| Path 63 | C00117->C00130:[10->8,12->3,6->10,6->11,6->17,6->4,7->12,8->10,8->11,8->13,8->4] | 0.80 | 554.827160494 | 36 | 81 | 0 | 0 |
| Path 64 | C00117->C00130:[10->8,12->3,6->10,6->17,7->12,8->13] | 0.60 | 627.342857143 | 26 | 35 | 0 | 0 |
| Path 65 | C00117->C00130:[6->10,6->11] | 0.20 | 505.55 | 21 | 40 | 0 | 0 |
| Path 66 | C00117->C00130:[10->8,12->3,6->10,6->11,6->17,6->2,6->4,7->12,8->13] | 0.90 | 594.710144928 | 39 | 69 | 0 | 1 |
| Path 67 | C00117->C00130:[6->10,6->11] | 0.20 | 613.983050847 | 27 | 59 | 0 | 0 |
| Path 68 | C00117->C00130:[10->8,12->3,6->10,6->11,6->17,6->4,7->12,8->13] | 0.80 | 584.842105263 | 26 | 38 | 0 | 0 |
| Path 69 | C00117->C00130:[6->10,6->11] | 0.20 | 572.193548387 | 19 | 31 | 0 | 0 |
| Path 70 | C00117->C00130:[6->11] | 0.10 | 632.0 | 14 | 23 | 0 | 0 |
| Path 71 | C00117->C00130:[10->8,12->11,12->3,6->17,7->12,8->13] | 0.60 | 504.47826087 | 14 | 23 | 0 | 0 |
| Path 72 | C00117->C00130:[10->8,12->3,6->10,6->11,6->17,6->2,6->4,7->12,8->13] | 0.90 | 589.772727273 | 38 | 66 | 0 | 1 |
| Path 73 | C00117->C00130:[6->11] | 0.10 | 625.27027027 | 16 | 37 | 0 | 0 |
| Path 74 | C00117->C00130:[10->8,12->3,6->10,6->11,6->17,6->4,7->12,8->10,8->11,8->13,8->4] | 0.80 | 555.0375 | 36 | 80 | 0 | 0 |
| Path 75 | C00117->C00130:[10->8,12->3,6->11,6->17,7->12,8->13] | 0.60 | 566.928571429 | 18 | 28 | 0 | 0 |
| Path 76 | C00117->C00130:[10->8,12->3,6->10,6->11,6->17,6->4,7->12,8->13] | 0.80 | 640.114285714 | 27 | 35 | 0 | 0 |
| Path 77 | C00117->C00130:[10->8,12->3,6->11,6->17,7->12,8->13] | 0.60 | 565.04 | 16 | 25 | 0 | 1 |
| Path 78 | C00117->C00130:[10->8,12->3,6->10,6->11,6->17,6->2,6->4,7->12,8->13] | 0.90 | 583.040816327 | 34 | 49 | 0 | 1 |
| Path 79 | C00117->C00130:[10->8,12->3,6->17,7->12,8->13] | 0.50 | 127.0 | 4 | 6 | 0 | 0 |
| Path 80 | C00117->C00130:[6->11,6->17,7->12,8->13] | 0.40 | 604.333333333 | 25 | 42 | 0 | 0 |
| Path 81 | C00117->C00130:[6->10,6->11] | 0.20 | 541.708333333 | 27 | 72 | 0 | 0 |
| Path 82 | C00117->C00130:[10->8,12->3,6->10,6->11,6->17,6->4,7->12,8->13] | 0.80 | 629.6 | 33 | 55 | 0 | 0 |
| Path 83 | C00117->C00130:[10->8,12->3,6->10,6->11,6->17,6->2,6->4,7->12,8->13] | 0.90 | 564.666666667 | 34 | 54 | 0 | 1 |
| Path 84 | C00117->C00130:[10->8,12->11,12->3,6->17,7->12,8->13] | 0.60 | 522.666666667 | 13 | 21 | 0 | 0 |
| Path 85 | C00117->C00130:[10->8,12->3,6->10,6->11,6->17,6->4,7->12,8->13] | 0.80 | 602.822580645 | 35 | 62 | 0 | 0 |
| Path 86 | C00117->C00130:[6->11] | 0.10 | 670.80952381 | 13 | 21 | 0 | 0 |
| Path 87 | C00117->C00130:[10->8,12->3,6->17,7->12,8->13] | 0.50 | 273.25 | 3 | 4 | 0 | 1 |
| Path 88 | C00117->C00130:[10->8,12->3,6->11,6->17,7->12,8->13] | 0.60 | 603.571428571 | 20 | 42 | 0 | 0 |
| Path 89 | C00117->C00130:[6->11] | 0.10 | 629.066666667 | 17 | 45 | 0 | 0 |
| Path 90 | C00117->C00130:[6->11] | 0.10 | 634.945945946 | 17 | 37 | 0 | 0 |
| Path 91 | C00117->C00130:[6->11] | 0.10 | 629.743589744 | 17 | 39 | 0 | 0 |
| Path 92 | C00117->C00130:[10->8,12->3,6->10,6->11,6->17,6->4,7->12,8->13] | 0.80 | 604.023809524 | 30 | 42 | 0 | 0 |
| Path 93 | C00117->C00130:[10->8,12->3,6->10,6->17,6->2,7->12,8->13] | 0.70 | 562.851851852 | 34 | 54 | 0 | 1 |
| Path 94 | C00117->C00130:[10->8,12->3,6->11,6->17,7->12,8->13] | 0.60 | 454.03030303 | 16 | 33 | 0 | 0 |
| Path 95 | C00117->C00130:[10->8,12->3,6->10,6->11,6->17,6->4,7->12,8->13] | 0.80 | 549.012345679 | 35 | 81 | 0 | 0 |
| Path 96 | C00117->C00130:[10->8,12->3,6->11,6->17,7->12,8->13] | 0.60 | 542.566666667 | 19 | 30 | 0 | 0 |
| Path 97 | C00117->C00130:[10->8,12->3,6->10,6->11,6->17,6->2,6->4,7->12,8->13] | 0.90 | 543.830508475 | 35 | 59 | 0 | 1 |
| Path 98 | C00117->C00130:[6->11] | 0.10 | 630.75 | 14 | 20 | 0 | 0 |
| Path 99 | C00117->C00130:[10->8,12->3,6->10,6->11,6->17,6->4,7->12,8->13] | 0.80 | 607.916666667 | 34 | 60 | 0 | 0 |
| Path 100 | C00117->C00130:[10->8,12->3,6->11,6->17,7->12,8->13] | 0.60 | 520.75 | 17 | 32 | 0 | 0 |
| Path 101 | C00117->C00130:[10->8,12->3,6->11,6->17,7->12,8->13] | 0.60 | 541.653846154 | 16 | 26 | 0 | 0 |
| Path 102 | C00117->C00130:[10->8,12->3,6->10,6->11,6->17,6->4,7->12,8->13] | 0.80 | 553.134146341 | 36 | 82 | 0 | 0 |
| Path 103 | C00117->C00130:[6->10,6->11] | 0.20 | 521.230769231 | 26 | 65 | 0 | 0 |
| Path 104 | C00117->C00130:[6->11,6->17,7->12,8->13] | 0.40 | 607.926829268 | 25 | 41 | 0 | 0 |
| Path 105 | C00117->C00130:[10->8,12->3,6->10,6->11,6->17,6->2,6->4,7->12,8->13] | 0.90 | 588.468085106 | 32 | 47 | 0 | 1 |
| Path 106 | C00117->C00130:[10->8,12->3,6->10,6->11,6->17,6->4,7->12,8->13] | 0.80 | 617.086956522 | 36 | 69 | 0 | 0 |
| Path 107 | C00117->C00130:[10->8,12->3,6->10,6->11,6->17,6->4,7->12,8->13] | 0.80 | 574.0 | 32 | 50 | 0 | 0 |
| Path 108 | C00117->C00130:[10->8,12->3,6->10,6->11,6->17,6->2,6->4,7->12,8->13] | 0.90 | 574.253521127 | 39 | 71 | 0 | 1 |
| Path 109 | C00117->C00130:[10->8,12->3,6->10,6->11,6->17,6->2,6->4,7->12,8->13] | 0.90 | 534.104477612 | 40 | 67 | 0 | 1 |
| Path 110 | C00117->C00130:[10->8,12->11,12->3,6->17,7->12,8->13] | 0.60 | 537.0 | 14 | 23 | 0 | 0 |
| Path 111 | C00117->C00130:[10->8,12->3,6->11,6->17,7->12,8->13] | 0.60 | 539.384615385 | 17 | 26 | 0 | 0 |
| Path 112 | C00117->C00130:[10->8,12->3,6->10,6->11,6->17,6->4,7->12,8->13] | 0.80 | 532.861111111 | 33 | 72 | 0 | 0 |
| Path 113 | C00117->C00130:[10->8,12->3,6->10,6->11,6->17,6->4,7->12,8->13] | 0.80 | 557.895833333 | 32 | 48 | 0 | 0 |
| Path 114 | C00117->C00130:[10->8,12->3,6->10,6->11,6->17,6->2,6->4,7->12,8->13] | 0.90 | 530.456140351 | 34 | 57 | 0 | 1 |
| Path 115 | C00117->C00130:[6->10,6->11] | 0.20 | 536.845070423 | 26 | 71 | 0 | 0 |
| Path 116 | C00117->C00130:[6->11,6->17,7->12,8->13] | 0.40 | 589.846153846 | 25 | 39 | 0 | 0 |
| Path 117 | C00117->C00130:[6->10,6->11] | 0.20 | 642.0 | 18 | 25 | 0 | 0 |
| Path 118 | C00117->C00130:[6->10,6->11] | 0.20 | 553.459459459 | 21 | 37 | 0 | 0 |
| Path 119 | C00117->C00130:[6->11] | 0.10 | 563.909090909 | 12 | 22 | 0 | 0 |
| Path 120 | C00117->C00130:[6->11,6->17,7->12,8->13] | 0.40 | 627.59375 | 21 | 32 | 0 | 0 |
| Path 121 | C00117->C00130:[10->8,12->3,6->10,6->11,6->17,6->2,6->4,7->12,8->13] | 0.90 | 563.086956522 | 31 | 46 | 0 | 1 |
| Path 122 | C00117->C00130:[10->8,12->3,6->10,6->11,6->17,6->4,7->12,8->13] | 0.80 | 599.95 | 28 | 40 | 0 | 0 |
| Path 123 | C00117->C00130:[6->11] | 0.10 | 605.72 | 14 | 25 | 0 | 0 |
| Path 124 | C00117->C00130:[10->8,12->3,6->10,6->11,6->17,6->2,6->4,7->12,8->13] | 0.90 | 540.740740741 | 39 | 81 | 0 | 1 |
| Path 125 | C00117->C00130:[10->8,12->3,6->11,6->17,7->12,8->13] | 0.60 | 613.851851852 | 18 | 27 | 0 | 0 |
| Path 126 | C00117->C00130:[6->10,6->11] | 0.20 | 525.523809524 | 22 | 42 | 0 | 0 |
| Path 127 | C00117->C00130:[10->8,12->3,6->11,6->17,7->12,8->13] | 0.60 | 577.785714286 | 17 | 28 | 0 | 0 |
| Path 128 | C00117->C00130:[10->8,12->3,6->10,6->11,6->17,6->2,6->4,7->12,8->13] | 0.90 | 546.931818182 | 39 | 88 | 0 | 1 |
| Path 129 | C00117->C00130:[10->8,12->3,6->10,6->11,6->17,6->2,6->4,7->12,8->13] | 0.90 | 636.630434783 | 34 | 46 | 0 | 1 |
| Path 130 | C00117->C00130:[6->11] | 0.10 | 594.0 | 13 | 24 | 0 | 0 |
| Path 131 | C00117->C00130:[10->8,12->3,6->10,6->11,6->17,6->2,6->4,7->12,8->13] | 0.90 | 564.895833333 | 33 | 48 | 0 | 1 |
| Path 132 | C00117->C00130:[10->8,12->3,6->11,6->17,7->12,8->13] | 0.60 | 442.592592593 | 16 | 27 | 0 | 0 |
| Path 133 | C00117->C00130:[10->8,12->3,6->11,6->17,7->12,8->13] | 0.60 | 569.035714286 | 17 | 28 | 0 | 0 |
| Path 134 | C00117->C00130:[10->8,12->3,6->11,6->17,7->12,8->13] | 0.60 | 557.259259259 | 16 | 27 | 0 | 0 |
| Path 135 | C00117->C00130:[10->8,12->3,6->10,6->11,6->17,6->2,6->4,7->12,8->13] | 0.90 | 573.511111111 | 31 | 45 | 0 | 1 |
| Path 136 | C00117->C00130:[10->8,12->3,6->11,6->17,7->12,8->13] | 0.60 | 611.974358974 | 19 | 39 | 0 | 0 |
| Path 137 | C00117->C00130:[6->11,6->17,7->12,8->13] | 0.40 | 556.12195122 | 23 | 41 | 0 | 0 |
| Path 138 | C00117->C00130:[10->8,12->3,6->11,6->17,7->12,8->13] | 0.60 | 611.454545455 | 15 | 22 | 0 | 0 |
| Path 139 | C00117->C00130:[10->8,12->3,6->11,6->17,7->12,8->13] | 0.60 | 598.125 | 19 | 40 | 0 | 0 |
| Path 140 | C00117->C00130:[10->8,12->3,6->10,6->11,6->17,6->4,7->12,8->13] | 0.80 | 570.307692308 | 27 | 39 | 0 | 0 |
| Path 141 | C00117->C00130:[10->8,12->3,6->11,6->17,7->12,8->13] | 0.60 | 630.8 | 18 | 35 | 0 | 0 |
| Path 142 | C00117->C00130:[10->8,12->3,6->10,6->11,6->17,6->2,6->4,7->12,8->13] | 0.90 | 620.711111111 | 33 | 45 | 0 | 1 |
| Path 143 | C00117->C00130:[10->8,12->3,6->10,6->11,6->17,6->2,6->4,7->12,8->13] | 0.90 | 574.914893617 | 33 | 47 | 0 | 1 |
| Path 144 | C00117->C00130:[10->8,12->11,12->3,6->17,7->12,8->13] | 0.60 | 495.396226415 | 18 | 53 | 0 | 0 |
| Path 145 | C00117->C00130:[10->8,12->3,6->11,6->17,7->12,8->13] | 0.60 | 598.782608696 | 16 | 23 | 0 | 0 |
| Path 146 | C00117->C00130:[6->11] | 0.10 | 615.52 | 14 | 25 | 0 | 0 |
| Path 147 | C00117->C00130:[6->10] | 0.10 | 616.88 | 19 | 25 | 0 | 0 |
| Path 148 | C00117->C00130:[10->8,12->3,6->10,6->11,6->17,6->2,6->4,7->12,8->13] | 0.90 | 600.042857143 | 40 | 70 | 0 | 1 |
| Path 149 | C00117->C00130:[6->10,6->11] | 0.20 | 586.461538462 | 26 | 52 | 0 | 0 |
| Path 150 | C00117->C00130:[10->8,12->3,6->10,6->11,6->17,6->2,6->4,7->12,8->13] | 0.90 | 561.456521739 | 31 | 46 | 0 | 1 |
| Path 151 | C00117->C00130:[6->11,6->17,7->12,8->13] | 0.40 | 666.555555556 | 24 | 36 | 0 | 0 |
| Path 152 | C00117->C00130:[6->11,6->17,7->12,8->13] | 0.40 | 697.515151515 | 23 | 33 | 0 | 0 |
| Path 153 | C00117->C00130:[10->8,12->3,6->10,6->11,6->17,6->2,6->4,7->12,8->13] | 0.90 | 531.974683544 | 37 | 79 | 0 | 1 |
| Path 154 | C00117->C00130:[6->10,6->11] | 0.20 | 558.65 | 23 | 40 | 0 | 0 |
| Path 155 | C00117->C00130:[10->8,12->3,6->10,6->11,6->17,6->4,7->12,8->13] | 0.80 | 565.944444444 | 25 | 36 | 0 | 0 |
| Path 156 | C00117->C00130:[10->8,12->3,6->11,6->17,7->12,8->13] | 0.60 | 527.84 | 15 | 25 | 0 | 0 |
| Path 157 | C00117->C00130:[10->8,12->3,6->11,6->17,7->12,8->13] | 0.60 | 623.434782609 | 16 | 23 | 0 | 0 |
| Path 158 | C00117->C00130:[10->8,12->3,6->10,6->11,6->17,6->4,7->12,8->10,8->11,8->13,8->4] | 0.80 | 564.348837209 | 39 | 86 | 0 | 0 |
| Path 159 | C00117->C00130:[10->8,12->3,6->11,6->17,7->12,8->13] | 0.60 | 567.965517241 | 17 | 29 | 0 | 0 |
| Path 160 | C00117->C00130:[10->8,12->3,6->10,6->11,6->17,6->4,7->12,8->13] | 0.80 | 507.209876543 | 35 | 81 | 0 | 0 |
| Path 161 | C00117->C00130:[10->11,10->8,12->3,6->17,7->12,8->13] | 0.60 | 555.103448276 | 15 | 29 | 0 | 0 |
| Path 162 | C00117->C00130:[10->8,12->3,6->10,6->11,6->17,6->4,7->12,8->13] | 0.80 | 536.453333333 | 35 | 75 | 0 | 0 |
| Path 163 | C00117->C00130:[10->8,12->3,6->10,6->11,6->17,6->2,6->4,7->12,8->13] | 0.90 | 570.106382979 | 32 | 47 | 0 | 1 |
| Path 164 | C00117->C00130:[10->8,12->3,6->10,6->11,6->17,6->4,7->12,8->13] | 0.80 | 531.52 | 30 | 50 | 0 | 0 |
| Path 165 | C00117->C00130:[10->8,12->3,6->10,6->11,6->17,6->4,7->12,8->10,8->11,8->13,8->4] | 0.80 | 520.724137931 | 36 | 87 | 0 | 0 |
| Path 166 | C00117->C00130:[10->11,10->8,12->11,12->3,6->17,7->12,8->13] | 0.60 | 536.2 | 22 | 35 | 0 | 0 |
| Path 167 | C00117->C00130:[6->10,6->11] | 0.20 | 604.833333333 | 19 | 30 | 0 | 0 |
| Path 168 | C00117->C00130:[6->10,6->11] | 0.20 | 615.405405405 | 24 | 37 | 0 | 0 |
| Path 169 | C00117->C00130:[10->8,12->3,6->11,6->17,7->12,8->13] | 0.60 | 538.115384615 | 16 | 26 | 0 | 0 |
| Path 170 | C00117->C00130:[6->11,6->17,7->12,8->13] | 0.40 | 659.214285714 | 27 | 56 | 0 | 0 |
| Path 171 | C00117->C00130:[10->8,12->3,6->10,6->11,6->17,6->2,6->4,7->12,8->13] | 0.90 | 535.292682927 | 39 | 82 | 0 | 1 |
| Path 172 | C00117->C00130:[10->8,12->3,6->10,6->11,6->17,6->4,7->12,8->13] | 0.80 | 537.712328767 | 34 | 73 | 0 | 0 |
| Path 173 | C00117->C00130:[6->11,6->17,7->12,8->13] | 0.40 | 693.96875 | 23 | 32 | 0 | 0 |
| Path 174 | C00117->C00130:[10->8,12->3,6->10,6->17,6->2,7->12,8->13] | 0.70 | 618.238095238 | 31 | 42 | 0 | 1 |
| Path 175 | C00117->C00130:[6->10,6->11,8->10,8->11] | 0.20 | 505.831168831 | 27 | 77 | 0 | 0 |
| Path 176 | C00117->C00130:[10->8,12->3,6->10,6->11,6->17,6->2,6->4,7->12,8->13] | 0.90 | 533.033898305 | 36 | 59 | 0 | 1 |
| Path 177 | C00117->C00130:[10->8,12->3,6->11,6->17,7->12,8->13] | 0.60 | 536.142857143 | 16 | 21 | 0 | 0 |
| Path 178 | C00117->C00130:[6->10,6->11,8->10,8->11] | 0.20 | 543.557142857 | 27 | 70 | 0 | 0 |
| Path 179 | C00117->C00130:[10->8,12->3,6->10,6->11,6->17,6->4,7->12,8->13] | 0.80 | 608.619047619 | 36 | 63 | 0 | 0 |
| Path 180 | C00117->C00130:[10->8,12->3,6->10,6->11,6->17,6->2,6->4,7->12,8->13] | 0.90 | 526.711864407 | 37 | 59 | 0 | 1 |
| Path 181 | C00117->C00130:[10->8,12->3,6->11,6->17,7->12,8->13] | 0.60 | 585.357142857 | 17 | 28 | 0 | 0 |
| Path 182 | C00117->C00130:[10->8,12->3,6->10,6->11,6->17,6->4,7->12,8->13] | 0.80 | 634.125 | 37 | 64 | 0 | 0 |
| Path 183 | C00117->C00130:[6->10] | 0.10 | 627.708333333 | 17 | 24 | 0 | 0 |
| Path 184 | C00117->C00130:[6->10,6->11] | 0.20 | 516.322580645 | 24 | 62 | 0 | 0 |
| Path 185 | C00117->C00130:[10->8,12->3,6->11,6->17,7->12,8->13] | 0.60 | 607.88 | 17 | 25 | 0 | 0 |
| Path 186 | C00117->C00130:[6->10,6->11] | 0.20 | 526.772727273 | 27 | 66 | 0 | 0 |
| Path 187 | C00117->C00130:[6->11,6->17,7->12,8->13] | 0.40 | 645.088235294 | 22 | 34 | 0 | 0 |
| Path 188 | C00117->C00130:[10->8,12->3,6->10,6->17,7->12,8->13] | 0.60 | 628.235294118 | 27 | 34 | 0 | 0 |
| Path 189 | C00117->C00130:[10->8,12->3,6->10,6->11,6->17,6->2,6->4,7->12,8->13] | 0.90 | 608.325581395 | 31 | 43 | 0 | 1 |
| Path 190 | C00117->C00130:[10->8,12->3,6->10,6->11,6->17,6->2,6->4,7->12,8->13] | 0.90 | 554.488888889 | 41 | 90 | 0 | 1 |
| Path 191 | C00117->C00130:[10->8,12->3,6->10,6->11,6->17,6->4,7->12,8->13] | 0.80 | 537.716216216 | 34 | 74 | 0 | 0 |
| Path 192 | C00117->C00130:[6->10,6->11] | 0.20 | 629.927272727 | 29 | 55 | 0 | 0 |
| Path 193 | C00117->C00130:[10->8,12->3,6->10,6->11,6->17,6->4,7->12,8->13] | 0.80 | 624.944444444 | 27 | 36 | 0 | 0 |
| Path 194 | C00117->C00130:[10->8,12->3,6->10,6->11,6->17,6->2,6->4,7->12,8->13] | 0.90 | 580.456521739 | 32 | 46 | 0 | 1 |
| Path 195 | C00117->C00130:[6->10,6->11] | 0.20 | 628.311111111 | 24 | 45 | 0 | 0 |
| Path 196 | C00117->C00130:[10->8,12->3,6->10,6->11,6->17,6->4,7->12,8->13] | 0.80 | 594.35483871 | 35 | 62 | 0 | 0 |
| Path 197 | C00117->C00130:[6->10,6->11] | 0.20 | 540.324324324 | 28 | 74 | 0 | 0 |
| Path 198 | C00117->C00130:[10->8,12->3,6->10,6->11,6->17,6->4,7->12,8->13] | 0.80 | 626.815789474 | 29 | 38 | 0 | 0 |
| Path 199 | C00117->C00130:[6->11,6->17,7->12,8->13] | 0.40 | 627.171428571 | 23 | 35 | 0 | 0 |
| Path 200 | C00117->C00130:[6->11,6->17,7->12,8->13] | 0.40 | 573.279069767 | 24 | 43 | 0 | 0 |
| Path 201 | C00117->C00130:[6->11] | 0.10 | 662.19047619 | 15 | 21 | 0 | 0 |
| Path 202 | C00117->C00130:[10->8,12->3,6->10,6->11,6->17,6->2,6->4,7->12,8->13] | 0.90 | 567.102040816 | 35 | 49 | 0 | 1 |
| Path 203 | C00117->C00130:[10->8,12->3,6->10,6->11,6->17,6->2,6->4,7->12,8->13] | 0.90 | 607.111111111 | 37 | 54 | 0 | 1 |
| Path 204 | C00117->C00130:[10->8,12->3,6->11,6->17,7->12,8->13] | 0.60 | 507.966666667 | 16 | 30 | 0 | 0 |
| Path 205 | C00117->C00130:[6->10,6->11] | 0.20 | 489.154929577 | 26 | 71 | 0 | 0 |
| Path 206 | C00117->C00130:[10->8,12->3,6->11,6->17,7->12,8->13] | 0.60 | 589.461538462 | 17 | 26 | 0 | 0 |
| Path 207 | C00117->C00130:[10->8,12->3,6->10,6->11,6->17,6->2,6->4,7->12,8->13] | 0.90 | 578.166666667 | 32 | 48 | 0 | 1 |
| Path 208 | C00117->C00130:[10->8,12->3,6->10,6->11,6->17,6->2,6->4,7->12,8->13] | 0.90 | 603.355555556 | 34 | 45 | 0 | 1 |
| Path 209 | C00117->C00130:[10->8,12->3,6->17,7->12,8->13] | 0.50 | 476.875 | 7 | 8 | 0 | 0 |
| Path 210 | C00117->C00130:[10->8,12->3,6->10,6->11,6->17,6->2,6->4,7->12,8->13] | 0.90 | 566.522727273 | 30 | 44 | 0 | 1 |
| Path 211 | C00117->C00130:[6->10,6->11] | 0.20 | 497.168831169 | 26 | 77 | 0 | 0 |
| Path 212 | C00117->C00130:[10->8,12->3,6->11,6->17,7->12,8->13] | 0.60 | 526.055555556 | 20 | 36 | 0 | 0 |
| Path 213 | C00117->C00130:[10->8,12->11,12->3,6->17,7->12,8->13] | 0.60 | 519.315789474 | 22 | 38 | 0 | 0 |
| Path 214 | C00117->C00130:[6->10,6->11] | 0.20 | 602.42 | 25 | 50 | 0 | 0 |
| Path 215 | C00117->C00130:[10->8,12->3,6->10,6->11,6->17,6->2,6->4,7->12,8->13] | 0.90 | 558.354166667 | 32 | 48 | 0 | 1 |
| Path 216 | C00117->C00130:[6->11,6->17,7->12,8->13] | 0.40 | 676.151515152 | 25 | 33 | 0 | 0 |
| Path 217 | C00117->C00130:[10->8,12->11,12->3,6->17,7->12,8->13] | 0.60 | 549.696969697 | 21 | 33 | 0 | 0 |
| Path 218 | C00117->C00130:[10->8,12->3,6->10,6->11,6->17,6->4,7->12,8->13] | 0.80 | 638.736842105 | 29 | 38 | 0 | 0 |
| Path 219 | C00117->C00130:[10->8,12->3,6->10,6->11,6->17,6->2,6->4,7->12,8->13] | 0.90 | 585.95 | 41 | 80 | 0 | 1 |
| Path 220 | C00117->C00130:[6->10,6->11] | 0.20 | 620.695652174 | 16 | 23 | 0 | 0 |
| Path 221 | C00117->C00130:[10->8,12->3,6->10,6->11,6->17,6->2,6->4,7->12,8->13] | 0.90 | 556.25 | 41 | 88 | 0 | 1 |
| Path 222 | C00117->C00130:[6->11] | 0.10 | 645.80952381 | 13 | 21 | 0 | 0 |
| Path 223 | C00117->C00130:[6->11,6->17,7->12,8->13] | 0.40 | 660.195652174 | 24 | 46 | 0 | 0 |
| Path 224 | C00117->C00130:[10->8,12->3,6->10,6->11,6->17,6->2,6->4,7->12,8->13] | 0.90 | 617.548387097 | 37 | 62 | 0 | 1 |
| Path 225 | C00117->C00130:[10->8,12->3,6->10,6->11,6->17,6->4,7->12,8->13] | 0.80 | 608.216216216 | 27 | 37 | 0 | 0 |
| Path 226 | C00117->C00130:[6->10,6->11] | 0.20 | 607.04 | 17 | 25 | 0 | 0 |
| Path 227 | C00117->C00130:[10->8,12->3,6->10,6->11,6->17,6->2,6->4,7->12,8->13] | 0.90 | 587.101449275 | 39 | 69 | 0 | 1 |
| Path 228 | C00117->C00130:[6->11] | 0.10 | 577.956521739 | 13 | 23 | 0 | 0 |
| Path 229 | C00117->C00130:[6->10,6->11] | 0.20 | 603.566037736 | 27 | 53 | 0 | 0 |
| Path 230 | C00117->C00130:[6->10,6->11,8->10,8->11] | 0.20 | 553.194805195 | 31 | 77 | 0 | 0 |
| Path 231 | C00117->C00130:[6->10,6->11] | 0.20 | 588.133333333 | 19 | 30 | 0 | 0 |
| Path 232 | C00117->C00130:[6->10] | 0.10 | 624.12 | 17 | 25 | 0 | 0 |
| Path 233 | C00117->C00130:[10->8,12->3,6->11,6->17,7->12,8->13] | 0.60 | 541.518518519 | 16 | 27 | 0 | 0 |
| Path 234 | C00117->C00130:[10->8,12->3,6->10,6->11,6->17,6->2,6->4,7->12,8->13] | 0.90 | 599.127659574 | 32 | 47 | 0 | 1 |
| Path 235 | C00117->C00130:[6->11] | 0.10 | 616.282051282 | 17 | 39 | 0 | 0 |
| Path 236 | C00117->C00130:[10->8,12->3,6->10,6->11,6->17,6->2,6->4,7->12,8->13] | 0.90 | 576.224137931 | 38 | 58 | 0 | 1 |
| Path 237 | C00117->C00130:[6->11,6->17,7->12,8->13] | 0.40 | 692.0 | 25 | 34 | 0 | 0 |
| Path 238 | C00117->C00130:[6->11,6->17,7->12,8->13] | 0.40 | 635.454545455 | 22 | 33 | 0 | 0 |
| Path 239 | C00117->C00130:[10->8,12->3,6->10,6->11,6->17,6->2,6->4,7->12,8->13] | 0.90 | 563.657534247 | 40 | 73 | 0 | 1 |
| Path 240 | C00117->C00130:[6->11] | 0.10 | 576.291666667 | 13 | 24 | 0 | 0 |
| Path 241 | C00117->C00130:[10->8,12->3,6->10,6->11,6->17,6->2,6->4,7->12,8->13] | 0.90 | 543.666666667 | 41 | 84 | 0 | 1 |
| Path 242 | C00117->C00130:[10->8,12->3,6->10,6->11,6->17,6->2,6->4,7->12,8->13] | 0.90 | 594.636363636 | 31 | 44 | 0 | 1 |
| Path 243 | C00117->C00130:[6->10,6->11,8->10,8->11] | 0.20 | 543.478873239 | 27 | 71 | 0 | 0 |
| Path 244 | C00117->C00130:[6->10,6->11] | 0.20 | 537.5 | 23 | 38 | 0 | 0 |
| Path 245 | C00117->C00130:[6->11,6->17,7->12,8->13] | 0.40 | 673.121212121 | 23 | 33 | 0 | 0 |
| Path 246 | C00117->C00130:[10->8,12->3,6->10,6->11,6->17,6->2,6->4,7->12,8->13] | 0.90 | 598.44 | 35 | 50 | 0 | 1 |
| Path 247 | C00117->C00130:[10->8,12->3,6->11,6->17,7->12,8->13] | 0.60 | 480.942857143 | 17 | 35 | 0 | 0 |
| Path 248 | C00117->C00130:[10->8,12->3,6->10,6->11,6->17,6->2,6->4,7->12,8->13] | 0.90 | 596.48 | 35 | 50 | 0 | 1 |
| Path 249 | C00117->C00130:[10->8,12->3,6->10,6->11,6->17,6->4,7->12,8->13] | 0.80 | 570.893617021 | 30 | 47 | 0 | 0 |
| Path 250 | C00117->C00130:[10->8,12->3,6->10,6->11,6->17,6->2,6->4,7->12,8->13] | 0.90 | 573.795918367 | 34 | 49 | 0 | 1 |
| Path 251 | C00117->C00130:[6->10,6->11] | 0.20 | 620.923076923 | 18 | 26 | 0 | 0 |
| Path 252 | C00117->C00130:[6->11] | 0.10 | 465.0 | 13 | 24 | 0 | 0 |
| Path 253 | C00117->C00130:[10->8,12->3,6->10,6->11,6->17,6->2,6->4,7->12,8->13] | 0.90 | 578.326086957 | 32 | 46 | 0 | 1 |
| Path 254 | C00117->C00130:[10->8,12->3,6->10,6->11,6->17,6->4,7->12,8->13] | 0.80 | 562.285714286 | 35 | 56 | 0 | 0 |
| Path 255 | C00117->C00130:[10->8,12->3,6->10,6->11,6->17,6->4,7->12,8->13] | 0.80 | 551.642857143 | 37 | 84 | 0 | 0 |
| Path 256 | C00117->C00130:[10->8,12->3,6->10,6->11,6->17,6->2,6->4,7->12,8->13] | 0.90 | 607.25 | 29 | 40 | 0 | 1 |
| Path 257 | C00117->C00130:[10->8,12->3,6->10,6->11,6->17,6->4,7->12,8->13] | 0.80 | 625.151515152 | 25 | 33 | 0 | 0 |
| Path 258 | C00117->C00130:[10->8,12->3,6->11,6->17,7->12,8->13] | 0.60 | 514.153846154 | 26 | 65 | 0 | 0 |
| Path 259 | C00117->C00130:[10->8,12->3,6->10,6->11,6->17,6->2,6->4,7->12,8->13] | 0.90 | 536.706896552 | 35 | 58 | 0 | 1 |
| Path 260 | C00117->C00130:[10->8,12->3,6->10,6->11,6->17,6->2,6->4,7->12,8->13] | 0.90 | 535.017241379 | 35 | 58 | 0 | 1 |
| Path 261 | C00117->C00130:[10->8,12->3,6->10,6->11,6->17,6->4,7->12,8->13] | 0.80 | 619.659574468 | 33 | 47 | 0 | 0 |
| Path 262 | C00117->C00130:[6->11,6->17,7->12,8->13] | 0.40 | 685.129032258 | 22 | 31 | 0 | 0 |
| Path 263 | C00117->C00130:[10->8,12->3,6->10,6->11,6->17,6->4,7->12,8->13] | 0.80 | 582.842105263 | 27 | 38 | 0 | 0 |
| Path 264 | C00117->C00130:[6->10,6->11] | 0.20 | 612.035714286 | 21 | 28 | 0 | 0 |
| Path 265 | C00117->C00130:[6->10,6->11] | 0.20 | 596.557692308 | 26 | 52 | 0 | 0 |
| Path 266 | C00117->C00130:[10->8,12->3,6->10,6->11,6->17,7->12,8->13] | 0.70 | 547.130434783 | 17 | 23 | 0 | 0 |
| Path 267 | C00117->C00130:[10->8,12->3,6->10,6->11,6->17,6->4,7->12,8->13] | 0.80 | 511.333333333 | 37 | 84 | 0 | 0 |
| Path 268 | C00117->C00130:[10->8,12->3,6->10,6->11,6->17,6->2,6->4,7->12,8->13] | 0.90 | 610.644444444 | 33 | 45 | 0 | 1 |
| Path 269 | C00117->C00130:[10->8,12->3,6->10,6->11,6->17,6->4,7->12,8->13] | 0.80 | 541.065789474 | 36 | 76 | 0 | 0 |
| Path 270 | C00117->C00130:[10->8,12->3,6->10,6->11,6->17,6->2,6->4,7->12,8->13] | 0.90 | 605.470588235 | 35 | 51 | 0 | 1 |
| Path 271 | C00117->C00130:[6->10,6->11] | 0.20 | 546.391304348 | 26 | 46 | 0 | 0 |
| Path 272 | C00117->C00130:[10->8,12->3,6->11,6->17,7->12,8->13] | 0.60 | 541.68 | 16 | 25 | 0 | 0 |
| Path 273 | C00117->C00130:[10->8,12->3,6->10,6->11,6->17,6->2,6->4,7->12,8->13] | 0.90 | 521.833333333 | 44 | 96 | 0 | 1 |
| Path 274 | C00117->C00130:[6->11] | 0.10 | 473.1 | 13 | 30 | 0 | 0 |
| Path 275 | C00117->C00130:[10->8,12->3,6->10,6->11,6->17,6->4,7->12,8->13] | 0.80 | 657.051282051 | 30 | 39 | 0 | 0 |
| Path 276 | C00117->C00130:[10->8,12->3,6->10,6->11,6->17,6->4,7->12,8->13] | 0.80 | 614.0 | 29 | 41 | 0 | 0 |
| Path 277 | C00117->C00130:[10->8,12->3,6->11,6->17,7->12,8->13] | 0.60 | 548.785714286 | 17 | 28 | 0 | 0 |
| Path 278 | C00117->C00130:[10->8,12->3,6->10,6->11,6->17,6->2,6->4,7->12,8->13] | 0.90 | 567.719298246 | 36 | 57 | 0 | 1 |
| Path 279 | C00117->C00130:[6->11,6->17,7->12,8->13] | 0.40 | 672.444444444 | 24 | 36 | 0 | 0 |
| Path 280 | C00117->C00130:[10->8,12->3,6->10,6->11,6->17,7->12,8->13] | 0.70 | 562.666666667 | 18 | 27 | 0 | 1 |
| Path 281 | C00117->C00130:[10->8,12->3,6->10,6->11,6->17,6->4,7->12,8->13] | 0.80 | 618.184210526 | 30 | 38 | 0 | 0 |
| Path 282 | C00117->C00130:[10->8,12->3,6->11,6->17,7->12,8->13] | 0.60 | 548.476190476 | 15 | 21 | 0 | 0 |
| Path 283 | C00117->C00130:[6->10] | 0.10 | 515.0 | 17 | 31 | 0 | 0 |
| Path 284 | C00117->C00130:[10->8,12->3,6->10,6->11,6->17,6->2,6->4,7->12,8->13] | 0.90 | 551.056179775 | 41 | 89 | 0 | 1 |
| Path 285 | C00117->C00130:[6->11] | 0.10 | 603.115384615 | 14 | 26 | 0 | 0 |
| Path 286 | C00117->C00130:[6->11,6->17,7->12,8->13] | 0.40 | 652.0 | 23 | 35 | 0 | 0 |
| Path 287 | C00117->C00130:[6->11] | 0.10 | 549.939393939 | 17 | 33 | 0 | 0 |
| Path 288 | C00117->C00130:[6->11,6->17,7->12,8->13] | 0.40 | 657.897959184 | 26 | 49 | 0 | 0 |
| Path 289 | C00117->C00130:[10->8,12->3,6->11,6->17,7->12,8->13] | 0.60 | 598.0 | 16 | 24 | 0 | 0 |
| Path 290 | C00117->C00130:[6->11,6->17,7->12,8->13] | 0.40 | 695.658536585 | 23 | 41 | 0 | 0 |
| Path 291 | C00117->C00130:[6->11] | 0.10 | 624.0 | 14 | 25 | 0 | 0 |
| Path 292 | C00117->C00130:[6->11] | 0.10 | 596.153846154 | 16 | 26 | 0 | 0 |
| Path 293 | C00117->C00130:[6->11] | 0.10 | 568.75 | 15 | 24 | 0 | 0 |
| Path 294 | C00117->C00130:[10->8,12->3,6->10,6->11,6->17,7->12,8->13] | 0.70 | 541.037037037 | 18 | 27 | 0 | 0 |
| Path 295 | C00117->C00130:[10->8,12->3,6->10,6->11,6->17,6->2,6->4,7->12,8->13] | 0.90 | 592.980392157 | 36 | 51 | 0 | 1 |
| Path 296 | C00117->C00130:[6->10,6->11] | 0.20 | 598.148148148 | 18 | 27 | 0 | 0 |
| Path 297 | C00117->C00130:[10->8,12->3,6->10,6->11,6->17,6->2,6->4,7->12,8->13] | 0.90 | 565.159574468 | 44 | 94 | 0 | 1 |
| Path 298 | C00117->C00130:[6->10,6->11,8->10,8->11] | 0.20 | 540.929577465 | 29 | 71 | 0 | 0 |
| Path 299 | C00117->C00130:[6->10,6->11] | 0.20 | 564.071428571 | 18 | 28 | 0 | 0 |
| Path 300 | C00117->C00130:[6->11] | 0.10 | 665.25 | 15 | 32 | 0 | 0 |
| Path 301 | C00117->C00130:[10->8,12->3,6->17,7->12,8->13] | 0.50 | 376.75 | 4 | 4 | 0 | 0 |
| Path 302 | C00117->C00130:[6->11,6->17,7->12,8->13] | 0.40 | 640.5 | 25 | 48 | 0 | 0 |
| Path 303 | C00117->C00130:[10->8,12->3,6->10,6->11,6->17,6->2,6->4,7->12,8->13] | 0.90 | 599.029850746 | 38 | 67 | 0 | 1 |
| Path 304 | C00117->C00130:[6->11] | 0.10 | 581.611111111 | 13 | 18 | 0 | 0 |
| Path 305 | C00117->C00130:[6->10,6->11] | 0.20 | 608.028571429 | 23 | 35 | 0 | 0 |
| Path 306 | C00117->C00130:[10->8,12->3,6->10,6->11,6->17,6->4,7->12,8->13] | 0.80 | 550.835443038 | 35 | 79 | 0 | 0 |
| Path 307 | C00117->C00130:[6->11] | 0.10 | 602.820512821 | 17 | 39 | 0 | 0 |
| Path 308 | C00117->C00130:[10->8,12->3,6->10,6->11,6->17,6->2,6->4,7->12,8->13] | 0.90 | 575.2 | 30 | 45 | 0 | 1 |
| Path 309 | C00117->C00130:[10->8,12->3,6->11,6->17,7->12,8->13] | 0.60 | 591.071428571 | 20 | 42 | 0 | 0 |
| Path 310 | C00117->C00130:[10->8,12->3,6->10,6->11,6->17,6->2,6->4,7->12,8->13] | 0.90 | 561.044444444 | 31 | 45 | 0 | 1 |
| Path 311 | C00117->C00130:[10->8,12->3,6->10,6->11,6->17,6->2,6->4,7->12,8->13] | 0.90 | 527.11 | 43 | 100 | 0 | 1 |
| Path 312 | C00117->C00130:[10->8,12->3,6->10,6->11,6->17,6->2,6->4,7->12,8->13] | 0.90 | 567.076086957 | 44 | 92 | 0 | 1 |
| Path 313 | C00117->C00130:[10->8,12->3,6->10,6->11,6->17,6->2,6->4,7->12,8->13] | 0.90 | 533.78 | 44 | 100 | 0 | 1 |
| Path 314 | C00117->C00130:[10->8,12->3,6->10,6->11,6->17,6->4,7->12,8->13] | 0.80 | 618.613636364 | 31 | 44 | 0 | 0 |
| Path 315 | C00117->C00130:[10->8,12->3,6->10,6->17,6->2,7->12,8->13] | 0.70 | 604.8 | 29 | 40 | 0 | 1 |
| Path 316 | C00117->C00130:[6->10,6->11] | 0.20 | 538.579710145 | 26 | 69 | 0 | 0 |
| Path 317 | C00117->C00130:[10->8,12->3,6->10,6->11,6->17,6->2,6->4,7->12,8->13] | 0.90 | 598.413333333 | 42 | 75 | 0 | 1 |
| Path 318 | C00117->C00130:[10->8,12->3,6->10,6->11,6->17,6->2,6->4,7->12,8->13] | 0.90 | 518.580645161 | 42 | 93 | 0 | 1 |
| Path 319 | C00117->C00130:[10->8,12->3,6->10,6->11,6->17,6->2,6->4,7->12,8->13] | 0.90 | 564.295454545 | 30 | 44 | 0 | 1 |
| Path 320 | C00117->C00130:[10->8,12->3,6->10,6->11,6->17,6->2,6->4,7->12,8->13] | 0.90 | 620.571428571 | 31 | 42 | 0 | 1 |
