# Supplementary material for: 13C-metabolic flux ratio and novel carbon path analyses confirmed that Trichoderma reesei uses primarily the respirative pathway also on the preferred carbon source glucose
Source: BMC Syst Biol. 2009 Oct 29;3:104. doi: 10.1186/1752-0509-3-104 (PMC2776023; doi:10.1186/1752-0509-3-104)
Supplement: Additional file 1 — Pathways discovered in ReTrace carbon path analysis. Graphical and tabular representations of amino acid synthesis pathways discovered in ReTrace carbon path analysis [21]. Self-contained web site: unpack zip archive and open index.html with a web browser. [file 1752-0509-3-104-S1.zip › AF1-treesei/pathways-C00049-to-C00188.html]

Pathways from C00049 to C00188


**Pathways from C00049 to C00188**

**Sources:** L-Aspartate; (C00049)

**Target:**L-Threonine; (C00188)

|  | Composite mapping | Z | Average score | Rpairs | Reactions | Zero scores | Scores under threshold |
| --- | --- | --- | --- | --- | --- | --- | --- |
| Path 1 | C00049->C00188:[1->1,2->2,3->3,5->5] | 1.00 | 399.166666667 | 5 | 6 | 0 | 0 |
| Path 2 | C00049->C00188:[2->5,5->2,5->3] | 0.75 | 261.023809524 | 19 | 42 | 1 | 2 |
| Path 3 | C00049->C00188:[2->5,5->2,5->3] | 0.75 | 350.756410256 | 25 | 78 | 1 | 2 |
| Path 4 | C00049->C00188:[2->5,5->2,5->3] | 0.75 | 378.977777778 | 23 | 45 | 1 | 2 |
| Path 5 | C00049->C00188:[1->5,3->2,5->3] | 0.75 | 309.180555556 | 23 | 72 | 1 | 2 |
| Path 6 | C00049->C00188:[5->3] | 0.25 | 281.873015873 | 14 | 63 | 1 | 2 |
| Path 7 | C00049->C00188:[5->3] | 0.25 | 248.433333333 | 12 | 30 | 1 | 2 |
| Path 8 | C00049->C00188:[5->2,5->3] | 0.50 | 308.6 | 20 | 40 | 1 | 2 |
| Path 9 | C00049->C00188:[1->5,3->2,5->3] | 0.75 | 306.564102564 | 21 | 39 | 1 | 2 |
| Path 10 | C00049->C00188:[1->2,2->5,5->2,5->3] | 0.75 | 293.647058824 | 24 | 85 | 1 | 2 |
| Path 11 | C00049->C00188:[1->2,2->5,5->3] | 0.75 | 287.931818182 | 19 | 44 | 1 | 2 |
| Path 12 | C00049->C00188:[1->2,2->5,5->2,5->3] | 0.75 | 281.826923077 | 22 | 52 | 1 | 2 |
| Path 13 | C00049->C00188:[2->5,5->2,5->3] | 0.75 | 283.573333333 | 21 | 75 | 1 | 2 |
| Path 14 | C00049->C00188:[2->5,5->2,5->3] | 0.75 | 338.681818182 | 21 | 44 | 1 | 2 |
| Path 15 | C00049->C00188:[2->5,5->2,5->3] | 0.75 | 320.493975904 | 24 | 83 | 1 | 2 |
| Path 16 | C00049->C00188:[1->2,2->5,5->3] | 0.75 | 298.363636364 | 21 | 77 | 1 | 2 |
| Path 17 | C00049->C00188:[2->5,5->2,5->3] | 0.75 | 283.697368421 | 22 | 76 | 1 | 2 |
| Path 18 | C00049->C00188:[2->5,5->2,5->3] | 0.75 | 261.76744186 | 20 | 43 | 1 | 2 |
| Path 19 | C00049->C00188:[5->2,5->3] | 0.50 | 304.341772152 | 23 | 79 | 1 | 2 |
