# Supplementary material for: 13C-metabolic flux ratio and novel carbon path analyses confirmed that Trichoderma reesei uses primarily the respirative pathway also on the preferred carbon source glucose
Source: BMC Syst Biol. 2009 Oct 29;3:104. doi: 10.1186/1752-0509-3-104 (PMC2776023; doi:10.1186/1752-0509-3-104)
Supplement: Additional file 1 — Pathways discovered in ReTrace carbon path analysis. Graphical and tabular representations of amino acid synthesis pathways discovered in ReTrace carbon path analysis [21]. Self-contained web site: unpack zip archive and open index.html with a web browser. [file 1752-0509-3-104-S1.zip › AF1-treesei/pathways-C00031-to-C00073.html]

Pathways from C00031 to C00073


**Pathways from C00031 to C00073**

**Sources:** D-Glucose; (C00031)

**Target:**L-Methionine; (C00073)

|  | Composite mapping | Z | Average score | Rpairs | Reactions | Zero scores | Scores under threshold |
| --- | --- | --- | --- | --- | --- | --- | --- |
| Path 1 | C00031->C00073:[7->1,7->2,7->3,7->9,9->7] | 1.00 | 365.0 | 35 | 113 | 0 | 0 |
| Path 2 | C00031->C00073:[4->1,4->2,7->3,7->9,9->7] | 1.00 | 376.9921875 | 40 | 128 | 0 | 0 |
| Path 3 | C00031->C00073:[4->2,4->7,7->3,7->9,9->1] | 1.00 | 380.913043478 | 37 | 115 | 0 | 0 |
| Path 4 | C00031->C00073:[4->1,4->2,7->3,7->9,9->7] | 1.00 | 370.603448276 | 33 | 116 | 0 | 0 |
| Path 5 | C00031->C00073:[4->2,4->7,7->3,7->9,9->1] | 1.00 | 378.770491803 | 40 | 122 | 0 | 0 |
| Path 6 | C00031->C00073:[4->1,4->2,7->3,7->9,9->7] | 1.00 | 374.504 | 39 | 125 | 0 | 0 |
| Path 7 | C00031->C00073:[4->1,4->2,7->3,7->9,9->7] | 1.00 | 339.889830508 | 39 | 118 | 0 | 0 |
| Path 8 | C00031->C00073:[7->1,7->2,7->3,7->9,9->7] | 1.00 | 337.150442478 | 35 | 113 | 0 | 0 |
| Path 9 | C00031->C00073:[4->2,4->7,7->3,7->9,9->1] | 1.00 | 384.56779661 | 39 | 118 | 0 | 0 |
| Path 10 | C00031->C00073:[4->1,4->2,7->3,7->9,9->7] | 1.00 | 332.128205128 | 38 | 117 | 0 | 0 |
| Path 11 | C00031->C00073:[4->2,4->7,7->3,7->9,9->1] | 1.00 | 381.104347826 | 38 | 115 | 0 | 0 |
| Path 12 | C00031->C00073:[4->1,4->2,7->3,7->9,9->7] | 1.00 | 372.992 | 38 | 125 | 0 | 0 |
| Path 13 | C00031->C00073:[4->2,4->7,7->3,7->9,9->1] | 1.00 | 380.4 | 38 | 120 | 0 | 0 |
| Path 14 | C00031->C00073:[4->1,4->2,7->3,7->9,9->7] | 1.00 | 303.833333333 | 28 | 84 | 0 | 0 |
| Path 15 | C00031->C00073:[4->1,4->2,7->3,7->9,9->7] | 1.00 | 373.358333333 | 38 | 120 | 0 | 0 |
| Path 16 | C00031->C00073:[4->1,4->2,7->3,7->9,9->7] | 1.00 | 339.3125 | 34 | 112 | 0 | 0 |
| Path 17 | C00031->C00073:[4->1,4->2,7->1,7->2,7->3,7->9,9->7] | 1.00 | 314.609195402 | 31 | 87 | 0 | 0 |
| Path 18 | C00031->C00073:[4->1,4->2,7->3,7->9,9->7] | 1.00 | 371.866666667 | 37 | 120 | 0 | 0 |
| Path 19 | C00031->C00073:[7->1,7->2,7->3,7->7,7->9,9->7] | 1.00 | 320.823529412 | 31 | 85 | 0 | 0 |
| Path 20 | C00031->C00073:[4->2,4->7,7->3,7->9,9->1] | 1.00 | 327.174418605 | 31 | 86 | 0 | 0 |
| Path 21 | C00031->C00073:[4->1,4->2,7->3,7->9,9->7] | 1.00 | 373.448 | 38 | 125 | 0 | 0 |
| Path 22 | C00031->C00073:[4->2,4->7,7->3,7->9,9->1] | 1.00 | 384.381355932 | 38 | 118 | 0 | 0 |
| Path 23 | C00031->C00073:[7->1,7->2,7->3,7->9,9->7] | 1.00 | 334.219298246 | 37 | 114 | 0 | 0 |
| Path 24 | C00031->C00073:[4->2,4->7,7->3,7->9,9->1] | 1.00 | 378.37007874 | 41 | 127 | 0 | 0 |
| Path 25 | C00031->C00073:[4->2,4->7,7->3,7->9,9->1] | 1.00 | 366.666666667 | 41 | 129 | 0 | 0 |
| Path 26 | C00031->C00073:[4->2,4->7,7->3,7->9,9->1] | 1.00 | 382.015384615 | 42 | 130 | 0 | 0 |
| Path 27 | C00031->C00073:[4->2,4->7,7->3,7->9,9->1] | 1.00 | 325.294117647 | 30 | 85 | 0 | 0 |
| Path 28 | C00031->C00073:[4->1,4->2,7->1,7->2,7->3,7->9,9->7] | 1.00 | 308.43902439 | 29 | 82 | 0 | 0 |
| Path 29 | C00031->C00073:[7->1,7->2,7->3,7->9,9->7] | 1.00 | 360.172131148 | 38 | 122 | 0 | 0 |
| Path 30 | C00031->C00073:[4->2,4->7,7->3,7->9,9->1] | 1.00 | 354.915966387 | 41 | 119 | 0 | 0 |
| Path 31 | C00031->C00073:[4->1,4->2,7->3,7->9,9->7] | 1.00 | 356.671755725 | 40 | 131 | 0 | 0 |
| Path 32 | C00031->C00073:[4->1,4->2,7->3,7->9,9->7] | 1.00 | 346.95 | 37 | 120 | 0 | 0 |
| Path 33 | C00031->C00073:[4->1,4->2,7->3,7->9,9->7] | 1.00 | 347.816 | 38 | 125 | 0 | 0 |
| Path 34 | C00031->C00073:[4->1,4->2,7->3,7->9,9->7] | 1.00 | 368.016129032 | 39 | 124 | 0 | 0 |
| Path 35 | C00031->C00073:[4->1,4->2,7->3,7->9,9->7] | 1.00 | 307.012048193 | 28 | 83 | 0 | 0 |
| Path 36 | C00031->C00073:[4->1,4->2,4->7,7->3,7->9,9->1] | 1.00 | 330.8 | 31 | 85 | 0 | 0 |
| Path 37 | C00031->C00073:[7->1,7->2,7->3,7->9,9->7] | 1.00 | 348.290322581 | 38 | 124 | 0 | 0 |
| Path 38 | C00031->C00073:[4->1,4->2,4->7,7->3,7->9,9->1] | 1.00 | 342.873563218 | 33 | 87 | 0 | 0 |
| Path 39 | C00031->C00073:[4->1,4->2,7->3,7->9,9->7] | 1.00 | 362.363636364 | 37 | 121 | 0 | 0 |
| Path 40 | C00031->C00073:[7->1,7->2,7->3,7->9,9->7] | 1.00 | 365.25 | 37 | 120 | 0 | 0 |
| Path 41 | C00031->C00073:[4->1,4->2,4->7,7->3,7->9,9->7] | 1.00 | 369.076923077 | 36 | 117 | 0 | 0 |
| Path 42 | C00031->C00073:[4->1,4->2,4->7,7->3,7->9,9->7] | 1.00 | 328.696629213 | 32 | 89 | 0 | 0 |
| Path 43 | C00031->C00073:[4->1,4->2,7->3,7->9,9->7] | 1.00 | 370.520661157 | 34 | 121 | 0 | 0 |
| Path 44 | C00031->C00073:[4->2,4->7,7->3,7->9,9->1] | 1.00 | 354.731092437 | 40 | 119 | 0 | 0 |
| Path 45 | C00031->C00073:[7->1,7->2,7->3,7->9,9->7] | 1.00 | 364.4 | 39 | 125 | 0 | 0 |
| Path 46 | C00031->C00073:[4->1,4->2,4->7,7->3,7->9,9->1] | 1.00 | 381.975 | 39 | 120 | 0 | 0 |
| Path 47 | C00031->C00073:[4->1,4->2,7->3,7->9,9->7] | 1.00 | 340.564102564 | 35 | 117 | 0 | 0 |
| Path 48 | C00031->C00073:[4->2,4->7,7->3,7->9,9->1] | 1.00 | 384.203252033 | 39 | 123 | 0 | 0 |
| Path 49 | C00031->C00073:[4->2,4->7,7->3,7->9,9->1] | 1.00 | 354.175 | 38 | 120 | 0 | 0 |
| Path 50 | C00031->C00073:[4->2,4->7,7->3,7->9,9->1] | 1.00 | 382.967741935 | 37 | 124 | 0 | 0 |
| Path 51 | C00031->C00073:[4->2,4->7,7->3,7->9,9->1] | 1.00 | 363.079365079 | 40 | 126 | 0 | 0 |
| Path 52 | C00031->C00073:[7->1,7->2,7->3,7->9,9->7] | 1.00 | 368.262295082 | 35 | 122 | 0 | 0 |
| Path 53 | C00031->C00073:[4->1,4->2,7->3,7->9,9->7] | 1.00 | 336.530973451 | 37 | 113 | 0 | 0 |
| Path 54 | C00031->C00073:[4->1,4->2,7->3,7->9,9->7] | 1.00 | 367.410714286 | 34 | 112 | 0 | 0 |
| Path 55 | C00031->C00073:[7->1,7->2,7->3,7->9,9->7] | 1.00 | 368.247863248 | 34 | 117 | 0 | 0 |
| Path 56 | C00031->C00073:[4->2,4->7,7->3,7->9,9->1] | 1.00 | 386.860655738 | 37 | 122 | 0 | 0 |
| Path 57 | C00031->C00073:[4->1,4->2,7->3,7->9,9->7] | 1.00 | 350.349593496 | 37 | 123 | 0 | 0 |
| Path 58 | C00031->C00073:[4->1,4->2,7->3,7->9,9->7] | 1.00 | 339.92 | 41 | 125 | 0 | 0 |
| Path 59 | C00031->C00073:[4->2,4->7,7->3,7->9,9->1] | 1.00 | 387.547619048 | 41 | 126 | 0 | 0 |
| Path 60 | C00031->C00073:[4->2,4->7,7->2,7->3,7->7,7->9,9->1] | 1.00 | 335.215909091 | 33 | 88 | 0 | 0 |
| Path 61 | C00031->C00073:[4->1,4->2,7->3,7->9,9->7] | 1.00 | 334.847826087 | 33 | 92 | 0 | 0 |
| Path 62 | C00031->C00073:[4->1,4->2,4->7,7->3,7->9,9->7] | 1.00 | 310.524390244 | 28 | 82 | 0 | 0 |
| Path 63 | C00031->C00073:[4->1,4->2,7->3,7->9,9->7] | 1.00 | 368.03875969 | 40 | 129 | 0 | 0 |
| Path 64 | C00031->C00073:[4->1,4->2,7->3,7->9,9->7] | 1.00 | 343.647540984 | 37 | 122 | 0 | 0 |
