# Supplementary material for: 13C-metabolic flux ratio and novel carbon path analyses confirmed that Trichoderma reesei uses primarily the respirative pathway also on the preferred carbon source glucose
Source: BMC Syst Biol. 2009 Oct 29;3:104. doi: 10.1186/1752-0509-3-104 (PMC2776023; doi:10.1186/1752-0509-3-104)
Supplement: Additional file 1 — Pathways discovered in ReTrace carbon path analysis. Graphical and tabular representations of amino acid synthesis pathways discovered in ReTrace carbon path analysis [21]. Self-contained web site: unpack zip archive and open index.html with a web browser. [file 1752-0509-3-104-S1.zip › AF1-treesei/pathways-C00036-to-C00188.html]

Pathways from C00036 to C00188


**Pathways from C00036 to C00188**

**Sources:** Oxaloacetate; (C00036)

**Target:**L-Threonine; (C00188)

|  | Composite mapping | Z | Average score | Rpairs | Reactions | Zero scores | Scores under threshold |
| --- | --- | --- | --- | --- | --- | --- | --- |
| Path 1 | C00036->C00188:[1->1,2->5,3->2,3->3] | 1.00 | 527.227272727 | 12 | 44 | 0 | 0 |
| Path 2 | C00036->C00188:[1->1,1->5,3->2,3->3] | 1.00 | 548.894736842 | 13 | 38 | 0 | 0 |
| Path 3 | C00036->C00188:[1->1,2->5,3->3,5->2] | 1.00 | 483.133333333 | 9 | 30 | 0 | 0 |
| Path 4 | C00036->C00188:[1->1,1->2,2->5,3->3] | 1.00 | 466.214285714 | 7 | 14 | 0 | 0 |
| Path 5 | C00036->C00188:[1->1,2->5,3->3,5->2] | 1.00 | 502.8 | 10 | 20 | 0 | 0 |
| Path 6 | C00036->C00188:[2->5,5->1,5->2,5->3] | 1.00 | 312.4 | 12 | 40 | 0 | 1 |
| Path 7 | C00036->C00188:[1->1,1->5,3->2,3->3] | 1.00 | 568.541666667 | 12 | 24 | 0 | 0 |
| Path 8 | C00036->C00188:[1->1,2->5,3->2,3->3] | 1.00 | 645.95 | 12 | 20 | 0 | 0 |
| Path 9 | C00036->C00188:[1->1,1->2,2->5,3->3,5->2] | 1.00 | 357.541666667 | 15 | 48 | 0 | 1 |
| Path 10 | C00036->C00188:[1->1,2->5,3->3,5->2] | 1.00 | 521.763157895 | 12 | 38 | 0 | 0 |
| Path 11 | C00036->C00188:[1->1,2->5,3->2,3->3] | 1.00 | 597.105263158 | 14 | 38 | 0 | 0 |
| Path 12 | C00036->C00188:[1->1,1->2,2->5,3->3,5->2] | 1.00 | 396.648148148 | 18 | 54 | 0 | 1 |
| Path 13 | C00036->C00188:[2->1,2->5,5->2,5->3] | 1.00 | 425.892857143 | 12 | 28 | 0 | 0 |
| Path 14 | C00036->C00188:[2->1,2->5,5->2,5->3] | 1.00 | 328.88 | 9 | 25 | 0 | 0 |
| Path 15 | C00036->C00188:[1->1,1->2,2->5,3->3] | 1.00 | 509.3125 | 9 | 32 | 0 | 0 |
| Path 16 | C00036->C00188:[1->1,2->5,3->3,5->2] | 1.00 | 455.0625 | 8 | 16 | 0 | 0 |
| Path 17 | C00036->C00188:[1->1,2->5,3->2,3->3] | 1.00 | 516.423076923 | 10 | 26 | 0 | 0 |
| Path 18 | C00036->C00188:[2->1,2->5,5->2,5->3] | 1.00 | 358.346153846 | 10 | 26 | 0 | 0 |
| Path 19 | C00036->C00188:[2->5,3->2,3->3,5->2,5->3] | 0.75 | 405.428571429 | 16 | 84 | 0 | 1 |
| Path 20 | C00036->C00188:[2->5,3->2] | 0.50 | 586.40625 | 11 | 32 | 0 | 0 |
| Path 21 | C00036->C00188:[3->2] | 0.25 | 642.428571429 | 9 | 14 | 0 | 0 |
| Path 22 | C00036->C00188:[5->2,5->3] | 0.50 | 653.92 | 14 | 25 | 0 | 1 |
| Path 23 | C00036->C00188:[2->5,3->3,5->2] | 0.75 | 490.130434783 | 12 | 23 | 0 | 1 |
| Path 24 | C00036->C00188:[2->5,3->2] | 0.50 | 641.777777778 | 10 | 18 | 0 | 0 |
| Path 25 | C00036->C00188:[1->1,3->3] | 0.50 | 620.888888889 | 6 | 9 | 0 | 0 |
| Path 26 | C00036->C00188:[2->5,5->2,5->3] | 0.75 | 477.0 | 14 | 51 | 0 | 1 |
| Path 27 | C00036->C00188:[2->5,3->2,3->3,5->2,5->3] | 0.75 | 462.540540541 | 13 | 37 | 0 | 1 |
| Path 28 | C00036->C00188:[2->5,5->2,5->3] | 0.75 | 365.652173913 | 10 | 23 | 0 | 1 |
| Path 29 | C00036->C00188:[2->5,3->2] | 0.50 | 502.5 | 8 | 24 | 0 | 0 |
| Path 30 | C00036->C00188:[2->5,5->2,5->3] | 0.75 | 529.311111111 | 16 | 45 | 0 | 1 |
| Path 31 | C00036->C00188:[1->5,3->2,3->3] | 0.75 | 507.563636364 | 16 | 55 | 0 | 1 |
| Path 32 | C00036->C00188:[3->2,3->3,5->2,5->3] | 0.50 | 396.283333333 | 16 | 60 | 0 | 1 |
| Path 33 | C00036->C00188:[2->5,3->2] | 0.50 | 519.785714286 | 10 | 42 | 0 | 0 |
| Path 34 | C00036->C00188:[1->2,2->5,3->1,3->3,5->1,5->3] | 1.00 | 383.255813953 | 14 | 43 | 0 | 1 |
| Path 35 | C00036->C00188:[1->1,1->2,2->5,3->3,5->2] | 1.00 | 440.636363636 | 16 | 44 | 0 | 1 |
| Path 36 | C00036->C00188:[1->2,2->5,3->3] | 0.75 | 476.653061224 | 12 | 49 | 0 | 1 |
| Path 37 | C00036->C00188:[2->5,3->2,3->3] | 0.75 | 604.679245283 | 17 | 53 | 0 | 1 |
| Path 38 | C00036->C00188:[3->3] | 0.25 | 496.475 | 10 | 40 | 0 | 1 |
| Path 39 | C00036->C00188:[2->5,3->2,3->3,5->2,5->3] | 0.75 | 601.836734694 | 16 | 49 | 0 | 1 |
| Path 40 | C00036->C00188:[2->5,5->2,5->3] | 0.75 | 646.333333333 | 12 | 21 | 0 | 1 |
| Path 41 | C00036->C00188:[2->1,2->5,5->2] | 0.75 | 478.681818182 | 11 | 22 | 0 | 0 |
| Path 42 | C00036->C00188:[2->5,3->2,3->3] | 0.75 | 383.471428571 | 15 | 70 | 0 | 1 |
| Path 43 | C00036->C00188:[5->2,5->3] | 0.50 | 520.296296296 | 14 | 27 | 0 | 1 |
| Path 44 | C00036->C00188:[2->5,3->2,3->3,5->2,5->3] | 0.75 | 750.655172414 | 17 | 29 | 0 | 1 |
| Path 45 | C00036->C00188:[1->2,2->5,3->1,3->3,5->1,5->3] | 1.00 | 430.344262295 | 16 | 61 | 0 | 1 |
| Path 46 | C00036->C00188:[2->5,5->1,5->2] | 0.75 | 348.833333333 | 10 | 30 | 0 | 1 |
| Path 47 | C00036->C00188:[3->3] | 0.25 | 486.384615385 | 9 | 26 | 0 | 1 |
| Path 48 | C00036->C00188:[2->5,5->2,5->3] | 0.75 | 501.58974359 | 13 | 39 | 0 | 1 |
| Path 49 | C00036->C00188:[5->3] | 0.25 | 419.8125 | 8 | 16 | 0 | 1 |
| Path 50 | C00036->C00188:[2->5,5->2] | 0.50 | 482.722222222 | 8 | 18 | 0 | 0 |
| Path 51 | C00036->C00188:[2->5,5->1,5->2,5->3] | 1.00 | 329.416666667 | 11 | 36 | 0 | 1 |
| Path 52 | C00036->C00188:[1->2,2->5,5->1,5->3] | 1.00 | 431.675675676 | 16 | 37 | 0 | 1 |
| Path 53 | C00036->C00188:[3->3] | 0.25 | 598.75 | 10 | 16 | 0 | 1 |
| Path 54 | C00036->C00188:[1->1,1->2,2->5,3->3,5->2] | 1.00 | 420.75 | 15 | 40 | 0 | 1 |
| Path 55 | C00036->C00188:[5->1,5->3] | 0.50 | 445.535714286 | 14 | 28 | 0 | 1 |
| Path 56 | C00036->C00188:[1->1,1->2,2->5,3->3,5->2] | 1.00 | 415.64 | 17 | 50 | 0 | 1 |
| Path 57 | C00036->C00188:[1->2,2->5,5->3] | 0.75 | 408.56 | 10 | 25 | 0 | 1 |
| Path 58 | C00036->C00188:[3->2,3->3,5->2,5->3] | 0.50 | 768.44 | 16 | 25 | 0 | 1 |
| Path 59 | C00036->C00188:[2->5,3->2] | 0.50 | 507.184210526 | 9 | 38 | 0 | 0 |
| Path 60 | C00036->C00188:[1->1,2->5,3->2,3->3] | 1.00 | 592.117647059 | 13 | 34 | 0 | 0 |
| Path 61 | C00036->C00188:[2->5,3->2,3->3] | 0.75 | 416.068181818 | 17 | 88 | 0 | 1 |
| Path 62 | C00036->C00188:[5->1,5->3] | 0.50 | 373.545454545 | 11 | 22 | 0 | 1 |
| Path 63 | C00036->C00188:[2->5,5->2,5->3] | 0.75 | 366.75 | 15 | 64 | 0 | 1 |
| Path 64 | C00036->C00188:[3->1,3->3,5->1,5->3] | 0.50 | 408.973684211 | 13 | 38 | 0 | 1 |
| Path 65 | C00036->C00188:[1->2,2->5,5->1,5->3] | 1.00 | 377.903225806 | 13 | 31 | 0 | 1 |
| Path 66 | C00036->C00188:[1->2,2->5,3->3] | 0.75 | 523.08 | 12 | 25 | 0 | 1 |
| Path 67 | C00036->C00188:[1->1,2->5,3->3,5->2] | 1.00 | 507.911764706 | 11 | 34 | 0 | 0 |
| Path 68 | C00036->C00188:[1->1,1->2,2->5,3->3,5->2] | 1.00 | 375.568181818 | 14 | 44 | 0 | 1 |
| Path 69 | C00036->C00188:[5->2] | 0.25 | 437.928571429 | 7 | 14 | 0 | 0 |
| Path 70 | C00036->C00188:[2->5,3->2,3->3] | 0.75 | 636.485714286 | 15 | 35 | 0 | 1 |
| Path 71 | C00036->C00188:[2->5,5->2,5->3] | 0.75 | 493.96 | 12 | 25 | 0 | 1 |
| Path 72 | C00036->C00188:[5->1,5->3] | 0.50 | 437.576923077 | 14 | 52 | 0 | 1 |
| Path 73 | C00036->C00188:[2->5,5->2,5->3] | 0.75 | 535.677419355 | 15 | 31 | 0 | 1 |
| Path 74 | C00036->C00188:[2->5,5->2,5->3] | 0.75 | 323.910714286 | 12 | 56 | 0 | 1 |
| Path 75 | C00036->C00188:[2->5,5->2] | 0.50 | 496.9375 | 9 | 32 | 0 | 0 |
| Path 76 | C00036->C00188:[2->5,3->2,3->3,5->2,5->3] | 0.75 | 488.818181818 | 15 | 55 | 0 | 1 |
| Path 77 | C00036->C00188:[2->5,3->2,3->3] | 0.75 | 507.096774194 | 12 | 31 | 0 | 1 |
| Path 78 | C00036->C00188:[2->5,3->3,5->2] | 0.75 | 458.553191489 | 12 | 47 | 0 | 1 |
| Path 79 | C00036->C00188:[1->1,3->3] | 0.50 | 556.565217391 | 7 | 23 | 0 | 0 |
| Path 80 | C00036->C00188:[2->5,5->2,5->3] | 0.75 | 651.931034483 | 15 | 29 | 0 | 1 |
| Path 81 | C00036->C00188:[2->5,3->3,5->2] | 0.75 | 434.515151515 | 11 | 33 | 0 | 1 |
| Path 82 | C00036->C00188:[2->5,5->2] | 0.50 | 335.6 | 5 | 10 | 0 | 0 |
| Path 83 | C00036->C00188:[1->1,2->5,3->2,3->3] | 1.00 | 516.0 | 11 | 40 | 0 | 0 |
| Path 84 | C00036->C00188:[2->5,3->2,3->3] | 0.75 | 608.48 | 14 | 25 | 0 | 1 |
| Path 85 | C00036->C00188:[1->5,3->2,3->3] | 0.75 | 504.951219512 | 15 | 41 | 0 | 1 |
| Path 86 | C00036->C00188:[2->5,3->2,3->3,5->2,5->3] | 0.75 | 411.484375 | 17 | 64 | 0 | 1 |
| Path 87 | C00036->C00188:[1->2,2->5,3->3] | 0.75 | 438.225806452 | 10 | 31 | 0 | 1 |
| Path 88 | C00036->C00188:[2->5,3->2,3->3] | 0.75 | 540.872727273 | 17 | 55 | 0 | 1 |
| Path 89 | C00036->C00188:[2->5,3->2,3->3] | 0.75 | 520.224489796 | 14 | 49 | 0 | 1 |
| Path 90 | C00036->C00188:[5->2,5->3] | 0.50 | 466.238095238 | 11 | 21 | 0 | 1 |
| Path 91 | C00036->C00188:[5->2,5->3] | 0.50 | 348.566666667 | 14 | 60 | 0 | 1 |
| Path 92 | C00036->C00188:[3->2,3->3] | 0.50 | 602.571428571 | 13 | 21 | 0 | 1 |
| Path 93 | C00036->C00188:[1->5,3->2,5->3] | 0.75 | 476.580645161 | 14 | 31 | 0 | 1 |
| Path 94 | C00036->C00188:[2->5,3->2,3->3] | 0.75 | 539.918918919 | 15 | 37 | 0 | 1 |
| Path 95 | C00036->C00188:[2->5,3->2,3->3] | 0.75 | 509.622222222 | 13 | 45 | 0 | 1 |
| Path 96 | C00036->C00188:[1->1,1->2,2->5,3->3,5->2,5->3] | 1.00 | 349.175 | 13 | 40 | 0 | 1 |
| Path 97 | C00036->C00188:[2->5,3->2,3->3] | 0.75 | 533.137254902 | 16 | 51 | 0 | 1 |
