# Supplementary material for: 13C-metabolic flux ratio and novel carbon path analyses confirmed that Trichoderma reesei uses primarily the respirative pathway also on the preferred carbon source glucose
Source: BMC Syst Biol. 2009 Oct 29;3:104. doi: 10.1186/1752-0509-3-104 (PMC2776023; doi:10.1186/1752-0509-3-104)
Supplement: Additional file 1 — Pathways discovered in ReTrace carbon path analysis. Graphical and tabular representations of amino acid synthesis pathways discovered in ReTrace carbon path analysis [21]. Self-contained web site: unpack zip archive and open index.html with a web browser. [file 1752-0509-3-104-S1.zip › AF1-treesei/pathways-C00026-to-C00025.html]

Pathways from C00026 to C00025


**Pathways from C00026 to C00025**

**Sources:** 2-Oxoglutarate; (C00026)

**Target:**L-Glutamate; (C00025)

|  | Composite mapping | Z | Average score | Rpairs | Reactions | Zero scores | Scores under threshold |
| --- | --- | --- | --- | --- | --- | --- | --- |
| Path 1 | C00026->C00025:[1->1,2->2,3->3,5->5,8->8] | 1.00 | 226.4375 | 16 | 240 | 0 | 0 |
| Path 2 | C00026->C00025:[1->1,2->2,3->3,5->5,8->8] | 1.00 | 225.404958678 | 17 | 242 | 0 | 0 |
| Path 3 | C00026->C00025:[1->1,2->2,3->3,5->5,8->8] | 1.00 | 426.25 | 2 | 4 | 0 | 0 |
| Path 4 | C00026->C00025:[1->1,2->2,3->3,5->5,8->8] | 1.00 | 222.80334728 | 15 | 239 | 0 | 0 |
| Path 5 | C00026->C00025:[1->1,2->2,3->3,5->5,8->8] | 1.00 | 248.125874126 | 8 | 143 | 0 | 0 |
| Path 6 | C00026->C00025:[1->1,2->2,3->3,5->5,8->8] | 1.00 | 248.465838509 | 10 | 161 | 0 | 0 |
| Path 7 | C00026->C00025:[1->1,2->2,3->3,5->5,8->8] | 1.00 | 370.649484536 | 9 | 97 | 0 | 0 |
| Path 8 | C00026->C00025:[1->1,2->2,3->3,5->5,8->8] | 1.00 | 222.80334728 | 15 | 239 | 0 | 0 |
| Path 9 | C00026->C00025:[1->1,2->2,3->3,5->5,8->8] | 1.00 | 346.652173913 | 1 | 69 | 0 | 0 |
| Path 10 | C00026->C00025:[1->1,2->2,3->3,5->5,8->8] | 1.00 | 207.854077253 | 17 | 233 | 0 | 2 |
| Path 11 | C00026->C00025:[1->1,2->2,3->3,5->5,8->8] | 1.00 | 204.030172414 | 16 | 232 | 0 | 2 |
| Path 12 | C00026->C00025:[1->1,2->2,3->3,5->5,8->8] | 1.00 | 209.298165138 | 11 | 218 | 0 | 0 |
| Path 13 | C00026->C00025:[1->1,2->2,3->3,5->5,8->8] | 1.00 | 210.152073733 | 10 | 217 | 0 | 0 |
| Path 14 | C00026->C00025:[1->1,2->2,3->3,5->5,8->8] | 1.00 | 213.238738739 | 15 | 222 | 0 | 2 |
| Path 15 | C00026->C00025:[1->1,2->2,3->3,5->5,8->8] | 1.00 | 269.438596491 | 11 | 171 | 0 | 0 |
| Path 16 | C00026->C00025:[1->1,2->2,3->3,5->5,8->8] | 1.00 | 212.390134529 | 16 | 223 | 0 | 2 |
| Path 17 | C00026->C00025:[1->1,2->2,3->3,5->5,8->8] | 1.00 | 217.461187215 | 12 | 219 | 0 | 0 |
| Path 18 | C00026->C00025:[1->1,2->2,3->3,5->5,8->8] | 1.00 | 215.335849057 | 18 | 265 | 0 | 2 |
| Path 19 | C00026->C00025:[1->1,2->2,3->3,5->5,8->8] | 1.00 | 208.414414414 | 15 | 222 | 0 | 2 |
| Path 20 | C00026->C00025:[1->1,2->2,3->3,5->5,8->8] | 1.00 | 216.411764706 | 13 | 221 | 0 | 0 |
| Path 21 | C00026->C00025:[1->1,2->2,3->3,5->5,8->8] | 1.00 | 212.570776256 | 12 | 219 | 0 | 0 |
| Path 22 | C00026->C00025:[1->1,2->2,3->3,5->5,8->8] | 1.00 | 212.5 | 13 | 242 | 0 | 0 |
| Path 23 | C00026->C00025:[1->1,2->2,3->3,5->5,8->8] | 1.00 | 219.848739496 | 14 | 238 | 0 | 0 |
| Path 24 | C00026->C00025:[1->1,2->2,3->3,5->5,8->8] | 1.00 | 214.211009174 | 11 | 218 | 0 | 0 |
| Path 25 | C00026->C00025:[1->1,2->2,3->3,5->5,8->8] | 1.00 | 219.848739496 | 14 | 238 | 0 | 0 |
| Path 26 | C00026->C00025:[1->1,2->2,3->3,5->5,8->8] | 1.00 | 275.988571429 | 14 | 175 | 0 | 0 |
| Path 27 | C00026->C00025:[1->1,2->2,3->3,5->5,8->8] | 1.00 | 209.373443983 | 12 | 241 | 0 | 0 |
| Path 28 | C00026->C00025:[1->1,2->2,3->3,5->5,8->8] | 1.00 | 196.770642202 | 12 | 218 | 0 | 0 |
| Path 29 | C00026->C00025:[1->1,2->2,3->3,5->5,8->8] | 1.00 | 220.369369369 | 14 | 222 | 0 | 0 |
| Path 30 | C00026->C00025:[1->1,2->2,3->3,5->5,8->8] | 1.00 | 209.248868778 | 14 | 221 | 0 | 2 |
| Path 31 | C00026->C00025:[1->1,2->2,3->3,5->5,8->8] | 1.00 | 212.5 | 13 | 242 | 0 | 0 |
| Path 32 | C00026->C00025:[1->1,2->2,3->3,5->5,8->8] | 1.00 | 209.373443983 | 12 | 241 | 0 | 0 |
| Path 33 | C00026->C00025:[1->1,2->2,3->3,5->5,8->8] | 1.00 | 219.029288703 | 15 | 239 | 0 | 0 |
| Path 34 | C00026->C00025:[1->1,2->2,3->3,5->5,8->8] | 1.00 | 273.255813953 | 12 | 172 | 0 | 0 |
| Path 35 | C00026->C00025:[1->1,2->2,3->3,5->5,8->8] | 1.00 | 213.435779817 | 11 | 218 | 0 | 0 |
| Path 36 | C00026->C00025:[1->1,2->2,3->3,5->5,8->8] | 1.00 | 223.510460251 | 15 | 239 | 0 | 0 |
