# Supplementary material for: 13C-metabolic flux ratio and novel carbon path analyses confirmed that Trichoderma reesei uses primarily the respirative pathway also on the preferred carbon source glucose
Source: BMC Syst Biol. 2009 Oct 29;3:104. doi: 10.1186/1752-0509-3-104 (PMC2776023; doi:10.1186/1752-0509-3-104)
Supplement: Additional file 1 — Pathways discovered in ReTrace carbon path analysis. Graphical and tabular representations of amino acid synthesis pathways discovered in ReTrace carbon path analysis [21]. Self-contained web site: unpack zip archive and open index.html with a web browser. [file 1752-0509-3-104-S1.zip › AF1-treesei/pathways-C00031-to-C00148.html]

Pathways from C00031 to C00148


**Pathways from C00031 to C00148**

**Sources:** D-Glucose; (C00031)

**Target:**L-Proline; (C00148)

|  | Composite mapping | Z | Average score | Rpairs | Reactions | Zero scores | Scores under threshold |
| --- | --- | --- | --- | --- | --- | --- | --- |
| Path 1 | C00031->C00148:[4->2,7->1,7->7,9->3,9->8] | 1.00 | 410.6 | 24 | 135 | 0 | 0 |
| Path 2 | C00031->C00148:[4->2,7->1,7->7,9->3,9->8] | 1.00 | 369.569536424 | 23 | 151 | 0 | 0 |
| Path 3 | C00031->C00148:[7->1,7->2,7->7,9->3,9->8] | 1.00 | 387.891472868 | 19 | 129 | 0 | 0 |
| Path 4 | C00031->C00148:[7->1,7->2,7->7,9->3,9->8] | 1.00 | 383.015503876 | 24 | 129 | 0 | 0 |
| Path 5 | C00031->C00148:[4->2,4->8,7->1,7->7,9->3] | 1.00 | 374.224719101 | 26 | 178 | 0 | 0 |
| Path 6 | C00031->C00148:[7->1,7->2,7->7,9->3,9->8] | 1.00 | 344.350282486 | 30 | 177 | 0 | 0 |
| Path 7 | C00031->C00148:[4->2,4->8,7->1,7->7,9->3] | 1.00 | 395.057553957 | 23 | 139 | 0 | 0 |
| Path 8 | C00031->C00148:[4->2,7->1,7->7,9->3,9->8] | 1.00 | 347.701657459 | 31 | 181 | 0 | 0 |
| Path 9 | C00031->C00148:[1->3,4->1,4->2,7->7,9->8] | 1.00 | 366.78238342 | 27 | 193 | 0 | 0 |
| Path 10 | C00031->C00148:[4->2,4->8,7->1,7->7,9->3] | 1.00 | 407.932330827 | 22 | 133 | 0 | 0 |
| Path 11 | C00031->C00148:[7->1,7->2,7->7,9->3,9->8] | 1.00 | 362.530201342 | 24 | 149 | 0 | 0 |
| Path 12 | C00031->C00148:[4->2,7->1,7->7,9->3,9->8] | 1.00 | 336.036144578 | 23 | 166 | 0 | 0 |
| Path 13 | C00031->C00148:[4->2,7->1,7->7,9->3,9->8] | 1.00 | 441.939130435 | 24 | 115 | 0 | 0 |
| Path 14 | C00031->C00148:[7->1,7->2,7->7,9->3,9->8] | 1.00 | 332.62804878 | 24 | 164 | 0 | 0 |
| Path 15 | C00031->C00148:[4->2,4->8,7->1,7->7,9->3,9->8] | 1.00 | 364.969325153 | 27 | 163 | 0 | 0 |
| Path 16 | C00031->C00148:[4->2,4->8,7->1,7->7,9->3,9->8] | 1.00 | 337.213483146 | 27 | 178 | 0 | 0 |
| Path 17 | C00031->C00148:[4->2,4->8,7->1,7->7,9->3] | 1.00 | 401.934210526 | 27 | 152 | 0 | 0 |
| Path 18 | C00031->C00148:[4->2,4->8,7->1,7->7,9->3] | 1.00 | 373.868421053 | 24 | 152 | 0 | 0 |
| Path 19 | C00031->C00148:[4->1,4->2,7->7,9->3,9->8] | 1.00 | 404.824175824 | 36 | 91 | 0 | 0 |
| Path 20 | C00031->C00148:[7->1,7->2,7->7,9->3,9->8] | 1.00 | 347.475177305 | 19 | 141 | 0 | 0 |
| Path 21 | C00031->C00148:[4->1,4->2,7->7,9->3,9->8] | 1.00 | 444.37037037 | 33 | 108 | 0 | 0 |
| Path 22 | C00031->C00148:[4->2,4->8,7->1,7->7,9->3] | 1.00 | 410.905109489 | 24 | 137 | 0 | 0 |
| Path 23 | C00031->C00148:[7->1,7->2,7->7,9->3,9->8] | 1.00 | 432.357798165 | 21 | 109 | 0 | 0 |
| Path 24 | C00031->C00148:[7->1,7->2,7->7,9->3,9->8] | 1.00 | 439.990990991 | 23 | 111 | 0 | 0 |
| Path 25 | C00031->C00148:[1->3,4->1,4->2,4->8,7->7] | 1.00 | 392.993006993 | 31 | 143 | 0 | 0 |
| Path 26 | C00031->C00148:[4->2,7->1,7->7,9->3,9->8] | 1.00 | 395.835616438 | 31 | 146 | 0 | 0 |
| Path 27 | C00031->C00148:[4->2,7->1,7->7,9->3,9->8] | 1.00 | 391.142857143 | 20 | 133 | 0 | 0 |
| Path 28 | C00031->C00148:[4->2,7->1,7->7,9->3,9->8] | 1.00 | 406.404411765 | 23 | 136 | 0 | 0 |
| Path 29 | C00031->C00148:[4->2,7->1,7->7,9->3,9->8] | 1.00 | 329.695121951 | 21 | 164 | 0 | 0 |
| Path 30 | C00031->C00148:[4->2,4->8,7->1,7->7,9->3,9->8] | 1.00 | 361.327044025 | 25 | 159 | 0 | 0 |
| Path 31 | C00031->C00148:[4->2,4->8,7->1,7->7,9->3] | 1.00 | 371.109195402 | 24 | 174 | 0 | 0 |
| Path 32 | C00031->C00148:[4->1,4->2,7->7,9->3,9->8] | 1.00 | 408.028985507 | 26 | 69 | 0 | 0 |
| Path 33 | C00031->C00148:[4->1,4->2,7->7,9->3,9->8] | 1.00 | 424.383561644 | 29 | 73 | 0 | 0 |
| Path 34 | C00031->C00148:[1->3,4->1,4->2,7->7,9->8] | 1.00 | 369.086734694 | 28 | 196 | 0 | 0 |
| Path 35 | C00031->C00148:[4->2,7->1,7->7,9->3,9->8] | 1.00 | 359.302013423 | 21 | 149 | 0 | 0 |
| Path 36 | C00031->C00148:[4->1,4->2,7->7,9->3,9->8] | 1.00 | 411.556818182 | 32 | 88 | 0 | 0 |
| Path 37 | C00031->C00148:[4->2,4->8,7->1,7->7,9->3] | 1.00 | 391.659259259 | 21 | 135 | 0 | 0 |
| Path 38 | C00031->C00148:[7->1,7->2,7->7,9->3,9->8] | 1.00 | 416.788990826 | 19 | 109 | 0 | 0 |
| Path 39 | C00031->C00148:[4->1,4->2,7->7,9->3,9->8] | 1.00 | 403.060606061 | 25 | 66 | 0 | 0 |
| Path 40 | C00031->C00148:[4->2,7->1,7->7,9->3,9->8] | 1.00 | 386.413533835 | 25 | 133 | 0 | 0 |
| Path 41 | C00031->C00148:[7->1,7->2,7->7,9->3,9->8] | 1.00 | 332.086419753 | 22 | 162 | 0 | 0 |
| Path 42 | C00031->C00148:[4->1,4->2,7->1,7->2,7->7,9->3,9->8] | 1.00 | 412.333333333 | 28 | 69 | 0 | 0 |
| Path 43 | C00031->C00148:[4->2,7->1,7->7,9->3,9->8] | 1.00 | 378.414201183 | 32 | 169 | 0 | 0 |
| Path 44 | C00031->C00148:[4->1,4->2,7->7,9->3,9->8] | 1.00 | 419.083333333 | 28 | 72 | 0 | 0 |
| Path 45 | C00031->C00148:[4->2,7->1,7->7,9->3,9->8] | 1.00 | 375.928104575 | 25 | 153 | 0 | 0 |
| Path 46 | C00031->C00148:[4->2,4->8,7->1,7->7,9->3] | 1.00 | 373.119205298 | 23 | 151 | 0 | 0 |
| Path 47 | C00031->C00148:[4->2,7->1,7->7,9->3,9->8] | 1.00 | 412.920289855 | 25 | 138 | 0 | 0 |
| Path 48 | C00031->C00148:[4->2,7->1,7->7,9->3,9->8] | 1.00 | 368.831168831 | 24 | 154 | 0 | 0 |
| Path 49 | C00031->C00148:[4->2,4->8,7->1,7->7,9->3] | 1.00 | 407.45323741 | 26 | 139 | 0 | 0 |
| Path 50 | C00031->C00148:[1->3,4->1,7->2,7->7,9->8] | 1.00 | 336.090277778 | 23 | 144 | 0 | 0 |
| Path 51 | C00031->C00148:[4->2,7->1,7->7,9->3,9->8] | 1.00 | 339.25443787 | 24 | 169 | 0 | 0 |
| Path 52 | C00031->C00148:[4->2,4->8,7->1,7->7,9->3] | 1.00 | 378.025316456 | 27 | 158 | 0 | 0 |
| Path 53 | C00031->C00148:[1->3,4->1,4->2,7->7,9->8] | 1.00 | 343.927152318 | 25 | 151 | 0 | 0 |
| Path 54 | C00031->C00148:[4->2,7->1,7->7,9->3,9->8] | 1.00 | 362.421052632 | 22 | 152 | 0 | 0 |
| Path 55 | C00031->C00148:[4->2,7->1,7->7,9->3,9->8] | 1.00 | 386.564885496 | 23 | 131 | 0 | 0 |
| Path 56 | C00031->C00148:[7->1,7->2,7->7,9->3,9->8] | 1.00 | 375.552 | 20 | 125 | 0 | 0 |
| Path 57 | C00031->C00148:[4->2,4->8,7->1,7->7,9->3] | 1.00 | 370.195945946 | 22 | 148 | 0 | 0 |
| Path 58 | C00031->C00148:[4->2,7->1,7->7,9->3,9->8] | 1.00 | 366.019607843 | 25 | 153 | 0 | 0 |
| Path 59 | C00031->C00148:[4->1,4->2,7->1,7->2,7->7,9->3,9->8] | 1.00 | 416.708333333 | 29 | 72 | 0 | 0 |
| Path 60 | C00031->C00148:[4->1,4->2,7->7,9->3,9->8] | 1.00 | 428.052631579 | 30 | 76 | 0 | 0 |
