# Supplementary material for: 13C-metabolic flux ratio and novel carbon path analyses confirmed that Trichoderma reesei uses primarily the respirative pathway also on the preferred carbon source glucose
Source: BMC Syst Biol. 2009 Oct 29;3:104. doi: 10.1186/1752-0509-3-104 (PMC2776023; doi:10.1186/1752-0509-3-104)
Supplement: Additional file 1 — Pathways discovered in ReTrace carbon path analysis. Graphical and tabular representations of amino acid synthesis pathways discovered in ReTrace carbon path analysis [21]. Self-contained web site: unpack zip archive and open index.html with a web browser. [file 1752-0509-3-104-S1.zip › AF1-treesei/pathways-C00031-to-C00025.html]

Pathways from C00031 to C00025


**Pathways from C00031 to C00025**

**Sources:** D-Glucose; (C00031)

**Target:**L-Glutamate; (C00025)

|  | Composite mapping | Z | Average score | Rpairs | Reactions | Zero scores | Scores under threshold |
| --- | --- | --- | --- | --- | --- | --- | --- |
| Path 1 | C00031->C00025:[4->3,7->1,7->5,9->2,9->8] | 1.00 | 406.59375 | 18 | 128 | 0 | 0 |
| Path 2 | C00031->C00025:[1->2,4->1,4->3,7->5,9->8] | 1.00 | 353.197183099 | 25 | 142 | 0 | 0 |
| Path 3 | C00031->C00025:[1->2,4->1,4->3,4->8,7->5] | 1.00 | 384.223880597 | 29 | 134 | 0 | 0 |
| Path 4 | C00031->C00025:[7->1,7->3,7->5,9->2,9->8] | 1.00 | 387.101910828 | 26 | 157 | 0 | 0 |
| Path 5 | C00031->C00025:[1->2,4->1,4->3,4->8,7->5] | 1.00 | 394.364963504 | 31 | 137 | 0 | 0 |
| Path 6 | C00031->C00025:[1->2,4->1,4->3,4->8,7->5] | 1.00 | 401.406504065 | 23 | 123 | 0 | 0 |
| Path 7 | C00031->C00025:[1->2,4->1,7->3,7->5,9->8] | 1.00 | 346.142857143 | 22 | 140 | 0 | 0 |
| Path 8 | C00031->C00025:[1->2,4->1,4->3,4->8,7->5] | 1.00 | 407.276119403 | 31 | 134 | 0 | 0 |
| Path 9 | C00031->C00025:[1->2,4->1,4->3,4->8,7->5] | 1.00 | 402.0 | 28 | 130 | 0 | 0 |
| Path 10 | C00031->C00025:[1->2,4->1,4->3,4->8,7->5] | 1.00 | 403.007407407 | 30 | 135 | 0 | 0 |
| Path 11 | C00031->C00025:[1->2,4->1,4->3,4->8,7->5] | 1.00 | 396.787401575 | 28 | 127 | 0 | 0 |
| Path 12 | C00031->C00025:[1->2,4->1,7->3,7->5,9->8] | 1.00 | 345.246478873 | 25 | 142 | 0 | 0 |
| Path 13 | C00031->C00025:[7->1,7->3,7->5,9->2,9->8] | 1.00 | 375.112676056 | 20 | 142 | 0 | 0 |
| Path 14 | C00031->C00025:[1->2,4->1,4->3,4->8,7->5] | 1.00 | 400.62406015 | 27 | 133 | 0 | 0 |
| Path 15 | C00031->C00025:[1->2,4->1,4->3,4->8,7->5] | 1.00 | 398.874074074 | 30 | 135 | 0 | 0 |
| Path 16 | C00031->C00025:[4->3,7->1,7->5,9->2,9->8] | 1.00 | 381.462068966 | 22 | 145 | 0 | 0 |
| Path 17 | C00031->C00025:[4->1,4->3,7->5,9->2,9->8] | 1.00 | 421.605633803 | 28 | 71 | 0 | 0 |
| Path 18 | C00031->C00025:[1->2,4->1,4->3,7->5,9->8] | 1.00 | 345.454545455 | 25 | 143 | 0 | 0 |
| Path 19 | C00031->C00025:[1->2,4->1,4->3,4->8,7->5] | 1.00 | 405.877697842 | 30 | 139 | 0 | 0 |
| Path 20 | C00031->C00025:[7->1,7->3,7->5,9->2,9->8] | 1.00 | 423.571428571 | 21 | 126 | 0 | 0 |
| Path 21 | C00031->C00025:[1->2,4->1,4->3,5->5,9->8] | 1.00 | 378.131782946 | 26 | 129 | 0 | 0 |
| Path 22 | C00031->C00025:[4->3,5->1,7->1,7->5,9->2,9->8] | 1.00 | 381.059602649 | 24 | 151 | 0 | 0 |
| Path 23 | C00031->C00025:[7->1,7->3,7->5,9->2,9->8] | 1.00 | 396.053254438 | 24 | 169 | 0 | 0 |
| Path 24 | C00031->C00025:[4->3,7->1,7->5,9->2,9->8] | 1.00 | 374.825174825 | 20 | 143 | 0 | 0 |
| Path 25 | C00031->C00025:[1->2,4->1,4->3,4->8,7->5] | 1.00 | 413.582733813 | 30 | 139 | 0 | 0 |
| Path 26 | C00031->C00025:[1->2,4->1,4->3,4->8,7->5] | 1.00 | 408.676691729 | 27 | 133 | 0 | 0 |
| Path 27 | C00031->C00025:[4->3,7->1,7->5,9->2,9->8] | 1.00 | 363.65034965 | 18 | 143 | 0 | 0 |
| Path 28 | C00031->C00025:[7->1,7->3,7->5,9->2,9->8] | 1.00 | 409.97810219 | 26 | 137 | 0 | 0 |
| Path 29 | C00031->C00025:[1->2,4->1,4->3,4->8,7->5] | 1.00 | 406.432835821 | 30 | 134 | 0 | 0 |
| Path 30 | C00031->C00025:[4->3,7->1,7->5,9->2,9->8] | 1.00 | 412.418439716 | 27 | 141 | 0 | 0 |
| Path 31 | C00031->C00025:[4->3,7->1,7->5,9->2,9->8] | 1.00 | 427.864661654 | 23 | 133 | 0 | 0 |
| Path 32 | C00031->C00025:[7->1,7->3,7->5,9->2,9->8] | 1.00 | 368.242857143 | 18 | 140 | 0 | 0 |
| Path 33 | C00031->C00025:[4->3,4->8,7->1,7->5,9->2] | 1.00 | 408.646616541 | 20 | 133 | 0 | 0 |
| Path 34 | C00031->C00025:[4->3,7->1,7->5,9->2,9->8] | 1.00 | 439.222222222 | 18 | 108 | 0 | 0 |
| Path 35 | C00031->C00025:[4->3,4->8,7->1,7->5,9->2] | 1.00 | 412.695945946 | 26 | 148 | 0 | 0 |
| Path 36 | C00031->C00025:[1->2,4->1,7->3,7->5,9->8] | 1.00 | 347.679104478 | 20 | 134 | 0 | 0 |
| Path 37 | C00031->C00025:[7->1,7->3,7->5,9->2,9->8] | 1.00 | 355.229166667 | 23 | 144 | 0 | 0 |
| Path 38 | C00031->C00025:[1->2,4->1,4->3,7->5,9->8] | 1.00 | 355.97260274 | 23 | 146 | 0 | 0 |
| Path 39 | C00031->C00025:[1->2,4->1,4->3,7->5,9->8] | 1.00 | 352.587412587 | 22 | 143 | 0 | 0 |
| Path 40 | C00031->C00025:[1->2,4->1,7->3,7->5,9->8] | 1.00 | 348.460431655 | 21 | 139 | 0 | 0 |
| Path 41 | C00031->C00025:[1->2,4->1,4->3,5->5,9->8] | 1.00 | 382.046153846 | 27 | 130 | 0 | 0 |
| Path 42 | C00031->C00025:[4->3,7->1,7->5,9->2,9->8] | 1.00 | 359.044692737 | 28 | 179 | 0 | 0 |
| Path 43 | C00031->C00025:[1->2,4->1,4->3,4->8,7->5] | 1.00 | 392.1875 | 27 | 128 | 0 | 0 |
| Path 44 | C00031->C00025:[1->2,4->1,4->3,5->5,9->8] | 1.00 | 384.251968504 | 25 | 127 | 0 | 0 |
| Path 45 | C00031->C00025:[1->2,4->1,4->3,7->5,9->8] | 1.00 | 348.841726619 | 23 | 139 | 0 | 0 |
| Path 46 | C00031->C00025:[4->3,4->8,7->1,7->5,9->2] | 1.00 | 415.340136054 | 25 | 147 | 0 | 0 |
| Path 47 | C00031->C00025:[4->3,7->1,7->5,9->2,9->8] | 1.00 | 381.22147651 | 22 | 149 | 0 | 0 |
| Path 48 | C00031->C00025:[4->3,7->1,7->5,9->2,9->8] | 1.00 | 364.142857143 | 18 | 140 | 0 | 0 |
| Path 49 | C00031->C00025:[1->2,4->1,4->3,7->5,9->8] | 1.00 | 348.98630137 | 26 | 146 | 0 | 0 |
| Path 50 | C00031->C00025:[1->2,4->1,4->3,4->8,7->5] | 1.00 | 403.639097744 | 30 | 133 | 0 | 0 |
| Path 51 | C00031->C00025:[1->2,4->1,4->3,7->5,9->8] | 1.00 | 352.646258503 | 27 | 147 | 0 | 0 |
| Path 52 | C00031->C00025:[7->1,7->3,7->5,9->2,9->8] | 1.00 | 371.338129496 | 19 | 139 | 0 | 0 |
| Path 53 | C00031->C00025:[7->1,7->3,7->5,9->2,9->8] | 1.00 | 398.836065574 | 20 | 122 | 0 | 0 |
| Path 54 | C00031->C00025:[1->2,4->1,7->3,7->5,9->8] | 1.00 | 352.728571429 | 25 | 140 | 0 | 0 |
| Path 55 | C00031->C00025:[4->3,5->1,5->5,7->1,7->5,9->2,9->8] | 1.00 | 405.75 | 21 | 128 | 0 | 0 |
| Path 56 | C00031->C00025:[1->2,4->1,4->3,4->8,7->5] | 1.00 | 395.488549618 | 28 | 131 | 0 | 0 |
| Path 57 | C00031->C00025:[4->3,7->1,7->5,9->2,9->8] | 1.00 | 366.808219178 | 19 | 146 | 0 | 0 |
| Path 58 | C00031->C00025:[1->2,4->1,4->3,5->5,9->8] | 1.00 | 366.610687023 | 26 | 131 | 0 | 0 |
| Path 59 | C00031->C00025:[1->2,4->1,4->3,4->8,7->5] | 1.00 | 398.403225806 | 26 | 124 | 0 | 0 |
| Path 60 | C00031->C00025:[7->1,7->3,7->5,9->2,9->8] | 1.00 | 391.216666667 | 18 | 120 | 0 | 0 |
| Path 61 | C00031->C00025:[4->3,7->1,7->5,9->2,9->8] | 1.00 | 342.906832298 | 21 | 161 | 0 | 0 |
| Path 62 | C00031->C00025:[4->3,4->8,7->1,7->3,7->5,7->8,9->2] | 1.00 | 410.879699248 | 22 | 133 | 0 | 0 |
| Path 63 | C00031->C00025:[4->3,4->8,7->1,7->3,7->5,7->8,9->2] | 1.00 | 427.694656489 | 23 | 131 | 0 | 0 |
| Path 64 | C00031->C00025:[4->3,4->8,7->1,7->3,7->5,7->8,9->2,9->8] | 1.00 | 380.95 | 27 | 160 | 0 | 0 |
| Path 65 | C00031->C00025:[4->3,4->8,7->1,7->5,9->2] | 1.00 | 423.2734375 | 20 | 128 | 0 | 0 |
| Path 66 | C00031->C00025:[4->3,4->8,7->1,7->5,9->2] | 1.00 | 409.774647887 | 27 | 142 | 0 | 0 |
| Path 67 | C00031->C00025:[4->1,4->3,7->1,7->3,7->5,9->2,9->8] | 1.00 | 429.5 | 31 | 74 | 0 | 0 |
| Path 68 | C00031->C00025:[1->2,4->1,7->3,7->5,9->8] | 1.00 | 330.943262411 | 24 | 141 | 0 | 0 |
| Path 69 | C00031->C00025:[4->3,4->8,7->1,7->5,9->2] | 1.00 | 410.96 | 29 | 150 | 0 | 0 |
| Path 70 | C00031->C00025:[1->2,4->1,4->3,5->5,9->8] | 1.00 | 389.892307692 | 24 | 130 | 0 | 0 |
| Path 71 | C00031->C00025:[1->2,4->1,4->3,7->5,9->8] | 1.00 | 352.758389262 | 27 | 149 | 0 | 0 |
| Path 72 | C00031->C00025:[1->2,4->1,4->3,4->8,7->5] | 1.00 | 400.15625 | 24 | 128 | 0 | 0 |
| Path 73 | C00031->C00025:[4->3,4->8,7->1,7->5,9->2] | 1.00 | 414.244755245 | 27 | 143 | 0 | 0 |
| Path 74 | C00031->C00025:[4->3,4->8,7->1,7->3,7->5,7->8,9->2] | 1.00 | 429.701492537 | 24 | 134 | 0 | 0 |
| Path 75 | C00031->C00025:[1->2,4->1,4->3,4->8,7->5] | 1.00 | 380.361538462 | 27 | 130 | 0 | 0 |
| Path 76 | C00031->C00025:[7->1,7->3,7->5,9->2,9->8] | 1.00 | 465.425373134 | 24 | 134 | 0 | 0 |
| Path 77 | C00031->C00025:[4->3,4->8,7->1,7->5,9->2] | 1.00 | 416.957746479 | 24 | 142 | 0 | 0 |
| Path 78 | C00031->C00025:[1->2,4->1,4->3,4->8,7->5] | 1.00 | 401.0 | 27 | 128 | 0 | 0 |
| Path 79 | C00031->C00025:[1->2,4->1,4->3,7->5,9->8] | 1.00 | 361.0 | 24 | 147 | 0 | 0 |
| Path 80 | C00031->C00025:[1->2,4->1,4->3,4->8,7->5] | 1.00 | 398.579365079 | 26 | 126 | 0 | 0 |
| Path 81 | C00031->C00025:[1->2,4->1,4->3,4->8,7->5] | 1.00 | 410.120300752 | 28 | 133 | 0 | 0 |
| Path 82 | C00031->C00025:[7->1,7->3,7->5,9->2,9->8] | 1.00 | 353.098837209 | 26 | 172 | 0 | 0 |
| Path 83 | C00031->C00025:[4->3,7->1,7->5,9->2,9->8] | 1.00 | 356.346590909 | 27 | 176 | 0 | 0 |
| Path 84 | C00031->C00025:[4->1,4->3,7->5,9->2,9->8] | 1.00 | 422.513888889 | 29 | 72 | 0 | 0 |
| Path 85 | C00031->C00025:[4->3,7->1,7->5,9->2,9->8] | 1.00 | 461.327272727 | 22 | 110 | 0 | 0 |
| Path 86 | C00031->C00025:[4->3,4->8,7->1,7->5,9->2] | 1.00 | 425.427480916 | 21 | 131 | 0 | 0 |
| Path 87 | C00031->C00025:[1->2,4->1,4->3,7->5,9->8] | 1.00 | 335.496551724 | 25 | 145 | 0 | 0 |
| Path 88 | C00031->C00025:[4->3,4->8,7->1,7->5,9->2] | 1.00 | 411.635135135 | 29 | 148 | 0 | 0 |
| Path 89 | C00031->C00025:[4->3,4->8,7->1,7->5,9->2] | 1.00 | 425.893939394 | 22 | 132 | 0 | 0 |
| Path 90 | C00031->C00025:[1->2,4->1,4->3,5->5,9->8] | 1.00 | 386.944444444 | 25 | 126 | 0 | 0 |
| Path 91 | C00031->C00025:[1->2,4->1,4->3,7->5,9->8] | 1.00 | 352.401408451 | 24 | 142 | 0 | 0 |
| Path 92 | C00031->C00025:[7->1,7->3,7->5,9->2,9->8] | 1.00 | 359.841726619 | 17 | 139 | 0 | 0 |
| Path 93 | C00031->C00025:[1->2,4->1,7->3,7->5,9->8] | 1.00 | 341.122302158 | 24 | 139 | 0 | 0 |
| Path 94 | C00031->C00025:[4->3,7->1,7->5,9->2,9->8] | 1.00 | 401.920634921 | 21 | 126 | 0 | 0 |
| Path 95 | C00031->C00025:[4->3,4->8,7->1,7->5,9->2] | 1.00 | 409.231343284 | 21 | 134 | 0 | 0 |
| Path 96 | C00031->C00025:[4->3,7->1,7->5,9->2,9->8] | 1.00 | 454.018518519 | 20 | 108 | 0 | 0 |
| Path 97 | C00031->C00025:[1->2,4->1,4->3,7->5,9->8] | 1.00 | 354.896551724 | 26 | 145 | 0 | 0 |
| Path 98 | C00031->C00025:[4->3,7->1,7->5,9->2,9->8] | 1.00 | 362.178807947 | 25 | 151 | 0 | 0 |
| Path 99 | C00031->C00025:[1->2,4->1,4->3,5->5,9->8] | 1.00 | 381.727272727 | 27 | 132 | 0 | 0 |
| Path 100 | C00031->C00025:[7->1,7->3,7->5,9->2,9->8] | 1.00 | 452.403846154 | 19 | 104 | 0 | 0 |
| Path 101 | C00031->C00025:[7->1,7->3,7->5,9->2,9->8] | 1.00 | 378.212765957 | 21 | 141 | 0 | 0 |
| Path 102 | C00031->C00025:[4->3,7->1,7->5,9->2,9->8] | 1.00 | 377.753424658 | 21 | 146 | 0 | 0 |
| Path 103 | C00031->C00025:[7->1,7->3,7->5,9->2,9->8] | 1.00 | 460.018867925 | 21 | 106 | 0 | 0 |
| Path 104 | C00031->C00025:[4->3,4->8,7->1,7->5,9->2] | 1.00 | 419.932432432 | 26 | 148 | 0 | 0 |
| Path 105 | C00031->C00025:[4->3,4->8,7->1,7->5,9->2] | 1.00 | 413.454545455 | 26 | 143 | 0 | 0 |
| Path 106 | C00031->C00025:[1->2,4->1,7->3,7->5,9->8] | 1.00 | 341.26119403 | 22 | 134 | 0 | 0 |
| Path 107 | C00031->C00025:[1->2,4->1,4->3,7->5,9->8] | 1.00 | 353.714285714 | 24 | 147 | 0 | 0 |
| Path 108 | C00031->C00025:[4->3,7->1,7->5,9->2,9->8] | 1.00 | 374.761904762 | 20 | 147 | 0 | 0 |
| Path 109 | C00031->C00025:[7->1,7->3,7->5,9->2,9->8] | 1.00 | 339.006369427 | 20 | 157 | 0 | 0 |
| Path 110 | C00031->C00025:[1->2,4->1,4->3,4->8,7->5] | 1.00 | 408.644927536 | 29 | 138 | 0 | 0 |
| Path 111 | C00031->C00025:[1->2,4->1,7->3,7->5,9->8] | 1.00 | 340.067164179 | 23 | 134 | 0 | 0 |
| Path 112 | C00031->C00025:[1->2,4->1,4->3,5->5,9->8] | 1.00 | 379.774193548 | 24 | 124 | 0 | 0 |
| Path 113 | C00031->C00025:[4->3,4->8,7->1,7->5,9->2] | 1.00 | 419.21875 | 21 | 128 | 0 | 0 |
| Path 114 | C00031->C00025:[4->3,5->1,7->1,7->5,9->2,9->8] | 1.00 | 378.297297297 | 23 | 148 | 0 | 0 |
| Path 115 | C00031->C00025:[4->3,7->1,7->5,9->2,9->8] | 1.00 | 466.311594203 | 25 | 138 | 0 | 0 |
| Path 116 | C00031->C00025:[4->3,7->1,7->5,9->2,9->8] | 1.00 | 389.807453416 | 27 | 161 | 0 | 0 |
| Path 117 | C00031->C00025:[1->2,4->1,4->3,7->5,9->8] | 1.00 | 357.743055556 | 23 | 144 | 0 | 0 |
| Path 118 | C00031->C00025:[4->3,7->1,7->5,9->2,9->8] | 1.00 | 384.216216216 | 23 | 148 | 0 | 0 |
| Path 119 | C00031->C00025:[1->2,4->1,4->3,4->8,7->5] | 1.00 | 397.491935484 | 25 | 124 | 0 | 0 |
| Path 120 | C00031->C00025:[4->1,4->3,7->5,9->2,9->8] | 1.00 | 417.382352941 | 27 | 68 | 0 | 0 |
| Path 121 | C00031->C00025:[4->3,7->1,7->5,9->2,9->8] | 1.00 | 359.033783784 | 24 | 148 | 0 | 0 |
| Path 122 | C00031->C00025:[1->2,4->1,4->3,4->8,7->5] | 1.00 | 404.818897638 | 25 | 127 | 0 | 0 |
| Path 123 | C00031->C00025:[1->2,4->1,4->3,4->8,7->5] | 1.00 | 399.977272727 | 29 | 132 | 0 | 0 |
| Path 124 | C00031->C00025:[1->2,4->1,4->3,4->8,7->5] | 1.00 | 403.477272727 | 26 | 132 | 0 | 0 |
| Path 125 | C00031->C00025:[1->2,4->1,4->3,7->5,9->8] | 1.00 | 349.376712329 | 26 | 146 | 0 | 0 |
| Path 126 | C00031->C00025:[1->2,4->1,4->3,4->8,7->5] | 1.00 | 399.443609023 | 30 | 133 | 0 | 0 |
| Path 127 | C00031->C00025:[4->1,4->3,7->1,7->3,7->5,9->2,9->8] | 1.00 | 425.788732394 | 30 | 71 | 0 | 0 |
| Path 128 | C00031->C00025:[4->3,4->8,7->1,7->5,9->2] | 1.00 | 416.298387097 | 19 | 124 | 0 | 0 |
| Path 129 | C00031->C00025:[4->3,7->1,7->5,9->2,9->8] | 1.00 | 409.129770992 | 19 | 131 | 0 | 0 |
| Path 130 | C00031->C00025:[1->2,4->1,4->3,5->5,9->8] | 1.00 | 378.483870968 | 25 | 124 | 0 | 0 |
| Path 131 | C00031->C00025:[1->2,4->1,4->3,5->5,9->8] | 1.00 | 395.27480916 | 25 | 131 | 0 | 0 |
| Path 132 | C00031->C00025:[1->2,4->1,4->3,4->8,7->5] | 1.00 | 396.023255814 | 28 | 129 | 0 | 0 |
| Path 133 | C00031->C00025:[7->1,7->3,7->5,9->2,9->8] | 1.00 | 416.596774194 | 19 | 124 | 0 | 0 |
| Path 134 | C00031->C00025:[4->3,7->1,7->5,9->2,9->8] | 1.00 | 419.078125 | 20 | 128 | 0 | 0 |
| Path 135 | C00031->C00025:[4->3,4->8,7->1,7->5,9->2] | 1.00 | 423.835820896 | 24 | 134 | 0 | 0 |
| Path 136 | C00031->C00025:[1->2,4->1,4->3,5->5,9->8] | 1.00 | 390.712 | 23 | 125 | 0 | 0 |
| Path 137 | C00031->C00025:[4->3,4->8,7->1,7->5,9->2] | 1.00 | 397.865771812 | 28 | 149 | 0 | 0 |
| Path 138 | C00031->C00025:[4->3,7->1,7->5,9->2,9->8] | 1.00 | 421.328244275 | 21 | 131 | 0 | 0 |
| Path 139 | C00031->C00025:[4->3,7->1,7->5,9->2,9->8] | 1.00 | 367.356643357 | 19 | 143 | 0 | 0 |
| Path 140 | C00031->C00025:[7->1,7->3,7->5,9->2,9->8] | 1.00 | 403.709677419 | 17 | 124 | 0 | 0 |
| Path 141 | C00031->C00025:[4->3,7->1,7->5,9->2,9->8] | 1.00 | 378.424657534 | 21 | 146 | 0 | 0 |
| Path 142 | C00031->C00025:[4->3,7->1,7->5,9->2,9->8] | 1.00 | 336.452830189 | 19 | 159 | 0 | 0 |
| Path 143 | C00031->C00025:[4->3,7->1,7->5,9->2,9->8] | 1.00 | 400.392045455 | 26 | 176 | 0 | 0 |
| Path 144 | C00031->C00025:[4->3,7->1,7->5,9->2,9->8] | 1.00 | 376.452229299 | 24 | 157 | 0 | 0 |
| Path 145 | C00031->C00025:[1->2,4->1,4->3,7->5,9->8] | 1.00 | 339.182432432 | 26 | 148 | 0 | 0 |
| Path 146 | C00031->C00025:[1->2,4->1,4->3,7->5,9->8] | 1.00 | 349.654676259 | 24 | 139 | 0 | 0 |
| Path 147 | C00031->C00025:[1->2,4->1,4->3,4->8,7->5] | 1.00 | 401.181102362 | 28 | 127 | 0 | 0 |
| Path 148 | C00031->C00025:[4->3,7->1,7->5,9->2,9->8] | 1.00 | 346.097560976 | 22 | 164 | 0 | 0 |
| Path 149 | C00031->C00025:[1->2,4->1,4->3,4->8,7->5] | 1.00 | 397.240310078 | 25 | 129 | 0 | 0 |
| Path 150 | C00031->C00025:[4->3,4->8,7->1,7->3,7->5,7->8,9->2] | 1.00 | 413.227941176 | 23 | 136 | 0 | 0 |
| Path 151 | C00031->C00025:[4->3,7->1,7->5,9->2,9->8] | 1.00 | 394.596774194 | 19 | 124 | 0 | 0 |
| Path 152 | C00031->C00025:[1->2,4->1,4->3,7->5,9->8] | 1.00 | 349.215277778 | 26 | 144 | 0 | 0 |
| Path 153 | C00031->C00025:[1->2,4->1,4->3,4->8,7->5] | 1.00 | 393.113821138 | 26 | 123 | 0 | 0 |
| Path 154 | C00031->C00025:[1->2,4->1,4->3,5->5,9->8] | 1.00 | 387.099236641 | 25 | 131 | 0 | 0 |
| Path 155 | C00031->C00025:[1->2,4->1,4->3,7->5,9->8] | 1.00 | 350.503546099 | 24 | 141 | 0 | 0 |
| Path 156 | C00031->C00025:[4->3,4->8,7->1,7->5,9->2,9->8] | 1.00 | 377.151898734 | 25 | 158 | 0 | 0 |
| Path 157 | C00031->C00025:[4->3,7->1,7->5,9->2,9->8] | 1.00 | 392.140243902 | 28 | 164 | 0 | 0 |
| Path 158 | C00031->C00025:[4->3,7->1,7->5,9->2,9->8] | 1.00 | 398.36416185 | 25 | 173 | 0 | 0 |
| Path 159 | C00031->C00025:[1->2,4->1,4->3,7->5,9->8] | 1.00 | 355.496453901 | 22 | 141 | 0 | 0 |
| Path 160 | C00031->C00025:[4->3,7->1,7->5,9->2,9->8] | 1.00 | 425.8 | 22 | 130 | 0 | 0 |
| Path 161 | C00031->C00025:[4->3,4->8,7->1,7->5,9->2,9->8] | 1.00 | 373.707792208 | 23 | 154 | 0 | 0 |
| Path 162 | C00031->C00025:[4->3,7->1,7->5,9->2,9->8] | 1.00 | 371.791666667 | 19 | 144 | 0 | 0 |
| Path 163 | C00031->C00025:[1->2,4->1,7->3,7->5,9->8] | 1.00 | 344.481481481 | 22 | 135 | 0 | 0 |
| Path 164 | C00031->C00025:[1->2,4->1,4->3,7->5,9->8] | 1.00 | 350.305555556 | 23 | 144 | 0 | 0 |
| Path 165 | C00031->C00025:[4->3,4->8,7->1,7->3,7->5,7->8,9->2,9->8] | 1.00 | 378.343949045 | 26 | 157 | 0 | 0 |
| Path 166 | C00031->C00025:[1->2,4->1,4->3,7->5,9->8] | 1.00 | 348.262411348 | 25 | 141 | 0 | 0 |
| Path 167 | C00031->C00025:[1->2,4->1,7->3,7->5,9->8] | 1.00 | 345.021428571 | 25 | 140 | 0 | 0 |
| Path 168 | C00031->C00025:[7->1,7->3,7->5,9->2,9->8] | 1.00 | 332.335483871 | 18 | 155 | 0 | 0 |
| Path 169 | C00031->C00025:[7->1,7->3,7->5,9->2,9->8] | 1.00 | 437.038461538 | 17 | 104 | 0 | 0 |
| Path 170 | C00031->C00025:[1->2,4->1,4->3,7->5,9->8] | 1.00 | 351.97826087 | 21 | 138 | 0 | 0 |
| Path 171 | C00031->C00025:[1->2,4->1,4->3,4->8,7->5] | 1.00 | 395.75 | 29 | 132 | 0 | 0 |
| Path 172 | C00031->C00025:[4->3,4->8,7->1,7->5,9->2] | 1.00 | 414.179310345 | 27 | 145 | 0 | 0 |
| Path 173 | C00031->C00025:[4->3,7->1,7->5,9->2,9->8] | 1.00 | 339.802469136 | 20 | 162 | 0 | 0 |
| Path 174 | C00031->C00025:[7->1,7->3,7->5,9->2,9->8] | 1.00 | 360.264705882 | 17 | 136 | 0 | 0 |
| Path 175 | C00031->C00025:[4->3,4->8,7->1,7->5,9->2] | 1.00 | 408.401360544 | 28 | 147 | 0 | 0 |
| Path 176 | C00031->C00025:[4->3,4->8,7->1,7->5,9->2] | 1.00 | 406.138461538 | 19 | 130 | 0 | 0 |
| Path 177 | C00031->C00025:[1->2,4->1,7->3,7->5,9->8] | 1.00 | 353.792857143 | 22 | 140 | 0 | 0 |
| Path 178 | C00031->C00025:[5->1,7->1,7->3,7->5,9->2,9->8] | 1.00 | 372.281690141 | 20 | 142 | 0 | 0 |
| Path 179 | C00031->C00025:[1->2,4->1,4->3,7->5,9->8] | 1.00 | 344.586956522 | 24 | 138 | 0 | 0 |
| Path 180 | C00031->C00025:[1->2,4->1,4->3,4->8,7->5] | 1.00 | 401.8828125 | 28 | 128 | 0 | 0 |
| Path 181 | C00031->C00025:[1->2,4->1,4->3,4->8,7->5] | 1.00 | 405.542635659 | 25 | 129 | 0 | 0 |
| Path 182 | C00031->C00025:[5->1,7->3,9->2] | 0.60 | 400.009708738 | 16 | 103 | 0 | 0 |
| Path 183 | C00031->C00025:[1->2,4->1,4->3,7->5,9->8] | 1.00 | 362.632653061 | 31 | 196 | 0 | 0 |
| Path 184 | C00031->C00025:[4->1,4->3,7->5,9->2,9->8] | 1.00 | 242.797250859 | 35 | 291 | 0 | 3 |
| Path 185 | C00031->C00025:[4->1,4->3] | 0.40 | 573.375 | 16 | 24 | 0 | 0 |
| Path 186 | C00031->C00025:[4->1,4->3] | 0.40 | 212.607438017 | 18 | 242 | 0 | 2 |
| Path 187 | C00031->C00025:[4->3,7->5,9->8] | 0.60 | 347.917293233 | 18 | 133 | 0 | 0 |
| Path 188 | C00031->C00025:[4->1,4->3] | 0.40 | 211.513157895 | 18 | 228 | 0 | 2 |
| Path 189 | C00031->C00025:[1->2,4->1,4->3] | 0.60 | 391.810344828 | 18 | 116 | 0 | 0 |
| Path 190 | C00031->C00025:[1->2,4->1] | 0.40 | 434.9 | 20 | 120 | 0 | 0 |
| Path 191 | C00031->C00025:[4->3,7->5,9->8] | 0.60 | 462.009259259 | 20 | 108 | 0 | 0 |
| Path 192 | C00031->C00025:[1->2,4->1,4->3,4->8] | 0.80 | 399.592307692 | 24 | 130 | 0 | 0 |
| Path 193 | C00031->C00025:[4->1,4->3,7->1,7->3] | 0.40 | 224.193415638 | 22 | 243 | 0 | 2 |
| Path 194 | C00031->C00025:[1->2,4->1,4->3] | 0.60 | 398.84 | 20 | 125 | 0 | 0 |
| Path 195 | C00031->C00025:[1->2,4->1,4->3] | 0.60 | 396.927272727 | 19 | 110 | 0 | 0 |
| Path 196 | C00031->C00025:[4->3,4->8,7->1,7->5,9->2] | 1.00 | 395.834782609 | 19 | 115 | 0 | 0 |
| Path 197 | C00031->C00025:[4->1,4->3] | 0.40 | 585.75 | 17 | 28 | 0 | 0 |
| Path 198 | C00031->C00025:[4->3,7->1,7->5,9->2,9->8] | 1.00 | 337.585798817 | 24 | 169 | 0 | 0 |
| Path 199 | C00031->C00025:[4->3,7->5,9->8] | 0.60 | 349.183206107 | 18 | 131 | 0 | 0 |
| Path 200 | C00031->C00025:[4->1,4->3] | 0.40 | 219.709677419 | 27 | 279 | 0 | 2 |
| Path 201 | C00031->C00025:[7->1,7->5,9->2,9->8] | 0.80 | 352.081300813 | 17 | 123 | 0 | 0 |
| Path 202 | C00031->C00025:[4->3,7->5,9->8] | 0.60 | 352.947761194 | 19 | 134 | 0 | 0 |
| Path 203 | C00031->C00025:[1->2,4->1,4->3] | 0.60 | 393.47826087 | 21 | 115 | 0 | 0 |
| Path 204 | C00031->C00025:[7->3,7->5,9->8] | 0.60 | 339.182539683 | 16 | 126 | 0 | 0 |
| Path 205 | C00031->C00025:[1->2,4->1,4->3,4->8] | 0.80 | 402.225 | 24 | 120 | 0 | 0 |
| Path 206 | C00031->C00025:[4->3,4->8,7->1,7->5,9->2] | 1.00 | 391.02238806 | 24 | 134 | 0 | 0 |
| Path 207 | C00031->C00025:[7->1,7->3,9->2] | 0.60 | 404.895652174 | 15 | 115 | 0 | 0 |
| Path 208 | C00031->C00025:[1->2,4->1,4->3] | 0.60 | 389.651785714 | 20 | 112 | 0 | 0 |
| Path 209 | C00031->C00025:[5->1,5->5,9->2,9->8] | 0.80 | 393.759259259 | 17 | 108 | 0 | 0 |
| Path 210 | C00031->C00025:[1->2,4->1,4->3,4->8] | 0.80 | 402.925619835 | 25 | 121 | 0 | 0 |
| Path 211 | C00031->C00025:[4->2,4->3,4->8,7->1,7->2,7->3,7->5,7->8,9->2,9->8] | 1.00 | 353.727272727 | 24 | 143 | 0 | 0 |
| Path 212 | C00031->C00025:[1->5,4->1,4->2,4->3,4->8] | 1.00 | 263.017857143 | 27 | 280 | 0 | 2 |
| Path 213 | C00031->C00025:[4->2,4->3,4->8,7->1,7->5,9->2,9->8] | 1.00 | 348.1 | 21 | 140 | 0 | 0 |
| Path 214 | C00031->C00025:[9->3] | 0.20 | 196.64516129 | 17 | 248 | 0 | 2 |
| Path 215 | C00031->C00025:[4->1,4->3,7->1,7->3] | 0.40 | 220.529166667 | 21 | 240 | 0 | 2 |
| Path 216 | C00031->C00025:[4->3,7->5,9->8] | 0.60 | 341.880503145 | 19 | 159 | 0 | 0 |
| Path 217 | C00031->C00025:[1->2,4->1,4->3] | 0.60 | 396.627118644 | 22 | 118 | 0 | 0 |
| Path 218 | C00031->C00025:[4->3,7->1,7->5,9->2,9->8] | 1.00 | 346.507462687 | 19 | 134 | 0 | 0 |
| Path 219 | C00031->C00025:[4->3,7->1,9->2] | 0.60 | 359.146666667 | 22 | 150 | 0 | 0 |
| Path 220 | C00031->C00025:[1->2,4->1,4->3,4->8] | 0.80 | 404.370689655 | 24 | 116 | 0 | 0 |
| Path 221 | C00031->C00025:[4->1,4->3] | 0.40 | 205.153846154 | 12 | 221 | 0 | 0 |
| Path 222 | C00031->C00025:[4->3,7->5,9->8] | 0.60 | 398.669565217 | 19 | 115 | 0 | 0 |
| Path 223 | C00031->C00025:[1->2,4->1,4->3,4->8] | 0.80 | 402.503875969 | 23 | 129 | 0 | 0 |
| Path 224 | C00031->C00025:[7->3,7->5,9->8] | 0.60 | 389.463636364 | 17 | 110 | 0 | 0 |
| Path 225 | C00031->C00025:[1->8,4->5,7->3] | 0.60 | 351.1 | 24 | 170 | 0 | 0 |
| Path 226 | C00031->C00025:[1->2,4->1,4->3,4->8] | 0.80 | 397.64 | 25 | 125 | 0 | 0 |
| Path 227 | C00031->C00025:[4->3,7->5,9->8] | 0.60 | 379.262068966 | 20 | 145 | 0 | 0 |
| Path 228 | C00031->C00025:[5->1,7->3,9->2] | 0.60 | 406.692307692 | 17 | 104 | 0 | 0 |
| Path 229 | C00031->C00025:[1->2,4->1,7->5,9->8] | 0.80 | 348.04379562 | 19 | 137 | 0 | 0 |
| Path 230 | C00031->C00025:[1->8,4->3,4->5] | 0.60 | 371.782608696 | 23 | 138 | 0 | 0 |
| Path 231 | C00031->C00025:[4->3,5->1,7->5,9->2,9->8] | 1.00 | 366.174242424 | 23 | 132 | 0 | 0 |
| Path 232 | C00031->C00025:[1->2,4->1,4->3,7->5,9->8] | 1.00 | 363.533333333 | 24 | 150 | 0 | 0 |
| Path 233 | C00031->C00025:[1->8,4->5,7->3] | 0.60 | 408.303703704 | 24 | 135 | 0 | 0 |
| Path 234 | C00031->C00025:[4->1,4->3] | 0.40 | 213.755725191 | 25 | 262 | 0 | 2 |
| Path 235 | C00031->C00025:[5->1,5->5,7->1,7->5,9->2,9->8] | 0.80 | 398.756756757 | 20 | 111 | 0 | 0 |
| Path 236 | C00031->C00025:[1->2,4->1,4->3,4->5,4->8] | 1.00 | 389.365853659 | 23 | 123 | 0 | 0 |
| Path 237 | C00031->C00025:[4->1,4->3,7->1,7->3] | 0.40 | 215.366533865 | 21 | 251 | 0 | 2 |
| Path 238 | C00031->C00025:[1->2,4->1,7->3] | 0.60 | 374.405405405 | 19 | 111 | 0 | 0 |
| Path 239 | C00031->C00025:[4->3,5->1,5->5,9->2,9->8] | 1.00 | 397.052631579 | 20 | 114 | 0 | 0 |
| Path 240 | C00031->C00025:[1->2,4->1,4->3,4->5,4->8] | 1.00 | 393.379032258 | 24 | 124 | 0 | 0 |
| Path 241 | C00031->C00025:[4->1,4->3,4->8,7->1,7->2,7->3,7->8,9->5] | 1.00 | 490.457142857 | 34 | 70 | 0 | 1 |
| Path 242 | C00031->C00025:[4->1,4->3,4->8,7->1,7->2,7->3,7->8,9->5] | 1.00 | 491.5 | 36 | 74 | 0 | 1 |
| Path 243 | C00031->C00025:[7->1,9->2] | 0.40 | 400.715686275 | 15 | 102 | 0 | 0 |
| Path 244 | C00031->C00025:[4->1,4->3] | 0.40 | 260.003584229 | 28 | 279 | 0 | 2 |
| Path 245 | C00031->C00025:[4->3,7->5,9->8] | 0.60 | 396.384615385 | 19 | 117 | 0 | 0 |
| Path 246 | C00031->C00025:[4->3] | 0.20 | 203.372197309 | 14 | 223 | 0 | 2 |
| Path 247 | C00031->C00025:[1->2,4->1,7->5,9->8] | 0.80 | 338.303030303 | 19 | 132 | 0 | 0 |
| Path 248 | C00031->C00025:[4->1,4->3] | 0.40 | 225.25 | 25 | 252 | 0 | 2 |
| Path 249 | C00031->C00025:[7->3,7->5,9->8] | 0.60 | 333.461038961 | 17 | 154 | 0 | 0 |
| Path 250 | C00031->C00025:[4->3,7->1,9->2] | 0.60 | 370.649006623 | 23 | 151 | 0 | 0 |
| Path 251 | C00031->C00025:[1->2,4->1,4->3,4->8] | 0.80 | 405.244094488 | 23 | 127 | 0 | 0 |
| Path 252 | C00031->C00025:[7->5,9->8] | 0.40 | 331.694444444 | 10 | 108 | 0 | 0 |
| Path 253 | C00031->C00025:[7->1,7->3,7->5,9->2,9->8] | 1.00 | 337.763779528 | 17 | 127 | 0 | 0 |
| Path 254 | C00031->C00025:[5->1,5->5,7->1,7->5,9->2,9->8] | 0.80 | 397.545454545 | 17 | 121 | 0 | 0 |
| Path 255 | C00031->C00025:[4->1,4->3] | 0.40 | 214.037037037 | 18 | 243 | 0 | 2 |
| Path 256 | C00031->C00025:[1->2,4->1,7->5,9->8] | 0.80 | 341.526717557 | 17 | 131 | 0 | 0 |
| Path 257 | C00031->C00025:[4->3,5->1,5->5,9->2,9->8] | 1.00 | 347.443708609 | 21 | 151 | 0 | 0 |
| Path 258 | C00031->C00025:[4->1,4->3] | 0.40 | 255.203883495 | 19 | 206 | 0 | 0 |
| Path 259 | C00031->C00025:[1->2,4->1,4->3,4->8] | 0.80 | 402.7 | 24 | 120 | 0 | 0 |
| Path 260 | C00031->C00025:[7->1,7->5,9->2,9->8] | 0.80 | 370.014388489 | 24 | 139 | 0 | 0 |
| Path 261 | C00031->C00025:[1->2,1->8,4->1,4->3,4->5] | 1.00 | 415.748251748 | 22 | 143 | 0 | 0 |
| Path 262 | C00031->C00025:[4->3,7->1,9->2] | 0.60 | 358.288590604 | 21 | 149 | 0 | 0 |
| Path 263 | C00031->C00025:[4->3,4->8,7->3,7->8] | 0.40 | 396.817391304 | 19 | 115 | 0 | 0 |
| Path 264 | C00031->C00025:[4->1,4->3] | 0.40 | 217.06504065 | 21 | 246 | 0 | 2 |
| Path 265 | C00031->C00025:[7->1,7->3,9->2] | 0.60 | 346.290780142 | 18 | 141 | 0 | 0 |
| Path 266 | C00031->C00025:[1->2,4->1,4->3,4->8] | 0.80 | 400.171641791 | 26 | 134 | 0 | 0 |
| Path 267 | C00031->C00025:[1->2,4->1,4->3,7->3,7->5,9->8] | 1.00 | 366.457286432 | 34 | 199 | 0 | 0 |
| Path 268 | C00031->C00025:[4->3,4->8,7->1,7->5,9->2] | 1.00 | 383.410447761 | 27 | 134 | 0 | 0 |
| Path 269 | C00031->C00025:[1->2,4->1,5->5,9->8] | 0.80 | 378.040322581 | 21 | 124 | 0 | 0 |
| Path 270 | C00031->C00025:[4->3,4->8,7->1,7->5,9->2] | 1.00 | 387.140740741 | 28 | 135 | 0 | 0 |
| Path 271 | C00031->C00025:[1->2,4->1,4->3] | 0.60 | 387.87704918 | 20 | 122 | 0 | 0 |
| Path 272 | C00031->C00025:[1->2,4->1,4->3,4->8] | 0.80 | 411.467213115 | 26 | 122 | 0 | 0 |
| Path 273 | C00031->C00025:[1->2,4->1] | 0.40 | 378.156521739 | 16 | 115 | 0 | 0 |
| Path 274 | C00031->C00025:[4->1,4->3,7->1,7->3] | 0.40 | 215.559322034 | 20 | 236 | 0 | 2 |
| Path 275 | C00031->C00025:[7->1,7->3] | 0.40 | 205.410480349 | 16 | 229 | 0 | 2 |
| Path 276 | C00031->C00025:[4->3,7->5,9->8] | 0.60 | 376.345070423 | 19 | 142 | 0 | 0 |
| Path 277 | C00031->C00025:[7->1,7->3,9->2] | 0.60 | 400.259615385 | 17 | 104 | 0 | 0 |
| Path 278 | C00031->C00025:[7->1,7->3,9->2] | 0.60 | 406.180952381 | 18 | 105 | 0 | 0 |
| Path 279 | C00031->C00025:[4->1,4->3] | 0.40 | 199.539170507 | 10 | 217 | 0 | 0 |
| Path 280 | C00031->C00025:[7->1,7->3,9->2] | 0.60 | 351.563380282 | 19 | 142 | 0 | 0 |
| Path 281 | C00031->C00025:[4->1,4->3] | 0.40 | 216.828897338 | 26 | 263 | 0 | 2 |
| Path 282 | C00031->C00025:[4->3,7->1,9->2] | 0.60 | 367.360544218 | 21 | 147 | 0 | 0 |
| Path 283 | C00031->C00025:[1->8,4->3,4->5] | 0.60 | 363.75147929 | 25 | 169 | 0 | 0 |
| Path 284 | C00031->C00025:[4->3,7->1,7->5,9->2,9->8] | 1.00 | 429.825174825 | 26 | 143 | 0 | 0 |
| Path 285 | C00031->C00025:[1->2,4->1,4->3] | 0.60 | 438.801470588 | 23 | 136 | 0 | 0 |
| Path 286 | C00031->C00025:[4->1,4->3] | 0.40 | 213.361538462 | 25 | 260 | 0 | 2 |
| Path 287 | C00031->C00025:[4->3,5->1,5->5,7->1,7->5,9->2,9->8] | 1.00 | 407.584745763 | 24 | 118 | 0 | 0 |
| Path 288 | C00031->C00025:[1->2,4->1,4->3] | 0.60 | 390.884297521 | 19 | 121 | 0 | 0 |
| Path 289 | C00031->C00025:[4->3,7->1,7->5,9->2,9->8] | 1.00 | 341.566473988 | 26 | 173 | 0 | 0 |
| Path 290 | C00031->C00025:[5->1,5->5,7->1,7->5,9->2,9->8] | 0.80 | 398.099099099 | 20 | 111 | 0 | 0 |
| Path 291 | C00031->C00025:[4->1,4->3] | 0.40 | 219.379928315 | 28 | 279 | 0 | 2 |
| Path 292 | C00031->C00025:[1->2,1->8,4->1,4->5,7->3] | 1.00 | 413.438848921 | 21 | 139 | 0 | 0 |
| Path 293 | C00031->C00025:[7->1,7->3,7->5,9->2,9->8] | 1.00 | 395.681415929 | 20 | 113 | 0 | 0 |
| Path 294 | C00031->C00025:[1->2,4->1,4->3,4->8] | 0.80 | 408.164179104 | 26 | 134 | 0 | 0 |
| Path 295 | C00031->C00025:[1->2,4->1,4->3] | 0.60 | 440.496402878 | 24 | 139 | 0 | 0 |
| Path 296 | C00031->C00025:[4->3,7->1,7->5,9->2,9->8] | 1.00 | 327.119047619 | 23 | 168 | 0 | 0 |
| Path 297 | C00031->C00025:[4->3,7->1,9->2] | 0.60 | 353.804054054 | 20 | 148 | 0 | 0 |
| Path 298 | C00031->C00025:[1->2,1->8,4->1,4->5,7->3] | 1.00 | 466.045454545 | 22 | 132 | 0 | 0 |
| Path 299 | C00031->C00025:[1->2,4->1,4->3,4->8] | 0.80 | 404.504 | 23 | 125 | 0 | 0 |
| Path 300 | C00031->C00025:[1->2,4->1,4->3,4->8] | 0.80 | 403.153846154 | 24 | 130 | 0 | 0 |
| Path 301 | C00031->C00025:[4->1,4->3] | 0.40 | 259.615646259 | 28 | 294 | 0 | 2 |
| Path 302 | C00031->C00025:[4->1,4->3,7->5,9->8] | 0.80 | 413.492537313 | 27 | 67 | 0 | 0 |
| Path 303 | C00031->C00025:[4->1,4->3] | 0.40 | 214.778688525 | 20 | 244 | 0 | 2 |
| Path 304 | C00031->C00025:[4->3,5->1,7->3,7->5,9->2,9->8] | 1.00 | 345.552325581 | 27 | 172 | 0 | 0 |
| Path 305 | C00031->C00025:[4->3,7->1,7->5,9->2,9->8] | 1.00 | 354.0 | 21 | 136 | 0 | 0 |
| Path 306 | C00031->C00025:[9->1] | 0.20 | 376.919191919 | 11 | 99 | 0 | 0 |
| Path 307 | C00031->C00025:[4->3,4->8,7->1,7->5,9->2,9->8] | 1.00 | 351.255172414 | 24 | 145 | 0 | 0 |
| Path 308 | C00031->C00025:[7->3] | 0.20 | 203.044843049 | 16 | 223 | 0 | 2 |
| Path 309 | C00031->C00025:[1->2,4->1,4->3,4->8] | 0.80 | 413.423357664 | 28 | 137 | 0 | 0 |
| Path 310 | C00031->C00025:[4->1,4->3] | 0.40 | 215.673076923 | 24 | 260 | 0 | 2 |
| Path 311 | C00031->C00025:[4->1,4->3] | 0.40 | 306.698198198 | 21 | 222 | 0 | 0 |
| Path 312 | C00031->C00025:[7->1,7->3,9->2] | 0.60 | 351.049295775 | 19 | 142 | 0 | 0 |
| Path 313 | C00031->C00025:[4->1,4->3] | 0.40 | 212.716157205 | 19 | 229 | 0 | 2 |
| Path 314 | C00031->C00025:[4->3,7->5,9->8] | 0.60 | 401.556451613 | 19 | 124 | 0 | 0 |
| Path 315 | C00031->C00025:[4->3,5->1,9->2] | 0.60 | 610.25 | 22 | 120 | 0 | 0 |
| Path 316 | C00031->C00025:[4->3,4->8,7->1,7->5,9->2] | 1.00 | 391.860465116 | 23 | 129 | 0 | 0 |
| Path 317 | C00031->C00025:[4->3,7->5,9->8] | 0.60 | 344.007692308 | 17 | 130 | 0 | 0 |
| Path 318 | C00031->C00025:[4->3,5->1,7->5,9->2,9->8] | 1.00 | 342.931034483 | 27 | 174 | 0 | 0 |
| Path 319 | C00031->C00025:[4->3,4->8,7->1,7->5,9->2,9->8] | 1.00 | 346.758865248 | 22 | 141 | 0 | 0 |
| Path 320 | C00031->C00025:[1->2,4->1,4->3,4->8] | 0.80 | 405.605839416 | 28 | 137 | 0 | 0 |
| Path 321 | C00031->C00025:[1->2,4->1,7->3] | 0.60 | 383.911504425 | 19 | 113 | 0 | 0 |
| Path 322 | C00031->C00025:[4->3,7->1,9->2] | 0.60 | 371.132450331 | 23 | 151 | 0 | 0 |
| Path 323 | C00031->C00025:[1->2,4->1,4->3,7->5,9->8] | 1.00 | 397.46835443 | 26 | 158 | 0 | 0 |
| Path 324 | C00031->C00025:[4->3,5->1,9->2] | 0.60 | 406.651376147 | 19 | 109 | 0 | 0 |
| Path 325 | C00031->C00025:[4->3,7->1,9->2] | 0.60 | 358.77852349 | 21 | 149 | 0 | 0 |
| Path 326 | C00031->C00025:[4->1,4->3,4->8] | 0.60 | 240.57751938 | 29 | 258 | 0 | 3 |
| Path 327 | C00031->C00025:[4->3] | 0.20 | 397.196078431 | 13 | 102 | 0 | 0 |
| Path 328 | C00031->C00025:[1->2,4->1,4->3] | 0.60 | 532.154285714 | 28 | 175 | 0 | 0 |
| Path 329 | C00031->C00025:[1->2,4->1,7->3] | 0.60 | 374.189655172 | 20 | 116 | 0 | 0 |
| Path 330 | C00031->C00025:[4->1,4->3] | 0.40 | 564.285714286 | 18 | 28 | 0 | 0 |
| Path 331 | C00031->C00025:[4->3,7->1,7->3,9->2] | 0.60 | 360.771812081 | 23 | 149 | 0 | 0 |
| Path 332 | C00031->C00025:[4->3,7->1,7->5,9->2,9->8] | 1.00 | 362.236842105 | 24 | 152 | 0 | 0 |
| Path 333 | C00031->C00025:[1->2,4->1,4->3,4->8] | 0.80 | 397.609375 | 26 | 128 | 0 | 0 |
| Path 334 | C00031->C00025:[1->2,4->1,7->3] | 0.60 | 387.794871795 | 21 | 117 | 0 | 0 |
| Path 335 | C00031->C00025:[4->3,5->1,7->5,9->2,9->8] | 1.00 | 339.005882353 | 25 | 170 | 0 | 0 |
| Path 336 | C00031->C00025:[4->3,7->5,9->8] | 0.60 | 383.664383562 | 21 | 146 | 0 | 0 |
| Path 337 | C00031->C00025:[1->2,4->1,7->5,9->8] | 0.80 | 333.740458015 | 20 | 131 | 0 | 0 |
| Path 338 | C00031->C00025:[4->1,4->3,4->8,7->2,9->5] | 1.00 | 468.057142857 | 32 | 70 | 0 | 1 |
| Path 339 | C00031->C00025:[1->2,4->1,7->3] | 0.60 | 361.466101695 | 20 | 118 | 0 | 0 |
| Path 340 | C00031->C00025:[1->2,4->1,4->3] | 0.60 | 376.210526316 | 20 | 114 | 0 | 0 |
| Path 341 | C00031->C00025:[4->3,7->1,7->5,9->2,9->8] | 1.00 | 365.547297297 | 26 | 148 | 0 | 0 |
| Path 342 | C00031->C00025:[1->8,4->3,4->5] | 0.60 | 360.975903614 | 24 | 166 | 0 | 0 |
| Path 343 | C00031->C00025:[7->1,7->5,9->2,9->8] | 0.80 | 384.122641509 | 15 | 106 | 0 | 0 |
| Path 344 | C00031->C00025:[4->3,7->1,9->2] | 0.60 | 354.697986577 | 21 | 149 | 0 | 0 |
| Path 345 | C00031->C00025:[4->3,7->1,7->5,9->2,9->8] | 1.00 | 351.65034965 | 22 | 143 | 0 | 0 |
| Path 346 | C00031->C00025:[4->1,4->3,7->1,7->3] | 0.40 | 264.437837838 | 16 | 185 | 0 | 0 |
| Path 347 | C00031->C00025:[4->1,4->3] | 0.40 | 261.973063973 | 30 | 297 | 0 | 2 |
| Path 348 | C00031->C00025:[4->3,5->1,5->5,9->2,9->8] | 1.00 | 403.12173913 | 21 | 115 | 0 | 0 |
| Path 349 | C00031->C00025:[4->1,4->3,4->8,7->1,7->3,7->8] | 0.60 | 248.007326007 | 35 | 273 | 0 | 3 |
| Path 350 | C00031->C00025:[4->1,4->3] | 0.40 | 210.106666667 | 16 | 225 | 0 | 2 |
| Path 351 | C00031->C00025:[1->2,4->1,4->3,7->5,9->8] | 1.00 | 353.743589744 | 30 | 195 | 0 | 0 |
| Path 352 | C00031->C00025:[4->3,4->8,7->1,7->5,9->2] | 1.00 | 383.953488372 | 26 | 129 | 0 | 0 |
| Path 353 | C00031->C00025:[4->3,7->1,7->3,9->2] | 0.60 | 371.853333333 | 24 | 150 | 0 | 0 |
| Path 354 | C00031->C00025:[4->3,5->1,7->5,9->2,9->8] | 1.00 | 371.654135338 | 24 | 133 | 0 | 0 |
| Path 355 | C00031->C00025:[4->1,4->3] | 0.40 | 220.463519313 | 19 | 233 | 0 | 2 |
| Path 356 | C00031->C00025:[4->1,4->3] | 0.40 | 252.298507463 | 20 | 268 | 0 | 0 |
| Path 357 | C00031->C00025:[1->2,4->1,5->5,9->8] | 0.80 | 369.735042735 | 20 | 117 | 0 | 0 |
| Path 358 | C00031->C00025:[4->1,4->3] | 0.40 | 261.771428571 | 13 | 175 | 0 | 0 |
| Path 359 | C00031->C00025:[4->3,7->1,9->2] | 0.60 | 355.020547945 | 20 | 146 | 0 | 0 |
| Path 360 | C00031->C00025:[7->1,7->3,9->2] | 0.60 | 406.876190476 | 18 | 105 | 0 | 0 |
| Path 361 | C00031->C00025:[4->1,4->3] | 0.40 | 216.049618321 | 24 | 262 | 0 | 2 |
| Path 362 | C00031->C00025:[1->2,4->1,4->3,4->8] | 0.80 | 393.933884298 | 23 | 121 | 0 | 0 |
| Path 363 | C00031->C00025:[1->2,4->1,4->3] | 0.60 | 396.655737705 | 20 | 122 | 0 | 0 |
| Path 364 | C00031->C00025:[4->1,4->3] | 0.40 | 224.945378151 | 25 | 238 | 0 | 2 |
| Path 365 | C00031->C00025:[1->2,4->1,4->3] | 0.60 | 393.943548387 | 20 | 124 | 0 | 0 |
| Path 366 | C00031->C00025:[4->1,4->3,7->1,7->3] | 0.40 | 222.13229572 | 24 | 257 | 0 | 2 |
| Path 367 | C00031->C00025:[7->3,7->5,9->8] | 0.60 | 455.252427184 | 18 | 103 | 0 | 0 |
| Path 368 | C00031->C00025:[1->2,4->1,4->3,4->8] | 0.80 | 407.830769231 | 24 | 130 | 0 | 0 |
| Path 369 | C00031->C00025:[1->2,4->1] | 0.40 | 432.899224806 | 19 | 129 | 0 | 0 |
| Path 370 | C00031->C00025:[1->2,4->1,4->3,7->3] | 0.60 | 381.822857143 | 29 | 175 | 0 | 0 |
| Path 371 | C00031->C00025:[1->2,4->1,4->3] | 0.60 | 387.743589744 | 20 | 117 | 0 | 0 |
| Path 372 | C00031->C00025:[4->1,4->3,7->1,7->3] | 0.40 | 219.347280335 | 21 | 239 | 0 | 2 |
| Path 373 | C00031->C00025:[4->3,4->8,7->1,7->5,9->2] | 1.00 | 399.663865546 | 21 | 119 | 0 | 0 |
| Path 374 | C00031->C00025:[1->2,4->1,4->3,7->5,9->8] | 1.00 | 391.012658228 | 29 | 158 | 0 | 0 |
| Path 375 | C00031->C00025:[5->1,7->3,9->2] | 0.60 | 351.035460993 | 18 | 141 | 0 | 0 |
| Path 376 | C00031->C00025:[1->2,4->1,7->5,9->8] | 0.80 | 324.739130435 | 21 | 138 | 0 | 0 |
| Path 377 | C00031->C00025:[1->2,4->1,4->3,4->5,4->8] | 1.00 | 395.812030075 | 23 | 133 | 0 | 0 |
| Path 378 | C00031->C00025:[4->1,4->3,7->1,7->3] | 0.40 | 219.623529412 | 24 | 255 | 0 | 2 |
| Path 379 | C00031->C00025:[1->2,1->8,4->1,4->3,4->5] | 1.00 | 417.835616438 | 23 | 146 | 0 | 0 |
| Path 380 | C00031->C00025:[4->1,4->3] | 0.40 | 579.64516129 | 17 | 31 | 0 | 0 |
| Path 381 | C00031->C00025:[4->1,4->3] | 0.40 | 215.340611354 | 18 | 229 | 0 | 2 |
| Path 382 | C00031->C00025:[4->1,4->3] | 0.40 | 229.6953125 | 27 | 256 | 0 | 2 |
| Path 383 | C00031->C00025:[4->3,7->1,7->3,9->2] | 0.60 | 367.489932886 | 23 | 149 | 0 | 0 |
| Path 384 | C00031->C00025:[1->2,4->1] | 0.40 | 365.0 | 16 | 107 | 0 | 0 |
| Path 385 | C00031->C00025:[1->2,4->1,4->3] | 0.60 | 398.943820225 | 27 | 178 | 0 | 0 |
| Path 386 | C00031->C00025:[1->8,4->5] | 0.40 | 365.586206897 | 16 | 116 | 0 | 0 |
| Path 387 | C00031->C00025:[4->3,4->8,7->1,7->3,7->5,7->8,9->2] | 1.00 | 404.314049587 | 23 | 121 | 0 | 0 |
| Path 388 | C00031->C00025:[1->2,4->1,4->3,7->3,7->5,9->8] | 1.00 | 371.582914573 | 31 | 199 | 0 | 0 |
| Path 389 | C00031->C00025:[4->3,5->1,7->5,9->2,9->8] | 1.00 | 347.228571429 | 28 | 175 | 0 | 0 |
| Path 390 | C00031->C00025:[1->2,4->1,4->3,4->8] | 0.80 | 404.3125 | 24 | 128 | 0 | 0 |
| Path 391 | C00031->C00025:[4->2,4->3,4->8] | 0.60 | 398.353982301 | 18 | 113 | 0 | 0 |
| Path 392 | C00031->C00025:[7->3,7->5,9->8] | 0.60 | 460.701923077 | 19 | 104 | 0 | 0 |
| Path 393 | C00031->C00025:[4->3,5->1,7->3,9->2] | 0.60 | 371.979865772 | 23 | 149 | 0 | 0 |
| Path 394 | C00031->C00025:[4->3,7->1,7->5,9->2,9->8] | 1.00 | 368.562913907 | 27 | 151 | 0 | 0 |
| Path 395 | C00031->C00025:[1->2,4->1,4->3,4->8] | 0.80 | 408.435114504 | 25 | 131 | 0 | 0 |
| Path 396 | C00031->C00025:[1->2,4->1,7->5,9->8] | 0.80 | 340.226277372 | 19 | 137 | 0 | 0 |
| Path 397 | C00031->C00025:[1->2,4->1,4->3,4->8] | 0.80 | 406.4 | 22 | 115 | 0 | 0 |
| Path 398 | C00031->C00025:[4->3,7->1,7->5,9->2,9->8] | 1.00 | 362.607692308 | 21 | 130 | 0 | 0 |
| Path 399 | C00031->C00025:[4->3,7->1,7->5,9->2,9->8] | 1.00 | 474.089385475 | 26 | 179 | 0 | 0 |
| Path 400 | C00031->C00025:[4->1,4->3,7->5,9->2,9->8] | 1.00 | 252.378737542 | 42 | 301 | 0 | 3 |
| Path 401 | C00031->C00025:[1->2,4->1,4->3] | 0.60 | 397.24137931 | 22 | 116 | 0 | 0 |
| Path 402 | C00031->C00025:[4->1,4->3] | 0.40 | 225.396491228 | 31 | 285 | 0 | 2 |
| Path 403 | C00031->C00025:[4->3,7->1,7->5,9->2,9->8] | 1.00 | 331.377245509 | 22 | 167 | 0 | 0 |
| Path 404 | C00031->C00025:[4->1,4->3,7->1,7->3] | 0.40 | 216.832635983 | 22 | 239 | 0 | 2 |
| Path 405 | C00031->C00025:[4->1,4->3] | 0.40 | 206.75 | 18 | 252 | 0 | 0 |
| Path 406 | C00031->C00025:[4->3,5->1,5->5,7->1,7->5,9->2,9->8] | 1.00 | 351.948051948 | 24 | 154 | 0 | 0 |
| Path 407 | C00031->C00025:[1->2,4->1,4->3] | 0.60 | 388.801136364 | 25 | 176 | 0 | 0 |
| Path 408 | C00031->C00025:[4->3,7->1,7->3,9->2] | 0.60 | 355.810810811 | 22 | 148 | 0 | 0 |
| Path 409 | C00031->C00025:[7->5,9->3,9->8] | 0.60 | 210.799212598 | 20 | 254 | 0 | 2 |
| Path 410 | C00031->C00025:[4->1,4->3,7->1,7->3] | 0.40 | 224.104247104 | 26 | 259 | 0 | 2 |
| Path 411 | C00031->C00025:[2->3,7->5,9->8] | 0.60 | 249.839590444 | 26 | 293 | 0 | 2 |
| Path 412 | C00031->C00025:[1->2,4->1,4->3,4->5,4->8] | 1.00 | 392.865079365 | 24 | 126 | 0 | 0 |
| Path 413 | C00031->C00025:[1->2,4->1,4->3] | 0.60 | 388.452380952 | 27 | 168 | 0 | 0 |
| Path 414 | C00031->C00025:[1->2,4->1,7->3] | 0.60 | 387.290598291 | 18 | 117 | 0 | 0 |
| Path 415 | C00031->C00025:[4->3,5->1,7->3,9->2] | 0.60 | 355.333333333 | 21 | 147 | 0 | 0 |
| Path 416 | C00031->C00025:[1->2,4->1,4->3] | 0.60 | 379.829059829 | 21 | 117 | 0 | 0 |
| Path 417 | C00031->C00025:[1->2,4->1,7->3] | 0.60 | 378.572649573 | 21 | 117 | 0 | 0 |
| Path 418 | C00031->C00025:[4->3,5->1,9->2] | 0.60 | 364.768707483 | 21 | 147 | 0 | 0 |
| Path 419 | C00031->C00025:[7->5,9->8] | 0.40 | 390.510869565 | 11 | 92 | 0 | 0 |
| Path 420 | C00031->C00025:[7->1,9->2] | 0.40 | 400.0 | 15 | 102 | 0 | 0 |
| Path 421 | C00031->C00025:[4->2,4->3,4->8] | 0.60 | 394.266055046 | 16 | 109 | 0 | 0 |
| Path 422 | C00031->C00025:[4->1,4->3,4->8,7->1,7->2,7->3,7->8,9->5] | 1.00 | 250.230215827 | 38 | 278 | 0 | 3 |
| Path 423 | C00031->C00025:[4->1,4->3,7->1,7->3] | 0.40 | 513.205128205 | 20 | 39 | 0 | 0 |
| Path 424 | C00031->C00025:[1->2,4->1,7->5,9->8] | 0.80 | 335.051470588 | 21 | 136 | 0 | 0 |
| Path 425 | C00031->C00025:[1->2,4->1,4->3,4->8] | 0.80 | 401.134920635 | 27 | 126 | 0 | 0 |
| Path 426 | C00031->C00025:[4->1,4->3] | 0.40 | 232.202479339 | 25 | 242 | 0 | 2 |
| Path 427 | C00031->C00025:[4->3,7->1,7->5,9->2,9->8] | 1.00 | 364.549618321 | 22 | 131 | 0 | 0 |
| Path 428 | C00031->C00025:[4->3,7->5,9->8] | 0.60 | 456.775700935 | 19 | 107 | 0 | 0 |
| Path 429 | C00031->C00025:[4->1,4->3,7->1,7->3] | 0.40 | 262.216216216 | 31 | 296 | 0 | 2 |
| Path 430 | C00031->C00025:[2->3] | 0.20 | 243.496212121 | 19 | 264 | 0 | 2 |
| Path 431 | C00031->C00025:[4->3,7->1,7->5,9->2,9->8] | 1.00 | 399.111111111 | 21 | 117 | 0 | 0 |
| Path 432 | C00031->C00025:[1->2,4->1] | 0.40 | 381.80733945 | 14 | 109 | 0 | 0 |
| Path 433 | C00031->C00025:[1->2,4->1,4->3,4->5,4->8] | 1.00 | 391.346456693 | 21 | 127 | 0 | 0 |
| Path 434 | C00031->C00025:[4->3,7->1,9->2] | 0.60 | 362.376712329 | 20 | 146 | 0 | 0 |
| Path 435 | C00031->C00025:[7->5,9->8] | 0.40 | 370.838709677 | 10 | 93 | 0 | 0 |
| Path 436 | C00031->C00025:[4->1,4->3,4->8,7->2,9->5] | 1.00 | 257.964539007 | 41 | 282 | 0 | 3 |
| Path 437 | C00031->C00025:[4->1,4->3] | 0.40 | 203.872727273 | 11 | 220 | 0 | 0 |
| Path 438 | C00031->C00025:[1->2,4->1,4->3,4->5,4->8] | 1.00 | 377.112 | 23 | 125 | 0 | 0 |
| Path 439 | C00031->C00025:[1->2,4->1,4->3] | 0.60 | 396.396551724 | 25 | 174 | 0 | 0 |
| Path 440 | C00031->C00025:[4->3,7->1,9->2] | 0.60 | 407.957983193 | 16 | 119 | 0 | 0 |
| Path 441 | C00031->C00025:[5->1,7->3,7->5,9->2,9->8] | 1.00 | 344.1171875 | 18 | 128 | 0 | 0 |
| Path 442 | C00031->C00025:[4->1,4->3] | 0.40 | 567.148148148 | 17 | 27 | 0 | 0 |
| Path 443 | C00031->C00025:[4->1,4->3,7->1,7->3] | 0.40 | 513.473684211 | 20 | 38 | 0 | 0 |
| Path 444 | C00031->C00025:[4->3,7->1,9->2] | 0.60 | 608.764705882 | 21 | 119 | 0 | 0 |
| Path 445 | C00031->C00025:[4->3,5->1,5->5,9->2,9->8] | 1.00 | 377.674033149 | 31 | 181 | 0 | 0 |
| Path 446 | C00031->C00025:[4->1,4->3,4->8,7->2,9->5] | 1.00 | 254.341726619 | 39 | 278 | 0 | 3 |
| Path 447 | C00031->C00025:[4->3,5->1,9->2] | 0.60 | 388.837837838 | 17 | 111 | 0 | 0 |
| Path 448 | C00031->C00025:[1->2,4->1,4->3] | 0.60 | 394.974789916 | 19 | 119 | 0 | 0 |
| Path 449 | C00031->C00025:[7->3,9->1] | 0.40 | 383.960784314 | 14 | 102 | 0 | 0 |
| Path 450 | C00031->C00025:[4->1,4->3,7->1,7->3] | 0.40 | 218.933070866 | 22 | 254 | 0 | 2 |
| Path 451 | C00031->C00025:[1->2,4->1,4->3,4->8] | 0.80 | 399.273504274 | 23 | 117 | 0 | 0 |
| Path 452 | C00031->C00025:[4->2,4->3,4->8] | 0.60 | 397.5625 | 17 | 112 | 0 | 0 |
| Path 453 | C00031->C00025:[1->2,4->1,4->3] | 0.60 | 387.764705882 | 21 | 119 | 0 | 0 |
| Path 454 | C00031->C00025:[4->1,4->3] | 0.40 | 260.074576271 | 30 | 295 | 0 | 2 |
| Path 455 | C00031->C00025:[1->2,4->1,7->3] | 0.60 | 384.211864407 | 19 | 118 | 0 | 0 |
| Path 456 | C00031->C00025:[7->1,7->5,9->2,9->8] | 0.80 | 399.198113208 | 17 | 106 | 0 | 0 |
| Path 457 | C00031->C00025:[4->3,7->1,7->5,9->2,9->8] | 1.00 | 335.922619048 | 23 | 168 | 0 | 0 |
| Path 458 | C00031->C00025:[4->1,4->3,7->1,7->3] | 0.40 | 513.5 | 21 | 42 | 0 | 0 |
| Path 459 | C00031->C00025:[4->1,4->3] | 0.40 | 262.157706093 | 27 | 279 | 0 | 2 |
| Path 460 | C00031->C00025:[4->1,4->3,4->8,7->1,7->2,7->3,7->8,9->5] | 1.00 | 474.150684932 | 35 | 73 | 0 | 1 |
| Path 461 | C00031->C00025:[7->1,7->3,7->5,9->2,9->8] | 1.00 | 345.798449612 | 19 | 129 | 0 | 0 |
| Path 462 | C00031->C00025:[1->2,4->1,4->3,4->8] | 0.80 | 406.159090909 | 28 | 132 | 0 | 0 |
| Path 463 | C00031->C00025:[4->3,4->8,7->1,7->5,9->2] | 1.00 | 380.766666667 | 19 | 120 | 0 | 0 |
| Path 464 | C00031->C00025:[7->1,7->3] | 0.40 | 255.904411765 | 25 | 272 | 0 | 2 |
| Path 465 | C00031->C00025:[4->1,4->3,7->1,7->3] | 0.40 | 213.012711864 | 21 | 236 | 0 | 2 |
| Path 466 | C00031->C00025:[7->3,7->5,9->8] | 0.60 | 344.559055118 | 17 | 127 | 0 | 0 |
| Path 467 | C00031->C00025:[4->1,4->3] | 0.40 | 264.96013289 | 33 | 301 | 0 | 2 |
| Path 468 | C00031->C00025:[4->3,4->8] | 0.40 | 387.220183486 | 15 | 109 | 0 | 0 |
| Path 469 | C00031->C00025:[4->3,5->1,5->5,9->2,9->8] | 1.00 | 352.361842105 | 22 | 152 | 0 | 0 |
| Path 470 | C00031->C00025:[4->3,7->5,9->8] | 0.60 | 214.558139535 | 22 | 258 | 0 | 2 |
| Path 471 | C00031->C00025:[1->2,4->1,4->3,4->5,4->8] | 1.00 | 390.515151515 | 22 | 132 | 0 | 0 |
| Path 472 | C00031->C00025:[7->1,7->5,9->2,9->8] | 0.80 | 345.991803279 | 16 | 122 | 0 | 0 |
| Path 473 | C00031->C00025:[4->3,5->1,9->2] | 0.60 | 412.909090909 | 20 | 110 | 0 | 0 |
| Path 474 | C00031->C00025:[4->1,4->3,4->8,7->1,7->2,7->3,7->8,9->5] | 1.00 | 251.938181818 | 37 | 275 | 0 | 3 |
| Path 475 | C00031->C00025:[5->1,7->3,9->2] | 0.60 | 381.304761905 | 14 | 105 | 0 | 0 |
| Path 476 | C00031->C00025:[4->1,4->3] | 0.40 | 214.601449275 | 25 | 276 | 0 | 2 |
| Path 477 | C00031->C00025:[4->1,4->3,4->8,7->2,9->5] | 1.00 | 246.236363636 | 35 | 275 | 0 | 3 |
| Path 478 | C00031->C00025:[7->1,7->3] | 0.40 | 504.535714286 | 15 | 28 | 0 | 0 |
| Path 479 | C00031->C00025:[4->1,4->3,7->1,7->3] | 0.40 | 225.517374517 | 26 | 259 | 0 | 2 |
| Path 480 | C00031->C00025:[9->1,9->3] | 0.40 | 339.577235772 | 15 | 123 | 0 | 0 |
| Path 481 | C00031->C00025:[5->1,5->5,9->2,9->8] | 0.80 | 429.224637681 | 24 | 138 | 0 | 0 |
| Path 482 | C00031->C00025:[5->1,5->5,9->2,9->8] | 0.80 | 418.437956204 | 26 | 137 | 0 | 0 |
| Path 483 | C00031->C00025:[1->2,4->1,4->3] | 0.60 | 384.413793103 | 20 | 116 | 0 | 0 |
| Path 484 | C00031->C00025:[7->1,9->2] | 0.40 | 399.232142857 | 12 | 112 | 0 | 0 |
| Path 485 | C00031->C00025:[4->3,7->1,7->3,7->5,9->2,9->8] | 1.00 | 340.842105263 | 26 | 171 | 0 | 0 |
| Path 486 | C00031->C00025:[4->3,4->8,7->3,7->8] | 0.40 | 378.230769231 | 18 | 117 | 0 | 0 |
| Path 487 | C00031->C00025:[1->8,4->5] | 0.40 | 417.801652893 | 15 | 121 | 0 | 0 |
| Path 488 | C00031->C00025:[7->3] | 0.20 | 194.486607143 | 13 | 224 | 0 | 2 |
| Path 489 | C00031->C00025:[4->3,5->1,5->5,9->2,9->8] | 1.00 | 434.937931034 | 28 | 145 | 0 | 0 |
| Path 490 | C00031->C00025:[4->3,7->1,9->2] | 0.60 | 413.017857143 | 20 | 112 | 0 | 0 |
| Path 491 | C00031->C00025:[7->5,9->3,9->8] | 0.60 | 215.83984375 | 22 | 256 | 0 | 2 |
| Path 492 | C00031->C00025:[4->1,4->3] | 0.40 | 261.663299663 | 31 | 297 | 0 | 2 |
| Path 493 | C00031->C00025:[1->2,4->1,4->3] | 0.60 | 394.054545455 | 20 | 110 | 0 | 0 |
| Path 494 | C00031->C00025:[1->2,4->1,4->3,4->8] | 0.80 | 400.259541985 | 25 | 131 | 0 | 0 |
| Path 495 | C00031->C00025:[4->1,4->3,7->1,7->3] | 0.40 | 216.920577617 | 27 | 277 | 0 | 2 |
| Path 496 | C00031->C00025:[1->2,4->1,7->3] | 0.60 | 437.068181818 | 22 | 132 | 0 | 0 |
| Path 497 | C00031->C00025:[1->2,4->1,5->5,9->8] | 0.80 | 372.62601626 | 23 | 123 | 0 | 0 |
| Path 498 | C00031->C00025:[1->2,1->8,4->1,4->3,4->5] | 1.00 | 466.926470588 | 23 | 136 | 0 | 0 |
| Path 499 | C00031->C00025:[7->3] | 0.20 | 375.990291262 | 12 | 103 | 0 | 0 |
| Path 500 | C00031->C00025:[1->2,4->1,7->5,9->8] | 0.80 | 342.551470588 | 18 | 136 | 0 | 0 |
| Path 501 | C00031->C00025:[4->1,4->3] | 0.40 | 229.3359375 | 28 | 256 | 0 | 2 |
| Path 502 | C00031->C00025:[4->1,4->3] | 0.40 | 218.104347826 | 18 | 230 | 0 | 0 |
| Path 503 | C00031->C00025:[4->3,4->8,7->1,7->5,9->2] | 1.00 | 389.069230769 | 26 | 130 | 0 | 0 |
| Path 504 | C00031->C00025:[1->2,4->1,7->3] | 0.60 | 375.846846847 | 18 | 111 | 0 | 0 |
| Path 505 | C00031->C00025:[4->3,5->1,7->5,9->2,9->8] | 1.00 | 478.099447514 | 28 | 181 | 0 | 0 |
| Path 506 | C00031->C00025:[1->2,4->1,5->5,9->8] | 0.80 | 386.677419355 | 21 | 124 | 0 | 0 |
| Path 507 | C00031->C00025:[1->2,4->1,4->3,4->5,4->8] | 1.00 | 390.211864407 | 22 | 118 | 0 | 0 |
| Path 508 | C00031->C00025:[4->1,4->3] | 0.40 | 215.155737705 | 19 | 244 | 0 | 2 |
| Path 509 | C00031->C00025:[4->1,4->3] | 0.40 | 210.403921569 | 19 | 255 | 0 | 0 |
| Path 510 | C00031->C00025:[4->1,4->3] | 0.40 | 215.490909091 | 25 | 275 | 0 | 2 |
| Path 511 | C00031->C00025:[4->3,7->1,7->3,9->2] | 0.60 | 372.34 | 24 | 150 | 0 | 0 |
| Path 512 | C00031->C00025:[4->1,4->3,7->1,7->3] | 0.40 | 260.269230769 | 15 | 182 | 0 | 0 |
| Path 513 | C00031->C00025:[4->1,4->3] | 0.40 | 589.407407407 | 16 | 27 | 0 | 0 |
| Path 514 | C00031->C00025:[1->2,4->1,5->5,9->8] | 0.80 | 380.918699187 | 20 | 123 | 0 | 0 |
| Path 515 | C00031->C00025:[4->1,4->3,4->8] | 0.60 | 244.687022901 | 31 | 262 | 0 | 3 |
| Path 516 | C00031->C00025:[7->3] | 0.20 | 421.875 | 8 | 16 | 0 | 0 |
| Path 517 | C00031->C00025:[4->3] | 0.20 | 569.133333333 | 9 | 15 | 0 | 0 |
| Path 518 | C00031->C00025:[4->1,4->3] | 0.40 | 215.410480349 | 17 | 229 | 0 | 2 |
| Path 519 | C00031->C00025:[4->3,7->1,7->5,9->2,9->8] | 1.00 | 373.568627451 | 25 | 153 | 0 | 0 |
| Path 520 | C00031->C00025:[4->1,4->3,7->1,7->3] | 0.40 | 205.692982456 | 14 | 228 | 0 | 0 |
| Path 521 | C00031->C00025:[4->1,4->3,4->8,7->1,7->3,7->8] | 0.60 | 244.115241636 | 33 | 269 | 0 | 3 |
| Path 522 | C00031->C00025:[1->5,4->1,4->2,4->3,4->8] | 1.00 | 265.713780919 | 28 | 283 | 0 | 2 |
| Path 523 | C00031->C00025:[1->2,4->1,4->3,4->8] | 0.80 | 409.87394958 | 24 | 119 | 0 | 0 |
| Path 524 | C00031->C00025:[4->3,5->1,5->5,9->2,9->8] | 1.00 | 386.076923077 | 29 | 182 | 0 | 0 |
| Path 525 | C00031->C00025:[1->2,4->1,4->3,4->8] | 0.80 | 404.760330579 | 24 | 121 | 0 | 0 |
| Path 526 | C00031->C00025:[4->1,4->3] | 0.40 | 227.236220472 | 27 | 254 | 0 | 2 |
| Path 527 | C00031->C00025:[1->2,4->1,4->3] | 0.60 | 400.885964912 | 21 | 114 | 0 | 0 |
| Path 528 | C00031->C00025:[4->3,5->1,9->2] | 0.60 | 415.681415929 | 21 | 113 | 0 | 0 |
| Path 529 | C00031->C00025:[4->3,4->8,7->1,7->5,9->2] | 1.00 | 372.235294118 | 27 | 136 | 0 | 0 |
| Path 530 | C00031->C00025:[1->2,4->1,7->5,9->8] | 0.80 | 390.509933775 | 22 | 151 | 0 | 0 |
| Path 531 | C00031->C00025:[4->3,4->8,7->1,7->5,9->2] | 1.00 | 388.2 | 25 | 130 | 0 | 0 |
| Path 532 | C00031->C00025:[1->2,4->1,5->5,9->8] | 0.80 | 372.44 | 23 | 125 | 0 | 0 |
| Path 533 | C00031->C00025:[1->2,4->1,4->3] | 0.60 | 441.11023622 | 24 | 127 | 0 | 0 |
| Path 534 | C00031->C00025:[1->2,4->1] | 0.40 | 383.490740741 | 17 | 108 | 0 | 0 |
| Path 535 | C00031->C00025:[4->3,7->1,9->2] | 0.60 | 409.47706422 | 19 | 109 | 0 | 0 |
| Path 536 | C00031->C00025:[7->3,7->5,9->8] | 0.60 | 210.125984252 | 21 | 254 | 0 | 2 |
| Path 537 | C00031->C00025:[4->3,7->1,9->2] | 0.60 | 359.633333333 | 22 | 150 | 0 | 0 |
| Path 538 | C00031->C00025:[1->2,4->1,4->3] | 0.60 | 398.556213018 | 28 | 169 | 0 | 0 |
| Path 539 | C00031->C00025:[4->3,7->1,7->5,9->2,9->8] | 1.00 | 402.066666667 | 22 | 120 | 0 | 0 |
| Path 540 | C00031->C00025:[7->1,7->5,9->2,9->8] | 0.80 | 423.764705882 | 22 | 136 | 0 | 0 |
| Path 541 | C00031->C00025:[1->2,4->1,5->5,9->8] | 0.80 | 381.398373984 | 23 | 123 | 0 | 0 |
| Path 542 | C00031->C00025:[1->2,4->1,4->3,7->5,9->8] | 1.00 | 484.980582524 | 30 | 206 | 0 | 0 |
| Path 543 | C00031->C00025:[4->3,5->1,5->5,9->2,9->8] | 1.00 | 358.947368421 | 22 | 152 | 0 | 0 |
| Path 544 | C00031->C00025:[1->2,4->1,4->3,4->8] | 0.80 | 409.181034483 | 24 | 116 | 0 | 0 |
| Path 545 | C00031->C00025:[4->3,7->1,7->5,9->2,9->8] | 1.00 | 408.389380531 | 21 | 113 | 0 | 0 |
| Path 546 | C00031->C00025:[4->1,4->3,4->8,7->2,9->5] | 1.00 | 247.919117647 | 34 | 272 | 0 | 3 |
| Path 547 | C00031->C00025:[4->3,7->1,9->2] | 0.60 | 521.095541401 | 23 | 157 | 0 | 0 |
| Path 548 | C00031->C00025:[1->2,4->1,5->5,9->8] | 0.80 | 368.409836066 | 22 | 122 | 0 | 0 |
| Path 549 | C00031->C00025:[1->2,4->1,7->3] | 0.60 | 395.628099174 | 19 | 121 | 0 | 0 |
| Path 550 | C00031->C00025:[4->3,7->1,9->2] | 0.60 | 366.306666667 | 22 | 150 | 0 | 0 |
| Path 551 | C00031->C00025:[4->1,4->3,4->8,7->1,7->3,7->8] | 0.60 | 481.691176471 | 32 | 68 | 0 | 1 |
| Path 552 | C00031->C00025:[4->3,7->1,7->5,9->2,9->8] | 1.00 | 335.549707602 | 24 | 171 | 0 | 0 |
| Path 553 | C00031->C00025:[4->3,5->1,7->5,9->2,9->8] | 1.00 | 328.609467456 | 24 | 169 | 0 | 0 |
| Path 554 | C00031->C00025:[4->1,4->3,4->8,7->1,7->2,7->3,7->8,9->5] | 1.00 | 248.132841328 | 35 | 271 | 0 | 3 |
| Path 555 | C00031->C00025:[4->3,7->1,7->5,9->2,9->8] | 1.00 | 390.944751381 | 28 | 181 | 0 | 0 |
| Path 556 | C00031->C00025:[4->1,4->3,4->8,7->2,9->5] | 1.00 | 244.01119403 | 32 | 268 | 0 | 3 |
| Path 557 | C00031->C00025:[1->2,4->1,7->5,9->8] | 0.80 | 339.080291971 | 22 | 137 | 0 | 0 |
| Path 558 | C00031->C00025:[4->1,4->3,7->1,7->3] | 0.40 | 497.657894737 | 21 | 38 | 0 | 0 |
| Path 559 | C00031->C00025:[4->3,4->8,7->3,7->8] | 0.40 | 381.708333333 | 19 | 120 | 0 | 0 |
| Path 560 | C00031->C00025:[4->1,4->3] | 0.40 | 216.823741007 | 26 | 278 | 0 | 2 |
| Path 561 | C00031->C00025:[1->2,4->1,4->3,7->5,9->8] | 1.00 | 360.394557823 | 23 | 147 | 0 | 0 |
| Path 562 | C00031->C00025:[1->2,4->1,5->5,9->8] | 0.80 | 368.367521368 | 21 | 117 | 0 | 0 |
| Path 563 | C00031->C00025:[4->3,7->1,7->5,9->2,9->8] | 1.00 | 416.173913043 | 23 | 115 | 0 | 0 |
| Path 564 | C00031->C00025:[4->1,4->3] | 0.40 | 233.65 | 30 | 260 | 0 | 2 |
| Path 565 | C00031->C00025:[7->1,7->3,7->5,9->2,9->8] | 1.00 | 413.595744681 | 23 | 141 | 0 | 0 |
| Path 566 | C00031->C00025:[5->1,7->5,9->2,9->8] | 0.80 | 361.880952381 | 20 | 126 | 0 | 0 |
| Path 567 | C00031->C00025:[5->1,5->5,9->2,9->8] | 0.80 | 387.205607477 | 16 | 107 | 0 | 0 |
| Path 568 | C00031->C00025:[5->1,7->5,9->2,9->8] | 0.80 | 356.016 | 19 | 125 | 0 | 0 |
| Path 569 | C00031->C00025:[4->3,5->1,5->5,9->2,9->8] | 1.00 | 424.715277778 | 30 | 144 | 0 | 0 |
| Path 570 | C00031->C00025:[4->1,4->3,7->5,9->2,9->8] | 1.00 | 246.416949153 | 37 | 295 | 0 | 3 |
| Path 571 | C00031->C00025:[1->2,4->1,4->3,4->8] | 0.80 | 400.626984127 | 25 | 126 | 0 | 0 |
| Path 572 | C00031->C00025:[4->1,4->3,7->1,7->3] | 0.40 | 219.816546763 | 28 | 278 | 0 | 2 |
| Path 573 | C00031->C00025:[4->1,4->3,4->8,7->2,9->5] | 1.00 | 256.235087719 | 42 | 285 | 0 | 3 |
| Path 574 | C00031->C00025:[4->1,4->3,4->8] | 0.60 | 511.526315789 | 28 | 57 | 0 | 1 |
| Path 575 | C00031->C00025:[4->3,4->8,7->1,7->3,7->5,7->8,9->2] | 1.00 | 386.512195122 | 22 | 123 | 0 | 0 |
| Path 576 | C00031->C00025:[4->1,4->3] | 0.40 | 218.489285714 | 28 | 280 | 0 | 2 |
| Path 577 | C00031->C00025:[1->2,4->1] | 0.40 | 387.469565217 | 16 | 115 | 0 | 0 |
| Path 578 | C00031->C00025:[4->1,4->3] | 0.40 | 207.208661417 | 18 | 254 | 0 | 0 |
| Path 579 | C00031->C00025:[4->1,4->3,7->1,7->3,7->5,9->2,9->8] | 1.00 | 246.608843537 | 38 | 294 | 0 | 3 |
| Path 580 | C00031->C00025:[1->2,4->1] | 0.40 | 394.094339623 | 17 | 106 | 0 | 0 |
| Path 581 | C00031->C00025:[4->1,4->3] | 0.40 | 257.206521739 | 27 | 276 | 0 | 2 |
| Path 582 | C00031->C00025:[7->1,7->3,7->5,9->2,9->8] | 1.00 | 361.923611111 | 25 | 144 | 0 | 0 |
| Path 583 | C00031->C00025:[1->2,4->1,4->3,7->5,9->8] | 1.00 | 367.836734694 | 28 | 196 | 0 | 0 |
| Path 584 | C00031->C00025:[1->2,4->1,5->5,9->8] | 0.80 | 356.39516129 | 22 | 124 | 0 | 0 |
| Path 585 | C00031->C00025:[4->2,4->3,4->8,7->2,7->3,7->8] | 0.60 | 391.260162602 | 21 | 123 | 0 | 0 |
| Path 586 | C00031->C00025:[4->3,5->1,5->5,7->1,7->5,9->2,9->8] | 1.00 | 367.423076923 | 26 | 156 | 0 | 0 |
| Path 587 | C00031->C00025:[7->1,7->5,9->2,9->8] | 0.80 | 407.657407407 | 19 | 108 | 0 | 0 |
| Path 588 | C00031->C00025:[1->2,4->1,4->3,4->5,4->8] | 1.00 | 395.975206612 | 22 | 121 | 0 | 0 |
| Path 589 | C00031->C00025:[1->2,1->8,4->1,4->3,4->5] | 1.00 | 398.058479532 | 23 | 171 | 0 | 0 |
| Path 590 | C00031->C00025:[5->1,9->2] | 0.40 | 396.980392157 | 15 | 102 | 0 | 0 |
| Path 591 | C00031->C00025:[4->1,4->3] | 0.40 | 219.314655172 | 18 | 232 | 0 | 2 |
| Path 592 | C00031->C00025:[4->3,5->1,5->5,9->2,9->8] | 1.00 | 387.115384615 | 32 | 182 | 0 | 0 |
| Path 593 | C00031->C00025:[4->3,7->1,7->5,9->2,9->8] | 1.00 | 469.774011299 | 24 | 177 | 0 | 0 |
| Path 594 | C00031->C00025:[1->2,4->1,4->3,4->8] | 0.80 | 399.76984127 | 22 | 126 | 0 | 0 |
| Path 595 | C00031->C00025:[4->3,5->1,7->5,9->2,9->8] | 1.00 | 474.672222222 | 27 | 180 | 0 | 0 |
| Path 596 | C00031->C00025:[4->1,4->3] | 0.40 | 275.190217391 | 19 | 184 | 0 | 0 |
| Path 597 | C00031->C00025:[4->3,4->8] | 0.40 | 404.192307692 | 15 | 104 | 0 | 0 |
| Path 598 | C00031->C00025:[4->1,4->3] | 0.40 | 228.623430962 | 24 | 239 | 0 | 2 |
| Path 599 | C00031->C00025:[4->1,4->3] | 0.40 | 214.149779736 | 17 | 227 | 0 | 0 |
| Path 600 | C00031->C00025:[1->2,4->1,4->3,4->5,4->8] | 1.00 | 384.590551181 | 23 | 127 | 0 | 0 |
| Path 601 | C00031->C00025:[4->3,7->1,7->5,9->2,9->8] | 1.00 | 394.372881356 | 20 | 118 | 0 | 0 |
| Path 602 | C00031->C00025:[4->1,4->3] | 0.40 | 259.384057971 | 26 | 276 | 0 | 2 |
| Path 603 | C00031->C00025:[4->3,7->5,9->8] | 0.60 | 337.575949367 | 18 | 158 | 0 | 0 |
| Path 604 | C00031->C00025:[1->2,4->1] | 0.40 | 381.263157895 | 15 | 114 | 0 | 0 |
| Path 605 | C00031->C00025:[4->3,7->1,9->2] | 0.60 | 406.873873874 | 19 | 111 | 0 | 0 |
| Path 606 | C00031->C00025:[4->1,4->3] | 0.40 | 260.386440678 | 29 | 295 | 0 | 2 |
| Path 607 | C00031->C00025:[4->1,4->3] | 0.40 | 212.55984556 | 23 | 259 | 0 | 2 |
| Path 608 | C00031->C00025:[4->3,7->1,7->5,9->2,9->8] | 1.00 | 341.338797814 | 29 | 183 | 0 | 0 |
| Path 609 | C00031->C00025:[1->2,4->1,4->3] | 0.60 | 392.982608696 | 21 | 115 | 0 | 0 |
| Path 610 | C00031->C00025:[1->2,1->8,4->1,4->5,7->3] | 1.00 | 395.71257485 | 22 | 167 | 0 | 0 |
| Path 611 | C00031->C00025:[4->3,7->5,9->8] | 0.60 | 218.038314176 | 23 | 261 | 0 | 2 |
| Path 612 | C00031->C00025:[7->1,9->2] | 0.40 | 393.841584158 | 14 | 101 | 0 | 0 |
| Path 613 | C00031->C00025:[1->2,4->1,4->3,4->5,4->8] | 1.00 | 387.6328125 | 23 | 128 | 0 | 0 |
| Path 614 | C00031->C00025:[4->3,7->1,9->2] | 0.60 | 403.805555556 | 18 | 108 | 0 | 0 |
| Path 615 | C00031->C00025:[1->2,4->1,4->3,7->5,9->8] | 1.00 | 480.029126214 | 33 | 206 | 0 | 0 |
| Path 616 | C00031->C00025:[4->1,4->3] | 0.40 | 222.591549296 | 30 | 284 | 0 | 2 |
| Path 617 | C00031->C00025:[5->1,7->3,9->2] | 0.60 | 345.721428571 | 17 | 140 | 0 | 0 |
| Path 618 | C00031->C00025:[4->3,5->1,5->5,9->2,9->8] | 1.00 | 395.420765027 | 30 | 183 | 0 | 0 |
| Path 619 | C00031->C00025:[1->8,4->3,4->5] | 0.60 | 422.564885496 | 24 | 131 | 0 | 0 |
| Path 620 | C00031->C00025:[4->1,4->3] | 0.40 | 219.114068441 | 25 | 263 | 0 | 2 |
| Path 621 | C00031->C00025:[1->2,4->1,4->3] | 0.60 | 389.355932203 | 26 | 177 | 0 | 0 |
| Path 622 | C00031->C00025:[1->2,4->1,4->3,4->8] | 0.80 | 389.459016393 | 24 | 122 | 0 | 0 |
| Path 623 | C00031->C00025:[4->3,7->1,7->5,9->2,9->8] | 1.00 | 417.925675676 | 25 | 148 | 0 | 0 |
| Path 624 | C00031->C00025:[4->3,7->1,7->3,9->2] | 0.60 | 360.281879195 | 23 | 149 | 0 | 0 |
| Path 625 | C00031->C00025:[4->1,4->3,4->8] | 0.60 | 251.421641791 | 36 | 268 | 0 | 3 |
| Path 626 | C00031->C00025:[1->2,4->1,7->3,7->5,9->8] | 1.00 | 356.601398601 | 22 | 143 | 0 | 0 |
| Path 627 | C00031->C00025:[7->5,9->8] | 0.40 | 397.301075269 | 12 | 93 | 0 | 0 |
| Path 628 | C00031->C00025:[4->1,4->3] | 0.40 | 598.416666667 | 15 | 24 | 0 | 0 |
| Path 629 | C00031->C00025:[4->1,4->3,4->8,7->2,9->5] | 1.00 | 242.346863469 | 33 | 271 | 0 | 3 |
| Path 630 | C00031->C00025:[1->2,4->1,5->5,9->8] | 0.80 | 377.495798319 | 21 | 119 | 0 | 0 |
| Path 631 | C00031->C00025:[1->2,4->1,4->3] | 0.60 | 442.869230769 | 25 | 130 | 0 | 0 |
| Path 632 | C00031->C00025:[4->3,4->8,7->1,7->5,9->2] | 1.00 | 388.303703704 | 25 | 135 | 0 | 0 |
| Path 633 | C00031->C00025:[1->2,4->1,4->3,7->3] | 0.60 | 391.5625 | 30 | 176 | 0 | 0 |
| Path 634 | C00031->C00025:[4->3,7->1,9->2] | 0.60 | 366.863945578 | 21 | 147 | 0 | 0 |
| Path 635 | C00031->C00025:[1->2,1->8,4->1,4->3,4->5] | 1.00 | 400.114942529 | 24 | 174 | 0 | 0 |
| Path 636 | C00031->C00025:[4->3,5->1,9->2] | 0.60 | 352.910958904 | 20 | 146 | 0 | 0 |
| Path 637 | C00031->C00025:[1->8,4->5,7->3] | 0.60 | 359.654929577 | 23 | 142 | 0 | 0 |
| Path 638 | C00031->C00025:[4->3,5->1,9->2] | 0.60 | 522.316455696 | 24 | 158 | 0 | 0 |
| Path 639 | C00031->C00025:[4->3,7->1,7->5,9->2,9->8] | 1.00 | 356.59602649 | 21 | 151 | 0 | 0 |
| Path 640 | C00031->C00025:[4->1,4->3] | 0.40 | 258.590443686 | 28 | 293 | 0 | 2 |
| Path 641 | C00031->C00025:[4->3,4->8] | 0.40 | 383.537735849 | 14 | 106 | 0 | 0 |
| Path 642 | C00031->C00025:[4->1,4->3] | 0.40 | 255.232472325 | 21 | 271 | 0 | 0 |
| Path 643 | C00031->C00025:[4->3,7->5,9->8] | 0.60 | 345.12962963 | 20 | 162 | 0 | 0 |
| Path 644 | C00031->C00025:[4->3,5->1,5->5,7->1,7->5,9->2,9->8] | 1.00 | 356.741935484 | 25 | 155 | 0 | 0 |
| Path 645 | C00031->C00025:[4->1,4->3] | 0.40 | 260.477011494 | 12 | 174 | 0 | 0 |
| Path 646 | C00031->C00025:[4->3,7->1,7->5,9->2,9->8] | 1.00 | 350.315789474 | 20 | 133 | 0 | 0 |
| Path 647 | C00031->C00025:[4->3,4->8,7->1,7->5,9->2] | 1.00 | 377.264957265 | 18 | 117 | 0 | 0 |
| Path 648 | C00031->C00025:[1->2,4->1,4->3,4->8] | 0.80 | 406.231404959 | 25 | 121 | 0 | 0 |
| Path 649 | C00031->C00025:[4->3,4->8] | 0.40 | 408.101851852 | 17 | 108 | 0 | 0 |
| Path 650 | C00031->C00025:[4->1,4->3,4->8,7->1,7->2,7->3,7->8,9->5] | 1.00 | 476.0 | 37 | 77 | 0 | 1 |
| Path 651 | C00031->C00025:[4->3,7->5,9->8] | 0.60 | 380.86013986 | 20 | 143 | 0 | 0 |
| Path 652 | C00031->C00025:[1->2,4->1,4->3,4->8] | 0.80 | 403.406779661 | 24 | 118 | 0 | 0 |
| Path 653 | C00031->C00025:[1->2,4->1,7->5,9->8] | 0.80 | 339.395683453 | 22 | 139 | 0 | 0 |
| Path 654 | C00031->C00025:[1->2,4->1,4->3,4->8] | 0.80 | 401.415254237 | 23 | 118 | 0 | 0 |
| Path 655 | C00031->C00025:[9->3] | 0.20 | 193.303643725 | 16 | 247 | 0 | 2 |
| Path 656 | C00031->C00025:[4->3,7->1,9->2] | 0.60 | 605.262711864 | 20 | 118 | 0 | 0 |
| Path 657 | C00031->C00025:[4->3,7->1,7->5,9->2,9->8] | 1.00 | 356.930232558 | 20 | 129 | 0 | 0 |
| Path 658 | C00031->C00025:[4->1,4->3,4->8] | 0.60 | 511.409836066 | 30 | 61 | 0 | 1 |
| Path 659 | C00031->C00025:[4->3,5->1,5->5,7->1,7->5,9->2,9->8] | 1.00 | 356.270967742 | 25 | 155 | 0 | 0 |
| Path 660 | C00031->C00025:[4->1,4->3] | 0.40 | 258.966507177 | 20 | 209 | 0 | 0 |
| Path 661 | C00031->C00025:[4->1,4->3] | 0.40 | 212.547445255 | 24 | 274 | 0 | 2 |
| Path 662 | C00031->C00025:[1->2,4->1,4->3,4->8] | 0.80 | 410.653225806 | 26 | 124 | 0 | 0 |
| Path 663 | C00031->C00025:[4->1,4->3,7->1,7->3] | 0.40 | 227.590038314 | 27 | 261 | 0 | 2 |
| Path 664 | C00031->C00025:[4->3,5->1,9->2] | 0.60 | 369.702702703 | 22 | 148 | 0 | 0 |
| Path 665 | C00031->C00025:[1->2,4->1,4->3] | 0.60 | 386.572254335 | 24 | 173 | 0 | 0 |
| Path 666 | C00031->C00025:[1->2,4->1,4->3,7->5,9->8] | 1.00 | 358.974358974 | 27 | 195 | 0 | 0 |
| Path 667 | C00031->C00025:[1->2,4->1,4->3,4->8] | 0.80 | 396.919354839 | 24 | 124 | 0 | 0 |
| Path 668 | C00031->C00025:[4->1,4->3] | 0.40 | 214.149122807 | 17 | 228 | 0 | 2 |
| Path 669 | C00031->C00025:[4->3,5->1,9->2] | 0.60 | 392.219298246 | 18 | 114 | 0 | 0 |
| Path 670 | C00031->C00025:[4->3,7->1,7->5,9->2,9->8] | 1.00 | 339.965116279 | 25 | 172 | 0 | 0 |
| Path 671 | C00031->C00025:[4->3,4->8,7->1,7->3,7->5,7->8,9->2] | 1.00 | 401.440677966 | 22 | 118 | 0 | 0 |
| Path 672 | C00031->C00025:[1->2,4->1,4->3,4->8] | 0.80 | 403.652173913 | 23 | 115 | 0 | 0 |
| Path 673 | C00031->C00025:[1->2,4->1,4->3,4->5,4->8] | 1.00 | 387.759398496 | 23 | 133 | 0 | 0 |
| Path 674 | C00031->C00025:[1->2,4->1,4->3,4->8] | 0.80 | 401.024793388 | 21 | 121 | 0 | 0 |
| Path 675 | C00031->C00025:[4->1,4->3,7->1,7->3] | 0.40 | 209.74025974 | 15 | 231 | 0 | 0 |
| Path 676 | C00031->C00025:[4->3,9->1] | 0.40 | 391.743119266 | 16 | 109 | 0 | 0 |
| Path 677 | C00031->C00025:[1->2,4->1,4->3,4->8] | 0.80 | 405.495934959 | 25 | 123 | 0 | 0 |
| Path 678 | C00031->C00025:[4->1,4->3,4->8,7->2,9->5] | 1.00 | 252.62633452 | 40 | 281 | 0 | 3 |
| Path 679 | C00031->C00025:[4->2,4->3,4->8,7->1,7->5,9->2,9->8] | 1.00 | 352.590277778 | 23 | 144 | 0 | 0 |
| Path 680 | C00031->C00025:[4->3,5->1,9->2] | 0.60 | 368.609271523 | 23 | 151 | 0 | 0 |
| Path 681 | C00031->C00025:[4->1,4->3,7->1,7->3,7->5,9->8] | 0.80 | 416.878787879 | 28 | 66 | 0 | 0 |
| Path 682 | C00031->C00025:[1->2,4->1,4->3] | 0.60 | 390.984 | 21 | 125 | 0 | 0 |
| Path 683 | C00031->C00025:[1->2,4->1,4->3,4->8] | 0.80 | 408.411764706 | 27 | 136 | 0 | 0 |
| Path 684 | C00031->C00025:[4->3,4->8,7->1,7->3,7->5,7->8,9->2] | 1.00 | 383.241666667 | 21 | 120 | 0 | 0 |
| Path 685 | C00031->C00025:[4->3,7->1,7->5,9->2,9->8] | 1.00 | 415.868965517 | 24 | 145 | 0 | 0 |
| Path 686 | C00031->C00025:[1->2,4->1,4->3,4->8] | 0.80 | 400.607142857 | 22 | 112 | 0 | 0 |
| Path 687 | C00031->C00025:[7->1,7->3,7->5,9->2,9->8] | 1.00 | 387.387387387 | 18 | 111 | 0 | 0 |
| Path 688 | C00031->C00025:[1->2,4->1] | 0.40 | 379.123809524 | 16 | 105 | 0 | 0 |
| Path 689 | C00031->C00025:[4->3,7->1,9->2] | 0.60 | 410.647540984 | 17 | 122 | 0 | 0 |
| Path 690 | C00031->C00025:[4->3,4->8,7->1,7->5,9->2] | 1.00 | 381.644628099 | 20 | 121 | 0 | 0 |
| Path 691 | C00031->C00025:[4->3,4->8,7->1,7->5,9->2] | 1.00 | 418.68503937 | 20 | 127 | 0 | 0 |
| Path 692 | C00031->C00025:[4->1,4->3,4->8,7->5,9->2,9->8] | 1.00 | 247.19047619 | 37 | 294 | 0 | 3 |
| Path 693 | C00031->C00025:[4->3,5->1,5->5,9->2,9->8] | 1.00 | 363.758169935 | 23 | 153 | 0 | 0 |
| Path 694 | C00031->C00025:[4->1,4->3] | 0.40 | 217.516245487 | 26 | 277 | 0 | 2 |
| Path 695 | C00031->C00025:[4->3,4->8,7->1,7->5,9->2] | 1.00 | 398.923728814 | 20 | 118 | 0 | 0 |
| Path 696 | C00031->C00025:[4->3,5->1,7->3,9->2] | 0.60 | 360.331081081 | 22 | 148 | 0 | 0 |
| Path 697 | C00031->C00025:[1->2,4->1,4->3] | 0.60 | 394.053097345 | 21 | 113 | 0 | 0 |
| Path 698 | C00031->C00025:[1->8,4->3,4->5] | 0.60 | 374.879432624 | 24 | 141 | 0 | 0 |
| Path 699 | C00031->C00025:[1->2,4->1,4->3] | 0.60 | 399.552 | 21 | 125 | 0 | 0 |
| Path 700 | C00031->C00025:[7->1,7->3] | 0.40 | 216.839662447 | 21 | 237 | 0 | 2 |
| Path 701 | C00031->C00025:[4->1,4->3] | 0.40 | 221.676156584 | 28 | 281 | 0 | 2 |
| Path 702 | C00031->C00025:[4->3,4->8,7->1,7->5,9->2] | 1.00 | 396.237037037 | 25 | 135 | 0 | 0 |
| Path 703 | C00031->C00025:[4->3,5->1,7->5,9->2,9->8] | 1.00 | 333.117647059 | 25 | 170 | 0 | 0 |
| Path 704 | C00031->C00025:[4->1,4->3,4->8,7->1,7->3,7->8] | 0.60 | 483.25 | 34 | 72 | 0 | 1 |
| Path 705 | C00031->C00025:[1->2,4->1,7->3] | 0.60 | 388.089285714 | 17 | 112 | 0 | 0 |
| Path 706 | C00031->C00025:[1->2,4->1,4->3] | 0.60 | 401.6171875 | 21 | 128 | 0 | 0 |
| Path 707 | C00031->C00025:[1->2,4->1,4->3] | 0.60 | 390.598130841 | 19 | 107 | 0 | 0 |
| Path 708 | C00031->C00025:[4->1,4->3,7->1,7->3] | 0.40 | 495.971428571 | 20 | 35 | 0 | 0 |
| Path 709 | C00031->C00025:[4->1,4->3] | 0.40 | 223.770212766 | 23 | 235 | 0 | 2 |
| Path 710 | C00031->C00025:[4->3,7->1,9->2] | 0.60 | 517.416666667 | 22 | 156 | 0 | 0 |
| Path 711 | C00031->C00025:[4->3,5->1,5->5,7->1,7->5,9->2,9->8] | 1.00 | 401.709401709 | 23 | 117 | 0 | 0 |
| Path 712 | C00031->C00025:[4->1,4->3,7->1,7->3] | 0.40 | 221.774319066 | 25 | 257 | 0 | 2 |
| Path 713 | C00031->C00025:[4->3,5->1,9->2] | 0.60 | 357.093333333 | 22 | 150 | 0 | 0 |
| Path 714 | C00031->C00025:[4->1,4->3] | 0.40 | 256.958762887 | 27 | 291 | 0 | 2 |
| Path 715 | C00031->C00025:[4->1,4->3,7->1,7->3] | 0.40 | 218.921146953 | 28 | 279 | 0 | 2 |
| Path 716 | C00031->C00025:[1->2,4->1] | 0.40 | 383.91509434 | 17 | 106 | 0 | 0 |
| Path 717 | C00031->C00025:[5->1,5->5,7->1,7->5,9->2,9->8] | 0.80 | 392.427272727 | 19 | 110 | 0 | 0 |
| Path 718 | C00031->C00025:[1->2,4->1,7->3] | 0.60 | 383.982248521 | 23 | 169 | 0 | 0 |
| Path 719 | C00031->C00025:[4->3,7->1,7->5,9->2,9->8] | 1.00 | 378.52739726 | 28 | 146 | 0 | 0 |
| Path 720 | C00031->C00025:[4->1,4->3,4->8,7->2,9->5] | 1.00 | 470.310810811 | 34 | 74 | 0 | 1 |
| Path 721 | C00031->C00025:[4->3,7->1,7->3,7->5,9->2,9->8] | 1.00 | 342.447674419 | 27 | 172 | 0 | 0 |
| Path 722 | C00031->C00025:[4->1,4->3] | 0.40 | 227.470588235 | 24 | 238 | 0 | 2 |
| Path 723 | C00031->C00025:[4->3,7->1,7->5,9->2,9->8] | 1.00 | 391.165217391 | 19 | 115 | 0 | 0 |
| Path 724 | C00031->C00025:[1->2,4->1,7->5,9->8] | 0.80 | 346.95620438 | 22 | 137 | 0 | 0 |
| Path 725 | C00031->C00025:[4->1,4->3] | 0.40 | 207.435555556 | 17 | 225 | 0 | 2 |
| Path 726 | C00031->C00025:[4->3,7->1,7->5,9->2,9->8] | 1.00 | 380.830508475 | 18 | 118 | 0 | 0 |
| Path 727 | C00031->C00025:[4->3,5->1,9->2] | 0.60 | 361.146666667 | 22 | 150 | 0 | 0 |
| Path 728 | C00031->C00025:[7->1,7->5,9->2,9->8] | 0.80 | 354.488721805 | 14 | 133 | 0 | 0 |
| Path 729 | C00031->C00025:[4->1,4->3,7->5,9->8] | 0.80 | 407.380952381 | 25 | 63 | 0 | 0 |
| Path 730 | C00031->C00025:[4->2,4->3,4->8,7->2,7->3,7->8] | 0.60 | 388.108333333 | 20 | 120 | 0 | 0 |
| Path 731 | C00031->C00025:[4->3,5->1,7->3,9->2] | 0.60 | 367.094594595 | 22 | 148 | 0 | 0 |
| Path 732 | C00031->C00025:[4->1,4->3,4->8,7->5,9->2,9->8] | 1.00 | 251.304054054 | 39 | 296 | 0 | 3 |
| Path 733 | C00031->C00025:[4->1,4->3] | 0.40 | 251.463414634 | 18 | 205 | 0 | 0 |
| Path 734 | C00031->C00025:[4->1,4->3,4->8,7->2,9->5] | 1.00 | 486.225352113 | 33 | 71 | 0 | 1 |
| Path 735 | C00031->C00025:[4->3] | 0.20 | 199.090909091 | 13 | 220 | 0 | 2 |
| Path 736 | C00031->C00025:[4->3,4->8,7->1,7->3,7->5,7->8,9->2,9->8] | 1.00 | 355.741496599 | 26 | 147 | 0 | 0 |
| Path 737 | C00031->C00025:[4->1,4->3] | 0.40 | 255.298076923 | 19 | 208 | 0 | 0 |
| Path 738 | C00031->C00025:[1->2,4->1,4->3,4->8] | 0.80 | 403.0 | 25 | 133 | 0 | 0 |
| Path 739 | C00031->C00025:[7->3,9->1] | 0.40 | 391.073394495 | 18 | 109 | 0 | 0 |
| Path 740 | C00031->C00025:[4->3,5->1,9->2] | 0.60 | 357.959183673 | 21 | 147 | 0 | 0 |
| Path 741 | C00031->C00025:[4->3,7->1,7->5,9->2,9->8] | 1.00 | 394.247787611 | 19 | 113 | 0 | 0 |
| Path 742 | C00031->C00025:[4->3] | 0.20 | 393.555555556 | 12 | 99 | 0 | 0 |
| Path 743 | C00031->C00025:[1->2,4->1,4->3,7->3] | 0.60 | 400.124293785 | 28 | 177 | 0 | 0 |
| Path 744 | C00031->C00025:[4->3,5->1,9->2] | 0.60 | 356.22147651 | 21 | 149 | 0 | 0 |
| Path 745 | C00031->C00025:[1->2,4->1,4->3] | 0.60 | 387.862275449 | 26 | 167 | 0 | 0 |
| Path 746 | C00031->C00025:[1->2,4->1] | 0.40 | 377.572727273 | 16 | 110 | 0 | 0 |
| Path 747 | C00031->C00025:[1->2,4->1,4->3,4->8] | 0.80 | 386.235294118 | 23 | 119 | 0 | 0 |
| Path 748 | C00031->C00025:[1->2,4->1,7->3] | 0.60 | 393.288135593 | 19 | 118 | 0 | 0 |
| Path 749 | C00031->C00025:[1->2,4->1,4->3] | 0.60 | 599.226027397 | 25 | 146 | 0 | 0 |
| Path 750 | C00031->C00025:[4->1,4->3,7->1,7->3,7->5,9->8] | 0.80 | 421.246376812 | 29 | 69 | 0 | 0 |
| Path 751 | C00031->C00025:[4->3,5->1,7->5,9->2,9->8] | 1.00 | 352.02238806 | 21 | 134 | 0 | 0 |
| Path 752 | C00031->C00025:[4->1,4->3] | 0.40 | 218.748201439 | 26 | 278 | 0 | 2 |
| Path 753 | C00031->C00025:[4->3,5->1,9->2] | 0.60 | 409.616071429 | 20 | 112 | 0 | 0 |
| Path 754 | C00031->C00025:[1->2,4->1,4->3] | 0.60 | 525.994565217 | 27 | 184 | 0 | 0 |
| Path 755 | C00031->C00025:[1->2,4->1,4->3,4->8] | 0.80 | 403.838709677 | 22 | 124 | 0 | 0 |
| Path 756 | C00031->C00025:[7->1,7->3] | 0.40 | 208.11372549 | 22 | 255 | 0 | 2 |
| Path 757 | C00031->C00025:[4->3,7->1,7->5,9->2,9->8] | 1.00 | 381.472222222 | 27 | 180 | 0 | 0 |
| Path 758 | C00031->C00025:[4->3,7->1,7->5,9->2,9->8] | 1.00 | 345.0 | 20 | 150 | 0 | 0 |
| Path 759 | C00031->C00025:[7->5,9->8] | 0.40 | 338.027522936 | 11 | 109 | 0 | 0 |
| Path 760 | C00031->C00025:[4->1,4->3] | 0.40 | 309.506666667 | 22 | 225 | 0 | 0 |
| Path 761 | C00031->C00025:[4->1,4->3] | 0.40 | 231.61627907 | 29 | 258 | 0 | 2 |
| Path 762 | C00031->C00025:[4->3,7->1,7->5,9->2,9->8] | 1.00 | 350.875 | 30 | 184 | 0 | 0 |
| Path 763 | C00031->C00025:[1->2,4->1,4->3] | 0.60 | 611.905109489 | 26 | 137 | 0 | 0 |
| Path 764 | C00031->C00025:[4->3,9->1] | 0.40 | 388.188679245 | 15 | 106 | 0 | 0 |
| Path 765 | C00031->C00025:[4->3,7->1,7->5,9->2,9->8] | 1.00 | 325.383233533 | 22 | 167 | 0 | 0 |
| Path 766 | C00031->C00025:[4->1,4->3] | 0.40 | 263.414715719 | 32 | 299 | 0 | 2 |
| Path 767 | C00031->C00025:[4->2,4->3,4->8,7->1,7->2,7->3,7->5,7->8,9->2,9->8] | 1.00 | 357.089041096 | 25 | 146 | 0 | 0 |
| Path 768 | C00031->C00025:[4->1,4->3] | 0.40 | 219.536290323 | 22 | 248 | 0 | 2 |
| Path 769 | C00031->C00025:[4->3,7->1,9->2] | 0.60 | 355.520547945 | 20 | 146 | 0 | 0 |
| Path 770 | C00031->C00025:[4->3,7->1,7->5,9->2,9->8] | 1.00 | 350.3125 | 23 | 144 | 0 | 0 |
| Path 771 | C00031->C00025:[7->3,7->5,9->8] | 0.60 | 217.75862069 | 25 | 261 | 0 | 2 |
| Path 772 | C00031->C00025:[4->3,4->8] | 0.40 | 388.127272727 | 16 | 110 | 0 | 0 |
| Path 773 | C00031->C00025:[1->2,4->1,7->3] | 0.60 | 374.916666667 | 25 | 168 | 0 | 0 |
| Path 774 | C00031->C00025:[4->3,5->1,7->5,9->2,9->8] | 1.00 | 343.426900585 | 26 | 171 | 0 | 0 |
| Path 775 | C00031->C00025:[4->3,4->8,7->1,7->5,9->2] | 1.00 | 389.378787879 | 26 | 132 | 0 | 0 |
| Path 776 | C00031->C00025:[4->1,4->3] | 0.40 | 210.245833333 | 17 | 240 | 0 | 2 |
| Path 777 | C00031->C00025:[4->1,4->3] | 0.40 | 218.817857143 | 27 | 280 | 0 | 2 |
| Path 778 | C00031->C00025:[4->1,4->3] | 0.40 | 203.501992032 | 17 | 251 | 0 | 0 |
| Path 779 | C00031->C00025:[1->2,4->1,4->3,4->8] | 0.80 | 395.105691057 | 25 | 123 | 0 | 0 |
| Path 780 | C00031->C00025:[4->3,5->1,7->3,7->5,9->2,9->8] | 1.00 | 341.169590643 | 26 | 171 | 0 | 0 |
| Path 781 | C00031->C00025:[4->1,4->3,7->1,7->3] | 0.40 | 219.984313725 | 23 | 255 | 0 | 2 |
| Path 782 | C00031->C00025:[7->3,7->5,9->8] | 0.60 | 377.54676259 | 19 | 139 | 0 | 0 |
| Path 783 | C00031->C00025:[4->3,7->5,9->8] | 0.60 | 393.201754386 | 18 | 114 | 0 | 0 |
| Path 784 | C00031->C00025:[1->2,4->1,4->3] | 0.60 | 390.983333333 | 21 | 120 | 0 | 0 |
| Path 785 | C00031->C00025:[4->3,7->1,7->5,9->2,9->8] | 1.00 | 320.746987952 | 21 | 166 | 0 | 0 |
| Path 786 | C00031->C00025:[1->2,4->1,4->3,4->8] | 0.80 | 406.153225806 | 26 | 124 | 0 | 0 |
| Path 787 | C00031->C00025:[7->3,7->5,9->8] | 0.60 | 395.162162162 | 18 | 111 | 0 | 0 |
| Path 788 | C00031->C00025:[1->2,4->1,4->3,7->5,9->8] | 1.00 | 365.575 | 33 | 200 | 0 | 0 |
| Path 789 | C00031->C00025:[4->3,4->8,7->3,7->8] | 0.40 | 399.881355932 | 20 | 118 | 0 | 0 |
| Path 790 | C00031->C00025:[4->3,7->1,7->5,9->2,9->8] | 1.00 | 342.595419847 | 18 | 131 | 0 | 0 |
| Path 791 | C00031->C00025:[4->3,5->1,9->2] | 0.60 | 518.668789809 | 23 | 157 | 0 | 0 |
| Path 792 | C00031->C00025:[4->3,4->8,7->1,7->5,9->2] | 1.00 | 418.694656489 | 22 | 131 | 0 | 0 |
| Path 793 | C00031->C00025:[4->3,7->1,7->5,9->2,9->8] | 1.00 | 377.269565217 | 17 | 115 | 0 | 0 |
| Path 794 | C00031->C00025:[1->2,4->1,4->3,4->8] | 0.80 | 400.527131783 | 26 | 129 | 0 | 0 |
| Path 795 | C00031->C00025:[4->3,5->1,7->5,9->2,9->8] | 1.00 | 355.642335766 | 22 | 137 | 0 | 0 |
| Path 796 | C00031->C00025:[4->3,7->5,9->8] | 0.60 | 401.686440678 | 20 | 118 | 0 | 0 |
| Path 797 | C00031->C00025:[4->1,4->3] | 0.40 | 221.212765957 | 24 | 235 | 0 | 2 |
| Path 798 | C00031->C00025:[7->3,7->5,9->8] | 0.60 | 393.168067227 | 17 | 119 | 0 | 0 |
| Path 799 | C00031->C00025:[4->3,4->8,7->1,7->5,9->2] | 1.00 | 386.759124088 | 28 | 137 | 0 | 0 |
| Path 800 | C00031->C00025:[4->3,5->1,5->5,7->1,7->5,9->2,9->8] | 1.00 | 406.966101695 | 24 | 118 | 0 | 0 |
| Path 801 | C00031->C00025:[4->3,7->1,7->5,9->2,9->8] | 1.00 | 367.178807947 | 23 | 151 | 0 | 0 |
| Path 802 | C00031->C00025:[7->3,7->5,9->8] | 0.60 | 372.876811594 | 18 | 138 | 0 | 0 |
| Path 803 | C00031->C00025:[4->1,4->3] | 0.40 | 223.084 | 24 | 250 | 0 | 2 |
| Path 804 | C00031->C00025:[1->2,4->1,4->3,4->8] | 0.80 | 396.811023622 | 23 | 127 | 0 | 0 |
| Path 805 | C00031->C00025:[4->3,5->1,9->2] | 0.60 | 361.98013245 | 23 | 151 | 0 | 0 |
| Path 806 | C00031->C00025:[4->1,4->3,7->1,7->3] | 0.40 | 260.62585034 | 30 | 294 | 0 | 2 |
| Path 807 | C00031->C00025:[1->2,4->1] | 0.40 | 379.61 | 15 | 100 | 0 | 0 |
| Path 808 | C00031->C00025:[4->3,7->1,9->2] | 0.60 | 609.378151261 | 21 | 119 | 0 | 0 |
| Path 809 | C00031->C00025:[1->2,4->1,7->5,9->8] | 0.80 | 339.159090909 | 20 | 132 | 0 | 0 |
| Path 810 | C00031->C00025:[9->1,9->3] | 0.40 | 347.84 | 17 | 125 | 0 | 0 |
| Path 811 | C00031->C00025:[4->1,4->3] | 0.40 | 221.748 | 23 | 250 | 0 | 2 |
| Path 812 | C00031->C00025:[1->2,4->1,4->3,7->3] | 0.60 | 390.488636364 | 27 | 176 | 0 | 0 |
| Path 813 | C00031->C00025:[4->1,4->3] | 0.40 | 217.43902439 | 20 | 246 | 0 | 2 |
| Path 814 | C00031->C00025:[1->2,4->1,4->3,4->8] | 0.80 | 403.351145038 | 28 | 131 | 0 | 0 |
| Path 815 | C00031->C00025:[4->1,4->3,7->1,7->3] | 0.40 | 513.142857143 | 19 | 35 | 0 | 0 |
| Path 816 | C00031->C00025:[1->2,4->1,7->5,9->8] | 0.80 | 383.754966887 | 25 | 151 | 0 | 0 |
| Path 817 | C00031->C00025:[4->1,4->3] | 0.40 | 227.598425197 | 26 | 254 | 0 | 2 |
| Path 818 | C00031->C00025:[4->1,4->3] | 0.40 | 226.57312253 | 25 | 253 | 0 | 2 |
| Path 819 | C00031->C00025:[4->1,4->3] | 0.40 | 576.75 | 18 | 32 | 0 | 0 |
| Path 820 | C00031->C00025:[4->1,4->3] | 0.40 | 223.48409894 | 30 | 283 | 0 | 2 |
| Path 821 | C00031->C00025:[4->3,7->1,7->5,9->2,9->8] | 1.00 | 355.653333333 | 22 | 150 | 0 | 0 |
| Path 822 | C00031->C00025:[4->1,4->3,7->1,7->3] | 0.40 | 217.584980237 | 22 | 253 | 0 | 2 |
| Path 823 | C00031->C00025:[4->1,4->3] | 0.40 | 221.348754448 | 29 | 281 | 0 | 2 |
| Path 824 | C00031->C00025:[1->2,4->1,7->3] | 0.60 | 425.847328244 | 24 | 131 | 0 | 0 |
| Path 825 | C00031->C00025:[7->1,7->5,9->2,9->8] | 0.80 | 354.217741935 | 18 | 124 | 0 | 0 |
| Path 826 | C00031->C00025:[1->2,4->1,4->3,4->8] | 0.80 | 397.049180328 | 23 | 122 | 0 | 0 |
| Path 827 | C00031->C00025:[4->1,4->3] | 0.40 | 216.492805755 | 27 | 278 | 0 | 2 |
| Path 828 | C00031->C00025:[1->2,4->1,4->3,7->5,9->8] | 1.00 | 370.675 | 30 | 200 | 0 | 0 |
| Path 829 | C00031->C00025:[7->1,7->3] | 0.40 | 211.29296875 | 23 | 256 | 0 | 2 |
| Path 830 | C00031->C00025:[4->1,4->3] | 0.40 | 271.17679558 | 18 | 181 | 0 | 0 |
| Path 831 | C00031->C00025:[1->2,4->1,4->3,4->8] | 0.80 | 399.936 | 24 | 125 | 0 | 0 |
| Path 832 | C00031->C00025:[7->3,7->5,9->8] | 0.60 | 337.903225806 | 18 | 155 | 0 | 0 |
| Path 833 | C00031->C00025:[4->3,7->1,7->3,7->5,9->2,9->8] | 1.00 | 336.405882353 | 25 | 170 | 0 | 0 |
| Path 834 | C00031->C00025:[4->3,7->1,7->5,9->2,9->8] | 1.00 | 473.286516854 | 25 | 178 | 0 | 0 |
| Path 835 | C00031->C00025:[4->3,4->8,7->1,7->3,7->5,7->8,9->2,9->8] | 1.00 | 352.375 | 25 | 144 | 0 | 0 |
| Path 836 | C00031->C00025:[7->1,7->3,7->5,9->2,9->8] | 1.00 | 372.990990991 | 16 | 111 | 0 | 0 |
| Path 837 | C00031->C00025:[1->2,4->1] | 0.40 | 373.935779817 | 16 | 109 | 0 | 0 |
| Path 838 | C00031->C00025:[4->1,4->3,4->8,7->1,7->2,7->3,7->8,9->5] | 1.00 | 246.441605839 | 36 | 274 | 0 | 3 |
| Path 839 | C00031->C00025:[4->3,7->5,9->8] | 0.60 | 340.925465839 | 19 | 161 | 0 | 0 |
| Path 840 | C00031->C00025:[4->3,7->1,9->2] | 0.60 | 412.366071429 | 20 | 112 | 0 | 0 |
| Path 841 | C00031->C00025:[1->2,4->1,4->3,4->8] | 0.80 | 409.900763359 | 26 | 131 | 0 | 0 |
| Path 842 | C00031->C00025:[4->1,4->3] | 0.40 | 210.239382239 | 24 | 259 | 0 | 2 |
| Path 843 | C00031->C00025:[4->3,5->1,5->5,7->1,7->5,9->2,9->8] | 1.00 | 367.891025641 | 26 | 156 | 0 | 0 |
| Path 844 | C00031->C00025:[4->3,7->1,9->2] | 0.60 | 520.630573248 | 23 | 157 | 0 | 0 |
| Path 845 | C00031->C00025:[4->3,7->5,9->8] | 0.60 | 396.512195122 | 18 | 123 | 0 | 0 |
| Path 846 | C00031->C00025:[4->3,5->1,5->5,7->1,7->5,9->2,9->8] | 1.00 | 363.2 | 25 | 155 | 0 | 0 |
| Path 847 | C00031->C00025:[1->2,4->1,4->3,4->8] | 0.80 | 407.537190083 | 25 | 121 | 0 | 0 |
| Path 848 | C00031->C00025:[4->3,5->1,9->2] | 0.60 | 373.388157895 | 24 | 152 | 0 | 0 |
| Path 849 | C00031->C00025:[4->1,4->3,4->8] | 0.60 | 255.220588235 | 38 | 272 | 0 | 3 |
| Path 850 | C00031->C00025:[4->1,4->3] | 0.40 | 586.321428571 | 16 | 28 | 0 | 0 |
| Path 851 | C00031->C00025:[1->2,4->1,5->5,9->8] | 0.80 | 381.406779661 | 19 | 118 | 0 | 0 |
| Path 852 | C00031->C00025:[4->1,4->3] | 0.40 | 255.970760234 | 11 | 171 | 0 | 0 |
| Path 853 | C00031->C00025:[1->2,4->1,4->3,4->8] | 0.80 | 402.831858407 | 22 | 113 | 0 | 0 |
| Path 854 | C00031->C00025:[4->3,7->1,9->2] | 0.60 | 350.420689655 | 19 | 145 | 0 | 0 |
| Path 855 | C00031->C00025:[1->2,4->1,4->3] | 0.60 | 385.493902439 | 25 | 164 | 0 | 0 |
| Path 856 | C00031->C00025:[5->1,9->2] | 0.40 | 403.757281553 | 16 | 103 | 0 | 0 |
| Path 857 | C00031->C00025:[4->3] | 0.20 | 582.083333333 | 8 | 12 | 0 | 0 |
| Path 858 | C00031->C00025:[4->1,4->3,7->5,9->8] | 0.80 | 412.378787879 | 26 | 66 | 0 | 0 |
| Path 859 | C00031->C00025:[4->1,4->3,7->1,7->3] | 0.40 | 226.187739464 | 27 | 261 | 0 | 2 |
| Path 860 | C00031->C00025:[4->3,7->1,9->2] | 0.60 | 410.146788991 | 19 | 109 | 0 | 0 |
| Path 861 | C00031->C00025:[1->2,4->1,7->3] | 0.60 | 378.277310924 | 21 | 119 | 0 | 0 |
| Path 862 | C00031->C00025:[1->2,4->1,4->3,4->8] | 0.80 | 390.25203252 | 25 | 123 | 0 | 0 |
| Path 863 | C00031->C00025:[7->3,7->5,9->8] | 0.60 | 398.408333333 | 18 | 120 | 0 | 0 |
| Path 864 | C00031->C00025:[4->1,4->3] | 0.40 | 220.65248227 | 29 | 282 | 0 | 2 |
| Path 865 | C00031->C00025:[4->1,4->3] | 0.40 | 215.848375451 | 25 | 277 | 0 | 2 |
| Path 866 | C00031->C00025:[1->2,4->1,4->3,4->8] | 0.80 | 406.893442623 | 26 | 122 | 0 | 0 |
| Path 867 | C00031->C00025:[1->2,4->1,4->3] | 0.60 | 395.860606061 | 26 | 165 | 0 | 0 |
| Path 868 | C00031->C00025:[1->2,4->1,4->3,4->8] | 0.80 | 405.495798319 | 24 | 119 | 0 | 0 |
| Path 869 | C00031->C00025:[4->1,4->3,4->8,7->2,9->5] | 1.00 | 484.820895522 | 31 | 67 | 0 | 1 |
| Path 870 | C00031->C00025:[4->3,5->1,9->2] | 0.60 | 606.176470588 | 21 | 119 | 0 | 0 |
| Path 871 | C00031->C00025:[4->3,4->8] | 0.40 | 407.364485981 | 16 | 107 | 0 | 0 |
| Path 872 | C00031->C00025:[4->1,4->3,7->1,7->3] | 0.40 | 221.789285714 | 29 | 280 | 0 | 2 |
