# Supplementary material for: 13C-metabolic flux ratio and novel carbon path analyses confirmed that Trichoderma reesei uses primarily the respirative pathway also on the preferred carbon source glucose
Source: BMC Syst Biol. 2009 Oct 29;3:104. doi: 10.1186/1752-0509-3-104 (PMC2776023; doi:10.1186/1752-0509-3-104)
Supplement: Additional file 1 — Pathways discovered in ReTrace carbon path analysis. Graphical and tabular representations of amino acid synthesis pathways discovered in ReTrace carbon path analysis [21]. Self-contained web site: unpack zip archive and open index.html with a web browser. [file 1752-0509-3-104-S1.zip › AF1-treesei/pathways-C00024-to-C00031.html]

Pathways from C00024 to C00031


**Pathways from C00024 to C00031**

**Sources:** Acetyl-CoA; (C00024)

**Target:**D-Glucose; (C00031)

|  | Composite mapping | Z | Average score | Rpairs | Reactions | Zero scores | Scores under threshold |
| --- | --- | --- | --- | --- | --- | --- | --- |
| Path 1 | C00024->C00031:[10->1,11->2,15->4,49->9,50->7,6->5] | 1.00 | 315.25477707 | 17 | 157 | 0 | 0 |
| Path 2 | C00024->C00031:[49->1,49->2,49->9,50->4,50->5,50->7] | 1.00 | 338.431818182 | 27 | 88 | 0 | 1 |
| Path 3 | C00024->C00031:[49->1,49->2,49->9,50->4,50->5,50->7] | 1.00 | 488.72972973 | 28 | 74 | 0 | 1 |
| Path 4 | C00024->C00031:[10->1,11->2,15->4,49->9,50->7,6->5] | 1.00 | 338.065217391 | 15 | 138 | 0 | 0 |
| Path 5 | C00024->C00031:[10->1,11->2,15->4,49->9,50->7,6->5] | 1.00 | 324.573099415 | 24 | 171 | 0 | 0 |
| Path 6 | C00024->C00031:[10->1,11->2,15->4,49->9,50->7,6->5] | 1.00 | 327.955555556 | 14 | 135 | 0 | 0 |
| Path 7 | C00024->C00031:[49->1,49->2,49->9,50->4,50->5,50->7] | 1.00 | 425.1875 | 29 | 96 | 0 | 1 |
| Path 8 | C00024->C00031:[10->1,11->2,15->4,49->9,50->7,6->5] | 1.00 | 336.082089552 | 13 | 134 | 0 | 0 |
| Path 9 | C00024->C00031:[10->1,11->2,15->4,49->9,50->7,6->5] | 1.00 | 297.562091503 | 13 | 153 | 0 | 0 |
| Path 10 | C00024->C00031:[49->1,49->2,49->9,50->4,50->5,50->7] | 1.00 | 503.071428571 | 28 | 70 | 0 | 1 |
| Path 11 | C00024->C00031:[49->1,49->2,49->9,50->4,50->5,50->7] | 1.00 | 407.589473684 | 28 | 95 | 0 | 1 |
| Path 12 | C00024->C00031:[49->1,49->2,49->9,50->4,50->5,50->7] | 1.00 | 359.395833333 | 32 | 96 | 0 | 1 |
| Path 13 | C00024->C00031:[10->1,11->2,15->4,49->9,50->7,6->5] | 1.00 | 342.414814815 | 17 | 135 | 0 | 0 |
| Path 14 | C00024->C00031:[10->1,11->2,15->4,49->9,50->7,6->5] | 1.00 | 294.644859813 | 18 | 107 | 0 | 0 |
| Path 15 | C00024->C00031:[10->1,11->2,15->4,49->9,50->7,6->5] | 1.00 | 336.03649635 | 17 | 137 | 0 | 0 |
| Path 16 | C00024->C00031:[49->1,49->2,49->9,50->4,50->5,50->7] | 1.00 | 350.322580645 | 29 | 93 | 0 | 1 |
| Path 17 | C00024->C00031:[10->1,11->2,15->4,49->9,50->7,6->5] | 1.00 | 306.515021459 | 26 | 233 | 0 | 0 |
| Path 18 | C00024->C00031:[10->1,11->2,15->4,49->9,50->7,6->5] | 1.00 | 320.251572327 | 18 | 159 | 0 | 0 |
| Path 19 | C00024->C00031:[49->1,49->2,49->9,50->4,50->5,50->7] | 1.00 | 352.477777778 | 29 | 90 | 0 | 1 |
| Path 20 | C00024->C00031:[49->1,49->2,49->9,50->4,50->5,50->7] | 1.00 | 495.014285714 | 28 | 70 | 0 | 1 |
| Path 21 | C00024->C00031:[10->1,11->2,15->4,49->9,50->7,6->5] | 1.00 | 303.0 | 25 | 231 | 0 | 0 |
| Path 22 | C00024->C00031:[49->1,49->2,49->9,50->4,50->5,50->7] | 1.00 | 387.288659794 | 28 | 97 | 0 | 1 |
| Path 23 | C00024->C00031:[49->1,49->2,49->9,50->4,50->5,50->7] | 1.00 | 415.343434343 | 29 | 99 | 0 | 1 |
| Path 24 | C00024->C00031:[10->1,11->2,15->4,49->9,50->7,6->5] | 1.00 | 338.323943662 | 17 | 142 | 0 | 0 |
| Path 25 | C00024->C00031:[10->1,11->2,15->4,49->9,50->7,6->5] | 1.00 | 300.229813665 | 17 | 161 | 0 | 0 |
| Path 26 | C00024->C00031:[10->1,11->2,15->4,49->9,50->7,6->5] | 1.00 | 312.912790698 | 23 | 172 | 0 | 0 |
| Path 27 | C00024->C00031:[49->1,49->2,49->9,50->4,50->5,50->7] | 1.00 | 347.731182796 | 29 | 93 | 0 | 1 |
| Path 28 | C00024->C00031:[10->1,11->2,15->4,2->5,49->9,50->7] | 1.00 | 328.179012346 | 21 | 162 | 0 | 0 |
| Path 29 | C00024->C00031:[49->1,49->2,49->9,50->4,50->5,50->7] | 1.00 | 377.923913043 | 26 | 92 | 0 | 1 |
| Path 30 | C00024->C00031:[10->1,11->2,15->4,49->9,50->7,6->5] | 1.00 | 350.566176471 | 18 | 136 | 0 | 0 |
| Path 31 | C00024->C00031:[10->1,11->2,15->4,49->9,50->7,6->5] | 1.00 | 333.007092199 | 15 | 141 | 0 | 0 |
| Path 32 | C00024->C00031:[49->1,49->2,49->9,50->4,50->5,50->7] | 1.00 | 408.446601942 | 29 | 103 | 0 | 1 |
| Path 33 | C00024->C00031:[10->1,11->2,15->4,49->9,50->7,6->5] | 1.00 | 305.288343558 | 18 | 163 | 0 | 0 |
| Path 34 | C00024->C00031:[10->1,11->2,15->4,49->9,50->7,6->5] | 1.00 | 298.021367521 | 25 | 234 | 0 | 0 |
| Path 35 | C00024->C00031:[10->1,11->2,15->4,49->9,50->7,6->5] | 1.00 | 324.409090909 | 25 | 176 | 0 | 0 |
| Path 36 | C00024->C00031:[10->1,11->2,15->4,49->9,50->7,6->5] | 1.00 | 278.771428571 | 16 | 105 | 0 | 0 |
| Path 37 | C00024->C00031:[49->1,49->2,49->9,50->4,50->5,50->7] | 1.00 | 507.816901408 | 29 | 71 | 0 | 1 |
| Path 38 | C00024->C00031:[49->1,49->2,49->9,50->4,50->5,50->7] | 1.00 | 336.415730337 | 28 | 89 | 0 | 1 |
| Path 39 | C00024->C00031:[10->1,11->2,15->4,49->9,50->7,6->5] | 1.00 | 341.528571429 | 16 | 140 | 0 | 0 |
| Path 40 | C00024->C00031:[49->1,49->2,49->7,49->9,50->4,50->5] | 1.00 | 428.133333333 | 17 | 60 | 0 | 1 |
| Path 41 | C00024->C00031:[10->1,11->2,15->4,49->9,50->7,6->5] | 1.00 | 309.961783439 | 16 | 157 | 0 | 0 |
| Path 42 | C00024->C00031:[49->1,49->2,49->9,50->4,50->5,50->7] | 1.00 | 497.53030303 | 26 | 66 | 0 | 1 |
| Path 43 | C00024->C00031:[10->1,11->2,15->4,49->9,50->7,6->5] | 1.00 | 343.460431655 | 17 | 139 | 0 | 0 |
| Path 44 | C00024->C00031:[49->1,49->2,49->9,50->4,50->5,50->7] | 1.00 | 408.826530612 | 29 | 98 | 0 | 1 |
| Path 45 | C00024->C00031:[49->1,49->2,49->9,50->4,50->5,50->7] | 1.00 | 375.569892473 | 27 | 93 | 0 | 1 |
| Path 46 | C00024->C00031:[10->1,11->2,15->4,49->9,50->7,6->5] | 1.00 | 303.43697479 | 24 | 119 | 0 | 0 |
| Path 47 | C00024->C00031:[10->1,11->2,15->4,49->9,50->7,6->5] | 1.00 | 341.239130435 | 17 | 138 | 0 | 0 |
| Path 48 | C00024->C00031:[49->1,49->2,49->9,50->4,50->5,50->7] | 1.00 | 508.478873239 | 29 | 71 | 0 | 1 |
| Path 49 | C00024->C00031:[10->1,11->2,15->4,49->9,50->7,6->5] | 1.00 | 319.982248521 | 23 | 169 | 0 | 0 |
| Path 50 | C00024->C00031:[49->1,49->2,49->9,50->4,50->5,50->7] | 1.00 | 396.446808511 | 28 | 94 | 0 | 1 |
| Path 51 | C00024->C00031:[49->1,49->2,49->9,50->4,50->5,50->7] | 1.00 | 433.051020408 | 31 | 98 | 0 | 1 |
| Path 52 | C00024->C00031:[49->1,49->2,49->9,50->4,50->5,50->7] | 1.00 | 380.947368421 | 29 | 95 | 0 | 1 |
| Path 53 | C00024->C00031:[49->1,49->2,49->9,50->4,50->5,50->7] | 1.00 | 339.123595506 | 28 | 89 | 0 | 1 |
| Path 54 | C00024->C00031:[10->1,11->2,15->4,49->9,50->7,6->5] | 1.00 | 333.058823529 | 15 | 136 | 0 | 0 |
| Path 55 | C00024->C00031:[10->1,11->2,15->4,49->9,50->7,6->5] | 1.00 | 325.28057554 | 14 | 139 | 0 | 0 |
| Path 56 | C00024->C00031:[49->1,49->2,49->9,50->4,50->5,50->7] | 1.00 | 342.89010989 | 30 | 91 | 0 | 1 |
| Path 57 | C00024->C00031:[49->1,49->2,49->9,50->4,50->5,50->7] | 1.00 | 394.89 | 31 | 100 | 0 | 1 |
| Path 58 | C00024->C00031:[49->1,49->2,49->9,50->4,50->5,50->7] | 1.00 | 358.655555556 | 29 | 90 | 0 | 1 |
| Path 59 | C00024->C00031:[10->1,11->2,15->4,49->9,50->7,6->5] | 1.00 | 317.505747126 | 24 | 174 | 0 | 0 |
| Path 60 | C00024->C00031:[49->1,49->2,49->9,50->4,50->5,50->7] | 1.00 | 429.948453608 | 30 | 97 | 0 | 1 |
| Path 61 | C00024->C00031:[10->1,11->2,15->4,49->9,50->7,6->5] | 1.00 | 346.592592593 | 17 | 135 | 0 | 0 |
| Path 62 | C00024->C00031:[49->1,49->2,49->9,50->4,50->5,50->7] | 1.00 | 384.804123711 | 28 | 97 | 0 | 1 |
| Path 63 | C00024->C00031:[10->1,11->2,15->4,49->9,50->7,6->5] | 1.00 | 303.334745763 | 26 | 236 | 0 | 0 |
| Path 64 | C00024->C00031:[49->1,49->2,49->9,50->4,50->5,50->7] | 1.00 | 340.241758242 | 30 | 91 | 0 | 1 |
| Path 65 | C00024->C00031:[10->1,11->2,15->4,49->9,50->7,6->5] | 1.00 | 319.948275862 | 24 | 174 | 0 | 0 |
| Path 66 | C00024->C00031:[49->1,49->2,49->9,50->4,50->5,50->7] | 1.00 | 417.69 | 29 | 100 | 0 | 1 |
| Path 67 | C00024->C00031:[49->1,49->2,49->9,50->4,50->5,50->7] | 1.00 | 486.520547945 | 28 | 73 | 0 | 1 |
| Path 68 | C00024->C00031:[49->1,49->2,49->9,50->4,50->5,50->7] | 1.00 | 478.638888889 | 28 | 72 | 0 | 1 |
| Path 69 | C00024->C00031:[10->1,11->2,15->4,49->9,50->7,6->5] | 1.00 | 320.231884058 | 13 | 138 | 0 | 0 |
| Path 70 | C00024->C00031:[10->1,11->2,15->4,2->9,49->7,6->5] | 1.00 | 345.342105263 | 18 | 152 | 0 | 0 |
| Path 71 | C00024->C00031:[49->1,49->2,49->9,50->4,50->5,50->7] | 1.00 | 378.161290323 | 27 | 93 | 0 | 1 |
| Path 72 | C00024->C00031:[49->1,49->2,49->9,50->4,50->5,50->7] | 1.00 | 355.977777778 | 29 | 90 | 0 | 1 |
| Path 73 | C00024->C00031:[49->1,49->2,49->9,50->4,50->5,50->7] | 1.00 | 393.882978723 | 28 | 94 | 0 | 1 |
| Path 74 | C00024->C00031:[10->1,11->2,15->4,49->9,50->7,6->5] | 1.00 | 328.085714286 | 14 | 140 | 0 | 0 |
| Path 75 | C00024->C00031:[49->1,49->2,49->9,50->4,50->5,50->7] | 1.00 | 473.597402597 | 28 | 77 | 0 | 1 |
| Path 76 | C00024->C00031:[10->1,11->2,15->4,49->9,50->7,6->5] | 1.00 | 333.072992701 | 14 | 137 | 0 | 0 |
| Path 77 | C00024->C00031:[10->1,11->2,15->4,49->9,50->7,6->5] | 1.00 | 273.594594595 | 18 | 111 | 0 | 0 |
| Path 78 | C00024->C00031:[10->1,11->2,15->4,49->9,50->7,6->5] | 1.00 | 304.767741935 | 15 | 155 | 0 | 0 |
| Path 79 | C00024->C00031:[10->1,11->2,15->4,49->9,50->7,6->5] | 1.00 | 333.463235294 | 16 | 136 | 0 | 0 |
| Path 80 | C00024->C00031:[49->1,49->2,49->9,50->4,50->5,50->7] | 1.00 | 378.410526316 | 29 | 95 | 0 | 1 |
| Path 81 | C00024->C00031:[10->1,11->2,15->4,49->9,50->7,6->5] | 1.00 | 350.220588235 | 18 | 136 | 0 | 0 |
| Path 82 | C00024->C00031:[49->1,49->2,49->9,50->4,50->5,50->7] | 1.00 | 390.531914894 | 28 | 94 | 0 | 1 |
| Path 83 | C00024->C00031:[49->1,49->2,49->9,50->4,50->5,50->7] | 1.00 | 419.3125 | 29 | 96 | 0 | 1 |
| Path 84 | C00024->C00031:[49->9,50->4,50->7] | 0.50 | 501.571428571 | 18 | 49 | 0 | 0 |
| Path 85 | C00024->C00031:[49->1,49->2,50->4] | 0.50 | 397.714285714 | 18 | 77 | 0 | 1 |
| Path 86 | C00024->C00031:[49->1,49->9,50->4,50->7] | 0.67 | 354.315789474 | 17 | 76 | 0 | 1 |
| Path 87 | C00024->C00031:[49->1,49->2,49->9,50->4,50->7] | 0.83 | 393.0 | 21 | 62 | 0 | 1 |
| Path 88 | C00024->C00031:[2->5,49->9,50->7] | 0.50 | 315.864197531 | 19 | 162 | 0 | 0 |
| Path 89 | C00024->C00031:[49->9,50->7] | 0.33 | 595.625 | 11 | 16 | 0 | 0 |
| Path 90 | C00024->C00031:[49->4,49->9,50->7] | 0.50 | 525.28125 | 19 | 32 | 0 | 1 |
| Path 91 | C00024->C00031:[49->4] | 0.17 | 617.5 | 14 | 22 | 0 | 1 |
| Path 92 | C00024->C00031:[49->1,49->2,49->9,50->4,50->7] | 0.83 | 388.432835821 | 23 | 67 | 0 | 1 |
| Path 93 | C00024->C00031:[49->4,49->9,50->7] | 0.50 | 440.181818182 | 20 | 44 | 0 | 1 |
| Path 94 | C00024->C00031:[49->1,49->9,50->4,50->7] | 0.67 | 512.850746269 | 23 | 67 | 0 | 1 |
| Path 95 | C00024->C00031:[49->1,49->2,49->9,50->4,50->5,50->7] | 1.00 | 515.363636364 | 27 | 66 | 0 | 1 |
| Path 96 | C00024->C00031:[2->5,49->9,50->7] | 0.50 | 322.079754601 | 21 | 163 | 0 | 0 |
| Path 97 | C00024->C00031:[49->1,49->2,50->4] | 0.50 | 486.352941176 | 20 | 68 | 0 | 1 |
| Path 98 | C00024->C00031:[49->9,50->7] | 0.33 | 538.6 | 10 | 15 | 0 | 0 |
| Path 99 | C00024->C00031:[2->5,49->9,50->7] | 0.50 | 348.333333333 | 19 | 141 | 0 | 0 |
| Path 100 | C00024->C00031:[49->1,49->9,50->4,50->7] | 0.67 | 383.111111111 | 19 | 54 | 0 | 1 |
| Path 101 | C00024->C00031:[10->1,11->2,15->4,2->5,49->9,50->7] | 1.00 | 334.319018405 | 23 | 163 | 0 | 0 |
| Path 102 | C00024->C00031:[10->1,11->2,15->4,49->9,50->7,6->5] | 1.00 | 304.327433628 | 22 | 113 | 0 | 0 |
| Path 103 | C00024->C00031:[49->1,49->9,50->4,50->7] | 0.67 | 410.644067797 | 21 | 59 | 0 | 1 |
| Path 104 | C00024->C00031:[49->7,49->9,50->5] | 0.50 | 309.844155844 | 14 | 77 | 0 | 1 |
| Path 105 | C00024->C00031:[49->4,49->9,50->7] | 0.50 | 361.24 | 18 | 50 | 0 | 1 |
| Path 106 | C00024->C00031:[49->9,50->5,50->7] | 0.50 | 557.833333333 | 16 | 24 | 0 | 0 |
| Path 107 | C00024->C00031:[49->4,49->9,50->7] | 0.50 | 538.264705882 | 19 | 34 | 0 | 1 |
| Path 108 | C00024->C00031:[49->1,49->2,49->9,50->4,50->7] | 0.83 | 455.314285714 | 23 | 70 | 0 | 1 |
| Path 109 | C00024->C00031:[49->1,49->2,49->9,50->4,50->7] | 0.83 | 402.984126984 | 22 | 63 | 0 | 1 |
| Path 110 | C00024->C00031:[2->5,49->9,50->7] | 0.50 | 345.510638298 | 18 | 141 | 0 | 0 |
| Path 111 | C00024->C00031:[2->5,49->1,49->9,50->4,50->7] | 0.83 | 335.873737374 | 26 | 198 | 0 | 1 |
| Path 112 | C00024->C00031:[10->1,11->2,15->4,49->9,50->7,6->5] | 1.00 | 305.112359551 | 15 | 89 | 0 | 0 |
| Path 113 | C00024->C00031:[49->1,49->9,50->4,50->5,50->7] | 0.83 | 507.841269841 | 25 | 63 | 0 | 1 |
| Path 114 | C00024->C00031:[49->4,49->9,50->7] | 0.50 | 477.914285714 | 18 | 35 | 0 | 1 |
| Path 115 | C00024->C00031:[49->1,50->2,50->4] | 0.50 | 486.352941176 | 20 | 68 | 0 | 1 |
| Path 116 | C00024->C00031:[2->5,49->9,50->7] | 0.50 | 346.178571429 | 19 | 140 | 0 | 0 |
| Path 117 | C00024->C00031:[49->9,50->7] | 0.33 | 371.947368421 | 7 | 19 | 0 | 0 |
| Path 118 | C00024->C00031:[49->9,50->4,50->7] | 0.50 | 389.870967742 | 17 | 62 | 0 | 0 |
| Path 119 | C00024->C00031:[49->1,49->2,49->9,50->4,50->7] | 0.83 | 519.057142857 | 25 | 70 | 0 | 1 |
| Path 120 | C00024->C00031:[10->1,11->2,15->4,2->5,49->9,50->7] | 1.00 | 332.987341772 | 22 | 158 | 0 | 0 |
| Path 121 | C00024->C00031:[49->1,49->2,49->9,50->4,50->7] | 0.83 | 393.016129032 | 23 | 62 | 0 | 1 |
| Path 122 | C00024->C00031:[49->1,49->9,50->4,50->5,50->7] | 0.83 | 500.134328358 | 25 | 67 | 0 | 1 |
| Path 123 | C00024->C00031:[2->5,49->9,50->7] | 0.50 | 322.26875 | 21 | 160 | 0 | 0 |
| Path 124 | C00024->C00031:[2->5,49->9,50->7] | 0.50 | 311.219512195 | 21 | 164 | 0 | 0 |
| Path 125 | C00024->C00031:[2->5,49->9,50->7] | 0.50 | 314.117647059 | 18 | 153 | 0 | 0 |
| Path 126 | C00024->C00031:[49->1,49->2,50->4] | 0.50 | 484.074626866 | 18 | 67 | 0 | 1 |
| Path 127 | C00024->C00031:[49->1,49->9,50->4,50->7] | 0.67 | 532.022222222 | 19 | 45 | 0 | 0 |
| Path 128 | C00024->C00031:[49->9,50->7] | 0.33 | 592.6875 | 11 | 16 | 0 | 0 |
| Path 129 | C00024->C00031:[10->1,11->2,15->4,49->9,50->7,6->5] | 1.00 | 325.175757576 | 22 | 165 | 0 | 0 |
| Path 130 | C00024->C00031:[49->9,50->7] | 0.33 | 576.2 | 10 | 15 | 0 | 0 |
| Path 131 | C00024->C00031:[10->1,11->2,15->4,2->7,6->5] | 0.83 | 325.90776699 | 22 | 206 | 0 | 0 |
| Path 132 | C00024->C00031:[49->9,50->7] | 0.33 | 440.235294118 | 7 | 17 | 0 | 0 |
| Path 133 | C00024->C00031:[2->5,49->9,50->7] | 0.50 | 312.171974522 | 18 | 157 | 0 | 0 |
| Path 134 | C00024->C00031:[49->4,49->9,50->7] | 0.50 | 418.489361702 | 20 | 47 | 0 | 1 |
| Path 135 | C00024->C00031:[49->4,49->9,50->7] | 0.50 | 512.868421053 | 20 | 38 | 0 | 1 |
| Path 136 | C00024->C00031:[49->1,49->9,50->4,50->7] | 0.67 | 450.533333333 | 18 | 60 | 0 | 1 |
| Path 137 | C00024->C00031:[49->4] | 0.17 | 555.722222222 | 15 | 36 | 0 | 1 |
| Path 138 | C00024->C00031:[2->5,49->9,50->7] | 0.50 | 304.975460123 | 19 | 163 | 0 | 0 |
| Path 139 | C00024->C00031:[49->4,49->9,50->7] | 0.50 | 384.25 | 18 | 44 | 0 | 1 |
| Path 140 | C00024->C00031:[10->1,11->2,15->4,49->9,50->7,6->5] | 1.00 | 317.238095238 | 22 | 168 | 0 | 0 |
| Path 141 | C00024->C00031:[2->5,49->1,49->2,49->9,50->4,50->7] | 1.00 | 341.82 | 27 | 200 | 0 | 1 |
| Path 142 | C00024->C00031:[2->5,49->1,49->2,49->9,50->4,50->7] | 1.00 | 345.956097561 | 29 | 205 | 0 | 1 |
| Path 143 | C00024->C00031:[49->1,49->9,50->4,50->7] | 0.67 | 440.25 | 16 | 56 | 0 | 1 |
| Path 144 | C00024->C00031:[49->9,50->7] | 0.33 | 223.636363636 | 6 | 33 | 0 | 0 |
| Path 145 | C00024->C00031:[2->5,49->9,50->7] | 0.50 | 313.05625 | 21 | 160 | 0 | 0 |
| Path 146 | C00024->C00031:[2->5,49->1,49->9,50->4,50->7] | 0.83 | 341.094059406 | 27 | 202 | 0 | 1 |
| Path 147 | C00024->C00031:[49->1,49->2,50->4] | 0.50 | 467.452054795 | 20 | 73 | 0 | 1 |
| Path 148 | C00024->C00031:[49->1,49->2,49->9,50->4,50->7] | 0.83 | 366.430379747 | 19 | 79 | 0 | 1 |
| Path 149 | C00024->C00031:[49->9,50->5,50->7] | 0.50 | 518.290322581 | 18 | 31 | 0 | 0 |
| Path 150 | C00024->C00031:[49->1,49->2,49->9,50->4,50->7] | 0.83 | 407.881355932 | 21 | 59 | 0 | 1 |
| Path 151 | C00024->C00031:[49->1,50->4] | 0.33 | 445.620689655 | 13 | 58 | 0 | 1 |
| Path 152 | C00024->C00031:[2->5,49->9,50->7] | 0.50 | 326.2375 | 21 | 160 | 0 | 0 |
| Path 153 | C00024->C00031:[49->1,49->2,49->9,50->4,50->7] | 0.83 | 428.428571429 | 21 | 70 | 0 | 1 |
| Path 154 | C00024->C00031:[10->1,11->2,15->4,6->5] | 0.67 | 331.015037594 | 12 | 133 | 0 | 0 |
| Path 155 | C00024->C00031:[14->4] | 0.17 | 320.802325581 | 17 | 86 | 0 | 1 |
| Path 156 | C00024->C00031:[49->1,49->2,49->9,50->4,50->7] | 0.83 | 398.385964912 | 21 | 57 | 0 | 1 |
| Path 157 | C00024->C00031:[49->1,49->9,50->4,50->7] | 0.67 | 433.857142857 | 18 | 63 | 0 | 1 |
| Path 158 | C00024->C00031:[49->1,49->2,49->9,50->4,50->7] | 0.83 | 481.584415584 | 25 | 77 | 0 | 1 |
| Path 159 | C00024->C00031:[49->1,49->2,49->9,50->4,50->7] | 0.83 | 444.757575758 | 22 | 66 | 0 | 1 |
| Path 160 | C00024->C00031:[14->2,49->1,50->4] | 0.50 | 361.06779661 | 20 | 177 | 0 | 1 |
| Path 161 | C00024->C00031:[2->5,49->1,49->2,49->9,50->4,50->7] | 1.00 | 349.603960396 | 29 | 202 | 0 | 1 |
| Path 162 | C00024->C00031:[49->1,49->9,50->4,50->5,50->7] | 0.83 | 511.525423729 | 23 | 59 | 0 | 1 |
| Path 163 | C00024->C00031:[49->1,49->2,49->7,49->9,50->4,50->5] | 1.00 | 460.426470588 | 21 | 68 | 0 | 1 |
| Path 164 | C00024->C00031:[49->1,49->9,50->4,50->7] | 0.67 | 443.046875 | 19 | 64 | 0 | 1 |
| Path 165 | C00024->C00031:[49->1,49->2,49->9,50->4,50->5,50->7] | 1.00 | 497.426470588 | 27 | 68 | 0 | 1 |
| Path 166 | C00024->C00031:[2->5,49->9,50->7] | 0.50 | 343.166666667 | 19 | 144 | 0 | 0 |
| Path 167 | C00024->C00031:[49->1,49->9,50->4,50->7] | 0.67 | 475.698630137 | 22 | 73 | 0 | 1 |
| Path 168 | C00024->C00031:[10->1,11->2,15->4,6->5] | 0.67 | 313.939759036 | 13 | 83 | 0 | 0 |
| Path 169 | C00024->C00031:[49->9,50->4,50->7] | 0.50 | 511.98 | 19 | 50 | 0 | 0 |
| Path 170 | C00024->C00031:[49->1,49->2,49->9,50->4,50->7] | 0.83 | 487.219178082 | 25 | 73 | 0 | 1 |
| Path 171 | C00024->C00031:[49->4,49->9,50->7] | 0.50 | 549.972972973 | 21 | 37 | 0 | 1 |
| Path 172 | C00024->C00031:[49->1,49->9,50->4,50->5,50->7] | 0.83 | 522.578125 | 26 | 64 | 0 | 1 |
| Path 173 | C00024->C00031:[49->1,49->9,50->4,50->7] | 0.67 | 394.727272727 | 20 | 55 | 0 | 1 |
| Path 174 | C00024->C00031:[10->1,11->2,15->4,49->9,50->7,6->5] | 1.00 | 306.942307692 | 15 | 156 | 0 | 0 |
| Path 175 | C00024->C00031:[49->5,49->7,50->9] | 0.50 | 408.40625 | 17 | 64 | 0 | 0 |
| Path 176 | C00024->C00031:[49->1,49->2,49->7,49->9,50->4,50->5] | 1.00 | 481.707692308 | 22 | 65 | 0 | 1 |
| Path 177 | C00024->C00031:[49->1,49->2,49->9,50->4,50->7] | 0.83 | 389.1 | 21 | 60 | 0 | 1 |
| Path 178 | C00024->C00031:[49->4,49->9,50->7] | 0.50 | 350.510204082 | 17 | 49 | 0 | 1 |
| Path 179 | C00024->C00031:[49->4,49->9,50->7] | 0.50 | 465.882352941 | 17 | 34 | 0 | 1 |
| Path 180 | C00024->C00031:[49->1,49->2,49->9,50->4,50->7] | 0.83 | 489.309859155 | 24 | 71 | 0 | 1 |
| Path 181 | C00024->C00031:[14->4] | 0.17 | 331.3359375 | 14 | 128 | 0 | 1 |
| Path 182 | C00024->C00031:[14->7,49->9,50->5] | 0.50 | 327.366666667 | 16 | 150 | 0 | 1 |
| Path 183 | C00024->C00031:[49->1,49->2,49->9,50->4,50->7] | 0.83 | 499.246575342 | 24 | 73 | 0 | 1 |
| Path 184 | C00024->C00031:[49->1,49->2,49->9,50->4,50->7] | 0.83 | 401.9375 | 23 | 64 | 0 | 1 |
| Path 185 | C00024->C00031:[49->1,49->2,49->9,50->4,50->7] | 0.83 | 378.372881356 | 20 | 59 | 0 | 1 |
| Path 186 | C00024->C00031:[49->1,49->2,49->9,50->4,50->7] | 0.83 | 483.5 | 24 | 76 | 0 | 1 |
| Path 187 | C00024->C00031:[10->1,11->2,15->4,49->9,50->7,6->5] | 1.00 | 325.842424242 | 22 | 165 | 0 | 0 |
| Path 188 | C00024->C00031:[49->4,49->9,50->7] | 0.50 | 428.833333333 | 20 | 42 | 0 | 1 |
| Path 189 | C00024->C00031:[10->1,11->2,15->4,49->9,50->7,6->5] | 1.00 | 337.405797101 | 15 | 138 | 0 | 0 |
| Path 190 | C00024->C00031:[10->1,11->2,15->4,49->9,50->7,6->5] | 1.00 | 295.948497854 | 24 | 233 | 0 | 0 |
| Path 191 | C00024->C00031:[49->9,50->7] | 0.33 | 483.35 | 9 | 20 | 0 | 0 |
| Path 192 | C00024->C00031:[14->4] | 0.17 | 320.925925926 | 16 | 81 | 0 | 1 |
| Path 193 | C00024->C00031:[49->4,49->9,50->7] | 0.50 | 405.369565217 | 19 | 46 | 0 | 1 |
| Path 194 | C00024->C00031:[49->1,49->2,50->4] | 0.50 | 407.507042254 | 16 | 71 | 0 | 1 |
| Path 195 | C00024->C00031:[49->4,49->9,50->7] | 0.50 | 540.333333333 | 20 | 33 | 0 | 1 |
| Path 196 | C00024->C00031:[14->4] | 0.17 | 335.595419847 | 15 | 131 | 0 | 1 |
| Path 197 | C00024->C00031:[49->1,49->9,50->4,50->7] | 0.67 | 501.548387097 | 20 | 62 | 0 | 1 |
| Path 198 | C00024->C00031:[49->1,49->2,49->7,49->9,50->4,50->5] | 1.00 | 428.133333333 | 17 | 60 | 0 | 1 |
| Path 199 | C00024->C00031:[49->1,49->2,49->9,50->4,50->7] | 0.83 | 453.358208955 | 21 | 67 | 0 | 1 |
| Path 200 | C00024->C00031:[49->1,50->2,50->4] | 0.50 | 477.723076923 | 18 | 65 | 0 | 1 |
| Path 201 | C00024->C00031:[10->1,11->2,15->4,2->7,6->5] | 0.83 | 356.465277778 | 20 | 144 | 0 | 0 |
| Path 202 | C00024->C00031:[49->1,49->2,49->9,50->4,50->7] | 0.83 | 454.578125 | 20 | 64 | 0 | 1 |
| Path 203 | C00024->C00031:[49->7,49->9,50->5] | 0.50 | 440.725 | 14 | 40 | 0 | 1 |
| Path 204 | C00024->C00031:[2->5] | 0.17 | 336.288888889 | 14 | 135 | 0 | 0 |
| Path 205 | C00024->C00031:[49->1,49->2,49->9,50->4,50->7] | 0.83 | 479.368421053 | 25 | 76 | 0 | 1 |
| Path 206 | C00024->C00031:[49->9,50->5,50->7] | 0.50 | 563.071428571 | 18 | 28 | 0 | 0 |
| Path 207 | C00024->C00031:[2->5,49->9,50->7] | 0.50 | 328.506410256 | 21 | 156 | 0 | 0 |
| Path 208 | C00024->C00031:[2->5,49->1,49->2,49->9,50->4,50->7] | 1.00 | 340.910447761 | 28 | 201 | 0 | 1 |
| Path 209 | C00024->C00031:[49->7,49->9,50->5] | 0.50 | 449.348837209 | 17 | 43 | 0 | 1 |
| Path 210 | C00024->C00031:[2->5,49->9,50->7] | 0.50 | 316.088607595 | 19 | 158 | 0 | 0 |
| Path 211 | C00024->C00031:[49->1,49->9,50->4,50->7] | 0.67 | 378.211538462 | 17 | 52 | 0 | 1 |
| Path 212 | C00024->C00031:[49->7,49->9,50->5] | 0.50 | 316.4 | 14 | 50 | 0 | 1 |
| Path 213 | C00024->C00031:[49->1,49->2,49->9,50->4,50->7] | 0.83 | 442.703125 | 20 | 64 | 0 | 1 |
| Path 214 | C00024->C00031:[49->1,49->2,49->9,50->4,50->7] | 0.83 | 461.142857143 | 20 | 63 | 0 | 1 |
| Path 215 | C00024->C00031:[49->1,49->2,49->9,50->4,50->7] | 0.83 | 451.632352941 | 22 | 68 | 0 | 1 |
| Path 216 | C00024->C00031:[49->1,49->2,49->9,50->4,50->7] | 0.83 | 499.608108108 | 26 | 74 | 0 | 1 |
| Path 217 | C00024->C00031:[2->5,49->9,50->7] | 0.50 | 318.123376623 | 19 | 154 | 0 | 0 |
| Path 218 | C00024->C00031:[2->5,49->1,49->9,50->4,50->7] | 0.83 | 336.771573604 | 25 | 197 | 0 | 1 |
| Path 219 | C00024->C00031:[10->1,11->2,15->4,49->9,50->7,6->5] | 1.00 | 303.769565217 | 24 | 230 | 0 | 0 |
| Path 220 | C00024->C00031:[10->1,11->2,15->4,2->5,49->9,50->7] | 1.00 | 336.748427673 | 23 | 159 | 0 | 0 |
| Path 221 | C00024->C00031:[2->5,49->9,50->7] | 0.50 | 351.562043796 | 19 | 137 | 0 | 0 |
| Path 222 | C00024->C00031:[2->5,49->9,50->7] | 0.50 | 309.636942675 | 17 | 157 | 0 | 0 |
| Path 223 | C00024->C00031:[49->1,49->9,50->4,50->5,50->7] | 0.83 | 483.0 | 25 | 70 | 0 | 1 |
| Path 224 | C00024->C00031:[49->1,49->9,50->4,50->7] | 0.67 | 497.0625 | 18 | 48 | 0 | 0 |
| Path 225 | C00024->C00031:[49->4,49->7,49->9] | 0.50 | 434.25974026 | 29 | 77 | 0 | 1 |
| Path 226 | C00024->C00031:[49->1,49->9,50->4,50->7] | 0.67 | 539.979591837 | 20 | 49 | 0 | 0 |
| Path 227 | C00024->C00031:[49->1,49->2,49->9,50->4,50->7] | 0.83 | 401.766666667 | 21 | 60 | 0 | 1 |
| Path 228 | C00024->C00031:[49->4,49->9,50->7] | 0.50 | 534.294117647 | 19 | 34 | 0 | 1 |
| Path 229 | C00024->C00031:[49->9,50->5,50->7] | 0.50 | 572.620689655 | 19 | 29 | 0 | 0 |
| Path 230 | C00024->C00031:[10->1,11->2,15->4,49->9,50->7,6->5] | 1.00 | 307.039647577 | 24 | 227 | 0 | 0 |
| Path 231 | C00024->C00031:[49->1,49->2,49->9,50->4,50->7] | 0.83 | 494.945205479 | 25 | 73 | 0 | 1 |
| Path 232 | C00024->C00031:[49->7,49->9,50->5] | 0.50 | 445.56097561 | 16 | 41 | 0 | 1 |
| Path 233 | C00024->C00031:[49->4,49->9,50->7] | 0.50 | 309.186440678 | 18 | 59 | 0 | 1 |
| Path 234 | C00024->C00031:[49->9,50->5,50->7] | 0.50 | 574.24137931 | 19 | 29 | 0 | 0 |
| Path 235 | C00024->C00031:[49->1,49->9,50->4,50->7] | 0.67 | 452.491525424 | 19 | 59 | 0 | 1 |
| Path 236 | C00024->C00031:[10->1,11->2,15->4,49->9,50->7,6->5] | 1.00 | 328.584337349 | 23 | 166 | 0 | 0 |
| Path 237 | C00024->C00031:[49->1,49->2,49->9,50->4,50->7] | 0.83 | 382.704918033 | 22 | 61 | 0 | 1 |
| Path 238 | C00024->C00031:[49->1,49->2,49->9,50->4,50->5,50->7] | 1.00 | 529.328358209 | 28 | 67 | 0 | 1 |
| Path 239 | C00024->C00031:[49->1,49->2,49->9,50->4,50->5,50->7] | 1.00 | 505.492753623 | 27 | 69 | 0 | 1 |
| Path 240 | C00024->C00031:[49->1,49->2,49->9,50->4,50->7] | 0.83 | 444.742424242 | 20 | 66 | 0 | 1 |
| Path 241 | C00024->C00031:[49->1,49->9,50->4,50->5,50->7] | 0.83 | 497.863636364 | 25 | 66 | 0 | 1 |
| Path 242 | C00024->C00031:[49->1,49->9,50->4,50->7] | 0.67 | 389.466666667 | 20 | 60 | 0 | 1 |
| Path 243 | C00024->C00031:[10->1,11->2,15->4,49->9,50->7,6->5] | 1.00 | 299.389830508 | 23 | 118 | 0 | 0 |
| Path 244 | C00024->C00031:[49->1,49->2,49->9,50->4,50->5,50->7] | 1.00 | 528.626865672 | 28 | 67 | 0 | 1 |
| Path 245 | C00024->C00031:[14->4] | 0.17 | 330.864661654 | 15 | 133 | 0 | 1 |
| Path 246 | C00024->C00031:[49->1,49->2,49->9,50->4,50->7] | 0.83 | 423.35483871 | 23 | 62 | 0 | 1 |
| Path 247 | C00024->C00031:[49->4] | 0.17 | 554.8125 | 10 | 16 | 0 | 1 |
| Path 248 | C00024->C00031:[49->1,49->2,49->9,50->4,50->7] | 0.83 | 405.517857143 | 20 | 56 | 0 | 1 |
| Path 249 | C00024->C00031:[49->1,49->2,49->9,50->4,50->7] | 0.83 | 433.507936508 | 19 | 63 | 0 | 1 |
| Path 250 | C00024->C00031:[49->9,50->5,50->7] | 0.50 | 413.27027027 | 14 | 37 | 0 | 1 |
| Path 251 | C00024->C00031:[10->1,11->2,15->4,2->5,49->9,50->7] | 1.00 | 330.635802469 | 22 | 162 | 0 | 0 |
| Path 252 | C00024->C00031:[49->7,49->9,50->5] | 0.50 | 384.176470588 | 11 | 34 | 0 | 1 |
| Path 253 | C00024->C00031:[49->1,49->2,49->9,50->4,50->7] | 0.83 | 378.833333333 | 22 | 66 | 0 | 1 |
| Path 254 | C00024->C00031:[2->5,49->9,50->7] | 0.50 | 324.201257862 | 21 | 159 | 0 | 0 |
| Path 255 | C00024->C00031:[49->9,50->7] | 0.33 | 389.25 | 7 | 20 | 0 | 0 |
| Path 256 | C00024->C00031:[49->4,49->9,50->7] | 0.50 | 446.282608696 | 21 | 46 | 0 | 1 |
| Path 257 | C00024->C00031:[49->1,49->2,49->9,50->4,50->7] | 0.83 | 471.706666667 | 25 | 75 | 0 | 1 |
| Path 258 | C00024->C00031:[49->1,49->9,50->4,50->7] | 0.67 | 485.893617021 | 17 | 47 | 0 | 0 |
| Path 259 | C00024->C00031:[49->1,49->9,50->4,50->7] | 0.67 | 498.636363636 | 22 | 66 | 0 | 1 |
| Path 260 | C00024->C00031:[49->1,49->2,49->9,50->4,50->7] | 0.83 | 443.119402985 | 21 | 67 | 0 | 1 |
| Path 261 | C00024->C00031:[49->1,49->2,50->4] | 0.50 | 456.819672131 | 15 | 61 | 0 | 1 |
| Path 262 | C00024->C00031:[49->1,49->9,50->4,50->7] | 0.67 | 442.672413793 | 18 | 58 | 0 | 1 |
| Path 263 | C00024->C00031:[49->1,49->9,50->4,50->7] | 0.67 | 463.789473684 | 17 | 57 | 0 | 1 |
| Path 264 | C00024->C00031:[49->1,49->2,49->9,50->4,50->7] | 0.83 | 463.177419355 | 21 | 62 | 0 | 1 |
| Path 265 | C00024->C00031:[49->5,49->7,50->9] | 0.50 | 546.708333333 | 18 | 48 | 0 | 0 |
| Path 266 | C00024->C00031:[2->5] | 0.17 | 339.237037037 | 15 | 135 | 0 | 0 |
| Path 267 | C00024->C00031:[10->1,11->2,15->4,49->9,50->7,6->5] | 1.00 | 304.469827586 | 25 | 232 | 0 | 0 |
| Path 268 | C00024->C00031:[49->9,50->7] | 0.33 | 408.466666667 | 7 | 15 | 0 | 0 |
| Path 269 | C00024->C00031:[10->1,11->2,15->4,49->9,50->7,6->5] | 1.00 | 300.92173913 | 24 | 230 | 0 | 0 |
| Path 270 | C00024->C00031:[49->1,49->9,50->4,50->7] | 0.67 | 507.181818182 | 22 | 66 | 0 | 1 |
| Path 271 | C00024->C00031:[10->1,11->2,15->4,2->5,49->9,50->7] | 1.00 | 325.366459627 | 20 | 161 | 0 | 0 |
| Path 272 | C00024->C00031:[14->4] | 0.17 | 313.698795181 | 16 | 83 | 0 | 1 |
| Path 273 | C00024->C00031:[49->1,49->9,50->4,50->7] | 0.67 | 393.660714286 | 19 | 56 | 0 | 1 |
| Path 274 | C00024->C00031:[49->4,49->9,50->7] | 0.50 | 523.606060606 | 18 | 33 | 0 | 1 |
| Path 275 | C00024->C00031:[49->4,49->9,50->7] | 0.50 | 499.108108108 | 19 | 37 | 0 | 1 |
| Path 276 | C00024->C00031:[49->1,49->2,49->7,49->9,50->4,50->5] | 1.00 | 386.236111111 | 19 | 72 | 0 | 1 |
| Path 277 | C00024->C00031:[10->1,11->2,15->4,49->9,50->7,6->5] | 1.00 | 318.607142857 | 22 | 168 | 0 | 0 |
| Path 278 | C00024->C00031:[49->9,50->7] | 0.33 | 419.380952381 | 8 | 21 | 0 | 0 |
| Path 279 | C00024->C00031:[49->1,49->2,50->4] | 0.50 | 487.514285714 | 21 | 70 | 0 | 1 |
| Path 280 | C00024->C00031:[49->1,49->9,50->4,50->5,50->7] | 0.83 | 521.84375 | 26 | 64 | 0 | 1 |
| Path 281 | C00024->C00031:[2->5,49->9,50->7] | 0.50 | 317.532467532 | 19 | 154 | 0 | 0 |
| Path 282 | C00024->C00031:[10->1,11->2,15->4,49->9,50->7,6->5] | 1.00 | 321.136904762 | 22 | 168 | 0 | 0 |
| Path 283 | C00024->C00031:[49->1,49->9,50->4,50->7] | 0.67 | 463.73015873 | 20 | 63 | 0 | 1 |
| Path 284 | C00024->C00031:[10->1,11->2,15->4,49->9,50->7,6->5] | 1.00 | 306.475770925 | 24 | 227 | 0 | 0 |
| Path 285 | C00024->C00031:[49->1,49->2,49->9,50->4,50->7] | 0.83 | 418.116666667 | 22 | 60 | 0 | 1 |
| Path 286 | C00024->C00031:[2->5,49->9,50->7] | 0.50 | 355.101449275 | 20 | 138 | 0 | 0 |
| Path 287 | C00024->C00031:[49->1,49->2,49->9,50->4,50->7] | 0.83 | 394.309090909 | 19 | 55 | 0 | 1 |
| Path 288 | C00024->C00031:[49->1,49->9,50->4,50->5,50->7] | 0.83 | 489.307692308 | 25 | 65 | 0 | 1 |
| Path 289 | C00024->C00031:[49->4,49->9,50->7] | 0.50 | 529.5 | 19 | 32 | 0 | 1 |
| Path 290 | C00024->C00031:[13->4] | 0.17 | 332.122137405 | 16 | 131 | 0 | 1 |
| Path 291 | C00024->C00031:[49->4,49->9,50->7] | 0.50 | 540.611111111 | 20 | 36 | 0 | 1 |
| Path 292 | C00024->C00031:[10->1,11->2,15->4,49->9,50->7,6->5] | 1.00 | 330.127659574 | 15 | 141 | 0 | 0 |
| Path 293 | C00024->C00031:[49->4,49->9,50->7] | 0.50 | 426.651162791 | 19 | 43 | 0 | 1 |
| Path 294 | C00024->C00031:[49->1,49->2,49->9,50->4,50->7] | 0.83 | 514.405797101 | 24 | 69 | 0 | 1 |
| Path 295 | C00024->C00031:[10->1,11->2,15->4,49->9,50->7,6->5] | 1.00 | 314.826589595 | 23 | 173 | 0 | 0 |
| Path 296 | C00024->C00031:[49->1,49->9,50->4,50->7] | 0.67 | 481.191176471 | 22 | 68 | 0 | 1 |
| Path 297 | C00024->C00031:[49->9,50->7] | 0.33 | 450.25 | 9 | 16 | 0 | 0 |
| Path 298 | C00024->C00031:[49->4] | 0.17 | 574.970588235 | 15 | 34 | 0 | 1 |
| Path 299 | C00024->C00031:[49->9,50->4,50->7] | 0.50 | 527.739130435 | 18 | 46 | 0 | 0 |
| Path 300 | C00024->C00031:[49->9,50->7] | 0.33 | 449.772727273 | 10 | 22 | 0 | 0 |
| Path 301 | C00024->C00031:[49->1,49->2,49->9,50->4,50->5,50->7] | 1.00 | 507.557142857 | 27 | 70 | 0 | 1 |
| Path 302 | C00024->C00031:[49->1,49->2,49->9,50->4,50->7] | 0.83 | 435.876923077 | 21 | 65 | 0 | 1 |
| Path 303 | C00024->C00031:[49->1,49->2,49->9,50->4,50->7] | 0.83 | 392.095238095 | 22 | 63 | 0 | 1 |
| Path 304 | C00024->C00031:[49->1,49->2,49->9,50->4,50->7] | 0.83 | 467.2875 | 25 | 80 | 0 | 1 |
| Path 305 | C00024->C00031:[10->1,11->2,15->4,49->9,50->7,6->5] | 1.00 | 317.283236994 | 23 | 173 | 0 | 0 |
| Path 306 | C00024->C00031:[2->5,49->9,50->7] | 0.50 | 335.746478873 | 18 | 142 | 0 | 0 |
| Path 307 | C00024->C00031:[10->1,11->2,15->4,2->5] | 0.67 | 363.220588235 | 19 | 136 | 0 | 0 |
| Path 308 | C00024->C00031:[49->9,50->5] | 0.33 | 356.193548387 | 9 | 31 | 0 | 1 |
| Path 309 | C00024->C00031:[49->1,49->2,49->9,50->4,50->7] | 0.83 | 407.348484848 | 24 | 66 | 0 | 1 |
| Path 310 | C00024->C00031:[2->5,50->7] | 0.33 | 346.742857143 | 18 | 140 | 0 | 0 |
| Path 311 | C00024->C00031:[49->4,49->9,50->7] | 0.50 | 481.771428571 | 18 | 35 | 0 | 1 |
| Path 312 | C00024->C00031:[2->5] | 0.17 | 342.335877863 | 15 | 131 | 0 | 0 |
| Path 313 | C00024->C00031:[49->9,50->7] | 0.33 | 335.833333333 | 6 | 18 | 0 | 0 |
| Path 314 | C00024->C00031:[49->1,49->2,49->7,49->9,50->4,50->5] | 1.00 | 357.504761905 | 21 | 105 | 0 | 1 |
| Path 315 | C00024->C00031:[49->1,49->2,49->7,49->9,50->4,50->5] | 1.00 | 447.339285714 | 16 | 56 | 0 | 1 |
| Path 316 | C00024->C00031:[49->7,49->9,50->5] | 0.50 | 360.5 | 12 | 38 | 0 | 1 |
| Path 317 | C00024->C00031:[49->4,49->9,50->7] | 0.50 | 414.365853659 | 19 | 41 | 0 | 1 |
| Path 318 | C00024->C00031:[49->4,49->9,50->7] | 0.50 | 443.75 | 18 | 40 | 0 | 1 |
| Path 319 | C00024->C00031:[49->9,50->5,50->7] | 0.50 | 522.40625 | 18 | 32 | 0 | 0 |
| Path 320 | C00024->C00031:[49->1,49->9,50->4,50->7] | 0.67 | 304.736111111 | 18 | 72 | 0 | 1 |
| Path 321 | C00024->C00031:[49->9,50->4,50->7] | 0.50 | 550.697674419 | 17 | 43 | 0 | 0 |
| Path 322 | C00024->C00031:[49->4,49->9,50->7] | 0.50 | 488.333333333 | 18 | 36 | 0 | 1 |
| Path 323 | C00024->C00031:[49->1,49->2,49->9,50->4,50->7] | 0.83 | 454.016393443 | 20 | 61 | 0 | 1 |
| Path 324 | C00024->C00031:[49->1,49->9,50->4,50->5,50->7] | 0.83 | 516.793650794 | 25 | 63 | 0 | 1 |
| Path 325 | C00024->C00031:[49->1,49->2,49->9,50->4,50->7] | 0.83 | 319.48 | 20 | 75 | 0 | 1 |
| Path 326 | C00024->C00031:[49->4,49->9,50->7] | 0.50 | 493.027777778 | 19 | 36 | 0 | 1 |
| Path 327 | C00024->C00031:[49->7,49->9] | 0.33 | 387.590163934 | 18 | 61 | 0 | 0 |
| Path 328 | C00024->C00031:[49->7,49->9,50->5] | 0.50 | 321.113636364 | 12 | 44 | 0 | 1 |
| Path 329 | C00024->C00031:[49->1,49->2,49->9,50->4,50->7] | 0.83 | 500.243243243 | 26 | 74 | 0 | 1 |
| Path 330 | C00024->C00031:[49->1,49->2,49->9,50->4,50->7] | 0.83 | 469.90625 | 21 | 64 | 0 | 1 |
| Path 331 | C00024->C00031:[10->1,11->2,15->4,49->9,50->7,6->5] | 1.00 | 321.888235294 | 23 | 170 | 0 | 0 |
| Path 332 | C00024->C00031:[49->1,49->2,49->9,50->4,50->7] | 0.83 | 311.367088608 | 21 | 79 | 0 | 1 |
| Path 333 | C00024->C00031:[49->1,49->2,50->4] | 0.50 | 366.272222222 | 22 | 180 | 0 | 1 |
| Path 334 | C00024->C00031:[49->1,49->2,49->9,50->4,50->7] | 0.83 | 356.445783133 | 20 | 83 | 0 | 1 |
| Path 335 | C00024->C00031:[49->1,49->2,49->9,50->4,50->7] | 0.83 | 474.266666667 | 19 | 60 | 0 | 1 |
| Path 336 | C00024->C00031:[49->9,50->7] | 0.33 | 446.8125 | 8 | 16 | 0 | 0 |
| Path 337 | C00024->C00031:[49->9,50->7] | 0.33 | 492.0 | 6 | 14 | 0 | 0 |
| Path 338 | C00024->C00031:[49->1,49->2,49->9,50->4,50->7] | 0.83 | 509.476923077 | 22 | 65 | 0 | 1 |
| Path 339 | C00024->C00031:[49->9,50->7] | 0.33 | 472.555555556 | 8 | 18 | 0 | 0 |
| Path 340 | C00024->C00031:[2->5,49->9,50->7] | 0.50 | 329.765957447 | 16 | 141 | 0 | 0 |
| Path 341 | C00024->C00031:[49->1,49->9,50->4,50->7] | 0.67 | 512.149253731 | 23 | 67 | 0 | 1 |
| Path 342 | C00024->C00031:[49->1,49->9,50->4,50->7] | 0.67 | 489.492753623 | 22 | 69 | 0 | 1 |
| Path 343 | C00024->C00031:[10->1,11->2,15->4,49->9,50->7,6->5] | 1.00 | 301.30212766 | 25 | 235 | 0 | 0 |
| Path 344 | C00024->C00031:[2->5,49->9,50->7] | 0.50 | 319.93081761 | 19 | 159 | 0 | 0 |
| Path 345 | C00024->C00031:[49->1,49->2,50->4] | 0.50 | 371.75 | 18 | 104 | 0 | 1 |
| Path 346 | C00024->C00031:[49->1,49->2,49->7,49->9,50->4,50->5] | 1.00 | 360.651428571 | 23 | 175 | 0 | 1 |
| Path 347 | C00024->C00031:[10->1,11->2,15->4,49->9,50->7,6->5] | 1.00 | 301.675324675 | 14 | 154 | 0 | 0 |
| Path 348 | C00024->C00031:[49->1,49->2,49->7,49->9,50->4,50->5] | 1.00 | 480.26984127 | 21 | 63 | 0 | 1 |
| Path 349 | C00024->C00031:[49->9,50->5,50->7] | 0.50 | 427.578947368 | 14 | 38 | 0 | 1 |
| Path 350 | C00024->C00031:[49->4,49->9,50->7] | 0.50 | 502.756756757 | 19 | 37 | 0 | 1 |
| Path 351 | C00024->C00031:[10->1,11->2,15->4,49->9,50->7,6->5] | 1.00 | 313.861445783 | 21 | 166 | 0 | 0 |
| Path 352 | C00024->C00031:[14->4] | 0.17 | 313.371794872 | 15 | 78 | 0 | 1 |
| Path 353 | C00024->C00031:[14->4] | 0.17 | 334.977941176 | 16 | 136 | 0 | 1 |
| Path 354 | C00024->C00031:[49->1,49->2,49->9,50->4,50->7] | 0.83 | 489.173913043 | 23 | 69 | 0 | 1 |
| Path 355 | C00024->C00031:[10->1,11->2,15->4,49->9,50->7,6->5] | 1.00 | 342.764285714 | 16 | 140 | 0 | 0 |
| Path 356 | C00024->C00031:[10->1,11->2,15->4,49->9,50->7,6->5] | 1.00 | 324.931818182 | 16 | 88 | 0 | 0 |
| Path 357 | C00024->C00031:[2->5,49->1,49->9,50->4,50->7] | 0.83 | 344.72361809 | 27 | 199 | 0 | 1 |
| Path 358 | C00024->C00031:[49->1,49->9,50->4,50->7] | 0.67 | 378.745762712 | 19 | 59 | 0 | 1 |
| Path 359 | C00024->C00031:[49->1,49->2,49->9,50->4,50->7] | 0.83 | 419.089285714 | 20 | 56 | 0 | 1 |
| Path 360 | C00024->C00031:[2->5,49->9,50->7] | 0.50 | 315.512658228 | 19 | 158 | 0 | 0 |
| Path 361 | C00024->C00031:[10->1,11->2,15->4,49->9,50->7,6->5] | 1.00 | 273.942307692 | 15 | 104 | 0 | 0 |
| Path 362 | C00024->C00031:[49->7] | 0.17 | 498.742857143 | 12 | 35 | 0 | 0 |
| Path 363 | C00024->C00031:[49->9,50->5,50->7] | 0.50 | 486.228571429 | 18 | 35 | 0 | 0 |
| Path 364 | C00024->C00031:[49->1,49->2,50->4] | 0.50 | 366.845454545 | 20 | 110 | 0 | 1 |
| Path 365 | C00024->C00031:[49->4,49->9,50->7] | 0.50 | 548.228571429 | 20 | 35 | 0 | 1 |
| Path 366 | C00024->C00031:[10->1,11->2,15->4,49->9,50->7,6->5] | 1.00 | 310.175438596 | 22 | 171 | 0 | 0 |
| Path 367 | C00024->C00031:[49->9,50->7] | 0.33 | 496.888888889 | 10 | 18 | 0 | 0 |
| Path 368 | C00024->C00031:[49->1,49->9,50->4,50->7] | 0.67 | 390.358490566 | 18 | 53 | 0 | 1 |
| Path 369 | C00024->C00031:[2->5,49->9,50->7] | 0.50 | 324.435897436 | 21 | 156 | 0 | 0 |
| Path 370 | C00024->C00031:[49->7,49->9,50->5] | 0.50 | 334.150326797 | 18 | 153 | 0 | 1 |
| Path 371 | C00024->C00031:[49->7,49->9,50->5] | 0.50 | 420.0 | 16 | 46 | 0 | 1 |
| Path 372 | C00024->C00031:[49->4] | 0.17 | 559.212121212 | 14 | 33 | 0 | 1 |
| Path 373 | C00024->C00031:[49->4,49->9,50->7] | 0.50 | 556.1 | 17 | 30 | 0 | 1 |
| Path 374 | C00024->C00031:[49->1,49->2,49->9,50->4,50->7] | 0.83 | 506.231884058 | 24 | 69 | 0 | 1 |
| Path 375 | C00024->C00031:[10->1,11->2,15->4,49->9,50->7,6->5] | 1.00 | 298.320175439 | 23 | 228 | 0 | 0 |
| Path 376 | C00024->C00031:[10->1,11->2,15->4,49->9,50->7,6->5] | 1.00 | 327.921686747 | 23 | 166 | 0 | 0 |
| Path 377 | C00024->C00031:[49->1,49->2,49->7,49->9,50->4,50->5] | 1.00 | 453.590909091 | 22 | 66 | 0 | 1 |
| Path 378 | C00024->C00031:[49->9,50->4,50->7] | 0.50 | 538.255319149 | 19 | 47 | 0 | 0 |
| Path 379 | C00024->C00031:[49->1,49->2,49->9,50->4,50->7] | 0.83 | 436.788732394 | 22 | 71 | 0 | 1 |
| Path 380 | C00024->C00031:[49->4,49->9,50->7] | 0.50 | 513.612903226 | 18 | 31 | 0 | 1 |
| Path 381 | C00024->C00031:[2->5,49->9,50->7] | 0.50 | 334.607594937 | 23 | 158 | 0 | 0 |
| Path 382 | C00024->C00031:[2->5,49->9,50->7] | 0.50 | 332.216049383 | 23 | 162 | 0 | 0 |
| Path 383 | C00024->C00031:[10->1,11->2,15->4,6->5] | 0.67 | 336.666666667 | 13 | 135 | 0 | 0 |
| Path 384 | C00024->C00031:[49->1,49->2,49->9,50->4,50->7] | 0.83 | 497.152777778 | 24 | 72 | 0 | 1 |
| Path 385 | C00024->C00031:[49->1,49->2,49->9,50->4,50->5,50->7] | 1.00 | 519.35483871 | 25 | 62 | 0 | 1 |
| Path 386 | C00024->C00031:[49->4] | 0.17 | 563.461538462 | 9 | 13 | 0 | 1 |
| Path 387 | C00024->C00031:[49->1,49->2,49->9,50->4,50->5,50->7] | 1.00 | 490.821917808 | 27 | 73 | 0 | 1 |
| Path 388 | C00024->C00031:[49->9,50->5,50->7] | 0.50 | 542.928571429 | 18 | 28 | 0 | 0 |
| Path 389 | C00024->C00031:[2->5,49->9,50->7] | 0.50 | 306.615384615 | 16 | 156 | 0 | 0 |
| Path 390 | C00024->C00031:[2->5,49->9,50->7] | 0.50 | 351.795774648 | 20 | 142 | 0 | 0 |
| Path 391 | C00024->C00031:[49->1,49->9,50->4,50->7] | 0.67 | 404.698113208 | 18 | 53 | 0 | 1 |
| Path 392 | C00024->C00031:[49->7,49->9,50->5] | 0.50 | 407.909090909 | 17 | 44 | 0 | 1 |
| Path 393 | C00024->C00031:[49->9,50->5,50->7] | 0.50 | 445.56097561 | 16 | 41 | 0 | 1 |
| Path 394 | C00024->C00031:[49->9,50->5,50->7] | 0.50 | 500.433333333 | 18 | 30 | 0 | 0 |
| Path 395 | C00024->C00031:[10->1,11->2,15->4,49->9,50->7,6->5] | 1.00 | 325.951515152 | 22 | 165 | 0 | 0 |
| Path 396 | C00024->C00031:[49->1,49->9,50->4,50->7] | 0.67 | 491.785714286 | 22 | 70 | 0 | 1 |
| Path 397 | C00024->C00031:[49->1,49->2,49->9,50->4,50->7] | 0.83 | 461.6 | 19 | 60 | 0 | 1 |
| Path 398 | C00024->C00031:[2->5,49->9,50->7] | 0.50 | 338.586956522 | 18 | 138 | 0 | 0 |
| Path 399 | C00024->C00031:[10->1,11->2,15->4,2->5] | 0.67 | 356.792857143 | 18 | 140 | 0 | 0 |
| Path 400 | C00024->C00031:[49->1,49->9,50->4,50->7] | 0.67 | 459.901639344 | 19 | 61 | 0 | 1 |
| Path 401 | C00024->C00031:[49->1,49->2,49->9,50->4,50->7] | 0.83 | 519.728571429 | 25 | 70 | 0 | 1 |
| Path 402 | C00024->C00031:[10->1,11->2,15->4,49->9,50->7,6->5] | 1.00 | 321.208588957 | 21 | 163 | 0 | 0 |
| Path 403 | C00024->C00031:[49->4,49->9,50->7] | 0.50 | 398.422222222 | 19 | 45 | 0 | 1 |
| Path 404 | C00024->C00031:[10->1,11->2,15->4,2->7,6->5] | 0.83 | 355.701388889 | 20 | 144 | 0 | 0 |
| Path 405 | C00024->C00031:[10->1,11->2,15->4,49->9,50->7,6->5] | 1.00 | 325.741176471 | 23 | 170 | 0 | 0 |
| Path 406 | C00024->C00031:[10->1,11->2,15->4,49->9,50->7,6->5] | 1.00 | 324.625899281 | 14 | 139 | 0 | 0 |
| Path 407 | C00024->C00031:[49->1,49->2,49->9,50->4,50->7] | 0.83 | 409.137931034 | 22 | 58 | 0 | 1 |
| Path 408 | C00024->C00031:[49->1,49->2,50->4] | 0.50 | 394.266666667 | 17 | 75 | 0 | 1 |
| Path 409 | C00024->C00031:[49->1,49->2,49->9,50->4,50->7] | 0.83 | 473.257575758 | 22 | 66 | 0 | 1 |
| Path 410 | C00024->C00031:[49->1,49->2,49->9,50->4,50->5,50->7] | 1.00 | 523.909090909 | 27 | 66 | 0 | 1 |
| Path 411 | C00024->C00031:[10->1,11->2,15->4,49->9,50->7,6->5] | 1.00 | 308.557017544 | 25 | 228 | 0 | 0 |
| Path 412 | C00024->C00031:[49->4] | 0.17 | 310.68627451 | 12 | 51 | 0 | 1 |
| Path 413 | C00024->C00031:[49->4,49->9,50->7] | 0.50 | 566.774193548 | 18 | 31 | 0 | 1 |
| Path 414 | C00024->C00031:[10->1,11->2,15->4,49->9,50->7,6->5] | 1.00 | 303.435555556 | 23 | 225 | 0 | 0 |
| Path 415 | C00024->C00031:[49->1,49->9,50->4,50->7] | 0.67 | 520.886363636 | 18 | 44 | 0 | 0 |
| Path 416 | C00024->C00031:[14->4] | 0.17 | 321.79389313 | 14 | 131 | 0 | 1 |
| Path 417 | C00024->C00031:[49->1,49->9,50->4,50->7] | 0.67 | 450.456140351 | 17 | 57 | 0 | 1 |
| Path 418 | C00024->C00031:[2->5,49->9,50->7] | 0.50 | 342.4 | 17 | 140 | 0 | 0 |
| Path 419 | C00024->C00031:[49->1,49->2,49->9,50->4,50->7] | 0.83 | 452.101694915 | 18 | 59 | 0 | 1 |
| Path 420 | C00024->C00031:[49->9,50->7] | 0.33 | 504.947368421 | 10 | 19 | 0 | 0 |
| Path 421 | C00024->C00031:[10->1,11->2,15->4,49->9,50->7,6->5] | 1.00 | 321.8 | 24 | 175 | 0 | 0 |
| Path 422 | C00024->C00031:[49->9,50->7] | 0.33 | 464.117647059 | 10 | 17 | 0 | 0 |
| Path 423 | C00024->C00031:[49->1,49->9,50->4,50->7] | 0.67 | 404.684210526 | 20 | 57 | 0 | 1 |
| Path 424 | C00024->C00031:[10->1,11->2,15->4,2->5] | 0.67 | 359.635714286 | 19 | 140 | 0 | 0 |
| Path 425 | C00024->C00031:[49->1,49->2,50->4] | 0.50 | 438.507692308 | 16 | 65 | 0 | 1 |
| Path 426 | C00024->C00031:[49->1,49->9,50->4,50->5] | 0.67 | 434.547169811 | 14 | 53 | 0 | 1 |
| Path 427 | C00024->C00031:[2->5,49->9,50->7] | 0.50 | 337.321678322 | 17 | 143 | 0 | 0 |
| Path 428 | C00024->C00031:[49->7,49->9,50->5] | 0.50 | 307.819277108 | 16 | 83 | 0 | 1 |
