# Supplementary material for: 13C-metabolic flux ratio and novel carbon path analyses confirmed that Trichoderma reesei uses primarily the respirative pathway also on the preferred carbon source glucose
Source: BMC Syst Biol. 2009 Oct 29;3:104. doi: 10.1186/1752-0509-3-104 (PMC2776023; doi:10.1186/1752-0509-3-104)
Supplement: Additional file 1 — Pathways discovered in ReTrace carbon path analysis. Graphical and tabular representations of amino acid synthesis pathways discovered in ReTrace carbon path analysis [21]. Self-contained web site: unpack zip archive and open index.html with a web browser. [file 1752-0509-3-104-S1.zip › AF1-treesei/pathways-C00065-to-C00037.html]

Pathways from C00065 to C00037


**Pathways from C00065 to C00037**

**Sources:** L-Serine; (C00065)

**Target:**Glycine; (C00037)

|  | Composite mapping | Z | Average score | Rpairs | Reactions | Zero scores | Scores under threshold |
| --- | --- | --- | --- | --- | --- | --- | --- |
| Path 1 | C00065->C00037:[2->1,3->2] | 1.00 | 287.837209302 | 19 | 86 | 0 | 0 |
| Path 2 | C00065->C00037:[2->1,3->2] | 1.00 | 325.109756098 | 15 | 82 | 0 | 0 |
| Path 3 | C00065->C00037:[2->1,3->2] | 1.00 | 398.886363636 | 10 | 44 | 0 | 0 |
| Path 4 | C00065->C00037:[3->1,3->2] | 1.00 | 464.673913043 | 13 | 46 | 0 | 0 |
| Path 5 | C00065->C00037:[2->1,3->2] | 1.00 | 610.719512195 | 17 | 82 | 0 | 0 |
| Path 6 | C00065->C00037:[2->1,3->2] | 1.00 | 307.180722892 | 20 | 83 | 0 | 0 |
| Path 7 | C00065->C00037:[1->1,3->2] | 1.00 | 287.357142857 | 8 | 28 | 0 | 0 |
| Path 8 | C00065->C00037:[1->2,2->1] | 1.00 | 681.935064935 | 20 | 77 | 0 | 0 |
| Path 9 | C00065->C00037:[1->1,3->2] | 1.00 | 398.26 | 12 | 50 | 0 | 0 |
| Path 10 | C00065->C00037:[2->1,3->2] | 1.00 | 320.4625 | 19 | 80 | 0 | 0 |
| Path 11 | C00065->C00037:[2->1,3->2] | 1.00 | 279.423076923 | 16 | 78 | 0 | 0 |
| Path 12 | C00065->C00037:[2->1,3->2] | 1.00 | 320.054794521 | 16 | 73 | 0 | 0 |
| Path 13 | C00065->C00037:[2->1,3->2] | 1.00 | 621.3 | 16 | 80 | 0 | 0 |
| Path 14 | C00065->C00037:[1->2,2->1] | 1.00 | 389.532467532 | 23 | 77 | 0 | 0 |
| Path 15 | C00065->C00037:[2->1,3->2] | 1.00 | 315.928571429 | 9 | 28 | 0 | 0 |
| Path 16 | C00065->C00037:[2->1,3->2] | 1.00 | 333.453488372 | 18 | 86 | 0 | 0 |
| Path 17 | C00065->C00037:[2->1,3->2] | 1.00 | 323.788732394 | 15 | 71 | 0 | 0 |
| Path 18 | C00065->C00037:[2->1,3->2] | 1.00 | 627.148148148 | 17 | 81 | 0 | 0 |
| Path 19 | C00065->C00037:[1->2,2->1] | 1.00 | 394.444444444 | 22 | 72 | 0 | 0 |
| Path 20 | C00065->C00037:[1->1,3->2] | 1.00 | 413.076923077 | 9 | 26 | 0 | 0 |
| Path 21 | C00065->C00037:[1->1,3->2] | 1.00 | 303.894736842 | 11 | 38 | 0 | 0 |
| Path 22 | C00065->C00037:[2->1,3->2] | 1.00 | 336.146341463 | 20 | 82 | 0 | 0 |
| Path 23 | C00065->C00037:[2->1,3->2] | 1.00 | 616.543209877 | 17 | 81 | 0 | 0 |
| Path 24 | C00065->C00037:[2->1,3->2] | 1.00 | 311.690140845 | 15 | 71 | 0 | 0 |
| Path 25 | C00065->C00037:[2->1,3->2] | 1.00 | 563.693181818 | 18 | 88 | 0 | 0 |
| Path 26 | C00065->C00037:[2->1,3->2] | 1.00 | 318.628205128 | 17 | 78 | 0 | 0 |
| Path 27 | C00065->C00037:[2->1,3->2] | 1.00 | 326.702380952 | 17 | 84 | 0 | 0 |
| Path 28 | C00065->C00037:[2->1,3->2] | 1.00 | 351.184210526 | 19 | 76 | 0 | 0 |
| Path 29 | C00065->C00037:[1->1,3->2] | 1.00 | 407.041666667 | 11 | 48 | 0 | 0 |
| Path 30 | C00065->C00037:[1->1,3->2] | 1.00 | 421.081632653 | 12 | 49 | 0 | 0 |
| Path 31 | C00065->C00037:[1->1,3->2] | 1.00 | 347.52173913 | 15 | 69 | 0 | 0 |
| Path 32 | C00065->C00037:[2->1,3->2] | 1.00 | 616.554216867 | 18 | 83 | 0 | 0 |
| Path 33 | C00065->C00037:[2->1,3->2] | 1.00 | 346.987179487 | 20 | 78 | 0 | 0 |
| Path 34 | C00065->C00037:[3->1,3->2] | 1.00 | 376.222222222 | 13 | 36 | 0 | 0 |
| Path 35 | C00065->C00037:[2->1,3->2] | 1.00 | 341.266666667 | 18 | 75 | 0 | 0 |
| Path 36 | C00065->C00037:[2->1,3->2] | 1.00 | 334.385542169 | 16 | 83 | 0 | 0 |
| Path 37 | C00065->C00037:[2->1,3->2] | 1.00 | 309.291666667 | 15 | 72 | 0 | 0 |
| Path 38 | C00065->C00037:[2->1,3->2] | 1.00 | 324.494117647 | 17 | 85 | 0 | 0 |
| Path 39 | C00065->C00037:[2->1,3->2] | 1.00 | 404.854545455 | 11 | 55 | 0 | 0 |
| Path 40 | C00065->C00037:[1->1,3->2] | 1.00 | 360.462686567 | 14 | 67 | 0 | 0 |
| Path 41 | C00065->C00037:[2->1,3->2] | 1.00 | 423.377358491 | 10 | 53 | 0 | 0 |
| Path 42 | C00065->C00037:[2->1,3->2] | 1.00 | 327.695121951 | 20 | 82 | 0 | 0 |
| Path 43 | C00065->C00037:[1->1,3->2] | 1.00 | 396.964285714 | 10 | 28 | 0 | 0 |
| Path 44 | C00065->C00037:[1->1,3->2] | 1.00 | 349.333333333 | 13 | 66 | 0 | 0 |
| Path 45 | C00065->C00037:[2->1,3->2] | 1.00 | 295.111111111 | 17 | 90 | 0 | 0 |
| Path 46 | C00065->C00037:[2->1,3->2] | 1.00 | 424.906976744 | 10 | 43 | 0 | 0 |
| Path 47 | C00065->C00037:[2->1,3->2] | 1.00 | 328.455696203 | 18 | 79 | 0 | 0 |
| Path 48 | C00065->C00037:[3->1,3->2] | 1.00 | 450.666666667 | 12 | 45 | 0 | 0 |
| Path 49 | C00065->C00037:[2->1,3->2] | 1.00 | 342.473684211 | 9 | 38 | 0 | 0 |
| Path 50 | C00065->C00037:[3->1,3->2] | 1.00 | 439.468085106 | 13 | 47 | 0 | 0 |
| Path 51 | C00065->C00037:[1->1,3->2] | 1.00 | 414.538461538 | 14 | 52 | 0 | 0 |
| Path 52 | C00065->C00037:[2->1,3->2] | 1.00 | 338.580246914 | 20 | 81 | 0 | 0 |
| Path 53 | C00065->C00037:[2->1,3->2] | 1.00 | 408.952380952 | 9 | 42 | 0 | 0 |
| Path 54 | C00065->C00037:[2->1,3->2] | 1.00 | 318.222222222 | 19 | 81 | 0 | 0 |
| Path 55 | C00065->C00037:[2->1,3->2] | 1.00 | 345.289156627 | 21 | 83 | 0 | 0 |
| Path 56 | C00065->C00037:[2->1,3->2] | 1.00 | 307.840909091 | 21 | 88 | 0 | 0 |
| Path 57 | C00065->C00037:[2->1,3->2] | 1.00 | 337.272727273 | 19 | 77 | 0 | 0 |
| Path 58 | C00065->C00037:[1->2,2->1] | 1.00 | 368.641791045 | 18 | 67 | 0 | 0 |
| Path 59 | C00065->C00037:[2->1,3->2] | 1.00 | 349.185185185 | 20 | 81 | 0 | 0 |
| Path 60 | C00065->C00037:[1->2,2->1] | 1.00 | 819.0 | 1 | 1 | 0 | 0 |
| Path 61 | C00065->C00037:[2->1,3->2] | 1.00 | 339.8625 | 19 | 80 | 0 | 0 |
| Path 62 | C00065->C00037:[2->1,3->2] | 1.00 | 339.881578947 | 19 | 76 | 0 | 0 |
| Path 63 | C00065->C00037:[1->1,3->2] | 1.00 | 401.196078431 | 13 | 51 | 0 | 0 |
| Path 64 | C00065->C00037:[3->1,3->2] | 1.00 | 385.483870968 | 12 | 31 | 0 | 0 |
| Path 65 | C00065->C00037:[2->1,3->2] | 1.00 | 312.771428571 | 14 | 70 | 0 | 0 |
| Path 66 | C00065->C00037:[3->1,3->2] | 1.00 | 437.48 | 13 | 50 | 0 | 0 |
| Path 67 | C00065->C00037:[2->1,3->2] | 1.00 | 410.461538462 | 9 | 52 | 0 | 0 |
| Path 68 | C00065->C00037:[3->1,3->2] | 1.00 | 450.37254902 | 14 | 51 | 0 | 0 |
| Path 69 | C00065->C00037:[3->1,3->2] | 1.00 | 427.865384615 | 14 | 52 | 0 | 0 |
| Path 70 | C00065->C00037:[2->1,3->2] | 1.00 | 326.325301205 | 16 | 83 | 0 | 0 |
| Path 71 | C00065->C00037:[2->1,3->2] | 1.00 | 613.839506173 | 16 | 81 | 0 | 0 |
| Path 72 | C00065->C00037:[2->1,3->2] | 1.00 | 341.040540541 | 17 | 74 | 0 | 0 |
| Path 73 | C00065->C00037:[3->1,3->2] | 1.00 | 324.513513514 | 17 | 74 | 0 | 0 |
| Path 74 | C00065->C00037:[3->1,3->2] | 1.00 | 438.285714286 | 17 | 42 | 0 | 0 |
| Path 75 | C00065->C00037:[3->1,3->2] | 1.00 | 469.685714286 | 15 | 35 | 0 | 0 |
| Path 76 | C00065->C00037:[1->1,3->2] | 1.00 | 394.648648649 | 13 | 37 | 0 | 0 |
| Path 77 | C00065->C00037:[2->1,3->2] | 1.00 | 324.095238095 | 16 | 84 | 0 | 0 |
| Path 78 | C00065->C00037:[3->1,3->2] | 1.00 | 313.95890411 | 16 | 73 | 0 | 0 |
| Path 79 | C00065->C00037:[2->1,3->2] | 1.00 | 323.3 | 14 | 70 | 0 | 0 |
| Path 80 | C00065->C00037:[1->2,2->1] | 1.00 | 339.04 | 15 | 25 | 0 | 1 |
| Path 81 | C00065->C00037:[3->1,3->2] | 1.00 | 316.388888889 | 16 | 72 | 0 | 0 |
| Path 82 | C00065->C00037:[1->2,2->1] | 1.00 | 686.197368421 | 19 | 76 | 0 | 0 |
| Path 83 | C00065->C00037:[1->2,2->1] | 1.00 | 473.219512195 | 19 | 41 | 0 | 1 |
| Path 84 | C00065->C00037:[1->2,2->1] | 1.00 | 295.688888889 | 18 | 45 | 0 | 1 |
| Path 85 | C00065->C00037:[2->1,3->2] | 1.00 | 327.320987654 | 19 | 81 | 0 | 0 |
| Path 86 | C00065->C00037:[1->2,2->1] | 1.00 | 412.25 | 23 | 56 | 0 | 1 |
| Path 87 | C00065->C00037:[1->2,2->1] | 1.00 | 370.657142857 | 20 | 70 | 0 | 0 |
| Path 88 | C00065->C00037:[3->2] | 0.50 | 395.966666667 | 11 | 30 | 0 | 0 |
| Path 89 | C00065->C00037:[2->1,3->2] | 1.00 | 339.64 | 18 | 75 | 0 | 0 |
| Path 90 | C00065->C00037:[2->1,2->2] | 1.00 | 163.005347594 | 20 | 187 | 0 | 2 |
| Path 91 | C00065->C00037:[1->2,2->1] | 1.00 | 277.818181818 | 15 | 33 | 0 | 1 |
| Path 92 | C00065->C00037:[1->2,2->1] | 1.00 | 382.661016949 | 22 | 59 | 0 | 1 |
| Path 93 | C00065->C00037:[3->2] | 0.50 | 431.073170732 | 9 | 41 | 0 | 0 |
| Path 94 | C00065->C00037:[1->2,2->1] | 1.00 | 537.321428571 | 15 | 28 | 0 | 0 |
| Path 95 | C00065->C00037:[2->1,3->2] | 1.00 | 311.028571429 | 14 | 70 | 0 | 0 |
| Path 96 | C00065->C00037:[1->2,2->1] | 1.00 | 372.779411765 | 19 | 68 | 0 | 0 |
| Path 97 | C00065->C00037:[2->1,3->2] | 1.00 | 416.738095238 | 12 | 42 | 0 | 0 |
| Path 98 | C00065->C00037:[3->2] | 0.50 | 414.475 | 8 | 40 | 0 | 0 |
| Path 99 | C00065->C00037:[3->2] | 0.50 | 409.78125 | 12 | 32 | 0 | 0 |
| Path 100 | C00065->C00037:[3->2] | 0.50 | 401.482758621 | 10 | 29 | 0 | 0 |
| Path 101 | C00065->C00037:[2->1,3->2] | 1.00 | 323.828947368 | 18 | 76 | 0 | 0 |
| Path 102 | C00065->C00037:[2->1,3->2] | 1.00 | 304.743902439 | 15 | 82 | 0 | 0 |
| Path 103 | C00065->C00037:[2->1,3->2] | 1.00 | 294.404494382 | 16 | 89 | 0 | 0 |
| Path 104 | C00065->C00037:[2->1,2->2] | 1.00 | 182.418367347 | 25 | 196 | 0 | 2 |
| Path 105 | C00065->C00037:[2->1,3->2] | 1.00 | 339.632911392 | 18 | 79 | 0 | 0 |
| Path 106 | C00065->C00037:[2->1,3->2] | 1.00 | 334.097560976 | 15 | 82 | 0 | 0 |
| Path 107 | C00065->C00037:[1->2,2->1] | 1.00 | 508.41025641 | 20 | 39 | 0 | 1 |
| Path 108 | C00065->C00037:[2->1,3->2] | 1.00 | 302.746987952 | 15 | 83 | 0 | 0 |
| Path 109 | C00065->C00037:[1->2,2->1] | 1.00 | 437.846153846 | 16 | 26 | 0 | 1 |
| Path 110 | C00065->C00037:[2->1,3->2] | 1.00 | 630.5125 | 16 | 80 | 0 | 0 |
| Path 111 | C00065->C00037:[2->1,3->2] | 1.00 | 265.476190476 | 17 | 84 | 0 | 0 |
| Path 112 | C00065->C00037:[3->1,3->2] | 1.00 | 436.638888889 | 15 | 36 | 0 | 0 |
| Path 113 | C00065->C00037:[2->1,3->2] | 1.00 | 313.875 | 18 | 80 | 0 | 0 |
| Path 114 | C00065->C00037:[3->1,3->2] | 1.00 | 358.276595745 | 17 | 47 | 0 | 0 |
| Path 115 | C00065->C00037:[3->1,3->2] | 1.00 | 434.307692308 | 15 | 39 | 0 | 0 |
| Path 116 | C00065->C00037:[1->1,3->2] | 1.00 | 394.512820513 | 15 | 39 | 0 | 0 |
| Path 117 | C00065->C00037:[2->1,3->2] | 1.00 | 323.518518519 | 19 | 81 | 0 | 0 |
| Path 118 | C00065->C00037:[2->1,2->2] | 1.00 | 163.614035088 | 19 | 171 | 0 | 2 |
| Path 119 | C00065->C00037:[2->1,2->2,3->2] | 1.00 | 186.367875648 | 24 | 193 | 0 | 2 |
| Path 120 | C00065->C00037:[3->2] | 0.50 | 403.666666667 | 9 | 42 | 0 | 0 |
| Path 121 | C00065->C00037:[2->1,3->2] | 1.00 | 297.217948718 | 17 | 78 | 0 | 0 |
| Path 122 | C00065->C00037:[2->1,3->2] | 1.00 | 335.87654321 | 19 | 81 | 0 | 0 |
| Path 123 | C00065->C00037:[1->2,2->1] | 1.00 | 274.458333333 | 9 | 24 | 0 | 1 |
| Path 124 | C00065->C00037:[1->2,2->1] | 1.00 | 383.84 | 15 | 25 | 0 | 1 |
| Path 125 | C00065->C00037:[2->1,3->2] | 1.00 | 307.264367816 | 20 | 87 | 0 | 0 |
| Path 126 | C00065->C00037:[2->1,3->2] | 1.00 | 312.115942029 | 13 | 69 | 0 | 0 |
| Path 127 | C00065->C00037:[2->1,2->2] | 1.00 | 166.529100529 | 21 | 189 | 0 | 2 |
| Path 128 | C00065->C00037:[1->2,2->1] | 1.00 | 367.2 | 21 | 75 | 0 | 0 |
| Path 129 | C00065->C00037:[1->2,2->1] | 1.00 | 487.2 | 9 | 15 | 0 | 1 |
| Path 130 | C00065->C00037:[1->2,2->1] | 1.00 | 413.428571429 | 17 | 28 | 0 | 1 |
| Path 131 | C00065->C00037:[2->1,3->2] | 1.00 | 295.215189873 | 17 | 79 | 0 | 0 |
| Path 132 | C00065->C00037:[3->1] | 0.50 | 141.395061728 | 15 | 162 | 0 | 2 |
| Path 133 | C00065->C00037:[3->1,3->2] | 1.00 | 451.294117647 | 14 | 34 | 0 | 0 |
| Path 134 | C00065->C00037:[1->2,2->1] | 1.00 | 466.19047619 | 15 | 21 | 0 | 1 |
| Path 135 | C00065->C00037:[2->1,3->2] | 1.00 | 346.844155844 | 19 | 77 | 0 | 0 |
| Path 136 | C00065->C00037:[2->1,3->2] | 1.00 | 273.931818182 | 15 | 88 | 0 | 0 |
| Path 137 | C00065->C00037:[1->2,2->1] | 1.00 | 585.451612903 | 18 | 31 | 0 | 0 |
| Path 138 | C00065->C00037:[2->1,3->2] | 1.00 | 317.115384615 | 17 | 78 | 0 | 0 |
| Path 139 | C00065->C00037:[2->1,3->2] | 1.00 | 318.116883117 | 16 | 77 | 0 | 0 |
| Path 140 | C00065->C00037:[1->1,3->2] | 1.00 | 388.125 | 15 | 40 | 0 | 0 |
| Path 141 | C00065->C00037:[2->1,3->2] | 1.00 | 624.632911392 | 15 | 79 | 0 | 0 |
| Path 142 | C00065->C00037:[1->2,2->1] | 1.00 | 283.390243902 | 16 | 41 | 0 | 1 |
| Path 143 | C00065->C00037:[2->1,3->2] | 1.00 | 328.076923077 | 17 | 78 | 0 | 0 |
| Path 144 | C00065->C00037:[2->1,3->2] | 1.00 | 294.723684211 | 15 | 76 | 0 | 0 |
| Path 145 | C00065->C00037:[2->1,3->2] | 1.00 | 319.987341772 | 18 | 79 | 0 | 0 |
| Path 146 | C00065->C00037:[2->1,3->2] | 1.00 | 619.775 | 16 | 80 | 0 | 0 |
| Path 147 | C00065->C00037:[3->2] | 0.50 | 317.269230769 | 8 | 26 | 0 | 0 |
| Path 148 | C00065->C00037:[2->1,3->2] | 1.00 | 394.838709677 | 11 | 31 | 0 | 0 |
| Path 149 | C00065->C00037:[2->1,2->2] | 1.00 | 183.413265306 | 24 | 196 | 0 | 2 |
| Path 150 | C00065->C00037:[3->1,3->2] | 1.00 | 362.976190476 | 16 | 42 | 0 | 0 |
| Path 151 | C00065->C00037:[3->1,3->2] | 1.00 | 317.521126761 | 15 | 71 | 0 | 0 |
| Path 152 | C00065->C00037:[2->1,3->2] | 1.00 | 976.829268293 | 13 | 41 | 0 | 0 |
| Path 153 | C00065->C00037:[2->1,3->2] | 1.00 | 345.134146341 | 20 | 82 | 0 | 0 |
| Path 154 | C00065->C00037:[2->1,3->2] | 1.00 | 382.272727273 | 12 | 33 | 0 | 0 |
| Path 155 | C00065->C00037:[2->1,2->2] | 1.00 | 153.910179641 | 18 | 167 | 0 | 2 |
| Path 156 | C00065->C00037:[1->2,2->1] | 1.00 | 368.4375 | 9 | 16 | 0 | 1 |
| Path 157 | C00065->C00037:[1->2,2->1] | 1.00 | 434.0 | 16 | 23 | 0 | 1 |
| Path 158 | C00065->C00037:[1->2,2->1] | 1.00 | 394.285714286 | 8 | 14 | 0 | 1 |
| Path 159 | C00065->C00037:[2->1,3->2] | 1.00 | 349.075 | 19 | 80 | 0 | 0 |
| Path 160 | C00065->C00037:[2->1,2->2] | 1.00 | 179.869109948 | 23 | 191 | 0 | 2 |
| Path 161 | C00065->C00037:[1->1,3->2] | 1.00 | 405.365853659 | 16 | 41 | 0 | 0 |
| Path 162 | C00065->C00037:[1->2,2->1] | 1.00 | 1127.76315789 | 17 | 38 | 0 | 0 |
| Path 163 | C00065->C00037:[2->1,3->2] | 1.00 | 297.608695652 | 13 | 69 | 0 | 0 |
| Path 164 | C00065->C00037:[2->1,3->2] | 1.00 | 333.164705882 | 17 | 85 | 0 | 0 |
| Path 165 | C00065->C00037:[3->1,3->2] | 1.00 | 454.432432432 | 16 | 37 | 0 | 0 |
| Path 166 | C00065->C00037:[2->1,3->2] | 1.00 | 317.725 | 18 | 80 | 0 | 0 |
| Path 167 | C00065->C00037:[1->2,2->1] | 1.00 | 412.041666667 | 15 | 24 | 0 | 1 |
| Path 168 | C00065->C00037:[2->1,3->2] | 1.00 | 284.469135802 | 18 | 81 | 0 | 0 |
| Path 169 | C00065->C00037:[2->1,3->2] | 1.00 | 566.057471264 | 17 | 87 | 0 | 0 |
| Path 170 | C00065->C00037:[2->1,3->2] | 1.00 | 305.116883117 | 16 | 77 | 0 | 0 |
| Path 171 | C00065->C00037:[1->2,2->1] | 1.00 | 548.416666667 | 19 | 36 | 0 | 0 |
| Path 172 | C00065->C00037:[2->1,3->2] | 1.00 | 403.235294118 | 13 | 34 | 0 | 0 |
| Path 173 | C00065->C00037:[1->2,2->1] | 1.00 | 352.217391304 | 14 | 23 | 0 | 1 |
| Path 174 | C00065->C00037:[3->1,3->2] | 1.00 | 429.35 | 16 | 40 | 0 | 0 |
| Path 175 | C00065->C00037:[2->1,3->2] | 1.00 | 278.402597403 | 15 | 77 | 0 | 0 |
| Path 176 | C00065->C00037:[2->1,3->2] | 1.00 | 393.886363636 | 13 | 44 | 0 | 0 |
| Path 177 | C00065->C00037:[1->2,2->1] | 1.00 | 350.448275862 | 17 | 29 | 0 | 1 |
| Path 178 | C00065->C00037:[2->1,3->2] | 1.00 | 316.088607595 | 18 | 79 | 0 | 0 |
| Path 179 | C00065->C00037:[1->2,2->1] | 1.00 | 293.378378378 | 17 | 37 | 0 | 1 |
| Path 180 | C00065->C00037:[2->1,3->2] | 1.00 | 324.703703704 | 14 | 81 | 0 | 0 |
| Path 181 | C00065->C00037:[1->2,2->1] | 1.00 | 571.4 | 8 | 10 | 0 | 1 |
| Path 182 | C00065->C00037:[3->1,3->2] | 1.00 | 284.113924051 | 17 | 79 | 0 | 0 |
| Path 183 | C00065->C00037:[2->1,3->2] | 1.00 | 283.071428571 | 13 | 70 | 0 | 0 |
| Path 184 | C00065->C00037:[2->1,3->2] | 1.00 | 326.962025316 | 18 | 79 | 0 | 0 |
| Path 185 | C00065->C00037:[2->1,3->2] | 1.00 | 313.546666667 | 17 | 75 | 0 | 0 |
| Path 186 | C00065->C00037:[2->1,3->2] | 1.00 | 319.527777778 | 15 | 72 | 0 | 0 |
| Path 187 | C00065->C00037:[2->1,3->2] | 1.00 | 308.605633803 | 14 | 71 | 0 | 0 |
| Path 188 | C00065->C00037:[1->2,2->1] | 1.00 | 304.657142857 | 16 | 35 | 0 | 1 |
| Path 189 | C00065->C00037:[1->1,3->2] | 1.00 | 413.078947368 | 14 | 38 | 0 | 0 |
| Path 190 | C00065->C00037:[1->2,2->1] | 1.00 | 555.692307692 | 14 | 26 | 0 | 0 |
| Path 191 | C00065->C00037:[1->2,2->1] | 1.00 | 436.578947368 | 14 | 19 | 0 | 1 |
| Path 192 | C00065->C00037:[2->1,2->2] | 1.00 | 158.703488372 | 19 | 172 | 0 | 2 |
| Path 193 | C00065->C00037:[1->2,2->1] | 1.00 | 494.666666667 | 18 | 36 | 0 | 1 |
| Path 194 | C00065->C00037:[1->2,2->1] | 1.00 | 342.230769231 | 16 | 65 | 0 | 0 |
| Path 195 | C00065->C00037:[2->1,3->2] | 1.00 | 400.195121951 | 11 | 41 | 0 | 0 |
| Path 196 | C00065->C00037:[2->1,3->2] | 1.00 | 286.465116279 | 19 | 86 | 0 | 0 |
| Path 197 | C00065->C00037:[2->1,3->2] | 1.00 | 332.836734694 | 13 | 49 | 0 | 0 |
| Path 198 | C00065->C00037:[2->1,2->2,3->2] | 1.00 | 186.586734694 | 24 | 196 | 0 | 2 |
| Path 199 | C00065->C00037:[2->1,2->2,3->2] | 1.00 | 189.712121212 | 25 | 198 | 0 | 2 |
| Path 200 | C00065->C00037:[1->2,3->1] | 1.00 | 169.687150838 | 21 | 179 | 0 | 2 |
| Path 201 | C00065->C00037:[1->2,2->1] | 1.00 | 498.170731707 | 21 | 41 | 0 | 1 |
| Path 202 | C00065->C00037:[1->2,2->1] | 1.00 | 389.947368421 | 22 | 76 | 0 | 0 |
| Path 203 | C00065->C00037:[1->2,2->1] | 1.00 | 421.411764706 | 22 | 51 | 0 | 1 |
| Path 204 | C00065->C00037:[1->2,2->1] | 1.00 | 478.673913043 | 22 | 46 | 0 | 1 |
| Path 205 | C00065->C00037:[2->1,3->2] | 1.00 | 306.56097561 | 19 | 82 | 0 | 0 |
| Path 206 | C00065->C00037:[2->1,3->2] | 1.00 | 327.513513514 | 17 | 74 | 0 | 0 |
| Path 207 | C00065->C00037:[2->1,3->2] | 1.00 | 312.345679012 | 14 | 81 | 0 | 0 |
| Path 208 | C00065->C00037:[3->1,3->2] | 1.00 | 178.050847458 | 21 | 177 | 0 | 2 |
| Path 209 | C00065->C00037:[2->1,3->2] | 1.00 | 311.307692308 | 13 | 39 | 0 | 0 |
| Path 210 | C00065->C00037:[2->1,3->2] | 1.00 | 285.15942029 | 13 | 69 | 0 | 0 |
| Path 211 | C00065->C00037:[2->1,3->2] | 1.00 | 815.326530612 | 15 | 49 | 0 | 0 |
| Path 212 | C00065->C00037:[1->2,2->1] | 1.00 | 368.803030303 | 17 | 66 | 0 | 0 |
| Path 213 | C00065->C00037:[2->1,3->2] | 1.00 | 337.0 | 18 | 76 | 0 | 0 |
| Path 214 | C00065->C00037:[2->1,2->2] | 1.00 | 158.725274725 | 19 | 182 | 0 | 2 |
| Path 215 | C00065->C00037:[2->1,3->2] | 1.00 | 940.11627907 | 14 | 43 | 0 | 0 |
| Path 216 | C00065->C00037:[2->1,3->2] | 1.00 | 254.486842105 | 14 | 76 | 0 | 0 |
| Path 217 | C00065->C00037:[2->1,3->2] | 1.00 | 416.71875 | 12 | 32 | 0 | 0 |
| Path 218 | C00065->C00037:[2->1,2->2,3->2] | 1.00 | 183.12565445 | 23 | 191 | 0 | 2 |
| Path 219 | C00065->C00037:[2->1,3->2] | 1.00 | 959.19047619 | 14 | 42 | 0 | 0 |
| Path 220 | C00065->C00037:[2->1,2->2] | 1.00 | 180.190721649 | 23 | 194 | 0 | 2 |
| Path 221 | C00065->C00037:[2->1,3->2] | 1.00 | 338.3375 | 19 | 80 | 0 | 0 |
| Path 222 | C00065->C00037:[1->2,2->1] | 1.00 | 369.296296296 | 16 | 27 | 0 | 1 |
| Path 223 | C00065->C00037:[2->1,3->2] | 1.00 | 305.2125 | 18 | 80 | 0 | 0 |
| Path 224 | C00065->C00037:[1->2,2->1] | 1.00 | 394.957746479 | 21 | 71 | 0 | 0 |
| Path 225 | C00065->C00037:[3->2] | 0.50 | 424.6 | 11 | 30 | 0 | 0 |
| Path 226 | C00065->C00037:[2->1,2->2] | 1.00 | 176.523809524 | 22 | 189 | 0 | 2 |
| Path 227 | C00065->C00037:[2->1,3->2] | 1.00 | 315.905405405 | 17 | 74 | 0 | 0 |
| Path 228 | C00065->C00037:[2->1,3->2] | 1.00 | 285.882352941 | 12 | 68 | 0 | 0 |
| Path 229 | C00065->C00037:[3->2] | 0.50 | 312.0 | 12 | 37 | 0 | 0 |
| Path 230 | C00065->C00037:[3->2] | 0.50 | 387.677419355 | 11 | 31 | 0 | 0 |
| Path 231 | C00065->C00037:[2->1,3->2] | 1.00 | 317.0 | 16 | 73 | 0 | 0 |
| Path 232 | C00065->C00037:[2->1,3->2] | 1.00 | 399.813953488 | 13 | 43 | 0 | 0 |
| Path 233 | C00065->C00037:[2->1,3->2] | 1.00 | 409.466666667 | 14 | 45 | 0 | 0 |
| Path 234 | C00065->C00037:[3->1,3->2] | 1.00 | 445.142857143 | 15 | 35 | 0 | 0 |
| Path 235 | C00065->C00037:[2->1,2->2] | 1.00 | 168.022727273 | 20 | 176 | 0 | 2 |
| Path 236 | C00065->C00037:[2->1,3->2] | 1.00 | 302.5625 | 13 | 80 | 0 | 0 |
| Path 237 | C00065->C00037:[2->1,3->2] | 1.00 | 287.011764706 | 18 | 85 | 0 | 0 |
| Path 238 | C00065->C00037:[1->2,3->1] | 1.00 | 165.402298851 | 20 | 174 | 0 | 2 |
| Path 239 | C00065->C00037:[3->1,3->2] | 1.00 | 450.825 | 16 | 40 | 0 | 0 |
| Path 240 | C00065->C00037:[1->1,3->2] | 1.00 | 322.288888889 | 15 | 45 | 0 | 0 |
| Path 241 | C00065->C00037:[1->2,2->1] | 1.00 | 377.671875 | 23 | 64 | 0 | 1 |
| Path 242 | C00065->C00037:[2->1,2->2] | 1.00 | 162.391304348 | 20 | 184 | 0 | 2 |
| Path 243 | C00065->C00037:[2->1,3->2] | 1.00 | 979.642857143 | 14 | 42 | 0 | 0 |
| Path 244 | C00065->C00037:[2->1,2->2] | 1.00 | 179.18556701 | 24 | 194 | 0 | 2 |
| Path 245 | C00065->C00037:[3->1,3->2] | 1.00 | 328.319444444 | 16 | 72 | 0 | 0 |
| Path 246 | C00065->C00037:[1->2,2->1] | 1.00 | 304.976744186 | 17 | 43 | 0 | 1 |
| Path 247 | C00065->C00037:[1->2,2->1] | 1.00 | 282.4375 | 10 | 32 | 0 | 1 |
| Path 248 | C00065->C00037:[3->1,3->2] | 1.00 | 422.268292683 | 16 | 41 | 0 | 0 |
| Path 249 | C00065->C00037:[1->2,2->1] | 1.00 | 362.518518519 | 16 | 27 | 0 | 1 |
| Path 250 | C00065->C00037:[2->1,3->2] | 1.00 | 389.875 | 12 | 32 | 0 | 0 |
| Path 251 | C00065->C00037:[2->1,2->2] | 1.00 | 182.693467337 | 25 | 199 | 0 | 2 |
| Path 252 | C00065->C00037:[1->2,2->1] | 1.00 | 485.333333333 | 22 | 45 | 0 | 1 |
| Path 253 | C00065->C00037:[2->1,3->2] | 1.00 | 312.178571429 | 16 | 84 | 0 | 0 |
| Path 254 | C00065->C00037:[3->1,3->2] | 1.00 | 173.959302326 | 20 | 172 | 0 | 2 |
| Path 255 | C00065->C00037:[2->1,3->2] | 1.00 | 294.507042254 | 14 | 71 | 0 | 0 |
| Path 256 | C00065->C00037:[2->1,3->2] | 1.00 | 943.636363636 | 15 | 44 | 0 | 0 |
| Path 257 | C00065->C00037:[2->1,3->2] | 1.00 | 351.093333333 | 18 | 75 | 0 | 0 |
| Path 258 | C00065->C00037:[3->1] | 0.50 | 146.706586826 | 16 | 167 | 0 | 2 |
| Path 259 | C00065->C00037:[2->1,2->2] | 1.00 | 185.810945274 | 26 | 201 | 0 | 2 |
| Path 260 | C00065->C00037:[2->1,3->2] | 1.00 | 619.707317073 | 17 | 82 | 0 | 0 |
