# Supplementary material for: 13C-metabolic flux ratio and novel carbon path analyses confirmed that Trichoderma reesei uses primarily the respirative pathway also on the preferred carbon source glucose
Source: BMC Syst Biol. 2009 Oct 29;3:104. doi: 10.1186/1752-0509-3-104 (PMC2776023; doi:10.1186/1752-0509-3-104)
Supplement: Additional file 1 — Pathways discovered in ReTrace carbon path analysis. Graphical and tabular representations of amino acid synthesis pathways discovered in ReTrace carbon path analysis [21]. Self-contained web site: unpack zip archive and open index.html with a web browser. [file 1752-0509-3-104-S1.zip › AF1-treesei/pathways-C00024-to-C00668.html]

Pathways from C00024 to C00668


**Pathways from C00024 to C00668**

**Sources:** Acetyl-CoA; (C00024)

**Target:**alpha-D-Glucose 6-phosphate (C00668)

|  | Composite mapping | Z | Average score | Rpairs | Reactions | Zero scores | Scores under threshold |
| --- | --- | --- | --- | --- | --- | --- | --- |
| Path 1 | C00024->C00668:[49->10,49->13,49->7,49->8,50->12,50->6] | 1.00 | 391.946666667 | 22 | 75 | 0 | 1 |
| Path 2 | C00024->C00668:[49->10,49->13,49->7,49->8,50->12,50->6] | 1.00 | 344.744 | 27 | 125 | 0 | 1 |
| Path 3 | C00024->C00668:[49->10,49->13,49->7,49->8,50->12,50->6] | 1.00 | 352.506493506 | 25 | 77 | 0 | 1 |
| Path 4 | C00024->C00668:[10->7,11->8,15->6,49->13,50->12,6->10] | 1.00 | 332.938931298 | 14 | 131 | 0 | 0 |
| Path 5 | C00024->C00668:[49->10,49->13,49->7,49->8,50->12,50->6] | 1.00 | 385.458823529 | 29 | 85 | 0 | 1 |
| Path 6 | C00024->C00668:[10->7,11->8,15->6,49->12,50->13,6->10] | 1.00 | 334.053030303 | 15 | 132 | 0 | 0 |
| Path 7 | C00024->C00668:[49->13,49->7,49->8,50->10,50->12,50->6] | 1.00 | 433.293478261 | 28 | 92 | 0 | 1 |
| Path 8 | C00024->C00668:[10->7,11->8,15->6,49->13,50->12,6->10] | 1.00 | 346.930769231 | 15 | 130 | 0 | 0 |
| Path 9 | C00024->C00668:[49->10,49->13,49->7,49->8,50->12,50->6] | 1.00 | 362.264367816 | 24 | 87 | 0 | 1 |
| Path 10 | C00024->C00668:[49->10,49->13,49->7,49->8,50->12,50->6] | 1.00 | 368.290322581 | 26 | 93 | 0 | 1 |
| Path 11 | C00024->C00668:[49->10,49->13,49->7,49->8,50->12,50->6] | 1.00 | 396.666666667 | 24 | 81 | 0 | 1 |
| Path 12 | C00024->C00668:[10->7,11->8,15->6,49->13,50->12,6->10] | 1.00 | 337.234848485 | 14 | 132 | 0 | 0 |
| Path 13 | C00024->C00668:[49->13,49->7,49->8,50->10,50->12,50->6] | 1.00 | 429.989010989 | 27 | 91 | 0 | 1 |
| Path 14 | C00024->C00668:[49->10,49->13,49->7,50->12,50->6,50->8] | 1.00 | 422.725 | 26 | 80 | 0 | 0 |
| Path 15 | C00024->C00668:[49->10,49->13,49->7,49->8,50->12,50->6] | 1.00 | 351.694736842 | 28 | 190 | 0 | 1 |
| Path 16 | C00024->C00668:[49->13,49->7,49->8,50->10,50->12,50->6] | 1.00 | 519.731343284 | 27 | 67 | 0 | 1 |
| Path 17 | C00024->C00668:[2->10,49->13,49->7,49->8,50->12,50->6] | 1.00 | 370.68503937 | 27 | 127 | 0 | 1 |
| Path 18 | C00024->C00668:[49->10,49->13,49->7,49->8,50->12,50->6] | 1.00 | 332.446236559 | 29 | 186 | 0 | 1 |
| Path 19 | C00024->C00668:[2->10,49->13,49->7,49->8,50->12,50->6] | 1.00 | 367.485875706 | 26 | 177 | 0 | 1 |
| Path 20 | C00024->C00668:[14->8,49->10,49->13,49->7,50->12,50->6] | 1.00 | 328.127659574 | 29 | 188 | 0 | 0 |
| Path 21 | C00024->C00668:[49->10,49->13,49->7,49->8,50->12,50->6] | 1.00 | 436.892857143 | 28 | 84 | 0 | 1 |
| Path 22 | C00024->C00668:[49->13,49->7,49->8,50->10,50->12,50->6] | 1.00 | 374.32183908 | 30 | 87 | 0 | 1 |
| Path 23 | C00024->C00668:[49->13,49->7,49->8,50->10,50->12,50->6] | 1.00 | 525.295081967 | 24 | 61 | 0 | 1 |
| Path 24 | C00024->C00668:[49->10,49->13,49->7,49->8,50->12,50->6] | 1.00 | 343.788732394 | 23 | 71 | 0 | 1 |
| Path 25 | C00024->C00668:[49->13,49->7,49->8,50->10,50->12,50->6] | 1.00 | 351.488372093 | 29 | 86 | 0 | 1 |
| Path 26 | C00024->C00668:[49->10,49->13,49->7,49->8,50->12,50->6] | 1.00 | 354.87755102 | 30 | 196 | 0 | 1 |
| Path 27 | C00024->C00668:[14->8,49->10,49->13,49->7,50->12,50->6] | 1.00 | 346.673796791 | 27 | 187 | 0 | 0 |
| Path 28 | C00024->C00668:[49->10,49->13,49->7,49->8,50->12,50->6] | 1.00 | 370.837837838 | 25 | 74 | 0 | 0 |
| Path 29 | C00024->C00668:[49->13,49->7,49->8,50->10,50->12,50->6] | 1.00 | 415.717391304 | 28 | 92 | 0 | 1 |
| Path 30 | C00024->C00668:[49->10,49->13,49->7,49->8,50->12,50->6] | 1.00 | 420.178082192 | 23 | 73 | 0 | 0 |
| Path 31 | C00024->C00668:[10->7,11->8,15->6,49->13,50->12,6->10] | 1.00 | 329.038461538 | 13 | 130 | 0 | 0 |
| Path 32 | C00024->C00668:[49->10,49->13,49->7,49->8,50->12,50->6] | 1.00 | 515.265625 | 24 | 64 | 0 | 0 |
| Path 33 | C00024->C00668:[14->8,49->10,49->13,49->7,50->12,50->6] | 1.00 | 356.421319797 | 30 | 197 | 0 | 0 |
| Path 34 | C00024->C00668:[49->13,49->7,49->8,50->10,50->12,50->6] | 1.00 | 362.511111111 | 30 | 90 | 0 | 1 |
| Path 35 | C00024->C00668:[10->7,11->8,15->6,49->13,50->12,6->10] | 1.00 | 341.015037594 | 15 | 133 | 0 | 0 |
| Path 36 | C00024->C00668:[49->12,49->7,49->8,50->10,50->13,50->6] | 1.00 | 501.050847458 | 22 | 59 | 0 | 1 |
| Path 37 | C00024->C00668:[2->10,49->13,49->7,49->8,50->12,50->6] | 1.00 | 366.238095238 | 26 | 126 | 0 | 1 |
| Path 38 | C00024->C00668:[49->13,49->7,49->8,50->10,50->12,50->6] | 1.00 | 496.764705882 | 26 | 68 | 0 | 1 |
| Path 39 | C00024->C00668:[49->13,49->7,49->8,50->10,50->12,50->6] | 1.00 | 510.619047619 | 26 | 63 | 0 | 1 |
| Path 40 | C00024->C00668:[49->10,49->13,49->7,49->8,50->12,50->6] | 1.00 | 468.526315789 | 25 | 57 | 0 | 0 |
| Path 41 | C00024->C00668:[10->7,11->8,15->6,49->13,50->12,6->10] | 1.00 | 329.474074074 | 14 | 135 | 0 | 0 |
| Path 42 | C00024->C00668:[10->7,11->8,15->6,49->13,50->12,6->10] | 1.00 | 337.364963504 | 15 | 137 | 0 | 0 |
| Path 43 | C00024->C00668:[49->13,49->7,49->8,50->10,50->12,50->6] | 1.00 | 429.869565217 | 28 | 92 | 0 | 1 |
| Path 44 | C00024->C00668:[49->10,49->13,49->7,49->8,50->12,50->6] | 1.00 | 532.964285714 | 23 | 56 | 0 | 0 |
| Path 45 | C00024->C00668:[49->13,49->7,49->8,50->10,50->12,50->6] | 1.00 | 426.527472527 | 27 | 91 | 0 | 1 |
| Path 46 | C00024->C00668:[49->10,49->13,49->7,49->8,50->12,50->6] | 1.00 | 390.189873418 | 28 | 79 | 0 | 1 |
| Path 47 | C00024->C00668:[49->10,49->13,49->7,49->8,50->12,50->6] | 1.00 | 394.544303797 | 27 | 79 | 0 | 0 |
| Path 48 | C00024->C00668:[49->13,49->7,49->8,50->10,50->12,50->6] | 1.00 | 406.6875 | 24 | 80 | 0 | 1 |
| Path 49 | C00024->C00668:[49->10,49->13,49->7,49->8,50->12,50->6] | 1.00 | 507.183333333 | 24 | 60 | 0 | 1 |
| Path 50 | C00024->C00668:[49->13,49->7,49->8,50->10,50->12,50->6] | 1.00 | 411.466666667 | 26 | 90 | 0 | 1 |
| Path 51 | C00024->C00668:[10->7,11->8,15->6,49->13,50->12,6->10] | 1.00 | 337.456692913 | 14 | 127 | 0 | 0 |
| Path 52 | C00024->C00668:[49->13,49->7,49->8,50->10,50->12,50->6] | 1.00 | 406.123595506 | 25 | 89 | 0 | 1 |
| Path 53 | C00024->C00668:[49->13,49->7,49->8,50->10,50->12,50->6] | 1.00 | 515.784615385 | 26 | 65 | 0 | 1 |
| Path 54 | C00024->C00668:[49->10,49->13,49->7,49->8,50->12,50->6] | 1.00 | 415.756410256 | 24 | 78 | 0 | 0 |
| Path 55 | C00024->C00668:[49->10,49->13,49->7,49->8,50->12,50->6] | 1.00 | 345.473684211 | 24 | 76 | 0 | 1 |
| Path 56 | C00024->C00668:[49->10,49->13,49->7,49->8,50->12,50->6] | 1.00 | 333.413612565 | 30 | 191 | 0 | 1 |
| Path 57 | C00024->C00668:[49->10,49->13,49->7,49->8,50->12,50->6] | 1.00 | 349.047619048 | 28 | 126 | 0 | 1 |
| Path 58 | C00024->C00668:[49->10,49->13,49->7,49->8,50->12,50->6] | 1.00 | 375.356321839 | 24 | 87 | 0 | 0 |
| Path 59 | C00024->C00668:[10->7,11->8,15->6,49->13,50->12,6->10] | 1.00 | 304.206896552 | 13 | 145 | 0 | 0 |
| Path 60 | C00024->C00668:[49->13,49->7,49->8,50->10,50->12,50->6] | 1.00 | 421.411111111 | 26 | 90 | 0 | 1 |
| Path 61 | C00024->C00668:[49->10,49->13,49->7,49->8,50->12,50->6] | 1.00 | 410.246753247 | 23 | 77 | 0 | 0 |
| Path 62 | C00024->C00668:[10->7,11->8,15->6,49->13,50->12,6->10] | 1.00 | 347.703703704 | 16 | 135 | 0 | 0 |
| Path 63 | C00024->C00668:[49->13,49->7,49->8,50->10,50->12,50->6] | 1.00 | 509.852459016 | 24 | 61 | 0 | 1 |
| Path 64 | C00024->C00668:[49->10,49->13,49->7,49->8,50->12,50->6] | 1.00 | 362.652173913 | 25 | 92 | 0 | 1 |
| Path 65 | C00024->C00668:[10->7,11->8,15->6,49->13,50->12,6->10] | 1.00 | 333.297709924 | 14 | 131 | 0 | 0 |
| Path 66 | C00024->C00668:[49->10,49->13,49->7,49->8,50->12,50->6] | 1.00 | 418.909090909 | 27 | 88 | 0 | 1 |
| Path 67 | C00024->C00668:[49->13,49->7,49->8,50->10,50->12,50->6] | 1.00 | 408.931818182 | 24 | 88 | 0 | 1 |
| Path 68 | C00024->C00668:[2->10,49->10,49->13,49->7,50->12,50->6,50->8,6->8] | 1.00 | 337.455621302 | 26 | 169 | 0 | 0 |
| Path 69 | C00024->C00668:[49->13,49->7,49->8,50->10,50->12,50->6] | 1.00 | 530.370967742 | 25 | 62 | 0 | 1 |
| Path 70 | C00024->C00668:[49->10,49->13,49->7,49->8,50->12,50->6] | 1.00 | 375.719512195 | 23 | 82 | 0 | 0 |
| Path 71 | C00024->C00668:[49->13,49->7,49->8,50->10,50->12,50->6] | 1.00 | 510.62295082 | 24 | 61 | 0 | 1 |
| Path 72 | C00024->C00668:[10->7,11->8,15->6,49->10,49->13,50->12] | 1.00 | 358.767605634 | 22 | 142 | 0 | 0 |
| Path 73 | C00024->C00668:[49->10,49->13,49->7,49->8,50->12,50->6] | 1.00 | 431.469879518 | 27 | 83 | 0 | 1 |
| Path 74 | C00024->C00668:[49->10,49->13,49->7,49->8,50->12,50->6] | 1.00 | 380.126582278 | 27 | 79 | 0 | 1 |
| Path 75 | C00024->C00668:[49->13,49->7,49->8,50->10,50->12,50->6] | 1.00 | 509.984375 | 25 | 64 | 0 | 1 |
| Path 76 | C00024->C00668:[49->13,49->7,49->8,50->10,50->12,50->6] | 1.00 | 419.468085106 | 27 | 94 | 0 | 1 |
| Path 77 | C00024->C00668:[49->13,49->7,49->8,50->10,50->12,50->6] | 1.00 | 400.138297872 | 29 | 94 | 0 | 1 |
| Path 78 | C00024->C00668:[49->10,49->13,49->7,49->8,50->12,50->6] | 1.00 | 319.638554217 | 25 | 83 | 0 | 1 |
| Path 79 | C00024->C00668:[49->13,49->7,49->8,50->10,50->12,50->6] | 1.00 | 415.010989011 | 27 | 91 | 0 | 1 |
| Path 80 | C00024->C00668:[49->13,49->7,49->8,50->10,50->12,50->6] | 1.00 | 413.619565217 | 28 | 92 | 0 | 1 |
| Path 81 | C00024->C00668:[10->7,11->8,15->6,49->10,49->13,50->12] | 1.00 | 362.769230769 | 23 | 143 | 0 | 0 |
| Path 82 | C00024->C00668:[49->10,49->13,49->7,49->8,50->12,50->6] | 1.00 | 333.361445783 | 25 | 83 | 0 | 0 |
| Path 83 | C00024->C00668:[49->13,49->7,49->8,50->10,50->12,50->6] | 1.00 | 491.641791045 | 25 | 67 | 0 | 1 |
| Path 84 | C00024->C00668:[49->10,49->13,49->7,49->8,50->12,50->6] | 1.00 | 379.488095238 | 28 | 84 | 0 | 1 |
| Path 85 | C00024->C00668:[49->13,49->7,49->8,50->10,50->12,50->6] | 1.00 | 515.061538462 | 26 | 65 | 0 | 1 |
| Path 86 | C00024->C00668:[49->10,49->13,49->7,49->8,50->12,50->6] | 1.00 | 312.577586207 | 27 | 116 | 0 | 1 |
| Path 87 | C00024->C00668:[10->7,11->8,15->6,49->13,50->12,6->10] | 1.00 | 350.433070866 | 14 | 127 | 0 | 0 |
| Path 88 | C00024->C00668:[49->10,49->13,49->7,50->12,50->6,50->8] | 1.00 | 439.505882353 | 28 | 85 | 0 | 0 |
| Path 89 | C00024->C00668:[49->10,49->13,49->7,49->8,50->12,50->6] | 1.00 | 336.296875 | 31 | 192 | 0 | 1 |
| Path 90 | C00024->C00668:[49->10,49->13,49->7,49->8,50->12,50->6] | 1.00 | 364.410958904 | 24 | 73 | 0 | 0 |
| Path 91 | C00024->C00668:[49->10,49->13,49->7,49->8,50->12,50->6] | 1.00 | 418.931506849 | 23 | 73 | 0 | 0 |
| Path 92 | C00024->C00668:[49->10,49->13,49->7,49->8,50->12,50->6] | 1.00 | 391.594594595 | 27 | 74 | 0 | 1 |
| Path 93 | C00024->C00668:[49->10,49->13,49->7,49->8,50->12,50->6] | 1.00 | 519.557377049 | 24 | 61 | 0 | 0 |
| Path 94 | C00024->C00668:[10->7,11->8,15->6,49->13,50->12,6->10] | 1.00 | 342.744186047 | 14 | 129 | 0 | 0 |
| Path 95 | C00024->C00668:[10->7,11->8,15->6,49->13,50->12,6->10] | 1.00 | 346.174603175 | 13 | 126 | 0 | 0 |
| Path 96 | C00024->C00668:[49->13,49->7,49->8,50->10,50->12,50->6] | 1.00 | 391.277777778 | 28 | 90 | 0 | 1 |
| Path 97 | C00024->C00668:[49->10,49->13,49->7,49->8,50->12,50->6] | 1.00 | 434.457831325 | 26 | 83 | 0 | 0 |
| Path 98 | C00024->C00668:[49->13,49->7,49->8,50->10,50->12,50->6] | 1.00 | 412.67032967 | 29 | 91 | 0 | 1 |
| Path 99 | C00024->C00668:[49->10,49->13,49->7,49->8,50->12,50->6] | 1.00 | 428.402439024 | 25 | 82 | 0 | 0 |
| Path 100 | C00024->C00668:[49->10,49->13,49->7,49->8,50->12,50->6] | 1.00 | 496.584615385 | 25 | 65 | 0 | 1 |
| Path 101 | C00024->C00668:[49->10,49->13,49->7,49->8,50->12,50->6] | 1.00 | 314.925619835 | 28 | 121 | 0 | 1 |
| Path 102 | C00024->C00668:[49->10,49->13,49->7,49->8,50->12,50->6] | 1.00 | 416.923076923 | 24 | 78 | 0 | 0 |
| Path 103 | C00024->C00668:[49->10,49->13,49->7,49->8,50->12,50->6] | 1.00 | 447.393442623 | 26 | 61 | 0 | 1 |
| Path 104 | C00024->C00668:[10->7,11->8,15->6,49->13,50->12,6->10] | 1.00 | 350.062992126 | 14 | 127 | 0 | 0 |
| Path 105 | C00024->C00668:[49->10,49->13,49->7,49->8,50->12,50->6] | 1.00 | 322.465909091 | 26 | 88 | 0 | 1 |
| Path 106 | C00024->C00668:[49->10,49->13,49->7,49->8,50->12,50->6] | 1.00 | 435.614457831 | 26 | 83 | 0 | 0 |
| Path 107 | C00024->C00668:[49->10,49->13,49->7,49->8,50->12,50->6] | 1.00 | 372.067567568 | 25 | 74 | 0 | 0 |
| Path 108 | C00024->C00668:[49->10,49->13,49->7,49->8,50->12,50->6] | 1.00 | 343.716666667 | 26 | 120 | 0 | 1 |
| Path 109 | C00024->C00668:[49->10,49->13,49->7,50->12,50->6,50->8] | 1.00 | 379.355263158 | 27 | 76 | 0 | 0 |
| Path 110 | C00024->C00668:[10->7,11->8,15->6,49->13,50->12,6->10] | 1.00 | 342.671641791 | 15 | 134 | 0 | 0 |
| Path 111 | C00024->C00668:[49->10,49->13,49->7,49->8,50->12,50->6] | 1.00 | 439.333333333 | 25 | 60 | 0 | 1 |
| Path 112 | C00024->C00668:[49->10,49->13,49->7,49->8,50->12,50->6] | 1.00 | 413.083333333 | 22 | 72 | 0 | 0 |
| Path 113 | C00024->C00668:[49->13,49->7,49->8,50->10,50->12,50->6] | 1.00 | 424.020833333 | 29 | 96 | 0 | 1 |
| Path 114 | C00024->C00668:[49->10,49->13,49->7,49->8,50->12,50->6] | 1.00 | 328.808988764 | 27 | 89 | 0 | 1 |
| Path 115 | C00024->C00668:[49->13,49->7,49->8,50->10,50->12,50->6] | 1.00 | 509.873015873 | 26 | 63 | 0 | 1 |
| Path 116 | C00024->C00668:[49->10,49->13,49->7,49->8,50->12,50->6] | 1.00 | 435.448717949 | 26 | 78 | 0 | 1 |
| Path 117 | C00024->C00668:[49->13,49->7,49->8,50->10,50->12,50->6] | 1.00 | 422.778947368 | 28 | 95 | 0 | 1 |
| Path 118 | C00024->C00668:[49->10,49->13,49->7,49->8,50->12,50->6] | 1.00 | 433.361445783 | 26 | 83 | 0 | 0 |
| Path 119 | C00024->C00668:[49->10,49->13,49->7,49->8,50->12,50->6] | 1.00 | 439.858974359 | 25 | 78 | 0 | 0 |
| Path 120 | C00024->C00668:[10->7,11->8,15->6,49->13,50->12,6->10] | 1.00 | 341.75 | 15 | 128 | 0 | 0 |
| Path 121 | C00024->C00668:[49->10,49->13,49->7,49->8,50->12,50->6] | 1.00 | 421.891566265 | 26 | 83 | 0 | 1 |
| Path 122 | C00024->C00668:[49->10,49->13,49->7,49->8,50->12,50->6] | 1.00 | 424.168539326 | 28 | 89 | 0 | 1 |
| Path 123 | C00024->C00668:[10->7,11->8,15->6,49->13,50->12,6->10] | 1.00 | 341.3828125 | 15 | 128 | 0 | 0 |
| Path 124 | C00024->C00668:[49->13,49->7,49->8,50->10,50->12,50->6] | 1.00 | 371.551724138 | 30 | 87 | 0 | 1 |
| Path 125 | C00024->C00668:[49->10,49->13,49->7,49->8,50->12,50->6] | 1.00 | 489.796875 | 24 | 64 | 0 | 1 |
| Path 126 | C00024->C00668:[49->13,49->7,49->8,50->10,50->12,50->6] | 1.00 | 531.129032258 | 25 | 62 | 0 | 1 |
| Path 127 | C00024->C00668:[10->7,11->8,15->6,49->13,50->12,6->10] | 1.00 | 307.876712329 | 14 | 146 | 0 | 0 |
| Path 128 | C00024->C00668:[49->10,49->13,49->7,49->8,50->12,50->6] | 1.00 | 396.4 | 29 | 80 | 0 | 1 |
| Path 129 | C00024->C00668:[49->10,49->13,49->7,49->8,50->12,50->6] | 1.00 | 352.148717949 | 29 | 195 | 0 | 1 |
| Path 130 | C00024->C00668:[10->7,11->8,15->6,49->13,50->12,6->10] | 1.00 | 351.492424242 | 16 | 132 | 0 | 0 |
| Path 131 | C00024->C00668:[2->10,49->13,49->7,49->8,50->12,50->6] | 1.00 | 364.284090909 | 25 | 176 | 0 | 1 |
| Path 132 | C00024->C00668:[49->10,49->13,49->7,49->8,50->12,50->6] | 1.00 | 319.614754098 | 29 | 122 | 0 | 1 |
| Path 133 | C00024->C00668:[49->13,49->7,49->8,50->10,50->12,50->6] | 1.00 | 504.548387097 | 25 | 62 | 0 | 1 |
| Path 134 | C00024->C00668:[49->13,49->7,49->8,50->10,50->12,50->6] | 1.00 | 410.021978022 | 29 | 91 | 0 | 1 |
| Path 135 | C00024->C00668:[49->10,49->13,49->7,50->12,50->6,50->8] | 1.00 | 426.28 | 25 | 75 | 0 | 0 |
| Path 136 | C00024->C00668:[49->13,49->7,49->8,50->10,50->12,50->6] | 1.00 | 414.440860215 | 26 | 93 | 0 | 1 |
| Path 137 | C00024->C00668:[49->13,49->7,49->8,50->10,50->12,50->6] | 1.00 | 408.417582418 | 27 | 91 | 0 | 1 |
| Path 138 | C00024->C00668:[49->10,49->13,49->7,49->8,50->12,50->6] | 1.00 | 451.090909091 | 28 | 88 | 0 | 0 |
| Path 139 | C00024->C00668:[10->7,11->8,15->6,49->13,50->12,6->10] | 1.00 | 341.368421053 | 15 | 133 | 0 | 0 |
| Path 140 | C00024->C00668:[49->10,49->13,49->7,49->8,50->12,50->6] | 1.00 | 500.0 | 23 | 59 | 0 | 1 |
| Path 141 | C00024->C00668:[49->10,49->13,49->7,49->8,50->12,50->6] | 1.00 | 390.5375 | 23 | 80 | 0 | 1 |
| Path 142 | C00024->C00668:[14->8,49->10,49->13,49->7,50->12,50->6] | 1.00 | 347.265625 | 28 | 192 | 0 | 0 |
| Path 143 | C00024->C00668:[49->10,49->13,49->7,49->8,50->12,50->6] | 1.00 | 393.434782609 | 26 | 92 | 0 | 0 |
| Path 144 | C00024->C00668:[49->13,49->7,49->8,50->10,50->12,50->6] | 1.00 | 424.911111111 | 26 | 90 | 0 | 1 |
| Path 145 | C00024->C00668:[49->13,49->7,49->8,50->10,50->12,50->6] | 1.00 | 497.455882353 | 26 | 68 | 0 | 1 |
| Path 146 | C00024->C00668:[49->13,49->7,49->8,50->10,50->12,50->6] | 1.00 | 504.35 | 23 | 60 | 0 | 1 |
| Path 147 | C00024->C00668:[49->13,49->7,49->8,50->10,50->12,50->6] | 1.00 | 417.064516129 | 29 | 93 | 0 | 1 |
| Path 148 | C00024->C00668:[10->7,11->8,15->6,49->13,50->12,6->10] | 1.00 | 346.569230769 | 15 | 130 | 0 | 0 |
| Path 149 | C00024->C00668:[49->13,49->7,50->12,50->6] | 0.67 | 417.68852459 | 16 | 61 | 0 | 0 |
| Path 150 | C00024->C00668:[49->12,49->7,50->10,50->13,50->6] | 0.83 | 517.403846154 | 19 | 52 | 0 | 1 |
| Path 151 | C00024->C00668:[49->13,50->12,50->6] | 0.50 | 581.477272727 | 20 | 44 | 0 | 0 |
| Path 152 | C00024->C00668:[49->13,49->7,50->12,50->6] | 0.67 | 435.88372093 | 15 | 43 | 0 | 0 |
| Path 153 | C00024->C00668:[2->10,49->13,50->12] | 0.50 | 316.901960784 | 21 | 102 | 0 | 0 |
| Path 154 | C00024->C00668:[49->13,49->7,50->12,50->6] | 0.67 | 515.728813559 | 20 | 59 | 0 | 1 |
| Path 155 | C00024->C00668:[49->7,49->8,50->6] | 0.50 | 364.301204819 | 21 | 166 | 0 | 1 |
| Path 156 | C00024->C00668:[49->13,49->7,50->12,50->6] | 0.67 | 495.222222222 | 19 | 63 | 0 | 1 |
| Path 157 | C00024->C00668:[10->7,11->8,15->6,2->10] | 0.67 | 360.169230769 | 16 | 130 | 0 | 0 |
| Path 158 | C00024->C00668:[2->10,49->13,50->12] | 0.50 | 320.24 | 18 | 150 | 0 | 0 |
| Path 159 | C00024->C00668:[49->13,49->7,49->8,50->12,50->6] | 0.83 | 349.230769231 | 25 | 130 | 0 | 1 |
| Path 160 | C00024->C00668:[49->13,49->7,50->12,50->6] | 0.67 | 515.912280702 | 18 | 57 | 0 | 1 |
| Path 161 | C00024->C00668:[2->10,49->13,50->12] | 0.50 | 348.0 | 16 | 131 | 0 | 0 |
| Path 162 | C00024->C00668:[49->13,49->7,49->8,50->12,50->6] | 0.83 | 389.052631579 | 25 | 76 | 0 | 1 |
| Path 163 | C00024->C00668:[49->13,49->7,49->8,50->12,50->6] | 0.83 | 444.872093023 | 30 | 86 | 0 | 1 |
| Path 164 | C00024->C00668:[49->7,49->8,50->6] | 0.50 | 364.333333333 | 18 | 93 | 0 | 1 |
| Path 165 | C00024->C00668:[49->13,49->7,49->8,50->10,50->12,50->6] | 1.00 | 542.619047619 | 26 | 63 | 0 | 1 |
| Path 166 | C00024->C00668:[49->13,50->12] | 0.33 | 901.571428571 | 7 | 7 | 0 | 0 |
| Path 167 | C00024->C00668:[49->13,49->7,49->8,50->12,50->6] | 0.83 | 501.0 | 21 | 64 | 0 | 1 |
| Path 168 | C00024->C00668:[49->13,49->7,50->12,50->6] | 0.67 | 531.614035088 | 18 | 57 | 0 | 1 |
| Path 169 | C00024->C00668:[49->13,49->7,50->10,50->12,50->6] | 0.83 | 520.454545455 | 22 | 55 | 0 | 1 |
| Path 170 | C00024->C00668:[49->7,49->8,50->6] | 0.50 | 402.566666667 | 16 | 60 | 0 | 1 |
| Path 171 | C00024->C00668:[49->13,49->7,50->12,50->6] | 0.67 | 407.224489796 | 18 | 49 | 0 | 1 |
| Path 172 | C00024->C00668:[49->13,49->7,49->8,50->12,50->6] | 0.83 | 486.716666667 | 20 | 60 | 0 | 1 |
| Path 173 | C00024->C00668:[14->8,49->7,50->6] | 0.50 | 358.613496933 | 19 | 163 | 0 | 1 |
| Path 174 | C00024->C00668:[49->13,50->12] | 0.33 | 530.666666667 | 7 | 12 | 0 | 0 |
| Path 175 | C00024->C00668:[14->8,49->7,50->6] | 0.50 | 355.351851852 | 18 | 162 | 0 | 1 |
| Path 176 | C00024->C00668:[14->8,49->13,49->7,50->12,50->6] | 0.83 | 347.49726776 | 23 | 183 | 0 | 0 |
| Path 177 | C00024->C00668:[49->13,49->7,49->8,50->12,50->6] | 0.83 | 494.873015873 | 20 | 63 | 0 | 1 |
| Path 178 | C00024->C00668:[49->13,49->7,49->8,50->12,50->6] | 0.83 | 336.678571429 | 28 | 196 | 0 | 1 |
| Path 179 | C00024->C00668:[49->13,50->12] | 0.33 | 678.076923077 | 9 | 13 | 0 | 0 |
| Path 180 | C00024->C00668:[49->13,49->7,49->8,50->10,50->12,50->6] | 1.00 | 419.710526316 | 23 | 76 | 0 | 1 |
| Path 181 | C00024->C00668:[49->7,49->8,50->6] | 0.50 | 373.829268293 | 14 | 41 | 0 | 1 |
| Path 182 | C00024->C00668:[49->13,49->7,49->8,50->12,50->6] | 0.83 | 517.393442623 | 21 | 61 | 0 | 1 |
| Path 183 | C00024->C00668:[49->13,49->7,50->12,50->6] | 0.67 | 330.076923077 | 17 | 65 | 0 | 1 |
| Path 184 | C00024->C00668:[14->8,49->7,50->6] | 0.50 | 364.83125 | 19 | 160 | 0 | 0 |
| Path 185 | C00024->C00668:[49->13,49->7,50->12,50->6,50->8] | 0.83 | 425.0 | 24 | 85 | 0 | 0 |
| Path 186 | C00024->C00668:[49->13,49->7,50->12,50->6] | 0.67 | 390.414285714 | 17 | 70 | 0 | 1 |
| Path 187 | C00024->C00668:[10->7,11->8,15->6,2->10,49->13,50->12] | 1.00 | 311.08372093 | 22 | 215 | 0 | 0 |
| Path 188 | C00024->C00668:[49->13,49->7,49->8,50->12,50->6] | 0.83 | 431.9375 | 24 | 80 | 0 | 1 |
| Path 189 | C00024->C00668:[49->13,49->7,49->8,50->12,50->6] | 0.83 | 500.820895522 | 22 | 67 | 0 | 1 |
| Path 190 | C00024->C00668:[49->7,49->8,50->6] | 0.50 | 364.916666667 | 21 | 168 | 0 | 1 |
| Path 191 | C00024->C00668:[10->7,11->8,15->6,2->10] | 0.67 | 326.760416667 | 18 | 192 | 0 | 0 |
| Path 192 | C00024->C00668:[2->10,49->13,49->7,50->12,50->6] | 0.83 | 343.888198758 | 22 | 161 | 0 | 0 |
| Path 193 | C00024->C00668:[49->13,49->7,50->12,50->6,50->8] | 0.83 | 370.652777778 | 23 | 72 | 0 | 0 |
| Path 194 | C00024->C00668:[49->13,49->7,49->8,50->12,50->6] | 0.83 | 450.459770115 | 31 | 87 | 0 | 1 |
| Path 195 | C00024->C00668:[49->13,49->7,50->10,50->12,50->6] | 0.83 | 520.830188679 | 20 | 53 | 0 | 1 |
| Path 196 | C00024->C00668:[49->13,49->7,49->8,50->12,50->6] | 0.83 | 492.145833333 | 20 | 48 | 0 | 0 |
| Path 197 | C00024->C00668:[49->7,49->8,50->6] | 0.50 | 342.06779661 | 18 | 59 | 0 | 1 |
| Path 198 | C00024->C00668:[49->13,49->7,49->8,50->12,50->6] | 0.83 | 522.507936508 | 21 | 63 | 0 | 1 |
| Path 199 | C00024->C00668:[49->13,49->7,49->8,50->12,50->6] | 0.83 | 354.88 | 27 | 200 | 0 | 1 |
| Path 200 | C00024->C00668:[49->13,49->7,49->8,50->12,50->6] | 0.83 | 527.46875 | 22 | 64 | 0 | 1 |
| Path 201 | C00024->C00668:[49->13,49->7,49->8,50->12,50->6] | 0.83 | 500.265625 | 21 | 64 | 0 | 1 |
| Path 202 | C00024->C00668:[49->13,49->7,49->8,50->12,50->6] | 0.83 | 514.984375 | 21 | 64 | 0 | 1 |
| Path 203 | C00024->C00668:[2->10,49->13,50->12] | 0.50 | 284.086538462 | 19 | 104 | 0 | 0 |
| Path 204 | C00024->C00668:[49->13,49->7,49->8,50->12,50->6] | 0.83 | 501.287878788 | 23 | 66 | 0 | 1 |
| Path 205 | C00024->C00668:[49->13,49->7,49->8,50->10,50->12,50->6] | 1.00 | 533.366666667 | 24 | 60 | 0 | 1 |
| Path 206 | C00024->C00668:[49->13,49->7,50->12,50->6] | 0.67 | 460.333333333 | 19 | 60 | 0 | 1 |
| Path 207 | C00024->C00668:[14->8,49->7,50->6] | 0.50 | 336.270440252 | 20 | 159 | 0 | 1 |
| Path 208 | C00024->C00668:[49->13,50->10,50->12] | 0.50 | 599.545454545 | 15 | 22 | 0 | 0 |
| Path 209 | C00024->C00668:[49->13,49->7,50->10,50->12,50->6] | 0.83 | 526.0 | 22 | 57 | 0 | 1 |
| Path 210 | C00024->C00668:[49->13,49->7,49->8,50->12,50->6] | 0.83 | 383.830985915 | 18 | 71 | 0 | 1 |
| Path 211 | C00024->C00668:[49->7,50->6,50->8] | 0.50 | 451.192307692 | 20 | 52 | 0 | 1 |
| Path 212 | C00024->C00668:[49->13,50->10,50->12] | 0.50 | 613.263157895 | 14 | 19 | 0 | 0 |
| Path 213 | C00024->C00668:[49->13,49->7,50->12,50->6] | 0.67 | 383.217391304 | 16 | 69 | 0 | 1 |
| Path 214 | C00024->C00668:[49->7,49->8,50->6] | 0.50 | 364.591836735 | 19 | 98 | 0 | 1 |
| Path 215 | C00024->C00668:[49->13,50->12] | 0.33 | 554.461538462 | 8 | 13 | 0 | 0 |
| Path 216 | C00024->C00668:[49->13,49->7,49->8,50->12,50->6] | 0.83 | 382.413333333 | 24 | 75 | 0 | 1 |
| Path 217 | C00024->C00668:[49->13,49->7,49->8,50->12,50->6] | 0.83 | 449.38372093 | 30 | 86 | 0 | 1 |
| Path 218 | C00024->C00668:[49->7,49->8,50->6] | 0.50 | 415.981481481 | 14 | 54 | 0 | 1 |
| Path 219 | C00024->C00668:[14->8,49->13,49->7,50->12,50->6] | 0.83 | 354.390625 | 25 | 192 | 0 | 0 |
| Path 220 | C00024->C00668:[10->7,11->8,15->6,2->10,49->13,50->12] | 1.00 | 329.184210526 | 19 | 152 | 0 | 0 |
| Path 221 | C00024->C00668:[49->13,50->12] | 0.33 | 462.8 | 6 | 10 | 0 | 0 |
| Path 222 | C00024->C00668:[49->12,49->7,49->8,50->10,50->13,50->6] | 1.00 | 525.909090909 | 21 | 55 | 0 | 1 |
| Path 223 | C00024->C00668:[49->7,49->8,50->6] | 0.50 | 338.305084746 | 17 | 59 | 0 | 1 |
| Path 224 | C00024->C00668:[2->10,49->13,49->7,50->12,50->6] | 0.83 | 370.166666667 | 24 | 120 | 0 | 1 |
| Path 225 | C00024->C00668:[49->13,49->7,49->8,50->12,50->6] | 0.83 | 353.891566265 | 20 | 83 | 0 | 1 |
| Path 226 | C00024->C00668:[49->13,50->10,50->12] | 0.50 | 344.368421053 | 14 | 38 | 0 | 0 |
| Path 227 | C00024->C00668:[49->13,49->7,49->8,50->12,50->6] | 0.83 | 308.683544304 | 21 | 79 | 0 | 1 |
| Path 228 | C00024->C00668:[49->7,49->8,50->6] | 0.50 | 559.905660377 | 18 | 53 | 0 | 0 |
| Path 229 | C00024->C00668:[49->13,49->7,49->8,50->12,50->6] | 0.83 | 517.49122807 | 20 | 57 | 0 | 1 |
| Path 230 | C00024->C00668:[49->13,49->7,50->12,50->6] | 0.67 | 525.545454545 | 18 | 44 | 0 | 0 |
| Path 231 | C00024->C00668:[49->13,49->7,49->8,50->12,50->6] | 0.83 | 452.550724638 | 20 | 69 | 0 | 0 |
| Path 232 | C00024->C00668:[2->10,49->13,50->12] | 0.50 | 330.868421053 | 20 | 152 | 0 | 0 |
| Path 233 | C00024->C00668:[14->8,49->13,49->7,50->12,50->6] | 0.83 | 327.402234637 | 24 | 179 | 0 | 0 |
| Path 234 | C00024->C00668:[49->7,49->8,50->6] | 0.50 | 443.22 | 14 | 50 | 0 | 1 |
| Path 235 | C00024->C00668:[49->13,49->7,49->8,50->10,50->12,50->6] | 1.00 | 555.965517241 | 24 | 58 | 0 | 1 |
| Path 236 | C00024->C00668:[49->13,49->7,50->12,50->6] | 0.67 | 418.067567568 | 18 | 74 | 0 | 0 |
| Path 237 | C00024->C00668:[49->7,49->8,50->6] | 0.50 | 459.020833333 | 14 | 48 | 0 | 1 |
| Path 238 | C00024->C00668:[49->13,49->7,50->12,50->6] | 0.67 | 500.609375 | 20 | 64 | 0 | 1 |
| Path 239 | C00024->C00668:[49->13,49->7,49->8,50->12,50->6] | 0.83 | 488.676056338 | 23 | 71 | 0 | 1 |
| Path 240 | C00024->C00668:[14->8,49->7,50->6] | 0.50 | 331.588235294 | 18 | 153 | 0 | 1 |
| Path 241 | C00024->C00668:[2->10,49->13,49->7,50->12,50->6] | 0.83 | 336.918918919 | 23 | 111 | 0 | 0 |
| Path 242 | C00024->C00668:[49->13,49->7,50->12,50->6] | 0.67 | 525.095238095 | 21 | 63 | 0 | 1 |
| Path 243 | C00024->C00668:[49->7,49->8,50->6] | 0.50 | 452.6 | 20 | 50 | 0 | 1 |
| Path 244 | C00024->C00668:[49->13,49->7,49->8,50->12,50->6] | 0.83 | 403.767857143 | 21 | 56 | 0 | 1 |
| Path 245 | C00024->C00668:[49->13,49->7,50->12,50->6] | 0.67 | 509.327586207 | 19 | 58 | 0 | 1 |
| Path 246 | C00024->C00668:[49->13,49->7,49->8,50->12,50->6] | 0.83 | 332.243243243 | 20 | 74 | 0 | 0 |
| Path 247 | C00024->C00668:[49->13,49->7,50->12,50->6] | 0.67 | 449.8 | 19 | 50 | 0 | 1 |
| Path 248 | C00024->C00668:[49->13,50->10,50->12] | 0.50 | 551.192307692 | 16 | 26 | 0 | 0 |
| Path 249 | C00024->C00668:[49->7,49->8,50->6] | 0.50 | 447.509803922 | 18 | 51 | 0 | 1 |
| Path 250 | C00024->C00668:[49->13,49->7,49->8,50->12,50->6] | 0.83 | 319.058823529 | 23 | 85 | 0 | 1 |
| Path 251 | C00024->C00668:[2->10,49->13,50->12] | 0.50 | 338.96124031 | 14 | 129 | 0 | 0 |
| Path 252 | C00024->C00668:[49->7,49->8,50->6] | 0.50 | 511.425925926 | 19 | 54 | 0 | 1 |
| Path 253 | C00024->C00668:[49->7,49->8,50->6] | 0.50 | 327.053191489 | 20 | 94 | 0 | 1 |
| Path 254 | C00024->C00668:[49->13,49->7,49->8,50->12,50->6] | 0.83 | 307.367521368 | 24 | 117 | 0 | 1 |
| Path 255 | C00024->C00668:[49->13,49->7,49->8,50->12,50->6] | 0.83 | 334.888888889 | 20 | 72 | 0 | 1 |
| Path 256 | C00024->C00668:[49->7,49->8,50->6] | 0.50 | 504.339622642 | 18 | 53 | 0 | 1 |
| Path 257 | C00024->C00668:[49->7,49->8,50->6] | 0.50 | 384.276595745 | 16 | 47 | 0 | 1 |
| Path 258 | C00024->C00668:[49->13,49->7,49->8,50->12,50->6] | 0.83 | 374.753846154 | 20 | 65 | 0 | 0 |
| Path 259 | C00024->C00668:[49->7,49->8,50->6] | 0.50 | 364.779141104 | 20 | 163 | 0 | 1 |
| Path 260 | C00024->C00668:[49->7,49->8,50->6] | 0.50 | 338.538461538 | 15 | 52 | 0 | 1 |
| Path 261 | C00024->C00668:[49->13,49->7,50->12,50->6] | 0.67 | 515.087719298 | 18 | 57 | 0 | 1 |
| Path 262 | C00024->C00668:[49->7,49->8,50->6] | 0.50 | 393.738095238 | 14 | 42 | 0 | 1 |
| Path 263 | C00024->C00668:[49->13,49->7,49->8,50->12,50->6] | 0.83 | 432.428571429 | 21 | 56 | 0 | 1 |
| Path 264 | C00024->C00668:[49->13,49->7,49->8,50->12,50->6] | 0.83 | 354.772727273 | 21 | 88 | 0 | 1 |
| Path 265 | C00024->C00668:[49->13,49->7,49->8,50->12,50->6] | 0.83 | 378.024691358 | 25 | 81 | 0 | 1 |
| Path 266 | C00024->C00668:[49->13,49->7,49->8,50->12,50->6] | 0.83 | 353.072463768 | 20 | 69 | 0 | 1 |
| Path 267 | C00024->C00668:[49->7,49->8,50->6] | 0.50 | 454.0 | 21 | 54 | 0 | 1 |
| Path 268 | C00024->C00668:[49->7,49->8,50->6] | 0.50 | 321.450980392 | 15 | 51 | 0 | 1 |
| Path 269 | C00024->C00668:[49->13,49->7,49->8,50->12,50->6] | 0.83 | 327.972527473 | 25 | 182 | 0 | 1 |
| Path 270 | C00024->C00668:[49->12,49->7,50->13,50->6] | 0.67 | 514.482758621 | 19 | 58 | 0 | 1 |
| Path 271 | C00024->C00668:[49->13,49->7,49->8,50->12,50->6] | 0.83 | 398.951807229 | 21 | 83 | 0 | 0 |
| Path 272 | C00024->C00668:[49->7,50->6] | 0.33 | 416.028571429 | 12 | 35 | 0 | 0 |
| Path 273 | C00024->C00668:[49->13,49->7,49->8,50->12,50->6] | 0.83 | 443.717647059 | 29 | 85 | 0 | 1 |
| Path 274 | C00024->C00668:[49->13,49->7,49->8,50->12,50->6] | 0.83 | 312.297619048 | 22 | 84 | 0 | 1 |
| Path 275 | C00024->C00668:[49->7,49->8,50->6] | 0.50 | 427.510204082 | 18 | 49 | 0 | 1 |
| Path 276 | C00024->C00668:[49->13,50->12] | 0.33 | 672.857142857 | 7 | 7 | 0 | 0 |
| Path 277 | C00024->C00668:[49->13,49->7,50->12,50->6] | 0.67 | 421.358490566 | 20 | 53 | 0 | 1 |
| Path 278 | C00024->C00668:[49->13,49->7,50->10,50->12,50->6] | 0.83 | 527.0 | 23 | 56 | 0 | 1 |
| Path 279 | C00024->C00668:[49->7,49->8,50->6] | 0.50 | 391.64516129 | 16 | 62 | 0 | 1 |
| Path 280 | C00024->C00668:[49->13,50->10,50->12] | 0.50 | 605.714285714 | 16 | 21 | 0 | 0 |
| Path 281 | C00024->C00668:[49->13,49->7,49->8,50->12,50->6] | 0.83 | 391.08045977 | 21 | 87 | 0 | 0 |
| Path 282 | C00024->C00668:[14->8,49->7,50->6] | 0.50 | 360.853503185 | 18 | 157 | 0 | 0 |
| Path 283 | C00024->C00668:[49->7,50->6,50->8] | 0.50 | 438.510638298 | 18 | 47 | 0 | 1 |
| Path 284 | C00024->C00668:[49->13,50->12] | 0.33 | 699.625 | 8 | 8 | 0 | 0 |
| Path 285 | C00024->C00668:[49->13,49->7,50->12,50->6] | 0.67 | 537.74137931 | 19 | 58 | 0 | 1 |
| Path 286 | C00024->C00668:[49->7,49->8,50->6] | 0.50 | 325.732142857 | 16 | 56 | 0 | 1 |
| Path 287 | C00024->C00668:[14->8,49->7,50->6] | 0.50 | 337.692810458 | 19 | 153 | 0 | 0 |
| Path 288 | C00024->C00668:[49->13,49->7,49->8,50->12,50->6] | 0.83 | 304.598214286 | 23 | 112 | 0 | 1 |
| Path 289 | C00024->C00668:[49->13,49->7,49->8,50->10,50->12,50->6] | 1.00 | 517.859375 | 25 | 64 | 0 | 1 |
| Path 290 | C00024->C00668:[49->13,49->7,49->8,50->12,50->6] | 0.83 | 509.044776119 | 22 | 67 | 0 | 1 |
| Path 291 | C00024->C00668:[49->13,49->7,50->12,50->6] | 0.67 | 404.160714286 | 20 | 56 | 0 | 1 |
| Path 292 | C00024->C00668:[49->13,49->7,49->8,50->12,50->6] | 0.83 | 517.627118644 | 19 | 59 | 0 | 1 |
| Path 293 | C00024->C00668:[49->13,50->12] | 0.33 | 687.5 | 9 | 12 | 0 | 0 |
| Path 294 | C00024->C00668:[49->13,49->7,49->8,50->12,50->6] | 0.83 | 379.808219178 | 18 | 73 | 0 | 0 |
| Path 295 | C00024->C00668:[14->8,49->7,50->6] | 0.50 | 360.572368421 | 17 | 152 | 0 | 0 |
| Path 296 | C00024->C00668:[2->10,49->7,49->8,50->6] | 0.67 | 386.188235294 | 25 | 170 | 0 | 0 |
| Path 297 | C00024->C00668:[49->13,50->12] | 0.33 | 674.111111111 | 7 | 9 | 0 | 0 |
| Path 298 | C00024->C00668:[49->13,49->7,50->12,50->6] | 0.67 | 407.476923077 | 16 | 65 | 0 | 0 |
| Path 299 | C00024->C00668:[10->7,11->8,15->6,49->13,50->12] | 0.83 | 364.126865672 | 18 | 134 | 0 | 0 |
| Path 300 | C00024->C00668:[49->13,49->7,50->12,50->6] | 0.67 | 410.65 | 15 | 60 | 0 | 0 |
| Path 301 | C00024->C00668:[49->7,49->8,50->6] | 0.50 | 393.596491228 | 15 | 57 | 0 | 1 |
| Path 302 | C00024->C00668:[49->13,49->7,49->8,50->12,50->6] | 0.83 | 531.730769231 | 19 | 52 | 0 | 1 |
| Path 303 | C00024->C00668:[14->8,49->13,49->7,50->12,50->6] | 0.83 | 357.085106383 | 25 | 188 | 0 | 0 |
| Path 304 | C00024->C00668:[49->13,50->10,50->12] | 0.50 | 662.842105263 | 14 | 19 | 0 | 0 |
| Path 305 | C00024->C00668:[49->7,49->8,50->6] | 0.50 | 534.212765957 | 15 | 47 | 0 | 0 |
| Path 306 | C00024->C00668:[49->7,49->8,50->6] | 0.50 | 335.255319149 | 14 | 47 | 0 | 1 |
| Path 307 | C00024->C00668:[49->13,49->7,49->8,50->12,50->6] | 0.83 | 531.833333333 | 23 | 66 | 0 | 1 |
| Path 308 | C00024->C00668:[49->7,49->8,50->6] | 0.50 | 564.837209302 | 15 | 43 | 0 | 0 |
| Path 309 | C00024->C00668:[49->13,50->10,50->12] | 0.50 | 553.0 | 16 | 26 | 0 | 0 |
| Path 310 | C00024->C00668:[49->13,49->7,50->12,50->6] | 0.67 | 657.64 | 14 | 25 | 0 | 0 |
| Path 311 | C00024->C00668:[49->7,50->6,50->8] | 0.50 | 508.644444444 | 16 | 45 | 0 | 0 |
| Path 312 | C00024->C00668:[49->7,49->8,50->6] | 0.50 | 344.491803279 | 18 | 61 | 0 | 1 |
| Path 313 | C00024->C00668:[49->13,49->7,49->8,50->12,50->6] | 0.83 | 348.424 | 24 | 125 | 0 | 1 |
| Path 314 | C00024->C00668:[49->13,49->7,49->8,50->12,50->6] | 0.83 | 337.086206897 | 22 | 116 | 0 | 1 |
| Path 315 | C00024->C00668:[10->7,11->8,15->6,2->10,49->13,50->12] | 1.00 | 308.186915888 | 21 | 214 | 0 | 0 |
| Path 316 | C00024->C00668:[49->13,49->7,49->8,50->12,50->6] | 0.83 | 397.511627907 | 27 | 86 | 0 | 1 |
| Path 317 | C00024->C00668:[49->13,49->7,49->8,50->12,50->6] | 0.83 | 489.338028169 | 23 | 71 | 0 | 1 |
| Path 318 | C00024->C00668:[2->10,49->13,50->12] | 0.50 | 289.346938776 | 17 | 98 | 0 | 0 |
| Path 319 | C00024->C00668:[49->7,49->8,50->6] | 0.50 | 388.745454545 | 14 | 55 | 0 | 1 |
| Path 320 | C00024->C00668:[10->7,11->8,15->6,2->10,49->13,50->12] | 1.00 | 333.117647059 | 20 | 153 | 0 | 0 |
| Path 321 | C00024->C00668:[49->13,49->7,49->8,50->12,50->6] | 0.83 | 343.301587302 | 18 | 63 | 0 | 1 |
| Path 322 | C00024->C00668:[49->13,49->7,49->8,50->12,50->6] | 0.83 | 408.763157895 | 19 | 76 | 0 | 1 |
| Path 323 | C00024->C00668:[49->13,49->7,49->8,50->12,50->6] | 0.83 | 444.405405405 | 21 | 74 | 0 | 0 |
| Path 324 | C00024->C00668:[49->13,49->7,49->8,50->12,50->6] | 0.83 | 382.881578947 | 19 | 76 | 0 | 1 |
| Path 325 | C00024->C00668:[49->7,49->8,50->6] | 0.50 | 362.773809524 | 15 | 84 | 0 | 1 |
| Path 326 | C00024->C00668:[49->13,49->7,49->8,50->12,50->6] | 0.83 | 362.675324675 | 21 | 77 | 0 | 1 |
| Path 327 | C00024->C00668:[49->7,50->6] | 0.33 | 596.8 | 11 | 20 | 0 | 0 |
| Path 328 | C00024->C00668:[49->13,49->7,49->8,50->12,50->6] | 0.83 | 415.367088608 | 20 | 79 | 0 | 1 |
| Path 329 | C00024->C00668:[49->7,49->8,50->6] | 0.50 | 318.575 | 16 | 80 | 0 | 1 |
| Path 330 | C00024->C00668:[49->13,49->7,49->8,50->12,50->6] | 0.83 | 503.318181818 | 21 | 66 | 0 | 1 |
| Path 331 | C00024->C00668:[49->13,49->7,49->8,50->12,50->6] | 0.83 | 332.04787234 | 27 | 188 | 0 | 1 |
| Path 332 | C00024->C00668:[49->7,49->8,50->6] | 0.50 | 430.581818182 | 20 | 55 | 0 | 1 |
| Path 333 | C00024->C00668:[49->13,49->7,49->8,50->10,50->12,50->6] | 1.00 | 529.0 | 22 | 56 | 0 | 1 |
| Path 334 | C00024->C00668:[49->13,49->7,49->8,50->12,50->6] | 0.83 | 312.279661017 | 25 | 118 | 0 | 1 |
| Path 335 | C00024->C00668:[49->13,49->7,49->8,50->12,50->6] | 0.83 | 345.220588235 | 19 | 68 | 0 | 1 |
| Path 336 | C00024->C00668:[49->7,49->8,50->6] | 0.50 | 521.607843137 | 18 | 51 | 0 | 1 |
| Path 337 | C00024->C00668:[49->13,49->7,49->8,50->12,50->6] | 0.83 | 488.983333333 | 21 | 60 | 0 | 1 |
| Path 338 | C00024->C00668:[49->13,49->7,49->8,50->12,50->6] | 0.83 | 437.012048193 | 24 | 83 | 0 | 1 |
| Path 339 | C00024->C00668:[49->13,49->7,49->8,50->12,50->6] | 0.83 | 329.935483871 | 24 | 93 | 0 | 1 |
| Path 340 | C00024->C00668:[49->7,49->8,50->6] | 0.50 | 509.018867925 | 17 | 53 | 0 | 1 |
| Path 341 | C00024->C00668:[49->7,50->6,50->8] | 0.50 | 514.109090909 | 19 | 55 | 0 | 0 |
| Path 342 | C00024->C00668:[49->13,49->7,49->8,50->12,50->6] | 0.83 | 360.752808989 | 22 | 89 | 0 | 1 |
| Path 343 | C00024->C00668:[49->7,49->8,50->6] | 0.50 | 451.422222222 | 13 | 45 | 0 | 1 |
| Path 344 | C00024->C00668:[49->12,49->7,49->8,50->13,50->6] | 0.83 | 499.953846154 | 22 | 65 | 0 | 1 |
| Path 345 | C00024->C00668:[49->7,49->8,50->6] | 0.50 | 387.306818182 | 17 | 88 | 0 | 0 |
| Path 346 | C00024->C00668:[49->13,49->7,49->8,50->12,50->6] | 0.83 | 505.808823529 | 23 | 68 | 0 | 1 |
| Path 347 | C00024->C00668:[49->7,50->6] | 0.33 | 464.657894737 | 10 | 38 | 0 | 1 |
| Path 348 | C00024->C00668:[49->13,49->7,50->12,50->6] | 0.67 | 542.653061224 | 17 | 49 | 0 | 0 |
| Path 349 | C00024->C00668:[49->7,49->8,50->6] | 0.50 | 430.070175439 | 20 | 57 | 0 | 1 |
| Path 350 | C00024->C00668:[49->13,49->7,49->8,50->12,50->6] | 0.83 | 338.421487603 | 23 | 121 | 0 | 1 |
| Path 351 | C00024->C00668:[49->13,50->12] | 0.33 | 555.071428571 | 8 | 14 | 0 | 0 |
| Path 352 | C00024->C00668:[2->10,49->13,50->12] | 0.50 | 347.073170732 | 18 | 82 | 0 | 0 |
| Path 353 | C00024->C00668:[49->7,50->6,50->8] | 0.50 | 501.901960784 | 17 | 51 | 0 | 1 |
| Path 354 | C00024->C00668:[49->13,49->7,50->10,50->12,50->6] | 0.83 | 550.418181818 | 22 | 55 | 0 | 1 |
| Path 355 | C00024->C00668:[49->13,49->7,50->12,50->6] | 0.67 | 521.06557377 | 20 | 61 | 0 | 1 |
| Path 356 | C00024->C00668:[2->10,49->13,49->7,49->8,50->12,50->6] | 1.00 | 369.052325581 | 24 | 172 | 0 | 1 |
| Path 357 | C00024->C00668:[49->13,49->7,50->10,50->12,50->6] | 0.83 | 536.083333333 | 24 | 60 | 0 | 1 |
| Path 358 | C00024->C00668:[49->7,50->6,50->8] | 0.50 | 436.0 | 16 | 41 | 0 | 1 |
| Path 359 | C00024->C00668:[49->13,49->7,49->8,50->12,50->6] | 0.83 | 419.205882353 | 18 | 68 | 0 | 0 |
| Path 360 | C00024->C00668:[49->13,49->7,50->10,50->12,50->6] | 0.83 | 510.983606557 | 23 | 61 | 0 | 1 |
| Path 361 | C00024->C00668:[49->7,49->8,50->6] | 0.50 | 335.578947368 | 17 | 57 | 0 | 1 |
| Path 362 | C00024->C00668:[49->13,49->7,49->8,50->12,50->6] | 0.83 | 389.428571429 | 20 | 77 | 0 | 1 |
| Path 363 | C00024->C00668:[49->6] | 0.17 | 569.785714286 | 9 | 14 | 0 | 1 |
| Path 364 | C00024->C00668:[49->13,49->7,49->8,50->12,50->6] | 0.83 | 403.024096386 | 21 | 83 | 0 | 1 |
| Path 365 | C00024->C00668:[49->13,49->7,49->8,50->12,50->6] | 0.83 | 342.452054795 | 21 | 73 | 0 | 1 |
| Path 366 | C00024->C00668:[49->13,49->7,49->8,50->12,50->6] | 0.83 | 497.721311475 | 22 | 61 | 0 | 1 |
| Path 367 | C00024->C00668:[49->7,50->6] | 0.33 | 386.571428571 | 13 | 42 | 0 | 1 |
| Path 368 | C00024->C00668:[49->7,49->8,50->6] | 0.50 | 369.989130435 | 17 | 92 | 0 | 1 |
| Path 369 | C00024->C00668:[49->13,49->7,50->12,50->6] | 0.67 | 419.180327869 | 16 | 61 | 0 | 0 |
| Path 370 | C00024->C00668:[49->13,49->7,49->8,50->12,50->6] | 0.83 | 536.691176471 | 29 | 68 | 0 | 1 |
| Path 371 | C00024->C00668:[49->13,50->12] | 0.33 | 422.133333333 | 7 | 15 | 0 | 0 |
| Path 372 | C00024->C00668:[49->13,49->7,49->8,50->10,50->12,50->6] | 1.00 | 518.59375 | 25 | 64 | 0 | 1 |
| Path 373 | C00024->C00668:[49->13,49->7,49->8,50->12,50->6] | 0.83 | 523.0 | 20 | 60 | 0 | 1 |
| Path 374 | C00024->C00668:[49->13,49->7,49->8,50->12,50->6] | 0.83 | 510.107142857 | 19 | 56 | 0 | 1 |
| Path 375 | C00024->C00668:[49->7,49->8,50->6] | 0.50 | 400.015384615 | 17 | 65 | 0 | 1 |
| Path 376 | C00024->C00668:[49->13,50->12] | 0.33 | 908.285714286 | 7 | 7 | 0 | 0 |
| Path 377 | C00024->C00668:[49->13,49->7,49->8,50->12,50->6] | 0.83 | 530.805970149 | 28 | 67 | 0 | 1 |
| Path 378 | C00024->C00668:[49->13,49->7,50->10,50->12,50->6] | 0.83 | 544.185185185 | 21 | 54 | 0 | 1 |
| Path 379 | C00024->C00668:[49->13,49->7,49->8,50->12,50->6] | 0.83 | 367.652173913 | 22 | 92 | 0 | 1 |
| Path 380 | C00024->C00668:[49->7,49->8,50->6] | 0.50 | 385.571428571 | 16 | 49 | 0 | 1 |
| Path 381 | C00024->C00668:[49->7,50->6] | 0.33 | 453.581395349 | 11 | 43 | 0 | 1 |
| Path 382 | C00024->C00668:[2->10,49->13,49->7,50->12,50->6] | 0.83 | 342.223214286 | 24 | 112 | 0 | 0 |
| Path 383 | C00024->C00668:[49->13,50->12] | 0.33 | 911.833333333 | 6 | 6 | 0 | 0 |
| Path 384 | C00024->C00668:[49->7,50->6] | 0.33 | 372.647058824 | 11 | 34 | 0 | 1 |
| Path 385 | C00024->C00668:[49->13,49->7,49->8,50->12,50->6] | 0.83 | 520.707692308 | 22 | 65 | 0 | 1 |
| Path 386 | C00024->C00668:[49->7,49->8,50->6] | 0.50 | 451.192307692 | 20 | 52 | 0 | 1 |
| Path 387 | C00024->C00668:[49->7,49->8,50->6] | 0.50 | 422.12962963 | 19 | 54 | 0 | 1 |
| Path 388 | C00024->C00668:[14->8,49->7,50->6] | 0.50 | 367.318181818 | 20 | 110 | 0 | 0 |
| Path 389 | C00024->C00668:[49->13,49->7,49->8,50->12,50->6] | 0.83 | 426.623188406 | 19 | 69 | 0 | 0 |
| Path 390 | C00024->C00668:[49->13,49->7,49->8,50->12,50->6] | 0.83 | 441.175438596 | 22 | 57 | 0 | 1 |
| Path 391 | C00024->C00668:[49->7,49->8,50->6] | 0.50 | 342.512345679 | 22 | 162 | 0 | 1 |
| Path 392 | C00024->C00668:[14->6] | 0.17 | 333.821705426 | 14 | 129 | 0 | 1 |
| Path 393 | C00024->C00668:[49->7,49->8,50->6] | 0.50 | 436.342105263 | 14 | 38 | 0 | 0 |
| Path 394 | C00024->C00668:[2->10,49->7,50->6] | 0.50 | 374.619565217 | 21 | 92 | 0 | 0 |
| Path 395 | C00024->C00668:[49->7,49->8,50->6] | 0.50 | 450.566037736 | 15 | 53 | 0 | 1 |
| Path 396 | C00024->C00668:[49->13,49->7,49->8,50->12,50->6] | 0.83 | 397.23880597 | 17 | 67 | 0 | 1 |
| Path 397 | C00024->C00668:[14->8,49->7,50->6] | 0.50 | 354.904458599 | 17 | 157 | 0 | 1 |
| Path 398 | C00024->C00668:[49->13,49->7,50->10,50->12,50->6] | 0.83 | 527.611111111 | 21 | 54 | 0 | 1 |
| Path 399 | C00024->C00668:[49->13,49->7,50->12,50->6,50->8] | 0.83 | 434.740740741 | 24 | 81 | 0 | 0 |
| Path 400 | C00024->C00668:[49->13,49->7,49->8,50->12,50->6] | 0.83 | 435.255555556 | 26 | 90 | 0 | 1 |
| Path 401 | C00024->C00668:[49->7,50->6] | 0.33 | 372.230769231 | 12 | 39 | 0 | 1 |
| Path 402 | C00024->C00668:[49->7,50->6] | 0.33 | 501.230769231 | 11 | 39 | 0 | 0 |
| Path 403 | C00024->C00668:[49->13,50->12] | 0.33 | 695.4 | 8 | 10 | 0 | 0 |
| Path 404 | C00024->C00668:[49->7,49->8,50->6] | 0.50 | 533.0 | 12 | 37 | 0 | 0 |
| Path 405 | C00024->C00668:[49->13,49->7,50->12,50->6] | 0.67 | 520.295081967 | 20 | 61 | 0 | 1 |
| Path 406 | C00024->C00668:[49->13,49->7,49->8,50->12,50->6] | 0.83 | 422.576923077 | 20 | 52 | 0 | 1 |
| Path 407 | C00024->C00668:[49->13,49->7,49->8,50->10,50->12,50->6] | 1.00 | 556.775862069 | 24 | 58 | 0 | 1 |
| Path 408 | C00024->C00668:[49->13,50->10,50->12] | 0.50 | 600.666666667 | 13 | 18 | 0 | 0 |
| Path 409 | C00024->C00668:[49->13,49->7,49->8,50->12,50->6] | 0.83 | 396.2875 | 20 | 80 | 0 | 1 |
| Path 410 | C00024->C00668:[49->13,49->7,49->8,50->12,50->6] | 0.83 | 371.6625 | 24 | 80 | 0 | 1 |
| Path 411 | C00024->C00668:[49->13,49->7,49->8,50->12,50->6] | 0.83 | 468.375 | 22 | 64 | 0 | 1 |
| Path 412 | C00024->C00668:[2->10,49->13,49->7,50->12,50->6] | 0.83 | 363.650887574 | 22 | 169 | 0 | 1 |
| Path 413 | C00024->C00668:[49->13,49->7,49->8,50->10,50->12,50->6] | 1.00 | 528.362068966 | 24 | 58 | 0 | 1 |
| Path 414 | C00024->C00668:[2->10,49->13,49->7,49->8,50->12,50->6] | 1.00 | 358.677083333 | 28 | 192 | 0 | 0 |
| Path 415 | C00024->C00668:[49->7,49->8,50->6] | 0.50 | 330.136363636 | 18 | 88 | 0 | 1 |
| Path 416 | C00024->C00668:[49->12,50->13] | 0.33 | 495.666666667 | 8 | 12 | 0 | 0 |
| Path 417 | C00024->C00668:[49->13,49->7,49->8,50->12,50->6] | 0.83 | 481.446428571 | 19 | 56 | 0 | 1 |
| Path 418 | C00024->C00668:[49->13,50->10,50->12] | 0.50 | 614.92 | 17 | 25 | 0 | 0 |
| Path 419 | C00024->C00668:[49->7,50->6] | 0.33 | 524.727272727 | 13 | 44 | 0 | 0 |
| Path 420 | C00024->C00668:[49->7,49->8,50->6] | 0.50 | 405.209302326 | 15 | 43 | 0 | 1 |
| Path 421 | C00024->C00668:[49->7,49->8,50->6] | 0.50 | 339.130434783 | 21 | 161 | 0 | 1 |
| Path 422 | C00024->C00668:[49->13,49->7,50->12,50->6] | 0.67 | 509.285714286 | 17 | 56 | 0 | 1 |
| Path 423 | C00024->C00668:[49->7,49->8,50->6] | 0.50 | 395.984126984 | 16 | 63 | 0 | 1 |
| Path 424 | C00024->C00668:[2->10,49->13,50->12] | 0.50 | 307.03 | 19 | 100 | 0 | 0 |
| Path 425 | C00024->C00668:[2->10,49->13,50->12] | 0.50 | 326.050632911 | 15 | 79 | 0 | 0 |
| Path 426 | C00024->C00668:[49->7,49->8,50->6] | 0.50 | 363.146067416 | 16 | 89 | 0 | 1 |
| Path 427 | C00024->C00668:[49->13,49->7,49->8,50->12,50->6] | 0.83 | 523.901960784 | 18 | 51 | 0 | 1 |
| Path 428 | C00024->C00668:[49->13,49->7,50->12,50->6] | 0.67 | 432.395348837 | 15 | 43 | 0 | 0 |
| Path 429 | C00024->C00668:[49->7,50->6,50->8] | 0.50 | 512.125 | 16 | 48 | 0 | 1 |
| Path 430 | C00024->C00668:[49->7,49->8,50->6] | 0.50 | 447.837209302 | 16 | 43 | 0 | 1 |
| Path 431 | C00024->C00668:[49->7,49->8,50->6] | 0.50 | 395.327868852 | 16 | 61 | 0 | 1 |
| Path 432 | C00024->C00668:[49->13,49->7,49->8,50->12,50->6] | 0.83 | 384.08988764 | 26 | 89 | 0 | 1 |
| Path 433 | C00024->C00668:[49->13,49->7,49->8,50->12,50->6] | 0.83 | 528.203125 | 22 | 64 | 0 | 1 |
| Path 434 | C00024->C00668:[49->7,49->8,50->6] | 0.50 | 459.210526316 | 16 | 57 | 0 | 0 |
| Path 435 | C00024->C00668:[49->13,49->7,49->8,50->12,50->6] | 0.83 | 424.136363636 | 24 | 88 | 0 | 1 |
| Path 436 | C00024->C00668:[14->6] | 0.17 | 333.26119403 | 15 | 134 | 0 | 1 |
| Path 437 | C00024->C00668:[14->8,49->13,49->7,50->12,50->6] | 0.83 | 346.882022472 | 22 | 178 | 0 | 0 |
| Path 438 | C00024->C00668:[49->7,49->8,50->6] | 0.50 | 442.8 | 15 | 55 | 0 | 0 |
| Path 439 | C00024->C00668:[49->13,50->12] | 0.33 | 474.823529412 | 8 | 17 | 0 | 0 |
| Path 440 | C00024->C00668:[2->10,49->13,50->12] | 0.50 | 342.455223881 | 16 | 134 | 0 | 0 |
| Path 441 | C00024->C00668:[49->13,49->7,49->8,50->12,50->6] | 0.83 | 416.113924051 | 22 | 79 | 0 | 1 |
| Path 442 | C00024->C00668:[49->13,50->10,50->12] | 0.50 | 591.65 | 15 | 20 | 0 | 0 |
| Path 443 | C00024->C00668:[49->7,49->8,50->6] | 0.50 | 367.666666667 | 15 | 48 | 0 | 0 |
| Path 444 | C00024->C00668:[49->13,49->7,49->8,50->12,50->6] | 0.83 | 497.381818182 | 19 | 55 | 0 | 1 |
| Path 445 | C00024->C00668:[49->13,50->10,50->12] | 0.50 | 671.7 | 15 | 20 | 0 | 0 |
| Path 446 | C00024->C00668:[49->13,49->7,49->8,50->12,50->6] | 0.83 | 418.905882353 | 24 | 85 | 0 | 1 |
| Path 447 | C00024->C00668:[2->10,49->13,49->7,49->8,50->12,50->6] | 1.00 | 372.300578035 | 25 | 173 | 0 | 1 |
| Path 448 | C00024->C00668:[49->13,49->7,50->12,50->6] | 0.67 | 415.409090909 | 17 | 66 | 0 | 0 |
| Path 449 | C00024->C00668:[49->13,49->7,50->12,50->6] | 0.67 | 480.066666667 | 18 | 45 | 0 | 0 |
| Path 450 | C00024->C00668:[2->10,49->13,50->12] | 0.50 | 332.392857143 | 17 | 84 | 0 | 0 |
| Path 451 | C00024->C00668:[2->10,49->13,50->12] | 0.50 | 290.275510204 | 17 | 98 | 0 | 0 |
| Path 452 | C00024->C00668:[49->13,49->7,49->8,50->12,50->6] | 0.83 | 431.09375 | 18 | 64 | 0 | 0 |
| Path 453 | C00024->C00668:[49->13,49->7,49->8,50->12,50->6] | 0.83 | 470.476190476 | 21 | 63 | 0 | 1 |
| Path 454 | C00024->C00668:[49->13,49->7,50->10,50->12,50->6] | 0.83 | 510.213114754 | 23 | 61 | 0 | 1 |
| Path 455 | C00024->C00668:[49->13,49->7,49->8,50->12,50->6] | 0.83 | 522.596774194 | 22 | 62 | 0 | 1 |
| Path 456 | C00024->C00668:[49->13,49->7,50->12,50->6] | 0.67 | 396.064102564 | 18 | 78 | 0 | 1 |
| Path 457 | C00024->C00668:[49->13,49->7,49->8,50->12,50->6] | 0.83 | 505.125 | 20 | 56 | 0 | 1 |
| Path 458 | C00024->C00668:[49->7,49->8,50->6] | 0.50 | 321.564705882 | 17 | 85 | 0 | 1 |
| Path 459 | C00024->C00668:[49->7,49->8,50->6] | 0.50 | 442.29787234 | 13 | 47 | 0 | 0 |
| Path 460 | C00024->C00668:[10->7,11->8,15->6,6->10] | 0.67 | 332.487804878 | 10 | 123 | 0 | 0 |
| Path 461 | C00024->C00668:[49->13,49->7,49->8,50->12,50->6] | 0.83 | 402.04109589 | 19 | 73 | 0 | 1 |
| Path 462 | C00024->C00668:[49->13,49->7,49->8,50->12,50->6] | 0.83 | 354.507692308 | 26 | 195 | 0 | 1 |
| Path 463 | C00024->C00668:[49->13,49->7,49->8,50->12,50->6] | 0.83 | 401.428571429 | 23 | 63 | 0 | 1 |
| Path 464 | C00024->C00668:[49->13,49->7,49->8,50->12,50->6] | 0.83 | 428.012987013 | 20 | 77 | 0 | 0 |
| Path 465 | C00024->C00668:[49->6] | 0.17 | 561.941176471 | 14 | 34 | 0 | 1 |
| Path 466 | C00024->C00668:[49->7,49->8,50->6] | 0.50 | 373.347826087 | 15 | 46 | 0 | 1 |
| Path 467 | C00024->C00668:[49->7,49->8,50->6] | 0.50 | 501.75 | 16 | 52 | 0 | 1 |
| Path 468 | C00024->C00668:[49->13,49->7,49->8,50->12,50->6] | 0.83 | 523.35483871 | 22 | 62 | 0 | 1 |
| Path 469 | C00024->C00668:[49->13,49->7,50->10,50->12,50->6] | 0.83 | 531.413793103 | 23 | 58 | 0 | 1 |
| Path 470 | C00024->C00668:[49->13,49->7,49->8,50->12,50->6] | 0.83 | 550.192307692 | 19 | 52 | 0 | 0 |
| Path 471 | C00024->C00668:[49->7,49->8,50->6] | 0.50 | 329.20754717 | 16 | 53 | 0 | 1 |
| Path 472 | C00024->C00668:[49->13,49->7,49->8,50->12,50->6] | 0.83 | 394.428571429 | 26 | 84 | 0 | 1 |
| Path 473 | C00024->C00668:[49->7,49->8,50->6] | 0.50 | 508.017857143 | 19 | 56 | 0 | 1 |
| Path 474 | C00024->C00668:[49->13,49->7,49->8,50->12,50->6] | 0.83 | 523.783333333 | 20 | 60 | 0 | 1 |
| Path 475 | C00024->C00668:[49->13,49->7,49->8,50->10,50->12,50->6] | 1.00 | 550.98245614 | 23 | 57 | 0 | 1 |
| Path 476 | C00024->C00668:[49->13,49->7,49->8,50->12,50->6] | 0.83 | 569.425531915 | 18 | 47 | 0 | 0 |
| Path 477 | C00024->C00668:[49->13,49->7,49->8,50->12,50->6] | 0.83 | 352.62962963 | 22 | 81 | 0 | 1 |
| Path 478 | C00024->C00668:[49->13,49->7,50->12,50->6,50->8] | 0.83 | 420.098591549 | 21 | 71 | 0 | 0 |
| Path 479 | C00024->C00668:[49->7,49->8,50->6] | 0.50 | 483.62295082 | 19 | 61 | 0 | 1 |
| Path 480 | C00024->C00668:[49->13,49->7,49->8,50->12,50->6] | 0.83 | 495.353846154 | 22 | 65 | 0 | 1 |
| Path 481 | C00024->C00668:[49->13,49->7,49->8,50->12,50->6] | 0.83 | 371.813333333 | 23 | 75 | 0 | 1 |
| Path 482 | C00024->C00668:[2->10,49->7,50->6,6->8] | 0.67 | 357.735714286 | 18 | 140 | 0 | 0 |
| Path 483 | C00024->C00668:[49->13,49->7,49->8,50->12,50->6] | 0.83 | 439.129411765 | 25 | 85 | 0 | 1 |
| Path 484 | C00024->C00668:[49->13,49->7,49->8,50->12,50->6] | 0.83 | 416.483333333 | 23 | 60 | 0 | 1 |
| Path 485 | C00024->C00668:[49->7,49->8,50->6] | 0.50 | 508.672413793 | 20 | 58 | 0 | 1 |
| Path 486 | C00024->C00668:[49->13,50->12] | 0.33 | 497.090909091 | 7 | 11 | 0 | 0 |
| Path 487 | C00024->C00668:[49->7,49->8,50->6] | 0.50 | 332.672413793 | 17 | 58 | 0 | 1 |
| Path 488 | C00024->C00668:[10->7,11->8,15->6,2->12,6->10] | 0.83 | 358.895522388 | 18 | 134 | 0 | 0 |
| Path 489 | C00024->C00668:[49->7,49->8,50->6] | 0.50 | 351.327272727 | 16 | 55 | 0 | 1 |
| Path 490 | C00024->C00668:[49->13,49->7,50->12,50->6] | 0.67 | 514.5625 | 16 | 48 | 0 | 1 |
| Path 491 | C00024->C00668:[49->7,49->8,50->6] | 0.50 | 439.666666667 | 17 | 48 | 0 | 1 |
| Path 492 | C00024->C00668:[49->13,49->7,49->8,50->12,50->6] | 0.83 | 379.141025641 | 19 | 78 | 0 | 0 |
| Path 493 | C00024->C00668:[49->13,49->7,50->12,50->6] | 0.67 | 523.06122449 | 17 | 49 | 0 | 1 |
| Path 494 | C00024->C00668:[2->10,49->13,49->7,50->12,50->6] | 0.83 | 366.988235294 | 23 | 170 | 0 | 1 |
| Path 495 | C00024->C00668:[49->13,50->12,50->6] | 0.50 | 540.52173913 | 19 | 46 | 0 | 0 |
| Path 496 | C00024->C00668:[49->7,50->6,50->8] | 0.50 | 499.150943396 | 18 | 53 | 0 | 0 |
| Path 497 | C00024->C00668:[2->10,49->13,50->12] | 0.50 | 309.414965986 | 15 | 147 | 0 | 0 |
| Path 498 | C00024->C00668:[14->8,49->7,50->6] | 0.50 | 359.309090909 | 19 | 165 | 0 | 1 |
| Path 499 | C00024->C00668:[49->13,49->7,49->8,50->12,50->6] | 0.83 | 451.895522388 | 22 | 67 | 0 | 1 |
| Path 500 | C00024->C00668:[49->13,49->7,49->8,50->12,50->6] | 0.83 | 543.639344262 | 21 | 61 | 0 | 1 |
| Path 501 | C00024->C00668:[49->13,49->7,50->12,50->6] | 0.67 | 565.774193548 | 16 | 31 | 0 | 0 |
| Path 502 | C00024->C00668:[2->10] | 0.17 | 324.026666667 | 13 | 75 | 0 | 0 |
| Path 503 | C00024->C00668:[49->13,49->7,50->12,50->6] | 0.67 | 361.774193548 | 18 | 62 | 0 | 0 |
| Path 504 | C00024->C00668:[10->7,11->8,15->6,49->13,50->12,6->10] | 1.00 | 328.884615385 | 21 | 156 | 0 | 0 |
| Path 505 | C00024->C00668:[49->13,49->7,49->8,50->12,50->6] | 0.83 | 506.5 | 23 | 68 | 0 | 1 |
| Path 506 | C00024->C00668:[49->7,49->8,50->6] | 0.50 | 411.894736842 | 15 | 57 | 0 | 1 |
| Path 507 | C00024->C00668:[49->13,49->7,49->8,50->12,50->6] | 0.83 | 543.527272727 | 19 | 55 | 0 | 0 |
| Path 508 | C00024->C00668:[49->13,49->7,49->8,50->12,50->6] | 0.83 | 395.305555556 | 18 | 72 | 0 | 1 |
| Path 509 | C00024->C00668:[2->10,49->13,50->12] | 0.50 | 300.68 | 19 | 100 | 0 | 0 |
| Path 510 | C00024->C00668:[49->7,50->6] | 0.33 | 385.1 | 13 | 40 | 0 | 1 |
| Path 511 | C00024->C00668:[14->8,49->7,50->6] | 0.50 | 371.567901235 | 20 | 162 | 0 | 0 |
| Path 512 | C00024->C00668:[49->13,49->7,49->8,50->12,50->6] | 0.83 | 426.409836066 | 24 | 61 | 0 | 1 |
| Path 513 | C00024->C00668:[49->13,49->7,49->8,50->12,50->6] | 0.83 | 367.484375 | 19 | 64 | 0 | 0 |
| Path 514 | C00024->C00668:[2->10,49->13,49->7,49->8,50->12,50->6] | 1.00 | 373.024590164 | 25 | 122 | 0 | 1 |
| Path 515 | C00024->C00668:[49->7,49->8,50->6] | 0.50 | 513.523809524 | 13 | 42 | 0 | 0 |
| Path 516 | C00024->C00668:[49->13,49->7,50->12,50->6] | 0.67 | 439.979591837 | 18 | 49 | 0 | 1 |
| Path 517 | C00024->C00668:[10->7,11->8,15->6,49->13,50->12] | 0.83 | 359.864661654 | 17 | 133 | 0 | 0 |
| Path 518 | C00024->C00668:[14->8,49->13,49->7,50->12,50->6] | 0.83 | 352.64084507 | 26 | 142 | 0 | 0 |
| Path 519 | C00024->C00668:[49->7,49->8,50->6] | 0.50 | 403.410714286 | 14 | 56 | 0 | 1 |
| Path 520 | C00024->C00668:[49->7,49->8,50->6] | 0.50 | 443.734693878 | 19 | 49 | 0 | 1 |
| Path 521 | C00024->C00668:[49->13,49->7,49->8,50->12,50->6] | 0.83 | 348.298429319 | 25 | 191 | 0 | 1 |
| Path 522 | C00024->C00668:[49->7,49->8,50->6] | 0.50 | 518.395833333 | 17 | 48 | 0 | 1 |
| Path 523 | C00024->C00668:[49->7,50->6,50->8] | 0.50 | 437.591836735 | 18 | 49 | 0 | 1 |
| Path 524 | C00024->C00668:[49->13,49->7,50->12,50->6] | 0.67 | 508.833333333 | 18 | 54 | 0 | 1 |
| Path 525 | C00024->C00668:[49->13,49->7,50->12,50->6] | 0.67 | 432.481481481 | 21 | 54 | 0 | 1 |
| Path 526 | C00024->C00668:[49->7,49->8,50->6] | 0.50 | 406.745098039 | 13 | 51 | 0 | 1 |
| Path 527 | C00024->C00668:[49->13,49->7,50->12,50->6] | 0.67 | 384.296875 | 15 | 64 | 0 | 1 |
| Path 528 | C00024->C00668:[2->10,49->13,49->7,49->8,50->12,50->6] | 1.00 | 361.642487047 | 29 | 193 | 0 | 0 |
| Path 529 | C00024->C00668:[49->7,49->8,50->6] | 0.50 | 452.181818182 | 18 | 44 | 0 | 1 |
| Path 530 | C00024->C00668:[49->13,49->7,49->8,50->12,50->6] | 0.83 | 394.705882353 | 21 | 85 | 0 | 1 |
| Path 531 | C00024->C00668:[49->13,49->7,49->8,50->12,50->6] | 0.83 | 383.342857143 | 23 | 70 | 0 | 1 |
| Path 532 | C00024->C00668:[49->7,49->8,50->6] | 0.50 | 318.945054945 | 19 | 91 | 0 | 1 |
| Path 533 | C00024->C00668:[49->7,49->8,50->6] | 0.50 | 387.133333333 | 15 | 60 | 0 | 1 |
| Path 534 | C00024->C00668:[2->10,49->7,50->6] | 0.50 | 381.02994012 | 23 | 167 | 0 | 0 |
| Path 535 | C00024->C00668:[49->13,50->12] | 0.33 | 501.363636364 | 7 | 11 | 0 | 0 |
| Path 536 | C00024->C00668:[14->8,49->7,50->6] | 0.50 | 337.260869565 | 20 | 161 | 0 | 1 |
| Path 537 | C00024->C00668:[49->7,49->8,50->6] | 0.50 | 328.139534884 | 18 | 86 | 0 | 1 |
| Path 538 | C00024->C00668:[49->7,50->6] | 0.33 | 520.617647059 | 10 | 34 | 0 | 0 |
| Path 539 | C00024->C00668:[49->13,49->7,50->12,50->6] | 0.67 | 326.8 | 16 | 60 | 0 | 1 |
| Path 540 | C00024->C00668:[49->7,49->8,50->6] | 0.50 | 357.377777778 | 17 | 90 | 0 | 1 |
| Path 541 | C00024->C00668:[49->7,49->8,50->6] | 0.50 | 480.636363636 | 17 | 44 | 0 | 0 |
| Path 542 | C00024->C00668:[49->13,49->7,49->8,50->12,50->6] | 0.83 | 342.918032787 | 24 | 122 | 0 | 1 |
| Path 543 | C00024->C00668:[49->13,50->10,50->12] | 0.50 | 674.05 | 15 | 20 | 0 | 0 |
| Path 544 | C00024->C00668:[49->13,49->7,50->10,50->12,50->6] | 0.83 | 409.287671233 | 21 | 73 | 0 | 1 |
| Path 545 | C00024->C00668:[49->7,50->6] | 0.33 | 506.357142857 | 12 | 42 | 0 | 0 |
| Path 546 | C00024->C00668:[49->13,49->7,49->8,50->12,50->6] | 0.83 | 500.575757576 | 23 | 66 | 0 | 1 |
| Path 547 | C00024->C00668:[49->7,49->8,50->6] | 0.50 | 316.011627907 | 18 | 86 | 0 | 1 |
| Path 548 | C00024->C00668:[49->13,49->7,49->8,50->12,50->6] | 0.83 | 430.013513514 | 22 | 74 | 0 | 1 |
| Path 549 | C00024->C00668:[49->7,49->8,50->6] | 0.50 | 348.886792453 | 16 | 53 | 0 | 1 |
| Path 550 | C00024->C00668:[49->13,49->7,49->8,50->12,50->6] | 0.83 | 433.170454545 | 25 | 88 | 0 | 1 |
| Path 551 | C00024->C00668:[49->7,49->8,50->6] | 0.50 | 493.821428571 | 18 | 56 | 0 | 1 |
| Path 552 | C00024->C00668:[2->10,49->13,50->12] | 0.50 | 308.525974026 | 18 | 154 | 0 | 0 |
| Path 553 | C00024->C00668:[49->13,49->7,50->12,50->6] | 0.67 | 405.184210526 | 18 | 76 | 0 | 1 |
| Path 554 | C00024->C00668:[14->8,49->7,50->6] | 0.50 | 358.99375 | 18 | 160 | 0 | 1 |
| Path 555 | C00024->C00668:[49->13,49->7,49->8,50->12,50->6] | 0.83 | 376.153846154 | 20 | 65 | 0 | 0 |
| Path 556 | C00024->C00668:[2->10,49->13,50->12] | 0.50 | 319.280487805 | 16 | 82 | 0 | 0 |
| Path 557 | C00024->C00668:[49->13,49->7,50->12,50->6] | 0.67 | 500.867924528 | 17 | 53 | 0 | 1 |
| Path 558 | C00024->C00668:[49->7,49->8,50->6] | 0.50 | 546.156862745 | 17 | 51 | 0 | 0 |
| Path 559 | C00024->C00668:[49->7,49->8,50->6] | 0.50 | 338.16025641 | 20 | 156 | 0 | 1 |
| Path 560 | C00024->C00668:[49->13,49->7,49->8,50->12,50->6] | 0.83 | 434.857142857 | 22 | 56 | 0 | 1 |
| Path 561 | C00024->C00668:[49->13,49->7,49->8,50->10,50->12,50->6] | 1.00 | 534.456140351 | 23 | 57 | 0 | 1 |
| Path 562 | C00024->C00668:[49->7,49->8,50->6] | 0.50 | 489.339622642 | 17 | 53 | 0 | 1 |
| Path 563 | C00024->C00668:[49->7,49->8,50->6] | 0.50 | 370.022988506 | 16 | 87 | 0 | 1 |
| Path 564 | C00024->C00668:[49->7,49->8,50->6] | 0.50 | 479.926829268 | 12 | 41 | 0 | 1 |
| Path 565 | C00024->C00668:[49->7,50->6,50->8] | 0.50 | 428.760869565 | 17 | 46 | 0 | 1 |
| Path 566 | C00024->C00668:[49->7,49->8,50->6] | 0.50 | 451.921568627 | 15 | 51 | 0 | 1 |
| Path 567 | C00024->C00668:[49->13,49->7,50->12,50->6] | 0.67 | 397.890410959 | 17 | 73 | 0 | 1 |
| Path 568 | C00024->C00668:[2->10,49->13,50->12] | 0.50 | 320.078431373 | 18 | 153 | 0 | 0 |
| Path 569 | C00024->C00668:[49->13,50->12] | 0.33 | 558.076923077 | 8 | 13 | 0 | 0 |
| Path 570 | C00024->C00668:[49->13,49->7,49->8,50->12,50->6] | 0.83 | 538.7 | 20 | 60 | 0 | 1 |
| Path 571 | C00024->C00668:[49->13,49->7,50->12,50->6] | 0.67 | 338.515151515 | 18 | 66 | 0 | 1 |
| Path 572 | C00024->C00668:[49->13,50->12] | 0.33 | 238.52 | 6 | 25 | 0 | 0 |
| Path 573 | C00024->C00668:[49->13,49->7,49->8,50->12,50->6] | 0.83 | 364.608333333 | 23 | 120 | 0 | 0 |
| Path 574 | C00024->C00668:[49->13,49->7,50->12,50->6] | 0.67 | 443.727272727 | 16 | 44 | 0 | 0 |
| Path 575 | C00024->C00668:[49->13,49->7,49->8,50->12,50->6] | 0.83 | 332.313432836 | 19 | 67 | 0 | 1 |
| Path 576 | C00024->C00668:[49->13,49->7,49->8,50->12,50->6] | 0.83 | 510.542857143 | 24 | 70 | 0 | 1 |
| Path 577 | C00024->C00668:[49->13,49->7,49->8,50->12,50->6] | 0.83 | 367.742268041 | 23 | 97 | 0 | 1 |
| Path 578 | C00024->C00668:[49->7,49->8,50->6] | 0.50 | 325.119565217 | 20 | 92 | 0 | 1 |
| Path 579 | C00024->C00668:[49->13,49->7,50->12,50->6] | 0.67 | 352.442622951 | 17 | 61 | 0 | 0 |
| Path 580 | C00024->C00668:[49->7,49->8,50->6] | 0.50 | 398.275862069 | 15 | 58 | 0 | 1 |
| Path 581 | C00024->C00668:[49->7,49->8,50->6] | 0.50 | 505.636363636 | 17 | 55 | 0 | 1 |
| Path 582 | C00024->C00668:[2->10,49->13,49->7,50->12,50->6,6->8] | 1.00 | 337.16875 | 21 | 160 | 0 | 0 |
| Path 583 | C00024->C00668:[49->13,49->7,49->8,50->12,50->6] | 0.83 | 476.892307692 | 23 | 65 | 0 | 1 |
| Path 584 | C00024->C00668:[49->7,49->8,50->6] | 0.50 | 343.408536585 | 22 | 164 | 0 | 1 |
| Path 585 | C00024->C00668:[2->10,49->13,50->12] | 0.50 | 313.0 | 16 | 148 | 0 | 0 |
| Path 586 | C00024->C00668:[49->7,49->8,50->6] | 0.50 | 435.288461538 | 14 | 52 | 0 | 0 |
| Path 587 | C00024->C00668:[49->13,49->7,49->8,50->12,50->6] | 0.83 | 453.442307692 | 20 | 52 | 0 | 1 |
| Path 588 | C00024->C00668:[49->7,50->6,50->8] | 0.50 | 494.2 | 16 | 50 | 0 | 1 |
| Path 589 | C00024->C00668:[2->10,49->13,49->7,49->8,50->12,50->6] | 1.00 | 377.56097561 | 26 | 123 | 0 | 1 |
| Path 590 | C00024->C00668:[49->13,49->7,50->10,50->12,50->6] | 0.83 | 532.224137931 | 23 | 58 | 0 | 1 |
| Path 591 | C00024->C00668:[2->10,49->13,50->12] | 0.50 | 343.515384615 | 15 | 130 | 0 | 0 |
| Path 592 | C00024->C00668:[49->13,49->7,49->8,50->12,50->6] | 0.83 | 421.193548387 | 25 | 93 | 0 | 1 |
| Path 593 | C00024->C00668:[49->13,49->7,49->8,50->12,50->6] | 0.83 | 462.452830189 | 21 | 53 | 0 | 1 |
| Path 594 | C00024->C00668:[49->13,49->7,49->8,50->12,50->6] | 0.83 | 519.984615385 | 22 | 65 | 0 | 1 |
| Path 595 | C00024->C00668:[49->13,50->10,50->12] | 0.50 | 610.0 | 16 | 23 | 0 | 0 |
| Path 596 | C00024->C00668:[49->7,49->8,50->6] | 0.50 | 405.688888889 | 15 | 45 | 0 | 1 |
| Path 597 | C00024->C00668:[2->10] | 0.17 | 338.16 | 12 | 125 | 0 | 0 |
| Path 598 | C00024->C00668:[49->7,50->6] | 0.33 | 463.431818182 | 12 | 44 | 0 | 1 |
| Path 599 | C00024->C00668:[49->7,50->6,50->8] | 0.50 | 521.607843137 | 18 | 51 | 0 | 1 |
| Path 600 | C00024->C00668:[49->13,50->10,50->12] | 0.50 | 603.476190476 | 16 | 21 | 0 | 0 |
| Path 601 | C00024->C00668:[49->7,49->8,50->6] | 0.50 | 399.507936508 | 17 | 63 | 0 | 1 |
| Path 602 | C00024->C00668:[49->13,49->7,49->8,50->12,50->6] | 0.83 | 446.932432432 | 21 | 74 | 0 | 0 |
| Path 603 | C00024->C00668:[2->10,49->13,50->12] | 0.50 | 313.614864865 | 16 | 148 | 0 | 0 |
| Path 604 | C00024->C00668:[49->13,50->10,50->12] | 0.50 | 539.64 | 15 | 25 | 0 | 0 |
| Path 605 | C00024->C00668:[49->13,49->7,50->12,50->6] | 0.67 | 470.58490566 | 17 | 53 | 0 | 1 |
| Path 606 | C00024->C00668:[49->13,49->7,49->8,50->12,50->6] | 0.83 | 413.333333333 | 23 | 84 | 0 | 1 |
| Path 607 | C00024->C00668:[49->7,49->8,50->6] | 0.50 | 517.488888889 | 14 | 45 | 0 | 0 |
| Path 608 | C00024->C00668:[49->13,49->7,49->8,50->12,50->6] | 0.83 | 438.98630137 | 20 | 73 | 0 | 0 |
| Path 609 | C00024->C00668:[49->7,49->8,50->6] | 0.50 | 412.033898305 | 15 | 59 | 0 | 1 |
| Path 610 | C00024->C00668:[49->7,49->8,50->6] | 0.50 | 448.795918367 | 18 | 49 | 0 | 1 |
| Path 611 | C00024->C00668:[49->7,49->8,50->6] | 0.50 | 476.829787234 | 14 | 47 | 0 | 1 |
| Path 612 | C00024->C00668:[49->13,49->7,50->12,50->6] | 0.67 | 434.732394366 | 19 | 71 | 0 | 0 |
| Path 613 | C00024->C00668:[49->13,50->12] | 0.33 | 693.75 | 8 | 8 | 0 | 0 |
| Path 614 | C00024->C00668:[49->13,49->7,50->10,50->12,50->6] | 0.83 | 549.563636364 | 22 | 55 | 0 | 1 |
| Path 615 | C00024->C00668:[49->13,49->7,49->8,50->12,50->6] | 0.83 | 351.104166667 | 26 | 192 | 0 | 1 |
| Path 616 | C00024->C00668:[2->10,49->7,50->6] | 0.50 | 369.246478873 | 20 | 142 | 0 | 0 |
| Path 617 | C00024->C00668:[2->10,49->13,50->12] | 0.50 | 340.987654321 | 17 | 81 | 0 | 0 |
| Path 618 | C00024->C00668:[49->13,49->7,49->8,50->10,50->12,50->6] | 1.00 | 534.440677966 | 25 | 59 | 0 | 1 |
| Path 619 | C00024->C00668:[49->13,49->7,49->8,50->12,50->6] | 0.83 | 401.228571429 | 22 | 70 | 0 | 0 |
| Path 620 | C00024->C00668:[2->10,49->13,50->12] | 0.50 | 334.462121212 | 15 | 132 | 0 | 0 |
| Path 621 | C00024->C00668:[49->7,50->6,50->8] | 0.50 | 508.017857143 | 19 | 56 | 0 | 1 |
| Path 622 | C00024->C00668:[49->13,49->7,49->8,50->12,50->6] | 0.83 | 544.409836066 | 21 | 61 | 0 | 1 |
| Path 623 | C00024->C00668:[49->13,49->7,49->8,50->10,50->12,50->6] | 1.00 | 512.746031746 | 24 | 63 | 0 | 1 |
| Path 624 | C00024->C00668:[49->7,50->6,50->8] | 0.50 | 498.660377358 | 17 | 53 | 0 | 1 |
| Path 625 | C00024->C00668:[49->13,49->7,49->8,50->12,50->6] | 0.83 | 508.343283582 | 22 | 67 | 0 | 1 |
| Path 626 | C00024->C00668:[49->13,49->7,50->12,50->6] | 0.67 | 536.038461538 | 17 | 52 | 0 | 0 |
| Path 627 | C00024->C00668:[2->10,49->13,50->12] | 0.50 | 324.473333333 | 18 | 150 | 0 | 0 |
| Path 628 | C00024->C00668:[49->7,49->8,50->6] | 0.50 | 361.133333333 | 20 | 165 | 0 | 1 |
| Path 629 | C00024->C00668:[49->13,50->12,50->6] | 0.50 | 571.23255814 | 19 | 43 | 0 | 0 |
| Path 630 | C00024->C00668:[49->13,49->7,49->8,50->10,50->12,50->6] | 1.00 | 535.280701754 | 23 | 57 | 0 | 1 |
| Path 631 | C00024->C00668:[49->13,49->7,49->8,50->12,50->6] | 0.83 | 494.0 | 21 | 61 | 0 | 1 |
| Path 632 | C00024->C00668:[49->7,49->8,50->6] | 0.50 | 358.010526316 | 18 | 95 | 0 | 1 |
| Path 633 | C00024->C00668:[49->13,49->7,50->12,50->6] | 0.67 | 479.280701754 | 19 | 57 | 0 | 1 |
| Path 634 | C00024->C00668:[49->13,49->7,49->8,50->12,50->6] | 0.83 | 459.966666667 | 20 | 60 | 0 | 1 |
| Path 635 | C00024->C00668:[49->13,49->7,50->12,50->6] | 0.67 | 360.306451613 | 18 | 62 | 0 | 0 |
| Path 636 | C00024->C00668:[49->7,49->8,50->6] | 0.50 | 544.479166667 | 16 | 48 | 0 | 0 |
| Path 637 | C00024->C00668:[49->13,49->7,49->8,50->12,50->6] | 0.83 | 429.671875 | 18 | 64 | 0 | 0 |
| Path 638 | C00024->C00668:[14->8,49->7,50->6] | 0.50 | 332.784810127 | 19 | 158 | 0 | 1 |
| Path 639 | C00024->C00668:[49->13,49->7,49->8,50->12,50->6] | 0.83 | 329.080213904 | 26 | 187 | 0 | 1 |
| Path 640 | C00024->C00668:[49->13,49->7,50->12,50->6] | 0.67 | 514.93220339 | 20 | 59 | 0 | 1 |
| Path 641 | C00024->C00668:[49->13,49->7,49->8,50->12,50->6] | 0.83 | 426.17721519 | 23 | 79 | 0 | 1 |
| Path 642 | C00024->C00668:[49->13,49->7,50->12,50->6] | 0.67 | 436.014084507 | 19 | 71 | 0 | 0 |
| Path 643 | C00024->C00668:[49->13,50->12] | 0.33 | 696.142857143 | 10 | 14 | 0 | 0 |
| Path 644 | C00024->C00668:[49->13,49->7,49->8,50->10,50->12,50->6] | 1.00 | 539.163934426 | 25 | 61 | 0 | 1 |
| Path 645 | C00024->C00668:[49->13,50->12] | 0.33 | 690.7 | 8 | 10 | 0 | 0 |
| Path 646 | C00024->C00668:[49->13,49->7,49->8,50->12,50->6] | 0.83 | 406.333333333 | 20 | 81 | 0 | 1 |
| Path 647 | C00024->C00668:[49->13,49->7,49->8,50->10,50->12,50->6] | 1.00 | 538.393442623 | 25 | 61 | 0 | 1 |
| Path 648 | C00024->C00668:[49->13,49->7,49->8,50->10,50->12,50->6] | 1.00 | 533.644067797 | 25 | 59 | 0 | 1 |
| Path 649 | C00024->C00668:[49->13,49->7,49->8,50->12,50->6] | 0.83 | 423.158730159 | 17 | 63 | 0 | 0 |
| Path 650 | C00024->C00668:[2->10,49->13,50->12] | 0.50 | 301.009708738 | 19 | 103 | 0 | 0 |
| Path 651 | C00024->C00668:[49->13,49->7,49->8,50->12,50->6] | 0.83 | 483.657142857 | 22 | 70 | 0 | 1 |
| Path 652 | C00024->C00668:[49->13,50->12] | 0.33 | 586.2 | 9 | 15 | 0 | 0 |
| Path 653 | C00024->C00668:[49->7,49->8,50->6] | 0.50 | 360.875 | 19 | 160 | 0 | 1 |
| Path 654 | C00024->C00668:[49->7,49->8,50->6] | 0.50 | 521.811320755 | 19 | 53 | 0 | 1 |
| Path 655 | C00024->C00668:[49->13,49->7,50->12,50->6] | 0.67 | 488.637931034 | 20 | 58 | 0 | 1 |
| Path 656 | C00024->C00668:[49->13,49->7,49->8,50->12,50->6] | 0.83 | 320.738095238 | 26 | 126 | 0 | 1 |
| Path 657 | C00024->C00668:[49->13,49->7,50->10,50->12,50->6] | 0.83 | 526.740740741 | 21 | 54 | 0 | 1 |
| Path 658 | C00024->C00668:[49->13,49->7,50->12,50->6] | 0.67 | 414.03030303 | 17 | 66 | 0 | 0 |
| Path 659 | C00024->C00668:[49->7,49->8,50->6] | 0.50 | 368.966666667 | 17 | 90 | 0 | 1 |
| Path 660 | C00024->C00668:[49->12,49->7,49->8,50->13,50->6] | 0.83 | 522.295081967 | 21 | 61 | 0 | 1 |
| Path 661 | C00024->C00668:[49->13,49->7,49->8,50->12,50->6] | 0.83 | 445.635135135 | 21 | 74 | 0 | 0 |
| Path 662 | C00024->C00668:[49->7,49->8,50->6] | 0.50 | 474.346938776 | 14 | 49 | 0 | 1 |
| Path 663 | C00024->C00668:[49->13,49->7,49->8,50->12,50->6] | 0.83 | 425.304347826 | 19 | 69 | 0 | 0 |
| Path 664 | C00024->C00668:[2->10,49->13,50->12] | 0.50 | 351.727272727 | 17 | 132 | 0 | 0 |
| Path 665 | C00024->C00668:[49->7,49->8,50->6] | 0.50 | 467.913043478 | 13 | 46 | 0 | 1 |
| Path 666 | C00024->C00668:[49->7,49->8,50->6] | 0.50 | 397.027027027 | 13 | 37 | 0 | 1 |
| Path 667 | C00024->C00668:[49->13,49->7,50->12,50->6] | 0.67 | 536.931034483 | 19 | 58 | 0 | 1 |
| Path 668 | C00024->C00668:[49->7,50->6,50->8] | 0.50 | 494.72 | 17 | 50 | 0 | 0 |
| Path 669 | C00024->C00668:[2->10,49->13,50->12] | 0.50 | 283.670103093 | 16 | 97 | 0 | 0 |
| Path 670 | C00024->C00668:[49->7,50->6] | 0.33 | 472.585365854 | 11 | 41 | 0 | 1 |
| Path 671 | C00024->C00668:[49->7,50->6,50->8] | 0.50 | 429.326086957 | 18 | 46 | 0 | 0 |
| Path 672 | C00024->C00668:[49->13,49->7,49->8,50->12,50->6] | 0.83 | 452.096385542 | 23 | 83 | 0 | 0 |
| Path 673 | C00024->C00668:[49->13,49->7,50->10,50->12,50->6] | 0.83 | 526.160714286 | 23 | 56 | 0 | 1 |
| Path 674 | C00024->C00668:[49->13,49->7,49->8,50->12,50->6] | 0.83 | 417.847457627 | 22 | 59 | 0 | 1 |
| Path 675 | C00024->C00668:[2->10,49->13,50->12] | 0.50 | 333.6125 | 16 | 80 | 0 | 0 |
| Path 676 | C00024->C00668:[49->13,49->7,50->12,50->6,50->8] | 0.83 | 416.763157895 | 22 | 76 | 0 | 0 |
| Path 677 | C00024->C00668:[49->7,50->6] | 0.33 | 461.369565217 | 12 | 46 | 0 | 1 |
| Path 678 | C00024->C00668:[49->13,50->10,50->12] | 0.50 | 612.043478261 | 16 | 23 | 0 | 0 |
| Path 679 | C00024->C00668:[49->7,49->8,50->6] | 0.50 | 485.915254237 | 19 | 59 | 0 | 1 |
| Path 680 | C00024->C00668:[49->13,49->7,49->8,50->12,50->6] | 0.83 | 463.455696203 | 23 | 79 | 0 | 0 |
| Path 681 | C00024->C00668:[49->7,50->6,50->8] | 0.50 | 508.066666667 | 15 | 45 | 0 | 1 |
| Path 682 | C00024->C00668:[49->13,49->7,50->12,50->6] | 0.67 | 501.34375 | 20 | 64 | 0 | 1 |
| Path 683 | C00024->C00668:[49->13,49->7,49->8,50->12,50->6] | 0.83 | 445.157894737 | 23 | 57 | 0 | 1 |
| Path 684 | C00024->C00668:[49->13,49->7,50->12,50->6] | 0.67 | 562.340909091 | 16 | 44 | 0 | 0 |
| Path 685 | C00024->C00668:[49->7,49->8,50->6] | 0.50 | 479.0 | 18 | 58 | 0 | 1 |
| Path 686 | C00024->C00668:[49->13,49->7,50->12,50->6] | 0.67 | 514.966666667 | 19 | 60 | 0 | 1 |
| Path 687 | C00024->C00668:[49->12,50->10,50->13] | 0.50 | 594.882352941 | 12 | 17 | 0 | 0 |
| Path 688 | C00024->C00668:[49->13,49->7,50->12,50->6] | 0.67 | 428.942857143 | 18 | 70 | 0 | 0 |
| Path 689 | C00024->C00668:[2->10,49->13,49->7,50->12,50->6] | 0.83 | 347.512345679 | 23 | 162 | 0 | 0 |
| Path 690 | C00024->C00668:[49->7,49->8,50->6] | 0.50 | 519.26 | 16 | 50 | 0 | 1 |
| Path 691 | C00024->C00668:[49->13,49->7,49->8,50->12,50->6] | 0.83 | 347.731182796 | 24 | 186 | 0 | 1 |
| Path 692 | C00024->C00668:[49->7,49->8,50->6] | 0.50 | 363.520833333 | 19 | 96 | 0 | 1 |
| Path 693 | C00024->C00668:[49->13,49->7,50->12,50->6] | 0.67 | 350.081081081 | 19 | 74 | 0 | 1 |
| Path 694 | C00024->C00668:[49->7,49->8,50->6] | 0.50 | 515.829787234 | 15 | 47 | 0 | 1 |
| Path 695 | C00024->C00668:[49->7,49->8,50->6] | 0.50 | 486.272727273 | 13 | 44 | 0 | 1 |
| Path 696 | C00024->C00668:[2->10,49->13,49->7,50->12,50->6] | 0.83 | 365.453781513 | 23 | 119 | 0 | 1 |
| Path 697 | C00024->C00668:[49->13,49->7,50->10,50->12,50->6] | 0.83 | 504.716666667 | 22 | 60 | 0 | 1 |
| Path 698 | C00024->C00668:[49->13,50->10,50->12] | 0.50 | 615.736842105 | 14 | 19 | 0 | 0 |
