# Supplementary material for: 13C-metabolic flux ratio and novel carbon path analyses confirmed that Trichoderma reesei uses primarily the respirative pathway also on the preferred carbon source glucose
Source: BMC Syst Biol. 2009 Oct 29;3:104. doi: 10.1186/1752-0509-3-104 (PMC2776023; doi:10.1186/1752-0509-3-104)
Supplement: Additional file 1 — Pathways discovered in ReTrace carbon path analysis. Graphical and tabular representations of amino acid synthesis pathways discovered in ReTrace carbon path analysis [21]. Self-contained web site: unpack zip archive and open index.html with a web browser. [file 1752-0509-3-104-S1.zip › AF1-treesei/pathways-C00031-to-C00047.html]

Pathways from C00031 to C00047


**Pathways from C00031 to C00047**

**Sources:** D-Glucose; (C00031)

**Target:**L-Lysine; (C00047)

|  | Composite mapping | Z | Average score | Rpairs | Reactions | Zero scores | Scores under threshold |
| --- | --- | --- | --- | --- | --- | --- | --- |
| Path 1 | C00031->C00047:[7->1,7->2,7->3,7->9,9->1] | 0.67 | 407.827586207 | 25 | 58 | 0 | 0 |
| Path 2 | C00031->C00047:[4->3,4->9,7->2,9->1] | 0.67 | 359.381818182 | 21 | 55 | 0 | 0 |
| Path 3 | C00031->C00047:[4->1,4->3,4->9,7->2,9->1] | 0.67 | 377.666666667 | 26 | 66 | 0 | 0 |
| Path 4 | C00031->C00047:[7->1,7->2,7->8,9->3,9->5,9->9] | 1.00 | 450.322580645 | 26 | 62 | 0 | 0 |
| Path 5 | C00031->C00047:[4->3,4->9,7->2,7->3,7->9,9->1] | 0.67 | 395.06557377 | 26 | 61 | 0 | 0 |
| Path 6 | C00031->C00047:[4->3,4->9,7->2,9->1] | 0.67 | 398.412698413 | 24 | 63 | 0 | 0 |
| Path 7 | C00031->C00047:[7->1,7->2,7->3,7->9,9->1] | 0.67 | 370.032258065 | 25 | 62 | 0 | 0 |
| Path 8 | C00031->C00047:[7->1,7->2,7->3,7->9,9->1] | 0.67 | 391.821428571 | 23 | 56 | 0 | 0 |
| Path 9 | C00031->C00047:[4->3,4->9,7->2,9->1] | 0.67 | 392.0 | 24 | 63 | 0 | 0 |
| Path 10 | C00031->C00047:[7->2,9->1,9->3,9->9] | 0.67 | 429.329787234 | 26 | 94 | 0 | 0 |
| Path 11 | C00031->C00047:[4->1,4->3,4->9,7->2,9->1] | 0.67 | 401.836065574 | 24 | 61 | 0 | 0 |
| Path 12 | C00031->C00047:[4->3,4->9,7->2,9->1] | 0.67 | 415.75 | 25 | 64 | 0 | 0 |
| Path 13 | C00031->C00047:[4->1,4->3,4->9,7->2,9->1] | 0.67 | 360.276923077 | 25 | 65 | 0 | 0 |
| Path 14 | C00031->C00047:[4->3,4->9,7->2,9->1] | 0.67 | 429.434343434 | 29 | 99 | 0 | 0 |
| Path 15 | C00031->C00047:[4->3,4->9,7->2,9->1] | 0.67 | 432.891089109 | 29 | 101 | 0 | 0 |
| Path 16 | C00031->C00047:[4->1,4->3,4->9,7->2,9->1] | 0.67 | 366.711864407 | 22 | 59 | 0 | 0 |
| Path 17 | C00031->C00047:[4->3,4->9,7->2,9->1] | 0.67 | 378.508474576 | 21 | 59 | 0 | 0 |
| Path 18 | C00031->C00047:[4->1,4->3,4->9,7->2,9->1] | 0.67 | 379.966101695 | 23 | 59 | 0 | 0 |
| Path 19 | C00031->C00047:[4->1,4->3,4->9,7->2,9->1] | 0.67 | 421.047619048 | 27 | 63 | 0 | 0 |
| Path 20 | C00031->C00047:[7->1,7->2,7->8,9->3,9->5,9->9] | 1.00 | 400.280701754 | 21 | 57 | 0 | 0 |
| Path 21 | C00031->C00047:[7->2,9->1,9->3,9->9] | 0.67 | 393.018518519 | 20 | 54 | 0 | 0 |
| Path 22 | C00031->C00047:[4->3,4->9,7->2,9->1] | 0.67 | 391.758064516 | 25 | 62 | 0 | 0 |
| Path 23 | C00031->C00047:[2->3,2->9,7->2,9->1] | 0.67 | 421.285714286 | 24 | 91 | 0 | 0 |
| Path 24 | C00031->C00047:[4->3,4->9,7->2,9->1] | 0.67 | 419.195876289 | 27 | 97 | 0 | 0 |
| Path 25 | C00031->C00047:[4->3,4->9,7->2,9->1] | 0.67 | 424.288659794 | 29 | 97 | 0 | 0 |
| Path 26 | C00031->C00047:[7->1,7->2,7->3,7->9,9->1] | 0.67 | 357.639344262 | 23 | 61 | 0 | 0 |
| Path 27 | C00031->C00047:[7->2,9->1,9->3,9->9] | 0.67 | 382.944444444 | 20 | 54 | 0 | 0 |
| Path 28 | C00031->C00047:[7->1,7->2,7->8,9->3,9->5,9->9] | 1.00 | 443.0 | 24 | 60 | 0 | 0 |
| Path 29 | C00031->C00047:[4->1,4->3,4->9,7->2,9->1] | 0.67 | 400.7 | 25 | 60 | 0 | 0 |
| Path 30 | C00031->C00047:[4->3,4->9,7->2,9->1] | 0.67 | 350.285714286 | 20 | 56 | 0 | 0 |
| Path 31 | C00031->C00047:[4->3,4->9,7->2,9->1] | 0.67 | 364.25 | 21 | 56 | 0 | 0 |
| Path 32 | C00031->C00047:[4->3,4->9,7->2,9->1] | 0.67 | 374.491803279 | 22 | 61 | 0 | 0 |
| Path 33 | C00031->C00047:[4->3,4->9,7->2,9->1] | 0.67 | 372.823529412 | 25 | 68 | 0 | 0 |
| Path 34 | C00031->C00047:[4->1,4->3,4->9,7->2,9->1] | 0.67 | 348.3125 | 23 | 64 | 0 | 0 |
| Path 35 | C00031->C00047:[1->5,4->2,7->1,7->8,9->3,9->9] | 1.00 | 412.533333333 | 27 | 75 | 0 | 0 |
| Path 36 | C00031->C00047:[7->1,7->2,7->3,7->9,9->1] | 0.67 | 357.419354839 | 24 | 62 | 0 | 0 |
| Path 37 | C00031->C00047:[4->3,4->9,7->2,7->3,7->9,9->1] | 0.67 | 390.016129032 | 24 | 62 | 0 | 0 |
| Path 38 | C00031->C00047:[7->1,7->2,7->8,9->3,9->5,9->9] | 1.00 | 435.621212121 | 27 | 66 | 0 | 0 |
| Path 39 | C00031->C00047:[7->2,7->3,7->9,9->1] | 0.67 | 426.631578947 | 28 | 95 | 0 | 0 |
| Path 40 | C00031->C00047:[4->3,4->9,7->2,9->1] | 0.67 | 407.25 | 25 | 64 | 0 | 0 |
| Path 41 | C00031->C00047:[7->1,7->2,7->8,9->3,9->5,9->9] | 1.00 | 419.379310345 | 22 | 58 | 0 | 0 |
| Path 42 | C00031->C00047:[7->1,7->2,7->3,7->9,9->1] | 0.67 | 404.771929825 | 23 | 57 | 0 | 0 |
| Path 43 | C00031->C00047:[4->3,4->9,7->2,7->3,7->9,9->1] | 0.67 | 382.883333333 | 24 | 60 | 0 | 0 |
| Path 44 | C00031->C00047:[4->1,4->3,4->9,7->2,9->1] | 0.67 | 412.412698413 | 27 | 63 | 0 | 0 |
| Path 45 | C00031->C00047:[4->3,4->9,7->2,9->1] | 0.67 | 383.620689655 | 23 | 58 | 0 | 0 |
| Path 46 | C00031->C00047:[7->2,9->1,9->3,9->9] | 0.67 | 443.885416667 | 28 | 96 | 0 | 0 |
| Path 47 | C00031->C00047:[7->1,7->2,7->3,7->9,9->1] | 0.67 | 375.357142857 | 22 | 56 | 0 | 0 |
