# Supplementary material for: 13C-metabolic flux ratio and novel carbon path analyses confirmed that Trichoderma reesei uses primarily the respirative pathway also on the preferred carbon source glucose
Source: BMC Syst Biol. 2009 Oct 29;3:104. doi: 10.1186/1752-0509-3-104 (PMC2776023; doi:10.1186/1752-0509-3-104)
Supplement: Additional file 1 — Pathways discovered in ReTrace carbon path analysis. Graphical and tabular representations of amino acid synthesis pathways discovered in ReTrace carbon path analysis [21]. Self-contained web site: unpack zip archive and open index.html with a web browser. [file 1752-0509-3-104-S1.zip › AF1-treesei/pathways-C00197-to-C00065.html]

Pathways from C00197 to C00065


**Pathways from C00197 to C00065**

**Sources:** 3-Phospho-D-glycerate; (C00197)

**Target:**L-Serine; (C00065)

|  | Composite mapping | Z | Average score | Rpairs | Reactions | Zero scores | Scores under threshold |
| --- | --- | --- | --- | --- | --- | --- | --- |
| Path 1 | C00197->C00065:[6->3,7->1,8->2] | 1.00 | 454.0 | 5 | 12 | 0 | 0 |
| Path 2 | C00197->C00065:[6->3,7->1,8->2] | 1.00 | 470.0 | 17 | 38 | 0 | 0 |
| Path 3 | C00197->C00065:[6->3,7->1,8->2] | 1.00 | 530.895833333 | 16 | 48 | 0 | 0 |
| Path 4 | C00197->C00065:[6->3,7->1,8->2] | 1.00 | 510.425 | 14 | 40 | 0 | 0 |
| Path 5 | C00197->C00065:[6->3,7->1,8->2] | 1.00 | 275.0 | 3 | 4 | 0 | 0 |
| Path 6 | C00197->C00065:[6->3,7->1,8->2] | 1.00 | 518.893617021 | 15 | 47 | 0 | 0 |
| Path 7 | C00197->C00065:[6->3,7->1,8->2] | 1.00 | 427.782608696 | 17 | 46 | 0 | 0 |
| Path 8 | C00197->C00065:[6->3,7->1,8->2] | 1.00 | 479.085106383 | 16 | 47 | 0 | 0 |
| Path 9 | C00197->C00065:[6->3,7->1,8->2] | 1.00 | 481.905660377 | 14 | 53 | 0 | 0 |
| Path 10 | C00197->C00065:[6->3,7->1,8->2] | 1.00 | 422.058823529 | 18 | 51 | 0 | 0 |
| Path 11 | C00197->C00065:[6->3,7->1,8->2] | 1.00 | 485.242424242 | 16 | 33 | 0 | 0 |
| Path 12 | C00197->C00065:[6->3,7->1,8->2] | 1.00 | 498.975609756 | 17 | 41 | 0 | 0 |
| Path 13 | C00197->C00065:[6->3,7->1,8->2] | 1.00 | 492.964912281 | 15 | 57 | 0 | 0 |
| Path 14 | C00197->C00065:[6->3,7->1,8->2] | 1.00 | 493.259259259 | 15 | 54 | 0 | 0 |
| Path 15 | C00197->C00065:[6->3,7->1,8->2] | 1.00 | 652.1875 | 4 | 16 | 0 | 0 |
| Path 16 | C00197->C00065:[6->3,7->1,8->2] | 1.00 | 428.611111111 | 15 | 36 | 0 | 0 |
| Path 17 | C00197->C00065:[6->3,7->1,8->2] | 1.00 | 516.972222222 | 16 | 36 | 0 | 0 |
| Path 18 | C00197->C00065:[6->3,7->1,8->2] | 1.00 | 398.287878788 | 17 | 66 | 0 | 0 |
| Path 19 | C00197->C00065:[6->3,7->1,8->2] | 1.00 | 521.022727273 | 15 | 44 | 0 | 0 |
| Path 20 | C00197->C00065:[6->3,7->1,8->2] | 1.00 | 561.578947368 | 5 | 19 | 0 | 0 |
| Path 21 | C00197->C00065:[6->3,7->1,8->2] | 1.00 | 503.344827586 | 16 | 58 | 0 | 0 |
| Path 22 | C00197->C00065:[6->3,7->1,8->2] | 1.00 | 421.390243902 | 16 | 41 | 0 | 0 |
| Path 23 | C00197->C00065:[6->3,7->1,8->2] | 1.00 | 406.372881356 | 19 | 59 | 0 | 0 |
| Path 24 | C00197->C00065:[6->3,7->1,8->2] | 1.00 | 670.111111111 | 6 | 9 | 0 | 0 |
| Path 25 | C00197->C00065:[6->3,7->1,8->2] | 1.00 | 494.755555556 | 15 | 45 | 0 | 0 |
| Path 26 | C00197->C00065:[6->1,7->3,8->2] | 1.00 | 458.419354839 | 17 | 31 | 0 | 0 |
| Path 27 | C00197->C00065:[6->3,7->1,8->2] | 1.00 | 507.674418605 | 14 | 43 | 0 | 0 |
| Path 28 | C00197->C00065:[6->3,7->1,8->2] | 1.00 | 409.796296296 | 18 | 54 | 0 | 0 |
| Path 29 | C00197->C00065:[6->1,7->3,8->2] | 1.00 | 475.538461538 | 16 | 26 | 0 | 0 |
| Path 30 | C00197->C00065:[6->3,7->1,8->2] | 1.00 | 418.135135135 | 18 | 37 | 0 | 0 |
| Path 31 | C00197->C00065:[6->3,7->1,8->2] | 1.00 | 425.75 | 17 | 32 | 0 | 0 |
| Path 32 | C00197->C00065:[6->3,7->1,8->2] | 1.00 | 600.555555556 | 5 | 18 | 0 | 0 |
| Path 33 | C00197->C00065:[6->3,7->1,8->2] | 1.00 | 378.866666667 | 6 | 15 | 0 | 0 |
| Path 34 | C00197->C00065:[6->3,7->1,8->2] | 1.00 | 408.686567164 | 18 | 67 | 0 | 0 |
| Path 35 | C00197->C00065:[6->3,7->1,8->2] | 1.00 | 491.916666667 | 17 | 48 | 0 | 0 |
| Path 36 | C00197->C00065:[6->3,7->1,8->2] | 1.00 | 414.612903226 | 19 | 93 | 0 | 0 |
| Path 37 | C00197->C00065:[6->3,7->1,8->2] | 1.00 | 461.428571429 | 19 | 63 | 0 | 0 |
| Path 38 | C00197->C00065:[6->2] | 0.33 | 441.153846154 | 13 | 26 | 0 | 2 |
| Path 39 | C00197->C00065:[6->1,7->3] | 0.67 | 443.461538462 | 10 | 13 | 0 | 0 |
| Path 40 | C00197->C00065:[6->3,7->1,8->2] | 1.00 | 358.752136752 | 22 | 117 | 0 | 0 |
| Path 41 | C00197->C00065:[6->3,7->1,8->2] | 1.00 | 506.731707317 | 13 | 41 | 0 | 0 |
| Path 42 | C00197->C00065:[6->3,7->1,8->2] | 1.00 | 567.822222222 | 17 | 45 | 0 | 0 |
| Path 43 | C00197->C00065:[6->3,7->1,8->2] | 1.00 | 566.48 | 16 | 50 | 0 | 0 |
| Path 44 | C00197->C00065:[6->3,7->1,8->2] | 1.00 | 619.973684211 | 15 | 38 | 0 | 0 |
| Path 45 | C00197->C00065:[6->1,7->3] | 0.67 | 422.888888889 | 11 | 18 | 0 | 0 |
| Path 46 | C00197->C00065:[6->3,7->1,8->2] | 1.00 | 389.836734694 | 21 | 98 | 0 | 0 |
| Path 47 | C00197->C00065:[6->1] | 0.33 | 486.043478261 | 11 | 23 | 0 | 0 |
| Path 48 | C00197->C00065:[6->3,7->1,8->2] | 1.00 | 399.117021277 | 19 | 94 | 0 | 0 |
| Path 49 | C00197->C00065:[6->3,7->1,8->2] | 1.00 | 366.383928571 | 21 | 112 | 0 | 0 |
| Path 50 | C00197->C00065:[6->3,7->1,8->2] | 1.00 | 373.474576271 | 23 | 118 | 0 | 0 |
| Path 51 | C00197->C00065:[6->3,7->1,8->2] | 1.00 | 415.540983607 | 16 | 61 | 0 | 0 |
| Path 52 | C00197->C00065:[6->2] | 0.33 | 451.170212766 | 21 | 47 | 0 | 1 |
| Path 53 | C00197->C00065:[6->3,7->1,8->2] | 1.00 | 407.070707071 | 22 | 99 | 0 | 0 |
| Path 54 | C00197->C00065:[6->3,7->1,8->2] | 1.00 | 524.428571429 | 14 | 49 | 0 | 0 |
| Path 55 | C00197->C00065:[6->3,7->1,8->2] | 1.00 | 396.336956522 | 18 | 92 | 0 | 0 |
| Path 56 | C00197->C00065:[6->1] | 0.33 | 511.416666667 | 12 | 24 | 0 | 0 |
| Path 57 | C00197->C00065:[6->3,7->1,8->2] | 1.00 | 512.541666667 | 13 | 48 | 0 | 0 |
| Path 58 | C00197->C00065:[6->3,7->1,8->2] | 1.00 | 480.714285714 | 15 | 35 | 0 | 0 |
| Path 59 | C00197->C00065:[6->3,7->1,8->2] | 1.00 | 408.32183908 | 17 | 87 | 0 | 0 |
| Path 60 | C00197->C00065:[6->3,7->1,8->2] | 1.00 | 569.720930233 | 16 | 43 | 0 | 0 |
| Path 61 | C00197->C00065:[6->3,7->1,8->2] | 1.00 | 532.054545455 | 17 | 55 | 0 | 0 |
| Path 62 | C00197->C00065:[6->3,7->1,8->2] | 1.00 | 426.5 | 17 | 62 | 0 | 0 |
| Path 63 | C00197->C00065:[6->2,7->1,8->3] | 1.00 | 461.296296296 | 26 | 54 | 0 | 1 |
| Path 64 | C00197->C00065:[6->3,7->1,8->2] | 1.00 | 520.738095238 | 14 | 42 | 0 | 0 |
| Path 65 | C00197->C00065:[6->3,7->1,8->2] | 1.00 | 462.647058824 | 14 | 34 | 0 | 0 |
| Path 66 | C00197->C00065:[6->3,7->1,8->2] | 1.00 | 477.909090909 | 14 | 33 | 0 | 0 |
| Path 67 | C00197->C00065:[6->3,7->1,8->2] | 1.00 | 416.978947368 | 20 | 95 | 0 | 0 |
| Path 68 | C00197->C00065:[6->3,7->1,8->2] | 1.00 | 535.489795918 | 19 | 49 | 0 | 0 |
| Path 69 | C00197->C00065:[6->3,7->1,8->2] | 1.00 | 458.625 | 13 | 32 | 0 | 0 |
