# Supplementary material for: 13C-metabolic flux ratio and novel carbon path analyses confirmed that Trichoderma reesei uses primarily the respirative pathway also on the preferred carbon source glucose
Source: BMC Syst Biol. 2009 Oct 29;3:104. doi: 10.1186/1752-0509-3-104 (PMC2776023; doi:10.1186/1752-0509-3-104)
Supplement: Additional file 1 — Pathways discovered in ReTrace carbon path analysis. Graphical and tabular representations of amino acid synthesis pathways discovered in ReTrace carbon path analysis [21]. Self-contained web site: unpack zip archive and open index.html with a web browser. [file 1752-0509-3-104-S1.zip › AF1-treesei/pathways-C00022-C00036-to-C00407.html]

Pathways from C00022,C00036 to C00407


**Pathways from C00022-C00036 to C00407**

**Sources:** Pyruvate; (C00022)
Oxaloacetate; (C00036)

**Target:**L-Isoleucine; (C00407)

|  | Composite mapping | Z | Average score | Rpairs | Reactions | Zero scores | Scores under threshold |
| --- | --- | --- | --- | --- | --- | --- | --- |
| Path 1 | C00022->C00407:[1->2,1->9,3->5,3->6], C00036->C00407:[1->9,2->5,5->1,5->3] | 1.00 | 324.948717949 | 21 | 78 | 0 | 0 |
| Path 2 | C00022->C00407:[1->1,1->2,1->5,2->3,3->6,3->9] | 1.00 | 502.590909091 | 15 | 22 | 0 | 0 |
| Path 3 | C00022->C00407:[1->1,1->2,1->5,2->3,3->6,3->9] | 1.00 | 325.02 | 18 | 50 | 0 | 0 |
| Path 4 | C00022->C00407:[1->2,3->6], C00036->C00407:[1->9,2->5,5->1,5->3] | 1.00 | 296.056603774 | 21 | 53 | 0 | 0 |
| Path 5 | C00022->C00407:[1->2,3->6], C00036->C00407:[1->9,2->5,5->1,5->3] | 1.00 | 318.512195122 | 18 | 41 | 0 | 0 |
| Path 6 | C00022->C00407:[1->1,1->2,1->5,2->3,3->6,3->9] | 1.00 | 409.878787879 | 17 | 33 | 0 | 0 |
| Path 7 | C00022->C00407:[1->1,1->2,1->5,2->3,3->6,3->9] | 1.00 | 536.45 | 17 | 40 | 0 | 0 |
| Path 8 | C00022->C00407:[1->1,1->2,1->5,2->3,3->6,3->9] | 1.00 | 437.068965517 | 16 | 29 | 0 | 0 |
| Path 9 | C00022->C00407:[1->1,1->2,1->5,2->3,3->6,3->9] | 1.00 | 520.7 | 17 | 40 | 0 | 0 |
| Path 10 | C00022->C00407:[1->2,3->6], C00036->C00407:[1->9,2->5,5->1,5->3] | 1.00 | 334.745098039 | 22 | 51 | 0 | 0 |
| Path 11 | C00022->C00407:[1->2,3->6], C00036->C00407:[1->9,2->5,5->1,5->3] | 1.00 | 386.949152542 | 20 | 59 | 0 | 0 |
| Path 12 | C00022->C00407:[1->2,3->6], C00036->C00407:[1->9,2->5,5->1,5->3] | 1.00 | 295.581395349 | 23 | 86 | 0 | 0 |
| Path 13 | C00022->C00407:[1->1,1->2,2->3,3->6,3->9], C00036->C00407:[2->9,5->5] | 1.00 | 316.857142857 | 17 | 49 | 0 | 0 |
| Path 14 | C00022->C00407:[1->1,1->2,1->5,2->3,3->6,3->9] | 1.00 | 485.12195122 | 21 | 41 | 0 | 0 |
| Path 15 | C00022->C00407:[1->1,1->2,2->3,2->5,3->6,3->9], C00036->C00407:[1->1,2->9,3->3,5->5] | 1.00 | 310.202702703 | 20 | 74 | 0 | 0 |
| Path 16 | C00022->C00407:[1->2,1->9,3->5,3->6], C00036->C00407:[1->9,2->5,5->1,5->3] | 1.00 | 330.873417722 | 22 | 79 | 0 | 0 |
| Path 17 | C00022->C00407:[1->1,1->2,1->5,2->3,3->6,3->9] | 1.00 | 536.45 | 17 | 40 | 0 | 0 |
| Path 18 | C00022->C00407:[1->2,1->9,2->3,3->5,3->6], C00036->C00407:[1->9,2->5,5->1,5->3] | 1.00 | 364.104477612 | 24 | 67 | 0 | 0 |
| Path 19 | C00022->C00407:[1->1,1->2,2->3,3->6,3->9], C00036->C00407:[5->5] | 1.00 | 345.301886792 | 19 | 53 | 0 | 0 |
| Path 20 | C00022->C00407:[1->2,3->6], C00036->C00407:[1->9,2->5,5->1,5->3] | 1.00 | 353.902173913 | 22 | 92 | 0 | 0 |
| Path 21 | C00022->C00407:[1->2,1->5,3->6,3->9], C00036->C00407:[1->1,3->3] | 1.00 | 300.59375 | 14 | 32 | 0 | 0 |
| Path 22 | C00022->C00407:[1->2,1->9,3->5,3->6], C00036->C00407:[1->9,2->5,5->1,5->3] | 1.00 | 347.044444444 | 19 | 45 | 0 | 0 |
| Path 23 | C00022->C00407:[1->1,1->2,1->5,2->3,3->6,3->9] | 1.00 | 529.0 | 16 | 39 | 0 | 0 |
| Path 24 | C00022->C00407:[1->1,1->5,2->3,3->6,3->9], C00036->C00407:[2->6,5->2] | 1.00 | 441.896551724 | 17 | 29 | 0 | 0 |
| Path 25 | C00022->C00407:[1->1,1->2,1->5,2->3,3->6,3->9] | 1.00 | 531.227272727 | 15 | 22 | 0 | 0 |
| Path 26 | C00022->C00407:[1->2,1->5,3->6,3->9], C00036->C00407:[1->1,3->3] | 1.00 | 387.8 | 16 | 50 | 0 | 0 |
| Path 27 | C00022->C00407:[1->1,1->2,1->5,2->3,3->6,3->9] | 1.00 | 531.227272727 | 15 | 22 | 0 | 0 |
| Path 28 | C00022->C00407:[1->1,1->2,1->5,2->3,3->6,3->9] | 1.00 | 518.761904762 | 14 | 21 | 0 | 0 |
| Path 29 | C00022->C00407:[1->1,1->2,1->5,2->3,3->6,3->9], C00036->C00407:[1->1,3->3] | 1.00 | 338.25 | 15 | 36 | 0 | 0 |
| Path 30 | C00022->C00407:[1->2,3->6], C00036->C00407:[1->1,2->9,3->3,5->5] | 1.00 | 250.444444444 | 16 | 54 | 0 | 0 |
| Path 31 | C00022->C00407:[1->1,1->2,1->5,2->3,3->6,3->9] | 1.00 | 544.027027027 | 21 | 37 | 0 | 0 |
| Path 32 | C00022->C00407:[1->2,3->6], C00036->C00407:[1->9,2->5,5->1,5->3] | 1.00 | 313.277777778 | 25 | 90 | 0 | 0 |
| Path 33 | C00022->C00407:[1->1,1->2,2->3,3->6,3->9], C00036->C00407:[1->1,2->9,3->3,5->5] | 1.00 | 266.03125 | 19 | 64 | 0 | 0 |
| Path 34 | C00022->C00407:[1->1,1->2,2->3,2->5,3->6,3->9] | 1.00 | 367.322033898 | 19 | 59 | 0 | 0 |
| Path 35 | C00022->C00407:[1->1,1->2,1->5,2->3,3->6,3->9] | 1.00 | 529.871794872 | 16 | 39 | 0 | 0 |
| Path 36 | C00022->C00407:[1->2,1->9,2->3,3->5,3->6], C00036->C00407:[1->9,2->5,5->1] | 1.00 | 341.24 | 26 | 100 | 0 | 0 |
| Path 37 | C00022->C00407:[1->2,3->6], C00036->C00407:[1->9,2->5,5->1,5->3] | 1.00 | 307.945945946 | 20 | 74 | 0 | 0 |
| Path 38 | C00022->C00407:[1->1,1->2,1->5,2->3,3->6,3->9] | 1.00 | 517.142857143 | 14 | 21 | 0 | 0 |
| Path 39 | C00022->C00407:[1->2,1->9,3->5,3->6], C00036->C00407:[1->9,2->5,5->1,5->3] | 1.00 | 356.739130435 | 20 | 46 | 0 | 0 |
| Path 40 | C00022->C00407:[1->2,1->5,2->3,3->6,3->9] | 0.83 | 500.280701754 | 20 | 57 | 0 | 1 |
| Path 41 | C00022->C00407:[1->2,3->6], C00036->C00407:[2->9,5->3,5->5] | 0.83 | 362.92 | 22 | 75 | 0 | 1 |
| Path 42 | C00022->C00407:[1->1,1->2,2->3,3->6], C00036->C00407:[5->3] | 0.67 | 473.1 | 23 | 50 | 0 | 1 |
| Path 43 | C00022->C00407:[1->2,1->5,3->6,3->9], C00036->C00407:[5->3] | 0.83 | 326.852459016 | 21 | 61 | 0 | 1 |
| Path 44 | C00036->C00407:[2->6,2->9,5->2,5->3,5->5] | 0.83 | 296.192982456 | 20 | 57 | 0 | 1 |
| Path 45 | C00022->C00407:[1->1,1->2,1->5,2->3,3->6,3->9] | 1.00 | 379.672131148 | 20 | 61 | 0 | 0 |
| Path 46 | C00022->C00407:[1->2,1->5,3->6,3->9] | 0.67 | 436.936170213 | 22 | 47 | 0 | 0 |
| Path 47 | C00022->C00407:[1->2,2->3,2->5,3->6,3->9] | 0.83 | 411.414634146 | 23 | 82 | 0 | 1 |
| Path 48 | C00036->C00407:[2->9,5->3,5->5] | 0.50 | 396.698113208 | 20 | 53 | 0 | 1 |
| Path 49 | C00022->C00407:[2->3], C00036->C00407:[2->9,5->5] | 0.50 | 367.139240506 | 20 | 79 | 0 | 1 |
| Path 50 | C00022->C00407:[1->1,1->2,1->5,2->3,3->6,3->9] | 1.00 | 479.678571429 | 18 | 28 | 0 | 0 |
| Path 51 | C00022->C00407:[1->2,1->5,3->6,3->9], C00036->C00407:[5->3] | 0.83 | 370.803030303 | 20 | 66 | 0 | 1 |
| Path 52 | C00022->C00407:[1->2,2->3,2->5,3->6,3->9] | 0.83 | 403.171428571 | 24 | 70 | 0 | 1 |
| Path 53 | C00022->C00407:[1->2,3->6], C00036->C00407:[5->3] | 0.50 | 654.655172414 | 18 | 29 | 0 | 1 |
| Path 54 | C00022->C00407:[1->1,2->3,3->9], C00036->C00407:[5->5] | 0.67 | 336.692307692 | 18 | 52 | 0 | 0 |
| Path 55 | C00022->C00407:[1->2,3->6] | 0.33 | 365.171052632 | 21 | 76 | 0 | 0 |
| Path 56 | C00022->C00407:[1->2,3->6], C00036->C00407:[2->9,5->3,5->5] | 0.83 | 404.037037037 | 21 | 54 | 0 | 1 |
| Path 57 | C00022->C00407:[1->2,1->5,3->6,3->9] | 0.67 | 516.0 | 13 | 20 | 0 | 0 |
| Path 58 | C00022->C00407:[1->1,1->2,1->5,2->3,3->6,3->9] | 1.00 | 368.763157895 | 24 | 76 | 0 | 0 |
| Path 59 | C00022->C00407:[1->2,1->5,3->6,3->9], C00036->C00407:[5->3] | 0.83 | 486.36 | 19 | 50 | 0 | 1 |
| Path 60 | C00022->C00407:[1->2,1->5,3->6,3->9], C00036->C00407:[5->3] | 0.83 | 467.170212766 | 19 | 47 | 0 | 1 |
| Path 61 | C00022->C00407:[1->2,3->6,3->9], C00036->C00407:[2->9,5->3,5->5] | 0.83 | 327.967213115 | 21 | 61 | 0 | 1 |
| Path 62 | C00022->C00407:[1->2,1->5,3->6,3->9] | 0.67 | 515.676470588 | 14 | 34 | 0 | 0 |
| Path 63 | C00022->C00407:[1->2,1->5,3->6,3->9], C00036->C00407:[5->3] | 0.83 | 362.013333333 | 22 | 75 | 0 | 1 |
| Path 64 | C00022->C00407:[1->2,3->6] | 0.33 | 563.533333333 | 9 | 15 | 0 | 0 |
| Path 65 | C00022->C00407:[1->2,3->6] | 0.33 | 531.178571429 | 9 | 28 | 0 | 0 |
| Path 66 | C00022->C00407:[1->1,2->3,2->5,3->9], C00036->C00407:[1->1,2->9,3->3,5->5] | 0.67 | 303.589041096 | 19 | 73 | 0 | 0 |
| Path 67 | C00022->C00407:[1->2,2->3,3->6] | 0.50 | 404.591836735 | 19 | 49 | 0 | 1 |
| Path 68 | C00022->C00407:[1->1,1->2,1->5,2->3,3->6,3->9], C00036->C00407:[5->3] | 1.00 | 371.685393258 | 29 | 89 | 0 | 1 |
| Path 69 | C00022->C00407:[1->2,3->6], C00036->C00407:[5->3] | 0.50 | 334.911764706 | 19 | 68 | 0 | 1 |
| Path 70 | C00022->C00407:[1->1,1->5,2->3,3->9], C00036->C00407:[1->1,3->3] | 0.67 | 325.257142857 | 14 | 35 | 0 | 0 |
| Path 71 | C00022->C00407:[1->2,1->3,1->5,3->6,3->9], C00036->C00407:[5->3] | 0.83 | 448.074074074 | 26 | 54 | 0 | 1 |
| Path 72 | C00022->C00407:[1->2,3->6], C00036->C00407:[5->3] | 0.50 | 456.71875 | 17 | 32 | 0 | 1 |
| Path 73 | C00022->C00407:[1->2,1->5,3->6,3->9] | 0.67 | 475.86440678 | 19 | 59 | 0 | 0 |
| Path 74 | C00022->C00407:[1->2,1->5,2->3,3->6,3->9] | 0.83 | 471.526315789 | 17 | 38 | 0 | 1 |
| Path 75 | C00022->C00407:[1->2,3->6] | 0.33 | 535.814814815 | 15 | 27 | 0 | 0 |
| Path 76 | C00022->C00407:[1->1,1->2,1->5,2->3,3->6,3->9], C00036->C00407:[5->3] | 1.00 | 389.951807229 | 27 | 83 | 0 | 1 |
| Path 77 | C00022->C00407:[1->1,1->2,1->5,2->3,3->6,3->9] | 1.00 | 365.679012346 | 25 | 81 | 0 | 0 |
| Path 78 | C00022->C00407:[1->2,3->6], C00036->C00407:[5->3] | 0.50 | 609.255813953 | 19 | 43 | 0 | 1 |
| Path 79 | C00022->C00407:[1->1,1->2,1->5,2->3,3->6,3->9], C00036->C00407:[5->3] | 1.00 | 482.795454545 | 24 | 44 | 0 | 1 |
| Path 80 | C00022->C00407:[1->2,1->5,3->6,3->9], C00036->C00407:[5->3] | 0.83 | 422.0 | 20 | 38 | 0 | 1 |
| Path 81 | C00022->C00407:[1->2,1->5,3->6,3->9] | 0.67 | 528.710526316 | 15 | 38 | 0 | 0 |
| Path 82 | C00022->C00407:[1->2,3->6], C00036->C00407:[2->9,5->5] | 0.67 | 269.790697674 | 14 | 43 | 0 | 0 |
| Path 83 | C00022->C00407:[1->2,3->6], C00036->C00407:[5->3] | 0.50 | 408.862068966 | 14 | 29 | 0 | 0 |
| Path 84 | C00022->C00407:[1->2,1->5,3->6,3->9] | 0.67 | 463.963636364 | 18 | 55 | 0 | 0 |
| Path 85 | C00022->C00407:[1->2,1->5,3->6,3->9], C00036->C00407:[5->3] | 0.83 | 487.04 | 19 | 50 | 0 | 1 |
| Path 86 | C00022->C00407:[1->5,3->9], C00036->C00407:[5->3] | 0.50 | 267.147058824 | 14 | 34 | 0 | 2 |
| Path 87 | C00022->C00407:[1->2,1->5,3->6,3->9] | 0.67 | 348.596491228 | 20 | 57 | 0 | 0 |
| Path 88 | C00036->C00407:[5->3] | 0.17 | 342.372881356 | 16 | 59 | 0 | 1 |
| Path 89 | C00022->C00407:[1->2,3->6] | 0.33 | 510.2 | 7 | 10 | 0 | 0 |
| Path 90 | C00022->C00407:[1->2,1->5,3->6,3->9], C00036->C00407:[5->3] | 0.83 | 366.375 | 19 | 40 | 0 | 1 |
| Path 91 | C00022->C00407:[1->2,1->5,3->6,3->9] | 0.67 | 388.731707317 | 20 | 82 | 0 | 0 |
| Path 92 | C00022->C00407:[1->2,1->5,3->6,3->9] | 0.67 | 426.685714286 | 17 | 35 | 0 | 0 |
| Path 93 | C00022->C00407:[1->1,1->2,2->3,3->6], C00036->C00407:[5->3] | 0.67 | 376.095890411 | 24 | 73 | 0 | 1 |
| Path 94 | C00022->C00407:[1->2,3->6,3->9], C00036->C00407:[2->9,5->5] | 0.67 | 311.5 | 16 | 48 | 0 | 0 |
| Path 95 | C00022->C00407:[1->2,3->6], C00036->C00407:[5->3] | 0.50 | 373.413333333 | 16 | 75 | 0 | 0 |
| Path 96 | C00022->C00407:[1->1,1->2,2->3,3->6], C00036->C00407:[5->3] | 0.67 | 480.352941176 | 21 | 34 | 0 | 1 |
| Path 97 | C00022->C00407:[1->2,1->5,2->3,3->6,3->9], C00036->C00407:[5->3] | 0.83 | 438.803030303 | 27 | 66 | 0 | 1 |
| Path 98 | C00022->C00407:[1->2,3->6] | 0.33 | 419.162790698 | 19 | 43 | 0 | 0 |
| Path 99 | C00022->C00407:[1->2,1->5,3->6,3->9] | 0.67 | 474.338028169 | 24 | 71 | 0 | 0 |
| Path 100 | C00022->C00407:[1->2,1->5,3->6,3->9], C00036->C00407:[5->3] | 0.83 | 446.787878788 | 18 | 33 | 0 | 1 |
| Path 101 | C00022->C00407:[1->2,1->5,3->6,3->9], C00036->C00407:[5->3] | 0.83 | 380.348484848 | 20 | 66 | 0 | 1 |
| Path 102 | C00022->C00407:[1->2,1->5,3->6,3->9], C00036->C00407:[5->3] | 0.83 | 403.95 | 21 | 80 | 0 | 1 |
| Path 103 | C00022->C00407:[1->5,3->9], C00036->C00407:[3->3] | 0.50 | 521.962962963 | 17 | 27 | 0 | 1 |
| Path 104 | C00022->C00407:[1->2,1->5,3->6,3->9], C00036->C00407:[5->3] | 0.83 | 373.476923077 | 19 | 65 | 0 | 1 |
| Path 105 | C00022->C00407:[1->2,1->5,3->6,3->9] | 0.67 | 515.676470588 | 14 | 34 | 0 | 0 |
| Path 106 | C00022->C00407:[1->2,1->5,2->3,3->6,3->9], C00036->C00407:[5->3] | 0.83 | 461.095238095 | 29 | 84 | 0 | 1 |
| Path 107 | C00022->C00407:[1->2,1->5,3->6,3->9], C00036->C00407:[5->3] | 0.83 | 496.166666667 | 24 | 48 | 0 | 1 |
| Path 108 | C00022->C00407:[1->1,1->2,1->5,2->3,3->6,3->9], C00036->C00407:[5->3] | 1.00 | 358.517241379 | 28 | 87 | 0 | 1 |
| Path 109 | C00022->C00407:[1->2,1->5,3->6,3->9], C00036->C00407:[5->3] | 0.83 | 493.039215686 | 20 | 51 | 0 | 1 |
| Path 110 | C00022->C00407:[1->2,3->6] | 0.33 | 540.206896552 | 10 | 29 | 0 | 0 |
| Path 111 | C00022->C00407:[1->2,1->5,3->6,3->9], C00036->C00407:[5->3] | 0.83 | 341.057971014 | 20 | 69 | 0 | 1 |
| Path 112 | C00022->C00407:[1->2,3->6], C00036->C00407:[2->9,5->3,5->5] | 0.83 | 296.449438202 | 21 | 89 | 0 | 1 |
| Path 113 | C00022->C00407:[1->2,2->3,3->6], C00036->C00407:[5->3] | 0.50 | 444.970149254 | 22 | 67 | 0 | 1 |
| Path 114 | C00036->C00407:[2->6,5->2,5->3] | 0.50 | 376.714285714 | 18 | 35 | 0 | 1 |
| Path 115 | C00022->C00407:[1->1,1->2,1->5,2->3,3->6,3->9] | 1.00 | 572.195121951 | 24 | 41 | 0 | 0 |
| Path 116 | C00022->C00407:[2->3,3->6], C00036->C00407:[2->6,5->2] | 0.50 | 424.755555556 | 19 | 45 | 0 | 1 |
| Path 117 | C00022->C00407:[1->2,1->5,3->6,3->9] | 0.67 | 342.586206897 | 20 | 58 | 0 | 0 |
| Path 118 | C00022->C00407:[1->2,3->6,3->9], C00036->C00407:[2->9,5->3,5->5] | 0.83 | 320.216666667 | 20 | 60 | 0 | 1 |
| Path 119 | C00022->C00407:[1->2,2->3,3->6], C00036->C00407:[5->3] | 0.50 | 426.41509434 | 21 | 53 | 0 | 1 |
| Path 120 | C00022->C00407:[1->2,2->3,3->6] | 0.50 | 459.456140351 | 23 | 57 | 0 | 1 |
| Path 121 | C00022->C00407:[1->2,3->6] | 0.33 | 375.196721311 | 17 | 61 | 0 | 0 |
| Path 122 | C00022->C00407:[1->1,1->2,2->3,3->6] | 0.67 | 520.380952381 | 14 | 21 | 0 | 0 |
| Path 123 | C00022->C00407:[1->1,1->2,2->3,3->6] | 0.67 | 425.571428571 | 15 | 28 | 0 | 0 |
| Path 124 | C00022->C00407:[1->1,1->2,1->5,2->3,3->6,3->9] | 1.00 | 375.060606061 | 21 | 66 | 0 | 0 |
| Path 125 | C00022->C00407:[1->1,1->2,1->5,2->3,3->6,3->9] | 1.00 | 353.202702703 | 23 | 74 | 0 | 0 |
| Path 126 | C00022->C00407:[1->2,3->6] | 0.33 | 547.142857143 | 8 | 14 | 0 | 0 |
| Path 127 | C00036->C00407:[5->3] | 0.17 | 185.95 | 7 | 20 | 0 | 2 |
| Path 128 | C00022->C00407:[1->2,1->5,3->6,3->9], C00036->C00407:[5->3] | 0.83 | 453.403846154 | 24 | 52 | 0 | 1 |
| Path 129 | C00022->C00407:[1->2,2->3,3->6] | 0.50 | 495.660714286 | 19 | 56 | 0 | 1 |
| Path 130 | C00022->C00407:[1->2,2->3,3->6] | 0.50 | 478.549019608 | 17 | 51 | 0 | 1 |
| Path 131 | C00022->C00407:[1->2,3->6] | 0.33 | 538.622222222 | 17 | 45 | 0 | 0 |
| Path 132 | C00022->C00407:[1->2,3->6] | 0.33 | 653.0 | 14 | 24 | 0 | 0 |
| Path 133 | C00022->C00407:[1->2,3->6] | 0.33 | 549.193548387 | 16 | 31 | 0 | 0 |
| Path 134 | C00022->C00407:[1->2,1->3,1->5,3->6,3->9], C00036->C00407:[5->3] | 0.83 | 530.224137931 | 29 | 58 | 0 | 1 |
| Path 135 | C00022->C00407:[1->2,1->5,3->6,3->9], C00036->C00407:[5->3] | 0.83 | 461.204545455 | 23 | 44 | 0 | 1 |
| Path 136 | C00022->C00407:[1->2,1->5,3->6,3->9] | 0.67 | 508.485714286 | 19 | 35 | 0 | 0 |
| Path 137 | C00022->C00407:[1->2,1->5,3->6,3->9], C00036->C00407:[5->3] | 0.83 | 398.594936709 | 20 | 79 | 0 | 1 |
| Path 138 | C00036->C00407:[2->6,2->9,5->2,5->3,5->5] | 0.83 | 296.803571429 | 19 | 56 | 0 | 1 |
| Path 139 | C00022->C00407:[1->2,3->6] | 0.33 | 537.862068966 | 10 | 29 | 0 | 0 |
| Path 140 | C00022->C00407:[1->2,1->5,3->6,3->9] | 0.67 | 362.691176471 | 19 | 68 | 0 | 0 |
| Path 141 | C00022->C00407:[1->2,3->6], C00036->C00407:[5->3] | 0.50 | 374.8625 | 24 | 80 | 0 | 1 |
| Path 142 | C00022->C00407:[1->2,3->6], C00036->C00407:[2->9,5->5] | 0.67 | 347.338709677 | 17 | 62 | 0 | 0 |
| Path 143 | C00022->C00407:[2->3,2->5,3->9], C00036->C00407:[5->3,5->5] | 0.50 | 356.028985507 | 21 | 69 | 0 | 1 |
| Path 144 | C00022->C00407:[1->2,1->5,3->6,3->9] | 0.67 | 521.567567568 | 14 | 37 | 0 | 0 |
| Path 145 | C00022->C00407:[1->2,3->6], C00036->C00407:[5->3] | 0.50 | 352.814285714 | 21 | 70 | 0 | 0 |
| Path 146 | C00022->C00407:[1->1,1->2,1->5,2->3,3->6,3->9], C00036->C00407:[5->3] | 1.00 | 368.872340426 | 30 | 94 | 0 | 1 |
| Path 147 | C00022->C00407:[1->2,3->6], C00036->C00407:[2->9,5->5] | 0.67 | 357.5 | 17 | 62 | 0 | 0 |
| Path 148 | C00022->C00407:[1->1,1->2,1->5,2->3,3->6,3->9], C00036->C00407:[5->3] | 1.00 | 408.716981132 | 26 | 53 | 0 | 1 |
| Path 149 | C00022->C00407:[1->2,1->5,3->6,3->9], C00036->C00407:[5->3] | 0.83 | 480.574468085 | 19 | 47 | 0 | 1 |
| Path 150 | C00022->C00407:[1->2,1->5,3->6,3->9], C00036->C00407:[5->3] | 0.83 | 473.782608696 | 18 | 46 | 0 | 1 |
| Path 151 | C00022->C00407:[1->2,1->5,3->6,3->9] | 0.67 | 378.3125 | 24 | 80 | 0 | 0 |
| Path 152 | C00022->C00407:[1->5,3->6,3->9], C00036->C00407:[2->6,5->2] | 0.67 | 424.0 | 15 | 27 | 0 | 0 |
| Path 153 | C00036->C00407:[2->9,5->3,5->5] | 0.50 | 290.806818182 | 20 | 88 | 0 | 1 |
| Path 154 | C00022->C00407:[1->2,1->5,3->6,3->9] | 0.67 | 446.463414634 | 17 | 41 | 0 | 0 |
| Path 155 | C00036->C00407:[3->3] | 0.17 | 512.846153846 | 16 | 26 | 0 | 1 |
| Path 156 | C00022->C00407:[1->2,2->3,3->6] | 0.50 | 464.675675676 | 16 | 37 | 0 | 1 |
| Path 157 | C00022->C00407:[1->1,1->2,1->5,2->3,3->6,3->9] | 1.00 | 351.025316456 | 24 | 79 | 0 | 0 |
| Path 158 | C00022->C00407:[1->2,1->5,3->6,3->9] | 0.67 | 510.408163265 | 20 | 49 | 0 | 0 |
| Path 159 | C00022->C00407:[1->2,1->5,3->6,3->9], C00036->C00407:[5->3] | 0.83 | 455.65625 | 17 | 32 | 0 | 1 |
| Path 160 | C00022->C00407:[1->2,3->6], C00036->C00407:[5->3] | 0.50 | 328.125 | 20 | 72 | 0 | 1 |
| Path 161 | C00022->C00407:[1->2,3->6], C00036->C00407:[5->3] | 0.50 | 364.830769231 | 19 | 65 | 0 | 1 |
| Path 162 | C00022->C00407:[1->2,1->5,3->6,3->9], C00036->C00407:[3->3] | 0.83 | 536.02173913 | 20 | 46 | 0 | 1 |
| Path 163 | C00022->C00407:[1->2,2->3,3->6,3->9], C00036->C00407:[2->9,5->5] | 0.83 | 342.181818182 | 20 | 66 | 0 | 1 |
| Path 164 | C00022->C00407:[1->2,1->5,3->6,3->9], C00036->C00407:[5->3] | 0.83 | 484.903225806 | 25 | 62 | 0 | 1 |
| Path 165 | C00022->C00407:[1->1,1->2,1->5,2->3,3->6,3->9] | 1.00 | 395.661538462 | 21 | 65 | 0 | 0 |
| Path 166 | C00022->C00407:[1->2,2->3,2->5,3->6,3->9] | 0.83 | 448.852941176 | 24 | 68 | 0 | 1 |
| Path 167 | C00022->C00407:[1->2,1->5,2->3,3->6,3->9], C00036->C00407:[5->3] | 0.83 | 460.138888889 | 24 | 72 | 0 | 1 |
| Path 168 | C00022->C00407:[1->2,1->5,3->6,3->9], C00036->C00407:[5->3] | 0.83 | 488.693877551 | 25 | 49 | 0 | 1 |
| Path 169 | C00022->C00407:[1->2,1->5,2->3,3->6,3->9] | 0.83 | 464.620689655 | 24 | 58 | 0 | 1 |
| Path 170 | C00022->C00407:[1->2,1->5,3->6,3->9], C00036->C00407:[5->3] | 0.83 | 334.02739726 | 21 | 73 | 0 | 1 |
| Path 171 | C00022->C00407:[1->2,1->5,3->6,3->9], C00036->C00407:[5->3] | 0.83 | 351.042253521 | 25 | 71 | 0 | 1 |
| Path 172 | C00022->C00407:[1->2,3->6] | 0.33 | 456.611111111 | 13 | 36 | 0 | 0 |
| Path 173 | C00022->C00407:[1->2,3->6], C00036->C00407:[5->3] | 0.50 | 354.416666667 | 12 | 24 | 0 | 0 |
| Path 174 | C00022->C00407:[1->2,3->6] | 0.33 | 343.016949153 | 14 | 59 | 0 | 0 |
| Path 175 | C00022->C00407:[1->2,3->6], C00036->C00407:[5->3] | 0.50 | 657.08 | 17 | 25 | 0 | 1 |
| Path 176 | C00022->C00407:[1->2,1->5,3->6,3->9] | 0.67 | 392.225806452 | 15 | 31 | 0 | 0 |
| Path 177 | C00036->C00407:[1->1,2->9,3->3,5->5] | 0.67 | 240.20754717 | 15 | 53 | 0 | 0 |
| Path 178 | C00036->C00407:[2->6,2->9,5->2,5->3,5->5] | 0.83 | 296.06741573 | 21 | 89 | 0 | 1 |
| Path 179 | C00022->C00407:[1->2,3->6], C00036->C00407:[5->3] | 0.50 | 445.870967742 | 16 | 31 | 0 | 1 |
| Path 180 | C00022->C00407:[1->2,1->5,3->6,3->9] | 0.67 | 491.580645161 | 18 | 31 | 0 | 0 |
| Path 181 | C00022->C00407:[1->2,3->6] | 0.33 | 473.02 | 14 | 50 | 0 | 0 |
| Path 182 | C00022->C00407:[1->2,1->5,3->6,3->9], C00036->C00407:[5->3] | 0.83 | 403.95 | 21 | 80 | 0 | 1 |
| Path 183 | C00022->C00407:[1->1,1->2,1->5,2->3,3->6,3->9] | 1.00 | 457.094339623 | 23 | 53 | 0 | 0 |
| Path 184 | C00022->C00407:[1->2,3->6], C00036->C00407:[5->3] | 0.50 | 395.142857143 | 13 | 28 | 0 | 0 |
| Path 185 | C00022->C00407:[1->5,2->3,3->9] | 0.50 | 489.636363636 | 18 | 55 | 0 | 1 |
| Path 186 | C00022->C00407:[1->2,3->6], C00036->C00407:[1->1,3->3] | 0.67 | 380.224489796 | 15 | 49 | 0 | 0 |
| Path 187 | C00022->C00407:[1->5,2->3,3->9] | 0.50 | 477.882352941 | 17 | 51 | 0 | 1 |
| Path 188 | C00036->C00407:[1->1,3->3] | 0.33 | 268.9 | 12 | 30 | 0 | 0 |
| Path 189 | C00022->C00407:[1->5,2->3,3->9] | 0.50 | 284.967741935 | 15 | 31 | 0 | 2 |
| Path 190 | C00022->C00407:[1->2,1->5,2->3,3->6,3->9] | 0.83 | 480.641025641 | 18 | 39 | 0 | 1 |
| Path 191 | C00022->C00407:[1->2,3->6] | 0.33 | 599.805555556 | 20 | 36 | 0 | 0 |
| Path 192 | C00022->C00407:[1->2,3->6], C00036->C00407:[5->3] | 0.50 | 460.826086957 | 18 | 46 | 0 | 1 |
| Path 193 | C00022->C00407:[1->1,1->2,1->5,2->3,3->6,3->9], C00036->C00407:[5->3] | 1.00 | 528.037037037 | 29 | 54 | 0 | 1 |
| Path 194 | C00022->C00407:[1->1,1->2,1->5,2->3,3->6,3->9], C00036->C00407:[5->3] | 1.00 | 377.316455696 | 26 | 79 | 0 | 1 |
| Path 195 | C00022->C00407:[1->1,2->3,3->6,3->9], C00036->C00407:[2->6,2->9,5->2,5->5] | 1.00 | 316.163265306 | 17 | 49 | 0 | 0 |
| Path 196 | C00022->C00407:[1->2,1->5,3->6,3->9] | 0.67 | 456.142857143 | 18 | 35 | 0 | 0 |
| Path 197 | C00022->C00407:[1->2,3->6], C00036->C00407:[5->3] | 0.50 | 468.840909091 | 23 | 44 | 0 | 1 |
| Path 198 | C00022->C00407:[1->2,1->5,2->3,3->6,3->9] | 0.83 | 489.228070175 | 20 | 57 | 0 | 1 |
| Path 199 | C00022->C00407:[1->2,3->6], C00036->C00407:[5->3] | 0.50 | 323.863013699 | 19 | 73 | 0 | 0 |
| Path 200 | C00022->C00407:[1->2,3->6] | 0.33 | 435.566666667 | 13 | 30 | 0 | 0 |
| Path 201 | C00022->C00407:[1->2,3->6] | 0.33 | 460.909090909 | 14 | 44 | 0 | 0 |
| Path 202 | C00022->C00407:[1->2,3->6] | 0.33 | 651.071428571 | 15 | 28 | 0 | 0 |
| Path 203 | C00022->C00407:[1->2,3->6] | 0.33 | 389.727272727 | 16 | 77 | 0 | 0 |
| Path 204 | C00022->C00407:[1->2,1->5,2->3,3->6,3->9], C00036->C00407:[5->3] | 0.83 | 432.574074074 | 22 | 54 | 0 | 1 |
| Path 205 | C00022->C00407:[1->1,1->2,2->3,3->6] | 0.67 | 530.743589744 | 16 | 39 | 0 | 0 |
| Path 206 | C00022->C00407:[1->2,2->3,3->6] | 0.50 | 495.660714286 | 19 | 56 | 0 | 1 |
| Path 207 | C00022->C00407:[1->1,1->2,1->5,2->3,3->6,3->9], C00036->C00407:[5->3] | 1.00 | 467.125 | 23 | 40 | 0 | 1 |
| Path 208 | C00022->C00407:[1->2,3->6] | 0.33 | 459.166666667 | 18 | 48 | 0 | 0 |
| Path 209 | C00022->C00407:[1->2,3->6] | 0.33 | 465.8125 | 19 | 48 | 0 | 0 |
| Path 210 | C00022->C00407:[1->2,3->6,3->9], C00036->C00407:[5->3,5->5] | 0.83 | 424.017241379 | 23 | 58 | 0 | 1 |
| Path 211 | C00022->C00407:[1->2,3->6], C00036->C00407:[5->3] | 0.50 | 349.883333333 | 17 | 60 | 0 | 1 |
| Path 212 | C00022->C00407:[1->1,2->3,3->9], C00036->C00407:[2->9,5->5] | 0.67 | 306.9375 | 16 | 48 | 0 | 0 |
| Path 213 | C00022->C00407:[1->2,1->5,3->6,3->9], C00036->C00407:[5->3] | 0.83 | 480.574468085 | 19 | 47 | 0 | 1 |
| Path 214 | C00022->C00407:[1->2,3->6] | 0.33 | 393.536585366 | 18 | 41 | 0 | 0 |
| Path 215 | C00022->C00407:[1->2,3->6,3->9], C00036->C00407:[2->9,5->5] | 0.67 | 298.375 | 16 | 48 | 0 | 0 |
| Path 216 | C00022->C00407:[1->1,1->2,2->3,3->6] | 0.67 | 530.743589744 | 16 | 39 | 0 | 0 |
| Path 217 | C00022->C00407:[1->2,3->6] | 0.33 | 563.533333333 | 9 | 15 | 0 | 0 |
| Path 218 | C00022->C00407:[1->1,1->2,1->5,2->3,3->6,3->9] | 1.00 | 437.980392157 | 22 | 51 | 0 | 0 |
| Path 219 | C00022->C00407:[1->2,3->6] | 0.33 | 485.351851852 | 15 | 54 | 0 | 0 |
| Path 220 | C00022->C00407:[1->2,1->5,3->6,3->9], C00036->C00407:[5->3] | 0.83 | 402.428571429 | 25 | 77 | 0 | 1 |
| Path 221 | C00022->C00407:[1->2,1->5,3->6,3->9], C00036->C00407:[5->3] | 0.83 | 366.87654321 | 24 | 81 | 0 | 1 |
| Path 222 | C00022->C00407:[1->2,2->3,3->6] | 0.50 | 473.315789474 | 17 | 38 | 0 | 1 |
| Path 223 | C00022->C00407:[1->2,3->6] | 0.33 | 404.192307692 | 12 | 26 | 0 | 0 |
| Path 224 | C00022->C00407:[1->2,1->5,3->6,3->9], C00036->C00407:[5->3] | 0.83 | 537.928571429 | 23 | 42 | 0 | 1 |
| Path 225 | C00022->C00407:[1->2,1->5,3->6,3->9], C00036->C00407:[5->3] | 0.83 | 658.133333333 | 19 | 30 | 0 | 1 |
| Path 226 | C00022->C00407:[1->2,3->6], C00036->C00407:[5->3] | 0.50 | 367.984375 | 18 | 64 | 0 | 1 |
| Path 227 | C00036->C00407:[2->9,3->3,5->5] | 0.50 | 340.454545455 | 20 | 55 | 0 | 1 |
| Path 228 | C00022->C00407:[1->2,1->5,3->6,3->9] | 0.67 | 507.272727273 | 13 | 33 | 0 | 0 |
| Path 229 | C00022->C00407:[1->2,3->6] | 0.33 | 796.523809524 | 16 | 21 | 0 | 0 |
| Path 230 | C00022->C00407:[1->2,1->5,3->6,3->9], C00036->C00407:[5->3] | 0.83 | 356.057142857 | 25 | 70 | 0 | 1 |
| Path 231 | C00022->C00407:[1->2,1->5,3->6,3->9], C00036->C00407:[5->3] | 0.83 | 465.878787879 | 18 | 33 | 0 | 1 |
| Path 232 | C00022->C00407:[1->5,3->9], C00036->C00407:[5->3] | 0.50 | 367.453125 | 18 | 64 | 0 | 1 |
| Path 233 | C00022->C00407:[1->2,3->6] | 0.33 | 521.533333333 | 9 | 15 | 0 | 0 |
| Path 234 | C00022->C00407:[1->1,1->2,2->3,3->6] | 0.67 | 514.58974359 | 16 | 39 | 0 | 0 |
| Path 235 | C00022->C00407:[1->1,1->2,1->5,2->3,3->6,3->9] | 1.00 | 509.473684211 | 19 | 38 | 0 | 0 |
| Path 236 | C00022->C00407:[1->2,1->5,3->6,3->9], C00036->C00407:[5->3] | 0.83 | 494.587301587 | 26 | 63 | 0 | 1 |
| Path 237 | C00022->C00407:[1->2,1->5,3->6,3->9] | 0.67 | 512.131578947 | 15 | 38 | 0 | 0 |
| Path 238 | C00022->C00407:[1->2,2->3,3->6], C00036->C00407:[2->9,5->5] | 0.83 | 372.4625 | 21 | 80 | 0 | 1 |
| Path 239 | C00022->C00407:[1->1,1->2,1->5,2->3,3->6,3->9] | 1.00 | 705.066666667 | 21 | 30 | 0 | 0 |
| Path 240 | C00022->C00407:[1->2,3->6,3->9], C00036->C00407:[5->3,5->5] | 0.83 | 371.55 | 23 | 60 | 0 | 1 |
| Path 241 | C00022->C00407:[1->2,3->6] | 0.33 | 518.482758621 | 10 | 29 | 0 | 0 |
| Path 242 | C00022->C00407:[1->2,1->5,2->3,3->6,3->9], C00036->C00407:[3->3] | 0.83 | 545.125 | 19 | 32 | 0 | 1 |
| Path 243 | C00022->C00407:[1->1,1->5,2->3,3->9] | 0.67 | 505.05 | 13 | 20 | 0 | 0 |
| Path 244 | C00022->C00407:[1->2,1->5,3->6,3->9] | 0.67 | 345.390625 | 18 | 64 | 0 | 0 |
| Path 245 | C00022->C00407:[1->2,1->5,3->6,3->9], C00036->C00407:[5->3] | 0.83 | 546.760869565 | 24 | 46 | 0 | 1 |
| Path 246 | C00022->C00407:[1->2,2->3,2->5,3->6,3->9], C00036->C00407:[5->3,5->5] | 0.83 | 387.761904762 | 23 | 84 | 0 | 1 |
| Path 247 | C00022->C00407:[1->2,1->5,2->3,3->6,3->9] | 0.83 | 500.280701754 | 20 | 57 | 0 | 1 |
| Path 248 | C00022->C00407:[1->1,1->2,1->5,2->3,3->6,3->9] | 1.00 | 715.153846154 | 20 | 26 | 0 | 0 |
| Path 249 | C00022->C00407:[2->3] | 0.17 | 455.555555556 | 15 | 36 | 0 | 1 |
| Path 250 | C00022->C00407:[1->2,1->5,3->6,3->9] | 0.67 | 474.948717949 | 19 | 39 | 0 | 0 |
| Path 251 | C00022->C00407:[1->1,1->2,2->3,3->6] | 0.67 | 398.96875 | 16 | 32 | 0 | 0 |
| Path 252 | C00022->C00407:[1->5,3->6,3->9], C00036->C00407:[2->6,5->2,5->3] | 0.83 | 412.55 | 20 | 40 | 0 | 1 |
| Path 253 | C00022->C00407:[1->2,1->5,3->6,3->9] | 0.67 | 520.648648649 | 14 | 37 | 0 | 0 |
| Path 254 | C00022->C00407:[1->2,1->5,3->6,3->9] | 0.67 | 609.0 | 19 | 33 | 0 | 0 |
| Path 255 | C00022->C00407:[1->2,3->6], C00036->C00407:[2->9,5->5] | 0.67 | 350.360655738 | 16 | 61 | 0 | 0 |
| Path 256 | C00022->C00407:[1->2,1->5,3->6,3->9] | 0.67 | 501.421052632 | 12 | 19 | 0 | 0 |
| Path 257 | C00022->C00407:[1->2,1->5,3->6,3->9] | 0.67 | 356.403225806 | 17 | 62 | 0 | 0 |
| Path 258 | C00022->C00407:[1->2,3->6], C00036->C00407:[3->3] | 0.50 | 531.066666667 | 19 | 45 | 0 | 1 |
| Path 259 | C00022->C00407:[2->3] | 0.17 | 272.692307692 | 7 | 26 | 0 | 2 |
| Path 260 | C00022->C00407:[1->1,1->2,2->3,3->6] | 0.67 | 523.842105263 | 15 | 38 | 0 | 0 |
| Path 261 | C00022->C00407:[1->5,2->3,3->6,3->9], C00036->C00407:[2->6,5->2] | 0.83 | 432.02173913 | 20 | 46 | 0 | 1 |
| Path 262 | C00022->C00407:[1->2,1->5,3->6,3->9] | 0.67 | 516.0 | 13 | 20 | 0 | 0 |
| Path 263 | C00022->C00407:[1->2,3->6], C00036->C00407:[5->3] | 0.50 | 374.523076923 | 19 | 65 | 0 | 1 |
| Path 264 | C00022->C00407:[1->2,3->6], C00036->C00407:[5->3] | 0.50 | 340.868852459 | 15 | 61 | 0 | 0 |
| Path 265 | C00022->C00407:[1->2,1->5,2->3,3->6,3->9] | 0.83 | 483.942307692 | 18 | 52 | 0 | 1 |
| Path 266 | C00022->C00407:[1->2,2->3,3->6] | 0.50 | 456.736842105 | 17 | 38 | 0 | 1 |
| Path 267 | C00022->C00407:[1->2,3->6] | 0.33 | 445.0 | 18 | 46 | 0 | 0 |
| Path 268 | C00022->C00407:[1->2,2->3,3->6] | 0.50 | 484.410714286 | 19 | 56 | 0 | 1 |
| Path 269 | C00022->C00407:[1->5,3->9], C00036->C00407:[2->6,5->2,5->3] | 0.83 | 343.086956522 | 21 | 69 | 0 | 1 |
| Path 270 | C00022->C00407:[1->5,2->3,3->9] | 0.50 | 463.756756757 | 16 | 37 | 0 | 1 |
| Path 271 | C00036->C00407:[5->3] | 0.17 | 651.416666667 | 16 | 24 | 0 | 1 |
| Path 272 | C00036->C00407:[5->3] | 0.17 | 335.347826087 | 11 | 23 | 0 | 0 |
| Path 273 | C00022->C00407:[2->3] | 0.17 | 204.117647059 | 8 | 17 | 0 | 2 |
| Path 274 | C00022->C00407:[1->2,3->6], C00036->C00407:[5->3] | 0.50 | 368.205882353 | 16 | 34 | 0 | 0 |
| Path 275 | C00022->C00407:[1->1,1->2,1->5,2->3,3->6,3->9], C00036->C00407:[5->3] | 1.00 | 609.441860465 | 26 | 43 | 0 | 1 |
| Path 276 | C00022->C00407:[1->2,3->6], C00036->C00407:[5->3] | 0.50 | 474.52173913 | 18 | 46 | 0 | 1 |
| Path 277 | C00022->C00407:[1->1,1->2,1->5,2->3,3->6,3->9] | 1.00 | 400.195121951 | 21 | 41 | 0 | 0 |
| Path 278 | C00022->C00407:[1->2,3->6], C00036->C00407:[5->3] | 0.50 | 399.455696203 | 20 | 79 | 0 | 1 |
| Path 279 | C00022->C00407:[1->2,1->5,3->6,3->9] | 0.67 | 522.709677419 | 18 | 31 | 0 | 0 |
| Path 280 | C00022->C00407:[1->2,3->6], C00036->C00407:[5->3] | 0.50 | 399.455696203 | 20 | 79 | 0 | 1 |
| Path 281 | C00022->C00407:[1->2,3->6], C00036->C00407:[5->3] | 0.50 | 391.481012658 | 20 | 79 | 0 | 1 |
| Path 282 | C00022->C00407:[1->2,2->3,2->5,3->6,3->9] | 0.83 | 374.453125 | 21 | 64 | 0 | 1 |
| Path 283 | C00022->C00407:[1->2,2->3,2->5,3->6,3->9] | 0.83 | 340.660194175 | 24 | 103 | 0 | 1 |
| Path 284 | C00022->C00407:[1->2,2->3,3->6] | 0.50 | 421.644444444 | 18 | 45 | 0 | 1 |
| Path 285 | C00022->C00407:[1->1,1->2,1->5,2->3,3->6,3->9], C00036->C00407:[5->3] | 1.00 | 381.27027027 | 25 | 74 | 0 | 1 |
| Path 286 | C00022->C00407:[1->2,3->6] | 0.33 | 531.0 | 15 | 30 | 0 | 0 |
| Path 287 | C00022->C00407:[1->2,1->5,3->6,3->9], C00036->C00407:[5->3] | 0.83 | 612.659090909 | 20 | 44 | 0 | 1 |
| Path 288 | C00022->C00407:[2->3,2->5,3->9] | 0.50 | 397.52173913 | 23 | 69 | 0 | 1 |
| Path 289 | C00022->C00407:[1->5,3->9], C00036->C00407:[2->6,5->2,5->3] | 0.83 | 387.333333333 | 19 | 36 | 0 | 1 |
| Path 290 | C00022->C00407:[1->2,3->6] | 0.33 | 380.232142857 | 16 | 56 | 0 | 0 |
| Path 291 | C00022->C00407:[1->2,3->6,3->9], C00036->C00407:[5->3,5->5] | 0.83 | 334.0 | 20 | 54 | 0 | 1 |
| Path 292 | C00022->C00407:[1->1,1->2,1->5,2->3,3->6,3->9], C00036->C00407:[5->3] | 1.00 | 418.083333333 | 25 | 48 | 0 | 1 |
| Path 293 | C00022->C00407:[1->1,1->2,1->5,2->3,3->6,3->9], C00036->C00407:[5->3] | 1.00 | 512.019230769 | 28 | 52 | 0 | 1 |
| Path 294 | C00022->C00407:[1->2,3->6] | 0.33 | 525.977272727 | 16 | 44 | 0 | 0 |
| Path 295 | C00022->C00407:[1->2,1->5,3->6,3->9] | 0.67 | 510.4375 | 19 | 32 | 0 | 0 |
| Path 296 | C00022->C00407:[1->2,1->5,3->6,3->9] | 0.67 | 524.777777778 | 20 | 36 | 0 | 0 |
| Path 297 | C00022->C00407:[1->2,1->5,3->6,3->9], C00036->C00407:[5->3] | 0.83 | 493.039215686 | 20 | 51 | 0 | 1 |
| Path 298 | C00022->C00407:[1->1,1->2,1->5,2->3,3->6,3->9] | 1.00 | 455.303030303 | 19 | 33 | 0 | 0 |
| Path 299 | C00022->C00407:[1->2,1->5,3->6,3->9] | 0.67 | 581.063829787 | 20 | 47 | 0 | 0 |
| Path 300 | C00022->C00407:[1->2,3->6], C00036->C00407:[2->9,3->3,5->5] | 0.83 | 348.535714286 | 21 | 56 | 0 | 1 |
| Path 301 | C00022->C00407:[2->3,3->6,3->9], C00036->C00407:[2->6,2->9,5->2,5->5] | 0.83 | 341.666666667 | 20 | 66 | 0 | 1 |
| Path 302 | C00036->C00407:[2->6,5->2] | 0.33 | 268.179487179 | 11 | 39 | 0 | 0 |
| Path 303 | C00036->C00407:[2->6,5->2,5->3] | 0.50 | 336.970588235 | 20 | 68 | 0 | 1 |
| Path 304 | C00022->C00407:[1->2,3->6] | 0.33 | 432.368421053 | 18 | 38 | 0 | 0 |
| Path 305 | C00022->C00407:[1->2,3->6,3->9], C00036->C00407:[2->9,5->5] | 0.67 | 281.222222222 | 15 | 45 | 0 | 0 |
| Path 306 | C00022->C00407:[1->2,1->5,3->6,3->9], C00036->C00407:[5->3] | 0.83 | 288.426229508 | 20 | 61 | 0 | 1 |
| Path 307 | C00022->C00407:[1->1,1->2,1->5,2->3,3->6,3->9], C00036->C00407:[5->3] | 1.00 | 503.820512821 | 23 | 39 | 0 | 1 |
| Path 308 | C00022->C00407:[1->2,3->6], C00036->C00407:[2->9,5->5] | 0.67 | 357.5 | 17 | 62 | 0 | 0 |
| Path 309 | C00022->C00407:[1->2,3->6] | 0.33 | 378.64 | 20 | 75 | 0 | 0 |
| Path 310 | C00022->C00407:[1->2,1->5,3->6,3->9] | 0.67 | 497.147058824 | 14 | 34 | 0 | 0 |
| Path 311 | C00022->C00407:[1->5,3->9], C00036->C00407:[5->3] | 0.50 | 444.774193548 | 16 | 31 | 0 | 1 |
| Path 312 | C00022->C00407:[1->2,1->5,3->6,3->9], C00036->C00407:[5->3] | 0.83 | 356.590163934 | 18 | 61 | 0 | 1 |
| Path 313 | C00022->C00407:[1->2,3->6] | 0.33 | 368.436619718 | 20 | 71 | 0 | 0 |
| Path 314 | C00022->C00407:[1->2,1->5,2->3,3->6,3->9] | 0.83 | 411.68 | 20 | 50 | 0 | 1 |
| Path 315 | C00022->C00407:[1->2,3->6], C00036->C00407:[5->3] | 0.50 | 372.714285714 | 17 | 35 | 0 | 1 |
| Path 316 | C00022->C00407:[1->2,1->5,2->3,3->6,3->9] | 0.83 | 428.97826087 | 19 | 46 | 0 | 1 |
| Path 317 | C00022->C00407:[1->2,1->5,3->6,3->9], C00036->C00407:[5->3] | 0.83 | 661.0 | 18 | 26 | 0 | 1 |
| Path 318 | C00022->C00407:[1->2,1->5,3->6,3->9], C00036->C00407:[5->3] | 0.83 | 391.204545455 | 20 | 44 | 0 | 1 |
| Path 319 | C00022->C00407:[2->3,2->5,3->9], C00036->C00407:[5->3,5->5] | 0.50 | 382.879518072 | 22 | 83 | 0 | 1 |
| Path 320 | C00022->C00407:[1->2,1->5,3->6,3->9], C00036->C00407:[5->3] | 0.83 | 396.075 | 21 | 80 | 0 | 1 |
| Path 321 | C00022->C00407:[1->1,1->2,1->5,2->3,3->6,3->9], C00036->C00407:[5->3] | 1.00 | 356.358695652 | 29 | 92 | 0 | 1 |
| Path 322 | C00022->C00407:[1->2,3->6,3->9], C00036->C00407:[2->9,5->5] | 0.67 | 311.5 | 16 | 48 | 0 | 0 |
| Path 323 | C00022->C00407:[1->2,1->5,3->6,3->9], C00036->C00407:[5->3] | 0.83 | 380.348484848 | 20 | 66 | 0 | 1 |
| Path 324 | C00022->C00407:[2->3], C00036->C00407:[3->3] | 0.17 | 380.833333333 | 10 | 24 | 0 | 2 |
| Path 325 | C00022->C00407:[1->2,1->5,3->6,3->9] | 0.67 | 571.088888889 | 24 | 45 | 0 | 0 |
| Path 326 | C00022->C00407:[1->2,1->5,3->6,3->9] | 0.67 | 536.057142857 | 19 | 35 | 0 | 0 |
| Path 327 | C00022->C00407:[1->2,3->6], C00036->C00407:[5->3] | 0.50 | 348.161290323 | 16 | 62 | 0 | 0 |
| Path 328 | C00022->C00407:[1->2,1->5,3->6,3->9], C00036->C00407:[5->3] | 0.83 | 304.912280702 | 20 | 57 | 0 | 1 |
| Path 329 | C00022->C00407:[1->1,1->2,2->3,3->6], C00036->C00407:[5->3] | 0.67 | 459.641025641 | 22 | 39 | 0 | 1 |
| Path 330 | C00022->C00407:[1->2,3->6], C00036->C00407:[3->3] | 0.50 | 523.222222222 | 17 | 27 | 0 | 1 |
| Path 331 | C00022->C00407:[1->2,1->5,2->3,3->6,3->9] | 0.83 | 347.895522388 | 21 | 67 | 0 | 1 |
| Path 332 | C00022->C00407:[1->1,1->2,1->5,2->3,3->6,3->9] | 1.00 | 425.511627907 | 22 | 43 | 0 | 0 |
| Path 333 | C00022->C00407:[1->2,1->5,3->6,3->9], C00036->C00407:[5->3] | 0.83 | 473.043478261 | 18 | 46 | 0 | 1 |
| Path 334 | C00022->C00407:[1->2,3->6], C00036->C00407:[5->3] | 0.50 | 474.52173913 | 18 | 46 | 0 | 1 |
| Path 335 | C00022->C00407:[1->2,1->5,3->6,3->9], C00036->C00407:[5->3] | 0.83 | 437.895833333 | 23 | 48 | 0 | 1 |
| Path 336 | C00022->C00407:[1->1,2->3,3->9], C00036->C00407:[1->1,2->9,3->3,5->5] | 0.67 | 257.666666667 | 18 | 63 | 0 | 0 |
| Path 337 | C00022->C00407:[1->2,2->3,3->6], C00036->C00407:[3->3] | 0.50 | 538.225806452 | 18 | 31 | 0 | 1 |
| Path 338 | C00022->C00407:[2->3,2->5,3->9] | 0.50 | 367.80952381 | 20 | 63 | 0 | 1 |
| Path 339 | C00022->C00407:[1->2,3->6] | 0.33 | 469.933333333 | 14 | 30 | 0 | 0 |
| Path 340 | C00022->C00407:[2->3] | 0.17 | 472.26 | 16 | 50 | 0 | 1 |
| Path 341 | C00022->C00407:[1->2,3->6], C00036->C00407:[5->3] | 0.50 | 467.444444444 | 17 | 45 | 0 | 1 |
| Path 342 | C00022->C00407:[1->1,1->2,2->3,3->6] | 0.67 | 506.75 | 13 | 20 | 0 | 0 |
| Path 343 | C00022->C00407:[1->2,1->5,3->6,3->9] | 0.67 | 355.592592593 | 14 | 27 | 0 | 0 |
| Path 344 | C00036->C00407:[2->6,5->2] | 0.33 | 390.166666667 | 10 | 18 | 0 | 0 |
| Path 345 | C00022->C00407:[1->2,3->6] | 0.33 | 502.782608696 | 14 | 23 | 0 | 0 |
| Path 346 | C00022->C00407:[1->2,1->5,3->6,3->9], C00036->C00407:[5->3] | 0.83 | 483.136363636 | 23 | 44 | 0 | 1 |
| Path 347 | C00022->C00407:[1->2,3->6], C00036->C00407:[5->3] | 0.50 | 319.912280702 | 14 | 57 | 0 | 0 |
| Path 348 | C00022->C00407:[1->2,2->3,3->6] | 0.50 | 498.641509434 | 23 | 53 | 0 | 1 |
| Path 349 | C00022->C00407:[1->2,1->5,3->6,3->9] | 0.67 | 380.434782609 | 13 | 23 | 0 | 0 |
| Path 350 | C00022->C00407:[1->5,3->9], C00036->C00407:[5->3] | 0.50 | 349.316666667 | 17 | 60 | 0 | 1 |
| Path 351 | C00036->C00407:[2->6,2->9,5->2,5->5] | 0.67 | 268.840909091 | 15 | 44 | 0 | 0 |
| Path 352 | C00022->C00407:[1->2,3->6] | 0.33 | 514.307692308 | 14 | 26 | 0 | 0 |
| Path 353 | C00022->C00407:[2->3,3->9], C00036->C00407:[2->9,5->5] | 0.50 | 335.246153846 | 19 | 65 | 0 | 1 |
| Path 354 | C00022->C00407:[1->2,2->3,3->6] | 0.50 | 490.254545455 | 18 | 55 | 0 | 1 |
| Path 355 | C00022->C00407:[1->2,3->6], C00036->C00407:[2->9,5->3,5->5] | 0.83 | 354.52 | 22 | 75 | 0 | 1 |
| Path 356 | C00022->C00407:[1->2,1->5,3->6,3->9], C00036->C00407:[5->3] | 0.83 | 388.736842105 | 25 | 95 | 0 | 1 |
| Path 357 | C00022->C00407:[1->2,1->5,3->6,3->9], C00036->C00407:[5->3] | 0.83 | 429.392857143 | 16 | 28 | 0 | 1 |
| Path 358 | C00022->C00407:[1->2,1->5,3->6,3->9], C00036->C00407:[3->3] | 0.83 | 531.642857143 | 18 | 28 | 0 | 1 |
| Path 359 | C00022->C00407:[1->1,1->2,1->5,2->3,3->6,3->9], C00036->C00407:[5->3] | 1.00 | 428.2 | 27 | 55 | 0 | 1 |
| Path 360 | C00022->C00407:[1->1,2->3,3->6], C00036->C00407:[2->6,5->2] | 0.67 | 430.571428571 | 16 | 28 | 0 | 0 |
| Path 361 | C00022->C00407:[1->2,1->5,3->6,3->9] | 0.67 | 399.225806452 | 16 | 31 | 0 | 0 |
| Path 362 | C00022->C00407:[1->2,3->6,3->9], C00036->C00407:[2->9,5->5] | 0.67 | 301.255319149 | 15 | 47 | 0 | 0 |
| Path 363 | C00022->C00407:[1->2,1->5,3->6,3->9] | 0.67 | 310.083333333 | 16 | 48 | 0 | 0 |
| Path 364 | C00022->C00407:[1->2,3->6], C00036->C00407:[5->3] | 0.50 | 437.03125 | 17 | 32 | 0 | 1 |
| Path 365 | C00022->C00407:[1->1,1->2,1->5,2->3,3->6,3->9], C00036->C00407:[5->3] | 1.00 | 427.984375 | 27 | 64 | 0 | 1 |
| Path 366 | C00022->C00407:[1->1,1->2,2->3,3->6] | 0.67 | 538.055555556 | 20 | 36 | 0 | 0 |
| Path 367 | C00022->C00407:[1->2,3->6,3->9], C00036->C00407:[5->3,5->5] | 0.83 | 313.537634409 | 23 | 93 | 0 | 1 |
| Path 368 | C00022->C00407:[1->2,3->6], C00036->C00407:[2->9,5->3,5->5] | 0.83 | 357.108108108 | 21 | 74 | 0 | 1 |
| Path 369 | C00022->C00407:[1->2,1->5,3->6,3->9], C00036->C00407:[5->3] | 0.83 | 475.288888889 | 24 | 45 | 0 | 1 |
| Path 370 | C00022->C00407:[1->5,3->9], C00036->C00407:[5->3] | 0.50 | 655.72 | 17 | 25 | 0 | 1 |
| Path 371 | C00022->C00407:[1->2,1->5,3->6,3->9], C00036->C00407:[5->3] | 0.83 | 352.714285714 | 23 | 77 | 0 | 1 |
| Path 372 | C00022->C00407:[1->5,3->9], C00036->C00407:[2->6,5->2] | 0.67 | 386.52173913 | 14 | 23 | 0 | 0 |
| Path 373 | C00022->C00407:[1->2,1->5,3->6,3->9], C00036->C00407:[5->3] | 0.83 | 442.714285714 | 21 | 42 | 0 | 1 |
| Path 374 | C00022->C00407:[1->1,1->2,1->5,2->3,3->6,3->9], C00036->C00407:[5->3] | 1.00 | 439.14 | 26 | 50 | 0 | 1 |
| Path 375 | C00022->C00407:[1->5,3->9], C00036->C00407:[5->3] | 0.50 | 415.925925926 | 15 | 27 | 0 | 1 |
| Path 376 | C00036->C00407:[2->6,2->9,5->2,5->5] | 0.67 | 269.0 | 14 | 43 | 0 | 0 |
| Path 377 | C00022->C00407:[3->9], C00036->C00407:[5->3,5->5] | 0.50 | 325.339622642 | 19 | 53 | 0 | 1 |
| Path 378 | C00022->C00407:[1->1,1->2,1->5,2->3,3->6,3->9], C00036->C00407:[5->3] | 1.00 | 488.314285714 | 22 | 35 | 0 | 1 |
| Path 379 | C00022->C00407:[1->2,1->5,3->6,3->9] | 0.67 | 451.979591837 | 18 | 49 | 0 | 0 |
| Path 380 | C00022->C00407:[1->1,1->2,2->3,3->6], C00036->C00407:[5->3] | 0.67 | 372.423076923 | 25 | 78 | 0 | 1 |
| Path 381 | C00022->C00407:[1->2,1->5,3->6,3->9], C00036->C00407:[5->3] | 0.83 | 301.211111111 | 22 | 90 | 0 | 1 |
| Path 382 | C00022->C00407:[1->2,1->5,2->3,3->6,3->9] | 0.83 | 464.487179487 | 18 | 39 | 0 | 1 |
| Path 383 | C00022->C00407:[1->1,1->2,1->5,2->3,3->6,3->9] | 1.00 | 414.395833333 | 23 | 48 | 0 | 0 |
| Path 384 | C00022->C00407:[1->2,3->6] | 0.33 | 351.545454545 | 10 | 22 | 0 | 0 |
| Path 385 | C00022->C00407:[1->2,1->5,3->6,3->9], C00036->C00407:[5->3] | 0.83 | 374.0 | 19 | 65 | 0 | 1 |
| Path 386 | C00022->C00407:[1->2,1->5,3->6,3->9] | 0.67 | 261.25 | 15 | 48 | 0 | 0 |
| Path 387 | C00022->C00407:[1->2,1->5,3->6,3->9], C00036->C00407:[5->3] | 0.83 | 409.05 | 19 | 40 | 0 | 1 |
| Path 388 | C00022->C00407:[1->2,1->5,3->6,3->9], C00036->C00407:[5->3] | 0.83 | 460.839285714 | 22 | 56 | 0 | 1 |
| Path 389 | C00022->C00407:[1->2,1->5,3->6,3->9] | 0.67 | 484.5 | 13 | 20 | 0 | 0 |
| Path 390 | C00022->C00407:[1->2,3->6], C00036->C00407:[5->3] | 0.50 | 394.41025641 | 19 | 78 | 0 | 1 |
| Path 391 | C00022->C00407:[1->2,3->6], C00036->C00407:[5->3] | 0.50 | 437.870967742 | 18 | 31 | 0 | 0 |
| Path 392 | C00022->C00407:[1->2,1->5,2->3,3->6,3->9] | 0.83 | 472.421052632 | 17 | 38 | 0 | 1 |
| Path 393 | C00022->C00407:[1->1,1->2,1->5,2->3,3->6,3->9] | 1.00 | 391.347826087 | 22 | 46 | 0 | 0 |
| Path 394 | C00022->C00407:[1->2,3->6], C00036->C00407:[5->3] | 0.50 | 456.71875 | 17 | 32 | 0 | 1 |
| Path 395 | C00022->C00407:[1->2,3->6], C00036->C00407:[5->3] | 0.50 | 397.736842105 | 24 | 76 | 0 | 1 |
| Path 396 | C00022->C00407:[1->2,2->3,3->6] | 0.50 | 356.277108434 | 20 | 83 | 0 | 0 |
| Path 397 | C00022->C00407:[3->9], C00036->C00407:[5->3,5->5] | 0.50 | 417.543859649 | 22 | 57 | 0 | 1 |
| Path 398 | C00022->C00407:[1->2,1->5,2->3,3->6,3->9], C00036->C00407:[5->3] | 0.83 | 449.588235294 | 23 | 68 | 0 | 1 |
| Path 399 | C00022->C00407:[3->9], C00036->C00407:[5->3,5->5] | 0.50 | 308.326086957 | 22 | 92 | 0 | 1 |
| Path 400 | C00022->C00407:[1->2,3->6], C00036->C00407:[5->3] | 0.50 | 356.307692308 | 18 | 39 | 0 | 1 |
| Path 401 | C00022->C00407:[1->2,1->3,1->5,3->6,3->9], C00036->C00407:[5->3] | 0.83 | 379.774193548 | 29 | 93 | 0 | 1 |
| Path 402 | C00022->C00407:[1->2,3->6], C00036->C00407:[5->3] | 0.50 | 476.720930233 | 22 | 43 | 0 | 1 |
| Path 403 | C00036->C00407:[2->9,5->3,5->5] | 0.50 | 288.4 | 18 | 55 | 0 | 1 |
| Path 404 | C00036->C00407:[5->3] | 0.17 | 311.464285714 | 13 | 56 | 0 | 0 |
| Path 405 | C00022->C00407:[1->2,1->5,3->6,3->9] | 0.67 | 522.1 | 21 | 50 | 0 | 0 |
| Path 406 | C00022->C00407:[1->1,2->3,2->5,3->9] | 0.67 | 359.982758621 | 18 | 58 | 0 | 0 |
| Path 407 | C00022->C00407:[1->2,1->5,3->6,3->9] | 0.67 | 528.710526316 | 15 | 38 | 0 | 0 |
| Path 408 | C00022->C00407:[1->2,3->6], C00036->C00407:[5->3] | 0.50 | 412.891891892 | 19 | 37 | 0 | 1 |
| Path 409 | C00022->C00407:[1->2,3->6] | 0.33 | 361.841269841 | 15 | 63 | 0 | 0 |
| Path 410 | C00022->C00407:[1->2,1->5,3->6,3->9], C00036->C00407:[5->3] | 0.83 | 399.025316456 | 20 | 79 | 0 | 1 |
| Path 411 | C00022->C00407:[1->1,1->2,1->5,2->3,3->6,3->9] | 1.00 | 475.216216216 | 20 | 37 | 0 | 0 |
| Path 412 | C00022->C00407:[1->2,1->5,3->6,3->9] | 0.67 | 418.814814815 | 14 | 27 | 0 | 0 |
| Path 413 | C00022->C00407:[1->2,3->6] | 0.33 | 469.928571429 | 15 | 28 | 0 | 0 |
| Path 414 | C00022->C00407:[1->1,1->2,1->5,2->3,3->6,3->9], C00036->C00407:[5->3] | 1.00 | 394.512820513 | 26 | 78 | 0 | 1 |
| Path 415 | C00022->C00407:[1->2,3->6], C00036->C00407:[5->3] | 0.50 | 374.523076923 | 19 | 65 | 0 | 1 |
| Path 416 | C00022->C00407:[1->2,3->6], C00036->C00407:[5->3] | 0.50 | 309.388888889 | 15 | 36 | 0 | 0 |
| Path 417 | C00022->C00407:[1->2,3->6] | 0.33 | 382.388888889 | 9 | 18 | 0 | 0 |
| Path 418 | C00022->C00407:[1->2,3->6], C00036->C00407:[1->1,3->3] | 0.67 | 285.806451613 | 13 | 31 | 0 | 0 |
| Path 419 | C00022->C00407:[1->1,1->2,1->5,2->3,3->6,3->9] | 1.00 | 390.171428571 | 22 | 70 | 0 | 0 |
| Path 420 | C00022->C00407:[1->2,1->5,3->6,3->9], C00036->C00407:[5->3] | 0.83 | 379.604938272 | 25 | 81 | 0 | 1 |
| Path 421 | C00022->C00407:[1->2,1->5,3->6,3->9] | 0.67 | 506.242424242 | 13 | 33 | 0 | 0 |
| Path 422 | C00022->C00407:[1->2,1->5,2->3,3->6,3->9] | 0.83 | 495.053571429 | 19 | 56 | 0 | 1 |
| Path 423 | C00022->C00407:[1->2,3->6] | 0.33 | 559.0 | 9 | 15 | 0 | 0 |
| Path 424 | C00022->C00407:[2->3,2->5,3->9] | 0.50 | 336.225490196 | 23 | 102 | 0 | 1 |
| Path 425 | C00022->C00407:[1->2,1->5,2->3,3->6,3->9] | 0.83 | 480.641025641 | 18 | 39 | 0 | 1 |
| Path 426 | C00022->C00407:[2->3,2->5,3->9] | 0.50 | 406.703703704 | 22 | 81 | 0 | 1 |
| Path 427 | C00022->C00407:[1->2,3->6,3->9], C00036->C00407:[2->9,5->3,5->5] | 0.83 | 327.967213115 | 21 | 61 | 0 | 1 |
| Path 428 | C00022->C00407:[1->2,3->6] | 0.33 | 530.090909091 | 15 | 33 | 0 | 0 |
| Path 429 | C00022->C00407:[1->5,2->3,3->9], C00036->C00407:[3->3] | 0.50 | 537.129032258 | 18 | 31 | 0 | 1 |
| Path 430 | C00022->C00407:[1->2,1->5,3->6,3->9] | 0.67 | 499.631578947 | 12 | 19 | 0 | 0 |
| Path 431 | C00022->C00407:[1->2,1->5,3->6,3->9] | 0.67 | 451.075471698 | 22 | 53 | 0 | 0 |
| Path 432 | C00022->C00407:[1->1,2->3] | 0.33 | 491.684210526 | 12 | 19 | 0 | 0 |
| Path 433 | C00022->C00407:[1->2,1->5,3->6,3->9], C00036->C00407:[5->3] | 0.83 | 454.59375 | 17 | 32 | 0 | 1 |
| Path 434 | C00022->C00407:[1->1,1->2,2->3,3->6], C00036->C00407:[5->3] | 0.67 | 602.342105263 | 24 | 38 | 0 | 1 |
| Path 435 | C00022->C00407:[1->2,3->6] | 0.33 | 595.8 | 20 | 40 | 0 | 0 |
| Path 436 | C00022->C00407:[1->2,3->6,3->9], C00036->C00407:[2->9,5->3,5->5] | 0.83 | 317.639344262 | 21 | 61 | 0 | 1 |
| Path 437 | C00022->C00407:[1->2,2->3,3->6], C00036->C00407:[5->3] | 0.50 | 396.84 | 18 | 50 | 0 | 0 |
| Path 438 | C00022->C00407:[1->2,3->6], C00036->C00407:[5->3] | 0.50 | 435.166666667 | 14 | 42 | 0 | 0 |
| Path 439 | C00022->C00407:[1->2,1->5,3->6,3->9] | 0.67 | 280.136363636 | 15 | 44 | 0 | 0 |
| Path 440 | C00022->C00407:[1->2,1->5,3->6,3->9], C00036->C00407:[5->3] | 0.83 | 383.444444444 | 18 | 36 | 0 | 1 |
| Path 441 | C00022->C00407:[1->1,1->2,2->3,3->6], C00036->C00407:[1->1,3->3] | 0.67 | 326.228571429 | 14 | 35 | 0 | 0 |
| Path 442 | C00022->C00407:[1->1,1->2,2->3,3->6] | 0.67 | 478.275 | 20 | 40 | 0 | 0 |
| Path 443 | C00022->C00407:[1->2,3->6] | 0.33 | 579.529411765 | 19 | 34 | 0 | 0 |
| Path 444 | C00022->C00407:[1->1,1->2,2->3,3->6] | 0.67 | 490.380952381 | 14 | 21 | 0 | 0 |
| Path 445 | C00022->C00407:[1->2,3->6] | 0.33 | 540.206896552 | 10 | 29 | 0 | 0 |
| Path 446 | C00022->C00407:[1->2,1->5,3->6,3->9], C00036->C00407:[5->3] | 0.83 | 476.0625 | 24 | 48 | 0 | 1 |
| Path 447 | C00022->C00407:[1->2,3->6] | 0.33 | 551.423076923 | 14 | 26 | 0 | 0 |
| Path 448 | C00022->C00407:[1->2,3->6], C00036->C00407:[2->9,5->3,5->5] | 0.83 | 362.92 | 22 | 75 | 0 | 1 |
| Path 449 | C00022->C00407:[1->2,3->6], C00036->C00407:[5->3] | 0.50 | 417.185185185 | 15 | 27 | 0 | 1 |
| Path 450 | C00022->C00407:[1->2,1->5,3->6,3->9], C00036->C00407:[5->3] | 0.83 | 465.878787879 | 18 | 33 | 0 | 1 |
| Path 451 | C00036->C00407:[5->3] | 0.17 | 402.730769231 | 14 | 26 | 0 | 1 |
| Path 452 | C00022->C00407:[1->2,3->6] | 0.33 | 481.984848485 | 20 | 66 | 0 | 0 |
| Path 453 | C00022->C00407:[1->1,1->2,1->5,2->3,3->6,3->9], C00036->C00407:[5->3] | 1.00 | 478.705882353 | 24 | 51 | 0 | 1 |
| Path 454 | C00022->C00407:[3->9], C00036->C00407:[5->3,5->5] | 0.50 | 364.406779661 | 22 | 59 | 0 | 1 |
| Path 455 | C00022->C00407:[1->2,1->5,3->6,3->9], C00036->C00407:[5->3] | 0.83 | 480.68627451 | 20 | 51 | 0 | 1 |
| Path 456 | C00022->C00407:[1->2,3->6] | 0.33 | 444.5 | 18 | 42 | 0 | 0 |
| Path 457 | C00022->C00407:[1->2,1->5,2->3,3->6,3->9] | 0.83 | 494.446428571 | 19 | 56 | 0 | 1 |
| Path 458 | C00022->C00407:[1->2,3->6] | 0.33 | 351.739130435 | 19 | 69 | 0 | 0 |
| Path 459 | C00022->C00407:[1->1,1->2,1->5,2->3,3->6,3->9], C00036->C00407:[5->3] | 1.00 | 443.636363636 | 28 | 66 | 0 | 1 |
| Path 460 | C00022->C00407:[1->2,3->6], C00036->C00407:[2->9,5->3,5->5] | 0.83 | 297.410714286 | 19 | 56 | 0 | 1 |
| Path 461 | C00022->C00407:[1->5,3->9], C00036->C00407:[5->3] | 0.50 | 653.482758621 | 18 | 29 | 0 | 1 |
| Path 462 | C00022->C00407:[1->2,3->6], C00036->C00407:[5->3] | 0.50 | 347.368421053 | 22 | 76 | 0 | 1 |
| Path 463 | C00022->C00407:[1->2,2->3,3->6] | 0.50 | 473.315789474 | 17 | 38 | 0 | 1 |
| Path 464 | C00022->C00407:[1->2,3->6] | 0.33 | 349.513513514 | 20 | 74 | 0 | 0 |
| Path 465 | C00022->C00407:[1->1,1->2,1->5,2->3,3->6,3->9] | 1.00 | 499.65625 | 19 | 32 | 0 | 0 |
| Path 466 | C00022->C00407:[1->2,3->6], C00036->C00407:[5->3] | 0.50 | 302.420289855 | 17 | 69 | 0 | 0 |
| Path 467 | C00022->C00407:[1->5,2->3,3->9] | 0.50 | 311.35 | 14 | 40 | 0 | 2 |
| Path 468 | C00022->C00407:[1->2,3->6], C00036->C00407:[5->3] | 0.50 | 431.063829787 | 22 | 47 | 0 | 1 |
| Path 469 | C00022->C00407:[1->2,2->3,2->5,3->6,3->9], C00036->C00407:[5->3,5->5] | 0.83 | 362.271428571 | 22 | 70 | 0 | 1 |
| Path 470 | C00022->C00407:[1->2,3->6,3->9], C00036->C00407:[2->9,5->3,5->5] | 0.83 | 305.327586207 | 20 | 58 | 0 | 1 |
| Path 471 | C00022->C00407:[1->2,1->5,3->6,3->9] | 0.67 | 464.6 | 11 | 15 | 0 | 0 |
| Path 472 | C00022->C00407:[1->2,3->6], C00036->C00407:[5->3] | 0.50 | 532.536585366 | 22 | 41 | 0 | 1 |
| Path 473 | C00022->C00407:[2->3,2->5,3->9] | 0.50 | 443.71641791 | 23 | 67 | 0 | 1 |
| Path 474 | C00022->C00407:[1->2,1->5,3->6,3->9], C00036->C00407:[5->3] | 0.83 | 539.4 | 25 | 60 | 0 | 1 |
| Path 475 | C00022->C00407:[1->1,1->2,1->5,2->3,3->6,3->9], C00036->C00407:[5->3] | 1.00 | 606.358974359 | 25 | 39 | 0 | 1 |
| Path 476 | C00022->C00407:[1->2,1->5,2->3,3->6,3->9] | 0.83 | 503.462962963 | 24 | 54 | 0 | 1 |
| Path 477 | C00022->C00407:[1->2,3->6], C00036->C00407:[5->3] | 0.50 | 454.279069767 | 22 | 43 | 0 | 1 |
| Path 478 | C00022->C00407:[1->5,3->9], C00036->C00407:[1->1,3->3] | 0.67 | 284.709677419 | 13 | 31 | 0 | 0 |
| Path 479 | C00022->C00407:[1->2,3->6] | 0.33 | 403.916666667 | 17 | 36 | 0 | 0 |
| Path 480 | C00022->C00407:[1->1,1->2,1->5,2->3,3->6,3->9] | 1.00 | 553.102564103 | 23 | 39 | 0 | 0 |
| Path 481 | C00022->C00407:[1->2,1->5,3->6,3->9] | 0.67 | 604.793103448 | 18 | 29 | 0 | 0 |
| Path 482 | C00022->C00407:[1->1,1->2,2->3,3->6] | 0.67 | 520.380952381 | 14 | 21 | 0 | 0 |
| Path 483 | C00022->C00407:[1->2,3->6] | 0.33 | 605.785714286 | 16 | 42 | 0 | 0 |
