# Supplementary material for: 13C-metabolic flux ratio and novel carbon path analyses confirmed that Trichoderma reesei uses primarily the respirative pathway also on the preferred carbon source glucose
Source: BMC Syst Biol. 2009 Oct 29;3:104. doi: 10.1186/1752-0509-3-104 (PMC2776023; doi:10.1186/1752-0509-3-104)
Supplement: Additional file 1 — Pathways discovered in ReTrace carbon path analysis. Graphical and tabular representations of amino acid synthesis pathways discovered in ReTrace carbon path analysis [21]. Self-contained web site: unpack zip archive and open index.html with a web browser. [file 1752-0509-3-104-S1.zip › AF1-treesei/pathways-C00026-to-C00062.html]

Pathways from C00026 to C00062


**Pathways from C00026 to C00062**

**Sources:** 2-Oxoglutarate; (C00026)

**Target:**L-Arginine; (C00062)

|  | Composite mapping | Z | Average score | Rpairs | Reactions | Zero scores | Scores under threshold |
| --- | --- | --- | --- | --- | --- | --- | --- |
| Path 1 | C00026->C00062:[1->3,2->1,3->5,5->2,8->10,8->4] | 1.00 | 210.144329897 | 14 | 97 | 0 | 0 |
| Path 2 | C00026->C00062:[1->3,2->1,3->5,5->2,8->10,8->4] | 1.00 | 416.818181818 | 14 | 88 | 0 | 0 |
| Path 3 | C00026->C00062:[1->3,2->1,3->10,3->5,5->2,8->4] | 1.00 | 287.582089552 | 20 | 201 | 0 | 0 |
| Path 4 | C00026->C00062:[1->3,2->1,3->5,5->2,8->10,8->4] | 1.00 | 434.369565217 | 15 | 92 | 0 | 0 |
| Path 5 | C00026->C00062:[1->3,2->1,3->10,3->5,5->2,8->4] | 1.00 | 278.384146341 | 18 | 164 | 0 | 0 |
| Path 6 | C00026->C00062:[1->3,2->1,3->5,5->2,8->10,8->4] | 1.00 | 370.992063492 | 17 | 126 | 0 | 0 |
| Path 7 | C00026->C00062:[1->3,2->1,3->5,5->2,8->10,8->4] | 1.00 | 405.034090909 | 14 | 88 | 0 | 0 |
| Path 8 | C00026->C00062:[1->3,2->1,3->10,3->5,5->2,8->4] | 1.00 | 221.193277311 | 17 | 238 | 0 | 0 |
| Path 9 | C00026->C00062:[1->3,2->1,3->10,3->5,5->2,8->4] | 1.00 | 284.914634146 | 18 | 164 | 0 | 0 |
| Path 10 | C00026->C00062:[1->3,2->1,3->10,3->5,5->2,8->4] | 1.00 | 272.55625 | 15 | 160 | 0 | 1 |
| Path 11 | C00026->C00062:[1->3,2->1,3->5,5->2,8->10,8->4] | 1.00 | 373.496 | 16 | 125 | 0 | 0 |
| Path 12 | C00026->C00062:[1->3,2->1,3->5,5->2,8->10,8->4] | 1.00 | 425.733333333 | 17 | 60 | 0 | 0 |
| Path 13 | C00026->C00062:[1->3,2->1,3->5,5->2,8->10,8->4] | 1.00 | 262.950617284 | 13 | 162 | 0 | 0 |
| Path 14 | C00026->C00062:[1->3,2->1,3->5,5->2,8->10,8->4] | 1.00 | 379.492063492 | 17 | 126 | 0 | 0 |
| Path 15 | C00026->C00062:[1->3,2->1,3->5,5->2,8->10,8->4] | 1.00 | 261.693251534 | 14 | 163 | 0 | 0 |
| Path 16 | C00026->C00062:[1->3,2->1,3->5,5->2,8->10,8->4] | 1.00 | 400.752808989 | 15 | 89 | 0 | 0 |
| Path 17 | C00026->C00062:[1->3,2->1,3->5,5->2,8->10,8->4] | 1.00 | 628.956521739 | 15 | 23 | 0 | 0 |
| Path 18 | C00026->C00062:[1->3,2->1,3->10,3->5,5->2,8->4] | 1.00 | 256.137566138 | 12 | 189 | 0 | 1 |
| Path 19 | C00026->C00062:[1->3,2->1,3->5,5->2,8->10,8->4] | 1.00 | 407.883333333 | 17 | 60 | 0 | 0 |
| Path 20 | C00026->C00062:[1->3,2->1,3->10,3->5,5->2,8->4] | 1.00 | 254.641618497 | 14 | 173 | 0 | 1 |
| Path 21 | C00026->C00062:[1->3,2->1,3->5,5->2,8->10,8->4] | 1.00 | 412.786516854 | 15 | 89 | 0 | 0 |
| Path 22 | C00026->C00062:[1->3,2->1,3->10,3->5,5->2,8->4] | 1.00 | 286.063291139 | 14 | 158 | 0 | 1 |
| Path 23 | C00026->C00062:[1->3,2->1,3->5,5->2,8->10,8->4] | 1.00 | 427.10989011 | 14 | 91 | 0 | 0 |
| Path 24 | C00026->C00062:[1->3,2->1,3->5,5->2,8->10,8->4] | 1.00 | 376.314516129 | 15 | 124 | 0 | 0 |
| Path 25 | C00026->C00062:[1->3,2->1,3->5,5->2,8->10,8->4] | 1.00 | 382.064 | 16 | 125 | 0 | 0 |
| Path 26 | C00026->C00062:[1->3,2->1,3->5,5->2,8->10,8->4] | 1.00 | 640.5 | 15 | 26 | 0 | 0 |
| Path 27 | C00026->C00062:[1->3,2->1,3->5,5->2,8->10,8->4] | 1.00 | 297.666666667 | 19 | 168 | 0 | 0 |
| Path 28 | C00026->C00062:[1->3,2->1,3->10,3->5,5->2,8->4] | 1.00 | 245.605263158 | 10 | 152 | 0 | 1 |
| Path 29 | C00026->C00062:[1->3,2->1,3->5,5->2,8->10,8->4] | 1.00 | 404.647727273 | 14 | 88 | 0 | 0 |
| Path 30 | C00026->C00062:[1->3,2->1,3->10,3->5,5->2,8->4] | 1.00 | 283.545 | 19 | 200 | 0 | 0 |
| Path 31 | C00026->C00062:[1->3,2->1,3->5,5->2,8->10,8->4] | 1.00 | 657.333333333 | 16 | 27 | 0 | 0 |
| Path 32 | C00026->C00062:[1->3,2->1,3->5,5->2,8->10,8->4] | 1.00 | 292.892215569 | 18 | 167 | 0 | 0 |
| Path 33 | C00026->C00062:[1->3,2->1,3->10,3->5,5->2,8->4] | 1.00 | 199.00456621 | 13 | 219 | 0 | 1 |
| Path 34 | C00026->C00062:[1->3,2->1,3->10,3->5,5->2,8->4] | 1.00 | 282.253731343 | 20 | 201 | 0 | 0 |
| Path 35 | C00026->C00062:[1->3,2->1,3->10,3->5,5->2,8->4] | 1.00 | 247.241830065 | 11 | 153 | 0 | 1 |
| Path 36 | C00026->C00062:[1->3,2->1,3->10,3->5,5->2,8->4] | 1.00 | 279.944785276 | 17 | 163 | 0 | 0 |
| Path 37 | C00026->C00062:[1->3,2->1,3->5,5->2,8->10,8->4] | 1.00 | 423.097826087 | 15 | 92 | 0 | 0 |
| Path 38 | C00026->C00062:[1->3,2->1,3->5,5->2,8->10,8->4] | 1.00 | 430.322580645 | 16 | 93 | 0 | 0 |
| Path 39 | C00026->C00062:[1->3,2->1,3->5,5->2,8->10,8->4] | 1.00 | 409.022988506 | 13 | 87 | 0 | 0 |
| Path 40 | C00026->C00062:[1->3,2->1,3->5,5->2,8->10,8->4] | 1.00 | 373.768 | 16 | 125 | 0 | 0 |
| Path 41 | C00026->C00062:[1->3,2->1,3->5,5->2,8->10,8->4] | 1.00 | 414.389830508 | 16 | 59 | 0 | 0 |
| Path 42 | C00026->C00062:[1->3,2->1,3->5,5->2,8->10,8->4] | 1.00 | 582.391304348 | 15 | 23 | 0 | 0 |
| Path 43 | C00026->C00062:[1->3,2->1,3->5,5->2,8->10,8->4] | 1.00 | 607.772727273 | 14 | 22 | 0 | 0 |
| Path 44 | C00026->C00062:[8->10,8->5] | 0.33 | 432.892857143 | 16 | 56 | 0 | 0 |
| Path 45 | C00026->C00062:[8->10,8->5] | 0.33 | 665.652173913 | 14 | 23 | 0 | 0 |
| Path 46 | C00026->C00062:[3->10,3->5] | 0.33 | 158.603658537 | 13 | 164 | 0 | 0 |
| Path 47 | C00026->C00062:[3->10,3->5] | 0.33 | 160.815286624 | 17 | 157 | 0 | 1 |
| Path 48 | C00026->C00062:[8->10,8->5] | 0.33 | 401.381818182 | 15 | 55 | 0 | 0 |
| Path 49 | C00026->C00062:[1->10,1->5] | 0.33 | 189.759036145 | 15 | 83 | 0 | 1 |
| Path 50 | C00026->C00062:[1->3,2->1,3->10,3->5,5->2,8->4] | 1.00 | 358.607594937 | 9 | 79 | 0 | 1 |
| Path 51 | C00026->C00062:[1->3,2->1,5->2,8->10,8->4] | 0.83 | 430.410526316 | 17 | 95 | 0 | 0 |
| Path 52 | C00026->C00062:[8->10] | 0.17 | 400.423076923 | 12 | 52 | 0 | 0 |
| Path 53 | C00026->C00062:[8->10,8->5] | 0.33 | 638.769230769 | 16 | 26 | 0 | 0 |
| Path 54 | C00026->C00062:[3->10,3->5] | 0.33 | 243.5234375 | 17 | 128 | 0 | 1 |
| Path 55 | C00026->C00062:[3->10,3->5] | 0.33 | 211.088888889 | 14 | 90 | 0 | 1 |
| Path 56 | C00026->C00062:[1->3,2->1,5->2,8->10,8->4,8->5] | 1.00 | 377.589147287 | 19 | 129 | 0 | 0 |
| Path 57 | C00026->C00062:[8->10] | 0.17 | 393.320754717 | 13 | 53 | 0 | 0 |
| Path 58 | C00026->C00062:[8->10,8->5] | 0.33 | 593.75 | 12 | 16 | 0 | 1 |
| Path 59 | C00026->C00062:[8->10,8->5] | 0.33 | 692.842105263 | 14 | 19 | 0 | 0 |
| Path 60 | C00026->C00062:[1->3,2->1,3->10,3->5,5->2,8->4] | 1.00 | 432.255813953 | 13 | 86 | 0 | 1 |
| Path 61 | C00026->C00062:[8->10,8->5] | 0.33 | 645.529411765 | 12 | 17 | 0 | 0 |
| Path 62 | C00026->C00062:[3->10,3->5] | 0.33 | 209.032967033 | 15 | 91 | 0 | 1 |
| Path 63 | C00026->C00062:[1->3,2->1,5->2,8->10,8->4,8->5] | 1.00 | 268.795180723 | 16 | 166 | 0 | 0 |
| Path 64 | C00026->C00062:[8->10,8->5] | 0.33 | 700.0 | 16 | 25 | 0 | 0 |
| Path 65 | C00026->C00062:[1->3,2->1,5->2,8->10,8->4] | 0.83 | 437.333333333 | 18 | 96 | 0 | 0 |
| Path 66 | C00026->C00062:[3->10,3->5] | 0.33 | 229.848214286 | 19 | 112 | 0 | 1 |
| Path 67 | C00026->C00062:[3->10,3->5] | 0.33 | 268.677083333 | 18 | 96 | 0 | 1 |
| Path 68 | C00026->C00062:[1->3,2->1,3->5,5->2,8->4] | 0.83 | 356.846153846 | 8 | 78 | 0 | 1 |
| Path 69 | C00026->C00062:[3->10,3->5] | 0.33 | 231.702702703 | 18 | 111 | 0 | 1 |
| Path 70 | C00026->C00062:[1->10,1->5] | 0.33 | 200.535714286 | 16 | 84 | 0 | 1 |
| Path 71 | C00026->C00062:[3->10,3->5] | 0.33 | 166.727848101 | 18 | 158 | 0 | 1 |
| Path 72 | C00026->C00062:[3->5,8->10] | 0.33 | 239.569892473 | 14 | 93 | 0 | 0 |
| Path 73 | C00026->C00062:[3->10,3->5] | 0.33 | 239.410714286 | 19 | 112 | 0 | 1 |
| Path 74 | C00026->C00062:[1->10,1->5] | 0.33 | 455.684210526 | 17 | 38 | 0 | 1 |
| Path 75 | C00026->C00062:[8->10,8->5] | 0.33 | 613.6 | 14 | 20 | 0 | 0 |
| Path 76 | C00026->C00062:[3->10,3->5] | 0.33 | 269.795031056 | 17 | 161 | 0 | 1 |
| Path 77 | C00026->C00062:[1->10,1->3,2->1,3->5,5->2,8->4] | 1.00 | 241.891156463 | 12 | 147 | 0 | 1 |
| Path 78 | C00026->C00062:[3->10,3->5] | 0.33 | 409.93258427 | 16 | 89 | 0 | 1 |
| Path 79 | C00026->C00062:[8->10,8->5] | 0.33 | 611.0 | 13 | 18 | 0 | 0 |
| Path 80 | C00026->C00062:[3->10,8->10,8->5] | 0.33 | 740.375 | 17 | 24 | 0 | 1 |
| Path 81 | C00026->C00062:[3->10,3->5] | 0.33 | 213.460674157 | 13 | 89 | 0 | 0 |
| Path 82 | C00026->C00062:[1->10,1->5] | 0.33 | 240.475247525 | 18 | 101 | 0 | 0 |
| Path 83 | C00026->C00062:[1->3,2->1,3->10,3->5,5->2,8->4] | 1.00 | 367.950617284 | 10 | 81 | 0 | 1 |
| Path 84 | C00026->C00062:[3->10,3->5] | 0.33 | 223.255555556 | 14 | 90 | 0 | 0 |
| Path 85 | C00026->C00062:[1->3,2->1,5->2,8->10,8->4,8->5] | 1.00 | 380.3515625 | 18 | 128 | 0 | 0 |
| Path 86 | C00026->C00062:[1->3,2->1,3->10,3->5,5->2,8->4] | 1.00 | 239.053333333 | 9 | 150 | 0 | 1 |
| Path 87 | C00026->C00062:[1->3,2->1,3->5,5->2,8->10,8->4] | 1.00 | 396.639534884 | 13 | 86 | 0 | 1 |
| Path 88 | C00026->C00062:[1->10,1->3,2->1,3->5,5->2,8->4] | 1.00 | 349.04040404 | 12 | 99 | 0 | 1 |
| Path 89 | C00026->C00062:[3->10,3->5] | 0.33 | 402.413793103 | 15 | 87 | 0 | 1 |
| Path 90 | C00026->C00062:[3->10,3->5] | 0.33 | 220.802197802 | 15 | 91 | 0 | 1 |
| Path 91 | C00026->C00062:[1->10,1->3,2->1,3->5,5->2,8->4] | 1.00 | 235.062068966 | 11 | 145 | 0 | 1 |
| Path 92 | C00026->C00062:[3->10,3->5] | 0.33 | 236.818897638 | 16 | 127 | 0 | 1 |
| Path 93 | C00026->C00062:[8->10,8->5] | 0.33 | 409.649122807 | 16 | 57 | 0 | 0 |
| Path 94 | C00026->C00062:[3->10,3->5] | 0.33 | 235.15625 | 17 | 128 | 0 | 1 |
| Path 95 | C00026->C00062:[8->10] | 0.17 | 413.528301887 | 13 | 53 | 0 | 0 |
| Path 96 | C00026->C00062:[3->10,3->5] | 0.33 | 271.33125 | 16 | 160 | 0 | 1 |
| Path 97 | C00026->C00062:[8->10,8->5] | 0.33 | 657.363636364 | 16 | 22 | 0 | 0 |
| Path 98 | C00026->C00062:[3->10,8->10,8->5] | 0.33 | 811.347826087 | 17 | 23 | 0 | 1 |
| Path 99 | C00026->C00062:[3->10,3->5] | 0.33 | 280.894736842 | 18 | 95 | 0 | 1 |
| Path 100 | C00026->C00062:[3->10,8->10,8->5] | 0.33 | 784.333333333 | 15 | 21 | 0 | 1 |
| Path 101 | C00026->C00062:[8->10] | 0.17 | 685.578947368 | 11 | 19 | 0 | 0 |
| Path 102 | C00026->C00062:[1->3,2->1,5->2,8->10,8->4,8->5] | 1.00 | 420.673913043 | 17 | 92 | 0 | 0 |
| Path 103 | C00026->C00062:[3->10,3->5] | 0.33 | 199.136363636 | 13 | 88 | 0 | 1 |
| Path 104 | C00026->C00062:[3->10,3->5] | 0.33 | 245.440944882 | 16 | 127 | 0 | 0 |
| Path 105 | C00026->C00062:[3->10,3->5] | 0.33 | 159.949367089 | 18 | 158 | 0 | 1 |
| Path 106 | C00026->C00062:[3->10,3->5] | 0.33 | 276.447204969 | 17 | 161 | 0 | 1 |
| Path 107 | C00026->C00062:[3->10,3->5] | 0.33 | 238.698412698 | 15 | 126 | 0 | 0 |
| Path 108 | C00026->C00062:[1->3,2->1,5->2,8->10,8->4,8->5] | 1.00 | 413.263736264 | 16 | 91 | 0 | 0 |
| Path 109 | C00026->C00062:[3->10,3->5] | 0.33 | 390.103448276 | 15 | 87 | 0 | 1 |
| Path 110 | C00026->C00062:[3->10,3->5] | 0.33 | 394.360465116 | 14 | 86 | 0 | 1 |
| Path 111 | C00026->C00062:[1->10,1->5] | 0.33 | 187.785714286 | 16 | 84 | 0 | 1 |
| Path 112 | C00026->C00062:[1->3,2->1,5->2,8->10,8->4,8->5] | 1.00 | 385.891472868 | 19 | 129 | 0 | 0 |
| Path 113 | C00026->C00062:[3->10,3->5] | 0.33 | 209.202247191 | 14 | 89 | 0 | 1 |
| Path 114 | C00026->C00062:[3->5,8->10] | 0.33 | 248.670212766 | 15 | 94 | 0 | 0 |
| Path 115 | C00026->C00062:[3->10,3->5] | 0.33 | 397.898876404 | 16 | 89 | 0 | 1 |
| Path 116 | C00026->C00062:[1->10,1->5] | 0.33 | 242.64 | 17 | 100 | 0 | 0 |
| Path 117 | C00026->C00062:[8->10] | 0.17 | 706.05 | 12 | 20 | 0 | 0 |
| Path 118 | C00026->C00062:[8->10,8->5] | 0.33 | 560.235294118 | 13 | 17 | 0 | 1 |
| Path 119 | C00026->C00062:[3->10,3->5] | 0.33 | 263.387096774 | 16 | 93 | 0 | 1 |
| Path 120 | C00026->C00062:[8->10,8->5] | 0.33 | 671.357142857 | 18 | 28 | 0 | 0 |
| Path 121 | C00026->C00062:[8->10] | 0.17 | 186.188888889 | 10 | 90 | 0 | 0 |
| Path 122 | C00026->C00062:[8->10] | 0.17 | 677.4375 | 11 | 16 | 0 | 0 |
| Path 123 | C00026->C00062:[8->10] | 0.17 | 610.5 | 11 | 16 | 0 | 0 |
| Path 124 | C00026->C00062:[3->10,3->5] | 0.33 | 211.355555556 | 14 | 90 | 0 | 0 |
| Path 125 | C00026->C00062:[8->10,8->5] | 0.33 | 432.881355932 | 18 | 59 | 0 | 0 |
| Path 126 | C00026->C00062:[8->10,8->5] | 0.33 | 408.37037037 | 14 | 54 | 0 | 0 |
| Path 127 | C00026->C00062:[8->10] | 0.17 | 649.6 | 10 | 15 | 0 | 0 |
| Path 128 | C00026->C00062:[3->10,3->5] | 0.33 | 197.168539326 | 14 | 89 | 0 | 1 |
| Path 129 | C00026->C00062:[3->10,3->5] | 0.33 | 237.007874016 | 16 | 127 | 0 | 0 |
| Path 130 | C00026->C00062:[1->10,1->5] | 0.33 | 427.5 | 17 | 38 | 0 | 1 |
| Path 131 | C00026->C00062:[1->3,2->1,5->2,8->10,8->4,8->5] | 1.00 | 409.032608696 | 17 | 92 | 0 | 0 |
| Path 132 | C00026->C00062:[3->10,3->5] | 0.33 | 402.147727273 | 15 | 88 | 0 | 1 |
| Path 133 | C00026->C00062:[8->10,8->5] | 0.33 | 623.235294118 | 13 | 17 | 0 | 1 |
| Path 134 | C00026->C00062:[1->10,1->5] | 0.33 | 438.405405405 | 16 | 37 | 0 | 1 |
