# Supplementary material for: 13C-metabolic flux ratio and novel carbon path analyses confirmed that Trichoderma reesei uses primarily the respirative pathway also on the preferred carbon source glucose
Source: BMC Syst Biol. 2009 Oct 29;3:104. doi: 10.1186/1752-0509-3-104 (PMC2776023; doi:10.1186/1752-0509-3-104)
Supplement: Additional file 1 — Pathways discovered in ReTrace carbon path analysis. Graphical and tabular representations of amino acid synthesis pathways discovered in ReTrace carbon path analysis [21]. Self-contained web site: unpack zip archive and open index.html with a web browser. [file 1752-0509-3-104-S1.zip › AF1-treesei/pathways-C00031-to-C00188.html]

Pathways from C00031 to C00188


**Pathways from C00031 to C00188**

**Sources:** D-Glucose; (C00031)

**Target:**L-Threonine; (C00188)

|  | Composite mapping | Z | Average score | Rpairs | Reactions | Zero scores | Scores under threshold |
| --- | --- | --- | --- | --- | --- | --- | --- |
| Path 1 | C00031->C00188:[4->2,4->3] | 0.50 | 433.03125 | 22 | 32 | 1 | 2 |
| Path 2 | C00031->C00188:[4->2,4->3,7->5] | 0.75 | 413.597402597 | 31 | 77 | 1 | 2 |
| Path 3 | C00031->C00188:[4->2,4->3] | 0.50 | 369.433962264 | 26 | 53 | 1 | 2 |
| Path 4 | C00031->C00188:[4->2,4->3,7->5] | 0.75 | 415.0 | 28 | 47 | 1 | 2 |
| Path 5 | C00031->C00188:[4->2,4->3,7->5] | 0.75 | 422.735849057 | 31 | 53 | 1 | 2 |
| Path 6 | C00031->C00188:[4->2,4->3,7->5] | 0.75 | 408.328767123 | 29 | 73 | 1 | 2 |
| Path 7 | C00031->C00188:[4->2,4->3,7->5] | 0.75 | 406.186046512 | 26 | 43 | 1 | 2 |
| Path 8 | C00031->C00188:[4->2,4->3,7->5] | 0.75 | 362.328125 | 30 | 64 | 1 | 2 |
| Path 9 | C00031->C00188:[4->2,4->3,7->5] | 0.75 | 403.613636364 | 27 | 44 | 1 | 2 |
| Path 10 | C00031->C00188:[1->2,4->3,4->5] | 0.75 | 439.028169014 | 29 | 71 | 1 | 2 |
| Path 11 | C00031->C00188:[4->2,4->3,7->5,9->2] | 0.75 | 321.057971014 | 31 | 69 | 1 | 2 |
| Path 12 | C00031->C00188:[4->2,4->3] | 0.50 | 428.787878788 | 23 | 33 | 1 | 2 |
| Path 13 | C00031->C00188:[4->2,4->3,7->5,9->2] | 0.75 | 309.446153846 | 29 | 65 | 1 | 2 |
| Path 14 | C00031->C00188:[4->2,4->3,7->5] | 0.75 | 412.458333333 | 29 | 48 | 1 | 2 |
| Path 15 | C00031->C00188:[4->2,4->3] | 0.50 | 544.028571429 | 26 | 35 | 1 | 2 |
| Path 16 | C00031->C00188:[4->2,4->3] | 0.50 | 437.540540541 | 25 | 37 | 1 | 2 |
| Path 17 | C00031->C00188:[4->2,4->3,7->5] | 0.75 | 352.5 | 28 | 60 | 1 | 2 |
| Path 18 | C00031->C00188:[4->3] | 0.25 | 420.72 | 15 | 25 | 1 | 2 |
| Path 19 | C00031->C00188:[4->2,4->3] | 0.50 | 540.512820513 | 28 | 39 | 1 | 2 |
| Path 20 | C00031->C00188:[4->2,4->3] | 0.50 | 441.555555556 | 24 | 36 | 1 | 2 |
| Path 21 | C00031->C00188:[4->2,4->3] | 0.50 | 357.979591837 | 24 | 49 | 1 | 2 |
| Path 22 | C00031->C00188:[4->3,7->5,9->2] | 0.75 | 286.0 | 24 | 58 | 1 | 2 |
| Path 23 | C00031->C00188:[4->2,4->3] | 0.50 | 440.257142857 | 23 | 35 | 1 | 2 |
| Path 24 | C00031->C00188:[4->2,4->3] | 0.50 | 452.177777778 | 28 | 45 | 1 | 2 |
| Path 25 | C00031->C00188:[4->2,4->3,7->5] | 0.75 | 413.434782609 | 27 | 46 | 1 | 2 |
| Path 26 | C00031->C00188:[4->3,7->5,9->2] | 0.75 | 297.37704918 | 25 | 61 | 1 | 2 |
| Path 27 | C00031->C00188:[4->2,4->3,7->5] | 0.75 | 427.803571429 | 32 | 56 | 1 | 2 |
| Path 28 | C00031->C00188:[4->3] | 0.25 | 407.545454545 | 14 | 22 | 1 | 2 |
| Path 29 | C00031->C00188:[4->2,4->3] | 0.50 | 447.523809524 | 27 | 42 | 1 | 2 |
| Path 30 | C00031->C00188:[1->2,4->3,4->5] | 0.75 | 442.202702703 | 30 | 74 | 1 | 2 |
