# Supplementary material for: 13C-metabolic flux ratio and novel carbon path analyses confirmed that Trichoderma reesei uses primarily the respirative pathway also on the preferred carbon source glucose
Source: BMC Syst Biol. 2009 Oct 29;3:104. doi: 10.1186/1752-0509-3-104 (PMC2776023; doi:10.1186/1752-0509-3-104)
Supplement: Additional file 1 — Pathways discovered in ReTrace carbon path analysis. Graphical and tabular representations of amino acid synthesis pathways discovered in ReTrace carbon path analysis [21]. Self-contained web site: unpack zip archive and open index.html with a web browser. [file 1752-0509-3-104-S1.zip › AF1-treesei/pathways-C00188-to-C00037.html]

Pathways from C00188 to C00037


**Pathways from C00188 to C00037**

**Sources:** L-Threonine; (C00188)

**Target:**Glycine; (C00037)

|  | Composite mapping | Z | Average score | Rpairs | Reactions | Zero scores | Scores under threshold |
| --- | --- | --- | --- | --- | --- | --- | --- |
| Path 1 | C00188->C00037:[2->1,5->2] | 1.00 | 415.019230769 | 14 | 52 | 0 | 0 |
| Path 2 | C00188->C00037:[2->1,2->2] | 1.00 | 273.566037736 | 10 | 53 | 0 | 0 |
| Path 3 | C00188->C00037:[2->1,5->2] | 1.00 | 462.0 | 9 | 22 | 0 | 0 |
| Path 4 | C00188->C00037:[2->1,2->2] | 1.00 | 268.384615385 | 10 | 52 | 0 | 0 |
| Path 5 | C00188->C00037:[2->1,2->2] | 1.00 | 432.666666667 | 10 | 27 | 0 | 0 |
| Path 6 | C00188->C00037:[2->1,2->2] | 1.00 | 306.880952381 | 9 | 42 | 0 | 0 |
| Path 7 | C00188->C00037:[2->1,5->2] | 1.00 | 229.065217391 | 9 | 46 | 0 | 0 |
| Path 8 | C00188->C00037:[2->1,5->2] | 1.00 | 433.208333333 | 14 | 48 | 0 | 0 |
| Path 9 | C00188->C00037:[2->1,2->2] | 1.00 | 326.111111111 | 9 | 36 | 0 | 0 |
| Path 10 | C00188->C00037:[2->1,5->2] | 1.00 | 425.553191489 | 13 | 47 | 0 | 0 |
| Path 11 | C00188->C00037:[2->1,5->2] | 1.00 | 346.779411765 | 14 | 68 | 0 | 0 |
| Path 12 | C00188->C00037:[2->1,2->2] | 1.00 | 405.2 | 8 | 25 | 0 | 0 |
| Path 13 | C00188->C00037:[1->2,3->1] | 1.00 | 455.5 | 2 | 2 | 0 | 0 |
| Path 14 | C00188->C00037:[2->1,2->2] | 1.00 | 354.909090909 | 10 | 33 | 0 | 0 |
| Path 15 | C00188->C00037:[2->1,2->2] | 1.00 | 247.079365079 | 11 | 63 | 0 | 0 |
| Path 16 | C00188->C00037:[1->2,3->1] | 1.00 | 280.0 | 1 | 1 | 0 | 0 |
| Path 17 | C00188->C00037:[2->1,5->2] | 1.00 | 400.58 | 12 | 50 | 0 | 0 |
| Path 18 | C00188->C00037:[2->1,2->2] | 1.00 | 278.283018868 | 11 | 53 | 0 | 0 |
| Path 19 | C00188->C00037:[2->1,5->2] | 1.00 | 340.119402985 | 13 | 67 | 0 | 0 |
| Path 20 | C00188->C00037:[2->1,2->2] | 1.00 | 318.186046512 | 10 | 43 | 0 | 0 |
| Path 21 | C00188->C00037:[2->1,5->2] | 1.00 | 300.704545455 | 10 | 44 | 0 | 0 |
| Path 22 | C00188->C00037:[2->1,5->2] | 1.00 | 311.25 | 8 | 24 | 0 | 0 |
| Path 23 | C00188->C00037:[2->1,2->2] | 1.00 | 473.695652174 | 10 | 23 | 0 | 0 |
| Path 24 | C00188->C00037:[2->1,2->2] | 1.00 | 459.181818182 | 9 | 22 | 0 | 0 |
| Path 25 | C00188->C00037:[2->1,2->2] | 1.00 | 356.967213115 | 22 | 61 | 0 | 1 |
| Path 26 | C00188->C00037:[2->1,2->2] | 1.00 | 276.720930233 | 17 | 43 | 0 | 1 |
| Path 27 | C00188->C00037:[2->1,5->2] | 1.00 | 446.131578947 | 17 | 38 | 0 | 0 |
| Path 28 | C00188->C00037:[2->1,2->2] | 1.00 | 353.627118644 | 20 | 59 | 0 | 1 |
| Path 29 | C00188->C00037:[2->1,5->2] | 1.00 | 422.380952381 | 17 | 42 | 0 | 0 |
| Path 30 | C00188->C00037:[2->1,5->2] | 1.00 | 415.19047619 | 17 | 42 | 0 | 0 |
| Path 31 | C00188->C00037:[2->2,5->1] | 1.00 | 521.5 | 11 | 14 | 0 | 1 |
| Path 32 | C00188->C00037:[2->1,5->2] | 1.00 | 340.344827586 | 17 | 58 | 0 | 0 |
| Path 33 | C00188->C00037:[2->1,5->2] | 1.00 | 428.594594595 | 16 | 37 | 0 | 0 |
| Path 34 | C00188->C00037:[2->1,2->2] | 1.00 | 346.637931034 | 19 | 58 | 0 | 1 |
| Path 35 | C00188->C00037:[2->2] | 0.50 | 313.607142857 | 6 | 28 | 0 | 0 |
| Path 36 | C00188->C00037:[2->1,5->2] | 1.00 | 418.472222222 | 15 | 36 | 0 | 0 |
| Path 37 | C00188->C00037:[2->1,5->2] | 1.00 | 335.137931034 | 17 | 58 | 0 | 0 |
| Path 38 | C00188->C00037:[2->1,5->2] | 1.00 | 436.756756757 | 16 | 37 | 0 | 0 |
| Path 39 | C00188->C00037:[2->1,5->2] | 1.00 | 318.063492063 | 18 | 63 | 0 | 0 |
| Path 40 | C00188->C00037:[2->1,5->2] | 1.00 | 404.7 | 15 | 40 | 0 | 0 |
| Path 41 | C00188->C00037:[5->2] | 0.50 | 415.418604651 | 10 | 43 | 0 | 0 |
| Path 42 | C00188->C00037:[5->2] | 0.50 | 403.96875 | 12 | 32 | 0 | 0 |
| Path 43 | C00188->C00037:[2->1,5->2] | 1.00 | 300.533333333 | 15 | 60 | 0 | 0 |
| Path 44 | C00188->C00037:[5->2] | 0.50 | 369.857142857 | 12 | 35 | 0 | 0 |
| Path 45 | C00188->C00037:[2->1,5->2] | 1.00 | 387.0 | 14 | 39 | 0 | 0 |
| Path 46 | C00188->C00037:[2->1,5->2] | 1.00 | 411.794871795 | 15 | 39 | 0 | 0 |
| Path 47 | C00188->C00037:[5->2] | 0.50 | 424.909090909 | 13 | 33 | 0 | 0 |
| Path 48 | C00188->C00037:[2->1,5->2] | 1.00 | 305.532258065 | 17 | 62 | 0 | 0 |
| Path 49 | C00188->C00037:[2->1] | 0.50 | 250.34375 | 9 | 32 | 0 | 1 |
| Path 50 | C00188->C00037:[2->2] | 0.50 | 442.444444444 | 6 | 18 | 0 | 0 |
| Path 51 | C00188->C00037:[5->2] | 0.50 | 413.40625 | 12 | 32 | 0 | 0 |
| Path 52 | C00188->C00037:[2->1,5->2] | 1.00 | 318.785714286 | 15 | 56 | 0 | 0 |
| Path 53 | C00188->C00037:[2->2] | 0.50 | 421.823529412 | 5 | 17 | 0 | 0 |
| Path 54 | C00188->C00037:[5->2] | 0.50 | 391.419354839 | 11 | 31 | 0 | 0 |
| Path 55 | C00188->C00037:[2->1,5->2] | 1.00 | 393.815789474 | 14 | 38 | 0 | 0 |
| Path 56 | C00188->C00037:[5->2] | 0.50 | 280.912280702 | 13 | 57 | 0 | 0 |
| Path 57 | C00188->C00037:[2->1,5->2] | 1.00 | 332.403508772 | 16 | 57 | 0 | 0 |
| Path 58 | C00188->C00037:[2->1,5->2] | 1.00 | 405.975609756 | 16 | 41 | 0 | 0 |
| Path 59 | C00188->C00037:[2->1,5->2] | 1.00 | 438.184210526 | 17 | 38 | 0 | 0 |
| Path 60 | C00188->C00037:[2->1,5->2] | 1.00 | 327.105263158 | 16 | 57 | 0 | 0 |
| Path 61 | C00188->C00037:[2->2,5->1] | 1.00 | 429.652173913 | 17 | 23 | 0 | 1 |
| Path 62 | C00188->C00037:[2->1,2->2] | 1.00 | 351.538461538 | 16 | 26 | 0 | 1 |
| Path 63 | C00188->C00037:[2->1] | 0.50 | 375.6875 | 9 | 16 | 0 | 1 |
| Path 64 | C00188->C00037:[2->1] | 0.50 | 494.4 | 8 | 10 | 0 | 1 |
| Path 65 | C00188->C00037:[2->1,2->2] | 1.00 | 350.266666667 | 21 | 60 | 0 | 1 |
| Path 66 | C00188->C00037:[2->1,2->2] | 1.00 | 244.641509434 | 18 | 53 | 0 | 1 |
| Path 67 | C00188->C00037:[5->2] | 0.50 | 415.757575758 | 13 | 33 | 0 | 0 |
| Path 68 | C00188->C00037:[2->1] | 0.50 | 216.142857143 | 10 | 42 | 0 | 1 |
| Path 69 | C00188->C00037:[5->2] | 0.50 | 406.428571429 | 9 | 42 | 0 | 0 |
| Path 70 | C00188->C00037:[5->2] | 0.50 | 390.0 | 13 | 36 | 0 | 0 |
| Path 71 | C00188->C00037:[2->1,2->2] | 1.00 | 396.173913043 | 17 | 23 | 0 | 1 |
