# Supplementary material for: 13C-metabolic flux ratio and novel carbon path analyses confirmed that Trichoderma reesei uses primarily the respirative pathway also on the preferred carbon source glucose
Source: BMC Syst Biol. 2009 Oct 29;3:104. doi: 10.1186/1752-0509-3-104 (PMC2776023; doi:10.1186/1752-0509-3-104)
Supplement: Additional file 1 — Pathways discovered in ReTrace carbon path analysis. Graphical and tabular representations of amino acid synthesis pathways discovered in ReTrace carbon path analysis [21]. Self-contained web site: unpack zip archive and open index.html with a web browser. [file 1752-0509-3-104-S1.zip › AF1-treesei/pathways-C00031-to-C00079.html]

Pathways from C00031 to C00079


**Pathways from C00031 to C00079**

**Sources:** D-Glucose; (C00031)

**Target:**L-Phenylalanine; (C00079)

|  | Composite mapping | Z | Average score | Rpairs | Reactions | Zero scores | Scores under threshold |
| --- | --- | --- | --- | --- | --- | --- | --- |
| Path 1 | C00031->C00079:[1->1,1->5,1->6,2->10,2->8,4->2,4->3,4->4,5->7] | 1.00 | 535.895833333 | 21 | 48 | 0 | 0 |
| Path 2 | C00031->C00079:[1->6,2->10,4->3,4->8,5->7,7->2,7->4,9->1,9->5] | 1.00 | 545.323076923 | 23 | 65 | 0 | 0 |
| Path 3 | C00031->C00079:[7->5,7->8] | 0.22 | 420.173913043 | 20 | 69 | 1 | 1 |
| Path 4 | C00031->C00079:[1->10,2->6,4->7,5->3,7->1,7->2,9->4,9->5,9->8] | 1.00 | 405.862068966 | 28 | 87 | 1 | 1 |
| Path 5 | C00031->C00079:[9->5,9->8] | 0.22 | 247.470588235 | 16 | 51 | 1 | 1 |
| Path 6 | C00031->C00079:[9->8] | 0.11 | 222.444444444 | 11 | 45 | 1 | 1 |
| Path 7 | C00031->C00079:[7->5,7->8] | 0.22 | 231.242424242 | 21 | 66 | 2 | 2 |
| Path 8 | C00031->C00079:[7->5,7->8] | 0.22 | 333.29787234 | 19 | 47 | 2 | 2 |
| Path 9 | C00031->C00079:[1->10,2->6,4->7,5->3,7->1,7->2,9->4,9->5,9->8] | 1.00 | 455.126582278 | 29 | 79 | 1 | 1 |
| Path 10 | C00031->C00079:[4->8] | 0.11 | 334.333333333 | 8 | 18 | 1 | 1 |
| Path 11 | C00031->C00079:[9->5,9->8] | 0.22 | 253.173076923 | 17 | 52 | 2 | 2 |
| Path 12 | C00031->C00079:[4->5,4->8] | 0.22 | 418.808219178 | 25 | 73 | 2 | 2 |
| Path 13 | C00031->C00079:[1->6,2->10,4->3,4->8,5->7,7->2,7->4,9->1,9->5] | 1.00 | 478.8 | 35 | 80 | 0 | 0 |
| Path 14 | C00031->C00079:[4->5,4->8] | 0.22 | 325.730769231 | 13 | 26 | 2 | 2 |
| Path 15 | C00031->C00079:[1->10,2->6,4->7,5->3,7->1,7->2,9->4,9->5,9->8] | 1.00 | 536.540983607 | 26 | 61 | 1 | 1 |
| Path 16 | C00031->C00079:[4->5,4->8] | 0.22 | 257.506849315 | 23 | 73 | 2 | 2 |
| Path 17 | C00031->C00079:[4->5,4->8,7->5,7->8] | 0.22 | 323.918918919 | 18 | 37 | 2 | 2 |
| Path 18 | C00031->C00079:[1->10,2->6,4->7,5->3,5->8] | 0.56 | 497.070175439 | 26 | 57 | 1 | 1 |
| Path 19 | C00031->C00079:[4->5,4->8] | 0.22 | 297.35483871 | 14 | 31 | 1 | 1 |
| Path 20 | C00031->C00079:[9->5,9->8] | 0.22 | 354.934065934 | 22 | 91 | 1 | 1 |
| Path 21 | C00031->C00079:[4->5,4->8] | 0.22 | 269.65 | 21 | 60 | 2 | 2 |
| Path 22 | C00031->C00079:[4->5,4->8] | 0.22 | 246.371428571 | 22 | 70 | 2 | 2 |
| Path 23 | C00031->C00079:[1->6,2->10,4->3,5->7,7->2,7->4,7->8,9->1,9->5] | 1.00 | 516.3375 | 33 | 80 | 0 | 0 |
| Path 24 | C00031->C00079:[1->10,2->6,4->7,5->3,9->8] | 0.56 | 403.587301587 | 25 | 63 | 1 | 1 |
| Path 25 | C00031->C00079:[4->5,4->8] | 0.22 | 425.333333333 | 26 | 81 | 2 | 2 |
| Path 26 | C00031->C00079:[4->5,4->8] | 0.22 | 294.6 | 24 | 65 | 2 | 2 |
| Path 27 | C00031->C00079:[1->10,2->6,4->5,4->7,4->8,5->3,5->4,7->1,7->2,7->5,7->8,9->4] | 1.00 | 535.328571429 | 35 | 70 | 2 | 2 |
| Path 28 | C00031->C00079:[4->5,4->8] | 0.22 | 310.224489796 | 20 | 49 | 1 | 1 |
| Path 29 | C00031->C00079:[1->6,2->10,4->3,5->7,7->8] | 0.56 | 529.526315789 | 31 | 76 | 0 | 0 |
| Path 30 | C00031->C00079:[1->10,2->6,4->7,5->3,7->8] | 0.56 | 539.487804878 | 23 | 41 | 1 | 1 |
| Path 31 | C00031->C00079:[1->6,2->10,4->3,4->8,5->7,7->2,7->4,9->1,9->5] | 1.00 | 480.192771084 | 36 | 83 | 0 | 0 |
| Path 32 | C00031->C00079:[4->5,4->8] | 0.22 | 409.724637681 | 23 | 69 | 2 | 2 |
| Path 33 | C00031->C00079:[7->5,7->8] | 0.22 | 349.756756757 | 19 | 37 | 2 | 2 |
| Path 34 | C00031->C00079:[2->8,7->2,9->5] | 0.33 | 429.935064935 | 24 | 77 | 3 | 3 |
| Path 35 | C00031->C00079:[4->5,4->8] | 0.22 | 422.897058824 | 25 | 68 | 2 | 2 |
| Path 36 | C00031->C00079:[4->5,4->8] | 0.22 | 348.729166667 | 21 | 48 | 1 | 1 |
| Path 37 | C00031->C00079:[1->6,2->10,4->3,4->8,5->7] | 0.56 | 490.013157895 | 33 | 76 | 0 | 0 |
| Path 38 | C00031->C00079:[1->6,2->10,4->3,4->8,5->7,7->2,7->4,9->1,9->5] | 1.00 | 412.010204082 | 36 | 98 | 0 | 0 |
| Path 39 | C00031->C00079:[9->8] | 0.11 | 351.482758621 | 12 | 29 | 1 | 1 |
| Path 40 | C00031->C00079:[7->5,7->8] | 0.22 | 308.189189189 | 17 | 37 | 1 | 1 |
| Path 41 | C00031->C00079:[4->5,4->8] | 0.22 | 260.608108108 | 24 | 74 | 2 | 2 |
| Path 42 | C00031->C00079:[1->10,2->6,4->7,5->3,5->8,7->1,7->2,9->4,9->5] | 1.00 | 526.819444444 | 29 | 72 | 1 | 1 |
| Path 43 | C00031->C00079:[1->6,2->10,4->3,5->7,7->2,7->4,7->8,9->1,9->5] | 1.00 | 442.653061224 | 34 | 98 | 0 | 0 |
| Path 44 | C00031->C00079:[4->5,4->8] | 0.22 | 257.016129032 | 20 | 62 | 1 | 1 |
| Path 45 | C00031->C00079:[7->5,7->8] | 0.22 | 393.014492754 | 23 | 69 | 2 | 2 |
| Path 46 | C00031->C00079:[7->5,7->8] | 0.22 | 416.835616438 | 23 | 73 | 2 | 2 |
| Path 47 | C00031->C00079:[4->5,4->8,7->5,7->8] | 0.22 | 426.605263158 | 28 | 76 | 2 | 2 |
| Path 48 | C00031->C00079:[1->6,2->10,4->3,4->8,5->7] | 0.56 | 551.450980392 | 24 | 51 | 0 | 0 |
| Path 49 | C00031->C00079:[1->10,2->6,4->5,4->7,4->8,5->3,5->4,7->1,7->2,9->4] | 1.00 | 507.988372093 | 38 | 86 | 2 | 2 |
| Path 50 | C00031->C00079:[7->5,7->8] | 0.22 | 396.597222222 | 25 | 72 | 2 | 2 |
| Path 51 | C00031->C00079:[4->5,4->8] | 0.22 | 291.033333333 | 13 | 30 | 1 | 1 |
| Path 52 | C00031->C00079:[4->5,4->8] | 0.22 | 314.409090909 | 11 | 22 | 1 | 1 |
| Path 53 | C00031->C00079:[7->5,7->8] | 0.22 | 414.333333333 | 24 | 69 | 2 | 2 |
| Path 54 | C00031->C00079:[4->5,4->8] | 0.22 | 414.208333333 | 24 | 72 | 2 | 2 |
| Path 55 | C00031->C00079:[1->10,2->6,4->5,4->7,4->8,5->3,5->4,7->1,7->2,9->4] | 1.00 | 439.549450549 | 36 | 91 | 2 | 2 |
| Path 56 | C00031->C00079:[7->5,7->8] | 0.22 | 411.393939394 | 22 | 66 | 2 | 2 |
| Path 57 | C00031->C00079:[4->5,4->8,7->5,7->8] | 0.22 | 261.575342466 | 25 | 73 | 2 | 2 |
| Path 58 | C00031->C00079:[4->5,4->8] | 0.22 | 400.487179487 | 22 | 78 | 1 | 1 |
| Path 59 | C00031->C00079:[4->5,4->8] | 0.22 | 420.383561644 | 24 | 73 | 2 | 2 |
| Path 60 | C00031->C00079:[7->5,7->8] | 0.22 | 414.171428571 | 21 | 70 | 2 | 2 |
| Path 61 | C00031->C00079:[7->5,7->8] | 0.22 | 253.5 | 19 | 54 | 2 | 2 |
| Path 62 | C00031->C00079:[4->5,4->8] | 0.22 | 341.166666667 | 22 | 48 | 2 | 2 |
| Path 63 | C00031->C00079:[9->5,9->8] | 0.22 | 216.527272727 | 16 | 55 | 1 | 1 |
| Path 64 | C00031->C00079:[4->5,4->8] | 0.22 | 428.0 | 22 | 76 | 1 | 1 |
| Path 65 | C00031->C00079:[7->5,7->8] | 0.22 | 300.078947368 | 18 | 38 | 2 | 2 |
| Path 66 | C00031->C00079:[9->5,9->8] | 0.22 | 258.137254902 | 16 | 51 | 1 | 1 |
| Path 67 | C00031->C00079:[4->8,7->2,9->5] | 0.33 | 376.9 | 17 | 50 | 2 | 2 |
| Path 68 | C00031->C00079:[4->5,4->8,7->5,7->8] | 0.22 | 428.275 | 27 | 80 | 2 | 2 |
| Path 69 | C00031->C00079:[4->5,4->8] | 0.22 | 422.697368421 | 26 | 76 | 2 | 2 |
| Path 70 | C00031->C00079:[4->5,4->8] | 0.22 | 364.581632653 | 30 | 98 | 2 | 2 |
| Path 71 | C00031->C00079:[9->8] | 0.11 | 288.5 | 11 | 30 | 1 | 1 |
| Path 72 | C00031->C00079:[9->5,9->8] | 0.22 | 346.375 | 25 | 88 | 2 | 2 |
| Path 73 | C00031->C00079:[4->5,4->8,7->5,7->8] | 0.22 | 336.111111111 | 16 | 36 | 1 | 1 |
| Path 74 | C00031->C00079:[9->5,9->8] | 0.22 | 210.161290323 | 17 | 62 | 2 | 2 |
| Path 75 | C00031->C00079:[4->5,4->8] | 0.22 | 418.594594595 | 22 | 74 | 2 | 2 |
| Path 76 | C00031->C00079:[4->5,4->8] | 0.22 | 248.014285714 | 21 | 70 | 2 | 2 |
| Path 77 | C00031->C00079:[7->2,9->5,9->8] | 0.33 | 338.976744186 | 19 | 43 | 3 | 3 |
| Path 78 | C00031->C00079:[1->10,2->6,4->5,4->7,4->8,5->3,5->4,7->1,7->2,9->4] | 1.00 | 513.133333333 | 35 | 75 | 2 | 2 |
| Path 79 | C00031->C00079:[4->5,4->8,7->5,7->8] | 0.22 | 414.432432432 | 27 | 74 | 2 | 2 |
| Path 80 | C00031->C00079:[1->6,2->10,4->3,4->8,5->7,7->2,7->4,9->1,9->5] | 1.00 | 493.792207792 | 34 | 77 | 0 | 0 |
| Path 81 | C00031->C00079:[4->5,4->8] | 0.22 | 404.814814815 | 23 | 81 | 1 | 1 |
| Path 82 | C00031->C00079:[9->5,9->8] | 0.22 | 351.076086957 | 23 | 92 | 2 | 2 |
| Path 83 | C00031->C00079:[4->5,4->8] | 0.22 | 269.030769231 | 21 | 65 | 1 | 1 |
| Path 84 | C00031->C00079:[1->6,2->10,4->3,5->7,7->2,7->4,9->1,9->5] | 0.89 | 545.051724138 | 19 | 58 | 0 | 0 |
| Path 85 | C00031->C00079:[7->2,9->5,9->8] | 0.33 | 291.836065574 | 22 | 61 | 3 | 3 |
| Path 86 | C00031->C00079:[4->5,4->8] | 0.22 | 346.058823529 | 20 | 51 | 2 | 2 |
| Path 87 | C00031->C00079:[9->5,9->8] | 0.22 | 242.711538462 | 17 | 52 | 2 | 2 |
| Path 88 | C00031->C00079:[7->2,7->8,9->5] | 0.33 | 356.023255814 | 15 | 43 | 2 | 2 |
| Path 89 | C00031->C00079:[4->5,4->8] | 0.22 | 254.166666667 | 23 | 72 | 2 | 2 |
| Path 90 | C00031->C00079:[1->10,2->6,4->7,5->3,9->8] | 0.56 | 544.872340426 | 26 | 47 | 1 | 1 |
| Path 91 | C00031->C00079:[7->5,7->8] | 0.22 | 359.45 | 21 | 40 | 2 | 2 |
| Path 92 | C00031->C00079:[4->5,4->8] | 0.22 | 339.522727273 | 19 | 44 | 1 | 1 |
| Path 93 | C00031->C00079:[4->5,4->8] | 0.22 | 371.863636364 | 22 | 44 | 2 | 2 |
| Path 94 | C00031->C00079:[1->10,2->6,4->7,4->8,5->3] | 0.56 | 534.479166667 | 25 | 48 | 1 | 1 |
| Path 95 | C00031->C00079:[7->5,7->8] | 0.22 | 253.482142857 | 20 | 56 | 2 | 2 |
| Path 96 | C00031->C00079:[1->6,2->10,4->3,4->8,5->7,7->2,7->4,9->1,9->5] | 1.00 | 446.819047619 | 36 | 105 | 0 | 0 |
| Path 97 | C00031->C00079:[4->8,7->2,9->5] | 0.33 | 370.19047619 | 20 | 42 | 3 | 3 |
| Path 98 | C00031->C00079:[7->5,7->8] | 0.22 | 271.733333333 | 14 | 30 | 2 | 2 |
| Path 99 | C00031->C00079:[4->5,4->8] | 0.22 | 271.566666667 | 20 | 60 | 2 | 2 |
| Path 100 | C00031->C00079:[7->5,7->8] | 0.22 | 248.703703704 | 12 | 27 | 2 | 2 |
| Path 101 | C00031->C00079:[2->8,7->2,9->5] | 0.33 | 428.589041096 | 26 | 73 | 3 | 3 |
| Path 102 | C00031->C00079:[1->10,2->6,4->7,5->3,9->8] | 0.56 | 362.917808219 | 26 | 73 | 1 | 1 |
| Path 103 | C00031->C00079:[1->6,2->10,4->3,4->8,5->7] | 0.56 | 491.050632911 | 34 | 79 | 0 | 0 |
| Path 104 | C00031->C00079:[1->10,2->6,4->7,4->8,5->3] | 0.56 | 535.622222222 | 24 | 45 | 1 | 1 |
| Path 105 | C00031->C00079:[9->5,9->8] | 0.22 | 222.632653061 | 14 | 49 | 1 | 1 |
| Path 106 | C00031->C00079:[4->5,4->8] | 0.22 | 318.738095238 | 19 | 42 | 2 | 2 |
| Path 107 | C00031->C00079:[1->10,2->6,4->7,5->3,7->1,7->2,9->4,9->5,9->8] | 1.00 | 564.360655738 | 28 | 61 | 1 | 1 |
| Path 108 | C00031->C00079:[1->6,2->10,4->3,5->7] | 0.44 | 564.612903226 | 15 | 31 | 0 | 0 |
| Path 109 | C00031->C00079:[1->10,2->6,4->7,4->8,5->3,7->1,7->2,9->4,9->5] | 1.00 | 559.571428571 | 28 | 63 | 1 | 1 |
| Path 110 | C00031->C00079:[1->10,2->6,4->7,4->8,5->3,7->1,7->2,9->4,9->5] | 1.00 | 561.683333333 | 27 | 60 | 1 | 1 |
| Path 111 | C00031->C00079:[7->5,7->8] | 0.22 | 400.52238806 | 23 | 67 | 2 | 2 |
| Path 112 | C00031->C00079:[4->5,4->8] | 0.22 | 331.703703704 | 14 | 27 | 2 | 2 |
| Path 113 | C00031->C00079:[7->5,7->8] | 0.22 | 359.472222222 | 18 | 36 | 1 | 1 |
| Path 114 | C00031->C00079:[7->5,7->8] | 0.22 | 245.346153846 | 17 | 52 | 1 | 1 |
| Path 115 | C00031->C00079:[4->5,4->8] | 0.22 | 291.59375 | 23 | 64 | 2 | 2 |
| Path 116 | C00031->C00079:[4->5,4->8] | 0.22 | 364.224137931 | 24 | 58 | 2 | 2 |
| Path 117 | C00031->C00079:[4->5,4->8] | 0.22 | 368.323529412 | 29 | 102 | 2 | 2 |
| Path 118 | C00031->C00079:[1->6,2->10,4->3,4->8,5->7] | 0.56 | 527.85 | 32 | 80 | 0 | 0 |
| Path 119 | C00031->C00079:[1->6,2->10,4->3,4->8,5->7,7->2,7->4,9->1,9->5] | 1.00 | 494.675 | 35 | 80 | 0 | 0 |
| Path 120 | C00031->C00079:[2->8] | 0.11 | 412.964912281 | 15 | 57 | 1 | 1 |
| Path 121 | C00031->C00079:[4->5,4->8,7->5,7->8] | 0.22 | 387.468085106 | 25 | 47 | 2 | 2 |
| Path 122 | C00031->C00079:[4->5,4->8,7->5,7->8] | 0.22 | 319.636363636 | 15 | 33 | 1 | 1 |
| Path 123 | C00031->C00079:[4->5,4->8] | 0.22 | 306.860759494 | 27 | 79 | 2 | 2 |
| Path 124 | C00031->C00079:[1->6,2->10,4->3,4->8,5->7,7->2,7->4,9->1,9->5] | 1.00 | 530.975308642 | 33 | 81 | 0 | 0 |
| Path 125 | C00031->C00079:[7->5,7->8] | 0.22 | 313.170731707 | 20 | 41 | 2 | 2 |
| Path 126 | C00031->C00079:[4->5,4->8] | 0.22 | 364.024390244 | 20 | 41 | 2 | 2 |
| Path 127 | C00031->C00079:[5->8,7->2,9->5] | 0.33 | 366.259259259 | 22 | 54 | 3 | 3 |
| Path 128 | C00031->C00079:[9->5,9->8] | 0.22 | 340.347826087 | 26 | 92 | 2 | 2 |
| Path 129 | C00031->C00079:[4->5,4->8] | 0.22 | 337.488888889 | 20 | 45 | 1 | 1 |
| Path 130 | C00031->C00079:[1->6,2->10,4->3,4->8,5->7] | 0.56 | 549.555555556 | 25 | 54 | 0 | 0 |
| Path 131 | C00031->C00079:[4->5,4->8,7->5,7->8] | 0.22 | 338.425 | 19 | 40 | 2 | 2 |
| Path 132 | C00031->C00079:[1->10,2->6,4->7,5->3,7->1,7->2,7->8,9->4,9->5] | 1.00 | 566.375 | 26 | 56 | 1 | 1 |
| Path 133 | C00031->C00079:[4->5,4->8] | 0.22 | 260.287878788 | 23 | 66 | 2 | 2 |
| Path 134 | C00031->C00079:[4->5,4->8] | 0.22 | 424.588235294 | 24 | 68 | 2 | 2 |
| Path 135 | C00031->C00079:[7->2,9->5] | 0.22 | 336.5 | 12 | 40 | 2 | 2 |
| Path 136 | C00031->C00079:[4->5,4->8,7->5,7->8] | 0.22 | 327.027027027 | 17 | 37 | 2 | 2 |
| Path 137 | C00031->C00079:[7->2,9->5,9->8] | 0.33 | 248.652173913 | 21 | 69 | 3 | 3 |
| Path 138 | C00031->C00079:[4->5,4->8] | 0.22 | 284.65625 | 23 | 64 | 2 | 2 |
| Path 139 | C00031->C00079:[4->5,4->8] | 0.22 | 417.723684211 | 27 | 76 | 2 | 2 |
| Path 140 | C00031->C00079:[7->5,7->8] | 0.22 | 342.04 | 21 | 50 | 2 | 2 |
| Path 141 | C00031->C00079:[4->5,4->8] | 0.22 | 281.444444444 | 22 | 63 | 2 | 2 |
| Path 142 | C00031->C00079:[7->5,7->8] | 0.22 | 258.283018868 | 18 | 53 | 1 | 1 |
| Path 143 | C00031->C00079:[4->5,4->8] | 0.22 | 276.169491525 | 19 | 59 | 1 | 1 |
| Path 144 | C00031->C00079:[4->5,4->8] | 0.22 | 420.948051948 | 24 | 77 | 2 | 2 |
| Path 145 | C00031->C00079:[5->8] | 0.11 | 331.205128205 | 12 | 39 | 1 | 1 |
| Path 146 | C00031->C00079:[1->10,2->6,4->5,4->7,4->8,5->3,5->4,7->1,7->2,9->4] | 1.00 | 406.594059406 | 37 | 101 | 2 | 2 |
| Path 147 | C00031->C00079:[4->5,4->8,7->5,7->8] | 0.22 | 342.048780488 | 20 | 41 | 2 | 2 |
| Path 148 | C00031->C00079:[9->8] | 0.11 | 201.4 | 12 | 55 | 1 | 1 |
| Path 149 | C00031->C00079:[2->8] | 0.11 | 420.75862069 | 15 | 58 | 1 | 1 |
| Path 150 | C00031->C00079:[7->2,9->5,9->8] | 0.33 | 272.711864407 | 20 | 59 | 3 | 3 |
| Path 151 | C00031->C00079:[1->10,1->4,1->5,2->6,2->8,4->1,4->2,4->7,5->3] | 1.00 | 520.75 | 28 | 80 | 1 | 1 |
| Path 152 | C00031->C00079:[4->5,4->8] | 0.22 | 383.186046512 | 20 | 43 | 1 | 1 |
| Path 153 | C00031->C00079:[1->10,2->6,4->7,5->3,7->1,7->2,9->4,9->5,9->8] | 1.00 | 444.714285714 | 27 | 77 | 1 | 1 |
| Path 154 | C00031->C00079:[4->5,4->8] | 0.22 | 274.964912281 | 19 | 57 | 1 | 1 |
| Path 155 | C00031->C00079:[4->5,4->8] | 0.22 | 258.6875 | 20 | 64 | 1 | 1 |
| Path 156 | C00031->C00079:[7->2,9->5,9->8] | 0.33 | 378.441860465 | 21 | 43 | 3 | 3 |
| Path 157 | C00031->C00079:[7->5,7->8] | 0.22 | 258.269230769 | 11 | 26 | 1 | 1 |
| Path 158 | C00031->C00079:[5->5,5->8,9->5,9->8] | 0.22 | 274.956521739 | 21 | 69 | 2 | 2 |
| Path 159 | C00031->C00079:[4->5,4->8] | 0.22 | 424.328767123 | 21 | 73 | 1 | 1 |
| Path 160 | C00031->C00079:[2->8] | 0.11 | 426.188679245 | 15 | 53 | 1 | 1 |
| Path 161 | C00031->C00079:[4->5,4->8,7->5,7->8] | 0.22 | 286.158730159 | 24 | 63 | 2 | 2 |
| Path 162 | C00031->C00079:[1->6,2->10,4->3,4->8,5->7,7->2,7->4,9->1,9->5] | 1.00 | 515.369047619 | 34 | 84 | 0 | 0 |
| Path 163 | C00031->C00079:[4->5,4->8] | 0.22 | 258.631578947 | 19 | 57 | 2 | 2 |
| Path 164 | C00031->C00079:[1->10,1->4,1->5,2->6,2->8,4->1,4->2,4->7,5->3] | 1.00 | 508.4125 | 31 | 80 | 1 | 1 |
| Path 165 | C00031->C00079:[4->5,4->8] | 0.22 | 329.422222222 | 21 | 45 | 2 | 2 |
| Path 166 | C00031->C00079:[4->5,4->8] | 0.22 | 321.307692308 | 14 | 26 | 2 | 2 |
| Path 167 | C00031->C00079:[4->5,4->8] | 0.22 | 270.224137931 | 20 | 58 | 2 | 2 |
| Path 168 | C00031->C00079:[7->5,7->8] | 0.22 | 219.444444444 | 19 | 63 | 2 | 2 |
| Path 169 | C00031->C00079:[4->5,4->8] | 0.22 | 374.477272727 | 21 | 44 | 2 | 2 |
| Path 170 | C00031->C00079:[7->5,7->8] | 0.22 | 396.84375 | 21 | 64 | 2 | 2 |
| Path 171 | C00031->C00079:[4->5,4->8] | 0.22 | 338.76 | 12 | 25 | 1 | 1 |
| Path 172 | C00031->C00079:[4->5,4->8,7->5,7->8] | 0.22 | 310.235294118 | 16 | 34 | 2 | 2 |
| Path 173 | C00031->C00079:[1->6,2->10,4->3,4->8,5->7,7->2,7->4,9->1,9->5] | 1.00 | 444.745098039 | 35 | 102 | 0 | 0 |
| Path 174 | C00031->C00079:[4->5,4->8] | 0.22 | 280.491803279 | 22 | 61 | 2 | 2 |
| Path 175 | C00031->C00079:[4->5,4->8] | 0.22 | 420.307692308 | 23 | 65 | 2 | 2 |
| Path 176 | C00031->C00079:[4->5,4->8] | 0.22 | 423.532467532 | 27 | 77 | 2 | 2 |
| Path 177 | C00031->C00079:[4->8,7->2,9->5] | 0.33 | 380.0 | 21 | 45 | 3 | 3 |
| Path 178 | C00031->C00079:[1->10,1->4,1->5,2->6,2->8,4->1,4->2,4->7,5->3] | 1.00 | 514.972972973 | 28 | 74 | 1 | 1 |
| Path 179 | C00031->C00079:[2->8,7->2,9->5] | 0.33 | 417.116883117 | 27 | 77 | 3 | 3 |
| Path 180 | C00031->C00079:[4->5,4->8] | 0.22 | 296.717391304 | 19 | 46 | 1 | 1 |
| Path 181 | C00031->C00079:[4->5,4->8] | 0.22 | 383.354166667 | 24 | 48 | 2 | 2 |
| Path 182 | C00031->C00079:[1->6,2->10,4->3,4->8,5->7,7->2,7->4,9->1,9->5] | 1.00 | 415.138613861 | 37 | 101 | 0 | 0 |
| Path 183 | C00031->C00079:[4->5,4->8] | 0.22 | 373.125 | 19 | 40 | 1 | 1 |
| Path 184 | C00031->C00079:[4->5,4->8,7->5,7->8] | 0.22 | 301.512195122 | 17 | 41 | 1 | 1 |
| Path 185 | C00031->C00079:[9->5,9->8] | 0.22 | 346.125 | 24 | 88 | 2 | 2 |
| Path 186 | C00031->C00079:[4->5,4->8] | 0.22 | 346.433333333 | 16 | 30 | 2 | 2 |
| Path 187 | C00031->C00079:[9->5,9->8] | 0.22 | 218.18 | 15 | 50 | 2 | 2 |
| Path 188 | C00031->C00079:[4->5,4->8] | 0.22 | 297.338709677 | 23 | 62 | 2 | 2 |
| Path 189 | C00031->C00079:[4->5,4->8] | 0.22 | 422.441558442 | 23 | 77 | 2 | 2 |
| Path 190 | C00031->C00079:[2->8] | 0.11 | 423.290322581 | 14 | 62 | 1 | 1 |
| Path 191 | C00031->C00079:[1->6,2->10,4->3,4->8,5->7,7->2,7->4,9->1,9->5] | 1.00 | 515.436781609 | 35 | 87 | 0 | 0 |
| Path 192 | C00031->C00079:[7->5,7->8] | 0.22 | 240.716981132 | 18 | 53 | 2 | 2 |
| Path 193 | C00031->C00079:[1->10,2->6,4->7,5->3,9->8] | 0.56 | 501.479166667 | 25 | 48 | 1 | 1 |
| Path 194 | C00031->C00079:[4->5,4->8] | 0.22 | 265.888888889 | 12 | 27 | 1 | 1 |
| Path 195 | C00031->C00079:[1->10,2->6,4->5,4->7,4->8,5->3,5->4,7->1,7->2,9->4] | 1.00 | 530.464788732 | 34 | 71 | 2 | 2 |
| Path 196 | C00031->C00079:[1->6,2->10,4->3,5->7,7->2,7->4,7->8,9->1,9->5] | 1.00 | 532.792207792 | 32 | 77 | 0 | 0 |
| Path 197 | C00031->C00079:[7->5,7->8] | 0.22 | 265.368421053 | 21 | 57 | 2 | 2 |
| Path 198 | C00031->C00079:[1->10,2->6,4->7,5->3,9->8] | 0.56 | 411.828125 | 26 | 64 | 1 | 1 |
| Path 199 | C00031->C00079:[1->6,2->10,4->3,4->8,5->7,7->2,7->4,9->1,9->5] | 1.00 | 530.488095238 | 34 | 84 | 0 | 0 |
| Path 200 | C00031->C00079:[4->5,4->8] | 0.22 | 416.228571429 | 23 | 70 | 2 | 2 |
| Path 201 | C00031->C00079:[4->8] | 0.11 | 360.476190476 | 9 | 21 | 1 | 1 |
| Path 202 | C00031->C00079:[4->5,4->8] | 0.22 | 416.8 | 26 | 75 | 2 | 2 |
| Path 203 | C00031->C00079:[4->5,4->8] | 0.22 | 412.611111111 | 25 | 72 | 2 | 2 |
| Path 204 | C00031->C00079:[7->8] | 0.11 | 264.363636364 | 8 | 22 | 1 | 1 |
| Path 205 | C00031->C00079:[4->5,4->8] | 0.22 | 426.887323944 | 26 | 71 | 2 | 2 |
| Path 206 | C00031->C00079:[4->5,4->8] | 0.22 | 245.967213115 | 19 | 61 | 1 | 1 |
| Path 207 | C00031->C00079:[1->10,2->6,4->5,4->7,4->8,5->3,5->4,7->1,7->2,9->4] | 1.00 | 448.505376344 | 38 | 93 | 2 | 2 |
| Path 208 | C00031->C00079:[4->5,4->8,7->5,7->8] | 0.22 | 296.234375 | 25 | 64 | 2 | 2 |
| Path 209 | C00031->C00079:[4->5,4->8] | 0.22 | 263.25 | 18 | 56 | 1 | 1 |
| Path 210 | C00031->C00079:[4->5,4->8] | 0.22 | 341.586206897 | 15 | 29 | 2 | 2 |
| Path 211 | C00031->C00079:[4->5,4->8] | 0.22 | 326.512195122 | 18 | 41 | 1 | 1 |
| Path 212 | C00031->C00079:[4->5,4->8] | 0.22 | 355.574074074 | 21 | 54 | 2 | 2 |
| Path 213 | C00031->C00079:[9->8] | 0.11 | 237.847826087 | 12 | 46 | 1 | 1 |
| Path 214 | C00031->C00079:[4->5,4->8] | 0.22 | 364.806122449 | 31 | 98 | 2 | 2 |
| Path 215 | C00031->C00079:[4->5,4->8] | 0.22 | 300.739130435 | 12 | 23 | 2 | 2 |
| Path 216 | C00031->C00079:[1->6,2->10,4->3,4->8,5->7] | 0.56 | 527.469879518 | 33 | 83 | 0 | 0 |
| Path 217 | C00031->C00079:[4->5,4->8] | 0.22 | 427.722222222 | 27 | 72 | 2 | 2 |
| Path 218 | C00031->C00079:[1->10,2->6,4->5,4->7,4->8,5->3,5->4,7->1,7->2,9->4] | 1.00 | 531.701492537 | 32 | 67 | 2 | 2 |
| Path 219 | C00031->C00079:[4->5,4->8,7->5,7->8] | 0.22 | 367.280701754 | 25 | 57 | 2 | 2 |
| Path 220 | C00031->C00079:[4->5,4->8] | 0.22 | 362.070175439 | 23 | 57 | 2 | 2 |
| Path 221 | C00031->C00079:[1->6,2->10,4->2,4->3,4->4,4->8,5->7] | 0.78 | 289.291666667 | 28 | 120 | 0 | 0 |
| Path 222 | C00031->C00079:[4->5,4->8] | 0.22 | 424.5625 | 25 | 80 | 2 | 2 |
| Path 223 | C00031->C00079:[1->10,1->4,1->5,2->6,2->8,4->1,4->2,4->7,5->3] | 1.00 | 524.236842105 | 30 | 76 | 1 | 1 |
| Path 224 | C00031->C00079:[7->2,7->8,9->5] | 0.33 | 356.947368421 | 19 | 38 | 3 | 3 |
| Path 225 | C00031->C00079:[1->10,2->6,4->5,4->7,4->8,5->3,5->4,7->1,7->2,9->4] | 1.00 | 535.76 | 37 | 75 | 2 | 2 |
| Path 226 | C00031->C00079:[9->5,9->8] | 0.22 | 212.660714286 | 17 | 56 | 2 | 2 |
| Path 227 | C00031->C00079:[4->5,4->8] | 0.22 | 287.083333333 | 20 | 60 | 1 | 1 |
| Path 228 | C00031->C00079:[4->5,4->8] | 0.22 | 344.142857143 | 23 | 49 | 2 | 2 |
| Path 229 | C00031->C00079:[1->6,2->10,4->2,4->3,4->4,4->8,5->7] | 0.78 | 281.689655172 | 26 | 116 | 0 | 0 |
| Path 230 | C00031->C00079:[4->5,4->8] | 0.22 | 381.14893617 | 23 | 47 | 2 | 2 |
| Path 231 | C00031->C00079:[4->5,4->8,7->5,7->8] | 0.22 | 409.974683544 | 29 | 79 | 2 | 2 |
| Path 232 | C00031->C00079:[2->8,7->2,9->5] | 0.33 | 428.287671233 | 25 | 73 | 3 | 3 |
| Path 233 | C00031->C00079:[4->5,4->8] | 0.22 | 235.955223881 | 20 | 67 | 2 | 2 |
| Path 234 | C00031->C00079:[4->5,4->8] | 0.22 | 331.977777778 | 20 | 45 | 2 | 2 |
| Path 235 | C00031->C00079:[4->8,7->2,9->5] | 0.33 | 367.936170213 | 16 | 47 | 2 | 2 |
| Path 236 | C00031->C00079:[4->5,4->8,7->5,7->8] | 0.22 | 347.354166667 | 24 | 48 | 2 | 2 |
| Path 237 | C00031->C00079:[4->5,4->8] | 0.22 | 288.564516129 | 23 | 62 | 2 | 2 |
| Path 238 | C00031->C00079:[4->5,4->8] | 0.22 | 344.461538462 | 13 | 26 | 1 | 1 |
| Path 239 | C00031->C00079:[4->5,4->8] | 0.22 | 282.37704918 | 21 | 61 | 2 | 2 |
| Path 240 | C00031->C00079:[4->5,4->8] | 0.22 | 358.647058824 | 32 | 102 | 2 | 2 |
| Path 241 | C00031->C00079:[4->5,4->8] | 0.22 | 353.444444444 | 22 | 54 | 2 | 2 |
| Path 242 | C00031->C00079:[4->5,4->8,7->5,7->8] | 0.22 | 284.473684211 | 16 | 38 | 1 | 1 |
