# Supplementary material for: 13C-metabolic flux ratio and novel carbon path analyses confirmed that Trichoderma reesei uses primarily the respirative pathway also on the preferred carbon source glucose
Source: BMC Syst Biol. 2009 Oct 29;3:104. doi: 10.1186/1752-0509-3-104 (PMC2776023; doi:10.1186/1752-0509-3-104)
Supplement: Additional file 1 — Pathways discovered in ReTrace carbon path analysis. Graphical and tabular representations of amino acid synthesis pathways discovered in ReTrace carbon path analysis [21]. Self-contained web site: unpack zip archive and open index.html with a web browser. [file 1752-0509-3-104-S1.zip › AF1-treesei/pathways-C00031-to-C00135.html]

Pathways from C00031 to C00135


**Pathways from C00031 to C00135**

**Sources:** D-Glucose; (C00031)

**Target:**L-Histidine; (C00135)

|  | Composite mapping | Z | Average score | Rpairs | Reactions | Zero scores | Scores under threshold |
| --- | --- | --- | --- | --- | --- | --- | --- |
| Path 1 | C00031->C00135:[1->5,2->2,4->7,4->8,5->1,5->3] | 1.00 | 373.717105263 | 31 | 152 | 1 | 1 |
| Path 2 | C00031->C00135:[1->5,2->2,4->7,4->8,5->1,9->3] | 1.00 | 347.401360544 | 25 | 147 | 1 | 1 |
| Path 3 | C00031->C00135:[1->5,2->2,4->7,4->8,5->1,7->3] | 1.00 | 370.383233533 | 35 | 167 | 1 | 1 |
| Path 4 | C00031->C00135:[1->5,2->2,4->7,4->8,5->1,7->3] | 1.00 | 379.436363636 | 35 | 165 | 1 | 1 |
| Path 5 | C00031->C00135:[1->5,2->2,4->7,4->8,5->1,5->3] | 1.00 | 367.25 | 27 | 152 | 1 | 1 |
| Path 6 | C00031->C00135:[1->5,2->2,4->7,4->8,5->1,9->3] | 1.00 | 345.763513514 | 26 | 148 | 1 | 1 |
| Path 7 | C00031->C00135:[1->5,2->2,4->7,4->8,5->1,5->3] | 1.00 | 371.791139241 | 31 | 158 | 1 | 1 |
| Path 8 | C00031->C00135:[1->5,2->2,2->3,4->8,5->1,7->7] | 1.00 | 378.208860759 | 29 | 158 | 1 | 1 |
| Path 9 | C00031->C00135:[1->5,2->2,4->7,4->8,5->1,5->3] | 1.00 | 360.2125 | 30 | 160 | 1 | 1 |
| Path 10 | C00031->C00135:[1->5,2->2,4->7,4->8,5->1,9->3] | 1.00 | 346.460526316 | 26 | 152 | 1 | 1 |
| Path 11 | C00031->C00135:[1->5,2->2,4->7,4->8,5->1,7->3] | 1.00 | 383.701863354 | 34 | 161 | 1 | 1 |
| Path 12 | C00031->C00135:[1->5,2->2,4->7,4->8,5->1,7->3] | 1.00 | 375.211180124 | 32 | 161 | 1 | 1 |
| Path 13 | C00031->C00135:[1->5,2->2,4->7,4->8,5->1,5->3] | 1.00 | 372.487179487 | 29 | 156 | 1 | 1 |
| Path 14 | C00031->C00135:[1->5,2->2,4->8,5->1,7->7,9->3] | 1.00 | 343.520833333 | 23 | 144 | 1 | 1 |
| Path 15 | C00031->C00135:[1->5,2->2,4->8,5->1,7->7,9->3] | 1.00 | 343.244755245 | 24 | 143 | 1 | 1 |
| Path 16 | C00031->C00135:[1->5,2->2,4->7,4->8,5->1,5->3] | 1.00 | 381.068322981 | 29 | 161 | 1 | 1 |
| Path 17 | C00031->C00135:[1->5,2->2,4->7,4->8,5->1,5->3] | 1.00 | 376.006329114 | 30 | 158 | 1 | 1 |
| Path 18 | C00031->C00135:[1->5,2->2,4->7,4->8,5->1,5->3] | 1.00 | 371.195945946 | 28 | 148 | 1 | 1 |
| Path 19 | C00031->C00135:[1->5,2->2,4->7,4->8,5->1,5->3] | 1.00 | 383.56097561 | 30 | 164 | 1 | 1 |
| Path 20 | C00031->C00135:[1->5,2->2,4->7,4->8,5->1,9->3] | 1.00 | 338.746666667 | 25 | 150 | 1 | 1 |
| Path 21 | C00031->C00135:[1->5,2->2,4->7,4->8,5->1,5->3] | 1.00 | 377.988023952 | 36 | 167 | 1 | 1 |
| Path 22 | C00031->C00135:[1->5,2->2,4->7,4->8,5->1,9->3] | 1.00 | 348.026666667 | 26 | 150 | 2 | 2 |
| Path 23 | C00031->C00135:[1->5,2->2,4->7,4->8,5->1,5->3] | 1.00 | 373.48427673 | 32 | 159 | 1 | 1 |
| Path 24 | C00031->C00135:[1->5,2->2,4->7,4->8,5->1,5->3] | 1.00 | 382.14375 | 32 | 160 | 1 | 1 |
| Path 25 | C00031->C00135:[1->5,2->2,4->7,4->8,5->1,7->3] | 1.00 | 376.679245283 | 31 | 159 | 1 | 1 |
| Path 26 | C00031->C00135:[1->5,2->2,4->7,4->8,5->1,7->3] | 1.00 | 382.380368098 | 34 | 163 | 1 | 1 |
| Path 27 | C00031->C00135:[1->5,2->2,4->7,4->8,5->1,5->3] | 1.00 | 376.756578947 | 30 | 152 | 1 | 1 |
| Path 28 | C00031->C00135:[1->5,2->2,4->7,4->8,5->1,5->3] | 1.00 | 369.537974684 | 30 | 158 | 1 | 1 |
| Path 29 | C00031->C00135:[1->5,2->2,4->8,5->1,7->3,7->7] | 1.00 | 373.6 | 30 | 155 | 1 | 1 |
| Path 30 | C00031->C00135:[1->5,2->2,4->7,4->8,5->1,9->3] | 1.00 | 350.8 | 26 | 150 | 1 | 1 |
| Path 31 | C00031->C00135:[1->5,2->2,4->7,4->8,5->1,5->3] | 1.00 | 379.192771084 | 30 | 166 | 1 | 1 |
| Path 32 | C00031->C00135:[1->5,2->2,4->7,4->8,5->1,5->3] | 1.00 | 373.74025974 | 29 | 154 | 1 | 1 |
| Path 33 | C00031->C00135:[1->5,2->2,4->7,4->8,5->1,9->3] | 1.00 | 408.760869565 | 25 | 46 | 1 | 1 |
| Path 34 | C00031->C00135:[1->5,2->2,2->3,4->7,4->8,5->1] | 1.00 | 379.62195122 | 31 | 164 | 1 | 1 |
| Path 35 | C00031->C00135:[1->5,2->2,4->7,4->8,5->1,5->3] | 1.00 | 382.194117647 | 35 | 170 | 1 | 1 |
| Path 36 | C00031->C00135:[1->5,2->2,4->8,5->1,9->3,9->7] | 1.00 | 317.521212121 | 26 | 165 | 1 | 1 |
| Path 37 | C00031->C00135:[1->5,2->2,4->7,4->8,5->1,5->3] | 1.00 | 377.890243902 | 33 | 164 | 1 | 1 |
| Path 38 | C00031->C00135:[1->5,2->2,4->7,4->8,5->1,5->3] | 1.00 | 367.111111111 | 30 | 153 | 1 | 1 |
| Path 39 | C00031->C00135:[1->5,2->2,2->3,4->7,4->8,5->1] | 1.00 | 381.117283951 | 30 | 162 | 1 | 1 |
| Path 40 | C00031->C00135:[1->5,2->2,4->8,5->1,5->3,7->7] | 1.00 | 363.77027027 | 26 | 148 | 1 | 1 |
| Path 41 | C00031->C00135:[1->5,2->2,4->8,5->1,5->3,7->7] | 1.00 | 378.140127389 | 28 | 157 | 1 | 1 |
| Path 42 | C00031->C00135:[1->5,2->2,4->8,5->1,5->3] | 0.83 | 556.8 | 20 | 30 | 1 | 1 |
| Path 43 | C00031->C00135:[1->5,2->2,4->8,5->1,5->3] | 0.83 | 517.953488372 | 27 | 43 | 1 | 1 |
| Path 44 | C00031->C00135:[1->5,2->2,4->7,4->8] | 0.67 | 370.64556962 | 29 | 158 | 2 | 2 |
| Path 45 | C00031->C00135:[4->7] | 0.17 | 346.380597015 | 19 | 134 | 1 | 1 |
| Path 46 | C00031->C00135:[1->5,2->2,4->8,7->7] | 0.67 | 367.38961039 | 28 | 154 | 2 | 2 |
| Path 47 | C00031->C00135:[1->5,2->2,4->8,5->1,7->3] | 0.83 | 557.642857143 | 27 | 42 | 1 | 1 |
| Path 48 | C00031->C00135:[7->3] | 0.17 | 525.090909091 | 21 | 33 | 1 | 1 |
| Path 49 | C00031->C00135:[1->5,2->2,4->7,4->8,5->1] | 0.83 | 371.653846154 | 28 | 156 | 1 | 1 |
| Path 50 | C00031->C00135:[4->8] | 0.17 | 414.230769231 | 8 | 13 | 3 | 3 |
| Path 51 | C00031->C00135:[1->5,2->2,4->7,4->8,5->1] | 0.83 | 367.215189873 | 28 | 158 | 1 | 1 |
| Path 52 | C00031->C00135:[1->5,2->2,4->8,5->1,5->3] | 0.83 | 526.205882353 | 22 | 34 | 1 | 1 |
| Path 53 | C00031->C00135:[2->3,7->7] | 0.33 | 372.083333333 | 28 | 156 | 1 | 1 |
| Path 54 | C00031->C00135:[1->5,2->2,4->8,5->1] | 0.67 | 519.962962963 | 17 | 27 | 1 | 1 |
| Path 55 | C00031->C00135:[1->5,2->2,4->8,5->1,7->3] | 0.83 | 528.567567568 | 23 | 37 | 1 | 1 |
| Path 56 | C00031->C00135:[1->5,2->2,4->8,5->1,7->3] | 0.83 | 544.0 | 22 | 35 | 1 | 1 |
| Path 57 | C00031->C00135:[1->5,2->2,4->7,4->8,5->1] | 0.83 | 363.516556291 | 26 | 151 | 1 | 1 |
| Path 58 | C00031->C00135:[4->8] | 0.17 | 383.3 | 7 | 10 | 3 | 3 |
| Path 59 | C00031->C00135:[1->5,2->2,4->8,5->1,5->3] | 0.83 | 466.166666667 | 21 | 36 | 1 | 1 |
| Path 60 | C00031->C00135:[1->5,2->2,4->8,5->1,7->3] | 0.83 | 530.609756098 | 26 | 41 | 1 | 1 |
| Path 61 | C00031->C00135:[2->3,4->7] | 0.33 | 375.18125 | 29 | 160 | 1 | 1 |
| Path 62 | C00031->C00135:[5->1,5->2,5->3,7->8,9->5] | 0.83 | 543.555555556 | 27 | 45 | 1 | 1 |
| Path 63 | C00031->C00135:[1->5,2->2,4->8,5->1] | 0.67 | 537.034482759 | 18 | 29 | 1 | 1 |
| Path 64 | C00031->C00135:[1->5,2->2,4->8,5->1,5->3] | 0.83 | 592.258064516 | 23 | 31 | 1 | 1 |
| Path 65 | C00031->C00135:[7->8] | 0.17 | 259.357142857 | 7 | 14 | 3 | 3 |
| Path 66 | C00031->C00135:[7->7] | 0.17 | 334.876811594 | 19 | 138 | 1 | 1 |
| Path 67 | C00031->C00135:[1->5,2->2,4->8,5->1,5->3] | 0.83 | 524.369565217 | 26 | 46 | 1 | 1 |
| Path 68 | C00031->C00135:[1->5,2->2,4->8,5->1,5->3] | 0.83 | 545.794117647 | 21 | 34 | 1 | 1 |
| Path 69 | C00031->C00135:[1->5,2->2,4->8,5->1,5->3] | 0.83 | 569.75 | 22 | 28 | 1 | 1 |
| Path 70 | C00031->C00135:[1->5,2->2,4->7,4->8,5->1] | 0.83 | 368.797385621 | 27 | 153 | 1 | 1 |
| Path 71 | C00031->C00135:[1->5,2->2,4->8,5->1,5->3] | 0.83 | 534.642857143 | 18 | 28 | 1 | 1 |
| Path 72 | C00031->C00135:[1->5,2->2,4->8,5->1,7->7] | 0.83 | 359.911564626 | 25 | 147 | 1 | 1 |
| Path 73 | C00031->C00135:[1->5,2->2,2->3,4->8,5->1] | 0.83 | 549.710526316 | 21 | 38 | 1 | 1 |
| Path 74 | C00031->C00135:[1->5,2->2,4->8,5->1,5->3] | 0.83 | 528.137931034 | 21 | 29 | 1 | 1 |
| Path 75 | C00031->C00135:[1->5,2->2,4->8,5->1,7->3] | 0.83 | 488.418604651 | 26 | 43 | 1 | 1 |
| Path 76 | C00031->C00135:[1->5,2->2,4->8,5->1,5->3,7->7] | 1.00 | 360.646258503 | 26 | 147 | 1 | 1 |
| Path 77 | C00031->C00135:[1->5,2->2,4->8,5->1,7->7] | 0.83 | 365.382550336 | 26 | 149 | 1 | 1 |
| Path 78 | C00031->C00135:[4->7,7->3] | 0.33 | 370.573248408 | 30 | 157 | 1 | 1 |
| Path 79 | C00031->C00135:[1->5,2->2,4->8,5->1,5->3] | 0.83 | 563.638888889 | 23 | 36 | 1 | 1 |
| Path 80 | C00031->C00135:[1->5,2->2,4->8,5->1,5->3] | 0.83 | 528.05 | 24 | 40 | 1 | 1 |
| Path 81 | C00031->C00135:[1->5,2->2,4->8,5->1,9->3] | 0.83 | 444.173913043 | 16 | 23 | 1 | 1 |
| Path 82 | C00031->C00135:[1->5,2->2,4->8,5->1,5->3] | 0.83 | 529.485714286 | 23 | 35 | 1 | 1 |
| Path 83 | C00031->C00135:[1->5,2->2,4->8,5->1,5->3,7->7] | 1.00 | 360.098684211 | 28 | 152 | 1 | 1 |
| Path 84 | C00031->C00135:[1->5,2->2,4->7,4->8,5->1,5->3] | 1.00 | 367.684210526 | 28 | 152 | 1 | 1 |
| Path 85 | C00031->C00135:[1->5,2->2,2->3,4->8,5->1] | 0.83 | 535.15 | 22 | 40 | 1 | 1 |
| Path 86 | C00031->C00135:[1->5,2->2,4->8,5->1,5->3] | 0.83 | 548.6 | 22 | 35 | 1 | 1 |
| Path 87 | C00031->C00135:[7->3,7->7] | 0.33 | 367.294117647 | 29 | 153 | 1 | 1 |
| Path 88 | C00031->C00135:[1->5,2->2,4->8,5->1,5->3] | 0.83 | 515.735294118 | 21 | 34 | 1 | 1 |
| Path 89 | C00031->C00135:[1->5,2->2,4->8,5->1,7->3] | 0.83 | 565.513513514 | 25 | 37 | 1 | 1 |
| Path 90 | C00031->C00135:[1->5,2->2,4->8,5->1,5->3] | 0.83 | 586.875 | 19 | 24 | 1 | 1 |
| Path 91 | C00031->C00135:[1->5,2->2,4->8,5->1,5->3] | 0.83 | 554.054054054 | 20 | 37 | 1 | 1 |
