# Supplementary material for: 13C-metabolic flux ratio and novel carbon path analyses confirmed that Trichoderma reesei uses primarily the respirative pathway also on the preferred carbon source glucose
Source: BMC Syst Biol. 2009 Oct 29;3:104. doi: 10.1186/1752-0509-3-104 (PMC2776023; doi:10.1186/1752-0509-3-104)
Supplement: Additional file 1 — Pathways discovered in ReTrace carbon path analysis. Graphical and tabular representations of amino acid synthesis pathways discovered in ReTrace carbon path analysis [21]. Self-contained web site: unpack zip archive and open index.html with a web browser. [file 1752-0509-3-104-S1.zip › AF1-treesei/pathways-C00024-C00026-to-C00047.html]

Pathways from C00024,C00026 to C00047


**Pathways from C00024-C00026 to C00047**

**Sources:** Acetyl-CoA; (C00024)
2-Oxoglutarate; (C00026)

**Target:**L-Lysine; (C00047)

|  | Composite mapping | Z | Average score | Rpairs | Reactions | Zero scores | Scores under threshold |
| --- | --- | --- | --- | --- | --- | --- | --- |
| Path 1 | C00026->C00047:[3->3,3->9], C00024->C00047:[49->1,50->2] | 0.67 | 269.534653465 | 20 | 101 | 0 | 0 |
| Path 2 | C00026->C00047:[3->3,3->9], C00024->C00047:[49->1,49->3,49->9,50->2] | 0.67 | 229.042553191 | 15 | 94 | 0 | 0 |
| Path 3 | C00026->C00047:[3->3,3->9], C00024->C00047:[49->1,50->2] | 0.67 | 270.889952153 | 20 | 209 | 0 | 0 |
| Path 4 | C00026->C00047:[3->3,3->9], C00024->C00047:[49->1,50->2] | 0.67 | 278.092592593 | 17 | 162 | 0 | 0 |
| Path 5 | C00026->C00047:[3->3,3->9], C00024->C00047:[49->1,50->2] | 0.67 | 433.551020408 | 19 | 98 | 0 | 0 |
| Path 6 | C00024->C00047:[49->1,49->3,49->9,50->2] | 0.67 | 616.964285714 | 16 | 28 | 0 | 0 |
| Path 7 | C00026->C00047:[1->3,1->9], C00024->C00047:[49->1,50->2] | 0.67 | 251.46 | 19 | 100 | 0 | 0 |
| Path 8 | C00026->C00047:[3->3,3->9], C00024->C00047:[49->1,50->2] | 0.67 | 203.95505618 | 13 | 89 | 0 | 0 |
| Path 9 | C00026->C00047:[3->3,3->9], C00024->C00047:[49->1,50->2] | 0.67 | 239.494845361 | 17 | 97 | 0 | 0 |
| Path 10 | C00026->C00047:[3->3,3->9], C00024->C00047:[49->1,50->2] | 0.67 | 214.395604396 | 13 | 91 | 0 | 0 |
| Path 11 | C00026->C00047:[3->3,3->9], C00024->C00047:[49->1,49->3,49->9,50->2] | 0.67 | 389.901960784 | 18 | 102 | 0 | 0 |
| Path 12 | C00024->C00047:[49->1,50->2,50->3,50->9] | 0.67 | 605.5 | 20 | 34 | 0 | 0 |
| Path 13 | C00026->C00047:[3->3,3->9], C00024->C00047:[49->1,50->2] | 0.67 | 356.468599034 | 22 | 207 | 0 | 0 |
| Path 14 | C00024->C00047:[49->1,49->3,49->9,50->2] | 0.67 | 649.454545455 | 15 | 22 | 0 | 0 |
| Path 15 | C00026->C00047:[3->3,3->9], C00024->C00047:[49->5,50->8] | 0.67 | 189.136363636 | 12 | 88 | 0 | 0 |
| Path 16 | C00026->C00047:[3->3,3->9], C00024->C00047:[49->1,50->2] | 0.67 | 274.961904762 | 22 | 210 | 0 | 0 |
| Path 17 | C00024->C00047:[49->1,49->3,49->9,50->2] | 0.67 | 508.974358974 | 18 | 39 | 0 | 0 |
| Path 18 | C00026->C00047:[3->3,3->9], C00024->C00047:[49->5,50->8] | 0.67 | 272.826086957 | 15 | 161 | 0 | 0 |
| Path 19 | C00026->C00047:[3->3,3->9], C00024->C00047:[49->1,50->2] | 0.67 | 266.946859903 | 20 | 207 | 0 | 0 |
| Path 20 | C00026->C00047:[3->3,3->9], C00024->C00047:[49->1,50->2] | 0.67 | 203.382352941 | 14 | 102 | 0 | 0 |
| Path 21 | C00026->C00047:[3->3,3->9], C00024->C00047:[49->1,50->2] | 0.67 | 268.79 | 19 | 100 | 0 | 0 |
| Path 22 | C00026->C00047:[3->3,3->9], C00024->C00047:[49->1,49->3,49->9,50->2] | 0.67 | 382.613861386 | 16 | 101 | 0 | 0 |
| Path 23 | C00026->C00047:[3->3,3->9], C00024->C00047:[49->1,50->2] | 0.67 | 216.048543689 | 15 | 103 | 0 | 0 |
| Path 24 | C00026->C00047:[3->3,3->9], C00024->C00047:[49->1,50->2] | 0.67 | 277.683229814 | 16 | 161 | 0 | 0 |
| Path 25 | C00024->C00047:[49->1,49->3,49->9,50->2] | 0.67 | 344.530201342 | 21 | 149 | 0 | 0 |
| Path 26 | C00026->C00047:[3->3,3->9], C00024->C00047:[49->5,50->8] | 0.67 | 222.989010989 | 14 | 91 | 0 | 0 |
| Path 27 | C00026->C00047:[3->3,3->9], C00024->C00047:[49->5,50->8] | 0.67 | 224.304347826 | 15 | 92 | 0 | 0 |
| Path 28 | C00024->C00047:[49->1,50->2,50->3,50->9] | 0.67 | 527.176470588 | 18 | 34 | 0 | 0 |
| Path 29 | C00026->C00047:[3->3,3->9], C00024->C00047:[49->1,50->2] | 0.67 | 208.225490196 | 16 | 102 | 0 | 0 |
| Path 30 | C00026->C00047:[3->3,3->9], C00024->C00047:[49->1,50->2] | 0.67 | 222.989010989 | 14 | 91 | 0 | 0 |
| Path 31 | C00026->C00047:[3->3,3->9], C00024->C00047:[49->1,50->2] | 0.67 | 217.278846154 | 16 | 104 | 0 | 0 |
| Path 32 | C00026->C00047:[3->3,3->9], C00024->C00047:[49->5,50->8] | 0.67 | 212.955555556 | 12 | 90 | 0 | 0 |
| Path 33 | C00026->C00047:[3->3,3->9], C00024->C00047:[49->1,49->3,49->9,50->2] | 0.67 | 213.891304348 | 13 | 92 | 0 | 0 |
| Path 34 | C00026->C00047:[3->3,3->9], C00024->C00047:[49->1,50->2] | 0.67 | 352.690821256 | 21 | 207 | 0 | 0 |
| Path 35 | C00026->C00047:[3->3,3->9], C00024->C00047:[49->1,50->2] | 0.67 | 412.168539326 | 15 | 89 | 0 | 0 |
| Path 36 | C00024->C00047:[49->1,49->3,49->9,50->2] | 0.67 | 663.633333333 | 19 | 30 | 0 | 0 |
| Path 37 | C00024->C00047:[49->1,50->2,50->3,50->9] | 0.67 | 644.851851852 | 18 | 27 | 0 | 0 |
| Path 38 | C00026->C00047:[3->3,3->9], C00024->C00047:[49->1,49->3,49->9,50->2] | 0.67 | 412.184782609 | 16 | 92 | 0 | 0 |
| Path 39 | C00026->C00047:[3->3,3->9], C00024->C00047:[49->1,50->2] | 0.67 | 247.572916667 | 16 | 96 | 0 | 0 |
| Path 40 | C00026->C00047:[3->3,3->9], C00024->C00047:[49->1,50->2] | 0.67 | 238.393617021 | 16 | 94 | 0 | 0 |
| Path 41 | C00024->C00047:[49->1,50->2,50->3,50->9] | 0.67 | 563.103448276 | 17 | 29 | 0 | 0 |
| Path 42 | C00026->C00047:[3->3,3->9], C00024->C00047:[49->1,50->2] | 0.67 | 212.955555556 | 12 | 90 | 0 | 0 |
| Path 43 | C00024->C00047:[49->1,49->3,49->9,50->2] | 0.67 | 582.7 | 12 | 20 | 0 | 0 |
| Path 44 | C00024->C00047:[49->1,49->3,49->9,50->2] | 0.67 | 669.961538462 | 16 | 26 | 0 | 0 |
| Path 45 | C00026->C00047:[3->3,3->9], C00024->C00047:[49->1,50->2] | 0.67 | 208.711111111 | 13 | 90 | 0 | 0 |
| Path 46 | C00026->C00047:[3->3,3->9], C00024->C00047:[49->1,50->2] | 0.67 | 246.557894737 | 15 | 95 | 0 | 0 |
| Path 47 | C00026->C00047:[3->3,3->9], C00024->C00047:[49->1,50->2] | 0.67 | 403.382022472 | 14 | 89 | 0 | 0 |
| Path 48 | C00026->C00047:[3->3,3->9], C00024->C00047:[49->3,49->5,49->9,50->8] | 0.67 | 213.891304348 | 13 | 92 | 0 | 0 |
| Path 49 | C00026->C00047:[3->3,3->9], C00024->C00047:[49->5,50->8] | 0.67 | 208.711111111 | 13 | 90 | 0 | 0 |
| Path 50 | C00024->C00047:[49->1,49->3,49->9,50->2] | 0.67 | 513.5 | 17 | 36 | 0 | 0 |
| Path 51 | C00026->C00047:[3->3,3->9], C00024->C00047:[49->1,50->2] | 0.67 | 195.356435644 | 15 | 101 | 0 | 0 |
| Path 52 | C00024->C00047:[14->3,14->9,49->1,50->2] | 0.67 | 372.375886525 | 21 | 141 | 0 | 0 |
| Path 53 | C00024->C00047:[49->1,49->3,49->9,50->2] | 0.67 | 353.333333333 | 19 | 138 | 0 | 0 |
| Path 54 | C00024->C00047:[49->1,49->3,49->9,50->2] | 0.67 | 459.617647059 | 16 | 34 | 0 | 0 |
| Path 55 | C00026->C00047:[3->3,3->9], C00024->C00047:[49->1,50->2] | 0.67 | 267.716981132 | 15 | 159 | 0 | 0 |
| Path 56 | C00024->C00047:[49->1,49->3,49->9,50->2] | 0.67 | 459.0 | 14 | 31 | 0 | 0 |
| Path 57 | C00026->C00047:[3->3,3->9], C00024->C00047:[49->1,50->2] | 0.67 | 200.022222222 | 12 | 90 | 0 | 0 |
| Path 58 | C00026->C00047:[3->3,3->9], C00024->C00047:[49->1,50->2] | 0.67 | 434.474226804 | 18 | 97 | 0 | 0 |
| Path 59 | C00026->C00047:[3->3,3->9], C00024->C00047:[49->1,50->2] | 0.67 | 272.38125 | 14 | 160 | 0 | 0 |
| Path 60 | C00026->C00047:[3->3,3->9], C00024->C00047:[49->5,50->8] | 0.67 | 203.95505618 | 13 | 89 | 0 | 0 |
| Path 61 | C00026->C00047:[3->3,3->9], C00024->C00047:[49->5,50->8] | 0.67 | 198.404494382 | 11 | 89 | 0 | 0 |
| Path 62 | C00026->C00047:[3->3,3->9], C00024->C00047:[49->1,49->3,49->9,50->2] | 0.67 | 246.75257732 | 16 | 97 | 0 | 0 |
| Path 63 | C00026->C00047:[3->3,3->9], C00024->C00047:[49->3,49->5,49->9,50->8] | 0.67 | 227.806451613 | 14 | 93 | 0 | 0 |
| Path 64 | C00026->C00047:[3->3,3->9], C00024->C00047:[49->1,50->2] | 0.67 | 441.530612245 | 20 | 98 | 0 | 0 |
| Path 65 | C00026->C00047:[3->3,3->9], C00024->C00047:[49->5,50->8] | 0.67 | 277.683229814 | 16 | 161 | 0 | 0 |
| Path 66 | C00026->C00047:[3->3,3->9], C00024->C00047:[49->1,50->2] | 0.67 | 212.339805825 | 16 | 103 | 0 | 0 |
| Path 67 | C00024->C00047:[49->1,49->3,49->9,50->2] | 0.67 | 430.46875 | 14 | 32 | 0 | 0 |
| Path 68 | C00026->C00047:[3->3,3->9], C00024->C00047:[49->1,49->3,49->9,50->2] | 0.67 | 259.62244898 | 17 | 98 | 0 | 0 |
| Path 69 | C00024->C00047:[49->1,50->2,50->3,50->9] | 0.67 | 677.125 | 21 | 32 | 0 | 0 |
| Path 70 | C00026->C00047:[3->3,3->9], C00024->C00047:[49->1,50->2] | 0.67 | 265.10625 | 14 | 160 | 0 | 0 |
| Path 71 | C00026->C00047:[3->3,3->9], C00024->C00047:[49->1,50->2] | 0.67 | 264.560386473 | 18 | 207 | 0 | 0 |
| Path 72 | C00024->C00047:[49->1,49->3,49->9,50->2] | 0.67 | 647.689655172 | 17 | 29 | 0 | 0 |
| Path 73 | C00026->C00047:[3->3,3->9], C00024->C00047:[49->1,50->2] | 0.67 | 259.867088608 | 14 | 158 | 0 | 0 |
| Path 74 | C00024->C00047:[49->1,49->3,49->9,50->2] | 0.67 | 613.909090909 | 14 | 22 | 0 | 0 |
| Path 75 | C00026->C00047:[3->3,3->9], C00024->C00047:[49->1,50->2] | 0.67 | 260.131313131 | 17 | 99 | 0 | 0 |
| Path 76 | C00026->C00047:[3->3,3->9], C00024->C00047:[49->1,50->2] | 0.67 | 224.741935484 | 15 | 93 | 0 | 0 |
| Path 77 | C00026->C00047:[3->3,3->9], C00024->C00047:[49->1,50->2] | 0.67 | 248.373737374 | 17 | 99 | 0 | 0 |
| Path 78 | C00024->C00047:[49->1,50->2,50->3,50->9] | 0.67 | 568.743589744 | 21 | 39 | 0 | 0 |
| Path 79 | C00026->C00047:[3->3,3->9], C00024->C00047:[49->1,49->3,49->9,50->2] | 0.67 | 215.290322581 | 14 | 93 | 0 | 0 |
| Path 80 | C00026->C00047:[3->3,3->9], C00024->C00047:[49->1,50->2] | 0.67 | 289.927272727 | 17 | 165 | 0 | 0 |
| Path 81 | C00026->C00047:[3->3,3->9], C00024->C00047:[49->1,50->2] | 0.67 | 256.628865979 | 18 | 97 | 0 | 0 |
| Path 82 | C00026->C00047:[3->3,3->9], C00024->C00047:[49->1,50->2] | 0.67 | 204.747572816 | 15 | 103 | 0 | 0 |
| Path 83 | C00026->C00047:[3->3,3->9], C00024->C00047:[49->1,50->2] | 0.67 | 252.43877551 | 18 | 98 | 0 | 0 |
| Path 84 | C00026->C00047:[3->3,3->9], C00024->C00047:[49->1,50->2] | 0.67 | 198.404494382 | 11 | 89 | 0 | 0 |
| Path 85 | C00026->C00047:[3->3,3->9], C00024->C00047:[49->1,49->3,49->9,50->2] | 0.67 | 237.361702128 | 16 | 94 | 0 | 0 |
| Path 86 | C00026->C00047:[3->3,3->9], C00024->C00047:[49->1,50->2] | 0.67 | 268.701923077 | 20 | 208 | 0 | 0 |
| Path 87 | C00024->C00047:[14->3,14->9,49->1,50->2] | 0.67 | 354.764705882 | 18 | 136 | 0 | 0 |
| Path 88 | C00026->C00047:[3->3,3->9], C00024->C00047:[49->1,49->3,49->9,50->2] | 0.67 | 219.260869565 | 15 | 92 | 0 | 0 |
| Path 89 | C00026->C00047:[3->3,3->9], C00024->C00047:[49->1,50->2] | 0.67 | 347.097560976 | 19 | 205 | 0 | 0 |
| Path 90 | C00026->C00047:[3->3,3->9], C00024->C00047:[49->1,50->2] | 0.67 | 282.5 | 16 | 164 | 0 | 0 |
| Path 91 | C00026->C00047:[3->3,3->9], C00024->C00047:[49->1,50->2] | 0.67 | 233.138297872 | 14 | 94 | 0 | 0 |
| Path 92 | C00026->C00047:[3->3,3->9], C00024->C00047:[49->1,49->3,49->9,50->2] | 0.67 | 412.934065934 | 15 | 91 | 0 | 0 |
| Path 93 | C00024->C00047:[49->1,49->3,49->9,50->2] | 0.67 | 367.1 | 22 | 140 | 0 | 0 |
| Path 94 | C00026->C00047:[3->3,3->9], C00024->C00047:[49->1,50->2] | 0.67 | 428.107526882 | 16 | 93 | 0 | 0 |
| Path 95 | C00026->C00047:[3->3,3->9], C00024->C00047:[49->3,49->5,49->9,50->8] | 0.67 | 229.042553191 | 15 | 94 | 0 | 0 |
| Path 96 | C00026->C00047:[3->3,3->9], C00024->C00047:[49->1,50->2] | 0.67 | 189.136363636 | 12 | 88 | 0 | 0 |
| Path 97 | C00026->C00047:[3->3,3->9], C00024->C00047:[49->1,50->2] | 0.67 | 391.367816092 | 12 | 87 | 0 | 0 |
| Path 98 | C00026->C00047:[3->3,3->9], C00024->C00047:[49->1,50->2] | 0.67 | 224.798076923 | 17 | 104 | 0 | 0 |
| Path 99 | C00026->C00047:[3->3,3->9], C00024->C00047:[49->1,49->3,49->9,50->2] | 0.67 | 420.684782609 | 17 | 92 | 0 | 0 |
| Path 100 | C00026->C00047:[3->3,3->9], C00024->C00047:[49->1,50->2] | 0.67 | 234.305263158 | 15 | 95 | 0 | 0 |
| Path 101 | C00026->C00047:[3->3,3->9], C00024->C00047:[49->1,50->2] | 0.67 | 264.942307692 | 19 | 208 | 0 | 0 |
| Path 102 | C00026->C00047:[3->3,3->9], C00024->C00047:[49->1,50->2] | 0.67 | 272.826086957 | 15 | 161 | 0 | 0 |
| Path 103 | C00026->C00047:[3->3,3->9], C00024->C00047:[49->1,50->2] | 0.67 | 255.71875 | 17 | 96 | 0 | 0 |
| Path 104 | C00026->C00047:[3->3,3->9], C00024->C00047:[49->3,49->5,49->9,50->8] | 0.67 | 237.361702128 | 16 | 94 | 0 | 0 |
| Path 105 | C00024->C00047:[49->1,49->3,49->9,50->2] | 0.67 | 361.514285714 | 21 | 140 | 0 | 0 |
| Path 106 | C00026->C00047:[3->3,3->9], C00024->C00047:[49->1,49->3,49->9,50->2] | 0.67 | 260.474747475 | 18 | 99 | 0 | 0 |
| Path 107 | C00026->C00047:[3->3,3->9], C00024->C00047:[49->1,49->3,49->9,50->2] | 0.67 | 371.36 | 15 | 100 | 0 | 0 |
| Path 108 | C00026->C00047:[3->3,3->9], C00024->C00047:[49->1,50->2] | 0.67 | 274.631578947 | 21 | 209 | 0 | 0 |
| Path 109 | C00026->C00047:[3->3,3->9], C00024->C00047:[49->1,50->2] | 0.67 | 260.922330097 | 19 | 206 | 0 | 0 |
| Path 110 | C00026->C00047:[3->3,3->9], C00024->C00047:[49->1,49->3,49->9,50->2] | 0.67 | 400.766666667 | 14 | 90 | 0 | 0 |
| Path 111 | C00026->C00047:[3->3,3->9], C00024->C00047:[49->5,50->8] | 0.67 | 214.395604396 | 13 | 91 | 0 | 0 |
| Path 112 | C00026->C00047:[3->3,3->9], C00024->C00047:[49->1,49->3,49->9,50->2] | 0.67 | 227.806451613 | 14 | 93 | 0 | 0 |
| Path 113 | C00026->C00047:[3->3,3->9], C00024->C00047:[49->1,50->2] | 0.67 | 264.610062893 | 13 | 159 | 0 | 0 |
| Path 114 | C00026->C00047:[3->3,3->9], C00024->C00047:[49->1,49->3,49->9,50->2] | 0.67 | 205.098901099 | 14 | 91 | 0 | 0 |
| Path 115 | C00026->C00047:[3->3,3->9], C00024->C00047:[49->1,50->2] | 0.67 | 260.97 | 18 | 100 | 0 | 0 |
| Path 116 | C00026->C00047:[3->3,3->9], C00024->C00047:[49->5,50->8] | 0.67 | 272.38125 | 14 | 160 | 0 | 0 |
| Path 117 | C00026->C00047:[3->3,3->9], C00024->C00047:[49->1,50->2] | 0.67 | 242.536842105 | 16 | 95 | 0 | 0 |
| Path 118 | C00026->C00047:[3->3,3->9], C00024->C00047:[49->5,50->8] | 0.67 | 200.022222222 | 12 | 90 | 0 | 0 |
| Path 119 | C00026->C00047:[3->3,3->9], C00024->C00047:[49->5,50->8] | 0.67 | 264.610062893 | 13 | 159 | 0 | 0 |
| Path 120 | C00024->C00047:[49->1,49->3,49->9,50->2] | 0.67 | 361.64028777 | 20 | 139 | 0 | 0 |
| Path 121 | C00026->C00047:[3->3,3->9], C00024->C00047:[49->1,50->2] | 0.67 | 225.933333333 | 18 | 105 | 0 | 0 |
| Path 122 | C00026->C00047:[3->3,3->9], C00024->C00047:[49->1,50->2] | 0.67 | 245.462962963 | 18 | 108 | 0 | 0 |
| Path 123 | C00024->C00047:[49->1,49->3,49->9,50->2] | 0.67 | 482.617647059 | 17 | 34 | 0 | 0 |
| Path 124 | C00024->C00047:[49->1,49->3,49->9,50->2] | 0.67 | 637.566666667 | 18 | 30 | 0 | 0 |
| Path 125 | C00024->C00047:[49->1,49->3,49->9,50->2] | 0.67 | 626.761904762 | 13 | 21 | 0 | 0 |
| Path 126 | C00026->C00047:[3->3,3->9], C00024->C00047:[49->1,50->2] | 0.67 | 256.272727273 | 18 | 99 | 0 | 0 |
| Path 127 | C00026->C00047:[3->3,3->9], C00024->C00047:[49->1,50->2] | 0.67 | 247.397959184 | 16 | 98 | 0 | 0 |
| Path 128 | C00026->C00047:[3->3,3->9], C00024->C00047:[49->1,50->2] | 0.67 | 269.99375 | 15 | 160 | 0 | 0 |
| Path 129 | C00024->C00047:[49->1,49->3,49->9,50->2] | 0.67 | 592.463414634 | 18 | 41 | 0 | 0 |
| Path 130 | C00026->C00047:[3->3,3->9], C00024->C00047:[49->1,50->2] | 0.67 | 404.056818182 | 13 | 88 | 0 | 0 |
| Path 131 | C00026->C00047:[3->3,3->9], C00024->C00047:[49->1,50->2] | 0.67 | 352.733009709 | 20 | 206 | 0 | 0 |
| Path 132 | C00026->C00047:[3->3,3->9], C00024->C00047:[49->1,49->3,49->9,50->2] | 0.67 | 382.235294118 | 17 | 102 | 0 | 0 |
| Path 133 | C00026->C00047:[3->3,3->9], C00024->C00047:[49->1,49->3,49->9,50->2] | 0.67 | 238.484210526 | 17 | 95 | 0 | 0 |
| Path 134 | C00026->C00047:[3->3,3->9], C00024->C00047:[49->1,50->2] | 0.67 | 290.253012048 | 18 | 166 | 0 | 0 |
| Path 135 | C00026->C00047:[3->3,3->9], C00024->C00047:[49->1,50->2] | 0.67 | 294.963855422 | 19 | 166 | 0 | 0 |
| Path 136 | C00024->C00047:[49->1,49->3,49->9,50->2] | 0.67 | 463.121212121 | 15 | 33 | 0 | 0 |
| Path 137 | C00026->C00047:[3->3,3->9], C00024->C00047:[49->5,50->8] | 0.67 | 216.048543689 | 15 | 103 | 0 | 0 |
| Path 138 | C00026->C00047:[3->3,3->9], C00024->C00047:[49->1,49->3,49->9,50->2] | 0.67 | 223.698924731 | 15 | 93 | 0 | 0 |
| Path 139 | C00026->C00047:[3->3,3->9], C00024->C00047:[49->1,50->2] | 0.67 | 270.538461538 | 19 | 208 | 0 | 0 |
| Path 140 | C00026->C00047:[3->3,3->9], C00024->C00047:[49->1,50->2] | 0.67 | 224.304347826 | 15 | 92 | 0 | 0 |
| Path 141 | C00026->C00047:[3->3,3->9], C00024->C00047:[49->1,49->3,49->9,50->2] | 0.67 | 268.373737374 | 19 | 99 | 0 | 0 |
| Path 142 | C00026->C00047:[3->3,3->9], C00024->C00047:[49->1,50->2] | 0.67 | 423.291666667 | 17 | 96 | 0 | 0 |
| Path 143 | C00024->C00047:[49->3,49->9] | 0.33 | 678.411764706 | 10 | 17 | 0 | 0 |
| Path 144 | C00026->C00047:[3->3,3->9] | 0.33 | 277.52866242 | 15 | 157 | 0 | 0 |
| Path 145 | C00026->C00047:[3->3,3->9] | 0.33 | 269.27804878 | 17 | 205 | 0 | 0 |
| Path 146 | C00026->C00047:[3->3,3->9] | 0.33 | 201.976744186 | 10 | 86 | 0 | 0 |
| Path 147 | C00026->C00047:[3->3,3->9] | 0.33 | 262.807881773 | 15 | 203 | 0 | 0 |
| Path 148 | C00026->C00047:[3->3,3->9] | 0.33 | 205.220930233 | 12 | 86 | 0 | 0 |
| Path 149 | C00026->C00047:[3->3,3->9] | 0.33 | 403.904761905 | 10 | 84 | 0 | 0 |
| Path 150 | C00026->C00047:[3->3,3->9], C00024->C00047:[49->1,50->2] | 0.67 | 401.613636364 | 13 | 88 | 0 | 0 |
| Path 151 | C00024->C00047:[49->1,49->3,49->9,50->2] | 0.67 | 657.888888889 | 17 | 27 | 0 | 0 |
| Path 152 | C00026->C00047:[3->3,3->9] | 0.33 | 194.388235294 | 10 | 85 | 0 | 0 |
| Path 153 | C00026->C00047:[3->3,3->9], C00024->C00047:[49->3,49->9] | 0.33 | 210.715909091 | 12 | 88 | 0 | 0 |
| Path 154 | C00026->C00047:[3->3,3->9], C00024->C00047:[49->3,49->9] | 0.33 | 391.387755102 | 15 | 98 | 0 | 0 |
| Path 155 | C00026->C00047:[3->3,3->9], C00024->C00047:[49->1,50->2] | 0.67 | 435.531914894 | 18 | 94 | 0 | 0 |
| Path 156 | C00026->C00047:[3->3,3->9] | 0.33 | 206.418604651 | 9 | 86 | 0 | 0 |
| Path 157 | C00026->C00047:[3->3,3->9] | 0.33 | 268.911764706 | 16 | 204 | 0 | 0 |
| Path 158 | C00024->C00047:[49->3,49->9] | 0.33 | 534.450980392 | 19 | 51 | 0 | 0 |
| Path 159 | C00026->C00047:[3->3,3->9], C00024->C00047:[49->1,50->2] | 0.67 | 226.403846154 | 16 | 104 | 0 | 0 |
| Path 160 | C00026->C00047:[3->3] | 0.17 | 425.585365854 | 11 | 82 | 0 | 0 |
| Path 161 | C00026->C00047:[3->3,3->9] | 0.33 | 282.189873418 | 14 | 158 | 0 | 0 |
| Path 162 | C00024->C00047:[49->3,49->9] | 0.33 | 681.269230769 | 15 | 26 | 0 | 0 |
| Path 163 | C00026->C00047:[3->3,3->9] | 0.33 | 263.205882353 | 16 | 204 | 0 | 0 |
| Path 164 | C00026->C00047:[3->3,3->9], C00024->C00047:[49->1,50->2] | 0.67 | 251.441176471 | 19 | 102 | 0 | 0 |
| Path 165 | C00024->C00047:[49->1,49->3,49->9,50->2] | 0.67 | 657.045454545 | 14 | 22 | 0 | 0 |
| Path 166 | C00026->C00047:[3->3,3->9] | 0.33 | 264.116129032 | 12 | 155 | 0 | 0 |
| Path 167 | C00024->C00047:[49->1,49->3,49->9,50->2] | 0.67 | 686.851851852 | 18 | 27 | 0 | 0 |
| Path 168 | C00026->C00047:[3->3,3->9] | 0.33 | 209.662790698 | 11 | 86 | 0 | 0 |
| Path 169 | C00026->C00047:[3->3,3->9] | 0.33 | 210.494949495 | 12 | 99 | 0 | 0 |
| Path 170 | C00026->C00047:[3->3] | 0.17 | 193.573170732 | 7 | 82 | 0 | 0 |
| Path 171 | C00026->C00047:[3->3,3->9], C00024->C00047:[49->1,50->2] | 0.67 | 212.955555556 | 12 | 90 | 0 | 0 |
| Path 172 | C00024->C00047:[49->3,49->9] | 0.33 | 494.769230769 | 18 | 39 | 0 | 0 |
| Path 173 | C00026->C00047:[3->3,3->9] | 0.33 | 242.882978723 | 13 | 94 | 0 | 0 |
| Path 174 | C00026->C00047:[3->3,3->9] | 0.33 | 346.970149254 | 16 | 201 | 0 | 0 |
| Path 175 | C00026->C00047:[3->3,3->9] | 0.33 | 352.674876847 | 18 | 203 | 0 | 0 |
| Path 176 | C00026->C00047:[3->3,3->9], C00024->C00047:[49->3,49->9] | 0.33 | 425.636363636 | 13 | 88 | 0 | 0 |
| Path 177 | C00024->C00047:[49->3,49->9] | 0.33 | 711.346153846 | 16 | 26 | 0 | 0 |
| Path 178 | C00026->C00047:[3->3,3->9] | 0.33 | 262.316129032 | 10 | 155 | 0 | 0 |
| Path 179 | C00026->C00047:[3->3,3->9] | 0.33 | 198.737373737 | 12 | 99 | 0 | 0 |
| Path 180 | C00026->C00047:[3->3,3->9], C00024->C00047:[49->3,49->9] | 0.33 | 433.505617978 | 15 | 89 | 0 | 0 |
| Path 181 | C00026->C00047:[3->3,3->9], C00024->C00047:[49->1,49->3,49->9,50->2] | 0.67 | 248.473684211 | 17 | 95 | 0 | 0 |
| Path 182 | C00026->C00047:[3->3] | 0.17 | 204.807228916 | 9 | 83 | 0 | 0 |
| Path 183 | C00026->C00047:[3->3,3->9], C00024->C00047:[49->3,49->9] | 0.33 | 393.091836735 | 14 | 98 | 0 | 0 |
| Path 184 | C00024->C00047:[49->3,49->9] | 0.33 | 691.058823529 | 10 | 17 | 0 | 0 |
| Path 185 | C00024->C00047:[49->3,49->9] | 0.33 | 344.282758621 | 18 | 145 | 0 | 0 |
| Path 186 | C00026->C00047:[3->3,3->9] | 0.33 | 257.435064935 | 11 | 154 | 0 | 0 |
| Path 187 | C00024->C00047:[49->1,49->3,49->9,50->2] | 0.67 | 609.142857143 | 19 | 42 | 0 | 0 |
| Path 188 | C00026->C00047:[3->3,3->9], C00024->C00047:[49->3,49->9] | 0.33 | 423.738636364 | 14 | 88 | 0 | 0 |
| Path 189 | C00026->C00047:[3->3,3->9], C00024->C00047:[49->3,49->9] | 0.33 | 414.852272727 | 13 | 88 | 0 | 0 |
| Path 190 | C00024->C00047:[49->1,49->3,49->9,50->2] | 0.67 | 513.5 | 17 | 36 | 0 | 0 |
| Path 191 | C00026->C00047:[3->3,3->9] | 0.33 | 243.947368421 | 14 | 95 | 0 | 0 |
| Path 192 | C00026->C00047:[3->3,3->9] | 0.33 | 265.503225806 | 12 | 155 | 0 | 0 |
| Path 193 | C00024->C00047:[49->3,49->9] | 0.33 | 473.766666667 | 13 | 30 | 0 | 0 |
| Path 194 | C00026->C00047:[3->3,3->9], C00024->C00047:[49->3,49->9] | 0.33 | 372.104166667 | 12 | 96 | 0 | 0 |
| Path 195 | C00026->C00047:[3->3,3->9] | 0.33 | 220.881188119 | 15 | 101 | 0 | 0 |
| Path 196 | C00024->C00047:[49->3] | 0.17 | 833.533333333 | 11 | 15 | 0 | 0 |
| Path 197 | C00024->C00047:[49->1,49->3,49->9,50->2] | 0.67 | 616.523809524 | 13 | 21 | 0 | 0 |
| Path 198 | C00026->C00047:[3->3,3->9] | 0.33 | 218.431818182 | 12 | 88 | 0 | 0 |
| Path 199 | C00024->C00047:[49->3,49->9] | 0.33 | 474.62962963 | 11 | 27 | 0 | 0 |
| Path 200 | C00026->C00047:[3->3,3->9], C00024->C00047:[49->3,49->9] | 0.33 | 381.597938144 | 13 | 97 | 0 | 0 |
| Path 201 | C00026->C00047:[3->3,3->9] | 0.33 | 259.089108911 | 16 | 202 | 0 | 0 |
| Path 202 | C00026->C00047:[3->3,3->9], C00024->C00047:[49->5,50->8] | 0.67 | 391.367816092 | 12 | 87 | 0 | 0 |
| Path 203 | C00024->C00047:[49->1,49->3,49->9,50->2] | 0.67 | 669.961538462 | 16 | 26 | 0 | 0 |
| Path 204 | C00024->C00047:[49->3,49->9] | 0.33 | 694.76 | 14 | 25 | 0 | 0 |
| Path 205 | C00026->C00047:[3->3,3->9], C00024->C00047:[49->3,49->9] | 0.33 | 229.811111111 | 13 | 90 | 0 | 0 |
| Path 206 | C00026->C00047:[3->3,3->9] | 0.33 | 275.751592357 | 13 | 157 | 0 | 0 |
| Path 207 | C00026->C00047:[3->3,3->9], C00024->C00047:[49->1,50->2] | 0.67 | 427.212765957 | 17 | 94 | 0 | 0 |
| Path 208 | C00026->C00047:[3->3,3->9], C00024->C00047:[49->1,50->2] | 0.67 | 234.619565217 | 15 | 92 | 0 | 0 |
| Path 209 | C00024->C00047:[49->3,49->9] | 0.33 | 638.780487805 | 18 | 41 | 0 | 0 |
| Path 210 | C00026->C00047:[3->3,3->9] | 0.33 | 273.436893204 | 19 | 206 | 0 | 0 |
| Path 211 | C00024->C00047:[49->1,50->2] | 0.33 | 619.0 | 11 | 18 | 0 | 0 |
| Path 212 | C00024->C00047:[49->3,49->5,49->9,50->8] | 0.67 | 649.454545455 | 15 | 22 | 0 | 0 |
| Path 213 | C00024->C00047:[49->3,49->9] | 0.33 | 503.857142857 | 12 | 28 | 0 | 0 |
| Path 214 | C00026->C00047:[3->3,3->9], C00024->C00047:[49->3,49->9] | 0.33 | 213.159090909 | 12 | 88 | 0 | 0 |
| Path 215 | C00026->C00047:[3->3,3->9], C00024->C00047:[49->1,50->2] | 0.67 | 294.807017544 | 20 | 114 | 0 | 0 |
| Path 216 | C00026->C00047:[3->3,3->9] | 0.33 | 214.517241379 | 11 | 87 | 0 | 0 |
| Path 217 | C00026->C00047:[3->3,3->9] | 0.33 | 206.636363636 | 13 | 99 | 0 | 0 |
| Path 218 | C00026->C00047:[3->3,3->9], C00024->C00047:[49->5,50->8] | 0.67 | 404.056818182 | 13 | 88 | 0 | 0 |
| Path 219 | C00026->C00047:[3->3,3->9] | 0.33 | 221.32 | 13 | 100 | 0 | 0 |
| Path 220 | C00026->C00047:[3->3,3->9] | 0.33 | 262.83974359 | 11 | 156 | 0 | 0 |
| Path 221 | C00026->C00047:[3->3,3->9] | 0.33 | 216.988505747 | 11 | 87 | 0 | 0 |
| Path 222 | C00026->C00047:[3->3,3->9] | 0.33 | 268.923076923 | 11 | 156 | 0 | 0 |
| Path 223 | C00026->C00047:[3->3,3->9], C00024->C00047:[49->1,50->2] | 0.67 | 414.04494382 | 14 | 89 | 0 | 0 |
| Path 224 | C00024->C00047:[49->3,49->9] | 0.33 | 671.777777778 | 11 | 18 | 0 | 0 |
| Path 225 | C00026->C00047:[3->3,3->9], C00024->C00047:[49->3,49->9] | 0.33 | 402.965116279 | 11 | 86 | 0 | 0 |
| Path 226 | C00026->C00047:[3->3,3->9] | 0.33 | 247.275510204 | 16 | 98 | 0 | 0 |
| Path 227 | C00026->C00047:[3->3,3->9] | 0.33 | 197.255102041 | 11 | 98 | 0 | 0 |
| Path 228 | C00024->C00047:[49->3,49->9] | 0.33 | 361.75 | 18 | 136 | 0 | 0 |
| Path 229 | C00026->C00047:[3->3,3->9] | 0.33 | 203.918604651 | 9 | 86 | 0 | 0 |
| Path 230 | C00024->C00047:[49->3,49->9] | 0.33 | 724.5 | 11 | 18 | 0 | 0 |
| Path 231 | C00026->C00047:[3->3,3->9] | 0.33 | 239.371134021 | 17 | 97 | 0 | 0 |
| Path 232 | C00026->C00047:[3->3,3->9] | 0.33 | 208.323232323 | 12 | 99 | 0 | 0 |
| Path 233 | C00026->C00047:[3->3,3->9], C00024->C00047:[49->1,50->2] | 0.67 | 220.626373626 | 14 | 91 | 0 | 0 |
| Path 234 | C00026->C00047:[3->3,3->9], C00024->C00047:[49->3,49->9] | 0.33 | 383.408163265 | 14 | 98 | 0 | 0 |
| Path 235 | C00026->C00047:[3->3] | 0.17 | 416.938271605 | 9 | 81 | 0 | 0 |
| Path 236 | C00026->C00047:[3->3,3->9] | 0.33 | 449.861111111 | 18 | 108 | 0 | 0 |
| Path 237 | C00026->C00047:[3->3,3->9] | 0.33 | 257.114583333 | 15 | 96 | 0 | 0 |
| Path 238 | C00024->C00047:[49->3,49->9] | 0.33 | 499.833333333 | 14 | 30 | 0 | 0 |
| Path 239 | C00026->C00047:[3->3,3->9] | 0.33 | 425.139534884 | 13 | 86 | 0 | 0 |
| Path 240 | C00024->C00047:[49->3,49->9] | 0.33 | 715.222222222 | 12 | 18 | 0 | 0 |
| Path 241 | C00026->C00047:[3->3,3->9] | 0.33 | 218.908045977 | 10 | 87 | 0 | 0 |
| Path 242 | C00024->C00047:[49->3,49->9] | 0.33 | 628.586206897 | 17 | 29 | 0 | 0 |
| Path 243 | C00024->C00047:[49->3,49->9] | 0.33 | 525.419354839 | 15 | 31 | 0 | 0 |
| Path 244 | C00024->C00047:[49->3,49->9] | 0.33 | 441.464285714 | 11 | 28 | 0 | 0 |
| Path 245 | C00026->C00047:[3->3,3->9] | 0.33 | 437.956989247 | 15 | 93 | 0 | 0 |
| Path 246 | C00026->C00047:[3->3,3->9] | 0.33 | 423.166666667 | 17 | 96 | 0 | 0 |
| Path 247 | C00026->C00047:[3->3,3->9], C00024->C00047:[49->3,49->9] | 0.33 | 219.741573034 | 11 | 89 | 0 | 0 |
| Path 248 | C00026->C00047:[3->3,3->9] | 0.33 | 286.055045872 | 18 | 109 | 0 | 0 |
| Path 249 | C00024->C00047:[49->3,49->5,49->9,50->8] | 0.67 | 626.761904762 | 13 | 21 | 0 | 0 |
| Path 250 | C00026->C00047:[3->3,3->9] | 0.33 | 393.192771084 | 9 | 83 | 0 | 0 |
| Path 251 | C00024->C00047:[49->3] | 0.17 | 661.941176471 | 14 | 34 | 0 | 0 |
| Path 252 | C00026->C00047:[3->3,3->9] | 0.33 | 414.929411765 | 12 | 85 | 0 | 0 |
| Path 253 | C00026->C00047:[3->3,3->9] | 0.33 | 405.729411765 | 11 | 85 | 0 | 0 |
| Path 254 | C00026->C00047:[3->3,3->9], C00024->C00047:[49->3,49->9] | 0.33 | 198.275862069 | 11 | 87 | 0 | 0 |
| Path 255 | C00026->C00047:[3->3,3->9] | 0.33 | 220.195402299 | 13 | 87 | 0 | 0 |
| Path 256 | C00024->C00047:[49->3,49->9] | 0.33 | 745.631578947 | 13 | 19 | 0 | 0 |
| Path 257 | C00026->C00047:[3->3,3->9] | 0.33 | 274.382165605 | 13 | 157 | 0 | 0 |
| Path 258 | C00024->C00047:[49->3,49->9] | 0.33 | 640.0 | 9 | 16 | 0 | 0 |
| Path 259 | C00026->C00047:[3->3,3->9], C00024->C00047:[49->1,50->2] | 0.67 | 224.824175824 | 13 | 91 | 0 | 0 |
| Path 260 | C00026->C00047:[3->3,3->9] | 0.33 | 252.178947368 | 15 | 95 | 0 | 0 |
| Path 261 | C00026->C00047:[3->3,3->9], C00024->C00047:[49->1,50->2] | 0.67 | 271.0375 | 14 | 160 | 0 | 0 |
| Path 262 | C00026->C00047:[3->3,3->9] | 0.33 | 208.0 | 10 | 87 | 0 | 0 |
| Path 263 | C00026->C00047:[3->3,3->9], C00024->C00047:[49->3,49->9] | 0.33 | 222.157303371 | 11 | 89 | 0 | 0 |
| Path 264 | C00026->C00047:[3->3,3->9] | 0.33 | 266.072164948 | 17 | 97 | 0 | 0 |
| Path 265 | C00026->C00047:[3->3,3->9] | 0.33 | 196.917647059 | 10 | 85 | 0 | 0 |
| Path 266 | C00026->C00047:[3->3,3->9], C00024->C00047:[49->3,49->9] | 0.33 | 233.428571429 | 14 | 91 | 0 | 0 |
| Path 267 | C00024->C00047:[49->3,49->5,49->9,50->8] | 0.67 | 582.7 | 12 | 20 | 0 | 0 |
| Path 268 | C00024->C00047:[49->1,49->3,49->9,50->2] | 0.67 | 636.44 | 15 | 25 | 0 | 0 |
| Path 269 | C00024->C00047:[49->3,49->9] | 0.33 | 505.4 | 13 | 30 | 0 | 0 |
| Path 270 | C00026->C00047:[3->3,3->9] | 0.33 | 213.313131313 | 14 | 99 | 0 | 0 |
| Path 271 | C00026->C00047:[3->3,3->9], C00024->C00047:[49->3,49->9] | 0.33 | 383.81443299 | 13 | 97 | 0 | 0 |
| Path 272 | C00026->C00047:[3->3,3->9] | 0.33 | 230.277227723 | 15 | 101 | 0 | 0 |
| Path 273 | C00026->C00047:[3->3,3->9] | 0.33 | 217.5 | 14 | 100 | 0 | 0 |
| Path 274 | C00026->C00047:[3->3,3->9], C00024->C00047:[49->1,50->2] | 0.67 | 283.950617284 | 17 | 162 | 0 | 0 |
| Path 275 | C00026->C00047:[3->3,3->9] | 0.33 | 267.039215686 | 17 | 204 | 0 | 0 |
| Path 276 | C00024->C00047:[49->1,50->2] | 0.33 | 669.52173913 | 14 | 23 | 0 | 0 |
| Path 277 | C00026->C00047:[3->3,3->9] | 0.33 | 234.591397849 | 14 | 93 | 0 | 0 |
| Path 278 | C00026->C00047:[3->3,3->9], C00024->C00047:[49->1,50->2] | 0.67 | 421.955555556 | 16 | 90 | 0 | 0 |
| Path 279 | C00026->C00047:[3->3,3->9], C00024->C00047:[49->3,49->9] | 0.33 | 217.865168539 | 12 | 89 | 0 | 0 |
| Path 280 | C00026->C00047:[3->3,3->9] | 0.33 | 219.65 | 14 | 100 | 0 | 0 |
| Path 281 | C00026->C00047:[3->3,3->9], C00024->C00047:[49->3,49->9] | 0.33 | 400.494949495 | 16 | 99 | 0 | 0 |
| Path 282 | C00026->C00047:[3->3] | 0.17 | 403.3 | 8 | 80 | 0 | 0 |
| Path 283 | C00026->C00047:[3->3,3->9] | 0.33 | 352.717821782 | 17 | 202 | 0 | 0 |
| Path 284 | C00026->C00047:[3->3,3->9], C00024->C00047:[49->1,50->2] | 0.67 | 246.557894737 | 15 | 95 | 0 | 0 |
| Path 285 | C00026->C00047:[3->3,3->9], C00024->C00047:[49->5,50->8] | 0.67 | 403.382022472 | 14 | 89 | 0 | 0 |
| Path 286 | C00026->C00047:[3->3] | 0.17 | 209.409638554 | 8 | 83 | 0 | 0 |
| Path 287 | C00026->C00047:[3->3,3->9] | 0.33 | 265.24137931 | 17 | 203 | 0 | 0 |
| Path 288 | C00024->C00047:[49->1,50->2,50->3,50->9] | 0.67 | 587.433333333 | 18 | 30 | 0 | 0 |
| Path 289 | C00026->C00047:[3->3,3->9] | 0.33 | 191.105882353 | 8 | 85 | 0 | 0 |
| Path 290 | C00024->C00047:[50->3] | 0.17 | 661.136363636 | 13 | 22 | 0 | 0 |
| Path 291 | C00024->C00047:[49->3,49->9] | 0.33 | 660.875 | 13 | 24 | 0 | 0 |
| Path 292 | C00026->C00047:[3->3,3->9] | 0.33 | 406.464285714 | 10 | 84 | 0 | 0 |
| Path 293 | C00026->C00047:[3->3,3->9], C00024->C00047:[49->3,49->9] | 0.33 | 207.545454545 | 10 | 88 | 0 | 0 |
| Path 294 | C00026->C00047:[3->3,3->9], C00024->C00047:[49->1,50->2] | 0.67 | 210.566666667 | 12 | 90 | 0 | 0 |
| Path 295 | C00024->C00047:[49->3,49->9] | 0.33 | 361.881481481 | 17 | 135 | 0 | 0 |
| Path 296 | C00024->C00047:[49->3] | 0.17 | 518.5 | 10 | 24 | 0 | 0 |
| Path 297 | C00026->C00047:[3->3,3->9], C00024->C00047:[49->3,49->9] | 0.33 | 243.857142857 | 14 | 91 | 0 | 0 |
| Path 298 | C00026->C00047:[3->3,3->9] | 0.33 | 256.2 | 14 | 95 | 0 | 0 |
| Path 299 | C00026->C00047:[3->3,3->9] | 0.33 | 426.326086957 | 14 | 92 | 0 | 0 |
| Path 300 | C00026->C00047:[3->3,3->9] | 0.33 | 270.770700637 | 12 | 157 | 0 | 0 |
| Path 301 | C00024->C00047:[49->3,49->5,49->9,50->8] | 0.67 | 613.909090909 | 14 | 22 | 0 | 0 |
| Path 302 | C00026->C00047:[3->3,3->9], C00024->C00047:[49->5,50->8] | 0.67 | 412.168539326 | 15 | 89 | 0 | 0 |
| Path 303 | C00026->C00047:[3->3,3->9] | 0.33 | 276.183544304 | 14 | 158 | 0 | 0 |
| Path 304 | C00026->C00047:[3->3,3->9] | 0.33 | 436.957446809 | 16 | 94 | 0 | 0 |
| Path 305 | C00024->C00047:[49->3] | 0.17 | 812.642857143 | 9 | 14 | 0 | 0 |
| Path 306 | C00024->C00047:[49->3,49->9] | 0.33 | 470.827586207 | 12 | 29 | 0 | 0 |
| Path 307 | C00026->C00047:[3->3,3->9], C00024->C00047:[49->3,49->9] | 0.33 | 223.511111111 | 12 | 90 | 0 | 0 |
| Path 308 | C00026->C00047:[3->3,3->9] | 0.33 | 292.672727273 | 17 | 110 | 0 | 0 |
| Path 309 | C00026->C00047:[3->3,3->9], C00024->C00047:[49->3,49->9] | 0.33 | 225.292134831 | 13 | 89 | 0 | 0 |
| Path 310 | C00024->C00047:[49->3] | 0.17 | 759.153846154 | 8 | 13 | 0 | 0 |
| Path 311 | C00026->C00047:[3->3] | 0.17 | 220.321428571 | 10 | 84 | 0 | 0 |
| Path 312 | C00026->C00047:[3->3,3->9], C00024->C00047:[49->1,49->3,49->9,50->2] | 0.67 | 225.494623656 | 14 | 93 | 0 | 0 |
| Path 313 | C00026->C00047:[3->3,3->9] | 0.33 | 270.301282051 | 11 | 156 | 0 | 0 |
| Path 314 | C00026->C00047:[3->3,3->9] | 0.33 | 356.527093596 | 19 | 203 | 0 | 0 |
| Path 315 | C00026->C00047:[3->3,3->9] | 0.33 | 188.835051546 | 12 | 97 | 0 | 0 |
| Path 316 | C00026->C00047:[3->3,3->9], C00024->C00047:[49->1,50->2] | 0.67 | 278.720496894 | 15 | 161 | 0 | 0 |
| Path 317 | C00026->C00047:[3->3,3->9] | 0.33 | 267.852564103 | 12 | 156 | 0 | 0 |
| Path 318 | C00026->C00047:[3->3,3->9], C00024->C00047:[49->1,49->3,49->9,50->2] | 0.67 | 239.138297872 | 15 | 94 | 0 | 0 |
| Path 319 | C00026->C00047:[3->3,3->9] | 0.33 | 416.894117647 | 11 | 85 | 0 | 0 |
| Path 320 | C00024->C00047:[49->1,49->3,49->9,50->2] | 0.67 | 485.0625 | 15 | 32 | 0 | 0 |
| Path 321 | C00024->C00047:[49->1,49->3,49->9,50->2] | 0.67 | 626.761904762 | 13 | 21 | 0 | 0 |
| Path 322 | C00026->C00047:[3->3,3->9], C00024->C00047:[49->3,49->9] | 0.33 | 235.3 | 15 | 90 | 0 | 0 |
| Path 323 | C00026->C00047:[3->3,3->9] | 0.33 | 272.08974359 | 13 | 156 | 0 | 0 |
| Path 324 | C00026->C00047:[3->3,3->9], C00024->C00047:[49->3,49->9] | 0.33 | 234.055555556 | 12 | 90 | 0 | 0 |
| Path 325 | C00026->C00047:[3->3,3->9] | 0.33 | 273.092682927 | 18 | 205 | 0 | 0 |
| Path 326 | C00024->C00047:[49->1,49->3,49->9,50->2] | 0.67 | 677.434782609 | 16 | 23 | 0 | 0 |
| Path 327 | C00026->C00047:[3->3,3->9], C00024->C00047:[49->3,49->9] | 0.33 | 232.2 | 13 | 90 | 0 | 0 |
| Path 328 | C00026->C00047:[3->3,3->9] | 0.33 | 265.260416667 | 16 | 96 | 0 | 0 |
| Path 329 | C00024->C00047:[49->3,49->9] | 0.33 | 478.24137931 | 12 | 29 | 0 | 0 |
| Path 330 | C00024->C00047:[49->3,49->5,49->9,50->8] | 0.67 | 459.0 | 14 | 31 | 0 | 0 |
| Path 331 | C00026->C00047:[3->3,3->9], C00024->C00047:[49->1,50->2] | 0.67 | 404.056818182 | 13 | 88 | 0 | 0 |
| Path 332 | C00026->C00047:[3->3,3->9] | 0.33 | 276.815286624 | 12 | 157 | 0 | 0 |
| Path 333 | C00026->C00047:[3->3,3->9] | 0.33 | 229.215909091 | 12 | 88 | 0 | 0 |
| Path 334 | C00026->C00047:[3->3,3->9] | 0.33 | 192.88372093 | 9 | 86 | 0 | 0 |
| Path 335 | C00024->C00047:[49->3,49->9] | 0.33 | 367.5 | 19 | 136 | 0 | 0 |
| Path 336 | C00026->C00047:[3->3,3->9], C00024->C00047:[49->1,50->2] | 0.67 | 428.107526882 | 16 | 93 | 0 | 0 |
| Path 337 | C00026->C00047:[3->3,3->9], C00024->C00047:[49->3,49->9] | 0.33 | 413.195402299 | 12 | 87 | 0 | 0 |
| Path 338 | C00026->C00047:[3->3,3->9] | 0.33 | 181.30952381 | 9 | 84 | 0 | 0 |
| Path 339 | C00026->C00047:[3->3,3->9] | 0.33 | 445.276595745 | 17 | 94 | 0 | 0 |
| Path 340 | C00024->C00047:[49->3,49->9] | 0.33 | 526.742857143 | 15 | 35 | 0 | 0 |
| Path 341 | C00026->C00047:[3->3,3->9] | 0.33 | 211.83 | 13 | 100 | 0 | 0 |
| Path 342 | C00026->C00047:[3->3,3->9], C00024->C00047:[49->1,50->2] | 0.67 | 416.369565217 | 15 | 92 | 0 | 0 |
| Path 343 | C00026->C00047:[3->3,3->9], C00024->C00047:[49->3,49->9] | 0.33 | 415.666666667 | 12 | 87 | 0 | 0 |
| Path 344 | C00026->C00047:[3->3,3->9] | 0.33 | 248.138297872 | 15 | 94 | 0 | 0 |
| Path 345 | C00026->C00047:[3->3,3->9], C00024->C00047:[49->3,49->9] | 0.33 | 209.078651685 | 11 | 89 | 0 | 0 |
| Path 346 | C00026->C00047:[3->3,3->9] | 0.33 | 202.295918367 | 13 | 98 | 0 | 0 |
| Path 347 | C00024->C00047:[49->3,49->9] | 0.33 | 353.328358209 | 16 | 134 | 0 | 0 |
