# Supplementary material for: 13C-metabolic flux ratio and novel carbon path analyses confirmed that Trichoderma reesei uses primarily the respirative pathway also on the preferred carbon source glucose
Source: BMC Syst Biol. 2009 Oct 29;3:104. doi: 10.1186/1752-0509-3-104 (PMC2776023; doi:10.1186/1752-0509-3-104)
Supplement: Additional file 1 — Pathways discovered in ReTrace carbon path analysis. Graphical and tabular representations of amino acid synthesis pathways discovered in ReTrace carbon path analysis [21]. Self-contained web site: unpack zip archive and open index.html with a web browser. [file 1752-0509-3-104-S1.zip › AF1-treesei/pathways-C00031-to-C00002.html]

Pathways from C00031 to C00002


**Pathways from C00031 to C00002**

**Sources:** D-Glucose; (C00031)

**Target:**ATP; (C00002)

|  | Composite mapping | Z | Average score | Rpairs | Reactions | Zero scores | Scores under threshold |
| --- | --- | --- | --- | --- | --- | --- | --- |
| Path 1 | C00031->C00002:[4->10,4->11,4->2,4->4] | 0.40 | 592.454545455 | 33 | 44 | 0 | 1 |
| Path 2 | C00031->C00002:[1->12,2->13,4->17,5->8,7->3] | 0.50 | 351.566433566 | 16 | 143 | 0 | 0 |
| Path 3 | C00031->C00002:[1->12,2->13,4->17,5->3,5->8,9->11] | 0.60 | 370.195945946 | 22 | 148 | 0 | 0 |
| Path 4 | C00031->C00002:[1->12,2->13,4->17,5->3,5->8,7->11] | 0.60 | 571.032258065 | 20 | 31 | 0 | 0 |
| Path 5 | C00031->C00002:[4->10,4->11,4->2,4->4,7->10,7->11,7->2,7->4] | 0.40 | 378.787878788 | 35 | 165 | 0 | 1 |
| Path 6 | C00031->C00002:[1->12,2->13,4->10,4->11,4->17,5->8,9->3] | 0.70 | 335.40397351 | 24 | 151 | 0 | 0 |
| Path 7 | C00031->C00002:[4->10,4->11] | 0.20 | 337.17791411 | 24 | 163 | 0 | 0 |
| Path 8 | C00031->C00002:[1->12,2->13,4->17,5->3,5->8,7->11] | 0.60 | 524.351351351 | 22 | 37 | 0 | 0 |
| Path 9 | C00031->C00002:[4->10,4->11,4->2,4->4,7->10,7->11,7->2,7->4] | 0.40 | 372.363095238 | 35 | 168 | 0 | 1 |
| Path 10 | C00031->C00002:[4->10,4->11,4->2,4->4,7->10,7->11,7->2,7->4] | 0.40 | 366.75 | 30 | 160 | 0 | 1 |
| Path 11 | C00031->C00002:[4->10,4->11,4->2,4->4,7->10,7->11,7->2,7->4] | 0.40 | 370.5 | 35 | 168 | 0 | 1 |
| Path 12 | C00031->C00002:[4->10,4->11,4->2,4->4,7->10,7->11,7->2,7->4] | 0.40 | 420.763157895 | 37 | 76 | 0 | 1 |
| Path 13 | C00031->C00002:[4->10,4->2,7->11,7->4] | 0.40 | 426.150684932 | 36 | 73 | 0 | 1 |
| Path 14 | C00031->C00002:[4->10,4->11,4->2,4->4] | 0.40 | 350.766839378 | 40 | 193 | 0 | 1 |
| Path 15 | C00031->C00002:[4->10,4->11,4->2,4->4,7->4] | 0.40 | 412.358974359 | 38 | 78 | 0 | 1 |
| Path 16 | C00031->C00002:[4->10,4->11,4->2,4->4,7->10,7->11,7->2,7->4] | 0.40 | 372.070512821 | 29 | 156 | 0 | 1 |
| Path 17 | C00031->C00002:[7->10,7->11] | 0.20 | 464.357142857 | 16 | 28 | 0 | 0 |
| Path 18 | C00031->C00002:[7->10,7->11,9->10,9->11] | 0.20 | 368.770491803 | 26 | 61 | 0 | 0 |
| Path 19 | C00031->C00002:[4->10,4->11,4->2,4->4,7->10,7->11,7->2,7->4] | 0.40 | 517.309090909 | 35 | 55 | 0 | 1 |
| Path 20 | C00031->C00002:[9->11] | 0.10 | 347.61971831 | 15 | 142 | 0 | 0 |
| Path 21 | C00031->C00002:[7->10,7->11] | 0.20 | 479.708333333 | 16 | 24 | 0 | 0 |
| Path 22 | C00031->C00002:[4->10,4->11,4->2,4->4,7->10,7->11,7->2,7->4] | 0.40 | 370.369426752 | 30 | 157 | 0 | 1 |
| Path 23 | C00031->C00002:[1->12,2->13,4->17,5->3,5->8,7->11] | 0.60 | 373.465838509 | 25 | 161 | 0 | 0 |
| Path 24 | C00031->C00002:[4->10,4->11,4->2,4->4,7->4] | 0.40 | 412.358974359 | 38 | 78 | 0 | 1 |
| Path 25 | C00031->C00002:[4->10,4->11,4->2,4->4] | 0.40 | 360.831578947 | 40 | 190 | 0 | 1 |
| Path 26 | C00031->C00002:[1->12,2->13,4->17,5->8,7->11] | 0.50 | 364.344594595 | 22 | 148 | 0 | 0 |
| Path 27 | C00031->C00002:[4->11] | 0.10 | 349.731343284 | 12 | 134 | 0 | 0 |
| Path 28 | C00031->C00002:[4->10,4->11,4->2,4->4,7->10,7->11,7->2,7->4] | 0.40 | 541.622641509 | 35 | 53 | 0 | 1 |
| Path 29 | C00031->C00002:[1->12,2->13,4->17,5->3,5->8,7->11] | 0.60 | 380.233128834 | 26 | 163 | 0 | 0 |
| Path 30 | C00031->C00002:[7->11] | 0.10 | 334.484615385 | 10 | 130 | 0 | 0 |
| Path 31 | C00031->C00002:[1->12,2->13,4->17,5->8,7->3,9->11] | 0.60 | 362.255555556 | 31 | 180 | 0 | 0 |
| Path 32 | C00031->C00002:[1->12,2->13,4->17,5->3,5->8] | 0.50 | 355.921985816 | 15 | 141 | 0 | 0 |
| Path 33 | C00031->C00002:[1->12,2->13,4->17,5->3,5->8,9->11] | 0.60 | 366.296703297 | 29 | 182 | 0 | 0 |
| Path 34 | C00031->C00002:[5->13,7->11,7->17,9->12] | 0.40 | 377.571428571 | 25 | 147 | 0 | 0 |
| Path 35 | C00031->C00002:[7->11] | 0.10 | 332.732824427 | 11 | 131 | 0 | 0 |
| Path 36 | C00031->C00002:[4->10,4->11,4->2,4->4,7->10,7->11,7->2,7->4] | 0.40 | 346.435897436 | 40 | 195 | 0 | 1 |
| Path 37 | C00031->C00002:[1->12,2->13,4->17,5->8,7->11] | 0.50 | 557.909090909 | 21 | 33 | 0 | 0 |
| Path 38 | C00031->C00002:[1->12,2->13,4->11,4->17,5->8] | 0.50 | 352.621621622 | 19 | 148 | 0 | 0 |
| Path 39 | C00031->C00002:[7->10,7->11] | 0.20 | 310.829268293 | 21 | 164 | 0 | 0 |
| Path 40 | C00031->C00002:[7->10,7->11] | 0.20 | 334.257142857 | 16 | 140 | 0 | 0 |
| Path 41 | C00031->C00002:[5->13,7->17,9->11,9->12] | 0.40 | 349.436781609 | 28 | 174 | 0 | 0 |
| Path 42 | C00031->C00002:[1->12,2->13,4->17,5->3,5->8,7->11] | 0.60 | 580.8 | 22 | 40 | 0 | 0 |
| Path 43 | C00031->C00002:[1->12,2->13,4->17,5->3,5->8,7->11] | 0.60 | 556.973684211 | 24 | 38 | 0 | 0 |
| Path 44 | C00031->C00002:[4->10,4->2,7->11,7->4] | 0.40 | 384.420118343 | 37 | 169 | 0 | 1 |
| Path 45 | C00031->C00002:[4->10,4->11,4->2,4->4,7->10,7->11,7->2,7->4] | 0.40 | 361.825 | 30 | 160 | 0 | 1 |
| Path 46 | C00031->C00002:[4->10,4->11,4->2,4->4] | 0.40 | 357.476683938 | 39 | 193 | 0 | 1 |
| Path 47 | C00031->C00002:[4->11,7->3] | 0.20 | 372.571428571 | 24 | 154 | 0 | 0 |
| Path 48 | C00031->C00002:[4->10,4->11,4->2,4->4,7->10,7->11,7->2,7->4] | 0.40 | 369.079754601 | 32 | 163 | 0 | 1 |
| Path 49 | C00031->C00002:[4->10,4->11,7->10,7->11] | 0.20 | 454.090909091 | 19 | 33 | 0 | 0 |
| Path 50 | C00031->C00002:[4->10,4->11,4->2,4->4] | 0.40 | 376.849056604 | 33 | 159 | 0 | 1 |
| Path 51 | C00031->C00002:[4->10,4->11,4->2,4->4,7->10,7->11,7->2,7->4] | 0.40 | 367.777777778 | 31 | 162 | 0 | 1 |
| Path 52 | C00031->C00002:[7->11] | 0.10 | 494.95 | 12 | 20 | 0 | 0 |
| Path 53 | C00031->C00002:[4->10,4->2,7->10,7->11,7->2,7->4] | 0.40 | 379.654320988 | 33 | 162 | 0 | 1 |
| Path 54 | C00031->C00002:[4->10,4->11,4->2,4->4,7->10,7->11,7->2,7->4] | 0.40 | 375.5125 | 31 | 160 | 0 | 1 |
| Path 55 | C00031->C00002:[1->12,2->13,4->17,5->3,5->8,7->11] | 0.60 | 541.97826087 | 24 | 46 | 0 | 0 |
| Path 56 | C00031->C00002:[4->10,4->11,7->10,7->11] | 0.20 | 349.503355705 | 20 | 149 | 0 | 0 |
| Path 57 | C00031->C00002:[1->12,2->13,4->17,5->3,5->8,7->11] | 0.60 | 382.17721519 | 25 | 158 | 0 | 0 |
| Path 58 | C00031->C00002:[4->11,7->3] | 0.20 | 369.695364238 | 23 | 151 | 0 | 0 |
| Path 59 | C00031->C00002:[4->10,4->11] | 0.20 | 323.847560976 | 21 | 164 | 0 | 0 |
| Path 60 | C00031->C00002:[7->3,9->11] | 0.20 | 347.324175824 | 30 | 182 | 0 | 0 |
| Path 61 | C00031->C00002:[4->10,4->11,4->2,4->4,7->10,7->11,7->2,7->4] | 0.40 | 370.781065089 | 36 | 169 | 0 | 1 |
| Path 62 | C00031->C00002:[4->10,4->11,4->2,4->4,7->10,7->11,7->2,7->4] | 0.40 | 363.440251572 | 29 | 159 | 0 | 1 |
| Path 63 | C00031->C00002:[7->11,7->3] | 0.20 | 559.75 | 23 | 36 | 0 | 0 |
| Path 64 | C00031->C00002:[1->12,2->13,4->17,5->3,5->8,7->11] | 0.60 | 366.545454545 | 24 | 154 | 0 | 0 |
| Path 65 | C00031->C00002:[1->12,2->13,4->17,5->8,7->11] | 0.50 | 362.85620915 | 23 | 153 | 0 | 0 |
| Path 66 | C00031->C00002:[1->12,2->13,4->17,5->8,9->11] | 0.50 | 322.277108434 | 20 | 166 | 0 | 0 |
| Path 67 | C00031->C00002:[4->10,4->11,4->2,4->4,7->10,7->11,7->2,7->4] | 0.40 | 507.724137931 | 37 | 58 | 0 | 1 |
| Path 68 | C00031->C00002:[5->13,7->11,7->17,9->12] | 0.40 | 384.919463087 | 26 | 149 | 0 | 0 |
| Path 69 | C00031->C00002:[4->11,7->3] | 0.20 | 370.880503145 | 25 | 159 | 0 | 0 |
| Path 70 | C00031->C00002:[4->10,4->11,4->2,4->4,7->10,7->11,7->2,7->4] | 0.40 | 372.089820359 | 34 | 167 | 0 | 1 |
| Path 71 | C00031->C00002:[4->10,4->11,4->2,4->4] | 0.40 | 366.141891892 | 25 | 148 | 0 | 1 |
| Path 72 | C00031->C00002:[9->10,9->11] | 0.20 | 313.872611465 | 17 | 157 | 0 | 0 |
| Path 73 | C00031->C00002:[1->12,2->13,4->17,5->8,7->3,9->11] | 0.60 | 377.83125 | 27 | 160 | 0 | 0 |
| Path 74 | C00031->C00002:[1->12,2->13,4->17,5->8,7->11] | 0.50 | 368.077419355 | 24 | 155 | 0 | 0 |
| Path 75 | C00031->C00002:[5->13,7->17,9->11,9->12] | 0.40 | 353.32748538 | 29 | 171 | 0 | 0 |
| Path 76 | C00031->C00002:[4->10,4->11,4->2,4->4,7->10,7->11,7->2,7->4] | 0.40 | 353.92746114 | 40 | 193 | 0 | 1 |
| Path 77 | C00031->C00002:[9->11] | 0.10 | 316.623376623 | 16 | 154 | 0 | 0 |
| Path 78 | C00031->C00002:[4->10,4->11,4->2,4->4,7->10,7->11,7->2,7->4] | 0.40 | 413.613333333 | 37 | 75 | 0 | 1 |
| Path 79 | C00031->C00002:[4->10,4->11,4->2,4->4] | 0.40 | 343.50273224 | 33 | 183 | 0 | 1 |
| Path 80 | C00031->C00002:[4->10,4->2,7->10,7->11,7->2,7->4] | 0.40 | 372.8375 | 32 | 160 | 0 | 1 |
| Path 81 | C00031->C00002:[1->12,2->13,4->17,5->8,7->11,7->3] | 0.60 | 370.487012987 | 24 | 154 | 0 | 0 |
| Path 82 | C00031->C00002:[1->12,2->13,4->17,5->3,5->8,9->11] | 0.60 | 350.029069767 | 26 | 172 | 0 | 0 |
| Path 83 | C00031->C00002:[1->12,2->13,4->17,5->8,7->3] | 0.50 | 368.216216216 | 20 | 148 | 0 | 0 |
| Path 84 | C00031->C00002:[1->12,2->13,4->17,5->8,7->11] | 0.50 | 354.376712329 | 19 | 146 | 0 | 0 |
| Path 85 | C00031->C00002:[5->13,7->17,9->11,9->12] | 0.40 | 355.450292398 | 28 | 171 | 0 | 0 |
| Path 86 | C00031->C00002:[7->10,7->11] | 0.20 | 330.695652174 | 15 | 138 | 0 | 0 |
| Path 87 | C00031->C00002:[4->10,4->11,4->2,4->4] | 0.40 | 379.836477987 | 33 | 159 | 0 | 1 |
| Path 88 | C00031->C00002:[7->10,7->11] | 0.20 | 545.037037037 | 19 | 27 | 0 | 0 |
| Path 89 | C00031->C00002:[4->10,4->2,7->11,7->4] | 0.40 | 539.275862069 | 38 | 58 | 0 | 1 |
| Path 90 | C00031->C00002:[4->10,4->11,4->2,4->4,7->10,7->11,7->2,7->4] | 0.40 | 352.049450549 | 35 | 182 | 0 | 1 |
| Path 91 | C00031->C00002:[4->10,4->11,4->2,4->4,7->10,7->11,7->2,7->4] | 0.40 | 357.348717949 | 41 | 195 | 0 | 1 |
| Path 92 | C00031->C00002:[4->10,4->11,7->10,7->11] | 0.20 | 346.830985915 | 19 | 142 | 0 | 0 |
| Path 93 | C00031->C00002:[1->12,2->13,4->17,5->8,7->11,7->3] | 0.60 | 543.395348837 | 25 | 43 | 0 | 0 |
| Path 94 | C00031->C00002:[1->12,2->13,4->11,4->17,5->8,9->3] | 0.60 | 333.551020408 | 20 | 147 | 0 | 0 |
| Path 95 | C00031->C00002:[4->10,4->11,4->2,4->4,7->10,7->11,7->2,7->4] | 0.40 | 384.819767442 | 38 | 172 | 0 | 1 |
| Path 96 | C00031->C00002:[7->10,7->11] | 0.20 | 342.383561644 | 20 | 146 | 0 | 0 |
| Path 97 | C00031->C00002:[7->10,7->11] | 0.20 | 346.531468531 | 20 | 143 | 0 | 0 |
| Path 98 | C00031->C00002:[4->10,4->11,4->2,4->4] | 0.40 | 377.393548387 | 29 | 155 | 0 | 1 |
| Path 99 | C00031->C00002:[7->10,7->11] | 0.20 | 336.64375 | 24 | 160 | 0 | 0 |
| Path 100 | C00031->C00002:[1->12,2->13,4->17,5->3,5->8,7->11] | 0.60 | 363.131944444 | 20 | 144 | 0 | 0 |
| Path 101 | C00031->C00002:[7->10,7->11] | 0.20 | 348.232394366 | 19 | 142 | 0 | 0 |
| Path 102 | C00031->C00002:[4->10,4->2,7->10,7->11,7->2,7->4] | 0.40 | 529.897959184 | 33 | 49 | 0 | 1 |
| Path 103 | C00031->C00002:[4->10,4->11,4->2,4->4] | 0.40 | 385.529032258 | 32 | 155 | 0 | 1 |
| Path 104 | C00031->C00002:[5->13,7->11,7->17,9->12] | 0.40 | 364.322580645 | 26 | 155 | 0 | 0 |
| Path 105 | C00031->C00002:[7->11] | 0.10 | 332.220588235 | 12 | 136 | 0 | 0 |
| Path 106 | C00031->C00002:[4->10,4->11,4->2,4->4] | 0.40 | 361.961956522 | 37 | 184 | 0 | 1 |
| Path 107 | C00031->C00002:[4->10,4->11,4->2,4->4,7->10,7->11,7->2,7->4] | 0.40 | 541.622641509 | 35 | 53 | 0 | 1 |
| Path 108 | C00031->C00002:[7->10,7->11] | 0.20 | 339.262068966 | 19 | 145 | 0 | 0 |
| Path 109 | C00031->C00002:[4->10,4->11] | 0.20 | 363.445945946 | 23 | 148 | 0 | 0 |
| Path 110 | C00031->C00002:[1->12,2->13,4->17,5->8,7->11] | 0.50 | 363.601398601 | 19 | 143 | 0 | 0 |
| Path 111 | C00031->C00002:[1->12,2->13,4->11,4->17,5->8,7->3] | 0.60 | 366.596153846 | 24 | 156 | 0 | 0 |
| Path 112 | C00031->C00002:[1->12,2->13,4->17,5->3,5->8,7->11] | 0.60 | 360.111888112 | 20 | 143 | 0 | 0 |
| Path 113 | C00031->C00002:[4->10,4->11,4->2,4->4] | 0.40 | 358.453551913 | 37 | 183 | 0 | 1 |
| Path 114 | C00031->C00002:[5->13,7->11,7->17,9->12] | 0.40 | 553.512195122 | 26 | 41 | 0 | 0 |
| Path 115 | C00031->C00002:[4->10,4->11,7->10,7->11] | 0.20 | 345.979310345 | 21 | 145 | 0 | 0 |
| Path 116 | C00031->C00002:[4->10,4->11,4->2,4->4] | 0.40 | 376.588607595 | 32 | 158 | 0 | 1 |
| Path 117 | C00031->C00002:[4->10,4->11,4->2,4->4,7->10,7->11,7->2,7->4] | 0.40 | 350.356020942 | 39 | 191 | 0 | 1 |
| Path 118 | C00031->C00002:[1->12,2->13,4->17,5->3,5->8,7->11] | 0.60 | 353.985915493 | 18 | 142 | 0 | 0 |
| Path 119 | C00031->C00002:[7->10,7->11] | 0.20 | 342.316176471 | 17 | 136 | 0 | 0 |
| Path 120 | C00031->C00002:[4->10,4->11,4->2,4->4] | 0.40 | 382.380368098 | 33 | 163 | 0 | 1 |
| Path 121 | C00031->C00002:[4->10,4->11,4->2,4->4] | 0.40 | 373.887417219 | 27 | 151 | 0 | 1 |
| Path 122 | C00031->C00002:[4->10,4->11,7->10,7->11] | 0.20 | 346.173333333 | 22 | 150 | 0 | 0 |
| Path 123 | C00031->C00002:[7->10,7->11] | 0.20 | 336.64375 | 24 | 160 | 0 | 0 |
| Path 124 | C00031->C00002:[4->10,4->11,4->2,4->4,7->10,7->11,7->2,7->4] | 0.40 | 374.060240964 | 33 | 166 | 0 | 1 |
| Path 125 | C00031->C00002:[4->10,4->11,7->10,7->11] | 0.20 | 350.569444444 | 19 | 144 | 0 | 0 |
| Path 126 | C00031->C00002:[7->11,9->10] | 0.20 | 356.137254902 | 21 | 51 | 0 | 0 |
| Path 127 | C00031->C00002:[1->12,2->13,4->17,5->8,9->11] | 0.50 | 345.514450867 | 26 | 173 | 0 | 0 |
| Path 128 | C00031->C00002:[1->12,2->13,4->17,5->8,7->11] | 0.50 | 500.454545455 | 19 | 33 | 0 | 0 |
| Path 129 | C00031->C00002:[7->11,7->3] | 0.20 | 520.238095238 | 25 | 42 | 0 | 0 |
| Path 130 | C00031->C00002:[1->12,2->13,4->17,5->3,5->8,7->11] | 0.60 | 534.325581395 | 22 | 43 | 0 | 0 |
| Path 131 | C00031->C00002:[4->10,4->11,7->10,7->11] | 0.20 | 341.691780822 | 20 | 146 | 0 | 0 |
| Path 132 | C00031->C00002:[1->12,2->13,4->17,5->8,7->3] | 0.50 | 357.346938776 | 19 | 147 | 0 | 0 |
| Path 133 | C00031->C00002:[4->10,4->11,4->2,4->4] | 0.40 | 556.354166667 | 34 | 48 | 0 | 1 |
| Path 134 | C00031->C00002:[4->10,4->11,4->2,4->4,7->10,7->11,7->2,7->4] | 0.40 | 366.459627329 | 31 | 161 | 0 | 1 |
| Path 135 | C00031->C00002:[4->10,4->11,4->2,4->4,7->10,7->11,7->2,7->4] | 0.40 | 522.413793103 | 36 | 58 | 0 | 1 |
| Path 136 | C00031->C00002:[7->10,7->11] | 0.20 | 338.463768116 | 17 | 138 | 0 | 0 |
| Path 137 | C00031->C00002:[7->10,7->11] | 0.20 | 336.544776119 | 14 | 134 | 0 | 0 |
| Path 138 | C00031->C00002:[4->10,4->11,4->2,4->4,7->10,7->11,7->2,7->4] | 0.40 | 427.943661972 | 36 | 71 | 0 | 1 |
| Path 139 | C00031->C00002:[1->12,2->13,4->17,5->8,7->11,7->3] | 0.60 | 368.981132075 | 27 | 159 | 0 | 0 |
| Path 140 | C00031->C00002:[4->10,4->11,4->2,4->4,7->10,7->11,7->2,7->4] | 0.40 | 377.023529412 | 36 | 170 | 0 | 1 |
| Path 141 | C00031->C00002:[7->10,7->11] | 0.20 | 508.387096774 | 20 | 31 | 0 | 0 |
| Path 142 | C00031->C00002:[4->10,4->11,4->2,4->4] | 0.40 | 350.768421053 | 38 | 190 | 0 | 1 |
| Path 143 | C00031->C00002:[1->12,2->13,4->17,5->8,7->11,7->3] | 0.60 | 372.22147651 | 23 | 149 | 0 | 0 |
| Path 144 | C00031->C00002:[1->12,2->13,4->17,5->8,7->11] | 0.50 | 355.734265734 | 18 | 143 | 0 | 0 |
| Path 145 | C00031->C00002:[1->12,2->13,4->17,5->8,7->11] | 0.50 | 354.823129252 | 20 | 147 | 0 | 0 |
| Path 146 | C00031->C00002:[1->12,2->13,4->17,5->8,7->11,7->3] | 0.60 | 373.251655629 | 24 | 151 | 0 | 0 |
| Path 147 | C00031->C00002:[5->13,7->11,7->17,9->12] | 0.40 | 570.790697674 | 27 | 43 | 0 | 0 |
| Path 148 | C00031->C00002:[1->12,2->13,4->17,5->8,7->11] | 0.50 | 369.72 | 23 | 150 | 0 | 0 |
| Path 149 | C00031->C00002:[1->12,2->13,4->11,4->17,5->3,5->8] | 0.60 | 368.637583893 | 21 | 149 | 0 | 0 |
| Path 150 | C00031->C00002:[1->12,2->13,4->17,5->8,7->11,7->3] | 0.60 | 375.888198758 | 28 | 161 | 0 | 0 |
| Path 151 | C00031->C00002:[4->10,4->11,4->2,4->4] | 0.40 | 386.715151515 | 36 | 165 | 0 | 1 |
| Path 152 | C00031->C00002:[1->12,2->13,4->11,4->17,5->8,7->3] | 0.60 | 373.664556962 | 25 | 158 | 0 | 0 |
| Path 153 | C00031->C00002:[1->12,2->13,4->11,4->17,5->8] | 0.50 | 358.153333333 | 20 | 150 | 0 | 0 |
| Path 154 | C00031->C00002:[4->10,4->11,4->2,4->4,7->11,7->4] | 0.40 | 511.596153846 | 32 | 52 | 0 | 1 |
| Path 155 | C00031->C00002:[4->10,4->11] | 0.20 | 352.26618705 | 17 | 139 | 0 | 0 |
| Path 156 | C00031->C00002:[4->10,4->11] | 0.20 | 327.075581395 | 23 | 172 | 0 | 0 |
| Path 157 | C00031->C00002:[4->10,4->11,7->10,7->11] | 0.20 | 347.021276596 | 18 | 141 | 0 | 0 |
| Path 158 | C00031->C00002:[1->12,2->13,4->17,5->8,7->3,9->11] | 0.60 | 353.296089385 | 30 | 179 | 0 | 0 |
| Path 159 | C00031->C00002:[5->13,7->11,7->17,9->12] | 0.40 | 573.3 | 26 | 40 | 0 | 0 |
| Path 160 | C00031->C00002:[4->10,4->11,4->2,4->4,7->10,7->11,7->2,7->4] | 0.40 | 351.160427807 | 36 | 187 | 0 | 1 |
| Path 161 | C00031->C00002:[1->12,2->13,4->17,5->3,5->8,7->11] | 0.60 | 367.954545455 | 21 | 154 | 0 | 0 |
| Path 162 | C00031->C00002:[7->10,7->11] | 0.20 | 337.446428571 | 25 | 168 | 0 | 0 |
| Path 163 | C00031->C00002:[4->10,4->11,4->2,4->4] | 0.40 | 367.082417582 | 39 | 182 | 0 | 1 |
| Path 164 | C00031->C00002:[4->10,4->11,4->2,4->4] | 0.40 | 386.524390244 | 35 | 164 | 0 | 1 |
| Path 165 | C00031->C00002:[9->10,9->11] | 0.20 | 314.024691358 | 18 | 162 | 0 | 0 |
| Path 166 | C00031->C00002:[7->10,7->11] | 0.20 | 482.055555556 | 21 | 36 | 0 | 0 |
| Path 167 | C00031->C00002:[7->10,7->11] | 0.20 | 351.962406015 | 17 | 133 | 0 | 0 |
| Path 168 | C00031->C00002:[4->10,4->11,4->2,4->4,7->11,7->4] | 0.40 | 342.262032086 | 36 | 187 | 0 | 1 |
| Path 169 | C00031->C00002:[1->12,2->13,4->17,5->8,7->11] | 0.50 | 521.533333333 | 18 | 30 | 0 | 0 |
| Path 170 | C00031->C00002:[4->10,4->11,4->2,4->4,7->11,7->4] | 0.40 | 341.85483871 | 35 | 186 | 0 | 1 |
| Path 171 | C00031->C00002:[1->12,2->13,4->17,5->8,7->11,7->3] | 0.60 | 361.503267974 | 24 | 153 | 0 | 0 |
| Path 172 | C00031->C00002:[4->10,4->11,4->2,4->4,7->10,7->11,7->2,7->4] | 0.40 | 343.666666667 | 36 | 186 | 0 | 1 |
| Path 173 | C00031->C00002:[7->10,7->11] | 0.20 | 315.036585366 | 21 | 164 | 0 | 0 |
| Path 174 | C00031->C00002:[4->10,4->11,4->2,4->4,7->10,7->11,7->2,7->4] | 0.40 | 541.2 | 30 | 45 | 0 | 1 |
| Path 175 | C00031->C00002:[1->12,2->13,4->11,4->17,5->3,5->8] | 0.60 | 371.196202532 | 22 | 158 | 0 | 0 |
| Path 176 | C00031->C00002:[4->10,4->11,7->10,7->11] | 0.20 | 346.054794521 | 19 | 146 | 0 | 0 |
| Path 177 | C00031->C00002:[5->13,7->11,7->17,9->12] | 0.40 | 371.464968153 | 27 | 157 | 0 | 0 |
| Path 178 | C00031->C00002:[7->10,7->11] | 0.20 | 319.360946746 | 24 | 169 | 0 | 0 |
| Path 179 | C00031->C00002:[7->10,7->11] | 0.20 | 496.0 | 15 | 23 | 0 | 0 |
| Path 180 | C00031->C00002:[7->11,9->10] | 0.20 | 320.074074074 | 20 | 162 | 0 | 0 |
| Path 181 | C00031->C00002:[7->10,7->11] | 0.20 | 312.09202454 | 20 | 163 | 0 | 0 |
| Path 182 | C00031->C00002:[7->10,7->11] | 0.20 | 495.78125 | 21 | 32 | 0 | 0 |
| Path 183 | C00031->C00002:[1->12,2->13,4->17,5->8,9->11] | 0.50 | 348.626436782 | 27 | 174 | 0 | 0 |
| Path 184 | C00031->C00002:[9->10,9->11] | 0.20 | 320.691823899 | 19 | 159 | 0 | 0 |
| Path 185 | C00031->C00002:[1->12,2->13,4->17,5->3,5->8,9->11] | 0.60 | 347.150289017 | 27 | 173 | 0 | 0 |
| Path 186 | C00031->C00002:[4->10,4->11,4->2,4->4,7->10,7->11,7->2,7->4] | 0.40 | 347.754010695 | 37 | 187 | 0 | 1 |
| Path 187 | C00031->C00002:[5->13,7->11,7->17,9->12] | 0.40 | 570.790697674 | 27 | 43 | 0 | 0 |
| Path 188 | C00031->C00002:[9->11] | 0.10 | 311.529411765 | 13 | 153 | 0 | 0 |
| Path 189 | C00031->C00002:[4->10,4->11,4->2,4->4,7->11,7->4] | 0.40 | 379.213872832 | 38 | 173 | 0 | 1 |
| Path 190 | C00031->C00002:[1->12,2->13,4->17,5->3,5->8,9->11] | 0.60 | 337.618181818 | 22 | 165 | 0 | 0 |
| Path 191 | C00031->C00002:[7->11,7->3] | 0.20 | 366.258503401 | 22 | 147 | 0 | 0 |
| Path 192 | C00031->C00002:[7->10,7->11,9->10,9->11] | 0.20 | 319.611764706 | 24 | 170 | 0 | 0 |
| Path 193 | C00031->C00002:[1->12,2->13,4->17,5->3,5->8,7->11] | 0.60 | 567.978723404 | 26 | 47 | 0 | 0 |
| Path 194 | C00031->C00002:[4->10,4->11,4->2,4->4] | 0.40 | 351.366666667 | 33 | 180 | 0 | 1 |
| Path 195 | C00031->C00002:[7->10,7->11] | 0.20 | 325.695652174 | 15 | 138 | 0 | 0 |
| Path 196 | C00031->C00002:[7->11,7->3] | 0.20 | 372.097402597 | 26 | 154 | 0 | 0 |
| Path 197 | C00031->C00002:[1->12,2->13,4->17,5->8,7->11,7->3] | 0.60 | 524.780487805 | 24 | 41 | 0 | 0 |
| Path 198 | C00031->C00002:[5->13,7->11,7->17,9->12] | 0.40 | 535.456521739 | 28 | 46 | 0 | 0 |
| Path 199 | C00031->C00002:[4->10,4->2,7->11,7->4] | 0.40 | 426.150684932 | 36 | 73 | 0 | 1 |
| Path 200 | C00031->C00002:[4->10,4->11,4->2,4->4,7->10,7->11,7->2,7->4] | 0.40 | 354.367021277 | 38 | 188 | 0 | 1 |
| Path 201 | C00031->C00002:[4->10,4->11,4->2,4->4,7->10,7->11,7->2,7->4] | 0.40 | 368.777777778 | 31 | 162 | 0 | 1 |
| Path 202 | C00031->C00002:[4->10,4->2,7->10,7->11,7->2,7->4] | 0.40 | 545.392156863 | 34 | 51 | 0 | 1 |
| Path 203 | C00031->C00002:[4->10,4->11,4->2,4->4,7->10,7->11,7->2,7->4] | 0.40 | 518.96 | 31 | 50 | 0 | 1 |
| Path 204 | C00031->C00002:[7->10,7->11] | 0.20 | 509.222222222 | 23 | 36 | 0 | 0 |
| Path 205 | C00031->C00002:[7->10,7->11] | 0.20 | 345.594594595 | 21 | 148 | 0 | 0 |
| Path 206 | C00031->C00002:[4->10,4->11,4->2,4->4] | 0.40 | 362.905027933 | 36 | 179 | 0 | 1 |
| Path 207 | C00031->C00002:[7->10,7->11] | 0.20 | 496.0 | 15 | 23 | 0 | 0 |
| Path 208 | C00031->C00002:[1->12,2->13,4->17,5->8,7->3] | 0.50 | 361.762237762 | 18 | 143 | 0 | 0 |
| Path 209 | C00031->C00002:[4->10,4->11,7->10,7->11] | 0.20 | 481.0 | 20 | 35 | 0 | 0 |
| Path 210 | C00031->C00002:[4->10,4->11,4->2,4->4,7->10,7->11,7->2,7->4] | 0.40 | 381.295180723 | 35 | 166 | 0 | 1 |
| Path 211 | C00031->C00002:[4->10,4->11,4->2,4->4,7->10,7->11,7->2,7->4] | 0.40 | 363.78125 | 30 | 160 | 0 | 1 |
| Path 212 | C00031->C00002:[4->10,4->11,4->2,4->4,7->10,7->11,7->2,7->4] | 0.40 | 344.956756757 | 35 | 185 | 0 | 1 |
| Path 213 | C00031->C00002:[7->11,7->3] | 0.20 | 357.226666667 | 22 | 150 | 0 | 0 |
| Path 214 | C00031->C00002:[1->12,2->13,4->17,5->3,5->8,9->11] | 0.60 | 347.452380952 | 25 | 168 | 0 | 0 |
| Path 215 | C00031->C00002:[7->10,7->11] | 0.20 | 509.222222222 | 23 | 36 | 0 | 0 |
| Path 216 | C00031->C00002:[1->12,2->13,4->11,4->17,5->3,5->8] | 0.60 | 382.639240506 | 23 | 158 | 0 | 0 |
| Path 217 | C00031->C00002:[4->10,4->11,7->10,7->11] | 0.20 | 344.414965986 | 20 | 147 | 0 | 0 |
| Path 218 | C00031->C00002:[7->10,7->11] | 0.20 | 349.730538922 | 28 | 167 | 0 | 0 |
| Path 219 | C00031->C00002:[7->10,7->11,9->10,9->11] | 0.20 | 326.651162791 | 25 | 172 | 0 | 0 |
| Path 220 | C00031->C00002:[4->10,4->11,4->2,4->4,7->10,7->11,7->2,7->4] | 0.40 | 517.309090909 | 35 | 55 | 0 | 1 |
| Path 221 | C00031->C00002:[7->3,9->11] | 0.20 | 372.35443038 | 26 | 158 | 0 | 0 |
| Path 222 | C00031->C00002:[7->10,7->11] | 0.20 | 354.530201342 | 22 | 149 | 0 | 0 |
| Path 223 | C00031->C00002:[1->12,2->13,4->11,4->17,5->8] | 0.50 | 364.837837838 | 20 | 148 | 0 | 0 |
| Path 224 | C00031->C00002:[4->10,4->11,4->2,4->4,7->10,7->11,7->2,7->4] | 0.40 | 506.456140351 | 36 | 57 | 0 | 1 |
| Path 225 | C00031->C00002:[4->10,4->11,4->2,4->4,7->10,7->11,7->2,7->4] | 0.40 | 374.604790419 | 35 | 167 | 0 | 1 |
| Path 226 | C00031->C00002:[4->10,4->11,4->2,4->4,7->10,7->11,7->2,7->4] | 0.40 | 510.843137255 | 32 | 51 | 0 | 1 |
| Path 227 | C00031->C00002:[1->12,2->13,4->17,5->8,7->11,7->3] | 0.60 | 363.190789474 | 23 | 152 | 0 | 0 |
| Path 228 | C00031->C00002:[1->12,2->13,4->17,5->8,7->11,7->3] | 0.60 | 370.44 | 24 | 150 | 0 | 0 |
| Path 229 | C00031->C00002:[5->13,7->17,9->11,9->12] | 0.40 | 368.38150289 | 31 | 173 | 0 | 0 |
| Path 230 | C00031->C00002:[4->10,4->11] | 0.20 | 359.560810811 | 22 | 148 | 0 | 0 |
| Path 231 | C00031->C00002:[1->12,2->13,4->11,4->17,5->3,5->8] | 0.60 | 364.033112583 | 21 | 151 | 0 | 0 |
| Path 232 | C00031->C00002:[4->10,4->11,4->2,4->4,7->10,7->11,7->2,7->4] | 0.40 | 416.662337662 | 38 | 77 | 0 | 1 |
| Path 233 | C00031->C00002:[4->10,4->11,4->2,4->4] | 0.40 | 380.488095238 | 34 | 168 | 0 | 1 |
| Path 234 | C00031->C00002:[4->10,4->11,4->2,4->4] | 0.40 | 363.85026738 | 41 | 187 | 0 | 1 |
| Path 235 | C00031->C00002:[1->12,2->13,4->17,5->8,9->11] | 0.50 | 329.452380952 | 21 | 168 | 0 | 0 |
| Path 236 | C00031->C00002:[4->10,4->11,4->2,4->4] | 0.40 | 366.069148936 | 41 | 188 | 0 | 1 |
| Path 237 | C00031->C00002:[1->12,2->13,4->17,5->8,7->3,9->11] | 0.60 | 336.201149425 | 25 | 174 | 0 | 0 |
| Path 238 | C00031->C00002:[1->12,2->13,4->17,5->3,5->8,7->11] | 0.60 | 569.195121951 | 23 | 41 | 0 | 0 |
| Path 239 | C00031->C00002:[1->12,2->13,4->17,5->8,7->11] | 0.50 | 536.90625 | 19 | 32 | 0 | 0 |
| Path 240 | C00031->C00002:[4->11,7->3] | 0.20 | 360.831168831 | 23 | 154 | 0 | 0 |
| Path 241 | C00031->C00002:[4->10,4->11,4->2,4->4,7->4] | 0.40 | 513.222222222 | 40 | 63 | 0 | 1 |
| Path 242 | C00031->C00002:[1->12,2->13,4->17,5->3,5->8,9->11] | 0.60 | 368.516339869 | 23 | 153 | 0 | 0 |
| Path 243 | C00031->C00002:[1->12,2->13,4->17,5->8,7->11] | 0.50 | 546.75 | 19 | 32 | 0 | 0 |
| Path 244 | C00031->C00002:[7->10,7->11] | 0.20 | 341.748201439 | 18 | 139 | 0 | 0 |
| Path 245 | C00031->C00002:[4->10,4->11,4->2,4->4,7->10,7->11,7->2,7->4] | 0.40 | 377.263157895 | 37 | 171 | 0 | 1 |
| Path 246 | C00031->C00002:[4->10,7->11] | 0.20 | 510.696969697 | 20 | 33 | 0 | 0 |
| Path 247 | C00031->C00002:[4->10,4->11,7->10,7->11] | 0.20 | 345.316901408 | 19 | 142 | 0 | 0 |
| Path 248 | C00031->C00002:[1->12,2->13,4->17,5->8,9->3] | 0.50 | 318.015503876 | 9 | 129 | 0 | 0 |
| Path 249 | C00031->C00002:[4->10,4->11,4->2,4->4,7->10,7->11,7->2,7->4] | 0.40 | 516.980392157 | 32 | 51 | 0 | 1 |
| Path 250 | C00031->C00002:[1->12,2->13,4->17,5->8,7->3,9->11] | 0.60 | 342.892045455 | 26 | 176 | 0 | 0 |
| Path 251 | C00031->C00002:[7->10,7->11,9->10,9->11] | 0.20 | 373.232142857 | 25 | 56 | 0 | 0 |
| Path 252 | C00031->C00002:[1->12,2->13,4->17,5->8,7->11,7->3] | 0.60 | 372.092715232 | 23 | 151 | 0 | 0 |
| Path 253 | C00031->C00002:[7->10,7->11] | 0.20 | 347.231292517 | 20 | 147 | 0 | 0 |
| Path 254 | C00031->C00002:[1->12,2->13,4->17,5->8,9->11] | 0.50 | 335.084848485 | 21 | 165 | 0 | 0 |
| Path 255 | C00031->C00002:[7->10,7->11] | 0.20 | 482.185185185 | 18 | 27 | 0 | 0 |
| Path 256 | C00031->C00002:[1->12,2->13,4->17,5->8,7->11] | 0.50 | 546.864864865 | 23 | 37 | 0 | 0 |
| Path 257 | C00031->C00002:[5->13,7->11,7->17,9->12] | 0.40 | 366.006493506 | 25 | 154 | 0 | 0 |
| Path 258 | C00031->C00002:[4->10,4->11,4->2,4->4,7->10,7->11,7->2,7->4] | 0.40 | 356.675257732 | 41 | 194 | 0 | 1 |
| Path 259 | C00031->C00002:[4->10,4->11,4->2,4->4,7->10,7->11,7->2,7->4] | 0.40 | 349.85106383 | 37 | 188 | 0 | 1 |
| Path 260 | C00031->C00002:[5->13,7->17,9->11,9->12] | 0.40 | 356.554216867 | 27 | 166 | 0 | 0 |
| Path 261 | C00031->C00002:[7->10,7->11] | 0.20 | 350.760869565 | 18 | 138 | 0 | 0 |
| Path 262 | C00031->C00002:[7->10,7->11] | 0.20 | 355.87755102 | 21 | 49 | 0 | 0 |
| Path 263 | C00031->C00002:[4->10,4->11,4->2,4->4,7->10,7->11,7->2,7->4] | 0.40 | 420.727272727 | 38 | 77 | 0 | 1 |
| Path 264 | C00031->C00002:[5->13,7->11,7->17,9->12] | 0.40 | 370.940789474 | 25 | 152 | 0 | 0 |
| Path 265 | C00031->C00002:[7->10,7->11] | 0.20 | 347.945454545 | 23 | 55 | 0 | 0 |
| Path 266 | C00031->C00002:[7->11,7->3] | 0.20 | 355.556291391 | 23 | 151 | 0 | 0 |
| Path 267 | C00031->C00002:[7->10,7->11] | 0.20 | 321.154320988 | 21 | 162 | 0 | 0 |
| Path 268 | C00031->C00002:[4->10,4->11,4->2,4->4,7->10,7->11,7->2,7->4] | 0.40 | 508.035714286 | 35 | 56 | 0 | 1 |
| Path 269 | C00031->C00002:[4->10,4->11,4->2,4->4] | 0.40 | 357.113989637 | 40 | 193 | 0 | 1 |
| Path 270 | C00031->C00002:[7->3,9->11] | 0.20 | 351.566473988 | 28 | 173 | 0 | 0 |
| Path 271 | C00031->C00002:[1->12,2->13,4->10,4->11,4->17,5->8,9->3] | 0.70 | 338.589403974 | 24 | 151 | 0 | 0 |
| Path 272 | C00031->C00002:[4->10,4->11,4->2,4->4] | 0.40 | 357.333333333 | 39 | 189 | 0 | 1 |
| Path 273 | C00031->C00002:[4->10,4->11,4->2,4->4,7->10,7->11,7->2,7->4] | 0.40 | 375.19047619 | 35 | 168 | 0 | 1 |
| Path 274 | C00031->C00002:[1->12,2->13,4->17,5->3,5->8,9->11] | 0.60 | 335.729885057 | 26 | 174 | 0 | 0 |
| Path 275 | C00031->C00002:[4->10,4->11,4->2,4->4,7->11,7->4] | 0.40 | 511.596153846 | 32 | 52 | 0 | 1 |
| Path 276 | C00031->C00002:[7->11,7->3] | 0.20 | 366.208053691 | 22 | 149 | 0 | 0 |
| Path 277 | C00031->C00002:[7->3,9->11] | 0.20 | 374.104575163 | 25 | 153 | 0 | 0 |
| Path 278 | C00031->C00002:[1->12,2->13,4->17,5->3,5->8,9->11] | 0.60 | 325.922155689 | 21 | 167 | 0 | 0 |
| Path 279 | C00031->C00002:[1->12,2->13,4->17,5->8,9->11] | 0.50 | 354.827586207 | 27 | 174 | 0 | 0 |
| Path 280 | C00031->C00002:[4->10,4->11,4->2,4->4] | 0.40 | 362.612903226 | 40 | 186 | 0 | 1 |
| Path 281 | C00031->C00002:[5->13,7->17,9->11,9->12] | 0.40 | 362.869047619 | 29 | 168 | 0 | 0 |
| Path 282 | C00031->C00002:[1->12,2->13,4->17,5->3,5->8,7->11] | 0.60 | 558.393939394 | 20 | 33 | 0 | 0 |
| Path 283 | C00031->C00002:[4->10,4->11,4->2,4->4] | 0.40 | 380.710059172 | 35 | 169 | 0 | 1 |
| Path 284 | C00031->C00002:[5->13,7->11,7->17,9->12] | 0.40 | 545.022222222 | 27 | 45 | 0 | 0 |
| Path 285 | C00031->C00002:[4->10,4->11,4->2,4->4] | 0.40 | 433.935897436 | 40 | 78 | 0 | 1 |
| Path 286 | C00031->C00002:[4->10,4->11,4->2,4->4] | 0.40 | 350.416666667 | 39 | 192 | 0 | 1 |
| Path 287 | C00031->C00002:[7->11,7->3] | 0.20 | 364.493243243 | 23 | 148 | 0 | 0 |
| Path 288 | C00031->C00002:[4->10,4->11] | 0.20 | 353.514925373 | 16 | 134 | 0 | 0 |
| Path 289 | C00031->C00002:[9->11] | 0.10 | 303.897435897 | 13 | 156 | 0 | 0 |
| Path 290 | C00031->C00002:[4->11,5->13,7->17,9->12] | 0.40 | 386.775510204 | 25 | 147 | 0 | 0 |
| Path 291 | C00031->C00002:[4->10,4->11,7->10,7->11] | 0.20 | 508.033333333 | 19 | 30 | 0 | 0 |
| Path 292 | C00031->C00002:[4->10,4->11,4->2,4->4,7->10,7->11,7->2,7->4] | 0.40 | 367.933333333 | 34 | 165 | 0 | 1 |
| Path 293 | C00031->C00002:[1->12,2->13,4->17,5->8,7->11] | 0.50 | 558.179487179 | 24 | 39 | 0 | 0 |
| Path 294 | C00031->C00002:[1->12,2->13,4->17,5->3,5->8,9->11] | 0.60 | 343.299435028 | 27 | 177 | 0 | 0 |
| Path 295 | C00031->C00002:[1->12,2->13,4->17,5->8,7->3,9->11] | 0.60 | 379.735483871 | 26 | 155 | 0 | 0 |
| Path 296 | C00031->C00002:[7->10,7->11] | 0.20 | 360.820512821 | 26 | 156 | 0 | 0 |
| Path 297 | C00031->C00002:[7->11,7->3] | 0.20 | 370.421383648 | 27 | 159 | 0 | 0 |
| Path 298 | C00031->C00002:[7->11] | 0.10 | 350.364341085 | 13 | 129 | 0 | 0 |
| Path 299 | C00031->C00002:[1->12,2->13,4->11,4->17,5->3,5->8] | 0.60 | 367.019480519 | 22 | 154 | 0 | 0 |
| Path 300 | C00031->C00002:[9->11] | 0.10 | 348.671532847 | 14 | 137 | 0 | 0 |
| Path 301 | C00031->C00002:[4->10,4->11,4->2,4->4] | 0.40 | 347.221621622 | 34 | 185 | 0 | 1 |
| Path 302 | C00031->C00002:[4->10,4->11,4->2,4->4,7->10,7->11,7->2,7->4] | 0.40 | 353.114130435 | 36 | 184 | 0 | 1 |
| Path 303 | C00031->C00002:[5->13,7->11,7->17,9->12] | 0.40 | 668.787878788 | 26 | 33 | 0 | 0 |
| Path 304 | C00031->C00002:[4->10,4->11,4->2,4->4,7->10,7->11,7->2,7->4] | 0.40 | 413.613333333 | 37 | 75 | 0 | 1 |
| Path 305 | C00031->C00002:[7->11,7->3] | 0.20 | 510.128205128 | 23 | 39 | 0 | 0 |
| Path 306 | C00031->C00002:[1->12,2->13,4->17,5->8,9->11] | 0.50 | 348.970414201 | 25 | 169 | 0 | 0 |
| Path 307 | C00031->C00002:[4->10,4->11,4->2,4->4,7->10,7->11,7->2,7->4] | 0.40 | 365.124223602 | 31 | 161 | 0 | 1 |
| Path 308 | C00031->C00002:[1->12,2->13,2->3,4->17,5->8] | 0.50 | 357.006849315 | 15 | 146 | 0 | 0 |
| Path 309 | C00031->C00002:[4->10,4->11,4->2,4->4,7->11,7->4] | 0.40 | 412.285714286 | 37 | 77 | 0 | 1 |
| Path 310 | C00031->C00002:[4->10,4->11,4->2,4->4] | 0.40 | 347.190217391 | 34 | 184 | 0 | 1 |
| Path 311 | C00031->C00002:[9->11] | 0.10 | 323.487179487 | 16 | 156 | 0 | 0 |
| Path 312 | C00031->C00002:[1->12,2->13,4->17,5->3,5->8,9->11] | 0.60 | 340.676136364 | 23 | 176 | 0 | 0 |
| Path 313 | C00031->C00002:[4->11,5->13,7->17,9->12] | 0.40 | 384.539473684 | 26 | 152 | 0 | 0 |
| Path 314 | C00031->C00002:[1->12,2->13,4->17,5->3,5->8,9->11] | 0.60 | 333.445121951 | 21 | 164 | 0 | 0 |
| Path 315 | C00031->C00002:[7->11] | 0.10 | 639.333333333 | 14 | 18 | 0 | 0 |
| Path 316 | C00031->C00002:[1->12,2->13,4->17,5->8,7->11] | 0.50 | 526.447368421 | 22 | 38 | 0 | 0 |
| Path 317 | C00031->C00002:[7->11,7->3] | 0.20 | 549.162790698 | 27 | 43 | 0 | 0 |
| Path 318 | C00031->C00002:[4->10,4->11] | 0.20 | 358.22875817 | 23 | 153 | 0 | 0 |
| Path 319 | C00031->C00002:[1->12,2->13,4->17,5->8,7->11,7->3] | 0.60 | 560.777777778 | 28 | 45 | 0 | 0 |
| Path 320 | C00031->C00002:[4->10,4->11,4->2,4->4] | 0.40 | 353.227979275 | 40 | 193 | 0 | 1 |
| Path 321 | C00031->C00002:[1->12,2->13,4->17,5->8,7->11] | 0.50 | 559.033333333 | 19 | 30 | 0 | 0 |
| Path 322 | C00031->C00002:[5->13,7->17,9->11,9->12] | 0.40 | 353.28 | 29 | 175 | 0 | 0 |
| Path 323 | C00031->C00002:[4->10,4->11,4->2,4->4] | 0.40 | 355.549450549 | 36 | 182 | 0 | 1 |
| Path 324 | C00031->C00002:[1->12,2->13,4->17,5->3,5->8,7->11] | 0.60 | 350.95890411 | 20 | 146 | 0 | 0 |
| Path 325 | C00031->C00002:[4->10,4->11] | 0.20 | 341.49689441 | 23 | 161 | 0 | 0 |
| Path 326 | C00031->C00002:[4->10,4->2,7->11,7->4] | 0.40 | 539.275862069 | 38 | 58 | 0 | 1 |
| Path 327 | C00031->C00002:[7->10,7->11] | 0.20 | 596.454545455 | 18 | 22 | 0 | 0 |
| Path 328 | C00031->C00002:[4->11,5->13,7->17,9->12] | 0.40 | 387.109677419 | 27 | 155 | 0 | 0 |
| Path 329 | C00031->C00002:[4->10,4->11,4->2,4->4] | 0.40 | 433.935897436 | 40 | 78 | 0 | 1 |
| Path 330 | C00031->C00002:[1->12,2->13,4->17,5->3,5->8,7->11] | 0.60 | 377.91503268 | 22 | 153 | 0 | 0 |
| Path 331 | C00031->C00002:[1->12,2->13,4->11,4->17,5->8] | 0.50 | 367.204081633 | 20 | 147 | 0 | 0 |
| Path 332 | C00031->C00002:[7->11,9->10] | 0.20 | 312.5125 | 19 | 160 | 0 | 0 |
| Path 333 | C00031->C00002:[7->10,7->11] | 0.20 | 596.454545455 | 18 | 22 | 0 | 0 |
| Path 334 | C00031->C00002:[1->12,2->13,4->17,5->8,7->11] | 0.50 | 346.95862069 | 19 | 145 | 0 | 0 |
| Path 335 | C00031->C00002:[4->10,4->11,4->2,4->4,7->10,7->11,7->2,7->4] | 0.40 | 386.608433735 | 36 | 166 | 0 | 1 |
| Path 336 | C00031->C00002:[1->12,2->13,4->17,5->8,9->3] | 0.50 | 314.286821705 | 9 | 129 | 0 | 0 |
| Path 337 | C00031->C00002:[4->10,4->11,4->2,4->4,7->10,7->11,7->2,7->4] | 0.40 | 373.793939394 | 32 | 165 | 0 | 1 |
| Path 338 | C00031->C00002:[1->12,2->13,4->17,5->8,7->3,9->11] | 0.60 | 356.261111111 | 31 | 180 | 0 | 0 |
| Path 339 | C00031->C00002:[1->12,2->13,4->11,4->17,5->8,7->3] | 0.60 | 375.45751634 | 24 | 153 | 0 | 0 |
| Path 340 | C00031->C00002:[4->10,4->11,7->10,7->11] | 0.20 | 338.013888889 | 18 | 144 | 0 | 0 |
| Path 341 | C00031->C00002:[1->12,2->13,4->17,5->3,5->8,7->11] | 0.60 | 552.675 | 21 | 40 | 0 | 0 |
| Path 342 | C00031->C00002:[4->10,4->11,4->2,4->4] | 0.40 | 564.530612245 | 34 | 49 | 0 | 1 |
| Path 343 | C00031->C00002:[1->12,2->13,4->17,5->3,5->8,7->11] | 0.60 | 361.916666667 | 19 | 144 | 0 | 0 |
| Path 344 | C00031->C00002:[7->11,7->3] | 0.20 | 363.356687898 | 26 | 157 | 0 | 0 |
| Path 345 | C00031->C00002:[4->10,4->11,4->2,4->4,7->10,7->11,7->2,7->4] | 0.40 | 369.933333333 | 33 | 165 | 0 | 1 |
| Path 346 | C00031->C00002:[4->10,4->11,4->2,4->4,7->10,7->11,7->2,7->4] | 0.40 | 501.924528302 | 34 | 53 | 0 | 1 |
| Path 347 | C00031->C00002:[1->12,2->13,4->17,5->3,5->8,7->11] | 0.60 | 358.716216216 | 21 | 148 | 0 | 0 |
| Path 348 | C00031->C00002:[1->12,2->13,4->17,5->3,5->8,7->11] | 0.60 | 369.509933775 | 20 | 151 | 0 | 0 |
| Path 349 | C00031->C00002:[1->12,2->13,4->17,5->8,7->11] | 0.50 | 519.714285714 | 24 | 42 | 0 | 0 |
| Path 350 | C00031->C00002:[4->10,4->11] | 0.20 | 584.655172414 | 22 | 29 | 0 | 0 |
| Path 351 | C00031->C00002:[7->10,7->11] | 0.20 | 428.923076923 | 15 | 26 | 0 | 0 |
| Path 352 | C00031->C00002:[4->10,4->11,4->2,4->4,7->4] | 0.40 | 509.830188679 | 33 | 53 | 0 | 1 |
| Path 353 | C00031->C00002:[7->10,7->11] | 0.20 | 362.21192053 | 25 | 151 | 0 | 0 |
| Path 354 | C00031->C00002:[4->10,4->11,4->2,4->4] | 0.40 | 355.23655914 | 35 | 186 | 0 | 1 |
| Path 355 | C00031->C00002:[1->12,2->13,4->17,5->3,5->8] | 0.50 | 340.947761194 | 11 | 134 | 0 | 0 |
| Path 356 | C00031->C00002:[7->3,9->11] | 0.20 | 330.686046512 | 24 | 172 | 0 | 0 |
| Path 357 | C00031->C00002:[4->11] | 0.10 | 350.930232558 | 11 | 129 | 0 | 0 |
| Path 358 | C00031->C00002:[7->3,9->11] | 0.20 | 340.033519553 | 29 | 179 | 0 | 0 |
| Path 359 | C00031->C00002:[1->12,2->13,4->17,5->3,5->8,7->11] | 0.60 | 580.8 | 22 | 40 | 0 | 0 |
| Path 360 | C00031->C00002:[5->13,7->17,9->11,9->12] | 0.40 | 383.435582822 | 30 | 163 | 0 | 0 |
| Path 361 | C00031->C00002:[4->10,4->11,4->2,4->4] | 0.40 | 347.058823529 | 35 | 187 | 0 | 1 |
| Path 362 | C00031->C00002:[4->10,4->11,4->2,4->4] | 0.40 | 362.612903226 | 40 | 186 | 0 | 1 |
| Path 363 | C00031->C00002:[1->12,2->13,4->17,5->3,5->8,7->11] | 0.60 | 366.258064516 | 22 | 155 | 0 | 0 |
| Path 364 | C00031->C00002:[4->10,4->11,4->2,4->4] | 0.40 | 346.819587629 | 39 | 194 | 0 | 1 |
| Path 365 | C00031->C00002:[4->10,4->11,4->2,4->4] | 0.40 | 366.489932886 | 26 | 149 | 0 | 1 |
| Path 366 | C00031->C00002:[1->12,2->13,4->17,5->8,7->3,9->11] | 0.60 | 352.358695652 | 31 | 184 | 0 | 0 |
| Path 367 | C00031->C00002:[1->12,2->13,4->17,5->8,7->3,9->11] | 0.60 | 356.811428571 | 29 | 175 | 0 | 0 |
| Path 368 | C00031->C00002:[7->10,7->11] | 0.20 | 331.023809524 | 25 | 168 | 0 | 0 |
| Path 369 | C00031->C00002:[4->10,4->2,7->11,7->4] | 0.40 | 368.184713376 | 29 | 157 | 0 | 1 |
| Path 370 | C00031->C00002:[1->12,2->13,4->17,5->8,7->11,7->3] | 0.60 | 543.342105263 | 23 | 38 | 0 | 0 |
| Path 371 | C00031->C00002:[1->12,2->13,4->17,5->3,5->8,7->11] | 0.60 | 529.279069767 | 25 | 43 | 0 | 0 |
| Path 372 | C00031->C00002:[1->12,2->13,4->17,5->3,5->8] | 0.50 | 344.421428571 | 14 | 140 | 0 | 0 |
| Path 373 | C00031->C00002:[4->10,4->11,4->2,4->4] | 0.40 | 368.647368421 | 42 | 190 | 0 | 1 |
| Path 374 | C00031->C00002:[4->10,4->11,4->2,4->4,7->10,7->11,7->2,7->4] | 0.40 | 358.608695652 | 37 | 184 | 0 | 1 |
| Path 375 | C00031->C00002:[5->13,7->11,7->17,9->12] | 0.40 | 378.136363636 | 26 | 154 | 0 | 0 |
| Path 376 | C00031->C00002:[1->12,2->13,4->17,5->3,5->8,7->11] | 0.60 | 556.46875 | 21 | 32 | 0 | 0 |
| Path 377 | C00031->C00002:[4->10,4->11,4->2,4->4] | 0.40 | 383.44375 | 33 | 160 | 0 | 1 |
| Path 378 | C00031->C00002:[4->10,4->11,4->2,4->4,7->10,7->11,7->2,7->4] | 0.40 | 510.020408163 | 31 | 49 | 0 | 1 |
| Path 379 | C00031->C00002:[4->10,4->11,4->2,4->4,7->10,7->11,7->2,7->4] | 0.40 | 373.591715976 | 36 | 169 | 0 | 1 |
| Path 380 | C00031->C00002:[1->12,2->13,4->17,5->8,7->11] | 0.50 | 359.160839161 | 19 | 143 | 0 | 0 |
| Path 381 | C00031->C00002:[4->10,4->11,4->2,4->4,7->11,7->4] | 0.40 | 373.093023256 | 38 | 172 | 0 | 1 |
| Path 382 | C00031->C00002:[4->10,4->11,4->2,4->4,7->10,7->11,7->2,7->4] | 0.40 | 514.789473684 | 36 | 57 | 0 | 1 |
| Path 383 | C00031->C00002:[1->12,2->13,4->17,5->8,7->11,7->3] | 0.60 | 368.774193548 | 25 | 155 | 0 | 0 |
| Path 384 | C00031->C00002:[5->13,7->17,9->12] | 0.30 | 364.207407407 | 16 | 135 | 0 | 0 |
| Path 385 | C00031->C00002:[9->10,9->11] | 0.20 | 331.203703704 | 22 | 162 | 0 | 0 |
| Path 386 | C00031->C00002:[7->3,9->11] | 0.20 | 357.219101124 | 30 | 178 | 0 | 0 |
| Path 387 | C00031->C00002:[4->10,4->11] | 0.20 | 316.497005988 | 21 | 167 | 0 | 0 |
| Path 388 | C00031->C00002:[7->10,7->11] | 0.20 | 323.906832298 | 21 | 161 | 0 | 0 |
| Path 389 | C00031->C00002:[1->12,2->13,4->17,5->3,5->8,9->11] | 0.60 | 361.028248588 | 27 | 177 | 0 | 0 |
| Path 390 | C00031->C00002:[4->10,4->11,4->2,4->4,7->10,7->11,7->2,7->4] | 0.40 | 373.056962025 | 30 | 158 | 0 | 1 |
| Path 391 | C00031->C00002:[5->13,5->3,5->8,7->17,9->12] | 0.50 | 366.013245033 | 20 | 151 | 0 | 0 |
| Path 392 | C00031->C00002:[7->10,7->11] | 0.20 | 355.87755102 | 21 | 49 | 0 | 0 |
| Path 393 | C00031->C00002:[1->12,2->13,4->17,5->3,5->8,7->11] | 0.60 | 536.0 | 21 | 36 | 0 | 0 |
| Path 394 | C00031->C00002:[4->10,4->11,4->2,4->4,7->11,7->4] | 0.40 | 412.285714286 | 37 | 77 | 0 | 1 |
| Path 395 | C00031->C00002:[4->10,4->11,4->2,4->4] | 0.40 | 355.572192513 | 36 | 187 | 0 | 1 |
| Path 396 | C00031->C00002:[1->12,2->13,4->17,5->8,7->11] | 0.50 | 544.387096774 | 20 | 31 | 0 | 0 |
| Path 397 | C00031->C00002:[7->10,7->11] | 0.20 | 346.006896552 | 21 | 145 | 0 | 0 |
| Path 398 | C00031->C00002:[4->10,4->11,7->10,7->11] | 0.20 | 350.35862069 | 20 | 145 | 0 | 0 |
| Path 399 | C00031->C00002:[4->10,4->11,4->2,4->4,7->10,7->11,7->2,7->4] | 0.40 | 500.37037037 | 35 | 54 | 0 | 1 |
| Path 400 | C00031->C00002:[4->10,4->11,4->2,4->4,7->10,7->11,7->2,7->4] | 0.40 | 344.064171123 | 37 | 187 | 0 | 1 |
| Path 401 | C00031->C00002:[5->13,7->11,7->17,9->12] | 0.40 | 527.348837209 | 26 | 43 | 0 | 0 |
| Path 402 | C00031->C00002:[1->12,2->13,4->11,4->17,5->8] | 0.50 | 363.333333333 | 21 | 153 | 0 | 0 |
| Path 403 | C00031->C00002:[1->12,2->13,4->17,5->3,5->8,7->11] | 0.60 | 571.032258065 | 20 | 31 | 0 | 0 |
| Path 404 | C00031->C00002:[1->12,2->13,4->17,5->8,7->3] | 0.50 | 347.496644295 | 19 | 149 | 0 | 0 |
| Path 405 | C00031->C00002:[7->11] | 0.10 | 518.454545455 | 12 | 22 | 0 | 0 |
| Path 406 | C00031->C00002:[4->11,7->3] | 0.20 | 368.064102564 | 24 | 156 | 0 | 0 |
| Path 407 | C00031->C00002:[4->10,4->11,4->2,4->4] | 0.40 | 358.505319149 | 38 | 188 | 0 | 1 |
| Path 408 | C00031->C00002:[1->12,2->13,4->17,5->3,5->8,7->11] | 0.60 | 375.096153846 | 22 | 156 | 0 | 0 |
| Path 409 | C00031->C00002:[1->12,2->13,4->17,5->3,5->8,9->11] | 0.60 | 353.387283237 | 27 | 173 | 0 | 0 |
| Path 410 | C00031->C00002:[1->12,2->13,4->17,5->8,7->3,9->11] | 0.60 | 343.596491228 | 25 | 171 | 0 | 0 |
| Path 411 | C00031->C00002:[4->10,4->11,4->2,4->4] | 0.40 | 373.593333333 | 26 | 150 | 0 | 1 |
| Path 412 | C00031->C00002:[7->11] | 0.10 | 639.333333333 | 14 | 18 | 0 | 0 |
| Path 413 | C00031->C00002:[1->12,2->13,4->17,5->3,5->8,9->11] | 0.60 | 349.43715847 | 28 | 183 | 0 | 0 |
| Path 414 | C00031->C00002:[4->10,4->11] | 0.20 | 361.742857143 | 21 | 140 | 0 | 0 |
| Path 415 | C00031->C00002:[1->12,2->13,4->17,5->8] | 0.40 | 336.511278195 | 10 | 133 | 0 | 0 |
| Path 416 | C00031->C00002:[7->10,7->11] | 0.20 | 350.86 | 22 | 50 | 0 | 0 |
| Path 417 | C00031->C00002:[1->12,2->13,4->11,4->17,5->8,9->3] | 0.60 | 336.823129252 | 20 | 147 | 0 | 0 |
| Path 418 | C00031->C00002:[9->10,9->11] | 0.20 | 327.760233918 | 24 | 171 | 0 | 0 |
| Path 419 | C00031->C00002:[1->12,2->13,4->10,4->11,4->17,4->2,4->4,5->8,9->3] | 0.90 | 356.8 | 34 | 165 | 0 | 1 |
| Path 420 | C00031->C00002:[4->10,4->11,4->2,4->4] | 0.40 | 369.677852349 | 26 | 149 | 0 | 1 |
| Path 421 | C00031->C00002:[4->10,4->11,4->2,4->4] | 0.40 | 367.082417582 | 39 | 182 | 0 | 1 |
| Path 422 | C00031->C00002:[9->11] | 0.10 | 325.743421053 | 16 | 152 | 0 | 0 |
| Path 423 | C00031->C00002:[4->10,4->11,4->2,4->4,7->11,7->4] | 0.40 | 499.721311475 | 39 | 61 | 0 | 1 |
| Path 424 | C00031->C00002:[7->11] | 0.10 | 569.652173913 | 15 | 23 | 0 | 0 |
| Path 425 | C00031->C00002:[4->10,4->11,4->2,4->4] | 0.40 | 347.620111732 | 32 | 179 | 0 | 1 |
| Path 426 | C00031->C00002:[1->12,2->13,4->17,5->8,7->11] | 0.50 | 352.680272109 | 20 | 147 | 0 | 0 |
| Path 427 | C00031->C00002:[4->10,4->11,4->2,4->4,7->10,7->11,7->2,7->4] | 0.40 | 427.943661972 | 36 | 71 | 0 | 1 |
| Path 428 | C00031->C00002:[4->10,4->11,4->2,4->4,7->10,7->11,7->2,7->4] | 0.40 | 541.2 | 30 | 45 | 0 | 1 |
| Path 429 | C00031->C00002:[4->10,4->11,4->2,4->4] | 0.40 | 359.565445026 | 42 | 191 | 0 | 1 |
| Path 430 | C00031->C00002:[7->10,7->11] | 0.20 | 319.521212121 | 21 | 165 | 0 | 0 |
| Path 431 | C00031->C00002:[4->10,4->11,4->2,4->4] | 0.40 | 377.12987013 | 28 | 154 | 0 | 1 |
| Path 432 | C00031->C00002:[7->3,9->11] | 0.20 | 338.609467456 | 24 | 169 | 0 | 0 |
| Path 433 | C00031->C00002:[4->10,4->11,4->2,4->4,7->10,7->11,7->2,7->4] | 0.40 | 362.173913043 | 31 | 161 | 0 | 1 |
| Path 434 | C00031->C00002:[4->10,4->11,4->2,4->4] | 0.40 | 354.354166667 | 39 | 192 | 0 | 1 |
| Path 435 | C00031->C00002:[4->10,4->11,4->2,4->4,7->11,7->4] | 0.40 | 493.549019608 | 32 | 51 | 0 | 1 |
| Path 436 | C00031->C00002:[4->10,4->11,7->10,7->11] | 0.20 | 348.461538462 | 19 | 143 | 0 | 0 |
| Path 437 | C00031->C00002:[4->10,4->11,4->2,4->4,7->10,7->11,7->2,7->4] | 0.40 | 350.211640212 | 38 | 189 | 0 | 1 |
| Path 438 | C00031->C00002:[4->10,4->2,7->11,7->4] | 0.40 | 412.098591549 | 35 | 71 | 0 | 1 |
| Path 439 | C00031->C00002:[9->10,9->11] | 0.20 | 306.3875 | 17 | 160 | 0 | 0 |
| Path 440 | C00031->C00002:[4->10,7->11] | 0.20 | 483.967741935 | 19 | 31 | 0 | 0 |
| Path 441 | C00031->C00002:[4->10,4->11,4->2,4->4] | 0.40 | 373.150537634 | 41 | 186 | 0 | 1 |
| Path 442 | C00031->C00002:[4->10,4->11,4->2,4->4,7->10,7->11,7->2,7->4] | 0.40 | 370.709876543 | 31 | 162 | 0 | 1 |
| Path 443 | C00031->C00002:[5->13,7->11,7->17,9->12] | 0.40 | 573.3 | 26 | 40 | 0 | 0 |
| Path 444 | C00031->C00002:[7->11,7->3] | 0.20 | 367.382550336 | 23 | 149 | 0 | 0 |
| Path 445 | C00031->C00002:[4->10,4->2,7->11,7->4] | 0.40 | 375.188679245 | 30 | 159 | 0 | 1 |
| Path 446 | C00031->C00002:[1->12,2->13,4->17,5->3,5->8] | 0.50 | 348.683823529 | 13 | 136 | 0 | 0 |
| Path 447 | C00031->C00002:[7->11,7->3] | 0.20 | 530.365853659 | 24 | 41 | 0 | 0 |
| Path 448 | C00031->C00002:[1->12,2->13,4->17,5->3,5->8] | 0.50 | 334.267605634 | 14 | 142 | 0 | 0 |
| Path 449 | C00031->C00002:[9->11] | 0.10 | 329.411764706 | 17 | 153 | 0 | 0 |
| Path 450 | C00031->C00002:[4->10,4->11,7->10,7->11] | 0.20 | 341.165517241 | 19 | 145 | 0 | 0 |
| Path 451 | C00031->C00002:[4->10,4->11] | 0.20 | 360.262068966 | 22 | 145 | 0 | 0 |
| Path 452 | C00031->C00002:[1->12,2->13,4->17,5->8,7->11] | 0.50 | 530.977272727 | 25 | 44 | 0 | 0 |
| Path 453 | C00031->C00002:[4->10,4->11,4->2,4->4,7->10,7->11,7->2,7->4] | 0.40 | 361.989304813 | 38 | 187 | 0 | 1 |
| Path 454 | C00031->C00002:[7->11,7->3] | 0.20 | 549.162790698 | 27 | 43 | 0 | 0 |
| Path 455 | C00031->C00002:[9->11] | 0.10 | 336.464052288 | 17 | 153 | 0 | 0 |
| Path 456 | C00031->C00002:[1->12,2->13,4->11,4->17,5->8] | 0.50 | 361.682758621 | 19 | 145 | 0 | 0 |
| Path 457 | C00031->C00002:[7->11] | 0.10 | 325.015037594 | 10 | 133 | 0 | 0 |
| Path 458 | C00031->C00002:[7->11] | 0.10 | 339.060150376 | 11 | 133 | 0 | 0 |
| Path 459 | C00031->C00002:[4->10,4->11,4->2,4->4] | 0.40 | 356.243093923 | 34 | 181 | 0 | 1 |
| Path 460 | C00031->C00002:[4->10,4->11,4->2,4->4] | 0.40 | 427.0 | 41 | 83 | 0 | 1 |
| Path 461 | C00031->C00002:[1->12,2->13,4->17,5->3,5->8,9->11] | 0.60 | 382.179012346 | 25 | 162 | 0 | 0 |
| Path 462 | C00031->C00002:[4->10,4->2,7->11,7->4] | 0.40 | 352.163043478 | 35 | 184 | 0 | 1 |
| Path 463 | C00031->C00002:[4->10,4->11,4->2,4->4] | 0.40 | 420.536585366 | 41 | 82 | 0 | 1 |
| Path 464 | C00031->C00002:[1->12,2->13,4->17,5->3,5->8,7->11] | 0.60 | 534.741935484 | 19 | 31 | 0 | 0 |
| Path 465 | C00031->C00002:[4->10,4->11,7->10,7->11] | 0.20 | 508.033333333 | 19 | 30 | 0 | 0 |
| Path 466 | C00031->C00002:[7->10,7->11] | 0.20 | 333.903030303 | 26 | 165 | 0 | 0 |
| Path 467 | C00031->C00002:[7->10,7->11] | 0.20 | 327.436046512 | 25 | 172 | 0 | 0 |
| Path 468 | C00031->C00002:[4->10,4->11,4->2,4->4,7->11,7->4] | 0.40 | 514.758064516 | 39 | 62 | 0 | 1 |
| Path 469 | C00031->C00002:[1->12,2->13,4->17,5->8,7->11] | 0.50 | 546.75 | 19 | 32 | 0 | 0 |
| Path 470 | C00031->C00002:[1->12,2->13,4->17,5->3,5->8,9->11] | 0.60 | 343.755813953 | 26 | 172 | 0 | 0 |
| Path 471 | C00031->C00002:[4->10,4->11,4->2,4->4,7->10,7->11,7->2,7->4] | 0.40 | 347.903225806 | 36 | 186 | 0 | 1 |
| Path 472 | C00031->C00002:[4->10,4->2,7->11,7->4] | 0.40 | 524.260869565 | 30 | 46 | 0 | 1 |
| Path 473 | C00031->C00002:[1->12,2->13,4->17,5->8,9->11] | 0.50 | 366.380952381 | 21 | 147 | 0 | 0 |
| Path 474 | C00031->C00002:[4->10,4->11] | 0.20 | 584.655172414 | 22 | 29 | 0 | 0 |
| Path 475 | C00031->C00002:[1->12,2->13,4->17,5->8,7->11] | 0.50 | 364.793103448 | 20 | 145 | 0 | 0 |
| Path 476 | C00031->C00002:[4->10,7->11] | 0.20 | 342.901408451 | 18 | 142 | 0 | 0 |
| Path 477 | C00031->C00002:[1->12,2->13,4->17,5->8,9->11] | 0.50 | 339.222891566 | 22 | 166 | 0 | 0 |
| Path 478 | C00031->C00002:[7->10,7->11] | 0.20 | 508.387096774 | 20 | 31 | 0 | 0 |
| Path 479 | C00031->C00002:[1->12,2->13,4->17,5->3,5->8,7->11] | 0.60 | 359.197368421 | 23 | 152 | 0 | 0 |
| Path 480 | C00031->C00002:[4->10,4->11,4->2,4->4] | 0.40 | 385.745762712 | 41 | 177 | 0 | 1 |
| Path 481 | C00031->C00002:[4->10,4->11,4->2,4->4,7->11,7->4] | 0.40 | 398.868421053 | 37 | 76 | 0 | 1 |
| Path 482 | C00031->C00002:[7->11,7->3] | 0.20 | 559.75 | 23 | 36 | 0 | 0 |
| Path 483 | C00031->C00002:[4->10,4->11,4->2,4->4,7->10,7->11,7->2,7->4] | 0.40 | 510.020408163 | 31 | 49 | 0 | 1 |
| Path 484 | C00031->C00002:[5->13,7->11,7->17,9->12] | 0.40 | 373.197368421 | 26 | 152 | 0 | 0 |
| Path 485 | C00031->C00002:[7->10,7->11] | 0.20 | 451.965517241 | 17 | 29 | 0 | 0 |
| Path 486 | C00031->C00002:[1->12,2->13,4->17,5->8,7->3,9->11] | 0.60 | 344.128654971 | 25 | 171 | 0 | 0 |
| Path 487 | C00031->C00002:[7->10,7->11] | 0.20 | 337.657534247 | 20 | 146 | 0 | 0 |
| Path 488 | C00031->C00002:[4->10,4->11] | 0.20 | 365.006993007 | 22 | 143 | 0 | 0 |
| Path 489 | C00031->C00002:[4->10,4->11,4->2,4->4,7->10,7->11,7->2,7->4] | 0.40 | 346.604278075 | 37 | 187 | 0 | 1 |
| Path 490 | C00031->C00002:[4->10,4->11,4->2,4->4,7->10,7->11,7->2,7->4] | 0.40 | 409.552631579 | 38 | 76 | 0 | 1 |
| Path 491 | C00031->C00002:[4->10,4->11,4->2,4->4,7->10,7->11,7->2,7->4] | 0.40 | 407.28 | 37 | 75 | 0 | 1 |
| Path 492 | C00031->C00002:[7->11,7->3] | 0.20 | 549.368421053 | 23 | 38 | 0 | 0 |
| Path 493 | C00031->C00002:[4->10,4->11,4->2,4->4,7->10,7->11,7->2,7->4] | 0.40 | 384.625730994 | 37 | 171 | 0 | 1 |
| Path 494 | C00031->C00002:[4->10,4->11,4->2,4->4,7->4] | 0.40 | 513.222222222 | 40 | 63 | 0 | 1 |
| Path 495 | C00031->C00002:[5->13,7->11,7->17,9->12] | 0.40 | 622.736842105 | 27 | 38 | 0 | 0 |
| Path 496 | C00031->C00002:[1->12,2->13,4->11,4->17,5->3,5->8] | 0.60 | 378.11875 | 23 | 160 | 0 | 0 |
| Path 497 | C00031->C00002:[9->10,9->11] | 0.20 | 319.642857143 | 23 | 168 | 0 | 0 |
| Path 498 | C00031->C00002:[4->10,4->11,4->2,4->4] | 0.40 | 375.482758621 | 25 | 145 | 0 | 1 |
| Path 499 | C00031->C00002:[1->12,2->13,4->17,5->3,5->8,9->11] | 0.60 | 333.01183432 | 22 | 169 | 0 | 0 |
| Path 500 | C00031->C00002:[7->11] | 0.10 | 518.454545455 | 12 | 22 | 0 | 0 |
| Path 501 | C00031->C00002:[4->10,4->11,4->2,4->4] | 0.40 | 356.340206186 | 40 | 194 | 0 | 1 |
| Path 502 | C00031->C00002:[5->13,7->11,7->17,9->12] | 0.40 | 668.787878788 | 26 | 33 | 0 | 0 |
| Path 503 | C00031->C00002:[1->12,2->13,4->17,5->3,5->8,7->11] | 0.60 | 570.404761905 | 22 | 42 | 0 | 0 |
| Path 504 | C00031->C00002:[7->3] | 0.10 | 346.302158273 | 14 | 139 | 0 | 0 |
| Path 505 | C00031->C00002:[1->12,2->13,4->17,5->3,5->8,9->11] | 0.60 | 360.368131868 | 29 | 182 | 0 | 0 |
| Path 506 | C00031->C00002:[4->10,4->11] | 0.20 | 323.698224852 | 22 | 169 | 0 | 0 |
| Path 507 | C00031->C00002:[4->10,4->11,4->2,4->4] | 0.40 | 383.658385093 | 34 | 161 | 0 | 1 |
| Path 508 | C00031->C00002:[7->11] | 0.10 | 474.5 | 12 | 24 | 0 | 0 |
| Path 509 | C00031->C00002:[4->10,4->11,4->2,4->4,7->11,7->4] | 0.40 | 348.058510638 | 36 | 188 | 0 | 1 |
| Path 510 | C00031->C00002:[4->10,4->11,4->2,4->4] | 0.40 | 388.95505618 | 41 | 178 | 0 | 1 |
| Path 511 | C00031->C00002:[4->10,4->11,4->2,4->4,7->10,7->11,7->2,7->4] | 0.40 | 381.258823529 | 37 | 170 | 0 | 1 |
| Path 512 | C00031->C00002:[7->11] | 0.10 | 336.234848485 | 11 | 132 | 0 | 0 |
| Path 513 | C00031->C00002:[1->12,2->13,4->17,5->8,7->11] | 0.50 | 363.586206897 | 19 | 145 | 0 | 0 |
| Path 514 | C00031->C00002:[9->10,9->11] | 0.20 | 328.030120482 | 23 | 166 | 0 | 0 |
| Path 515 | C00031->C00002:[1->12,2->13,4->11,4->17,5->8] | 0.50 | 360.253333333 | 20 | 150 | 0 | 0 |
| Path 516 | C00031->C00002:[1->12,2->13,4->17,5->3,5->8,9->11] | 0.60 | 384.197452229 | 24 | 157 | 0 | 0 |
| Path 517 | C00031->C00002:[7->10,7->11] | 0.20 | 327.694610778 | 24 | 167 | 0 | 0 |
| Path 518 | C00031->C00002:[7->10,7->11] | 0.20 | 335.90647482 | 15 | 139 | 0 | 0 |
| Path 519 | C00031->C00002:[7->10,7->11] | 0.20 | 334.82962963 | 15 | 135 | 0 | 0 |
| Path 520 | C00031->C00002:[7->10,7->11] | 0.20 | 327.395061728 | 22 | 162 | 0 | 0 |
| Path 521 | C00031->C00002:[4->10,4->11,4->2,4->4,7->10,7->11,7->2,7->4] | 0.40 | 370.237804878 | 32 | 164 | 0 | 1 |
| Path 522 | C00031->C00002:[4->10,4->11,4->2,4->4,7->11,7->4] | 0.40 | 501.083333333 | 38 | 60 | 0 | 1 |
| Path 523 | C00031->C00002:[4->10,4->11,4->2,4->4,7->11,7->4] | 0.40 | 362.99378882 | 30 | 161 | 0 | 1 |
| Path 524 | C00031->C00002:[4->10,4->11,4->2,4->4,7->10,7->11,7->2,7->4] | 0.40 | 378.864705882 | 36 | 170 | 0 | 1 |
| Path 525 | C00031->C00002:[4->10,4->11,4->2,4->4] | 0.40 | 349.043243243 | 35 | 185 | 0 | 1 |
| Path 526 | C00031->C00002:[1->12,2->13,4->17,5->8,9->11] | 0.50 | 327.577380952 | 21 | 168 | 0 | 0 |
| Path 527 | C00031->C00002:[1->12,2->13,4->17,5->8,7->11,7->3] | 0.60 | 572.947368421 | 24 | 38 | 0 | 0 |
| Path 528 | C00031->C00002:[7->11] | 0.10 | 340.462121212 | 13 | 132 | 0 | 0 |
| Path 529 | C00031->C00002:[7->10,7->11] | 0.20 | 349.645833333 | 20 | 144 | 0 | 0 |
| Path 530 | C00031->C00002:[4->10,4->11,7->10,7->11] | 0.20 | 352.201298701 | 25 | 154 | 0 | 0 |
| Path 531 | C00031->C00002:[7->10,7->11] | 0.20 | 456.0 | 20 | 34 | 0 | 0 |
| Path 532 | C00031->C00002:[1->12,2->13,4->17,5->8,9->11] | 0.50 | 344.764044944 | 27 | 178 | 0 | 0 |
| Path 533 | C00031->C00002:[1->12,2->13,4->17,5->3,5->8,7->11] | 0.60 | 551.688888889 | 23 | 45 | 0 | 0 |
| Path 534 | C00031->C00002:[4->10,4->11,4->2,4->4] | 0.40 | 426.892857143 | 42 | 84 | 0 | 1 |
| Path 535 | C00031->C00002:[9->10,9->11] | 0.20 | 331.377245509 | 24 | 167 | 0 | 0 |
| Path 536 | C00031->C00002:[1->12,2->13,4->17,5->3,5->8,9->11] | 0.60 | 356.419354839 | 29 | 186 | 0 | 0 |
| Path 537 | C00031->C00002:[4->10,4->11,4->2,4->4] | 0.40 | 377.035928144 | 34 | 167 | 0 | 1 |
| Path 538 | C00031->C00002:[4->10,4->11,4->2,4->4] | 0.40 | 420.536585366 | 41 | 82 | 0 | 1 |
| Path 539 | C00031->C00002:[7->10,7->11] | 0.20 | 484.764705882 | 22 | 34 | 0 | 0 |
| Path 540 | C00031->C00002:[1->12,2->13,4->11,4->17,5->8,7->3] | 0.60 | 376.341614907 | 26 | 161 | 0 | 0 |
| Path 541 | C00031->C00002:[7->11] | 0.10 | 349.186567164 | 14 | 134 | 0 | 0 |
| Path 542 | C00031->C00002:[1->12,2->13,4->17,5->3,5->8,9->11] | 0.60 | 347.241573034 | 24 | 178 | 0 | 0 |
| Path 543 | C00031->C00002:[4->10,4->2,7->10,7->11,7->2,7->4] | 0.40 | 545.392156863 | 34 | 51 | 0 | 1 |
| Path 544 | C00031->C00002:[4->10,4->11,4->2,4->4] | 0.40 | 362.47311828 | 37 | 186 | 0 | 1 |
| Path 545 | C00031->C00002:[9->10,9->11] | 0.20 | 324.113207547 | 19 | 159 | 0 | 0 |
| Path 546 | C00031->C00002:[4->10,4->11,4->2,4->4,7->4] | 0.40 | 348.428571429 | 37 | 189 | 0 | 1 |
| Path 547 | C00031->C00002:[4->10,4->11,4->2,4->4] | 0.40 | 359.616666667 | 35 | 180 | 0 | 1 |
| Path 548 | C00031->C00002:[1->12,2->13,4->17,5->8,7->11] | 0.50 | 524.714285714 | 20 | 35 | 0 | 0 |
| Path 549 | C00031->C00002:[4->10,4->11,4->2,4->4] | 0.40 | 592.454545455 | 33 | 44 | 0 | 1 |
| Path 550 | C00031->C00002:[1->12,2->13,4->17,5->3,5->8,7->11] | 0.60 | 570.404761905 | 22 | 42 | 0 | 0 |
| Path 551 | C00031->C00002:[1->12,2->13,4->17,5->8,7->3] | 0.50 | 352.886524823 | 15 | 141 | 0 | 0 |
| Path 552 | C00031->C00002:[1->12,2->13,4->17,5->8,7->11,7->3] | 0.60 | 533.431818182 | 26 | 44 | 0 | 0 |
| Path 553 | C00031->C00002:[7->11,7->3] | 0.20 | 547.459459459 | 24 | 37 | 0 | 0 |
| Path 554 | C00031->C00002:[7->3,9->11] | 0.20 | 348.129943503 | 29 | 177 | 0 | 0 |
| Path 555 | C00031->C00002:[5->13,7->11,7->17,9->12] | 0.40 | 387.215277778 | 25 | 144 | 0 | 0 |
| Path 556 | C00031->C00002:[4->10,4->11,4->2,4->4] | 0.40 | 390.98265896 | 40 | 173 | 0 | 1 |
| Path 557 | C00031->C00002:[1->12,2->13,4->17,5->8,7->3,9->11] | 0.60 | 345.232044199 | 30 | 181 | 0 | 0 |
| Path 558 | C00031->C00002:[1->12,2->13,4->17,5->3,5->8,7->11] | 0.60 | 360.442176871 | 20 | 147 | 0 | 0 |
| Path 559 | C00031->C00002:[1->12,2->13,4->17,5->8,7->11] | 0.50 | 357.937062937 | 18 | 143 | 0 | 0 |
| Path 560 | C00031->C00002:[7->10,7->11] | 0.20 | 330.423076923 | 21 | 52 | 0 | 0 |
| Path 561 | C00031->C00002:[4->10,4->11,4->2,4->4,7->10,7->11,7->2,7->4] | 0.40 | 350.699453552 | 36 | 183 | 0 | 1 |
| Path 562 | C00031->C00002:[4->10,4->11,7->10,7->11] | 0.20 | 352.068027211 | 23 | 147 | 0 | 0 |
| Path 563 | C00031->C00002:[4->10,4->11,4->2,4->4] | 0.40 | 383.024539877 | 35 | 163 | 0 | 1 |
| Path 564 | C00031->C00002:[1->12,2->13,4->11,4->17,5->8] | 0.50 | 365.611842105 | 21 | 152 | 0 | 0 |
| Path 565 | C00031->C00002:[4->10,4->11,4->2,4->4,7->10,7->11,7->2,7->4] | 0.40 | 378.633136095 | 35 | 169 | 0 | 1 |
| Path 566 | C00031->C00002:[1->12,2->13,4->11,4->17,5->3,5->8] | 0.60 | 365.582191781 | 20 | 146 | 0 | 0 |
| Path 567 | C00031->C00002:[1->12,2->13,4->17,5->8,9->11] | 0.50 | 334.0 | 21 | 164 | 0 | 0 |
| Path 568 | C00031->C00002:[7->10] | 0.10 | 335.813432836 | 14 | 134 | 0 | 0 |
| Path 569 | C00031->C00002:[4->10,4->11,4->2,4->4,7->10,7->11,7->2,7->4] | 0.40 | 374.863905325 | 36 | 169 | 0 | 1 |
| Path 570 | C00031->C00002:[1->12,2->13,4->10,4->11,4->17,4->2,4->4,5->8,9->3] | 0.90 | 352.124260355 | 35 | 169 | 0 | 1 |
| Path 571 | C00031->C00002:[4->10,4->11,4->2,4->4,7->10,7->11,7->2,7->4] | 0.40 | 357.036082474 | 40 | 194 | 0 | 1 |
| Path 572 | C00031->C00002:[1->12,2->13,4->17,5->8,7->11] | 0.50 | 356.091549296 | 19 | 142 | 0 | 0 |
| Path 573 | C00031->C00002:[1->12,2->13,4->17,5->8,9->11] | 0.50 | 370.045454545 | 23 | 154 | 0 | 0 |
| Path 574 | C00031->C00002:[1->12,2->13,4->17,5->8,7->11,7->3] | 0.60 | 560.777777778 | 28 | 45 | 0 | 0 |
| Path 575 | C00031->C00002:[4->10,4->11,4->2,4->4,7->10,7->11,7->2,7->4] | 0.40 | 380.457317073 | 34 | 164 | 0 | 1 |
| Path 576 | C00031->C00002:[5->13,7->17,9->12] | 0.30 | 363.226950355 | 14 | 141 | 0 | 0 |
| Path 577 | C00031->C00002:[4->10,4->11,4->2,4->4] | 0.40 | 359.960674157 | 35 | 178 | 0 | 1 |
| Path 578 | C00031->C00002:[7->10,7->11] | 0.20 | 327.306569343 | 14 | 137 | 0 | 0 |
| Path 579 | C00031->C00002:[1->12,2->13,4->17,5->8,7->11] | 0.50 | 355.410596026 | 22 | 151 | 0 | 0 |
| Path 580 | C00031->C00002:[4->10,4->2,7->11,7->4] | 0.40 | 525.5 | 37 | 56 | 0 | 1 |
| Path 581 | C00031->C00002:[4->10,4->11,4->2,4->4,7->10,7->11,7->2,7->4] | 0.40 | 531.717391304 | 31 | 46 | 0 | 1 |
| Path 582 | C00031->C00002:[1->12,2->13,4->17,5->8,7->11,7->3] | 0.60 | 562.425 | 24 | 40 | 0 | 0 |
| Path 583 | C00031->C00002:[4->10,4->11] | 0.20 | 348.707692308 | 14 | 130 | 0 | 0 |
| Path 584 | C00031->C00002:[1->12,2->13,4->17,5->8,7->11] | 0.50 | 559.735294118 | 20 | 34 | 0 | 0 |
| Path 585 | C00031->C00002:[7->10,7->11] | 0.20 | 353.884353741 | 22 | 147 | 0 | 0 |
| Path 586 | C00031->C00002:[7->10,7->11,9->10,9->11] | 0.20 | 326.886227545 | 24 | 167 | 0 | 0 |
| Path 587 | C00031->C00002:[4->10,4->11,4->2,4->4] | 0.40 | 357.274725275 | 35 | 182 | 0 | 1 |
| Path 588 | C00031->C00002:[4->10,4->11,4->2,4->4] | 0.40 | 556.354166667 | 34 | 48 | 0 | 1 |
| Path 589 | C00031->C00002:[7->3,9->11] | 0.20 | 337.517241379 | 25 | 174 | 0 | 0 |
| Path 590 | C00031->C00002:[4->10,4->11,4->2,4->4,7->10,7->11,7->2,7->4] | 0.40 | 533.537037037 | 36 | 54 | 0 | 1 |
| Path 591 | C00031->C00002:[4->10,4->11] | 0.20 | 328.527272727 | 24 | 165 | 0 | 0 |
| Path 592 | C00031->C00002:[4->10,4->11] | 0.20 | 328.078787879 | 22 | 165 | 0 | 0 |
| Path 593 | C00031->C00002:[4->10,4->11,4->2,4->4] | 0.40 | 347.592391304 | 36 | 184 | 0 | 1 |
| Path 594 | C00031->C00002:[1->12,2->13,4->17,5->8,7->11,7->3] | 0.60 | 562.425 | 24 | 40 | 0 | 0 |
| Path 595 | C00031->C00002:[4->10,4->11,4->2,4->4,7->10,7->11,7->2,7->4] | 0.40 | 358.047619048 | 39 | 189 | 0 | 1 |
| Path 596 | C00031->C00002:[5->13,7->11,7->17,9->12] | 0.40 | 375.954248366 | 26 | 153 | 0 | 0 |
| Path 597 | C00031->C00002:[4->10,4->11,4->2,4->4] | 0.40 | 359.157608696 | 37 | 184 | 0 | 1 |
| Path 598 | C00031->C00002:[1->12,2->13,4->17,5->8,7->11,7->3] | 0.60 | 377.717948718 | 27 | 156 | 0 | 0 |
| Path 599 | C00031->C00002:[1->12,2->13,4->17,5->3,5->8,9->11] | 0.60 | 340.578947368 | 25 | 171 | 0 | 0 |
| Path 600 | C00031->C00002:[7->11] | 0.10 | 515.473684211 | 11 | 19 | 0 | 0 |
| Path 601 | C00031->C00002:[1->12,2->13,4->17,5->3,5->8,7->11] | 0.60 | 352.655172414 | 19 | 145 | 0 | 0 |
| Path 602 | C00031->C00002:[4->11] | 0.10 | 336.914728682 | 10 | 129 | 0 | 0 |
| Path 603 | C00031->C00002:[1->12,2->13,4->17,5->3,5->8,9->11] | 0.60 | 357.458563536 | 28 | 181 | 0 | 0 |
| Path 604 | C00031->C00002:[4->10,4->11,4->2,4->4] | 0.40 | 356.656410256 | 41 | 195 | 0 | 1 |
| Path 605 | C00031->C00002:[1->12,2->13,4->17,5->8,7->11] | 0.50 | 558.179487179 | 24 | 39 | 0 | 0 |
| Path 606 | C00031->C00002:[1->12,2->13,4->17,5->8,7->11,7->3] | 0.60 | 536.58 | 29 | 50 | 0 | 0 |
| Path 607 | C00031->C00002:[9->11] | 0.10 | 311.759493671 | 14 | 158 | 0 | 0 |
| Path 608 | C00031->C00002:[1->12,2->13,4->11,4->17,5->3,5->8] | 0.60 | 380.032258065 | 22 | 155 | 0 | 0 |
| Path 609 | C00031->C00002:[4->11] | 0.10 | 345.893129771 | 11 | 131 | 0 | 0 |
| Path 610 | C00031->C00002:[4->10,4->11,4->2,4->4,7->11,7->4] | 0.40 | 372.830409357 | 37 | 171 | 0 | 1 |
| Path 611 | C00031->C00002:[4->10,4->11] | 0.20 | 347.6 | 15 | 135 | 0 | 0 |
| Path 612 | C00031->C00002:[7->11] | 0.10 | 323.373134328 | 11 | 134 | 0 | 0 |
| Path 613 | C00031->C00002:[1->12,2->13,4->17,5->3,5->8,7->11] | 0.60 | 513.117647059 | 20 | 34 | 0 | 0 |
| Path 614 | C00031->C00002:[1->12,2->13,4->17,5->8,7->11] | 0.50 | 348.638888889 | 18 | 144 | 0 | 0 |
| Path 615 | C00031->C00002:[1->12,2->13,4->17,5->3,5->8,7->11] | 0.60 | 376.960264901 | 21 | 151 | 0 | 0 |
| Path 616 | C00031->C00002:[9->11] | 0.10 | 325.522292994 | 17 | 157 | 0 | 0 |
| Path 617 | C00031->C00002:[4->10,4->11,4->2,4->4,7->10,7->11,7->2,7->4] | 0.40 | 407.135135135 | 36 | 74 | 0 | 1 |
| Path 618 | C00031->C00002:[4->10,4->11,4->2,4->4,7->4] | 0.40 | 379.436781609 | 39 | 174 | 0 | 1 |
| Path 619 | C00031->C00002:[4->10,4->11,4->2,4->4,7->10,7->11,7->2,7->4] | 0.40 | 374.343373494 | 34 | 166 | 0 | 1 |
| Path 620 | C00031->C00002:[7->10,7->11] | 0.20 | 336.422222222 | 15 | 135 | 0 | 0 |
| Path 621 | C00031->C00002:[4->11,5->13,7->17,9->12] | 0.40 | 389.386666667 | 26 | 150 | 0 | 0 |
| Path 622 | C00031->C00002:[4->10,4->11,4->2,4->4,7->10,7->11,7->2,7->4] | 0.40 | 367.62804878 | 33 | 164 | 0 | 1 |
| Path 623 | C00031->C00002:[4->10,4->11,4->2,4->4,7->10,7->11,7->2,7->4] | 0.40 | 370.506024096 | 35 | 166 | 0 | 1 |
| Path 624 | C00031->C00002:[1->12,2->13,4->17,5->3,5->8,7->11] | 0.60 | 544.019230769 | 27 | 52 | 0 | 0 |
| Path 625 | C00031->C00002:[4->10,4->11,4->2,4->4,7->10,7->11,7->2,7->4] | 0.40 | 365.347560976 | 33 | 164 | 0 | 1 |
| Path 626 | C00031->C00002:[1->12,2->13,4->17,5->8,7->11,7->3] | 0.60 | 572.947368421 | 24 | 38 | 0 | 0 |
| Path 627 | C00031->C00002:[1->12,2->13,4->17,5->8,7->11] | 0.50 | 357.872340426 | 18 | 141 | 0 | 0 |
| Path 628 | C00031->C00002:[1->12,2->13,4->17,5->8,7->11,7->3] | 0.60 | 560.948717949 | 25 | 39 | 0 | 0 |
| Path 629 | C00031->C00002:[1->12,2->13,4->11,4->17,5->8] | 0.50 | 368.548387097 | 22 | 155 | 0 | 0 |
| Path 630 | C00031->C00002:[7->11,7->3] | 0.20 | 364.697368421 | 23 | 152 | 0 | 0 |
| Path 631 | C00031->C00002:[4->10,4->11,7->10,7->11] | 0.20 | 336.406896552 | 19 | 145 | 0 | 0 |
| Path 632 | C00031->C00002:[9->10,9->11] | 0.20 | 323.951219512 | 20 | 164 | 0 | 0 |
| Path 633 | C00031->C00002:[4->10,4->11,4->2,4->4] | 0.40 | 379.087248322 | 27 | 149 | 0 | 1 |
| Path 634 | C00031->C00002:[4->10,4->11,4->2,4->4,7->10,7->11,7->2,7->4] | 0.40 | 348.989361702 | 37 | 188 | 0 | 1 |
| Path 635 | C00031->C00002:[1->12,2->13,4->17,5->3,5->8,7->11] | 0.60 | 558.393939394 | 20 | 33 | 0 | 0 |
| Path 636 | C00031->C00002:[7->10,7->11] | 0.20 | 338.213235294 | 15 | 136 | 0 | 0 |
| Path 637 | C00031->C00002:[4->10,4->11,4->2,4->4,7->10,7->11,7->2,7->4] | 0.40 | 509.057692308 | 33 | 52 | 0 | 1 |
| Path 638 | C00031->C00002:[1->12,2->13,4->17,5->8,7->11] | 0.50 | 559.033333333 | 19 | 30 | 0 | 0 |
| Path 639 | C00031->C00002:[4->10,4->11,7->10,7->11] | 0.20 | 470.555555556 | 21 | 36 | 0 | 0 |
| Path 640 | C00031->C00002:[4->10,4->11,4->2,4->4,7->10,7->11,7->2,7->4] | 0.40 | 501.92 | 32 | 50 | 0 | 1 |
| Path 641 | C00031->C00002:[4->10,4->11,4->2,4->4,7->10,7->11,7->2,7->4] | 0.40 | 355.710382514 | 36 | 183 | 0 | 1 |
| Path 642 | C00031->C00002:[4->10,4->11,4->2,4->4] | 0.40 | 351.163934426 | 34 | 183 | 0 | 1 |
| Path 643 | C00031->C00002:[4->10,4->11,4->2,4->4,7->10,7->11,7->2,7->4] | 0.40 | 520.644067797 | 37 | 59 | 0 | 1 |
| Path 644 | C00031->C00002:[7->10,7->11] | 0.20 | 355.770833333 | 21 | 144 | 0 | 0 |
| Path 645 | C00031->C00002:[1->12,2->13,4->17,5->8,9->11] | 0.50 | 364.815789474 | 22 | 152 | 0 | 0 |
| Path 646 | C00031->C00002:[4->10,4->11,4->2,4->4] | 0.40 | 365.79144385 | 40 | 187 | 0 | 1 |
| Path 647 | C00031->C00002:[4->10,4->11,4->2,4->4,7->10,7->11,7->2,7->4] | 0.40 | 513.716666667 | 38 | 60 | 0 | 1 |
| Path 648 | C00031->C00002:[4->10,4->2,7->11,7->4] | 0.40 | 377.946107784 | 36 | 167 | 0 | 1 |
| Path 649 | C00031->C00002:[5->13,7->17,9->11,9->12] | 0.40 | 342.06779661 | 28 | 177 | 0 | 0 |
| Path 650 | C00031->C00002:[1->12,2->13,4->17,5->3,5->8,9->11] | 0.60 | 339.960227273 | 26 | 176 | 0 | 0 |
| Path 651 | C00031->C00002:[4->10,4->11] | 0.20 | 352.363636364 | 21 | 143 | 0 | 0 |
| Path 652 | C00031->C00002:[4->10,4->11,4->2,4->4,7->10,7->11,7->2,7->4] | 0.40 | 360.831578947 | 40 | 190 | 0 | 1 |
| Path 653 | C00031->C00002:[4->10,4->11,4->2,4->4,7->4] | 0.40 | 509.830188679 | 33 | 53 | 0 | 1 |
| Path 654 | C00031->C00002:[4->10,4->11,4->2,4->4,7->10,7->11,7->2,7->4] | 0.40 | 345.349462366 | 36 | 186 | 0 | 1 |
| Path 655 | C00031->C00002:[7->10,7->11] | 0.20 | 319.54375 | 20 | 160 | 0 | 0 |
| Path 656 | C00031->C00002:[4->10,4->11,7->10,7->11] | 0.20 | 495.032258065 | 20 | 31 | 0 | 0 |
| Path 657 | C00031->C00002:[4->10,4->11,4->2,4->4] | 0.40 | 351.983783784 | 35 | 185 | 0 | 1 |
| Path 658 | C00031->C00002:[7->3,9->11] | 0.20 | 351.157303371 | 30 | 178 | 0 | 0 |
| Path 659 | C00031->C00002:[1->12,2->13,4->17,5->8,7->11] | 0.50 | 559.735294118 | 20 | 34 | 0 | 0 |
| Path 660 | C00031->C00002:[7->11] | 0.10 | 433.545454545 | 11 | 22 | 0 | 0 |
| Path 661 | C00031->C00002:[9->11] | 0.10 | 316.142857143 | 14 | 154 | 0 | 0 |
| Path 662 | C00031->C00002:[7->3,9->11] | 0.20 | 338.071005917 | 24 | 169 | 0 | 0 |
| Path 663 | C00031->C00002:[1->12,2->13,4->17,5->3,5->8,7->11] | 0.60 | 373.375796178 | 23 | 157 | 0 | 0 |
| Path 664 | C00031->C00002:[5->13,7->11,7->17,9->12] | 0.40 | 374.973509934 | 25 | 151 | 0 | 0 |
| Path 665 | C00031->C00002:[1->12,2->13,4->17,5->3,5->8,7->11] | 0.60 | 361.908450704 | 19 | 142 | 0 | 0 |
| Path 666 | C00031->C00002:[4->10,4->11,4->2,4->4,7->10,7->11,7->2,7->4] | 0.40 | 376.070588235 | 36 | 170 | 0 | 1 |
| Path 667 | C00031->C00002:[4->10,4->2,7->11,7->4] | 0.40 | 540.958333333 | 31 | 48 | 0 | 1 |
| Path 668 | C00031->C00002:[4->10,4->11,4->2,4->4] | 0.40 | 373.339869281 | 28 | 153 | 0 | 1 |
| Path 669 | C00031->C00002:[1->12,2->13,4->17,5->8,9->11] | 0.50 | 371.765100671 | 22 | 149 | 0 | 0 |
| Path 670 | C00031->C00002:[4->10,4->11,4->2,4->4] | 0.40 | 346.836956522 | 33 | 184 | 0 | 1 |
| Path 671 | C00031->C00002:[1->12,2->13,4->11,4->17,5->8] | 0.50 | 370.206666667 | 21 | 150 | 0 | 0 |
| Path 672 | C00031->C00002:[4->10,4->11,4->2,4->4] | 0.40 | 361.301587302 | 39 | 189 | 0 | 1 |
| Path 673 | C00031->C00002:[4->10,4->11,7->10,7->11] | 0.20 | 325.098837209 | 26 | 172 | 0 | 0 |
| Path 674 | C00031->C00002:[4->10,4->11,4->2,4->4,7->10,7->11,7->2,7->4] | 0.40 | 351.50802139 | 37 | 187 | 0 | 1 |
| Path 675 | C00031->C00002:[1->12,2->13,4->17,5->3,5->8,9->11] | 0.60 | 343.958083832 | 24 | 167 | 0 | 0 |
| Path 676 | C00031->C00002:[4->10,4->11] | 0.20 | 545.558823529 | 23 | 34 | 0 | 0 |
| Path 677 | C00031->C00002:[4->11] | 0.10 | 346.968253968 | 10 | 126 | 0 | 0 |
| Path 678 | C00031->C00002:[1->12,2->13,4->17,5->8,7->11] | 0.50 | 513.055555556 | 21 | 36 | 0 | 0 |
| Path 679 | C00031->C00002:[7->11,7->3] | 0.20 | 525.166666667 | 28 | 48 | 0 | 0 |
| Path 680 | C00031->C00002:[4->10,4->11,7->10,7->11] | 0.20 | 344.736486486 | 20 | 148 | 0 | 0 |
| Path 681 | C00031->C00002:[4->10,4->11,4->2,4->4,7->11,7->4] | 0.40 | 363.333333333 | 31 | 162 | 0 | 1 |
| Path 682 | C00031->C00002:[1->12,2->13,4->17,5->8,7->11] | 0.50 | 360.797385621 | 23 | 153 | 0 | 0 |
| Path 683 | C00031->C00002:[4->10,4->11,4->2,4->4] | 0.40 | 353.237569061 | 34 | 181 | 0 | 1 |
| Path 684 | C00031->C00002:[5->13,7->11,7->17,9->12] | 0.40 | 373.173076923 | 26 | 156 | 0 | 0 |
| Path 685 | C00031->C00002:[1->12,2->13,4->11,4->17,5->8,7->3] | 0.60 | 378.185897436 | 25 | 156 | 0 | 0 |
| Path 686 | C00031->C00002:[4->10,4->11] | 0.20 | 340.530487805 | 25 | 164 | 0 | 0 |
| Path 687 | C00031->C00002:[4->10,4->11,4->2,4->4] | 0.40 | 340.907103825 | 33 | 183 | 0 | 1 |
| Path 688 | C00031->C00002:[4->10,4->11,4->2,4->4] | 0.40 | 351.777777778 | 35 | 180 | 0 | 1 |
| Path 689 | C00031->C00002:[7->11] | 0.10 | 330.114503817 | 10 | 131 | 0 | 0 |
| Path 690 | C00031->C00002:[1->12,2->13,4->17,5->8,7->11] | 0.50 | 546.864864865 | 23 | 37 | 0 | 0 |
| Path 691 | C00031->C00002:[1->12,2->13,4->17,5->3,5->8,7->11] | 0.60 | 567.978723404 | 26 | 47 | 0 | 0 |
| Path 692 | C00031->C00002:[4->10,4->11,4->2,4->4,7->10,7->11,7->2,7->4] | 0.40 | 515.535714286 | 36 | 56 | 0 | 1 |
| Path 693 | C00031->C00002:[5->13,7->17,9->11,9->12] | 0.40 | 348.581005587 | 29 | 179 | 0 | 0 |
| Path 694 | C00031->C00002:[1->12,2->13,4->17,5->8,7->11] | 0.50 | 515.714285714 | 20 | 35 | 0 | 0 |
| Path 695 | C00031->C00002:[4->10,4->11,4->2,4->4,7->10,7->11,7->2,7->4] | 0.40 | 370.416149068 | 30 | 161 | 0 | 1 |
| Path 696 | C00031->C00002:[4->10,4->11,4->2,4->4,7->11,7->4] | 0.40 | 495.06 | 31 | 50 | 0 | 1 |
| Path 697 | C00031->C00002:[4->10,4->11,4->2,4->4,7->4] | 0.40 | 370.182926829 | 32 | 164 | 0 | 1 |
| Path 698 | C00031->C00002:[7->11,7->3] | 0.20 | 549.368421053 | 23 | 38 | 0 | 0 |
| Path 699 | C00031->C00002:[4->10,4->11,4->2,4->4,7->10,7->11,7->2,7->4] | 0.40 | 500.326530612 | 31 | 49 | 0 | 1 |
| Path 700 | C00031->C00002:[4->10,7->11] | 0.20 | 510.696969697 | 20 | 33 | 0 | 0 |
| Path 701 | C00031->C00002:[7->10,7->11,9->10,9->11] | 0.20 | 373.232142857 | 25 | 56 | 0 | 0 |
| Path 702 | C00031->C00002:[1->12,2->13,4->17,5->8,7->11] | 0.50 | 349.893617021 | 17 | 141 | 0 | 0 |
| Path 703 | C00031->C00002:[7->10,7->11] | 0.20 | 318.211180124 | 21 | 161 | 0 | 0 |
| Path 704 | C00031->C00002:[4->11,5->13,7->17,9->12] | 0.40 | 377.333333333 | 25 | 150 | 0 | 0 |
| Path 705 | C00031->C00002:[4->10,4->11,4->2,4->4,7->11,7->4] | 0.40 | 398.613333333 | 36 | 75 | 0 | 1 |
| Path 706 | C00031->C00002:[1->12,2->13,4->17,5->8,7->11] | 0.50 | 356.534246575 | 19 | 146 | 0 | 0 |
| Path 707 | C00031->C00002:[7->10,7->11] | 0.20 | 471.864864865 | 22 | 37 | 0 | 0 |
| Path 708 | C00031->C00002:[1->12,2->13,4->17,5->3,5->8,7->11] | 0.60 | 368.147651007 | 23 | 149 | 0 | 0 |
| Path 709 | C00031->C00002:[1->12,2->13,4->17,5->8,7->11] | 0.50 | 362.087837838 | 20 | 148 | 0 | 0 |
| Path 710 | C00031->C00002:[7->10,7->11] | 0.20 | 512.724137931 | 19 | 29 | 0 | 0 |
| Path 711 | C00031->C00002:[7->11] | 0.10 | 515.473684211 | 11 | 19 | 0 | 0 |
| Path 712 | C00031->C00002:[7->11] | 0.10 | 333.903703704 | 11 | 135 | 0 | 0 |
| Path 713 | C00031->C00002:[1->12,2->13,4->11,4->17,5->3,5->8] | 0.60 | 380.680981595 | 24 | 163 | 0 | 0 |
| Path 714 | C00031->C00002:[4->10,4->11,4->2,4->4] | 0.40 | 364.170454545 | 34 | 176 | 0 | 1 |
| Path 715 | C00031->C00002:[5->13,7->17,9->11,9->12] | 0.40 | 385.481012658 | 29 | 158 | 0 | 0 |
| Path 716 | C00031->C00002:[4->10,4->11,4->2,4->4] | 0.40 | 357.788659794 | 40 | 194 | 0 | 1 |
| Path 717 | C00031->C00002:[1->12,2->13,4->17,5->3,5->8] | 0.50 | 348.269503546 | 15 | 141 | 0 | 0 |
| Path 718 | C00031->C00002:[1->12,2->13,4->17,5->3,5->8,9->11] | 0.60 | 351.936781609 | 24 | 174 | 0 | 0 |
| Path 719 | C00031->C00002:[7->11] | 0.10 | 459.72 | 13 | 25 | 0 | 0 |
| Path 720 | C00031->C00002:[4->10,4->11,4->2,4->4] | 0.40 | 350.303191489 | 35 | 188 | 0 | 1 |
| Path 721 | C00031->C00002:[4->10,4->2,7->11,7->4] | 0.40 | 345.868131868 | 34 | 182 | 0 | 1 |
| Path 722 | C00031->C00002:[4->10,4->11,4->2,4->4] | 0.40 | 389.117318436 | 42 | 179 | 0 | 1 |
| Path 723 | C00031->C00002:[4->10,4->11,4->2,4->4,7->10,7->11,7->2,7->4] | 0.40 | 357.743455497 | 39 | 191 | 0 | 1 |
| Path 724 | C00031->C00002:[4->10,4->11,7->10,7->11] | 0.20 | 345.479166667 | 20 | 144 | 0 | 0 |
| Path 725 | C00031->C00002:[4->10,4->11,4->2,4->4,7->10,7->11,7->2,7->4] | 0.40 | 375.722222222 | 34 | 162 | 0 | 1 |
| Path 726 | C00031->C00002:[5->13,7->11,7->17,9->12] | 0.40 | 561.87804878 | 27 | 41 | 0 | 0 |
| Path 727 | C00031->C00002:[7->11,7->3] | 0.20 | 363.0 | 24 | 153 | 0 | 0 |
| Path 728 | C00031->C00002:[1->12,2->13,4->17,5->8,7->11] | 0.50 | 572.0625 | 20 | 32 | 0 | 0 |
| Path 729 | C00031->C00002:[4->10,4->11,4->2,4->4,7->10,7->11,7->2,7->4] | 0.40 | 502.041666667 | 30 | 48 | 0 | 1 |
| Path 730 | C00031->C00002:[7->10,7->11] | 0.20 | 318.228915663 | 22 | 166 | 0 | 0 |
| Path 731 | C00031->C00002:[4->10,4->2,7->11,7->4] | 0.40 | 540.958333333 | 31 | 48 | 0 | 1 |
| Path 732 | C00031->C00002:[7->10,7->11] | 0.20 | 512.724137931 | 19 | 29 | 0 | 0 |
| Path 733 | C00031->C00002:[1->12,2->13,4->17,5->8,7->11] | 0.50 | 572.0625 | 20 | 32 | 0 | 0 |
| Path 734 | C00031->C00002:[1->12,2->13,4->17,5->3,5->8,7->11] | 0.60 | 375.171052632 | 22 | 152 | 0 | 0 |
| Path 735 | C00031->C00002:[4->10,4->11,4->2,4->4] | 0.40 | 388.65408805 | 34 | 159 | 0 | 1 |
| Path 736 | C00031->C00002:[4->10,4->11,4->2,4->4] | 0.40 | 350.661375661 | 36 | 189 | 0 | 1 |
| Path 737 | C00031->C00002:[7->10,7->11] | 0.20 | 331.023809524 | 25 | 168 | 0 | 0 |
| Path 738 | C00031->C00002:[7->11,7->3] | 0.20 | 358.605442177 | 21 | 147 | 0 | 0 |
| Path 739 | C00031->C00002:[1->12,2->13,4->17,5->3,5->8,7->11] | 0.60 | 556.973684211 | 24 | 38 | 0 | 0 |
| Path 740 | C00031->C00002:[5->13,7->17,9->11,9->12] | 0.40 | 359.333333333 | 31 | 177 | 0 | 0 |
| Path 741 | C00031->C00002:[1->12,2->13,4->17,5->8,9->11] | 0.50 | 329.779141104 | 20 | 163 | 0 | 0 |
| Path 742 | C00031->C00002:[4->10,4->11,4->2,4->4,7->10,7->11,7->2,7->4] | 0.40 | 515.535714286 | 36 | 56 | 0 | 1 |
| Path 743 | C00031->C00002:[4->10,4->11] | 0.20 | 327.323353293 | 22 | 167 | 0 | 0 |
| Path 744 | C00031->C00002:[1->12,2->13,4->11,4->17,5->3,5->8] | 0.60 | 356.503355705 | 20 | 149 | 0 | 0 |
| Path 745 | C00031->C00002:[1->12,2->13,4->10,4->11,4->17,4->2,4->4,5->8,9->3] | 0.90 | 354.970414201 | 35 | 169 | 0 | 1 |
| Path 746 | C00031->C00002:[1->12,2->13,4->17,5->3,5->8,9->11] | 0.60 | 348.063583815 | 23 | 173 | 0 | 0 |
| Path 747 | C00031->C00002:[4->10,7->11] | 0.20 | 350.986111111 | 19 | 144 | 0 | 0 |
| Path 748 | C00031->C00002:[4->10,4->11] | 0.20 | 336.630952381 | 25 | 168 | 0 | 0 |
| Path 749 | C00031->C00002:[4->10,4->11] | 0.20 | 338.917293233 | 14 | 133 | 0 | 0 |
| Path 750 | C00031->C00002:[4->10,4->11,4->2,4->4,7->10,7->11,7->2,7->4] | 0.40 | 370.5 | 32 | 160 | 0 | 1 |
| Path 751 | C00031->C00002:[1->12,2->13,4->17,5->3,5->8,9->11] | 0.60 | 344.011627907 | 26 | 172 | 0 | 0 |
| Path 752 | C00031->C00002:[7->10,7->11] | 0.20 | 346.842857143 | 18 | 140 | 0 | 0 |
| Path 753 | C00031->C00002:[4->10,4->11,4->2,4->4] | 0.40 | 358.836065574 | 36 | 183 | 0 | 1 |
| Path 754 | C00031->C00002:[4->10,4->11,4->2,4->4,7->10,7->11,7->2,7->4] | 0.40 | 351.515957447 | 37 | 188 | 0 | 1 |
| Path 755 | C00031->C00002:[7->10,7->11] | 0.20 | 336.103030303 | 25 | 165 | 0 | 0 |
| Path 756 | C00031->C00002:[1->12,2->13,4->17,5->8,7->11] | 0.50 | 361.805555556 | 20 | 144 | 0 | 0 |
| Path 757 | C00031->C00002:[7->11,9->10] | 0.20 | 332.918367347 | 20 | 49 | 0 | 0 |
| Path 758 | C00031->C00002:[7->11,7->3] | 0.20 | 528.5 | 22 | 36 | 0 | 0 |
| Path 759 | C00031->C00002:[1->12,2->13,4->17,5->8,9->11] | 0.50 | 334.605882353 | 22 | 170 | 0 | 0 |
| Path 760 | C00031->C00002:[4->10,4->11,4->2,4->4] | 0.40 | 340.483516484 | 32 | 182 | 0 | 1 |
| Path 761 | C00031->C00002:[1->12,2->13,4->17,5->8,7->11] | 0.50 | 537.837837838 | 21 | 37 | 0 | 0 |
| Path 762 | C00031->C00002:[7->11,9->10] | 0.20 | 356.137254902 | 21 | 51 | 0 | 0 |
| Path 763 | C00031->C00002:[4->10,4->11,4->2,4->4,7->10,7->11,7->2,7->4] | 0.40 | 416.679487179 | 39 | 78 | 0 | 1 |
| Path 764 | C00031->C00002:[9->10,9->11] | 0.20 | 337.838323353 | 24 | 167 | 0 | 0 |
| Path 765 | C00031->C00002:[4->10,4->11,4->2,4->4] | 0.40 | 357.64893617 | 38 | 188 | 0 | 1 |
| Path 766 | C00031->C00002:[4->10,4->11,4->2,4->4,7->11,7->4] | 0.40 | 514.758064516 | 39 | 62 | 0 | 1 |
| Path 767 | C00031->C00002:[4->10,4->11,4->2,4->4,7->11,7->4] | 0.40 | 369.889570552 | 31 | 163 | 0 | 1 |
| Path 768 | C00031->C00002:[4->10,4->11,4->2,4->4] | 0.40 | 358.194736842 | 38 | 190 | 0 | 1 |
| Path 769 | C00031->C00002:[1->12,2->13,4->17,5->8,7->11,7->3] | 0.60 | 364.67114094 | 22 | 149 | 0 | 0 |
| Path 770 | C00031->C00002:[1->12,2->13,4->10,4->11,4->17,4->2,4->4,5->8,9->3] | 0.90 | 359.715151515 | 34 | 165 | 0 | 1 |
| Path 771 | C00031->C00002:[1->12,2->13,4->17,5->8,9->11] | 0.50 | 337.262857143 | 26 | 175 | 0 | 0 |
| Path 772 | C00031->C00002:[4->10,4->11,4->2,4->4] | 0.40 | 356.675257732 | 41 | 194 | 0 | 1 |
| Path 773 | C00031->C00002:[1->12,2->13,4->17,5->3,5->8,9->11] | 0.60 | 332.289017341 | 25 | 173 | 0 | 0 |
| Path 774 | C00031->C00002:[4->10,4->11,4->2,4->4] | 0.40 | 561.6 | 35 | 50 | 0 | 1 |
| Path 775 | C00031->C00002:[4->10,4->11,4->2,4->4,7->10,7->11,7->2,7->4] | 0.40 | 515.338983051 | 37 | 59 | 0 | 1 |
| Path 776 | C00031->C00002:[4->10,4->11] | 0.20 | 334.796407186 | 24 | 167 | 0 | 0 |
| Path 777 | C00031->C00002:[4->10,4->11,4->2,4->4,7->10,7->11,7->2,7->4] | 0.40 | 423.458333333 | 37 | 72 | 0 | 1 |
| Path 778 | C00031->C00002:[4->10,4->11,4->2,4->4,7->10,7->11,7->2,7->4] | 0.40 | 514.789473684 | 36 | 57 | 0 | 1 |
| Path 779 | C00031->C00002:[1->12,2->13,4->17,5->3,5->8,7->11] | 0.60 | 376.77124183 | 21 | 153 | 0 | 0 |
| Path 780 | C00031->C00002:[4->10,4->11,4->2,4->4] | 0.40 | 353.016129032 | 36 | 186 | 0 | 1 |
| Path 781 | C00031->C00002:[7->11] | 0.10 | 477.8 | 11 | 20 | 0 | 0 |
| Path 782 | C00031->C00002:[1->12,2->13,4->17,5->8,7->11] | 0.50 | 360.362416107 | 21 | 149 | 0 | 0 |
| Path 783 | C00031->C00002:[7->10,7->11] | 0.20 | 352.444444444 | 22 | 54 | 0 | 0 |
