# Supplementary material for: 13C-metabolic flux ratio and novel carbon path analyses confirmed that Trichoderma reesei uses primarily the respirative pathway also on the preferred carbon source glucose
Source: BMC Syst Biol. 2009 Oct 29;3:104. doi: 10.1186/1752-0509-3-104 (PMC2776023; doi:10.1186/1752-0509-3-104)
Supplement: Additional file 1 — Pathways discovered in ReTrace carbon path analysis. Graphical and tabular representations of amino acid synthesis pathways discovered in ReTrace carbon path analysis [21]. Self-contained web site: unpack zip archive and open index.html with a web browser. [file 1752-0509-3-104-S1.zip › AF1-treesei/pathways-C00031-to-C00041.html]

Pathways from C00031 to C00041


**Pathways from C00031 to C00041**

**Sources:** D-Glucose; (C00031)

**Target:**L-Alanine; (C00041)

|  | Composite mapping | Z | Average score | Rpairs | Reactions | Zero scores | Scores under threshold |
| --- | --- | --- | --- | --- | --- | --- | --- |
| Path 1 | C00031->C00041:[1->1,2->2,4->3] | 1.00 | 282.981132075 | 10 | 53 | 0 | 0 |
| Path 2 | C00031->C00041:[1->1,4->2,4->3] | 1.00 | 332.260869565 | 16 | 69 | 0 | 0 |
| Path 3 | C00031->C00041:[4->2,7->3,9->1] | 1.00 | 284.214285714 | 11 | 56 | 0 | 0 |
| Path 4 | C00031->C00041:[4->2,7->3,9->1] | 1.00 | 321.987804878 | 25 | 82 | 0 | 1 |
| Path 5 | C00031->C00041:[1->1,2->2,4->3] | 1.00 | 266.79245283 | 12 | 53 | 0 | 0 |
| Path 6 | C00031->C00041:[1->1,2->2,4->3] | 1.00 | 308.355932203 | 10 | 59 | 0 | 0 |
| Path 7 | C00031->C00041:[7->3,9->1,9->2] | 1.00 | 335.117647059 | 17 | 68 | 0 | 0 |
| Path 8 | C00031->C00041:[1->1,2->2,4->3] | 1.00 | 484.942857143 | 12 | 35 | 0 | 0 |
| Path 9 | C00031->C00041:[7->2,7->3,9->1] | 1.00 | 264.410714286 | 11 | 56 | 0 | 0 |
| Path 10 | C00031->C00041:[1->1,2->3,4->2] | 1.00 | 308.35483871 | 14 | 62 | 0 | 0 |
| Path 11 | C00031->C00041:[4->2,7->3,9->1] | 1.00 | 233.229885057 | 16 | 87 | 0 | 1 |
| Path 12 | C00031->C00041:[4->2,7->3,9->1] | 1.00 | 312.358974359 | 23 | 78 | 0 | 1 |
| Path 13 | C00031->C00041:[7->3,9->1,9->2] | 1.00 | 214.057142857 | 10 | 70 | 0 | 0 |
| Path 14 | C00031->C00041:[1->1,2->2,4->3] | 1.00 | 468.840909091 | 11 | 44 | 0 | 0 |
| Path 15 | C00031->C00041:[1->1,2->2,4->3] | 1.00 | 332.307692308 | 10 | 52 | 0 | 0 |
| Path 16 | C00031->C00041:[1->1,2->2,4->3] | 1.00 | 335.561403509 | 11 | 57 | 0 | 0 |
| Path 17 | C00031->C00041:[4->2,7->3,9->1] | 1.00 | 236.682926829 | 15 | 82 | 0 | 1 |
| Path 18 | C00031->C00041:[1->1,2->2,4->3] | 1.00 | 276.185185185 | 12 | 54 | 0 | 0 |
| Path 19 | C00031->C00041:[1->1,2->2,4->3] | 1.00 | 352.62295082 | 12 | 61 | 0 | 0 |
| Path 20 | C00031->C00041:[1->1,4->3,7->2] | 1.00 | 331.202898551 | 18 | 69 | 0 | 0 |
| Path 21 | C00031->C00041:[7->3,9->1,9->2] | 1.00 | 200.6375 | 11 | 80 | 0 | 0 |
| Path 22 | C00031->C00041:[1->1,2->3,4->2] | 1.00 | 325.34375 | 14 | 64 | 0 | 0 |
| Path 23 | C00031->C00041:[7->3,9->1,9->2] | 1.00 | 252.670212766 | 17 | 94 | 0 | 0 |
| Path 24 | C00031->C00041:[4->2,5->3,7->2,9->1] | 1.00 | 298.698630137 | 19 | 73 | 0 | 1 |
| Path 25 | C00031->C00041:[1->1,2->2,4->3] | 1.00 | 391.05 | 12 | 40 | 0 | 0 |
| Path 26 | C00031->C00041:[1->1,2->2,4->3] | 1.00 | 353.303571429 | 12 | 56 | 0 | 0 |
| Path 27 | C00031->C00041:[7->2,7->3,9->1] | 1.00 | 282.910714286 | 13 | 56 | 0 | 0 |
| Path 28 | C00031->C00041:[7->3,9->1,9->2] | 1.00 | 225.436619718 | 11 | 71 | 0 | 0 |
| Path 29 | C00031->C00041:[7->3,9->1,9->2] | 1.00 | 231.486486486 | 11 | 74 | 0 | 0 |
| Path 30 | C00031->C00041:[1->1,2->2,4->3] | 1.00 | 340.641509434 | 10 | 53 | 0 | 0 |
| Path 31 | C00031->C00041:[7->2,7->3,9->1] | 1.00 | 290.0 | 15 | 63 | 0 | 0 |
| Path 32 | C00031->C00041:[7->2,7->3,9->1] | 1.00 | 252.653061224 | 9 | 49 | 0 | 0 |
| Path 33 | C00031->C00041:[4->2,7->3,9->1] | 1.00 | 219.903614458 | 14 | 83 | 0 | 1 |
| Path 34 | C00031->C00041:[7->3,9->1,9->2] | 1.00 | 311.617647059 | 15 | 68 | 0 | 0 |
| Path 35 | C00031->C00041:[4->2,7->3,9->1] | 1.00 | 279.85 | 12 | 60 | 0 | 0 |
| Path 36 | C00031->C00041:[4->2,7->3,9->1] | 1.00 | 284.925925926 | 11 | 54 | 0 | 0 |
| Path 37 | C00031->C00041:[1->1,2->2,4->3] | 1.00 | 481.58974359 | 10 | 39 | 0 | 0 |
| Path 38 | C00031->C00041:[4->2,7->3,9->1] | 1.00 | 271.018867925 | 10 | 53 | 0 | 0 |
| Path 39 | C00031->C00041:[1->1,2->2,4->3] | 1.00 | 352.129032258 | 10 | 62 | 0 | 0 |
| Path 40 | C00031->C00041:[4->2,7->3,9->1] | 1.00 | 291.158730159 | 13 | 63 | 0 | 0 |
| Path 41 | C00031->C00041:[1->1,2->3,4->2] | 1.00 | 340.971014493 | 13 | 69 | 0 | 0 |
| Path 42 | C00031->C00041:[1->1,4->3,7->2] | 1.00 | 312.741935484 | 14 | 62 | 0 | 0 |
| Path 43 | C00031->C00041:[1->1,2->2,4->3] | 1.00 | 353.018518519 | 12 | 54 | 0 | 0 |
| Path 44 | C00031->C00041:[7->3,9->1,9->2] | 1.00 | 300.0 | 13 | 56 | 0 | 0 |
| Path 45 | C00031->C00041:[7->3,9->1,9->2] | 1.00 | 297.137931034 | 12 | 58 | 0 | 0 |
| Path 46 | C00031->C00041:[7->2,7->3,9->1] | 1.00 | 268.04 | 10 | 50 | 0 | 0 |
| Path 47 | C00031->C00041:[1->1,2->2,4->3] | 1.00 | 316.872727273 | 12 | 55 | 0 | 0 |
| Path 48 | C00031->C00041:[4->2,7->3,9->1] | 1.00 | 280.176470588 | 16 | 68 | 0 | 1 |
| Path 49 | C00031->C00041:[1->1,2->2,4->3] | 1.00 | 290.431034483 | 11 | 58 | 0 | 0 |
| Path 50 | C00031->C00041:[1->1,2->2,4->3] | 1.00 | 467.45 | 12 | 40 | 0 | 0 |
| Path 51 | C00031->C00041:[7->3,9->1,9->2] | 1.00 | 259.290322581 | 12 | 62 | 0 | 0 |
| Path 52 | C00031->C00041:[7->3,9->1,9->2] | 1.00 | 272.916666667 | 16 | 84 | 0 | 0 |
| Path 53 | C00031->C00041:[1->1,2->2,4->3] | 1.00 | 373.0 | 12 | 54 | 0 | 0 |
| Path 54 | C00031->C00041:[7->3,9->1,9->2] | 1.00 | 247.921052632 | 13 | 76 | 0 | 0 |
| Path 55 | C00031->C00041:[7->3,9->1,9->2] | 1.00 | 279.407407407 | 11 | 54 | 0 | 0 |
| Path 56 | C00031->C00041:[1->1,2->3,4->2] | 1.00 | 332.246153846 | 14 | 65 | 0 | 0 |
| Path 57 | C00031->C00041:[4->1,4->2,7->3,9->1] | 1.00 | 235.720930233 | 16 | 86 | 0 | 1 |
| Path 58 | C00031->C00041:[1->1,2->2,4->3] | 1.00 | 342.943396226 | 11 | 53 | 0 | 0 |
| Path 59 | C00031->C00041:[7->2,7->3,9->1] | 1.00 | 295.877192982 | 14 | 57 | 0 | 0 |
| Path 60 | C00031->C00041:[4->2,7->2,7->3,9->1] | 1.00 | 294.38028169 | 19 | 71 | 0 | 1 |
| Path 61 | C00031->C00041:[1->1,4->2,4->3] | 1.00 | 323.848484848 | 15 | 66 | 0 | 0 |
| Path 62 | C00031->C00041:[7->3,9->1,9->2] | 1.00 | 231.888888889 | 12 | 72 | 0 | 0 |
| Path 63 | C00031->C00041:[1->1,2->2,4->3] | 1.00 | 345.6 | 12 | 60 | 0 | 0 |
| Path 64 | C00031->C00041:[4->2,7->3,9->1] | 1.00 | 292.930555556 | 18 | 72 | 0 | 1 |
| Path 65 | C00031->C00041:[7->3,9->1,9->2] | 1.00 | 216.630952381 | 12 | 84 | 0 | 0 |
| Path 66 | C00031->C00041:[1->1,2->2,4->3] | 1.00 | 459.58974359 | 12 | 39 | 0 | 0 |
| Path 67 | C00031->C00041:[4->2,7->2,7->3,9->1] | 1.00 | 233.73255814 | 17 | 86 | 0 | 1 |
| Path 68 | C00031->C00041:[1->1,2->2,4->3] | 1.00 | 269.163265306 | 12 | 49 | 0 | 0 |
| Path 69 | C00031->C00041:[7->3,9->1,9->2] | 1.00 | 249.814814815 | 9 | 54 | 0 | 0 |
| Path 70 | C00031->C00041:[1->1,2->2,4->3] | 1.00 | 340.1875 | 10 | 48 | 0 | 0 |
| Path 71 | C00031->C00041:[7->3,9->1,9->2] | 1.00 | 270.047619048 | 16 | 84 | 0 | 0 |
| Path 72 | C00031->C00041:[5->3,7->2,9->1] | 1.00 | 185.142857143 | 6 | 35 | 0 | 0 |
| Path 73 | C00031->C00041:[1->1,2->3,4->2] | 1.00 | 331.183333333 | 14 | 60 | 0 | 0 |
| Path 74 | C00031->C00041:[4->2,5->3,7->2,9->1] | 1.00 | 286.463768116 | 17 | 69 | 0 | 1 |
| Path 75 | C00031->C00041:[1->1,2->2,4->3] | 1.00 | 316.781818182 | 11 | 55 | 0 | 0 |
| Path 76 | C00031->C00041:[4->2,7->2,7->3,9->1] | 1.00 | 305.866666667 | 21 | 75 | 0 | 1 |
| Path 77 | C00031->C00041:[4->2,7->3,9->1] | 1.00 | 297.157894737 | 12 | 57 | 0 | 0 |
| Path 78 | C00031->C00041:[7->3,9->1,9->2] | 1.00 | 271.464285714 | 11 | 56 | 0 | 0 |
| Path 79 | C00031->C00041:[7->3,9->1,9->2] | 1.00 | 216.62195122 | 13 | 82 | 0 | 0 |
| Path 80 | C00031->C00041:[4->2,7->3,9->1] | 1.00 | 253.376344086 | 21 | 93 | 0 | 1 |
| Path 81 | C00031->C00041:[1->1,2->2,4->3] | 1.00 | 350.614035088 | 9 | 57 | 0 | 0 |
| Path 82 | C00031->C00041:[1->1,2->2,4->3] | 1.00 | 343.120689655 | 11 | 58 | 0 | 0 |
| Path 83 | C00031->C00041:[4->2,5->1,9->3] | 1.00 | 340.093333333 | 24 | 75 | 0 | 1 |
| Path 84 | C00031->C00041:[4->2,7->3,9->1] | 1.00 | 295.154639175 | 27 | 97 | 0 | 1 |
| Path 85 | C00031->C00041:[4->1,4->2] | 0.67 | 352.455882353 | 22 | 68 | 0 | 1 |
| Path 86 | C00031->C00041:[4->2,7->3,9->1] | 1.00 | 230.279069767 | 15 | 86 | 0 | 1 |
| Path 87 | C00031->C00041:[4->1,4->2] | 0.67 | 311.626865672 | 19 | 67 | 0 | 1 |
| Path 88 | C00031->C00041:[4->1,4->2,7->1,7->2] | 0.67 | 289.5 | 15 | 64 | 0 | 1 |
| Path 89 | C00031->C00041:[4->2,7->3,9->1] | 1.00 | 326.662162162 | 23 | 74 | 0 | 1 |
| Path 90 | C00031->C00041:[4->2,7->3,9->1] | 1.00 | 314.588235294 | 29 | 102 | 0 | 1 |
| Path 91 | C00031->C00041:[4->2,7->2,7->3,9->1] | 1.00 | 358.1875 | 27 | 80 | 0 | 1 |
| Path 92 | C00031->C00041:[4->1,4->2] | 0.67 | 350.447761194 | 21 | 67 | 0 | 1 |
| Path 93 | C00031->C00041:[7->2,7->3,9->1] | 1.00 | 232.853658537 | 16 | 82 | 0 | 1 |
| Path 94 | C00031->C00041:[4->1,4->2] | 0.67 | 281.698113208 | 11 | 53 | 0 | 1 |
| Path 95 | C00031->C00041:[4->2,7->3,9->1] | 1.00 | 339.095890411 | 22 | 73 | 0 | 1 |
| Path 96 | C00031->C00041:[4->2,7->3,9->1] | 1.00 | 326.073684211 | 27 | 95 | 0 | 1 |
| Path 97 | C00031->C00041:[4->2] | 0.33 | 329.129032258 | 18 | 62 | 0 | 1 |
| Path 98 | C00031->C00041:[7->2,7->3,9->1] | 1.00 | 294.287234043 | 26 | 94 | 0 | 1 |
| Path 99 | C00031->C00041:[7->2,7->3,9->1] | 1.00 | 296.464646465 | 28 | 99 | 0 | 1 |
| Path 100 | C00031->C00041:[4->2,7->3,9->1] | 1.00 | 285.680412371 | 24 | 97 | 0 | 1 |
| Path 101 | C00031->C00041:[4->2,7->2,7->3,9->1] | 1.00 | 322.910891089 | 30 | 101 | 0 | 1 |
| Path 102 | C00031->C00041:[7->2,7->3,9->1] | 1.00 | 310.707692308 | 19 | 65 | 0 | 1 |
| Path 103 | C00031->C00041:[4->2,7->3,9->1] | 1.00 | 287.734375 | 15 | 64 | 0 | 1 |
| Path 104 | C00031->C00041:[7->2,7->3,9->1] | 1.00 | 292.673684211 | 27 | 95 | 0 | 1 |
| Path 105 | C00031->C00041:[7->3,9->1] | 0.67 | 191.416666667 | 9 | 72 | 0 | 1 |
| Path 106 | C00031->C00041:[7->2,7->3,9->1] | 1.00 | 332.847222222 | 24 | 72 | 0 | 1 |
| Path 107 | C00031->C00041:[4->2,7->3,9->1] | 1.00 | 298.014925373 | 16 | 67 | 0 | 1 |
| Path 108 | C00031->C00041:[4->2,7->3,9->1] | 1.00 | 348.12987013 | 24 | 77 | 0 | 1 |
| Path 109 | C00031->C00041:[4->1,4->2,7->1,7->2] | 0.67 | 294.5 | 18 | 72 | 0 | 1 |
| Path 110 | C00031->C00041:[4->2,7->3,9->1] | 1.00 | 318.0 | 25 | 91 | 0 | 1 |
| Path 111 | C00031->C00041:[4->2,7->3,9->1] | 1.00 | 349.91025641 | 25 | 78 | 0 | 1 |
| Path 112 | C00031->C00041:[4->2,7->3,9->1] | 1.00 | 354.875 | 27 | 80 | 0 | 1 |
| Path 113 | C00031->C00041:[4->1,4->2,7->1,7->2] | 0.67 | 281.838235294 | 16 | 68 | 0 | 1 |
| Path 114 | C00031->C00041:[7->3,9->1] | 0.67 | 244.490566038 | 8 | 53 | 0 | 0 |
| Path 115 | C00031->C00041:[4->2,7->3,9->1] | 1.00 | 329.611111111 | 21 | 72 | 0 | 1 |
| Path 116 | C00031->C00041:[4->1,4->2,7->1,7->2] | 0.67 | 340.346666667 | 24 | 75 | 0 | 1 |
| Path 117 | C00031->C00041:[5->1,7->2,9->3] | 1.00 | 323.102941176 | 22 | 68 | 0 | 1 |
| Path 118 | C00031->C00041:[4->2,7->3,9->1] | 1.00 | 334.333333333 | 25 | 81 | 0 | 1 |
| Path 119 | C00031->C00041:[4->2,7->3,9->1] | 1.00 | 298.772277228 | 28 | 101 | 0 | 1 |
| Path 120 | C00031->C00041:[7->2,7->3,9->1] | 1.00 | 268.611111111 | 22 | 90 | 0 | 1 |
| Path 121 | C00031->C00041:[4->2,7->3,9->1] | 1.00 | 293.442105263 | 25 | 95 | 0 | 1 |
| Path 122 | C00031->C00041:[4->2,7->3,9->1] | 1.00 | 311.653061224 | 28 | 98 | 0 | 1 |
| Path 123 | C00031->C00041:[4->2,7->3,9->1] | 1.00 | 334.090909091 | 24 | 77 | 0 | 1 |
| Path 124 | C00031->C00041:[4->2,7->3,9->1] | 1.00 | 307.254901961 | 29 | 102 | 0 | 1 |
| Path 125 | C00031->C00041:[7->2,7->3,9->1] | 1.00 | 205.924050633 | 13 | 79 | 0 | 1 |
| Path 126 | C00031->C00041:[4->1,4->2] | 0.67 | 330.212121212 | 19 | 66 | 0 | 1 |
| Path 127 | C00031->C00041:[7->2,7->3,9->1] | 1.00 | 318.638888889 | 24 | 72 | 0 | 1 |
| Path 128 | C00031->C00041:[4->2,5->1,9->3] | 1.00 | 332.708333333 | 23 | 72 | 0 | 1 |
| Path 129 | C00031->C00041:[4->1,4->2,7->1,7->2] | 0.67 | 347.153846154 | 25 | 78 | 0 | 1 |
| Path 130 | C00031->C00041:[4->2,7->3,9->1] | 1.00 | 286.141304348 | 24 | 92 | 0 | 1 |
| Path 131 | C00031->C00041:[7->2,7->3,9->1] | 1.00 | 276.602272727 | 23 | 88 | 0 | 1 |
| Path 132 | C00031->C00041:[4->2,7->3,9->1] | 1.00 | 281.344086022 | 23 | 93 | 0 | 1 |
| Path 133 | C00031->C00041:[4->1,4->2] | 0.67 | 288.62295082 | 14 | 61 | 0 | 1 |
| Path 134 | C00031->C00041:[4->2,7->2,7->3,9->1] | 1.00 | 327.521276596 | 28 | 94 | 0 | 1 |
| Path 135 | C00031->C00041:[4->2,7->3,9->1] | 1.00 | 320.173333333 | 22 | 75 | 0 | 1 |
| Path 136 | C00031->C00041:[7->3,9->1] | 0.67 | 228.934782609 | 6 | 46 | 0 | 0 |
| Path 137 | C00031->C00041:[7->2,7->3,9->1] | 1.00 | 208.413333333 | 12 | 75 | 0 | 1 |
| Path 138 | C00031->C00041:[4->1,4->2] | 0.67 | 321.301587302 | 18 | 63 | 0 | 1 |
| Path 139 | C00031->C00041:[7->2,7->3,9->1] | 1.00 | 281.380434783 | 24 | 92 | 0 | 1 |
| Path 140 | C00031->C00041:[4->1,4->2,7->1,7->2] | 0.67 | 299.701492537 | 16 | 67 | 0 | 1 |
| Path 141 | C00031->C00041:[4->2,7->3,9->1] | 1.00 | 313.928571429 | 27 | 98 | 0 | 1 |
| Path 142 | C00031->C00041:[4->2,7->3,9->1] | 1.00 | 308.444444444 | 28 | 99 | 0 | 1 |
| Path 143 | C00031->C00041:[7->2,7->3,9->1] | 1.00 | 301.132352941 | 20 | 68 | 0 | 1 |
| Path 144 | C00031->C00041:[4->2,7->2,7->3,9->1] | 1.00 | 348.626506024 | 28 | 83 | 0 | 1 |
| Path 145 | C00031->C00041:[7->2,7->3,9->1] | 1.00 | 263.127906977 | 21 | 86 | 0 | 1 |
| Path 146 | C00031->C00041:[4->2] | 0.33 | 319.559322034 | 17 | 59 | 0 | 1 |
| Path 147 | C00031->C00041:[4->2,7->3,9->1] | 1.00 | 340.869047619 | 26 | 84 | 0 | 1 |
| Path 148 | C00031->C00041:[4->1,4->2] | 0.67 | 316.197530864 | 22 | 81 | 0 | 1 |
| Path 149 | C00031->C00041:[4->2,7->3,9->1] | 1.00 | 341.185185185 | 27 | 81 | 0 | 1 |
| Path 150 | C00031->C00041:[4->2,7->3,9->1] | 1.00 | 353.367647059 | 23 | 68 | 0 | 1 |
| Path 151 | C00031->C00041:[4->2,7->3,9->1] | 1.00 | 290.322916667 | 25 | 96 | 0 | 1 |
| Path 152 | C00031->C00041:[4->2,7->3,9->1] | 1.00 | 300.888888889 | 28 | 99 | 0 | 1 |
| Path 153 | C00031->C00041:[4->2,7->3,9->1] | 1.00 | 340.419753086 | 26 | 81 | 0 | 1 |
| Path 154 | C00031->C00041:[4->2,7->3,9->1] | 1.00 | 321.607843137 | 29 | 102 | 0 | 1 |
| Path 155 | C00031->C00041:[4->2] | 0.33 | 526.1 | 7 | 10 | 0 | 0 |
| Path 156 | C00031->C00041:[4->2,7->3,9->1] | 1.00 | 273.477777778 | 22 | 90 | 0 | 1 |
| Path 157 | C00031->C00041:[4->2,7->3,9->1] | 1.00 | 222.974683544 | 13 | 79 | 0 | 1 |
| Path 158 | C00031->C00041:[4->2,7->3,9->1] | 1.00 | 233.743902439 | 14 | 82 | 0 | 1 |
| Path 159 | C00031->C00041:[1->1,4->3] | 0.67 | 297.305084746 | 11 | 59 | 0 | 0 |
| Path 160 | C00031->C00041:[4->2,7->2,7->3,9->1] | 1.00 | 320.288659794 | 29 | 97 | 0 | 1 |
| Path 161 | C00031->C00041:[4->2,7->3,9->1] | 1.00 | 353.76 | 24 | 75 | 0 | 1 |
| Path 162 | C00031->C00041:[4->2,7->3,9->1] | 1.00 | 338.5875 | 25 | 80 | 0 | 1 |
| Path 163 | C00031->C00041:[4->2,5->3,7->2,9->1] | 1.00 | 304.132352941 | 17 | 68 | 0 | 1 |
| Path 164 | C00031->C00041:[7->2,7->3,9->1] | 1.00 | 321.484848485 | 20 | 66 | 0 | 1 |
| Path 165 | C00031->C00041:[4->2,7->3,9->1] | 1.00 | 348.545454545 | 26 | 77 | 0 | 1 |
| Path 166 | C00031->C00041:[4->1,4->2] | 0.67 | 322.788732394 | 21 | 71 | 0 | 1 |
| Path 167 | C00031->C00041:[4->2,7->3,9->1] | 1.00 | 321.449275362 | 20 | 69 | 0 | 1 |
| Path 168 | C00031->C00041:[4->2,7->3,9->1] | 1.00 | 327.973684211 | 25 | 76 | 0 | 1 |
| Path 169 | C00031->C00041:[4->2,7->3,9->1] | 1.00 | 342.588235294 | 27 | 85 | 0 | 1 |
| Path 170 | C00031->C00041:[4->2,5->3,7->2,9->1] | 1.00 | 306.782608696 | 18 | 69 | 0 | 1 |
| Path 171 | C00031->C00041:[4->2,7->3,9->1] | 1.00 | 304.213592233 | 29 | 103 | 0 | 1 |
| Path 172 | C00031->C00041:[4->2,7->3,9->1] | 1.00 | 331.623376623 | 24 | 77 | 0 | 1 |
| Path 173 | C00031->C00041:[4->1,4->2] | 0.67 | 342.625 | 20 | 64 | 0 | 1 |
| Path 174 | C00031->C00041:[7->2,7->3,9->1] | 1.00 | 316.985714286 | 22 | 70 | 0 | 1 |
| Path 175 | C00031->C00041:[7->2,7->3,9->1] | 1.00 | 283.404255319 | 26 | 94 | 0 | 1 |
| Path 176 | C00031->C00041:[4->2,7->3,9->1] | 1.00 | 360.295774648 | 24 | 71 | 0 | 1 |
| Path 177 | C00031->C00041:[4->2,7->2,7->3,9->1] | 1.00 | 351.987012987 | 26 | 77 | 0 | 1 |
| Path 178 | C00031->C00041:[7->2,7->3,9->1] | 1.00 | 290.188888889 | 25 | 90 | 0 | 1 |
| Path 179 | C00031->C00041:[7->3,9->1] | 0.67 | 245.808510638 | 7 | 47 | 0 | 0 |
| Path 180 | C00031->C00041:[4->1,4->2] | 0.67 | 294.321428571 | 12 | 56 | 0 | 1 |
| Path 181 | C00031->C00041:[4->1,4->2,7->1,7->2] | 0.67 | 291.788732394 | 17 | 71 | 0 | 1 |
| Path 182 | C00031->C00041:[7->2,7->3,9->1] | 1.00 | 329.955223881 | 21 | 67 | 0 | 1 |
| Path 183 | C00031->C00041:[4->2,7->3,9->1] | 1.00 | 297.202020202 | 26 | 99 | 0 | 1 |
| Path 184 | C00031->C00041:[7->2,7->3,9->1] | 1.00 | 333.142857143 | 26 | 77 | 0 | 1 |
| Path 185 | C00031->C00041:[7->2,7->3,9->1] | 1.00 | 330.472222222 | 23 | 72 | 0 | 1 |
| Path 186 | C00031->C00041:[4->2,7->3,9->1] | 1.00 | 311.958333333 | 21 | 72 | 0 | 1 |
| Path 187 | C00031->C00041:[4->1,4->2] | 0.67 | 297.701754386 | 13 | 57 | 0 | 1 |
| Path 188 | C00031->C00041:[4->2,7->3,9->1] | 1.00 | 335.164556962 | 26 | 79 | 0 | 1 |
| Path 189 | C00031->C00041:[4->2,7->3,9->1] | 1.00 | 331.457142857 | 21 | 70 | 0 | 1 |
| Path 190 | C00031->C00041:[4->2,5->3,7->2,9->1] | 1.00 | 294.292307692 | 16 | 65 | 0 | 1 |
| Path 191 | C00031->C00041:[4->2,7->2,7->3,9->1] | 1.00 | 344.404761905 | 28 | 84 | 0 | 1 |
| Path 192 | C00031->C00041:[4->2,7->3,9->1] | 1.00 | 346.944444444 | 23 | 72 | 0 | 1 |
| Path 193 | C00031->C00041:[4->1,4->2,7->1,7->2] | 0.67 | 317.5 | 26 | 92 | 0 | 1 |
| Path 194 | C00031->C00041:[4->2,7->3,9->1] | 1.00 | 300.794117647 | 17 | 68 | 0 | 1 |
| Path 195 | C00031->C00041:[4->1,4->2] | 0.67 | 285.316666667 | 13 | 60 | 0 | 1 |
| Path 196 | C00031->C00041:[4->2,7->3,9->1] | 1.00 | 341.27027027 | 23 | 74 | 0 | 1 |
| Path 197 | C00031->C00041:[7->3,9->1,9->2] | 1.00 | 210.693333333 | 11 | 75 | 0 | 1 |
| Path 198 | C00031->C00041:[4->2,7->2,7->3,9->1] | 1.00 | 350.367816092 | 29 | 87 | 0 | 1 |
| Path 199 | C00031->C00041:[4->2] | 0.33 | 524.076923077 | 8 | 13 | 0 | 0 |
| Path 200 | C00031->C00041:[4->2,7->3,9->1] | 1.00 | 305.157894737 | 27 | 95 | 0 | 1 |
| Path 201 | C00031->C00041:[4->2,7->3,9->1] | 1.00 | 278.287234043 | 23 | 94 | 0 | 1 |
| Path 202 | C00031->C00041:[4->2,7->3,9->1] | 1.00 | 310.840425532 | 26 | 94 | 0 | 1 |
| Path 203 | C00031->C00041:[4->1,4->2] | 0.67 | 273.105263158 | 12 | 57 | 0 | 1 |
| Path 204 | C00031->C00041:[4->2,7->3,9->1] | 1.00 | 347.476190476 | 28 | 84 | 0 | 1 |
| Path 205 | C00031->C00041:[4->2,7->2,7->3,9->1] | 1.00 | 342.3 | 27 | 80 | 0 | 1 |
| Path 206 | C00031->C00041:[7->2] | 0.33 | 295.53968254 | 17 | 63 | 0 | 1 |
| Path 207 | C00031->C00041:[4->2,7->3,9->1] | 1.00 | 288.063829787 | 26 | 94 | 0 | 1 |
| Path 208 | C00031->C00041:[7->2,7->3,9->1] | 1.00 | 278.822222222 | 25 | 90 | 0 | 1 |
| Path 209 | C00031->C00041:[4->1,4->2] | 0.67 | 325.305882353 | 24 | 85 | 0 | 1 |
| Path 210 | C00031->C00041:[4->2,7->3,9->1] | 1.00 | 292.081632653 | 27 | 98 | 0 | 1 |
| Path 211 | C00031->C00041:[4->2,7->3,9->1] | 1.00 | 310.245283019 | 30 | 106 | 0 | 1 |
| Path 212 | C00031->C00041:[4->2,7->3,9->1] | 1.00 | 318.959183673 | 28 | 98 | 0 | 1 |
