# Supplementary material for: 13C-metabolic flux ratio and novel carbon path analyses confirmed that Trichoderma reesei uses primarily the respirative pathway also on the preferred carbon source glucose
Source: BMC Syst Biol. 2009 Oct 29;3:104. doi: 10.1186/1752-0509-3-104 (PMC2776023; doi:10.1186/1752-0509-3-104)
Supplement: Additional file 1 — Pathways discovered in ReTrace carbon path analysis. Graphical and tabular representations of amino acid synthesis pathways discovered in ReTrace carbon path analysis [21]. Self-contained web site: unpack zip archive and open index.html with a web browser. [file 1752-0509-3-104-S1.zip › AF1-treesei/index.html]

ReTrace analysis of T. reesei amino acid biosynthesis pathways


# ReTrace analysis of T. reesei amino acid biosynthesis pathways

This document describes in detail the results of the ReTrace analysis
of T. reesei amino acid biosynthesis pathways as described in the
manuscript

```
P. Jouhten, E. Pitk�nen, T. Pakula, M. Saloheimo, M. Penttil� and H. Maaheimo:
13C-metabolic flux ratio and novel carbon path analyses confirmed that
Trichoderma reesei uses primarily the respirative pathway also
on the preferred carbon source glucose. Submitted, 2009.
```

Result files can be accessed by the link below.

- Pathways found by ReTrace

## Result file descriptions

ReTrace generates result files of three different types: a query
summary, query HTML result, query text result and pathway result files.
In the following the contents of these files are explained.

Most tables in results are sortable by clicking column headings.

In addition to pathways discussed in the manuscript, the results
include also pathways for other metabolites. For example,
alanine biosynthesis pathways starting from both glucose and
pyruvate are provided.

## Query summary file

The query summary file contains a table which summarizes ReTrace
results for each pathway query performed. The table includes queries
from amino acid precursors to amino acids, as described in the
manuscript. Additionally, other queries are reported. For instance,
amino acids queries where glucose was assigned the source metabolite
were performed and are reported here.

Each line in the summary table corresponds to a single pathway
query. The two hyperlinks for each entry link to query HTML and text
result files, which are described in detail below.

The table gives the following information about query results.

- Source(s): query source metabolites.
- Target: query target metabolites.
- Pathways: link to query HTML result file.
- Summary: link to query HTML text file.
- NumPathways: the total number of pathways found.
- Zo: mean Zo score of pathways (fraction of target atoms transferred from source
  atoms), standard deviation in parentheses.
- BestZo: best Zo score.
- BestAvgScore: the highest average reaction score over all pathways.
- AvgScores: average reaction score.
- #RPAIRs: number of KEGG RPAIR entries utilized by the pathways on the average.
- #Reactions: number of KEGG reactions utilized by the pathways on the
  average. Note that all reactions corresponding to any KEGG RPAIR entry
  utilized in the pathway are counted in this number.
- MinZeroScores: minimum number of reactions with zero reaction scores
  over all pathways.
- ZeroScores: average number of zero reaction scores in pathways.
- MinScoresUnderThr: minimum number of reactions with scores under 50
  over all pathways.
- ScoresUnderThr: average number of reactions with scores under 50 in pathways.

## Query HTML result files

Query HTML result files, accessible from the query summary table, give
information about each result pathway found for the query, and
provide links to a more detailed description of each pathway (pathway
result file).

Query HTML result file contains a table with the following columns.

- Composite mapping: Overall atom mapping performed by this pathway.
- Z: Zo score of this pathway.
- Average score: Average of reaction scores.
- Rpairs: Number of KEGG RPAIR entries used in this pathway.
- Reactions: Number of KEGG reactions used in this pathway.
- Zero scores: Number of reactions with zero score.
- Scores under threshold: Number of reactions with a score under 50.

## Query text result files

Query text result files provide the same information as query HTML result files.
Additionally, for each pathway, the individual KEGG RPAIR and reaction
entries are given. Moreover, lines prefixed with
"#" report the query parameters given to ReTrace, in addition to the
total query time.

## Pathway result files

Each pathway result file reports an individual metabolic pathway in
html format.

Each file begins with a graphical description of the composite
mapping corresponding to the pathway by giving molecular structures
for source and target metabolites. In each image of a molecule,
green circles denote atoms that are involved in the composite
mapping. Further, the composite mapping is specified under the
images as a text string.
*Note:* images are drawn with an external program located on
the server sysdb.cs.helsinki.fi.

KEGG RPAIR entries used on the pathway are listed in a table, one
entry per line. For each entry, the table contains the following
data.

- Reaction: KEGG reaction identifier corresponding to the RPAIR
  entry. Note that only the best scoring reaction is reported for each
  RPAIR entry. A link to KEGG reaction database is provided.
- Score: Score of the reaction reported for this RPAIR entry.
  Score is derived from a pairwise Blast run as described in the manuscript.
  Score is
  color-coded as follows: green - score > 50, blue - 0 < score <= 50, red - score = 0.
- Seq1: Identifier of *T. reesei* sequence in the Blast hit from
  which the score was derived.
- Seq2: Identifier of UniProt sequence in the Blast hit from which the
  score was derived.
- Evalue: Blast E-value corresponding to the score.
- ECs: EC numbers corresponding to the KEGG reaction.
- Equation: Reaction equation.

Finally, the file concludes with a pathway diagram.
Reactions are drawn as boxes, metabolites as ellipses. Reactions have
been color-coded as in the table.
Source and target metabolites have been color-coded green and yellow,
respectively.
