# Supplementary material for: 13C-metabolic flux ratio and novel carbon path analyses confirmed that Trichoderma reesei uses primarily the respirative pathway also on the preferred carbon source glucose
Source: BMC Syst Biol. 2009 Oct 29;3:104. doi: 10.1186/1752-0509-3-104 (PMC2776023; doi:10.1186/1752-0509-3-104)
Supplement: Additional file 1 — Pathways discovered in ReTrace carbon path analysis. Graphical and tabular representations of amino acid synthesis pathways discovered in ReTrace carbon path analysis [21]. Self-contained web site: unpack zip archive and open index.html with a web browser. [file 1752-0509-3-104-S1.zip › AF1-treesei/pathways-C00022-to-C00183.html]

Pathways from C00022 to C00183


**Pathways from C00022 to C00183**

**Sources:** Pyruvate; (C00022)

**Target:**L-Valine; (C00183)

|  | Composite mapping | Z | Average score | Rpairs | Reactions | Zero scores | Scores under threshold |
| --- | --- | --- | --- | --- | --- | --- | --- |
| Path 1 | C00022->C00183:[1->1,1->2,2->3,3->5,3->6] | 1.00 | 488.422535211 | 20 | 71 | 0 | 0 |
| Path 2 | C00022->C00183:[1->2,3->5] | 0.40 | 379.954545455 | 14 | 44 | 0 | 0 |
| Path 3 | C00022->C00183:[1->2,3->6] | 0.40 | 492.396825397 | 20 | 63 | 0 | 0 |
| Path 4 | C00022->C00183:[1->1,1->2,2->3,3->5,3->6] | 1.00 | 554.418604651 | 17 | 43 | 0 | 0 |
| Path 5 | C00022->C00183:[1->1,1->2,2->3,3->5,3->6] | 1.00 | 509.418181818 | 18 | 55 | 0 | 0 |
| Path 6 | C00022->C00183:[1->1,2->3,3->6] | 0.60 | 482.734693878 | 15 | 49 | 0 | 0 |
| Path 7 | C00022->C00183:[1->2,3->6] | 0.40 | 534.741935484 | 13 | 31 | 0 | 0 |
| Path 8 | C00022->C00183:[1->2,3->5] | 0.40 | 529.444444444 | 13 | 27 | 0 | 0 |
| Path 9 | C00022->C00183:[1->1,2->3,3->6] | 0.60 | 542.780487805 | 15 | 41 | 0 | 0 |
| Path 10 | C00022->C00183:[1->2,3->5] | 0.40 | 504.863636364 | 18 | 44 | 0 | 0 |
| Path 11 | C00022->C00183:[1->2,3->5] | 0.40 | 412.603448276 | 15 | 58 | 0 | 0 |
| Path 12 | C00022->C00183:[1->1,1->2,2->3,3->5,3->6] | 1.00 | 489.412698413 | 21 | 63 | 0 | 0 |
| Path 13 | C00022->C00183:[1->1,2->3,3->6] | 0.60 | 499.725 | 16 | 40 | 0 | 0 |
| Path 14 | C00022->C00183:[1->2,3->6] | 0.40 | 485.87755102 | 19 | 49 | 0 | 0 |
| Path 15 | C00022->C00183:[1->1,2->2,2->3,3->5,3->6] | 1.00 | 431.363636364 | 20 | 66 | 0 | 0 |
| Path 16 | C00022->C00183:[1->2,3->5] | 0.40 | 395.844444444 | 15 | 45 | 0 | 0 |
| Path 17 | C00022->C00183:[1->1,1->2,2->3,3->5,3->6] | 1.00 | 569.314285714 | 17 | 35 | 0 | 0 |
| Path 18 | C00022->C00183:[1->2,3->5] | 0.40 | 507.692307692 | 12 | 26 | 0 | 0 |
| Path 19 | C00022->C00183:[1->2,3->6] | 0.40 | 498.283018868 | 17 | 53 | 0 | 0 |
| Path 20 | C00022->C00183:[1->1,2->3,3->6] | 0.60 | 534.413043478 | 14 | 46 | 0 | 0 |
| Path 21 | C00022->C00183:[1->1,1->2,2->3,3->5,3->6] | 1.00 | 594.705882353 | 18 | 34 | 0 | 0 |
| Path 22 | C00022->C00183:[1->1,1->2,2->3,3->5,3->6] | 1.00 | 557.529411765 | 15 | 34 | 0 | 0 |
| Path 23 | C00022->C00183:[1->1,2->3,3->6] | 0.60 | 483.823529412 | 17 | 51 | 0 | 0 |
| Path 24 | C00022->C00183:[1->2,3->6] | 0.40 | 507.692307692 | 12 | 26 | 0 | 0 |
| Path 25 | C00022->C00183:[1->2,3->6] | 0.40 | 510.325 | 13 | 40 | 0 | 0 |
| Path 26 | C00022->C00183:[1->1,1->2,2->3,3->5,3->6] | 1.00 | 525.408163265 | 19 | 49 | 0 | 0 |
| Path 27 | C00022->C00183:[1->1,1->2,2->3,3->5,3->6] | 1.00 | 474.606557377 | 17 | 61 | 0 | 0 |
| Path 28 | C00022->C00183:[1->1,1->2,2->3,3->5,3->6] | 1.00 | 431.041666667 | 12 | 24 | 0 | 0 |
| Path 29 | C00022->C00183:[1->1,2->3,3->6] | 0.60 | 401.077922078 | 17 | 77 | 0 | 0 |
| Path 30 | C00022->C00183:[1->1,1->2,2->3,3->5,3->6] | 1.00 | 417.571428571 | 14 | 63 | 0 | 0 |
| Path 31 | C00022->C00183:[1->2,3->6] | 0.40 | 510.157894737 | 16 | 38 | 0 | 0 |
| Path 32 | C00022->C00183:[1->1,1->2,2->3,3->5,3->6] | 1.00 | 471.545454545 | 6 | 11 | 0 | 0 |
| Path 33 | C00022->C00183:[1->2,3->6] | 0.40 | 559.666666667 | 15 | 33 | 0 | 0 |
| Path 34 | C00022->C00183:[1->2,3->6] | 0.40 | 601.615384615 | 13 | 26 | 0 | 0 |
| Path 35 | C00022->C00183:[1->1,1->2,2->3,3->5,3->6] | 1.00 | 474.606557377 | 17 | 61 | 0 | 0 |
| Path 36 | C00022->C00183:[1->1,2->3,3->6] | 0.60 | 503.740740741 | 17 | 54 | 0 | 0 |
| Path 37 | C00022->C00183:[1->1,2->3,3->6] | 0.60 | 542.8125 | 13 | 32 | 0 | 0 |
| Path 38 | C00022->C00183:[1->2,3->5] | 0.40 | 601.615384615 | 13 | 26 | 0 | 0 |
| Path 39 | C00022->C00183:[1->2,3->5] | 0.40 | 510.325 | 13 | 40 | 0 | 0 |
| Path 40 | C00022->C00183:[1->2,3->5] | 0.40 | 485.87755102 | 19 | 49 | 0 | 0 |
| Path 41 | C00022->C00183:[1->1,1->2,2->3,3->5,3->6] | 1.00 | 571.285714286 | 18 | 35 | 0 | 0 |
| Path 42 | C00022->C00183:[1->2,3->6] | 0.40 | 528.666666667 | 14 | 45 | 0 | 0 |
| Path 43 | C00022->C00183:[1->1,2->3,3->6] | 0.60 | 390.430555556 | 18 | 72 | 0 | 0 |
| Path 44 | C00022->C00183:[1->2,3->6] | 0.40 | 517.977777778 | 19 | 45 | 0 | 0 |
| Path 45 | C00022->C00183:[1->1,1->2,2->3,3->5,3->6] | 1.00 | 476.417721519 | 23 | 79 | 0 | 0 |
| Path 46 | C00022->C00183:[1->1,2->3,3->5] | 0.60 | 428.8 | 5 | 10 | 0 | 0 |
| Path 47 | C00022->C00183:[1->1,1->2,2->3,3->5,3->6] | 1.00 | 554.418604651 | 17 | 43 | 0 | 0 |
| Path 48 | C00022->C00183:[1->2,3->5] | 0.40 | 517.977777778 | 19 | 45 | 0 | 0 |
| Path 49 | C00022->C00183:[1->2,3->6] | 0.40 | 492.205128205 | 16 | 39 | 0 | 0 |
| Path 50 | C00022->C00183:[1->1,1->2,2->3,3->5,3->6] | 1.00 | 575.527777778 | 18 | 36 | 0 | 0 |
| Path 51 | C00022->C00183:[1->1,1->2,2->3,3->5,3->6] | 1.00 | 423.181818182 | 11 | 33 | 0 | 0 |
| Path 52 | C00022->C00183:[1->2,3->6] | 0.40 | 379.954545455 | 14 | 44 | 0 | 0 |
| Path 53 | C00022->C00183:[1->1,1->2,2->3,3->5,3->6] | 1.00 | 487.134615385 | 18 | 52 | 0 | 0 |
| Path 54 | C00022->C00183:[1->2,3->6] | 0.40 | 548.34375 | 15 | 32 | 0 | 0 |
| Path 55 | C00022->C00183:[1->2,3->6] | 0.40 | 507.956521739 | 17 | 46 | 0 | 0 |
| Path 56 | C00022->C00183:[1->1,1->2,2->3,3->5,3->6] | 1.00 | 500.935483871 | 21 | 62 | 0 | 0 |
| Path 57 | C00022->C00183:[1->1,2->3,3->6] | 0.60 | 463.813559322 | 15 | 59 | 0 | 0 |
| Path 58 | C00022->C00183:[1->2,3->6] | 0.40 | 498.06 | 20 | 50 | 0 | 0 |
| Path 59 | C00022->C00183:[1->2,3->5] | 0.40 | 351.0 | 4 | 8 | 0 | 0 |
| Path 60 | C00022->C00183:[1->1,2->3,3->6] | 0.60 | 566.529411765 | 15 | 34 | 0 | 0 |
| Path 61 | C00022->C00183:[1->1,1->2,2->3,3->5,3->6] | 1.00 | 516.612903226 | 14 | 31 | 0 | 0 |
| Path 62 | C00022->C00183:[1->2,3->6] | 0.40 | 395.844444444 | 15 | 45 | 0 | 0 |
| Path 63 | C00022->C00183:[1->2,3->6] | 0.40 | 507.362068966 | 19 | 58 | 0 | 0 |
| Path 64 | C00022->C00183:[1->2,3->5] | 0.40 | 498.06 | 20 | 50 | 0 | 0 |
| Path 65 | C00022->C00183:[1->2,3->6] | 0.40 | 412.603448276 | 15 | 58 | 0 | 0 |
| Path 66 | C00022->C00183:[1->1,1->2,2->3,3->5,3->6] | 1.00 | 498.333333333 | 7 | 12 | 0 | 0 |
| Path 67 | C00022->C00183:[1->1,2->2,2->3,3->5,3->6] | 1.00 | 420.4375 | 24 | 96 | 0 | 0 |
| Path 68 | C00022->C00183:[1->1,2->3,3->6] | 0.60 | 457.765957447 | 18 | 47 | 0 | 0 |
| Path 69 | C00022->C00183:[1->2,3->6] | 0.40 | 504.863636364 | 18 | 44 | 0 | 0 |
| Path 70 | C00022->C00183:[1->1,2->3,3->6] | 0.60 | 621.085714286 | 19 | 35 | 0 | 0 |
| Path 71 | C00022->C00183:[1->1,2->3,3->6] | 0.60 | 517.41025641 | 16 | 39 | 0 | 0 |
| Path 72 | C00022->C00183:[1->2,3->5] | 0.40 | 507.362068966 | 19 | 58 | 0 | 0 |
| Path 73 | C00022->C00183:[1->2,3->6] | 0.40 | 351.0 | 4 | 8 | 0 | 0 |
| Path 74 | C00022->C00183:[1->1,1->2,2->3,3->5,3->6] | 1.00 | 471.545454545 | 6 | 11 | 0 | 0 |
| Path 75 | C00022->C00183:[1->1,2->2,2->3,3->5,3->6] | 1.00 | 333.8 | 12 | 50 | 0 | 0 |
| Path 76 | C00022->C00183:[1->1,2->3,3->6] | 0.60 | 400.111111111 | 4 | 9 | 0 | 0 |
| Path 77 | C00022->C00183:[1->2,3->6] | 0.40 | 529.444444444 | 13 | 27 | 0 | 0 |
| Path 78 | C00022->C00183:[1->1,2->3,3->6] | 0.60 | 555.757575758 | 15 | 33 | 0 | 0 |
| Path 79 | C00022->C00183:[1->2,3->5] | 0.40 | 507.956521739 | 17 | 46 | 0 | 0 |
| Path 80 | C00022->C00183:[1->2,3->5] | 0.40 | 492.396825397 | 20 | 63 | 0 | 0 |
| Path 81 | C00022->C00183:[1->1,1->2,2->3,3->5,3->6] | 1.00 | 583.520408163 | 21 | 98 | 0 | 0 |
| Path 82 | C00022->C00183:[1->1,2->3,3->6] | 0.60 | 480.85106383 | 14 | 47 | 0 | 0 |
| Path 83 | C00022->C00183:[1->1,1->2,2->3,3->5,3->6] | 1.00 | 381.087912088 | 23 | 91 | 0 | 0 |
| Path 84 | C00022->C00183:[1->1,2->3,3->5] | 0.60 | 372.902439024 | 19 | 82 | 0 | 0 |
| Path 85 | C00022->C00183:[1->1,2->3,3->6] | 0.60 | 603.068965517 | 14 | 29 | 0 | 0 |
| Path 86 | C00022->C00183:[1->1,2->3,3->6] | 0.60 | 380.606741573 | 21 | 89 | 0 | 0 |
| Path 87 | C00022->C00183:[1->1,1->2,2->3,3->5,3->6] | 1.00 | 334.428571429 | 20 | 91 | 0 | 0 |
| Path 88 | C00022->C00183:[1->1,2->3,3->5] | 0.60 | 597.103448276 | 14 | 29 | 0 | 0 |
| Path 89 | C00022->C00183:[1->1,2->3,3->5] | 0.60 | 359.208333333 | 19 | 72 | 0 | 0 |
| Path 90 | C00022->C00183:[1->2,3->6] | 0.40 | 535.413793103 | 13 | 29 | 0 | 0 |
| Path 91 | C00022->C00183:[1->1,1->2,2->3,3->5,3->6] | 1.00 | 559.5625 | 15 | 32 | 0 | 0 |
| Path 92 | C00022->C00183:[1->1,1->2,2->3,3->5,3->6] | 1.00 | 518.978723404 | 17 | 47 | 0 | 0 |
| Path 93 | C00022->C00183:[1->1,2->3,3->5] | 0.60 | 552.032258065 | 14 | 31 | 0 | 0 |
| Path 94 | C00022->C00183:[1->1,2->3,3->5] | 0.60 | 930.690909091 | 20 | 55 | 0 | 0 |
| Path 95 | C00022->C00183:[1->1,2->3,3->6] | 0.60 | 544.0 | 13 | 30 | 0 | 0 |
| Path 96 | C00022->C00183:[1->1,2->3,3->6] | 0.60 | 352.67816092 | 19 | 87 | 0 | 0 |
| Path 97 | C00022->C00183:[1->1,1->2,2->3,3->5,3->6] | 1.00 | 588.513513514 | 20 | 37 | 0 | 0 |
| Path 98 | C00022->C00183:[1->1,2->3,3->6] | 0.60 | 405.036363636 | 19 | 55 | 0 | 0 |
| Path 99 | C00022->C00183:[1->1,1->2,2->3,3->5,3->6] | 1.00 | 573.563636364 | 22 | 55 | 0 | 0 |
| Path 100 | C00022->C00183:[1->1,1->2,2->3,3->5,3->6] | 1.00 | 498.089285714 | 17 | 56 | 0 | 0 |
| Path 101 | C00022->C00183:[1->1,1->2,2->3,3->5,3->6] | 1.00 | 390.024096386 | 20 | 83 | 0 | 0 |
| Path 102 | C00022->C00183:[1->1,2->3,3->6] | 0.60 | 508.025641026 | 18 | 39 | 0 | 0 |
| Path 103 | C00022->C00183:[1->2,3->6] | 0.40 | 607.090909091 | 12 | 22 | 0 | 0 |
| Path 104 | C00022->C00183:[1->1,2->3,3->5] | 0.60 | 392.459459459 | 15 | 37 | 0 | 0 |
| Path 105 | C00022->C00183:[1->1,2->3,3->5] | 0.60 | 492.727272727 | 16 | 55 | 0 | 0 |
| Path 106 | C00022->C00183:[1->1,1->2,2->3,3->5,3->6] | 1.00 | 541.58 | 17 | 50 | 0 | 0 |
| Path 107 | C00022->C00183:[1->2,3->5] | 0.40 | 583.857142857 | 11 | 21 | 0 | 0 |
| Path 108 | C00022->C00183:[1->1,1->2,2->3,3->5,3->6] | 1.00 | 925.2 | 20 | 55 | 0 | 0 |
| Path 109 | C00022->C00183:[1->1,1->2,2->3,3->5,3->6] | 1.00 | 863.75 | 21 | 60 | 0 | 0 |
| Path 110 | C00022->C00183:[1->1,2->3,3->6] | 0.60 | 507.0 | 17 | 37 | 0 | 0 |
| Path 111 | C00022->C00183:[1->1,1->2,2->3,3->5,3->6] | 1.00 | 554.878787879 | 17 | 33 | 0 | 0 |
| Path 112 | C00022->C00183:[1->1,2->3,3->6] | 0.60 | 348.576470588 | 18 | 85 | 0 | 0 |
| Path 113 | C00022->C00183:[1->1,1->2,2->3,3->5,3->6] | 1.00 | 480.264705882 | 18 | 68 | 0 | 0 |
| Path 114 | C00022->C00183:[1->1,2->3,3->5] | 0.60 | 552.606060606 | 16 | 33 | 0 | 0 |
| Path 115 | C00022->C00183:[1->1,2->3,3->5] | 0.60 | 569.5 | 21 | 54 | 0 | 0 |
| Path 116 | C00022->C00183:[1->1,2->3,3->5] | 0.60 | 385.109756098 | 19 | 82 | 0 | 0 |
| Path 117 | C00022->C00183:[1->1,1->2,2->3,3->5,3->6] | 1.00 | 365.04494382 | 21 | 89 | 0 | 0 |
| Path 118 | C00022->C00183:[1->1,1->2,2->3,3->5,3->6] | 1.00 | 394.953271028 | 23 | 107 | 0 | 0 |
| Path 119 | C00022->C00183:[1->2,3->6] | 0.40 | 556.4 | 12 | 35 | 0 | 0 |
| Path 120 | C00022->C00183:[1->1,1->2,2->3,3->5,3->6] | 1.00 | 352.98 | 13 | 50 | 0 | 0 |
| Path 121 | C00022->C00183:[1->1,1->2,2->3,3->5,3->6] | 1.00 | 365.150684932 | 20 | 73 | 0 | 0 |
| Path 122 | C00022->C00183:[1->1,2->3,3->5] | 0.60 | 564.03030303 | 17 | 33 | 0 | 0 |
| Path 123 | C00022->C00183:[1->1,2->3,3->6] | 0.60 | 393.136363636 | 19 | 66 | 0 | 0 |
| Path 124 | C00022->C00183:[1->1,1->2,2->3,3->5,3->6] | 1.00 | 367.637362637 | 23 | 91 | 0 | 0 |
| Path 125 | C00022->C00183:[1->1,1->2,2->3,3->5,3->6] | 1.00 | 566.636363636 | 16 | 33 | 0 | 0 |
| Path 126 | C00022->C00183:[1->1,1->2,2->3,3->5,3->6] | 1.00 | 353.576086957 | 21 | 92 | 0 | 0 |
| Path 127 | C00022->C00183:[1->1,1->2,2->3,3->5,3->6] | 1.00 | 553.886792453 | 20 | 53 | 0 | 0 |
| Path 128 | C00022->C00183:[1->1,1->2,2->3,3->5,3->6] | 1.00 | 369.658823529 | 21 | 85 | 0 | 0 |
| Path 129 | C00022->C00183:[1->1,2->3,3->6] | 0.60 | 467.270833333 | 17 | 48 | 0 | 0 |
| Path 130 | C00022->C00183:[1->1,2->3,3->5] | 0.60 | 522.186046512 | 14 | 43 | 0 | 0 |
| Path 131 | C00022->C00183:[2->3] | 0.20 | 282.76 | 6 | 25 | 0 | 1 |
| Path 132 | C00022->C00183:[1->1,2->3,3->6] | 0.60 | 409.315789474 | 20 | 57 | 0 | 0 |
| Path 133 | C00022->C00183:[1->2,3->6] | 0.40 | 583.857142857 | 11 | 21 | 0 | 0 |
| Path 134 | C00022->C00183:[1->1,1->2,2->3,3->5,3->6] | 1.00 | 372.135135135 | 23 | 111 | 0 | 0 |
| Path 135 | C00022->C00183:[1->1,1->2,2->3,3->5,3->6] | 1.00 | 392.325581395 | 22 | 86 | 0 | 0 |
| Path 136 | C00022->C00183:[1->1,2->3,3->6] | 0.60 | 492.578947368 | 17 | 38 | 0 | 0 |
| Path 137 | C00022->C00183:[1->1,1->2,2->3,3->5,3->6] | 1.00 | 603.633333333 | 15 | 30 | 0 | 0 |
| Path 138 | C00022->C00183:[1->1,2->3,3->6] | 0.60 | 516.0 | 16 | 46 | 0 | 0 |
| Path 139 | C00022->C00183:[1->1,2->3,3->6] | 0.60 | 397.071428571 | 19 | 56 | 0 | 0 |
| Path 140 | C00022->C00183:[1->1,1->2,2->3,3->5,3->6] | 1.00 | 367.159090909 | 21 | 88 | 0 | 0 |
| Path 141 | C00022->C00183:[1->1,1->2,2->3,3->5,3->6] | 1.00 | 525.456521739 | 19 | 46 | 0 | 0 |
| Path 142 | C00022->C00183:[1->1,2->3,3->6] | 0.60 | 367.716049383 | 18 | 81 | 0 | 0 |
| Path 143 | C00022->C00183:[2->3] | 0.20 | 170.766666667 | 12 | 90 | 0 | 1 |
| Path 144 | C00022->C00183:[1->1,1->2,2->3,3->5,3->6] | 1.00 | 359.555555556 | 22 | 90 | 0 | 0 |
| Path 145 | C00022->C00183:[3->3] | 0.20 | 339.45 | 11 | 20 | 0 | 1 |
| Path 146 | C00022->C00183:[1->1,2->3,3->6] | 0.60 | 489.37037037 | 14 | 54 | 0 | 0 |
| Path 147 | C00022->C00183:[1->1,2->3,3->6] | 0.60 | 532.2 | 14 | 40 | 0 | 0 |
| Path 148 | C00022->C00183:[1->1,2->3,3->6] | 0.60 | 446.717391304 | 17 | 46 | 0 | 0 |
| Path 149 | C00022->C00183:[1->1,2->3,3->5] | 0.60 | 548.833333333 | 19 | 36 | 0 | 0 |
| Path 150 | C00022->C00183:[1->1,1->2,2->3,3->5,3->6] | 1.00 | 358.793103448 | 20 | 87 | 0 | 0 |
| Path 151 | C00022->C00183:[3->3] | 0.20 | 344.894736842 | 10 | 19 | 0 | 1 |
| Path 152 | C00022->C00183:[1->1,1->2,2->3,3->5,3->6] | 1.00 | 333.806818182 | 19 | 88 | 0 | 0 |
| Path 153 | C00022->C00183:[1->1,2->3,3->6] | 0.60 | 499.76744186 | 16 | 43 | 0 | 0 |
| Path 154 | C00022->C00183:[1->1,2->3,3->5] | 0.60 | 307.976744186 | 17 | 86 | 0 | 0 |
| Path 155 | C00022->C00183:[1->1,1->2,2->3,3->5,3->6] | 1.00 | 559.571428571 | 18 | 35 | 0 | 0 |
| Path 156 | C00022->C00183:[1->1,2->3,3->5] | 0.60 | 549.288461538 | 19 | 52 | 0 | 0 |
| Path 157 | C00022->C00183:[1->1,2->3,3->5] | 0.60 | 576.90625 | 16 | 32 | 0 | 0 |
| Path 158 | C00022->C00183:[1->1,1->2,2->3,3->5,3->6] | 1.00 | 315.822222222 | 20 | 90 | 0 | 0 |
| Path 159 | C00022->C00183:[1->1,2->3,3->5] | 0.60 | 506.431818182 | 17 | 44 | 0 | 0 |
| Path 160 | C00022->C00183:[1->1,2->3,3->6] | 0.60 | 326.441441441 | 23 | 111 | 0 | 0 |
| Path 161 | C00022->C00183:[1->1,2->3,3->6] | 0.60 | 357.255813953 | 19 | 86 | 0 | 0 |
| Path 162 | C00022->C00183:[1->1,1->2,2->3,3->5,3->6] | 1.00 | 525.079365079 | 22 | 63 | 0 | 0 |
| Path 163 | C00022->C00183:[1->1,1->2,2->3,3->5,3->6] | 1.00 | 369.219512195 | 19 | 82 | 0 | 0 |
| Path 164 | C00022->C00183:[1->1,1->2,2->3,3->5,3->6] | 1.00 | 512.8 | 18 | 45 | 0 | 0 |
| Path 165 | C00022->C00183:[1->1,1->2,2->3,3->5,3->6] | 1.00 | 603.633333333 | 15 | 30 | 0 | 0 |
| Path 166 | C00022->C00183:[1->1,2->3,3->5] | 0.60 | 369.287671233 | 20 | 73 | 0 | 0 |
| Path 167 | C00022->C00183:[1->1,2->3,3->6] | 0.60 | 335.459459459 | 23 | 111 | 0 | 0 |
| Path 168 | C00022->C00183:[1->1,1->2,2->3,3->5,3->6] | 1.00 | 480.216666667 | 19 | 60 | 0 | 0 |
| Path 169 | C00022->C00183:[1->1,1->2,2->3,3->5,3->6] | 1.00 | 377.963855422 | 20 | 83 | 0 | 0 |
| Path 170 | C00022->C00183:[1->1,2->3,3->5] | 0.60 | 296.961038961 | 18 | 77 | 0 | 0 |
| Path 171 | C00022->C00183:[1->1,2->3,3->6] | 0.60 | 358.625 | 17 | 80 | 0 | 0 |
| Path 172 | C00022->C00183:[1->1,1->2,2->3,3->5,3->6] | 1.00 | 430.240740741 | 19 | 54 | 0 | 0 |
| Path 173 | C00022->C00183:[1->1,1->2,2->3,3->5,3->6] | 1.00 | 369.219512195 | 19 | 82 | 0 | 0 |
| Path 174 | C00022->C00183:[1->1,1->2,2->3,3->5,3->6] | 1.00 | 357.787234043 | 22 | 94 | 0 | 0 |
| Path 175 | C00022->C00183:[1->1,1->2,2->3,3->5,3->6] | 1.00 | 928.232142857 | 21 | 56 | 0 | 0 |
| Path 176 | C00022->C00183:[1->1,2->3,3->5] | 0.60 | 494.890909091 | 15 | 55 | 0 | 0 |
| Path 177 | C00022->C00183:[1->1,1->2,2->3,3->5,3->6] | 1.00 | 516.229166667 | 18 | 48 | 0 | 0 |
| Path 178 | C00022->C00183:[1->2,3->6] | 0.40 | 587.04 | 12 | 25 | 0 | 0 |
| Path 179 | C00022->C00183:[1->1,2->3,3->5] | 0.60 | 486.982758621 | 18 | 58 | 0 | 0 |
| Path 180 | C00022->C00183:[1->1,1->2,2->3,3->5,3->6] | 1.00 | 570.764705882 | 18 | 34 | 0 | 0 |
| Path 181 | C00022->C00183:[1->1,1->2,2->3,3->5,3->6] | 1.00 | 559.676470588 | 17 | 34 | 0 | 0 |
| Path 182 | C00022->C00183:[1->1,2->3,3->5] | 0.60 | 363.987654321 | 18 | 81 | 0 | 0 |
| Path 183 | C00022->C00183:[1->1,2->3,3->6] | 0.60 | 403.611940299 | 20 | 67 | 0 | 0 |
| Path 184 | C00022->C00183:[1->1,1->2,2->3,3->5,3->6] | 1.00 | 418.375 | 24 | 104 | 0 | 0 |
| Path 185 | C00022->C00183:[1->1,2->3,3->5] | 0.60 | 798.237288136 | 18 | 59 | 0 | 0 |
| Path 186 | C00022->C00183:[1->1,1->2,2->3,3->5,3->6] | 1.00 | 335.384615385 | 21 | 91 | 0 | 0 |
| Path 187 | C00022->C00183:[1->1,1->2,2->3,3->5,3->6] | 1.00 | 375.013513514 | 21 | 74 | 0 | 0 |
| Path 188 | C00022->C00183:[1->1,2->3,3->5] | 0.60 | 436.697674419 | 18 | 43 | 0 | 0 |
| Path 189 | C00022->C00183:[1->1,2->3,3->5] | 0.60 | 538.103448276 | 13 | 29 | 0 | 0 |
| Path 190 | C00022->C00183:[1->1,2->3,3->5] | 0.60 | 393.662650602 | 20 | 83 | 0 | 0 |
| Path 191 | C00022->C00183:[1->1,1->2,2->3,3->5,3->6] | 1.00 | 555.432432432 | 20 | 37 | 0 | 0 |
| Path 192 | C00022->C00183:[1->1,1->2,2->3,3->5,3->6] | 1.00 | 405.107843137 | 22 | 102 | 0 | 0 |
| Path 193 | C00022->C00183:[3->3] | 0.20 | 335.32 | 12 | 25 | 0 | 1 |
| Path 194 | C00022->C00183:[1->2,3->5] | 0.40 | 556.4 | 12 | 35 | 0 | 0 |
| Path 195 | C00022->C00183:[1->1,2->3,3->5] | 0.60 | 533.228571429 | 18 | 35 | 0 | 0 |
| Path 196 | C00022->C00183:[1->1,2->3,3->6] | 0.60 | 325.723809524 | 21 | 105 | 0 | 0 |
| Path 197 | C00022->C00183:[1->1,1->2,2->3,3->5,3->6] | 1.00 | 540.444444444 | 19 | 36 | 0 | 0 |
| Path 198 | C00022->C00183:[1->1,1->2,2->3,3->5,3->6] | 1.00 | 570.764705882 | 18 | 34 | 0 | 0 |
| Path 199 | C00022->C00183:[1->2,3->5] | 0.40 | 607.090909091 | 12 | 22 | 0 | 0 |
| Path 200 | C00022->C00183:[1->2,3->6] | 0.40 | 519.222222222 | 12 | 27 | 0 | 0 |
| Path 201 | C00022->C00183:[1->1,2->3,3->6] | 0.60 | 342.241071429 | 24 | 112 | 0 | 0 |
| Path 202 | C00022->C00183:[1->1,2->3,3->5] | 0.60 | 391.65 | 17 | 40 | 0 | 0 |
| Path 203 | C00022->C00183:[1->1,1->2,2->3,3->5,3->6] | 1.00 | 528.340909091 | 15 | 44 | 0 | 0 |
| Path 204 | C00022->C00183:[1->1,1->2,2->3,3->5,3->6] | 1.00 | 512.8 | 18 | 45 | 0 | 0 |
| Path 205 | C00022->C00183:[1->1,2->3,3->6] | 0.60 | 513.295454545 | 17 | 44 | 0 | 0 |
| Path 206 | C00022->C00183:[1->1,2->3,3->5] | 0.60 | 927.648148148 | 19 | 54 | 0 | 0 |
| Path 207 | C00022->C00183:[1->1,1->2,2->3,3->5,3->6] | 1.00 | 528.340909091 | 15 | 44 | 0 | 0 |
| Path 208 | C00022->C00183:[1->1,1->2,2->3,3->5,3->6] | 1.00 | 473.258064516 | 21 | 62 | 0 | 0 |
| Path 209 | C00022->C00183:[1->1,2->3,3->6] | 0.60 | 377.24137931 | 20 | 87 | 0 | 0 |
| Path 210 | C00022->C00183:[1->1,2->3,3->5] | 0.60 | 536.448979592 | 16 | 49 | 0 | 0 |
| Path 211 | C00022->C00183:[1->1,2->3,3->5] | 0.60 | 474.915254237 | 18 | 59 | 0 | 0 |
| Path 212 | C00022->C00183:[1->1,2->3,3->6] | 0.60 | 338.875 | 21 | 104 | 0 | 0 |
| Path 213 | C00022->C00183:[1->1,2->3,3->5] | 0.60 | 547.4375 | 16 | 32 | 0 | 0 |
| Path 214 | C00022->C00183:[1->1,2->3,3->6] | 0.60 | 539.516129032 | 15 | 31 | 0 | 0 |
| Path 215 | C00022->C00183:[1->1,2->3,3->6] | 0.60 | 392.259259259 | 18 | 54 | 0 | 0 |
| Path 216 | C00022->C00183:[1->1,1->2,2->3,3->5,3->6] | 1.00 | 583.454545455 | 17 | 33 | 0 | 0 |
| Path 217 | C00022->C00183:[1->1,1->2,2->3,3->5,3->6] | 1.00 | 444.795454545 | 19 | 44 | 0 | 0 |
| Path 218 | C00022->C00183:[1->1,1->2,2->3,3->5,3->6] | 1.00 | 525.456521739 | 19 | 46 | 0 | 0 |
| Path 219 | C00022->C00183:[1->1,1->2,2->3,3->5,3->6] | 1.00 | 492.169491525 | 19 | 59 | 0 | 0 |
| Path 220 | C00022->C00183:[1->1,2->3,3->5] | 0.60 | 513.02173913 | 16 | 46 | 0 | 0 |
| Path 221 | C00022->C00183:[1->1,2->3,3->6] | 0.60 | 372.488636364 | 20 | 88 | 0 | 0 |
| Path 222 | C00022->C00183:[3->3] | 0.20 | 339.45 | 11 | 20 | 0 | 1 |
| Path 223 | C00022->C00183:[1->1,2->3,3->5] | 0.60 | 475.597014925 | 17 | 67 | 0 | 0 |
| Path 224 | C00022->C00183:[1->1,1->2,2->3,3->5,3->6] | 1.00 | 358.793103448 | 20 | 87 | 0 | 0 |
| Path 225 | C00022->C00183:[1->1,2->3,3->5] | 0.60 | 538.56097561 | 15 | 41 | 0 | 0 |
| Path 226 | C00022->C00183:[1->1,1->2,2->3,3->5,3->6] | 1.00 | 500.279411765 | 23 | 68 | 0 | 0 |
| Path 227 | C00022->C00183:[1->1,1->2,2->3,3->5,3->6] | 1.00 | 544.619047619 | 16 | 42 | 0 | 0 |
| Path 228 | C00022->C00183:[1->1,1->2,2->3,3->5,3->6] | 1.00 | 303.320512821 | 19 | 78 | 0 | 0 |
| Path 229 | C00022->C00183:[3->3] | 0.20 | 335.32 | 12 | 25 | 0 | 1 |
| Path 230 | C00022->C00183:[1->1,1->2,2->3,3->5,3->6] | 1.00 | 339.096774194 | 21 | 93 | 0 | 0 |
| Path 231 | C00022->C00183:[1->1,2->3,3->5] | 0.60 | 439.4 | 5 | 10 | 0 | 0 |
| Path 232 | C00022->C00183:[1->1,1->2,2->3,3->5,3->6] | 1.00 | 378.093023256 | 22 | 86 | 0 | 0 |
| Path 233 | C00022->C00183:[3->3] | 0.20 | 335.32 | 12 | 25 | 0 | 1 |
| Path 234 | C00022->C00183:[1->1,1->2,2->3,3->5,3->6] | 1.00 | 403.0 | 16 | 38 | 0 | 0 |
| Path 235 | C00022->C00183:[1->1,1->2,2->3,3->5,3->6] | 1.00 | 407.798165138 | 25 | 109 | 0 | 0 |
| Path 236 | C00022->C00183:[1->1,1->2,2->3,3->5,3->6] | 1.00 | 313.551724138 | 18 | 87 | 0 | 0 |
| Path 237 | C00022->C00183:[1->1,1->2,2->3,3->5,3->6] | 1.00 | 401.58 | 17 | 50 | 0 | 0 |
| Path 238 | C00022->C00183:[1->1,2->3,3->6] | 0.60 | 361.113636364 | 20 | 88 | 0 | 0 |
| Path 239 | C00022->C00183:[1->1,1->2,2->3,3->5,3->6] | 1.00 | 546.6 | 14 | 30 | 0 | 0 |
| Path 240 | C00022->C00183:[1->1,2->3,3->6] | 0.60 | 480.081632653 | 18 | 49 | 0 | 0 |
| Path 241 | C00022->C00183:[1->1,2->3,3->6] | 0.60 | 346.076190476 | 22 | 105 | 0 | 0 |
| Path 242 | C00022->C00183:[3->3] | 0.20 | 339.45 | 11 | 20 | 0 | 1 |
| Path 243 | C00022->C00183:[1->1,1->2,2->3,3->5,3->6] | 1.00 | 367.159090909 | 21 | 88 | 0 | 0 |
| Path 244 | C00022->C00183:[2->3] | 0.20 | 360.0 | 13 | 44 | 0 | 1 |
| Path 245 | C00022->C00183:[1->1,1->2,2->3,3->5,3->6] | 1.00 | 398.416666667 | 21 | 84 | 0 | 0 |
| Path 246 | C00022->C00183:[3->3] | 0.20 | 339.458333333 | 11 | 24 | 0 | 1 |
| Path 247 | C00022->C00183:[1->1,2->3,3->6] | 0.60 | 321.815533981 | 20 | 103 | 0 | 0 |
| Path 248 | C00022->C00183:[3->3] | 0.20 | 329.904761905 | 11 | 21 | 0 | 1 |
| Path 249 | C00022->C00183:[1->1,1->2,2->3,3->5,3->6] | 1.00 | 867.540983607 | 22 | 61 | 0 | 0 |
| Path 250 | C00022->C00183:[1->1,2->2,2->3,3->5,3->6] | 1.00 | 423.138461538 | 19 | 65 | 0 | 0 |
| Path 251 | C00022->C00183:[1->1,2->3,3->6] | 0.60 | 401.815384615 | 16 | 65 | 0 | 0 |
| Path 252 | C00022->C00183:[1->1,2->3,3->6] | 0.60 | 345.573333333 | 18 | 75 | 0 | 0 |
| Path 253 | C00022->C00183:[1->1,2->3,3->6] | 0.60 | 490.666666667 | 16 | 36 | 0 | 0 |
| Path 254 | C00022->C00183:[1->1,1->2,2->3,3->5,3->6] | 1.00 | 500.214285714 | 16 | 56 | 0 | 0 |
| Path 255 | C00022->C00183:[1->1,1->2,2->3,3->5,3->6] | 1.00 | 500.214285714 | 16 | 56 | 0 | 0 |
| Path 256 | C00022->C00183:[1->1,2->3,3->6] | 0.60 | 590.107142857 | 13 | 28 | 0 | 0 |
| Path 257 | C00022->C00183:[1->1,1->2,2->3,3->5,3->6] | 1.00 | 439.714285714 | 18 | 42 | 0 | 0 |
| Path 258 | C00022->C00183:[1->2,3->5] | 0.40 | 587.04 | 12 | 25 | 0 | 0 |
| Path 259 | C00022->C00183:[1->1,2->3,3->6] | 0.60 | 319.454545455 | 22 | 110 | 0 | 0 |
| Path 260 | C00022->C00183:[1->1,2->3,3->5] | 0.60 | 582.833333333 | 19 | 36 | 0 | 0 |
| Path 261 | C00022->C00183:[1->1,2->3,3->6] | 0.60 | 405.552238806 | 17 | 67 | 0 | 0 |
| Path 262 | C00022->C00183:[1->1,2->3,3->5] | 0.60 | 552.705882353 | 17 | 34 | 0 | 0 |
| Path 263 | C00022->C00183:[1->1,2->3,3->5] | 0.60 | 519.511111111 | 18 | 45 | 0 | 0 |
| Path 264 | C00022->C00183:[1->1,2->3,3->5] | 0.60 | 393.591836735 | 16 | 49 | 0 | 0 |
| Path 265 | C00022->C00183:[1->1,1->2,2->3,3->5,3->6] | 1.00 | 470.141025641 | 22 | 78 | 0 | 0 |
| Path 266 | C00022->C00183:[1->1,2->3,3->6] | 0.60 | 556.875 | 16 | 32 | 0 | 0 |
| Path 267 | C00022->C00183:[1->1,2->2,2->3,3->5,3->6] | 1.00 | 414.694736842 | 23 | 95 | 0 | 0 |
| Path 268 | C00022->C00183:[1->1,2->3,3->6] | 0.60 | 610.941176471 | 18 | 34 | 0 | 0 |
| Path 269 | C00022->C00183:[1->1,2->3,3->5] | 0.60 | 550.393939394 | 14 | 33 | 0 | 0 |
| Path 270 | C00022->C00183:[1->1,2->3,3->6] | 0.60 | 529.0 | 12 | 28 | 0 | 0 |
| Path 271 | C00022->C00183:[1->1,1->2,2->3,3->5,3->6] | 1.00 | 375.595238095 | 20 | 84 | 0 | 0 |
| Path 272 | C00022->C00183:[1->1,2->3,3->6] | 0.60 | 329.25 | 21 | 104 | 0 | 0 |
| Path 273 | C00022->C00183:[1->1,2->3,3->5] | 0.60 | 468.016393443 | 20 | 61 | 0 | 0 |
| Path 274 | C00022->C00183:[1->1,1->2,2->3,3->5,3->6] | 1.00 | 500.666666667 | 13 | 30 | 0 | 0 |
| Path 275 | C00022->C00183:[1->1,1->2,2->3,3->5,3->6] | 1.00 | 377.963855422 | 20 | 83 | 0 | 0 |
| Path 276 | C00022->C00183:[1->1,2->3,3->6] | 0.60 | 355.434210526 | 19 | 76 | 0 | 0 |
| Path 277 | C00022->C00183:[1->1,1->2,2->3,3->5,3->6] | 1.00 | 798.15 | 19 | 60 | 0 | 0 |
| Path 278 | C00022->C00183:[1->1,1->2,2->3,3->5,3->6] | 1.00 | 544.619047619 | 16 | 42 | 0 | 0 |
| Path 279 | C00022->C00183:[1->1,1->2,2->3,3->5,3->6] | 1.00 | 387.526785714 | 24 | 112 | 0 | 0 |
| Path 280 | C00022->C00183:[1->1,2->3,3->6] | 0.60 | 368.895348837 | 19 | 86 | 0 | 0 |
| Path 281 | C00022->C00183:[1->1,2->3,3->6] | 0.60 | 515.738095238 | 13 | 42 | 0 | 0 |
| Path 282 | C00022->C00183:[1->1,1->2,2->3,3->5,3->6] | 1.00 | 401.43902439 | 18 | 41 | 0 | 0 |
| Path 283 | C00022->C00183:[1->1,2->3,3->6] | 0.60 | 332.981132075 | 22 | 106 | 0 | 0 |
| Path 284 | C00022->C00183:[1->1,2->3,3->5] | 0.60 | 328.528735632 | 18 | 87 | 0 | 0 |
| Path 285 | C00022->C00183:[1->1,1->2,2->3,3->5,3->6] | 1.00 | 554.878787879 | 17 | 33 | 0 | 0 |
