# Supplementary material for: 13C-metabolic flux ratio and novel carbon path analyses confirmed that Trichoderma reesei uses primarily the respirative pathway also on the preferred carbon source glucose
Source: BMC Syst Biol. 2009 Oct 29;3:104. doi: 10.1186/1752-0509-3-104 (PMC2776023; doi:10.1186/1752-0509-3-104)
Supplement: Additional file 2 — Estimation of subcellular localization of key enzymes by TargetP. TargetP machine learning program [33,34] was used to predict the subcellular localization of key enzymes of amino acid biosynthetic routes in T. reesei. Targeting sequences were predicted for T. reesei sequences that showed the highest homology to the aspartate aminotransferase, acetolactate synthase, serine hydroxymethyltransferase and glycine dehydrogenase in S. cerevisiae. [file 1752-0509-3-104-S2.doc]

Additional file 2.

>Q01802|AATM_YEAST Aspartate aminotransferase, mitochondrial precursor - Saccharomyces cerevisiae

74725,

>TRIRE0074725 | TRIRE0074725 | Trichoderma reesei | estExt_GeneWisePlus.C_20334

MASTTSFPPETVPQAPEDPLFGLARAYKADTSPIKVDLGIGAYRDNNAKPWVLPVVKKAD

DILRNNPELNHEYAPIAGLESFTSKAAELMLGADSPAIADRRVTSVQTISGTGAVHLGAL

FLAKFYKGNRTVYVSNPTWANHHQIFSNVGITVAQYPYFNKETRGLDFEGMTAAISAAPE

RSIILLHPCAHNPTGVDPTLDQWKQLAVIIREKKHFPFFDCAYQGFASGDLARDASAVRY

FIEQGFELVVAQSFAKNFGLYGERAGCFHFVGAPAADAAETVTRIASQLAILQRSEISNP

PLYGARIASTVLNDPQLFSEWEENLRTMSGRIIDMRKALRSKLEELETPGTWNHITDQIG

MFSFTGLTEPQVKKLREEYHIYMTKNGRISMAGLNTHNIDHVAQAIRKVVGETQ*

### targetp v1.1 prediction results ##################################

Number of query sequences: 1

Cleavage site predictions not included.

Using NON-PLANT networks.

Name Len mTP SP other Loc RC

----------------------------------------------------------------------

TRIRE0074725 414 0.099 0.092 0.870 _ 2

----------------------------------------------------------------------

cutoff 0.000 0.000 0.000

05233

>TRIRE0005233 | TRIRE0005233 | Trichoderma reesei | fgenesh1_pm.C_scaffold_21000016

MMLSTLRVASRRAVALRPATLRLTSRAALSTWINVPQGPPVRCITEAFKADKFEQKINLG

VGAYRDDAGKPYVLPSVREAERKIVDAKLNKEYAGITGVPEFPPLAAKLAYGPNQSVLDR

VAITQTISGTGALRVGAAFLQRFYSGDKKIFIPNPSWANHKAVFSDAGLKVETYRYYNKD

TIGLDFDGLIADLKAAPLASVFLFHACAHNPTGVDPTPEQWKEISKVVKEQSHFAFFDMA

YQGFASGDTDRDAFAVRYFVEQGHDIALCQSFAKNMGLYGERVGAFSLTTADADEKKRVE

SQLKILIRPMYSNPPIHGARIASEVLSNPKLYKQWLGEVKEMADRIITMRALLKENLEKL

GSKHDWSHITSQIGMFAYTGLNAEEMEKLAKEYSVYATKDGRISVAGITSSNVGRLAEAI

YKVKG*

### targetp v1.1 prediction results ##################################

Number of query sequences: 1

Cleavage site predictions not included.

Using NON-PLANT networks.

Name Len mTP SP other Loc RC

----------------------------------------------------------------------

TRIRE0005233 425 0.973 0.023 0.040 M 1

----------------------------------------------------------------------

cutoff 0.000 0.000 0.000

>P23542|AATC_YEAST Aspartate aminotransferase, cytoplasmic - Saccharomyces cerevisiae

74725, 05233

>P07342|ILVB_YEAST Acetolactate synthase catalytic subunit, mitochondrial precursor - Saccharomyces cerevisiae

74123

>TRIRE0074123 | TRIRE0074123 | Trichoderma reesei | estExt_GeneWisePlus.C_11137

MLRSRQVTARAVRALGQARAFTSTTKPVMIQSSQRKQANASAAPQVRPVPSPAFNAEDKD

RSHVQPLVNPSKPDMDESFIGKTGGEIFHEMMLRQGVKHIFGYPGGAILPVFDAIYNSKH

FDFILPRHEQGAGHMAEGYARASGKPGVVLVTSGPGATNVITPMQDALSDGTPLVVFCGQ

VPTTAIGSDAFQEADVVGISRACTKWNVMVKSVAELPRRINEAFEIATSGRPGPVLVDLP

KDVTAGILRRAIPTETALPSLPSAASRAAMELSSKQLNASIKRAADLINIAKKPVIYAGQ

GVIQSEGGVELLKQLADKASIPVTTTLHGLGAFDELDEKSLHMLGMHGSAYANMAMQQAD

LIIALGSRFDDRVTLNVSKFAPAARQAAAEGRGGIIHFEIMPKNINKVIQATEAVEGDVA

TNLKHLIPQIAEKSMADRGEWFGLINEWKKKWPLSNYQRAERAGLIKPQTVMEEISNLTA

NRKDKTYIATGVGQHQMWVAQHFRWRHPRSMITSGGLGTMGYGLPAAIGAKVAQPDALVI

DVDGDASFNMTLTELSTAAQFNIGVKVVVLNNEEQGMVTQWQNLFYEDRYAHTHQKNPDF

MKLADAMGVQHQRVTEPEKLVDALTWLINTDGPALLEVVTDKKVPVLPMVPAGSALHEFL

VFEPEKDKQRRELMKERTKGVHS*

### targetp v1.1 prediction results ##################################

Number of query sequences: 1

Cleavage site predictions not included.

Using NON-PLANT networks.

Name Len mTP SP other Loc RC

----------------------------------------------------------------------

TRIRE0074123 683 0.912 0.018 0.130 M 2

----------------------------------------------------------------------

cutoff 0.000 0.000 0.000

>P37292|GLYM_YEAST Serine hydroxymethyltransferase, mitochondrial precursor - Saccharomyces cerevisiae

121686, 65295

>P37291|GLYC_YEAST Serine hydroxymethyltransferase, cytosolic - Saccharomyces cerevisiae

121686, 65295

>TRIRE0121686 | TRIRE0121686 | Trichoderma reesei | estExt_fgenesh5_pg.C_80360

MSTYALPASHKEMLEKSLLESDPEVAAIMKDEVQRQRESIVLIASENITSRAVFDALGSP

MSNKYSEGLPGARYYGGNQHIDQIELLCQRRALEAFHLDPAKWGVNVQCLSGSPANLQVY

QAIMPPHGRLMGLDLPHGGHLSHGYQTPQRKISAVSTYFETMPYRVNLETGIIDYDQLQQ

NALLYRPKVLVAGTSAYCRLIDYERMRKIADSVGAYLVVDMAHISGLIAAEAIPSPFQWA

DIVTTTTHKSLRGPRGAMIFFRKGVRSVDPKTGKETLYDLEDPINFSVFPGHQGGPHNHT

ITALAVALKQAQTPEFKAYQEKVVSNAKTLEVKFKELGHKLVADGTDSHMVLLDLRQFNL

DGARVETVLEQINIACNKNAIPGDKSALTPCGIRIGTPAMTSRGFGEKDFERVALYIDQA

IKLCVEIQASLPKPNNKLKDFKAEVTSGKVAKLGELQKEIAAWASSFPLPVEGWRLDAGI

*

### targetp v1.1 prediction results ##################################

Number of query sequences: 1

Cleavage site predictions not included.

Using NON-PLANT networks.

Name Len mTP SP other Loc RC

----------------------------------------------------------------------

TRIRE0121686 480 0.075 0.073 0.925 _ 1

----------------------------------------------------------------------

cutoff 0.000 0.000 0.000

>TRIRE0065295 | TRIRE0065295 | Trichoderma reesei | e_gw1.16.86.1

MLTRGTRQLLSAHLAKADPAVFDIIEREKNRQKHFINLIPSENFTSQAVLDALGSVMQNK

YSEGYPGARYYGGNEFIDQSERLCQQRALEAFGLDPKSWGVNVQGSTSSNGDSSSALSGA

PANLYVYSALMDTHDRLMGLDLPHGGHLSHGYQTPTKKISAVSKYFETLPYQLDERTGYI

DYDNLEKMASIYRPKIIVAGTSAYSRLIDYKRIREICDKVNAYMVADMAHISGLVAAKVL

PGPFPFADIVTTTSHKSLRGPRGALIFFRKGVRRQNPKTKEEELYNLEGPINNSVFPGHQ

GGPHNHTITALAVALKQTQTPEFQAYQSQVLANAKAFAKRLGEEKGKGGLGYSLVSGGTD

NHLVLVDLKPHGIDGSRVERVLELVGVAANKNTVPGDRSALVPGGLRMGTPAMTTRGFHE

DDFVRVADVVDRAVTIATRINKTVRAAAEERGEKSPGKLKLFVEHLGNGDGDPEIVQLRS

EVADWVGTYPLPWDSTQ*

### targetp v1.1 prediction results ##################################

Number of query sequences: 1

Cleavage site predictions not included.

Using NON-PLANT networks.

Name Len mTP SP other Loc RC

----------------------------------------------------------------------

TRIRE0065295 497 0.549 0.051 0.371 M 5

----------------------------------------------------------------------

cutoff

>P49095|GCSP_YEAST Glycine dehydrogenase [decarboxylating], mitochondrial precursor - Saccharomyces cerevisiae

81339

>TRIRE0081339 | TRIRE0081339 | Trichoderma reesei | estExt_GeneWisePlus.C_240047

MRQIRAANKLPWKDFSARHIGPRDDEIEQMLKALGPGAETLDAFVSQVIPADVLDPPKKT

IQSHVYSESGIVKAFKNMNAHNDIRVWMNGGGYYPVEIPAVIKRNVLENPAWYTSYTPYQ

PEISQGRLESLLNFQTMVSDLTGLPVANASLLDEGTAASEAMTMSLTSLPASRQKRSGKT

YVVSHLVHDATIRVMHGRAEGFGIHIQVLDLGAPDAQDKIKALGDDLIGVMVQYPDSNGG

VSDFRELAELAHAQGTLLSAATDLSNLTLLTPPGEWGADIAFGNAQRFGVPLGYGGPHAA

FMAVQEGSKRRLPGRLIGVSRDRRGDRALRLALQTREQHIRREKATSNVCTAQALLANMS

AMYAIYHGPEQLREMAINNLRHARMIQAAAQHYGLNVSTRSVDADGKVLSDTVALHFDDP

VVCRALRRELMDQGISSGKAWQPNELTVAYKNHSDADLDVSAKYWKEGFAQSSEELIQSL

PESVRRQSKFLTHPVFNSHHSETEMLRYMYHLQSKDLSLVHSMIPLGSCTMKLNGTTQME

LIGLENASNIHPHAPYSCAKGYQRLFDATSAQLAALTGMDGTSLQPNSGAQGEFAGLRAI

RKYHEQQPGPKRDICLIPVSAHGTNPASAAMVGMRVVPIKCDTKTGNLDLEDLEAKCKKH

ASELGAIMITYPSTYGVFEPQVRKVCDIVHQYGGLVYMDGANMNAQIGLTSPGALGADVC

HLNLHKTFCIPHGGGGPGIGPICVKKHLIPYLPHKSTQTPVSSAAFGSASIVPISWSYIS

TMGDAGLRKATTVALLNANYLLTRLKDHYPILYTNDKGRCAHEFIVDARPFQKTAGIEAI

DIAKRLQDYGFHAPTMSWPVPNTLMIEPTESESKEELDRFVDAMISIRNEIREIEEGKQP

RQGNVLKNAPHPQRDLILGDAEGKWDRPYSREKAAYPLPYLLEKKFWPTVGRVDDTYGDT

NLFCTCPPVEDTTGSA*

### targetp v1.1 prediction results ##################################

Number of query sequences: 1

Cleavage site predictions not included.

Using NON-PLANT networks.

Name Len mTP SP other Loc RC

----------------------------------------------------------------------

TRIRE0081339 976 0.413 0.048 0.535 _ 5

----------------------------------------------------------------------

cutoff 0.000 0.000 0.000

>P49095|GCSP_YEAST Glycine dehydrogenase [decarboxylating], mitochondrial precursor - Saccharomyces cerevisiae

81339

>TRIRE0081339 | TRIRE0081339 | Trichoderma reesei | estExt_GeneWisePlus.C_240047

MRQIRAANKLPWKDFSARHIGPRDDEIEQMLKALGPGAETLDAFVSQVIPADVLDPPKKT

IQSHVYSESGIVKAFKNMNAHNDIRVWMNGGGYYPVEIPAVIKRNVLENPAWYTSYTPYQ

PEISQGRLESLLNFQTMVSDLTGLPVANASLLDEGTAASEAMTMSLTSLPASRQKRSGKT

YVVSHLVHDATIRVMHGRAEGFGIHIQVLDLGAPDAQDKIKALGDDLIGVMVQYPDSNGG

VSDFRELAELAHAQGTLLSAATDLSNLTLLTPPGEWGADIAFGNAQRFGVPLGYGGPHAA

FMAVQEGSKRRLPGRLIGVSRDRRGDRALRLALQTREQHIRREKATSNVCTAQALLANMS

AMYAIYHGPEQLREMAINNLRHARMIQAAAQHYGLNVSTRSVDADGKVLSDTVALHFDDP

VVCRALRRELMDQGISSGKAWQPNELTVAYKNHSDADLDVSAKYWKEGFAQSSEELIQSL

PESVRRQSKFLTHPVFNSHHSETEMLRYMYHLQSKDLSLVHSMIPLGSCTMKLNGTTQME

LIGLENASNIHPHAPYSCAKGYQRLFDATSAQLAALTGMDGTSLQPNSGAQGEFAGLRAI

RKYHEQQPGPKRDICLIPVSAHGTNPASAAMVGMRVVPIKCDTKTGNLDLEDLEAKCKKH

ASELGAIMITYPSTYGVFEPQVRKVCDIVHQYGGLVYMDGANMNAQIGLTSPGALGADVC

HLNLHKTFCIPHGGGGPGIGPICVKKHLIPYLPHKSTQTPVSSAAFGSASIVPISWSYIS

TMGDAGLRKATTVALLNANYLLTRLKDHYPILYTNDKGRCAHEFIVDARPFQKTAGIEAI

DIAKRLQDYGFHAPTMSWPVPNTLMIEPTESESKEELDRFVDAMISIRNEIREIEEGKQP

RQGNVLKNAPHPQRDLILGDAEGKWDRPYSREKAAYPLPYLLEKKFWPTVGRVDDTYGDT

NLFCTCPPVEDTTGSA*

### targetp v1.1 prediction results ##################################

Number of query sequences: 1

Cleavage site predictions not included.

Using NON-PLANT networks.

Name Len mTP SP other Loc RC

----------------------------------------------------------------------

TRIRE0081339 976 0.413 0.048 0.535 _ 5

----------------------------------------------------------------------

cutoff 0.000 0.000 0.000
